# Supplementary figures and images for: Variables associated with owner perceptions of the health of their dog: Further analysis of data from a large international survey
Source: PLoS One. 2024 May 15;19(5):e0280173. doi: 10.1371/journal.pone.0280173 (PMC11095744; doi:10.1371/journal.pone.0280173)

(a)

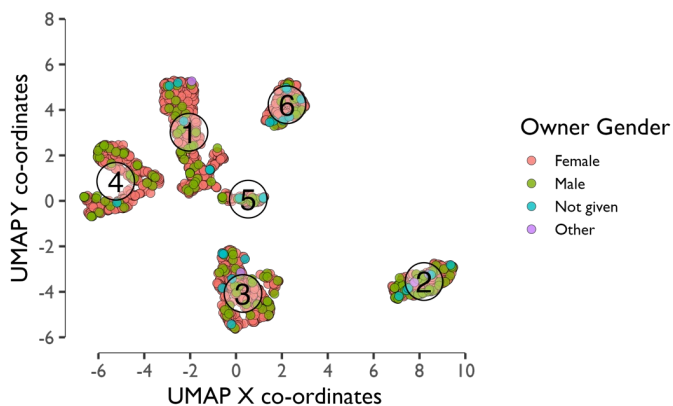

(b)

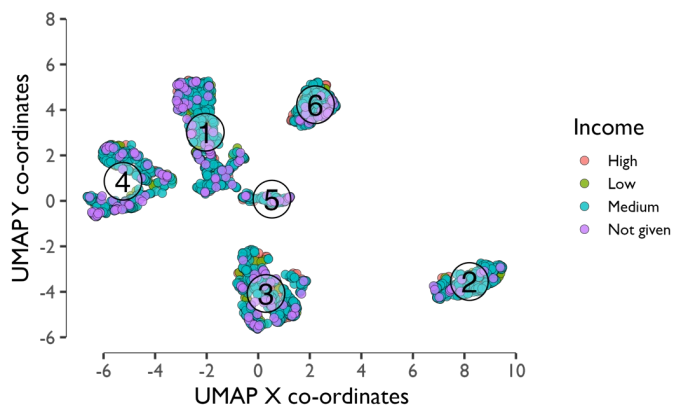

(c)

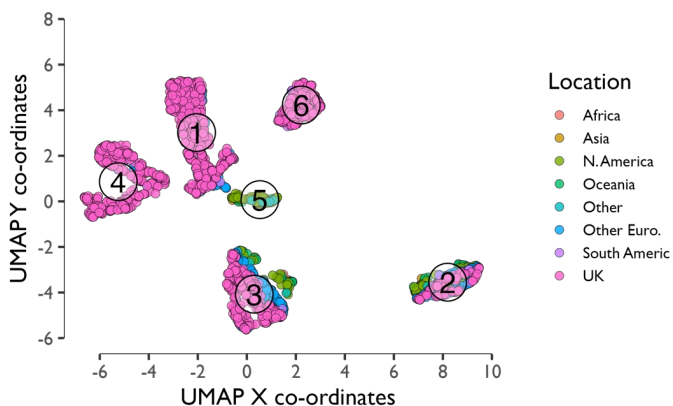

(d)

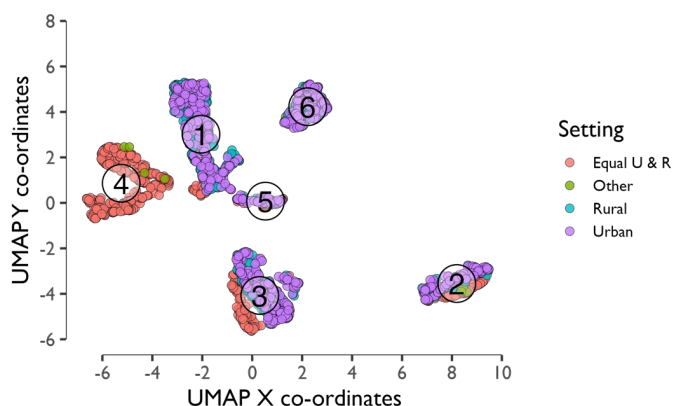

(e)

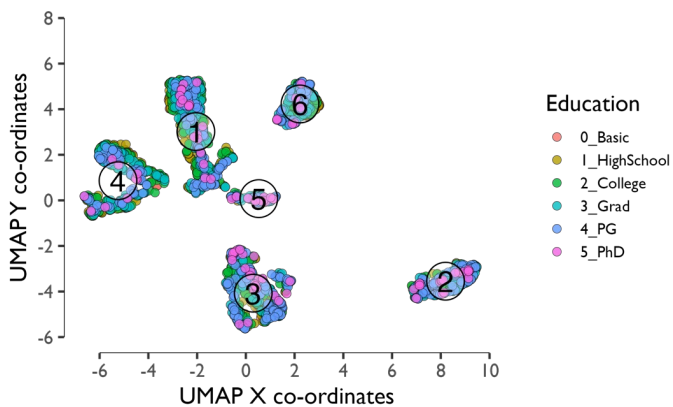

(f)

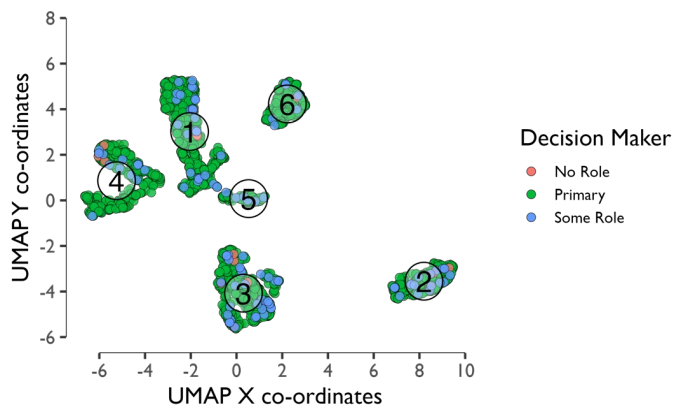

Supplement: S1 Fig — Owner-pet metadata were pre-processed with all factors as numeric and subject to dimension reduction with the UMAP projection technique. Owner variables included in this visualisation were diet, sex, location, education and income; animal variables included were age, sex, neuter status, diet and breed size category. Healthcare variables were not included. Each individual row of the data (owner-dog combination) contributes one UMAP x and y coordinate, analogous to PC1 and PC2 in a principal component analysis. Four or five distinct clusters were evident. Points are colour-coded by owner gender (a), income (b), location (c), setting (d), education € and decision-maker status (f) as indicated in the legend. (PDF) [file pone.0280173.s001.pdf]

(a)

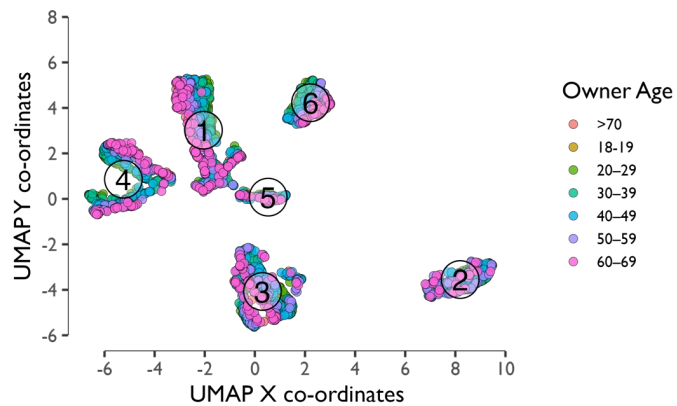

(b)

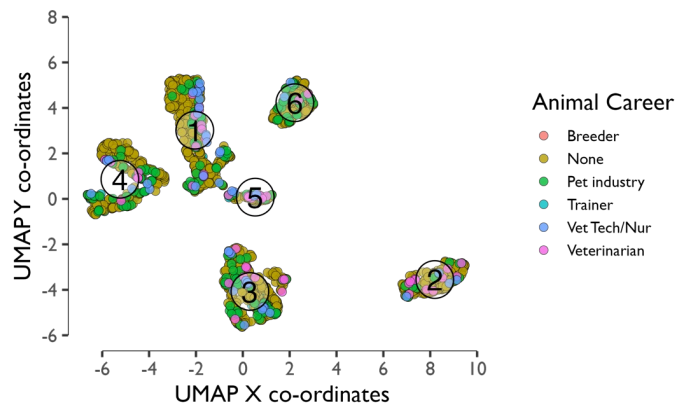

(c)

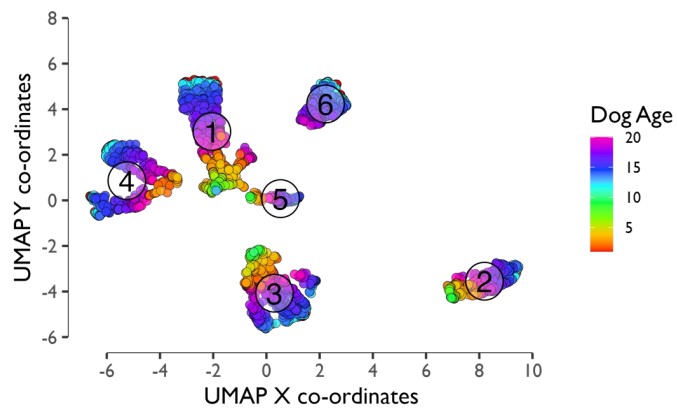

(d)

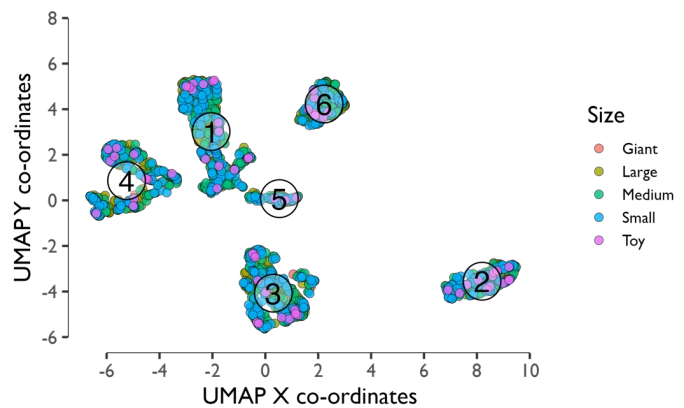

(e)

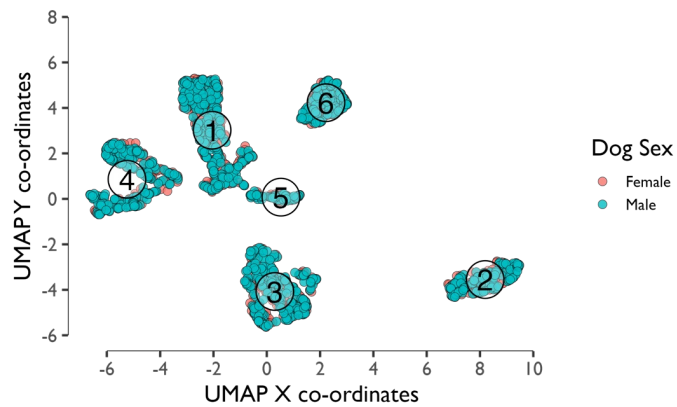

(f)

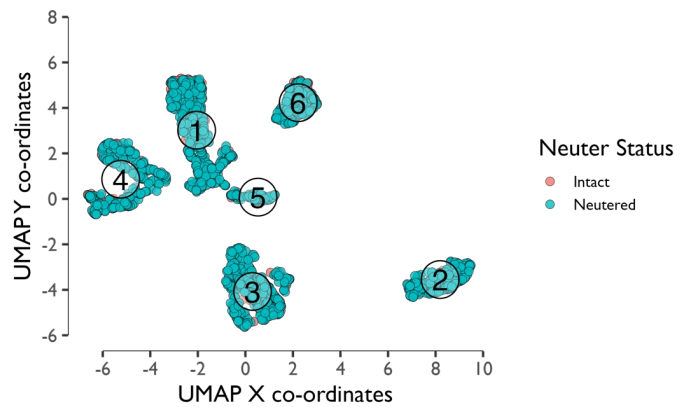

Supplement: S2 Fig — Owner-pet metadata were pre-processed with all factors as numeric and subject to dimension reduction with the UMAP projection technique. Owner variables included in this visualisation were diet, sex, location, education and income; animal variables included were age, sex, neuter status, diet and breed size category. Healthcare variables were not included. Each individual row of the data (owner-dog combination) contributes one UMAP x and y coordinate. Four or five distinct clusters were evident. Points are colour-coded by owner age (a), animal career (b), dog age (c), breed size category (d), dog sex € and neuter status (f) as indicated in the legend. (PDF) [file pone.0280173.s002.pdf]

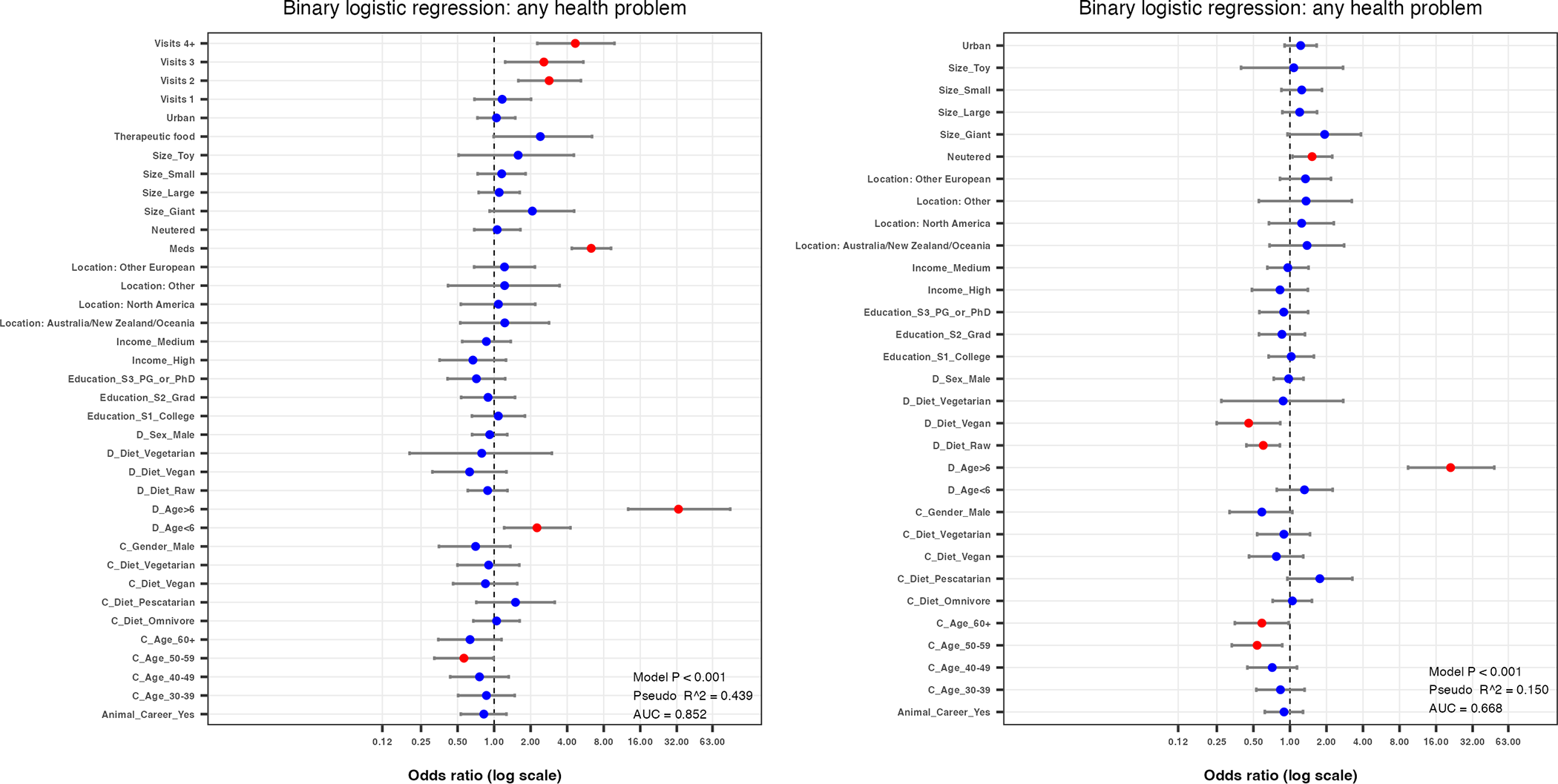

Supplement: S3 Fig — Multiple binary logistic regression model, on data from all owners, with any health problem as the outcome variable and including either all owner, animal and including either all healthcare variables (a) or only the owner-animal metadata (b). The dots represent the odds ratio for each variable, whilst the bars represent 99% confidence intervals (99%-CI). Variables where the 99%-CI range does not include 1.0 (vertical dotted line) are depicted in red, whilst those that include 1.0 are depicted in blue. Note the logarithmic scale for the X-axis. (TIF) [file pone.0280173.s003.tif]

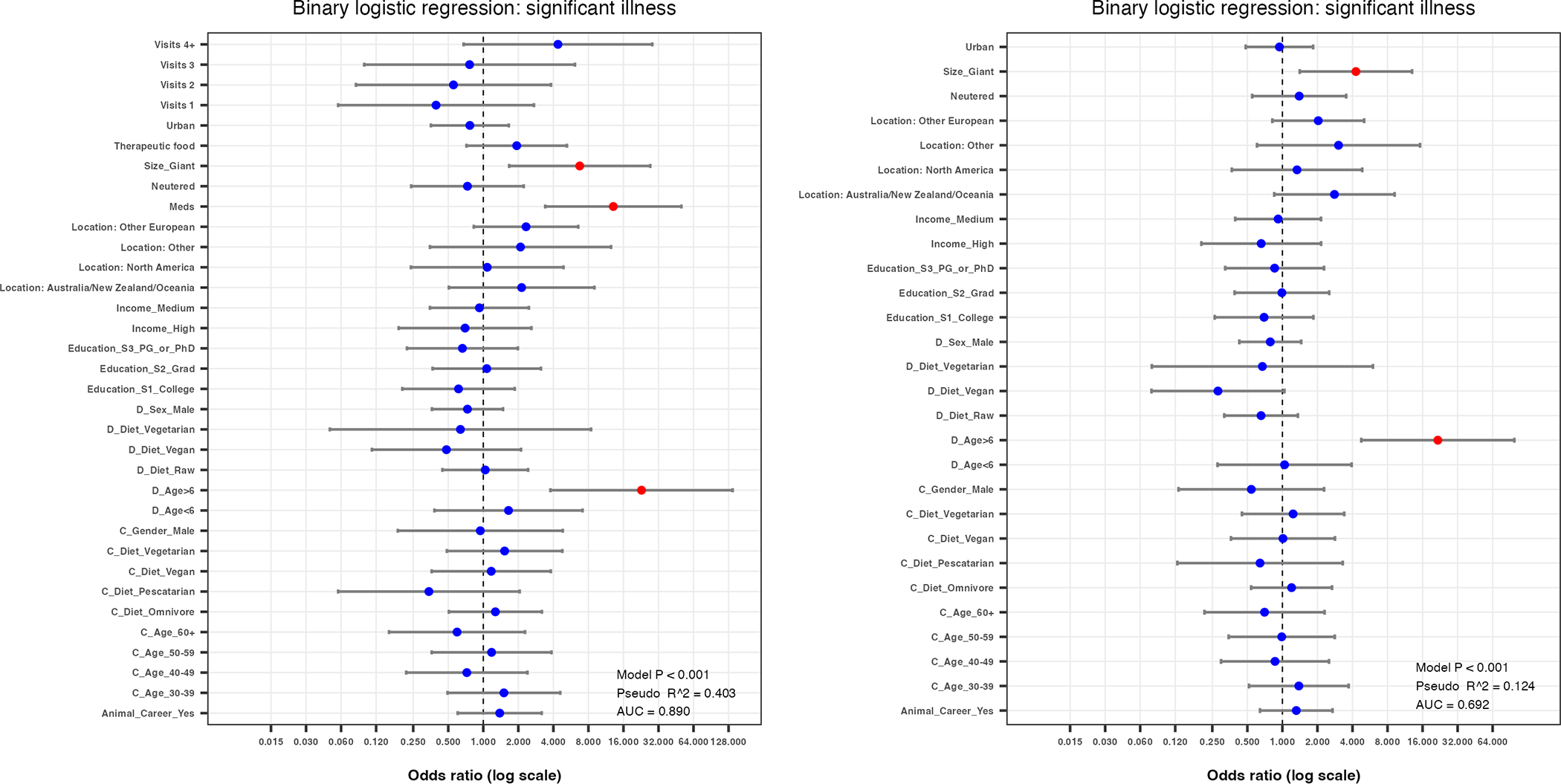

Supplement: S4 Fig — Multiple binary logistic regression model with significant illness as the outcome variable, on data from owners who were primary carers, either all owner, animal and including either all healthcare variables (a) or only the owner-animal metadata (b). The dots represent the odds ratio for each variable, whilst the bars represent 99% confidence intervals (99%-CI). Variables where the 99%-CI range does not include 1.0 (vertical dotted line) are depicted in red, whilst those that include 1.0 are depicted in blue. Note the logarithmic scale for the X-axis. (TIF) [file pone.0280173.s004.tif]

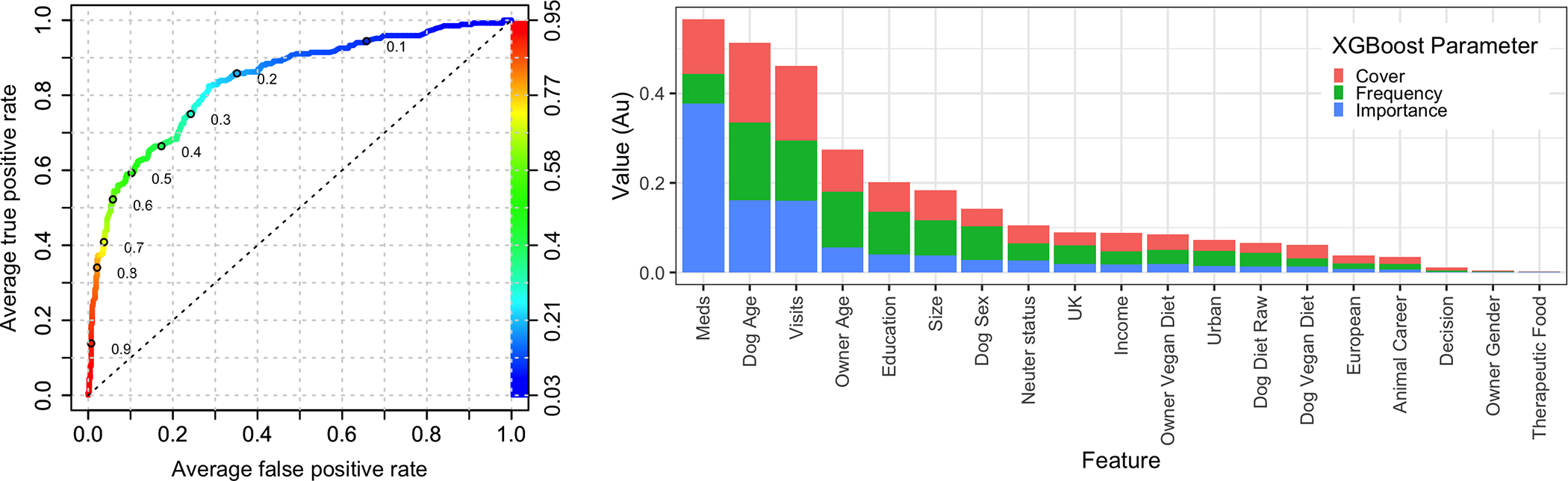

Supplement: S5 Fig — (a) Receiver operating characteristic curve of a prediction model containing all variables (owner, animal and healthcare). This shows the increasing true positive and false positive rates, with decrease of the threshold probability for prediction of any health problem. Prediction accuracy was good as assessed by ROC analysis (area under curve 0.838, 99%-CI: 0.797–0.879). Acceptance threshold is indicated by the colour bar on the right-hand side and shown at discrete points on the curve. (b) Graph depicting the relative contribution of predictor variables to the all-variable XGBoost model for the any health problem binary. ‘Importance’ represents fractional contribution of each feature to the model, based on the total gain from including each feature; ‘cover’ represents the number of observations related to this feature in the model); ‘frequency’ represents the relative number of times a feature has been used in trees). Variables are organised in order of importance left to right, based on the sum of the 3 metrics, whilst variables that are not shown did not contribute to the final model. (TIF) [file pone.0280173.s005.tif]

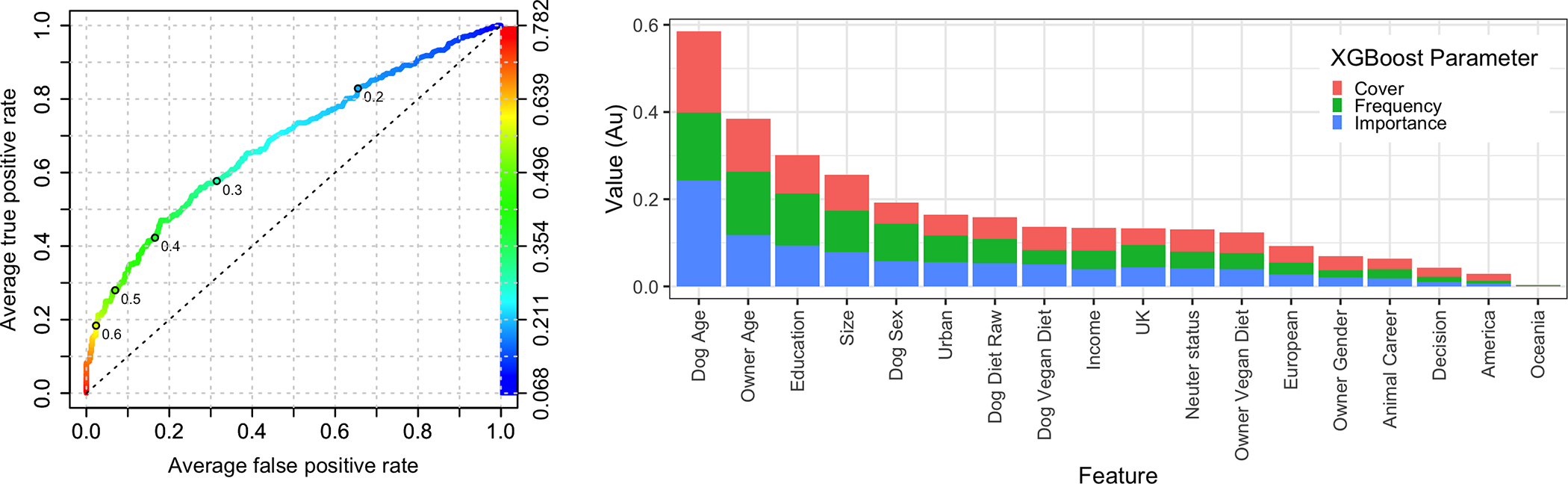

Supplement: S6 Fig — (a) Receiver operating characteristic curve of a reduced prediction model only containing owner and animal variables. This shows the increasing true positive and false positive rates, with decrease of the threshold probability for prediction of any health problem. Prediction accuracy was moderate, as assessed by ROC analysis (AUC 0.682, 99%-CI: 0.628–0.737). Acceptance threshold is indicated by the colour bar on the right-hand side and shown at discrete points on the curve. The fact that the prediction thresholds are all low (< 0.5) shows that this model struggles to predict health issues. (b) Graph depicting the relative contribution of predictor variables to the all-variable XGBoost model for the any health problem binary. ‘Importance’ represents fractional contribution of each feature to the model, based on the total gain from including each feature; ‘cover’ represents the number of observations related to this feature in the model); ‘frequency’ represents the relative number of times a feature has been used in trees). Variables are organised in order of importance left to right, based on the sum of the 3 metrics, whilst variables that are not shown did not contribute to the final model. (TIF) [file pone.0280173.s006.tif]

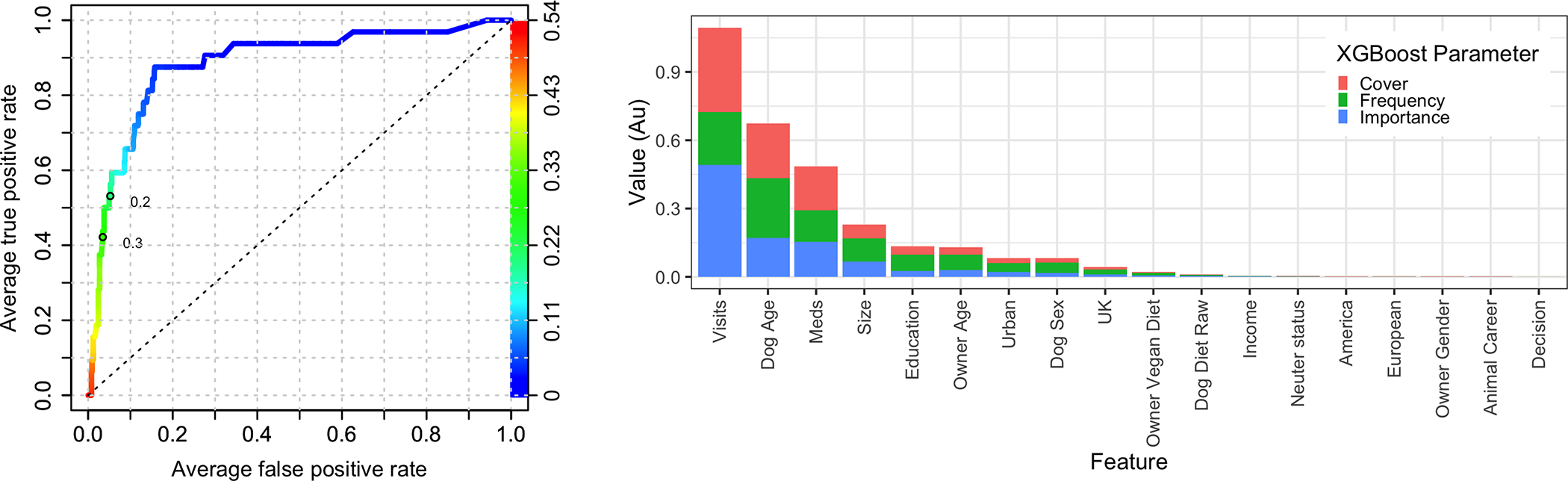

Supplement: S7 Fig — (a) Receiver operating characteristic curve of a prediction model containing all variables (owner, animal and healthcare). This shows the increasing true positive and false positive rates, with decrease of the threshold probability for prediction of significant illness. Prediction accuracy was good, as assessed by ROC analysis (area under curve 0.884, 99%-CI: 0.796–0.972). Acceptance threshold is indicated by the colour bar on the right-hand side and shown at discrete points on the curve. (b) Graph depicting the relative contribution of predictor variables to the all-variable XGBoost model for the significant illness binary. ‘Importance’ represents fractional contribution of each feature to the model, based on the total gain from including each feature; ‘cover’ represents the number of observations related to this feature in the model); ‘frequency’ represents the relative number of times a feature has been used in trees). Variables are organised in order of importance left to right, based on the sum of the 3 metrics, whilst variables that are not shown did not contribute to the final model. (TIF) [file pone.0280173.s007.tif]

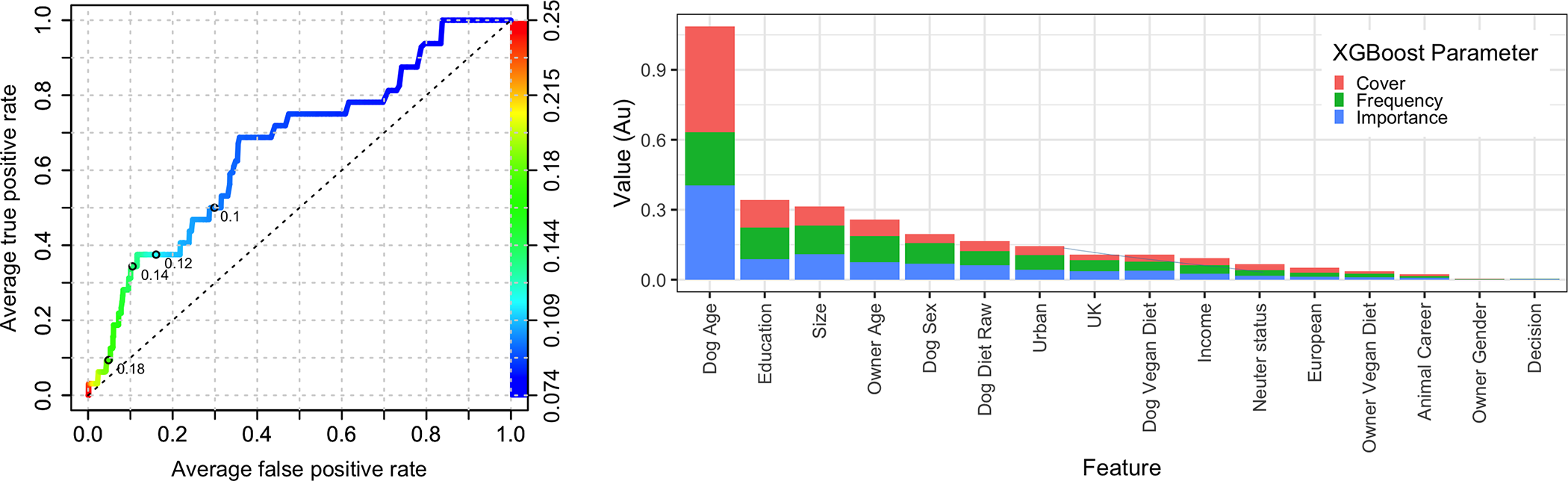

Supplement: S8 Fig — (a) Receiver operating characteristic curve of a reduced prediction model only containing owner and animal variables. This shows the increasing true positive and false positive rates, with decrease of the threshold probability for prediction of significant illness. Prediction accuracy was moderate, as assessed by ROC analysis (area under curve 0.664, 99%-CI: 0.535–0.693). Acceptance threshold is indicated by the colour bar on the right-hand side, and also shown at discrete points on the curve. (b) Graph depicting the relative contribution of predictor variables to the all-variable XGBoost model for the significant illness binary. ‘Importance’ represents fractional contribution of each feature to the model, based on the total gain from including each feature; ‘cover’ represents the number of observations related to this feature in the model); ‘frequency’ represents the relative number of times a feature has been used in trees). Variables are organised in order of importance left to right, based on the sum of the 3 metrics, whilst variables that are not shown did not contribute to the final model. (TIF) [file pone.0280173.s008.tif]
