# Supplementary material for: Variables associated with owner perceptions of the health of their dog: Further analysis of data from a large international survey
Source: PLoS One. 2024 May 15;19(5):e0280173. doi: 10.1371/journal.pone.0280173 (PMC11095744; doi:10.1371/journal.pone.0280173)
Supplement: S1 Table — (DOCX) [file pone.0280173.s009.docx]

**S1 Table. Results of simple (i.e., univariable) binary logistic regression analyses examining associations between owner, animal and veterinary variables and the *any health problem* binary (the presence of any illness as reported by the owner) for all owners.**

| **Variable ^1^** | **Estimate** | **Odds ratio** | **99%-CI** | ***P*-value** | **Pseudo-R^2^** | **BIC** | **AUC** |
| --- | --- | --- | --- | --- | --- | --- | --- |
| **Owner variables** |  |  |  |  |  |  |  |
| Location |  |  |  |  | 0.0030 | 3128 | 0.542 |
| United Kingdom | Ref | --- | --- | --- |  |  |  |
| Other European country | 0.17 (0.119) | 1.180 | 0.867, 1.600 | 0.165 |  |  |  |
| North America | 0.31 (0.185) | 1.359 | 0.840, 2.185 | 0.097 |  |  |  |
| Australia / New Zealand / Oceania | 0.24 (0.208) | 1.277 | 0.741, 2.177 | 0.240 |  |  |  |
| Other region | 0.08 (0.239) | 1.081 | 0.574, 1.984 | 0.745 |  |  |  |
| Setting ^2^ |  |  |  |  | 0.0015 | 3107 | 0.514 |
| Urban | Ref | --- | --- | --- |  |  |  |
| Rural | 0.14 (0.089) | 1.154 | 0.918, 1.450 | 0.107 |  |  |  |
| Owner age (years) |  |  |  |  | 0.0062 | 3122 | 0.539 |
| <30 | Ref | --- | --- | --- |  |  |  |
| 30-39 | -0.05 (0.137) | 0.950 | 0.668, 1.353 | 0.710 |  |  |  |
| 40-49 | -0.18 (0.140) | 0.838 | 0.585, 1.202 | 0.207 |  |  |  |
| 50-59 | -0.37 (0.138) | 0.690 | 0.483, 0.984 | 0.007 |  |  |  |
| ≥60 | -0.29 (0.144) | 0.749 | 0.517, 1.086 | 0.045 |  |  |  |
| Owner gender |  |  |  |  | 0.0034 | 3104 | 0.513 |
| Female | Ref | --- | --- | --- |  |  |  |
| Male | -0.40 (0.170) | 0.670 | 0.428, 1.028 | 0.019 |  |  |  |
| Education |  |  |  |  | 0.0005 | 3124 | 0.528 |
| Basic or high school | Ref | --- | --- | --- |  |  |  |
| College | -0.10 (0.126) | 0.904 | 0.654, 1.252 | 0.423 |  |  |  |
| Graduate | -0.09 (0.125) | 0.912 | 0.661, 1.260 | 0.462 |  |  |  |
| Postgraduate | -0.10 (0.132) | 0.909 | 0.647, 1.276 | 0.466 |  |  |  |
| Income |  |  |  |  | 0.0015 | 3115 | 0.516 |
| Low | Ref | --- | --- | --- |  |  |  |
| Medium | -0.15 (0.118) | 0.863 | 0.638, 1.172 | 0.214 |  |  |  |
| High | -0.24 (0.154) | 0.785 | 0.527, 1.168 | 0.117 |  |  |  |
| Animal-related career |  |  |  |  | 0.0017 | 3107 | 0.514 |
| No | Ref | --- | --- | --- |  |  |  |
| Yes | 0.19 (0.108) | 1.204 | 0.910, 1.589 | 0.086 |  |  |  |
| Owner diet ^3^ |  |  |  |  |  |  |  |
| Omnivore | Ref | --- | --- | --- | 0.0066 | 3122 | 0.554 |
| Omnivore (restricted) | 0.18 (0.114) | 1.196 | 0.891, 1.603 | 0.160 |  |  |  |
| Pescatarian | 0.58 (0.192) | 1.779 | 1.083, 2.924 | 0.003 |  |  |  |
| Vegetarian | 0.24 (0.149) | 1.273 | 0.865, 1.864 | 0.105 |  |  |  |
| Vegan | 0.05 (0.113) | 1.046 | 0.782, 1.398 | 0.689 |  |  |  |
| Owner on vegan diet ^3^ |  |  |  |  |  |  |  |
| No | Ref | --- | --- | --- |  |  |  |
| Yes | -0.08 (0.102) | 0.924 | 0.709, 1.200 | 0.441 | 0.0003 | 3109 | 0.508 |
| Decision maker status ^4^ |  |  |  |  | 0.0046 | 3102 | 0.513 |
| Other | Ref | --- | --- | --- |  |  |  |
| Primary | -0.55 (0.195) | 0.576 | 0.347, 0.953 | 0.005 |  |  |  |
| **Animal Variables** |  |  |  |  |  |  |  |
| Age (per year) ^5^ |  |  |  |  | 0.0877 | 2962 | 0.642 |
| 1 to 5 | 0.36 (0.158) | 1.437 | 0.959, 2.163 | 0.022 |  |  |  |
| 6 to 20 | 3.01 (0.257) | 20.345 | 10.575, 39,824 | <0.001 |  |  |  |
| Breed size category |  |  |  |  | 0.0043 | 3126 | 0.531 |
| Medium | Ref | --- | --- | --- |  |  |  |
| Toy | 0.16 (0.282) | 1.176 | 0.550, 2.410 | 0.566 |  |  |  |
| Small | 0.29 (0.117) | 1.338 | 0.990, 1.807 | 0.013 |  |  |  |
| Large | 0.14 (0.106) | 1.155 | 0.891, 1.497 | 0.152 |  |  |  |
| Giant | 0.32 (0.214) | 1.371 | 0.784, 2.371 | 0.140 |  |  |  |
| Sex |  |  |  |  | 0.0001 | 3110 | 0.504 |
| Female | Ref | --- | --- | --- |  |  |  |
| Male | 0.03 (0.085) | 1.031 | 0.827, 1.285 | 0.725 |  |  |  |
| Neuter status |  |  |  |  | 0.0192 | 3077 | 0.550 |
| Sexually intact | Ref | --- | --- | --- |  |  |  |
| Neutered | 0.61 (0.110) | 1.848 | 1.398, 2.461 | <0.001 |  |  |  |
| Dog diet ^3^ |  |  |  |  | 0.0252 | 3082 | 0.571 |
| Conventional | Ref | --- | --- | --- |  |  |  |
| Raw | -0.54 (0.097) | 0.580 | 0.451, 0.745 | <0.001 |  |  |  |
| Vegetarian | 0.05 (0.344) | 1.051 | 0.423, 2.558 | 0.884 |  |  |  |
| Vegan | -0.62 (0.136) | 0.536 | 0.375, 0.757 | <0.001 |  |  |  |
| Dog on vegan diet ^3^ |  |  |  |  | 0.0065 | 3098 | 0.524 |
| No | Ref | --- | --- | --- |  |  |  |
| Yes | -0.43 (0.131) | 0.650 | 0.460, 0.907 | 0.001 |  |  |  |
| **Healthcare variables** |  |  |  |  |  |  |  |
| Veterinary visits |  |  |  |  | 0.2516 | 2657 | 0.746 |
| None | Ref | --- | --- | --- |  |  |  |
| 1 | 0.55 (0.160) | 1.735 | 1.162, 2.647 | <0.001 |  |  |  |
| 2 | 1.83 (0.165) | 6.255 | 4.131, 9.678 | <0.001 |  |  |  |
| 3 | 2.16 (0.201) | 8.713 | 5.245, 14.759 | <0.001 |  |  |  |
| 4 or more | 2.99 (0.196) | 19.867 | 12.139, 33.398 | <0.001 |  |  |  |
| Received medication |  |  |  |  | 0.3256 | 2473 | 0.760 |
| No | Ref | --- | --- | --- |  |  |  |
| Yes | 2.33 (0.100) | 10.272 | 7.969, 13.315 | <0.001 |  |  |  |
| Switched to therapeutic diet |  |  |  |  | 0.0489 | 3025 | 0.542 |
| No | Ref | --- | --- | --- |  |  |  |
| Yes | 1.95 (0.242) | 7.080 | 3.916, 13.781 | <0.001 |  |  |  |

Results presented are from simple (i.e., univariable) binary logistic regression, whereby each independent predictor variable is tested separately in a logistic regression model. These results were then used to determine the variables to include in subsequent multiple regression analysis, as shown in Fig 4 and S4 Table. Results are reported as estimates of regression coefficients (β) with its standard error in brackets, odds ratios and 99% confidence intervals (99%-CI). Model performance assessed using the coefficient of determination (pseudo-R^2^) based on the method reported by Nagelkerke [63], with pseudo-R^2^, the Bayesian information criterion (BIC [60,61]) and area under the receiver operating characteristic curve (AUC) for the test dataset. For BIC, models having the best fit have lower BIC values; n.b., BIC can only be compared within the same family of models. For AUC, values can range from 0 to 1; a model that performed no better than chance would have an AUC of 0.5, and models predicting better than by chance would have AUC >0.5, with an AUC of 1.0 suggesting perfect prediction. ^1^ Definitions of the different categories are given in the original study [15]. ^2^ Please note that the urban category in *setting* variable combines the ‘urban’ and ‘equally urban and rural categories’. ^3^ Please see the footnote to Table 1 for details of how owner and dog diets were assigned. ^4^ Decision maker status (primary vs. other) variable created from data about the role owners played in making decisions about choosing a diet for their dog; for this, the ‘primary decision maker’ category was classified as ‘primary’, whilst the other two categories (‘play no role’, n=15; ‘play some lesser role’, n=96) were classified as ‘other’. ^5^ Dog age analysed as a continuous variable with B-splines, utilising boundary knots and an internal knot at the median value (6 years); therefore, odds ratios represent are the effect per year for each side of that knot.
