# Supplementary material for: Variables associated with owner perceptions of the health of their dog: Further analysis of data from a large international survey
Source: PLoS One. 2024 May 15;19(5):e0280173. doi: 10.1371/journal.pone.0280173 (PMC11095744; doi:10.1371/journal.pone.0280173)
Supplement: S2 Table — (DOCX) [file pone.0280173.s010.docx]

**S2 Table. Results of simple (i.e., univariable) binary logistic regression analyses examining associations between owner, animal and veterinary variables and the *significant illness* binary (the presence of any illness as reported by the owner) for all owners.**

| **Variable ^1^** | **Estimate** | **Odds ratio** | **99%-CI** | ***P*-value** | **Pseudo-R^2^** | **BIC** | **AUC** |
| --- | --- | --- | --- | --- | --- | --- | --- |
| **Owner variables** |  |  |  |  |  |  |  |
| Location |  |  |  |  | 0.0054 | 1042 | 0.542 |
| United Kingdom | Ref | --- | --- | --- |  |  |  |
| Other European country | 0.25 (0.243) | 1.287 | 0.663, 2.340 | 0.299 |  |  |  |
| North America | 0.46 (0.348) | 1.589 | 0.583, 3.611 | 0.183 |  |  |  |
| Australia / New Zealand / Oceania | 0.62 (0.367) | 1.854 | 0.639, 4.393 | 0.092 |  |  |  |
| Other region | 0.26 (0.476) | 1.302 | 0.299, 3.796 | 0.579 |  |  |  |
| Setting ^2^ |  |  |  |  |  |  |  |
| Urban | Ref | --- | --- | --- | 0.0005 | 1023 | 0.514 |
| Rural | 0.12 (0.185) | 1.130 | 0.694, 1.811 | 0.509 |  |  |  |
| Owner age (years) |  |  |  |  | 0.0029 | 1044 | 0.537 |
| <30 | Ref | --- | --- | --- |  |  |  |
| 30-39 | 0.36 (0.293) | 1.429 | 0.683, 3.144 | 0.224 |  |  |  |
| 40-49 | 0.03 (0.315) | 1.027 | 0.457, 2.363 | 0.932 |  |  |  |
| 50-59 | 0.18 (0.299) | 1.202 | 0.563, 2.677 | 0.540 |  |  |  |
| ≥60 | 0.04 (0.321) | 1.044 | 0.455, 2.432 | 0.894 |  |  |  |
| Owner gender |  |  |  |  | 0.0006 | 1023 | 0.508 |
| Female | Ref | --- | --- | --- |  |  |  |
| Male | -0.24 (0.374) | 0.783 | 0.258, 1.848 | 0.513 |  |  |  |
| Education |  |  |  |  | 0.0028 | 1036 | 0.532 |
| Basic or high school | Ref | --- | --- | --- |  |  |  |
| College | -0.28 (0.275) | 0.754 | 0.371, 1.551 | 0.305 |  |  |  |
| Graduate | 0.04 (0.257) | 1.042 | 0.543, 2.065 | 0.872 |  |  |  |
| Postgraduate | 0.04 (0.270) | 1.036 | 0.517, 2.110 | 0.896 |  |  |  |
| Income |  |  |  |  | 0.0013 | 1030 | 0.521 |
| Low | Ref | --- | --- | --- |  |  |  |
| Medium | -0.11 (0.241) | 0.898 | 0.497, 1.736 | 0.655 |  |  |  |
| High | -0.33 (0.335) | 0.717 | 0.293, 1.682 | 0.319 |  |  |  |
| Animal-related career |  |  |  |  | 0.0034 | 1020 | 0.530 |
| No | Ref | --- | --- | --- |  |  |  |
| Yes | 0.36 (0.211) | 1.434 | 0.813, 2.422 | 0.087 |  |  |  |
| Owner diet ^3^ |  |  |  |  | 0.0062 | 1041 | 0.555 |
| Omnivore | Ref | --- | --- | --- |  |  |  |
| Omnivore (restricted) | 0.41 (0.237) | 1.508 | 0.811, 2.764 | 0.083 |  |  |  |
| Pescatarian | 0.35 (0.398) | 1.424 | 0.449, 3.643 | 0.374 |  |  |  |
| Vegetarian | 0.54 (0.291) | 1.713 | 0.777, 3.530 | 0.064 |  |  |  |
| Vegan | 0.14 (0.249) | 1.147 | 0.593, 2.162 | 0.249 |  |  |  |
| Owner on vegan diet ^3^ |  |  |  |  | 0.0002 | 1023 | 0.508 |
| No | Ref | --- | --- | --- |  |  |  |
| Yes | -0.09 (0.219) | 0.910 | 0.502, 1.562 | 0.667 |  |  |  |
| Decision maker status ^4^ |  |  |  |  | 0.0015 | 1022 | 0.511 |
| Other | Ref | --- | --- | --- |  |  |  |
| Primary | -0.41 (0.360) | 0.662 | 0.259, 1.896 | 0.252 |  |  |  |
| **Animal Variables** |  |  |  |  |  |  |  |
| Age (per year) ^5^ |  |  |  |  | 0.0677 | 975 | 0.677 |
| 1 to 6 years | 0.54 (0.388) | 1.720 | 0.657, 4.905 | 0.162 |  |  |  |
| 6 to 20 years | 3.26 (0.427) | 25.962 | 8.678, 79.192 | <0.001 |  |  |  |
| Giant breed ^6^ |  |  |  |  | 0.0038 | 1020 | 0.518 |
| No | Ref | --- | --- | --- |  |  |  |
| Yes | 0.66 (0.346) | 1.929 | 0.710, 4.361 | 0.058 |  |  |  |
| Sex |  |  |  |  | 0.0002 | 1023 | 0.509 |
| Female | Ref | --- | --- | --- |  |  |  |
| Male | -0.07 (0.180) | 0.930 | 0.585, 1.483 | 0.689 |  |  |  |
| Neuter status |  |  |  |  | 0.0062 | 1018 | 0.540 |
| Sexually intact | Ref | --- | --- | --- |  |  |  |
| Neutered | 0.54 (0.253) | 1.711 | 0.928, 3.466 | 0.034 |  |  |  |
| Dog diet ^3^ |  |  |  |  | 0.0169 | 1025 | 0.583 |
| Conventional | Ref | --- | --- | --- |  |  |  |
| Raw | -0.66 (0.222) | 0.514 | 0.281, 0.891 | 0.003 |  |  |  |
| Vegetarian | 0.53 (0.543) | 1.695 | 0.308, 5.751 | 0.543 |  |  |  |
| Vegan | -0.65 (0.315) | 0.524 | 0.213, 1.104 | 0.039 |  |  |  |
| Dog on vegan diet ^2^ |  |  |  |  | 0.0030 | 1021 | 0.523 |
| No | Ref | --- | --- | --- |  |  |  |
| Yes | -0.46 (0.309) | 0.633 | 0.260, 1.311 | 0.139 |  |  |  |
| **Healthcare variables** |  |  |  |  |  |  |  |
| Veterinary visits |  |  |  |  | 0.2666 | 818 | 0.833 |
| None | Ref | --- | --- | --- |  |  |  |
| 1 | -0.11 (0.551) | 0.899 | 0.229, 4.427 | 0.846 |  |  |  |
| 2 | 1.03 (0.513) | 2.802 | 0.833, 12.985 | 0.045 |  |  |  |
| 3 | 1.95 (0.520) | 7.009 | 2.035, 32.877 | <0.001 |  |  |  |
| 4 or more | 3.42 (0.468) | 30.687 | 10.824, 131.903 | <0.001 |  |  |  |
| Received medication |  |  |  |  | 0.2057 | 849 | 0.780 |
| No | Ref | --- | --- | --- |  |  |  |
| Yes | 3.13 (0.348) | 22.931 | 10.291, 64.206 | <0.001 |  |  |  |
| Switched to therapeutic diet |  |  |  |  | 0.0466 | 985 | 0.578 |
| No | Ref | --- | --- | --- |  |  |  |
| Yes | 1.75 (0.248) | 5.773 | 2.959, 10.712 | <0.001 |  |  |  |

Results presented are from simple (i.e., univariable) binary logistic regression, whereby each independent predictor variable is tested separately in a logistic regression model. These results were then used to determine the variables to include in subsequent multiple regression analysis, as shown in Fig 4 and S4 Table. Results are reported as estimates of regression coefficients (β) with its standard error in brackets, odds ratios and 99% confidence intervals (99%-CI). Model performance assessed using the coefficient of determination (pseudo-R^2^) based on the method reported by Nagelkerke [63], with pseudo-R^2^, the Bayesian information criterion (BIC [60,61]) and area under the receiver operating characteristic curve (AUC) for the test dataset. For BIC, models having the best fit have lower BIC values; n.b., BIC can only be compared within the same family of models. For AUC, values can range from 0 to 1; a model that performed no better than chance would have an AUC of 0.5, and models predicting better than by chance would have AUC >0.5, with an AUC of 1.0 suggesting perfect prediction. ^1^ Definitions of the different categories are given in the original study [15]. ^2^ Please note that the urban category in *setting* variable combines the ‘urban’ and ‘equally urban and rural categories’. ^3^ Please see the footnote to Table 1 for details of how owner and dog diets were assigned. ^4^ Decision maker status (primary vs. other) variable created from data about the role owners played in making decisions about choosing a diet for their dog; for this, the ‘primary decision maker’ category was classified as ‘primary’, whilst the other two categories (‘play no role’, n=15; ‘play some lesser role’, n=96) were classified as ‘other’. ^5^ Dog age analysed as a continuous variable with B-splines, utilising boundary knots and an internal knot at the median value (6 years); therefore, odds ratios represent are the effect per year for each side of that knot. ^6^ Please note that breed was better categorised using the *breed size category* variable for regression models using the *significant illness* outcome.
