# Supplementary material for: Variables associated with owner perceptions of the health of their dog: Further analysis of data from a large international survey
Source: PLoS One. 2024 May 15;19(5):e0280173. doi: 10.1371/journal.pone.0280173 (PMC11095744; doi:10.1371/journal.pone.0280173)
Supplement: S5 Table — (DOCX) [file pone.0280173.s013.docx]

**S5 Table.**

Best-fit multiple binary logistic regression models examining associations between owner, animal and veterinary variables and the *any health problem* binary outcome variable, as reported by owners who were primary decision makers.

| **Variable ^1^** | **Estimate ^2^** | **Odds ratio** | **99% confidence interval** | ***P*-value** | **Pseudo-R^2^** | **BIC** | **AUC** |
| --- | --- | --- | --- | --- | --- | --- | --- |
| Model 1 |  |  |  |  | 0.4195 | 1656 | 0.842 |
| Age (per year) ^2^ |  |  |  |  |  |  |  |
| 1 to 6 years | 0.74 (0.229) | 2.085 | 1.162, 3.791 | 0.001 |  |  |  |
| 6 to 20 years | 3.09 (0.342) | 21.948 | 9.178, 53.652 | <0.001 |  |  |  |
| Visits |  |  |  |  |  |  |  |
| None | Ref | --- | --- | --- |  |  |  |
| 1 | 0.17 (0.202) | 1.182 | 0.710, 2.014 | 0.408 |  |  |  |
| 2 | 1.07 (0.221) | 2.926 | 1.671, 5.226 | <0.001 |  |  |  |
| 3 | 0.99 (0.278) | 2.694 | 1.326, 9.847 | <0.001 |  |  |  |
| 4 or more | 1.57 (0.272) | 4.819 | 2.415, 0.796 | 0.001 |  |  |  |
| Received medication |  |  |  |  |  |  |  |
| No | Ref | --- | --- | --- |  |  |  |
| Yes | 1.81 (0.139) | 6.123 | 4.294, 8.796 | <0.001 |  |  |  |
| Switched to therapeutic food |  |  |  |  |  |  |  |
| No | Ref | --- | --- | --- |  |  |  |
| Yes | 0.94 (0.354) | 2.567 | 1.071, 6.731 | 0.008 |  |  |  |
| Model 2 |  |  |  |  | 0.4216 | 1660 | 0.845 |
| Age (per year) ^2^ |  |  |  |  |  |  |  |
| 1 to 6 years | 0.75 (0.230) | 2.127 | 1.184, 3.870 | 0.001 |  |  |  |
| 6 to 20 years | 3.18 (0.347) | 24.067 | 9.958, 59.530 | <0.001 |  |  |  |
| Visits |  |  |  |  |  |  |  |
| None | Ref | --- | --- | --- |  |  |  |
| 1 | 0.17 (0.202) | 1.189 | 0.715, 2.026 | 0.390 |  |  |  |
| 2 | 1.08 (0.221) | 2.945 | 1.682, 5.263 | <0.001 |  |  |  |
| 3 | 1.01 (0.278) | 2.732 | 1.342, 5,649 | <0.001 |  |  |  |
| 4 or more | 1.56 (0.273) | 4.761 | 2.383, 9.738 | <0.001 |  |  |  |
| Received medication |  |  |  |  |  |  |  |
| No | Ref | --- | --- | --- |  |  |  |
| Yes | 1.80 (0.139) | 6.048 | 4.237, 8.695 | <0.001 |  |  |  |
| Switched to therapeutic food |  |  |  |  |  |  |  |
| No | Ref | --- | --- | --- |  |  |  |
| Yes | 0.96 (0.354) | 2.602 | 1.087, 6.811 | 0.007 |  |  |  |
| Dog on vegan diet |  |  |  |  |  |  |  |
| No | Ref | --- | --- | --- |  |  |  |
| Yes | -0.38 (0.195) | 0.687 | 0.412, 1.128 | 0.055 |  |  |  |
| Model 3 |  |  |  |  |  |  |  |
| Age (per year) ^3^ |  |  |  |  | 0.4212 | 1660 | 0.844 |
| 1 to 6 years | 0.76 (0.230) | 2.131 | 1.185, 3.881 | 0.001 |  |  |  |
| 6 to 20 years | 3.19 (0.348) | 24.324 | 10.019, 60.459 | <0.001 |  |  |  |
| Visits |  |  |  |  |  |  |  |
| None | Ref | --- | --- | --- |  |  |  |
| 1 | 0.17 (0.202) | 1.186 | 0.712, 2.022 | 0.394 |  |  |  |
| 2 | 1.08 (0.221) | 2.933 | 1.674, 5.244 | <0.001 |  |  |  |
| 3 | 1.00 (0.278) | 2.713 | 1.333, 5,606 | <0.001 |  |  |  |
| 4 or more | 1.57 (0.273) | 4.785 | 2.396, 9.786 | <0.001 |  |  |  |
| Received medication |  |  |  |  |  |  |  |
| No | Ref | --- | --- | --- |  |  |  |
| Yes | 1.81 (0.139) | 6.088 | 4.266, 8.753 | <0.001 |  |  |  |
| Switched to therapeutic food |  |  |  |  |  |  |  |
| No | Ref | --- | --- | --- |  |  |  |
| Yes | 0.94 (0.354) | 2.564 | 1.070, 6.726 | 0.008 |  |  |  |
| Owner on vegan diet |  |  |  |  |  |  |  |
| No | Ref | --- | --- | --- |  |  |  |
| Yes | -0.27 (0.156) | 0.761 | 0.507, 1.134 | 0.081 |  |  |  |

Results presented are from simple (i.e., univariable) binary logistic regression, whereby each independent predictor variable is tested separately in a logistic regression model. These results were then used to determine the variables to include in subsequent multiple regression analysis, as shown in Fig 4 and S4 Table. ^1^ Definitions of the different categories are given in the original study [15]. ^2^ The estimate of the regression coefficient (β) with its standard error in brackets. ^3^ Dog age analysed as a continuous variable with B-splines, with a single interior knot at 6 years; therefore, odds ratios represent are the effect per year for each side of that knot. Pseudo-R^2^: coefficient of determination based on the method reported by Nagelkerke [62]. BIC: Bayesian information criterion, with models having the best fit having lower BIC [58,59]; BIC values can only be compared within the same family of models. AUC: area under the receiver-operating characteristic curve for the test dataset, used as a measure of prediction accuracy; values can range from 0 to 1; a model that performed no better than chance would have an AUC of 0.5, and models predicting better than by chance would have AUC >0.5, with an AUC of 1.0 suggesting perfect prediction.
