# Supplementary material for: Variables associated with owner perceptions of the health of their dog: Further analysis of data from a large international survey
Source: PLoS One. 2024 May 15;19(5):e0280173. doi: 10.1371/journal.pone.0280173 (PMC11095744; doi:10.1371/journal.pone.0280173)
Supplement: S6 Table — (DOCX) [file pone.0280173.s014.docx]

**S6 Table.**

Best-fit multiple binary logistic regression models examining associations between owner, animal and veterinary variables and the *significant illness* binary variable, as reported by owners who were primary decision makers.

| **Variable ^1^** | **Estimate ^2^** | **Odds ratio** | **99% confidence interval** | ***P*-value** | **Pseudo-R^2^** | **BIC** | **AUC** | |
| --- | --- | --- | --- | --- | --- | --- | --- | --- |
| Model 1 |  |  |  |  | 0.3417 | 517 | 0.903 | |
| Age (per year) ^2^ |  |  |  |  |  |  |  | |
| 1 to 6 years | 0.51 (0.519) | 1.672 | 0.463, 6.901 | 0.322 |  |  |  | |
| 6 to 20 years | 2.72 (0.592) | 15.208 | 3.359, 72.565 | <0.001 |  |  |  | |
| Visits |  |  |  |  |  |  |  | |
| None | Ref | --- | --- | --- |  |  |  | |
| 1 | -0.65 (0.721) | 0.523 | 0.091, 4.638 | 0.368 |  |  |  | |
| 2 | -0.35 (0.730) | 0.706 | 0.124, 6.420 | 0.634 |  |  |  | |
| 3 | -0.11 (0.784) | 0.900 | 0.129, 8.934 | 0.893 |  |  |  | |
| 4 or more | 1.64 (0.702) | 5.146 | 1.007, 44.794 | 0.020 |  |  |  | |
| Received medication |  |  |  |  |  |  |  | |
| No | Ref | --- | --- | --- |  |  |  | |
| Yes | 2.37 (0.503) | 10.709 | 3.349, 47.086 | <0.001 |  |  |  | |
| Model 2 |  |  |  |  | 0.3419 | 524 | 0.904 |  |
| Age (per year) ^3^ |  |  |  |  |  |  |  | |
| 1 to 6 years | 0.52 (0.520) | 1.684 | 0.465, 6.960 | 0.316 |  |  |  | |
| 6 to 20 years | 2.74 (0.596) | 15.562 | 3.402, 75.019 | <0.001 |  |  |  | |
| Visits |  |  |  |  |  |  |  | |
| None | Ref | --- | --- | --- |  |  |  | |
| 1 | -0.65 (0.722) | 0.520 | 0.090, 4.625 | 0.366 |  |  |  | |
| 2 | -0.36 (0.733) | 0.697 | 0.121, 6.358 | 0.622 |  |  |  | |
| 3 | -0.11 (0.785) | 0.892 | 0.128, 8.888 | 0.885 |  |  |  | |
| 4 or more | 1.62 (0.705) | 5.045 | 0.972, 44.165 | 0.022 |  |  |  | |
| Received medication |  |  |  |  |  |  |  | |
| No | Ref | --- | --- | --- |  |  |  | |
| Yes | 2.37 (0.504) | 10.720 | 3.343, 47.331 | <0.001 |  |  |  | |
| Dog on vegan diet |  |  |  |  |  |  |  | |
| No | Ref | --- | --- | --- |  |  |  | |
| Yes | -0.15 (0.444) | 0.862 | 0.237, 2.470 | 0.738 |  |  |  | |
| Model 3 |  |  |  |  | 0.3420 | 524 | 0.903 |  |
| Age (per year) ^2^ |  |  |  |  |  |  |  | |
| 1 to 6 years | 0.50 (0.520) | 1.652 | 0.456, 6.831 | 0.334 |  |  |  | |
| 6 to 20 years | 2.68 (0.599) | 14.654 | 3.178, 71.231 | <0.001 |  |  |  | |
| Visits |  |  |  |  |  |  |  | |
| None | Ref | --- | --- | --- |  |  |  | |
| 1 | -0.65 (0.720) | 0.521 | 0.090, 4.619 | 0.365 |  |  |  | |
| 2 | -0.34 (0.729) | 0.711 | 0.125, 6.450 | 0.640 |  |  |  | |
| 3 | -0.10 (0.783) | 0.902 | 0.130, 8.947 | 0.895 |  |  |  | |
| 4 or more | 1.65 (0.701) | 5.203 | 1.018, 45.247 | 0.019 |  |  |  | |
| Received medication |  |  |  |  |  |  |  | |
| No | Ref | --- | --- | --- |  |  |  | |
| Yes | 2.37 (0.502) | 10.740 | 3.349, 47.114 | <0.001 |  |  |  | |
| Owner on vegan diet |  |  |  |  |  |  |  | |
| No | Ref | --- | --- | --- |  |  |  | |
| Yes | 0.12 (0.312) | 1.130 | 0.486, 2.463 | 0.695 |  |  |  | |

Results presented are from simple (i.e., univariable) binary logistic regression, whereby each independent predictor variable is tested separately in a logistic regression model. These results were then used to determine the variables to include in subsequent multiple regression analysis, as shown in Fig 4 and S4 Table. ^1^ Definitions of the different categories are given in the original study [15]. ^2^ The estimate of the regression coefficient (β) with its standard error in brackets. ^3^ Dog age analysed as a continuous variable with B-splines, with a single knot at 6 years; therefore, odds ratios represent are the effect per year for each side of that knot. Pseudo-R^2^: coefficient of determination based on the method reported by Nagelkerke [62]. BIC: Bayesian information criterion, with models having the best fit having lower BIC [58,59]; BIC values can only be compared within the same family of models. AUC: area under the receiver-operating characteristic curve for the test dataset, used as a measure of prediction accuracy; values can range from 0 to 1; a model that performed no better than chance would have an AUC of 0.5, and models predicting better than by chance would have AUC >0.5, with an AUC of 1.0 suggesting perfect prediction.
