# Supplementary material for: Variables associated with owner perceptions of the health of their dog: Further analysis of data from a large international survey
Source: PLoS One. 2024 May 15;19(5):e0280173. doi: 10.1371/journal.pone.0280173 (PMC11095744; doi:10.1371/journal.pone.0280173)
Supplement: S2 File — (HTML) [file pone.0280173.s016.html]

Read Data 102 - include all owners not just decision makers


# Read Data 102 - include all owners not just decision makers

Data from here: https://osf.io/nbepu
Original methods and full paper here: https://journals.plos.org/plosone/article?id=10.1371/journal.pone.0265662

# Load required packages

```
library(stringr)
library(dplyr)
library(DescTools)
library(car)
require(foreign)
require(ggplot2)
require(Hmisc)
require(reshape2)
library(tidyverse)
library(broom)
library(aod)
library(glmtoolbox)
library(fmsb)
library(umap)
library(ggthemes)
library(readxl) #leaves characters as characters
library(ROCR) # Use ROCR package to plot ROC curve & AUC
library(pROC)
library(caret)
library(xgboost)
library(smotefamily) # one method of ML data augmentation
library(rsample)
library(mltools) # Wrapper allows UMAP calculations
library(data.table)
library(heatmaply)
library(rstatix) # to calculate Kendall correlation with associated p-values.
library(IHW) # Bioconductor package to calculate BH threshold
library(dbscan)
library(corrplot)
library(showtext)
knitr::opts_chunk$set(tidy.opts = list(width.cutoff = 60), tidy = TRUE)
library(splines)
library(splines)
library(cluster)
library(factoextra)
library(clusterSim)
```

# Open Excel file

```
# Save the data so can run subsequent analysis ad lib
SAVE = TRUE

# quicker version readxl
ml <- read_excel("Canine health results.xlsx", sheet = "All")
cols <- c("Col_a")
for (col in 2:ncol(ml)) {
    if (col <= 26) {
        cols <- cbind(cols, paste0("Col_", letters[col]))
    } else if (col <= 52) {
        cols <- cbind(cols, paste0("Col_a", letters[col - 26]))
    } else {
        cols <- cbind(cols, paste0("Col_b", letters[col - 52]))
    }
}
names(ml) <- cols
```

# Rename variables

```
ml <- dplyr::rename(ml, unique.resp.number = Col_a)
ml <- dplyr::rename(ml, Location = Col_c)
ml <- dplyr::rename(ml, setting = Col_e)
ml <- dplyr::rename(ml, Education = Col_g)
ml <- dplyr::rename(ml, Animal_Career = Col_h)
ml <- dplyr::rename(ml, Income = Col_i)
ml <- dplyr::rename(ml, C_Age = Col_j)
ml <- dplyr::rename(ml, C_Gender = Col_k)
ml <- dplyr::rename(ml, C_Diet = Col_l)
ml <- dplyr::rename(ml, Size = Col_o)
ml <- dplyr::rename(ml, D_Age = Col_p)
ml <- dplyr::rename(ml, D_Gender = Col_r)
ml <- dplyr::rename(ml, Therapeutic_Food = Col_s)
ml <- dplyr::rename(ml, Visits = Col_t)
ml <- dplyr::rename(ml, Vet_Health = Col_u)
ml <- dplyr::rename(ml, Meds = Col_ag)
ml <- dplyr::rename(ml, Health = Col_ah)
ml <- dplyr::rename(ml, D_Diet = Col_ai)
ml <- dplyr::rename(ml, Decision_Maker = Col_at)
head(ml)
```

# Start by creating separate Sex and neuter status variables

```
ml$D_Sex <- rep("Male", nrow(ml))
for (i in 1:nrow(ml)) {
    if (grepl("Male", ml$D_Gender[i], fixed = TRUE)) {
        ml$D_Sex[i] <- "Male"
    } else {
        ml$D_Sex[i] <- "Female"
    }
}

# Checks and Balances
dplyr::count(ml, D_Sex)
```

```
ml$D_Neuter <- rep("BLANK", nrow(ml))
for (i in 1:nrow(ml)) {
    if (grepl("intact", ml$D_Gender[i], fixed = TRUE)) {
        ml$D_Neuter[i] <- "Intact"
    } else {
        ml$D_Neuter[i] <- "Neutered"
    }
}
dplyr::count(ml, D_Neuter)
```

# check D\_Age

```
dplyr::count(ml, D_Age)
```

# Dog Age remove <1 years and unsure, then change to numeric

```
ml <- subset(ml, D_Age != "<1" & D_Age != "Unsure")
ml$D_Age <- as.numeric(ml$D_Age)
dplyr::count(ml, D_Age)
```

# Create ml data frame

```
ml <- dplyr::select(ml, c(unique.resp.number, Location, setting,
    Education, Animal_Career, Income, C_Age, C_Gender, C_Diet,
    Size, D_Age, D_Gender, D_Sex, D_Neuter, Therapeutic_Food,
    Visits, Vet_Health, Meds, Health, D_Diet, Decision_Maker))

# check columns
head(ml)
```

# Categorise Dog Age into quintiles

```
ml <- subset(ml, D_Age != "NA")
ml$D_Age_quant <- cut(ml$D_Age, breaks = quantile(ml$D_Age, probs = seq(0,
    1, 0.2)), na.rm = TRUE, include.lowest = TRUE)
dplyr::count(ml, D_Age_quant)
```

# Check Sex

```
ml$D_Sex <- as.factor(ml$D_Sex)
# Checks and Balances
dplyr::count(ml, D_Sex)
```

# Check Neuter

```
ml$D_Neuter <- as.factor(ml$D_Neuter)
# Checks and Balances
dplyr::count(ml, D_Neuter)
```

# Check Location

```
dplyr::count(ml, Location)
```

# combine smaller groups into logical categories

```
for (i in 1:nrow(ml)) {
    if (grepl("UK", ml$Location[i], fixed = TRUE)) {
        ml$Location[i] <- "UK"
    } else if (grepl("Other European", ml$Location[i], fixed = TRUE)) {
        ml$Location[i] <- "Other European"
    } else if (grepl("North America", ml$Location[i], fixed = TRUE)) {
        ml$Location[i] <- "North America"
    } else if (grepl("Australia/New Zealand/Oceania", ml$Location[i],
        fixed = TRUE)) {
        ml$Location[i] <- "Australia/New Zealand/Oceania"
    } else {
        ml$Location[i] <- "Other"
    }
}
ml$Location <- factor(ml$Location, levels = c("UK", "Other European",
    "North America", "Australia/New Zealand/Oceania", "Other"))
dplyr::count(ml, Location)
```

# check setting

```
dplyr::count(ml, setting)
```

# Not much point including data where setting is “other”

```
ml <- subset(ml, setting != "Other" | setting != NA)
ml$setting <- as.factor(ml$setting)
dplyr::count(ml, setting)
```

# Reorder SETTING and check

```
# reorder categories so Urban is REF
ml$setting <- factor(ml$setting, levels = c("Urban", "Rural",
    "Equally urban and rural"))
dplyr::count(ml, setting)
```

# create binary for URBAN

```
# Create a binary URBAN variable
ml$Urban <- ml$setting
ml$Urban <- as.character(ml$Urban)
for (i in 1:nrow(ml)) {
    if (grepl("Urban", ml$Urban[i], fixed = TRUE)) {
        ml$Urban[i] <- "Yes"
    } else {
        ml$Urban[i] <- "No"
    }
}
dplyr::count(ml, Urban)
```

# Check Education

```
dplyr::count(ml, Education)
```

# Simplify education and order categories

```
Qualifications = c("0_Basic", "1_HighSchool", "2_College", "3_Grad",
    "4_PG", "5_PhD")
ml$Education <- str_replace(ml$Education, "Did not complete high school",
    "0_Basic")
ml$Education <- str_replace(ml$Education, "High school or equivalent",
    "1_HighSchool")
ml$Education <- str_replace(ml$Education, "College or University award lower than undergraduate degree",
    "2_College")
ml$Education <- str_replace(ml$Education, "University undergraduate degree",
    "3_Grad")
ml$Education <- str_replace(ml$Education, "University postgraduate degree lower than doctoral degree e.g. masters",
    "4_PG")
ml$Education <- str_replace(ml$Education, "Doctoral degree",
    "5_PhD")
ml$Education <- ordered(ml$Education)
dplyr::count(ml, Education)
```

#Simplify education further as small group sizes

```
Qualifications_S = c("0_Basic_or_HighSchool", "1_College", "2_Grad",
    "3_PG_or_PhD")
ml$Education_S <- ml$Education
ml$Education_S <- str_replace(ml$Education_S, "0_Basic", "0_Basic_or_HighSchool")
ml$Education_S <- str_replace(ml$Education_S, "1_HighSchool",
    "0_Basic_or_HighSchool")
ml$Education_S <- str_replace(ml$Education_S, "2_College", "1_College")
ml$Education_S <- str_replace(ml$Education_S, "3_Grad", "2_Grad")
ml$Education_S <- str_replace(ml$Education_S, "4_PG", "3_PG_or_PhD")
ml$Education_S <- str_replace(ml$Education_S, "5_PhD", "3_PG_or_PhD")
dplyr::count(ml, Education_S)
```

# Try non-ordered categories for Education\_S

```
ml$Education_S2 <- factor(ml$Education_S, ordered = FALSE)
dplyr::count(ml, Education_S2)
```

# check Animal\_Career variable

```
dplyr::count(ml, Animal_Career)
```

# create animal career variable where vet combined

```
ml$Animal_Career2 <- ml$Animal_Career
for (i in 1:nrow(ml)) {
    if (grepl("Vet", ml$Animal_Career2[i], fixed = TRUE)) {
        ml$Animal_Career2[i] <- "Vet professional"
    } else if (grepl("Animal breeder", ml$Animal_Career2[i], fixed = TRUE)) {
        ml$Animal_Career2[i] <- "breeder/trainer"
    } else if (grepl("Animal trainer", ml$Animal_Career2[i], fixed = TRUE)) {
        ml$Animal_Career2[i] <- "breeder/trainer"
    } else if (grepl("Pet industry worker", ml$Animal_Career2[i],
        fixed = TRUE)) {
        ml$Animal_Career2[i] <- "Pet industry worker"
    } else {
        ml$Animal_Career2[i] <- "None of the above"
    }
}
# reorder categories so None of the above is REF
ml$Animal_Career2 <- factor(ml$Animal_Career2, levels = c("None of the above",
    "Vet professional", "breeder/trainer", "Pet industry worker"))
dplyr::count(ml, Animal_Career2)
```

# create binary variable for animal career as ensures better balance

```
ml$Animal_Career_BINARY <- ml$Animal_Career

for (i in 1:nrow(ml)) {
    if (grepl("None of the above", ml$Animal_Career_BINARY[i],
        fixed = TRUE)) {
        ml$Animal_Career_BINARY[i] <- "No"
    } else {
        ml$Animal_Career_BINARY[i] <- "Yes"
    }
}
dplyr::count(ml, Animal_Career_BINARY)
```

# Check Income

```
dplyr::count(ml, Income)
```

# Two choice with Income; treat as factor or drop “prefer not to say” and treat as ordinal.

```
ml <- subset(ml, Income != "Prefer not to answer" | Income !=
    NA)
ml$Income <- ordered(ml$Income, levels = c("Low", "Medium", "High"))
dplyr::count(ml, Income)
```

# Try non-ordered categories for Income

```
ml$Income2 <- factor(ml$Income, ordered = FALSE)
dplyr::count(ml, Income2)
```

# Check C\_Age

```
dplyr::count(ml, C_Age)
```

# combine smallest categories and reorder

```
ml$C_Age <- str_replace(ml$C_Age, "18-19", "<30")
ml$C_Age <- str_replace(ml$C_Age, "20–29", "<30")
ml$C_Age <- str_replace(ml$C_Age, ">70", "60<")
ml$C_Age <- str_replace(ml$C_Age, "60–69", "60<")
ml$C_Age <- ordered(ml$C_Age)
# Check right order
ml$C_Age[nrow(ml)]
```

```
## [1] 60<
## Levels: <30 < 30–39 < 40–49 < 50–59 < 60<
```

```
dplyr::count(ml, C_Age)
```

# Try non-ordered categories for c\_Age

```
ml$C_Age2 <- factor(ml$C_Age, ordered = FALSE)
dplyr::count(ml, C_Age2)
```

# Check C\_Gender

```
dplyr::count(ml, C_Gender)
```

# Given small numbers in some categories, create male/female subset for C\_Gender

```
ml <- subset(ml, C_Gender == "Male" | C_Gender == "Female")
ml$C_Gender <- as.factor(ml$C_Gender)
dplyr::count(ml, C_Gender)
```

# Check C\_Diet

```
dplyr::count(ml, C_Diet)
```

# Not much point including other category as small numbers

```
ml <- subset(ml, C_Diet != "Other" | C_Diet != NA)
dplyr::count(ml, C_Diet)
```

# create binary C\_Vegan variable

```
ml$C_Diet_Vegan <- ml$C_Diet
for (i in 1:nrow(ml)) {
    if (grepl("Vegan", ml$C_Diet_Vegan[i], fixed = TRUE)) {
        ml$C_Diet_Vegan[i] <- "Yes"
    } else {
        ml$C_Diet_Vegan[i] <- "No"
    }
}
dplyr::count(ml, C_Diet_Vegan)
```

# create binary C\_Vegan\_Veggie variable

```
ml$C_Diet_Vegan_Veggie <- ml$C_Diet
for (i in 1:nrow(ml)) {
    if (grepl("Veg", ml$C_Diet_Vegan_Veggie[i], fixed = TRUE)) {
        ml$C_Diet_Vegan_Veggie[i] <- "Yes"
    } else {
        ml$C_Diet_Vegan_Veggie[i] <- "No"
    }
}
dplyr::count(ml, C_Diet_Vegan_Veggie)
```

# reorder and rename size categories

```
for (i in 1:nrow(ml)) {
    if (grepl("Toy", ml$Size[i], fixed = TRUE)) {
        ml$Size[i] <- "Toy"
    } else if (grepl("Small", ml$Size[i], fixed = TRUE)) {
        ml$Size[i] <- "Small"
    } else if (grepl("Medium", ml$Size[i], fixed = TRUE)) {
        ml$Size[i] <- "Medium"
    } else if (grepl("Large", ml$Size[i], fixed = TRUE)) {
        ml$Size[i] <- "Large"
    } else {
        ml$Size[i] <- "Giant"
    }
}
ml$Size <- ordered(ml$Size, levels = c("Toy", "Small", "Medium",
    "Large", "Giant"))
dplyr::count(ml, Size)
```

# Try non-ordered size categories with medium as reference

```
ml$Size2 <- factor(ml$Size, levels = c("Medium", "Toy", "Small",
    "Large", "Giant"), ordered = FALSE)
dplyr::count(ml, Size2)
```

# create binary Giant breed variable

```
Giant = c("Yes", "No")
ml$Size_Giant <- ml$Size2
ml$Size_Giant <- str_replace(ml$Size_Giant, "Giant", "Yes")
ml$Size_Giant <- str_replace(ml$Size_Giant, "Toy", "No")
ml$Size_Giant <- str_replace(ml$Size_Giant, "Medium", "No")
ml$Size_Giant <- str_replace(ml$Size_Giant, "Small", "No")
ml$Size_Giant <- str_replace(ml$Size_Giant, "Large", "No")
dplyr::count(ml, Size_Giant)
```

# create theraeputic food category

```
ml$Therapeutic_Food <- as.character(ml$Therapeutic_Food)
ml <- subset(ml, Therapeutic_Food != "NA" | Therapeutic_Food !=
    NA)
dplyr::count(ml, Therapeutic_Food)
```

# check visit data

```
ml <- subset(ml, Visits != "NA")
dplyr::count(ml, Visits)
```

# Not much point including data where clients are unsure of visit numbers.

```
ml <- subset(ml, Visits != "Unsure" | Visits != NA)
ml <- subset(ml, Visits != "NA" | Visits != NA)
dplyr::count(ml, Visits)
```

# rename and reorder Visits

```
ml$Visits <- str_replace(ml$Visits, ">3", "3<")
ml$Visits <- str_replace(ml$Visits, "0", "0")
ml$Visits <- str_replace(ml$Visits, "1", "1")
ml$Visits <- str_replace(ml$Visits, "2", "2")
ml$Visits <- str_replace(ml$Visits, "3", "3")
dplyr::count(ml, ml$Visits)
```

# Simplify as Visits2 to ensure proportionality

```
ml$Visits2 <- ml$Visits
ml$Visits2 <- str_replace(ml$Visits2, "3<", "3")
ml$Visits2 <- str_replace(ml$Visits2, "3", "3v")
ml$Visits2 <- str_replace(ml$Visits2, "0", "0v")
ml$Visits2 <- str_replace(ml$Visits2, "1", "1v")
ml$Visits2 <- str_replace(ml$Visits2, "2", "2v")

dplyr::count(ml, ml$Visits2)
```

# check Meds

```
ml$Meds <- as.factor(ml$Meds)
dplyr::count(ml, Meds)
```

# check Health category

```
dplyr::count(ml, ml$Health)
```

# Tidy up Health category

## Not much point including data where clients unsure of pet health.

## Also need to reorder categories

```
ml <- subset(ml, Health != "Unsure" | Health != NA)
ml$Health <- as.factor(ml$Health)
ml$Health <- ordered(ml$Health, levels = c("Healthy", "Generally healthy with minor or infrequent problems",
    "Significant or frequent problems", "Seriously ill"))
dplyr::count(ml, Health)
```

# create Health2 variable where serious and significant are combined

```
Health_severity = c("0_None", "1_Mild", "2_Significant")
ml$Health2 <- ml$Health
ml$Health2 <- str_replace(ml$Health2, "Healthy", "0_None")
ml$Health2 <- str_replace(ml$Health2, "Generally healthy with minor or infrequent problems",
    "1_Mild")
ml$Health2 <- str_replace(ml$Health2, "Significant or frequent problems",
    "2_Significant")
ml$Health2 <- str_replace(ml$Health2, "Seriously ill", "2_Significant")
ml$Health2 <- ordered(ml$Health2, levels = c("0_None", "1_Mild",
    "2_Significant"))
dplyr::count(ml, ml$Health2)
```

# create binary Health variable

```
bhealth <- rep(0, nrow(ml))
for (i in 1:nrow(ml)) {
    if (grepl("healthy", ml$Health[i], ignore.case = TRUE)) {
        # 0 is good health
        bhealth[i] <- 0
    } else {
        bhealth[i] <- 1
    }
}
# Keep as a variable not factor!
ml$Health_Binary <- as.numeric(bhealth)
rm(bhealth)
# Checks and Balances, hey it's VERY unbalanced
dplyr::count(ml, Health_Binary)
```

# create binary Any health problem variable

```
bhealth2 <- rep(0, nrow(ml))
for (i in 1:nrow(ml)) {
    if (grepl("0_None", ml$Health2[i], ignore.case = TRUE)) {
        # 0 is good health
        bhealth2[i] <- 0
    } else {
        bhealth2[i] <- 1
    }
}
# Keep as a variable not factor!
ml$Any_Health_Problem <- as.numeric(bhealth2)
rm(bhealth2)
# Checks and Balances, better balanced this time.
dplyr::count(ml, Any_Health_Problem)
```

# check D\_Diet

```
dplyr::count(ml, D_Diet)
```

# Not much point including those that are unsure.

```
ml <- subset(ml, D_Diet != "Unsure" | D_Diet != NA)
dplyr::count(ml, D_Diet)
```

# Not much point including diets where there are small numbers

```
ml <- subset(ml, D_Diet != "Mixture" | D_Diet != NA)
ml <- subset(ml, D_Diet != "Insect-based" | D_Diet != NA)
ml <- subset(ml, D_Diet != "Meat-based – lab-grown" | D_Diet !=
    NA)
dplyr::count(ml, D_Diet)
```

# create binary D\_Vegan variable

```
ml$D_Diet_Vegan <- ml$D_Diet
for (i in 1:nrow(ml)) {
    if (grepl("Vegan", ml$D_Diet_Vegan[i], fixed = TRUE)) {
        ml$D_Diet_Vegan[i] <- "Yes"
    } else {
        ml$D_Diet_Vegan[i] <- "No"
    }
}
dplyr::count(ml, D_Diet_Vegan)
```

# create binary D\_Raw variable

```
ml$D_Diet_Raw <- ml$D_Diet
for (i in 1:nrow(ml)) {
    if (grepl("raw", ml$D_Diet_Raw[i], fixed = TRUE)) {
        ml$D_Diet_Raw[i] <- "Yes"
    } else {
        ml$D_Diet_Raw[i] <- "No"
    }
}
dplyr::count(ml, D_Diet_Raw)
```

# create binary D\_Vegan\_Veggie variable

```
ml$D_Diet_Vegan_Veggie <- ml$D_Diet
for (i in 1:nrow(ml)) {
    if (grepl("Veg", ml$D_Diet_Vegan_Veggie[i], fixed = TRUE)) {
        ml$D_Diet_Vegan_Veggie[i] <- "Yes"
    } else {
        ml$D_Diet_Vegan_Veggie[i] <- "No"
    }
}
dplyr::count(ml, D_Diet_Vegan_Veggie)
```

# check Decision\_maker

```
dplyr::count(ml, Decision_Maker)
```

# create binary for primary decision maker

```
ml$Primary_Decision_Maker <- ml$Decision_Maker
for (i in 1:nrow(ml)) {
    if (grepl("Primary decision-maker", ml$Primary_Decision_Maker[i],
        fixed = TRUE)) {
        ml$Primary_Decision_Maker[i] <- "Yes"
    } else {
        ml$Primary_Decision_Maker[i] <- "No"
    }
}
dplyr::count(ml, ml$Primary_Decision_Maker)
```

# Let’s just keep those we will need

```
ml <- subset(ml, select = c("Location", "setting", "Urban", "Education",
    "Education_S", "Education_S2", "Animal_Career", "Animal_Career2",
    "Animal_Career_BINARY", "Income", "Income2", "C_Age", "C_Age2",
    "C_Gender", "C_Diet", "C_Diet_Vegan", "C_Diet_Vegan_Veggie",
    "D_Age", "D_Age_quant", "Size", "Size2", "Size_Giant", "D_Sex",
    "D_Neuter", "Therapeutic_Food", "Meds", "D_Diet", "D_Diet_Vegan",
    "D_Diet_Vegan_Veggie", "D_Diet_Raw", "Visits", "Visits2",
    "Health", "Health2", "Health_Binary", "Any_Health_Problem",
    "Primary_Decision_Maker"))

# check columns
head(ml)
```

# summary data

```
describe(ml)
```

```
## ml 
## 
##  37  Variables      2322  Observations
## --------------------------------------------------------------------------------
## Location 
##        n  missing distinct 
##     2322        0        5 
## 
## UK (1659, 0.714), Other European (358, 0.154), North America (128, 0.055),
## Australia/New Zealand/Oceania (100, 0.043), Other (77, 0.033)
## --------------------------------------------------------------------------------
## setting 
##        n  missing distinct 
##     2322        0        3 
##                                                           
## Value                        Urban                   Rural
## Frequency                      824                     758
## Proportion                   0.355                   0.326
##                                   
## Value      Equally urban and rural
## Frequency                      740
## Proportion                   0.319
## --------------------------------------------------------------------------------
## Urban 
##        n  missing distinct 
##     2322        0        2 
##                       
## Value         No   Yes
## Frequency   1498   824
## Proportion 0.645 0.355
## --------------------------------------------------------------------------------
## Education 
##        n  missing distinct 
##     2322        0        6 
##                                                                            
## Value           0_Basic 1_HighSchool    2_College       3_Grad         4_PG
## Frequency            29          410          662          682          460
## Proportion        0.012        0.177        0.285        0.294        0.198
##                        
## Value             5_PhD
## Frequency            79
## Proportion        0.034
## --------------------------------------------------------------------------------
## Education_S 
##        n  missing distinct 
##     2322        0        4 
##                                                                             
## Value      0_Basic_or_HighSchool             1_College                2_Grad
## Frequency                    439                   662                   682
## Proportion                 0.189                 0.285                 0.294
##                                 
## Value                3_PG_or_PhD
## Frequency                    539
## Proportion                 0.232
## --------------------------------------------------------------------------------
## Education_S2 
##        n  missing distinct 
##     2322        0        4 
##                                                                             
## Value      0_Basic_or_HighSchool             1_College                2_Grad
## Frequency                    439                   662                   682
## Proportion                 0.189                 0.285                 0.294
##                                 
## Value                3_PG_or_PhD
## Frequency                    539
## Proportion                 0.232
## --------------------------------------------------------------------------------
## Animal_Career 
##        n  missing distinct 
##     2322        0        6 
## 
## Animal breeder (15, 0.006), Animal trainer (124, 0.053), None of the above
## (1887, 0.813), Pet industry worker (186, 0.080), Veterinarian (59, 0.025),
## Veterinary technician or nurse (51, 0.022)
## --------------------------------------------------------------------------------
## Animal_Career2 
##        n  missing distinct 
##     2322        0        4 
##                                                                       
## Value        None of the above    Vet professional     breeder/trainer
## Frequency                 1887                 110                 139
## Proportion               0.813               0.047               0.060
##                               
## Value      Pet industry worker
## Frequency                  186
## Proportion               0.080
## --------------------------------------------------------------------------------
## Animal_Career_BINARY 
##        n  missing distinct 
##     2322        0        2 
##                       
## Value         No   Yes
## Frequency   1887   435
## Proportion 0.813 0.187
## --------------------------------------------------------------------------------
## Income 
##        n  missing distinct 
##     2322        0        3 
##                                
## Value         Low Medium   High
## Frequency     364   1611    347
## Proportion  0.157  0.694  0.149
## --------------------------------------------------------------------------------
## Income2 
##        n  missing distinct 
##     2322        0        3 
##                                
## Value         Low Medium   High
## Frequency     364   1611    347
## Proportion  0.157  0.694  0.149
## --------------------------------------------------------------------------------
## C_Age 
##        n  missing distinct 
##     2322        0        5 
##                                         
## Value        <30 30–39 40–49 50–59   60<
## Frequency    385   507   474   528   428
## Proportion 0.166 0.218 0.204 0.227 0.184
## --------------------------------------------------------------------------------
## C_Age2 
##        n  missing distinct 
##     2322        0        5 
##                                         
## Value        <30 30–39 40–49 50–59   60<
## Frequency    385   507   474   528   428
## Proportion 0.166 0.218 0.204 0.227 0.184
## --------------------------------------------------------------------------------
## C_Gender 
##        n  missing distinct 
##     2322        0        2 
##                         
## Value      Female   Male
## Frequency    2146    176
## Proportion  0.924  0.076
## --------------------------------------------------------------------------------
## C_Diet 
##        n  missing distinct 
##     2322        0        5 
## 
## lowest : Omnivore (i.e. eat meat)                        Omnivore reducing animal product consumption    Pescatarian (including fish but no other meats) Vegan (consuming no animal products)            Vegetarian (consuming plants, eggs and milk)   
## highest: Omnivore (i.e. eat meat)                        Omnivore reducing animal product consumption    Pescatarian (including fish but no other meats) Vegan (consuming no animal products)            Vegetarian (consuming plants, eggs and milk)   
## --------------------------------------------------------------------------------
## C_Diet_Vegan 
##        n  missing distinct 
##     2322        0        2 
##                       
## Value         No   Yes
## Frequency   1790   532
## Proportion 0.771 0.229
## --------------------------------------------------------------------------------
## C_Diet_Vegan_Veggie 
##        n  missing distinct 
##     2322        0        2 
##                     
## Value        No  Yes
## Frequency  1555  767
## Proportion 0.67 0.33
## --------------------------------------------------------------------------------
## D_Age 
##        n  missing distinct     Info     Mean      Gmd      .05      .10 
##     2322        0       19    0.993    6.187    4.111        1        2 
##      .25      .50      .75      .90      .95 
##        3        6        9       11       13 
##                                                                             
## Value          1     2     3     4     5     6     7     8     9    10    11
## Frequency    150   255   256   241   242   204   192   177   143   134   101
## Proportion 0.065 0.110 0.110 0.104 0.104 0.088 0.083 0.076 0.062 0.058 0.043
##                                                           
## Value         12    13    14    15    16    17    18    20
## Frequency     75    59    50    26     8     7     1     1
## Proportion 0.032 0.025 0.022 0.011 0.003 0.003 0.000 0.000
## 
## For the frequency table, variable is rounded to the nearest 0
## --------------------------------------------------------------------------------
## D_Age_quant 
##        n  missing distinct 
##     2322        0        5 
##                                                        
## Value         [1,3]    (3,5]    (5,7]  (7,9.4] (9.4,20]
## Frequency       661      483      396      320      462
## Proportion    0.285    0.208    0.171    0.138    0.199
## --------------------------------------------------------------------------------
## Size 
##        n  missing distinct 
##     2322        0        5 
##                                              
## Value         Toy  Small Medium  Large  Giant
## Frequency      56    467    896    803    100
## Proportion  0.024  0.201  0.386  0.346  0.043
## --------------------------------------------------------------------------------
## Size2 
##        n  missing distinct 
##     2322        0        5 
##                                              
## Value      Medium    Toy  Small  Large  Giant
## Frequency     896     56    467    803    100
## Proportion  0.386  0.024  0.201  0.346  0.043
## --------------------------------------------------------------------------------
## Size_Giant 
##        n  missing distinct 
##     2322        0        2 
##                       
## Value         No   Yes
## Frequency   2222   100
## Proportion 0.957 0.043
## --------------------------------------------------------------------------------
## D_Sex 
##        n  missing distinct 
##     2322        0        2 
##                         
## Value      Female   Male
## Frequency    1095   1227
## Proportion  0.472  0.528
## --------------------------------------------------------------------------------
## D_Neuter 
##        n  missing distinct 
##     2322        0        2 
##                             
## Value        Intact Neutered
## Frequency       512     1810
## Proportion     0.22     0.78
## --------------------------------------------------------------------------------
## Therapeutic_Food 
##        n  missing distinct 
##     2322        0        2 
##                       
## Value         No   Yes
## Frequency   2211   111
## Proportion 0.952 0.048
## --------------------------------------------------------------------------------
## Meds 
##        n  missing distinct 
##     2322        0        2 
##                       
## Value         No   Yes
## Frequency   1386   936
## Proportion 0.597 0.403
## --------------------------------------------------------------------------------
## D_Diet 
##        n  missing distinct 
##     2322        0        4 
## 
## Meat-based – conventional (1230, 0.530), Meat-based – raw (744, 0.320), Vegan
## (consuming no animal products) (313, 0.135), Vegetarian (including eggs or
## milk, but not meat) (35, 0.015)
## --------------------------------------------------------------------------------
## D_Diet_Vegan 
##        n  missing distinct 
##     2322        0        2 
##                       
## Value         No   Yes
## Frequency   2009   313
## Proportion 0.865 0.135
## --------------------------------------------------------------------------------
## D_Diet_Vegan_Veggie 
##        n  missing distinct 
##     2322        0        2 
##                     
## Value        No  Yes
## Frequency  1974  348
## Proportion 0.85 0.15
## --------------------------------------------------------------------------------
## D_Diet_Raw 
##        n  missing distinct 
##     2322        0        2 
##                     
## Value        No  Yes
## Frequency  1578  744
## Proportion 0.68 0.32
## --------------------------------------------------------------------------------
## Visits 
##        n  missing distinct 
##     2322        0        5 
##                                         
## Value          0     1     2     3    3<
## Frequency    408   907   506   200   301
## Proportion 0.176 0.391 0.218 0.086 0.130
## --------------------------------------------------------------------------------
## Visits2 
##        n  missing distinct 
##     2322        0        4 
##                                   
## Value         0v    1v    2v    3v
## Frequency    408   907   506   501
## Proportion 0.176 0.391 0.218 0.216
## --------------------------------------------------------------------------------
## Health 
##        n  missing distinct 
##     2322        0        4 
## 
## --------------------------------------------------------------------------------
## Health2 
##        n  missing distinct 
##     2322        0        3 
##                                                     
## Value             0_None        1_Mild 2_Significant
## Frequency           1429           762           131
## Proportion         0.615         0.328         0.056
## --------------------------------------------------------------------------------
## Health_Binary 
##        n  missing distinct     Info      Sum     Mean      Gmd 
##     2322        0        2     0.16      131  0.05642   0.1065 
## 
## --------------------------------------------------------------------------------
## Any_Health_Problem 
##        n  missing distinct     Info      Sum     Mean      Gmd 
##     2322        0        2     0.71      893   0.3846   0.4736 
## 
## --------------------------------------------------------------------------------
## Primary_Decision_Maker 
##        n  missing distinct 
##     2322        0        2 
##                       
## Value         No   Yes
## Frequency    111  2211
## Proportion 0.048 0.952
## --------------------------------------------------------------------------------
```

# check C\_Diet category numbers

```
dplyr::count(ml, C_Diet)
```

# check D\_Sex by D\_Neuter

```
## two way cross tabs (xtabs) and flatten the table
ftable(xtabs(~D_Sex + D_Neuter, data = ml))
```

```
##        D_Neuter Intact Neutered
## D_Sex                          
## Female             203      892
## Male               309      918
```

# Citations for packages

```
citation("stringr")
```

```
## 
## To cite package 'stringr' in publications use:
## 
##   Wickham H (2022). _stringr: Simple, Consistent Wrappers for Common
##   String Operations_. R package version 1.5.0,
##   <https://CRAN.R-project.org/package=stringr>.
## 
## A BibTeX entry for LaTeX users is
## 
##   @Manual{,
##     title = {stringr: Simple, Consistent Wrappers for Common String Operations},
##     author = {Hadley Wickham},
##     year = {2022},
##     note = {R package version 1.5.0},
##     url = {https://CRAN.R-project.org/package=stringr},
##   }
```

```
packageVersion("stringr")
```

```
## [1] '1.5.0'
```

```
citation("dplyr")
```

```
## 
## To cite package 'dplyr' in publications use:
## 
##   Wickham H, François R, Henry L, Müller K, Vaughan D (2023). _dplyr: A
##   Grammar of Data Manipulation_. R package version 1.1.3,
##   <https://CRAN.R-project.org/package=dplyr>.
## 
## A BibTeX entry for LaTeX users is
## 
##   @Manual{,
##     title = {dplyr: A Grammar of Data Manipulation},
##     author = {Hadley Wickham and Romain François and Lionel Henry and Kirill Müller and Davis Vaughan},
##     year = {2023},
##     note = {R package version 1.1.3},
##     url = {https://CRAN.R-project.org/package=dplyr},
##   }
```

```
packageVersion("dplyr")
```

```
## [1] '1.1.3'
```

```
citation("DescTools")
```

```
## 
## To cite package 'DescTools' in publications use:
## 
##   Signorell A (2023). _DescTools: Tools for Descriptive Statistics_. R
##   package version 0.99.50,
##   <https://CRAN.R-project.org/package=DescTools>.
## 
## A BibTeX entry for LaTeX users is
## 
##   @Manual{,
##     title = {DescTools: Tools for Descriptive Statistics},
##     author = {Andri Signorell},
##     year = {2023},
##     note = {R package version 0.99.50},
##     url = {https://CRAN.R-project.org/package=DescTools},
##   }
```

```
packageVersion("DescTools")
```

```
## [1] '0.99.50'
```

```
citation("car")
```

```
## 
## To cite the car package in publications use:
## 
##   Fox J, Weisberg S (2019). _An R Companion to Applied Regression_,
##   Third edition. Sage, Thousand Oaks CA.
##   <https://socialsciences.mcmaster.ca/jfox/Books/Companion/>.
## 
## A BibTeX entry for LaTeX users is
## 
##   @Book{,
##     title = {An {R} Companion to Applied Regression},
##     edition = {Third},
##     author = {John Fox and Sanford Weisberg},
##     year = {2019},
##     publisher = {Sage},
##     address = {Thousand Oaks {CA}},
##     url = {https://socialsciences.mcmaster.ca/jfox/Books/Companion/},
##   }
```

```
packageVersion("car")
```

```
## [1] '3.1.2'
```

```
citation("foreign")
```

```
## 
## To cite package 'foreign' in publications use:
## 
##   R Core Team (2023). _foreign: Read Data Stored by 'Minitab', 'S',
##   'SAS', 'SPSS', 'Stata', 'Systat', 'Weka', 'dBase', ..._. R package
##   version 0.8-85, <https://CRAN.R-project.org/package=foreign>.
## 
## A BibTeX entry for LaTeX users is
## 
##   @Manual{,
##     title = {foreign: Read Data Stored by 'Minitab', 'S', 'SAS', 'SPSS', 'Stata',
## 'Systat', 'Weka', 'dBase', ...},
##     author = {{R Core Team}},
##     year = {2023},
##     note = {R package version 0.8-85},
##     url = {https://CRAN.R-project.org/package=foreign},
##   }
```

```
packageVersion("foreign")
```

```
## [1] '0.8.85'
```

```
citation("ggplot2")
```

```
## 
## To cite ggplot2 in publications, please use
## 
##   H. Wickham. ggplot2: Elegant Graphics for Data Analysis.
##   Springer-Verlag New York, 2016.
## 
## A BibTeX entry for LaTeX users is
## 
##   @Book{,
##     author = {Hadley Wickham},
##     title = {ggplot2: Elegant Graphics for Data Analysis},
##     publisher = {Springer-Verlag New York},
##     year = {2016},
##     isbn = {978-3-319-24277-4},
##     url = {https://ggplot2.tidyverse.org},
##   }
```

```
packageVersion("ggplot2")
```

```
## [1] '3.4.3'
```

```
citation("Hmisc")
```

```
## 
## To cite package 'Hmisc' in publications use:
## 
##   Harrell Jr F (2023). _Hmisc: Harrell Miscellaneous_. R package
##   version 5.1-1, <https://CRAN.R-project.org/package=Hmisc>.
## 
## A BibTeX entry for LaTeX users is
## 
##   @Manual{,
##     title = {Hmisc: Harrell Miscellaneous},
##     author = {Frank E {Harrell Jr}},
##     year = {2023},
##     note = {R package version 5.1-1},
##     url = {https://CRAN.R-project.org/package=Hmisc},
##   }
```

```
packageVersion("Hmisc")
```

```
## [1] '5.1.1'
```

```
citation("reshape2")
```

```
## 
## To cite reshape2 in publications use:
## 
##   Hadley Wickham (2007). Reshaping Data with the reshape Package.
##   Journal of Statistical Software, 21(12), 1-20. URL
##   http://www.jstatsoft.org/v21/i12/.
## 
## A BibTeX entry for LaTeX users is
## 
##   @Article{,
##     title = {Reshaping Data with the {reshape} Package},
##     author = {Hadley Wickham},
##     journal = {Journal of Statistical Software},
##     year = {2007},
##     volume = {21},
##     number = {12},
##     pages = {1--20},
##     url = {http://www.jstatsoft.org/v21/i12/},
##   }
```

```
packageVersion("reshape2")
```

```
## [1] '1.4.4'
```

```
citation("tidyverse")
```

```
## 
## To cite package 'tidyverse' in publications use:
## 
##   Wickham H, Averick M, Bryan J, Chang W, McGowan LD, François R,
##   Grolemund G, Hayes A, Henry L, Hester J, Kuhn M, Pedersen TL, Miller
##   E, Bache SM, Müller K, Ooms J, Robinson D, Seidel DP, Spinu V,
##   Takahashi K, Vaughan D, Wilke C, Woo K, Yutani H (2019). "Welcome to
##   the tidyverse." _Journal of Open Source Software_, *4*(43), 1686.
##   doi:10.21105/joss.01686 <https://doi.org/10.21105/joss.01686>.
## 
## A BibTeX entry for LaTeX users is
## 
##   @Article{,
##     title = {Welcome to the {tidyverse}},
##     author = {Hadley Wickham and Mara Averick and Jennifer Bryan and Winston Chang and Lucy D'Agostino McGowan and Romain François and Garrett Grolemund and Alex Hayes and Lionel Henry and Jim Hester and Max Kuhn and Thomas Lin Pedersen and Evan Miller and Stephan Milton Bache and Kirill Müller and Jeroen Ooms and David Robinson and Dana Paige Seidel and Vitalie Spinu and Kohske Takahashi and Davis Vaughan and Claus Wilke and Kara Woo and Hiroaki Yutani},
##     year = {2019},
##     journal = {Journal of Open Source Software},
##     volume = {4},
##     number = {43},
##     pages = {1686},
##     doi = {10.21105/joss.01686},
##   }
```

```
packageVersion("tidyverse")
```

```
## [1] '2.0.0'
```

```
citation("broom")
```

```
## 
## To cite package 'broom' in publications use:
## 
##   Robinson D, Hayes A, Couch S (2023). _broom: Convert Statistical
##   Objects into Tidy Tibbles_. R package version 1.0.5,
##   <https://CRAN.R-project.org/package=broom>.
## 
## A BibTeX entry for LaTeX users is
## 
##   @Manual{,
##     title = {broom: Convert Statistical Objects into Tidy Tibbles},
##     author = {David Robinson and Alex Hayes and Simon Couch},
##     year = {2023},
##     note = {R package version 1.0.5},
##     url = {https://CRAN.R-project.org/package=broom},
##   }
```

```
packageVersion("broom")
```

```
## [1] '1.0.5'
```

```
citation("aod")
```

```
## 
## To cite package 'aod' in publications use:
## 
##   Lesnoff, M., Lancelot, R. (2012). aod: Analysis of Overdispersed
##   Data. R package version 1.3.2, URL
##   http://cran.r-project.org/package=aod
## 
## A BibTeX entry for LaTeX users is
## 
##   @Manual{,
##     title = {aod: Analysis of Overdispersed Data},
##     author = {{Lesnoff} and {M.} and {Lancelot} and {R.}},
##     year = {2012},
##     note = {R package version 1.3.2},
##     url = {https://cran.r-project.org/package=aod},
##   }
```

```
packageVersion("aod")
```

```
## [1] '1.3.2'
```

```
citation("glmtoolbox")
```

```
## 
## To cite package 'glmtoolbox' in publications use:
## 
##   Vanegas L, Rondón L, Paula G (2023). _glmtoolbox: Set of Tools to
##   Data Analysis using Generalized Linear Models_. R package version
##   0.1.9, <https://CRAN.R-project.org/package=glmtoolbox>.
## 
## A BibTeX entry for LaTeX users is
## 
##   @Manual{,
##     title = {glmtoolbox: Set of Tools to Data Analysis using Generalized Linear Models},
##     author = {Luis Hernando Vanegas and Luz Marina Rondón and Gilberto A. Paula},
##     year = {2023},
##     note = {R package version 0.1.9},
##     url = {https://CRAN.R-project.org/package=glmtoolbox},
##   }
```

```
packageVersion("glmtoolbox")
```

```
## [1] '0.1.9'
```

```
citation("fmsb")
```

```
## 
## To cite package 'fmsb' in publications use:
## 
##   Nakazawa M (2023). _fmsb: Functions for Medical Statistics Book with
##   some Demographic Data_. R package version 0.7.5,
##   <https://CRAN.R-project.org/package=fmsb>.
## 
## A BibTeX entry for LaTeX users is
## 
##   @Manual{,
##     title = {fmsb: Functions for Medical Statistics Book with some Demographic Data},
##     author = {Minato Nakazawa},
##     year = {2023},
##     note = {R package version 0.7.5},
##     url = {https://CRAN.R-project.org/package=fmsb},
##   }
## 
## ATTENTION: This citation information has been auto-generated from the
## package DESCRIPTION file and may need manual editing, see
## 'help("citation")'.
```

```
packageVersion("fmsb")
```

```
## [1] '0.7.5'
```

```
citation("uwot")
```

```
## 
## To cite package 'uwot' in publications use:
## 
##   Melville J (2023). _uwot: The Uniform Manifold Approximation and
##   Projection (UMAP) Method for Dimensionality Reduction_. R package
##   version 0.1.16, <https://CRAN.R-project.org/package=uwot>.
## 
## A BibTeX entry for LaTeX users is
## 
##   @Manual{,
##     title = {uwot: The Uniform Manifold Approximation and Projection (UMAP) Method
## for Dimensionality Reduction},
##     author = {James Melville},
##     year = {2023},
##     note = {R package version 0.1.16},
##     url = {https://CRAN.R-project.org/package=uwot},
##   }
```

```
packageVersion("uwot")
```

```
## [1] '0.1.16'
```

```
citation("ggthemes")
```

```
## 
## To cite package 'ggthemes' in publications use:
## 
##   Arnold J (2021). _ggthemes: Extra Themes, Scales and Geoms for
##   'ggplot2'_. R package version 4.2.4,
##   <https://CRAN.R-project.org/package=ggthemes>.
## 
## A BibTeX entry for LaTeX users is
## 
##   @Manual{,
##     title = {ggthemes: Extra Themes, Scales and Geoms for 'ggplot2'},
##     author = {Jeffrey B. Arnold},
##     year = {2021},
##     note = {R package version 4.2.4},
##     url = {https://CRAN.R-project.org/package=ggthemes},
##   }
```

```
packageVersion("ggthemes")
```

```
## [1] '4.2.4'
```

```
citation("ggforce")
```

```
## 
## To cite package 'ggforce' in publications use:
## 
##   Pedersen T (2022). _ggforce: Accelerating 'ggplot2'_. R package
##   version 0.4.1, <https://CRAN.R-project.org/package=ggforce>.
## 
## A BibTeX entry for LaTeX users is
## 
##   @Manual{,
##     title = {ggforce: Accelerating 'ggplot2'},
##     author = {Thomas Lin Pedersen},
##     year = {2022},
##     note = {R package version 0.4.1},
##     url = {https://CRAN.R-project.org/package=ggforce},
##   }
```

```
packageVersion("ggforce")
```

```
## [1] '0.4.1'
```

```
citation("readxl")
```

```
## 
## To cite package 'readxl' in publications use:
## 
##   Wickham H, Bryan J (2023). _readxl: Read Excel Files_. R package
##   version 1.4.3, <https://CRAN.R-project.org/package=readxl>.
## 
## A BibTeX entry for LaTeX users is
## 
##   @Manual{,
##     title = {readxl: Read Excel Files},
##     author = {Hadley Wickham and Jennifer Bryan},
##     year = {2023},
##     note = {R package version 1.4.3},
##     url = {https://CRAN.R-project.org/package=readxl},
##   }
```

```
packageVersion("readxl")
```

```
## [1] '1.4.3'
```

```
citation("ROCR")
```

```
## 
## To cite ROCR in publications use:
## 
##   Sing T, Sander O, Beerenwinkel N, Lengauer T (2005). "ROCR:
##   visualizing classifier performance in R." _Bioinformatics_, *21*(20),
##   7881. <http://rocr.bioinf.mpi-sb.mpg.de>.
## 
## A BibTeX entry for LaTeX users is
## 
##   @Article{,
##     entry = {article},
##     title = {ROCR: visualizing classifier performance in R},
##     author = {T. Sing and O. Sander and N. Beerenwinkel and T. Lengauer},
##     year = {2005},
##     journal = {Bioinformatics},
##     volume = {21},
##     number = {20},
##     pages = {7881},
##     url = {http://rocr.bioinf.mpi-sb.mpg.de},
##   }
## 
## We have invested a lot of time and effort in creating ROCR, please cite
## it when using it for data analysis.
```

```
packageVersion("ROCR")
```

```
## [1] '1.0.11'
```

```
citation("pROC")
```

```
## 
## If you use pROC in published research, please cite the following paper:
## 
##   Xavier Robin, Natacha Turck, Alexandre Hainard, Natalia Tiberti,
##   Frédérique Lisacek, Jean-Charles Sanchez and Markus Müller (2011).
##   pROC: an open-source package for R and S+ to analyze and compare ROC
##   curves. BMC Bioinformatics, 12, p. 77.  DOI: 10.1186/1471-2105-12-77
##   <http://www.biomedcentral.com/1471-2105/12/77/>
## 
## A BibTeX entry for LaTeX users is
## 
##   @Article{,
##     title = {pROC: an open-source package for R and S+ to analyze and compare ROC curves},
##     author = {Xavier Robin and Natacha Turck and Alexandre Hainard and Natalia Tiberti and Frédérique Lisacek and Jean-Charles Sanchez and Markus Müller},
##     year = {2011},
##     journal = {BMC Bioinformatics},
##     volume = {12},
##     pages = {77},
##   }
```

```
packageVersion("pROC")
```

```
## [1] '1.18.4'
```

```
citation("caret")
```

```
## 
## To cite caret in publications use:
## 
##   Kuhn, M. (2008). Building Predictive Models in R Using the caret
##   Package. Journal of Statistical Software, 28(5), 1–26.
##   https://doi.org/10.18637/jss.v028.i05
## 
## A BibTeX entry for LaTeX users is
## 
##   @Article{,
##     title = {Building Predictive Models in R Using the caret Package},
##     volume = {28},
##     url = {https://www.jstatsoft.org/index.php/jss/article/view/v028i05},
##     doi = {10.18637/jss.v028.i05},
##     number = {5},
##     journal = {Journal of Statistical Software},
##     author = {{Kuhn} and {Max}},
##     year = {2008},
##     pages = {1–26},
##   }
```

```
packageVersion("caret")
```

```
## [1] '6.0.94'
```

```
citation("xgboost")
```

```
## 
## To cite package 'xgboost' in publications use:
## 
##   Chen T, He T, Benesty M, Khotilovich V, Tang Y, Cho H, Chen K,
##   Mitchell R, Cano I, Zhou T, Li M, Xie J, Lin M, Geng Y, Li Y, Yuan J
##   (2023). _xgboost: Extreme Gradient Boosting_. R package version
##   1.7.5.1, <https://CRAN.R-project.org/package=xgboost>.
## 
## A BibTeX entry for LaTeX users is
## 
##   @Manual{,
##     title = {xgboost: Extreme Gradient Boosting},
##     author = {Tianqi Chen and Tong He and Michael Benesty and Vadim Khotilovich and Yuan Tang and Hyunsu Cho and Kailong Chen and Rory Mitchell and Ignacio Cano and Tianyi Zhou and Mu Li and Junyuan Xie and Min Lin and Yifeng Geng and Yutian Li and Jiaming Yuan},
##     year = {2023},
##     note = {R package version 1.7.5.1},
##     url = {https://CRAN.R-project.org/package=xgboost},
##   }
```

```
packageVersion("xgboost")
```

```
## [1] '1.7.5.1'
```

```
citation("smotefamily")
```

```
## 
## To cite package 'smotefamily' in publications use:
## 
##   Siriseriwan W (2019). _smotefamily: A Collection of Oversampling
##   Techniques for Class Imbalance Problem Based on SMOTE_. R package
##   version 1.3.1, <https://CRAN.R-project.org/package=smotefamily>.
## 
## A BibTeX entry for LaTeX users is
## 
##   @Manual{,
##     title = {smotefamily: A Collection of Oversampling Techniques for Class Imbalance
## Problem Based on SMOTE},
##     author = {Wacharasak Siriseriwan},
##     year = {2019},
##     note = {R package version 1.3.1},
##     url = {https://CRAN.R-project.org/package=smotefamily},
##   }
```

```
packageVersion("smotefamily")
```

```
## [1] '1.3.1'
```

```
citation("rsample")
```

```
## 
## To cite package 'rsample' in publications use:
## 
##   Frick H, Chow F, Kuhn M, Mahoney M, Silge J, Wickham H (2023).
##   _rsample: General Resampling Infrastructure_. R package version
##   1.2.0, <https://CRAN.R-project.org/package=rsample>.
## 
## A BibTeX entry for LaTeX users is
## 
##   @Manual{,
##     title = {rsample: General Resampling Infrastructure},
##     author = {Hannah Frick and Fanny Chow and Max Kuhn and Michael Mahoney and Julia Silge and Hadley Wickham},
##     year = {2023},
##     note = {R package version 1.2.0},
##     url = {https://CRAN.R-project.org/package=rsample},
##   }
```

```
packageVersion("rsample")
```

```
## [1] '1.2.0'
```

```
citation("mltools")
```

```
## 
## To cite package 'mltools' in publications use:
## 
##   Gorman B (2018). _mltools: Machine Learning Tools_. R package version
##   0.3.5, <https://CRAN.R-project.org/package=mltools>.
## 
## A BibTeX entry for LaTeX users is
## 
##   @Manual{,
##     title = {mltools: Machine Learning Tools},
##     author = {Ben Gorman},
##     year = {2018},
##     note = {R package version 0.3.5},
##     url = {https://CRAN.R-project.org/package=mltools},
##   }
## 
## ATTENTION: This citation information has been auto-generated from the
## package DESCRIPTION file and may need manual editing, see
## 'help("citation")'.
```

```
packageVersion("mltools")
```

```
## [1] '0.3.5'
```

```
citation("data.table")
```

```
## 
## To cite package 'data.table' in publications use:
## 
##   Dowle M, Srinivasan A (2023). _data.table: Extension of
##   `data.frame`_. R package version 1.14.8,
##   <https://CRAN.R-project.org/package=data.table>.
## 
## A BibTeX entry for LaTeX users is
## 
##   @Manual{,
##     title = {data.table: Extension of `data.frame`},
##     author = {Matt Dowle and Arun Srinivasan},
##     year = {2023},
##     note = {R package version 1.14.8},
##     url = {https://CRAN.R-project.org/package=data.table},
##   }
```

```
packageVersion("data.table")
```

```
## [1] '1.14.8'
```

```
citation("heatmaply")
```

```
## 
## The methods within the package can be cited as:
## 
##   Galili, Tal, O'Callaghan, Alan, Sidi, Jonathan, Sievert, Carson
##   (2017). "heatmaply: an R package for creating interactive cluster
##   heatmaps for online publishing." _Bioinformatics_.
##   doi:10.1093/bioinformatics/btx657
##   <https://doi.org/10.1093/bioinformatics/btx657>,
##   <https://academic.oup.com/bioinformatics/article-pdf/doi/10.1093/bioinformatics/btx657/21358327/btx657.pdf>.
## 
## A BibTeX entry for LaTeX users is
## 
##   @Article{,
##     author = {{Galili} and {Tal} and {O'Callaghan} and {Alan} and {Sidi} and {Jonathan} and {Sievert} and {Carson}},
##     title = {heatmaply: an R package for creating interactive cluster heatmaps for online publishing},
##     journal = {Bioinformatics},
##     year = {2017},
##     doi = {10.1093/bioinformatics/btx657},
##     url = {https://academic.oup.com/bioinformatics/article-pdf/doi/10.1093/bioinformatics/btx657/21358327/btx657.pdf},
##   }
## 
## This free open-source software implements academic research by the
## authors and co-workers. If you use it, please support the project by
## citing the appropriate journal articles.
```

```
packageVersion("heatmaply")
```

```
## [1] '1.5.0'
```

```
citation("rstatix")
```

```
## 
## To cite package 'rstatix' in publications use:
## 
##   Kassambara A (2023). _rstatix: Pipe-Friendly Framework for Basic
##   Statistical Tests_. R package version 0.7.2,
##   <https://CRAN.R-project.org/package=rstatix>.
## 
## A BibTeX entry for LaTeX users is
## 
##   @Manual{,
##     title = {rstatix: Pipe-Friendly Framework for Basic Statistical Tests},
##     author = {Alboukadel Kassambara},
##     year = {2023},
##     note = {R package version 0.7.2},
##     url = {https://CRAN.R-project.org/package=rstatix},
##   }
```

```
packageVersion("rstatix")
```

```
## [1] '0.7.2'
```

```
citation("dbscan")
```

```
## 
## Hahsler M, Piekenbrock M (2022). _dbscan: Density-Based Spatial
## Clustering of Applications with Noise (DBSCAN) and Related Algorithms_.
## R package version 1.1-11, <https://CRAN.R-project.org/package=dbscan>.
## 
## To cite dbscan in publications use:
## 
##   Hahsler M, Piekenbrock M, Doran D (2019). "dbscan: Fast Density-Based
##   Clustering with R." _Journal of Statistical Software_, *91*(1), 1-30.
##   doi:10.18637/jss.v091.i01 <https://doi.org/10.18637/jss.v091.i01>.
## 
## To see these entries in BibTeX format, use 'print(<citation>,
## bibtex=TRUE)', 'toBibtex(.)', or set
## 'options(citation.bibtex.max=999)'.
```

```
packageVersion("dbscan")
```

```
## [1] '1.1.11'
```

```
citation("corrplot")
```

```
## 
## To cite corrplot in publications use:
## 
##   Taiyun Wei and Viliam Simko (2021). R package 'corrplot':
##   Visualization of a Correlation Matrix (Version 0.92). Available from
##   https://github.com/taiyun/corrplot
## 
## A BibTeX entry for LaTeX users is
## 
##   @Manual{corrplot2021,
##     title = {R package 'corrplot': Visualization of a Correlation Matrix},
##     author = {Taiyun Wei and Viliam Simko},
##     year = {2021},
##     note = {(Version 0.92)},
##     url = {https://github.com/taiyun/corrplot},
##   }
```

```
packageVersion("corrplot")
```

```
## [1] '0.92'
```

```
citation("showtext")
```

```
## 
## To cite package 'showtext' in publications use:
## 
##   Qiu Y, details. aotisSfAf (2023). _showtext: Using Fonts More Easily
##   in R Graphs_. R package version 0.9-6,
##   <https://CRAN.R-project.org/package=showtext>.
## 
## A BibTeX entry for LaTeX users is
## 
##   @Manual{,
##     title = {showtext: Using Fonts More Easily in R Graphs},
##     author = {Yixuan Qiu and authors/contributors of the included software. See file AUTHORS for details.},
##     year = {2023},
##     note = {R package version 0.9-6},
##     url = {https://CRAN.R-project.org/package=showtext},
##   }
## 
## ATTENTION: This citation information has been auto-generated from the
## package DESCRIPTION file and may need manual editing, see
## 'help("citation")'.
```

```
packageVersion("showtext")
```

```
## [1] '0.9.6'
```

```
citation("cluster")
```

```
## 
## To cite the R package 'cluster' in publications use:
## 
##   Maechler, M., Rousseeuw, P., Struyf, A., Hubert, M., Hornik,
##   K.(2022).  cluster: Cluster Analysis Basics and Extensions. R package
##   version 2.1.4.
## 
## A BibTeX entry for LaTeX users is
## 
##   @Manual{,
##     title = {cluster: Cluster Analysis Basics and Extensions},
##     author = {Martin Maechler and Peter Rousseeuw and Anja Struyf and Mia Hubert and Kurt Hornik},
##     year = {2022},
##     url = {https://CRAN.R-project.org/package=cluster},
##     note = {R package version 2.1.4 --- For new features, see the 'Changelog' file (in the package source)},
##   }
```

```
packageVersion("cluster")
```

```
## [1] '2.1.4'
```

```
citation("factoextra")
```

```
## 
## To cite package 'factoextra' in publications use:
## 
##   Kassambara A, Mundt F (2020). _factoextra: Extract and Visualize the
##   Results of Multivariate Data Analyses_. R package version 1.0.7,
##   <https://CRAN.R-project.org/package=factoextra>.
## 
## A BibTeX entry for LaTeX users is
## 
##   @Manual{,
##     title = {factoextra: Extract and Visualize the Results of Multivariate Data Analyses},
##     author = {Alboukadel Kassambara and Fabian Mundt},
##     year = {2020},
##     note = {R package version 1.0.7},
##     url = {https://CRAN.R-project.org/package=factoextra},
##   }
```

```
packageVersion("factoextra")
```

```
## [1] '1.0.7'
```

```
citation("clusterSim")
```

```
## 
## To cite clusterSim in publications use:
## 
##   Walesiak M, Dudek A (2020). "The Choice of Variable Normalization
##   Method in Cluster Analysis." In Soliman KS (ed.), _Education
##   Excellence and Innovation Management: A 2025 Vision to Sustain
##   Economic Development During Global Challenges_, 325-340. ISBN
##   978-0-9998551-4-1.
## 
## A BibTeX entry for LaTeX users is
## 
##   @InProceedings{,
##     title = {The Choice of Variable Normalization Method in Cluster Analysis},
##     author = {Marek Walesiak and Andrzej Dudek},
##     booktitle = {Education Excellence and Innovation Management: A 2025 Vision to Sustain Economic Development During Global Challenges},
##     booksubtitle = {Proceedings of the 35th International Business Information Management Association Conference (IBIMA), 1-2 April 2020, Seville, Spain},
##     year = {2020},
##     publisher = {International Business Information Management Association (IBIMA)},
##     editor = {Khalid S. Soliman},
##     pages = {325-340},
##     isbn = {978-0-9998551-4-1},
##   }
```

```
packageVersion("clusterSim")
```

```
## [1] '0.51.3'
```

```
citation("RColorBrewer")
```

```
## 
## To cite package 'RColorBrewer' in publications use:
## 
##   Neuwirth E (2022). _RColorBrewer: ColorBrewer Palettes_. R package
##   version 1.1-3, <https://CRAN.R-project.org/package=RColorBrewer>.
## 
## A BibTeX entry for LaTeX users is
## 
##   @Manual{,
##     title = {RColorBrewer: ColorBrewer Palettes},
##     author = {Erich Neuwirth},
##     year = {2022},
##     note = {R package version 1.1-3},
##     url = {https://CRAN.R-project.org/package=RColorBrewer},
##   }
```

```
packageVersion("RColorBrewer")
```

```
## [1] '1.1.3'
```

```
citation("IHW")
```

```
## 
## To cite package 'IHW' in publications use:
## 
##   Nikolaos Ignatiadis, Bernd Klaus, Judith Zaugg and Wolfgang Huber
##   (2016): Data-driven hypothesis weighting increases detection power in
##   genome-scale multiple testing. Nature Methods 13:577, doi:
##   10.1038/nmeth.3885
## 
##   Nikolaos Ignatiadis and Wolfgang Huber (2016): Covariate-powered
##   weighted multiple testing with false discovery rate control.
##   arXiv:1701.05179
## 
## To see these entries in BibTeX format, use 'print(<citation>,
## bibtex=TRUE)', 'toBibtex(.)', or set
## 'options(citation.bibtex.max=999)'.
```

```
packageVersion("IHW")
```

```
## [1] '1.26.0'
```

```
if (SAVE) {
    save(ml, file = "ml_read_data_102.RData")
}
```
