# Supplementary material for: Variables associated with owner perceptions of the health of their dog: Further analysis of data from a large international survey
Source: PLoS One. 2024 May 15;19(5):e0280173. doi: 10.1371/journal.pone.0280173 (PMC11095744; doi:10.1371/journal.pone.0280173)
Supplement: S3 File — (HTML) [file pone.0280173.s017.html]

XGBoost 101 - Any Health Issues - just decision makers


# XGBoost 101 - Any Health Issues - just decision makers

# SETUP

#Functions

```
head(ml)
```

```
## # A tibble: 6 × 37
##   Location       setting  Urban Education Education_S Education_S2 Animal_Career
##   <fct>          <fct>    <chr> <ord>     <chr>       <fct>        <chr>        
## 1 UK             Equally… No    3_Grad    2_Grad      2_Grad       None of the …
## 2 UK             Rural    No    2_College 1_College   1_College    None of the …
## 3 UK             Equally… No    1_HighSc… 0_Basic_or… 0_Basic_or_… None of the …
## 4 Other European Urban    Yes   3_Grad    2_Grad      2_Grad       None of the …
## 5 UK             Equally… No    2_College 1_College   1_College    None of the …
## 6 UK             Urban    Yes   1_HighSc… 0_Basic_or… 0_Basic_or_… None of the …
## # ℹ 30 more variables: Animal_Career2 <fct>, Animal_Career_BINARY <chr>,
## #   Income <ord>, Income2 <fct>, C_Age <ord>, C_Age2 <fct>, C_Gender <fct>,
## #   C_Diet <chr>, C_Diet_Vegan <chr>, C_Diet_Vegan_Veggie <chr>, D_Age <dbl>,
## #   D_Age_quant <fct>, Size <ord>, Size2 <fct>, Size_Giant <chr>, D_Sex <fct>,
## #   D_Neuter <fct>, Therapeutic_Food <chr>, Meds <fct>, D_Diet <chr>,
## #   D_Diet_Vegan <chr>, D_Diet_Vegan_Veggie <chr>, D_Diet_Raw <chr>,
## #   Visits <chr>, Visits2 <chr>, Health <ord>, Health2 <ord>, …
```

```
nicenames <- function(strings, find, replacement) {
    # replace cumbersome names with nice ones
    strings[grep(find, strings)] <- replacement
    strings
}

fprint <- function(data) {
    print(data)
    if (is.data.frame(data)) {
        write.table(data, "status.txt", append = TRUE)
    } else {
        write(data, "status.txt", append = TRUE)
    }
}
```

# XGBoost ALL FEATURES

## Pre-process to numeric

```
fprint(paste("\n\n\nBEGINNING THE FULL MODE WITH:", health, "_",
    version))
```

```
## [1] "\n\n\nBEGINNING THE FULL MODE WITH: Any _ 101"
```

```
# Remove these models from the environment in case
# confusion later.  If they don't exist a warning is
# displayed but code continues to run
suppressWarnings(rm(cv_res))
suppressWarnings(rm(bstSparse))

locations <- dplyr::select(ml, Location)
locations <- one_hot(as.data.table(locations), dropUnusedLevels = TRUE)
factors <- cbind(locations)  #legacy line!

fnames <- names(factors)
fnames <- nicenames(fnames, "European", "European")
fnames <- nicenames(fnames, "UK", "UK")
fnames <- nicenames(fnames, "Oceania", "Oceania")
fnames <- nicenames(fnames, "America", "America")
fnames <- nicenames(fnames, "Other", "Other")

names(factors) <- fnames

if (tolower(DAge == "quant")) {
    ordered <- as.data.frame(cbind(ordered(ml$Income), ordered(ml$Education),
        ordered(ml$D_Age_quant), ordered(ml$C_Age), ordered(ml$Size),
        factor(ml$Visits, levels = c("0", "1", "2", "3", "3<"),
            order = TRUE)))

} else {
    ordered <- as.data.frame(cbind(ml$Income, ml$Education, ml$D_Age,
        ml$C_Age, ml$Size, factor(ml$Visits, levels = c("0",
            "1", "2", "3", "3<"), order = TRUE)))
}

names(ordered) <- c("Income", "Education", "Dog Age", "Owner Age",
    "Size", "Visits")

numeric <- dplyr::select(ml, C_Gender)
numeric$C_Gender <- as.numeric(numeric$C_Gender) - 1
numeric$Urban <- as.numeric(factor(ml$Urban, levels = c("No",
    "Yes"), order = TRUE)) - 1
numeric$D_Sex <- as.numeric(ml$D_Sex) - 1
numeric$Neuter <- as.numeric(ml$D_Neuter) - 1
numeric$Meds <- as.numeric(ml$Meds) - 1
numeric$D_Diet_Vegan <- as.numeric(as.factor(ml$D_Diet_Vegan)) -
    1
numeric$D_Diet_Raw <- as.numeric(as.factor(ml$D_Diet_Raw)) -
    1
numeric$C_Diet_Vegan <- as.numeric(as.factor(ml$C_Diet_Vegan)) -
    1

numeric$BIN_Animal_Career <- as.numeric(as.factor(ml$Animal_Career_BINARY)) -
    1
numeric$Therapeutic_Food <- as.numeric(as.factor(ml$Therapeutic_Food)) -
    1
if (tolower(health) == "any") {
    numeric$Health_Binary <- ml$Any_Health_Problem
} else if (tolower(health) == "severe") {
    numeric$Health_Binary <- ml$Health_Binary
} else {
    stop(paste("OOPS ERROR, YOU'RE HEALTH VARIABLE WAS", health))
}

names(numeric) <- c("Owner Gender", "Urban", "Dog Sex", "Neuter status",
    "Meds", "Dog Vegan Diet", "Dog Diet Raw", "Owner Vegan Diet",
    "Animal Career", "Therapeutic Food", "Health_Binary")
if (version == "102") {
    Decision <- as.numeric(as.factor(ml$Primary_Decision_Maker))
    Decision <- data.frame(Decision = Decision)
    numeric <- cbind(Decision, numeric)
}

XGdata <- cbind(factors, ordered, numeric)
```

# Pseudo correlation matrix given all data as numeric

threshold for significance removed from corrplots following Reviewer
request sig.level=bht changed to sig.level=1

```
# Only do this with Any Health since idental at this point
if ((tolower(health) == "any") & (corri == TRUE)) {

    WRITE = TRUE
    cdata <- dplyr::select(XGdata, -c("Health_Binary"))
    cdata <- cdata[complete.cases(cdata), ]

    # Old method cor.mat <-
    # round(cor(cdata,use='pairwise.complete.obs',
    # method='kendall'),2)

    # get the correlation matrix using rstatix, slower but
    # allows direct p-val return; and the correlation
    # values itself are identical.
    corMatrix <- rstatix::cor_mat(cdata, method = "kendall")
    # Convert first column to rownames
    corMatrix <- corMatrix %>%
        remove_rownames %>%
        column_to_rownames(var = "rowname")

    # get the p.values
    corMatrix_p <- as.data.frame(corMatrix %>%
        rstatix::cor_get_pval())
    # drop the p-values to allow extra functions
    cor.Mat <- as.matrix(DescTools::StripAttr(corMatrix, attr_names = "pvalue"))

    # Log all but the rownames
    tmpnames <- dplyr::select(corMatrix_p, rowname)
    corMatrix_p <- dplyr::select(corMatrix_p, -rowname)
    lgcorMatrix_p <- log10(corMatrix_p)
    logmatrix <- cbind(tmpnames, round(lgcorMatrix_p, 2))

    if (WRITE) {
        write.csv(corMatrix_p, paste0("corr-p-values_", version,
            ".csv"))
        write.csv(round(corMatrix, 2), paste0("corr-values_",
            version, ".csv"))
        write.csv(logmatrix, paste0("corr-logp-values_", version,
            ".csv"))
    }

    rm(lgcorMatrix_p, tmpnames)

    hc <- hclust(as.dist(1 - cor.Mat), method = "ward.D2")
    # Plot here first

    # Then save figure
    tiff(paste0("figures/hclust_", version, ".tif"), width = 3.25,
        height = 3.25, units = "in", res = 300, pointsize = 4)
    plot(hc, hang = -1, cex = 0.9)
    dev.off()

    # Using Kendall which is good for ranks
    cex = 1.3
    srt = 45
    tmp <- as.matrix(corMatrix_p)
    rownames(tmp) <- rownames(cor.Mat)

    # Calculate a BH threshold Get all the p-values as a
    # Vector, just get the bottom part (don't duplicate)
    ps <- tmp[lower.tri(tmp, diag = FALSE)]
    # Calculating bht would allow us to label the
    # significant correlations Reviewers prefer us to leave
    # in even the insignificant correlations.
    bht <- get_bh_threshold(ps, alpha = 0.05)

    corrplot::corrplot(cor.Mat, p.mat = tmp, type = "lower",
        order = "hclust", hclust.method = "ward.D2", tl.col = "black",
        insig = "blank", sig.level = 1, tl.srt = 45, tl.cex = cex/2,
        na.label = " ", cl.cex = cex/2)

    # Then save figure
    tiff(paste0("figures/HealthBinaryCorPlot_", version, ".tif"),
        width = 3.25, height = 3.25, units = "in", res = 300,
        pointsize = 4)
    par(bg = NA)
    corrplot::corrplot(cor.Mat, p.mat = tmp, type = "lower",
        order = "hclust", hclust.method = "ward.D2", insig = "blank",
        tl.col = "black", sig.level = 1, tl.srt = 45, tl.cex = cex,
        na.label = " ", cl.cex = cex)

    dev.off()
}  #End of the Corriplot routines
```

```
## quartz_off_screen 
##                 2
```

#Functional Importance

```
do_importance <- function(model, RedOrFull) {

    importance_matrix <- xgb.importance(model = model)
    fprint(importance_matrix)
    xgb.plot.importance(importance_matrix = importance_matrix)
    importance_matrix <- importance_matrix %>%
        dplyr::select(-Gain)

    if (BW) {
        tiff(paste0("figures/XGImpBWReduced_Simple_", version,
            health, ".tif"))
        xgb.plot.importance(importance_matrix = importance_matrix)
        dev.off()
    }
    # importance_matrix <-
    # dplyr::select(importance_matrix,c(Feature,Importance,
    # Frequency, Cover))

    m_imp <- reshape::melt(importance_matrix, id_vars = Feature)

    # Check the order of columns is right
    names(m_imp) <- c("Feature", "XGBoost Parameter", "Value")

    tiff(paste0("figures/XGImp", RedOrFull, "_", version, health,
        ".tif"), width = 6.5, height = 3.25, units = "in", res = 300,
        pointsize = 4)
    par(bg = NA)
    p <- ggplot(m_imp, aes(x = reorder(Feature, -Value), y = Value,
        fill = `XGBoost Parameter`)) + geom_bar(position = "stack",
        stat = "identity") + xlab("Feature") + ylab("Value (Au)") +
        theme_bw() + theme(axis.text.x = element_text(angle = 90,
        vjust = 0.5, hjust = 1), legend.position = c(0.98, 0.75),
        legend.justification = "right")
    print(p)
    dev.off()
    print(p)
    max_i <- max(importance_matrix$Importance)
    output <- importance_matrix %>%
        mutate(Percentage = 100 * Importance/max_i)

    write.table(output, "status.csv", sep = ",", row.names = FALSE,
        append = TRUE)
}
```

#Augment Train Data Function

```
augment <- function(trainX, trainY, method) {
    if (method == "SMOTE") {
        train.SMOTE <- SMOTE(trainX, trainY, K = 50, dup_size = 100)
        trainX <- train.SMOTE$data[, 1:ncol(train) - 1]
        trainY <- as.numeric(train.SMOTE$data[, ncol(train)])
        train.SMOTE <- SMOTE(trainX, trainY, K = 5, dup_size = 6)
        trainX <- train.SMOTE$data[, 1:ncol(train) - 1]
        trainY <- as.numeric(train.SMOTE$data[, ncol(train)])
        value <- list(trainX, trainY)
        names(value) <- c("trainX", "trainY")
        return(value)
    } else if (method == "bruteforce") {
        # trouble with this method is it may create extra
        # levels... somehow need to constrain to 0 to max
        # level AND add or subtract but still not go <0.
        # get column max and min
        cmax <- train %>%
            summarise_if(is.numeric, max)
        cmin <- train %>%
            summarise_if(is.numeric, min)
        # Firstly BALANCE the dataset reps set at the top [
        # reps!]
        bigTrain <- train
        classtrain <- subset(train, Health_Binary == 0)
        for (i in 1:reps) {
            AugmentMe <- as.data.frame(matrix(rbinom(ncol(classtrain) *
                nrow(classtrain), 1, 0.2), ncol = ncol(classtrain)))
            names(AugmentMe) <- names(classtrain)
            classtrain <- subset(train, Health_Binary == 0)
            # last column is the outcome, dont change that
            AugmentMe[, ncol(AugmentMe)] <- 0
            if ((reps%%2) == 0) {
                newbatch <- classtrain + AugmentMe
            } else {
                newbatch <- classtrain - AugmentMe
            }

            for (c in 1:ncol(train)) {
                mycol = names(cmax[c])
                newbatch <- subset(newbatch, get(mycol) <= as.numeric(cmax[c]))
                newbatch <- subset(newbatch, get(mycol) >= as.numeric(cmin[c]))
            }
            bigTrain <- rbind(bigTrain, newbatch)
        }
        train <- bigTrain
        # NOW just augment generally.

        bigTrain <- train
        for (i in 1:reps) {
            AugmentMe <- as.data.frame(matrix(rbinom(ncol(train) *
                nrow(train), 1, 0.2), ncol = ncol(train)))
            names(AugmentMe) <- names(train)
            # last column is the outcome, dont change that
            AugmentMe[, ncol(AugmentMe)] <- 0
            if ((reps%%2) == 0) {
                newbatch <- train + AugmentMe
            } else {
                newbatch <- train - AugmentMe
            }

            for (c in 1:ncol(train)) {
                mycol = names(cmax[c])
                newbatch <- subset(newbatch, get(mycol) <= as.numeric(cmax[c]))
                newbatch <- subset(newbatch, get(mycol) >= as.numeric(cmin[c]))
            }
            bigTrain <- rbind(bigTrain, newbatch)
        }
        train <- bigTrain
        trainX <- train[, 1:ncol(train) - 1]
        trainY <- train[, ncol(train)]
        value <- list(trainX, trainY)
        names(value) <- c("trainX", "trainY")
        return(value)
    } else {
        value <- list(trainX, trainY)
        names(value) <- c("trainX", "trainY")
        return(value)

    }
}
```

#Train XGboost FULL

```
# Remove these models from the environment in case
# confusion later.  If they don't exist a warning is
# displayed but code continues to run
if (SKIPFULL == FALSE) {
    fprint(paste("TRAINING XGBOOST WITH THE FULL MODEL WITH:",
        health, "_", version))

    update <- paste("Full Model (Training):", health, "_", version)
    con <- file("status.csv", "a")
    writeLines(update, con)
    close(con)
    suppressWarnings(rm(cv_res))
    suppressWarnings(rm(bstSparse))
    XGdata <- XGdata[complete.cases(XGdata), ]
    # XGdata <- dplyr::select(XGdata, -D_Age_quant )

    # OR Better split with stratification to avoid a
    # potentially empty dependent variable set.
    set.seed(2020)
    XGd <- initial_split(XGdata, prop = 0.7, strata = Health_Binary)

    XGd_valid <- initial_split(training(XGd), prop = 0.7, strata = Health_Binary)

    train <- training(XGd_valid)
    train <- as.data.frame(training(XGd))

    validXG <- testing(XGd_valid)
    train <- as.data.frame(train)
    trainX <- train[, 1:(ncol(train) - 1)]
    trainY <- train[, ncol(train)]
    augments <- augment(trainX, trainY, method = Meth_augment)
    trainX <- augments[["trainX"]]
    trainY <- augments[["trainY"]]

    train <- xgb.DMatrix(as.matrix(trainX), label = trainY)

    validXG <- as.data.frame(validXG)
    validX <- validXG[, 1:(ncol(validXG) - 1)]

    validY <- validXG[, ncol(validXG)]
    valid <- xgb.DMatrix(as.matrix(validX), label = validY)

    # Models work best if training data augmented

    watchlist = list(eval = valid, train = train)

    # Two alternative methods. Bruteforce seems OK.
    length(trainY[trainY == 0])
    length(trainY[trainY > 0])
    # new method
    test <- as.data.frame(testing(XGd))
    testX <- test[, 1:ncol(test) - 1]
    testY <- test[, ncol(test)]
    # weight<-nrow(ml)/(1-sum(ml$Bhealth)) wont work now
    # changed to factor wat above
    weight = 1
    # weight <- weight^0.5 #some say otherwise skews
    param <- list(max.depth = depth, eta = 0.01, nthread = 12,
        objective = "multi:softprob", num_class = 2, min_child_weight = min_child_weight,
        subsample = 0.5, gamma = 0.1, booster = "gbtree")
    #'binary:logistic' or 'multi:softprob' 
    # If using binary:logistic then delete the class number
    # parameter!

    if (nrounds == 0) {
        # This is one quick way to tune, remembering that
        # iterations is kind of the same as number of
        # trees.
        cv_res <- xgb.cv(data = as.matrix(trainX), label = trainY,
            params = param, nrounds = nrounds, early_stopping_rounds = 5,
            print_every_n = 500, nfold = 5, eval_metric = "auc")

        nrounds <- cv_res$best_iteration
    }

    bstSparse <- xgb.train(params = param, data = train, nrounds = nrounds,
        print_every_n = 100, eval_metric = "auc", watchlist = watchlist,
        early_stopping_rounds = 100)

    # Recreate the original train data to avoid confusion
    # later: Never want to accidental mess with the
    # augmented data again.
    train <- as.data.frame(training(XGd))
    trainX <- train[, 1:(ncol(train) - 1)]
    trainY <- train[, ncol(train)]
    #########

    # Facile test on train.... to delete
    pred <- predict(bstSparse, as.matrix(trainX), reshape = TRUE)
    prediction <- as.numeric(pred[, 2] > 0.3)
    rocker <- roc(trainY, pred[, 2])
    confusionMatrix(as.factor(prediction), as.factor(trainY))
    ######
}
```

#ROC FULL

```
if (!SKIPFULL) {
    fprint(paste("\nFULL MODEL ROC WITH:", health, "_", version))

    pred <- predict(bstSparse, as.matrix(testX), reshape = TRUE)
    pred_obj <- prediction(pred[, 2], testY)

    xgb.perf <- performance(pred_obj, "tpr", "fpr")

    # Remember this is the Full model
    if (tolower(health) == "any") {
        pts <- seq(0.1, 0.9, by = 0.1)
    } else if (tolower(health) == "severe") {
        pts <- c(0.14, 0.18, 0.22)
    }

    text = 1.5  # Text size
    par(bg = NA, cex = 1.5/2, cex.axis = 1.5/2, cex.lab = 1.5/2)
    ROCR::plot(xgb.perf, avg = "threshold", colorize = TRUE,
        lwd = 1, main = "XGBoost: Mild, significant or serious illness",
        print.cutoffs.at = pts, cutoff.label.function = function(x) {
            round(x, 2)
        }, text.adj = c(-1, 1), colorkey.relwidth = 1)

    grid(col = "lightgray")
    axis(1, at = seq(0, 1, by = 0.1))
    axis(2, at = seq(0, 1, by = 0.1))
    abline(v = c(0.1, 0.3, 0.5, 0.7, 0.9), col = "lightgray",
        lty = "dotted")
    abline(h = c(0.1, 0.3, 0.5, 0.7, 0.9), col = "lightgray",
        lty = "dotted")
    lines(x = c(0, 1), y = c(0, 1), col = "black", lty = "dotted")

    tiff(paste0("figures/xgROCsimpleFull_", version, health,
        ".tif"), width = 3.25, height = 3.25, units = "in", res = 300,
        pointsize = 4)
    par(bg = NA, cex = 1.5, cex.axis = 1.5, cex.lab = 1.5)
    ROCR::plot(xgb.perf, avg = "threshold", colorize = TRUE,
        lwd = 3, main = "", print.cutoffs.at = pts, cutoff.label.function = function(x) {
            round(x, 2)
        }, text.adj = c(-1, 1), colorkey.relwidth = 1)
    grid(col = "lightgray")
    axis(1, at = seq(0, 1, by = 0.1))
    axis(2, at = seq(0, 1, by = 0.1))
    abline(v = c(0.1, 0.3, 0.5, 0.7, 0.9), col = "lightgray",
        lty = "dotted")
    abline(h = c(0.1, 0.3, 0.5, 0.7, 0.9), col = "lightgray",
        lty = "dotted")
    lines(x = c(0, 1), y = c(0, 1), col = "black", lty = "dotted")
    dev.off()

    res <- pROC::roc(testY, pred[, 2], ci = TRUE, conf.level = 0.99)
    res$auc
    res$ci
    fprint(paste("\nFull model ROC, AUC:", res$auc))
    fprint(paste("99% confidence:", res$ci))
    cis <- paste(round(res$ci, 3), collapse = ":")
    cis <- paste("Full ROC:", health, version, cis)
    con <- file("status.csv", "a")
    writeLines(cis, con)
    close(con)

}
```

```
## [1] "\nFULL MODEL ROC WITH: Any _ 101"
```

```
## Setting levels: control = 0, case = 1
```

```
## Setting direction: controls < cases
```

```
## [1] "\nFull model ROC, AUC: 0.836156584316487"
## [1] "99% confidence: 0.79404066597263"  "99% confidence: 0.836156584316487"
## [3] "99% confidence: 0.878272502660344"
```

# FULL XGboost Prediction

Just an example threshold

```
if (!SKIPFULL) {
    # CHOOSE A THRESHOLD FROM THE ROC
    thresh = 0.3
    pred <- predict(bstSparse, as.matrix(testX), reshape = TRUE)
    prediction <- as.numeric(pred[, 2] > thresh)
    confusionMatrix(as.factor(prediction), as.factor(testY))
}
```

```
## Confusion Matrix and Statistics
## 
##           Reference
## Prediction   0   1
##          0 317  61
##          1  96 190
##                                           
##                Accuracy : 0.7636          
##                  95% CI : (0.7294, 0.7954)
##     No Information Rate : 0.622           
##     P-Value [Acc > NIR] : 5.085e-15       
##                                           
##                   Kappa : 0.5106          
##                                           
##  Mcnemar's Test P-Value : 0.006658        
##                                           
##             Sensitivity : 0.7676          
##             Specificity : 0.7570          
##          Pos Pred Value : 0.8386          
##          Neg Pred Value : 0.6643          
##              Prevalence : 0.6220          
##          Detection Rate : 0.4774          
##    Detection Prevalence : 0.5693          
##       Balanced Accuracy : 0.7623          
##                                           
##        'Positive' Class : 0               
##
```

# Calculate importance FULL

```
if (!SKIPFULL) {
    fprint(paste("\nIMPORTANCE WITH FULL MODEL:", health, "_",
        version))

    do_importance(bstSparse, "Full")
}
```

```
## [1] "\nIMPORTANCE WITH FULL MODEL: Any _ 101"
##              Feature         Gain       Cover    Frequency
##  1:             Meds 0.3928450491 0.128511728 0.0648267496
##  2:          Dog Age 0.1750915620 0.182359312 0.1878838426
##  3:           Visits 0.1531162040 0.180563150 0.1552809159
##  4:        Owner Age 0.0476762402 0.082507848 0.1063007051
##  5:             Size 0.0464316143 0.069819319 0.0868905906
##  6:        Education 0.0443918195 0.077676141 0.1034953370
##  7:          Dog Sex 0.0289491413 0.040875635 0.0732428539
##  8:               UK 0.0173911391 0.027473051 0.0392751535
##  9:     Dog Diet Raw 0.0147524565 0.025741087 0.0316930776
## 10:            Urban 0.0133433784 0.024929757 0.0319205398
## 11:           Income 0.0130704537 0.034680652 0.0257790583
## 12:    Neuter status 0.0116705885 0.028233433 0.0273712943
## 13:     Owner Gender 0.0116619635 0.029621974 0.0128137084
## 14: Owner Vegan Diet 0.0094320320 0.018453691 0.0178936993
## 15:   Dog Vegan Diet 0.0084771763 0.020741866 0.0131928122
## 16:    Animal Career 0.0070239333 0.015805909 0.0140268405
## 17:         European 0.0042275688 0.010779780 0.0075820760
## 18:          America 0.0004476794 0.001225666 0.0005307453
```

```
## Warning in write.table(data, "status.txt", append = TRUE): appending column
## names to file
```

```
## Using Feature as id variables
```

```
## Warning in write.table(output, "status.csv", sep = ",", row.names = FALSE, :
## appending column names to file
```

#Reduced XGBoost Now XGBoost on the data without meds and visits or
therapeutic foods ##Simple XGBoost on reduced variable set, still Any
Health Issue binary # Preprocess reduced XGBoost

```
suppressWarnings(rm(cv_res))
suppressWarnings(rm(bstSparse))
fprint(paste("\n\n\nBEGINNING A REDUCED MODEL WITH:", health,
    "_", version))
locations <- dplyr::select(ml, Location)
locations <- one_hot(as.data.table(locations), dropUnusedLevels = TRUE)

factors <- cbind(locations)

fnames <- names(factors)
fnames <- nicenames(fnames, "European", "European")
fnames <- nicenames(fnames, "UK", "UK")
fnames <- nicenames(fnames, "Oceania", "Oceania")
fnames <- nicenames(fnames, "America", "America")
fnames <- nicenames(fnames, "Other", "Other")

names(factors) <- fnames

if (tolower(DAge == "quant")) {
    ordered <- as.data.frame(cbind(ml$Income, ml$Education, ordered(ml$D_Age_quant),
        ml$C_Age, ml$Size))

} else {
    ordered <- as.data.frame(cbind(ml$Income, ml$Education, ml$D_Age,
        ml$C_Age, ml$Size))
}

names(ordered) <- c("Income", "Education", "Dog Age", "Owner Age",
    "Size")

numeric <- dplyr::select(ml, C_Gender)
numeric$C_Gender <- as.numeric(numeric$C_Gender) - 1
numeric$Urban <- as.numeric(factor(ml$Urban, levels = c("No",
    "Yes"), order = TRUE)) - 1
numeric$D_Sex <- as.numeric(ml$D_Sex) - 1
numeric$Neuter <- as.numeric(ml$D_Neuter) - 1
numeric$D_Diet_Vegan <- as.numeric(as.factor(ml$D_Diet_Vegan)) -
    1

numeric$D_Diet_Raw <- as.numeric(as.factor(ml$D_Diet_Raw)) -
    1
numeric$C_Diet_Vegan <- as.numeric(as.factor(ml$C_Diet_Vegan)) -
    1

numeric$BIN_Animal_Career <- as.numeric(as.factor(ml$Animal_Career_BINARY)) -
    1
if (tolower(health) == "any") {
    numeric$Health_Binary <- ml$Any_Health_Problem
} else if (tolower(health) == "severe") {
    numeric$Health_Binary <- as.numeric(ml$Health_Binary)
} else {
    stop(paste("OOPS ERROR, YOU'RE HEALTH VARIABLE WAS", health))
}

names(numeric) <- c("Owner Gender", "Urban", "Dog Sex", "Neuter status",
    "Dog Vegan Diet", "Dog Diet Raw", "Owner Vegan Diet", "Animal Career",
    "Health_Binary")

if (version == "102") {
    Decision <- as.numeric(as.factor(ml$Primary_Decision_Maker))
    Decision <- data.frame(Decision = Decision)
    numeric <- cbind(Decision, numeric)
}

XGdata <- cbind(factors, ordered, numeric)
# XGdata$Health_Binary <- XGdata$Health_Binary-1
```

#Train Reduced XG

```
set.seed(2020)

update <- paste("Reduced Model (Training):", health, "_", version)
con <- file("status.csv", "a")
writeLines(update, con)
close(con)

XGd <- initial_split(XGdata, prop = 0.7, strata = Health_Binary)

XGd_valid <- initial_split(training(XGd), prop = 0.7, strata = Health_Binary)

train <- training(XGd_valid)
train <- as.data.frame(training(XGd))

validXG <- testing(XGd_valid)
train <- as.data.frame(train)
trainX <- train[, 1:(ncol(train) - 1)]
trainY <- train[, ncol(train)]
augments <- augment(trainX, trainY, method = Meth_augment)
trainX <- augments[["trainX"]]
trainY <- augments[["trainY"]]

train <- xgb.DMatrix(as.matrix(trainX), label = trainY)

validXG <- as.data.frame(validXG)
validX <- validXG[, 1:(ncol(validXG) - 1)]

validY <- validXG[, ncol(validXG)]
valid <- xgb.DMatrix(as.matrix(validX), label = validY)

# Models work best if training data augmented

watchlist = list(eval = valid, train = train)

# Two alternative methods. Bruteforce seems OK.
length(trainY[trainY == 0])
```

```
## [1] 2370
```

```
length(trainY[trainY > 0])
```

```
## [1] 910
```

```
# new method
test <- as.data.frame(testing(XGd))
testX <- test[, 1:ncol(test) - 1]
testY <- test[, ncol(test)]
# weight<-nrow(ml)/(1-sum(ml$Bhealth)) wont work now
# changed to factor wat above
weight = 1
# weight <- weight^0.5 #some say otherwise skews
param <- list(max.depth = depth, eta = 0.01, nthread = 10, objective = "multi:softprob",
    num_class = 2, min_child_weight = min_child_weight, subsample = 0.5,
    gamma = 0.1, booster = "gbtree")
#'binary:logistic' or 'multi:softprob' 
# If using binary:logistic then delete the class number
# parameter!

if (nrounds == 0) {
    # This is one quick way to tune, remembering that
    # iterations is kind of the same as number of trees.
    cv_res <- xgb.cv(data = as.matrix(trainX), label = trainY,
        params = param, nrounds = 2000, early_stopping_rounds = 5,
        print_every_n = 500, nfold = 5, eval_metric = "auc")

    nrounds <- cv_res$best_iteration
}

bstSparse <- xgb.train(params = param, data = train, nrounds = nrounds,
    print_every_n = 100, eval_metric = "auc", watchlist = watchlist,
    early_stopping_rounds = 100)
```

```
## [1]  eval-auc:0.674229   train-auc:0.683235 
## Multiple eval metrics are present. Will use train_auc for early stopping.
## Will train until train_auc hasn't improved in 100 rounds.
## 
## [101]    eval-auc:0.686468   train-auc:0.733386 
## [201]    eval-auc:0.697910   train-auc:0.746570 
## [301]    eval-auc:0.707563   train-auc:0.758750 
## [401]    eval-auc:0.718357   train-auc:0.770253 
## [501]    eval-auc:0.727224   train-auc:0.779647 
## [600]    eval-auc:0.735166   train-auc:0.788750
```

```
# Recreate the original train data to avoid confusion
# later: Never want to accidental mess with the augmented
# data again.
train <- as.data.frame(training(XGd))
trainX <- train[, 1:(ncol(train) - 1)]
trainY <- train[, ncol(train)]
#########
```

#ROC reduced

```
fprint(paste("\n\n\nREDUCED MODEL ROC WITH:", health, "_", version))
```

```
## [1] "\n\n\nREDUCED MODEL ROC WITH: Any _ 101"
```

```
pred <- predict(bstSparse, as.matrix(testX), reshape = TRUE)
pred_obj <- prediction(pred[, 2], testY)

xgb.perf <- performance(pred_obj, "tpr", "fpr")
# Remember this is the reduced model
if (tolower(health) == "any") {
    pts <- seq(0.2, 0.6, by = 0.1)
} else if (tolower(health) == "severe") {
    pts <- c(0.1, 0.06, 0.05)  # seq(0.10, 0.18, by=0.02)
} else pts <- c()  # empty vector safest
text = 1.5  # Text size
par(bg = NA, cex = 1.5/2, cex.axis = 1.5/2, cex.lab = 1.5/2)
ROCR::plot(xgb.perf, avg = "threshold", colorize = TRUE, lwd = 1,
    main = paste("XGBoost:Reduced:", health), print.cutoffs.at = pts,
    cutoff.label.function = function(x) {
        round(x, 3)
    }, text.adj = c(-0.5, 0.9), text.cex = 0.5)
grid(col = "lightgray")
axis(1, at = seq(0, 1, by = 0.1))
axis(2, at = seq(0, 1, by = 0.1))
abline(v = c(0.1, 0.3, 0.5, 0.7, 0.9), col = "lightgray", lty = "dotted")
abline(h = c(0.1, 0.3, 0.5, 0.7, 0.9), col = "lightgray", lty = "dotted")
lines(x = c(0, 1), y = c(0, 1), col = "black", lty = "dotted")
```

```
tiff(paste0("figures/XGROCsimpleReduced_", version, health, ".tif"),
    width = 3.25, height = 3.25, units = "in", res = 300, pointsize = 4)
par(bg = NA, cex = 1.5, cex.axis = 1.5, cex.lab = 1.5)
ROCR::plot(xgb.perf, avg = "threshold", colorize = TRUE, lwd = 3,
    main = "", print.cutoffs.at = pts, cutoff.label.function = function(x) {
        round(x, 3)
    }, text.adj = c(-0.3, 1.3), colorkey.relwidth = 1)
grid(col = "lightgray")
axis(1, at = seq(0, 1, by = 0.1))
axis(2, at = seq(0, 1, by = 0.1))
abline(v = c(0.1, 0.3, 0.5, 0.7, 0.9), col = "lightgray", lty = "dotted")
abline(h = c(0.1, 0.3, 0.5, 0.7, 0.9), col = "lightgray", lty = "dotted")
lines(x = c(0, 1), y = c(0, 1), col = "black", lty = "dotted")
dev.off()
```

```
## quartz_off_screen 
##                 2
```

```
res <- pROC::roc(testY, pred[, 2], ci = TRUE, conf.level = 0.99)
```

```
## Setting levels: control = 0, case = 1
```

```
## Setting direction: controls < cases
```

```
res$auc
```

```
## Area under the curve: 0.674
```

```
res$ci
```

```
## 99% CI: 0.6166-0.7313 (DeLong)
```

```
fprint(paste("Reduced model ROC, AUC:", res$auc))
```

```
## [1] "Reduced model ROC, AUC: 0.673962744662995"
```

```
fprint(paste("99% confidence:", res$ci))
```

```
## [1] "99% confidence: 0.616598086618907" "99% confidence: 0.673962744662995"
## [3] "99% confidence: 0.731327402707082"
```

```
cis <- paste(round(res$ci, 3), collapse = ":")
cis <- paste("Reduced ROC:", health, version, cis)
con <- file("status.csv", "a")
writeLines(cis, con)
close(con)
```

# XGboostPrediction REDUCED

```
# CHOOSE A THRESHOLD FROM THE ROC
thresh = 0.775
pred <- predict(bstSparse, as.matrix(testX), reshape = TRUE)
prediction <- as.numeric(pred[, 2] > thresh)
confusionMatrix(factor(prediction, levels = c(0, 1)), as.factor(testY))
```

```
## Confusion Matrix and Statistics
## 
##           Reference
## Prediction   0   1
##          0 413 251
##          1   0   0
##                                          
##                Accuracy : 0.622          
##                  95% CI : (0.5839, 0.659)
##     No Information Rate : 0.622          
##     P-Value [Acc > NIR] : 0.5173         
##                                          
##                   Kappa : 0              
##                                          
##  Mcnemar's Test P-Value : <2e-16         
##                                          
##             Sensitivity : 1.000          
##             Specificity : 0.000          
##          Pos Pred Value : 0.622          
##          Neg Pred Value :   NaN          
##              Prevalence : 0.622          
##          Detection Rate : 0.622          
##    Detection Prevalence : 1.000          
##       Balanced Accuracy : 0.500          
##                                          
##        'Positive' Class : 0              
##
```

# Calculate importance REDUCED

```
do_importance(bstSparse, "Reduced")
```

```
##              Feature         Gain       Cover    Frequency
##  1:          Dog Age 0.2612961889 0.195596855 0.1570613576
##  2:        Owner Age 0.1148178006 0.123016369 0.1411689370
##  3:        Education 0.0947007687 0.089118350 0.1242286646
##  4:             Size 0.0906022013 0.099484135 0.1081033881
##  5:    Neuter status 0.0604413165 0.062280855 0.0472115497
##  6:     Dog Diet Raw 0.0543083395 0.048253587 0.0603679124
##  7:          Dog Sex 0.0523145116 0.044600521 0.0797531727
##  8:               UK 0.0408392984 0.037468918 0.0492490395
##  9:            Urban 0.0401578819 0.042181729 0.0490743975
## 10:   Dog Vegan Diet 0.0394364333 0.044543369 0.0307952032
## 11:           Income 0.0373973061 0.051054440 0.0436604960
## 12: Owner Vegan Diet 0.0370418183 0.045238779 0.0352194668
## 13:    Animal Career 0.0297479825 0.034040138 0.0301548492
## 14:     Owner Gender 0.0226933634 0.039788198 0.0164745605
## 15:         European 0.0165737238 0.023456179 0.0201420421
## 16:          America 0.0068386205 0.017575638 0.0064617534
## 17:          Oceania 0.0007924448 0.002301938 0.0008732099
```

```
## Warning in write.table(data, "status.txt", append = TRUE): appending column
## names to file
```

```
## Using Feature as id variables
```

```
## Warning in write.table(output, "status.csv", sep = ",", row.names = FALSE, :
## appending column names to file
```
