# Supplementary material for: Variables associated with owner perceptions of the health of their dog: Further analysis of data from a large international survey
Source: PLoS One. 2024 May 15;19(5):e0280173. doi: 10.1371/journal.pone.0280173 (PMC11095744; doi:10.1371/journal.pone.0280173)
Supplement: S5 File — (HTML) [file pone.0280173.s019.html]

XGBoost 101 - Significant Health Issues - just decision makers


# XGBoost 101 - Significant Health Issues - just decision makers

# SETUP

#Functions

```
head(ml)
```

```
## # A tibble: 6 × 37
##   Location       setting  Urban Education Education_S Education_S2 Animal_Career
##   <fct>          <fct>    <chr> <ord>     <chr>       <fct>        <chr>        
## 1 UK             Equally… No    3_Grad    2_Grad      2_Grad       None of the …
## 2 UK             Rural    No    2_College 1_College   1_College    None of the …
## 3 UK             Equally… No    1_HighSc… 0_Basic_or… 0_Basic_or_… None of the …
## 4 Other European Urban    Yes   3_Grad    2_Grad      2_Grad       None of the …
## 5 UK             Equally… No    2_College 1_College   1_College    None of the …
## 6 UK             Urban    Yes   1_HighSc… 0_Basic_or… 0_Basic_or_… None of the …
## # ℹ 30 more variables: Animal_Career2 <fct>, Animal_Career_BINARY <chr>,
## #   Income <ord>, Income2 <fct>, C_Age <ord>, C_Age2 <fct>, C_Gender <fct>,
## #   C_Diet <chr>, C_Diet_Vegan <chr>, C_Diet_Vegan_Veggie <chr>, D_Age <dbl>,
## #   D_Age_quant <fct>, Size <ord>, Size2 <fct>, Size_Giant <chr>, D_Sex <fct>,
## #   D_Neuter <fct>, Therapeutic_Food <chr>, Meds <fct>, D_Diet <chr>,
## #   D_Diet_Vegan <chr>, D_Diet_Vegan_Veggie <chr>, D_Diet_Raw <chr>,
## #   Visits <chr>, Visits2 <chr>, Health <ord>, Health2 <ord>, …
```

```
nicenames <- function(strings, find, replacement) {
    # replace cumbersome names with nice ones
    strings[grep(find, strings)] <- replacement
    strings
}

fprint <- function(data) {
    print(data)
    if (is.data.frame(data)) {
        write.table(data, "status.txt", append = TRUE)
    } else {
        write(data, "status.txt", append = TRUE)
    }
}
```

# XGBoost ALL FEATURES

## Pre-process to numeric

```
fprint(paste("\n\n\nBEGINNING THE FULL MODE WITH:", health, "_",
    version))
```

```
## [1] "\n\n\nBEGINNING THE FULL MODE WITH: Severe _ 101"
```

```
# Remove these models from the environment in case
# confusion later.  If they don't exist a warning is
# displayed but code continues to run
suppressWarnings(rm(cv_res))
suppressWarnings(rm(bstSparse))

locations <- dplyr::select(ml, Location)
locations <- one_hot(as.data.table(locations), dropUnusedLevels = TRUE)
factors <- cbind(locations)  #legacy line!

fnames <- names(factors)
fnames <- nicenames(fnames, "European", "European")
fnames <- nicenames(fnames, "UK", "UK")
fnames <- nicenames(fnames, "Oceania", "Oceania")
fnames <- nicenames(fnames, "America", "America")
fnames <- nicenames(fnames, "Other", "Other")

names(factors) <- fnames

if (tolower(DAge == "quant")) {
    ordered <- as.data.frame(cbind(ordered(ml$Income), ordered(ml$Education),
        ordered(ml$D_Age_quant), ordered(ml$C_Age), ordered(ml$Size),
        factor(ml$Visits, levels = c("0", "1", "2", "3", "3<"),
            order = TRUE)))

} else {
    ordered <- as.data.frame(cbind(ml$Income, ml$Education, ml$D_Age,
        ml$C_Age, ml$Size, factor(ml$Visits, levels = c("0",
            "1", "2", "3", "3<"), order = TRUE)))
}

names(ordered) <- c("Income", "Education", "Dog Age", "Owner Age",
    "Size", "Visits")

numeric <- dplyr::select(ml, C_Gender)
numeric$C_Gender <- as.numeric(numeric$C_Gender) - 1
numeric$Urban <- as.numeric(factor(ml$Urban, levels = c("No",
    "Yes"), order = TRUE)) - 1
numeric$D_Sex <- as.numeric(ml$D_Sex) - 1
numeric$Neuter <- as.numeric(ml$D_Neuter) - 1
numeric$Meds <- as.numeric(ml$Meds) - 1
numeric$D_Diet_Vegan <- as.numeric(as.factor(ml$D_Diet_Vegan)) -
    1
numeric$D_Diet_Raw <- as.numeric(as.factor(ml$D_Diet_Raw)) -
    1
numeric$C_Diet_Vegan <- as.numeric(as.factor(ml$C_Diet_Vegan)) -
    1

numeric$BIN_Animal_Career <- as.numeric(as.factor(ml$Animal_Career_BINARY)) -
    1
numeric$Therapeutic_Food <- as.numeric(as.factor(ml$Therapeutic_Food)) -
    1
if (tolower(health) == "any") {
    numeric$Health_Binary <- ml$Any_Health_Problem
} else if (tolower(health) == "severe") {
    numeric$Health_Binary <- ml$Health_Binary
} else {
    stop(paste("OOPS ERROR, YOU'RE HEALTH VARIABLE WAS", health))
}

names(numeric) <- c("Owner Gender", "Urban", "Dog Sex", "Neuter status",
    "Meds", "Dog Vegan Diet", "Dog Diet Raw", "Owner Vegan Diet",
    "Animal Career", "Therapeutic Food", "Health_Binary")
if (version == "102") {
    Decision <- as.numeric(as.factor(ml$Primary_Decision_Maker))
    Decision <- data.frame(Decision = Decision)
    numeric <- cbind(Decision, numeric)
}

XGdata <- cbind(factors, ordered, numeric)
```

# Pseudo correlation matrix given all data as numeric

threshold for significance removed from corrplots following Reviewer
request sig.level=bht changed to sig.level=1

```
# Only do this with Any Health since idental at this point
if ((tolower(health) == "any") & (corri == TRUE)) {

    WRITE = TRUE
    cdata <- dplyr::select(XGdata, -c("Health_Binary"))
    cdata <- cdata[complete.cases(cdata), ]

    # Old method cor.mat <-
    # round(cor(cdata,use='pairwise.complete.obs',
    # method='kendall'),2)

    # get the correlation matrix using rstatix, slower but
    # allows direct p-val return; and the correlation
    # values itself are identical.
    corMatrix <- rstatix::cor_mat(cdata, method = "kendall")
    # Convert first column to rownames
    corMatrix <- corMatrix %>%
        remove_rownames %>%
        column_to_rownames(var = "rowname")

    # get the p.values
    corMatrix_p <- as.data.frame(corMatrix %>%
        rstatix::cor_get_pval())
    # drop the p-values to allow extra functions
    cor.Mat <- as.matrix(DescTools::StripAttr(corMatrix, attr_names = "pvalue"))

    # Log all but the rownames
    tmpnames <- dplyr::select(corMatrix_p, rowname)
    corMatrix_p <- dplyr::select(corMatrix_p, -rowname)
    lgcorMatrix_p <- log10(corMatrix_p)
    logmatrix <- cbind(tmpnames, round(lgcorMatrix_p, 2))

    if (WRITE) {
        write.csv(corMatrix_p, paste0("corr-p-values_", version,
            ".csv"))
        write.csv(round(corMatrix, 2), paste0("corr-values_",
            version, ".csv"))
        write.csv(logmatrix, paste0("corr-logp-values_", version,
            ".csv"))
    }

    rm(lgcorMatrix_p, tmpnames)

    hc <- hclust(as.dist(1 - cor.Mat), method = "ward.D2")
    # Plot here first

    # Then save figure
    tiff(paste0("figures/hclust_", version, ".tif"), width = 3.25,
        height = 3.25, units = "in", res = 300, pointsize = 4)
    plot(hc, hang = -1, cex = 0.9)
    dev.off()

    # Using Kendall which is good for ranks
    cex = 1.3
    srt = 45
    tmp <- as.matrix(corMatrix_p)
    rownames(tmp) <- rownames(cor.Mat)

    # Calculate a BH threshold Get all the p-values as a
    # Vector, just get the bottom part (don't duplicate)
    ps <- tmp[lower.tri(tmp, diag = FALSE)]
    # Calculating bht would allow us to label the
    # significant correlations Reviewers prefer us to leave
    # in even the insignificant correlations.
    bht <- get_bh_threshold(ps, alpha = 0.05)

    corrplot::corrplot(cor.Mat, p.mat = tmp, type = "lower",
        order = "hclust", hclust.method = "ward.D2", tl.col = "black",
        insig = "blank", sig.level = 1, tl.srt = 45, tl.cex = cex/2,
        na.label = " ", cl.cex = cex/2)

    # Then save figure
    tiff(paste0("figures/HealthBinaryCorPlot_", version, ".tif"),
        width = 3.25, height = 3.25, units = "in", res = 300,
        pointsize = 4)
    par(bg = NA)
    corrplot::corrplot(cor.Mat, p.mat = tmp, type = "lower",
        order = "hclust", hclust.method = "ward.D2", insig = "blank",
        tl.col = "black", sig.level = 1, tl.srt = 45, tl.cex = cex,
        na.label = " ", cl.cex = cex)

    dev.off()
}  #End of the Corriplot routines
```

#Functional Importance

```
do_importance <- function(model, RedOrFull) {

    importance_matrix <- xgb.importance(model = model)
    fprint(importance_matrix)
    xgb.plot.importance(importance_matrix = importance_matrix)
    importance_matrix <- importance_matrix %>%
        dplyr::select(-Gain)

    if (BW) {
        tiff(paste0("figures/XGImpBWReduced_Simple_", version,
            health, ".tif"))
        xgb.plot.importance(importance_matrix = importance_matrix)
        dev.off()
    }
    # importance_matrix <-
    # dplyr::select(importance_matrix,c(Feature,Importance,
    # Frequency, Cover))

    m_imp <- reshape::melt(importance_matrix, id_vars = Feature)

    # Check the order of columns is right
    names(m_imp) <- c("Feature", "XGBoost Parameter", "Value")

    tiff(paste0("figures/XGImp", RedOrFull, "_", version, health,
        ".tif"), width = 6.5, height = 3.25, units = "in", res = 300,
        pointsize = 4)
    par(bg = NA)
    p <- ggplot(m_imp, aes(x = reorder(Feature, -Value), y = Value,
        fill = `XGBoost Parameter`)) + geom_bar(position = "stack",
        stat = "identity") + xlab("Feature") + ylab("Value (Au)") +
        theme_bw() + theme(axis.text.x = element_text(angle = 90,
        vjust = 0.5, hjust = 1), legend.position = c(0.98, 0.75),
        legend.justification = "right")
    print(p)
    dev.off()
    print(p)
    max_i <- max(importance_matrix$Importance)
    output <- importance_matrix %>%
        mutate(Percentage = 100 * Importance/max_i)

    write.table(output, "status.csv", sep = ",", row.names = FALSE,
        append = TRUE)
}
```

#Augment Train Data Function

```
augment <- function(trainX, trainY, method) {
    if (method == "SMOTE") {
        train.SMOTE <- SMOTE(trainX, trainY, K = 50, dup_size = 100)
        trainX <- train.SMOTE$data[, 1:ncol(train) - 1]
        trainY <- as.numeric(train.SMOTE$data[, ncol(train)])
        train.SMOTE <- SMOTE(trainX, trainY, K = 5, dup_size = 6)
        trainX <- train.SMOTE$data[, 1:ncol(train) - 1]
        trainY <- as.numeric(train.SMOTE$data[, ncol(train)])
        value <- list(trainX, trainY)
        names(value) <- c("trainX", "trainY")
        return(value)
    } else if (method == "bruteforce") {
        # trouble with this method is it may create extra
        # levels... somehow need to constrain to 0 to max
        # level AND add or subtract but still not go <0.
        # get column max and min
        cmax <- train %>%
            summarise_if(is.numeric, max)
        cmin <- train %>%
            summarise_if(is.numeric, min)
        # Firstly BALANCE the dataset reps set at the top [
        # reps!]
        bigTrain <- train
        classtrain <- subset(train, Health_Binary == 0)
        for (i in 1:reps) {
            AugmentMe <- as.data.frame(matrix(rbinom(ncol(classtrain) *
                nrow(classtrain), 1, 0.2), ncol = ncol(classtrain)))
            names(AugmentMe) <- names(classtrain)
            classtrain <- subset(train, Health_Binary == 0)
            # last column is the outcome, dont change that
            AugmentMe[, ncol(AugmentMe)] <- 0
            if ((reps%%2) == 0) {
                newbatch <- classtrain + AugmentMe
            } else {
                newbatch <- classtrain - AugmentMe
            }

            for (c in 1:ncol(train)) {
                mycol = names(cmax[c])
                newbatch <- subset(newbatch, get(mycol) <= as.numeric(cmax[c]))
                newbatch <- subset(newbatch, get(mycol) >= as.numeric(cmin[c]))
            }
            bigTrain <- rbind(bigTrain, newbatch)
        }
        train <- bigTrain
        # NOW just augment generally.

        bigTrain <- train
        for (i in 1:reps) {
            AugmentMe <- as.data.frame(matrix(rbinom(ncol(train) *
                nrow(train), 1, 0.2), ncol = ncol(train)))
            names(AugmentMe) <- names(train)
            # last column is the outcome, dont change that
            AugmentMe[, ncol(AugmentMe)] <- 0
            if ((reps%%2) == 0) {
                newbatch <- train + AugmentMe
            } else {
                newbatch <- train - AugmentMe
            }

            for (c in 1:ncol(train)) {
                mycol = names(cmax[c])
                newbatch <- subset(newbatch, get(mycol) <= as.numeric(cmax[c]))
                newbatch <- subset(newbatch, get(mycol) >= as.numeric(cmin[c]))
            }
            bigTrain <- rbind(bigTrain, newbatch)
        }
        train <- bigTrain
        trainX <- train[, 1:ncol(train) - 1]
        trainY <- train[, ncol(train)]
        value <- list(trainX, trainY)
        names(value) <- c("trainX", "trainY")
        return(value)
    } else {
        value <- list(trainX, trainY)
        names(value) <- c("trainX", "trainY")
        return(value)

    }
}
```

#Train XGboost FULL

```
# Remove these models from the environment in case
# confusion later.  If they don't exist a warning is
# displayed but code continues to run
if (SKIPFULL == FALSE) {
    fprint(paste("TRAINING XGBOOST WITH THE FULL MODEL WITH:",
        health, "_", version))

    update <- paste("Full Model (Training):", health, "_", version)
    con <- file("status.csv", "a")
    writeLines(update, con)
    close(con)
    suppressWarnings(rm(cv_res))
    suppressWarnings(rm(bstSparse))
    XGdata <- XGdata[complete.cases(XGdata), ]
    # XGdata <- dplyr::select(XGdata, -D_Age_quant )

    # OR Better split with stratification to avoid a
    # potentially empty dependent variable set.
    set.seed(2020)
    XGd <- initial_split(XGdata, prop = 0.7, strata = Health_Binary)

    XGd_valid <- initial_split(training(XGd), prop = 0.7, strata = Health_Binary)

    train <- training(XGd_valid)
    train <- as.data.frame(training(XGd))

    validXG <- testing(XGd_valid)
    train <- as.data.frame(train)
    trainX <- train[, 1:(ncol(train) - 1)]
    trainY <- train[, ncol(train)]
    augments <- augment(trainX, trainY, method = Meth_augment)
    trainX <- augments[["trainX"]]
    trainY <- augments[["trainY"]]

    train <- xgb.DMatrix(as.matrix(trainX), label = trainY)

    validXG <- as.data.frame(validXG)
    validX <- validXG[, 1:(ncol(validXG) - 1)]

    validY <- validXG[, ncol(validXG)]
    valid <- xgb.DMatrix(as.matrix(validX), label = validY)

    # Models work best if training data augmented

    watchlist = list(eval = valid, train = train)

    # Two alternative methods. Bruteforce seems OK.
    length(trainY[trainY == 0])
    length(trainY[trainY > 0])
    # new method
    test <- as.data.frame(testing(XGd))
    testX <- test[, 1:ncol(test) - 1]
    testY <- test[, ncol(test)]
    # weight<-nrow(ml)/(1-sum(ml$Bhealth)) wont work now
    # changed to factor wat above
    weight = 1
    # weight <- weight^0.5 #some say otherwise skews
    param <- list(max.depth = depth, eta = 0.01, nthread = 12,
        objective = "multi:softprob", num_class = 2, min_child_weight = min_child_weight,
        subsample = 0.5, gamma = 0.1, booster = "gbtree")
    #'binary:logistic' or 'multi:softprob' 
    # If using binary:logistic then delete the class number
    # parameter!

    if (nrounds == 0) {
        # This is one quick way to tune, remembering that
        # iterations is kind of the same as number of
        # trees.
        cv_res <- xgb.cv(data = as.matrix(trainX), label = trainY,
            params = param, nrounds = nrounds, early_stopping_rounds = 5,
            print_every_n = 500, nfold = 5, eval_metric = "auc")

        nrounds <- cv_res$best_iteration
    }

    bstSparse <- xgb.train(params = param, data = train, nrounds = nrounds,
        print_every_n = 100, eval_metric = "auc", watchlist = watchlist,
        early_stopping_rounds = 100)

    # Recreate the original train data to avoid confusion
    # later: Never want to accidental mess with the
    # augmented data again.
    train <- as.data.frame(training(XGd))
    trainX <- train[, 1:(ncol(train) - 1)]
    trainY <- train[, ncol(train)]
    #########

    # Facile test on train.... to delete
    pred <- predict(bstSparse, as.matrix(trainX), reshape = TRUE)
    prediction <- as.numeric(pred[, 2] > 0.3)
    rocker <- roc(trainY, pred[, 2])
    confusionMatrix(as.factor(prediction), as.factor(trainY))
    ######
}
```

#ROC FULL

```
if (!SKIPFULL) {
    fprint(paste("\nFULL MODEL ROC WITH:", health, "_", version))

    pred <- predict(bstSparse, as.matrix(testX), reshape = TRUE)
    pred_obj <- prediction(pred[, 2], testY)

    xgb.perf <- performance(pred_obj, "tpr", "fpr")

    # Remember this is the Full model
    if (tolower(health) == "any") {
        pts <- seq(0.1, 0.9, by = 0.1)
    } else if (tolower(health) == "severe") {
        pts <- c(0.14, 0.18, 0.22)
    }

    text = 1.5  # Text size
    par(bg = NA, cex = 1.5/2, cex.axis = 1.5/2, cex.lab = 1.5/2)
    ROCR::plot(xgb.perf, avg = "threshold", colorize = TRUE,
        lwd = 1, main = "XGBoost: Mild, significant or serious illness",
        print.cutoffs.at = pts, cutoff.label.function = function(x) {
            round(x, 2)
        }, text.adj = c(-1, 1), colorkey.relwidth = 1)

    grid(col = "lightgray")
    axis(1, at = seq(0, 1, by = 0.1))
    axis(2, at = seq(0, 1, by = 0.1))
    abline(v = c(0.1, 0.3, 0.5, 0.7, 0.9), col = "lightgray",
        lty = "dotted")
    abline(h = c(0.1, 0.3, 0.5, 0.7, 0.9), col = "lightgray",
        lty = "dotted")
    lines(x = c(0, 1), y = c(0, 1), col = "black", lty = "dotted")

    tiff(paste0("figures/xgROCsimpleFull_", version, health,
        ".tif"), width = 3.25, height = 3.25, units = "in", res = 300,
        pointsize = 4)
    par(bg = NA, cex = 1.5, cex.axis = 1.5, cex.lab = 1.5)
    ROCR::plot(xgb.perf, avg = "threshold", colorize = TRUE,
        lwd = 3, main = "", print.cutoffs.at = pts, cutoff.label.function = function(x) {
            round(x, 2)
        }, text.adj = c(-1, 1), colorkey.relwidth = 1)
    grid(col = "lightgray")
    axis(1, at = seq(0, 1, by = 0.1))
    axis(2, at = seq(0, 1, by = 0.1))
    abline(v = c(0.1, 0.3, 0.5, 0.7, 0.9), col = "lightgray",
        lty = "dotted")
    abline(h = c(0.1, 0.3, 0.5, 0.7, 0.9), col = "lightgray",
        lty = "dotted")
    lines(x = c(0, 1), y = c(0, 1), col = "black", lty = "dotted")
    dev.off()

    res <- pROC::roc(testY, pred[, 2], ci = TRUE, conf.level = 0.99)
    res$auc
    res$ci
    fprint(paste("\nFull model ROC, AUC:", res$auc))
    fprint(paste("99% confidence:", res$ci))
    cis <- paste(round(res$ci, 3), collapse = ":")
    cis <- paste("Full ROC:", health, version, cis)
    con <- file("status.csv", "a")
    writeLines(cis, con)
    close(con)

}
```

```
## [1] "\nFULL MODEL ROC WITH: Severe _ 101"
```

```
## Setting levels: control = 0, case = 1
```

```
## Setting direction: controls < cases
```

```
## [1] "\nFull model ROC, AUC: 0.887042544937282"
## [1] "99% confidence: 0.815523707264776" "99% confidence: 0.887042544937282"
## [3] "99% confidence: 0.958561382609788"
```

# FULL XGboost Prediction

Just an example threshold

```
if (!SKIPFULL) {
    # CHOOSE A THRESHOLD FROM THE ROC
    thresh = 0.3
    pred <- predict(bstSparse, as.matrix(testX), reshape = TRUE)
    prediction <- as.numeric(pred[, 2] > thresh)
    confusionMatrix(as.factor(prediction), as.factor(testY))
}
```

```
## Confusion Matrix and Statistics
## 
##           Reference
## Prediction   0   1
##          0 612  27
##          1  15  10
##                                          
##                Accuracy : 0.9367         
##                  95% CI : (0.9155, 0.954)
##     No Information Rate : 0.9443         
##     P-Value [Acc > NIR] : 0.82498        
##                                          
##                   Kappa : 0.2907         
##                                          
##  Mcnemar's Test P-Value : 0.08963        
##                                          
##             Sensitivity : 0.9761         
##             Specificity : 0.2703         
##          Pos Pred Value : 0.9577         
##          Neg Pred Value : 0.4000         
##              Prevalence : 0.9443         
##          Detection Rate : 0.9217         
##    Detection Prevalence : 0.9623         
##       Balanced Accuracy : 0.6232         
##                                          
##        'Positive' Class : 0              
##
```

# Calculate importance FULL

```
if (!SKIPFULL) {
    fprint(paste("\nIMPORTANCE WITH FULL MODEL:", health, "_",
        version))

    do_importance(bstSparse, "Full")
}
```

```
## [1] "\nIMPORTANCE WITH FULL MODEL: Severe _ 101"
##              Feature         Gain        Cover    Frequency
##  1:           Visits 5.303995e-01 0.4734936032 0.2850734095
##  2:          Dog Age 1.819081e-01 0.2191939137 0.3022022838
##  3:             Meds 1.751356e-01 0.1904154752 0.1427406199
##  4:             Size 5.007180e-02 0.0342057141 0.0791190865
##  5:        Education 1.617003e-02 0.0195406322 0.0501631321
##  6:        Owner Age 1.171807e-02 0.0144728252 0.0362969005
##  7:               UK 1.104383e-02 0.0123635509 0.0297716150
##  8:            Urban 1.028643e-02 0.0105731408 0.0269168026
##  9:          Dog Sex 9.247078e-03 0.0102498819 0.0342577488
## 10:    Animal Career 1.091473e-03 0.0020314823 0.0028548124
## 11:    Neuter status 7.600563e-04 0.0004303305 0.0008156607
## 12:         European 7.314753e-04 0.0031119194 0.0028548124
## 13: Owner Vegan Diet 4.843322e-04 0.0008820155 0.0012234910
## 14:           Income 4.320734e-04 0.0008429190 0.0012234910
## 15:     Owner Gender 3.107740e-04 0.0056107986 0.0032626427
## 16: Therapeutic Food 1.478658e-04 0.0024268211 0.0008156607
## 17:     Dog Diet Raw 6.148987e-05 0.0001549765 0.0004078303
```

```
## Warning in write.table(data, "status.txt", append = TRUE): appending column
## names to file
```

```
## Using Feature as id variables
```

```
## Warning in write.table(output, "status.csv", sep = ",", row.names = FALSE, :
## appending column names to file
```

#Reduced XGBoost Now XGBoost on the data without meds and visits or
therapeutic foods ##Simple XGBoost on reduced variable set, still Any
Health Issue binary # Preprocess reduced XGBoost

```
suppressWarnings(rm(cv_res))
suppressWarnings(rm(bstSparse))
fprint(paste("\n\n\nBEGINNING A REDUCED MODEL WITH:", health,
    "_", version))
locations <- dplyr::select(ml, Location)
locations <- one_hot(as.data.table(locations), dropUnusedLevels = TRUE)

factors <- cbind(locations)

fnames <- names(factors)
fnames <- nicenames(fnames, "European", "European")
fnames <- nicenames(fnames, "UK", "UK")
fnames <- nicenames(fnames, "Oceania", "Oceania")
fnames <- nicenames(fnames, "America", "America")
fnames <- nicenames(fnames, "Other", "Other")

names(factors) <- fnames

if (tolower(DAge == "quant")) {
    ordered <- as.data.frame(cbind(ml$Income, ml$Education, ordered(ml$D_Age_quant),
        ml$C_Age, ml$Size))

} else {
    ordered <- as.data.frame(cbind(ml$Income, ml$Education, ml$D_Age,
        ml$C_Age, ml$Size))
}

names(ordered) <- c("Income", "Education", "Dog Age", "Owner Age",
    "Size")

numeric <- dplyr::select(ml, C_Gender)
numeric$C_Gender <- as.numeric(numeric$C_Gender) - 1
numeric$Urban <- as.numeric(factor(ml$Urban, levels = c("No",
    "Yes"), order = TRUE)) - 1
numeric$D_Sex <- as.numeric(ml$D_Sex) - 1
numeric$Neuter <- as.numeric(ml$D_Neuter) - 1
numeric$D_Diet_Vegan <- as.numeric(as.factor(ml$D_Diet_Vegan)) -
    1

numeric$D_Diet_Raw <- as.numeric(as.factor(ml$D_Diet_Raw)) -
    1
numeric$C_Diet_Vegan <- as.numeric(as.factor(ml$C_Diet_Vegan)) -
    1

numeric$BIN_Animal_Career <- as.numeric(as.factor(ml$Animal_Career_BINARY)) -
    1
if (tolower(health) == "any") {
    numeric$Health_Binary <- ml$Any_Health_Problem
} else if (tolower(health) == "severe") {
    numeric$Health_Binary <- as.numeric(ml$Health_Binary)
} else {
    stop(paste("OOPS ERROR, YOU'RE HEALTH VARIABLE WAS", health))
}

names(numeric) <- c("Owner Gender", "Urban", "Dog Sex", "Neuter status",
    "Dog Vegan Diet", "Dog Diet Raw", "Owner Vegan Diet", "Animal Career",
    "Health_Binary")

if (version == "102") {
    Decision <- as.numeric(as.factor(ml$Primary_Decision_Maker))
    Decision <- data.frame(Decision = Decision)
    numeric <- cbind(Decision, numeric)
}

XGdata <- cbind(factors, ordered, numeric)
# XGdata$Health_Binary <- XGdata$Health_Binary-1
```

#Train Reduced XG

```
set.seed(2020)

update <- paste("Reduced Model (Training):", health, "_", version)
con <- file("status.csv", "a")
writeLines(update, con)
close(con)

XGd <- initial_split(XGdata, prop = 0.7, strata = Health_Binary)

XGd_valid <- initial_split(training(XGd), prop = 0.7, strata = Health_Binary)

train <- training(XGd_valid)
train <- as.data.frame(training(XGd))

validXG <- testing(XGd_valid)
train <- as.data.frame(train)
trainX <- train[, 1:(ncol(train) - 1)]
trainY <- train[, ncol(train)]
augments <- augment(trainX, trainY, method = Meth_augment)
trainX <- augments[["trainX"]]
trainY <- augments[["trainY"]]

train <- xgb.DMatrix(as.matrix(trainX), label = trainY)

validXG <- as.data.frame(validXG)
validX <- validXG[, 1:(ncol(validXG) - 1)]

validY <- validXG[, ncol(validXG)]
valid <- xgb.DMatrix(as.matrix(validX), label = validY)

# Models work best if training data augmented

watchlist = list(eval = valid, train = train)

# Two alternative methods. Bruteforce seems OK.
length(trainY[trainY == 0])
```

```
## [1] 3497
```

```
length(trainY[trainY > 0])
```

```
## [1] 144
```

```
# new method
test <- as.data.frame(testing(XGd))
testX <- test[, 1:ncol(test) - 1]
testY <- test[, ncol(test)]
# weight<-nrow(ml)/(1-sum(ml$Bhealth)) wont work now
# changed to factor wat above
weight = 1
# weight <- weight^0.5 #some say otherwise skews
param <- list(max.depth = depth, eta = 0.01, nthread = 10, objective = "multi:softprob",
    num_class = 2, min_child_weight = min_child_weight, subsample = 0.5,
    gamma = 0.1, booster = "gbtree")
#'binary:logistic' or 'multi:softprob' 
# If using binary:logistic then delete the class number
# parameter!

if (nrounds == 0) {
    # This is one quick way to tune, remembering that
    # iterations is kind of the same as number of trees.
    cv_res <- xgb.cv(data = as.matrix(trainX), label = trainY,
        params = param, nrounds = 2000, early_stopping_rounds = 5,
        print_every_n = 500, nfold = 5, eval_metric = "auc")

    nrounds <- cv_res$best_iteration
}

bstSparse <- xgb.train(params = param, data = train, nrounds = nrounds,
    print_every_n = 100, eval_metric = "auc", watchlist = watchlist,
    early_stopping_rounds = 100)
```

```
## [1]  eval-auc:0.630805   train-auc:0.676853 
## Multiple eval metrics are present. Will use train_auc for early stopping.
## Will train until train_auc hasn't improved in 100 rounds.
## 
## [101]    eval-auc:0.677548   train-auc:0.756655 
## [201]    eval-auc:0.698391   train-auc:0.770445 
## [301]    eval-auc:0.695479   train-auc:0.771537 
## [401]    eval-auc:0.693180   train-auc:0.771422 
## Stopping. Best iteration:
## [336]    eval-auc:0.696092   train-auc:0.772503
```

```
# Recreate the original train data to avoid confusion
# later: Never want to accidental mess with the augmented
# data again.
train <- as.data.frame(training(XGd))
trainX <- train[, 1:(ncol(train) - 1)]
trainY <- train[, ncol(train)]
#########
```

#ROC reduced

```
fprint(paste("\n\n\nREDUCED MODEL ROC WITH:", health, "_", version))
```

```
## [1] "\n\n\nREDUCED MODEL ROC WITH: Severe _ 101"
```

```
pred <- predict(bstSparse, as.matrix(testX), reshape = TRUE)
pred_obj <- prediction(pred[, 2], testY)

xgb.perf <- performance(pred_obj, "tpr", "fpr")
# Remember this is the reduced model
if (tolower(health) == "any") {
    pts <- seq(0.2, 0.6, by = 0.1)
} else if (tolower(health) == "severe") {
    pts <- c(0.1, 0.06, 0.05)  # seq(0.10, 0.18, by=0.02)
} else pts <- c()  # empty vector safest
text = 1.5  # Text size
par(bg = NA, cex = 1.5/2, cex.axis = 1.5/2, cex.lab = 1.5/2)
ROCR::plot(xgb.perf, avg = "threshold", colorize = TRUE, lwd = 1,
    main = paste("XGBoost:Reduced:", health), print.cutoffs.at = pts,
    cutoff.label.function = function(x) {
        round(x, 3)
    }, text.adj = c(-0.5, 0.9), text.cex = 0.5)
grid(col = "lightgray")
axis(1, at = seq(0, 1, by = 0.1))
axis(2, at = seq(0, 1, by = 0.1))
abline(v = c(0.1, 0.3, 0.5, 0.7, 0.9), col = "lightgray", lty = "dotted")
abline(h = c(0.1, 0.3, 0.5, 0.7, 0.9), col = "lightgray", lty = "dotted")
lines(x = c(0, 1), y = c(0, 1), col = "black", lty = "dotted")
```

```
tiff(paste0("figures/XGROCsimpleReduced_", version, health, ".tif"),
    width = 3.25, height = 3.25, units = "in", res = 300, pointsize = 4)
par(bg = NA, cex = 1.5, cex.axis = 1.5, cex.lab = 1.5)
ROCR::plot(xgb.perf, avg = "threshold", colorize = TRUE, lwd = 3,
    main = "", print.cutoffs.at = pts, cutoff.label.function = function(x) {
        round(x, 3)
    }, text.adj = c(-0.3, 1.3), colorkey.relwidth = 1)
grid(col = "lightgray")
axis(1, at = seq(0, 1, by = 0.1))
axis(2, at = seq(0, 1, by = 0.1))
abline(v = c(0.1, 0.3, 0.5, 0.7, 0.9), col = "lightgray", lty = "dotted")
abline(h = c(0.1, 0.3, 0.5, 0.7, 0.9), col = "lightgray", lty = "dotted")
lines(x = c(0, 1), y = c(0, 1), col = "black", lty = "dotted")
dev.off()
```

```
## quartz_off_screen 
##                 2
```

```
res <- pROC::roc(testY, pred[, 2], ci = TRUE, conf.level = 0.99)
```

```
## Setting levels: control = 0, case = 1
```

```
## Setting direction: controls < cases
```

```
res$auc
```

```
## Area under the curve: 0.6886
```

```
res$ci
```

```
## 99% CI: 0.5722-0.805 (DeLong)
```

```
fprint(paste("Reduced model ROC, AUC:", res$auc))
```

```
## [1] "Reduced model ROC, AUC: 0.688607267554636"
```

```
fprint(paste("99% confidence:", res$ci))
```

```
## [1] "99% confidence: 0.572171208148505" "99% confidence: 0.688607267554636"
## [3] "99% confidence: 0.805043326960767"
```

```
cis <- paste(round(res$ci, 3), collapse = ":")
cis <- paste("Reduced ROC:", health, version, cis)
con <- file("status.csv", "a")
writeLines(cis, con)
close(con)
```

# XGboostPrediction REDUCED

```
# CHOOSE A THRESHOLD FROM THE ROC
thresh = 0.775
pred <- predict(bstSparse, as.matrix(testX), reshape = TRUE)
prediction <- as.numeric(pred[, 2] > thresh)
confusionMatrix(factor(prediction, levels = c(0, 1)), as.factor(testY))
```

```
## Confusion Matrix and Statistics
## 
##           Reference
## Prediction   0   1
##          0 627  37
##          1   0   0
##                                          
##                Accuracy : 0.9443         
##                  95% CI : (0.924, 0.9605)
##     No Information Rate : 0.9443         
##     P-Value [Acc > NIR] : 0.5436         
##                                          
##                   Kappa : 0              
##                                          
##  Mcnemar's Test P-Value : 3.252e-09      
##                                          
##             Sensitivity : 1.0000         
##             Specificity : 0.0000         
##          Pos Pred Value : 0.9443         
##          Neg Pred Value :    NaN         
##              Prevalence : 0.9443         
##          Detection Rate : 0.9443         
##    Detection Prevalence : 1.0000         
##       Balanced Accuracy : 0.5000         
##                                          
##        'Positive' Class : 0              
##
```

# Calculate importance REDUCED

```
do_importance(bstSparse, "Reduced")
```

```
##              Feature        Gain       Cover   Frequency
##  1:          Dog Age 0.304107474 0.361710921 0.223867403
##  2:             Size 0.195092956 0.225696979 0.186298343
##  3:        Owner Age 0.091391553 0.081600702 0.124640884
##  4:            Urban 0.077140721 0.066229642 0.085745856
##  5:        Education 0.072165861 0.060261510 0.094143646
##  6:     Dog Diet Raw 0.058843647 0.042311685 0.047071823
##  7:          Dog Sex 0.050254195 0.030389563 0.068950276
##  8:               UK 0.039205248 0.025109484 0.056132597
##  9:    Animal Career 0.027833745 0.025039299 0.024088398
## 10:    Neuter status 0.019628164 0.015284711 0.018784530
## 11:   Dog Vegan Diet 0.017960920 0.014215017 0.016132597
## 12:         European 0.016909382 0.021623858 0.017237569
## 13: Owner Vegan Diet 0.015331922 0.010457556 0.017900552
## 14:           Income 0.012911079 0.013434905 0.016132597
## 15:     Owner Gender 0.001223134 0.006634167 0.002872928
```

```
## Warning in write.table(data, "status.txt", append = TRUE): appending column
## names to file
```

```
## Using Feature as id variables
```

```
## Warning in write.table(output, "status.csv", sep = ",", row.names = FALSE, :
## appending column names to file
```
