# Supplementary material for: Variables associated with owner perceptions of the health of their dog: Further analysis of data from a large international survey
Source: PLoS One. 2024 May 15;19(5):e0280173. doi: 10.1371/journal.pone.0280173 (PMC11095744; doi:10.1371/journal.pone.0280173)
Supplement: S7 File — (HTML) [file pone.0280173.s021.html]

 

 

 

 
 
 


 

 

 R stats for binary logistic regression on the ANY HEALTH PROBLEM outcome variable 

 
 
 
 
 
 
 
 
 
 
 
 
 

 

 
 


 


 

 

 


 

 


 


 


 R stats for binary logistic regression on
the ANY HEALTH PROBLEM outcome variable 
 Alex German 
 7 December 2023 

 


 
 Create data frame for analysis 
 
 NB need to run “Read_data_101.Rtm” first to create dataset 
 
 
 
 LET’S DO SOME INTIAL TABULATION AND CHI SQUARE TESTS 
 
 Tabulate D_Sex + D_Neuter + D_Diet 
  ## three way cross tabs (xtabs) and flatten the table
ftable(xtabs(~ D_Sex + D_Neuter + D_Diet, data = ml))  
  ##                 D_Diet Meat-based – conventional Meat-based – raw Vegan (consuming no animal products) Vegetarian (including eggs or milk, but not meat)
## D_Sex  D_Neuter                                                                                                                                         
## Female Intact                                 79               92                                   20                                                 1
##        Neutered                              465              239                                  133                                                13
## Male   Intact                                139              131                                   21                                                 3
##        Neutered                              469              270                                  121                                                15  
 
 
 Tabulate D_Neuter + D_Diet 
  ## two way cross tabs (xtabs) and flatten the table
ftable(xtabs(~ D_Neuter + D_Diet, data = ml))  
  ##          D_Diet Meat-based – conventional Meat-based – raw Vegan (consuming no animal products) Vegetarian (including eggs or milk, but not meat)
## D_Neuter                                                                                                                                         
## Intact                                218              223                                   41                                                 4
## Neutered                              934              509                                  254                                                28  
 
 
 Tabulate C_Diet_Vegan + D_Diet_Vegan 
  ## two way cross tabs (xtabs) and flatten the table
ftable(xtabs(~ C_Diet_Vegan + D_Diet_Vegan, data = ml))  
  ##              D_Diet_Vegan   No  Yes
## C_Diet_Vegan                       
## No                        1696   22
## Yes                        220  273  
 
 
 Tabulate C_Diet_Vegan_Veggie + D_Diet_Vegan_Veggie 
  ## two way cross tabs (xtabs) and flatten the table
ftable(xtabs(~ C_Diet_Vegan_Veggie + D_Diet_Vegan_Veggie, data = ml))  
  ##                     D_Diet_Vegan_Veggie   No  Yes
## C_Diet_Vegan_Veggie                              
## No                                      1473   24
## Yes                                      411  303  
 
 
 Tabulate D_Sex, D_Neuter and D_Raw 
  ## three way cross tabs (xtabs) and flatten the table
ftable(xtabs(~ D_Sex + D_Neuter + D_Diet_Raw, data = ml))  
  ##                 D_Diet_Raw  No Yes
## D_Sex  D_Neuter                   
## Female Intact              100  92
##        Neutered            611 239
## Male   Intact              163 131
##        Neutered            605 270  
 
 
 
 Tabulate D_Neuter and D_Raw 
  ## two way cross tabs (xtabs) and flatten the table
ftable(xtabs(~ D_Neuter + D_Diet_Raw, data = ml))  
  ##          D_Diet_Raw   No  Yes
## D_Neuter                     
## Intact               263  223
## Neutered            1216  509  
 
 Chi squared test of C_VEGAN vs D-VEGAN 
  table(ml$C_Diet_Vegan, ml$D_Diet_Vegan)  
  ##      
##         No  Yes
##   No  1696   22
##   Yes  220  273  
  chisq.test(ml$C_Diet_Vegan, ml$D_Diet_Vegan, correct=FALSE)  
  ## 
##  Pearson's Chi-squared test
## 
## data:  ml$C_Diet_Vegan and ml$D_Diet_Vegan
## X-squared = 969.51, df = 1, p-value < 2.2e-16  
 
 
 Chi squared test of NEUTERED vs D-RAW 
  table(ml$D_Neuter, ml$D_Diet_Raw)  
  ##           
##              No  Yes
##   Intact    263  223
##   Neutered 1216  509  
  chisq.test(ml$D_Neuter, ml$D_Diet_Raw, correct=FALSE)  
  ## 
##  Pearson's Chi-squared test
## 
## data:  ml$D_Neuter and ml$D_Diet_Raw
## X-squared = 45.923, df = 1, p-value = 1.23e-11  
 
 
 Tabulate Health_Binary and Income 
  ## three way cross tabs (xtabs) and flatten the table
ftable(xtabs(~ Health_Binary + Income, data = ml))  
  ##               Income  Low Medium High
## Health_Binary                        
## 0                     330   1445  314
## 1                      22     85   15  
 
 
 Chi squared test of Health_Binary and Income 
  table(ml$Health_Binary, ml$Income)  
  ##    
##      Low Medium High
##   0  330   1445  314
##   1   22     85   15  
  chisq.test(ml$Health_Binary, ml$Income, correct=FALSE)  
  ## 
##  Pearson's Chi-squared test
## 
## data:  ml$Health_Binary and ml$Income
## X-squared = 0.94597, df = 2, p-value = 0.6231  
 
 
 
 BINARY LOGISTIC REGRESSION ON HEALTH 
 
 
 CHECK EFFECT OF OWNER CHARACTERISTICS - simple binary logistic
regression 
 
 CLIENT DIET Binary regression for Any_Health_Problem 
  # fit binary logit model and store results 'm'
m <- glm(Any_Health_Problem ~ C_Diet, data = ml_train,family = binomial)
# view a summary of the model
summary(m)  
  ## 
## Call:
## glm(formula = Any_Health_Problem ~ C_Diet, family = binomial, 
##     data = ml_train)
## 
## Coefficients:
##                                                       Estimate Std. Error
## (Intercept)                                           -0.60158    0.08031
## C_DietOmnivore reducing animal product consumption     0.20752    0.13357
## C_DietPescatarian (including fish but no other meats)  0.66612    0.22250
## C_DietVegan (consuming no animal products)             0.01291    0.13708
## C_DietVegetarian (consuming plants, eggs and milk)     0.18130    0.17658
##                                                       z value Pr(>|z|)    
## (Intercept)                                            -7.491 6.85e-14 ***
## C_DietOmnivore reducing animal product consumption      1.554  0.12026    
## C_DietPescatarian (including fish but no other meats)   2.994  0.00276 ** 
## C_DietVegan (consuming no animal products)              0.094  0.92496    
## C_DietVegetarian (consuming plants, eggs and milk)      1.027  0.30454    
## ---
## Signif. codes:  0 '***' 0.001 '**' 0.01 '*' 0.05 '.' 0.1 ' ' 1
## 
## (Dispersion parameter for binomial family taken to be 1)
## 
##     Null deviance: 2200.0  on 1657  degrees of freedom
## Residual deviance: 2189.2  on 1653  degrees of freedom
## AIC: 2199.2
## 
## Number of Fisher Scoring iterations: 4  
  # test model fit
with(m, null.deviance - deviance)  
  ## [1] 10.83013  
  with(m, df.null - df.residual)  
  ## [1] 4  
  with(m, pchisq(null.deviance - deviance, df.null - df.residual, lower.tail = FALSE))  
  ## [1] 0.02854094  
  BIC(m)  
  ## [1] 2226.265  
  ## CIs using profiled log-likelihood
confint(m, level=0.99)  
  ## Waiting for profiling to be done...  
  ##                                                             0.5 %     99.5 %
## (Intercept)                                           -0.81072414 -0.3966114
## C_DietOmnivore reducing animal product consumption    -0.13763543  0.5510344
## C_DietPescatarian (including fish but no other meats)  0.09200566  1.2434624
## C_DietVegan (consuming no animal products)            -0.34240322  0.3645036
## C_DietVegetarian (consuming plants, eggs and milk)    -0.27868901  0.6330898  
  ## CIs using standard errors
confint.default(m, level=0.99)  
  ##                                                             0.5 %     99.5 %
## (Intercept)                                           -0.80844603 -0.3947139
## C_DietOmnivore reducing animal product consumption    -0.13652350  0.5515585
## C_DietPescatarian (including fish but no other meats)  0.09300184  1.2392352
## C_DietVegan (consuming no animal products)            -0.34018879  0.3660125
## C_DietVegetarian (consuming plants, eggs and milk)    -0.27352971  0.6361293  
  # Wald test
wald.test(b = coef(m), Sigma = vcov(m), Terms = 2)  
  ## Wald test:
## ----------
## 
## Chi-squared test:
## X2 = 2.4, df = 1, P(> X2) = 0.12  
  ## odds ratios and 95% CI
exp(cbind(OR = coef(m), confint(m, level=0.99)))  
  ## Waiting for profiling to be done...  
  ##                                                              OR     0.5 %
## (Intercept)                                           0.5479452 0.4445360
## C_DietOmnivore reducing animal product consumption    1.2306193 0.8714163
## C_DietPescatarian (including fish but no other meats) 1.9466667 1.0963710
## C_DietVegan (consuming no animal products)            1.0129956 0.7100618
## C_DietVegetarian (consuming plants, eggs and milk)    1.1987745 0.7567752
##                                                          99.5 %
## (Intercept)                                           0.6725953
## C_DietOmnivore reducing animal product consumption    1.7350468
## C_DietPescatarian (including fish but no other meats) 3.4675989
## C_DietVegan (consuming no animal products)            1.4397991
## C_DietVegetarian (consuming plants, eggs and milk)    1.8834210  
 
 Calculate Nagelkerke R^2 
  NagelkerkeR2(m)  
  ## $N
## [1] 1658
## 
## $R2
## [1] 0.008861716  
 
 
 check assumptions of model 
 
 Cook’s distance 
  plot(m, which = 4, id.n = 3)  
   
 
 
 Extract model results and display data for top 3 values using Cook’s
distance 
  model.data <- augment(m) %>% 
  mutate(index = 1:n()) 
model.data %>% top_n(3, .cooksd)  
 
 
 
 
 
 plot standardised residuals 
  ggplot(model.data, aes(index, .std.resid)) + 
  geom_point(aes(color = Any_Health_Problem), alpha = .5) +
  theme_bw()  
   
 
 
 Filter potential influential data points with abs(.std.res) >
3: 
  model.data %>% 
  filter(abs(.std.resid) > 3)  
 
 
 
 
 
 
 Create ROCR for training and test data 
  ## training data
pred.mtt = predict(m, type = "response") #repeat risk predictions from model m
rocr.pred.mtt = ROCR::prediction(pred.mtt, labels = ml_train$Any_Health_Problem) #ROCR prediction object
roc.perf.mtt = ROCR::performance(rocr.pred.mtt, measure = "tpr", x.measure = "fpr") # #ROCR performance object
plot(roc.perf.mtt, col = "blue")


pred.te.1 = predict(m, newdata = ml_test, type = "response") #.te = "test"
rocr.pred.te.1 = ROCR::prediction(pred.te.1, labels = ml_test$Any_Health_Problem)
roc.perf.te.1 = ROCR::performance(rocr.pred.te.1, measure = "tpr", x.measure = "fpr")
plot(roc.perf.te.1, col = "red", add = T)

abline(a = 0, b = 1, lty = 2) #diagonal for random assignment
legend("bottomright", legend = c("train","test"),
col = c("blue","red"), lty = c(2,1), lwd =1.5)  
   
 
 
 Report AUC from ROC for training and test data 
    # Train AUC
aucTr <- ROCR::performance(rocr.pred.mtt, measure = "auc")
  aucTr <- aucTr@y.values[[1]]
  print(aucTr)  
  ## [1] 0.5366907  
     # Test AUC
  aucTe <- ROCR::performance(rocr.pred.te.1, measure = "auc")
  aucTe <- aucTe@y.values[[1]]
  print(aucTe)  
  ## [1] 0.525216  
 
 
 
 CLIENT DIET VEGAN Binary regression for Any_Health_Problem 
  # fit binary logit model and store results 'm'
m <- glm(Any_Health_Problem ~ C_Diet_Vegan, data = ml_train,family = binomial)
# view a summary of the model
summary(m)  
  ## 
## Call:
## glm(formula = Any_Health_Problem ~ C_Diet_Vegan, family = binomial, 
##     data = ml_train)
## 
## Coefficients:
##                 Estimate Std. Error z value Pr(>|z|)    
## (Intercept)      -0.4698     0.0569  -8.256   <2e-16 ***
## C_Diet_VeganYes  -0.1189     0.1248  -0.953    0.341    
## ---
## Signif. codes:  0 '***' 0.001 '**' 0.01 '*' 0.05 '.' 0.1 ' ' 1
## 
## (Dispersion parameter for binomial family taken to be 1)
## 
##     Null deviance: 2200.0  on 1657  degrees of freedom
## Residual deviance: 2199.1  on 1656  degrees of freedom
## AIC: 2203.1
## 
## Number of Fisher Scoring iterations: 4  
  # test model fit
with(m, null.deviance - deviance)  
  ## [1] 0.9135053  
  with(m, df.null - df.residual)  
  ## [1] 1  
  with(m, pchisq(null.deviance - deviance, df.null - df.residual, lower.tail = FALSE))  
  ## [1] 0.3391861  
  BIC(m)  
  ## [1] 2213.942  
  ## CIs using profiled log-likelihood
confint(m, level=0.99)  
  ## Waiting for profiling to be done...  
  ##                      0.5 %     99.5 %
## (Intercept)     -0.6172079 -0.3239558
## C_Diet_VeganYes -0.4437405  0.2000408  
  ## CIs using standard errors
confint.default(m, level=0.99)  
  ##                      0.5 %     99.5 %
## (Intercept)     -0.6163137 -0.3231955
## C_Diet_VeganYes -0.4404195  0.2025924  
  # Wald test
wald.test(b = coef(m), Sigma = vcov(m), Terms = 2)  
  ## Wald test:
## ----------
## 
## Chi-squared test:
## X2 = 0.91, df = 1, P(> X2) = 0.34  
  ## odds ratios and 95% CI
exp(cbind(OR = coef(m), confint(m, level=0.99)))  
  ## Waiting for profiling to be done...  
  ##                        OR     0.5 %    99.5 %
## (Intercept)     0.6251557 0.5394485 0.7232822
## C_Diet_VeganYes 0.8878846 0.6416319 1.2214526  
 
 Calculate Nagelkerke R^2 
  NagelkerkeR2(m)  
  ## $N
## [1] 1658
## 
## $R2
## [1] 0.0007497099  
 
 
 check assumptions of model 
 
 Cook’s distance 
  plot(m, which = 4, id.n = 3)  
   
 
 
 Extract model results and display data for top 3 values using Cook’s
distance 
  model.data <- augment(m) %>% 
  mutate(index = 1:n()) 
model.data %>% top_n(3, .cooksd)  
 
 
 
 
 
 plot standardised residuals 
  ggplot(model.data, aes(index, .std.resid)) + 
  geom_point(aes(color = Any_Health_Problem), alpha = .5) +
  theme_bw()  
   
 
 
 
 
 
 Filter potential influential data points with abs(.std.res) >
3: 
  model.data %>% 
  filter(abs(.std.resid) > 3)  
 
 
 
 
 Create ROCR for training and test data 
  ## training data
pred.mtt = predict(m, type = "response") #repeat risk predictions from model m
rocr.pred.mtt = ROCR::prediction(pred.mtt, labels = ml_train$Any_Health_Problem) #ROCR prediction object
roc.perf.mtt = ROCR::performance(rocr.pred.mtt, measure = "tpr", x.measure = "fpr") # #ROCR performance object
plot(roc.perf.mtt, col = "blue")


pred.te.1 = predict(m, newdata = ml_test, type = "response") #.te = "test"
rocr.pred.te.1 = ROCR::prediction(pred.te.1, labels = ml_test$Any_Health_Problem)
roc.perf.te.1 = ROCR::performance(rocr.pred.te.1, measure = "tpr", x.measure = "fpr")
plot(roc.perf.te.1, col = "red", add = T)

abline(a = 0, b = 1, lty = 2) #diagonal for random assignment
legend("bottomright", legend = c("train","test"),
col = c("blue","red"), lty = c(2,1), lwd =1.5)  
   
 
 
 Report AUC from ROC for training and test data 
    # Train AUC
aucTr <- ROCR::performance(rocr.pred.mtt, measure = "auc")
  aucTr <- aucTr@y.values[[1]]
  print(aucTr)  
  ## [1] 0.5098757  
     # Test AUC
  aucTe <- ROCR::performance(rocr.pred.te.1, measure = "auc")
  aucTe <- aucTe@y.values[[1]]
  print(aucTe)  
  ## [1] 0.5025362  
 
 
 C_Diet_Vegan_Veggie Binary logistic regression for HEALTH 
  # fit binary logit model and store results 'm'
m <- glm(Any_Health_Problem ~ C_Diet_Vegan_Veggie, data = ml_train,family = binomial)
# view a summary of the model
summary(m)  
  ## 
## Call:
## glm(formula = Any_Health_Problem ~ C_Diet_Vegan_Veggie, family = binomial, 
##     data = ml_train)
## 
## Coefficients:
##                        Estimate Std. Error z value Pr(>|z|)    
## (Intercept)            -0.47716    0.06104  -7.818 5.38e-15 ***
## C_Diet_Vegan_VeggieYes -0.05621    0.10930  -0.514    0.607    
## ---
## Signif. codes:  0 '***' 0.001 '**' 0.01 '*' 0.05 '.' 0.1 ' ' 1
## 
## (Dispersion parameter for binomial family taken to be 1)
## 
##     Null deviance: 2200.0  on 1657  degrees of freedom
## Residual deviance: 2199.8  on 1656  degrees of freedom
## AIC: 2203.8
## 
## Number of Fisher Scoring iterations: 4  
  # test model fit
with(m, null.deviance - deviance)  
  ## [1] 0.264927  
  with(m, df.null - df.residual)  
  ## [1] 1  
  with(m, pchisq(null.deviance - deviance, df.null - df.residual, lower.tail = FALSE))  
  ## [1] 0.6067552  
  BIC(m)  
  ## [1] 2214.59  
  ## CIs using profiled log-likelihood
confint(m, level=0.99)  
  ## Waiting for profiling to be done...  
  ##                             0.5 %     99.5 %
## (Intercept)            -0.6354292 -0.3208275
## C_Diet_Vegan_VeggieYes -0.3392630  0.2241818  
  ## CIs using standard errors
confint.default(m, level=0.99)  
  ##                            0.5 %     99.5 %
## (Intercept)            -0.634380 -0.3199437
## C_Diet_Vegan_VeggieYes -0.337741  0.2253295  
  # Wald test
wald.test(b = coef(m), Sigma = vcov(m), Terms = 2)  
  ## Wald test:
## ----------
## 
## Chi-squared test:
## X2 = 0.26, df = 1, P(> X2) = 0.61  
  ## odds ratios and 95% CI
exp(cbind(OR = coef(m), confint(m, level=0.99)))  
  ## Waiting for profiling to be done...  
  ##                               OR     0.5 %    99.5 %
## (Intercept)            0.6205421 0.5297081 0.7255484
## C_Diet_Vegan_VeggieYes 0.9453447 0.7122951 1.2512985  
 
 Calculate Nagelkerke R^2 
  NagelkerkeR2(m)  
  ## $N
## [1] 1658
## 
## $R2
## [1] 0.000217467  
 
 
 check assumptions of model 
 
 Cook’s distance 
  plot(m, which = 4, id.n = 3)  
   
 
 
 Extract model results and display data for top 3 values using Cook’s
distance 
  model.data <- augment(m) %>% 
  mutate(index = 1:n()) 
model.data %>% top_n(3, .cooksd)  
 
 
 
 
 
 plot standardised residuals 
  ggplot(model.data, aes(index, .std.resid)) + 
  geom_point(aes(color = Any_Health_Problem), alpha = .5) +
  theme_bw()  
   
 
 
 Filter potential influential data points with abs(.std.res) >
3: 
  model.data %>% 
  filter(abs(.std.resid) > 3)  
 
 
 
 
 
 
 Create ROCR for training and test data 
  ## training data
pred.mtt = predict(m, type = "response") #repeat risk predictions from model m
rocr.pred.mtt = ROCR::prediction(pred.mtt, labels = ml_train$Any_Health_Problem) #ROCR prediction object
roc.perf.mtt = ROCR::performance(rocr.pred.mtt, measure = "tpr", x.measure = "fpr") # #ROCR performance object
plot(roc.perf.mtt, col = "blue")


pred.te.1 = predict(m, newdata = ml_test, type = "response") #.te = "test"
rocr.pred.te.1 = ROCR::prediction(pred.te.1, labels = ml_test$Any_Health_Problem)
roc.perf.te.1 = ROCR::performance(rocr.pred.te.1, measure = "tpr", x.measure = "fpr")
plot(roc.perf.te.1, col = "red", add = T)

abline(a = 0, b = 1, lty = 2) #diagonal for random assignment
legend("bottomright", legend = c("train","test"),
col = c("blue","red"), lty = c(2,1), lwd =1.5)  
   
 
 
 Report AUC from ROC for training and test data 
    # Train AUC
aucTr <- ROCR::performance(rocr.pred.mtt, measure = "auc")
  aucTr <- aucTr@y.values[[1]]
  print(aucTr)  
  ## [1] 0.5060463  
     # Test AUC
  aucTe <- ROCR::performance(rocr.pred.te.1, measure = "auc")
  aucTe <- aucTe@y.values[[1]]
  print(aucTe)  
  ## [1] 0.4931299  
 
 
 
 LOCATION binary logistic regression for HEALTH 
  # fit binary logit model and store results 'm'
m <- glm(Any_Health_Problem ~ Location, data = ml_train,family = binomial)
# view a summary of the model
summary(m)  
  ## 
## Call:
## glm(formula = Any_Health_Problem ~ Location, family = binomial, 
##     data = ml_train)
## 
## Coefficients:
##                                       Estimate Std. Error z value Pr(>|z|)    
## (Intercept)                           -0.56609    0.05969  -9.484   <2e-16 ***
## LocationOther European                 0.20411    0.14700   1.388   0.1650    
## LocationNorth America                  0.30372    0.21862   1.389   0.1647    
## LocationAustralia/New Zealand/Oceania  0.40357    0.24078   1.676   0.0937 .  
## LocationOther                          0.22962    0.29879   0.768   0.4422    
## ---
## Signif. codes:  0 '***' 0.001 '**' 0.01 '*' 0.05 '.' 0.1 ' ' 1
## 
## (Dispersion parameter for binomial family taken to be 1)
## 
##     Null deviance: 2200.0  on 1657  degrees of freedom
## Residual deviance: 2194.1  on 1653  degrees of freedom
## AIC: 2204.1
## 
## Number of Fisher Scoring iterations: 4  
  # test model fit
with(m, null.deviance - deviance)  
  ## [1] 5.902871  
  with(m, df.null - df.residual)  
  ## [1] 4  
  with(m, pchisq(null.deviance - deviance, df.null - df.residual, lower.tail = FALSE))  
  ## [1] 0.2065203  
  BIC(m)  
  ## [1] 2231.193  
  # Hosmer-Lemeshow Goodness-of-Fit Test
glmtoolbox::hltest(m)  
  ## 
##    The Hosmer-Lemeshow goodness-of-fit test
## 
##  Group Size Observed Expected
##      1 1215      440      440
##      2  229       94       94
##      3   48       20       20
##      4   92       40       40
##      5   74       34       34
## 
##          Statistic =  0 
## degrees of freedom =  3 
##            p-value =  1  
  ## CIs using profiled log-likelihood
confint(m, level=0.99)  
  ## Waiting for profiling to be done...  
  ##                                            0.5 %     99.5 %
## (Intercept)                           -0.7210092 -0.4133443
## LocationOther European                -0.1780503  0.5806205
## LocationNorth America                 -0.2682864  0.8639613
## LocationAustralia/New Zealand/Oceania -0.2253643  1.0233643
## LocationOther                         -0.5639731  0.9924539  
  ## CIs using standard errors
confint.default(m, level=0.99)  
  ##                                            0.5 %     99.5 %
## (Intercept)                           -0.7198429 -0.4123337
## LocationOther European                -0.1745365  0.5827531
## LocationNorth America                 -0.2593985  0.8668466
## LocationAustralia/New Zealand/Oceania -0.2166378  1.0237765
## LocationOther                         -0.5400240  0.9992562  
  # Wald test
wald.test(b = coef(m), Sigma = vcov(m), Terms = 2)  
  ## Wald test:
## ----------
## 
## Chi-squared test:
## X2 = 1.9, df = 1, P(> X2) = 0.16  
  ## odds ratios and 95% CI
exp(cbind(OR = coef(m), confint(m, level=0.99)))  
  ## Waiting for profiling to be done...  
  ##                                              OR     0.5 %    99.5 %
## (Intercept)                           0.5677419 0.4862613 0.6614345
## LocationOther European                1.2264310 0.8369004 1.7871471
## LocationNorth America                 1.3548951 0.7646887 2.3725403
## LocationAustralia/New Zealand/Oceania 1.4971591 0.7982254 2.7825403
## LocationOther                         1.2581169 0.5689441 2.6978467  
 
 Calculate Nagelkerke R^2 
  NagelkerkeR2(m)  
  ## $N
## [1] 1658
## 
## $R2
## [1] 0.004837179  
 
 
 check assumptions of model 
 
 Cook’s distance 
  plot(m, which = 4, id.n = 3)  
   
 
 
 
 
 
 Extract model results and display data for top 3 values using Cook’s
distance 
  model.data <- augment(m) %>% 
  mutate(index = 1:n()) 
model.data %>% top_n(3, .cooksd)  
 
 
 
 
 plot standardised residuals 
  ggplot(model.data, aes(index, .std.resid)) + 
  geom_point(aes(color = Any_Health_Problem), alpha = .5) +
  theme_bw()  
   
 
 
 Filter potential influential data points with abs(.std.res) >
3: 
  model.data %>% 
  filter(abs(.std.resid) > 3)  
 
 
 
 
 
 Create ROCR for training and test data 
  ## training data
pred.mtt = predict(m, type = "response") #repeat risk predictions from model m
rocr.pred.mtt = ROCR::prediction(pred.mtt, labels = ml_train$Any_Health_Problem) #ROCR prediction object
roc.perf.mtt = ROCR::performance(rocr.pred.mtt, measure = "tpr", x.measure = "fpr") # #ROCR performance object
plot(roc.perf.mtt, col = "blue")


pred.te.1 = predict(m, newdata = ml_test, type = "response") #.te = "test"
rocr.pred.te.1 = ROCR::prediction(pred.te.1, labels = ml_test$Any_Health_Problem)
roc.perf.te.1 = ROCR::performance(rocr.pred.te.1, measure = "tpr", x.measure = "fpr")
plot(roc.perf.te.1, col = "red", add = T)

abline(a = 0, b = 1, lty = 2) #diagonal for random assignment
legend("bottomright", legend = c("train","test"),
col = c("blue","red"), lty = c(2,1), lwd =1.5)  
   
 
 
 Report AUC from ROC for training and test data 
    # Train AUC
aucTr <- ROCR::performance(rocr.pred.mtt, measure = "auc")
  aucTr <- aucTr@y.values[[1]]
  print(aucTr)  
  ## [1] 0.5272942  
     # Test AUC
  aucTe <- ROCR::performance(rocr.pred.te.1, measure = "auc")
  aucTe <- aucTe@y.values[[1]]
  print(aucTe)  
  ## [1] 0.4914785  
 
 
 SETTING binary logistic regression for HEALTH 
  # fit binary logit model and store results 'm'
m <- glm(Any_Health_Problem ~ setting, data = ml_train,family = binomial)
# view a summary of the model
summary(m)  
  ## 
## Call:
## glm(formula = Any_Health_Problem ~ setting, family = binomial, 
##     data = ml_train)
## 
## Coefficients:
##                                Estimate Std. Error z value Pr(>|z|)    
## (Intercept)                    -0.35499    0.08472  -4.190 2.79e-05 ***
## settingRural                   -0.21383    0.12235  -1.748   0.0805 .  
## settingEqually urban and rural -0.21807    0.12413  -1.757   0.0790 .  
## ---
## Signif. codes:  0 '***' 0.001 '**' 0.01 '*' 0.05 '.' 0.1 ' ' 1
## 
## (Dispersion parameter for binomial family taken to be 1)
## 
##     Null deviance: 2200.0  on 1657  degrees of freedom
## Residual deviance: 2195.9  on 1655  degrees of freedom
## AIC: 2201.9
## 
## Number of Fisher Scoring iterations: 4  
  # test model fit
with(m, null.deviance - deviance)  
  ## [1] 4.156139  
  with(m, df.null - df.residual)  
  ## [1] 2  
  with(m, pchisq(null.deviance - deviance, df.null - df.residual, lower.tail = FALSE))  
  ## [1] 0.1251716  
  BIC(m)  
  ## [1] 2218.113  
  # Hosmer-Lemeshow Goodness-of-Fit Test
glmtoolbox::hltest(m)  
  ## 
##    The Hosmer-Lemeshow goodness-of-fit test
## 
##  Group Size Observed Expected
##      1  527      190      190
##      2  556      201      201
##      3  575      237      237
## 
##          Statistic =  0 
## degrees of freedom =  1 
##            p-value =  1  
  ## CIs using profiled log-likelihood
confint(m, level=0.99)  
  ## Waiting for profiling to be done...  
  ##                                     0.5 %     99.5 %
## (Intercept)                    -0.5748352 -0.1379318
## settingRural                   -0.5297510  0.1009458
## settingEqually urban and rural -0.5387235  0.1011592  
  ## CIs using standard errors
confint.default(m, level=0.99)  
  ##                                     0.5 %     99.5 %
## (Intercept)                    -0.5732178 -0.1367537
## settingRural                   -0.5289850  0.1013308
## settingEqually urban and rural -0.5378134  0.1016672  
  # Wald test
wald.test(b = coef(m), Sigma = vcov(m), Terms = 2)  
  ## Wald test:
## ----------
## 
## Chi-squared test:
## X2 = 3.1, df = 1, P(> X2) = 0.081  
  ## odds ratios and 95% CI
exp(cbind(OR = coef(m), confint(m, level=0.99)))  
  ## Waiting for profiling to be done...  
  ##                                       OR     0.5 %    99.5 %
## (Intercept)                    0.7011834 0.5627976 0.8711581
## settingRural                   0.8074880 0.5887516 1.1062167
## settingEqually urban and rural 0.8040667 0.5834926 1.1064528  
 
 Calculate Nagelkerke R^2 
  NagelkerkeR2(m)  
  ## $N
## [1] 1658
## 
## $R2
## [1] 0.003407592  
 
 
 check assumptions of model 
 
 Cook’s distance 
  plot(m, which = 4, id.n = 3)  
   
 
 
 Extract model results and display data for top 3 values using Cook’s
distance 
  model.data <- augment(m) %>% 
  mutate(index = 1:n()) 
model.data %>% top_n(3, .cooksd)  
 
 
 
 
 
 plot standardised residuals 
  ggplot(model.data, aes(index, .std.resid)) + 
  geom_point(aes(color = Any_Health_Problem), alpha = .5) +
  theme_bw()  
   
 
 
 Filter potential influential data points with abs(.std.res) >
3: 
  model.data %>% 
  filter(abs(.std.resid) > 3)  
 
 
 
 
 
 
 Create ROCR for training and test data 
  ## training data
pred.mtt = predict(m, type = "response") #repeat risk predictions from model m
rocr.pred.mtt = ROCR::prediction(pred.mtt, labels = ml_train$Health_Binary) #ROCR prediction object
roc.perf.mtt = ROCR::performance(rocr.pred.mtt, measure = "tpr", x.measure = "fpr") # #ROCR performance object
plot(roc.perf.mtt, col = "blue")


pred.te.1 = predict(m, newdata = ml_test, type = "response") #.te = "test"
rocr.pred.te.1 = ROCR::prediction(pred.te.1, labels = ml_test$Health_Binary)
roc.perf.te.1 = ROCR::performance(rocr.pred.te.1, measure = "tpr", x.measure = "fpr")
plot(roc.perf.te.1, col = "red", add = T)

abline(a = 0, b = 1, lty = 2) #diagonal for random assignment
legend("bottomright", legend = c("train","test"),
col = c("blue","red"), lty = c(2,1), lwd =1.5)  
   
 
 
 Report AUC from ROC for training and test data 
    # Train AUC
aucTr <- ROCR::performance(rocr.pred.mtt, measure = "auc")
  aucTr <- aucTr@y.values[[1]]
  print(aucTr)  
  ## [1] 0.5198596  
     # Test AUC
  aucTe <- ROCR::performance(rocr.pred.te.1, measure = "auc")
  aucTe <- aucTe@y.values[[1]]
  print(aucTe)  
  ## [1] 0.5423977  
 
 
 
 URBAN binary logistic regression for HEALTH 
  # fit binary logit model and store results 'm'
m <- glm(Any_Health_Problem ~ Urban, data = ml_train,family = binomial)
# view a summary of the model
summary(m)  
  ## 
## Call:
## glm(formula = Any_Health_Problem ~ Urban, family = binomial, 
##     data = ml_train)
## 
## Coefficients:
##             Estimate Std. Error z value Pr(>|z|)    
## (Intercept) -0.57088    0.06327  -9.023   <2e-16 ***
## UrbanYes     0.21589    0.10574   2.042   0.0412 *  
## ---
## Signif. codes:  0 '***' 0.001 '**' 0.01 '*' 0.05 '.' 0.1 ' ' 1
## 
## (Dispersion parameter for binomial family taken to be 1)
## 
##     Null deviance: 2200.0  on 1657  degrees of freedom
## Residual deviance: 2195.9  on 1656  degrees of freedom
## AIC: 2199.9
## 
## Number of Fisher Scoring iterations: 4  
  # test model fit
with(m, null.deviance - deviance)  
  ## [1] 4.155014  
  with(m, df.null - df.residual)  
  ## [1] 1  
  with(m, pchisq(null.deviance - deviance, df.null - df.residual, lower.tail = FALSE))  
  ## [1] 0.04151144  
  BIC(m)  
  ## [1] 2210.7  
  ## CIs using profiled log-likelihood
confint(m, level=0.99)  
  ## Waiting for profiling to be done...  
  ##                   0.5 %     99.5 %
## (Intercept) -0.73516715 -0.4090549
## UrbanYes    -0.05707366  0.4879440  
  ## CIs using standard errors
confint.default(m, level=0.99)  
  ##                   0.5 %     99.5 %
## (Intercept) -0.73384171 -0.4079151
## UrbanYes    -0.05647156  0.4882568  
  # Wald test
wald.test(b = coef(m), Sigma = vcov(m), Terms = 2)  
  ## Wald test:
## ----------
## 
## Chi-squared test:
## X2 = 4.2, df = 1, P(> X2) = 0.041  
  ## odds ratios and 95% CI
exp(cbind(OR = coef(m), confint(m, level=0.99)))  
  ## Waiting for profiling to be done...  
  ##                    OR     0.5 %    99.5 %
## (Intercept) 0.5650289 0.4794253 0.6642778
## UrbanYes    1.2409691 0.9445245 1.6289637  
 
 Calculate Nagelkerke R^2 
  NagelkerkeR2(m)  
  ## $N
## [1] 1658
## 
## $R2
## [1] 0.003406671  
 
 
 check assumptions of model 
 
 Cook’s distance 
  plot(m, which = 4, id.n = 3)  
   
 
 
 Extract model results and display data for top 3 values using Cook’s
distance 
  model.data <- augment(m) %>% 
  mutate(index = 1:n()) 
model.data %>% top_n(3, .cooksd)  
 
 
 
 
 
 plot standardised residuals 
  ggplot(model.data, aes(index, .std.resid)) + 
  geom_point(aes(color = Any_Health_Problem), alpha = .5) +
  theme_bw()  
   
 
 
 Filter potential influential data points with abs(.std.res) >
3: 
  model.data %>% 
  filter(abs(.std.resid) > 3)  
 
 
 
 
 
 
 Create ROCR for training and test data 
  ## training data
pred.mtt = predict(m, type = "response") #repeat risk predictions from model m
rocr.pred.mtt = ROCR::prediction(pred.mtt, labels = ml_train$Health_Binary) #ROCR prediction object
roc.perf.mtt = ROCR::performance(rocr.pred.mtt, measure = "tpr", x.measure = "fpr") # #ROCR performance object
plot(roc.perf.mtt, col = "blue")


pred.te.1 = predict(m, newdata = ml_test, type = "response") #.te = "test"
rocr.pred.te.1 = ROCR::prediction(pred.te.1, labels = ml_test$Health_Binary)
roc.perf.te.1 = ROCR::performance(rocr.pred.te.1, measure = "tpr", x.measure = "fpr")
plot(roc.perf.te.1, col = "red", add = T)

abline(a = 0, b = 1, lty = 2) #diagonal for random assignment
legend("bottomright", legend = c("train","test"),
col = c("blue","red"), lty = c(2,1), lwd =1.5)  
   
 
 
 Report AUC from ROC for training and test data 
    # Train AUC
aucTr <- ROCR::performance(rocr.pred.mtt, measure = "auc")
  aucTr <- aucTr@y.values[[1]]
  print(aucTr)  
  ## [1] 0.5164356  
     # Test AUC
  aucTe <- ROCR::performance(rocr.pred.te.1, measure = "auc")
  aucTe <- aucTe@y.values[[1]]
  print(aucTe)  
  ## [1] 0.5157895  
 
 
 
 Location + URBAN binary logistic regression for HEALTH 
  # fit binary logit model and store results 'm'
m <- glm(Any_Health_Problem ~ Location + Urban, data = ml_train,family = binomial)
# view a summary of the model
summary(m)  
  ## 
## Call:
## glm(formula = Any_Health_Problem ~ Location + Urban, family = binomial, 
##     data = ml_train)
## 
## Coefficients:
##                                       Estimate Std. Error z value Pr(>|z|)    
## (Intercept)                            -0.6162     0.0680  -9.063   <2e-16 ***
## LocationOther European                  0.1796     0.1479   1.214    0.225    
## LocationNorth America                   0.2808     0.2193   1.281    0.200    
## LocationAustralia/New Zealand/Oceania   0.3377     0.2446   1.381    0.167    
## LocationOther                           0.1472     0.3035   0.485    0.628    
## UrbanYes                                0.1713     0.1093   1.568    0.117    
## ---
## Signif. codes:  0 '***' 0.001 '**' 0.01 '*' 0.05 '.' 0.1 ' ' 1
## 
## (Dispersion parameter for binomial family taken to be 1)
## 
##     Null deviance: 2200.0  on 1657  degrees of freedom
## Residual deviance: 2191.7  on 1652  degrees of freedom
## AIC: 2203.7
## 
## Number of Fisher Scoring iterations: 4  
  # test model fit
with(m, null.deviance - deviance)  
  ## [1] 8.35172  
  with(m, df.null - df.residual)  
  ## [1] 5  
  with(m, pchisq(null.deviance - deviance, df.null - df.residual, lower.tail = FALSE))  
  ## [1] 0.1378874  
  BIC(m)  
  ## [1] 2236.157  
  # Hosmer-Lemeshow Goodness-of-Fit Test
hltest(m)  
  ## 
##    The Hosmer-Lemeshow goodness-of-fit test
## 
##  Group Size Observed   Expected
##      1  865      306 303.301225
##      2   11        2   4.233396
##      3  350      134 136.698775
##      4  183       70  73.126037
##      5  160       76  69.076551
##      6   89       40  41.564016
## 
##          Statistic =  3.59394 
## degrees of freedom =  4 
##            p-value =  0.46374  
  ## CIs using profiled log-likelihood
confint(m, level=0.99)  
  ## Waiting for profiling to be done...  
  ##                                            0.5 %     99.5 %
## (Intercept)                           -0.7928951 -0.4423920
## LocationOther European                -0.2050921  0.5584383
## LocationNorth America                 -0.2929641  0.8426611
## LocationAustralia/New Zealand/Oceania -0.3009235  0.9669694
## LocationOther                         -0.6579659  0.9221767
## UrbanYes                              -0.1110495  0.4523258  
  ## CIs using standard errors
confint.default(m, level=0.99)  
  ##                                            0.5 %     99.5 %
## (Intercept)                           -0.7913862 -0.4410923
## LocationOther European                -0.2015139  0.5606447
## LocationNorth America                 -0.2840339  0.8456340
## LocationAustralia/New Zealand/Oceania -0.2922688  0.9676198
## LocationOther                         -0.6346040  0.9290928
## UrbanYes                              -0.1101998  0.4528272  
  # Wald test
wald.test(b = coef(m), Sigma = vcov(m), Terms = 2)  
  ## Wald test:
## ----------
## 
## Chi-squared test:
## X2 = 1.5, df = 1, P(> X2) = 0.22  
  ## odds ratios and 95% CI
exp(cbind(OR = coef(m), confint(m, level=0.99)))  
  ## Waiting for profiling to be done...  
  ##                                              OR     0.5 %    99.5 %
## (Intercept)                           0.5399713 0.4525328 0.6424977
## LocationOther European                1.1966972 0.8145723 1.7479407
## LocationNorth America                 1.3241888 0.7460489 2.3225393
## LocationAustralia/New Zealand/Oceania 1.4016856 0.7401344 2.6299619
## LocationOther                         1.1586371 0.5179038 2.5147582
## UrbanYes                              1.1868630 0.8948945 1.5719640  
 
 Calculate Nagelkerke R^2 
  NagelkerkeR2(m)  
  ## $N
## [1] 1658
## 
## $R2
## [1] 0.00683887  
 
 
 check assumptions of model 
 
 Cook’s distance 
  plot(m, which = 4, id.n = 3)  
   
 
 
 
 
 
 Extract model results and display data for top 3 values using Cook’s
distance 
  model.data <- augment(m) %>% 
  mutate(index = 1:n()) 
model.data %>% top_n(3, .cooksd)  
 
 
 
 
 plot standardised residuals 
  ggplot(model.data, aes(index, .std.resid)) + 
  geom_point(aes(color = Any_Health_Problem), alpha = .5) +
  theme_bw()  
   
 
 
 Filter potential influential data points with abs(.std.res) >
3: 
  model.data %>% 
  filter(abs(.std.resid) > 3)  
 
 
 
 
 
 check for multicollinearity 
  car::vif(m)  
  ##              GVIF Df GVIF^(1/(2*Df))
## Location 1.065118  4        1.007917
## Urban    1.065118  1        1.032045  
 
 
 Create ROCR for training and test data 
  ## training data
pred.mtt = predict(m, type = "response") #repeat risk predictions from model m
rocr.pred.mtt = ROCR::prediction(pred.mtt, labels = ml_train$Any_Health_Problem) #ROCR prediction object
roc.perf.mtt = ROCR::performance(rocr.pred.mtt, measure = "tpr", x.measure = "fpr") # #ROCR performance object
plot(roc.perf.mtt, col = "blue")


pred.te.1 = predict(m, newdata = ml_test, type = "response") #.te = "test"
rocr.pred.te.1 = ROCR::prediction(pred.te.1, labels = ml_test$Any_Health_Problem)
roc.perf.te.1 = ROCR::performance(rocr.pred.te.1, measure = "tpr", x.measure = "fpr")
plot(roc.perf.te.1, col = "red", add = T)

abline(a = 0, b = 1, lty = 2) #diagonal for random assignment
legend("bottomright", legend = c("train","test"),
col = c("blue","red"), lty = c(2,1), lwd =1.5)  
   
 
 
 Report AUC from ROC for training and test data 
    # Train AUC
aucTr <- ROCR::performance(rocr.pred.mtt, measure = "auc")
  aucTr <- aucTr@y.values[[1]]
  print(aucTr)  
  ## [1] 0.5372588  
     # Test AUC
  aucTe <- ROCR::performance(rocr.pred.te.1, measure = "auc")
  aucTe <- aucTe@y.values[[1]]
  print(aucTe)  
  ## [1] 0.492252  
 
 
 LOCATION * URBAN binary logistic regression for HEALTH 
  # fit binary logit model and store results 'm'
m <- glm(Any_Health_Problem ~ Location*Urban, data = ml_train,family = binomial)
# view a summary of the model
summary(m)  
  ## 
## Call:
## glm(formula = Any_Health_Problem ~ Location * Urban, family = binomial, 
##     data = ml_train)
## 
## Coefficients:
##                                                Estimate Std. Error z value
## (Intercept)                                    -0.60256    0.07111  -8.473
## LocationOther European                          0.09994    0.19445   0.514
## LocationNorth America                           0.18135    0.28970   0.626
## LocationAustralia/New Zealand/Oceania           0.76962    0.41580   1.851
## LocationOther                                  -0.90151    0.78496  -1.148
## UrbanYes                                        0.12513    0.13095   0.955
## LocationOther European:UrbanYes                 0.19518    0.30107   0.648
## LocationNorth America:UrbanYes                  0.24479    0.44570   0.549
## LocationAustralia/New Zealand/Oceania:UrbanYes -0.61495    0.51680  -1.190
## LocationOther:UrbanYes                          1.32488    0.85817   1.544
##                                                Pr(>|z|)    
## (Intercept)                                      <2e-16 ***
## LocationOther European                           0.6073    
## LocationNorth America                            0.5313    
## LocationAustralia/New Zealand/Oceania            0.0642 .  
## LocationOther                                    0.2508    
## UrbanYes                                         0.3393    
## LocationOther European:UrbanYes                  0.5168    
## LocationNorth America:UrbanYes                   0.5828    
## LocationAustralia/New Zealand/Oceania:UrbanYes   0.2341    
## LocationOther:UrbanYes                           0.1226    
## ---
## Signif. codes:  0 '***' 0.001 '**' 0.01 '*' 0.05 '.' 0.1 ' ' 1
## 
## (Dispersion parameter for binomial family taken to be 1)
## 
##     Null deviance: 2200.0  on 1657  degrees of freedom
## Residual deviance: 2186.6  on 1648  degrees of freedom
## AIC: 2206.6
## 
## Number of Fisher Scoring iterations: 4  
  # test model fit
with(m, null.deviance - deviance)  
  ## [1] 13.4362  
  with(m, df.null - df.residual)  
  ## [1] 9  
  with(m, pchisq(null.deviance - deviance, df.null - df.residual, lower.tail = FALSE))  
  ## [1] 0.1438413  
  BIC(m)  
  ## [1] 2260.726  
  # Hosmer-Lemeshow Goodness-of-Fit Test
hltest(m)  
  ## 
##    The Hosmer-Lemeshow goodness-of-fit test
## 
##  Group Size Observed Expected
##      1   11        2        2
##      2  865      306      306
##      3  130       49       49
##      4  350      134      134
##      5  202       87       87
##      6  100       50       50
## 
##          Statistic =  0 
## degrees of freedom =  4 
##            p-value =  1  
  ## CIs using profiled log-likelihood
confint(m, level=0.99)  
  ## Waiting for profiling to be done...  
  ##                                                     0.5 %     99.5 %
## (Intercept)                                    -0.7875074 -0.4208996
## LocationOther European                         -0.4103147  0.5950337
## LocationNorth America                          -0.5894560  0.9177774
## LocationAustralia/New Zealand/Oceania          -0.3096629  1.8798662
## LocationOther                                  -3.5858338  0.8504434
## UrbanYes                                       -0.2142658  0.4610421
## LocationOther European:UrbanYes                -0.5801074  0.9738647
## LocationNorth America:UrbanYes                 -0.9040696  1.4052371
## LocationAustralia/New Zealand/Oceania:UrbanYes -1.9744882  0.7158770
## LocationOther:UrbanYes                         -0.6593941  4.1186813  
  ## CIs using standard errors
confint.default(m, level=0.99)  
  ##                                                     0.5 %     99.5 %
## (Intercept)                                    -0.7857361 -0.4193926
## LocationOther European                         -0.4009340  0.6008050
## LocationNorth America                          -0.5648661  0.9275679
## LocationAustralia/New Zealand/Oceania          -0.3014095  1.8406465
## LocationOther                                  -2.9234454  1.1204194
## UrbanYes                                       -0.2121918  0.4624433
## LocationOther European:UrbanYes                -0.5803261  0.9706892
## LocationNorth America:UrbanYes                 -0.9032566  1.3928454
## LocationAustralia/New Zealand/Oceania:UrbanYes -1.9461441  0.7162377
## LocationOther:UrbanYes                         -0.8856025  3.5353713  
  # Wald test
wald.test(b = coef(m), Sigma = vcov(m), Terms = 2)  
  ## Wald test:
## ----------
## 
## Chi-squared test:
## X2 = 0.26, df = 1, P(> X2) = 0.61  
  ## odds ratios and 95% CI
exp(cbind(OR = coef(m), confint(m, level=0.99)))  
  ## Waiting for profiling to be done...  
  ##                                                       OR      0.5 %    99.5 %
## (Intercept)                                    0.5474061 0.45497748  0.656456
## LocationOther European                         1.1050997 0.66344143  1.813092
## LocationNorth America                          1.1988358 0.55462892  2.503719
## LocationAustralia/New Zealand/Oceania          2.1589424 0.73369425  6.552628
## LocationOther                                  0.4059550 0.02771355  2.340684
## UrbanYes                                       1.1332910 0.80713379  1.585726
## LocationOther European:UrbanYes                1.2155316 0.55983822  2.648159
## LocationNorth America:UrbanYes                 1.2773587 0.40491843  4.076493
## LocationAustralia/New Zealand/Oceania:UrbanYes 0.5406662 0.13883236  2.045980
## LocationOther:UrbanYes                         3.7617505 0.51716456 61.478118  
 
 Calculate Nagelkerke R^2 
  NagelkerkeR2(m)  
  ## $N
## [1] 1658
## 
## $R2
## [1] 0.0109855  
 
 
 check assumptions of model 
 
 Cook’s distance 
  plot(m, which = 4, id.n = 3)  
   
 
 
 Extract model results and display data for top 3 values using Cook’s
distance 
  model.data <- augment(m) %>% 
  mutate(index = 1:n()) 
model.data %>% top_n(3, .cooksd)  
 
 
 
 
 
 plot standardised residuals 
  ggplot(model.data, aes(index, .std.resid)) + 
  geom_point(aes(color = Any_Health_Problem), alpha = .5) +
  theme_bw()  
   
 
 
 Filter potential influential data points with abs(.std.res) >
3: 
  model.data %>% 
  filter(abs(.std.resid) > 3)  
 
 
 
 
 
 
 
 
 check for multicollinearity 
  car::vif(m)  
  ## there are higher-order terms (interactions) in this model
## consider setting type = 'predictor'; see ?vif  
  ##                     GVIF Df GVIF^(1/(2*Df))
## Location       58.818677  4        1.664138
## Urban           1.524618  1        1.234754
## Location:Urban 78.503967  4        1.725287  
 
 Create ROCR for training and test data 
  ## training data
pred.mtt = predict(m, type = "response") #repeat risk predictions from model m
rocr.pred.mtt = ROCR::prediction(pred.mtt, labels = ml_train$Any_Health_Problem) #ROCR prediction object
roc.perf.mtt = ROCR::performance(rocr.pred.mtt, measure = "tpr", x.measure = "fpr") # #ROCR performance object
plot(roc.perf.mtt, col = "blue")


pred.te.1 = predict(m, newdata = ml_test, type = "response") #.te = "test"
rocr.pred.te.1 = ROCR::prediction(pred.te.1, labels = ml_test$Any_Health_Problem)
roc.perf.te.1 = ROCR::performance(rocr.pred.te.1, measure = "tpr", x.measure = "fpr")
plot(roc.perf.te.1, col = "red", add = T)

abline(a = 0, b = 1, lty = 2) #diagonal for random assignment
legend("bottomright", legend = c("train","test"),
col = c("blue","red"), lty = c(2,1), lwd =1.5)  
   
 
 
 Report AUC from ROC for training and test data 
    # Train AUC
aucTr <- ROCR::performance(rocr.pred.mtt, measure = "auc")
  aucTr <- aucTr@y.values[[1]]
  print(aucTr)  
  ## [1] 0.541668  
     # Test AUC
  aucTe <- ROCR::performance(rocr.pred.te.1, measure = "auc")
  aucTe <- aucTe@y.values[[1]]
  print(aucTe)  
  ## [1] 0.5009615  
 
 
 EDUCATION Binary logistic regression for HEALTH 
  # fit binary logit model and store results 'm'
m <- glm(Any_Health_Problem ~ Education, data = ml_train,family = binomial)
# view a summary of the model
summary(m)  
  ## 
## Call:
## glm(formula = Any_Health_Problem ~ Education, family = binomial, 
##     data = ml_train)
## 
## Coefficients:
##             Estimate Std. Error z value Pr(>|z|)    
## (Intercept) -0.38893    0.08975  -4.333 1.47e-05 ***
## Education.L -0.28537    0.30164  -0.946    0.344    
## Education.Q  0.38816    0.27605   1.406    0.160    
## Education.C -0.18217    0.20795  -0.876    0.381    
## Education^4  0.09498    0.14305   0.664    0.507    
## Education^5 -0.12387    0.10381  -1.193    0.233    
## ---
## Signif. codes:  0 '***' 0.001 '**' 0.01 '*' 0.05 '.' 0.1 ' ' 1
## 
## (Dispersion parameter for binomial family taken to be 1)
## 
##     Null deviance: 2200.0  on 1657  degrees of freedom
## Residual deviance: 2196.9  on 1652  degrees of freedom
## AIC: 2208.9
## 
## Number of Fisher Scoring iterations: 4  
  # test model fit
with(m, null.deviance - deviance)  
  ## [1] 3.155165  
  with(m, df.null - df.residual)  
  ## [1] 5  
  with(m, pchisq(null.deviance - deviance, df.null - df.residual, lower.tail = FALSE))  
  ## [1] 0.6760781  
  BIC(m)  
  ## [1] 2241.354  
  # Hosmer-Lemeshow Goodness-of-Fit Test
hltest(m)  
  ## 
##    The Hosmer-Lemeshow goodness-of-fit test
## 
##  Group Size Observed Expected
##      1  496      178      178
##      2  265       99       99
##      3  482      186      186
##      4  332      129      129
##      5   60       24       24
##      6   23       12       12
## 
##          Statistic =  0 
## degrees of freedom =  4 
##            p-value =  1  
  ## CIs using profiled log-likelihood
confint(m, level=0.99)  
  ## Waiting for profiling to be done...  
  ##                  0.5 %     99.5 %
## (Intercept) -0.6221441 -0.1553046
## Education.L -1.0778440  0.4952147
## Education.Q -0.3305065  1.1091259
## Education.C -0.7254481  0.3547633
## Education^4 -0.2734692  0.4654772
## Education^5 -0.3916543  0.1433863  
  ## CIs using standard errors
confint.default(m, level=0.99)  
  ##                  0.5 %     99.5 %
## (Intercept) -0.6201233 -0.1577419
## Education.L -1.0623385  0.4915894
## Education.Q -0.3229093  1.0992227
## Education.C -0.7178158  0.3534735
## Education^4 -0.2734996  0.4634530
## Education^5 -0.3912594  0.1435164  
  # Wald test
wald.test(b = coef(m), Sigma = vcov(m), Terms = 2)  
  ## Wald test:
## ----------
## 
## Chi-squared test:
## X2 = 0.9, df = 1, P(> X2) = 0.34  
  ## odds ratios and 95% CI
exp(cbind(OR = coef(m), confint(m, level=0.99)))  
  ## Waiting for profiling to be done...  
  ##                    OR     0.5 %    99.5 %
## (Intercept) 0.6777799 0.5367923 0.8561543
## Education.L 0.7517327 0.3403285 1.6408506
## Education.Q 1.4742608 0.7185597 3.0317071
## Education.C 0.8334587 0.4841076 1.4258430
## Education^4 1.0996333 0.7607358 1.5927740
## Education^5 0.8834934 0.6759377 1.1541756  
 
 Calculate Nagelkerke R^2 
  NagelkerkeR2(m)  
  ## $N
## [1] 1658
## 
## $R2
## [1] 0.002587681  
 
 
 check assumptions of model 
 
 Cook’s distance 
  plot(m, which = 4, id.n = 3)  
   
 
 
 Extract model results and display data for top 3 values using Cook’s
distance 
  model.data <- augment(m) %>% 
  mutate(index = 1:n()) 
model.data %>% top_n(3, .cooksd)  
 
 
 
 
 
 plot standardised residuals 
  ggplot(model.data, aes(index, .std.resid)) + 
  geom_point(aes(color = Any_Health_Problem), alpha = .5) +
  theme_bw()  
   
 
 
 
 
 
 Filter potential influential data points with abs(.std.res) >
3: 
  model.data %>% 
  filter(abs(.std.resid) > 3)  
 
 
 
 
 Create ROCR for training and test data 
  ## training data
pred.mtt = predict(m, type = "response") #repeat risk predictions from model m
rocr.pred.mtt = ROCR::prediction(pred.mtt, labels = ml_train$Any_Health_Problem) #ROCR prediction object
roc.perf.mtt = ROCR::performance(rocr.pred.mtt, measure = "tpr", x.measure = "fpr") # #ROCR performance object
plot(roc.perf.mtt, col = "blue")


pred.te.1 = predict(m, newdata = ml_test, type = "response") #.te = "test"
rocr.pred.te.1 = ROCR::prediction(pred.te.1, labels = ml_test$Any_Health_Problem)
roc.perf.te.1 = ROCR::performance(rocr.pred.te.1, measure = "tpr", x.measure = "fpr")
plot(roc.perf.te.1, col = "red", add = T)

abline(a = 0, b = 1, lty = 2) #diagonal for random assignment
legend("bottomright", legend = c("train","test"),
col = c("blue","red"), lty = c(2,1), lwd =1.5)  
   
 
 
 Report AUC from ROC for training and test data 
    # Train AUC
aucTr <- ROCR::performance(rocr.pred.mtt, measure = "auc")
  aucTr <- aucTr@y.values[[1]]
  print(aucTr)  
  ## [1] 0.518806  
     # Test AUC
  aucTe <- ROCR::performance(rocr.pred.te.1, measure = "auc")
  aucTe <- aucTe@y.values[[1]]
  print(aucTe)  
  ## [1] 0.4467113  
 
 
 EDUCATION_S Binary logistic regression for HEALTH 
  # fit binary logit model and store results 'm'
m <- glm(Any_Health_Problem ~ Education_S, data = ml_train,family = binomial)
# view a summary of the model
summary(m)  
  ## 
## Call:
## glm(formula = Any_Health_Problem ~ Education_S, family = binomial, 
##     data = ml_train)
## 
## Coefficients:
##                         Estimate Std. Error z value Pr(>|z|)    
## (Intercept)            -0.466620   0.121073  -3.854 0.000116 ***
## Education_S1_College    0.002007   0.153015   0.013 0.989536    
## Education_S2_Grad      -0.113648   0.153040  -0.743 0.457722    
## Education_S3_PG_or_PhD  0.020594   0.159307   0.129 0.897143    
## ---
## Signif. codes:  0 '***' 0.001 '**' 0.01 '*' 0.05 '.' 0.1 ' ' 1
## 
## (Dispersion parameter for binomial family taken to be 1)
## 
##     Null deviance: 2200.0  on 1657  degrees of freedom
## Residual deviance: 2198.8  on 1654  degrees of freedom
## AIC: 2206.8
## 
## Number of Fisher Scoring iterations: 4  
  # test model fit
with(m, null.deviance - deviance)  
  ## [1] 1.219139  
  with(m, df.null - df.residual)  
  ## [1] 3  
  with(m, pchisq(null.deviance - deviance, df.null - df.residual, lower.tail = FALSE))  
  ## [1] 0.7484177  
  BIC(m)  
  ## [1] 2228.463  
  # Hosmer-Lemeshow Goodness-of-Fit Test
hltest(m, G=3)  
  ## 
##    The Hosmer-Lemeshow goodness-of-fit test
## 
##  Group Size Observed Expected
##      1  496      178      178
##      2  288      111      111
##      3  482      186      186
##      4  392      153      153
## 
##          Statistic =  0 
## degrees of freedom =  2 
##            p-value =  1  
  ## CIs using profiled log-likelihood
confint(m, level=0.99)  
  ## Waiting for profiling to be done...  
  ##                             0.5 %     99.5 %
## (Intercept)            -0.7828572 -0.1578385
## Education_S1_College   -0.3910516  0.3980878
## Education_S2_Grad      -0.5069794  0.2823099
## Education_S3_PG_or_PhD -0.3891407  0.4324399  
  ## CIs using standard errors
confint.default(m, level=0.99)  
  ##                             0.5 %     99.5 %
## (Intercept)            -0.7784835 -0.1547556
## Education_S1_College   -0.3921326  0.3961461
## Education_S2_Grad      -0.5078542  0.2805576
## Education_S3_PG_or_PhD -0.3897542  0.4309420  
  # Wald test
wald.test(b = coef(m), Sigma = vcov(m), Terms = 2)  
  ## Wald test:
## ----------
## 
## Chi-squared test:
## X2 = 0.00017, df = 1, P(> X2) = 0.99  
  ## odds ratios and 95% CI
exp(cbind(OR = coef(m), confint(m, level=0.99)))  
  ## Waiting for profiling to be done...  
  ##                               OR     0.5 %    99.5 %
## (Intercept)            0.6271186 0.4570981 0.8539877
## Education_S1_College   1.0020088 0.6763453 1.4889748
## Education_S2_Grad      0.8925718 0.6023122 1.3261897
## Education_S3_PG_or_PhD 1.0208074 0.6776389 1.5410128  
 
 Calculate Nagelkerke R^2 
  NagelkerkeR2(m)  
  ## $N
## [1] 1658
## 
## $R2
## [1] 0.00100045  
 
 
 check assumptions of model 
 
 Cook’s distance 
  plot(m, which = 4, id.n = 3)  
   
 
 
 Extract model results and display data for top 3 values using Cook’s
distance 
  model.data <- augment(m) %>% 
  mutate(index = 1:n()) 
model.data %>% top_n(3, .cooksd)  
 
 
 
 
 
 plot standardised residuals 
  ggplot(model.data, aes(index, .std.resid)) + 
  geom_point(aes(color = Any_Health_Problem), alpha = .5) +
  theme_bw()  
   
 
 
 Filter potential influential data points with abs(.std.res) >
3: 
  model.data %>% 
  filter(abs(.std.resid) > 3)  
 
 
 
 
 
 
 Create ROCR for training and test data 
  ## training data
pred.mtt = predict(m, type = "response") #repeat risk predictions from model m
rocr.pred.mtt = ROCR::prediction(pred.mtt, labels = ml_train$Health_Binary) #ROCR prediction object
roc.perf.mtt = ROCR::performance(rocr.pred.mtt, measure = "tpr", x.measure = "fpr") # #ROCR performance object
plot(roc.perf.mtt, col = "blue")


pred.te.1 = predict(m, newdata = ml_test, type = "response") #.te = "test"
rocr.pred.te.1 = ROCR::prediction(pred.te.1, labels = ml_test$Health_Binary)
roc.perf.te.1 = ROCR::performance(rocr.pred.te.1, measure = "tpr", x.measure = "fpr")
plot(roc.perf.te.1, col = "red", add = T)

abline(a = 0, b = 1, lty = 2) #diagonal for random assignment
legend("bottomright", legend = c("train","test"),
col = c("blue","red"), lty = c(2,1), lwd =1.5)  
   
 
 
 Report AUC from ROC for training and test data 
    # Train AUC
aucTr <- ROCR::performance(rocr.pred.mtt, measure = "auc")
  aucTr <- aucTr@y.values[[1]]
  print(aucTr)  
  ## [1] 0.5041089  
     # Test AUC
  aucTe <- ROCR::performance(rocr.pred.te.1, measure = "auc")
  aucTe <- aucTe@y.values[[1]]
  print(aucTe)  
  ## [1] 0.4436891  
 
 
 
 EDUCATION_S Binary logistic regression for HEALTH 
  # fit binary logit model and store results 'm'
m <- glm(Any_Health_Problem ~ Education_S2, data = ml_train,family = binomial)
# view a summary of the model
summary(m)  
  ## 
## Call:
## glm(formula = Any_Health_Problem ~ Education_S2, family = binomial, 
##     data = ml_train)
## 
## Coefficients:
##                          Estimate Std. Error z value Pr(>|z|)    
## (Intercept)             -0.466620   0.121073  -3.854 0.000116 ***
## Education_S21_College    0.002007   0.153015   0.013 0.989536    
## Education_S22_Grad      -0.113648   0.153040  -0.743 0.457722    
## Education_S23_PG_or_PhD  0.020594   0.159307   0.129 0.897143    
## ---
## Signif. codes:  0 '***' 0.001 '**' 0.01 '*' 0.05 '.' 0.1 ' ' 1
## 
## (Dispersion parameter for binomial family taken to be 1)
## 
##     Null deviance: 2200.0  on 1657  degrees of freedom
## Residual deviance: 2198.8  on 1654  degrees of freedom
## AIC: 2206.8
## 
## Number of Fisher Scoring iterations: 4  
  # test model fit
with(m, null.deviance - deviance)  
  ## [1] 1.219139  
  with(m, df.null - df.residual)  
  ## [1] 3  
  with(m, pchisq(null.deviance - deviance, df.null - df.residual, lower.tail = FALSE))  
  ## [1] 0.7484177  
  BIC(m)  
  ## [1] 2228.463  
  # Hosmer-Lemeshow Goodness-of-Fit Test
hltest(m, G=3)  
  ## 
##    The Hosmer-Lemeshow goodness-of-fit test
## 
##  Group Size Observed Expected
##      1  496      178      178
##      2  288      111      111
##      3  482      186      186
##      4  392      153      153
## 
##          Statistic =  0 
## degrees of freedom =  2 
##            p-value =  1  
  ## CIs using profiled log-likelihood
confint(m, level=0.99)  
  ## Waiting for profiling to be done...  
  ##                              0.5 %     99.5 %
## (Intercept)             -0.7828572 -0.1578385
## Education_S21_College   -0.3910516  0.3980878
## Education_S22_Grad      -0.5069794  0.2823099
## Education_S23_PG_or_PhD -0.3891407  0.4324399  
  ## CIs using standard errors
confint.default(m, level=0.99)  
  ##                              0.5 %     99.5 %
## (Intercept)             -0.7784835 -0.1547556
## Education_S21_College   -0.3921326  0.3961461
## Education_S22_Grad      -0.5078542  0.2805576
## Education_S23_PG_or_PhD -0.3897542  0.4309420  
  # Wald test
wald.test(b = coef(m), Sigma = vcov(m), Terms = 2)  
  ## Wald test:
## ----------
## 
## Chi-squared test:
## X2 = 0.00017, df = 1, P(> X2) = 0.99  
  ## odds ratios and 95% CI
exp(cbind(OR = coef(m), confint(m, level=0.99)))  
  ## Waiting for profiling to be done...  
  ##                                OR     0.5 %    99.5 %
## (Intercept)             0.6271186 0.4570981 0.8539877
## Education_S21_College   1.0020088 0.6763453 1.4889748
## Education_S22_Grad      0.8925718 0.6023122 1.3261897
## Education_S23_PG_or_PhD 1.0208074 0.6776389 1.5410128  
 
 Calculate Nagelkerke R^2 
  NagelkerkeR2(m)  
  ## $N
## [1] 1658
## 
## $R2
## [1] 0.00100045  
 
 
 check assumptions of model 
 
 Cook’s distance 
  plot(m, which = 4, id.n = 3)  
   
 
 
 Extract model results and display data for top 3 values using Cook’s
distance 
  model.data <- augment(m) %>% 
  mutate(index = 1:n()) 
model.data %>% top_n(3, .cooksd)  
 
 
 
 
 
 plot standardised residuals 
  ggplot(model.data, aes(index, .std.resid)) + 
  geom_point(aes(color = Any_Health_Problem), alpha = .5) +
  theme_bw()  
   
 
 
 Filter potential influential data points with abs(.std.res) >
3: 
  model.data %>% 
  filter(abs(.std.resid) > 3)  
 
 
 
 
 
 
 Create ROCR for training and test data 
  ## training data
pred.mtt = predict(m, type = "response") #repeat risk predictions from model m
rocr.pred.mtt = ROCR::prediction(pred.mtt, labels = ml_train$Any_Health_Problem) #ROCR prediction object
roc.perf.mtt = ROCR::performance(rocr.pred.mtt, measure = "tpr", x.measure = "fpr") # #ROCR performance object
plot(roc.perf.mtt, col = "blue")


pred.te.1 = predict(m, newdata = ml_test, type = "response") #.te = "test"
rocr.pred.te.1 = ROCR::prediction(pred.te.1, labels = ml_test$Any_Health_Problem)
roc.perf.te.1 = ROCR::performance(rocr.pred.te.1, measure = "tpr", x.measure = "fpr")
plot(roc.perf.te.1, col = "red", add = T)

abline(a = 0, b = 1, lty = 2) #diagonal for random assignment
legend("bottomright", legend = c("train","test"),
col = c("blue","red"), lty = c(2,1), lwd =1.5)  
   
 
 
 Report AUC from ROC for training and test data 
    # Train AUC
aucTr <- ROCR::performance(rocr.pred.mtt, measure = "auc")
  aucTr <- aucTr@y.values[[1]]
  print(aucTr)  
  ## [1] 0.5137716  
     # Test AUC
  aucTe <- ROCR::performance(rocr.pred.te.1, measure = "auc")
  aucTe <- aucTe@y.values[[1]]
  print(aucTe)  
  ## [1] 0.4395764  
 
 
 
 ANIMAL CAREER 2 Binary logistic regression for HEALTH 
  # fit binary logit model and store results 'm'
m <- glm(Any_Health_Problem ~ Animal_Career2, data = ml_train,family = binomial)
# view a summary of the model
summary(m)  
  ## 
## Call:
## glm(formula = Any_Health_Problem ~ Animal_Career2, family = binomial, 
##     data = ml_train)
## 
## Coefficients:
##                                   Estimate Std. Error z value Pr(>|z|)    
## (Intercept)                       -0.51566    0.05680  -9.078   <2e-16 ***
## Animal_Career2Vet professional     0.13125    0.23612   0.556    0.578    
## Animal_Career2breeder/trainer      0.06575    0.20111   0.327    0.744    
## Animal_Career2Pet industry worker  0.11606    0.18037   0.643    0.520    
## ---
## Signif. codes:  0 '***' 0.001 '**' 0.01 '*' 0.05 '.' 0.1 ' ' 1
## 
## (Dispersion parameter for binomial family taken to be 1)
## 
##     Null deviance: 2200.0  on 1657  degrees of freedom
## Residual deviance: 2199.3  on 1654  degrees of freedom
## AIC: 2207.3
## 
## Number of Fisher Scoring iterations: 4  
  # test model fit
with(m, null.deviance - deviance)  
  ## [1] 0.7265285  
  with(m, df.null - df.residual)  
  ## [1] 3  
  with(m, pchisq(null.deviance - deviance, df.null - df.residual, lower.tail = FALSE))  
  ## [1] 0.8669471  
  BIC(m)  
  ## [1] 2228.956  
  ## CIs using profiled log-likelihood
confint(m, level=0.99)  
  ## Waiting for profiling to be done...  
  ##                                        0.5 %     99.5 %
## (Intercept)                       -0.6629434 -0.3701848
## Animal_Career2Vet professional    -0.4914322  0.7330182
## Animal_Career2breeder/trainer     -0.4630080  0.5777318
## Animal_Career2Pet industry worker -0.3558442  0.5765426  
  ## CIs using standard errors
confint.default(m, level=0.99)  
  ##                                        0.5 %     99.5 %
## (Intercept)                       -0.6619748 -0.3693500
## Animal_Career2Vet professional    -0.4769564  0.7394578
## Animal_Career2breeder/trainer     -0.4522876  0.5837787
## Animal_Career2Pet industry worker -0.3485521  0.5806769  
  # Wald test
wald.test(b = coef(m), Sigma = vcov(m), Terms = 2)  
  ## Wald test:
## ----------
## 
## Chi-squared test:
## X2 = 0.31, df = 1, P(> X2) = 0.58  
  ## odds ratios and 95% CI
exp(cbind(OR = coef(m), confint(m, level=0.99)))  
  ## Waiting for profiling to be done...  
  ##                                          OR     0.5 %    99.5 %
## (Intercept)                       0.5971049 0.5153322 0.6906067
## Animal_Career2Vet professional    1.1402536 0.6117496 2.0813530
## Animal_Career2breeder/trainer     1.0679549 0.6293876 1.7819919
## Animal_Career2Pet industry worker 1.1230660 0.7005818 1.7798741  
 
 Calculate Nagelkerke R^2 
  NagelkerkeR2(m)  
  ## $N
## [1] 1658
## 
## $R2
## [1] 0.0005962924  
 
 
 check assumptions of model 
 
 Cook’s distance 
  plot(m, which = 4, id.n = 3)  
   
 
 
 Extract model results and display data for top 3 values using Cook’s
distance 
  model.data <- augment(m) %>% 
  mutate(index = 1:n()) 
model.data %>% top_n(3, .cooksd)  
 
 
 
 
 
 plot standardised residuals 
  ggplot(model.data, aes(index, .std.resid)) + 
  geom_point(aes(color = Any_Health_Problem), alpha = .5) +
  theme_bw()  
   
 
 
 Filter potential influential data points with abs(.std.res) >
3: 
  model.data %>% 
  filter(abs(.std.resid) > 3)  
 
 
 
 
 
 
 Create ROCR for training and test data 
  ## training data
pred.mtt = predict(m, type = "response") #repeat risk predictions from model m
rocr.pred.mtt = ROCR::prediction(pred.mtt, labels = ml_train$Any_Health_Problem) #ROCR prediction object
roc.perf.mtt = ROCR::performance(rocr.pred.mtt, measure = "tpr", x.measure = "fpr") # #ROCR performance object
plot(roc.perf.mtt, col = "blue")


pred.te.1 = predict(m, newdata = ml_test, type = "response") #.te = "test"
rocr.pred.te.1 = ROCR::prediction(pred.te.1, labels = ml_test$Any_Health_Problem)
roc.perf.te.1 = ROCR::performance(rocr.pred.te.1, measure = "tpr", x.measure = "fpr")
plot(roc.perf.te.1, col = "red", add = T)

abline(a = 0, b = 1, lty = 2) #diagonal for random assignment
legend("bottomright", legend = c("train","test"),
col = c("blue","red"), lty = c(2,1), lwd =1.5)  
   
 
 
 Report AUC from ROC for training and test data 
    # Train AUC
aucTr <- ROCR::performance(rocr.pred.mtt, measure = "auc")
  aucTr <- aucTr@y.values[[1]]
  print(aucTr)  
  ## [1] 0.508608  
     # Test AUC
  aucTe <- ROCR::performance(rocr.pred.te.1, measure = "auc")
  aucTe <- aucTe@y.values[[1]]
  print(aucTe)  
  ## [1] 0.543743  
 
 
 
 ANIMAL_CAREER_BINARY Binary logistic regression for HEALTH 
  # fit binary logit model and store results 'm'
m <- glm(Any_Health_Problem ~ Animal_Career_BINARY, data = ml_train,family = binomial)
# view a summary of the model
summary(m)  
  ## 
## Call:
## glm(formula = Any_Health_Problem ~ Animal_Career_BINARY, family = binomial, 
##     data = ml_train)
## 
## Coefficients:
##                         Estimate Std. Error z value Pr(>|z|)    
## (Intercept)              -0.5157     0.0568  -9.078   <2e-16 ***
## Animal_Career_BINARYYes   0.1027     0.1254   0.819    0.413    
## ---
## Signif. codes:  0 '***' 0.001 '**' 0.01 '*' 0.05 '.' 0.1 ' ' 1
## 
## (Dispersion parameter for binomial family taken to be 1)
## 
##     Null deviance: 2200.0  on 1657  degrees of freedom
## Residual deviance: 2199.4  on 1656  degrees of freedom
## AIC: 2203.4
## 
## Number of Fisher Scoring iterations: 4  
  # test model fit
with(m, null.deviance - deviance)  
  ## [1] 0.6681625  
  with(m, df.null - df.residual)  
  ## [1] 1  
  with(m, pchisq(null.deviance - deviance, df.null - df.residual, lower.tail = FALSE))  
  ## [1] 0.413693  
  BIC(m)  
  ## [1] 2214.187  
  ## CIs using profiled log-likelihood
confint(m, level=0.99)  
  ## Waiting for profiling to be done...  
  ##                              0.5 %     99.5 %
## (Intercept)             -0.6629434 -0.3701848
## Animal_Career_BINARYYes -0.2226944  0.4239842  
  ## CIs using standard errors
confint.default(m, level=0.99)  
  ##                              0.5 %     99.5 %
## (Intercept)             -0.6619748 -0.3693500
## Animal_Career_BINARYYes -0.2202534  0.4256667  
  # Wald test
wald.test(b = coef(m), Sigma = vcov(m), Terms = 2)  
  ## Wald test:
## ----------
## 
## Chi-squared test:
## X2 = 0.67, df = 1, P(> X2) = 0.41  
  ## odds ratios and 95% CI
exp(cbind(OR = coef(m), confint(m, level=0.99)))  
  ## Waiting for profiling to be done...  
  ##                                OR     0.5 %    99.5 %
## (Intercept)             0.5971049 0.5153322 0.6906067
## Animal_Career_BINARYYes 1.1081662 0.8003594 1.5280375  
 
 Calculate Nagelkerke R^2 
  NagelkerkeR2(m)  
  ## $N
## [1] 1658
## 
## $R2
## [1] 0.0005483987  
 
 
 check assumptions of model 
 
 Cook’s distance 
  plot(m, which = 4, id.n = 3)  
   
 
 
 Extract model results and display data for top 3 values using Cook’s
distance 
  model.data <- augment(m) %>% 
  mutate(index = 1:n()) 
model.data %>% top_n(3, .cooksd)  
 
 
 
 
 
 plot standardised residuals 
  ggplot(model.data, aes(index, .std.resid)) + 
  geom_point(aes(color = Any_Health_Problem), alpha = .5) +
  theme_bw()  
   
 
 
 Filter potential influential data points with abs(.std.res) >
3: 
  model.data %>% 
  filter(abs(.std.resid) > 3)  
 
 
 
 
 
 
 Create ROCR for training and test data 
  ## training data
pred.mtt = predict(m, type = "response") #repeat risk predictions from model m
rocr.pred.mtt = ROCR::prediction(pred.mtt, labels = ml_train$Any_Health_Problem) #ROCR prediction object
roc.perf.mtt = ROCR::performance(rocr.pred.mtt, measure = "tpr", x.measure = "fpr") # #ROCR performance object
plot(roc.perf.mtt, col = "blue")


pred.te.1 = predict(m, newdata = ml_test, type = "response") #.te = "test"
rocr.pred.te.1 = ROCR::prediction(pred.te.1, labels = ml_test$Any_Health_Problem)
roc.perf.te.1 = ROCR::performance(rocr.pred.te.1, measure = "tpr", x.measure = "fpr")
plot(roc.perf.te.1, col = "red", add = T)

abline(a = 0, b = 1, lty = 2) #diagonal for random assignment
legend("bottomright", legend = c("train","test"),
col = c("blue","red"), lty = c(2,1), lwd =1.5)  
   
 
 
 Report AUC from ROC for training and test data 
    # Train AUC
aucTr <- ROCR::performance(rocr.pred.mtt, measure = "auc")
  aucTr <- aucTr@y.values[[1]]
  print(aucTr)  
  ## [1] 0.5083189  
     # Test AUC
  aucTe <- ROCR::performance(rocr.pred.te.1, measure = "auc")
  aucTe <- aucTe@y.values[[1]]
  print(aucTe)  
  ## [1] 0.5415064  
 
 
 
 INCOME Binary logistic regression for HEALTH 
  # fit binary logit model and store results 'm'
m <- glm(Any_Health_Problem ~ Income, data = ml_train,family = binomial)
# view a summary of the model
summary(m)  
  ## 
## Call:
## glm(formula = Any_Health_Problem ~ Income, family = binomial, 
##     data = ml_train)
## 
## Coefficients:
##              Estimate Std. Error z value Pr(>|z|)    
## (Intercept) -0.497287   0.064906  -7.662 1.84e-14 ***
## Income.L    -0.091045   0.130860  -0.696    0.487    
## Income.Q    -0.003104   0.090292  -0.034    0.973    
## ---
## Signif. codes:  0 '***' 0.001 '**' 0.01 '*' 0.05 '.' 0.1 ' ' 1
## 
## (Dispersion parameter for binomial family taken to be 1)
## 
##     Null deviance: 2200.0  on 1657  degrees of freedom
## Residual deviance: 2199.5  on 1655  degrees of freedom
## AIC: 2205.5
## 
## Number of Fisher Scoring iterations: 4  
  # test model fit
with(m, null.deviance - deviance)  
  ## [1] 0.4846146  
  with(m, df.null - df.residual)  
  ## [1] 2  
  with(m, pchisq(null.deviance - deviance, df.null - df.residual, lower.tail = FALSE))  
  ## [1] 0.784815  
  BIC(m)  
  ## [1] 2221.784  
  # Hosmer-Lemeshow Goodness-of-Fit Test
glmtoolbox::hltest(m)  
  ## 
##    The Hosmer-Lemeshow goodness-of-fit test
## 
##  Group Size Observed Expected
##      1  237       86       86
##      2 1159      439      439
##      3  262      103      103
## 
##          Statistic =  0 
## degrees of freedom =  1 
##            p-value =  1  
  ## CIs using profiled log-likelihood
confint(m, level=0.99)  
  ## Waiting for profiling to be done...  
  ##                  0.5 %     99.5 %
## (Intercept) -0.6661010 -0.3313846
## Income.L    -0.4294393  0.2456634
## Income.Q    -0.2369586  0.2285247  
  ## CIs using standard errors
confint.default(m, level=0.99)  
  ##                  0.5 %     99.5 %
## (Intercept) -0.6644736 -0.3300994
## Income.L    -0.4281172  0.2460268
## Income.Q    -0.2356806  0.2294718  
  # Wald test
wald.test(b = coef(m), Sigma = vcov(m), Terms = 2)  
  ## Wald test:
## ----------
## 
## Chi-squared test:
## X2 = 0.48, df = 1, P(> X2) = 0.49  
  ## odds ratios and 95% CI
exp(cbind(OR = coef(m), confint(m, level=0.99)))  
  ## Waiting for profiling to be done...  
  ##                    OR     0.5 %   99.5 %
## (Intercept) 0.6081787 0.5137076 0.717929
## Income.L    0.9129765 0.6508739 1.278469
## Income.Q    0.9969004 0.7890239 1.256745  
 
 Calculate Nagelkerke R^2 
  NagelkerkeR2(m)  
  ## $N
## [1] 1658
## 
## $R2
## [1] 0.0003977725  
 
 
 check assumptions of model 
 
 Cook’s distance 
  plot(m, which = 4, id.n = 3)  
   
 
 
 Extract model results and display data for top 3 values using Cook’s
distance 
  model.data <- augment(m) %>% 
  mutate(index = 1:n()) 
model.data %>% top_n(3, .cooksd)  
 
 
 
 
 
 plot standardised residuals 
  ggplot(model.data, aes(index, .std.resid)) + 
  geom_point(aes(color = Any_Health_Problem), alpha = .5) +
  theme_bw()  
   
 
 
 Filter potential influential data points with abs(.std.res) >
3: 
  model.data %>% 
  filter(abs(.std.resid) > 3)  
 
 
 
 
 
 
 Create ROCR for training and test data 
  ## training data
pred.mtt = predict(m, type = "response") #repeat risk predictions from model m
rocr.pred.mtt = ROCR::prediction(pred.mtt, labels = ml_train$Any_Health_Problem) #ROCR prediction object
roc.perf.mtt = ROCR::performance(rocr.pred.mtt, measure = "tpr", x.measure = "fpr") # #ROCR performance object
plot(roc.perf.mtt, col = "blue")


pred.te.1 = predict(m, newdata = ml_test, type = "response") #.te = "test"
rocr.pred.te.1 = ROCR::prediction(pred.te.1, labels = ml_test$Any_Health_Problem)
roc.perf.te.1 = ROCR::performance(rocr.pred.te.1, measure = "tpr", x.measure = "fpr")
plot(roc.perf.te.1, col = "red", add = T)

abline(a = 0, b = 1, lty = 2) #diagonal for random assignment
legend("bottomright", legend = c("train","test"),
col = c("blue","red"), lty = c(2,1), lwd =1.5)  
   
 
 
 Report AUC from ROC for training and test data 
    # Train AUC
aucTr <- ROCR::performance(rocr.pred.mtt, measure = "auc")
  aucTr <- aucTr@y.values[[1]]
  print(aucTr)  
  ## [1] 0.5081991  
     # Test AUC
  aucTe <- ROCR::performance(rocr.pred.te.1, measure = "auc")
  aucTe <- aucTe@y.values[[1]]
  print(aucTe)  
  ## [1] 0.5377857  
 
 
 
 INCOME2 Binary logistic regression for HEALTH 
  # fit binary logit model and store results 'm'
m <- glm(Any_Health_Problem ~ Income2, data = ml_train,family = binomial)
# view a summary of the model
summary(m)  
  ## 
## Call:
## glm(formula = Any_Health_Problem ~ Income2, family = binomial, 
##     data = ml_train)
## 
## Coefficients:
##               Estimate Std. Error z value Pr(>|z|)    
## (Intercept)   -0.43418    0.12648  -3.433 0.000598 ***
## Income2Medium -0.06058    0.14023  -0.432 0.665759    
## Income2High   -0.12876    0.18506  -0.696 0.486587    
## ---
## Signif. codes:  0 '***' 0.001 '**' 0.01 '*' 0.05 '.' 0.1 ' ' 1
## 
## (Dispersion parameter for binomial family taken to be 1)
## 
##     Null deviance: 2200.0  on 1657  degrees of freedom
## Residual deviance: 2199.5  on 1655  degrees of freedom
## AIC: 2205.5
## 
## Number of Fisher Scoring iterations: 4  
  # test model fit
with(m, null.deviance - deviance)  
  ## [1] 0.4846146  
  with(m, df.null - df.residual)  
  ## [1] 2  
  with(m, pchisq(null.deviance - deviance, df.null - df.residual, lower.tail = FALSE))  
  ## [1] 0.784815  
  BIC(m)  
  ## [1] 2221.784  
  # Hosmer-Lemeshow Goodness-of-Fit Test
glmtoolbox::hltest(m)  
  ## 
##    The Hosmer-Lemeshow goodness-of-fit test
## 
##  Group Size Observed Expected
##      1  237       86       86
##      2 1159      439      439
##      3  262      103      103
## 
##          Statistic =  0 
## degrees of freedom =  1 
##            p-value =  1  
  ## CIs using profiled log-likelihood
confint(m, level=0.99)  
  ## Waiting for profiling to be done...  
  ##                    0.5 %     99.5 %
## (Intercept)   -0.7645050 -0.1114372
## Income2Medium -0.4194435  0.3041113
## Income2High   -0.6073189  0.3474206  
  ## CIs using standard errors
confint.default(m, level=0.99)  
  ##                    0.5 %     99.5 %
## (Intercept)   -0.7599748 -0.1083756
## Income2Medium -0.4217887  0.3006355
## Income2High   -0.6054491  0.3479344  
  # Wald test
wald.test(b = coef(m), Sigma = vcov(m), Terms = 2)  
  ## Wald test:
## ----------
## 
## Chi-squared test:
## X2 = 0.19, df = 1, P(> X2) = 0.67  
  ## odds ratios and 95% CI
exp(cbind(OR = coef(m), confint(m, level=0.99)))  
  ## Waiting for profiling to be done...  
  ##                      OR     0.5 %    99.5 %
## (Intercept)   0.6477987 0.4655643 0.8945476
## Income2Medium 0.9412217 0.6574125 1.3554199
## Income2High   0.8791873 0.5448096 1.4154119  
 
 Calculate Nagelkerke R^2 
  NagelkerkeR2(m)  
  ## $N
## [1] 1658
## 
## $R2
## [1] 0.0003977725  
 
 
 check assumptions of model 
 
 Cook’s distance 
  plot(m, which = 4, id.n = 3)  
   
 
 
 Extract model results and display data for top 3 values using Cook’s
distance 
  model.data <- augment(m) %>% 
  mutate(index = 1:n()) 
model.data %>% top_n(3, .cooksd)  
 
 
 
 
 
 plot standardised residuals 
  ggplot(model.data, aes(index, .std.resid)) + 
  geom_point(aes(color = Any_Health_Problem), alpha = .5) +
  theme_bw()  
   
 
 
 Filter potential influential data points with abs(.std.res) >
3: 
  model.data %>% 
  filter(abs(.std.resid) > 3)  
 
 
 
 
 
 
 Create ROCR for training and test data 
  ## training data
pred.mtt = predict(m, type = "response") #repeat risk predictions from model m
rocr.pred.mtt = ROCR::prediction(pred.mtt, labels = ml_train$Any_Health_Problem) #ROCR prediction object
roc.perf.mtt = ROCR::performance(rocr.pred.mtt, measure = "tpr", x.measure = "fpr") # #ROCR performance object
plot(roc.perf.mtt, col = "blue")


pred.te.1 = predict(m, newdata = ml_test, type = "response") #.te = "test"
rocr.pred.te.1 = ROCR::prediction(pred.te.1, labels = ml_test$Any_Health_Problem)
roc.perf.te.1 = ROCR::performance(rocr.pred.te.1, measure = "tpr", x.measure = "fpr")
plot(roc.perf.te.1, col = "red", add = T)

abline(a = 0, b = 1, lty = 2) #diagonal for random assignment
legend("bottomright", legend = c("train","test"),
col = c("blue","red"), lty = c(2,1), lwd =1.5)  
   
 
 
 Report AUC from ROC for training and test data 
    # Train AUC
aucTr <- ROCR::performance(rocr.pred.mtt, measure = "auc")
  aucTr <- aucTr@y.values[[1]]
  print(aucTr)  
  ## [1] 0.5081991  
     # Test AUC
  aucTe <- ROCR::performance(rocr.pred.te.1, measure = "auc")
  aucTe <- aucTe@y.values[[1]]
  print(aucTe)  
  ## [1] 0.5377857  
 
 
 
 C_AGE Binary logistic regression for HEALTH 
  # fit binary logit model and store results 'm'
m <- glm(Any_Health_Problem ~ C_Age, data = ml_train,family = binomial)
# view a summary of the model
summary(m)  
  ## 
## Call:
## glm(formula = Any_Health_Problem ~ C_Age, family = binomial, 
##     data = ml_train)
## 
## Coefficients:
##             Estimate Std. Error z value Pr(>|z|)    
## (Intercept) -0.49024    0.05089  -9.633   <2e-16 ***
## C_Age.L     -0.14536    0.11749  -1.237    0.216    
## C_Age.Q      0.02686    0.11565   0.232    0.816    
## C_Age.C      0.10542    0.11145   0.946    0.344    
## C_Age^4      0.07337    0.11046   0.664    0.507    
## ---
## Signif. codes:  0 '***' 0.001 '**' 0.01 '*' 0.05 '.' 0.1 ' ' 1
## 
## (Dispersion parameter for binomial family taken to be 1)
## 
##     Null deviance: 2200.0  on 1657  degrees of freedom
## Residual deviance: 2196.9  on 1653  degrees of freedom
## AIC: 2206.9
## 
## Number of Fisher Scoring iterations: 4  
  # test model fit
with(m, null.deviance - deviance)  
  ## [1] 3.096409  
  with(m, df.null - df.residual)  
  ## [1] 4  
  with(m, pchisq(null.deviance - deviance, df.null - df.residual, lower.tail = FALSE))  
  ## [1] 0.5418231  
  BIC(m)  
  ## [1] 2233.999  
  # Hosmer-Lemeshow Goodness-of-Fit Test
hltest(m, G=4)  
  ## 
##    The Hosmer-Lemeshow goodness-of-fit test
## 
##  Group Size Observed Expected
##      1  372      128      128
##      2  323      120      120
##      3  342      133      133
##      4  348      138      138
##      5  273      109      109
## 
##          Statistic =  0 
## degrees of freedom =  3 
##            p-value =  1  
  ## CIs using profiled log-likelihood
confint(m, level=0.99)  
  ## Waiting for profiling to be done...  
  ##                  0.5 %     99.5 %
## (Intercept) -0.6220686 -0.3597831
## C_Age.L     -0.4482493  0.1575287
## C_Age.Q     -0.2718387  0.3243072
## C_Age.C     -0.1815083  0.3930126
## C_Age^4     -0.2117869  0.3577003  
  ## CIs using standard errors
confint.default(m, level=0.99)  
  ##                  0.5 %     99.5 %
## (Intercept) -0.6213351 -0.3591487
## C_Age.L     -0.4480023  0.1572779
## C_Age.Q     -0.2710433  0.3247610
## C_Age.C     -0.1816509  0.3924942
## C_Age^4     -0.2111708  0.3579028  
  # Wald test
wald.test(b = coef(m), Sigma = vcov(m), Terms = 2)  
  ## Wald test:
## ----------
## 
## Chi-squared test:
## X2 = 1.5, df = 1, P(> X2) = 0.22  
  ## odds ratios and 95% CI
exp(cbind(OR = coef(m), confint(m, level=0.99)))  
  ## Waiting for profiling to be done...  
  ##                    OR     0.5 %    99.5 %
## (Intercept) 0.6124782 0.5368328 0.6978277
## C_Age.L     0.8647091 0.6387454 1.1706144
## C_Age.Q     1.0272228 0.7619771 1.3830721
## C_Age.C     1.1111790 0.8340113 1.4814370
## C_Age^4     1.0761243 0.8091371 1.4300370  
 
 Calculate Nagelkerke R^2 
  NagelkerkeR2(m)  
  ## $N
## [1] 1658
## 
## $R2
## [1] 0.002539538  
 
 
 check assumptions of model 
 
 Cook’s distance 
  plot(m, which = 4, id.n = 3)  
   
 
 
 Extract model results and display data for top 3 values using Cook’s
distance 
  model.data <- augment(m) %>% 
  mutate(index = 1:n()) 
model.data %>% top_n(3, .cooksd)  
 
 
 
 
 
 plot standardised residuals 
  ggplot(model.data, aes(index, .std.resid)) + 
  geom_point(aes(color = Any_Health_Problem), alpha = .5) +
  theme_bw()  
   
 
 
 Filter potential influential data points with abs(.std.res) >
3: 
  model.data %>% 
  filter(abs(.std.resid) > 3)  
 
 
 
 
 
 
 Create ROCR for training and test data 
  ## training data
pred.mtt = predict(m, type = "response") #repeat risk predictions from model m
rocr.pred.mtt = ROCR::prediction(pred.mtt, labels = ml_train$Any_Health_Problem) #ROCR prediction object
roc.perf.mtt = ROCR::performance(rocr.pred.mtt, measure = "tpr", x.measure = "fpr") # #ROCR performance object
plot(roc.perf.mtt, col = "blue")


pred.te.1 = predict(m, newdata = ml_test, type = "response") #.te = "test"
rocr.pred.te.1 = ROCR::prediction(pred.te.1, labels = ml_test$Any_Health_Problem)
roc.perf.te.1 = ROCR::performance(rocr.pred.te.1, measure = "tpr", x.measure = "fpr")
plot(roc.perf.te.1, col = "red", add = T)

abline(a = 0, b = 1, lty = 2) #diagonal for random assignment
legend("bottomright", legend = c("train","test"),
col = c("blue","red"), lty = c(2,1), lwd =1.5)  
   
 
 
 Report AUC from ROC for training and test data 
    # Train AUC
aucTr <- ROCR::performance(rocr.pred.mtt, measure = "auc")
  aucTr <- aucTr@y.values[[1]]
  print(aucTr)  
  ## [1] 0.5237393  
     # Test AUC
  aucTe <- ROCR::performance(rocr.pred.te.1, measure = "auc")
  aucTe <- aucTe@y.values[[1]]
  print(aucTe)  
  ## [1] 0.555595  
 
 
 
 C_AGE Binary logistic regression for HEALTH 
  # fit binary logit model and store results 'm'
m <- glm(Any_Health_Problem ~ C_Age2, data = ml_train,family = binomial)
# view a summary of the model
summary(m)  
  ## 
## Call:
## glm(formula = Any_Health_Problem ~ C_Age2, family = binomial, 
##     data = ml_train)
## 
## Coefficients:
##             Estimate Std. Error z value Pr(>|z|)    
## (Intercept) -0.40852    0.12358  -3.306 0.000947 ***
## C_Age230–39 -0.01134    0.16517  -0.069 0.945285    
## C_Age240–49 -0.04347    0.16606  -0.262 0.793510    
## C_Age250–59 -0.23662    0.16487  -1.435 0.151238    
## C_Age260<   -0.11720    0.16891  -0.694 0.487791    
## ---
## Signif. codes:  0 '***' 0.001 '**' 0.01 '*' 0.05 '.' 0.1 ' ' 1
## 
## (Dispersion parameter for binomial family taken to be 1)
## 
##     Null deviance: 2200.0  on 1657  degrees of freedom
## Residual deviance: 2196.9  on 1653  degrees of freedom
## AIC: 2206.9
## 
## Number of Fisher Scoring iterations: 4  
  # test model fit
with(m, null.deviance - deviance)  
  ## [1] 3.096409  
  with(m, df.null - df.residual)  
  ## [1] 4  
  with(m, pchisq(null.deviance - deviance, df.null - df.residual, lower.tail = FALSE))  
  ## [1] 0.5418231  
  BIC(m)  
  ## [1] 2233.999  
  # Hosmer-Lemeshow Goodness-of-Fit Test
hltest(m, G=4)  
  ## 
##    The Hosmer-Lemeshow goodness-of-fit test
## 
##  Group Size Observed Expected
##      1  372      128      128
##      2  323      120      120
##      3  342      133      133
##      4  348      138      138
##      5  273      109      109
## 
##          Statistic =  0 
## degrees of freedom =  3 
##            p-value =  1  
  ## CIs using profiled log-likelihood
confint(m, level=0.99)  
  ## Waiting for profiling to be done...  
  ##                  0.5 %      99.5 %
## (Intercept) -0.7309370 -0.09293041
## C_Age230–39 -0.4365457  0.41527907
## C_Age240–49 -0.4711412  0.38529063
## C_Age250–59 -0.6616767  0.18867384
## C_Age260<   -0.5527150  0.31847112  
  ## CIs using standard errors
confint.default(m, level=0.99)  
  ##                  0.5 %      99.5 %
## (Intercept) -0.7268380 -0.09019906
## C_Age230–39 -0.4367774  0.41410683
## C_Age240–49 -0.4712044  0.38427125
## C_Age250–59 -0.6613012  0.18806235
## C_Age260<   -0.5522845  0.31789317  
  # Wald test
wald.test(b = coef(m), Sigma = vcov(m), Terms = 2)  
  ## Wald test:
## ----------
## 
## Chi-squared test:
## X2 = 0.0047, df = 1, P(> X2) = 0.95  
  ## odds ratios and 95% CI
exp(cbind(OR = coef(m), confint(m, level=0.99)))  
  ## Waiting for profiling to be done...  
  ##                    OR     0.5 %    99.5 %
## (Intercept) 0.6646341 0.4814577 0.9112569
## C_Age230–39 0.9887287 0.6462650 1.5147934
## C_Age240–49 0.9574646 0.6242894 1.4700415
## C_Age250–59 0.7892916 0.5159855 1.2076470
## C_Age260<   0.8894111 0.5753855 1.3750239  
 
 Calculate Nagelkerke R^2 
  NagelkerkeR2(m)  
  ## $N
## [1] 1658
## 
## $R2
## [1] 0.002539538  
 
 
 check assumptions of model 
 
 Cook’s distance 
  plot(m, which = 4, id.n = 3)  
   
 
 
 Extract model results and display data for top 3 values using Cook’s
distance 
  model.data <- augment(m) %>% 
  mutate(index = 1:n()) 
model.data %>% top_n(3, .cooksd)  
 
 
 
 
 
 plot standardised residuals 
  ggplot(model.data, aes(index, .std.resid)) + 
  geom_point(aes(color = Any_Health_Problem), alpha = .5) +
  theme_bw()  
   
 
 
 Filter potential influential data points with abs(.std.res) >
3: 
  model.data %>% 
  filter(abs(.std.resid) > 3)  
 
 
 
 
 
 
 Create ROCR for training and test data 
  ## training data
pred.mtt = predict(m, type = "response") #repeat risk predictions from model m
rocr.pred.mtt = ROCR::prediction(pred.mtt, labels = ml_train$Any_Health_Problem) #ROCR prediction object
roc.perf.mtt = ROCR::performance(rocr.pred.mtt, measure = "tpr", x.measure = "fpr") # #ROCR performance object
plot(roc.perf.mtt, col = "blue")


pred.te.1 = predict(m, newdata = ml_test, type = "response") #.te = "test"
rocr.pred.te.1 = ROCR::prediction(pred.te.1, labels = ml_test$Any_Health_Problem)
roc.perf.te.1 = ROCR::performance(rocr.pred.te.1, measure = "tpr", x.measure = "fpr")
plot(roc.perf.te.1, col = "red", add = T)

abline(a = 0, b = 1, lty = 2) #diagonal for random assignment
legend("bottomright", legend = c("train","test"),
col = c("blue","red"), lty = c(2,1), lwd =1.5)  
   
 
 
 Report AUC from ROC for training and test data 
    # Train AUC
aucTr <- ROCR::performance(rocr.pred.mtt, measure = "auc")
  aucTr <- aucTr@y.values[[1]]
  print(aucTr)  
  ## [1] 0.5237393  
     # Test AUC
  aucTe <- ROCR::performance(rocr.pred.te.1, measure = "auc")
  aucTe <- aucTe@y.values[[1]]
  print(aucTe)  
  ## [1] 0.555595  
 
 
 
 C_GENDER Binary logistic regression for HEALTH 
  # fit binary logit model and store results 'm'
m <- glm(Any_Health_Problem ~ C_Gender, data = ml_train,family = binomial)
# view a summary of the model
summary(m)  
  ## 
## Call:
## glm(formula = Any_Health_Problem ~ C_Gender, family = binomial, 
##     data = ml_train)
## 
## Coefficients:
##              Estimate Std. Error z value Pr(>|z|)    
## (Intercept)  -0.46958    0.05231  -8.977   <2e-16 ***
## C_GenderMale -0.38608    0.21130  -1.827   0.0677 .  
## ---
## Signif. codes:  0 '***' 0.001 '**' 0.01 '*' 0.05 '.' 0.1 ' ' 1
## 
## (Dispersion parameter for binomial family taken to be 1)
## 
##     Null deviance: 2200.0  on 1657  degrees of freedom
## Residual deviance: 2196.5  on 1656  degrees of freedom
## AIC: 2200.5
## 
## Number of Fisher Scoring iterations: 4  
  # test model fit
with(m, null.deviance - deviance)  
  ## [1] 3.479778  
  with(m, df.null - df.residual)  
  ## [1] 1  
  with(m, pchisq(null.deviance - deviance, df.null - df.residual, lower.tail = FALSE))  
  ## [1] 0.06212308  
  BIC(m)  
  ## [1] 2211.376  
  ## CIs using profiled log-likelihood
confint(m, level=0.99)  
  ## Waiting for profiling to be done...  
  ##                   0.5 %     99.5 %
## (Intercept)  -0.6050702 -0.3354928
## C_GenderMale -0.9510360  0.1435398  
  ## CIs using standard errors
confint.default(m, level=0.99)  
  ##                   0.5 %     99.5 %
## (Intercept)  -0.6043193 -0.3348460
## C_GenderMale -0.9303572  0.1581903  
  # Wald test
wald.test(b = coef(m), Sigma = vcov(m), Terms = 2)  
  ## Wald test:
## ----------
## 
## Chi-squared test:
## X2 = 3.3, df = 1, P(> X2) = 0.068  
  ## odds ratios and 95% CI
exp(cbind(OR = coef(m), confint(m, level=0.99)))  
  ## Waiting for profiling to be done...  
  ##                     OR     0.5 %    99.5 %
## (Intercept)  0.6252632 0.5460361 0.7149856
## C_GenderMale 0.6797138 0.3863406 1.1543527  
 
 Calculate Nagelkerke R^2 
  NagelkerkeR2(m)  
  ## $N
## [1] 1658
## 
## $R2
## [1] 0.00285363  
 
 
 check assumptions of model 
 
 Cook’s distance 
  plot(m, which = 4, id.n = 3)  
   
 
 
 Extract model results and display data for top 3 values using Cook’s
distance 
  model.data <- augment(m) %>% 
  mutate(index = 1:n()) 
model.data %>% top_n(3, .cooksd)  
 
 
 
 
 
 plot standardised residuals 
  ggplot(model.data, aes(index, .std.resid)) + 
  geom_point(aes(color = Any_Health_Problem), alpha = .5) +
  theme_bw()  
   
 
 
 Filter potential influential data points with abs(.std.res) >
3: 
  model.data %>% 
  filter(abs(.std.resid) > 3)  
 
 
 
 
 
 
 Create ROCR for training and test data 
  ## training data
pred.mtt = predict(m, type = "response") #repeat risk predictions from model m
rocr.pred.mtt = ROCR::prediction(pred.mtt, labels = ml_train$Any_Health_Problem) #ROCR prediction object
roc.perf.mtt = ROCR::performance(rocr.pred.mtt, measure = "tpr", x.measure = "fpr") # #ROCR performance object
plot(roc.perf.mtt, col = "blue")


pred.te.1 = predict(m, newdata = ml_test, type = "response") #.te = "test"
rocr.pred.te.1 = ROCR::prediction(pred.te.1, labels = ml_test$Any_Health_Problem)
roc.perf.te.1 = ROCR::performance(rocr.pred.te.1, measure = "tpr", x.measure = "fpr")
plot(roc.perf.te.1, col = "red", add = T)

abline(a = 0, b = 1, lty = 2) #diagonal for random assignment
legend("bottomright", legend = c("train","test"),
col = c("blue","red"), lty = c(2,1), lwd =1.5)  
   
 
 
 Report AUC from ROC for training and test data 
    # Train AUC
aucTr <- ROCR::performance(rocr.pred.mtt, measure = "auc")
  aucTr <- aucTr@y.values[[1]]
  print(aucTr)  
  ## [1] 0.5117649  
     # Test AUC
  aucTe <- ROCR::performance(rocr.pred.te.1, measure = "auc")
  aucTe <- aucTe@y.values[[1]]
  print(aucTe)  
  ## [1] 0.510779  
 
 
 
 
 NOW CHECK ASSOCIATIONS WITH DOG CHARACTERISTICS - simple BINARY
LOGISTIC regression 
 
 DOG DIET ordinal regression for HEALTH 
  # fit binary logit model and store results 'm'
m <- glm(Any_Health_Problem ~ D_Diet, data = ml_train,family = binomial)
# view a summary of the model
summary(m)  
  ## 
## Call:
## glm(formula = Any_Health_Problem ~ D_Diet, family = binomial, 
##     data = ml_train)
## 
## Coefficients:
##                                                         Estimate Std. Error
## (Intercept)                                             -0.23361    0.06867
## D_DietMeat-based – raw                                  -0.60947    0.11458
## D_DietVegan (consuming no animal products)              -0.52549    0.16419
## D_DietVegetarian (including eggs or milk, but not meat)  0.07946    0.39935
##                                                         z value Pr(>|z|)    
## (Intercept)                                              -3.402 0.000668 ***
## D_DietMeat-based – raw                                   -5.319 1.04e-07 ***
## D_DietVegan (consuming no animal products)               -3.200 0.001372 ** 
## D_DietVegetarian (including eggs or milk, but not meat)   0.199 0.842274    
## ---
## Signif. codes:  0 '***' 0.001 '**' 0.01 '*' 0.05 '.' 0.1 ' ' 1
## 
## (Dispersion parameter for binomial family taken to be 1)
## 
##     Null deviance: 2200.0  on 1657  degrees of freedom
## Residual deviance: 2166.7  on 1654  degrees of freedom
## AIC: 2174.7
## 
## Number of Fisher Scoring iterations: 4  
  # test model fit
with(m, null.deviance - deviance)  
  ## [1] 33.29736  
  with(m, df.null - df.residual)  
  ## [1] 3  
  with(m, pchisq(null.deviance - deviance, df.null - df.residual, lower.tail = FALSE))  
  ## [1] 2.787508e-07  
  BIC(m)  
  ## [1] 2196.385  
  ## CIs using profiled log-likelihood
confint(m, level=0.99)  
  ## Waiting for profiling to be done...  
  ##                                                              0.5 %      99.5 %
## (Intercept)                                             -0.4112086 -0.05723623
## D_DietMeat-based – raw                                  -0.9069829 -0.31629177
## D_DietVegan (consuming no animal products)              -0.9567135 -0.10886089
## D_DietVegetarian (including eggs or milk, but not meat) -0.9832784  1.11600684  
  ## CIs using standard errors
confint.default(m, level=0.99)  
  ##                                                              0.5 %      99.5 %
## (Intercept)                                             -0.4104847 -0.05674497
## D_DietMeat-based – raw                                  -0.9046147 -0.31433029
## D_DietVegan (consuming no animal products)              -0.9484177 -0.10256287
## D_DietVegetarian (including eggs or milk, but not meat) -0.9491816  1.10810998  
  # Wald test
wald.test(b = coef(m), Sigma = vcov(m), Terms = 2)  
  ## Wald test:
## ----------
## 
## Chi-squared test:
## X2 = 28.3, df = 1, P(> X2) = 1e-07  
  ## odds ratios and 95% CI
exp(cbind(OR = coef(m), confint(m, level=0.99)))  
  ## Waiting for profiling to be done...  
  ##                                                                OR     0.5 %
## (Intercept)                                             0.7916667 0.6628487
## D_DietMeat-based – raw                                  0.5436376 0.4037405
## D_DietVegan (consuming no animal products)              0.5912654 0.3841533
## D_DietVegetarian (including eggs or milk, but not meat) 1.0827068 0.3740827
##                                                            99.5 %
## (Intercept)                                             0.9443710
## D_DietMeat-based – raw                                  0.7288468
## D_DietVegan (consuming no animal products)              0.8968552
## D_DietVegetarian (including eggs or milk, but not meat) 3.0526402  
 
 Calculate Nagelkerke R^2 
  NagelkerkeR2(m)  
  ## $N
## [1] 1658
## 
## $R2
## [1] 0.02706188  
 
 
 check assumptions of model 
 
 Cook’s distance 
  plot(m, which = 4, id.n = 3)  
   
 
 
 Extract model results and display data for top 3 values using Cook’s
distance 
  model.data <- augment(m) %>% 
  mutate(index = 1:n()) 
model.data %>% top_n(3, .cooksd)  
 
 
 
 
 
 plot standardised residuals 
  ggplot(model.data, aes(index, .std.resid)) + 
  geom_point(aes(color = Any_Health_Problem), alpha = .5) +
  theme_bw()  
   
 
 
 Filter potential influential data points with abs(.std.res) >
3: 
  model.data %>% 
  filter(abs(.std.resid) > 3)  
 
 
 
 
 
 
 Create ROCR for training and test data 
  ## training data
pred.mtt = predict(m, type = "response") #repeat risk predictions from model m
rocr.pred.mtt = ROCR::prediction(pred.mtt, labels = ml_train$Any_Health_Problem) #ROCR prediction object
roc.perf.mtt = ROCR::performance(rocr.pred.mtt, measure = "tpr", x.measure = "fpr") # #ROCR performance object
plot(roc.perf.mtt, col = "blue")


pred.te.1 = predict(m, newdata = ml_test, type = "response") #.te = "test"
rocr.pred.te.1 = ROCR::prediction(pred.te.1, labels = ml_test$Any_Health_Problem)
roc.perf.te.1 = ROCR::performance(rocr.pred.te.1, measure = "tpr", x.measure = "fpr")
plot(roc.perf.te.1, col = "red", add = T)

abline(a = 0, b = 1, lty = 2) #diagonal for random assignment
legend("bottomright", legend = c("train","test"),
col = c("blue","red"), lty = c(2,1), lwd =1.5)  
   
 
 
 Report AUC from ROC for training and test data 
    # Train AUC
aucTr <- ROCR::performance(rocr.pred.mtt, measure = "auc")
  aucTr <- aucTr@y.values[[1]]
  print(aucTr)  
  ## [1] 0.5742595  
     # Test AUC
  aucTe <- ROCR::performance(rocr.pred.te.1, measure = "auc")
  aucTe <- aucTe@y.values[[1]]
  print(aucTe)  
  ## [1] 0.5360089  
 
 
 
 DOG DIET VEGAN ordinal regression for HEALTH 
  # fit binary logit model and store results 'm'
m <- glm(Any_Health_Problem ~ D_Diet_Vegan, data = ml_train,family = binomial)
# view a summary of the model
summary(m)  
  ## 
## Call:
## glm(formula = Any_Health_Problem ~ D_Diet_Vegan, family = binomial, 
##     data = ml_train)
## 
## Coefficients:
##                 Estimate Std. Error z value Pr(>|z|)    
## (Intercept)     -0.45860    0.05389  -8.510   <2e-16 ***
## D_Diet_VeganYes -0.30051    0.15858  -1.895   0.0581 .  
## ---
## Signif. codes:  0 '***' 0.001 '**' 0.01 '*' 0.05 '.' 0.1 ' ' 1
## 
## (Dispersion parameter for binomial family taken to be 1)
## 
##     Null deviance: 2200.0  on 1657  degrees of freedom
## Residual deviance: 2196.3  on 1656  degrees of freedom
## AIC: 2200.3
## 
## Number of Fisher Scoring iterations: 4  
  # test model fit
with(m, null.deviance - deviance)  
  ## [1] 3.684778  
  with(m, df.null - df.residual)  
  ## [1] 1  
  with(m, pchisq(null.deviance - deviance, df.null - df.residual, lower.tail = FALSE))  
  ## [1] 0.0549113  
  BIC(m)  
  ## [1] 2211.171  
  ## CIs using profiled log-likelihood
confint(m, level=0.99)  
  ## Waiting for profiling to be done...  
  ##                      0.5 %    99.5 %
## (Intercept)     -0.5981909 -0.320450
## D_Diet_VeganYes -0.7179134  0.101198  
  ## CIs using standard errors
confint.default(m, level=0.99)  
  ##                      0.5 %     99.5 %
## (Intercept)     -0.5974089 -0.3197819
## D_Diet_VeganYes -0.7089872  0.1079677  
  # Wald test
wald.test(b = coef(m), Sigma = vcov(m), Terms = 2)  
  ## Wald test:
## ----------
## 
## Chi-squared test:
## X2 = 3.6, df = 1, P(> X2) = 0.058  
  ## odds ratios and 95% CI
exp(cbind(OR = coef(m), confint(m, level=0.99)))  
  ## Waiting for profiling to be done...  
  ##                        OR     0.5 %    99.5 %
## (Intercept)     0.6321710 0.5498054 0.7258223
## D_Diet_VeganYes 0.7404407 0.4877690 1.1064957  
 
 Calculate Nagelkerke R^2 
  NagelkerkeR2(m)  
  ## $N
## [1] 1658
## 
## $R2
## [1] 0.003021556  
 
 
 check assumptions of model 
 
 Cook’s distance 
  plot(m, which = 4, id.n = 3)  
   
 
 
 Extract model results and display data for top 3 values using Cook’s
distance 
  model.data <- augment(m) %>% 
  mutate(index = 1:n()) 
model.data %>% top_n(3, .cooksd)  
 
 
 
 
 
 plot standardised residuals 
  ggplot(model.data, aes(index, .std.resid)) + 
  geom_point(aes(color = Any_Health_Problem), alpha = .5) +
  theme_bw()  
   
 
 
 Filter potential influential data points with abs(.std.res) >
3: 
  model.data %>% 
  filter(abs(.std.resid) > 3)  
 
 
 
 
 
 
 Create ROCR for training and test data 
  ## training data
pred.mtt = predict(m, type = "response") #repeat risk predictions from model m
rocr.pred.mtt = ROCR::prediction(pred.mtt, labels = ml_train$Any_Health_Problem) #ROCR prediction object
roc.perf.mtt = ROCR::performance(rocr.pred.mtt, measure = "tpr", x.measure = "fpr") # #ROCR performance object
plot(roc.perf.mtt, col = "blue")


pred.te.1 = predict(m, newdata = ml_test, type = "response") #.te = "test"
rocr.pred.te.1 = ROCR::prediction(pred.te.1, labels = ml_test$Any_Health_Problem)
roc.perf.te.1 = ROCR::performance(rocr.pred.te.1, measure = "tpr", x.measure = "fpr")
plot(roc.perf.te.1, col = "red", add = T)

abline(a = 0, b = 1, lty = 2) #diagonal for random assignment
legend("bottomright", legend = c("train","test"),
col = c("blue","red"), lty = c(2,1), lwd =1.5)  
   
 
 
 Report AUC from ROC for training and test data 
    # Train AUC
aucTr <- ROCR::performance(rocr.pred.mtt, measure = "auc")
  aucTr <- aucTr@y.values[[1]]
  print(aucTr)  
  ## [1] 0.5158988  
     # Test AUC
  aucTe <- ROCR::performance(rocr.pred.te.1, measure = "auc")
  aucTe <- aucTe@y.values[[1]]
  print(aucTe)  
  ## [1] 0.5350613  
 
 
 
 CLIENT DIET VEGAN + DOG DIET VEGAN ordinal regression for
HEALTH 
  # fit binary logit model and store results 'm'
m <- glm(Any_Health_Problem ~ C_Diet_Vegan + D_Diet_Vegan, data = ml_train,family = binomial)
# view a summary of the model
summary(m)  
  ## 
## Call:
## glm(formula = Any_Health_Problem ~ C_Diet_Vegan + D_Diet_Vegan, 
##     family = binomial, data = ml_train)
## 
## Coefficients:
##                 Estimate Std. Error z value Pr(>|z|)    
## (Intercept)     -0.46588    0.05695  -8.181 2.81e-16 ***
## C_Diet_VeganYes  0.06527    0.16376   0.399   0.6902    
## D_Diet_VeganYes -0.35381    0.20746  -1.705   0.0881 .  
## ---
## Signif. codes:  0 '***' 0.001 '**' 0.01 '*' 0.05 '.' 0.1 ' ' 1
## 
## (Dispersion parameter for binomial family taken to be 1)
## 
##     Null deviance: 2200.0  on 1657  degrees of freedom
## Residual deviance: 2196.2  on 1655  degrees of freedom
## AIC: 2202.2
## 
## Number of Fisher Scoring iterations: 4  
  # test model fit
with(m, null.deviance - deviance)  
  ## [1] 3.843213  
  with(m, df.null - df.residual)  
  ## [1] 2  
  with(m, pchisq(null.deviance - deviance, df.null - df.residual, lower.tail = FALSE))  
  ## [1] 0.1463716  
  BIC(m)  
  ## [1] 2218.426  
  ## CIs using profiled log-likelihood
confint(m, level=0.99)  
  ## Waiting for profiling to be done...  
  ##                      0.5 %     99.5 %
## (Intercept)     -0.6134590 -0.3199550
## C_Diet_VeganYes -0.3610133  0.4847235
## D_Diet_VeganYes -0.8930176  0.1780489  
  ## CIs using standard errors
confint.default(m, level=0.99)  
  ##                      0.5 %     99.5 %
## (Intercept)     -0.6125680 -0.3191980
## C_Diet_VeganYes -0.3565409  0.4870733
## D_Diet_VeganYes -0.8882025  0.1805795  
  # Wald test
wald.test(b = coef(m), Sigma = vcov(m), Terms = 2)  
  ## Wald test:
## ----------
## 
## Chi-squared test:
## X2 = 0.16, df = 1, P(> X2) = 0.69  
  ## odds ratios and 95% CI
exp(cbind(OR = coef(m), confint(m, level=0.99)))  
  ## Waiting for profiling to be done...  
  ##                        OR     0.5 %    99.5 %
## (Intercept)     0.6275807 0.5414747 0.7261817
## C_Diet_VeganYes 1.0674431 0.6969697 1.6237261
## D_Diet_VeganYes 0.7020073 0.4094184 1.1948837  
 
 Calculate Nagelkerke R^2 
  NagelkerkeR2(m)  
  ## $N
## [1] 1658
## 
## $R2
## [1] 0.003151324  
 
 
 check assumptions of model 
 
 Cook’s distance 
  plot(m, which = 4, id.n = 3)  
   
 
 
 Extract model results and display data for top 3 values using Cook’s
distance 
  model.data <- augment(m) %>% 
  mutate(index = 1:n()) 
model.data %>% top_n(3, .cooksd)  
 
 
 
 
 
 plot standardised residuals 
  ggplot(model.data, aes(index, .std.resid)) + 
  geom_point(aes(color = Any_Health_Problem), alpha = .5) +
  theme_bw()  
   
 
 
 Filter potential influential data points with abs(.std.res) >
3: 
  model.data %>% 
  filter(abs(.std.resid) > 3)  
 
 
 
 
 
 check for multicollinearity 
  car::vif(m)  
  ## C_Diet_Vegan D_Diet_Vegan 
##     1.711408     1.711408  
 
 
 
 Create ROCR for training and test data 
  ## training data
pred.mtt = predict(m, type = "response") #repeat risk predictions from model m
rocr.pred.mtt = ROCR::prediction(pred.mtt, labels = ml_train$Any_Health_Problem) #ROCR prediction object
roc.perf.mtt = ROCR::performance(rocr.pred.mtt, measure = "tpr", x.measure = "fpr") # #ROCR performance object
plot(roc.perf.mtt, col = "blue")


pred.te.1 = predict(m, newdata = ml_test, type = "response") #.te = "test"
rocr.pred.te.1 = ROCR::prediction(pred.te.1, labels = ml_test$Any_Health_Problem)
roc.perf.te.1 = ROCR::performance(rocr.pred.te.1, measure = "tpr", x.measure = "fpr")
plot(roc.perf.te.1, col = "red", add = T)

abline(a = 0, b = 1, lty = 2) #diagonal for random assignment
legend("bottomright", legend = c("train","test"),
col = c("blue","red"), lty = c(2,1), lwd =1.5)  
   
 
 
 Report AUC from ROC for training and test data 
    # Train AUC
aucTr <- ROCR::performance(rocr.pred.mtt, measure = "auc")
  aucTr <- aucTr@y.values[[1]]
  print(aucTr)  
  ## [1] 0.5207501  
     # Test AUC
  aucTe <- ROCR::performance(rocr.pred.te.1, measure = "auc")
  aucTe <- aucTe@y.values[[1]]
  print(aucTe)  
  ## [1] 0.5565635  
 
 
 
 CLIENT DIET VEGAN * DOG DIET VEGAN ordinal regression for
HEALTH 
  # fit binary logit model and store results 'm2'
m <- glm(Any_Health_Problem ~ C_Diet_Vegan*D_Diet_Vegan, data = ml_train,family = binomial)
# view a summary of the model
summary(m)  
  ## 
## Call:
## glm(formula = Any_Health_Problem ~ C_Diet_Vegan * D_Diet_Vegan, 
##     family = binomial, data = ml_train)
## 
## Coefficients:
##                                 Estimate Std. Error z value Pr(>|z|)    
## (Intercept)                     -0.47378    0.05725  -8.275   <2e-16 ***
## C_Diet_VeganYes                  0.13518    0.16983   0.796    0.426    
## D_Diet_VeganYes                  0.34025    0.52071   0.653    0.513    
## C_Diet_VeganYes:D_Diet_VeganYes -0.81446    0.56672  -1.437    0.151    
## ---
## Signif. codes:  0 '***' 0.001 '**' 0.01 '*' 0.05 '.' 0.1 ' ' 1
## 
## (Dispersion parameter for binomial family taken to be 1)
## 
##     Null deviance: 2200.0  on 1657  degrees of freedom
## Residual deviance: 2194.2  on 1654  degrees of freedom
## AIC: 2202.2
## 
## Number of Fisher Scoring iterations: 4  
  # test model fit
with(m, null.deviance - deviance)  
  ## [1] 5.851622  
  with(m, df.null - df.residual)  
  ## [1] 3  
  with(m, pchisq(null.deviance - deviance, df.null - df.residual, lower.tail = FALSE))  
  ## [1] 0.1190566  
  BIC(m)  
  ## [1] 2223.83  
  # Hosmer-Lemeshow Goodness-of-Fit Test
glmtoolbox::hltest(m)  
  ## 
##    The Hosmer-Lemeshow goodness-of-fit test
## 
##  Group Size Observed Expected
##      1  192       59       59
##      2 1290      495      495
##      3  161       67       67
##      4   15        7        7
## 
##          Statistic =  0 
## degrees of freedom =  2 
##            p-value =  1  
  ## CIs using profiled log-likelihood
confint(m, level=0.99)  
  ## Waiting for profiling to be done...  
  ##                                      0.5 %     99.5 %
## (Intercept)                     -0.6221747 -0.3270831
## C_Diet_VeganYes                 -0.3076545  0.5697445
## D_Diet_VeganYes                 -1.0706306  1.7103539
## C_Diet_VeganYes:D_Diet_VeganYes -2.2961081  0.7014966  
  ## CIs using standard errors
confint.default(m, level=0.99)  
  ##                                      0.5 %     99.5 %
## (Intercept)                     -0.6212619 -0.3263069
## C_Diet_VeganYes                 -0.3022672  0.5726315
## D_Diet_VeganYes                 -1.0009980  1.6815039
## C_Diet_VeganYes:D_Diet_VeganYes -2.2742257  0.6453007  
  # Wald test
wald.test(b = coef(m), Sigma = vcov(m), Terms = 2)  
  ## Wald test:
## ----------
## 
## Chi-squared test:
## X2 = 0.63, df = 1, P(> X2) = 0.43  
  ## odds ratios and 95% CI
exp(cbind(OR = coef(m), confint(m, level=0.99)))  
  ## Waiting for profiling to be done...  
  ##                                        OR     0.5 %    99.5 %
## (Intercept)                     0.6226415 0.5367758 0.7210238
## C_Diet_VeganYes                 1.1447453 0.7351693 1.7678153
## D_Diet_VeganYes                 1.4053030 0.3427923 5.5309185
## C_Diet_VeganYes:D_Diet_VeganYes 0.4428773 0.1006498 2.0167688  
 
 Calculate Nagelkerke R^2 
  NagelkerkeR2(m)  
  ## $N
## [1] 1658
## 
## $R2
## [1] 0.004795257  
 
 
 check assumptions of model 
 
 Cook’s distance 
  plot(m, which = 4, id.n = 3)  
   
 
 
 Extract model results and display data for top 3 values using Cook’s
distance 
  model.data <- augment(m) %>% 
  mutate(index = 1:n()) 
model.data %>% top_n(3, .cooksd)  
 
 
 
 
 
 plot standardised residuals 
  ggplot(model.data, aes(index, .std.resid)) + 
  geom_point(aes(color = Any_Health_Problem), alpha = .5) +
  theme_bw()  
   
 
 
 Filter potential influential data points with abs(.std.res) >
3: 
  model.data %>% 
  filter(abs(.std.resid) > 3)  
 
 
 
 
 
 check for multicollinearity 
  car::vif(m)  
  ## there are higher-order terms (interactions) in this model
## consider setting type = 'predictor'; see ?vif  
  ##              C_Diet_Vegan              D_Diet_Vegan C_Diet_Vegan:D_Diet_Vegan 
##                  1.832346                 10.706047                 11.746177  
 
 
 
 Create ROCR for training and test data 
  ## training data
pred.mtt = predict(m, type = "response") #repeat risk predictions from model m
rocr.pred.mtt = ROCR::prediction(pred.mtt, labels = ml_train$Any_Health_Problem) #ROCR prediction object
roc.perf.mtt = ROCR::performance(rocr.pred.mtt, measure = "tpr", x.measure = "fpr") # #ROCR performance object
plot(roc.perf.mtt, col = "blue")


pred.te.1 = predict(m, newdata = ml_test, type = "response") #.te = "test"
rocr.pred.te.1 = ROCR::prediction(pred.te.1, labels = ml_test$Any_Health_Problem)
roc.perf.te.1 = ROCR::performance(rocr.pred.te.1, measure = "tpr", x.measure = "fpr")
plot(roc.perf.te.1, col = "red", add = T)

abline(a = 0, b = 1, lty = 2) #diagonal for random assignment
legend("bottomright", legend = c("train","test"),
col = c("blue","red"), lty = c(2,1), lwd =1.5)  
   
 
 
 Report AUC from ROC for training and test data 
    # Train AUC
aucTr <- ROCR::performance(rocr.pred.mtt, measure = "auc")
  aucTr <- aucTr@y.values[[1]]
  print(aucTr)  
  ## [1] 0.5241296  
     # Test AUC
  aucTe <- ROCR::performance(rocr.pred.te.1, measure = "auc")
  aucTe <- aucTe@y.values[[1]]
  print(aucTe)  
  ## [1] 0.5516862  
 
 
 
 DOG DIET RAW ordinal regression for HEALTH 
  # fit binary logit model and store results 'm'
m <- glm(Any_Health_Problem ~ D_Diet_Raw, data = ml_train,family = binomial)
# view a summary of the model
summary(m)  
  ## 
## Call:
## glm(formula = Any_Health_Problem ~ D_Diet_Raw, family = binomial, 
##     data = ml_train)
## 
## Coefficients:
##               Estimate Std. Error z value Pr(>|z|)    
## (Intercept)    -0.3268     0.0613   -5.33 9.82e-08 ***
## D_Diet_RawYes  -0.5163     0.1103   -4.68 2.87e-06 ***
## ---
## Signif. codes:  0 '***' 0.001 '**' 0.01 '*' 0.05 '.' 0.1 ' ' 1
## 
## (Dispersion parameter for binomial family taken to be 1)
## 
##     Null deviance: 2200.0  on 1657  degrees of freedom
## Residual deviance: 2177.6  on 1656  degrees of freedom
## AIC: 2181.6
## 
## Number of Fisher Scoring iterations: 4  
  # test model fit
with(m, null.deviance - deviance)  
  ## [1] 22.47696  
  with(m, df.null - df.residual)  
  ## [1] 1  
  with(m, pchisq(null.deviance - deviance, df.null - df.residual, lower.tail = FALSE))  
  ## [1] 2.126789e-06  
  BIC(m)  
  ## [1] 2192.378  
  ## CIs using profiled log-likelihood
confint(m, level=0.99)  
  ## Waiting for profiling to be done...  
  ##                    0.5 %     99.5 %
## (Intercept)   -0.4854225 -0.1694378
## D_Diet_RawYes -0.8030896 -0.2342984  
  ## CIs using standard errors
confint.default(m, level=0.99)  
  ##                    0.5 %     99.5 %
## (Intercept)   -0.4846651 -0.1688466
## D_Diet_RawYes -0.8005168 -0.2321462  
  # Wald test
wald.test(b = coef(m), Sigma = vcov(m), Terms = 2)  
  ## Wald test:
## ----------
## 
## Chi-squared test:
## X2 = 21.9, df = 1, P(> X2) = 2.9e-06  
  ## odds ratios and 95% CI
exp(cbind(OR = coef(m), confint(m, level=0.99)))  
  ## Waiting for profiling to be done...  
  ##                      OR     0.5 %    99.5 %
## (Intercept)   0.7212598 0.6154371 0.8441393
## D_Diet_RawYes 0.5967055 0.4479429 0.7911257  
 
 Calculate Nagelkerke R^2 
  NagelkerkeR2(m)  
  ## $N
## [1] 1658
## 
## $R2
## [1] 0.01832732  
 
 
 check assumptions of model 
 
 Cook’s distance 
  plot(m, which = 4, id.n = 3)  
   
 
 
 Extract model results and display data for top 3 values using Cook’s
distance 
  model.data <- augment(m) %>% 
  mutate(index = 1:n()) 
model.data %>% top_n(3, .cooksd)  
 
 
 
 
 
 plot standardised residuals 
  ggplot(model.data, aes(index, .std.resid)) + 
  geom_point(aes(color = Any_Health_Problem), alpha = .5) +
  theme_bw()  
   
 
 
 Filter potential influential data points with abs(.std.res) >
3: 
  model.data %>% 
  filter(abs(.std.resid) > 3)  
 
 
 
 
 
 
 Create ROCR for training and test data 
  ## training data
pred.mtt = predict(m, type = "response") #repeat risk predictions from model m
rocr.pred.mtt = ROCR::prediction(pred.mtt, labels = ml_train$Any_Health_Problem) #ROCR prediction object
roc.perf.mtt = ROCR::performance(rocr.pred.mtt, measure = "tpr", x.measure = "fpr") # #ROCR performance object
plot(roc.perf.mtt, col = "blue")


pred.te.1 = predict(m, newdata = ml_test, type = "response") #.te = "test"
rocr.pred.te.1 = ROCR::prediction(pred.te.1, labels = ml_test$Any_Health_Problem)
roc.perf.te.1 = ROCR::performance(rocr.pred.te.1, measure = "tpr", x.measure = "fpr")
plot(roc.perf.te.1, col = "red", add = T)

abline(a = 0, b = 1, lty = 2) #diagonal for random assignment
legend("bottomright", legend = c("train","test"),
col = c("blue","red"), lty = c(2,1), lwd =1.5)  
   
 
 
 Report AUC from ROC for training and test data 
    # Train AUC
aucTr <- ROCR::performance(rocr.pred.mtt, measure = "auc")
  aucTr <- aucTr@y.values[[1]]
  print(aucTr)  
  ## [1] 0.5563973  
     # Test AUC
  aucTe <- ROCR::performance(rocr.pred.te.1, measure = "auc")
  aucTe <- aucTe@y.values[[1]]
  print(aucTe)  
  ## [1] 0.5108417  
 
 
 
 DOG DIET + CLIENT DIET binary logistic regression for HEALTH 
  # fit binary logit model and store results 'm'
m <- glm(Any_Health_Problem ~ D_Diet + C_Diet , data = ml_train,family = binomial)
# view a summary of the model
summary(m)  
  ## 
## Call:
## glm(formula = Any_Health_Problem ~ D_Diet + C_Diet, family = binomial, 
##     data = ml_train)
## 
## Coefficients:
##                                                         Estimate Std. Error
## (Intercept)                                             -0.35580    0.09248
## D_DietMeat-based – raw                                  -0.60393    0.11552
## D_DietVegan (consuming no animal products)              -0.54981    0.21205
## D_DietVegetarian (including eggs or milk, but not meat)  0.08246    0.40569
## C_DietOmnivore reducing animal product consumption       0.21267    0.13498
## C_DietPescatarian (including fish but no other meats)    0.64532    0.22495
## C_DietVegan (consuming no animal products)               0.13853    0.17693
## C_DietVegetarian (consuming plants, eggs and milk)       0.08535    0.18019
##                                                         z value Pr(>|z|)    
## (Intercept)                                              -3.847 0.000119 ***
## D_DietMeat-based – raw                                   -5.228 1.72e-07 ***
## D_DietVegan (consuming no animal products)               -2.593 0.009519 ** 
## D_DietVegetarian (including eggs or milk, but not meat)   0.203 0.838935    
## C_DietOmnivore reducing animal product consumption        1.576 0.115132    
## C_DietPescatarian (including fish but no other meats)     2.869 0.004122 ** 
## C_DietVegan (consuming no animal products)                0.783 0.433647    
## C_DietVegetarian (consuming plants, eggs and milk)        0.474 0.635717    
## ---
## Signif. codes:  0 '***' 0.001 '**' 0.01 '*' 0.05 '.' 0.1 ' ' 1
## 
## (Dispersion parameter for binomial family taken to be 1)
## 
##     Null deviance: 2200.0  on 1657  degrees of freedom
## Residual deviance: 2157.5  on 1650  degrees of freedom
## AIC: 2173.5
## 
## Number of Fisher Scoring iterations: 4  
  # test model fit
with(m, null.deviance - deviance)  
  ## [1] 42.52866  
  with(m, df.null - df.residual)  
  ## [1] 7  
  with(m, pchisq(null.deviance - deviance, df.null - df.residual, lower.tail = FALSE))  
  ## [1] 4.112728e-07  
  BIC(m)  
  ## [1] 2216.807  
  # Hosmer-Lemeshow Goodness-of-Fit Test
hltest(m, G=7)  
  ## 
##    The Hosmer-Lemeshow goodness-of-fit test
## 
##  Group Size Observed  Expected
##      1  289       86  80.03321
##      2   98       29  29.39616
##      3  192       59  60.88536
##      4  158       49  50.87420
##      5  382      151 157.37474
##      6  152       63  65.44545
##      7  112       53  50.01265
##      8  206       98  95.64097
##      9   69       40  38.33725
## 
##          Statistic =  2.00308 
## degrees of freedom =  7 
##            p-value =  0.95967  
  ## CIs using profiled log-likelihood
confint(m, level=0.99)  
  ## Waiting for profiling to be done...  
  ##                                                               0.5 %
## (Intercept)                                             -0.59563412
## D_DietMeat-based – raw                                  -0.90384869
## D_DietVegan (consuming no animal products)              -1.10062543
## D_DietVegetarian (including eggs or milk, but not meat) -0.99541229
## C_DietOmnivore reducing animal product consumption      -0.13609477
## C_DietPescatarian (including fish but no other meats)    0.06490232
## C_DietVegan (consuming no animal products)              -0.32105551
## C_DietVegetarian (consuming plants, eggs and milk)      -0.38419001
##                                                               99.5 %
## (Intercept)                                             -0.118723468
## D_DietMeat-based – raw                                  -0.308295502
## D_DietVegan (consuming no animal products)              -0.005935627
## D_DietVegetarian (including eggs or milk, but not meat)  1.134853749
## C_DietOmnivore reducing animal product consumption       0.559878361
## C_DietPescatarian (including fish but no other meats)    1.228894553
## C_DietVegan (consuming no animal products)               0.592264951
## C_DietVegetarian (consuming plants, eggs and milk)       0.546160380  
  ## CIs using standard errors
confint.default(m, level=0.99)  
  ##                                                               0.5 %
## (Intercept)                                             -0.59402464
## D_DietMeat-based – raw                                  -0.90149785
## D_DietVegan (consuming no animal products)              -1.09601237
## D_DietVegetarian (including eggs or milk, but not meat) -0.96254050
## C_DietOmnivore reducing animal product consumption      -0.13501902
## C_DietPescatarian (including fish but no other meats)    0.06587327
## C_DietVegan (consuming no animal products)              -0.31721345
## C_DietVegetarian (consuming plants, eggs and milk)      -0.37878079
##                                                               99.5 %
## (Intercept)                                             -0.117582604
## D_DietMeat-based – raw                                  -0.306362108
## D_DietVegan (consuming no animal products)              -0.003601304
## D_DietVegetarian (including eggs or milk, but not meat)  1.127459999
## C_DietOmnivore reducing animal product consumption       0.560350884
## C_DietPescatarian (including fish but no other meats)    1.224762030
## C_DietVegan (consuming no animal products)               0.594275794
## C_DietVegetarian (consuming plants, eggs and milk)       0.549489801  
  # Wald test
wald.test(b = coef(m), Sigma = vcov(m), Terms = 2)  
  ## Wald test:
## ----------
## 
## Chi-squared test:
## X2 = 27.3, df = 1, P(> X2) = 1.7e-07  
  ## odds ratios and 95% CI
exp(cbind(OR = coef(m), confint(m, level=0.99)))  
  ## Waiting for profiling to be done...  
  ##                                                                OR     0.5 %
## (Intercept)                                             0.7006102 0.5512129
## D_DietMeat-based – raw                                  0.5466591 0.4050079
## D_DietVegan (consuming no animal products)              0.5770613 0.3326630
## D_DietVegetarian (including eggs or milk, but not meat) 1.0859550 0.3695710
## C_DietOmnivore reducing animal product consumption      1.2369713 0.8727599
## C_DietPescatarian (including fish but no other meats)   1.9065926 1.0670548
## C_DietVegan (consuming no animal products)              1.1485855 0.7253830
## C_DietVegetarian (consuming plants, eggs and milk)      1.0891031 0.6810020
##                                                            99.5 %
## (Intercept)                                             0.8880533
## D_DietMeat-based – raw                                  0.7346982
## D_DietVegan (consuming no animal products)              0.9940820
## D_DietVegetarian (including eggs or milk, but not meat) 3.1107186
## C_DietOmnivore reducing animal product consumption      1.7504596
## C_DietPescatarian (including fish but no other meats)   3.4174496
## C_DietVegan (consuming no animal products)              1.8080790
## C_DietVegetarian (consuming plants, eggs and milk)      1.7266107  
 
 Calculate Nagelkerke R^2 
  NagelkerkeR2(m)  
  ## $N
## [1] 1658
## 
## $R2
## [1] 0.03446875  
 
 
 check assumptions of model 
 
 Cook’s distance 
  plot(m, which = 4, id.n = 3)  
   
 
 
 Extract model results and display data for top 3 values using Cook’s
distance 
  model.data <- augment(m) %>% 
  mutate(index = 1:n()) 
model.data %>% top_n(3, .cooksd)  
 
 
 
 
 
 plot standardised residuals 
  ggplot(model.data, aes(index, .std.resid)) + 
  geom_point(aes(color = Any_Health_Problem), alpha = .5) +
  theme_bw()  
   
 
 
 Filter potential influential data points with abs(.std.res) >
3: 
  model.data %>% 
  filter(abs(.std.resid) > 3)  
 
 
 
 
 
 check for multicollinearity 
  car::vif(m)  
  ##            GVIF Df GVIF^(1/(2*Df))
## D_Diet 1.783459  3        1.101228
## C_Diet 1.783459  4        1.074999  
 
 
 
 Create ROCR for training and test data 
  ## training data
pred.mtt = predict(m, type = "response") #repeat risk predictions from model m
rocr.pred.mtt = ROCR::prediction(pred.mtt, labels = ml_train$Any_Health_Problem) #ROCR prediction object
roc.perf.mtt = ROCR::performance(rocr.pred.mtt, measure = "tpr", x.measure = "fpr") # #ROCR performance object
plot(roc.perf.mtt, col = "blue")


pred.te.1 = predict(m, newdata = ml_test, type = "response") #.te = "test"
rocr.pred.te.1 = ROCR::prediction(pred.te.1, labels = ml_test$Any_Health_Problem)
roc.perf.te.1 = ROCR::performance(rocr.pred.te.1, measure = "tpr", x.measure = "fpr")
plot(roc.perf.te.1, col = "red", add = T)

abline(a = 0, b = 1, lty = 2) #diagonal for random assignment
legend("bottomright", legend = c("train","test"),
col = c("blue","red"), lty = c(2,1), lwd =1.5)  
   
 
 
 Report AUC from ROC for training and test data 
    # Train AUC
aucTr <- ROCR::performance(rocr.pred.mtt, measure = "auc")
  aucTr <- aucTr@y.values[[1]]
  print(aucTr)  
  ## [1] 0.591164  
     # Test AUC
  aucTe <- ROCR::performance(rocr.pred.te.1, measure = "auc")
  aucTe <- aucTe@y.values[[1]]
  print(aucTe)  
  ## [1] 0.5559016  
 
 
 
 THERAPEUTIC DIET binary logistic regression for HEALTH 
  # fit binary logit model and store results 'm'
m <- glm(Any_Health_Problem ~ Therapeutic_Food, data = ml_train,family = binomial)
# view a summary of the model
summary(m)  
  ## 
## Call:
## glm(formula = Any_Health_Problem ~ Therapeutic_Food, family = binomial, 
##     data = ml_train)
## 
## Coefficients:
##                     Estimate Std. Error z value Pr(>|z|)    
## (Intercept)         -0.57701    0.05234 -11.024  < 2e-16 ***
## Therapeutic_FoodYes  1.92940    0.29436   6.555 5.58e-11 ***
## ---
## Signif. codes:  0 '***' 0.001 '**' 0.01 '*' 0.05 '.' 0.1 ' ' 1
## 
## (Dispersion parameter for binomial family taken to be 1)
## 
##     Null deviance: 2200.0  on 1657  degrees of freedom
## Residual deviance: 2144.8  on 1656  degrees of freedom
## AIC: 2148.8
## 
## Number of Fisher Scoring iterations: 4  
  # test model fit
with(m, null.deviance - deviance)  
  ## [1] 55.23019  
  with(m, df.null - df.residual)  
  ## [1] 1  
  with(m, pchisq(null.deviance - deviance, df.null - df.residual, lower.tail = FALSE))  
  ## [1] 1.072093e-13  
  BIC(m)  
  ## [1] 2159.625  
  ## CIs using profiled log-likelihood
confint(m, level=0.99)  
  ## Waiting for profiling to be done...  
  ##                          0.5 %     99.5 %
## (Intercept)         -0.7127343 -0.4429849
## Therapeutic_FoodYes  1.2154551  2.7509045  
  ## CIs using standard errors
confint.default(m, level=0.99)  
  ##                          0.5 %     99.5 %
## (Intercept)         -0.7118297 -0.4421854
## Therapeutic_FoodYes  1.1711802  2.6876205  
  # Wald test
wald.test(b = coef(m), Sigma = vcov(m), Terms = 2)  
  ## Wald test:
## ----------
## 
## Chi-squared test:
## X2 = 43.0, df = 1, P(> X2) = 5.6e-11  
  ## odds ratios and 95% CI
exp(cbind(OR = coef(m), confint(m, level=0.99)))  
  ## Waiting for profiling to be done...  
  ##                            OR     0.5 %     99.5 %
## (Intercept)         0.5615764 0.4903017  0.6421169
## Therapeutic_FoodYes 6.8853801 3.3718283 15.6567867  
 
 Calculate Nagelkerke R^2 
  NagelkerkeR2(m)  
  ## $N
## [1] 1658
## 
## $R2
## [1] 0.04459282  
 
 
 check assumptions of model 
 
 Cook’s distance 
  plot(m, which = 4, id.n = 3)  
   
 
 
 Extract model results and display data for top 3 values using Cook’s
distance 
  model.data <- augment(m) %>% 
  mutate(index = 1:n()) 
model.data %>% top_n(3, .cooksd)  
 
 
 
 
 
 plot standardised residuals 
  ggplot(model.data, aes(index, .std.resid)) + 
  geom_point(aes(color = Any_Health_Problem), alpha = .5) +
  theme_bw()  
   
 
 
 Filter potential influential data points with abs(.std.res) >
3: 
  model.data %>% 
  filter(abs(.std.resid) > 3)  
 
 
 
 
 
 
 Create ROCR for training and test data 
  ## training data
pred.mtt = predict(m, type = "response") #repeat risk predictions from model m
rocr.pred.mtt = ROCR::prediction(pred.mtt, labels = ml_train$Any_Health_Problem) #ROCR prediction object
roc.perf.mtt = ROCR::performance(rocr.pred.mtt, measure = "tpr", x.measure = "fpr") # #ROCR performance object
plot(roc.perf.mtt, col = "blue")


pred.te.1 = predict(m, newdata = ml_test, type = "response") #.te = "test"
rocr.pred.te.1 = ROCR::prediction(pred.te.1, labels = ml_test$Any_Health_Problem)
roc.perf.te.1 = ROCR::performance(rocr.pred.te.1, measure = "tpr", x.measure = "fpr")
plot(roc.perf.te.1, col = "red", add = T)

abline(a = 0, b = 1, lty = 2) #diagonal for random assignment
legend("bottomright", legend = c("train","test"),
col = c("blue","red"), lty = c(2,1), lwd =1.5)  
   
 
 
 Report AUC from ROC for training and test data 
    # Train AUC
aucTr <- ROCR::performance(rocr.pred.mtt, measure = "auc")
  aucTr <- aucTr@y.values[[1]]
  print(aucTr)  
  ## [1] 0.5388968  
     # Test AUC
  aucTe <- ROCR::performance(rocr.pred.te.1, measure = "auc")
  aucTe <- aucTe@y.values[[1]]
  print(aucTe)  
  ## [1] 0.5417851  
 
 
 
 DOG DIET + THERAPEUTIC DIET binary logistic regression for
HEALTH 
  # fit binary logit model and store results 'm'
m <- glm(Any_Health_Problem ~ D_Diet + Therapeutic_Food, data = ml_train,family = binomial)
# view a summary of the model
summary(m)  
  ## 
## Call:
## glm(formula = Any_Health_Problem ~ D_Diet + Therapeutic_Food, 
##     family = binomial, data = ml_train)
## 
## Coefficients:
##                                                         Estimate Std. Error
## (Intercept)                                             -0.33069    0.07081
## D_DietMeat-based – raw                                  -0.53787    0.11599
## D_DietVegan (consuming no animal products)              -0.54583    0.16809
## D_DietVegetarian (including eggs or milk, but not meat) -0.01214    0.41327
## Therapeutic_FoodYes                                      1.84152    0.29709
##                                                         z value Pr(>|z|)    
## (Intercept)                                              -4.670 3.01e-06 ***
## D_DietMeat-based – raw                                   -4.637 3.53e-06 ***
## D_DietVegan (consuming no animal products)               -3.247  0.00117 ** 
## D_DietVegetarian (including eggs or milk, but not meat)  -0.029  0.97657    
## Therapeutic_FoodYes                                       6.198 5.70e-10 ***
## ---
## Signif. codes:  0 '***' 0.001 '**' 0.01 '*' 0.05 '.' 0.1 ' ' 1
## 
## (Dispersion parameter for binomial family taken to be 1)
## 
##     Null deviance: 2200  on 1657  degrees of freedom
## Residual deviance: 2118  on 1653  degrees of freedom
## AIC: 2128
## 
## Number of Fisher Scoring iterations: 4  
  # test model fit
with(m, null.deviance - deviance)  
  ## [1] 82.03845  
  with(m, df.null - df.residual)  
  ## [1] 4  
  with(m, pchisq(null.deviance - deviance, df.null - df.residual, lower.tail = FALSE))  
  ## [1] 6.442074e-17  
  BIC(m)  
  ## [1] 2155.057  
  # Hosmer-Lemeshow Goodness-of-Fit Test
hltest(m, G=3)  
  ## 
##    The Hosmer-Lemeshow goodness-of-fit test
## 
##  Group Size Observed   Expected
##      1  195       56  57.310532
##      2  558      163 164.920026
##      3   23        9   9.547859
##      4  809      342 338.221583
##      5   12       10   8.689468
##      6    7        7   5.079974
##      7    3        3   2.452141
##      8   51       38  41.778417
## 
##          Statistic =  6.12276 
## degrees of freedom =  6 
##            p-value =  0.40958  
  ## CIs using profiled log-likelihood
confint(m, level=0.99)  
  ## Waiting for profiling to be done...  
  ##                                                              0.5 %     99.5 %
## (Intercept)                                             -0.5141051 -0.1490794
## D_DietMeat-based – raw                                  -0.8388553 -0.2408882
## D_DietVegan (consuming no animal products)              -0.9877495 -0.1197432
## D_DietVegetarian (including eggs or milk, but not meat) -1.1171981  1.0528944
## Therapeutic_FoodYes                                      1.1198256  2.6691085  
  ## CIs using standard errors
confint.default(m, level=0.99)  
  ##                                                              0.5 %     99.5 %
## (Intercept)                                             -0.5130720 -0.1483006
## D_DietMeat-based – raw                                  -0.8366421 -0.2390902
## D_DietVegan (consuming no animal products)              -0.9788011 -0.1128594
## D_DietVegetarian (including eggs or milk, but not meat) -1.0766564  1.0523864
## Therapeutic_FoodYes                                      1.0762569  2.6067821  
  # Wald test
wald.test(b = coef(m), Sigma = vcov(m), Terms = 2)  
  ## Wald test:
## ----------
## 
## Chi-squared test:
## X2 = 21.5, df = 1, P(> X2) = 3.5e-06  
  ## odds ratios and 95% CI
exp(cbind(OR = coef(m), confint(m, level=0.99)))  
  ## Waiting for profiling to be done...  
  ##                                                                OR     0.5 %
## (Intercept)                                             0.7184305 0.5980356
## D_DietMeat-based – raw                                  0.5839931 0.4322050
## D_DietVegan (consuming no animal products)              0.5793606 0.3724139
## D_DietVegetarian (including eggs or milk, but not meat) 0.9879383 0.3271953
## Therapeutic_FoodYes                                     6.3061130 3.0643198
##                                                             99.5 %
## (Intercept)                                              0.8615007
## D_DietMeat-based – raw                                   0.7859295
## D_DietVegan (consuming no animal products)               0.8871482
## D_DietVegetarian (including eggs or milk, but not meat)  2.8659343
## Therapeutic_FoodYes                                     14.4271015  
 
 Calculate Nagelkerke R^2 
  NagelkerkeR2(m)  
  ## $N
## [1] 1658
## 
## $R2
## [1] 0.06570812  
 
 
 check assumptions of model 
 
 Cook’s distance 
  plot(m, which = 4, id.n = 3)  
   
 
 
 Extract model results and display data for top 3 values using Cook’s
distance 
  model.data <- augment(m) %>% 
  mutate(index = 1:n()) 
model.data %>% top_n(3, .cooksd)  
 
 
 
 
 
 plot standardised residuals 
  ggplot(model.data, aes(index, .std.resid)) + 
  geom_point(aes(color = Any_Health_Problem), alpha = .5) +
  theme_bw()  
   
 
 
 Filter potential influential data points with abs(.std.res) >
3: 
  model.data %>% 
  filter(abs(.std.resid) > 3)  
 
 
 
 
 
 check for multicollinearity 
  car::vif(m)  
  ##                      GVIF Df GVIF^(1/(2*Df))
## D_Diet           1.007771  3        1.001291
## Therapeutic_Food 1.007771  1        1.003878  
 
 
 
 Create ROCR for training and test data 
  ## training data
pred.mtt = predict(m, type = "response") #repeat risk predictions from model m
rocr.pred.mtt = ROCR::prediction(pred.mtt, labels = ml_train$Any_Health_Problem) #ROCR prediction object
roc.perf.mtt = ROCR::performance(rocr.pred.mtt, measure = "tpr", x.measure = "fpr") # #ROCR performance object
plot(roc.perf.mtt, col = "blue")


pred.te.1 = predict(m, newdata = ml_test, type = "response") #.te = "test"
rocr.pred.te.1 = ROCR::prediction(pred.te.1, labels = ml_test$Any_Health_Problem)
roc.perf.te.1 = ROCR::performance(rocr.pred.te.1, measure = "tpr", x.measure = "fpr")
plot(roc.perf.te.1, col = "red", add = T)

abline(a = 0, b = 1, lty = 2) #diagonal for random assignment
legend("bottomright", legend = c("train","test"),
col = c("blue","red"), lty = c(2,1), lwd =1.5)  
   
 
 
 Report AUC from ROC for training and test data 
    # Train AUC
aucTr <- ROCR::performance(rocr.pred.mtt, measure = "auc")
  aucTr <- aucTr@y.values[[1]]
  print(aucTr)  
  ## [1] 0.6030958  
     # Test AUC
  aucTe <- ROCR::performance(rocr.pred.te.1, measure = "auc")
  aucTe <- aucTe@y.values[[1]]
  print(aucTe)  
  ## [1] 0.5774108  
 
 
 
 DOG DIET * THERAPEUTIC DIET binary logistic regression for
HEALTH 
  # fit binary logit model and store results 'm'
m <- glm(Any_Health_Problem ~ D_Diet*Therapeutic_Food, data = ml_train,family = binomial)
# view a summary of the model
summary(m)  
  ## 
## Call:
## glm(formula = Any_Health_Problem ~ D_Diet * Therapeutic_Food, 
##     family = binomial, data = ml_train)
## 
## Coefficients:
##                                                                              Estimate
## (Intercept)                                                                  -0.31152
## D_DietMeat-based – raw                                                       -0.57362
## D_DietVegan (consuming no animal products)                                   -0.59760
## D_DietVegetarian (including eggs or milk, but not meat)                      -0.13031
## Therapeutic_FoodYes                                                           1.38416
## D_DietMeat-based – raw:Therapeutic_FoodYes                                   14.06705
## D_DietVegan (consuming no animal products):Therapeutic_FoodYes                1.13440
## D_DietVegetarian (including eggs or milk, but not meat):Therapeutic_FoodYes  13.62375
##                                                                             Std. Error
## (Intercept)                                                                    0.07117
## D_DietMeat-based – raw                                                         0.11718
## D_DietVegan (consuming no animal products)                                     0.17354
## D_DietVegetarian (including eggs or milk, but not meat)                        0.43313
## Therapeutic_FoodYes                                                            0.32910
## D_DietMeat-based – raw:Therapeutic_FoodYes                                   333.64581
## D_DietVegan (consuming no animal products):Therapeutic_FoodYes                 0.85636
## D_DietVegetarian (including eggs or milk, but not meat):Therapeutic_FoodYes  509.65241
##                                                                             z value
## (Intercept)                                                                  -4.377
## D_DietMeat-based – raw                                                       -4.895
## D_DietVegan (consuming no animal products)                                   -3.444
## D_DietVegetarian (including eggs or milk, but not meat)                      -0.301
## Therapeutic_FoodYes                                                           4.206
## D_DietMeat-based – raw:Therapeutic_FoodYes                                    0.042
## D_DietVegan (consuming no animal products):Therapeutic_FoodYes                1.325
## D_DietVegetarian (including eggs or milk, but not meat):Therapeutic_FoodYes   0.027
##                                                                             Pr(>|z|)
## (Intercept)                                                                 1.20e-05
## D_DietMeat-based – raw                                                      9.83e-07
## D_DietVegan (consuming no animal products)                                  0.000574
## D_DietVegetarian (including eggs or milk, but not meat)                     0.763519
## Therapeutic_FoodYes                                                         2.60e-05
## D_DietMeat-based – raw:Therapeutic_FoodYes                                  0.966370
## D_DietVegan (consuming no animal products):Therapeutic_FoodYes              0.185278
## D_DietVegetarian (including eggs or milk, but not meat):Therapeutic_FoodYes 0.978674
##                                                                                
## (Intercept)                                                                 ***
## D_DietMeat-based – raw                                                      ***
## D_DietVegan (consuming no animal products)                                  ***
## D_DietVegetarian (including eggs or milk, but not meat)                        
## Therapeutic_FoodYes                                                         ***
## D_DietMeat-based – raw:Therapeutic_FoodYes                                     
## D_DietVegan (consuming no animal products):Therapeutic_FoodYes                 
## D_DietVegetarian (including eggs or milk, but not meat):Therapeutic_FoodYes    
## ---
## Signif. codes:  0 '***' 0.001 '**' 0.01 '*' 0.05 '.' 0.1 ' ' 1
## 
## (Dispersion parameter for binomial family taken to be 1)
## 
##     Null deviance: 2200.0  on 1657  degrees of freedom
## Residual deviance: 2109.6  on 1650  degrees of freedom
## AIC: 2125.6
## 
## Number of Fisher Scoring iterations: 13  
  # test model fit
with(m, null.deviance - deviance)  
  ## [1] 90.45554  
  with(m, df.null - df.residual)  
  ## [1] 7  
  with(m, pchisq(null.deviance - deviance, df.null - df.residual, lower.tail = FALSE))  
  ## [1] 9.974578e-17  
  BIC(m)  
  ## [1] 2168.88  
  # Hosmer-Lemeshow Goodness-of-Fit Test
hltest(m, G=7)  
  ## 
##    The Hosmer-Lemeshow goodness-of-fit test
## 
##  Group Size Observed   Expected
##      1  195       56  56.000000
##      2  558      163 163.000000
##      3   23        9   9.000000
##      4  809      342 342.000000
##      5   51       38  38.000000
##      6   12       10  10.000000
##      7   10       10   9.999995
## 
##          Statistic =  0 
## degrees of freedom =  5 
##            p-value =  1  
  ## CIs using profiled log-likelihood
confint(m, level=0.99)  
  ## Waiting for profiling to be done...  
  ## Warning: glm.fit: fitted probabilities numerically 0 or 1 occurred

## Warning: glm.fit: fitted probabilities numerically 0 or 1 occurred

## Warning: glm.fit: fitted probabilities numerically 0 or 1 occurred

## Warning: glm.fit: fitted probabilities numerically 0 or 1 occurred

## Warning: glm.fit: fitted probabilities numerically 0 or 1 occurred

## Warning: glm.fit: fitted probabilities numerically 0 or 1 occurred

## Warning: glm.fit: fitted probabilities numerically 0 or 1 occurred

## Warning: glm.fit: fitted probabilities numerically 0 or 1 occurred

## Warning: glm.fit: fitted probabilities numerically 0 or 1 occurred

## Warning: glm.fit: fitted probabilities numerically 0 or 1 occurred

## Warning: glm.fit: fitted probabilities numerically 0 or 1 occurred

## Warning: glm.fit: fitted probabilities numerically 0 or 1 occurred

## Warning: glm.fit: fitted probabilities numerically 0 or 1 occurred

## Warning: glm.fit: fitted probabilities numerically 0 or 1 occurred

## Warning: glm.fit: fitted probabilities numerically 0 or 1 occurred

## Warning: glm.fit: fitted probabilities numerically 0 or 1 occurred

## Warning: glm.fit: fitted probabilities numerically 0 or 1 occurred

## Warning: glm.fit: fitted probabilities numerically 0 or 1 occurred

## Warning: glm.fit: fitted probabilities numerically 0 or 1 occurred

## Warning: glm.fit: fitted probabilities numerically 0 or 1 occurred

## Warning: glm.fit: fitted probabilities numerically 0 or 1 occurred

## Warning: glm.fit: fitted probabilities numerically 0 or 1 occurred

## Warning: glm.fit: fitted probabilities numerically 0 or 1 occurred

## Warning: glm.fit: fitted probabilities numerically 0 or 1 occurred

## Warning: glm.fit: fitted probabilities numerically 0 or 1 occurred  
  ##                                                                                   0.5 %
## (Intercept)                                                                  -0.4958401
## D_DietMeat-based – raw                                                       -0.8778840
## D_DietVegan (consuming no animal products)                                   -1.0555998
## D_DietVegetarian (including eggs or milk, but not meat)                      -1.3176151
## Therapeutic_FoodYes                                                           0.5785993
## D_DietMeat-based – raw:Therapeutic_FoodYes                                  -31.6598124
## D_DietVegan (consuming no animal products):Therapeutic_FoodYes               -0.8481864
## D_DietVegetarian (including eggs or milk, but not meat):Therapeutic_FoodYes -89.1693657
##                                                                                 99.5 %
## (Intercept)                                                                 -0.1289316
## D_DietMeat-based – raw                                                      -0.2737581
## D_DietVegan (consuming no animal products)                                  -0.1590734
## D_DietVegetarian (including eggs or milk, but not meat)                      0.9687654
## Therapeutic_FoodYes                                                          2.2986144
## D_DietMeat-based – raw:Therapeutic_FoodYes                                          NA
## D_DietVegan (consuming no animal products):Therapeutic_FoodYes               3.9240025
## D_DietVegetarian (including eggs or milk, but not meat):Therapeutic_FoodYes         NA  
  ## CIs using standard errors
confint.default(m, level=0.99)  
  ##                                                                                     0.5 %
## (Intercept)                                                                    -0.4948427
## D_DietMeat-based – raw                                                         -0.8754611
## D_DietVegan (consuming no animal products)                                     -1.0446173
## D_DietVegetarian (including eggs or milk, but not meat)                        -1.2459933
## Therapeutic_FoodYes                                                             0.5364602
## D_DietMeat-based – raw:Therapeutic_FoodYes                                   -845.3476061
## D_DietVegan (consuming no animal products):Therapeutic_FoodYes                 -1.0714370
## D_DietVegetarian (including eggs or milk, but not meat):Therapeutic_FoodYes -1299.1538686
##                                                                                   99.5 %
## (Intercept)                                                                   -0.1281943
## D_DietMeat-based – raw                                                        -0.2717729
## D_DietVegan (consuming no animal products)                                    -0.1505901
## D_DietVegetarian (including eggs or milk, but not meat)                        0.9853648
## Therapeutic_FoodYes                                                            2.2318504
## D_DietMeat-based – raw:Therapeutic_FoodYes                                   873.4817021
## D_DietVegan (consuming no animal products):Therapeutic_FoodYes                 3.3402467
## D_DietVegetarian (including eggs or milk, but not meat):Therapeutic_FoodYes 1326.4013591  
  # Wald test
wald.test(b = coef(m), Sigma = vcov(m), Terms = 2)  
  ## Wald test:
## ----------
## 
## Chi-squared test:
## X2 = 24.0, df = 1, P(> X2) = 9.8e-07  
  ## odds ratios and 95% CI
exp(cbind(OR = coef(m), confint(m, level=0.99)))  
  ## Waiting for profiling to be done...  
  ## Warning: glm.fit: fitted probabilities numerically 0 or 1 occurred

## Warning: glm.fit: fitted probabilities numerically 0 or 1 occurred

## Warning: glm.fit: fitted probabilities numerically 0 or 1 occurred

## Warning: glm.fit: fitted probabilities numerically 0 or 1 occurred

## Warning: glm.fit: fitted probabilities numerically 0 or 1 occurred

## Warning: glm.fit: fitted probabilities numerically 0 or 1 occurred

## Warning: glm.fit: fitted probabilities numerically 0 or 1 occurred

## Warning: glm.fit: fitted probabilities numerically 0 or 1 occurred

## Warning: glm.fit: fitted probabilities numerically 0 or 1 occurred

## Warning: glm.fit: fitted probabilities numerically 0 or 1 occurred

## Warning: glm.fit: fitted probabilities numerically 0 or 1 occurred

## Warning: glm.fit: fitted probabilities numerically 0 or 1 occurred

## Warning: glm.fit: fitted probabilities numerically 0 or 1 occurred

## Warning: glm.fit: fitted probabilities numerically 0 or 1 occurred

## Warning: glm.fit: fitted probabilities numerically 0 or 1 occurred

## Warning: glm.fit: fitted probabilities numerically 0 or 1 occurred

## Warning: glm.fit: fitted probabilities numerically 0 or 1 occurred

## Warning: glm.fit: fitted probabilities numerically 0 or 1 occurred

## Warning: glm.fit: fitted probabilities numerically 0 or 1 occurred

## Warning: glm.fit: fitted probabilities numerically 0 or 1 occurred

## Warning: glm.fit: fitted probabilities numerically 0 or 1 occurred

## Warning: glm.fit: fitted probabilities numerically 0 or 1 occurred

## Warning: glm.fit: fitted probabilities numerically 0 or 1 occurred

## Warning: glm.fit: fitted probabilities numerically 0 or 1 occurred

## Warning: glm.fit: fitted probabilities numerically 0 or 1 occurred  
  ##                                                                                       OR
## (Intercept)                                                                 7.323340e-01
## D_DietMeat-based – raw                                                      5.634836e-01
## D_DietVegan (consuming no animal products)                                  5.501283e-01
## D_DietVegetarian (including eggs or milk, but not meat)                     8.778195e-01
## Therapeutic_FoodYes                                                         3.991453e+00
## D_DietMeat-based – raw:Therapeutic_FoodYes                                  1.286001e+06
## D_DietVegan (consuming no animal products):Therapeutic_FoodYes              3.109322e+00
## D_DietVegetarian (including eggs or milk, but not meat):Therapeutic_FoodYes 8.255005e+05
##                                                                                    0.5 %
## (Intercept)                                                                 6.090590e-01
## D_DietMeat-based – raw                                                      4.156615e-01
## D_DietVegan (consuming no animal products)                                  3.479836e-01
## D_DietVegetarian (including eggs or milk, but not meat)                     2.677731e-01
## Therapeutic_FoodYes                                                         1.783539e+00
## D_DietMeat-based – raw:Therapeutic_FoodYes                                  1.779583e-14
## D_DietVegan (consuming no animal products):Therapeutic_FoodYes              4.281908e-01
## D_DietVegetarian (including eggs or milk, but not meat):Therapeutic_FoodYes 1.880341e-39
##                                                                                 99.5 %
## (Intercept)                                                                  0.8790341
## D_DietMeat-based – raw                                                       0.7605160
## D_DietVegan (consuming no animal products)                                   0.8529337
## D_DietVegetarian (including eggs or milk, but not meat)                      2.6346896
## Therapeutic_FoodYes                                                          9.9603715
## D_DietMeat-based – raw:Therapeutic_FoodYes                                          NA
## D_DietVegan (consuming no animal products):Therapeutic_FoodYes              50.6025790
## D_DietVegetarian (including eggs or milk, but not meat):Therapeutic_FoodYes         NA  
 
 Calculate Nagelkerke R^2 
  NagelkerkeR2(m)  
  ## $N
## [1] 1658
## 
## $R2
## [1] 0.07226765  
 
 
 check assumptions of model 
 
 Cook’s distance 
  plot(m, which = 4, id.n = 3)  
   
 
 
 Extract model results and display data for top 3 values using Cook’s
distance 
  model.data <- augment(m) %>% 
  mutate(index = 1:n()) 
model.data %>% top_n(3, .cooksd)  
 
 
 
 
 
 plot standardised residuals 
  ggplot(model.data, aes(index, .std.resid)) + 
  geom_point(aes(color = Any_Health_Problem), alpha = .5) +
  theme_bw()  
   
 
 
 Filter potential influential data points with abs(.std.res) >
3: 
  model.data %>% 
  filter(abs(.std.resid) > 3)  
 
 
 
 
 
 check for multicollinearity 
 
 
 Note interactions 
  car::vif(m)  
  ## there are higher-order terms (interactions) in this model
## consider setting type = 'predictor'; see ?vif  
  ##                             GVIF Df GVIF^(1/(2*Df))
## D_Diet                  1.059292  3        1.009646
## Therapeutic_Food        1.191799  1        1.091696
## D_Diet:Therapeutic_Food 1.216749  3        1.033238  
 
 
 
 Create ROCR for training and test data 
  ## training data
pred.mtt = predict(m, type = "response") #repeat risk predictions from model m
rocr.pred.mtt = ROCR::prediction(pred.mtt, labels = ml_train$Any_Health_Problem) #ROCR prediction object
roc.perf.mtt = ROCR::performance(rocr.pred.mtt, measure = "tpr", x.measure = "fpr") # #ROCR performance object
plot(roc.perf.mtt, col = "blue")


pred.te.1 = predict(m, newdata = ml_test, type = "response") #.te = "test"
rocr.pred.te.1 = ROCR::prediction(pred.te.1, labels = ml_test$Any_Health_Problem)
roc.perf.te.1 = ROCR::performance(rocr.pred.te.1, measure = "tpr", x.measure = "fpr")
plot(roc.perf.te.1, col = "red", add = T)

abline(a = 0, b = 1, lty = 2) #diagonal for random assignment
legend("bottomright", legend = c("train","test"),
col = c("blue","red"), lty = c(2,1), lwd =1.5)  
   
 
 
 Report AUC from ROC for training and test data 
    # Train AUC
aucTr <- ROCR::performance(rocr.pred.mtt, measure = "auc")
  aucTr <- aucTr@y.values[[1]]
  print(aucTr)  
  ## [1] 0.6033803  
     # Test AUC
  aucTe <- ROCR::performance(rocr.pred.te.1, measure = "auc")
  aucTe <- aucTe@y.values[[1]]
  print(aucTe)  
  ## [1] 0.5770764  
 
 
 
 SIZE Binary logistic regression for HEALTH 
  # fit binary logit model and store results 'm'
m <- glm(Any_Health_Problem ~ Size, data = ml_train,family = binomial)
# view a summary of the model
summary(m)  
  ## 
## Call:
## glm(formula = Any_Health_Problem ~ Size, family = binomial, data = ml_train)
## 
## Coefficients:
##             Estimate Std. Error z value Pr(>|z|)    
## (Intercept) -0.47358    0.08814  -5.373 7.74e-08 ***
## Size.L       0.07352    0.26265   0.280   0.7796    
## Size.Q       0.02524    0.22633   0.112   0.9112    
## Size.C       0.16418    0.15720   1.044   0.2963    
## Size^4      -0.17831    0.10222  -1.744   0.0811 .  
## ---
## Signif. codes:  0 '***' 0.001 '**' 0.01 '*' 0.05 '.' 0.1 ' ' 1
## 
## (Dispersion parameter for binomial family taken to be 1)
## 
##     Null deviance: 2200  on 1657  degrees of freedom
## Residual deviance: 2195  on 1653  degrees of freedom
## AIC: 2205
## 
## Number of Fisher Scoring iterations: 4  
  # test model fit
with(m, null.deviance - deviance)  
  ## [1] 5.043677  
  with(m, df.null - df.residual)  
  ## [1] 4  
  with(m, pchisq(null.deviance - deviance, df.null - df.residual, lower.tail = FALSE))  
  ## [1] 0.2828453  
  BIC(m)  
  ## [1] 2232.052  
  # Hosmer-Lemeshow Goodness-of-Fit Test
hltest(m)  
  ## 
##    The Hosmer-Lemeshow goodness-of-fit test
## 
##  Group Size Observed Expected
##      1  644      226      226
##      2   39       14       14
##      3  574      220      220
##      4   74       30       30
##      5  327      138      138
## 
##          Statistic =  0 
## degrees of freedom =  3 
##            p-value =  1  
  ## CIs using profiled log-likelihood
confint(m, level=0.99)  
  ## Waiting for profiling to be done...  
  ##                  0.5 %      99.5 %
## (Intercept) -0.7068659 -0.24996619
## Size.L      -0.5964467  0.76801029
## Size.Q      -0.5757175  0.59869881
## Size.C      -0.2383461  0.57498997
## Size^4      -0.4426779  0.08433946  
  ## CIs using standard errors
confint.default(m, level=0.99)  
  ##                  0.5 %      99.5 %
## (Intercept) -0.7006161 -0.24655185
## Size.L      -0.6030330  0.75006400
## Size.Q      -0.5577404  0.60821751
## Size.C      -0.2407430  0.56910023
## Size^4      -0.4416107  0.08499312  
  # Wald test
wald.test(b = coef(m), Sigma = vcov(m), Terms = 2)  
  ## Wald test:
## ----------
## 
## Chi-squared test:
## X2 = 0.078, df = 1, P(> X2) = 0.78  
  ## odds ratios and 95% CI
exp(cbind(OR = coef(m), confint(m, level=0.99)))  
  ## Waiting for profiling to be done...  
  ##                    OR     0.5 %    99.5 %
## (Intercept) 0.6227663 0.4931875 0.7788271
## Size.L      1.0762852 0.5507652 2.1554732
## Size.Q      1.0255598 0.5623012 1.8197494
## Size.C      1.1784248 0.7879299 1.7771127
## Size^4      0.8366840 0.6423141 1.0879982  
 
 Calculate Nagelkerke R^2 
  NagelkerkeR2(m)  
  ## $N
## [1] 1658
## 
## $R2
## [1] 0.004134173  
 
 
 check assumptions of model 
 
 Cook’s distance 
  plot(m, which = 4, id.n = 3)  
   
 
 
 Extract model results and display data for top 3 values using Cook’s
distance 
  model.data <- augment(m) %>% 
  mutate(index = 1:n()) 
model.data %>% top_n(3, .cooksd)  
 
 
 
 
 
 plot standardised residuals 
  ggplot(model.data, aes(index, .std.resid)) + 
  geom_point(aes(color = Any_Health_Problem), alpha = .5) +
  theme_bw()  
   
 
 
 Filter potential influential data points with abs(.std.res) >
3: 
  model.data %>% 
  filter(abs(.std.resid) > 3)  
 
 
 
 
 
 
 Create ROCR for training and test data 
  ## training data
pred.mtt = predict(m, type = "response") #repeat risk predictions from model m
rocr.pred.mtt = ROCR::prediction(pred.mtt, labels = ml_train$Any_Health_Problem) #ROCR prediction object
roc.perf.mtt = ROCR::performance(rocr.pred.mtt, measure = "tpr", x.measure = "fpr") # #ROCR performance object
plot(roc.perf.mtt, col = "blue")


pred.te.1 = predict(m, newdata = ml_test, type = "response") #.te = "test"
rocr.pred.te.1 = ROCR::prediction(pred.te.1, labels = ml_test$Any_Health_Problem)
roc.perf.te.1 = ROCR::performance(rocr.pred.te.1, measure = "tpr", x.measure = "fpr")
plot(roc.perf.te.1, col = "red", add = T)

abline(a = 0, b = 1, lty = 2) #diagonal for random assignment
legend("bottomright", legend = c("train","test"),
col = c("blue","red"), lty = c(2,1), lwd =1.5)  
   
 
 
 Report AUC from ROC for training and test data 
    # Train AUC
aucTr <- ROCR::performance(rocr.pred.mtt, measure = "auc")
  aucTr <- aucTr@y.values[[1]]
  print(aucTr)  
  ## [1] 0.5307804  
     # Test AUC
  aucTe <- ROCR::performance(rocr.pred.te.1, measure = "auc")
  aucTe <- aucTe@y.values[[1]]
  print(aucTe)  
  ## [1] 0.5326157  
 
 
 
 SIZE2 Binary logistic regression for HEALTH 
  # fit binary logit model and store results 'm'
m <- glm(Any_Health_Problem ~ Size2, data = ml_train,family = binomial)
# view a summary of the model
summary(m)  
  ## 
## Call:
## glm(formula = Any_Health_Problem ~ Size2, family = binomial, 
##     data = ml_train)
## 
## Coefficients:
##             Estimate Std. Error z value Pr(>|z|)    
## (Intercept) -0.61495    0.08257  -7.448 9.48e-14 ***
## Size2Toy     0.03513    0.34387   0.102   0.9186    
## Size2Small   0.30045    0.13912   2.160   0.0308 *  
## Size2Large   0.13928    0.11911   1.169   0.2423    
## Size2Giant   0.23195    0.25075   0.925   0.3550    
## ---
## Signif. codes:  0 '***' 0.001 '**' 0.01 '*' 0.05 '.' 0.1 ' ' 1
## 
## (Dispersion parameter for binomial family taken to be 1)
## 
##     Null deviance: 2200  on 1657  degrees of freedom
## Residual deviance: 2195  on 1653  degrees of freedom
## AIC: 2205
## 
## Number of Fisher Scoring iterations: 4  
  # test model fit
with(m, null.deviance - deviance)  
  ## [1] 5.043677  
  with(m, df.null - df.residual)  
  ## [1] 4  
  with(m, pchisq(null.deviance - deviance, df.null - df.residual, lower.tail = FALSE))  
  ## [1] 0.2828453  
  BIC(m)  
  ## [1] 2232.052  
  # Hosmer-Lemeshow Goodness-of-Fit Test
hltest(m)  
  ## 
##    The Hosmer-Lemeshow goodness-of-fit test
## 
##  Group Size Observed Expected
##      1  644      226      226
##      2   39       14       14
##      3  574      220      220
##      4   74       30       30
##      5  327      138      138
## 
##          Statistic =  0 
## degrees of freedom =  3 
##            p-value =  1  
  ## CIs using profiled log-likelihood
confint(m, level=0.99)  
  ## Waiting for profiling to be done...  
  ##                   0.5 %     99.5 %
## (Intercept) -0.83008197 -0.4043168
## Size2Toy    -0.89671166  0.9002316
## Size2Small  -0.05884591  0.6585199
## Size2Large  -0.16762496  0.4463614
## Size2Giant  -0.42843213  0.8716162  
  ## CIs using standard errors
confint.default(m, level=0.99)  
  ##                   0.5 %     99.5 %
## (Intercept) -0.82762201 -0.4022709
## Size2Toy    -0.85061912  0.9208750
## Size2Small  -0.05789747  0.6588037
## Size2Large  -0.16753259  0.4460867
## Size2Giant  -0.41394614  0.8778545  
  # Wald test
wald.test(b = coef(m), Sigma = vcov(m), Terms = 2)  
  ## Wald test:
## ----------
## 
## Chi-squared test:
## X2 = 0.01, df = 1, P(> X2) = 0.92  
  ## odds ratios and 95% CI
exp(cbind(OR = coef(m), confint(m, level=0.99)))  
  ## Waiting for profiling to be done...  
  ##                    OR     0.5 %    99.5 %
## (Intercept) 0.5406699 0.4360135 0.6674327
## Size2Toy    1.0357522 0.4079088 2.4601729
## Size2Small  1.3504706 0.9428520 1.9319308
## Size2Large  1.1494425 0.8456709 1.5626161
## Size2Giant  1.2610619 0.6515298 2.3907718  
 
 Calculate Nagelkerke R^2 
  NagelkerkeR2(m)  
  ## $N
## [1] 1658
## 
## $R2
## [1] 0.004134173  
 
 
 check assumptions of model 
 
 Cook’s distance 
  plot(m, which = 4, id.n = 3)  
   
 
 
 Extract model results and display data for top 3 values using Cook’s
distance 
  model.data <- augment(m) %>% 
  mutate(index = 1:n()) 
model.data %>% top_n(3, .cooksd)  
 
 
 
 
 
 plot standardised residuals 
  ggplot(model.data, aes(index, .std.resid)) + 
  geom_point(aes(color = Any_Health_Problem), alpha = .5) +
  theme_bw()  
   
 
 
 Filter potential influential data points with abs(.std.res) >
3: 
  model.data %>% 
  filter(abs(.std.resid) > 3)  
 
 
 
 
 
 
 Create ROCR for training and test data 
  ## training data
pred.mtt = predict(m, type = "response") #repeat risk predictions from model m
rocr.pred.mtt = ROCR::prediction(pred.mtt, labels = ml_train$Any_Health_Problem) #ROCR prediction object
roc.perf.mtt = ROCR::performance(rocr.pred.mtt, measure = "tpr", x.measure = "fpr") # #ROCR performance object
plot(roc.perf.mtt, col = "blue")


pred.te.1 = predict(m, newdata = ml_test, type = "response") #.te = "test"
rocr.pred.te.1 = ROCR::prediction(pred.te.1, labels = ml_test$Any_Health_Problem)
roc.perf.te.1 = ROCR::performance(rocr.pred.te.1, measure = "tpr", x.measure = "fpr")
plot(roc.perf.te.1, col = "red", add = T)

abline(a = 0, b = 1, lty = 2) #diagonal for random assignment
legend("bottomright", legend = c("train","test"),
col = c("blue","red"), lty = c(2,1), lwd =1.5)  
   
 
 
 Report AUC from ROC for training and test data 
    # Train AUC
aucTr <- ROCR::performance(rocr.pred.mtt, measure = "auc")
  aucTr <- aucTr@y.values[[1]]
  print(aucTr)  
  ## [1] 0.5307804  
     # Test AUC
  aucTe <- ROCR::performance(rocr.pred.te.1, measure = "auc")
  aucTe <- aucTe@y.values[[1]]
  print(aucTe)  
  ## [1] 0.5326157  
 
 
 
 SIZE_GIANT Binary logistic regression for HEALTH 
  # fit binary logit model and store results 'm'
m <- glm(Any_Health_Problem ~ Size_Giant, data = ml_train,family = binomial)
# view a summary of the model
summary(m)  
  ## 
## Call:
## glm(formula = Any_Health_Problem ~ Size_Giant, family = binomial, 
##     data = ml_train)
## 
## Coefficients:
##               Estimate Std. Error z value Pr(>|z|)    
## (Intercept)   -0.50007    0.05183  -9.648   <2e-16 ***
## Size_GiantYes  0.11707    0.24238   0.483    0.629    
## ---
## Signif. codes:  0 '***' 0.001 '**' 0.01 '*' 0.05 '.' 0.1 ' ' 1
## 
## (Dispersion parameter for binomial family taken to be 1)
## 
##     Null deviance: 2200.0  on 1657  degrees of freedom
## Residual deviance: 2199.8  on 1656  degrees of freedom
## AIC: 2203.8
## 
## Number of Fisher Scoring iterations: 4  
  # test model fit
with(m, null.deviance - deviance)  
  ## [1] 0.2316414  
  with(m, df.null - df.residual)  
  ## [1] 1  
  with(m, pchisq(null.deviance - deviance, df.null - df.residual, lower.tail = FALSE))  
  ## [1] 0.6303094  
  BIC(m)  
  ## [1] 2214.624  
  ## CIs using profiled log-likelihood
confint(m, level=0.99)  
  ## Waiting for profiling to be done...  
  ##                    0.5 %     99.5 %
## (Intercept)   -0.6343528 -0.3672363
## Size_GiantYes -0.5230473  0.7346156  
  ## CIs using standard errors
confint.default(m, level=0.99)  
  ##                    0.5 %     99.5 %
## (Intercept)   -0.6335731 -0.3665581
## Size_GiantYes -0.5072507  0.7413974  
  # Wald test
wald.test(b = coef(m), Sigma = vcov(m), Terms = 2)  
  ## Wald test:
## ----------
## 
## Chi-squared test:
## X2 = 0.23, df = 1, P(> X2) = 0.63  
  ## odds ratios and 95% CI
exp(cbind(OR = coef(m), confint(m, level=0.99)))  
  ## Waiting for profiling to be done...  
  ##                      OR     0.5 %    99.5 %
## (Intercept)   0.6064909 0.5302786 0.6926459
## Size_GiantYes 1.1242019 0.5927116 2.0846804  
 
 Calculate Nagelkerke R^2 
  NagelkerkeR2(m)  
  ## $N
## [1] 1658
## 
## $R2
## [1] 0.0001901462  
 
 
 check assumptions of model 
 
 Cook’s distance 
  plot(m, which = 4, id.n = 3)  
   
 
 
 Extract model results and display data for top 3 values using Cook’s
distance 
  model.data <- augment(m) %>% 
  mutate(index = 1:n()) 
model.data %>% top_n(3, .cooksd)  
 
 
 
 
 
 plot standardised residuals 
  ggplot(model.data, aes(index, .std.resid)) + 
  geom_point(aes(color = Any_Health_Problem), alpha = .5) +
  theme_bw()  
   
 
 
 Filter potential influential data points with abs(.std.res) >
3: 
  model.data %>% 
  filter(abs(.std.resid) > 3)  
 
 
 
 
 
 
 Create ROCR for training and test data 
  ## training data
pred.mtt = predict(m, type = "response") #repeat risk predictions from model m
rocr.pred.mtt = ROCR::prediction(pred.mtt, labels = ml_train$Any_Health_Problem) #ROCR prediction object
roc.perf.mtt = ROCR::performance(rocr.pred.mtt, measure = "tpr", x.measure = "fpr") # #ROCR performance object
plot(roc.perf.mtt, col = "blue")


pred.te.1 = predict(m, newdata = ml_test, type = "response") #.te = "test"
rocr.pred.te.1 = ROCR::prediction(pred.te.1, labels = ml_test$Any_Health_Problem)
roc.perf.te.1 = ROCR::performance(rocr.pred.te.1, measure = "tpr", x.measure = "fpr")
plot(roc.perf.te.1, col = "red", add = T)

abline(a = 0, b = 1, lty = 2) #diagonal for random assignment
legend("bottomright", legend = c("train","test"),
col = c("blue","red"), lty = c(2,1), lwd =1.5)  
   
 
 
 Report AUC from ROC for training and test data 
    # Train AUC
aucTr <- ROCR::performance(rocr.pred.mtt, measure = "auc")
  aucTr <- aucTr@y.values[[1]]
  print(aucTr)  
  ## [1] 0.5025261  
     # Test AUC
  aucTe <- ROCR::performance(rocr.pred.te.1, measure = "auc")
  aucTe <- aucTe@y.values[[1]]
  print(aucTe)  
  ## [1] 0.5051979  
 
 
 
 D_AGE Binary logistic regression for HEALTH 
  # fit binary logit model and store results 'm'
m <- glm(Any_Health_Problem ~ D_Age, data = ml_train,family = binomial)
# view a summary of the model
summary(m)  
  ## 
## Call:
## glm(formula = Any_Health_Problem ~ D_Age, family = binomial, 
##     data = ml_train)
## 
## Coefficients:
##             Estimate Std. Error z value Pr(>|z|)    
## (Intercept) -1.40277    0.10748 -13.051   <2e-16 ***
## D_Age        0.14396    0.01458   9.873   <2e-16 ***
## ---
## Signif. codes:  0 '***' 0.001 '**' 0.01 '*' 0.05 '.' 0.1 ' ' 1
## 
## (Dispersion parameter for binomial family taken to be 1)
## 
##     Null deviance: 2200.0  on 1657  degrees of freedom
## Residual deviance: 2096.4  on 1656  degrees of freedom
## AIC: 2100.4
## 
## Number of Fisher Scoring iterations: 4  
  # test model fit
with(m, null.deviance - deviance)  
  ## [1] 103.643  
  with(m, df.null - df.residual)  
  ## [1] 1  
  with(m, pchisq(null.deviance - deviance, df.null - df.residual, lower.tail = FALSE))  
  ## [1] 2.422557e-24  
  BIC(m)  
  ## [1] 2111.212  
  # Hosmer-Lemeshow Goodness-of-Fit Test
hltest(m)  
  ## 
##    The Hosmer-Lemeshow goodness-of-fit test
## 
##  Group Size Observed Expected
##      1  114       28 25.21441
##      2  177       49 43.71338
##      3  194       54 53.29254
##      4  173       48 52.64312
##      5  173       55 58.05879
##      6  143       53 52.68397
##      7  134       40 53.93553
##      8  130       60 56.88244
##      9  191       95 93.66255
##     10  159      104 90.99484
##     11   70       42 46.91842
## 
##          Statistic =  14.36815 
## degrees of freedom =  9 
##            p-value =  0.10982  
  ## CIs using profiled log-likelihood
confint(m, level=0.99)  
  ## Waiting for profiling to be done...  
  ##                  0.5 %     99.5 %
## (Intercept) -1.6836141 -1.1295224
## D_Age        0.1067895  0.1819739  
  ## CIs using standard errors
confint.default(m, level=0.99)  
  ##                  0.5 %     99.5 %
## (Intercept) -1.6796215 -1.1259175
## D_Age        0.1064016  0.1815203  
  # Wald test
wald.test(b = coef(m), Sigma = vcov(m), Terms = 2)  
  ## Wald test:
## ----------
## 
## Chi-squared test:
## X2 = 97.5, df = 1, P(> X2) = 0.0  
  ## odds ratios and 95% CI
exp(cbind(OR = coef(m), confint(m, level=0.99)))  
  ## Waiting for profiling to be done...  
  ##                   OR     0.5 %    99.5 %
## (Intercept) 0.245915 0.1857016 0.3231876
## D_Age       1.154839 1.1127000 1.1995829  
 
 Calculate Nagelkerke R^2 
  NagelkerkeR2(m)  
  ## $N
## [1] 1658
## 
## $R2
## [1] 0.0824781  
 
 
 Check age is linear with logit of outcome 
 
 Note lack of linearity 
  ypred = predict(m)
res = residuals(m, type = 'deviance')
plot(ypred,res)  
   
 
 
 
 Box Tidwell test to check that D_Age is linearly associated with the
logit of the outcome 
 
 suggests not linear 
  boxTidwell(ml$Health_Binary ~ ml$D_Age)  
  ##  MLE of lambda Score Statistic (t) Pr(>|t|)   
##         2.5903              3.1841 0.001473 **
## ---
## Signif. codes:  0 '***' 0.001 '**' 0.01 '*' 0.05 '.' 0.1 ' ' 1
## 
## iterations =  4  
 
 
 
 check assumptions of model 
 
 Cook’s distance 
  plot(m, which = 4, id.n = 3)  
   
 
 
 Extract model results and display data for top 3 values using Cook’s
distance 
  model.data <- augment(m) %>% 
  mutate(index = 1:n()) 
model.data %>% top_n(3, .cooksd)  
 
 
 
 
 
 plot standardised residuals 
  ggplot(model.data, aes(index, .std.resid)) + 
  geom_point(aes(color = Any_Health_Problem), alpha = .5) +
  theme_bw()  
   
 
 
 Filter potential influential data points with abs(.std.res) >
3: 
  model.data %>% 
  filter(abs(.std.resid) > 3)  
 
 
 
 
 
 
 Create ROCR for training and test data 
  ## training data
pred.mtt = predict(m, type = "response") #repeat risk predictions from model m
rocr.pred.mtt = ROCR::prediction(pred.mtt, labels = ml_train$Any_Health_Problem) #ROCR prediction object
roc.perf.mtt = ROCR::performance(rocr.pred.mtt, measure = "tpr", x.measure = "fpr") # #ROCR performance object
plot(roc.perf.mtt, col = "blue")


pred.te.1 = predict(m, newdata = ml_test, type = "response") #.te = "test"
rocr.pred.te.1 = ROCR::prediction(pred.te.1, labels = ml_test$Any_Health_Problem)
roc.perf.te.1 = ROCR::performance(rocr.pred.te.1, measure = "tpr", x.measure = "fpr")
plot(roc.perf.te.1, col = "red", add = T)

abline(a = 0, b = 1, lty = 2) #diagonal for random assignment
legend("bottomright", legend = c("train","test"),
col = c("blue","red"), lty = c(2,1), lwd =1.5)  
   
 
 
 Report AUC from ROC for training and test data 
    # Train AUC
aucTr <- ROCR::performance(rocr.pred.mtt, measure = "auc")
  aucTr <- aucTr@y.values[[1]]
  print(aucTr)  
  ## [1] 0.6423876  
     # Test AUC
  aucTe <- ROCR::performance(rocr.pred.te.1, measure = "auc")
  aucTe <- aucTe@y.values[[1]]
  print(aucTe)  
  ## [1] 0.6336538  
 
 
 
 D_AGE Binary logistic regression for HEALTH - with splines 
  # fit binary logit model and store results 'm'
m <- glm(Any_Health_Problem ~ bs(D_Age,degree=1,df=2), data = ml_train,family = binomial)
# view a summary of the model
summary(m)  
  ## 
## Call:
## glm(formula = Any_Health_Problem ~ bs(D_Age, degree = 1, df = 2), 
##     family = binomial, data = ml_train)
## 
## Coefficients:
##                                Estimate Std. Error z value Pr(>|z|)    
## (Intercept)                     -1.0942     0.1435  -7.626 2.41e-14 ***
## bs(D_Age, degree = 1, df = 2)1   0.3003     0.1924   1.561    0.119    
## bs(D_Age, degree = 1, df = 2)2   2.8299     0.2849   9.932  < 2e-16 ***
## ---
## Signif. codes:  0 '***' 0.001 '**' 0.01 '*' 0.05 '.' 0.1 ' ' 1
## 
## (Dispersion parameter for binomial family taken to be 1)
## 
##     Null deviance: 2200.0  on 1657  degrees of freedom
## Residual deviance: 2094.2  on 1655  degrees of freedom
## AIC: 2100.2
## 
## Number of Fisher Scoring iterations: 4  
  # test model fit
with(m, null.deviance - deviance)  
  ## [1] 105.8707  
  with(m, df.null - df.residual)  
  ## [1] 2  
  with(m, pchisq(null.deviance - deviance, df.null - df.residual, lower.tail = FALSE))  
  ## [1] 1.024413e-23  
  BIC(m)  
  ## [1] 2116.398  
  # Hosmer-Lemeshow Goodness-of-Fit Test
hltest(m)  
  ## 
##    The Hosmer-Lemeshow goodness-of-fit test
## 
##  Group Size Observed Expected
##      1  114       28 28.59532
##      2  177       49 46.94161
##      3  194       54 54.33805
##      4  173       48 51.11783
##      5  173       55 53.86255
##      6  143       53 49.84958
##      7  134       40 51.96523
##      8  130       60 55.70635
##      9  191       95 93.64349
##     10  159      104 93.15238
##     11   70       42 48.82763
## 
##          Statistic =  12.07734 
## degrees of freedom =  9 
##            p-value =  0.20898  
  ## CIs using profiled log-likelihood
confint(m, level=0.99)  
  ## Waiting for profiling to be done...  
  ##                                     0.5 %     99.5 %
## (Intercept)                    -1.4725010 -0.7320867
## bs(D_Age, degree = 1, df = 2)1 -0.1909103  0.8014133
## bs(D_Age, degree = 1, df = 2)2  2.1040198  3.5734357  
  ## CIs using standard errors
confint.default(m, level=0.99)  
  ##                                     0.5 %     99.5 %
## (Intercept)                    -1.4637075 -0.7246085
## bs(D_Age, degree = 1, df = 2)1 -0.1952474  0.7958785
## bs(D_Age, degree = 1, df = 2)2  2.0959351  3.5638072  
  # Wald test
wald.test(b = coef(m), Sigma = vcov(m), Terms = 2)  
  ## Wald test:
## ----------
## 
## Chi-squared test:
## X2 = 2.4, df = 1, P(> X2) = 0.12  
  ## odds ratios and 95% CI
exp(cbind(OR = coef(m), confint(m, level=0.99)))  
  ## Waiting for profiling to be done...  
  ##                                        OR     0.5 %     99.5 %
## (Intercept)                     0.3348214 0.2293512  0.4809045
## bs(D_Age, degree = 1, df = 2)1  1.3502848 0.8262067  2.2286884
## bs(D_Age, degree = 1, df = 2)2 16.9432774 8.1990624 35.6388256  
 
 Where are the knots? 
  ## To see what knots were selected
attr(terms(m), "predvars")  
  ## list(Any_Health_Problem, bs(D_Age, degree = 1L, knots = 5, Boundary.knots = c(1, 
## 20), intercept = FALSE))  
 
 
 Calculate Nagelkerke R^2 
  NagelkerkeR2(m)  
  ## $N
## [1] 1658
## 
## $R2
## [1] 0.08419483  
 
 
 check assumptions of model 
 
 Cook’s distance 
  plot(m, which = 4, id.n = 3)  
   
 
 
 Extract model results and display data for top 3 values using Cook’s
distance 
  model.data <- augment(m) %>% 
  mutate(index = 1:n()) 
model.data %>% top_n(3, .cooksd)  
 
 
 
 
 
 plot standardised residuals 
  ggplot(model.data, aes(index, .std.resid)) + 
  geom_point(aes(color = Any_Health_Problem), alpha = .5) +
  theme_bw()  
   
 
 
 Filter potential influential data points with abs(.std.res) >
3: 
  model.data %>% 
  filter(abs(.std.resid) > 3)  
 
 
 
 
 
 
 Create ROCR for training and test data 
  ## training data
pred.mtt = predict(m, type = "response") #repeat risk predictions from model m
rocr.pred.mtt = ROCR::prediction(pred.mtt, labels = ml_train$Any_Health_Problem) #ROCR prediction object
roc.perf.mtt = ROCR::performance(rocr.pred.mtt, measure = "tpr", x.measure = "fpr") # #ROCR performance object
plot(roc.perf.mtt, col = "blue")


pred.te.1 = predict(m, newdata = ml_test, type = "response") #.te = "test"
rocr.pred.te.1 = ROCR::prediction(pred.te.1, labels = ml_test$Any_Health_Problem)
roc.perf.te.1 = ROCR::performance(rocr.pred.te.1, measure = "tpr", x.measure = "fpr")
plot(roc.perf.te.1, col = "red", add = T)

abline(a = 0, b = 1, lty = 2) #diagonal for random assignment
legend("bottomright", legend = c("train","test"),
col = c("blue","red"), lty = c(2,1), lwd =1.5)  
   
 
 
 Report AUC from ROC for training and test data 
    # Train AUC
aucTr <- ROCR::performance(rocr.pred.mtt, measure = "auc")
  aucTr <- aucTr@y.values[[1]]
  print(aucTr)  
  ## [1] 0.6423876  
     # Test AUC
  aucTe <- ROCR::performance(rocr.pred.te.1, measure = "auc")
  aucTe <- aucTe@y.values[[1]]
  print(aucTe)  
  ## [1] 0.6336538  
 
 
 
 D_Age_quant logistic regression for HEALTH 
 
 Note worse model fit than D-Age 
  # fit binary logit model and store results 'm'
m <- glm(Any_Health_Problem ~ D_Age_quant, data = ml_train,family = binomial)
# view a summary of the model
summary(m)  
  ## 
## Call:
## glm(formula = Any_Health_Problem ~ D_Age_quant, family = binomial, 
##     data = ml_train)
## 
## Coefficients:
##                     Estimate Std. Error z value Pr(>|z|)    
## (Intercept)          -0.9941     0.1023  -9.721  < 2e-16 ***
## D_Age_quant(3,5]      0.1358     0.1558   0.871   0.3836    
## D_Age_quant(5,7]      0.3118     0.1632   1.910   0.0561 .  
## D_Age_quant(7,9.4]    0.8547     0.1671   5.114 3.15e-07 ***
## D_Age_quant(9.4,20]   1.4257     0.1535   9.290  < 2e-16 ***
## ---
## Signif. codes:  0 '***' 0.001 '**' 0.01 '*' 0.05 '.' 0.1 ' ' 1
## 
## (Dispersion parameter for binomial family taken to be 1)
## 
##     Null deviance: 2200.0  on 1657  degrees of freedom
## Residual deviance: 2087.5  on 1653  degrees of freedom
## AIC: 2097.5
## 
## Number of Fisher Scoring iterations: 4  
  # test model fit
with(m, null.deviance - deviance)  
  ## [1] 112.4815  
  with(m, df.null - df.residual)  
  ## [1] 4  
  with(m, pchisq(null.deviance - deviance, df.null - df.residual, lower.tail = FALSE))  
  ## [1] 2.151094e-23  
  BIC(m)  
  ## [1] 2124.614  
  # Hosmer-Lemeshow Goodness-of-Fit Test
hltest(m, G=4)  
  ## 
##    The Hosmer-Lemeshow goodness-of-fit test
## 
##  Group Size Observed Expected
##      1  485      131      131
##      2  346      103      103
##      3  277       93       93
##      4  230      107      107
##      5  320      194      194
## 
##          Statistic =  0 
## degrees of freedom =  3 
##            p-value =  1  
  ## CIs using profiled log-likelihood
confint(m, level=0.99)  
  ## Waiting for profiling to be done...  
  ##                          0.5 %     99.5 %
## (Intercept)         -1.2632612 -0.7356029
## D_Age_quant(3,5]    -0.2674043  0.5364536
## D_Age_quant(5,7]    -0.1108130  0.7313335
## D_Age_quant(7,9.4]   0.4248215  1.2869519
## D_Age_quant(9.4,20]  1.0341194  1.8253916  
  ## CIs using standard errors
confint.default(m, level=0.99)  
  ##                          0.5 %     99.5 %
## (Intercept)         -1.2575206 -0.7306786
## D_Age_quant(3,5]    -0.2656193  0.5371536
## D_Age_quant(5,7]    -0.1087040  0.7322306
## D_Age_quant(7,9.4]   0.4242313  1.2852568
## D_Age_quant(9.4,20]  1.0303924  1.8209593  
  # Wald test
wald.test(b = coef(m), Sigma = vcov(m), Terms = 2)  
  ## Wald test:
## ----------
## 
## Chi-squared test:
## X2 = 0.76, df = 1, P(> X2) = 0.38  
  ## odds ratios and 95% CI
exp(cbind(OR = coef(m), confint(m, level=0.99)))  
  ## Waiting for profiling to be done...  
  ##                            OR     0.5 %    99.5 %
## (Intercept)         0.3700565 0.2827305 0.4792164
## D_Age_quant(3,5]    1.1454151 0.7653636 1.7099319
## D_Age_quant(5,7]    1.3658314 0.8951061 2.0778496
## D_Age_quant(7,9.4]  2.3507727 1.5293174 3.6217305
## D_Age_quant(9.4,20] 4.1606688 2.8126284 6.2052242  
 
 
 Calculate Nagelkerke R^2 
  NagelkerkeR2(m)  
  ## $N
## [1] 1658
## 
## $R2
## [1] 0.08927596  
 
 
 check assumptions of model 
 
 Cook’s distance 
  plot(m, which = 4, id.n = 3)  
   
 
 
 Extract model results and display data for top 3 values using Cook’s
distance 
  model.data <- augment(m) %>% 
  mutate(index = 1:n()) 
model.data %>% top_n(3, .cooksd)  
 
 
 
 
 
 plot standardised residuals 
  ggplot(model.data, aes(index, .std.resid)) + 
  geom_point(aes(color = Any_Health_Problem), alpha = .5) +
  theme_bw()  
   
 
 
 Filter potential influential data points with abs(.std.res) >
3: 
  model.data %>% 
  filter(abs(.std.resid) > 3)  
 
 
 
 
 
 
 Create ROCR for training and test data 
  ## training data
pred.mtt = predict(m, type = "response") #repeat risk predictions from model m
rocr.pred.mtt = ROCR::prediction(pred.mtt, labels = ml_train$Any_Health_Problem) #ROCR prediction object
roc.perf.mtt = ROCR::performance(rocr.pred.mtt, measure = "tpr", x.measure = "fpr") # #ROCR performance object
plot(roc.perf.mtt, col = "blue")


pred.te.1 = predict(m, newdata = ml_test, type = "response") #.te = "test"
rocr.pred.te.1 = ROCR::prediction(pred.te.1, labels = ml_test$Any_Health_Problem)
roc.perf.te.1 = ROCR::performance(rocr.pred.te.1, measure = "tpr", x.measure = "fpr")
plot(roc.perf.te.1, col = "red", add = T)

abline(a = 0, b = 1, lty = 2) #diagonal for random assignment
legend("bottomright", legend = c("train","test"),
col = c("blue","red"), lty = c(2,1), lwd =1.5)  
   
 
 
 Report AUC from ROC for training and test data 
    # Train AUC
aucTr <- ROCR::performance(rocr.pred.mtt, measure = "auc")
  aucTr <- aucTr@y.values[[1]]
  print(aucTr)  
  ## [1] 0.640013  
     # Test AUC
  aucTe <- ROCR::performance(rocr.pred.te.1, measure = "auc")
  aucTe <- aucTe@y.values[[1]]
  print(aucTe)  
  ## [1] 0.6296753  
 
 
 
 DOG SEX binary logistic regression for HEALTH 
  # fit binary logit model and store results 'm'
m <- glm(Any_Health_Problem ~ D_Sex, data = ml_train,family = binomial)
# view a summary of the model
summary(m)  
  ## 
## Call:
## glm(formula = Any_Health_Problem ~ D_Sex, family = binomial, 
##     data = ml_train)
## 
## Coefficients:
##             Estimate Std. Error z value Pr(>|z|)    
## (Intercept) -0.46590    0.07305  -6.378  1.8e-10 ***
## D_SexMale   -0.05540    0.10134  -0.547    0.585    
## ---
## Signif. codes:  0 '***' 0.001 '**' 0.01 '*' 0.05 '.' 0.1 ' ' 1
## 
## (Dispersion parameter for binomial family taken to be 1)
## 
##     Null deviance: 2200.0  on 1657  degrees of freedom
## Residual deviance: 2199.7  on 1656  degrees of freedom
## AIC: 2203.7
## 
## Number of Fisher Scoring iterations: 4  
  # test model fit
with(m, null.deviance - deviance)  
  ## [1] 0.2988056  
  with(m, df.null - df.residual)  
  ## [1] 1  
  with(m, pchisq(null.deviance - deviance, df.null - df.residual, lower.tail = FALSE))  
  ## [1] 0.5846322  
  BIC(m)  
  ## [1] 2214.557  
  ## CIs using profiled log-likelihood
confint(m, level=0.99)  
  ## Waiting for profiling to be done...  
  ##                  0.5 %    99.5 %
## (Intercept) -0.6555562 -0.278944
## D_SexMale   -0.3165198  0.205787  
  ## CIs using standard errors
confint.default(m, level=0.99)  
  ##                  0.5 %     99.5 %
## (Intercept) -0.6540612 -0.2777325
## D_SexMale   -0.3164437  0.2056436  
  # Wald test
wald.test(b = coef(m), Sigma = vcov(m), Terms = 2)  
  ## Wald test:
## ----------
## 
## Chi-squared test:
## X2 = 0.3, df = 1, P(> X2) = 0.58  
  ## odds ratios and 95% CI
exp(cbind(OR = coef(m), confint(m, level=0.99)))  
  ## Waiting for profiling to be done...  
  ##                    OR     0.5 %    99.5 %
## (Intercept) 0.6275720 0.5191532 0.7565822
## D_SexMale   0.9461066 0.7286806 1.2284915  
 
 Calculate Nagelkerke R^2 
  NagelkerkeR2(m)  
  ## $N
## [1] 1658
## 
## $R2
## [1] 0.0002452739  
 
 
 check assumptions of model 
 
 Cook’s distance 
  plot(m, which = 4, id.n = 3)  
   
 
 
 Extract model results and display data for top 3 values using Cook’s
distance 
  model.data <- augment(m) %>% 
  mutate(index = 1:n()) 
model.data %>% top_n(3, .cooksd)  
 
 
 
 
 
 plot standardised residuals 
  ggplot(model.data, aes(index, .std.resid)) + 
  geom_point(aes(color = Any_Health_Problem), alpha = .5) +
  theme_bw()  
   
 
 
 Filter potential influential data points with abs(.std.res) >
3: 
  model.data %>% 
  filter(abs(.std.resid) > 3)  
 
 
 
 
 
 
 Create ROCR for training and test data 
  ## training data
pred.mtt = predict(m, type = "response") #repeat risk predictions from model m
rocr.pred.mtt = ROCR::prediction(pred.mtt, labels = ml_train$Any_Health_Problem) #ROCR prediction object
roc.perf.mtt = ROCR::performance(rocr.pred.mtt, measure = "tpr", x.measure = "fpr") # #ROCR performance object
plot(roc.perf.mtt, col = "blue")


pred.te.1 = predict(m, newdata = ml_test, type = "response") #.te = "test"
rocr.pred.te.1 = ROCR::prediction(pred.te.1, labels = ml_test$Any_Health_Problem)
roc.perf.te.1 = ROCR::performance(rocr.pred.te.1, measure = "tpr", x.measure = "fpr")
plot(roc.perf.te.1, col = "red", add = T)

abline(a = 0, b = 1, lty = 2) #diagonal for random assignment
legend("bottomright", legend = c("train","test"),
col = c("blue","red"), lty = c(2,1), lwd =1.5)  
   
 
 
 Report AUC from ROC for training and test data 
    # Train AUC
aucTr <- ROCR::performance(rocr.pred.mtt, measure = "auc")
  aucTr <- aucTr@y.values[[1]]
  print(aucTr)  
  ## [1] 0.5069121  
     # Test AUC
  aucTe <- ROCR::performance(rocr.pred.te.1, measure = "auc")
  aucTe <- aucTe@y.values[[1]]
  print(aucTe)  
  ## [1] 0.4714535  
 
 
 
 DOG NEUTER binary logistic regression for HEALTH 
  # fit binary logit model and store results 'm'
m <- glm(Any_Health_Problem ~ D_Neuter, data = ml_train,family = binomial)
# view a summary of the model
summary(m)  
  ## 
## Call:
## glm(formula = Any_Health_Problem ~ D_Neuter, family = binomial, 
##     data = ml_train)
## 
## Coefficients:
##                  Estimate Std. Error z value Pr(>|z|)    
## (Intercept)       -1.0771     0.1194  -9.019  < 2e-16 ***
## D_NeuterNeutered   0.7321     0.1321   5.541 3.02e-08 ***
## ---
## Signif. codes:  0 '***' 0.001 '**' 0.01 '*' 0.05 '.' 0.1 ' ' 1
## 
## (Dispersion parameter for binomial family taken to be 1)
## 
##     Null deviance: 2200.0  on 1657  degrees of freedom
## Residual deviance: 2167.2  on 1656  degrees of freedom
## AIC: 2171.2
## 
## Number of Fisher Scoring iterations: 4  
  # test model fit
with(m, null.deviance - deviance)  
  ## [1] 32.85249  
  with(m, df.null - df.residual)  
  ## [1] 1  
  with(m, pchisq(null.deviance - deviance, df.null - df.residual, lower.tail = FALSE))  
  ## [1] 9.942349e-09  
  BIC(m)  
  ## [1] 2182.003  
  ## CIs using profiled log-likelihood
confint(m, level=0.99)  
  ## Waiting for profiling to be done...  
  ##                       0.5 %    99.5 %
## (Intercept)      -1.3931530 -0.776624
## D_NeuterNeutered  0.3974485  1.079281  
  ## CIs using standard errors
confint.default(m, level=0.99)  
  ##                       0.5 %    99.5 %
## (Intercept)      -1.3847151 -0.769497
## D_NeuterNeutered  0.3917453  1.072474  
  # Wald test
wald.test(b = coef(m), Sigma = vcov(m), Terms = 2)  
  ## Wald test:
## ----------
## 
## Chi-squared test:
## X2 = 30.7, df = 1, P(> X2) = 3e-08  
  ## odds ratios and 95% CI
exp(cbind(OR = coef(m), confint(m, level=0.99)))  
  ## Waiting for profiling to be done...  
  ##                         OR     0.5 %    99.5 %
## (Intercept)      0.3405797 0.2482912 0.4599562
## D_NeuterNeutered 2.0794627 1.4880231 2.9425630  
 
 Calculate Nagelkerke R^2 
  NagelkerkeR2(m)  
  ## $N
## [1] 1658
## 
## $R2
## [1] 0.02670389  
 
 
 check assumptions of model 
 
 Cook’s distance 
  plot(m, which = 4, id.n = 3)  
   
 
 
 Extract model results and display data for top 3 values using Cook’s
distance 
  model.data <- augment(m) %>% 
  mutate(index = 1:n()) 
model.data %>% top_n(3, .cooksd)  
 
 
 
 
 
 plot standardised residuals 
  ggplot(model.data, aes(index, .std.resid)) + 
  geom_point(aes(color = Any_Health_Problem), alpha = .5) +
  theme_bw()  
   
 
 
 Filter potential influential data points with abs(.std.res) >
3: 
  model.data %>% 
  filter(abs(.std.resid) > 3)  
 
 
 
 
 
 
 Create ROCR for training and test data 
  ## training data
pred.mtt = predict(m, type = "response") #repeat risk predictions from model m
rocr.pred.mtt = ROCR::prediction(pred.mtt, labels = ml_train$Any_Health_Problem) #ROCR prediction object
roc.perf.mtt = ROCR::performance(rocr.pred.mtt, measure = "tpr", x.measure = "fpr") # #ROCR performance object
plot(roc.perf.mtt, col = "blue")


pred.te.1 = predict(m, newdata = ml_test, type = "response") #.te = "test"
rocr.pred.te.1 = ROCR::prediction(pred.te.1, labels = ml_test$Any_Health_Problem)
roc.perf.te.1 = ROCR::performance(rocr.pred.te.1, measure = "tpr", x.measure = "fpr")
plot(roc.perf.te.1, col = "red", add = T)

abline(a = 0, b = 1, lty = 2) #diagonal for random assignment
legend("bottomright", legend = c("train","test"),
col = c("blue","red"), lty = c(2,1), lwd =1.5)  
   
 
 
 Report AUC from ROC for training and test data 
    # Train AUC
aucTr <- ROCR::performance(rocr.pred.mtt, measure = "auc")
  aucTr <- aucTr@y.values[[1]]
  print(aucTr)  
  ## [1] 0.5591398  
     # Test AUC
  aucTe <- ROCR::performance(rocr.pred.te.1, measure = "auc")
  aucTe <- aucTe@y.values[[1]]
  print(aucTe)  
  ## [1] 0.5216973  
 
 
 
 D_Sex + D_Neuter Binary logistic regression for HEALTH 
  # fit binary logit model and store results 'm'
m <- glm(Any_Health_Problem ~ D_Sex + D_Neuter, data = ml_train,family = binomial)
# view a summary of the model
summary(m)  
  ## 
## Call:
## glm(formula = Any_Health_Problem ~ D_Sex + D_Neuter, family = binomial, 
##     data = ml_train)
## 
## Coefficients:
##                  Estimate Std. Error z value Pr(>|z|)    
## (Intercept)       -1.0672     0.1336  -7.987 1.38e-15 ***
## D_SexMale         -0.0170     0.1025  -0.166    0.868    
## D_NeuterNeutered   0.7307     0.1324   5.519 3.40e-08 ***
## ---
## Signif. codes:  0 '***' 0.001 '**' 0.01 '*' 0.05 '.' 0.1 ' ' 1
## 
## (Dispersion parameter for binomial family taken to be 1)
## 
##     Null deviance: 2200.0  on 1657  degrees of freedom
## Residual deviance: 2167.1  on 1655  degrees of freedom
## AIC: 2173.1
## 
## Number of Fisher Scoring iterations: 4  
  # test model fit
with(m, null.deviance - deviance)  
  ## [1] 32.87998  
  with(m, df.null - df.residual)  
  ## [1] 2  
  with(m, pchisq(null.deviance - deviance, df.null - df.residual, lower.tail = FALSE))  
  ## [1] 7.247745e-08  
  BIC(m)  
  ## [1] 2189.389  
  # Hosmer-Lemeshow Goodness-of-Fit Test
hltest(m)  
  ## 
##    The Hosmer-Lemeshow goodness-of-fit test
## 
##  Group Size Observed  Expected
##      1  217       53  54.84052
##      2  153       41  39.15948
##      3  650      270 268.15948
##      4  638      264 265.84052
## 
##          Statistic =  0.24227 
## degrees of freedom =  2 
##            p-value =  0.88591  
  ## CIs using profiled log-likelihood
confint(m, level=0.99)  
  ## Waiting for profiling to be done...  
  ##                       0.5 %     99.5 %
## (Intercept)      -1.4189268 -0.7294726
## D_SexMale        -0.2809802  0.2473153
## D_NeuterNeutered  0.3953847  1.0785480  
  ## CIs using standard errors
confint.default(m, level=0.99)  
  ##                       0.5 %     99.5 %
## (Intercept)      -1.4113042 -0.7230075
## D_SexMale        -0.2810273  0.2470369
## D_NeuterNeutered  0.3896999  1.0717600  
  # Wald test
wald.test(b = coef(m), Sigma = vcov(m), Terms = 2)  
  ## Wald test:
## ----------
## 
## Chi-squared test:
## X2 = 0.027, df = 1, P(> X2) = 0.87  
  ## odds ratios and 95% CI
exp(cbind(OR = coef(m), confint(m, level=0.99)))  
  ## Waiting for profiling to be done...  
  ##                         OR     0.5 %    99.5 %
## (Intercept)      0.3439855 0.2419736 0.4821632
## D_SexMale        0.9831484 0.7550433 1.2805828
## D_NeuterNeutered 2.0765958 1.4849553 2.9404071  
 
 Calculate Nagelkerke R^2 
  NagelkerkeR2(m)  
  ## $N
## [1] 1658
## 
## $R2
## [1] 0.02672601  
 
 
 check assumptions of model 
 
 Cook’s distance 
  plot(m, which = 4, id.n = 3)  
   
 
 
 Extract model results and display data for top 3 values using Cook’s
distance 
  model.data <- augment(m) %>% 
  mutate(index = 1:n()) 
model.data %>% top_n(3, .cooksd)  
 
 
 
 
 
 plot standardised residuals 
  ggplot(model.data, aes(index, .std.resid)) + 
  geom_point(aes(color = Any_Health_Problem), alpha = .5) +
  theme_bw()  
   
 
 
 Filter potential influential data points with abs(.std.res) >
3: 
  model.data %>% 
  filter(abs(.std.resid) > 3)  
 
 
 
 
 
 check for multicollinearity 
  car::vif(m)  
  ##    D_Sex D_Neuter 
## 1.003902 1.003902  
 
 
 
 Create ROCR for training and test data 
  ## training data
pred.mtt = predict(m, type = "response") #repeat risk predictions from model m
rocr.pred.mtt = ROCR::prediction(pred.mtt, labels = ml_train$Any_Health_Problem) #ROCR prediction object
roc.perf.mtt = ROCR::performance(rocr.pred.mtt, measure = "tpr", x.measure = "fpr") # #ROCR performance object
plot(roc.perf.mtt, col = "blue")


pred.te.1 = predict(m, newdata = ml_test, type = "response") #.te = "test"
rocr.pred.te.1 = ROCR::prediction(pred.te.1, labels = ml_test$Any_Health_Problem)
roc.perf.te.1 = ROCR::performance(rocr.pred.te.1, measure = "tpr", x.measure = "fpr")
plot(roc.perf.te.1, col = "red", add = T)

abline(a = 0, b = 1, lty = 2) #diagonal for random assignment
legend("bottomright", legend = c("train","test"),
col = c("blue","red"), lty = c(2,1), lwd =1.5)  
   
 
 
 Report AUC from ROC for training and test data 
    # Train AUC
aucTr <- ROCR::performance(rocr.pred.mtt, measure = "auc")
  aucTr <- aucTr@y.values[[1]]
  print(aucTr)  
  ## [1] 0.5592388  
     # Test AUC
  aucTe <- ROCR::performance(rocr.pred.te.1, measure = "auc")
  aucTe <- aucTe@y.values[[1]]
  print(aucTe)  
  ## [1] 0.502794  
 
 
 
 D_Sex * D_Neuter Binary logistic regression for HEALTH 
  # fit binary logit model and store results 'm'
m <- glm(Any_Health_Problem ~ D_Sex*D_Neuter, data = ml_train,family = binomial)
# view a summary of the model
summary(m)  
  ## 
## Call:
## glm(formula = Any_Health_Problem ~ D_Sex * D_Neuter, family = binomial, 
##     data = ml_train)
## 
## Coefficients:
##                            Estimate Std. Error z value Pr(>|z|)    
## (Intercept)                 -1.0049     0.1825  -5.505 3.68e-08 ***
## D_SexMale                   -0.1246     0.2414  -0.516 0.605639    
## D_NeuterNeutered             0.6566     0.1995   3.292 0.000994 ***
## D_SexMale:D_NeuterNeutered   0.1312     0.2666   0.492 0.622633    
## ---
## Signif. codes:  0 '***' 0.001 '**' 0.01 '*' 0.05 '.' 0.1 ' ' 1
## 
## (Dispersion parameter for binomial family taken to be 1)
## 
##     Null deviance: 2200.0  on 1657  degrees of freedom
## Residual deviance: 2166.9  on 1654  degrees of freedom
## AIC: 2174.9
## 
## Number of Fisher Scoring iterations: 4  
  # test model fit
with(m, null.deviance - deviance)  
  ## [1] 33.12171  
  with(m, df.null - df.residual)  
  ## [1] 3  
  with(m, pchisq(null.deviance - deviance, df.null - df.residual, lower.tail = FALSE))  
  ## [1] 3.035792e-07  
  BIC(m)  
  ## [1] 2196.56  
  # Hosmer-Lemeshow Goodness-of-Fit Test
hltest(m)  
  ## 
##    The Hosmer-Lemeshow goodness-of-fit test
## 
##  Group Size Observed Expected
##      1  217       53       53
##      2  153       41       41
##      3  638      264      264
##      4  650      270      270
## 
##          Statistic =  0 
## degrees of freedom =  2 
##            p-value =  1  
  ## CIs using profiled log-likelihood
confint(m, level=0.99)  
  ## Waiting for profiling to be done...  
  ##                                 0.5 %     99.5 %
## (Intercept)                -1.4946213 -0.5496257
## D_SexMale                  -0.7449314  0.5032259
## D_NeuterNeutered            0.1551414  1.1865919
## D_SexMale:D_NeuterNeutered -0.5603107  0.8165097  
  ## CIs using standard errors
confint.default(m, level=0.99)  
  ##                                 0.5 %     99.5 %
## (Intercept)                -1.4751039 -0.5347497
## D_SexMale                  -0.7465076  0.4972121
## D_NeuterNeutered            0.1428701  1.1703701
## D_SexMale:D_NeuterNeutered -0.5555381  0.8179483  
  # Wald test
wald.test(b = coef(m), Sigma = vcov(m), Terms = 2)  
  ## Wald test:
## ----------
## 
## Chi-squared test:
## X2 = 0.27, df = 1, P(> X2) = 0.61  
  ## odds ratios and 95% CI
exp(cbind(OR = coef(m), confint(m, level=0.99)))  
  ## Waiting for profiling to be done...  
  ##                                   OR     0.5 %    99.5 %
## (Intercept)                0.3660714 0.2243335 0.5771658
## D_SexMale                  0.8828079 0.4747669 1.6540485
## D_NeuterNeutered           1.9282640 1.1678231 3.2758975
## D_SexMale:D_NeuterNeutered 1.1402016 0.5710316 2.2625888  
 
 Calculate Nagelkerke R^2 
  NagelkerkeR2(m)  
  ## $N
## [1] 1658
## 
## $R2
## [1] 0.02692055  
 
 
 check assumptions of model 
 
 Cook’s distance 
  plot(m, which = 4, id.n = 3)  
   
 
 
 Extract model results and display data for top 3 values using Cook’s
distance 
  model.data <- augment(m) %>% 
  mutate(index = 1:n()) 
model.data %>% top_n(3, .cooksd)  
 
 
 
 
 
 plot standardised residuals 
  ggplot(model.data, aes(index, .std.resid)) + 
  geom_point(aes(color = Any_Health_Problem), alpha = .5) +
  theme_bw()  
   
 
 
 Filter potential influential data points with abs(.std.res) >
3: 
  model.data %>% 
  filter(abs(.std.resid) > 3)  
 
 
 
 
 
 check for multicollinearity 
  car::vif(m)  
  ## there are higher-order terms (interactions) in this model
## consider setting type = 'predictor'; see ?vif  
  ##          D_Sex       D_Neuter D_Sex:D_Neuter 
##       5.569388       2.276983       6.591877  
 
 
 
 Create ROCR for training and test data 
  ## training data
pred.mtt = predict(m, type = "response") #repeat risk predictions from model m
rocr.pred.mtt = ROCR::prediction(pred.mtt, labels = ml_train$Any_Health_Problem) #ROCR prediction object
roc.perf.mtt = ROCR::performance(rocr.pred.mtt, measure = "tpr", x.measure = "fpr") # #ROCR performance object
plot(roc.perf.mtt, col = "blue")


pred.te.1 = predict(m, newdata = ml_test, type = "response") #.te = "test"
rocr.pred.te.1 = ROCR::prediction(pred.te.1, labels = ml_test$Any_Health_Problem)
roc.perf.te.1 = ROCR::performance(rocr.pred.te.1, measure = "tpr", x.measure = "fpr")
plot(roc.perf.te.1, col = "red", add = T)

abline(a = 0, b = 1, lty = 2) #diagonal for random assignment
legend("bottomright", legend = c("train","test"),
col = c("blue","red"), lty = c(2,1), lwd =1.5)  
   
 
 
 Report AUC from ROC for training and test data 
    # Train AUC
aucTr <- ROCR::performance(rocr.pred.mtt, measure = "auc")
  aucTr <- aucTr@y.values[[1]]
  print(aucTr)  
  ## [1] 0.5602591  
     # Test AUC
  aucTe <- ROCR::performance(rocr.pred.te.1, measure = "auc")
  aucTe <- aucTe@y.values[[1]]
  print(aucTe)  
  ## [1] 0.5361134  
 
 
 
 
 CHECK EFFECT OF DOG HEALTH CHARACTERISTICS - simple binary
regression 
 
 THERAPEUTIC FOOD Binary logistic regression for HEALTH 
  # fit binary logit model and store results 'm'
m <- glm(Any_Health_Problem ~ Therapeutic_Food, data = ml_train,family = binomial)
# view a summary of the model
summary(m)  
  ## 
## Call:
## glm(formula = Any_Health_Problem ~ Therapeutic_Food, family = binomial, 
##     data = ml_train)
## 
## Coefficients:
##                     Estimate Std. Error z value Pr(>|z|)    
## (Intercept)         -0.57701    0.05234 -11.024  < 2e-16 ***
## Therapeutic_FoodYes  1.92940    0.29436   6.555 5.58e-11 ***
## ---
## Signif. codes:  0 '***' 0.001 '**' 0.01 '*' 0.05 '.' 0.1 ' ' 1
## 
## (Dispersion parameter for binomial family taken to be 1)
## 
##     Null deviance: 2200.0  on 1657  degrees of freedom
## Residual deviance: 2144.8  on 1656  degrees of freedom
## AIC: 2148.8
## 
## Number of Fisher Scoring iterations: 4  
  # test model fit
with(m, null.deviance - deviance)  
  ## [1] 55.23019  
  with(m, df.null - df.residual)  
  ## [1] 1  
  with(m, pchisq(null.deviance - deviance, df.null - df.residual, lower.tail = FALSE))  
  ## [1] 1.072093e-13  
  BIC(m)  
  ## [1] 2159.625  
  ## CIs using profiled log-likelihood
confint(m, level=0.99)  
  ## Waiting for profiling to be done...  
  ##                          0.5 %     99.5 %
## (Intercept)         -0.7127343 -0.4429849
## Therapeutic_FoodYes  1.2154551  2.7509045  
  ## CIs using standard errors
confint.default(m, level=0.99)  
  ##                          0.5 %     99.5 %
## (Intercept)         -0.7118297 -0.4421854
## Therapeutic_FoodYes  1.1711802  2.6876205  
  # Wald test
wald.test(b = coef(m), Sigma = vcov(m), Terms = 2)  
  ## Wald test:
## ----------
## 
## Chi-squared test:
## X2 = 43.0, df = 1, P(> X2) = 5.6e-11  
  ## odds ratios and 95% CI
exp(cbind(OR = coef(m), confint(m, level=0.99)))  
  ## Waiting for profiling to be done...  
  ##                            OR     0.5 %     99.5 %
## (Intercept)         0.5615764 0.4903017  0.6421169
## Therapeutic_FoodYes 6.8853801 3.3718283 15.6567867  
 
 Calculate Nagelkerke R^2 
  NagelkerkeR2(m)  
  ## $N
## [1] 1658
## 
## $R2
## [1] 0.04459282  
 
 
 check assumptions of model 
 
 Cook’s distance 
  plot(m, which = 4, id.n = 3)  
   
 
 
 Extract model results and display data for top 3 values using Cook’s
distance 
  model.data <- augment(m) %>% 
  mutate(index = 1:n()) 
model.data %>% top_n(3, .cooksd)  
 
 
 
 
 
 plot standardised residuals 
  ggplot(model.data, aes(index, .std.resid)) + 
  geom_point(aes(color = Any_Health_Problem), alpha = .5) +
  theme_bw()  
   
 
 
 Filter potential influential data points with abs(.std.res) >
3: 
  model.data %>% 
  filter(abs(.std.resid) > 3)  
 
 
 
 
 
 
 Create ROCR for training and test data 
  ## training data
pred.mtt = predict(m, type = "response") #repeat risk predictions from model m
rocr.pred.mtt = ROCR::prediction(pred.mtt, labels = ml_train$Any_Health_Problem) #ROCR prediction object
roc.perf.mtt = ROCR::performance(rocr.pred.mtt, measure = "tpr", x.measure = "fpr") # #ROCR performance object
plot(roc.perf.mtt, col = "blue")


pred.te.1 = predict(m, newdata = ml_test, type = "response") #.te = "test"
rocr.pred.te.1 = ROCR::prediction(pred.te.1, labels = ml_test$Any_Health_Problem)
roc.perf.te.1 = ROCR::performance(rocr.pred.te.1, measure = "tpr", x.measure = "fpr")
plot(roc.perf.te.1, col = "red", add = T)

abline(a = 0, b = 1, lty = 2) #diagonal for random assignment
legend("bottomright", legend = c("train","test"),
col = c("blue","red"), lty = c(2,1), lwd =1.5)  
   
 
 
 Report AUC from ROC for training and test data 
    # Train AUC
aucTr <- ROCR::performance(rocr.pred.mtt, measure = "auc")
  aucTr <- aucTr@y.values[[1]]
  print(aucTr)  
  ## [1] 0.5388968  
     # Test AUC
  aucTe <- ROCR::performance(rocr.pred.te.1, measure = "auc")
  aucTe <- aucTe@y.values[[1]]
  print(aucTe)  
  ## [1] 0.5417851  
 
 
 
 VISITS Binary logistic regression for HEALTH 
  # fit binary logit model and store results 'm'
m <- glm(Any_Health_Problem ~ Visits, data = ml_train,family = binomial)
# view a summary of the model
summary(m)  
  ## 
## Call:
## glm(formula = Any_Health_Problem ~ Visits, family = binomial, 
##     data = ml_train)
## 
## Coefficients:
##             Estimate Std. Error z value Pr(>|z|)    
## (Intercept)  -1.7610     0.1632 -10.791  < 2e-16 ***
## Visits1       0.6361     0.1864   3.412 0.000645 ***
## Visits2       1.9373     0.1942   9.976  < 2e-16 ***
## Visits3       2.0874     0.2384   8.756  < 2e-16 ***
## Visits3<      2.8390     0.2323  12.219  < 2e-16 ***
## ---
## Signif. codes:  0 '***' 0.001 '**' 0.01 '*' 0.05 '.' 0.1 ' ' 1
## 
## (Dispersion parameter for binomial family taken to be 1)
## 
##     Null deviance: 2200.0  on 1657  degrees of freedom
## Residual deviance: 1896.3  on 1653  degrees of freedom
## AIC: 1906.3
## 
## Number of Fisher Scoring iterations: 4  
  # test model fit
with(m, null.deviance - deviance)  
  ## [1] 303.7694  
  with(m, df.null - df.residual)  
  ## [1] 4  
  with(m, pchisq(null.deviance - deviance, df.null - df.residual, lower.tail = FALSE))  
  ## [1] 1.666039e-64  
  BIC(m)  
  ## [1] 1933.326  
  # Hosmer-Lemeshow Goodness-of-Fit Test
hltest(m)  
  ## 
##    The Hosmer-Lemeshow goodness-of-fit test
## 
##  Group Size Observed Expected
##      1  300       44       44
##      2  665      163      163
##      3  364      198      198
##      4  136       79       79
##      5  193      144      144
## 
##          Statistic =  0 
## degrees of freedom =  3 
##            p-value =  1  
  ## CIs using profiled log-likelihood
confint(m, level=0.99)  
  ## Waiting for profiling to be done...  
  ##                  0.5 %    99.5 %
## (Intercept) -2.2040828 -1.359713
## Visits1      0.1691605  1.133095
## Visits2      1.4507370  2.454044
## Visits3      1.4844532  2.715634
## Visits3<     2.2570716  3.456616  
  ## CIs using standard errors
confint.default(m, level=0.99)  
  ##                  0.5 %    99.5 %
## (Intercept) -2.1813575 -1.340618
## Visits1      0.1558959  1.116380
## Visits2      1.4370776  2.437457
## Visits3      1.4733010  2.701468
## Visits3<     2.2404893  3.437472  
  # Wald test
wald.test(b = coef(m), Sigma = vcov(m), Terms = 2)  
  ## Wald test:
## ----------
## 
## Chi-squared test:
## X2 = 11.6, df = 1, P(> X2) = 0.00064  
  ## odds ratios and 95% CI
exp(cbind(OR = coef(m), confint(m, level=0.99)))  
  ## Waiting for profiling to be done...  
  ##                    OR     0.5 %     99.5 %
## (Intercept)  0.171875 0.1103517  0.2567345
## Visits1      1.889171 1.1843102  3.1052535
## Visits2      6.939759 4.2662576 11.6353015
## Visits3      8.063796 4.4125518 15.1141860
## Visits3<    17.098330 9.5550672 31.7094759  
 
 Calculate Nagelkerke R^2 
  NagelkerkeR2(m)  
  ## $N
## [1] 1658
## 
## $R2
## [1] 0.2278602  
 
 
 check assumptions of model 
 
 Cook’s distance 
  plot(m, which = 4, id.n = 3)  
   
 
 
 Extract model results and display data for top 3 values using Cook’s
distance 
  model.data <- augment(m) %>% 
  mutate(index = 1:n()) 
model.data %>% top_n(3, .cooksd)  
 
 
 
 
 
 plot standardised residuals 
  ggplot(model.data, aes(index, .std.resid)) + 
  geom_point(aes(color = Any_Health_Problem), alpha = .5) +
  theme_bw()  
   
 
 
 Filter potential influential data points with abs(.std.res) >
3: 
  model.data %>% 
  filter(abs(.std.resid) > 3)  
 
 
 
 
 
 
 Create ROCR for training and test data 
  ## training data
pred.mtt = predict(m, type = "response") #repeat risk predictions from model m
rocr.pred.mtt = ROCR::prediction(pred.mtt, labels = ml_train$Any_Health_Problem) #ROCR prediction object
roc.perf.mtt = ROCR::performance(rocr.pred.mtt, measure = "tpr", x.measure = "fpr") # #ROCR performance object
plot(roc.perf.mtt, col = "blue")


pred.te.1 = predict(m, newdata = ml_test, type = "response") #.te = "test"
rocr.pred.te.1 = ROCR::prediction(pred.te.1, labels = ml_test$Any_Health_Problem)
roc.perf.te.1 = ROCR::performance(rocr.pred.te.1, measure = "tpr", x.measure = "fpr")
plot(roc.perf.te.1, col = "red", add = T)

abline(a = 0, b = 1, lty = 2) #diagonal for random assignment
legend("bottomright", legend = c("train","test"),
col = c("blue","red"), lty = c(2,1), lwd =1.5)  
   
 
 
 Report AUC from ROC for training and test data 
    # Train AUC
aucTr <- ROCR::performance(rocr.pred.mtt, measure = "auc")
  aucTr <- aucTr@y.values[[1]]
  print(aucTr)  
  ## [1] 0.7340772  
     # Test AUC
  aucTe <- ROCR::performance(rocr.pred.te.1, measure = "auc")
  aucTe <- aucTe@y.values[[1]]
  print(aucTe)  
  ## [1] 0.7696976  
 
 
 
 VISITS2 Binary logistic regression for HEALTH 
 
 Note Visits better fit than Visits2 
  # fit binary logit model and store results 'm'
m <- glm(Any_Health_Problem ~ Visits2, data = ml_train,family = binomial)
# view a summary of the model
summary(m)  
  ## 
## Call:
## glm(formula = Any_Health_Problem ~ Visits2, family = binomial, 
##     data = ml_train)
## 
## Coefficients:
##             Estimate Std. Error z value Pr(>|z|)    
## (Intercept)  -1.7610     0.1632 -10.791  < 2e-16 ***
## Visits21v     0.6361     0.1864   3.412 0.000645 ***
## Visits22v     1.9373     0.1942   9.976  < 2e-16 ***
## Visits23v     2.5047     0.2014  12.438  < 2e-16 ***
## ---
## Signif. codes:  0 '***' 0.001 '**' 0.01 '*' 0.05 '.' 0.1 ' ' 1
## 
## (Dispersion parameter for binomial family taken to be 1)
## 
##     Null deviance: 2200.0  on 1657  degrees of freedom
## Residual deviance: 1906.2  on 1654  degrees of freedom
## AIC: 1914.2
## 
## Number of Fisher Scoring iterations: 4  
  # test model fit
with(m, null.deviance - deviance)  
  ## [1] 293.8664  
  with(m, df.null - df.residual)  
  ## [1] 3  
  with(m, pchisq(null.deviance - deviance, df.null - df.residual, lower.tail = FALSE))  
  ## [1] 2.114523e-63  
  BIC(m)  
  ## [1] 1935.816  
  # Hosmer-Lemeshow Goodness-of-Fit Test
hltest(m)  
  ## 
##    The Hosmer-Lemeshow goodness-of-fit test
## 
##  Group Size Observed Expected
##      1  300       44       44
##      2  665      163      163
##      3  364      198      198
##      4  329      223      223
## 
##          Statistic =  0 
## degrees of freedom =  2 
##            p-value =  1  
  ## CIs using profiled log-likelihood
confint(m, level=0.99)  
  ## Waiting for profiling to be done...  
  ##                  0.5 %    99.5 %
## (Intercept) -2.2040828 -1.359713
## Visits21v    0.1691605  1.133095
## Visits22v    1.4507370  2.454044
## Visits23v    2.0003620  3.040357  
  ## CIs using standard errors
confint.default(m, level=0.99)  
  ##                  0.5 %    99.5 %
## (Intercept) -2.1813575 -1.340618
## Visits21v    0.1558959  1.116380
## Visits22v    1.4370776  2.437457
## Visits23v    1.9860136  3.023427  
  # Wald test
wald.test(b = coef(m), Sigma = vcov(m), Terms = 2)  
  ## Wald test:
## ----------
## 
## Chi-squared test:
## X2 = 11.6, df = 1, P(> X2) = 0.00064  
  ## odds ratios and 95% CI
exp(cbind(OR = coef(m), confint(m, level=0.99)))  
  ## Waiting for profiling to be done...  
  ##                    OR     0.5 %     99.5 %
## (Intercept)  0.171875 0.1103517  0.2567345
## Visits21v    1.889171 1.1843102  3.1052535
## Visits22v    6.939759 4.2662576 11.6353015
## Visits23v   12.240137 7.3917317 20.9126977  
 
 
 Calculate Nagelkerke R^2 
  NagelkerkeR2(m)  
  ## $N
## [1] 1658
## 
## $R2
## [1] 0.2210714  
 
 
 check assumptions of model 
 
 Cook’s distance 
  plot(m, which = 4, id.n = 3)  
   
 
 
 Extract model results and display data for top 3 values using Cook’s
distance 
  model.data <- augment(m) %>% 
  mutate(index = 1:n()) 
model.data %>% top_n(3, .cooksd)  
 
 
 
 
 
 plot standardised residuals 
  ggplot(model.data, aes(index, .std.resid)) + 
  geom_point(aes(color = Any_Health_Problem), alpha = .5) +
  theme_bw()  
   
 
 
 Filter potential influential data points with abs(.std.res) >
3: 
  model.data %>% 
  filter(abs(.std.resid) > 3)  
 
 
 
 
 
 
 Create ROCR for training and test data 
  ## training data
pred.mtt = predict(m, type = "response") #repeat risk predictions from model m
rocr.pred.mtt = ROCR::prediction(pred.mtt, labels = ml_train$Any_Health_Problem) #ROCR prediction object
roc.perf.mtt = ROCR::performance(rocr.pred.mtt, measure = "tpr", x.measure = "fpr") # #ROCR performance object
plot(roc.perf.mtt, col = "blue")


pred.te.1 = predict(m, newdata = ml_test, type = "response") #.te = "test"
rocr.pred.te.1 = ROCR::prediction(pred.te.1, labels = ml_test$Any_Health_Problem)
roc.perf.te.1 = ROCR::performance(rocr.pred.te.1, measure = "tpr", x.measure = "fpr")
plot(roc.perf.te.1, col = "red", add = T)

abline(a = 0, b = 1, lty = 2) #diagonal for random assignment
legend("bottomright", legend = c("train","test"),
col = c("blue","red"), lty = c(2,1), lwd =1.5)  
   
 
 
 Report AUC from ROC for training and test data 
    # Train AUC
aucTr <- ROCR::performance(rocr.pred.mtt, measure = "auc")
  aucTr <- aucTr@y.values[[1]]
  print(aucTr)  
  ## [1] 0.7307248  
     # Test AUC
  aucTe <- ROCR::performance(rocr.pred.te.1, measure = "auc")
  aucTe <- aucTe@y.values[[1]]
  print(aucTe)  
  ## [1] 0.765078  
 
 
 
 MEDS Binary logistic regression for HEALTH 
  # fit binary logit model and store results 'm'
m <- glm(Any_Health_Problem ~ Meds, data = ml_train,family = binomial)
# view a summary of the model
summary(m)  
  ## 
## Call:
## glm(formula = Any_Health_Problem ~ Meds, family = binomial, data = ml_train)
## 
## Coefficients:
##             Estimate Std. Error z value Pr(>|z|)    
## (Intercept) -1.52243    0.08227  -18.51   <2e-16 ***
## MedsYes      2.30421    0.11780   19.56   <2e-16 ***
## ---
## Signif. codes:  0 '***' 0.001 '**' 0.01 '*' 0.05 '.' 0.1 ' ' 1
## 
## (Dispersion parameter for binomial family taken to be 1)
## 
##     Null deviance: 2200.0  on 1657  degrees of freedom
## Residual deviance: 1757.4  on 1656  degrees of freedom
## AIC: 1761.4
## 
## Number of Fisher Scoring iterations: 4  
  # test model fit
with(m, null.deviance - deviance)  
  ## [1] 442.6517  
  with(m, df.null - df.residual)  
  ## [1] 1  
  with(m, pchisq(null.deviance - deviance, df.null - df.residual, lower.tail = FALSE))  
  ## [1] 2.866393e-98  
  BIC(m)  
  ## [1] 1772.204  
  # Hosmer-Lemeshow Goodness-of-Fit Test
## CIs using profiled log-likelihood
confint(m, level=0.99)  
  ## Waiting for profiling to be done...  
  ##                 0.5 %    99.5 %
## (Intercept) -1.739368 -1.315112
## MedsYes      2.004450  2.611667  
  ## CIs using standard errors
confint.default(m, level=0.99)  
  ##                 0.5 %    99.5 %
## (Intercept) -1.734329 -1.310524
## MedsYes      2.000766  2.607654  
  # Wald test
wald.test(b = coef(m), Sigma = vcov(m), Terms = 2)  
  ## Wald test:
## ----------
## 
## Chi-squared test:
## X2 = 382.6, df = 1, P(> X2) = 0.0  
  ## odds ratios and 95% CI
exp(cbind(OR = coef(m), confint(m, level=0.99)))  
  ## Waiting for profiling to be done...  
  ##                     OR     0.5 %     99.5 %
## (Intercept)  0.2181818 0.1756314  0.2684443
## MedsYes     10.0162602 7.4220070 13.6217365  
 
 Calculate Nagelkerke R^2 
  NagelkerkeR2(m)  
  ## $N
## [1] 1658
## 
## $R2
## [1] 0.3189181  
 
 
 check assumptions of model 
 
 Cook’s distance 
  plot(m, which = 4, id.n = 3)  
   
 
 
 Extract model results and display data for top 3 values using Cook’s
distance 
  model.data <- augment(m) %>% 
  mutate(index = 1:n()) 
model.data %>% top_n(3, .cooksd)  
 
 
 
 
 
 plot standardised residuals 
  ggplot(model.data, aes(index, .std.resid)) + 
  geom_point(aes(color = Any_Health_Problem), alpha = .5) +
  theme_bw()  
   
 
 
 
 
 
 Filter potential influential data points with abs(.std.res) >
3: 
  model.data %>% 
  filter(abs(.std.resid) > 3)  
 
 
 
 
 Create ROCR for training and test data 
  ## training data
pred.mtt = predict(m, type = "response") #repeat risk predictions from model m
rocr.pred.mtt = ROCR::prediction(pred.mtt, labels = ml_train$Any_Health_Problem) #ROCR prediction object
roc.perf.mtt = ROCR::performance(rocr.pred.mtt, measure = "tpr", x.measure = "fpr") # #ROCR performance object
plot(roc.perf.mtt, col = "blue")


pred.te.1 = predict(m, newdata = ml_test, type = "response") #.te = "test"
rocr.pred.te.1 = ROCR::prediction(pred.te.1, labels = ml_test$Any_Health_Problem)
roc.perf.te.1 = ROCR::performance(rocr.pred.te.1, measure = "tpr", x.measure = "fpr")
plot(roc.perf.te.1, col = "red", add = T)

abline(a = 0, b = 1, lty = 2) #diagonal for random assignment
legend("bottomright", legend = c("train","test"),
col = c("blue","red"), lty = c(2,1), lwd =1.5)  
   
 
 
 Report AUC from ROC for training and test data 
    # Train AUC
aucTr <- ROCR::performance(rocr.pred.mtt, measure = "auc")
  aucTr <- aucTr@y.values[[1]]
  print(aucTr)  
  ## [1] 0.7571733  
     # Test AUC
  aucTe <- ROCR::performance(rocr.pred.te.1, measure = "auc")
  aucTe <- aucTe@y.values[[1]]
  print(aucTe)  
  ## [1] 0.7682483  
 
 
 
 MULTIPLE REGRESSION WITH BACKWARDS ELIMINATION 
 
 ROUND 1: Model with all variables 
 
 D_Age included as continuous and Animal Career as a binary 
 
 
 D_Size included as factor not ordered 
  # fit binary logit model and store results 'm'
m <- glm(Any_Health_Problem ~  Location + Urban + Education_S + Animal_Career_BINARY 
         + Income2 + C_Age2 + C_Gender + C_Diet + Size2 + D_Sex + D_Neuter  + D_Diet + Therapeutic_Food + Visits 
         + Meds + bs(D_Age,degree=1,df=2), data = ml_train,family = binomial)
# view a summary of the model
summary(m)  
  ## 
## Call:
## glm(formula = Any_Health_Problem ~ Location + Urban + Education_S + 
##     Animal_Career_BINARY + Income2 + C_Age2 + C_Gender + C_Diet + 
##     Size2 + D_Sex + D_Neuter + D_Diet + Therapeutic_Food + Visits + 
##     Meds + bs(D_Age, degree = 1, df = 2), family = binomial, 
##     data = ml_train)
## 
## Coefficients:
##                                                         Estimate Std. Error
## (Intercept)                                             -2.39957    0.37442
## LocationOther European                                   0.19675    0.22344
## LocationNorth America                                    0.08156    0.27319
## LocationAustralia/New Zealand/Oceania                    0.20214    0.32593
## LocationOther                                            0.19928    0.40893
## UrbanYes                                                 0.04375    0.13918
## Education_S1_College                                     0.07988    0.19485
## Education_S2_Grad                                       -0.11565    0.19806
## Education_S3_PG_or_PhD                                  -0.33582    0.21181
## Animal_Career_BINARYYes                                 -0.19537    0.16773
## Income2Medium                                           -0.14742    0.17852
## Income2High                                             -0.40230    0.24428
## C_Age230–39                                             -0.14607    0.20797
## C_Age240–49                                             -0.27724    0.21507
## C_Age250–59                                             -0.57275    0.21658
## C_Age260<                                               -0.45710    0.23249
## C_GenderMale                                            -0.35129    0.26208
## C_DietOmnivore reducing animal product consumption       0.04701    0.17029
## C_DietPescatarian (including fish but no other meats)    0.40571    0.28809
## C_DietVegan (consuming no animal products)              -0.16492    0.23577
## C_DietVegetarian (consuming plants, eggs and milk)      -0.10366    0.22716
## Size2Toy                                                 0.45581    0.42127
## Size2Small                                               0.14378    0.17632
## Size2Large                                               0.09640    0.15084
## Size2Giant                                               0.72541    0.31004
## D_SexMale                                               -0.08397    0.12928
## D_NeuterNeutered                                         0.05776    0.16957
## D_DietMeat-based – raw                                  -0.12326    0.14605
## D_DietVegan (consuming no animal products)              -0.46503    0.27220
## D_DietVegetarian (including eggs or milk, but not meat) -0.23490    0.51801
## Therapeutic_FoodYes                                      0.87672    0.35990
## Visits1                                                  0.15278    0.20763
## Visits2                                                  1.04364    0.22986
## Visits3                                                  0.94397    0.28732
## Visits3<                                                 1.53993    0.28362
## MedsYes                                                  1.84168    0.14426
## bs(D_Age, degree = 1, df = 2)1                           0.81300    0.24358
## bs(D_Age, degree = 1, df = 2)2                           3.49739    0.37527
##                                                         z value Pr(>|z|)    
## (Intercept)                                              -6.409 1.47e-10 ***
## LocationOther European                                    0.881 0.378561    
## LocationNorth America                                     0.299 0.765283    
## LocationAustralia/New Zealand/Oceania                     0.620 0.535135    
## LocationOther                                             0.487 0.626033    
## UrbanYes                                                  0.314 0.753273    
## Education_S1_College                                      0.410 0.681836    
## Education_S2_Grad                                        -0.584 0.559293    
## Education_S3_PG_or_PhD                                   -1.585 0.112857    
## Animal_Career_BINARYYes                                  -1.165 0.244115    
## Income2Medium                                            -0.826 0.408912    
## Income2High                                              -1.647 0.099580 .  
## C_Age230–39                                              -0.702 0.482449    
## C_Age240–49                                              -1.289 0.197373    
## C_Age250–59                                              -2.645 0.008181 ** 
## C_Age260<                                                -1.966 0.049291 *  
## C_GenderMale                                             -1.340 0.180122    
## C_DietOmnivore reducing animal product consumption        0.276 0.782503    
## C_DietPescatarian (including fish but no other meats)     1.408 0.159056    
## C_DietVegan (consuming no animal products)               -0.699 0.484252    
## C_DietVegetarian (consuming plants, eggs and milk)       -0.456 0.648173    
## Size2Toy                                                  1.082 0.279261    
## Size2Small                                                0.815 0.414803    
## Size2Large                                                0.639 0.522786    
## Size2Giant                                                2.340 0.019296 *  
## D_SexMale                                                -0.650 0.516000    
## D_NeuterNeutered                                          0.341 0.733394    
## D_DietMeat-based – raw                                   -0.844 0.398707    
## D_DietVegan (consuming no animal products)               -1.708 0.087557 .  
## D_DietVegetarian (including eggs or milk, but not meat)  -0.453 0.650216    
## Therapeutic_FoodYes                                       2.436 0.014849 *  
## Visits1                                                   0.736 0.461842    
## Visits2                                                   4.540 5.62e-06 ***
## Visits3                                                   3.285 0.001018 ** 
## Visits3<                                                  5.430 5.65e-08 ***
## MedsYes                                                  12.767  < 2e-16 ***
## bs(D_Age, degree = 1, df = 2)1                            3.338 0.000845 ***
## bs(D_Age, degree = 1, df = 2)2                            9.320  < 2e-16 ***
## ---
## Signif. codes:  0 '***' 0.001 '**' 0.01 '*' 0.05 '.' 0.1 ' ' 1
## 
## (Dispersion parameter for binomial family taken to be 1)
## 
##     Null deviance: 2200.0  on 1657  degrees of freedom
## Residual deviance: 1553.9  on 1620  degrees of freedom
## AIC: 1629.9
## 
## Number of Fisher Scoring iterations: 4  
  # test model fit
with(m, null.deviance - deviance)  
  ## [1] 646.1275  
  with(m, df.null - df.residual)  
  ## [1] 37  
  with(m, pchisq(null.deviance - deviance, df.null - df.residual, lower.tail = FALSE))  
  ## [1] 2.858622e-112  
  BIC(m)  
  ## [1] 1835.609  
  # Hosmer-Lemeshow Goodness-of-Fit Test
hltest(m)  
  ## 
##    The Hosmer-Lemeshow goodness-of-fit test
## 
##  Group Size Observed   Expected
##      1  166       10   9.628249
##      2  166       19  14.735388
##      3  166       19  20.308452
##      4  166       27  26.891500
##      5  166       40  37.332721
##      6  166       55  55.156303
##      7  166       66  82.080121
##      8  166      111 106.255022
##      9  166      131 127.785132
##     10  164      150 147.827113
## 
##          Statistic =  9.20813 
## degrees of freedom =  8 
##            p-value =  0.32504  
  ## CIs using profiled log-likelihood
confint(m, level=0.99)  
  ## Waiting for profiling to be done...  
  ##                                                                0.5 %
## (Intercept)                                             -3.379491383
## LocationOther European                                  -0.379000052
## LocationNorth America                                   -0.631058660
## LocationAustralia/New Zealand/Oceania                   -0.641778380
## LocationOther                                           -0.874083059
## UrbanYes                                                -0.316238689
## Education_S1_College                                    -0.420739835
## Education_S2_Grad                                       -0.625251044
## Education_S3_PG_or_PhD                                  -0.882629940
## Animal_Career_BINARYYes                                 -0.631309921
## Income2Medium                                           -0.605431581
## Income2High                                             -1.034440917
## C_Age230–39                                             -0.682484619
## C_Age240–49                                             -0.832706899
## C_Age250–59                                             -1.133281066
## C_Age260<                                               -1.058636854
## C_GenderMale                                            -1.046912969
## C_DietOmnivore reducing animal product consumption      -0.393796364
## C_DietPescatarian (including fish but no other meats)   -0.337363299
## C_DietVegan (consuming no animal products)              -0.777760183
## C_DietVegetarian (consuming plants, eggs and milk)      -0.694486751
## Size2Toy                                                -0.674265101
## Size2Small                                              -0.312085256
## Size2Large                                              -0.292702959
## Size2Giant                                              -0.084933633
## D_SexMale                                               -0.417533708
## D_NeuterNeutered                                        -0.376423443
## D_DietMeat-based – raw                                  -0.499831338
## D_DietVegan (consuming no animal products)              -1.170809089
## D_DietVegetarian (including eggs or milk, but not meat) -1.604029257
## Therapeutic_FoodYes                                     -0.009091493
## Visits1                                                 -0.372647645
## Visits2                                                  0.459495321
## Visits3                                                  0.209594404
## Visits3<                                                 0.819306658
## MedsYes                                                  1.474009214
## bs(D_Age, degree = 1, df = 2)1                           0.191311875
## bs(D_Age, degree = 1, df = 2)2                           2.543044814
##                                                              99.5 %
## (Intercept)                                             -1.44757246
## LocationOther European                                   0.77384075
## LocationNorth America                                    0.78056520
## LocationAustralia/New Zealand/Oceania                    1.04240821
## LocationOther                                            1.24126811
## UrbanYes                                                 0.40155196
## Education_S1_College                                     0.58444767
## Education_S2_Grad                                        0.39650422
## Education_S3_PG_or_PhD                                   0.20998343
## Animal_Career_BINARYYes                                  0.23392726
## Income2Medium                                            0.31559867
## Income2High                                              0.22576963
## C_Age230–39                                              0.39028323
## C_Age240–49                                              0.27668551
## C_Age250–59                                             -0.01601719
## C_Age260<                                                0.14070941
## C_GenderMale                                             0.30820716
## C_DietOmnivore reducing animal product consumption       0.48459266
## C_DietPescatarian (including fish but no other meats)    1.15000258
## C_DietVegan (consuming no animal products)               0.43870221
## C_DietVegetarian (consuming plants, eggs and milk)       0.47790283
## Size2Toy                                                 1.51288373
## Size2Small                                               0.59740350
## Size2Large                                               0.48527932
## Size2Giant                                               1.51787229
## D_SexMale                                                0.24918477
## D_NeuterNeutered                                         0.49850484
## D_DietMeat-based – raw                                   0.25345814
## D_DietVegan (consuming no animal products)               0.23394252
## D_DietVegetarian (including eggs or milk, but not meat)  1.09504821
## Therapeutic_FoodYes                                      1.85726710
## Visits1                                                  0.70048062
## Visits2                                                  1.64664414
## Visits3                                                  1.69279474
## Visits3<                                                 2.28316784
## MedsYes                                                  2.21800969
## bs(D_Age, degree = 1, df = 2)1                           1.44781493
## bs(D_Age, degree = 1, df = 2)2                           4.47911007  
  ## CIs using standard errors
confint.default(m, level=0.99)  
  ##                                                               0.5 %      99.5 %
## (Intercept)                                             -3.36402444 -1.43512056
## LocationOther European                                  -0.37879654  0.77230102
## LocationNorth America                                   -0.62212707  0.78524832
## LocationAustralia/New Zealand/Oceania                   -0.63740020  1.04167353
## LocationOther                                           -0.85405258  1.25260964
## UrbanYes                                                -0.31474837  0.40224234
## Education_S1_College                                    -0.42202335  0.58178578
## Education_S2_Grad                                       -0.62582389  0.39452909
## Education_S3_PG_or_PhD                                  -0.88141739  0.20976746
## Animal_Career_BINARYYes                                 -0.62742708  0.23668502
## Income2Medium                                           -0.60725616  0.31241118
## Income2High                                             -1.03152962  0.22692158
## C_Age230–39                                             -0.68175630  0.38961763
## C_Age240–49                                             -0.83121259  0.27673870
## C_Age250–59                                             -1.13063645 -0.01487351
## C_Age260<                                               -1.05596173  0.14176566
## C_GenderMale                                            -1.02636825  0.32378944
## C_DietOmnivore reducing animal product consumption      -0.39163864  0.48566113
## C_DietPescatarian (including fish but no other meats)   -0.33637202  1.14779059
## C_DietVegan (consuming no animal products)              -0.77223435  0.44239509
## C_DietVegetarian (consuming plants, eggs and milk)      -0.68878880  0.48147855
## Size2Toy                                                -0.62931250  1.54092478
## Size2Small                                              -0.31038518  0.59795179
## Size2Large                                              -0.29214477  0.48493656
## Size2Giant                                              -0.07318830  1.52400800
## D_SexMale                                               -0.41696943  0.24903031
## D_NeuterNeutered                                        -0.37902123  0.49453586
## D_DietMeat-based – raw                                  -0.49945794  0.25294433
## D_DietVegan (consuming no animal products)              -1.16617363  0.23610685
## D_DietVegetarian (including eggs or milk, but not meat) -1.56919862  1.09940445
## Therapeutic_FoodYes                                     -0.05031172  1.80375712
## Visits1                                                 -0.38204727  0.68760728
## Visits2                                                  0.45156073  1.63571519
## Visits3                                                  0.20387520  1.68406982
## Visits3<                                                 0.80937343  2.27048943
## MedsYes                                                  1.47010069  2.21325847
## bs(D_Age, degree = 1, df = 2)1                           0.18557245  1.44042240
## bs(D_Age, degree = 1, df = 2)2                           2.53077361  4.46401610  
  # Wald test
wald.test(b = coef(m), Sigma = vcov(m), Terms = 2)  
  ## Wald test:
## ----------
## 
## Chi-squared test:
## X2 = 0.78, df = 1, P(> X2) = 0.38  
  ## odds ratios and 95% CI
exp(cbind(OR = coef(m), confint(m, level=0.99)))  
  ## Waiting for profiling to be done...  
  ##                                                                  OR       0.5 %
## (Intercept)                                              0.09075674  0.03406478
## LocationOther European                                   1.21744237  0.68454558
## LocationNorth America                                    1.08497899  0.53202827
## LocationAustralia/New Zealand/Oceania                    1.22401527  0.52635553
## LocationOther                                            1.22052187  0.41724443
## UrbanYes                                                 1.04471799  0.72888545
## Education_S1_College                                     1.08315840  0.65656089
## Education_S2_Grad                                        0.89078926  0.53512707
## Education_S3_PG_or_PhD                                   0.71474820  0.41369349
## Animal_Career_BINARYYes                                  0.82252942  0.53189460
## Income2Medium                                            0.86292932  0.54583880
## Income2High                                              0.66877739  0.35542504
## C_Age230–39                                              0.86409779  0.50535980
## C_Age240–49                                              0.75787490  0.43487054
## C_Age250–59                                              0.56396957  0.32197510
## C_Age260<                                                0.63311827  0.34692840
## C_GenderMale                                             0.70378005  0.35101969
## C_DietOmnivore reducing animal product consumption       1.04813380  0.67449139
## C_DietPescatarian (including fish but no other meats)    1.50036631  0.71364952
## C_DietVegan (consuming no animal products)               0.84796185  0.45943391
## C_DietVegetarian (consuming plants, eggs and milk)       0.90153616  0.49933066
## Size2Toy                                                 1.57744451  0.50953074
## Size2Small                                               1.15463387  0.73191912
## Size2Large                                               1.10119493  0.74624377
## Size2Giant                                               2.06557750  0.91857325
## D_SexMale                                                0.91945924  0.65866929
## D_NeuterNeutered                                         1.05945785  0.68631166
## D_DietMeat-based – raw                                   0.88403661  0.60663297
## D_DietVegan (consuming no animal products)               0.62811413  0.31011593
## D_DietVegetarian (including eggs or milk, but not meat)  0.79065222  0.20108466
## Therapeutic_FoodYes                                      2.40301140  0.99094971
## Visits1                                                  1.16506864  0.68890793
## Visits2                                                  2.83952833  1.58327474
## Visits3                                                  2.57017120  1.23317779
## Visits3<                                                 4.66427042  2.26892615
## MedsYes                                                  6.30712269  4.36670715
## bs(D_Age, degree = 1, df = 2)1                           2.25465603  1.21083702
## bs(D_Age, degree = 1, df = 2)2                          33.02929365 12.71833705
##                                                             99.5 %
## (Intercept)                                              0.2351404
## LocationOther European                                   2.1680773
## LocationNorth America                                    2.1827056
## LocationAustralia/New Zealand/Oceania                    2.8360386
## LocationOther                                            3.4599984
## UrbanYes                                                 1.4941417
## Education_S1_College                                     1.7939998
## Education_S2_Grad                                        1.4866187
## Education_S3_PG_or_PhD                                   1.2336576
## Animal_Career_BINARYYes                                  1.2635526
## Income2Medium                                            1.3710799
## Income2High                                              1.2532869
## C_Age230–39                                              1.4773992
## C_Age240–49                                              1.3187516
## C_Age250–59                                              0.9841104
## C_Age260<                                                1.1510901
## C_GenderMale                                             1.3609829
## C_DietOmnivore reducing animal product consumption       1.6235135
## C_DietPescatarian (including fish but no other meats)    3.1582011
## C_DietVegan (consuming no animal products)               1.5506934
## C_DietVegetarian (consuming plants, eggs and milk)       1.6126888
## Size2Toy                                                 4.5398035
## Size2Small                                               1.8173938
## Size2Large                                               1.6246287
## Size2Giant                                               4.5625072
## D_SexMale                                                1.2829791
## D_NeuterNeutered                                         1.6462580
## D_DietMeat-based – raw                                   1.2884734
## D_DietVegan (consuming no animal products)               1.2635719
## D_DietVegetarian (including eggs or milk, but not meat)  2.9893268
## Therapeutic_FoodYes                                      6.4062053
## Visits1                                                  2.0147208
## Visits2                                                  5.1895352
## Visits3                                                  5.4346479
## Visits3<                                                 9.8077004
## MedsYes                                                  9.1890237
## bs(D_Age, degree = 1, df = 2)1                           4.2538095
## bs(D_Age, degree = 1, df = 2)2                          88.1561849  
 
 
 Calculate Nagelkerke R^2 
  NagelkerkeR2(m)  
  ## $N
## [1] 1658
## 
## $R2
## [1] 0.4392804  
 
 
 check assumptions of model 
 
 Cook’s distance 
  plot(m, which = 4, id.n = 3)  
   
 
 
 Extract model results and display data for top 3 values using Cook’s
distance 
  model.data <- augment(m) %>% 
  mutate(index = 1:n()) 
model.data %>% top_n(3, .cooksd)  
 
 
 
 
 
 plot standardised residuals 
  ggplot(model.data, aes(index, .std.resid)) + 
  geom_point(aes(color = Any_Health_Problem), alpha = .5) +
  theme_bw()  
   
 
 
 Filter potential influential data points with abs(.std.res) >
3: 
  model.data %>% 
  filter(abs(.std.resid) > 3)  
 
 
 
 
 
 check for multicollinearity 
  car::vif(m)  
  ##                                   GVIF Df GVIF^(1/(2*Df))
## Location                      1.801049  4        1.076318
## Urban                         1.110659  1        1.053878
## Education_S                   1.254420  3        1.038502
## Animal_Career_BINARY          1.119209  1        1.057927
## Income2                       1.158812  2        1.037536
## C_Age2                        1.437957  4        1.046449
## C_Gender                      1.063264  1        1.031147
## C_Diet                        2.298575  4        1.109641
## Size2                         1.154567  4        1.018128
## D_Sex                         1.045261  1        1.022380
## D_Neuter                      1.170904  1        1.082083
## D_Diet                        2.243887  3        1.144195
## Therapeutic_Food              1.040118  1        1.019862
## Visits                        1.472418  4        1.049552
## Meds                          1.302021  1        1.141061
## bs(D_Age, degree = 1, df = 2) 1.369622  2        1.081808  
 
 
 
 Create ROCR for training and test data 
  ## training data
pred.mtt = predict(m, type = "response") #repeat risk predictions from model m
rocr.pred.mtt = ROCR::prediction(pred.mtt, labels = ml_train$Any_Health_Problem) #ROCR prediction object
roc.perf.mtt = ROCR::performance(rocr.pred.mtt, measure = "tpr", x.measure = "fpr") # #ROCR performance object
plot(roc.perf.mtt, col = "blue")


pred.te.1 = predict(m, newdata = ml_test, type = "response") #.te = "test"
rocr.pred.te.1 = ROCR::prediction(pred.te.1, labels = ml_test$Any_Health_Problem)
roc.perf.te.1 = ROCR::performance(rocr.pred.te.1, measure = "tpr", x.measure = "fpr")
plot(roc.perf.te.1, col = "red", add = T)

abline(a = 0, b = 1, lty = 2) #diagonal for random assignment
legend("bottomright", legend = c("train","test"),
col = c("blue","red"), lty = c(2,1), lwd =1.5)  
   
 
 
 Report AUC from ROC for training and test data 
    # Train AUC
aucTr <- ROCR::performance(rocr.pred.mtt, measure = "auc")
  aucTr <- aucTr@y.values[[1]]
  print(aucTr)  
  ## [1] 0.8425182  
     # Test AUC
  aucTe <- ROCR::performance(rocr.pred.te.1, measure = "auc")
  aucTe <- aucTe@y.values[[1]]
  print(aucTe)  
  ## [1] 0.8520555  
 
 
 
 
 MULTIPLE REGRESSION WITH BACKWARDS ELIMINATION 
 
 ROUND 2: Model with all variables 
 Switch Location + Urban with Location*Urban given simple regression
results 
  # fit binary logit model and store results 'm'
m <- glm(Any_Health_Problem ~  Location*Urban + Education_S + Animal_Career_BINARY 
         + Income2 + C_Age2 + C_Gender + C_Diet + Size2 + D_Sex + D_Neuter  + D_Diet + Therapeutic_Food + Visits 
         + Meds + bs(D_Age,degree=1,df=2), data = ml_train,family = binomial)
# view a summary of the model
summary(m)  
  ## 
## Call:
## glm(formula = Any_Health_Problem ~ Location * Urban + Education_S + 
##     Animal_Career_BINARY + Income2 + C_Age2 + C_Gender + C_Diet + 
##     Size2 + D_Sex + D_Neuter + D_Diet + Therapeutic_Food + Visits + 
##     Meds + bs(D_Age, degree = 1, df = 2), family = binomial, 
##     data = ml_train)
## 
## Coefficients:
##                                                         Estimate Std. Error
## (Intercept)                                             -2.37665    0.37551
## LocationOther European                                   0.11243    0.27009
## LocationNorth America                                   -0.10000    0.35644
## LocationAustralia/New Zealand/Oceania                    0.59923    0.54294
## LocationOther                                           -0.34767    0.88528
## UrbanYes                                                -0.01374    0.16482
## Education_S1_College                                     0.07418    0.19523
## Education_S2_Grad                                       -0.11989    0.19843
## Education_S3_PG_or_PhD                                  -0.33721    0.21209
## Animal_Career_BINARYYes                                 -0.19512    0.16795
## Income2Medium                                           -0.14923    0.17857
## Income2High                                             -0.41075    0.24493
## C_Age230–39                                             -0.15106    0.20825
## C_Age240–49                                             -0.29633    0.21584
## C_Age250–59                                             -0.57767    0.21709
## C_Age260<                                               -0.47105    0.23294
## C_GenderMale                                            -0.36259    0.26310
## C_DietOmnivore reducing animal product consumption       0.04373    0.17040
## C_DietPescatarian (including fish but no other meats)    0.42933    0.28856
## C_DietVegan (consuming no animal products)              -0.15508    0.23681
## C_DietVegetarian (consuming plants, eggs and milk)      -0.09784    0.22789
## Size2Toy                                                 0.45032    0.42233
## Size2Small                                               0.14414    0.17637
## Size2Large                                               0.10467    0.15123
## Size2Giant                                               0.76147    0.31261
## D_SexMale                                               -0.08351    0.12943
## D_NeuterNeutered                                         0.05083    0.17002
## D_DietMeat-based – raw                                  -0.12563    0.14636
## D_DietVegan (consuming no animal products)              -0.49701    0.27387
## D_DietVegetarian (including eggs or milk, but not meat) -0.24939    0.52374
## Therapeutic_FoodYes                                      0.86228    0.36108
## Visits1                                                  0.15787    0.20776
## Visits2                                                  1.04857    0.23018
## Visits3                                                  0.94792    0.28745
## Visits3<                                                 1.54154    0.28425
## MedsYes                                                  1.83738    0.14435
## bs(D_Age, degree = 1, df = 2)1                           0.82411    0.24455
## bs(D_Age, degree = 1, df = 2)2                           3.50856    0.37643
## LocationOther European:UrbanYes                          0.22063    0.38665
## LocationNorth America:UrbanYes                           0.44965    0.54150
## LocationAustralia/New Zealand/Oceania:UrbanYes          -0.54913    0.66463
## LocationOther:UrbanYes                                   0.74896    0.98948
##                                                         z value Pr(>|z|)    
## (Intercept)                                              -6.329 2.47e-10 ***
## LocationOther European                                    0.416 0.677200    
## LocationNorth America                                    -0.281 0.779054    
## LocationAustralia/New Zealand/Oceania                     1.104 0.269735    
## LocationOther                                            -0.393 0.694524    
## UrbanYes                                                 -0.083 0.933544    
## Education_S1_College                                      0.380 0.703980    
## Education_S2_Grad                                        -0.604 0.545703    
## Education_S3_PG_or_PhD                                   -1.590 0.111844    
## Animal_Career_BINARYYes                                  -1.162 0.245335    
## Income2Medium                                            -0.836 0.403331    
## Income2High                                              -1.677 0.093537 .  
## C_Age230–39                                              -0.725 0.468221    
## C_Age240–49                                              -1.373 0.169770    
## C_Age250–59                                              -2.661 0.007793 ** 
## C_Age260<                                                -2.022 0.043158 *  
## C_GenderMale                                             -1.378 0.168161    
## C_DietOmnivore reducing animal product consumption        0.257 0.797437    
## C_DietPescatarian (including fish but no other meats)     1.488 0.136791    
## C_DietVegan (consuming no animal products)               -0.655 0.512564    
## C_DietVegetarian (consuming plants, eggs and milk)       -0.429 0.667700    
## Size2Toy                                                  1.066 0.286301    
## Size2Small                                                0.817 0.413768    
## Size2Large                                                0.692 0.488874    
## Size2Giant                                                2.436 0.014858 *  
## D_SexMale                                                -0.645 0.518794    
## D_NeuterNeutered                                          0.299 0.764989    
## D_DietMeat-based – raw                                   -0.858 0.390693    
## D_DietVegan (consuming no animal products)               -1.815 0.069560 .  
## D_DietVegetarian (including eggs or milk, but not meat)  -0.476 0.633950    
## Therapeutic_FoodYes                                       2.388 0.016939 *  
## Visits1                                                   0.760 0.447340    
## Visits2                                                   4.555 5.23e-06 ***
## Visits3                                                   3.298 0.000975 ***
## Visits3<                                                  5.423 5.86e-08 ***
## MedsYes                                                  12.729  < 2e-16 ***
## bs(D_Age, degree = 1, df = 2)1                            3.370 0.000752 ***
## bs(D_Age, degree = 1, df = 2)2                            9.321  < 2e-16 ***
## LocationOther European:UrbanYes                           0.571 0.568257    
## LocationNorth America:UrbanYes                            0.830 0.406321    
## LocationAustralia/New Zealand/Oceania:UrbanYes           -0.826 0.408677    
## LocationOther:UrbanYes                                    0.757 0.449093    
## ---
## Signif. codes:  0 '***' 0.001 '**' 0.01 '*' 0.05 '.' 0.1 ' ' 1
## 
## (Dispersion parameter for binomial family taken to be 1)
## 
##     Null deviance: 2200.0  on 1657  degrees of freedom
## Residual deviance: 1551.6  on 1616  degrees of freedom
## AIC: 1635.6
## 
## Number of Fisher Scoring iterations: 4  
  # test model fit
with(m, null.deviance - deviance)  
  ## [1] 648.4609  
  with(m, df.null - df.residual)  
  ## [1] 41  
  with(m, pchisq(null.deviance - deviance, df.null - df.residual, lower.tail = FALSE))  
  ## [1] 2.780453e-110  
  BIC(m)  
  ## [1] 1862.929  
  # Hosmer-Lemeshow Goodness-of-Fit Test
hltest(m)  
  ## 
##    The Hosmer-Lemeshow goodness-of-fit test
## 
##  Group Size Observed   Expected
##      1  166        9   9.540567
##      2  166       19  14.528454
##      3  166       18  20.225872
##      4  166       28  26.978084
##      5  166       42  37.334526
##      6  166       52  55.305552
##      7  166       71  82.124990
##      8  166      105 106.177704
##      9  166      133 127.778445
##     10  164      151 148.005804
## 
##          Statistic =  7.48107 
## degrees of freedom =  8 
##            p-value =  0.48573  
  ## CIs using profiled log-likelihood
confint(m, level=0.99)  
  ## Waiting for profiling to be done...  
  ##                                                               0.5 %      99.5 %
## (Intercept)                                             -3.35926515 -1.42173393
## LocationOther European                                  -0.58888093  0.80525955
## LocationNorth America                                   -1.04050240  0.80545906
## LocationAustralia/New Zealand/Oceania                   -0.80735586  2.01571282
## LocationOther                                           -3.19195414  1.68731128
## UrbanYes                                                -0.44097609  0.40917479
## Education_S1_College                                    -0.42744769  0.57968380
## Education_S2_Grad                                       -0.63042177  0.39324568
## Education_S3_PG_or_PhD                                  -0.88476637  0.20928041
## Animal_Career_BINARYYes                                 -0.63160193  0.23475186
## Income2Medium                                           -0.60737964  0.31392147
## Income2High                                             -1.04462632  0.21892638
## C_Age230–39                                             -0.68820144  0.38601653
## C_Age240–49                                             -0.85389985  0.25947022
## C_Age250–59                                             -1.13947588 -0.01956889
## C_Age260<                                               -1.07375431  0.12791365
## C_GenderMale                                            -1.06121913  0.29923286
## C_DietOmnivore reducing animal product consumption      -0.39733197  0.48159078
## C_DietPescatarian (including fish but no other meats)   -0.31502350  1.17476968
## C_DietVegan (consuming no animal products)              -0.77055831  0.45131517
## C_DietVegetarian (consuming plants, eggs and milk)      -0.69058828  0.48556692
## Size2Toy                                                -0.68214137  1.51049078
## Size2Small                                              -0.31188147  0.59787672
## Size2Large                                              -0.28539312  0.49460951
## Size2Giant                                              -0.05513042  1.56085310
## D_SexMale                                               -0.41746927  0.25003575
## D_NeuterNeutered                                        -0.38450924  0.49277139
## D_DietMeat-based – raw                                  -0.50304761  0.25187038
## D_DietVegan (consuming no animal products)              -1.20731845  0.20618707
## D_DietVegetarian (including eggs or milk, but not meat) -1.63393162  1.09530912
## Therapeutic_FoodYes                                     -0.02669317  1.84575018
## Visits1                                                 -0.36789153  0.70589173
## Visits2                                                  0.46362034  1.65244594
## Visits3                                                  0.21322285  1.69708238
## Visits3<                                                 0.81930090  2.28643523
## MedsYes                                                  1.46946577  2.21393313
## bs(D_Age, degree = 1, df = 2)1                           0.19996921  1.46150073
## bs(D_Age, degree = 1, df = 2)2                           2.55130263  4.49337499
## LocationOther European:UrbanYes                         -0.77458618  1.22086603
## LocationNorth America:UrbanYes                          -0.94692477  1.85327335
## LocationAustralia/New Zealand/Oceania:UrbanYes          -2.27534707  1.16655751
## LocationOther:UrbanYes                                  -1.60352065  3.76807595  
  ## CIs using standard errors
confint.default(m, level=0.99)  
  ##                                                               0.5 %      99.5 %
## (Intercept)                                             -3.34389849 -1.40940008
## LocationOther European                                  -0.58326848  0.80813724
## LocationNorth America                                   -1.01813731  0.81813604
## LocationAustralia/New Zealand/Oceania                   -0.79929577  1.99775566
## LocationOther                                           -2.62800255  1.93266255
## UrbanYes                                                -0.43828192  0.41079485
## Education_S1_College                                    -0.42869407  0.57704825
## Education_S2_Grad                                       -0.63101973  0.39123087
## Education_S3_PG_or_PhD                                  -0.88350768  0.20908987
## Animal_Career_BINARYYes                                 -0.62772668  0.23749346
## Income2Medium                                           -0.60919282  0.31073617
## Income2High                                             -1.04163445  0.22014021
## C_Age230–39                                             -0.68746184  0.38534861
## C_Age240–49                                             -0.85229030  0.25962731
## C_Age250–59                                             -1.13686321 -0.01847046
## C_Age260<                                               -1.07106494  0.12896748
## C_GenderMale                                            -1.04028896  0.31511338
## C_DietOmnivore reducing animal product consumption      -0.39517629  0.48264595
## C_DietPescatarian (including fish but no other meats)   -0.31394697  1.17260925
## C_DietVegan (consuming no animal products)              -0.76505867  0.45490791
## C_DietVegetarian (consuming plants, eggs and milk)      -0.68484334  0.48917318
## Size2Toy                                                -0.63753369  1.53817484
## Size2Small                                              -0.31015604  0.59844429
## Size2Large                                              -0.28487872  0.49421322
## Size2Giant                                              -0.04376959  1.56670405
## D_SexMale                                               -0.41690098  0.24988202
## D_NeuterNeutered                                        -0.38711828  0.48876856
## D_DietMeat-based – raw                                  -0.50264372  0.25137634
## D_DietVegan (consuming no animal products)              -1.20245250  0.20843166
## D_DietVegetarian (including eggs or milk, but not meat) -1.59844254  1.09966307
## Therapeutic_FoodYes                                     -0.06780829  1.79236695
## Visits1                                                 -0.37728528  0.69301984
## Visits2                                                  0.45565856  1.64148402
## Visits3                                                  0.20749858  1.68834173
## Visits3<                                                 0.80935264  2.27373491
## MedsYes                                                  1.46556449  2.20918579
## bs(D_Age, degree = 1, df = 2)1                           0.19418263  1.45404505
## bs(D_Age, degree = 1, df = 2)2                           2.53895246  4.47816794
## LocationOther European:UrbanYes                         -0.77530651  1.21656288
## LocationNorth America:UrbanYes                          -0.94515042  1.84445077
## LocationAustralia/New Zealand/Oceania:UrbanYes          -2.26110410  1.16283939
## LocationOther:UrbanYes                                  -1.79976479  3.29769135  
  # Wald test
wald.test(b = coef(m), Sigma = vcov(m), Terms = 2)  
  ## Wald test:
## ----------
## 
## Chi-squared test:
## X2 = 0.17, df = 1, P(> X2) = 0.68  
  ## odds ratios and 95% CI
exp(cbind(OR = coef(m), confint(m, level=0.99)))  
  ## Waiting for profiling to be done...  
  ##                                                                  OR       0.5 %
## (Intercept)                                              0.09286121  0.03476079
## LocationOther European                                   1.11899883  0.55494796
## LocationNorth America                                    0.90483684  0.35327715
## LocationAustralia/New Zealand/Oceania                    1.82071621  0.44603589
## LocationOther                                            0.70633193  0.04109149
## UrbanYes                                                 0.98635048  0.64340809
## Education_S1_College                                     1.07699752  0.65217152
## Education_S2_Grad                                        0.88701407  0.53236722
## Education_S3_PG_or_PhD                                   0.71375972  0.41281061
## Animal_Career_BINARYYes                                  0.82273871  0.53173931
## Income2Medium                                            0.86137242  0.54477651
## Income2High                                              0.66315461  0.35182326
## C_Age230–39                                              0.85979902  0.50247899
## C_Age240–49                                              0.74354091  0.42575132
## C_Age250–59                                              0.56120623  0.31998669
## C_Age260<                                                0.62434715  0.34172317
## C_GenderMale                                             0.69587322  0.34603369
## C_DietOmnivore reducing animal product consumption       1.04470530  0.67211087
## C_DietPescatarian (including fish but no other meats)    1.53622965  0.72977172
## C_DietVegan (consuming no animal products)               0.85635062  0.46275463
## C_DietVegetarian (consuming plants, eggs and milk)       0.90679844  0.50128109
## Size2Toy                                                 1.56881503  0.50553330
## Size2Small                                               1.15505057  0.73206830
## Size2Large                                               1.11034108  0.75171868
## Size2Giant                                               2.14141587  0.94636172
## D_SexMale                                                0.91988236  0.65871173
## D_NeuterNeutered                                         1.05213890  0.68078466
## D_DietMeat-based – raw                                   0.88193785  0.60468501
## D_DietVegan (consuming no animal products)               0.60834665  0.29899798
## D_DietVegetarian (including eggs or milk, but not meat)  0.77927620  0.19516077
## Therapeutic_FoodYes                                      2.36855327  0.97365995
## Visits1                                                  1.17101076  0.69219226
## Visits2                                                  2.85357127  1.58981927
## Visits3                                                  2.58033737  1.23766043
## Visits3<                                                 4.67179691  2.26891309
## MedsYes                                                  6.28003239  4.34691227
## bs(D_Age, degree = 1, df = 2)1                           2.27985955  1.22136515
## bs(D_Age, degree = 1, df = 2)2                          33.40014365 12.82379754
## LocationOther European:UrbanYes                          1.24685974  0.46089447
## LocationNorth America:UrbanYes                           1.56776365  0.38793217
## LocationAustralia/New Zealand/Oceania:UrbanYes           0.57745061  0.10276124
## LocationOther:UrbanYes                                   2.11480642  0.20118696
##                                                             99.5 %
## (Intercept)                                              0.2412953
## LocationOther European                                   2.2372771
## LocationNorth America                                    2.2377235
## LocationAustralia/New Zealand/Oceania                    7.5060760
## LocationOther                                            5.4049288
## UrbanYes                                                 1.5055749
## Education_S1_College                                     1.7854738
## Education_S2_Grad                                        1.4817824
## Education_S3_PG_or_PhD                                   1.2327906
## Animal_Career_BINARYYes                                  1.2645949
## Income2Medium                                            1.3687822
## Income2High                                              1.2447396
## C_Age230–39                                              1.4711090
## C_Age240–49                                              1.2962432
## C_Age250–59                                              0.9806213
## C_Age260<                                                1.1364549
## C_GenderMale                                             1.3488237
## C_DietOmnivore reducing animal product consumption       1.6186473
## C_DietPescatarian (including fish but no other meats)    3.2373972
## C_DietVegan (consuming no animal products)               1.5703761
## C_DietVegetarian (consuming plants, eggs and milk)       1.6250960
## Size2Toy                                                 4.5289530
## Size2Small                                               1.8182540
## Size2Large                                               1.6398578
## Size2Giant                                               4.7628827
## D_SexMale                                                1.2840713
## D_NeuterNeutered                                         1.6368463
## D_DietMeat-based – raw                                   1.2864293
## D_DietVegan (consuming no animal products)               1.2289831
## D_DietVegetarian (including eggs or milk, but not meat)  2.9901068
## Therapeutic_FoodYes                                      6.3328488
## Visits1                                                  2.0256522
## Visits2                                                  5.2197314
## Visits3                                                  5.4579997
## Visits3<                                                 9.8397984
## MedsYes                                                  9.1516403
## bs(D_Age, degree = 1, df = 2)1                           4.3124265
## bs(D_Age, degree = 1, df = 2)2                          89.4227384
## LocationOther European:UrbanYes                          3.3901224
## LocationNorth America:UrbanYes                           6.3806716
## LocationAustralia/New Zealand/Oceania:UrbanYes           3.2109200
## LocationOther:UrbanYes                                  43.2966799  
 
 Calculate Nagelkerke R^2 
  NagelkerkeR2(m)  
  ## $N
## [1] 1658
## 
## $R2
## [1] 0.4405767  
 
 
 check assumptions of model 
 
 Cook’s distance 
  plot(m, which = 4, id.n = 3)  
   
 
 
 Extract model results and display data for top 3 values using Cook’s
distance 
  model.data <- augment(m) %>% 
  mutate(index = 1:n()) 
model.data %>% top_n(3, .cooksd)  
 
 
 
 
 
 plot standardised residuals 
  ggplot(model.data, aes(index, .std.resid)) + 
  geom_point(aes(color = Any_Health_Problem), alpha = .5) +
  theme_bw()  
   
 
 
 Filter potential influential data points with abs(.std.res) >
3: 
  model.data %>% 
  filter(abs(.std.resid) > 3)  
 
 
 
 
 
 check for multicollinearity 
  car::vif(m)  
  ## there are higher-order terms (interactions) in this model
## consider setting type = 'predictor'; see ?vif  
  ##                                    GVIF Df GVIF^(1/(2*Df))
## Location                      60.459611  4        1.669872
## Urban                          1.555266  1        1.247103
## Education_S                    1.260061  3        1.039279
## Animal_Career_BINARY           1.120274  1        1.058430
## Income2                        1.164329  2        1.038769
## C_Age2                         1.464092  4        1.048808
## C_Gender                       1.065008  1        1.031992
## C_Diet                         2.341857  4        1.112231
## Size2                          1.173052  4        1.020151
## D_Sex                          1.046010  1        1.022746
## D_Neuter                       1.177315  1        1.085041
## D_Diet                         2.274536  3        1.146785
## Therapeutic_Food               1.040795  1        1.020194
## Visits                         1.480577  4        1.050277
## Meds                           1.301784  1        1.140957
## bs(D_Age, degree = 1, df = 2)  1.385604  2        1.084950
## Location:Urban                64.619393  4        1.683819  
 
 
 
 Create ROCR for training and test data 
  ## training data
pred.mtt = predict(m, type = "response") #repeat risk predictions from model m
rocr.pred.mtt = ROCR::prediction(pred.mtt, labels = ml_train$Any_Health_Problem) #ROCR prediction object
roc.perf.mtt = ROCR::performance(rocr.pred.mtt, measure = "tpr", x.measure = "fpr") # #ROCR performance object
plot(roc.perf.mtt, col = "blue")


pred.te.1 = predict(m, newdata = ml_test, type = "response") #.te = "test"
rocr.pred.te.1 = ROCR::prediction(pred.te.1, labels = ml_test$Any_Health_Problem)
roc.perf.te.1 = ROCR::performance(rocr.pred.te.1, measure = "tpr", x.measure = "fpr")
plot(roc.perf.te.1, col = "red", add = T)

abline(a = 0, b = 1, lty = 2) #diagonal for random assignment
legend("bottomright", legend = c("train","test"),
col = c("blue","red"), lty = c(2,1), lwd =1.5)  
   
 
 
 Report AUC from ROC for training and test data 
    # Train AUC
aucTr <- ROCR::performance(rocr.pred.mtt, measure = "auc")
  aucTr <- aucTr@y.values[[1]]
  print(aucTr)  
  ## [1] 0.8430593  
     # Test AUC
  aucTe <- ROCR::performance(rocr.pred.te.1, measure = "auc")
  aucTe <- aucTe@y.values[[1]]
  print(aucTe)  
  ## [1] 0.8529195  
 
 
 
 
 MULTIPLE REGRESSION WITH BACKWARDS ELIMINATION 
 
 ROUND 3: Model with all variables 
 Try removing Location 
  # fit binary logit model and store results 'm'
m <- glm(Any_Health_Problem ~  Urban + Education_S + Animal_Career_BINARY 
         + Income2 + C_Age2 + C_Gender + C_Diet + Size2 + D_Sex + D_Neuter  + D_Diet + Therapeutic_Food + Visits 
         + Meds + bs(D_Age,degree=1,df=2), data = ml_train,family = binomial)
# view a summary of the model
summary(m)  
  ## 
## Call:
## glm(formula = Any_Health_Problem ~ Urban + Education_S + Animal_Career_BINARY + 
##     Income2 + C_Age2 + C_Gender + C_Diet + Size2 + D_Sex + D_Neuter + 
##     D_Diet + Therapeutic_Food + Visits + Meds + bs(D_Age, degree = 1, 
##     df = 2), family = binomial, data = ml_train)
## 
## Coefficients:
##                                                         Estimate Std. Error
## (Intercept)                                             -2.39428    0.37306
## UrbanYes                                                 0.06143    0.13669
## Education_S1_College                                     0.07958    0.19463
## Education_S2_Grad                                       -0.10767    0.19754
## Education_S3_PG_or_PhD                                  -0.32524    0.21122
## Animal_Career_BINARYYes                                 -0.20327    0.16697
## Income2Medium                                           -0.13951    0.17809
## Income2High                                             -0.39044    0.24311
## C_Age230–39                                             -0.15197    0.20769
## C_Age240–49                                             -0.28070    0.21375
## C_Age250–59                                             -0.58836    0.21553
## C_Age260<                                               -0.47959    0.23029
## C_GenderMale                                            -0.33439    0.26095
## C_DietOmnivore reducing animal product consumption       0.06608    0.16896
## C_DietPescatarian (including fish but no other meats)    0.43450    0.28658
## C_DietVegan (consuming no animal products)              -0.10662    0.22691
## C_DietVegetarian (consuming plants, eggs and milk)      -0.07539    0.22497
## Size2Toy                                                 0.44328    0.41972
## Size2Small                                               0.14591    0.17580
## Size2Large                                               0.09483    0.15045
## Size2Giant                                               0.73769    0.30920
## D_SexMale                                               -0.08423    0.12912
## D_NeuterNeutered                                         0.04640    0.16826
## D_DietMeat-based – raw                                  -0.13438    0.14538
## D_DietVegan (consuming no animal products)              -0.40640    0.26465
## D_DietVegetarian (including eggs or milk, but not meat) -0.17724    0.50938
## Therapeutic_FoodYes                                      0.89583    0.35859
## Visits1                                                  0.16185    0.20718
## Visits2                                                  1.06311    0.22755
## Visits3                                                  0.95199    0.28684
## Visits3<                                                 1.55725    0.28233
## MedsYes                                                  1.83583    0.14343
## bs(D_Age, degree = 1, df = 2)1                           0.82162    0.24293
## bs(D_Age, degree = 1, df = 2)2                           3.51170    0.37472
##                                                         z value Pr(>|z|)    
## (Intercept)                                              -6.418 1.38e-10 ***
## UrbanYes                                                  0.449 0.653152    
## Education_S1_College                                      0.409 0.682623    
## Education_S2_Grad                                        -0.545 0.585725    
## Education_S3_PG_or_PhD                                   -1.540 0.123609    
## Animal_Career_BINARYYes                                  -1.217 0.223464    
## Income2Medium                                            -0.783 0.433436    
## Income2High                                              -1.606 0.108261    
## C_Age230–39                                              -0.732 0.464359    
## C_Age240–49                                              -1.313 0.189102    
## C_Age250–59                                              -2.730 0.006337 ** 
## C_Age260<                                                -2.083 0.037295 *  
## C_GenderMale                                             -1.281 0.200036    
## C_DietOmnivore reducing animal product consumption        0.391 0.695720    
## C_DietPescatarian (including fish but no other meats)     1.516 0.129482    
## C_DietVegan (consuming no animal products)               -0.470 0.638441    
## C_DietVegetarian (consuming plants, eggs and milk)       -0.335 0.737551    
## Size2Toy                                                  1.056 0.290908    
## Size2Small                                                0.830 0.406552    
## Size2Large                                                0.630 0.528501    
## Size2Giant                                                2.386 0.017044 *  
## D_SexMale                                                -0.652 0.514160    
## D_NeuterNeutered                                          0.276 0.782722    
## D_DietMeat-based – raw                                   -0.924 0.355328    
## D_DietVegan (consuming no animal products)               -1.536 0.124639    
## D_DietVegetarian (including eggs or milk, but not meat)  -0.348 0.727880    
## Therapeutic_FoodYes                                       2.498 0.012483 *  
## Visits1                                                   0.781 0.434678    
## Visits2                                                   4.672 2.98e-06 ***
## Visits3                                                   3.319 0.000904 ***
## Visits3<                                                  5.516 3.47e-08 ***
## MedsYes                                                  12.800  < 2e-16 ***
## bs(D_Age, degree = 1, df = 2)1                            3.382 0.000719 ***
## bs(D_Age, degree = 1, df = 2)2                            9.371  < 2e-16 ***
## ---
## Signif. codes:  0 '***' 0.001 '**' 0.01 '*' 0.05 '.' 0.1 ' ' 1
## 
## (Dispersion parameter for binomial family taken to be 1)
## 
##     Null deviance: 2200  on 1657  degrees of freedom
## Residual deviance: 1555  on 1624  degrees of freedom
## AIC: 1623
## 
## Number of Fisher Scoring iterations: 4  
  # test model fit
with(m, null.deviance - deviance)  
  ## [1] 645.055  
  with(m, df.null - df.residual)  
  ## [1] 33  
  with(m, pchisq(null.deviance - deviance, df.null - df.residual, lower.tail = FALSE))  
  ## [1] 1.309234e-114  
  BIC(m)  
  ## [1] 1807.028  
  # Hosmer-Lemeshow Goodness-of-Fit Test
hltest(m)  
  ## 
##    The Hosmer-Lemeshow goodness-of-fit test
## 
##  Group Size Observed   Expected
##      1  166        9   9.664812
##      2  166       18  14.797085
##      3  166       18  20.280730
##      4  166       31  26.982821
##      5  166       37  37.242609
##      6  166       57  55.134776
##      7  166       66  82.260184
##      8  166      109 106.165266
##      9  166      135 127.697553
##     10  164      148 147.774164
## 
##          Statistic =  10.30729 
## degrees of freedom =  8 
##            p-value =  0.24412  
  ## CIs using profiled log-likelihood
confint(m, level=0.99)  
  ## Waiting for profiling to be done...  
  ##                                                               0.5 %      99.5 %
## (Intercept)                                             -3.37047051 -1.44564165
## UrbanYes                                                -0.29202279  0.41294429
## Education_S1_College                                    -0.42050574  0.58352970
## Education_S2_Grad                                       -0.61587739  0.40314771
## Education_S3_PG_or_PhD                                  -0.87052568  0.21905030
## Animal_Career_BINARYYes                                 -0.63728998  0.22403260
## Income2Medium                                           -0.59634850  0.32248924
## Income2High                                             -1.01954287  0.23461244
## C_Age230–39                                             -0.68768550  0.38367505
## C_Age240–49                                             -0.83269607  0.26987408
## C_Age250–59                                             -1.14621353 -0.03438417
## C_Age260<                                               -1.07562533  0.11235859
## C_GenderMale                                            -1.02639592  0.32275913
## C_DietOmnivore reducing animal product consumption      -0.37107775  0.50041220
## C_DietPescatarian (including fish but no other meats)   -0.30490354  1.17464531
## C_DietVegan (consuming no animal products)              -0.69588410  0.47487048
## C_DietVegetarian (consuming plants, eggs and milk)      -0.66060935  0.50047391
## Size2Toy                                                -0.68187335  1.49723063
## Size2Small                                              -0.30861750  0.59820115
## Size2Large                                              -0.29329834  0.48268491
## Size2Giant                                              -0.07062739  1.52794867
## D_SexMale                                               -0.41738724  0.24850848
## D_NeuterNeutered                                        -0.38438223  0.48382870
## D_DietMeat-based – raw                                  -0.50931075  0.24052298
## D_DietVegan (consuming no animal products)              -1.09260480  0.27327596
## D_DietVegetarian (including eggs or milk, but not meat) -1.52287726  1.12876061
## Therapeutic_FoodYes                                      0.01322340  1.87282589
## Visits1                                                 -0.36230585  0.70850257
## Visits2                                                  0.48522293  1.66045602
## Visits3                                                  0.21889234  1.69954943
## Visits3<                                                 0.84003472  2.29722689
## MedsYes                                                  1.47017762  2.20989530
## bs(D_Age, degree = 1, df = 2)1                           0.20157970  1.45469955
## bs(D_Age, degree = 1, df = 2)2                           2.55872586  4.49200339  
  ## CIs using standard errors
confint.default(m, level=0.99)  
  ##                                                               0.5 %     99.5 %
## (Intercept)                                             -3.35521297 -1.4333477
## UrbanYes                                                -0.29066622  0.4135212
## Education_S1_College                                    -0.42175329  0.5809167
## Education_S2_Grad                                       -0.61648499  0.4011541
## Education_S3_PG_or_PhD                                  -0.86932043  0.2188338
## Animal_Career_BINARYYes                                 -0.63336677  0.2268281
## Income2Medium                                           -0.59824487  0.3192340
## Income2High                                             -1.01664616  0.2357579
## C_Age230–39                                             -0.68695461  0.3830186
## C_Age240–49                                             -0.83127108  0.2698730
## C_Age250–59                                             -1.14353547 -0.0331866
## C_Age260<                                               -1.07279105  0.1136063
## C_GenderMale                                            -1.00655846  0.3377694
## C_DietOmnivore reducing animal product consumption      -0.36912928  0.5012908
## C_DietPescatarian (including fish but no other meats)   -0.30368437  1.1726844
## C_DietVegan (consuming no animal products)              -0.69109629  0.4778574
## C_DietVegetarian (consuming plants, eggs and milk)      -0.65488165  0.5041050
## Size2Toy                                                -0.63784957  1.5244114
## Size2Small                                              -0.30692574  0.5987505
## Size2Large                                              -0.29271436  0.4823762
## Size2Giant                                              -0.05876888  1.5341408
## D_SexMale                                               -0.41682334  0.2483553
## D_NeuterNeutered                                        -0.38701218  0.4798167
## D_DietMeat-based – raw                                  -0.50885618  0.2401019
## D_DietVegan (consuming no animal products)              -1.08810536  0.2753036
## D_DietVegetarian (including eggs or milk, but not meat) -1.48930513  1.1348327
## Therapeutic_FoodYes                                     -0.02784097  1.8194919
## Visits1                                                 -0.37181666  0.6955264
## Visits2                                                  0.47698531  1.6492366
## Visits3                                                  0.21314558  1.6908356
## Visits3<                                                 0.83001398  2.2844791
## MedsYes                                                  1.46637730  2.2052733
## bs(D_Age, degree = 1, df = 2)1                           0.19588061  1.4473568
## bs(D_Age, degree = 1, df = 2)2                           2.54648148  4.4769215  
  # Wald test
wald.test(b = coef(m), Sigma = vcov(m), Terms = 2)  
  ## Wald test:
## ----------
## 
## Chi-squared test:
## X2 = 0.2, df = 1, P(> X2) = 0.65  
  ## odds ratios and 95% CI
exp(cbind(OR = coef(m), confint(m, level=0.99)))  
  ## Waiting for profiling to be done...  
  ##                                                                  OR       0.5 %
## (Intercept)                                              0.09123832  0.03437346
## UrbanYes                                                 1.06335341  0.74675152
## Education_S1_College                                     1.08283405  0.65671461
## Education_S2_Grad                                        0.89792793  0.54016675
## Education_S3_PG_or_PhD                                   0.72235158  0.41873137
## Animal_Career_BINARYYes                                  0.81605843  0.52872334
## Income2Medium                                            0.86978828  0.55081929
## Income2High                                              0.67675622  0.36075982
## C_Age230–39                                              0.85901575  0.50273831
## C_Age240–49                                              0.75525559  0.43487525
## C_Age250–59                                              0.55523655  0.31783798
## C_Age260<                                                0.61903566  0.34108440
## C_GenderMale                                             0.71577135  0.35829596
## C_DietOmnivore reducing animal product consumption       1.06831301  0.68999029
## C_DietPescatarian (including fish but no other meats)    1.54419080  0.73719448
## C_DietVegan (consuming no animal products)               0.89886768  0.49863341
## C_DietVegetarian (consuming plants, eggs and milk)       0.92738327  0.51653649
## Size2Toy                                                 1.55780991  0.50566881
## Size2Small                                               1.15709483  0.73446165
## Size2Large                                               1.09947293  0.74579961
## Size2Giant                                               2.09109101  0.93180903
## D_SexMale                                                0.91921609  0.65876577
## D_NeuterNeutered                                         1.04749569  0.68087113
## D_DietMeat-based – raw                                   0.87426030  0.60090961
## D_DietVegan (consuming no animal products)               0.66604313  0.33534186
## D_DietVegetarian (including eggs or milk, but not meat)  0.83758193  0.21808350
## Therapeutic_FoodYes                                      2.44935677  1.01331121
## Visits1                                                  1.17568963  0.69606944
## Visits2                                                  2.89536436  1.62453713
## Visits3                                                  2.59086188  1.24469726
## Visits3<                                                 4.74573612  2.31644741
## MedsYes                                                  6.27030680  4.35000771
## bs(D_Age, degree = 1, df = 2)1                           2.27417808  1.22333373
## bs(D_Age, degree = 1, df = 2)2                          33.50522782 12.91934574
##                                                             99.5 %
## (Intercept)                                              0.2355949
## UrbanYes                                                 1.5112608
## Education_S1_College                                     1.7923537
## Education_S2_Grad                                        1.4965279
## Education_S3_PG_or_PhD                                   1.2448939
## Animal_Career_BINARYYes                                  1.2511118
## Income2Medium                                            1.3805600
## Income2High                                              1.2644186
## C_Age230–39                                              1.4676684
## C_Age240–49                                              1.3097995
## C_Age250–59                                              0.9662002
## C_Age260<                                                1.1189140
## C_GenderMale                                             1.3809327
## C_DietOmnivore reducing animal product consumption       1.6494010
## C_DietPescatarian (including fish but no other meats)    3.2369946
## C_DietVegan (consuming no animal products)               1.6078059
## C_DietVegetarian (consuming plants, eggs and milk)       1.6495028
## Size2Toy                                                 4.4692948
## Size2Small                                               1.8188440
## Size2Large                                               1.6204192
## Size2Giant                                               4.6087131
## D_SexMale                                                1.2821117
## D_NeuterNeutered                                         1.6222737
## D_DietMeat-based – raw                                   1.2719142
## D_DietVegan (consuming no animal products)               1.3142629
## D_DietVegetarian (including eggs or milk, but not meat)  3.0918222
## Therapeutic_FoodYes                                      6.5066575
## Visits1                                                  2.0309478
## Visits2                                                  5.2617098
## Visits3                                                  5.4714815
## Visits3<                                                 9.9465612
## MedsYes                                                  9.1147621
## bs(D_Age, degree = 1, df = 2)1                           4.2831964
## bs(D_Age, degree = 1, df = 2)2                          89.3001701  
 
 Calculate Nagelkerke R^2 
  NagelkerkeR2(m)  
  ## $N
## [1] 1658
## 
## $R2
## [1] 0.4386839  
 
 
 check assumptions of model 
 
 Cook’s distance 
  plot(m, which = 4, id.n = 3)  
   
 
 
 Extract model results and display data for top 3 values using Cook’s
distance 
  model.data <- augment(m) %>% 
  mutate(index = 1:n()) 
model.data %>% top_n(3, .cooksd)  
 
 
 
 
 
 plot standardised residuals 
  ggplot(model.data, aes(index, .std.resid)) + 
  geom_point(aes(color = Any_Health_Problem), alpha = .5) +
  theme_bw()  
   
 
 
 Filter potential influential data points with abs(.std.res) >
3: 
  model.data %>% 
  filter(abs(.std.resid) > 3)  
 
 
 
 
 
 check for multicollinearity 
  car::vif(m)  
  ##                                   GVIF Df GVIF^(1/(2*Df))
## Urban                         1.072898  1        1.035808
## Education_S                   1.239284  3        1.036403
## Animal_Career_BINARY          1.108019  1        1.052625
## Income2                       1.146956  2        1.034872
## C_Age2                        1.391491  4        1.042162
## C_Gender                      1.056919  1        1.028066
## C_Diet                        2.095668  4        1.096896
## Size2                         1.139787  4        1.016490
## D_Sex                         1.043496  1        1.021516
## D_Neuter                      1.154496  1        1.074475
## D_Diet                        2.028249  3        1.125089
## Therapeutic_Food              1.035366  1        1.017530
## Visits                        1.427557  4        1.045500
## Meds                          1.288170  1        1.134976
## bs(D_Age, degree = 1, df = 2) 1.364581  2        1.080811  
 
 
 
 Create ROCR for training and test data 
  ## training data
pred.mtt = predict(m, type = "response") #repeat risk predictions from model m
rocr.pred.mtt = ROCR::prediction(pred.mtt, labels = ml_train$Any_Health_Problem) #ROCR prediction object
roc.perf.mtt = ROCR::performance(rocr.pred.mtt, measure = "tpr", x.measure = "fpr") # #ROCR performance object
plot(roc.perf.mtt, col = "blue")


pred.te.1 = predict(m, newdata = ml_test, type = "response") #.te = "test"
rocr.pred.te.1 = ROCR::prediction(pred.te.1, labels = ml_test$Any_Health_Problem)
roc.perf.te.1 = ROCR::performance(rocr.pred.te.1, measure = "tpr", x.measure = "fpr")
plot(roc.perf.te.1, col = "red", add = T)

abline(a = 0, b = 1, lty = 2) #diagonal for random assignment
legend("bottomright", legend = c("train","test"),
col = c("blue","red"), lty = c(2,1), lwd =1.5)  
   
 
 
 Report AUC from ROC for training and test data 
    # Train AUC
aucTr <- ROCR::performance(rocr.pred.mtt, measure = "auc")
  aucTr <- aucTr@y.values[[1]]
  print(aucTr)  
  ## [1] 0.8425662  
     # Test AUC
  aucTe <- ROCR::performance(rocr.pred.te.1, measure = "auc")
  aucTe <- aucTe@y.values[[1]]
  print(aucTe)  
  ## [1] 0.8520415  
 
 
 
 
 MULTIPLE REGRESSION WITH BACKWARDS ELIMINATION 
 
 ROUND 4: Model with all variables 
 Try removing Urban 
  # fit binary logit model and store results 'm'
m <- glm(Any_Health_Problem ~  Education_S + Animal_Career_BINARY 
         + Income2 + C_Age2 + C_Gender + C_Diet + Size2 + D_Sex + D_Neuter  + D_Diet + Therapeutic_Food + Visits 
         + Meds + bs(D_Age,degree=1,df=2), data = ml_train,family = binomial)
# view a summary of the model
summary(m)  
  ## 
## Call:
## glm(formula = Any_Health_Problem ~ Education_S + Animal_Career_BINARY + 
##     Income2 + C_Age2 + C_Gender + C_Diet + Size2 + D_Sex + D_Neuter + 
##     D_Diet + Therapeutic_Food + Visits + Meds + bs(D_Age, degree = 1, 
##     df = 2), family = binomial, data = ml_train)
## 
## Coefficients:
##                                                         Estimate Std. Error
## (Intercept)                                             -2.37771    0.37127
## Education_S1_College                                     0.07590    0.19446
## Education_S2_Grad                                       -0.10629    0.19756
## Education_S3_PG_or_PhD                                  -0.32254    0.21116
## Animal_Career_BINARYYes                                 -0.20799    0.16665
## Income2Medium                                           -0.13794    0.17802
## Income2High                                             -0.38860    0.24297
## C_Age230–39                                             -0.15400    0.20767
## C_Age240–49                                             -0.28157    0.21370
## C_Age250–59                                             -0.58976    0.21545
## C_Age260<                                               -0.49012    0.22906
## C_GenderMale                                            -0.33031    0.26046
## C_DietOmnivore reducing animal product consumption       0.06631    0.16892
## C_DietPescatarian (including fish but no other meats)    0.43467    0.28655
## C_DietVegan (consuming no animal products)              -0.09794    0.22605
## C_DietVegetarian (consuming plants, eggs and milk)      -0.07478    0.22485
## Size2Toy                                                 0.44740    0.41984
## Size2Small                                               0.14984    0.17561
## Size2Large                                               0.09711    0.15034
## Size2Giant                                               0.73849    0.30922
## D_SexMale                                               -0.08460    0.12910
## D_NeuterNeutered                                         0.05066    0.16797
## D_DietMeat-based – raw                                  -0.13569    0.14538
## D_DietVegan (consuming no animal products)              -0.40636    0.26465
## D_DietVegetarian (including eggs or milk, but not meat) -0.17837    0.50924
## Therapeutic_FoodYes                                      0.89688    0.35894
## Visits1                                                  0.16177    0.20719
## Visits2                                                  1.06480    0.22750
## Visits3                                                  0.95379    0.28690
## Visits3<                                                 1.55849    0.28235
## MedsYes                                                  1.83854    0.14332
## bs(D_Age, degree = 1, df = 2)1                           0.82127    0.24292
## bs(D_Age, degree = 1, df = 2)2                           3.50573    0.37434
##                                                         z value Pr(>|z|)    
## (Intercept)                                              -6.404 1.51e-10 ***
## Education_S1_College                                      0.390 0.696324    
## Education_S2_Grad                                        -0.538 0.590554    
## Education_S3_PG_or_PhD                                   -1.527 0.126642    
## Animal_Career_BINARYYes                                  -1.248 0.212000    
## Income2Medium                                            -0.775 0.438435    
## Income2High                                              -1.599 0.109744    
## C_Age230–39                                              -0.742 0.458348    
## C_Age240–49                                              -1.318 0.187640    
## C_Age250–59                                              -2.737 0.006195 ** 
## C_Age260<                                                -2.140 0.032379 *  
## C_GenderMale                                             -1.268 0.204731    
## C_DietOmnivore reducing animal product consumption        0.393 0.694675    
## C_DietPescatarian (including fish but no other meats)     1.517 0.129295    
## C_DietVegan (consuming no animal products)               -0.433 0.664822    
## C_DietVegetarian (consuming plants, eggs and milk)       -0.333 0.739438    
## Size2Toy                                                  1.066 0.286587    
## Size2Small                                                0.853 0.393524    
## Size2Large                                                0.646 0.518314    
## Size2Giant                                                2.388 0.016929 *  
## D_SexMale                                                -0.655 0.512266    
## D_NeuterNeutered                                          0.302 0.762964    
## D_DietMeat-based – raw                                   -0.933 0.350643    
## D_DietVegan (consuming no animal products)               -1.535 0.124665    
## D_DietVegetarian (including eggs or milk, but not meat)  -0.350 0.726138    
## Therapeutic_FoodYes                                       2.499 0.012465 *  
## Visits1                                                   0.781 0.434925    
## Visits2                                                   4.680 2.86e-06 ***
## Visits3                                                   3.324 0.000886 ***
## Visits3<                                                  5.520 3.40e-08 ***
## MedsYes                                                  12.828  < 2e-16 ***
## bs(D_Age, degree = 1, df = 2)1                            3.381 0.000723 ***
## bs(D_Age, degree = 1, df = 2)2                            9.365  < 2e-16 ***
## ---
## Signif. codes:  0 '***' 0.001 '**' 0.01 '*' 0.05 '.' 0.1 ' ' 1
## 
## (Dispersion parameter for binomial family taken to be 1)
## 
##     Null deviance: 2200.0  on 1657  degrees of freedom
## Residual deviance: 1555.2  on 1625  degrees of freedom
## AIC: 1621.2
## 
## Number of Fisher Scoring iterations: 4  
  # test model fit
with(m, null.deviance - deviance)  
  ## [1] 644.8533  
  with(m, df.null - df.residual)  
  ## [1] 32  
  with(m, pchisq(null.deviance - deviance, df.null - df.residual, lower.tail = FALSE))  
  ## [1] 3.18032e-115  
  BIC(m)  
  ## [1] 1799.816  
  # Hosmer-Lemeshow Goodness-of-Fit Test
hltest(m)  
  ## 
##    The Hosmer-Lemeshow goodness-of-fit test
## 
##  Group Size Observed   Expected
##      1  166        9   9.688965
##      2  167       19  14.868367
##      3  166       17  20.338265
##      4  166       32  27.034026
##      5  166       38  37.344657
##      6  166       56  55.270363
##      7  166       67  82.383745
##      8  166      108 106.384821
##      9  166      135 127.774093
##     10  163      147 146.912698
## 
##          Statistic =  10.60206 
## degrees of freedom =  8 
##            p-value =  0.22528  
  ## CIs using profiled log-likelihood
confint(m, level=0.99)  
  ## Waiting for profiling to be done...  
  ##                                                               0.5 %      99.5 %
## (Intercept)                                             -3.34949631 -1.43384525
## Education_S1_College                                    -0.42375256  0.57940741
## Education_S2_Grad                                       -0.61457389  0.40457093
## Education_S3_PG_or_PhD                                  -0.86766217  0.22158084
## Animal_Career_BINARYYes                                 -0.64118588  0.21845107
## Income2Medium                                           -0.59459277  0.32387784
## Income2High                                             -1.01732428  0.23614176
## C_Age230–39                                             -0.68964387  0.38156161
## C_Age240–49                                             -0.83345362  0.26887455
## C_Age250–59                                             -1.14742462 -0.03599738
## C_Age260<                                               -1.08307623  0.09852844
## C_GenderMale                                            -1.02080860  0.32579059
## C_DietOmnivore reducing animal product consumption      -0.37075174  0.50055598
## C_DietPescatarian (including fish but no other meats)   -0.30469834  1.17470136
## C_DietVegan (consuming no animal products)              -0.68495926  0.48136625
## C_DietVegetarian (consuming plants, eggs and milk)      -0.65964948  0.50077414
## Size2Toy                                                -0.67819426  1.50149810
## Size2Small                                              -0.30419369  0.60161106
## Size2Large                                              -0.29070646  0.48470177
## Size2Giant                                              -0.06984379  1.52882963
## D_SexMale                                               -0.41771532  0.24809904
## D_NeuterNeutered                                        -0.37932119  0.48736335
## D_DietMeat-based – raw                                  -0.51060356  0.23919660
## D_DietVegan (consuming no animal products)              -1.09258517  0.27326786
## D_DietVegetarian (including eggs or milk, but not meat) -1.52287681  1.12803972
## Therapeutic_FoodYes                                      0.01346141  1.87478051
## Visits1                                                 -0.36239736  0.70844863
## Visits2                                                  0.48706225  1.66204381
## Visits3                                                  0.22054265  1.70152352
## Visits3<                                                 0.84122373  2.29852882
## MedsYes                                                  1.47318296  2.21232140
## bs(D_Age, degree = 1, df = 2)1                           0.20126651  1.45434140
## bs(D_Age, degree = 1, df = 2)2                           2.55369202  4.48498221  
  ## CIs using standard errors
confint.default(m, level=0.99)  
  ##                                                               0.5 %      99.5 %
## (Intercept)                                             -3.33404495 -1.42137742
## Education_S1_College                                    -0.42500146  0.57679258
## Education_S2_Grad                                       -0.61517394  0.40258657
## Education_S3_PG_or_PhD                                  -0.86645339  0.22136907
## Animal_Career_BINARYYes                                 -0.63724573  0.22126531
## Income2Medium                                           -0.59649426  0.32061686
## Income2High                                             -1.01445728  0.23726017
## C_Age230–39                                             -0.68890930  0.38091214
## C_Age240–49                                             -0.83201936  0.26888377
## C_Age250–59                                             -1.14473248 -0.03478338
## C_Age260<                                               -1.08013675  0.09990074
## C_GenderMale                                            -1.00121386  0.34058873
## C_DietOmnivore reducing animal product consumption      -0.36881380  0.50142552
## C_DietPescatarian (including fish but no other meats)   -0.30344197  1.17277853
## C_DietVegan (consuming no animal products)              -0.68019860  0.48432131
## C_DietVegetarian (consuming plants, eggs and milk)      -0.65395283  0.50438526
## Size2Toy                                                -0.63404182  1.52884330
## Size2Small                                              -0.30249764  0.60216818
## Size2Large                                              -0.29014541  0.48437342
## Size2Giant                                              -0.05800643  1.53499397
## D_SexMale                                               -0.41715214  0.24794543
## D_NeuterNeutered                                        -0.38199507  0.48330926
## D_DietMeat-based – raw                                  -0.51014860  0.23877775
## D_DietVegan (consuming no animal products)              -1.08805233  0.27532567
## D_DietVegetarian (including eggs or milk, but not meat) -1.49007332  1.13333575
## Therapeutic_FoodYes                                     -0.02768602  1.82144164
## Visits1                                                 -0.37191642  0.69546333
## Visits2                                                  0.47880311  1.65080439
## Visits3                                                  0.21477641  1.69279945
## Visits3<                                                 0.83120435  2.28578353
## MedsYes                                                  1.46937644  2.20769499
## bs(D_Age, degree = 1, df = 2)1                           0.19555840  1.44699022
## bs(D_Age, degree = 1, df = 2)2                           2.54150374  4.46995741  
  # Wald test
wald.test(b = coef(m), Sigma = vcov(m), Terms = 2)  
  ## Wald test:
## ----------
## 
## Chi-squared test:
## X2 = 0.15, df = 1, P(> X2) = 0.7  
  ## odds ratios and 95% CI
exp(cbind(OR = coef(m), confint(m, level=0.99)))  
  ## Waiting for profiling to be done...  
  ##                                                                  OR       0.5 %
## (Intercept)                                              0.09276265  0.03510203
## Education_S1_College                                     1.07884990  0.65458583
## Education_S2_Grad                                        0.89916054  0.54087132
## Education_S3_PG_or_PhD                                   0.72430539  0.41993213
## Animal_Career_BINARYYes                                  0.81221499  0.52666749
## Income2Medium                                            0.87115209  0.55178722
## Income2High                                              0.67800640  0.36156108
## C_Age230–39                                              0.85727324  0.50175473
## C_Age240–49                                              0.75459976  0.43454594
## C_Age250–59                                              0.55446149  0.31745328
## C_Age260<                                                0.61255411  0.33855246
## C_GenderMale                                             0.71869905  0.36030348
## C_DietOmnivore reducing animal product consumption       1.06855349  0.69021527
## C_DietPescatarian (including fish but no other meats)    1.54445065  0.73734577
## C_DietVegan (consuming no animal products)               0.90670453  0.50411077
## C_DietVegetarian (consuming plants, eggs and milk)       0.92794410  0.51703253
## Size2Toy                                                 1.56424103  0.50753264
## Size2Small                                               1.16164287  0.73771796
## Size2Large                                               1.10198600  0.74773513
## Size2Giant                                               2.09278093  0.93253948
## D_SexMale                                                0.91887668  0.65854968
## D_NeuterNeutered                                         1.05196211  0.68432578
## D_DietMeat-based – raw                                   0.87311725  0.60013325
## D_DietVegan (consuming no animal products)               0.66606812  0.33534844
## D_DietVegetarian (including eggs or milk, but not meat)  0.83663383  0.21808360
## Therapeutic_FoodYes                                      2.45193573  1.01355243
## Visits1                                                  1.17559389  0.69600575
## Visits2                                                  2.90026976  1.62752792
## Visits3                                                  2.59552272  1.24675310
## Visits3<                                                 4.75165956  2.31920332
## MedsYes                                                  6.28732508  4.36310062
## bs(D_Age, degree = 1, df = 2)1                           2.27339501  1.22295066
## bs(D_Age, degree = 1, df = 2)2                          33.30576731 12.85447528
##                                                             99.5 %
## (Intercept)                                              0.2383905
## Education_S1_College                                     1.7849804
## Education_S2_Grad                                        1.4986593
## Education_S3_PG_or_PhD                                   1.2480481
## Animal_Career_BINARYYes                                  1.2441481
## Income2Medium                                            1.3824784
## Income2High                                              1.2663538
## C_Age230–39                                              1.4645699
## C_Age240–49                                              1.3084910
## C_Age250–59                                              0.9646428
## C_Age260<                                                1.1035458
## C_GenderMale                                             1.3851253
## C_DietOmnivore reducing animal product consumption       1.6496382
## C_DietPescatarian (including fish but no other meats)    3.2371760
## C_DietVegan (consuming no animal products)               1.6182839
## C_DietVegetarian (consuming plants, eggs and milk)       1.6499981
## Size2Toy                                                 4.4884081
## Size2Small                                               1.8250567
## Size2Large                                               1.6236907
## Size2Giant                                               4.6127750
## D_SexMale                                                1.2815868
## D_NeuterNeutered                                         1.6280180
## D_DietMeat-based – raw                                   1.2702282
## D_DietVegan (consuming no animal products)               1.3142522
## D_DietVegetarian (including eggs or milk, but not meat)  3.0895941
## Therapeutic_FoodYes                                      6.5193880
## Visits1                                                  2.0308382
## Visits2                                                  5.2700709
## Visits3                                                  5.4822934
## Visits3<                                                 9.9595194
## MedsYes                                                  9.1369022
## bs(D_Age, degree = 1, df = 2)1                           4.2816626
## bs(D_Age, degree = 1, df = 2)2                          88.6753737  
 
 Calculate Nagelkerke R^2 
  NagelkerkeR2(m)  
  ## $N
## [1] 1658
## 
## $R2
## [1] 0.4385717  
 
 
 check assumptions of model 
 
 Cook’s distance 
  plot(m, which = 4, id.n = 3)  
   
 
 
 Extract model results and display data for top 3 values using Cook’s
distance 
  model.data <- augment(m) %>% 
  mutate(index = 1:n()) 
model.data %>% top_n(3, .cooksd)  
 
 
 
 
 
 plot standardised residuals 
  ggplot(model.data, aes(index, .std.resid)) + 
  geom_point(aes(color = Any_Health_Problem), alpha = .5) +
  theme_bw()  
   
 
 
 Filter potential influential data points with abs(.std.res) >
3: 
  model.data %>% 
  filter(abs(.std.resid) > 3)  
 
 
 
 
 
 check for multicollinearity 
  car::vif(m)  
  ##                                   GVIF Df GVIF^(1/(2*Df))
## Education_S                   1.229937  3        1.035096
## Animal_Career_BINARY          1.103668  1        1.050556
## Income2                       1.145995  2        1.034655
## C_Age2                        1.370166  4        1.040152
## C_Gender                      1.055730  1        1.027487
## C_Diet                        2.077900  4        1.095729
## Size2                         1.136680  4        1.016143
## D_Sex                         1.043307  1        1.021424
## D_Neuter                      1.150670  1        1.072693
## D_Diet                        2.026891  3        1.124963
## Therapeutic_Food              1.035264  1        1.017479
## Visits                        1.426173  4        1.045374
## Meds                          1.286311  1        1.134157
## bs(D_Age, degree = 1, df = 2) 1.363125  2        1.080523  
 
 
 
 Create ROCR for training and test data 
  ## training data
pred.mtt = predict(m, type = "response") #repeat risk predictions from model m
rocr.pred.mtt = ROCR::prediction(pred.mtt, labels = ml_train$Any_Health_Problem) #ROCR prediction object
roc.perf.mtt = ROCR::performance(rocr.pred.mtt, measure = "tpr", x.measure = "fpr") # #ROCR performance object
plot(roc.perf.mtt, col = "blue")


pred.te.1 = predict(m, newdata = ml_test, type = "response") #.te = "test"
rocr.pred.te.1 = ROCR::prediction(pred.te.1, labels = ml_test$Any_Health_Problem)
roc.perf.te.1 = ROCR::performance(rocr.pred.te.1, measure = "tpr", x.measure = "fpr")
plot(roc.perf.te.1, col = "red", add = T)

abline(a = 0, b = 1, lty = 2) #diagonal for random assignment
legend("bottomright", legend = c("train","test"),
col = c("blue","red"), lty = c(2,1), lwd =1.5)  
   
 
 
 Report AUC from ROC for training and test data 
    # Train AUC
aucTr <- ROCR::performance(rocr.pred.mtt, measure = "auc")
  aucTr <- aucTr@y.values[[1]]
  print(aucTr)  
  ## [1] 0.8426311  
     # Test AUC
  aucTe <- ROCR::performance(rocr.pred.te.1, measure = "auc")
  aucTe <- aucTe@y.values[[1]]
  print(aucTe)  
  ## [1] 0.8524596  
 
 
 
 
 MULTIPLE REGRESSION WITH BACKWARDS ELIMINATION 
 
 ROUND 6: Model with all variables 
 Try removing C_Diet 
  # fit binary logit model and store results 'm'
m <- glm(Any_Health_Problem ~  Animal_Career_BINARY 
         + Income2 + C_Age2 + C_Gender + Size2 +D_Neuter  + D_Diet + Therapeutic_Food + Visits 
         + Meds + bs(D_Age,degree=1,df=2), data = ml_train,family = binomial)
# view a summary of the model
summary(m)  
  ## 
## Call:
## glm(formula = Any_Health_Problem ~ Animal_Career_BINARY + Income2 + 
##     C_Age2 + C_Gender + Size2 + D_Neuter + D_Diet + Therapeutic_Food + 
##     Visits + Meds + bs(D_Age, degree = 1, df = 2), family = binomial, 
##     data = ml_train)
## 
## Coefficients:
##                                                         Estimate Std. Error
## (Intercept)                                             -2.45360    0.33123
## Animal_Career_BINARYYes                                 -0.19228    0.16468
## Income2Medium                                           -0.19180    0.17574
## Income2High                                             -0.49424    0.23608
## C_Age230–39                                             -0.17755    0.20558
## C_Age240–49                                             -0.27674    0.21101
## C_Age250–59                                             -0.54705    0.21150
## C_Age260<                                               -0.39653    0.21953
## C_GenderMale                                            -0.35374    0.25964
## Size2Toy                                                 0.47887    0.41146
## Size2Small                                               0.15226    0.17485
## Size2Large                                               0.09575    0.14929
## Size2Giant                                               0.70214    0.30661
## D_NeuterNeutered                                         0.06787    0.16709
## D_DietMeat-based – raw                                  -0.10072    0.14388
## D_DietVegan (consuming no animal products)              -0.52245    0.20955
## D_DietVegetarian (including eggs or milk, but not meat) -0.26358    0.50076
## Therapeutic_FoodYes                                      0.88198    0.35259
## Visits1                                                  0.14502    0.20559
## Visits2                                                  1.05417    0.22636
## Visits3                                                  0.96864    0.28401
## Visits3<                                                 1.54446    0.28057
## MedsYes                                                  1.81798    0.14214
## bs(D_Age, degree = 1, df = 2)1                           0.82849    0.24154
## bs(D_Age, degree = 1, df = 2)2                           3.43623    0.36847
##                                                         z value Pr(>|z|)    
## (Intercept)                                              -7.408 1.29e-13 ***
## Animal_Career_BINARYYes                                  -1.168 0.242980    
## Income2Medium                                            -1.091 0.275103    
## Income2High                                              -2.094 0.036300 *  
## C_Age230–39                                              -0.864 0.387782    
## C_Age240–49                                              -1.311 0.189689    
## C_Age250–59                                              -2.587 0.009695 ** 
## C_Age260<                                                -1.806 0.070874 .  
## C_GenderMale                                             -1.362 0.173062    
## Size2Toy                                                  1.164 0.244496    
## Size2Small                                                0.871 0.383854    
## Size2Large                                                0.641 0.521283    
## Size2Giant                                                2.290 0.022023 *  
## D_NeuterNeutered                                          0.406 0.684604    
## D_DietMeat-based – raw                                   -0.700 0.483908    
## D_DietVegan (consuming no animal products)               -2.493 0.012661 *  
## D_DietVegetarian (including eggs or milk, but not meat)  -0.526 0.598633    
## Therapeutic_FoodYes                                       2.501 0.012370 *  
## Visits1                                                   0.705 0.480579    
## Visits2                                                   4.657 3.21e-06 ***
## Visits3                                                   3.411 0.000648 ***
## Visits3<                                                  5.505 3.70e-08 ***
## MedsYes                                                  12.790  < 2e-16 ***
## bs(D_Age, degree = 1, df = 2)1                            3.430 0.000603 ***
## bs(D_Age, degree = 1, df = 2)2                            9.326  < 2e-16 ***
## ---
## Signif. codes:  0 '***' 0.001 '**' 0.01 '*' 0.05 '.' 0.1 ' ' 1
## 
## (Dispersion parameter for binomial family taken to be 1)
## 
##     Null deviance: 2200.0  on 1657  degrees of freedom
## Residual deviance: 1563.8  on 1633  degrees of freedom
## AIC: 1613.8
## 
## Number of Fisher Scoring iterations: 4  
  # test model fit
with(m, null.deviance - deviance)  
  ## [1] 636.2399  
  with(m, df.null - df.residual)  
  ## [1] 24  
  with(m, pchisq(null.deviance - deviance, df.null - df.residual, lower.tail = FALSE))  
  ## [1] 6.093081e-119  
  BIC(m)  
  ## [1] 1749.123  
  # Hosmer-Lemeshow Goodness-of-Fit Test
hltest(m)  
  ## 
##    The Hosmer-Lemeshow goodness-of-fit test
## 
##  Group Size Observed  Expected
##      1  166       14  10.03940
##      2  166       15  15.18707
##      3  166       19  20.56867
##      4  166       24  26.85058
##      5  166       43  36.77065
##      6  166       58  56.02081
##      7  166       66  81.90388
##      8  166      104 106.64703
##      9  166      135 126.77597
##     10  164      150 147.23594
## 
##          Statistic =  12.66935 
## degrees of freedom =  8 
##            p-value =  0.12374  
  ## CIs using profiled log-likelihood
confint(m, level=0.99)  
  ## Waiting for profiling to be done...  
  ##                                                               0.5 %
## (Intercept)                                             -3.32278535
## Animal_Career_BINARYYes                                 -0.62027348
## Income2Medium                                           -0.64272029
## Income2High                                             -1.10578190
## C_Age230–39                                             -0.70792302
## C_Age240–49                                             -0.82172434
## C_Age250–59                                             -1.09409256
## C_Age260<                                               -0.96409543
## C_GenderMale                                            -1.04245310
## Size2Toy                                                -0.62272429
## Size2Small                                              -0.29976461
## Size2Large                                              -0.28936089
## Size2Giant                                              -0.09995361
## D_NeuterNeutered                                        -0.35979557
## D_DietMeat-based – raw                                  -0.47164708
## D_DietVegan (consuming no animal products)              -1.07076686
## D_DietVegetarian (including eggs or milk, but not meat) -1.58229908
## Therapeutic_FoodYes                                      0.01238530
## Visits1                                                 -0.37503960
## Visits2                                                  0.47938193
## Visits3                                                  0.24302427
## Visits3<                                                 0.83194028
## MedsYes                                                  1.45551728
## bs(D_Age, degree = 1, df = 2)1                           0.21218610
## bs(D_Age, degree = 1, df = 2)2                           2.49913154
##                                                               99.5 %
## (Intercept)                                             -1.613965821
## Animal_Career_BINARYYes                                  0.229213114
## Income2Medium                                            0.263982475
## Income2High                                              0.112096627
## C_Age230–39                                              0.352515867
## C_Age240–49                                              0.266705743
## C_Age250–59                                             -0.003097981
## C_Age260<                                                0.168318680
## C_GenderMale                                             0.299878877
## Size2Toy                                                 1.514235477
## Size2Small                                               0.602109152
## Size2Large                                               0.480598023
## Size2Giant                                               1.485450920
## D_NeuterNeutered                                         0.502360269
## D_DietMeat-based – raw                                   0.270439773
## D_DietVegan (consuming no animal products)               0.010777022
## D_DietVegetarian (including eggs or milk, but not meat)  1.023430221
## Therapeutic_FoodYes                                      1.841793458
## Visits1                                                  0.687563610
## Visits2                                                  1.648461083
## Visits3                                                  1.709089652
## Visits3<                                                 2.280034674
## MedsYes                                                  2.188549134
## bs(D_Age, degree = 1, df = 2)1                           1.458101356
## bs(D_Age, degree = 1, df = 2)2                           4.400146583  
  ## CIs using standard errors
confint.default(m, level=0.99)  
  ##                                                               0.5 %
## (Intercept)                                             -3.30679515
## Animal_Career_BINARYYes                                 -0.61646435
## Income2Medium                                           -0.64446708
## Income2High                                             -1.10233587
## C_Age230–39                                             -0.70709268
## C_Age240–49                                             -0.82026425
## C_Age250–59                                             -1.09183605
## C_Age260<                                               -0.96200271
## C_GenderMale                                            -1.02251905
## Size2Toy                                                -0.58098455
## Size2Small                                              -0.29812102
## Size2Large                                              -0.28879496
## Size2Giant                                              -0.08764801
## D_NeuterNeutered                                        -0.36253052
## D_DietMeat-based – raw                                  -0.47134093
## D_DietVegan (consuming no animal products)              -1.06222570
## D_DietVegetarian (including eggs or milk, but not meat) -1.55346025
## Therapeutic_FoodYes                                     -0.02623833
## Visits1                                                 -0.38454284
## Visits2                                                  0.47111597
## Visits3                                                  0.23707386
## Visits3<                                                 0.82175972
## MedsYes                                                  1.45186573
## bs(D_Age, degree = 1, df = 2)1                           0.20632972
## bs(D_Age, degree = 1, df = 2)2                           2.48710960
##                                                               99.5 %
## (Intercept)                                             -1.600414550
## Animal_Career_BINARYYes                                  0.231912727
## Income2Medium                                            0.260871971
## Income2High                                              0.113853866
## C_Age230–39                                              0.351993245
## C_Age240–49                                              0.266785778
## C_Age250–59                                             -0.002264573
## C_Age260<                                                0.168937192
## C_GenderMale                                             0.315043906
## Size2Toy                                                 1.538716025
## Size2Small                                               0.602645654
## Size2Large                                               0.480294222
## Size2Giant                                               1.491924020
## D_NeuterNeutered                                         0.498272404
## D_DietMeat-based – raw                                   0.269895799
## D_DietVegan (consuming no animal products)               0.017323776
## D_DietVegetarian (including eggs or milk, but not meat)  1.026290943
## Therapeutic_FoodYes                                      1.790206089
## Visits1                                                  0.674573827
## Visits2                                                  1.637221720
## Visits3                                                  1.700210347
## Visits3<                                                 2.267163434
## MedsYes                                                  2.184099501
## bs(D_Age, degree = 1, df = 2)1                           1.450659474
## bs(D_Age, degree = 1, df = 2)2                           4.385340524  
  # Wald test
wald.test(b = coef(m), Sigma = vcov(m), Terms = 2)  
  ## Wald test:
## ----------
## 
## Chi-squared test:
## X2 = 1.4, df = 1, P(> X2) = 0.24  
  ## odds ratios and 95% CI
exp(cbind(OR = coef(m), confint(m, level=0.99)))  
  ## Waiting for profiling to be done...  
  ##                                                                  OR       0.5 %
## (Intercept)                                              0.08598307  0.03605227
## Animal_Career_BINARYYes                                  0.82507927  0.53779734
## Income2Medium                                            0.82547396  0.52585999
## Income2High                                              0.61003374  0.33095201
## C_Age230–39                                              0.83731937  0.49266640
## C_Age240–49                                              0.75825219  0.43967286
## C_Age250–59                                              0.57865414  0.33484332
## C_Age260<                                                0.67264824  0.38132798
## C_GenderMale                                             0.70205918  0.35258869
## Size2Toy                                                 1.61424239  0.53648092
## Size2Small                                               1.16446565  0.74099262
## Size2Large                                               1.10048350  0.74874194
## Size2Giant                                               2.01806273  0.90487939
## D_NeuterNeutered                                         1.07022718  0.69781896
## D_DietMeat-based – raw                                   0.90418385  0.62397369
## D_DietVegan (consuming no animal products)               0.59306518  0.34274558
## D_DietVegetarian (including eggs or milk, but not meat)  0.76829258  0.20550209
## Therapeutic_FoodYes                                      2.41568739  1.01246232
## Visits1                                                  1.15605748  0.68726206
## Visits2                                                  2.86958909  1.61507586
## Visits3                                                  2.63436483  1.27509957
## Visits3<                                                 4.68544819  2.29777274
## MedsYes                                                  6.15941997  4.28670030
## bs(D_Age, degree = 1, df = 2)1                           2.28986896  1.23637796
## bs(D_Age, degree = 1, df = 2)2                          31.06945131 12.17191861
##                                                             99.5 %
## (Intercept)                                              0.1990965
## Animal_Career_BINARYYes                                  1.2576100
## Income2Medium                                            1.3021054
## Income2High                                              1.1186209
## C_Age230–39                                              1.4226422
## C_Age240–49                                              1.3056562
## C_Age250–59                                              0.9969068
## C_Age260<                                                1.1833136
## C_GenderMale                                             1.3496953
## Size2Toy                                                 4.5459443
## Size2Small                                               1.8259660
## Size2Large                                               1.6170411
## Size2Giant                                               4.4169567
## D_NeuterNeutered                                         1.6526173
## D_DietMeat-based – raw                                   1.3105407
## D_DietVegan (consuming no animal products)               1.0108353
## D_DietVegetarian (including eggs or milk, but not meat)  2.7827238
## Therapeutic_FoodYes                                      6.3078410
## Visits1                                                  1.9888640
## Visits2                                                  5.1989729
## Visits3                                                  5.5239305
## Visits3<                                                 9.7770194
## MedsYes                                                  8.9222587
## bs(D_Age, degree = 1, df = 2)1                           4.2977918
## bs(D_Age, degree = 1, df = 2)2                          81.4628088  
 
 Calculate Nagelkerke R^2 
  NagelkerkeR2(m)  
  ## $N
## [1] 1658
## 
## $R2
## [1] 0.4337667  
 
 
 check assumptions of model 
 
 Cook’s distance 
  plot(m, which = 4, id.n = 3)  
   
 
 
 Extract model results and display data for top 3 values using Cook’s
distance 
  model.data <- augment(m) %>% 
  mutate(index = 1:n()) 
model.data %>% top_n(3, .cooksd)  
 
 
 
 
 
 plot standardised residuals 
  ggplot(model.data, aes(index, .std.resid)) + 
  geom_point(aes(color = Any_Health_Problem), alpha = .5) +
  theme_bw()  
   
 
 
 Filter potential influential data points with abs(.std.res) >
3: 
  model.data %>% 
  filter(abs(.std.resid) > 3)  
 
 
 
 
 
 check for multicollinearity 
  car::vif(m)  
  ##                                   GVIF Df GVIF^(1/(2*Df))
## Animal_Career_BINARY          1.089934  1        1.043999
## Income2                       1.084043  2        1.020379
## C_Age2                        1.208686  4        1.023975
## C_Gender                      1.044421  1        1.021969
## Size2                         1.104661  4        1.012520
## D_Neuter                      1.144910  1        1.070005
## D_Diet                        1.182871  3        1.028386
## Therapeutic_Food              1.030315  1        1.015044
## Visits                        1.388997  4        1.041928
## Meds                          1.273345  1        1.128426
## bs(D_Age, degree = 1, df = 2) 1.320350  2        1.071944  
 
 
 
 Create ROCR for training and test data 
  ## training data
pred.mtt = predict(m, type = "response") #repeat risk predictions from model m
rocr.pred.mtt = ROCR::prediction(pred.mtt, labels = ml_train$Any_Health_Problem) #ROCR prediction object
roc.perf.mtt = ROCR::performance(rocr.pred.mtt, measure = "tpr", x.measure = "fpr") # #ROCR performance object
plot(roc.perf.mtt, col = "blue")


pred.te.1 = predict(m, newdata = ml_test, type = "response") #.te = "test"
rocr.pred.te.1 = ROCR::prediction(pred.te.1, labels = ml_test$Any_Health_Problem)
roc.perf.te.1 = ROCR::performance(rocr.pred.te.1, measure = "tpr", x.measure = "fpr")
plot(roc.perf.te.1, col = "red", add = T)

abline(a = 0, b = 1, lty = 2) #diagonal for random assignment
legend("bottomright", legend = c("train","test"),
col = c("blue","red"), lty = c(2,1), lwd =1.5)  
   
 
 
 Report AUC from ROC for training and test data 
    # Train AUC
aucTr <- ROCR::performance(rocr.pred.mtt, measure = "auc")
  aucTr <- aucTr@y.values[[1]]
  print(aucTr)  
  ## [1] 0.8401792  
     # Test AUC
  aucTe <- ROCR::performance(rocr.pred.te.1, measure = "auc")
  aucTe <- aucTe@y.values[[1]]
  print(aucTe)  
  ## [1] 0.8540552  
 
 
 
 
 MULTIPLE REGRESSION WITH BACKWARDS ELIMINATION 
 
 ROUND 7: Model with all variables 
 Try removing Animal_Career_BINARY 
  # fit binary logit model and store results 'm'
m <- glm(Any_Health_Problem ~  Income2 + C_Age2 + C_Gender + Size2 +D_Neuter  + D_Diet + Therapeutic_Food + Visits 
         + Meds + bs(D_Age,degree=1,df=2), data = ml_train,family = binomial)
# view a summary of the model
summary(m)  
  ## 
## Call:
## glm(formula = Any_Health_Problem ~ Income2 + C_Age2 + C_Gender + 
##     Size2 + D_Neuter + D_Diet + Therapeutic_Food + Visits + Meds + 
##     bs(D_Age, degree = 1, df = 2), family = binomial, data = ml_train)
## 
## Coefficients:
##                                                         Estimate Std. Error
## (Intercept)                                             -2.52704    0.32556
## Income2Medium                                           -0.17588    0.17512
## Income2High                                             -0.47577    0.23539
## C_Age230–39                                             -0.16649    0.20523
## C_Age240–49                                             -0.24656    0.20927
## C_Age250–59                                             -0.50721    0.20858
## C_Age260<                                               -0.34723    0.21527
## C_GenderMale                                            -0.32826    0.25829
## Size2Toy                                                 0.46504    0.41091
## Size2Small                                               0.14987    0.17475
## Size2Large                                               0.09761    0.14925
## Size2Giant                                               0.69923    0.30594
## D_NeuterNeutered                                         0.07582    0.16690
## D_DietMeat-based – raw                                  -0.09855    0.14389
## D_DietVegan (consuming no animal products)              -0.49112    0.20759
## D_DietVegetarian (including eggs or milk, but not meat) -0.24450    0.49877
## Therapeutic_FoodYes                                      0.89286    0.35346
## Visits1                                                  0.15095    0.20537
## Visits2                                                  1.05836    0.22616
## Visits3                                                  0.98313    0.28371
## Visits3<                                                 1.54438    0.28033
## MedsYes                                                  1.81233    0.14193
## bs(D_Age, degree = 1, df = 2)1                           0.80545    0.24070
## bs(D_Age, degree = 1, df = 2)2                           3.39748    0.36652
##                                                         z value Pr(>|z|)    
## (Intercept)                                              -7.762 8.35e-15 ***
## Income2Medium                                            -1.004 0.315221    
## Income2High                                              -2.021 0.043256 *  
## C_Age230–39                                              -0.811 0.417213    
## C_Age240–49                                              -1.178 0.238724    
## C_Age250–59                                              -2.432 0.015025 *  
## C_Age260<                                                -1.613 0.106748    
## C_GenderMale                                             -1.271 0.203777    
## Size2Toy                                                  1.132 0.257742    
## Size2Small                                                0.858 0.391108    
## Size2Large                                                0.654 0.513100    
## Size2Giant                                                2.286 0.022283 *  
## D_NeuterNeutered                                          0.454 0.649640    
## D_DietMeat-based – raw                                   -0.685 0.493406    
## D_DietVegan (consuming no animal products)               -2.366 0.017988 *  
## D_DietVegetarian (including eggs or milk, but not meat)  -0.490 0.623989    
## Therapeutic_FoodYes                                       2.526 0.011536 *  
## Visits1                                                   0.735 0.462342    
## Visits2                                                   4.680 2.87e-06 ***
## Visits3                                                   3.465 0.000530 ***
## Visits3<                                                  5.509 3.60e-08 ***
## MedsYes                                                  12.769  < 2e-16 ***
## bs(D_Age, degree = 1, df = 2)1                            3.346 0.000819 ***
## bs(D_Age, degree = 1, df = 2)2                            9.270  < 2e-16 ***
## ---
## Signif. codes:  0 '***' 0.001 '**' 0.01 '*' 0.05 '.' 0.1 ' ' 1
## 
## (Dispersion parameter for binomial family taken to be 1)
## 
##     Null deviance: 2200.0  on 1657  degrees of freedom
## Residual deviance: 1565.2  on 1634  degrees of freedom
## AIC: 1613.2
## 
## Number of Fisher Scoring iterations: 4  
  # test model fit
with(m, null.deviance - deviance)  
  ## [1] 634.868  
  with(m, df.null - df.residual)  
  ## [1] 23  
  with(m, pchisq(null.deviance - deviance, df.null - df.residual, lower.tail = FALSE))  
  ## [1] 2.221117e-119  
  BIC(m)  
  ## [1] 1743.081  
  # Hosmer-Lemeshow Goodness-of-Fit Test
hltest(m)  
  ## 
##    The Hosmer-Lemeshow goodness-of-fit test
## 
##  Group Size Observed  Expected
##      1  166       12  10.15624
##      2  166       18  15.28253
##      3  166       23  20.61440
##      4  166       23  26.77162
##      5  166       41  36.78634
##      6  166       57  56.24111
##      7  166       68  81.75368
##      8  166      102 106.24646
##      9  166      135 126.94611
##     10  164      149 147.20152
## 
##          Statistic =  9.89011 
## degrees of freedom =  8 
##            p-value =  0.27282  
  ## CIs using profiled log-likelihood
confint(m, level=0.99)  
  ## Waiting for profiling to be done...  
  ##                                                               0.5 %      99.5 %
## (Intercept)                                             -3.38206637 -1.70263938
## Income2Medium                                           -0.62509351  0.27842437
## Income2High                                             -1.08543479  0.12888515
## C_Age230–39                                             -0.69587973  0.36272741
## C_Age240–49                                             -0.78688760  0.29256867
## C_Age250–59                                             -1.04649954  0.02941906
## C_Age260<                                               -0.90351069  0.20692383
## C_GenderMale                                            -1.01351087  0.32200995
## Size2Toy                                                -0.63504984  1.49887063
## Size2Small                                              -0.30191605  0.59946007
## Size2Large                                              -0.28738146  0.48237841
## Size2Giant                                              -0.10095092  1.48097594
## D_NeuterNeutered                                        -0.35133248  0.50984819
## D_DietMeat-based – raw                                  -0.46951080  0.27263323
## D_DietVegan (consuming no animal products)              -1.03418072  0.03721018
## D_DietVegetarian (including eggs or milk, but not meat) -1.55877707  1.03683972
## Therapeutic_FoodYes                                      0.02069753  1.85434584
## Visits1                                                 -0.36851159  0.69295971
## Visits2                                                  0.48409250  1.65218028
## Visits3                                                  0.25838372  1.72287551
## Visits3<                                                 0.83247525  2.27930488
## MedsYes                                                  1.45036270  2.18231854
## bs(D_Age, degree = 1, df = 2)1                           0.19125822  1.43282748
## bs(D_Age, degree = 1, df = 2)2                           2.46506773  4.35594325  
  ## CIs using standard errors
confint.default(m, level=0.99)  
  ##                                                               0.5 %      99.5 %
## (Intercept)                                             -3.36562716 -1.68845807
## Income2Medium                                           -0.62696152  0.27520328
## Income2High                                             -1.08208702  0.13054515
## C_Age230–39                                             -0.69512407  0.36213619
## C_Age240–49                                             -0.78559728  0.29248378
## C_Age250–59                                             -1.04445987  0.03004746
## C_Age260<                                               -0.90172331  0.20727070
## C_GenderMale                                            -0.99357991  0.33706490
## Size2Toy                                                -0.59338349  1.52346320
## Size2Small                                              -0.30026488  0.60000566
## Size2Large                                              -0.28683262  0.48205890
## Size2Giant                                              -0.08882316  1.48727895
## D_NeuterNeutered                                        -0.35409974  0.50573682
## D_DietMeat-based – raw                                  -0.46920084  0.27209373
## D_DietVegan (consuming no animal products)              -1.02582364  0.04358349
## D_DietVegetarian (including eggs or milk, but not meat) -1.52924167  1.04024424
## Therapeutic_FoodYes                                     -0.01759991  1.80331236
## Visits1                                                 -0.37805006  0.67994083
## Visits2                                                  0.47579922  1.64091960
## Visits3                                                  0.25234029  1.71391592
## Visits3<                                                 0.82231015  2.26645044
## MedsYes                                                  1.44675026  2.17791215
## bs(D_Age, degree = 1, df = 2)1                           0.18544753  1.42544845
## bs(D_Age, degree = 1, df = 2)2                           2.45339364  4.34156091  
  # Wald test
wald.test(b = coef(m), Sigma = vcov(m), Terms = 2)  
  ## Wald test:
## ----------
## 
## Chi-squared test:
## X2 = 1.0, df = 1, P(> X2) = 0.32  
  ## odds ratios and 95% CI
exp(cbind(OR = coef(m), confint(m, level=0.99)))  
  ## Waiting for profiling to be done...  
  ##                                                                  OR       0.5 %
## (Intercept)                                              0.07989495  0.03397717
## Income2Medium                                            0.83871936  0.53521138
## Income2High                                              0.62140581  0.33775490
## C_Age230–39                                              0.84662795  0.49863559
## C_Age240–49                                              0.78148701  0.45525954
## C_Age250–59                                              0.60217559  0.35116484
## C_Age260<                                                0.70664539  0.40514482
## C_GenderMale                                             0.72017755  0.36294250
## Size2Toy                                                 1.59207764  0.52990908
## Size2Small                                               1.16168366  0.73940014
## Size2Large                                               1.10253617  0.75022550
## Size2Giant                                               2.01219848  0.90397740
## D_NeuterNeutered                                         1.07876680  0.70374973
## D_DietMeat-based – raw                                   0.90614716  0.62530809
## D_DietVegan (consuming no animal products)               0.61194059  0.35551753
## D_DietVegetarian (including eggs or milk, but not meat)  0.78309699  0.21039321
## Therapeutic_FoodYes                                      2.44209487  1.02091321
## Visits1                                                  1.16293315  0.69176319
## Visits2                                                  2.88163951  1.62270174
## Visits3                                                  2.67280399  1.29483558
## Visits3<                                                 4.68506737  2.29900232
## MedsYes                                                  6.12470875  4.26466105
## bs(D_Age, degree = 1, df = 2)1                           2.23769875  1.21077206
## bs(D_Age, degree = 1, df = 2)2                          29.88860404 11.76427897
##                                                            99.5 %
## (Intercept)                                              0.182202
## Income2Medium                                            1.321047
## Income2High                                              1.137559
## C_Age230–39                                              1.437244
## C_Age240–49                                              1.339865
## C_Age250–59                                              1.029856
## C_Age260<                                                1.229889
## C_GenderMale                                             1.379899
## Size2Toy                                                 4.476630
## Size2Small                                               1.821135
## Size2Large                                               1.619923
## Size2Giant                                               4.397235
## D_NeuterNeutered                                         1.665038
## D_DietMeat-based – raw                                   1.313418
## D_DietVegan (consuming no animal products)               1.037911
## D_DietVegetarian (including eggs or milk, but not meat)  2.820290
## Therapeutic_FoodYes                                      6.387518
## Visits1                                                  1.999625
## Visits2                                                  5.218345
## Visits3                                                  5.600610
## Visits3<                                                 9.769887
## MedsYes                                                  8.866841
## bs(D_Age, degree = 1, df = 2)1                           4.190531
## bs(D_Age, degree = 1, df = 2)2                          77.940308  
 
 Calculate Nagelkerke R^2 
  NagelkerkeR2(m)  
  ## $N
## [1] 1658
## 
## $R2
## [1] 0.432999  
 
 
 check assumptions of model 
 
 Cook’s distance 
  plot(m, which = 4, id.n = 3)  
   
 
 
 Extract model results and display data for top 3 values using Cook’s
distance 
  model.data <- augment(m) %>% 
  mutate(index = 1:n()) 
model.data %>% top_n(3, .cooksd)  
 
 
 
 
 
 plot standardised residuals 
  ggplot(model.data, aes(index, .std.resid)) + 
  geom_point(aes(color = Any_Health_Problem), alpha = .5) +
  theme_bw()  
   
 
 
 Filter potential influential data points with abs(.std.res) >
3: 
  model.data %>% 
  filter(abs(.std.resid) > 3)  
 
 
 
 
 
 check for multicollinearity 
  car::vif(m)  
  ##                                   GVIF Df GVIF^(1/(2*Df))
## Income2                       1.076476  2        1.018594
## C_Age2                        1.146559  4        1.017243
## C_Gender                      1.037290  1        1.018475
## Size2                         1.103136  4        1.012345
## D_Neuter                      1.144352  1        1.069744
## D_Diet                        1.161798  3        1.025310
## Therapeutic_Food              1.029478  1        1.014632
## Visits                        1.384226  4        1.041480
## Meds                          1.270879  1        1.127333
## bs(D_Age, degree = 1, df = 2) 1.302436  2        1.068290  
 
 
 
 Create ROCR for training and test data 
  ## training data
pred.mtt = predict(m, type = "response") #repeat risk predictions from model m
rocr.pred.mtt = ROCR::prediction(pred.mtt, labels = ml_train$Any_Health_Problem) #ROCR prediction object
roc.perf.mtt = ROCR::performance(rocr.pred.mtt, measure = "tpr", x.measure = "fpr") # #ROCR performance object
plot(roc.perf.mtt, col = "blue")


pred.te.1 = predict(m, newdata = ml_test, type = "response") #.te = "test"
rocr.pred.te.1 = ROCR::prediction(pred.te.1, labels = ml_test$Any_Health_Problem)
roc.perf.te.1 = ROCR::performance(rocr.pred.te.1, measure = "tpr", x.measure = "fpr")
plot(roc.perf.te.1, col = "red", add = T)

abline(a = 0, b = 1, lty = 2) #diagonal for random assignment
legend("bottomright", legend = c("train","test"),
col = c("blue","red"), lty = c(2,1), lwd =1.5)  
   
 
 
 Report AUC from ROC for training and test data 
    # Train AUC
aucTr <- ROCR::performance(rocr.pred.mtt, measure = "auc")
  aucTr <- aucTr@y.values[[1]]
  print(aucTr)  
  ## [1] 0.8397502  
     # Test AUC
  aucTe <- ROCR::performance(rocr.pred.te.1, measure = "auc")
  aucTe <- aucTe@y.values[[1]]
  print(aucTe)  
  ## [1] 0.8546823  
 
 
 
 
 MULTIPLE REGRESSION WITH BACKWARDS ELIMINATION 
 
 ROUND 7b: Model with all variables 
 Replace Size2 with Size_Giant 
  # fit binary logit model and store results 'm'
m <- glm(Any_Health_Problem ~  Income2 + C_Age2 + C_Gender + Size_Giant + D_Diet + Therapeutic_Food + Visits 
         + Meds + bs(D_Age,degree=1,df=2), data = ml_train,family = binomial)
# view a summary of the model
summary(m)  
  ## 
## Call:
## glm(formula = Any_Health_Problem ~ Income2 + C_Age2 + C_Gender + 
##     Size_Giant + D_Diet + Therapeutic_Food + Visits + Meds + 
##     bs(D_Age, degree = 1, df = 2), family = binomial, data = ml_train)
## 
## Coefficients:
##                                                         Estimate Std. Error
## (Intercept)                                              -2.4125     0.3095
## Income2Medium                                            -0.1781     0.1747
## Income2High                                              -0.4765     0.2348
## C_Age230–39                                              -0.1646     0.2049
## C_Age240–49                                              -0.2514     0.2090
## C_Age250–59                                              -0.5135     0.2080
## C_Age260<                                                -0.3469     0.2147
## C_GenderMale                                             -0.3519     0.2575
## Size_GiantYes                                             0.6072     0.2935
## D_DietMeat-based – raw                                   -0.1047     0.1434
## D_DietVegan (consuming no animal products)               -0.4849     0.2075
## D_DietVegetarian (including eggs or milk, but not meat)  -0.2111     0.4983
## Therapeutic_FoodYes                                       0.8973     0.3536
## Visits1                                                   0.1627     0.2051
## Visits2                                                   1.0569     0.2260
## Visits3                                                   0.9920     0.2832
## Visits3<                                                  1.5567     0.2795
## MedsYes                                                   1.8174     0.1413
## bs(D_Age, degree = 1, df = 2)1                            0.8314     0.2333
## bs(D_Age, degree = 1, df = 2)2                            3.4359     0.3599
##                                                         z value Pr(>|z|)    
## (Intercept)                                              -7.796 6.39e-15 ***
## Income2Medium                                            -1.019 0.308010    
## Income2High                                              -2.029 0.042416 *  
## C_Age230–39                                              -0.803 0.421939    
## C_Age240–49                                              -1.203 0.229038    
## C_Age250–59                                              -2.469 0.013549 *  
## C_Age260<                                                -1.616 0.106125    
## C_GenderMale                                             -1.366 0.171799    
## Size_GiantYes                                             2.069 0.038546 *  
## D_DietMeat-based – raw                                   -0.731 0.465062    
## D_DietVegan (consuming no animal products)               -2.337 0.019433 *  
## D_DietVegetarian (including eggs or milk, but not meat)  -0.424 0.671827    
## Therapeutic_FoodYes                                       2.537 0.011169 *  
## Visits1                                                   0.794 0.427473    
## Visits2                                                   4.676 2.93e-06 ***
## Visits3                                                   3.503 0.000460 ***
## Visits3<                                                  5.570 2.55e-08 ***
## MedsYes                                                  12.862  < 2e-16 ***
## bs(D_Age, degree = 1, df = 2)1                            3.564 0.000365 ***
## bs(D_Age, degree = 1, df = 2)2                            9.547  < 2e-16 ***
## ---
## Signif. codes:  0 '***' 0.001 '**' 0.01 '*' 0.05 '.' 0.1 ' ' 1
## 
## (Dispersion parameter for binomial family taken to be 1)
## 
##     Null deviance: 2200.0  on 1657  degrees of freedom
## Residual deviance: 1567.1  on 1638  degrees of freedom
## AIC: 1607.1
## 
## Number of Fisher Scoring iterations: 4  
  # test model fit
with(m, null.deviance - deviance)  
  ## [1] 632.9038  
  with(m, df.null - df.residual)  
  ## [1] 19  
  with(m, pchisq(null.deviance - deviance, df.null - df.residual, lower.tail = FALSE))  
  ## [1] 5.681626e-122  
  BIC(m)  
  ## [1] 1715.392  
  # Hosmer-Lemeshow Goodness-of-Fit Test
hltest(m)  
  ## 
##    The Hosmer-Lemeshow goodness-of-fit test
## 
##  Group Size Observed  Expected
##      1  166       12  10.29234
##      2  167       21  15.49616
##      3  167       21  20.69958
##      4  166       26  26.77944
##      5  166       40  37.41247
##      6  166       55  56.22746
##      7  165       65  81.51970
##      8  166      105 106.34726
##      9  166      132 126.89582
##     10  163      151 146.32978
## 
##          Statistic =  11.75357 
## degrees of freedom =  8 
##            p-value =  0.16254  
  ## CIs using profiled log-likelihood
confint(m, level=0.99)  
  ## Waiting for profiling to be done...  
  ##                                                               0.5 %      99.5 %
## (Intercept)                                             -3.22600969 -1.62962722
## Income2Medium                                           -0.62628915  0.27518486
## Income2High                                             -1.08463875  0.12656701
## C_Age230–39                                             -0.69325309  0.36391179
## C_Age240–49                                             -0.79104903  0.28701982
## C_Age250–59                                             -1.05119557  0.02160971
## C_Age260<                                               -0.90157163  0.20574449
## C_GenderMale                                            -1.03528691  0.29609997
## Size_GiantYes                                           -0.16225072  1.35603800
## D_DietMeat-based – raw                                  -0.47433099  0.26501374
## D_DietVegan (consuming no animal products)              -1.02768420  0.04316951
## D_DietVegetarian (including eggs or milk, but not meat) -1.52544828  1.06848726
## Therapeutic_FoodYes                                      0.02420041  1.85835443
## Visits1                                                 -0.35580022  0.70402881
## Visits2                                                  0.48299178  1.65037038
## Visits3                                                  0.26870932  1.73035637
## Visits3<                                                 0.84704618  2.28954598
## MedsYes                                                  1.45703661  2.18574022
## bs(D_Age, degree = 1, df = 2)1                           0.23652681  1.43986693
## bs(D_Age, degree = 1, df = 2)2                           2.52040813  4.37728346  
  ## CIs using standard errors
confint.default(m, level=0.99)  
  ##                                                               0.5 %      99.5 %
## (Intercept)                                             -3.20959761 -1.61539663
## Income2Medium                                           -0.62817514  0.27194488
## Income2High                                             -1.08123315  0.12828911
## C_Age230–39                                             -0.69249592  0.36332442
## C_Age240–49                                             -0.78974015  0.28695418
## C_Age250–59                                             -1.04917757  0.02221882
## C_Age260<                                               -0.89980201  0.20607122
## C_GenderMale                                            -1.01510991  0.31140801
## Size_GiantYes                                           -0.14874884  1.36319853
## D_DietMeat-based – raw                                  -0.47397323  0.26452567
## D_DietVegan (consuming no animal products)              -1.01935596  0.04953037
## D_DietVegetarian (including eggs or milk, but not meat) -1.49473281  1.07250437
## Therapeutic_FoodYes                                     -0.01359876  1.80812733
## Visits1                                                 -0.36546452  0.69089050
## Visits2                                                  0.47468291  1.63909029
## Visits3                                                  0.26259339  1.72131477
## Visits3<                                                 0.83680245  2.27664256
## MedsYes                                                  1.45346509  2.18138327
## bs(D_Age, degree = 1, df = 2)1                           0.23053614  1.43230758
## bs(D_Age, degree = 1, df = 2)2                           2.50884729  4.36299120  
  # Wald test
wald.test(b = coef(m), Sigma = vcov(m), Terms = 2)  
  ## Wald test:
## ----------
## 
## Chi-squared test:
## X2 = 1.0, df = 1, P(> X2) = 0.31  
  ## odds ratios and 95% CI
exp(cbind(OR = coef(m), confint(m, level=0.99)))  
  ## Waiting for profiling to be done...  
  ##                                                                  OR       0.5 %
## (Intercept)                                              0.08959129  0.03971566
## Income2Medium                                            0.83684607  0.53457184
## Income2High                                              0.62097030  0.33802387
## C_Age230–39                                              0.84824502  0.49994705
## C_Age240–49                                              0.77771668  0.45336895
## C_Age250–59                                              0.59840986  0.34951962
## C_Age260<                                                0.70690047  0.40593118
## C_GenderMale                                             0.70338495  0.35512448
## Size_GiantYes                                            1.83533099  0.85022801
## D_DietMeat-based – raw                                   0.90057324  0.62230124
## D_DietVegan (consuming no animal products)               0.61575089  0.35783467
## D_DietVegetarian (including eggs or milk, but not meat)  0.80968158  0.21752352
## Therapeutic_FoodYes                                      2.45288354  1.02449561
## Visits1                                                  1.17669892  0.70061258
## Visits2                                                  2.87739854  1.62091659
## Visits3                                                  2.69649850  1.30827480
## Visits3<                                                 4.74324974  2.33274615
## MedsYes                                                  6.15598131  4.29321820
## bs(D_Age, degree = 1, df = 2)1                           2.29658184  1.26684151
## bs(D_Age, degree = 1, df = 2)2                          31.05995123 12.43367023
##                                                             99.5 %
## (Intercept)                                              0.1960026
## Income2Medium                                            1.3167741
## Income2High                                              1.1349255
## C_Age230–39                                              1.4389473
## C_Age240–49                                              1.3324506
## C_Age250–59                                              1.0218449
## C_Age260<                                                1.2284393
## C_GenderMale                                             1.3446046
## Size_GiantYes                                            3.8807871
## D_DietMeat-based – raw                                   1.3034489
## D_DietVegan (consuming no animal products)               1.0441149
## D_DietVegetarian (including eggs or milk, but not meat)  2.9109726
## Therapeutic_FoodYes                                      6.4131748
## Visits1                                                  2.0218821
## Visits2                                                  5.2089087
## Visits3                                                  5.6426644
## Visits3<                                                 9.8704553
## MedsYes                                                  8.8972320
## bs(D_Age, degree = 1, df = 2)1                           4.2201342
## bs(D_Age, degree = 1, df = 2)2                          79.6214448  
 
 Calculate Nagelkerke R^2 
  NagelkerkeR2(m)  
  ## $N
## [1] 1658
## 
## $R2
## [1] 0.4318989  
 
 
 check assumptions of model 
 
 Cook’s distance 
  plot(m, which = 4, id.n = 3)  
   
 
 
 Extract model results and display data for top 3 values using Cook’s
distance 
  model.data <- augment(m) %>% 
  mutate(index = 1:n()) 
model.data %>% top_n(3, .cooksd)  
 
 
 
 
 
 plot standardised residuals 
  ggplot(model.data, aes(index, .std.resid)) + 
  geom_point(aes(color = Any_Health_Problem), alpha = .5) +
  theme_bw()  
   
 
 
 Filter potential influential data points with abs(.std.res) >
3: 
  model.data %>% 
  filter(abs(.std.resid) > 3)  
 
 
 
 
 
 check for multicollinearity 
  car::vif(m)  
  ##                                   GVIF Df GVIF^(1/(2*Df))
## Income2                       1.073125  2        1.017800
## C_Age2                        1.132269  4        1.015649
## C_Gender                      1.030295  1        1.015034
## Size_Giant                    1.034368  1        1.017039
## D_Diet                        1.148676  3        1.023371
## Therapeutic_Food              1.024635  1        1.012242
## Visits                        1.366951  4        1.039846
## Meds                          1.261513  1        1.123171
## bs(D_Age, degree = 1, df = 2) 1.192555  2        1.045008  
 
 
 
 Create ROCR for training and test data 
  ## training data
pred.mtt = predict(m, type = "response") #repeat risk predictions from model m
rocr.pred.mtt = ROCR::prediction(pred.mtt, labels = ml_train$Any_Health_Problem) #ROCR prediction object
roc.perf.mtt = ROCR::performance(rocr.pred.mtt, measure = "tpr", x.measure = "fpr") # #ROCR performance object
plot(roc.perf.mtt, col = "blue")


pred.te.1 = predict(m, newdata = ml_test, type = "response") #.te = "test"
rocr.pred.te.1 = ROCR::prediction(pred.te.1, labels = ml_test$Any_Health_Problem)
roc.perf.te.1 = ROCR::performance(rocr.pred.te.1, measure = "tpr", x.measure = "fpr")
plot(roc.perf.te.1, col = "red", add = T)

abline(a = 0, b = 1, lty = 2) #diagonal for random assignment
legend("bottomright", legend = c("train","test"),
col = c("blue","red"), lty = c(2,1), lwd =1.5)  
   
 
 
 Report AUC from ROC for training and test data 
    # Train AUC
aucTr <- ROCR::performance(rocr.pred.mtt, measure = "auc")
  aucTr <- aucTr@y.values[[1]]
  print(aucTr)  
  ## [1] 0.8388373  
     # Test AUC
  aucTe <- ROCR::performance(rocr.pred.te.1, measure = "auc")
  aucTe <- aucTe@y.values[[1]]
  print(aucTe)  
  ## [1] 0.8540831  
 
 
 
 
 MULTIPLE REGRESSION WITH BACKWARDS ELIMINATION 
 
 ROUND 8: Model with all variables 
 Remove C_Gender 
  # fit binary logit model and store results 'm'
m <- glm(Any_Health_Problem ~  Income2 + C_Age2 + Size_Giant + D_Diet + Therapeutic_Food + Visits 
         + Meds + bs(D_Age,degree=1,df=2), data = ml_train,family = binomial)
# view a summary of the model
summary(m)  
  ## 
## Call:
## glm(formula = Any_Health_Problem ~ Income2 + C_Age2 + Size_Giant + 
##     D_Diet + Therapeutic_Food + Visits + Meds + bs(D_Age, degree = 1, 
##     df = 2), family = binomial, data = ml_train)
## 
## Coefficients:
##                                                         Estimate Std. Error
## (Intercept)                                             -2.42105    0.30922
## Income2Medium                                           -0.17927    0.17467
## Income2High                                             -0.49901    0.23411
## C_Age230–39                                             -0.16778    0.20467
## C_Age240–49                                             -0.24416    0.20885
## C_Age250–59                                             -0.50499    0.20776
## C_Age260<                                               -0.35579    0.21441
## Size_GiantYes                                            0.58647    0.29244
## D_DietMeat-based – raw                                  -0.09457    0.14311
## D_DietVegan (consuming no animal products)              -0.50094    0.20673
## D_DietVegetarian (including eggs or milk, but not meat) -0.20642    0.49972
## Therapeutic_FoodYes                                      0.90537    0.35393
## Visits1                                                  0.14909    0.20488
## Visits2                                                  1.05005    0.22583
## Visits3                                                  0.99554    0.28274
## Visits3<                                                 1.55197    0.27916
## MedsYes                                                  1.82266    0.14103
## bs(D_Age, degree = 1, df = 2)1                           0.82233    0.23290
## bs(D_Age, degree = 1, df = 2)2                           3.41800    0.35892
##                                                         z value Pr(>|z|)    
## (Intercept)                                              -7.830 4.90e-15 ***
## Income2Medium                                            -1.026 0.304736    
## Income2High                                              -2.131 0.033049 *  
## C_Age230–39                                              -0.820 0.412339    
## C_Age240–49                                              -1.169 0.242366    
## C_Age250–59                                              -2.431 0.015071 *  
## C_Age260<                                                -1.659 0.097037 .  
## Size_GiantYes                                             2.005 0.044920 *  
## D_DietMeat-based – raw                                   -0.661 0.508736    
## D_DietVegan (consuming no animal products)               -2.423 0.015387 *  
## D_DietVegetarian (including eggs or milk, but not meat)  -0.413 0.679553    
## Therapeutic_FoodYes                                       2.558 0.010525 *  
## Visits1                                                   0.728 0.466796    
## Visits2                                                   4.650 3.32e-06 ***
## Visits3                                                   3.521 0.000430 ***
## Visits3<                                                  5.560 2.71e-08 ***
## MedsYes                                                  12.924  < 2e-16 ***
## bs(D_Age, degree = 1, df = 2)1                            3.531 0.000414 ***
## bs(D_Age, degree = 1, df = 2)2                            9.523  < 2e-16 ***
## ---
## Signif. codes:  0 '***' 0.001 '**' 0.01 '*' 0.05 '.' 0.1 ' ' 1
## 
## (Dispersion parameter for binomial family taken to be 1)
## 
##     Null deviance: 2200  on 1657  degrees of freedom
## Residual deviance: 1569  on 1639  degrees of freedom
## AIC: 1607
## 
## Number of Fisher Scoring iterations: 4  
  # test model fit
with(m, null.deviance - deviance)  
  ## [1] 630.9862  
  with(m, df.null - df.residual)  
  ## [1] 18  
  with(m, pchisq(null.deviance - deviance, df.null - df.residual, lower.tail = FALSE))  
  ## [1] 2.402219e-122  
  BIC(m)  
  ## [1] 1709.896  
  # Hosmer-Lemeshow Goodness-of-Fit Test
hltest(m)  
  ## 
##    The Hosmer-Lemeshow goodness-of-fit test
## 
##  Group Size Observed  Expected
##      1  166       10  10.37486
##      2  167       24  15.59094
##      3  166       20  20.49306
##      4  167       25  26.97976
##      5  167       39  37.57308
##      6  166       55  56.39741
##      7  166       68  82.97775
##      8  166      106 106.02768
##      9  166      133 126.98388
##     10  161      148 144.60156
## 
##          Statistic =  12.7286 
## degrees of freedom =  8 
##            p-value =  0.12154  
  ## CIs using profiled log-likelihood
confint(m, level=0.99)  
  ## Waiting for profiling to be done...  
  ##                                                               0.5 %      99.5 %
## (Intercept)                                             -3.23404157 -1.63887711
## Income2Medium                                           -0.62734285  0.27387379
## Income2High                                             -1.10550871  0.10224083
## C_Age230–39                                             -0.69576036  0.35996343
## C_Age240–49                                             -0.78341586  0.29386860
## C_Age250–59                                             -1.04213233  0.02957798
## C_Age260<                                               -0.90989492  0.19611420
## Size_GiantYes                                           -0.18007187  1.33294160
## D_DietMeat-based – raw                                  -0.46351431  0.27459922
## D_DietVegan (consuming no animal products)              -1.04196181  0.02501299
## D_DietVegetarian (including eggs or milk, but not meat) -1.52551601  1.07519071
## Therapeutic_FoodYes                                      0.03148903  1.86728236
## Visits1                                                 -0.36897316  0.68996812
## Visits2                                                  0.47665798  1.64304640
## Visits3                                                  0.27331248  1.73280848
## Visits3<                                                 0.84318787  2.28396933
## MedsYes                                                  1.46298683  2.19030275
## bs(D_Age, degree = 1, df = 2)1                           0.22836179  1.42973058
## bs(D_Age, degree = 1, df = 2)2                           2.50481130  4.35655563  
  ## CIs using standard errors
confint.default(m, level=0.99)  
  ##                                                                0.5 %
## (Intercept)                                             -3.217548511
## Income2Medium                                           -0.629203857
## Income2High                                             -1.102037538
## C_Age230–39                                             -0.694977641
## C_Age240–49                                             -0.782116473
## C_Age250–59                                             -1.040144928
## C_Age260<                                               -0.908075633
## Size_GiantYes                                           -0.166816672
## D_DietMeat-based – raw                                  -0.463206492
## D_DietVegan (consuming no animal products)              -1.033450107
## D_DietVegetarian (including eggs or milk, but not meat) -1.493622848
## Therapeutic_FoodYes                                     -0.006278296
## Visits1                                                 -0.378640762
## Visits2                                                  0.468349499
## Visits3                                                  0.267254171
## Visits3<                                                 0.832911159
## MedsYes                                                  1.459392813
## bs(D_Age, degree = 1, df = 2)1                           0.222433433
## bs(D_Age, degree = 1, df = 2)2                           2.493480724
##                                                              99.5 %
## (Intercept)                                             -1.62455686
## Income2Medium                                            0.27065750
## Income2High                                              0.10402581
## C_Age230–39                                              0.35940815
## C_Age240–49                                              0.29379281
## C_Age250–59                                              0.03015882
## C_Age260<                                                0.19649333
## Size_GiantYes                                            1.33975151
## D_DietMeat-based – raw                                   0.27406555
## D_DietVegan (consuming no animal products)               0.03156742
## D_DietVegetarian (including eggs or milk, but not meat)  1.08077819
## Therapeutic_FoodYes                                      1.81702727
## Visits1                                                  0.67682082
## Visits2                                                  1.63176014
## Visits3                                                  1.72381942
## Visits3<                                                 2.27102614
## MedsYes                                                  2.18593158
## bs(D_Age, degree = 1, df = 2)1                           1.42223463
## bs(D_Age, degree = 1, df = 2)2                           4.34251225  
  # Wald test
wald.test(b = coef(m), Sigma = vcov(m), Terms = 2)  
  ## Wald test:
## ----------
## 
## Chi-squared test:
## X2 = 1.1, df = 1, P(> X2) = 0.3  
  ## odds ratios and 95% CI
exp(cbind(OR = coef(m), confint(m, level=0.99)))  
  ## Waiting for profiling to be done...  
  ##                                                                  OR       0.5 %
## (Intercept)                                              0.08882806  0.03939795
## Income2Medium                                            0.83587753  0.53400886
## Income2High                                              0.60713393  0.33104243
## C_Age230–39                                              0.84553582  0.49869512
## C_Age240–49                                              0.78336085  0.45684283
## C_Age250–59                                              0.60350977  0.35270180
## C_Age260<                                                0.70061893  0.40256652
## Size_GiantYes                                            1.79762692  0.83521018
## D_DietMeat-based – raw                                   0.90976362  0.62906901
## D_DietVegan (consuming no animal products)               0.60595998  0.35276195
## D_DietVegetarian (including eggs or milk, but not meat)  0.81348944  0.21750879
## Therapeutic_FoodYes                                      2.47285780  1.03199006
## Visits1                                                  1.16077749  0.69144397
## Visits2                                                  2.85780778  1.61068246
## Visits3                                                  2.70617662  1.31431088
## Visits3<                                                 4.72075455  2.32376304
## MedsYes                                                  6.18831103  4.31883992
## bs(D_Age, degree = 1, df = 2)1                           2.27580545  1.25653984
## bs(D_Age, degree = 1, df = 2)2                          30.50823009 12.24124880
##                                                            99.5 %
## (Intercept)                                              0.194198
## Income2Medium                                            1.315049
## Income2High                                              1.107650
## C_Age230–39                                              1.433277
## C_Age240–49                                              1.341608
## C_Age250–59                                              1.030020
## C_Age260<                                                1.216666
## Size_GiantYes                                            3.792182
## D_DietMeat-based – raw                                   1.316003
## D_DietVegan (consuming no animal products)               1.025328
## D_DietVegetarian (including eggs or milk, but not meat)  2.930552
## Therapeutic_FoodYes                                      6.470688
## Visits1                                                  1.993652
## Visits2                                                  5.170898
## Visits3                                                  5.656518
## Visits3<                                                 9.815564
## MedsYes                                                  8.937919
## bs(D_Age, degree = 1, df = 2)1                           4.177574
## bs(D_Age, degree = 1, df = 2)2                          77.988051  
 
 Calculate Nagelkerke R^2 
  NagelkerkeR2(m)  
  ## $N
## [1] 1658
## 
## $R2
## [1] 0.4308236  
 
 
 check assumptions of model 
 
 Cook’s distance 
  plot(m, which = 4, id.n = 3)  
   
 
 
 Extract model results and display data for top 3 values using Cook’s
distance 
  model.data <- augment(m) %>% 
  mutate(index = 1:n()) 
model.data %>% top_n(3, .cooksd)  
 
 
 
 
 
 plot standardised residuals 
  ggplot(model.data, aes(index, .std.resid)) + 
  geom_point(aes(color = Any_Health_Problem), alpha = .5) +
  theme_bw()  
   
 
 
 Filter potential influential data points with abs(.std.res) >
3: 
  model.data %>% 
  filter(abs(.std.resid) > 3)  
 
 
 
 
 
 check for multicollinearity 
  car::vif(m)  
  ##                                   GVIF Df GVIF^(1/(2*Df))
## Income2                       1.066750  2        1.016285
## C_Age2                        1.126663  4        1.015019
## Size_Giant                    1.031832  1        1.015791
## D_Diet                        1.142021  3        1.022380
## Therapeutic_Food              1.024249  1        1.012052
## Visits                        1.360814  4        1.039262
## Meds                          1.258905  1        1.122009
## bs(D_Age, degree = 1, df = 2) 1.190991  2        1.044665  
 
 
 
 Create ROCR for training and test data 
  ## training data
pred.mtt = predict(m, type = "response") #repeat risk predictions from model m
rocr.pred.mtt = ROCR::prediction(pred.mtt, labels = ml_train$Any_Health_Problem) #ROCR prediction object
roc.perf.mtt = ROCR::performance(rocr.pred.mtt, measure = "tpr", x.measure = "fpr") # #ROCR performance object
plot(roc.perf.mtt, col = "blue")


pred.te.1 = predict(m, newdata = ml_test, type = "response") #.te = "test"
rocr.pred.te.1 = ROCR::prediction(pred.te.1, labels = ml_test$Any_Health_Problem)
roc.perf.te.1 = ROCR::performance(rocr.pred.te.1, measure = "tpr", x.measure = "fpr")
plot(roc.perf.te.1, col = "red", add = T)

abline(a = 0, b = 1, lty = 2) #diagonal for random assignment
legend("bottomright", legend = c("train","test"),
col = c("blue","red"), lty = c(2,1), lwd =1.5)  
   
 
 
 Report AUC from ROC for training and test data 
    # Train AUC
aucTr <- ROCR::performance(rocr.pred.mtt, measure = "auc")
  aucTr <- aucTr@y.values[[1]]
  print(aucTr)  
  ## [1] 0.8387283  
     # Test AUC
  aucTe <- ROCR::performance(rocr.pred.te.1, measure = "auc")
  aucTe <- aucTe@y.values[[1]]
  print(aucTe)  
  ## [1] 0.8528846  
 
 
 
 
 MULTIPLE REGRESSION WITH BACKWARDS ELIMINATION 
 
 ROUND 8: Model with all variables 
 Remove Income2 
  # fit binary logit model and store results 'm'
m <- glm(Any_Health_Problem ~  C_Age2 + Size_Giant + D_Diet + Therapeutic_Food + Visits 
         + Meds + bs(D_Age,degree=1,df=2), data = ml_train,family = binomial)
# view a summary of the model
summary(m)  
  ## 
## Call:
## glm(formula = Any_Health_Problem ~ C_Age2 + Size_Giant + D_Diet + 
##     Therapeutic_Food + Visits + Meds + bs(D_Age, degree = 1, 
##     df = 2), family = binomial, data = ml_train)
## 
## Coefficients:
##                                                         Estimate Std. Error
## (Intercept)                                             -2.56832    0.28267
## C_Age230–39                                             -0.19308    0.20389
## C_Age240–49                                             -0.30057    0.20669
## C_Age250–59                                             -0.54064    0.20684
## C_Age260<                                               -0.37172    0.21400
## Size_GiantYes                                            0.58263    0.29308
## D_DietMeat-based – raw                                  -0.07686    0.14255
## D_DietVegan (consuming no animal products)              -0.47911    0.20614
## D_DietVegetarian (including eggs or milk, but not meat) -0.28741    0.49845
## Therapeutic_FoodYes                                      0.88424    0.35307
## Visits1                                                  0.13302    0.20412
## Visits2                                                  1.02471    0.22466
## Visits3                                                  0.97039    0.28176
## Visits3<                                                 1.50025    0.27731
## MedsYes                                                  1.82198    0.14081
## bs(D_Age, degree = 1, df = 2)1                           0.81561    0.23223
## bs(D_Age, degree = 1, df = 2)2                           3.40359    0.35767
##                                                         z value Pr(>|z|)    
## (Intercept)                                              -9.086  < 2e-16 ***
## C_Age230–39                                              -0.947 0.343627    
## C_Age240–49                                              -1.454 0.145878    
## C_Age250–59                                              -2.614 0.008954 ** 
## C_Age260<                                                -1.737 0.082383 .  
## Size_GiantYes                                             1.988 0.046820 *  
## D_DietMeat-based – raw                                   -0.539 0.589769    
## D_DietVegan (consuming no animal products)               -2.324 0.020118 *  
## D_DietVegetarian (including eggs or milk, but not meat)  -0.577 0.564202    
## Therapeutic_FoodYes                                       2.504 0.012266 *  
## Visits1                                                   0.652 0.514604    
## Visits2                                                   4.561 5.09e-06 ***
## Visits3                                                   3.444 0.000573 ***
## Visits3<                                                  5.410 6.30e-08 ***
## MedsYes                                                  12.940  < 2e-16 ***
## bs(D_Age, degree = 1, df = 2)1                            3.512 0.000445 ***
## bs(D_Age, degree = 1, df = 2)2                            9.516  < 2e-16 ***
## ---
## Signif. codes:  0 '***' 0.001 '**' 0.01 '*' 0.05 '.' 0.1 ' ' 1
## 
## (Dispersion parameter for binomial family taken to be 1)
## 
##     Null deviance: 2200.0  on 1657  degrees of freedom
## Residual deviance: 1573.8  on 1641  degrees of freedom
## AIC: 1607.8
## 
## Number of Fisher Scoring iterations: 4  
  # test model fit
with(m, null.deviance - deviance)  
  ## [1] 626.2676  
  with(m, df.null - df.residual)  
  ## [1] 16  
  with(m, pchisq(null.deviance - deviance, df.null - df.residual, lower.tail = FALSE))  
  ## [1] 6.097906e-123  
  BIC(m)  
  ## [1] 1699.788  
  # Hosmer-Lemeshow Goodness-of-Fit Test
hltest(m)  
  ## 
##    The Hosmer-Lemeshow goodness-of-fit test
## 
##  Group Size Observed  Expected
##      1  166       13  10.81019
##      2  167       19  15.58745
##      3  169       22  21.09403
##      4  165       22  26.52460
##      5  167       45  37.72296
##      6  166       52  56.28954
##      7  166       67  83.36258
##      8  167      109 107.09688
##      9  167      134 127.97024
##     10  158      145 141.54153
## 
##          Statistic =  13.14338 
## degrees of freedom =  8 
##            p-value =  0.10701  
  ## CIs using profiled log-likelihood
confint(m, level=0.99)  
  ## Waiting for profiling to be done...  
  ##                                                               0.5 %
## (Intercept)                                             -3.31249976
## C_Age230–39                                             -0.71913886
## C_Age240–49                                             -0.83442998
## C_Age250–59                                             -1.07553878
## C_Age260<                                               -0.92478242
## Size_GiantYes                                           -0.18542218
## D_DietMeat-based – raw                                  -0.44428602
## D_DietVegan (consuming no animal products)              -1.01838953
## D_DietVegetarian (including eggs or milk, but not meat) -1.60208385
## Therapeutic_FoodYes                                      0.01332823
## Visits1                                                 -0.38312044
## Visits2                                                  0.45419848
## Visits3                                                  0.25056182
## Visits3<                                                 0.79611638
## MedsYes                                                  1.46284603
## bs(D_Age, degree = 1, df = 2)1                           0.22336893
## bs(D_Age, degree = 1, df = 2)2                           2.49381458
##                                                               99.5 %
## (Intercept)                                             -1.854415585
## C_Age230–39                                              0.332533368
## C_Age240–49                                              0.231710821
## C_Age250–59                                             -0.008569285
## C_Age260<                                                0.179083007
## Size_GiantYes                                            1.330610135
## D_DietMeat-based – raw                                   0.290932845
## D_DietVegan (consuming no animal products)               0.045509178
## D_DietVegetarian (including eggs or milk, but not meat)  0.992410886
## Therapeutic_FoodYes                                      1.845392599
## Visits1                                                  0.671952765
## Visits2                                                  1.614565039
## Visits3                                                  1.704999592
## Visits3<                                                 2.227394631
## MedsYes                                                  2.189000923
## bs(D_Age, degree = 1, df = 2)1                           1.421309187
## bs(D_Age, degree = 1, df = 2)2                           4.339084374  
  ## CIs using standard errors
confint.default(m, level=0.99)  
  ##                                                               0.5 %
## (Intercept)                                             -3.29641651
## C_Age230–39                                             -0.71825973
## C_Age240–49                                             -0.83295997
## C_Age250–59                                             -1.07342969
## C_Age260<                                               -0.92293450
## Size_GiantYes                                           -0.17230398
## D_DietMeat-based – raw                                  -0.44405605
## D_DietVegan (consuming no animal products)              -1.01009491
## D_DietVegetarian (including eggs or milk, but not meat) -1.57133120
## Therapeutic_FoodYes                                     -0.02522076
## Visits1                                                 -0.39276352
## Visits2                                                  0.44601980
## Visits3                                                  0.24463795
## Visits3<                                                 0.78594314
## MedsYes                                                  1.45928444
## bs(D_Age, degree = 1, df = 2)1                           0.21741832
## bs(D_Age, degree = 1, df = 2)2                           2.48229107
##                                                               99.5 %
## (Intercept)                                             -1.840215754
## C_Age230–39                                              0.332090606
## C_Age240–49                                              0.231815279
## C_Age250–59                                             -0.007855286
## C_Age260<                                                0.179501343
## Size_GiantYes                                            1.337559124
## D_DietMeat-based – raw                                   0.290333889
## D_DietVegan (consuming no animal products)               0.051883920
[truncated: 174,822 more chars]
