# Supplementary material for: Variables associated with owner perceptions of the health of their dog: Further analysis of data from a large international survey
Source: PLoS One. 2024 May 15;19(5):e0280173. doi: 10.1371/journal.pone.0280173 (PMC11095744; doi:10.1371/journal.pone.0280173)
Supplement: S8 File — (HTML) [file pone.0280173.s022.html]

R stats for binary logistic regression on ANY HEALTH PROBLEM for the all-owner dataset


# R stats for binary logistic regression on ANY HEALTH PROBLEM for the all-owner dataset

#### Alex German

#### 7 December 2023

# Create data frame for analysis

### NB need to run “Read\_data\_102.Rtm” first to create dataset

## Chi squared test of Health\_Binary and Primary\_Decision\_Maker

```
table(ml$Health_Binary, ml$Primary_Decision_Maker)
```

```
##    
##       No  Yes
##   0  102 2089
##   1    9  122
```

```
chisq.test(ml$Health_Binary, ml$Primary_Decision_Maker, correct=FALSE)
```

```
## 
##  Pearson's Chi-squared test
## 
## data:  ml$Health_Binary and ml$Primary_Decision_Maker
## X-squared = 1.3321, df = 1, p-value = 0.2484
```

# BINARY LOGISTIC REGRESSION ON HEALTH

# CHECK EFFECT OF OWNER CHARACTERISTICS - simple binary logistic regression

## DECISION MAKER Binary regression for Any\_Health\_Problem

```
# fit binary logit model and store results 'm'
m <- glm(Any_Health_Problem ~ Primary_Decision_Maker, data = ml,family = binomial)
# view a summary of the model
summary(m)
```

```
## 
## Call:
## glm(formula = Any_Health_Problem ~ Primary_Decision_Maker, family = binomial, 
##     data = ml)
## 
## Coefficients:
##                           Estimate Std. Error z value Pr(>|z|)   
## (Intercept)                0.05407    0.18990   0.285  0.77586   
## Primary_Decision_MakerYes -0.55165    0.19490  -2.830  0.00465 **
## ---
## Signif. codes:  0 '***' 0.001 '**' 0.01 '*' 0.05 '.' 0.1 ' ' 1
## 
## (Dispersion parameter for binomial family taken to be 1)
## 
##     Null deviance: 3094.1  on 2321  degrees of freedom
## Residual deviance: 3086.2  on 2320  degrees of freedom
## AIC: 3090.2
## 
## Number of Fisher Scoring iterations: 4
```

```
# test model fit
with(m, null.deviance - deviance)
```

```
## [1] 7.961804
```

```
with(m, df.null - df.residual)
```

```
## [1] 1
```

```
with(m, pchisq(null.deviance - deviance, df.null - df.residual, lower.tail = FALSE))
```

```
## [1] 0.004777479
```

```
BIC(m)
```

```
## [1] 3101.663
```

```
## CIs using profiled log-likelihood
confint(m, level=0.99)
```

```
## Waiting for profiling to be done...
```

```
##                               0.5 %      99.5 %
## (Intercept)               -0.436452  0.54675736
## Primary_Decision_MakerYes -1.057004 -0.04840597
```

```
## CIs using standard errors
confint.default(m, level=0.99)
```

```
##                                0.5 %      99.5 %
## (Intercept)               -0.4350853  0.54321970
## Primary_Decision_MakerYes -1.0536755 -0.04961972
```

```
# Wald test
wald.test(b = coef(m), Sigma = vcov(m), Terms = 2)
```

```
## Wald test:
## ----------
## 
## Chi-squared test:
## X2 = 8.0, df = 1, P(> X2) = 0.0046
```

```
## odds ratios and 95% CI
exp(cbind(OR = coef(m), confint(m, level=0.99)))
```

```
## Waiting for profiling to be done...
```

```
##                                 OR     0.5 %    99.5 %
## (Intercept)               1.055556 0.6463255 1.7276418
## Primary_Decision_MakerYes 0.576000 0.3474954 0.9527469
```

### Create ROCR from data

```
pred.mtt = predict(m, type = "response") #repeat risk predictions from model m
rocr.pred.mtt = ROCR::prediction(pred.mtt, labels = ml$Any_Health_Problem) #ROCR prediction object
roc.perf.mtt = ROCR::performance(rocr.pred.mtt, measure = "tpr", x.measure = "fpr") # #ROCR performance object
plot(roc.perf.mtt, col = "blue")
abline(a = 0, b = 1, lty = 2) #diagonal for random assignment
```

### Report AUC from ROC for training and test data

```
  # Train AUC
auc <- ROCR::performance(rocr.pred.mtt, measure = "auc")
  auc <- auc@y.values[[1]]
  print(auc)
```

```
## [1] 0.5130206
```

### Calculate Nagelkerke R^2

```
NagelkerkeR2(m)
```

```
## $N
## [1] 2322
## 
## $R2
## [1] 0.004649594
```

### check assumptions of model

#### Cook’s distance

```
plot(m, which = 4, id.n = 3)
```

#### Extract model results and display data for top 3 values using Cook’s distance

```
model.data <- augment(m) %>% 
  mutate(index = 1:n()) 
model.data %>% top_n(3, .cooksd)
```

#### plot standardised residuals

```
ggplot(model.data, aes(index, .std.resid)) + 
  geom_point(aes(color = Any_Health_Problem), alpha = .5) +
  theme_bw()
```

#### Filter potential influential data points with abs(.std.res) > 3:

```
model.data %>% 
  filter(abs(.std.resid) > 3)
```

## CLIENT DIET Binary regression for Any\_Health\_Problem

```
# fit binary logit model and store results 'm'
m <- glm(Any_Health_Problem ~ C_Diet, data = ml,family = binomial)
# view a summary of the model
summary(m)
```

```
## 
## Call:
## glm(formula = Any_Health_Problem ~ C_Diet, family = binomial, 
##     data = ml)
## 
## Coefficients:
##                                                       Estimate Std. Error
## (Intercept)                                           -0.57611    0.06829
## C_DietOmnivore reducing animal product consumption     0.17897    0.11394
## C_DietPescatarian (including fish but no other meats)  0.57611    0.19215
## C_DietVegan (consuming no animal products)             0.04518    0.11280
## C_DietVegetarian (consuming plants, eggs and milk)     0.24110    0.14888
##                                                       z value Pr(>|z|)    
## (Intercept)                                            -8.437  < 2e-16 ***
## C_DietOmnivore reducing animal product consumption      1.571  0.11624    
## C_DietPescatarian (including fish but no other meats)   2.998  0.00272 ** 
## C_DietVegan (consuming no animal products)              0.401  0.68875    
## C_DietVegetarian (consuming plants, eggs and milk)      1.619  0.10537    
## ---
## Signif. codes:  0 '***' 0.001 '**' 0.01 '*' 0.05 '.' 0.1 ' ' 1
## 
## (Dispersion parameter for binomial family taken to be 1)
## 
##     Null deviance: 3094.1  on 2321  degrees of freedom
## Residual deviance: 3082.8  on 2317  degrees of freedom
## AIC: 3092.8
## 
## Number of Fisher Scoring iterations: 4
```

```
# test model fit
with(m, null.deviance - deviance)
```

```
## [1] 11.35382
```

```
with(m, df.null - df.residual)
```

```
## [1] 4
```

```
with(m, pchisq(null.deviance - deviance, df.null - df.residual, lower.tail = FALSE))
```

```
## [1] 0.0228626
```

```
BIC(m)
```

```
## [1] 3121.522
```

```
## CIs using profiled log-likelihood
confint(m, level=0.99)
```

```
## Waiting for profiling to be done...
```

```
##                                                             0.5 %     99.5 %
## (Intercept)                                           -0.75356757 -0.4015498
## C_DietOmnivore reducing animal product consumption    -0.11534403  0.4720064
## C_DietPescatarian (including fish but no other meats)  0.07964473  1.0729339
## C_DietVegan (consuming no animal products)            -0.24649573  0.3349825
## C_DietVegetarian (consuming plants, eggs and milk)    -0.14526831  0.6229633
```

```
## CIs using standard errors
confint.default(m, level=0.99)
```

```
##                                                             0.5 %     99.5 %
## (Intercept)                                           -0.75200225 -0.4002180
## C_DietOmnivore reducing animal product consumption    -0.11452088  0.4724638
## C_DietPescatarian (including fish but no other meats)  0.08116877  1.0710515
## C_DietVegan (consuming no animal products)            -0.24537367  0.3357403
## C_DietVegetarian (consuming plants, eggs and milk)    -0.14240163  0.6245950
```

```
# Wald test
wald.test(b = coef(m), Sigma = vcov(m), Terms = 2)
```

```
## Wald test:
## ----------
## 
## Chi-squared test:
## X2 = 2.5, df = 1, P(> X2) = 0.12
```

```
## odds ratios and 95% CI
exp(cbind(OR = coef(m), confint(m, level=0.99)))
```

```
## Waiting for profiling to be done...
```

```
##                                                              OR     0.5 %
## (Intercept)                                           0.5620805 0.4706844
## C_DietOmnivore reducing animal product consumption    1.1959866 0.8910595
## C_DietPescatarian (including fish but no other meats) 1.7791045 1.0829023
## C_DietVegan (consuming no animal products)            1.0462196 0.7815347
## C_DietVegetarian (consuming plants, eggs and milk)    1.2726441 0.8647902
##                                                         99.5 %
## (Intercept)                                           0.669282
## C_DietOmnivore reducing animal product consumption    1.603208
## C_DietPescatarian (including fish but no other meats) 2.923945
## C_DietVegan (consuming no animal products)            1.397916
## C_DietVegetarian (consuming plants, eggs and milk)    1.864445
```

### Create ROCR from data

```
pred.mtt = predict(m, type = "response") #repeat risk predictions from model m
rocr.pred.mtt = ROCR::prediction(pred.mtt, labels = ml$Any_Health_Problem) #ROCR prediction object
roc.perf.mtt = ROCR::performance(rocr.pred.mtt, measure = "tpr", x.measure = "fpr") # #ROCR performance object
plot(roc.perf.mtt, col = "blue")
abline(a = 0, b = 1, lty = 2) #diagonal for random assignment
```

### Report AUC from ROC for training and test data

```
  # Train AUC
auc <- ROCR::performance(rocr.pred.mtt, measure = "auc")
  auc <- auc@y.values[[1]]
  print(auc)
```

```
## [1] 0.533932
```

### Calculate Nagelkerke R^2

```
NagelkerkeR2(m)
```

```
## $N
## [1] 2322
## 
## $R2
## [1] 0.006625649
```

### check assumptions of model

#### Cook’s distance

```
plot(m, which = 4, id.n = 3)
```

#### Extract model results and display data for top 3 values using Cook’s distance

```
model.data <- augment(m) %>% 
  mutate(index = 1:n()) 
model.data %>% top_n(3, .cooksd)
```

#### plot standardised residuals

```
ggplot(model.data, aes(index, .std.resid)) + 
  geom_point(aes(color = Any_Health_Problem), alpha = .5) +
  theme_bw()
```

#### Filter potential influential data points with abs(.std.res) > 3:

```
model.data %>% 
  filter(abs(.std.resid) > 3)
```

## CLIENT DIET VEGAN Binary regression for Any\_Health\_Problem

```
# fit binary logit model and store results 'm'
m <- glm(Any_Health_Problem ~ C_Diet_Vegan, data = ml,family = binomial)
# view a summary of the model
summary(m)
```

```
## 
## Call:
## glm(formula = Any_Health_Problem ~ C_Diet_Vegan, family = binomial, 
##     data = ml)
## 
## Coefficients:
##                 Estimate Std. Error z value Pr(>|z|)    
## (Intercept)     -0.45225    0.04849  -9.327   <2e-16 ***
## C_Diet_VeganYes -0.07868    0.10204  -0.771    0.441    
## ---
## Signif. codes:  0 '***' 0.001 '**' 0.01 '*' 0.05 '.' 0.1 ' ' 1
## 
## (Dispersion parameter for binomial family taken to be 1)
## 
##     Null deviance: 3094.1  on 2321  degrees of freedom
## Residual deviance: 3093.5  on 2320  degrees of freedom
## AIC: 3097.5
## 
## Number of Fisher Scoring iterations: 4
```

```
# test model fit
with(m, null.deviance - deviance)
```

```
## [1] 0.5967405
```

```
with(m, df.null - df.residual)
```

```
## [1] 1
```

```
with(m, pchisq(null.deviance - deviance, df.null - df.residual, lower.tail = FALSE))
```

```
## [1] 0.4398244
```

```
BIC(m)
```

```
## [1] 3109.028
```

```
## CIs using profiled log-likelihood
confint(m, level=0.99)
```

```
## Waiting for profiling to be done...
```

```
##                      0.5 %     99.5 %
## (Intercept)     -0.5777575 -0.3278932
## C_Diet_VeganYes -0.3433791  0.1826859
```

```
## CIs using standard errors
confint.default(m, level=0.99)
```

```
##                      0.5 %     99.5 %
## (Intercept)     -0.5771371 -0.3273556
## C_Diet_VeganYes -0.3415170  0.1841560
```

```
# Wald test
wald.test(b = coef(m), Sigma = vcov(m), Terms = 2)
```

```
## Wald test:
## ----------
## 
## Chi-squared test:
## X2 = 0.59, df = 1, P(> X2) = 0.44
```

```
## odds ratios and 95% CI
exp(cbind(OR = coef(m), confint(m, level=0.99)))
```

```
## Waiting for profiling to be done...
```

```
##                        OR     0.5 %   99.5 %
## (Intercept)     0.6361974 0.5611553 0.720440
## C_Diet_VeganYes 0.9243352 0.7093693 1.200437
```

### Create ROCR from data

```
pred.mtt = predict(m, type = "response") #repeat risk predictions from model m
rocr.pred.mtt = ROCR::prediction(pred.mtt, labels = ml$Any_Health_Problem) #ROCR prediction object
roc.perf.mtt = ROCR::performance(rocr.pred.mtt, measure = "tpr", x.measure = "fpr") # #ROCR performance object
plot(roc.perf.mtt, col = "blue")
abline(a = 0, b = 1, lty = 2) #diagonal for random assignment
```

### Report AUC from ROC for training and test data

```
  # Train AUC
auc <- ROCR::performance(rocr.pred.mtt, measure = "auc")
  auc <- auc@y.values[[1]]
  print(auc)
```

```
## [1] 0.5069125
```

### Calculate Nagelkerke R^2

```
NagelkerkeR2(m)
```

```
## $N
## [1] 2322
## 
## $R2
## [1] 0.0003490419
```

### check assumptions of model

#### Cook’s distance

```
plot(m, which = 4, id.n = 3)
```

#### Extract model results and display data for top 3 values using Cook’s distance

```
model.data <- augment(m) %>% 
  mutate(index = 1:n()) 
model.data %>% top_n(3, .cooksd)
```

#### plot standardised residuals

```
ggplot(model.data, aes(index, .std.resid)) + 
  geom_point(aes(color = Any_Health_Problem), alpha = .5) +
  theme_bw()
```

# Filter potential influential data points with abs(.std.res) > 3:

```
model.data %>% 
  filter(abs(.std.resid) > 3)
```

## C\_Diet\_Vegan\_Veggie Binary logistic regression for HEALTH

```
# fit binary logit model and store results 'm'
m <- glm(Any_Health_Problem ~ C_Diet_Vegan_Veggie, data = ml,family = binomial)
# view a summary of the model
summary(m)
```

```
## 
## Call:
## glm(formula = Any_Health_Problem ~ C_Diet_Vegan_Veggie, family = binomial, 
##     data = ml)
## 
## Coefficients:
##                         Estimate Std. Error z value Pr(>|z|)    
## (Intercept)            -0.470213   0.052126  -9.021   <2e-16 ***
## C_Diet_Vegan_VeggieYes  0.000209   0.090695   0.002    0.998    
## ---
## Signif. codes:  0 '***' 0.001 '**' 0.01 '*' 0.05 '.' 0.1 ' ' 1
## 
## (Dispersion parameter for binomial family taken to be 1)
## 
##     Null deviance: 3094.1  on 2321  degrees of freedom
## Residual deviance: 3094.1  on 2320  degrees of freedom
## AIC: 3098.1
## 
## Number of Fisher Scoring iterations: 4
```

```
# test model fit
with(m, null.deviance - deviance)
```

```
## [1] 5.310776e-06
```

```
with(m, df.null - df.residual)
```

```
## [1] 1
```

```
with(m, pchisq(null.deviance - deviance, df.null - df.residual, lower.tail = FALSE))
```

```
## [1] 0.9981613
```

```
BIC(m)
```

```
## [1] 3109.625
```

```
## CIs using profiled log-likelihood
confint(m, level=0.99)
```

```
## Waiting for profiling to be done...
```

```
##                             0.5 %     99.5 %
## (Intercept)            -0.6052281 -0.3365870
## C_Diet_Vegan_VeggieYes -0.2342212  0.2332098
```

```
## CIs using standard errors
confint.default(m, level=0.99)
```

```
##                             0.5 %     99.5 %
## (Intercept)            -0.6044816 -0.3359437
## C_Diet_Vegan_VeggieYes -0.2334067  0.2338248
```

```
# Wald test
wald.test(b = coef(m), Sigma = vcov(m), Terms = 2)
```

```
## Wald test:
## ----------
## 
## Chi-squared test:
## X2 = 5.3e-06, df = 1, P(> X2) = 1.0
```

```
## odds ratios and 95% CI
exp(cbind(OR = coef(m), confint(m, level=0.99)))
```

```
## Waiting for profiling to be done...
```

```
##                               OR     0.5 %    99.5 %
## (Intercept)            0.6248694 0.5459499 0.7142037
## C_Diet_Vegan_VeggieYes 1.0002090 0.7911868 1.2626463
```

### Create ROCR from data

```
pred.mtt = predict(m, type = "response") #repeat risk predictions from model m
rocr.pred.mtt = ROCR::prediction(pred.mtt, labels = ml$Any_Health_Problem) #ROCR prediction object
roc.perf.mtt = ROCR::performance(rocr.pred.mtt, measure = "tpr", x.measure = "fpr") # #ROCR performance object
plot(roc.perf.mtt, col = "blue")
abline(a = 0, b = 1, lty = 2) #diagonal for random assignment
```

### Report AUC from ROC for training and test data

```
  # Train AUC
auc <- ROCR::performance(rocr.pred.mtt, measure = "auc")
  auc <- auc@y.values[[1]]
  print(auc)
```

```
## [1] 0.5000231
```

### Calculate Nagelkerke R^2

```
NagelkerkeR2(m)
```

```
## $N
## [1] 2322
## 
## $R2
## [1] 3.106747e-09
```

### check assumptions of model

#### Cook’s distance

```
plot(m, which = 4, id.n = 3)
```

#### Extract model results and display data for top 3 values using Cook’s distance

```
model.data <- augment(m) %>% 
  mutate(index = 1:n()) 
model.data %>% top_n(3, .cooksd)
```

#### plot standardised residuals

```
ggplot(model.data, aes(index, .std.resid)) + 
  geom_point(aes(color = Any_Health_Problem), alpha = .5) +
  theme_bw()
```

#### Filter potential influential data points with abs(.std.res) > 3:

```
model.data %>% 
  filter(abs(.std.resid) > 3)
```

## LOCATION binary logistic regression for HEALTH

```
# fit binary logit model and store results 'm'
m <- glm(Any_Health_Problem ~ Location, data = ml,family = binomial)
# view a summary of the model
summary(m)
```

```
## 
## Call:
## glm(formula = Any_Health_Problem ~ Location, family = binomial, 
##     data = ml)
## 
## Coefficients:
##                                       Estimate Std. Error z value Pr(>|z|)    
## (Intercept)                           -0.52661    0.05081 -10.363   <2e-16 ***
## LocationOther European                 0.16518    0.11885   1.390    0.165    
## LocationNorth America                  0.30698    0.18496   1.660    0.097 .  
## LocationAustralia/New Zealand/Oceania  0.24476    0.20828   1.175    0.240    
## LocationOther                          0.07766    0.23915   0.325    0.745    
## ---
## Signif. codes:  0 '***' 0.001 '**' 0.01 '*' 0.05 '.' 0.1 ' ' 1
## 
## (Dispersion parameter for binomial family taken to be 1)
## 
##     Null deviance: 3094.1  on 2321  degrees of freedom
## Residual deviance: 3089.0  on 2317  degrees of freedom
## AIC: 3099
## 
## Number of Fisher Scoring iterations: 4
```

```
# test model fit
with(m, null.deviance - deviance)
```

```
## [1] 5.087203
```

```
with(m, df.null - df.residual)
```

```
## [1] 4
```

```
with(m, pchisq(null.deviance - deviance, df.null - df.residual, lower.tail = FALSE))
```

```
## [1] 0.2784664
```

```
BIC(m)
```

```
## [1] 3127.788
```

```
# Hosmer-Lemeshow Goodness-of-Fit Test
glmtoolbox::hltest(m)
```

```
## 
##    The Hosmer-Lemeshow goodness-of-fit test
## 
##  Group Size Observed Expected
##      1 1659      616      616
##      2   77       30       30
##      3  358      147      147
##      4  100       43       43
##      5  128       57       57
## 
##          Statistic =  0 
## degrees of freedom =  3 
##            p-value =  1
```

```
## CIs using profiled log-likelihood
confint(m, level=0.99)
```

```
## Waiting for profiling to be done...
```

```
##                                            0.5 %     99.5 %
## (Intercept)                           -0.6582840 -0.3964074
## LocationOther European                -0.1430240  0.4699145
## LocationNorth America                 -0.1747627  0.7817039
## LocationAustralia/New Zealand/Oceania -0.3004182  0.7780286
## LocationOther                         -0.5555474  0.6851848
```

```
## CIs using standard errors
confint.default(m, level=0.99)
```

```
##                                            0.5 %     99.5 %
## (Intercept)                           -0.6574999 -0.3957191
## LocationOther European                -0.1409413  0.4713092
## LocationNorth America                 -0.1694467  0.7834085
## LocationAustralia/New Zealand/Oceania -0.2917432  0.7812599
## LocationOther                         -0.5383468  0.6936654
```

```
# Wald test
wald.test(b = coef(m), Sigma = vcov(m), Terms = 2)
```

```
## Wald test:
## ----------
## 
## Chi-squared test:
## X2 = 1.9, df = 1, P(> X2) = 0.16
```

```
## odds ratios and 95% CI
exp(cbind(OR = coef(m), confint(m, level=0.99)))
```

```
## Waiting for profiling to be done...
```

```
##                                             OR     0.5 %    99.5 %
## (Intercept)                           0.590604 0.5177390 0.6727326
## LocationOther European                1.179610 0.8667333 1.5998573
## LocationNorth America                 1.359315 0.8396563 2.1851925
## LocationAustralia/New Zealand/Oceania 1.277313 0.7405085 2.1771759
## LocationOther                         1.080754 0.5737581 1.9841384
```

### Create ROCR from data

```
pred.mtt = predict(m, type = "response") #repeat risk predictions from model m
rocr.pred.mtt = ROCR::prediction(pred.mtt, labels = ml$Any_Health_Problem) #ROCR prediction object
roc.perf.mtt = ROCR::performance(rocr.pred.mtt, measure = "tpr", x.measure = "fpr") # #ROCR performance object
plot(roc.perf.mtt, col = "blue")
abline(a = 0, b = 1, lty = 2) #diagonal for random assignment
```

### Report AUC from ROC for training and test data

```
  # Train AUC
auc <- ROCR::performance(rocr.pred.mtt, measure = "auc")
  auc <- auc@y.values[[1]]
  print(auc)
```

```
## [1] 0.5215712
```

### Calculate Nagelkerke R^2

```
NagelkerkeR2(m)
```

```
## $N
## [1] 2322
## 
## $R2
## [1] 0.002972702
```

### check assumptions of model

#### Cook’s distance

```
plot(m, which = 4, id.n = 3)
```

# Extract model results and display data for top 3 values using Cook’s distance

```
model.data <- augment(m) %>% 
  mutate(index = 1:n()) 
model.data %>% top_n(3, .cooksd)
```

#### plot standardised residuals

```
ggplot(model.data, aes(index, .std.resid)) + 
  geom_point(aes(color = Any_Health_Problem), alpha = .5) +
  theme_bw()
```

#### Filter potential influential data points with abs(.std.res) > 3:

```
model.data %>% 
  filter(abs(.std.resid) > 3)
```

## SETTING binary logistic regression for HEALTH

```
# fit binary logit model and store results 'm'
m <- glm(Any_Health_Problem ~ setting, data = ml,family = binomial)
# view a summary of the model
summary(m)
```

```
## 
## Call:
## glm(formula = Any_Health_Problem ~ setting, family = binomial, 
##     data = ml)
## 
## Coefficients:
##                                Estimate Std. Error z value Pr(>|z|)    
## (Intercept)                    -0.37823    0.07092  -5.333 9.66e-08 ***
## settingRural                   -0.14528    0.10333  -1.406    0.160    
## settingEqually urban and rural -0.14125    0.10396  -1.359    0.174    
## ---
## Signif. codes:  0 '***' 0.001 '**' 0.01 '*' 0.05 '.' 0.1 ' ' 1
## 
## (Dispersion parameter for binomial family taken to be 1)
## 
##     Null deviance: 3094.1  on 2321  degrees of freedom
## Residual deviance: 3091.5  on 2319  degrees of freedom
## AIC: 3097.5
## 
## Number of Fisher Scoring iterations: 4
```

```
# test model fit
with(m, null.deviance - deviance)
```

```
## [1] 2.598945
```

```
with(m, df.null - df.residual)
```

```
## [1] 2
```

```
with(m, pchisq(null.deviance - deviance, df.null - df.residual, lower.tail = FALSE))
```

```
## [1] 0.2726756
```

```
BIC(m)
```

```
## [1] 3114.776
```

```
# Hosmer-Lemeshow Goodness-of-Fit Test
glmtoolbox::hltest(m)
```

```
## 
##    The Hosmer-Lemeshow goodness-of-fit test
## 
##  Group Size Observed Expected
##      1  758      282      282
##      2  740      276      276
##      3  824      335      335
## 
##          Statistic =  0 
## degrees of freedom =  1 
##            p-value =  1
```

```
## CIs using profiled log-likelihood
confint(m, level=0.99)
```

```
## Waiting for profiling to be done...
```

```
##                                     0.5 %     99.5 %
## (Intercept)                    -0.5620882 -0.1964593
## settingRural                   -0.4119092  0.1206385
## settingEqually urban and rural -0.4095464  0.1262724
```

```
## CIs using standard errors
confint.default(m, level=0.99)
```

```
##                                     0.5 %     99.5 %
## (Intercept)                    -0.5609174 -0.1955466
## settingRural                   -0.4114382  0.1208806
## settingEqually urban and rural -0.4090442  0.1265407
```

```
# Wald test
wald.test(b = coef(m), Sigma = vcov(m), Terms = 2)
```

```
## Wald test:
## ----------
## 
## Chi-squared test:
## X2 = 2.0, df = 1, P(> X2) = 0.16
```

```
## odds ratios and 95% CI
exp(cbind(OR = coef(m), confint(m, level=0.99)))
```

```
## Waiting for profiling to be done...
```

```
##                                       OR     0.5 %    99.5 %
## (Intercept)                    0.6850716 0.5700175 0.8216347
## settingRural                   0.8647811 0.6623844 1.1282169
## settingEqually urban and rural 0.8682707 0.6639514 1.1345912
```

### Create ROCR from data

```
pred.mtt = predict(m, type = "response") #repeat risk predictions from model m
rocr.pred.mtt = ROCR::prediction(pred.mtt, labels = ml$Any_Health_Problem) #ROCR prediction object
roc.perf.mtt = ROCR::performance(rocr.pred.mtt, measure = "tpr", x.measure = "fpr") # #ROCR performance object
plot(roc.perf.mtt, col = "blue")
abline(a = 0, b = 1, lty = 2) #diagonal for random assignment
```

### Report AUC from ROC for training and test data

```
  # Train AUC
auc <- ROCR::performance(rocr.pred.mtt, measure = "auc")
  auc <- auc@y.values[[1]]
  print(auc)
```

```
## [1] 0.5166782
```

### Calculate Nagelkerke R^2

```
NagelkerkeR2(m)
```

```
## $N
## [1] 2322
## 
## $R2
## [1] 0.001519504
```

### check assumptions of model

#### Cook’s distance

```
plot(m, which = 4, id.n = 3)
```

#### Extract model results and display data for top 3 values using Cook’s distance

```
model.data <- augment(m) %>% 
  mutate(index = 1:n()) 
model.data %>% top_n(3, .cooksd)
```

#### plot standardised residuals

```
ggplot(model.data, aes(index, .std.resid)) + 
  geom_point(aes(color = Any_Health_Problem), alpha = .5) +
  theme_bw()
```

#### Filter potential influential data points with abs(.std.res) > 3:

```
model.data %>% 
  filter(abs(.std.resid) > 3)
```

## URBAN binary logistic regression for HEALTH

```
# fit binary logit model and store results 'm'
m <- glm(Any_Health_Problem ~ Urban, data = ml,family = binomial)
# view a summary of the model
summary(m)
```

```
## 
## Call:
## glm(formula = Any_Health_Problem ~ Urban, family = binomial, 
##     data = ml)
## 
## Coefficients:
##             Estimate Std. Error z value Pr(>|z|)    
## (Intercept) -0.52152    0.05344  -9.759   <2e-16 ***
## UrbanYes     0.14329    0.08880   1.614    0.107    
## ---
## Signif. codes:  0 '***' 0.001 '**' 0.01 '*' 0.05 '.' 0.1 ' ' 1
## 
## (Dispersion parameter for binomial family taken to be 1)
## 
##     Null deviance: 3094.1  on 2321  degrees of freedom
## Residual deviance: 3091.5  on 2320  degrees of freedom
## AIC: 3095.5
## 
## Number of Fisher Scoring iterations: 4
```

```
# test model fit
with(m, null.deviance - deviance)
```

```
## [1] 2.597525
```

```
with(m, df.null - df.residual)
```

```
## [1] 1
```

```
with(m, pchisq(null.deviance - deviance, df.null - df.residual, lower.tail = FALSE))
```

```
## [1] 0.1070307
```

```
BIC(m)
```

```
## [1] 3107.028
```

```
## CIs using profiled log-likelihood
confint(m, level=0.99)
```

```
## Waiting for profiling to be done...
```

```
##                   0.5 %     99.5 %
## (Intercept) -0.66003844 -0.3846170
## UrbanYes    -0.08591073  0.3717427
```

```
## CIs using standard errors
confint.default(m, level=0.99)
```

```
##                   0.5 %     99.5 %
## (Intercept) -0.65917590 -0.3838659
## UrbanYes    -0.08545293  0.3720308
```

```
# Wald test
wald.test(b = coef(m), Sigma = vcov(m), Terms = 2)
```

```
## Wald test:
## ----------
## 
## Chi-squared test:
## X2 = 2.6, df = 1, P(> X2) = 0.11
```

```
## odds ratios and 95% CI
exp(cbind(OR = coef(m), confint(m, level=0.99)))
```

```
## Waiting for profiling to be done...
```

```
##                   OR     0.5 %    99.5 %
## (Intercept) 0.593617 0.5168315 0.6807113
## UrbanYes    1.154063 0.9176761 1.4502598
```

### Create ROCR from data

```
pred.mtt = predict(m, type = "response") #repeat risk predictions from model m
rocr.pred.mtt = ROCR::prediction(pred.mtt, labels = ml$Any_Health_Problem) #ROCR prediction object
roc.perf.mtt = ROCR::performance(rocr.pred.mtt, measure = "tpr", x.measure = "fpr") # #ROCR performance object
plot(roc.perf.mtt, col = "blue")
abline(a = 0, b = 1, lty = 2) #diagonal for random assignment
```

### Report AUC from ROC for training and test data

```
  # Train AUC
auc <- ROCR::performance(rocr.pred.mtt, measure = "auc")
  auc <- auc@y.values[[1]]
  print(auc)
```

```
## [1] 0.5164713
```

### Calculate Nagelkerke R^2

```
NagelkerkeR2(m)
```

```
## $N
## [1] 2322
## 
## $R2
## [1] 0.001518675
```

### check assumptions of model

#### Cook’s distance

```
plot(m, which = 4, id.n = 3)
```

#### Extract model results and display data for top 3 values using Cook’s distance

```
model.data <- augment(m) %>% 
  mutate(index = 1:n()) 
model.data %>% top_n(3, .cooksd)
```

#### plot standardised residuals

```
ggplot(model.data, aes(index, .std.resid)) + 
  geom_point(aes(color = Any_Health_Problem), alpha = .5) +
  theme_bw()
```

#### Filter potential influential data points with abs(.std.res) > 3:

```
model.data %>% 
  filter(abs(.std.resid) > 3)
```

## Location + URBAN binary logistic regression for HEALTH

```
# fit binary logit model and store results 'm'
m <- glm(Any_Health_Problem ~ Location + Urban, data = ml,family = binomial)
# view a summary of the model
summary(m)
```

```
## 
## Call:
## glm(formula = Any_Health_Problem ~ Location + Urban, family = binomial, 
##     data = ml)
## 
## Coefficients:
##                                       Estimate Std. Error z value Pr(>|z|)    
## (Intercept)                           -0.56026    0.05766  -9.716   <2e-16 ***
## LocationOther European                 0.14871    0.11962   1.243    0.214    
## LocationNorth America                  0.28919    0.18557   1.558    0.119    
## LocationAustralia/New Zealand/Oceania  0.19762    0.21173   0.933    0.351    
## LocationOther                          0.01838    0.24386   0.075    0.940    
## UrbanYes                               0.11511    0.09222   1.248    0.212    
## ---
## Signif. codes:  0 '***' 0.001 '**' 0.01 '*' 0.05 '.' 0.1 ' ' 1
## 
## (Dispersion parameter for binomial family taken to be 1)
## 
##     Null deviance: 3094.1  on 2321  degrees of freedom
## Residual deviance: 3087.5  on 2316  degrees of freedom
## AIC: 3099.5
## 
## Number of Fisher Scoring iterations: 4
```

```
# test model fit
with(m, null.deviance - deviance)
```

```
## [1] 6.641709
```

```
with(m, df.null - df.residual)
```

```
## [1] 5
```

```
with(m, pchisq(null.deviance - deviance, df.null - df.residual, lower.tail = FALSE))
```

```
## [1] 0.2486792
```

```
BIC(m)
```

```
## [1] 3133.984
```

```
# Hosmer-Lemeshow Goodness-of-Fit Test
hltest(m)
```

```
## 
##    The Hosmer-Lemeshow goodness-of-fit test
## 
##  Group Size Observed   Expected
##      1 1179      431 428.552539
##      2   15        2   5.516268
##      3  480      185 187.447461
##      4  265      107 105.387408
##      5  256      114 109.123841
##      6  127       54  56.972483
## 
##          Statistic =  4.32151 
## degrees of freedom =  4 
##            p-value =  0.36424
```

```
## CIs using profiled log-likelihood
confint(m, level=0.99)
```

```
## Waiting for profiling to be done...
```

```
##                                            0.5 %     99.5 %
## (Intercept)                           -0.7097682 -0.4125851
## LocationOther European                -0.1615166  0.4553955
## LocationNorth America                 -0.1941458  0.7654458
## LocationAustralia/New Zealand/Oceania -0.3562468  0.7397514
## LocationOther                         -0.6263776  0.6382278
## UrbanYes                              -0.1230368  0.3522765
```

```
## CIs using standard errors
confint.default(m, level=0.99)
```

```
##                                            0.5 %     99.5 %
## (Intercept)                           -0.7087844 -0.4117282
## LocationOther European                -0.1594078  0.4568184
## LocationNorth America                 -0.1888121  0.7671957
## LocationAustralia/New Zealand/Oceania -0.3477605  0.7430063
## LocationOther                         -0.6097717  0.6465316
## UrbanYes                              -0.1224416  0.3526629
```

```
# Wald test
wald.test(b = coef(m), Sigma = vcov(m), Terms = 2)
```

```
## Wald test:
## ----------
## 
## Chi-squared test:
## X2 = 1.5, df = 1, P(> X2) = 0.21
```

```
## odds ratios and 95% CI
exp(cbind(OR = coef(m), confint(m, level=0.99)))
```

```
## Waiting for profiling to be done...
```

```
##                                              OR     0.5 %    99.5 %
## (Intercept)                           0.5710627 0.4917582 0.6619369
## LocationOther European                1.1603310 0.8508524 1.5767969
## LocationNorth America                 1.3353478 0.8235378 2.1499527
## LocationAustralia/New Zealand/Oceania 1.2185028 0.7002998 2.0954146
## LocationOther                         1.0185499 0.5345246 1.8931229
## UrbanYes                              1.1219976 0.8842311 1.4223017
```

### Create ROCR from data

```
pred.mtt = predict(m, type = "response") #repeat risk predictions from model m
rocr.pred.mtt = ROCR::prediction(pred.mtt, labels = ml$Any_Health_Problem) #ROCR prediction object
roc.perf.mtt = ROCR::performance(rocr.pred.mtt, measure = "tpr", x.measure = "fpr") # #ROCR performance object
plot(roc.perf.mtt, col = "blue")
abline(a = 0, b = 1, lty = 2) #diagonal for random assignment
```

### Report AUC from ROC for training and test data

```
  # Train AUC
auc <- ROCR::performance(rocr.pred.mtt, measure = "auc")
  auc <- auc@y.values[[1]]
  print(auc)
```

```
## [1] 0.5277052
```

### Calculate Nagelkerke R^2

```
NagelkerkeR2(m)
```

```
## $N
## [1] 2322
## 
## $R2
## [1] 0.003879777
```

### check assumptions of model

#### Cook’s distance

```
plot(m, which = 4, id.n = 3)
```

# Extract model results and display data for top 3 values using Cook’s distance

```
model.data <- augment(m) %>% 
  mutate(index = 1:n()) 
model.data %>% top_n(3, .cooksd)
```

#### plot standardised residuals

```
ggplot(model.data, aes(index, .std.resid)) + 
  geom_point(aes(color = Any_Health_Problem), alpha = .5) +
  theme_bw()
```

#### Filter potential influential data points with abs(.std.res) > 3:

```
model.data %>% 
  filter(abs(.std.resid) > 3)
```

#### check for multicollinearity

```
car::vif(m)
```

```
##              GVIF Df GVIF^(1/(2*Df))
## Location 1.076421  4        1.009248
## Urban    1.076421  1        1.037507
```

## LOCATION \* URBAN binary logistic regression for HEALTH

```
# fit binary logit model and store results 'm'
m <- glm(Any_Health_Problem ~ Location*Urban, data = ml,family = binomial)
# view a summary of the model
summary(m)
```

```
## 
## Call:
## glm(formula = Any_Health_Problem ~ Location * Urban, family = binomial, 
##     data = ml)
## 
## Coefficients:
##                                                 Estimate Std. Error z value
## (Intercept)                                    -0.551295   0.060474  -9.116
## LocationOther European                          0.100461   0.156140   0.643
## LocationNorth America                           0.296403   0.246810   1.201
## LocationAustralia/New Zealand/Oceania           0.551295   0.370122   1.489
## LocationOther                                  -1.320507   0.761957  -1.733
## UrbanYes                                        0.084675   0.111590   0.759
## LocationOther European:UrbanYes                 0.119758   0.243670   0.491
## LocationNorth America:UrbanYes                 -0.005674   0.374740  -0.015
## LocationAustralia/New Zealand/Oceania:UrbanYes -0.490140   0.453111  -1.082
## LocationOther:UrbanYes                          1.592971   0.809012   1.969
##                                                Pr(>|z|)    
## (Intercept)                                      <2e-16 ***
## LocationOther European                           0.5200    
## LocationNorth America                            0.2298    
## LocationAustralia/New Zealand/Oceania            0.1364    
## LocationOther                                    0.0831 .  
## UrbanYes                                         0.4480    
## LocationOther European:UrbanYes                  0.6231    
## LocationNorth America:UrbanYes                   0.9879    
## LocationAustralia/New Zealand/Oceania:UrbanYes   0.2794    
## LocationOther:UrbanYes                           0.0489 *  
## ---
## Signif. codes:  0 '***' 0.001 '**' 0.01 '*' 0.05 '.' 0.1 ' ' 1
## 
## (Dispersion parameter for binomial family taken to be 1)
## 
##     Null deviance: 3094.1  on 2321  degrees of freedom
## Residual deviance: 3080.9  on 2312  degrees of freedom
## AIC: 3100.9
## 
## Number of Fisher Scoring iterations: 4
```

```
# test model fit
with(m, null.deviance - deviance)
```

```
## [1] 13.26466
```

```
with(m, df.null - df.residual)
```

```
## [1] 9
```

```
with(m, pchisq(null.deviance - deviance, df.null - df.residual, lower.tail = FALSE))
```

```
## [1] 0.150992
```

```
BIC(m)
```

```
## [1] 3158.362
```

```
# Hosmer-Lemeshow Goodness-of-Fit Test
hltest(m)
```

```
## 
##    The Hosmer-Lemeshow goodness-of-fit test
## 
##  Group Size Observed Expected
##      1   15        2        2
##      2 1179      431      431
##      3  480      185      185
##      4  203       79       79
##      5  296      127      127
##      6  149       69       69
## 
##          Statistic =  0 
## degrees of freedom =  4 
##            p-value =  1
```

```
## CIs using profiled log-likelihood
confint(m, level=0.99)
```

```
## Waiting for profiling to be done...
```

```
##                                                     0.5 %     99.5 %
## (Intercept)                                    -0.7082355 -0.3965331
## LocationOther European                         -0.3067928  0.4993486
## LocationNorth America                          -0.3514315  0.9290856
## LocationAustralia/New Zealand/Oceania          -0.4188685  1.5215106
## LocationOther                                  -3.9749634  0.3243378
## UrbanYes                                       -0.2042295  0.3710581
## LocationOther European:UrbanYes                -0.5081721  0.7486114
## LocationNorth America:UrbanYes                 -0.9748740  0.9632933
## LocationAustralia/New Zealand/Oceania:UrbanYes -1.6710413  0.6835282
## LocationOther:UrbanYes                         -0.2132031  4.3153980
```

```
## CIs using standard errors
confint.default(m, level=0.99)
```

```
##                                                     0.5 %     99.5 %
## (Intercept)                                    -0.7070651 -0.3955246
## LocationOther European                         -0.3017299  0.5026522
## LocationNorth America                          -0.3393376  0.9321429
## LocationAustralia/New Zealand/Oceania          -0.4020766  1.5046664
## LocationOther                                  -3.2831793  0.6421648
## UrbanYes                                       -0.2027613  0.3721120
## LocationOther European:UrbanYes                -0.5078951  0.7474110
## LocationNorth America:UrbanYes                 -0.9709398  0.9595922
## LocationAustralia/New Zealand/Oceania:UrbanYes -1.6572767  0.6769958
## LocationOther:UrbanYes                         -0.4909058  3.6768474
```

```
# Wald test
wald.test(b = coef(m), Sigma = vcov(m), Terms = 2)
```

```
## Wald test:
## ----------
## 
## Chi-squared test:
## X2 = 0.41, df = 1, P(> X2) = 0.52
```

```
## odds ratios and 95% CI
exp(cbind(OR = coef(m), confint(m, level=0.99)))
```

```
## Waiting for profiling to be done...
```

```
##                                                       OR      0.5 %    99.5 %
## (Intercept)                                    0.5762032 0.49251246  0.672648
## LocationOther European                         1.1056807 0.73580303  1.647648
## LocationNorth America                          1.3450116 0.70368004  2.532193
## LocationAustralia/New Zealand/Oceania          1.7354988 0.65779067  4.579137
## LocationOther                                  0.2669998 0.01877999  1.383114
## UrbanYes                                       1.0883637 0.81527526  1.449267
## LocationOther European:UrbanYes                1.1272240 0.60159425  2.114062
## LocationNorth America:UrbanYes                 0.9943423 0.37723989  2.620312
## LocationAustralia/New Zealand/Oceania:UrbanYes 0.6125403 0.18805115  1.980854
## LocationOther:UrbanYes                         4.9183387 0.80799201 74.843402
```

### Create ROCR from data

```
pred.mtt = predict(m, type = "response") #repeat risk predictions from model m
rocr.pred.mtt = ROCR::prediction(pred.mtt, labels = ml$Any_Health_Problem) #ROCR prediction object
roc.perf.mtt = ROCR::performance(rocr.pred.mtt, measure = "tpr", x.measure = "fpr") # #ROCR performance object
plot(roc.perf.mtt, col = "blue")
abline(a = 0, b = 1, lty = 2) #diagonal for random assignment
```

### Report AUC from ROC for training and test data

```
  # Train AUC
auc <- ROCR::performance(rocr.pred.mtt, measure = "auc")
  auc <- auc@y.values[[1]]
  print(auc)
```

```
## [1] 0.5329062
```

### Calculate Nagelkerke R^2

```
NagelkerkeR2(m)
```

```
## $N
## [1] 2322
## 
## $R2
## [1] 0.00773756
```

### check assumptions of model

#### Cook’s distance

```
plot(m, which = 4, id.n = 3)
```

#### Extract model results and display data for top 3 values using Cook’s distance

```
model.data <- augment(m) %>% 
  mutate(index = 1:n()) 
model.data %>% top_n(3, .cooksd)
```

#### plot standardised residuals

```
ggplot(model.data, aes(index, .std.resid)) + 
  geom_point(aes(color = Any_Health_Problem), alpha = .5) +
  theme_bw()
```

#### Filter potential influential data points with abs(.std.res) > 3:

```
model.data %>% 
  filter(abs(.std.resid) > 3)
```

# check for multicollinearity

```
car::vif(m)
```

```
## there are higher-order terms (interactions) in this model
## consider setting type = 'predictor'; see ?vif
```

```
##                      GVIF Df GVIF^(1/(2*Df))
## Location        93.427325  4        1.763231
## Urban            1.572918  1        1.254160
## Location:Urban 126.616139  4        1.831518
```

## EDUCATION Binary logistic regression for HEALTH

```
# fit binary logit model and store results 'm'
m <- glm(Any_Health_Problem ~ Education, data = ml,family = binomial)
# view a summary of the model
summary(m)
```

```
## 
## Call:
## glm(formula = Any_Health_Problem ~ Education, family = binomial, 
##     data = ml)
## 
## Coefficients:
##             Estimate Std. Error z value Pr(>|z|)    
## (Intercept) -0.38690    0.07953  -4.865 1.15e-06 ***
## Education.L -0.52335    0.26938  -1.943   0.0520 .  
## Education.Q  0.28010    0.24674   1.135   0.2563    
## Education.C -0.31171    0.18325  -1.701   0.0889 .  
## Education^4  0.05402    0.12258   0.441   0.6595    
## Education^5 -0.04123    0.08783  -0.469   0.6387    
## ---
## Signif. codes:  0 '***' 0.001 '**' 0.01 '*' 0.05 '.' 0.1 ' ' 1
## 
## (Dispersion parameter for binomial family taken to be 1)
## 
##     Null deviance: 3094.1  on 2321  degrees of freedom
## Residual deviance: 3090.0  on 2316  degrees of freedom
## AIC: 3102
## 
## Number of Fisher Scoring iterations: 4
```

```
# test model fit
with(m, null.deviance - deviance)
```

```
## [1] 4.169829
```

```
with(m, df.null - df.residual)
```

```
## [1] 5
```

```
with(m, pchisq(null.deviance - deviance, df.null - df.residual, lower.tail = FALSE))
```

```
## [1] 0.525233
```

```
BIC(m)
```

```
## [1] 3136.456
```

```
# Hosmer-Lemeshow Goodness-of-Fit Test
hltest(m)
```

```
## 
##    The Hosmer-Lemeshow goodness-of-fit test
## 
##  Group Size Observed Expected
##      1   79       27       27
##      2  662      251      251
##      3  682      260      260
##      4  460      178      178
##      5  410      161      161
##      6   29       16       16
## 
##          Statistic =  0 
## degrees of freedom =  4 
##            p-value =  1
```

```
## CIs using profiled log-likelihood
confint(m, level=0.99)
```

```
## Waiting for profiling to be done...
```

```
##                  0.5 %     99.5 %
## (Intercept) -0.5925898 -0.1794892
## Education.L -1.2340155  0.1675013
## Education.Q -0.3585546  0.9254636
## Education.C -0.7923258  0.1584270
## Education^4 -0.2615278  0.3715823
## Education^5 -0.2676360  0.1849834
```

```
## CIs using standard errors
confint.default(m, level=0.99)
```

```
##                  0.5 %     99.5 %
## (Intercept) -0.5917628 -0.1820384
## Education.L -1.2172132  0.1705182
## Education.Q -0.3554454  0.9156522
## Education.C -0.7837294  0.1603177
## Education^4 -0.2617379  0.3697680
## Education^5 -0.2674543  0.1849944
```

```
# Wald test
wald.test(b = coef(m), Sigma = vcov(m), Terms = 2)
```

```
## Wald test:
## ----------
## 
## Chi-squared test:
## X2 = 3.8, df = 1, P(> X2) = 0.052
```

```
## odds ratios and 95% CI
exp(cbind(OR = coef(m), confint(m, level=0.99)))
```

```
## Waiting for profiling to be done...
```

```
##                    OR     0.5 %    99.5 %
## (Intercept) 0.6791586 0.5528935 0.8356969
## Education.L 0.5925337 0.2911212 1.1823468
## Education.Q 1.3232667 0.6986855 2.5230376
## Education.C 0.7321969 0.4527905 1.1716664
## Education^4 1.0555005 0.7698745 1.4500272
## Education^5 0.9596084 0.7651863 1.2031984
```

### Create ROCR from data

```
pred.mtt = predict(m, type = "response") #repeat risk predictions from model m
rocr.pred.mtt = ROCR::prediction(pred.mtt, labels = ml$Any_Health_Problem) #ROCR prediction object
roc.perf.mtt = ROCR::performance(rocr.pred.mtt, measure = "tpr", x.measure = "fpr") # #ROCR performance object
plot(roc.perf.mtt, col = "blue")
abline(a = 0, b = 1, lty = 2) #diagonal for random assignment
```

### Report AUC from ROC for training and test data

```
  # Train AUC
auc <- ROCR::performance(rocr.pred.mtt, measure = "auc")
  auc <- auc@y.values[[1]]
  print(auc)
```

```
## [1] 0.5124172
```

### Calculate Nagelkerke R^2

```
NagelkerkeR2(m)
```

```
## $N
## [1] 2322
## 
## $R2
## [1] 0.002437116
```

### check assumptions of model

#### Cook’s distance

```
plot(m, which = 4, id.n = 3)
```

#### Extract model results and display data for top 3 values using Cook’s distance

```
model.data <- augment(m) %>% 
  mutate(index = 1:n()) 
model.data %>% top_n(3, .cooksd)
```

#### plot standardised residuals

```
ggplot(model.data, aes(index, .std.resid)) + 
  geom_point(aes(color = Any_Health_Problem), alpha = .5) +
  theme_bw()
```

# Filter potential influential data points with abs(.std.res) > 3:

```
model.data %>% 
  filter(abs(.std.resid) > 3)
```

## EDUCATION\_S Binary logistic regression for HEALTH

```
# fit binary logit model and store results 'm'
m <- glm(Any_Health_Problem ~ Education_S, data = ml,family = binomial)
# view a summary of the model
summary(m)
```

```
## 
## Call:
## glm(formula = Any_Health_Problem ~ Education_S, family = binomial, 
##     data = ml)
## 
## Coefficients:
##                        Estimate Std. Error z value Pr(>|z|)    
## (Intercept)            -0.39219    0.09730  -4.031 5.56e-05 ***
## Education_S1_College   -0.10095    0.12603  -0.801    0.423    
## Education_S2_Grad      -0.09213    0.12523  -0.736    0.462    
## Education_S3_PG_or_PhD -0.09594    0.13168  -0.729    0.466    
## ---
## Signif. codes:  0 '***' 0.001 '**' 0.01 '*' 0.05 '.' 0.1 ' ' 1
## 
## (Dispersion parameter for binomial family taken to be 1)
## 
##     Null deviance: 3094.1  on 2321  degrees of freedom
## Residual deviance: 3093.3  on 2318  degrees of freedom
## AIC: 3101.3
## 
## Number of Fisher Scoring iterations: 4
```

```
# test model fit
with(m, null.deviance - deviance)
```

```
## [1] 0.7945791
```

```
with(m, df.null - df.residual)
```

```
## [1] 3
```

```
with(m, pchisq(null.deviance - deviance, df.null - df.residual, lower.tail = FALSE))
```

```
## [1] 0.8507632
```

```
BIC(m)
```

```
## [1] 3124.331
```

```
# Hosmer-Lemeshow Goodness-of-Fit Test
hltest(m, G=3)
```

```
## 
##    The Hosmer-Lemeshow goodness-of-fit test
## 
##  Group Size Observed Expected
##      1  662      251      251
##      2  539      205      205
##      3  682      260      260
##      4  439      177      177
## 
##          Statistic =  0 
## degrees of freedom =  2 
##            p-value =  1
```

```
## CIs using profiled log-likelihood
confint(m, level=0.99)
```

```
## Waiting for profiling to be done...
```

```
##                             0.5 %     99.5 %
## (Intercept)            -0.6451787 -0.1432762
## Education_S1_College   -0.4252820  0.2244287
## Education_S2_Grad      -0.4143384  0.2312413
## Education_S3_PG_or_PhD -0.4351858  0.2436386
```

```
## CIs using standard errors
confint.default(m, level=0.99)
```

```
##                             0.5 %     99.5 %
## (Intercept)            -0.6428127 -0.1415769
## Education_S1_College   -0.4255783  0.2236873
## Education_S2_Grad      -0.4146978  0.2304399
## Education_S3_PG_or_PhD -0.4351112  0.2432387
```

```
# Wald test
wald.test(b = coef(m), Sigma = vcov(m), Terms = 2)
```

```
## Wald test:
## ----------
## 
## Chi-squared test:
## X2 = 0.64, df = 1, P(> X2) = 0.42
```

```
## odds ratios and 95% CI
exp(cbind(OR = coef(m), confint(m, level=0.99)))
```

```
## Waiting for profiling to be done...
```

```
##                               OR     0.5 %    99.5 %
## (Intercept)            0.6755725 0.5245688 0.8665147
## Education_S1_College   0.9039823 0.6535854 1.2516075
## Education_S2_Grad      0.9119876 0.6607773 1.2601633
## Education_S3_PG_or_PhD 0.9085219 0.6471444 1.2758832
```

### Create ROCR from data

```
pred.mtt = predict(m, type = "response") #repeat risk predictions from model m
rocr.pred.mtt = ROCR::prediction(pred.mtt, labels = ml$Any_Health_Problem) #ROCR prediction object
roc.perf.mtt = ROCR::performance(rocr.pred.mtt, measure = "tpr", x.measure = "fpr") # #ROCR performance object
plot(roc.perf.mtt, col = "blue")
abline(a = 0, b = 1, lty = 2) #diagonal for random assignment
```

### Report AUC from ROC for training and test data

```
  # Train AUC
auc <- ROCR::performance(rocr.pred.mtt, measure = "auc")
  auc <- auc@y.values[[1]]
  print(auc)
```

```
## [1] 0.5080934
```

### Calculate Nagelkerke R^2

```
NagelkerkeR2(m)
```

```
## $N
## [1] 2322
## 
## $R2
## [1] 0.0004647407
```

### check assumptions of model

#### Cook’s distance

```
plot(m, which = 4, id.n = 3)
```

#### Extract model results and display data for top 3 values using Cook’s distance

```
model.data <- augment(m) %>% 
  mutate(index = 1:n()) 
model.data %>% top_n(3, .cooksd)
```

#### plot standardised residuals

```
ggplot(model.data, aes(index, .std.resid)) + 
  geom_point(aes(color = Any_Health_Problem), alpha = .5) +
  theme_bw()
```

#### Filter potential influential data points with abs(.std.res) > 3:

```
model.data %>% 
  filter(abs(.std.resid) > 3)
```

## EDUCATION\_S Binary logistic regression for HEALTH

```
# fit binary logit model and store results 'm'
m <- glm(Any_Health_Problem ~ Education_S2, data = ml,family = binomial)
# view a summary of the model
summary(m)
```

```
## 
## Call:
## glm(formula = Any_Health_Problem ~ Education_S2, family = binomial, 
##     data = ml)
## 
## Coefficients:
##                         Estimate Std. Error z value Pr(>|z|)    
## (Intercept)             -0.39219    0.09730  -4.031 5.56e-05 ***
## Education_S21_College   -0.10095    0.12603  -0.801    0.423    
## Education_S22_Grad      -0.09213    0.12523  -0.736    0.462    
## Education_S23_PG_or_PhD -0.09594    0.13168  -0.729    0.466    
## ---
## Signif. codes:  0 '***' 0.001 '**' 0.01 '*' 0.05 '.' 0.1 ' ' 1
## 
## (Dispersion parameter for binomial family taken to be 1)
## 
##     Null deviance: 3094.1  on 2321  degrees of freedom
## Residual deviance: 3093.3  on 2318  degrees of freedom
## AIC: 3101.3
## 
## Number of Fisher Scoring iterations: 4
```

```
# test model fit
with(m, null.deviance - deviance)
```

```
## [1] 0.7945791
```

```
with(m, df.null - df.residual)
```

```
## [1] 3
```

```
with(m, pchisq(null.deviance - deviance, df.null - df.residual, lower.tail = FALSE))
```

```
## [1] 0.8507632
```

```
BIC(m)
```

```
## [1] 3124.331
```

```
# Hosmer-Lemeshow Goodness-of-Fit Test
hltest(m, G=3)
```

```
## 
##    The Hosmer-Lemeshow goodness-of-fit test
## 
##  Group Size Observed Expected
##      1  662      251      251
##      2  539      205      205
##      3  682      260      260
##      4  439      177      177
## 
##          Statistic =  0 
## degrees of freedom =  2 
##            p-value =  1
```

```
## CIs using profiled log-likelihood
confint(m, level=0.99)
```

```
## Waiting for profiling to be done...
```

```
##                              0.5 %     99.5 %
## (Intercept)             -0.6451787 -0.1432762
## Education_S21_College   -0.4252820  0.2244287
## Education_S22_Grad      -0.4143384  0.2312413
## Education_S23_PG_or_PhD -0.4351858  0.2436386
```

```
## CIs using standard errors
confint.default(m, level=0.99)
```

```
##                              0.5 %     99.5 %
## (Intercept)             -0.6428127 -0.1415769
## Education_S21_College   -0.4255783  0.2236873
## Education_S22_Grad      -0.4146978  0.2304399
## Education_S23_PG_or_PhD -0.4351112  0.2432387
```

```
# Wald test
wald.test(b = coef(m), Sigma = vcov(m), Terms = 2)
```

```
## Wald test:
## ----------
## 
## Chi-squared test:
## X2 = 0.64, df = 1, P(> X2) = 0.42
```

```
## odds ratios and 95% CI
exp(cbind(OR = coef(m), confint(m, level=0.99)))
```

```
## Waiting for profiling to be done...
```

```
##                                OR     0.5 %    99.5 %
## (Intercept)             0.6755725 0.5245688 0.8665147
## Education_S21_College   0.9039823 0.6535854 1.2516075
## Education_S22_Grad      0.9119876 0.6607773 1.2601633
## Education_S23_PG_or_PhD 0.9085219 0.6471444 1.2758832
```

### Create ROCR from data

```
pred.mtt = predict(m, type = "response") #repeat risk predictions from model m
rocr.pred.mtt = ROCR::prediction(pred.mtt, labels = ml$Any_Health_Problem) #ROCR prediction object
roc.perf.mtt = ROCR::performance(rocr.pred.mtt, measure = "tpr", x.measure = "fpr") # #ROCR performance object
plot(roc.perf.mtt, col = "blue")
abline(a = 0, b = 1, lty = 2) #diagonal for random assignment
```

### Report AUC from ROC for training and test data

```
  # Train AUC
auc <- ROCR::performance(rocr.pred.mtt, measure = "auc")
  auc <- auc@y.values[[1]]
  print(auc)
```

```
## [1] 0.5080934
```

### Calculate Nagelkerke R^2

```
NagelkerkeR2(m)
```

```
## $N
## [1] 2322
## 
## $R2
## [1] 0.0004647407
```

### check assumptions of model

#### Cook’s distance

```
plot(m, which = 4, id.n = 3)
```

#### Extract model results and display data for top 3 values using Cook’s distance

```
model.data <- augment(m) %>% 
  mutate(index = 1:n()) 
model.data %>% top_n(3, .cooksd)
```

#### plot standardised residuals

```
ggplot(model.data, aes(index, .std.resid)) + 
  geom_point(aes(color = Any_Health_Problem), alpha = .5) +
  theme_bw()
```

#### Filter potential influential data points with abs(.std.res) > 3:

```
model.data %>% 
  filter(abs(.std.resid) > 3)
```

## ANIMAL CAREER 2 Binary logistic regression for HEALTH

```
# fit binary logit model and store results 'm'
m <- glm(Any_Health_Problem ~ Animal_Career2, data = ml,family = binomial)
# view a summary of the model
summary(m)
```

```
## 
## Call:
## glm(formula = Any_Health_Problem ~ Animal_Career2, family = binomial, 
##     data = ml)
## 
## Coefficients:
##                                   Estimate Std. Error z value Pr(>|z|)    
## (Intercept)                       -0.50546    0.04752 -10.637   <2e-16 ***
## Animal_Career2Vet professional     0.28641    0.19764   1.449    0.147    
## Animal_Career2breeder/trainer      0.02140    0.18098   0.118    0.906    
## Animal_Career2Pet industry worker  0.24595    0.15533   1.583    0.113    
## ---
## Signif. codes:  0 '***' 0.001 '**' 0.01 '*' 0.05 '.' 0.1 ' ' 1
## 
## (Dispersion parameter for binomial family taken to be 1)
## 
##     Null deviance: 3094.1  on 2321  degrees of freedom
## Residual deviance: 3089.9  on 2318  degrees of freedom
## AIC: 3097.9
## 
## Number of Fisher Scoring iterations: 4
```

```
# test model fit
with(m, null.deviance - deviance)
```

```
## [1] 4.260352
```

```
with(m, df.null - df.residual)
```

```
## [1] 3
```

```
with(m, pchisq(null.deviance - deviance, df.null - df.residual, lower.tail = FALSE))
```

```
## [1] 0.2346886
```

```
BIC(m)
```

```
## [1] 3120.865
```

```
## CIs using profiled log-likelihood
confint(m, level=0.99)
```

```
## Waiting for profiling to be done...
```

```
##                                        0.5 %     99.5 %
## (Intercept)                       -0.6285185 -0.3836378
## Animal_Career2Vet professional    -0.2291815  0.7936247
## Animal_Career2breeder/trainer     -0.4540031  0.4818767
## Animal_Career2Pet industry worker -0.1579574  0.6442762
```

```
## CIs using standard errors
confint.default(m, level=0.99)
```

```
##                                        0.5 %     99.5 %
## (Intercept)                       -0.6278603 -0.3830580
## Animal_Career2Vet professional    -0.2226690  0.7954801
## Animal_Career2breeder/trainer     -0.4447708  0.4875783
## Animal_Career2Pet industry worker -0.1541569  0.6460527
```

```
# Wald test
wald.test(b = coef(m), Sigma = vcov(m), Terms = 2)
```

```
## Wald test:
## ----------
## 
## Chi-squared test:
## X2 = 2.1, df = 1, P(> X2) = 0.15
```

```
## odds ratios and 95% CI
exp(cbind(OR = coef(m), confint(m, level=0.99)))
```

```
## Waiting for profiling to be done...
```

```
##                                          OR     0.5 %    99.5 %
## (Intercept)                       0.6032285 0.5333814 0.6813782
## Animal_Career2Vet professional    1.3316324 0.7951842 2.2113976
## Animal_Career2breeder/trainer     1.0216345 0.6350807 1.6191101
## Animal_Career2Pet industry worker 1.2788330 0.8538862 1.9046079
```

### Create ROCR from data

```
pred.mtt = predict(m, type = "response") #repeat risk predictions from model m
rocr.pred.mtt = ROCR::prediction(pred.mtt, labels = ml$Any_Health_Problem) #ROCR prediction object
roc.perf.mtt = ROCR::performance(rocr.pred.mtt, measure = "tpr", x.measure = "fpr") # #ROCR performance object
plot(roc.perf.mtt, col = "blue")
abline(a = 0, b = 1, lty = 2) #diagonal for random assignment
```

### Report AUC from ROC for training and test data

```
  # Train AUC
auc <- ROCR::performance(rocr.pred.mtt, measure = "auc")
  auc <- auc@y.values[[1]]
  print(auc)
```

```
## [1] 0.5153033
```

### Calculate Nagelkerke R^2

```
NagelkerkeR2(m)
```

```
## $N
## [1] 2322
## 
## $R2
## [1] 0.002489975
```

### check assumptions of model

#### Cook’s distance

```
plot(m, which = 4, id.n = 3)
```

#### Extract model results and display data for top 3 values using Cook’s distance

```
model.data <- augment(m) %>% 
  mutate(index = 1:n()) 
model.data %>% top_n(3, .cooksd)
```

#### plot standardised residuals

```
ggplot(model.data, aes(index, .std.resid)) + 
  geom_point(aes(color = Any_Health_Problem), alpha = .5) +
  theme_bw()
```

#### Filter potential influential data points with abs(.std.res) > 3:

```
model.data %>% 
  filter(abs(.std.resid) > 3)
```

## ANIMAL\_CAREER\_BINARY Binary logistic regression for HEALTH

```
# fit binary logit model and store results 'm'
m <- glm(Any_Health_Problem ~ Animal_Career_BINARY, data = ml,family = binomial)
# view a summary of the model
summary(m)
```

```
## 
## Call:
## glm(formula = Any_Health_Problem ~ Animal_Career_BINARY, family = binomial, 
##     data = ml)
## 
## Coefficients:
##                         Estimate Std. Error z value Pr(>|z|)    
## (Intercept)             -0.50546    0.04752 -10.637   <2e-16 ***
## Animal_Career_BINARYYes  0.18552    0.10812   1.716   0.0862 .  
## ---
## Signif. codes:  0 '***' 0.001 '**' 0.01 '*' 0.05 '.' 0.1 ' ' 1
## 
## (Dispersion parameter for binomial family taken to be 1)
## 
##     Null deviance: 3094.1  on 2321  degrees of freedom
## Residual deviance: 3091.2  on 2320  degrees of freedom
## AIC: 3095.2
## 
## Number of Fisher Scoring iterations: 4
```

```
# test model fit
with(m, null.deviance - deviance)
```

```
## [1] 2.924578
```

```
with(m, df.null - df.residual)
```

```
## [1] 1
```

```
with(m, pchisq(null.deviance - deviance, df.null - df.residual, lower.tail = FALSE))
```

```
## [1] 0.08724002
```

```
BIC(m)
```

```
## [1] 3106.701
```

```
## CIs using profiled log-likelihood
confint(m, level=0.99)
```

```
## Waiting for profiling to be done...
```

```
##                               0.5 %     99.5 %
## (Intercept)             -0.62851848 -0.3836378
## Animal_Career_BINARYYes -0.09446253  0.4630529
```

```
## CIs using standard errors
confint.default(m, level=0.99)
```

```
##                               0.5 %     99.5 %
## (Intercept)             -0.62786032 -0.3830580
## Animal_Career_BINARYYes -0.09299281  0.4640252
```

```
# Wald test
wald.test(b = coef(m), Sigma = vcov(m), Terms = 2)
```

```
## Wald test:
## ----------
## 
## Chi-squared test:
## X2 = 2.9, df = 1, P(> X2) = 0.086
```

```
## odds ratios and 95% CI
exp(cbind(OR = coef(m), confint(m, level=0.99)))
```

```
## Waiting for profiling to be done...
```

```
##                                OR     0.5 %    99.5 %
## (Intercept)             0.6032285 0.5333814 0.6813782
## Animal_Career_BINARYYes 1.2038397 0.9098618 1.5889175
```

### Create ROCR from data

```
pred.mtt = predict(m, type = "response") #repeat risk predictions from model m
rocr.pred.mtt = ROCR::prediction(pred.mtt, labels = ml$Any_Health_Problem) #ROCR prediction object
roc.perf.mtt = ROCR::performance(rocr.pred.mtt, measure = "tpr", x.measure = "fpr") # #ROCR performance object
plot(roc.perf.mtt, col = "blue")
abline(a = 0, b = 1, lty = 2) #diagonal for random assignment
```

### Report AUC from ROC for training and test data

```
  # Train AUC
auc <- ROCR::performance(rocr.pred.mtt, measure = "auc")
  auc <- auc@y.values[[1]]
  print(auc)
```

```
## [1] 0.5142901
```

### Calculate Nagelkerke R^2

```
NagelkerkeR2(m)
```

```
## $N
## [1] 2322
## 
## $R2
## [1] 0.00170977
```

### check assumptions of model

#### Cook’s distance

```
plot(m, which = 4, id.n = 3)
```

#### Extract model results and display data for top 3 values using Cook’s distance

```
model.data <- augment(m) %>% 
  mutate(index = 1:n()) 
model.data %>% top_n(3, .cooksd)
```

#### plot standardised residuals

```
ggplot(model.data, aes(index, .std.resid)) + 
  geom_point(aes(color = Any_Health_Problem), alpha = .5) +
  theme_bw()
```

#### Filter potential influential data points with abs(.std.res) > 3:

```
model.data %>% 
  filter(abs(.std.resid) > 3)
```

## INCOME Binary logistic regression for HEALTH

```
# fit binary logit model and store results 'm'
m <- glm(Any_Health_Problem ~ Income, data = ml,family = binomial)
# view a summary of the model
summary(m)
```

```
## 
## Call:
## glm(formula = Any_Health_Problem ~ Income, family = binomial, 
##     data = ml)
## 
## Coefficients:
##             Estimate Std. Error z value Pr(>|z|)    
## (Intercept) -0.46219    0.05419  -8.529   <2e-16 ***
## Income.L    -0.17088    0.10909  -1.566    0.117    
## Income.Q     0.02120    0.07562   0.280    0.779    
## ---
## Signif. codes:  0 '***' 0.001 '**' 0.01 '*' 0.05 '.' 0.1 ' ' 1
## 
## (Dispersion parameter for binomial family taken to be 1)
## 
##     Null deviance: 3094.1  on 2321  degrees of freedom
## Residual deviance: 3091.6  on 2319  degrees of freedom
## AIC: 3097.6
## 
## Number of Fisher Scoring iterations: 4
```

```
# test model fit
with(m, null.deviance - deviance)
```

```
## [1] 2.568457
```

```
with(m, df.null - df.residual)
```

```
## [1] 2
```

```
with(m, pchisq(null.deviance - deviance, df.null - df.residual, lower.tail = FALSE))
```

```
## [1] 0.2768641
```

```
BIC(m)
```

```
## [1] 3114.807
```

```
# Hosmer-Lemeshow Goodness-of-Fit Test
glmtoolbox::hltest(m)
```

```
## 
##    The Hosmer-Lemeshow goodness-of-fit test
## 
##  Group Size Observed Expected
##      1  347      125      125
##      2 1611      616      616
##      3  364      152      152
## 
##          Statistic =  0 
## degrees of freedom =  1 
##            p-value =  1
```

```
## CIs using profiled log-likelihood
confint(m, level=0.99)
```

```
## Waiting for profiling to be done...
```

```
##                  0.5 %     99.5 %
## (Intercept) -0.6027987 -0.3234343
## Income.L    -0.4528881  0.1096361
## Income.Q    -0.1743753  0.2154029
```

```
## CIs using standard errors
confint.default(m, level=0.99)
```

```
##                  0.5 %     99.5 %
## (Intercept) -0.6017721 -0.3226047
## Income.L    -0.4518703  0.1101144
## Income.Q    -0.1735977  0.2159919
```

```
# Wald test
wald.test(b = coef(m), Sigma = vcov(m), Terms = 2)
```

```
## Wald test:
## ----------
## 
## Chi-squared test:
## X2 = 2.5, df = 1, P(> X2) = 0.12
```

```
## odds ratios and 95% CI
exp(cbind(OR = coef(m), confint(m, level=0.99)))
```

```
## Waiting for profiling to be done...
```

```
##                    OR     0.5 %    99.5 %
## (Intercept) 0.6299037 0.5472778 0.7236595
## Income.L    0.8429245 0.6357892 1.1158719
## Income.Q    1.0214234 0.8399816 1.2403615
```

### Create ROCR from data

```
pred.mtt = predict(m, type = "response") #repeat risk predictions from model m
rocr.pred.mtt = ROCR::prediction(pred.mtt, labels = ml$Any_Health_Problem) #ROCR prediction object
roc.perf.mtt = ROCR::performance(rocr.pred.mtt, measure = "tpr", x.measure = "fpr") # #ROCR performance object
plot(roc.perf.mtt, col = "blue")
abline(a = 0, b = 1, lty = 2) #diagonal for random assignment
```

### Report AUC from ROC for training and test data

```
  # Train AUC
auc <- ROCR::performance(rocr.pred.mtt, measure = "auc")
  auc <- auc@y.values[[1]]
  print(auc)
```

```
## [1] 0.5157782
```

### Calculate Nagelkerke R^2

```
NagelkerkeR2(m)
```

```
## $N
## [1] 2322
## 
## $R2
## [1] 0.001501689
```

### check assumptions of model

#### Cook’s distance

```
plot(m, which = 4, id.n = 3)
```

#### Extract model results and display data for top 3 values using Cook’s distance

```
model.data <- augment(m) %>% 
  mutate(index = 1:n()) 
model.data %>% top_n(3, .cooksd)
```

#### plot standardised residuals

```
ggplot(model.data, aes(index, .std.resid)) + 
  geom_point(aes(color = Any_Health_Problem), alpha = .5) +
  theme_bw()
```

#### Filter potential influential data points with abs(.std.res) > 3:

```
model.data %>% 
  filter(abs(.std.resid) > 3)
```

## INCOME2 Binary logistic regression for HEALTH

```
# fit binary logit model and store results 'm'
m <- glm(Any_Health_Problem ~ Income2, data = ml,family = binomial)
# view a summary of the model
summary(m)
```

```
## 
## Call:
## glm(formula = Any_Health_Problem ~ Income2, family = binomial, 
##     data = ml)
## 
## Coefficients:
##               Estimate Std. Error z value Pr(>|z|)   
## (Intercept)    -0.3327     0.1063  -3.130  0.00175 **
## Income2Medium  -0.1468     0.1180  -1.244  0.21351   
## Income2High    -0.2417     0.1543  -1.566  0.11725   
## ---
## Signif. codes:  0 '***' 0.001 '**' 0.01 '*' 0.05 '.' 0.1 ' ' 1
## 
## (Dispersion parameter for binomial family taken to be 1)
## 
##     Null deviance: 3094.1  on 2321  degrees of freedom
## Residual deviance: 3091.6  on 2319  degrees of freedom
## AIC: 3097.6
## 
## Number of Fisher Scoring iterations: 4
```

```
# test model fit
with(m, null.deviance - deviance)
```

```
## [1] 2.568457
```

```
with(m, df.null - df.residual)
```

```
## [1] 2
```

```
with(m, pchisq(null.deviance - deviance, df.null - df.residual, lower.tail = FALSE))
```

```
## [1] 0.2768641
```

```
BIC(m)
```

```
## [1] 3114.807
```

```
# Hosmer-Lemeshow Goodness-of-Fit Test
glmtoolbox::hltest(m)
```

```
## 
##    The Hosmer-Lemeshow goodness-of-fit test
## 
##  Group Size Observed Expected
##      1  347      125      125
##      2 1611      616      616
##      3  364      152      152
## 
##          Statistic =  0 
## degrees of freedom =  1 
##            p-value =  1
```

```
## CIs using profiled log-likelihood
confint(m, level=0.99)
```

```
## Waiting for profiling to be done...
```

```
##                    0.5 %      99.5 %
## (Intercept)   -0.6089695 -0.06057305
## Income2Medium -0.4495211  0.15903870
## Income2High   -0.6404806  0.15504888
```

```
## CIs using standard errors
confint.default(m, level=0.99)
```

```
##                    0.5 %      99.5 %
## (Intercept)   -0.6064708 -0.05894068
## Income2Medium -0.4507415  0.15716144
## Income2High   -0.6390411  0.15572530
```

```
# Wald test
wald.test(b = coef(m), Sigma = vcov(m), Terms = 2)
```

```
## Wald test:
## ----------
## 
## Chi-squared test:
## X2 = 1.5, df = 1, P(> X2) = 0.21
```

```
## odds ratios and 95% CI
exp(cbind(OR = coef(m), confint(m, level=0.99)))
```

```
## Waiting for profiling to be done...
```

```
##                      OR     0.5 %   99.5 %
## (Intercept)   0.7169811 0.5439111 0.941225
## Income2Medium 0.8634753 0.6379336 1.172383
## Income2High   0.7853248 0.5270391 1.167715
```

### Create ROCR from data

```
pred.mtt = predict(m, type = "response") #repeat risk predictions from model m
rocr.pred.mtt = ROCR::prediction(pred.mtt, labels = ml$Any_Health_Problem) #ROCR prediction object
roc.perf.mtt = ROCR::performance(rocr.pred.mtt, measure = "tpr", x.measure = "fpr") # #ROCR performance object
plot(roc.perf.mtt, col = "blue")
abline(a = 0, b = 1, lty = 2) #diagonal for random assignment
```

### Report AUC from ROC for training and test data

```
  # Train AUC
auc <- ROCR::performance(rocr.pred.mtt, measure = "auc")
  auc <- auc@y.values[[1]]
  print(auc)
```

```
## [1] 0.5157782
```

### Calculate Nagelkerke R^2

```
NagelkerkeR2(m)
```

```
## $N
## [1] 2322
## 
## $R2
## [1] 0.001501689
```

### check assumptions of model

#### Cook’s distance

```
plot(m, which = 4, id.n = 3)
```

#### Extract model results and display data for top 3 values using Cook’s distance

```
model.data <- augment(m) %>% 
  mutate(index = 1:n()) 
model.data %>% top_n(3, .cooksd)
```

#### plot standardised residuals

```
ggplot(model.data, aes(index, .std.resid)) + 
  geom_point(aes(color = Any_Health_Problem), alpha = .5) +
  theme_bw()
```

#### Filter potential influential data points with abs(.std.res) > 3:

```
model.data %>% 
  filter(abs(.std.resid) > 3)
```

## C\_AGE Binary logistic regression for HEALTH

```
# fit binary logit model and store results 'm'
m <- glm(Any_Health_Problem ~ C_Age, data = ml,family = binomial)
# view a summary of the model
summary(m)
```

```
## 
## Call:
## glm(formula = Any_Health_Problem ~ C_Age, family = binomial, 
##     data = ml)
## 
## Coefficients:
##             Estimate Std. Error z value Pr(>|z|)    
## (Intercept) -0.46509    0.04299 -10.819  < 2e-16 ***
## C_Age.L     -0.28389    0.09977  -2.845  0.00444 ** 
## C_Age.Q      0.05284    0.09825   0.538  0.59072    
## C_Age.C      0.11163    0.09324   1.197  0.23123    
## C_Age^4      0.04113    0.09304   0.442  0.65844    
## ---
## Signif. codes:  0 '***' 0.001 '**' 0.01 '*' 0.05 '.' 0.1 ' ' 1
## 
## (Dispersion parameter for binomial family taken to be 1)
## 
##     Null deviance: 3094.1  on 2321  degrees of freedom
## Residual deviance: 3083.5  on 2317  degrees of freedom
## AIC: 3093.5
## 
## Number of Fisher Scoring iterations: 4
```

```
# test model fit
with(m, null.deviance - deviance)
```

```
## [1] 10.6717
```

```
with(m, df.null - df.residual)
```

```
## [1] 4
```

```
with(m, pchisq(null.deviance - deviance, df.null - df.residual, lower.tail = FALSE))
```

```
## [1] 0.03051233
```

```
BIC(m)
```

```
## [1] 3122.204
```

```
# Hosmer-Lemeshow Goodness-of-Fit Test
hltest(m, G=4)
```

```
## 
##    The Hosmer-Lemeshow goodness-of-fit test
## 
##  Group Size Observed Expected
##      1  528      180      180
##      2  428      154      154
##      3  474      183      183
##      4  507      211      211
##      5  385      165      165
## 
##          Statistic =  0 
## degrees of freedom =  3 
##            p-value =  1
```

```
## CIs using profiled log-likelihood
confint(m, level=0.99)
```

```
## Waiting for profiling to be done...
```

```
##                  0.5 %      99.5 %
## (Intercept) -0.5763089 -0.35479443
## C_Age.L     -0.5414334 -0.02714238
## C_Age.Q     -0.2007430  0.30562593
## C_Age.C     -0.1283278  0.35222470
## C_Age^4     -0.1990419  0.28051323
```

```
## CIs using standard errors
confint.default(m, level=0.99)
```

```
##                  0.5 %      99.5 %
## (Intercept) -0.5758154 -0.35436129
## C_Age.L     -0.5408820 -0.02689302
## C_Age.Q     -0.2002402  0.30591849
## C_Age.C     -0.1285428  0.35179658
## C_Age^4     -0.1985225  0.28077997
```

```
# Wald test
wald.test(b = coef(m), Sigma = vcov(m), Terms = 2)
```

```
## Wald test:
## ----------
## 
## Chi-squared test:
## X2 = 8.1, df = 1, P(> X2) = 0.0044
```

```
## odds ratios and 95% CI
exp(cbind(OR = coef(m), confint(m, level=0.99)))
```

```
## Waiting for profiling to be done...
```

```
##                    OR     0.5 %    99.5 %
## (Intercept) 0.6280796 0.5619688 0.7013176
## C_Age.L     0.7528513 0.5819136 0.9732227
## C_Age.Q     1.0542601 0.8181226 1.3574744
## C_Age.C     1.1180956 0.8795650 1.4222281
## C_Age^4     1.0419862 0.8195155 1.3238091
```

### Create ROCR from data

```
pred.mtt = predict(m, type = "response") #repeat risk predictions from model m
rocr.pred.mtt = ROCR::prediction(pred.mtt, labels = ml$Any_Health_Problem) #ROCR prediction object
roc.perf.mtt = ROCR::performance(rocr.pred.mtt, measure = "tpr", x.measure = "fpr") # #ROCR performance object
plot(roc.perf.mtt, col = "blue")
abline(a = 0, b = 1, lty = 2) #diagonal for random assignment
```

### Report AUC from ROC for training and test data

```
  # Train AUC
auc <- ROCR::performance(rocr.pred.mtt, measure = "auc")
  auc <- auc@y.values[[1]]
  print(auc)
```

```
## [1] 0.5391193
```

### Calculate Nagelkerke R^2

```
NagelkerkeR2(m)
```

```
## $N
## [1] 2322
## 
## $R2
## [1] 0.006228504
```

### check assumptions of model

#### Cook’s distance

```
plot(m, which = 4, id.n = 3)
```

#### Extract model results and display data for top 3 values using Cook’s distance

```
model.data <- augment(m) %>% 
  mutate(index = 1:n()) 
model.data %>% top_n(3, .cooksd)
```

#### plot standardised residuals

```
ggplot(model.data, aes(index, .std.resid)) + 
  geom_point(aes(color = Any_Health_Problem), alpha = .5) +
  theme_bw()
```

#### Filter potential influential data points with abs(.std.res) > 3:

```
model.data %>% 
  filter(abs(.std.resid) > 3)
```

## C\_AGE Binary logistic regression for HEALTH

```
# fit binary logit model and store results 'm'
m <- glm(Any_Health_Problem ~ C_Age2, data = ml,family = binomial)
# view a summary of the model
summary(m)
```

```
## 
## Call:
## glm(formula = Any_Health_Problem ~ C_Age2, family = binomial, 
##     data = ml)
## 
## Coefficients:
##             Estimate Std. Error z value Pr(>|z|)   
## (Intercept) -0.28768    0.10299  -2.793  0.00522 **
## C_Age230–39 -0.05082    0.13683  -0.371  0.71035   
## C_Age240–49 -0.17616    0.13967  -1.261  0.20722   
## C_Age250–59 -0.37156    0.13797  -2.693  0.00708 **
## C_Age260<   -0.28849    0.14405  -2.003  0.04520 * 
## ---
## Signif. codes:  0 '***' 0.001 '**' 0.01 '*' 0.05 '.' 0.1 ' ' 1
## 
## (Dispersion parameter for binomial family taken to be 1)
## 
##     Null deviance: 3094.1  on 2321  degrees of freedom
## Residual deviance: 3083.5  on 2317  degrees of freedom
## AIC: 3093.5
## 
## Number of Fisher Scoring iterations: 4
```

```
# test model fit
with(m, null.deviance - deviance)
```

```
## [1] 10.6717
```

```
with(m, df.null - df.residual)
```

```
## [1] 4
```

```
with(m, pchisq(null.deviance - deviance, df.null - df.residual, lower.tail = FALSE))
```

```
## [1] 0.03051233
```

```
BIC(m)
```

```
## [1] 3122.204
```

```
# Hosmer-Lemeshow Goodness-of-Fit Test
hltest(m, G=4)
```

```
## 
##    The Hosmer-Lemeshow goodness-of-fit test
## 
##  Group Size Observed Expected
##      1  528      180      180
##      2  428      154      154
##      3  474      183      183
##      4  507      211      211
##      5  385      165      165
## 
##          Statistic =  0 
## degrees of freedom =  3 
##            p-value =  1
```

```
## CIs using profiled log-likelihood
confint(m, level=0.99)
```

```
## Waiting for profiling to be done...
```

```
##                  0.5 %      99.5 %
## (Intercept) -0.5550296 -0.02369581
## C_Age230–39 -0.4032406  0.30220040
## C_Age240–49 -0.5362985  0.18376642
## C_Age250–59 -0.7276020 -0.01628170
## C_Age260<   -0.6605163  0.08216549
```

```
## CIs using standard errors
confint.default(m, level=0.99)
```

```
##                  0.5 %      99.5 %
## (Intercept) -0.5529557 -0.02240841
## C_Age230–39 -0.4032825  0.30164396
## C_Age240–49 -0.5359143  0.18360420
## C_Age250–59 -0.7269456 -0.01618146
## C_Age260<   -0.6595309  0.08254404
```

```
# Wald test
wald.test(b = coef(m), Sigma = vcov(m), Terms = 2)
```

```
## Wald test:
## ----------
## 
## Chi-squared test:
## X2 = 0.14, df = 1, P(> X2) = 0.71
```

```
## odds ratios and 95% CI
exp(cbind(OR = coef(m), confint(m, level=0.99)))
```

```
## Waiting for profiling to be done...
```

```
##                    OR     0.5 %    99.5 %
## (Intercept) 0.7500000 0.5740553 0.9765827
## C_Age230–39 0.9504505 0.6681513 1.3528323
## C_Age240–49 0.8384880 0.5849093 1.2017351
## C_Age250–59 0.6896552 0.4830660 0.9838501
## C_Age260<   0.7493917 0.5165846 1.0856355
```

### Create ROCR from data

```
pred.mtt = predict(m, type = "response") #repeat risk predictions from model m
rocr.pred.mtt = ROCR::prediction(pred.mtt, labels = ml$Any_Health_Problem) #ROCR prediction object
roc.perf.mtt = ROCR::performance(rocr.pred.mtt, measure = "tpr", x.measure = "fpr") # #ROCR performance object
plot(roc.perf.mtt, col = "blue")
abline(a = 0, b = 1, lty = 2) #diagonal for random assignment
```

### Report AUC from ROC for training and test data

```
  # Train AUC
auc <- ROCR::performance(rocr.pred.mtt, measure = "auc")
  auc <- auc@y.values[[1]]
  print(auc)
```

```
## [1] 0.5391193
```

### Calculate Nagelkerke R^2

```
NagelkerkeR2(m)
```

```
## $N
## [1] 2322
## 
## $R2
## [1] 0.006228504
```

### check assumptions of model

#### Cook’s distance

```
plot(m, which = 4, id.n = 3)
```

#### Extract model results and display data for top 3 values using Cook’s distance

```
model.data <- augment(m) %>% 
  mutate(index = 1:n()) 
model.data %>% top_n(3, .cooksd)
```

#### plot standardised residuals

```
ggplot(model.data, aes(index, .std.resid)) + 
  geom_point(aes(color = Any_Health_Problem), alpha = .5) +
  theme_bw()
```

#### Filter potential influential data points with abs(.std.res) > 3:

```
model.data %>% 
  filter(abs(.std.resid) > 3)
```

## C\_GENDER Binary logistic regression for HEALTH

```
# fit binary logit model and store results 'm'
m <- glm(Any_Health_Problem ~ C_Gender, data = ml,family = binomial)
# view a summary of the model
summary(m)
```

```
## 
## Call:
## glm(formula = Any_Health_Problem ~ C_Gender, family = binomial, 
##     data = ml)
## 
## Coefficients:
##              Estimate Std. Error z value Pr(>|z|)    
## (Intercept)  -0.44132    0.04423  -9.978   <2e-16 ***
## C_GenderMale -0.40057    0.17016  -2.354   0.0186 *  
## ---
## Signif. codes:  0 '***' 0.001 '**' 0.01 '*' 0.05 '.' 0.1 ' ' 1
## 
## (Dispersion parameter for binomial family taken to be 1)
## 
##     Null deviance: 3094.1  on 2321  degrees of freedom
## Residual deviance: 3088.3  on 2320  degrees of freedom
## AIC: 3092.3
## 
## Number of Fisher Scoring iterations: 4
```

```
# test model fit
with(m, null.deviance - deviance)
```

```
## [1] 5.776689
```

```
with(m, df.null - df.residual)
```

```
## [1] 1
```

```
with(m, pchisq(null.deviance - deviance, df.null - df.residual, lower.tail = FALSE))
```

```
## [1] 0.01624011
```

```
BIC(m)
```

```
## [1] 3103.848
```

```
## CIs using profiled log-likelihood
confint(m, level=0.99)
```

```
## Waiting for profiling to be done...
```

```
##                   0.5 %      99.5 %
## (Intercept)  -0.5557498 -0.32783607
## C_GenderMale -0.8515255  0.02816629
```

```
## CIs using standard errors
confint.default(m, level=0.99)
```

```
##                   0.5 %      99.5 %
## (Intercept)  -0.5552479 -0.32739698
## C_GenderMale -0.8388711  0.03773103
```

```
# Wald test
wald.test(b = coef(m), Sigma = vcov(m), Terms = 2)
```

```
## Wald test:
## ----------
## 
## Chi-squared test:
## X2 = 5.5, df = 1, P(> X2) = 0.019
```

```
## odds ratios and 95% CI
exp(cbind(OR = coef(m), confint(m, level=0.99)))
```

```
## Waiting for profiling to be done...
```

```
##                     OR     0.5 %    99.5 %
## (Intercept)  0.6431853 0.5736420 0.7204811
## C_GenderMale 0.6699381 0.4267634 1.0285667
```

### Create ROCR from data

```
pred.mtt = predict(m, type = "response") #repeat risk predictions from model m
rocr.pred.mtt = ROCR::prediction(pred.mtt, labels = ml$Any_Health_Problem) #ROCR prediction object
roc.perf.mtt = ROCR::performance(rocr.pred.mtt, measure = "tpr", x.measure = "fpr") # #ROCR performance object
plot(roc.perf.mtt, col = "blue")
abline(a = 0, b = 1, lty = 2) #diagonal for random assignment
```

### Report AUC from ROC for training and test data

```
  # Train AUC
auc <- ROCR::performance(rocr.pred.mtt, measure = "auc")
  auc <- auc@y.values[[1]]
  print(auc)
```

```
## [1] 0.5133618
```

### Calculate Nagelkerke R^2

```
NagelkerkeR2(m)
```

```
## $N
## [1] 2322
## 
## $R2
## [1] 0.003375101
```

### check assumptions of model

#### Cook’s distance

```
plot(m, which = 4, id.n = 3)
```

#### Extract model results and display data for top 3 values using Cook’s distance

```
model.data <- augment(m) %>% 
  mutate(index = 1:n()) 
model.data %>% top_n(3, .cooksd)
```

#### plot standardised residuals

```
ggplot(model.data, aes(index, .std.resid)) + 
  geom_point(aes(color = Any_Health_Problem), alpha = .5) +
  theme_bw()
```

#### Filter potential influential data points with abs(.std.res) > 3:

```
model.data %>% 
  filter(abs(.std.resid) > 3)
```

# NOW CHECK ASSOCIATIONS WITH DOG CHARACTERISTICS - simple BINARY LOGISTIC regression

## DOG DIET ordinal regression for HEALTH

```
# fit binary logit model and store results 'm'
m <- glm(Any_Health_Problem ~ D_Diet, data = ml,family = binomial)
# view a summary of the model
summary(m)
```

```
## 
## Call:
## glm(formula = Any_Health_Problem ~ D_Diet, family = binomial, 
##     data = ml)
## 
## Coefficients:
##                                                         Estimate Std. Error
## (Intercept)                                             -0.22205    0.05738
## D_DietMeat-based – raw                                  -0.54460    0.09746
## D_DietVegan (consuming no animal products)              -0.62373    0.13600
## D_DietVegetarian (including eggs or milk, but not meat)  0.05020    0.34413
##                                                         z value Pr(>|z|)    
## (Intercept)                                              -3.870 0.000109 ***
## D_DietMeat-based – raw                                   -5.588 2.30e-08 ***
## D_DietVegan (consuming no animal products)               -4.586 4.51e-06 ***
## D_DietVegetarian (including eggs or milk, but not meat)   0.146 0.884029    
## ---
## Signif. codes:  0 '***' 0.001 '**' 0.01 '*' 0.05 '.' 0.1 ' ' 1
## 
## (Dispersion parameter for binomial family taken to be 1)
## 
##     Null deviance: 3094.1  on 2321  degrees of freedom
## Residual deviance: 3050.5  on 2318  degrees of freedom
## AIC: 3058.5
## 
## Number of Fisher Scoring iterations: 4
```

```
# test model fit
with(m, null.deviance - deviance)
```

```
## [1] 43.59839
```

```
with(m, df.null - df.residual)
```

```
## [1] 3
```

```
with(m, pchisq(null.deviance - deviance, df.null - df.residual, lower.tail = FALSE))
```

```
## [1] 1.836725e-09
```

```
BIC(m)
```

```
## [1] 3081.527
```

```
## CIs using profiled log-likelihood
confint(m, level=0.99)
```

```
## Waiting for profiling to be done...
```

```
##                                                              0.5 %      99.5 %
## (Intercept)                                             -0.3703142 -0.07458454
## D_DietMeat-based – raw                                  -0.7972689 -0.29494693
## D_DietVegan (consuming no animal products)              -0.9801000 -0.27831262
## D_DietVegetarian (including eggs or milk, but not meat) -0.8605297  0.93928702
```

```
## CIs using standard errors
confint.default(m, level=0.99)
```

```
##                                                              0.5 %      99.5 %
## (Intercept)                                             -0.3698431 -0.07424906
## D_DietMeat-based – raw                                  -0.7956389 -0.29356830
## D_DietVegan (consuming no animal products)              -0.9740510 -0.27341076
## D_DietVegetarian (including eggs or milk, but not meat) -0.8362184  0.93660997
```

```
# Wald test
wald.test(b = coef(m), Sigma = vcov(m), Terms = 2)
```

```
## Wald test:
## ----------
## 
## Chi-squared test:
## X2 = 31.2, df = 1, P(> X2) = 2.3e-08
```

```
## odds ratios and 95% CI
exp(cbind(OR = coef(m), confint(m, level=0.99)))
```

```
## Waiting for profiling to be done...
```

```
##                                                                OR     0.5 %
## (Intercept)                                             0.8008785 0.6905174
## D_DietMeat-based – raw                                  0.5800717 0.4505578
## D_DietVegan (consuming no animal products)              0.5359412 0.3752736
## D_DietVegetarian (including eggs or milk, but not meat) 1.0514770 0.4229380
##                                                            99.5 %
## (Intercept)                                             0.9281290
## D_DietMeat-based – raw                                  0.7445711
## D_DietVegan (consuming no animal products)              0.7570601
## D_DietVegetarian (including eggs or milk, but not meat) 2.5581569
```

### Create ROCR from data

```
pred.mtt = predict(m, type = "response") #repeat risk predictions from model m
rocr.pred.mtt = ROCR::prediction(pred.mtt, labels = ml$Any_Health_Problem) #ROCR prediction object
roc.perf.mtt = ROCR::performance(rocr.pred.mtt, measure = "tpr", x.measure = "fpr") # #ROCR performance object
plot(roc.perf.mtt, col = "blue")
abline(a = 0, b = 1, lty = 2) #diagonal for random assignment
```

### Report AUC from ROC for training and test data

```
  # Train AUC
auc <- ROCR::performance(rocr.pred.mtt, measure = "auc")
  auc <- auc@y.values[[1]]
  print(auc)
```

```
## [1] 0.5713535
```

### Calculate Nagelkerke R^2

```
NagelkerkeR2(m)
```

```
## $N
## [1] 2322
## 
## $R2
## [1] 0.02526664
```

### check assumptions of model

#### Cook’s distance

```
plot(m, which = 4, id.n = 3)
```

#### Extract model results and display data for top 3 values using Cook’s distance

```
model.data <- augment(m) %>% 
  mutate(index = 1:n()) 
model.data %>% top_n(3, .cooksd)
```

#### plot standardised residuals

```
ggplot(model.data, aes(index, .std.resid)) + 
  geom_point(aes(color = Any_Health_Problem), alpha = .5) +
  theme_bw()
```

#### Filter potential influential data points with abs(.std.res) > 3:

```
model.data %>% 
  filter(abs(.std.resid) > 3)
```

## DOG DIET VEGAN ordinal regression for HEALTH

```
# fit binary logit model and store results 'm'
m <- glm(Any_Health_Problem ~ D_Diet_Vegan, data = ml,family = binomial)
# view a summary of the model
summary(m)
```

```
## 
## Call:
## glm(formula = Any_Health_Problem ~ D_Diet_Vegan, family = binomial, 
##     data = ml)
## 
## Coefficients:
##                 Estimate Std. Error z value Pr(>|z|)    
## (Intercept)     -0.41501    0.04559  -9.104  < 2e-16 ***
## D_Diet_VeganYes -0.43076    0.13146  -3.277  0.00105 ** 
## ---
## Signif. codes:  0 '***' 0.001 '**' 0.01 '*' 0.05 '.' 0.1 ' ' 1
## 
## (Dispersion parameter for binomial family taken to be 1)
## 
##     Null deviance: 3094.1  on 2321  degrees of freedom
## Residual deviance: 3083.0  on 2320  degrees of freedom
## AIC: 3087
## 
## Number of Fisher Scoring iterations: 4
```

```
# test model fit
with(m, null.deviance - deviance)
```

```
## [1] 11.16772
```

```
with(m, df.null - df.residual)
```

```
## [1] 1
```

```
with(m, pchisq(null.deviance - deviance, df.null - df.residual, lower.tail = FALSE))
```

```
## [1] 0.0008323277
```

```
BIC(m)
```

```
## [1] 3098.457
```

```
## CIs using profiled log-likelihood
confint(m, level=0.99)
```

```
## Waiting for profiling to be done...
```

```
##                      0.5 %      99.5 %
## (Intercept)     -0.5329396 -0.29803154
## D_Diet_VeganYes -0.7758822 -0.09739852
```

```
## CIs using standard errors
confint.default(m, level=0.99)
```

```
##                      0.5 %      99.5 %
## (Intercept)     -0.5324344 -0.29759499
## D_Diet_VeganYes -0.7693883 -0.09213617
```

```
# Wald test
wald.test(b = coef(m), Sigma = vcov(m), Terms = 2)
```

```
## Wald test:
## ----------
## 
## Chi-squared test:
## X2 = 10.7, df = 1, P(> X2) = 0.0011
```

```
## odds ratios and 95% CI
exp(cbind(OR = coef(m), confint(m, level=0.99)))
```

```
## Waiting for profiling to be done...
```

```
##                        OR     0.5 %    99.5 %
## (Intercept)     0.6603306 0.5868772 0.7422779
## D_Diet_VeganYes 0.6500134 0.4602975 0.9071944
```

### Create ROCR from data

```
pred.mtt = predict(m, type = "response") #repeat risk predictions from model m
rocr.pred.mtt = ROCR::prediction(pred.mtt, labels = ml$Any_Health_Problem) #ROCR prediction object
roc.perf.mtt = ROCR::performance(rocr.pred.mtt, measure = "tpr", x.measure = "fpr") # #ROCR performance object
plot(roc.perf.mtt, col = "blue")
abline(a = 0, b = 1, lty = 2) #diagonal for random assignment
```

### Report AUC from ROC for training and test data

```
  # Train AUC
auc <- ROCR::performance(rocr.pred.mtt, measure = "auc")
  auc <- auc@y.values[[1]]
  print(auc)
```

```
## [1] 0.5239954
```

### Calculate Nagelkerke R^2

```
NagelkerkeR2(m)
```

```
## $N
## [1] 2322
## 
## $R2
## [1] 0.006517313
```

### check assumptions of model

#### Cook’s distance

```
plot(m, which = 4, id.n = 3)
```

#### Extract model results and display data for top 3 values using Cook’s distance

```
model.data <- augment(m) %>% 
  mutate(index = 1:n()) 
model.data %>% top_n(3, .cooksd)
```

#### plot standardised residuals

```
ggplot(model.data, aes(index, .std.resid)) + 
  geom_point(aes(color = Any_Health_Problem), alpha = .5) +
  theme_bw()
```

#### Filter potential influential data points with abs(.std.res) > 3:

```
model.data %>% 
  filter(abs(.std.resid) > 3)
```

## CLIENT DIET VEGAN + DOG DIET VEGAN ordinal regression for HEALTH

```
# fit binary logit model and store results 'm'
m <- glm(Any_Health_Problem ~ C_Diet_Vegan + D_Diet_Vegan, data = ml,family = binomial)
# view a summary of the model
summary(m)
```

```
## 
## Call:
## glm(formula = Any_Health_Problem ~ C_Diet_Vegan + D_Diet_Vegan, 
##     family = binomial, data = ml)
## 
## Coefficients:
##                 Estimate Std. Error z value Pr(>|z|)    
## (Intercept)     -0.44436    0.04855  -9.153  < 2e-16 ***
## C_Diet_VeganYes  0.23722    0.13223   1.794  0.07282 .  
## D_Diet_VeganYes -0.62055    0.16894  -3.673  0.00024 ***
## ---
## Signif. codes:  0 '***' 0.001 '**' 0.01 '*' 0.05 '.' 0.1 ' ' 1
## 
## (Dispersion parameter for binomial family taken to be 1)
## 
##     Null deviance: 3094.1  on 2321  degrees of freedom
## Residual deviance: 3079.8  on 2319  degrees of freedom
## AIC: 3085.8
## 
## Number of Fisher Scoring iterations: 4
```

```
# test model fit
with(m, null.deviance - deviance)
```

```
## [1] 14.37171
```

```
with(m, df.null - df.residual)
```

```
## [1] 2
```

```
with(m, pchisq(null.deviance - deviance, df.null - df.residual, lower.tail = FALSE))
```

```
## [1] 0.0007572202
```

```
BIC(m)
```

```
## [1] 3103.004
```

```
## CIs using profiled log-likelihood
confint(m, level=0.99)
```

```
## Waiting for profiling to be done...
```

```
##                      0.5 %     99.5 %
## (Intercept)     -0.5700256 -0.3198400
## C_Diet_VeganYes -0.1046437  0.5776232
## D_Diet_VeganYes -1.0602445 -0.1887643
```

```
## CIs using standard errors
confint.default(m, level=0.99)
```

```
##                      0.5 %     99.5 %
## (Intercept)     -0.5694099 -0.3193071
## C_Diet_VeganYes -0.1033898  0.5778230
## D_Diet_VeganYes -1.0557080 -0.1853834
```

```
# Wald test
wald.test(b = coef(m), Sigma = vcov(m), Terms = 2)
```

```
## Wald test:
## ----------
## 
## Chi-squared test:
## X2 = 3.2, df = 1, P(> X2) = 0.073
```

```
## odds ratios and 95% CI
exp(cbind(OR = coef(m), confint(m, level=0.99)))
```

```
## Waiting for profiling to be done...
```

```
##                        OR     0.5 %    99.5 %
## (Intercept)     0.6412355 0.5655109 0.7262652
## C_Diet_VeganYes 1.2677157 0.9006454 1.7817984
## D_Diet_VeganYes 0.5376510 0.3463711 0.8279817
```

### Create ROCR from data

```
pred.mtt = predict(m, type = "response") #repeat risk predictions from model m
rocr.pred.mtt = ROCR::prediction(pred.mtt, labels = ml$Any_Health_Problem) #ROCR prediction object
roc.perf.mtt = ROCR::performance(rocr.pred.mtt, measure = "tpr", x.measure = "fpr") # #ROCR performance object
plot(roc.perf.mtt, col = "blue")
abline(a = 0, b = 1, lty = 2) #diagonal for random assignment
```

### Report AUC from ROC for training and test data

```
  # Train AUC
auc <- ROCR::performance(rocr.pred.mtt, measure = "auc")
  auc <- auc@y.values[[1]]
  print(auc)
```

```
## [1] 0.5362519
```

### Calculate Nagelkerke R^2

```
NagelkerkeR2(m)
```

```
## $N
## [1] 2322
## 
## $R2
## [1] 0.008381334
```

### check assumptions of model

#### Cook’s distance

```
plot(m, which = 4, id.n = 3)
```

#### Extract model results and display data for top 3 values using Cook’s distance

```
model.data <- augment(m) %>% 
  mutate(index = 1:n()) 
model.data %>% top_n(3, .cooksd)
```

#### plot standardised residuals

```
ggplot(model.data, aes(index, .std.resid)) + 
  geom_point(aes(color = Any_Health_Problem), alpha = .5) +
  theme_bw()
```

#### Filter potential influential data points with abs(.std.res) > 3:

```
model.data %>% 
  filter(abs(.std.resid) > 3)
```

#### check for multicollinearity

```
car::vif(m)
```

```
## C_Diet_Vegan D_Diet_Vegan 
##     1.649982     1.649982
```

## CLIENT DIET VEGAN \* DOG DIET VEGAN ordinal regression for HEALTH

```
# fit binary logit model and store results 'm2'
m <- glm(Any_Health_Problem ~ C_Diet_Vegan*D_Diet_Vegan, data = ml,family = binomial)
# view a summary of the model
summary(m)
```

```
## 
## Call:
## glm(formula = Any_Health_Problem ~ C_Diet_Vegan * D_Diet_Vegan, 
##     family = binomial, data = ml)
## 
## Coefficients:
##                                 Estimate Std. Error z value Pr(>|z|)    
## (Intercept)                     -0.45291    0.04883  -9.275   <2e-16 ***
## C_Diet_VeganYes                  0.30510    0.13736   2.221   0.0263 *  
## D_Diet_VeganYes                  0.04745    0.41116   0.115   0.9081    
## C_Diet_VeganYes:D_Diet_VeganYes -0.78694    0.44982  -1.749   0.0802 .  
## ---
## Signif. codes:  0 '***' 0.001 '**' 0.01 '*' 0.05 '.' 0.1 ' ' 1
## 
## (Dispersion parameter for binomial family taken to be 1)
## 
##     Null deviance: 3094.1  on 2321  degrees of freedom
## Residual deviance: 3076.8  on 2318  degrees of freedom
## AIC: 3084.8
## 
## Number of Fisher Scoring iterations: 4
```

```
# test model fit
with(m, null.deviance - deviance)
```

```
## [1] 17.28716
```

```
with(m, df.null - df.residual)
```

```
## [1] 3
```

```
with(m, pchisq(null.deviance - deviance, df.null - df.residual, lower.tail = FALSE))
```

```
## [1] 0.0006168489
```

```
BIC(m)
```

```
## [1] 3107.838
```

```
# Hosmer-Lemeshow Goodness-of-Fit Test
glmtoolbox::hltest(m)
```

```
## 
##    The Hosmer-Lemeshow goodness-of-fit test
## 
##  Group Size Observed Expected
##      1  288       84       84
##      2 1765      686      686
##      3   25       10       10
##      4  244      113      113
## 
##          Statistic =  0 
## degrees of freedom =  2 
##            p-value =  1
```

```
## CIs using profiled log-likelihood
confint(m, level=0.99)
```

```
## Waiting for profiling to be done...
```

```
##                                       0.5 %     99.5 %
## (Intercept)                     -0.57932426 -0.3276767
## C_Diet_VeganYes                 -0.05044338  0.6584344
## D_Diet_VeganYes                 -1.07334936  1.0934453
## C_Diet_VeganYes:D_Diet_VeganYes -1.93365082  0.4220574
```

```
## CIs using standard errors
confint.default(m, level=0.99)
```

```
##                                       0.5 %     99.5 %
## (Intercept)                     -0.57869380 -0.3271309
## C_Diet_VeganYes                 -0.04871201  0.6589177
## D_Diet_VeganYes                 -1.01162645  1.1065209
## C_Diet_VeganYes:D_Diet_VeganYes -1.94560902  0.3717272
```

```
# Wald test
wald.test(b = coef(m), Sigma = vcov(m), Terms = 2)
```

```
## Wald test:
## ----------
## 
## Chi-squared test:
## X2 = 4.9, df = 1, P(> X2) = 0.026
```

```
## odds ratios and 95% CI
exp(cbind(OR = coef(m), confint(m, level=0.99)))
```

```
## Waiting for profiling to be done...
```

```
##                                        OR     0.5 %   99.5 %
## (Intercept)                     0.6357739 0.5602768 0.720596
## C_Diet_VeganYes                 1.3567645 0.9508078 1.931766
## D_Diet_VeganYes                 1.0485909 0.3418616 2.984539
## C_Diet_VeganYes:D_Diet_VeganYes 0.4552353 0.1446193 1.525096
```

### Create ROCR from data

```
pred.mtt = predict(m, type = "response") #repeat risk predictions from model m
rocr.pred.mtt = ROCR::prediction(pred.mtt, labels = ml$Any_Health_Problem) #ROCR prediction object
roc.perf.mtt = ROCR::performance(rocr.pred.mtt, measure = "tpr", x.measure = "fpr") # #ROCR performance object
plot(roc.perf.mtt, col = "blue")
abline(a = 0, b = 1, lty = 2) #diagonal for random assignment
```

### Report AUC from ROC for training and test data

```
  # Train AUC
auc <- ROCR::performance(rocr.pred.mtt, measure = "auc")
  auc <- auc@y.values[[1]]
  print(auc)
```

```
## [1] 0.537255
```

### Calculate Nagelkerke R^2

```
NagelkerkeR2(m)
```

```
## $N
## [1] 2322
## 
## $R2
## [1] 0.01007525
```

### check assumptions of model

#### Cook’s distance

```
plot(m, which = 4, id.n = 3)
```

#### Extract model results and display data for top 3 values using Cook’s distance

```
model.data <- augment(m) %>% 
  mutate(index = 1:n()) 
model.data %>% top_n(3, .cooksd)
```

#### plot standardised residuals

```
ggplot(model.data, aes(index, .std.resid)) + 
  geom_point(aes(color = Any_Health_Problem), alpha = .5) +
  theme_bw()
```

#### Filter potential influential data points with abs(.std.res) > 3:

```
model.data %>% 
  filter(abs(.std.resid) > 3)
```

#### check for multicollinearity

```
car::vif(m)
```

```
## there are higher-order terms (interactions) in this model
## consider setting type = 'predictor'; see ?vif
```

```
##              C_Diet_Vegan              D_Diet_Vegan C_Diet_Vegan:D_Diet_Vegan 
##                  1.767867                  9.743408                 10.726225
```

## DOG DIET RAW ordinal regression for HEALTH

```
# fit binary logit model and store results 'm'
m <- glm(Any_Health_Problem ~ D_Diet_Raw, data = ml,family = binomial)
# view a summary of the model
summary(m)
```

```
## 
## Call:
## glm(formula = Any_Health_Problem ~ D_Diet_Raw, family = binomial, 
##     data = ml)
## 
## Coefficients:
##               Estimate Std. Error z value Pr(>|z|)    
## (Intercept)   -0.33778    0.05107  -6.614 3.73e-11 ***
## D_Diet_RawYes -0.42887    0.09388  -4.568 4.92e-06 ***
## ---
## Signif. codes:  0 '***' 0.001 '**' 0.01 '*' 0.05 '.' 0.1 ' ' 1
## 
## (Dispersion parameter for binomial family taken to be 1)
## 
##     Null deviance: 3094.1  on 2321  degrees of freedom
## Residual deviance: 3072.8  on 2320  degrees of freedom
## AIC: 3076.8
## 
## Number of Fisher Scoring iterations: 4
```

```
# test model fit
with(m, null.deviance - deviance)
```

```
## [1] 21.30865
```

```
with(m, df.null - df.residual)
```

```
## [1] 1
```

```
with(m, pchisq(null.deviance - deviance, df.null - df.residual, lower.tail = FALSE))
```

```
## [1] 3.90964e-06
```

```
BIC(m)
```

```
## [1] 3088.316
```

```
## CIs using profiled log-likelihood
confint(m, level=0.99)
```

```
## Waiting for profiling to be done...
```

```
##                    0.5 %     99.5 %
## (Intercept)   -0.4698475 -0.2066712
## D_Diet_RawYes -0.6724551 -0.1885501
```

```
## CIs using standard errors
confint.default(m, level=0.99)
```

```
##                    0.5 %     99.5 %
## (Intercept)   -0.4693161 -0.2062359
## D_Diet_RawYes -0.6706950 -0.1870523
```

```
# Wald test
wald.test(b = coef(m), Sigma = vcov(m), Terms = 2)
```

```
## Wald test:
## ----------
## 
## Chi-squared test:
## X2 = 20.9, df = 1, P(> X2) = 4.9e-06
```

```
## odds ratios and 95% CI
exp(cbind(OR = coef(m), confint(m, level=0.99)))
```

```
## Waiting for profiling to be done...
```

```
##                      OR     0.5 %   99.5 %
## (Intercept)   0.7133550 0.6250976 0.813287
## D_Diet_RawYes 0.6512422 0.5104538 0.828159
```

### Create ROCR from data

```
pred.mtt = predict(m, type = "response") #repeat risk predictions from model m
rocr.pred.mtt = ROCR::prediction(pred.mtt, labels = ml$Any_Health_Problem) #ROCR prediction object
roc.perf.mtt = ROCR::performance(rocr.pred.mtt, measure = "tpr", x.measure = "fpr") # #ROCR performance object
plot(roc.perf.mtt, col = "blue")
abline(a = 0, b = 1, lty = 2) #diagonal for random assignment
```

### Report AUC from ROC for training and test data

```
  # Train AUC
auc <- ROCR::performance(rocr.pred.mtt, measure = "auc")
  auc <- auc@y.values[[1]]
  print(auc)
```

```
## [1] 0.5456078
```

### Calculate Nagelkerke R^2

```
NagelkerkeR2(m)
```

```
## $N
## [1] 2322
## 
## $R2
## [1] 0.01240831
```

### check assumptions of model

#### Cook’s distance

```
plot(m, which = 4, id.n = 3)
```

#### Extract model results and display data for top 3 values using Cook’s distance

```
model.data <- augment(m) %>% 
  mutate(index = 1:n()) 
model.data %>% top_n(3, .cooksd)
```

#### plot standardised residuals

```
ggplot(model.data, aes(index, .std.resid)) + 
  geom_point(aes(color = Any_Health_Problem), alpha = .5) +
  theme_bw()
```

#### Filter potential influential data points with abs(.std.res) > 3:

```
model.data %>% 
  filter(abs(.std.resid) > 3)
```

## DOG DIET + CLIENT DIET binary logistic regression for HEALTH

```
# fit binary logit model and store results 'm'
m <- glm(Any_Health_Problem ~ D_Diet + C_Diet , data = ml,family = binomial)
# view a summary of the model
summary(m)
```

```
## 
## Call:
## glm(formula = Any_Health_Problem ~ D_Diet + C_Diet, family = binomial, 
##     data = ml)
## 
## Coefficients:
##                                                         Estimate Std. Error
## (Intercept)                                             -0.36495    0.07833
## D_DietMeat-based – raw                                  -0.52325    0.09826
## D_DietVegan (consuming no animal products)              -0.77899    0.17197
## D_DietVegetarian (including eggs or milk, but not meat) -0.04204    0.34971
## C_DietOmnivore reducing animal product consumption       0.17792    0.11488
## C_DietPescatarian (including fish but no other meats)    0.57525    0.19412
## C_DietVegan (consuming no animal products)               0.30204    0.14383
## C_DietVegetarian (consuming plants, eggs and milk)       0.18073    0.15153
##                                                         z value Pr(>|z|)    
## (Intercept)                                              -4.659 3.17e-06 ***
## D_DietMeat-based – raw                                   -5.325 1.01e-07 ***
## D_DietVegan (consuming no animal products)               -4.530 5.90e-06 ***
## D_DietVegetarian (including eggs or milk, but not meat)  -0.120  0.90432    
## C_DietOmnivore reducing animal product consumption        1.549  0.12146    
## C_DietPescatarian (including fish but no other meats)     2.963  0.00304 ** 
## C_DietVegan (consuming no animal products)                2.100  0.03573 *  
## C_DietVegetarian (consuming plants, eggs and milk)        1.193  0.23301    
## ---
## Signif. codes:  0 '***' 0.001 '**' 0.01 '*' 0.05 '.' 0.1 ' ' 1
## 
## (Dispersion parameter for binomial family taken to be 1)
## 
##     Null deviance: 3094.1  on 2321  degrees of freedom
## Residual deviance: 3039.0  on 2314  degrees of freedom
## AIC: 3055
## 
## Number of Fisher Scoring iterations: 4
```

```
# test model fit
with(m, null.deviance - deviance)
```

```
## [1] 55.14379
```

```
with(m, df.null - df.residual)
```

```
## [1] 7
```

```
with(m, pchisq(null.deviance - deviance, df.null - df.residual, lower.tail = FALSE))
```

```
## [1] 1.396228e-09
```

```
BIC(m)
```

```
## [1] 3100.982
```

```
# Hosmer-Lemeshow Goodness-of-Fit Test
hltest(m, G=7)
```

```
## 
##    The Hosmer-Lemeshow goodness-of-fit test
## 
##  Group Size Observed  Expected
##      1  406      122 117.90744
##      2  288       84  86.72646
##      3  255       85  84.07026
##      4   71       26  25.57618
##      5  536      216 219.63194
##      6  351      152 157.72868
##      7  170       80  77.48812
##      8  245      128 123.87092
## 
##          Statistic =  1.25679 
## degrees of freedom =  6 
##            p-value =  0.97399
```

```
## CIs using profiled log-likelihood
confint(m, level=0.99)
```

```
## Waiting for profiling to be done...
```

```
##                                                               0.5 %     99.5 %
## (Intercept)                                             -0.56786207 -0.1640722
## D_DietMeat-based – raw                                  -0.77791250 -0.2714631
## D_DietVegan (consuming no animal products)              -1.22632692 -0.3392231
## D_DietVegetarian (including eggs or milk, but not meat) -0.96636340  0.8610264
## C_DietOmnivore reducing animal product consumption      -0.11883719  0.4733821
## C_DietPescatarian (including fish but no other meats)    0.07376129  1.0771531
## C_DietVegan (consuming no animal products)              -0.06946517  0.6723768
## C_DietVegetarian (consuming plants, eggs and milk)      -0.21258639  0.5692892
```

```
## CIs using standard errors
confint.default(m, level=0.99)
```

```
##                                                               0.5 %     99.5 %
## (Intercept)                                             -0.56670543 -0.1632009
## D_DietMeat-based – raw                                  -0.77634410 -0.2701539
## D_DietVegan (consuming no animal products)              -1.22195493 -0.3360222
## D_DietVegetarian (including eggs or milk, but not meat) -0.94282524  0.8587519
## C_DietOmnivore reducing animal product consumption      -0.11800407  0.4738438
## C_DietPescatarian (including fish but no other meats)    0.07522318  1.0752815
## C_DietVegan (consuming no animal products)              -0.06843410  0.6725058
## C_DietVegetarian (consuming plants, eggs and milk)      -0.20960141  0.5710542
```

```
# Wald test
wald.test(b = coef(m), Sigma = vcov(m), Terms = 2)
```

```
## Wald test:
## ----------
## 
## Chi-squared test:
## X2 = 28.4, df = 1, P(> X2) = 1e-07
```

```
## odds ratios and 95% CI
exp(cbind(OR = coef(m), confint(m, level=0.99)))
```

```
## Waiting for profiling to be done...
```

```
##                                                                OR     0.5 %
## (Intercept)                                             0.6942292 0.5667358
## D_DietMeat-based – raw                                  0.5925921 0.4593639
## D_DietVegan (consuming no animal products)              0.4588699 0.2933682
## D_DietVegetarian (including eggs or milk, but not meat) 0.9588346 0.3804641
## C_DietOmnivore reducing animal product consumption      1.1947296 0.8879524
## C_DietPescatarian (including fish but no other meats)   1.7775790 1.0765498
## C_DietVegan (consuming no animal products)              1.3526097 0.9328926
## C_DietVegetarian (consuming plants, eggs and milk)      1.1980874 0.8084905
##                                                            99.5 %
## (Intercept)                                             0.8486808
## D_DietMeat-based – raw                                  0.7622634
## D_DietVegan (consuming no animal products)              0.7123235
## D_DietVegetarian (including eggs or milk, but not meat) 2.3655875
## C_DietOmnivore reducing animal product consumption      1.6054146
## C_DietPescatarian (including fish but no other meats)   2.9363083
## C_DietVegan (consuming no animal products)              1.9588877
## C_DietVegetarian (consuming plants, eggs and milk)      1.7670106
```

### Create ROCR from data

```
pred.mtt = predict(m, type = "response") #repeat risk predictions from model m
rocr.pred.mtt = ROCR::prediction(pred.mtt, labels = ml$Any_Health_Problem) #ROCR prediction object
roc.perf.mtt = ROCR::performance(rocr.pred.mtt, measure = "tpr", x.measure = "fpr") # #ROCR performance object
plot(roc.perf.mtt, col = "blue")
abline(a = 0, b = 1, lty = 2) #diagonal for random assignment
```

### Report AUC from ROC for training and test data

```
  # Train AUC
auc <- ROCR::performance(rocr.pred.mtt, measure = "auc")
  auc <- auc@y.values[[1]]
  print(auc)
```

```
## [1] 0.5884854
```

### Calculate Nagelkerke R^2

```
NagelkerkeR2(m)
```

```
## $N
## [1] 2322
## 
## $R2
## [1] 0.0318785
```

### check assumptions of model

#### Cook’s distance

```
plot(m, which = 4, id.n = 3)
```

#### Extract model results and display data for top 3 values using Cook’s distance

```
model.data <- augment(m) %>% 
  mutate(index = 1:n()) 
model.data %>% top_n(3, .cooksd)
```

#### plot standardised residuals

```
ggplot(model.data, aes(index, .std.resid)) + 
  geom_point(aes(color = Any_Health_Problem), alpha = .5) +
  theme_bw()
```

#### Filter potential influential data points with abs(.std.res) > 3:

```
model.data %>% 
  filter(abs(.std.resid) > 3)
```

#### check for multicollinearity

```
car::vif(m)
```

```
##            GVIF Df GVIF^(1/(2*Df))
## D_Diet 1.715421  3        1.094112
## C_Diet 1.715421  4        1.069785
```

## THERAPEUTIC DIET binary logistic regression for HEALTH

```
# fit binary logit model and store results 'm'
m <- glm(Any_Health_Problem ~ Therapeutic_Food, data = ml,family = binomial)
# view a summary of the model
summary(m)
```

```
## 
## Call:
## glm(formula = Any_Health_Problem ~ Therapeutic_Food, family = binomial, 
##     data = ml)
## 
## Coefficients:
##                     Estimate Std. Error z value Pr(>|z|)    
## (Intercept)         -0.55962    0.04421 -12.658  < 2e-16 ***
## Therapeutic_FoodYes  1.95721    0.24217   8.082 6.37e-16 ***
## ---
## Signif. codes:  0 '***' 0.001 '**' 0.01 '*' 0.05 '.' 0.1 ' ' 1
## 
## (Dispersion parameter for binomial family taken to be 1)
## 
##     Null deviance: 3094.1  on 2321  degrees of freedom
## Residual deviance: 3009.1  on 2320  degrees of freedom
## AIC: 3013.1
## 
## Number of Fisher Scoring iterations: 4
```

```
# test model fit
with(m, null.deviance - deviance)
```

```
## [1] 85.0518
```

```
with(m, df.null - df.residual)
```

```
## [1] 1
```

```
with(m, pchisq(null.deviance - deviance, df.null - df.residual, lower.tail = FALSE))
```

```
## [1] 2.9065e-20
```

```
BIC(m)
```

```
## [1] 3024.573
```

```
## CIs using profiled log-likelihood
confint(m, level=0.99)
```

```
## Waiting for profiling to be done...
```

```
##                         0.5 %     99.5 %
## (Intercept)         -0.674115 -0.4462975
## Therapeutic_FoodYes  1.365113  2.6232872
```

```
## CIs using standard errors
confint.default(m, level=0.99)
```

```
##                          0.5 %     99.5 %
## (Intercept)         -0.6734929 -0.4457387
## Therapeutic_FoodYes  1.3334281  2.5809913
```

```
# Wald test
wald.test(b = coef(m), Sigma = vcov(m), Terms = 2)
```

```
## Wald test:
## ----------
## 
## Chi-squared test:
## X2 = 65.3, df = 1, P(> X2) = 6.7e-16
```

```
## odds ratios and 95% CI
exp(cbind(OR = coef(m), confint(m, level=0.99)))
```

```
## Waiting for profiling to be done...
```

```
##                            OR     0.5 %     99.5 %
## (Intercept)         0.5714286 0.5096072  0.6399934
## Therapeutic_FoodYes 7.0795455 3.9161644 13.7809496
```

### Create ROCR from data

```
pred.mtt = predict(m, type = "response") #repeat risk predictions from model m
rocr.pred.mtt = ROCR::prediction(pred.mtt, labels = ml$Any_Health_Problem) #ROCR prediction object
roc.perf.mtt = ROCR::performance(rocr.pred.mtt, measure = "tpr", x.measure = "fpr") # #ROCR performance object
plot(roc.perf.mtt, col = "blue")
abline(a = 0, b = 1, lty = 2) #diagonal for random assignment
```

### Report AUC from ROC for training and test data

```
  # Train AUC
auc <- ROCR::performance(rocr.pred.mtt, measure = "auc")
  auc <- auc@y.values[[1]]
  print(auc)
```

```
## [1] 0.5421343
```

### Calculate Nagelkerke R^2

```
NagelkerkeR2(m)
```

```
## $N
## [1] 2322
## 
## $R2
## [1] 0.0488542
```

### check assumptions of model

#### Cook’s distance

```
plot(m, which = 4, id.n = 3)
```

#### Extract model results and display data for top 3 values using Cook’s distance

```
model.data <- augment(m) %>% 
  mutate(index = 1:n()) 
model.data %>% top_n(3, .cooksd)
```

#### plot standardised residuals

```
ggplot(model.data, aes(index, .std.resid)) + 
  geom_point(aes(color = Any_Health_Problem), alpha = .5) +
  theme_bw()
```

#### Filter potential influential data points with abs(.std.res) > 3:

```
model.data %>% 
  filter(abs(.std.resid) > 3)
```

## DOG DIET + THERAPEUTIC DIET binary logistic regression for HEALTH

```
# fit binary logit model and store results 'm'
m <- glm(Any_Health_Problem ~ D_Diet + Therapeutic_Food, data = ml,family = binomial)
# view a summary of the model
summary(m)
```

```
## 
## Call:
## glm(formula = Any_Health_Problem ~ D_Diet + Therapeutic_Food, 
##     family = binomial, data = ml)
## 
## Coefficients:
##                                                         Estimate Std. Error
## (Intercept)                                             -0.33349    0.05949
## D_DietMeat-based – raw                                  -0.46020    0.09883
## D_DietVegan (consuming no animal products)              -0.60676    0.13875
## D_DietVegetarian (including eggs or milk, but not meat) -0.02816    0.35637
## Therapeutic_FoodYes                                      1.86559    0.24420
##                                                         z value Pr(>|z|)    
## (Intercept)                                              -5.606 2.08e-08 ***
## D_DietMeat-based – raw                                   -4.657 3.21e-06 ***
## D_DietVegan (consuming no animal products)               -4.373 1.22e-05 ***
## D_DietVegetarian (including eggs or milk, but not meat)  -0.079    0.937    
## Therapeutic_FoodYes                                       7.640 2.18e-14 ***
## ---
## Signif. codes:  0 '***' 0.001 '**' 0.01 '*' 0.05 '.' 0.1 ' ' 1
## 
## (Dispersion parameter for binomial family taken to be 1)
## 
##     Null deviance: 3094.1  on 2321  degrees of freedom
## Residual deviance: 2975.6  on 2317  degrees of freedom
## AIC: 2985.6
## 
## Number of Fisher Scoring iterations: 4
```

```
# test model fit
with(m, null.deviance - deviance)
```

```
## [1] 118.4856
```

```
with(m, df.null - df.residual)
```

```
## [1] 4
```

```
with(m, pchisq(null.deviance - deviance, df.null - df.residual, lower.tail = FALSE))
```

```
## [1] 1.124815e-24
```

```
BIC(m)
```

```
## [1] 3014.39
```

```
# Hosmer-Lemeshow Goodness-of-Fit Test
hltest(m, G=3)
```

```
## 
##    The Hosmer-Lemeshow goodness-of-fit test
## 
##  Group Size Observed   Expected
##      1  299       84  83.974196
##      2  734      226 228.550425
##      3   31       12  12.727356
##      4 1147      482 478.748023
##      5   14       10  10.025804
##      6   10       10   7.449575
##      7    4        4   3.272644
##      8   83       65  68.251977
## 
##          Statistic =  5.33462 
## degrees of freedom =  6 
##            p-value =  0.50167
```

```
## CIs using profiled log-likelihood
confint(m, level=0.99)
```

```
## Waiting for profiling to be done...
```

```
##                                                              0.5 %     99.5 %
## (Intercept)                                             -0.4874569 -0.1808202
## D_DietMeat-based – raw                                  -0.7162628 -0.2068877
## D_DietVegan (consuming no animal products)              -0.9704186 -0.2544882
## D_DietVegetarian (including eggs or milk, but not meat) -0.9753500  0.8868679
## Therapeutic_FoodYes                                      1.2677225  2.5362738
```

```
## CIs using standard errors
confint.default(m, level=0.99)
```

```
##                                                              0.5 %     99.5 %
## (Intercept)                                             -0.4867338 -0.1802480
## D_DietMeat-based – raw                                  -0.7147600 -0.2056410
## D_DietVegan (consuming no animal products)              -0.9641416 -0.2493735
## D_DietVegetarian (including eggs or milk, but not meat) -0.9460998  0.8897789
## Therapeutic_FoodYes                                      1.2365654  2.4946112
```

```
# Wald test
wald.test(b = coef(m), Sigma = vcov(m), Terms = 2)
```

```
## Wald test:
## ----------
## 
## Chi-squared test:
## X2 = 21.7, df = 1, P(> X2) = 3.2e-06
```

```
## odds ratios and 95% CI
exp(cbind(OR = coef(m), confint(m, level=0.99)))
```

```
## Waiting for profiling to be done...
```

```
##                                                                OR     0.5 %
## (Intercept)                                             0.7164184 0.6141863
## D_DietMeat-based – raw                                  0.6311571 0.4885748
## D_DietVegan (consuming no animal products)              0.5451155 0.3789244
## D_DietVegetarian (including eggs or milk, but not meat) 0.9722324 0.3770604
## Therapeutic_FoodYes                                     6.4597349 3.5527518
##                                                             99.5 %
## (Intercept)                                              0.8345854
## D_DietMeat-based – raw                                   0.8131110
## D_DietVegan (consuming no animal products)               0.7753132
## D_DietVegetarian (including eggs or milk, but not meat)  2.4275146
## Therapeutic_FoodYes                                     12.6325119
```

### Create ROCR from data

```
pred.mtt = predict(m, type = "response") #repeat risk predictions from model m
rocr.pred.mtt = ROCR::prediction(pred.mtt, labels = ml$Any_Health_Problem) #ROCR prediction object
roc.perf.mtt = ROCR::performance(rocr.pred.mtt, measure = "tpr", x.measure = "fpr") # #ROCR performance object
plot(roc.perf.mtt, col = "blue")
abline(a = 0, b = 1, lty = 2) #diagonal for random assignment
```

### Report AUC from ROC for training and test data

```
  # Train AUC
auc <- ROCR::performance(rocr.pred.mtt, measure = "auc")
  auc <- auc@y.values[[1]]
  print(auc)
```

```
## [1] 0.6017309
```

### Calculate Nagelkerke R^2

```
NagelkerkeR2(m)
```

```
## $N
## [1] 2322
## 
## $R2
## [1] 0.06757409
```

### check assumptions of model

#### Cook’s distance

```
plot(m, which = 4, id.n = 3)
```

#### Extract model results and display data for top 3 values using Cook’s distance

```
model.data <- augment(m) %>% 
  mutate(index = 1:n()) 
model.data %>% top_n(3, .cooksd)
```

#### plot standardised residuals

```
ggplot(model.data, aes(index, .std.resid)) + 
  geom_point(aes(color = Any_Health_Problem), alpha = .5) +
  theme_bw()
```

#### Filter potential influential data points with abs(.std.res) > 3:

```
model.data %>% 
  filter(abs(.std.resid) > 3)
```

#### check for multicollinearity

```
car::vif(m)
```

```
##                     GVIF Df GVIF^(1/(2*Df))
## D_Diet           1.00685  3        1.001138
## Therapeutic_Food 1.00685  1        1.003419
```

## DOG DIET \* THERAPEUTIC DIET binary logistic regression for HEALTH

```
# fit binary logit model and store results 'm'
m <- glm(Any_Health_Problem ~ D_Diet*Therapeutic_Food, data = ml,family = binomial)
# view a summary of the model
summary(m)
```

```
## 
## Call:
## glm(formula = Any_Health_Problem ~ D_Diet * Therapeutic_Food, 
##     family = binomial, data = ml)
## 
## Coefficients:
##                                                                              Estimate
## (Intercept)                                                                  -0.32184
## D_DietMeat-based – raw                                                       -0.48810
## D_DietVegan (consuming no animal products)                                   -0.61798
## D_DietVegetarian (including eggs or milk, but not meat)                      -0.13769
## Therapeutic_FoodYes                                                           1.60586
## D_DietMeat-based – raw:Therapeutic_FoodYes                                   13.77016
## D_DietVegan (consuming no animal products):Therapeutic_FoodYes                0.25025
## D_DietVegetarian (including eggs or milk, but not meat):Therapeutic_FoodYes  13.41974
##                                                                             Std. Error
## (Intercept)                                                                    0.05982
## D_DietMeat-based – raw                                                         0.09986
## D_DietVegan (consuming no animal products)                                     0.14190
## D_DietVegetarian (including eggs or milk, but not meat)                        0.37356
## Therapeutic_FoodYes                                                            0.27298
## D_DietMeat-based – raw:Therapeutic_FoodYes                                   279.14811
## D_DietVegan (consuming no animal products):Therapeutic_FoodYes                 0.66413
## D_DietVegetarian (including eggs or milk, but not meat):Therapeutic_FoodYes  441.37193
##                                                                             z value
## (Intercept)                                                                  -5.380
## D_DietMeat-based – raw                                                       -4.888
## D_DietVegan (consuming no animal products)                                   -4.355
## D_DietVegetarian (including eggs or milk, but not meat)                      -0.369
## Therapeutic_FoodYes                                                           5.883
## D_DietMeat-based – raw:Therapeutic_FoodYes                                    0.049
## D_DietVegan (consuming no animal products):Therapeutic_FoodYes                0.377
## D_DietVegetarian (including eggs or milk, but not meat):Therapeutic_FoodYes   0.030
##                                                                             Pr(>|z|)
## (Intercept)                                                                 7.44e-08
## D_DietMeat-based – raw                                                      1.02e-06
## D_DietVegan (consuming no animal products)                                  1.33e-05
## D_DietVegetarian (including eggs or milk, but not meat)                        0.712
## Therapeutic_FoodYes                                                         4.04e-09
## D_DietMeat-based – raw:Therapeutic_FoodYes                                     0.961
## D_DietVegan (consuming no animal products):Therapeutic_FoodYes                 0.706
## D_DietVegetarian (including eggs or milk, but not meat):Therapeutic_FoodYes    0.976
##                                                                                
## (Intercept)                                                                 ***
## D_DietMeat-based – raw                                                      ***
## D_DietVegan (consuming no animal products)                                  ***
## D_DietVegetarian (including eggs or milk, but not meat)                        
## Therapeutic_FoodYes                                                         ***
## D_DietMeat-based – raw:Therapeutic_FoodYes                                     
## D_DietVegan (consuming no animal products):Therapeutic_FoodYes                 
## D_DietVegetarian (including eggs or milk, but not meat):Therapeutic_FoodYes    
## ---
## Signif. codes:  0 '***' 0.001 '**' 0.01 '*' 0.05 '.' 0.1 ' ' 1
## 
## (Dispersion parameter for binomial family taken to be 1)
## 
##     Null deviance: 3094.1  on 2321  degrees of freedom
## Residual deviance: 2967.2  on 2314  degrees of freedom
## AIC: 2983.2
## 
## Number of Fisher Scoring iterations: 13
```

```
# test model fit
with(m, null.deviance - deviance)
```

```
## [1] 126.9572
```

```
with(m, df.null - df.residual)
```

```
## [1] 7
```

```
with(m, pchisq(null.deviance - deviance, df.null - df.residual, lower.tail = FALSE))
```

```
## [1] 2.714884e-24
```

```
BIC(m)
```

```
## [1] 3029.169
```

```
# Hosmer-Lemeshow Goodness-of-Fit Test
hltest(m, G=7)
```

```
## 
##    The Hosmer-Lemeshow goodness-of-fit test
## 
##  Group Size Observed  Expected
##      1  299       84  84.00000
##      2  734      226 226.00000
##      3   31       12  12.00000
##      4 1147      482 482.00000
##      5   14       10  10.00000
##      6   83       65  65.00000
##      7   14       14  13.99999
## 
##          Statistic =  1e-05 
## degrees of freedom =  5 
##            p-value =  1
```

```
## CIs using profiled log-likelihood
confint(m, level=0.99)
```

```
## Waiting for profiling to be done...
```

```
## Warning: glm.fit: fitted probabilities numerically 0 or 1 occurred

## Warning: glm.fit: fitted probabilities numerically 0 or 1 occurred

## Warning: glm.fit: fitted probabilities numerically 0 or 1 occurred

## Warning: glm.fit: fitted probabilities numerically 0 or 1 occurred

## Warning: glm.fit: fitted probabilities numerically 0 or 1 occurred

## Warning: glm.fit: fitted probabilities numerically 0 or 1 occurred

## Warning: glm.fit: fitted probabilities numerically 0 or 1 occurred

## Warning: glm.fit: fitted probabilities numerically 0 or 1 occurred

## Warning: glm.fit: fitted probabilities numerically 0 or 1 occurred

## Warning: glm.fit: fitted probabilities numerically 0 or 1 occurred

## Warning: glm.fit: fitted probabilities numerically 0 or 1 occurred

## Warning: glm.fit: fitted probabilities numerically 0 or 1 occurred

## Warning: glm.fit: fitted probabilities numerically 0 or 1 occurred

## Warning: glm.fit: fitted probabilities numerically 0 or 1 occurred

## Warning: glm.fit: fitted probabilities numerically 0 or 1 occurred

## Warning: glm.fit: fitted probabilities numerically 0 or 1 occurred

## Warning: glm.fit: fitted probabilities numerically 0 or 1 occurred

## Warning: glm.fit: fitted probabilities numerically 0 or 1 occurred

## Warning: glm.fit: fitted probabilities numerically 0 or 1 occurred

## Warning: glm.fit: fitted probabilities numerically 0 or 1 occurred

## Warning: glm.fit: fitted probabilities numerically 0 or 1 occurred
```

```
##                                                                                   0.5 %
## (Intercept)                                                                  -0.4766393
## D_DietMeat-based – raw                                                       -0.7469774
## D_DietVegan (consuming no animal products)                                   -0.9906653
## D_DietVegetarian (including eggs or milk, but not meat)                      -1.1514834
## Therapeutic_FoodYes                                                           0.9383835
## D_DietMeat-based – raw:Therapeutic_FoodYes                                  -18.9221530
## D_DietVegan (consuming no animal products):Therapeutic_FoodYes               -1.3890643
## D_DietVegetarian (including eggs or milk, but not meat):Therapeutic_FoodYes -64.5847734
##                                                                                 99.5 %
## (Intercept)                                                                 -0.1683117
## D_DietMeat-based – raw                                                      -0.2322693
## D_DietVegan (consuming no animal products)                                  -0.2583276
## D_DietVegetarian (including eggs or milk, but not meat)                      0.8083259
## Therapeutic_FoodYes                                                          2.3591431
## D_DietMeat-based – raw:Therapeutic_FoodYes                                          NA
## D_DietVegan (consuming no animal products):Therapeutic_FoodYes               2.1638587
## D_DietVegetarian (including eggs or milk, but not meat):Therapeutic_FoodYes         NA
```

```
## CIs using standard errors
confint.default(m, level=0.99)
```

```
##                                                                                     0.5 %
## (Intercept)                                                                    -0.4759294
## D_DietMeat-based – raw                                                         -0.7453221
## D_DietVegan (consuming no animal products)                                     -0.9834773
## D_DietVegetarian (including eggs or milk, but not meat)                        -1.0999034
## Therapeutic_FoodYes                                                             0.9027060
## D_DietMeat-based – raw:Therapeutic_FoodYes                                   -705.2677271
## D_DietVegan (consuming no animal products):Therapeutic_FoodYes                 -1.4604434
## D_DietVegetarian (including eggs or milk, but not meat):Therapeutic_FoodYes -1123.4789991
##                                                                                   99.5 %
## (Intercept)                                                                   -0.1677565
## D_DietMeat-based – raw                                                        -0.2308849
## D_DietVegan (consuming no animal products)                                    -0.2524794
## D_DietVegetarian (including eggs or milk, but not meat)                        0.8245246
## Therapeutic_FoodYes                                                            2.3090109
## D_DietMeat-based – raw:Therapeutic_FoodYes                                   732.8080386
## D_DietVegan (consuming no animal products):Therapeutic_FoodYes                 1.9609505
## D_DietVegetarian (including eggs or milk, but not meat):Therapeutic_FoodYes 1150.3184825
```

```
# Wald test
wald.test(b = coef(m), Sigma = vcov(m), Terms = 2)
```

```
## Wald test:
## ----------
## 
## Chi-squared test:
## X2 = 23.9, df = 1, P(> X2) = 1e-06
```

```
## odds ratios and 95% CI
exp(cbind(OR = coef(m), confint(m, level=0.99)))
```

```
## Waiting for profiling to be done...
```

```
## Warning: glm.fit: fitted probabilities numerically 0 or 1 occurred

## Warning: glm.fit: fitted probabilities numerically 0 or 1 occurred

## Warning: glm.fit: fitted probabilities numerically 0 or 1 occurred

## Warning: glm.fit: fitted probabilities numerically 0 or 1 occurred

## Warning: glm.fit: fitted probabilities numerically 0 or 1 occurred

## Warning: glm.fit: fitted probabilities numerically 0 or 1 occurred

## Warning: glm.fit: fitted probabilities numerically 0 or 1 occurred

## Warning: glm.fit: fitted probabilities numerically 0 or 1 occurred

## Warning: glm.fit: fitted probabilities numerically 0 or 1 occurred

## Warning: glm.fit: fitted probabilities numerically 0 or 1 occurred

## Warning: glm.fit: fitted probabilities numerically 0 or 1 occurred

## Warning: glm.fit: fitted probabilities numerically 0 or 1 occurred

## Warning: glm.fit: fitted probabilities numerically 0 or 1 occurred

## Warning: glm.fit: fitted probabilities numerically 0 or 1 occurred

## Warning: glm.fit: fitted probabilities numerically 0 or 1 occurred

## Warning: glm.fit: fitted probabilities numerically 0 or 1 occurred

## Warning: glm.fit: fitted probabilities numerically 0 or 1 occurred

## Warning: glm.fit: fitted probabilities numerically 0 or 1 occurred

## Warning: glm.fit: fitted probabilities numerically 0 or 1 occurred

## Warning: glm.fit: fitted probabilities numerically 0 or 1 occurred

## Warning: glm.fit: fitted probabilities numerically 0 or 1 occurred
```

```
##                                                                                       OR
## (Intercept)                                                                 7.248120e-01
## D_DietMeat-based – raw                                                      6.137893e-01
## D_DietVegan (consuming no animal products)                                  5.390331e-01
## D_DietVegetarian (including eggs or milk, but not meat)                     8.713693e-01
## Therapeutic_FoodYes                                                         4.982135e+00
## D_DietMeat-based – raw:Therapeutic_FoodYes                                  9.556584e+05
## D_DietVegan (consuming no animal products):Therapeutic_FoodYes              1.284351e+00
## D_DietVegetarian (including eggs or milk, but not meat):Therapeutic_FoodYes 6.731623e+05
##                                                                                    0.5 %
## (Intercept)                                                                 6.208665e-01
## D_DietMeat-based – raw                                                      4.737965e-01
## D_DietVegan (consuming no animal products)                                  3.713296e-01
## D_DietVegetarian (including eggs or milk, but not meat)                     3.161674e-01
## Therapeutic_FoodYes                                                         2.555847e+00
## D_DietMeat-based – raw:Therapeutic_FoodYes                                  6.056383e-09
## D_DietVegan (consuming no animal products):Therapeutic_FoodYes              2.493085e-01
## D_DietVegetarian (including eggs or milk, but not meat):Therapeutic_FoodYes 8.936949e-29
##                                                                                 99.5 %
## (Intercept)                                                                  0.8450904
## D_DietMeat-based – raw                                                       0.7927326
## D_DietVegan (consuming no animal products)                                   0.7723422
## D_DietVegetarian (including eggs or milk, but not meat)                      2.2441478
## Therapeutic_FoodYes                                                         10.5818803
## D_DietMeat-based – raw:Therapeutic_FoodYes                                          NA
## D_DietVegan (consuming no animal products):Therapeutic_FoodYes               8.7046618
## D_DietVegetarian (including eggs or milk, but not meat):Therapeutic_FoodYes         NA
```

### Create ROCR from data

```
pred.mtt = predict(m, type = "response") #repeat risk predictions from model m
rocr.pred.mtt = ROCR::prediction(pred.mtt, labels = ml$Any_Health_Problem) #ROCR prediction object
roc.perf.mtt = ROCR::performance(rocr.pred.mtt, measure = "tpr", x.measure = "fpr") # #ROCR performance object
plot(roc.perf.mtt, col = "blue")
abline(a = 0, b = 1, lty = 2) #diagonal for random assignment
```

### Report AUC from ROC for training and test data

```
  # Train AUC
auc <- ROCR::performance(rocr.pred.mtt, measure = "auc")
  auc <- auc@y.values[[1]]
  print(auc)
```

```
## [1] 0.6019284
```

### Calculate Nagelkerke R^2

```
NagelkerkeR2(m)
```

```
## $N
## [1] 2322
## 
## $R2
## [1] 0.07227477
```

### check assumptions of model

#### Cook’s distance

```
plot(m, which = 4, id.n = 3)
```

#### Extract model results and display data for top 3 values using Cook’s distance

```
model.data <- augment(m) %>% 
  mutate(index = 1:n()) 
model.data %>% top_n(3, .cooksd)
```

#### plot standardised residuals

```
ggplot(model.data, aes(index, .std.resid)) + 
  geom_point(aes(color = Any_Health_Problem), alpha = .5) +
  theme_bw()
```

#### Filter potential influential data points with abs(.std.res) > 3:

```
model.data %>% 
  filter(abs(.std.resid) > 3)
```

#### check for multicollinearity

#### Note interactions

```
car::vif(m)
```

```
## there are higher-order terms (interactions) in this model
## consider setting type = 'predictor'; see ?vif
```

```
##                             GVIF Df GVIF^(1/(2*Df))
## D_Diet                  1.064305  3        1.010441
## Therapeutic_Food        1.222211  1        1.105537
## D_Diet:Therapeutic_Food 1.253299  3        1.038347
```

## SIZE Binary logistic regression for HEALTH

```
# fit binary logit model and store results 'm'
m <- glm(Any_Health_Problem ~ Size, data = ml,family = binomial)
# view a summary of the model
summary(m)
```

```
## 
## Call:
## glm(formula = Any_Health_Problem ~ Size, family = binomial, data = ml)
## 
## Coefficients:
##             Estimate Std. Error z value Pr(>|z|)    
## (Intercept) -0.41492    0.07336  -5.656 1.55e-08 ***
## Size.L       0.05058    0.21833   0.232   0.8168    
## Size.Q       0.13913    0.18826   0.739   0.4599    
## Size.C       0.14150    0.13104   1.080   0.2802    
## Size^4      -0.15093    0.08580  -1.759   0.0786 .  
## ---
## Signif. codes:  0 '***' 0.001 '**' 0.01 '*' 0.05 '.' 0.1 ' ' 1
## 
## (Dispersion parameter for binomial family taken to be 1)
## 
##     Null deviance: 3094.1  on 2321  degrees of freedom
## Residual deviance: 3086.8  on 2317  degrees of freedom
## AIC: 3096.8
## 
## Number of Fisher Scoring iterations: 4
```

```
# test model fit
with(m, null.deviance - deviance)
```

```
## [1] 7.325022
```

```
with(m, df.null - df.residual)
```

```
## [1] 4
```

```
with(m, pchisq(null.deviance - deviance, df.null - df.residual, lower.tail = FALSE))
```

```
## [1] 0.1196772
```

```
BIC(m)
```

```
## [1] 3125.551
```

```
# Hosmer-Lemeshow Goodness-of-Fit Test
hltest(m)
```

```
## 
##    The Hosmer-Lemeshow goodness-of-fit test
## 
##  Group Size Observed Expected
##      1  896      318      318
##      2  803      312      312
##      3   56       22       22
##      4  467      198      198
##      5  100       43       43
## 
##          Statistic =  0 
## degrees of freedom =  3 
##            p-value =  1
```

```
## CIs using profiled log-likelihood
confint(m, level=0.99)
```

```
## Waiting for profiling to be done...
```

```
##                  0.5 %      99.5 %
## (Intercept) -0.6070605 -0.22763879
## Size.L      -0.5088845  0.62172183
## Size.Q      -0.3549063  0.61938890
## Size.C      -0.1949779  0.48184301
## Size^4      -0.3725053  0.06972608
```

```
## CIs using standard errors
confint.default(m, level=0.99)
```

```
##                  0.5 %      99.5 %
## (Intercept) -0.6038840 -0.22594676
## Size.L      -0.5118006  0.61295376
## Size.Q      -0.3457853  0.62405335
## Size.C      -0.1960278  0.47902637
## Size^4      -0.3719349  0.07007683
```

```
# Wald test
wald.test(b = coef(m), Sigma = vcov(m), Terms = 2)
```

```
## Wald test:
## ----------
## 
## Chi-squared test:
## X2 = 0.054, df = 1, P(> X2) = 0.82
```

```
## odds ratios and 95% CI
exp(cbind(OR = coef(m), confint(m, level=0.99)))
```

```
## Waiting for profiling to be done...
```

```
##                    OR     0.5 %    99.5 %
## (Intercept) 0.6603961 0.5449504 0.7964119
## Size.L      1.0518774 0.6011658 1.8621316
## Size.Q      1.1492781 0.7012391 1.8577924
## Size.C      1.1519997 0.8228529 1.6190556
## Size^4      0.8599087 0.6890060 1.0722144
```

### Create ROCR from data

```
pred.mtt = predict(m, type = "response") #repeat risk predictions from model m
rocr.pred.mtt = ROCR::prediction(pred.mtt, labels = ml$Any_Health_Problem) #ROCR prediction object
roc.perf.mtt = ROCR::performance(rocr.pred.mtt, measure = "tpr", x.measure = "fpr") # #ROCR performance object
plot(roc.perf.mtt, col = "blue")
abline(a = 0, b = 1, lty = 2) #diagonal for random assignment
```

### Report AUC from ROC for training and test data

```
  # Train AUC
auc <- ROCR::performance(rocr.pred.mtt, measure = "auc")
  auc <- auc@y.values[[1]]
  print(auc)
```

```
## [1] 0.531286
```

### Calculate Nagelkerke R^2

```
NagelkerkeR2(m)
```

```
## $N
## [1] 2322
## 
## $R2
## [1] 0.004278308
```

### check assumptions of model

#### Cook’s distance

```
plot(m, which = 4, id.n = 3)
```

#### Extract model results and display data for top 3 values using Cook’s distance

```
model.data <- augment(m) %>% 
  mutate(index = 1:n()) 
model.data %>% top_n(3, .cooksd)
```

#### plot standardised residuals

```
ggplot(model.data, aes(index, .std.resid)) + 
  geom_point(aes(color = Any_Health_Problem), alpha = .5) +
  theme_bw()
```

#### Filter potential influential data points with abs(.std.res) > 3:

```
model.data %>% 
  filter(abs(.std.resid) > 3)
```

## SIZE2 Binary logistic regression for HEALTH

```
# fit binary logit model and store results 'm'
m <- glm(Any_Health_Problem ~ Size2, data = ml,family = binomial)
# view a summary of the model
summary(m)
```

```
## 
## Call:
## glm(formula = Any_Health_Problem ~ Size2, family = binomial, 
##     data = ml)
## 
## Coefficients:
##             Estimate Std. Error z value Pr(>|z|)    
## (Intercept) -0.59752    0.06982  -8.558   <2e-16 ***
## Size2Toy     0.16220    0.28238   0.574   0.5657    
## Size2Small   0.29108    0.11680   2.492   0.0127 *  
## Size2Large   0.14408    0.10058   1.432   0.1520    
## Size2Giant   0.31567    0.21372   1.477   0.1397    
## ---
## Signif. codes:  0 '***' 0.001 '**' 0.01 '*' 0.05 '.' 0.1 ' ' 1
## 
## (Dispersion parameter for binomial family taken to be 1)
## 
##     Null deviance: 3094.1  on 2321  degrees of freedom
## Residual deviance: 3086.8  on 2317  degrees of freedom
## AIC: 3096.8
## 
## Number of Fisher Scoring iterations: 4
```

```
# test model fit
with(m, null.deviance - deviance)
```

```
## [1] 7.325022
```

```
with(m, df.null - df.residual)
```

```
## [1] 4
```

```
with(m, pchisq(null.deviance - deviance, df.null - df.residual, lower.tail = FALSE))
```

```
## [1] 0.1196772
```

```
BIC(m)
```

```
## [1] 3125.551
```

```
# Hosmer-Lemeshow Goodness-of-Fit Test
hltest(m)
```

```
## 
##    The Hosmer-Lemeshow goodness-of-fit test
## 
##  Group Size Observed Expected
##      1  896      318      318
##      2  803      312      312
##      3   56       22       22
##      4  467      198      198
##      5  100       43       43
## 
##          Statistic =  0 
## degrees of freedom =  3 
##            p-value =  1
```

```
## CIs using profiled log-likelihood
confint(m, level=0.99)
```

```
## Waiting for profiling to be done...
```

```
##                   0.5 %     99.5 %
## (Intercept) -0.77905807 -0.4191220
## Size2Toy    -0.58883357  0.8796012
## Size2Small  -0.01036949  0.5917420
## Size2Large  -0.11502302  0.4033544
## Size2Giant  -0.24289917  0.8631665
```

```
## CIs using standard errors
confint.default(m, level=0.99)
```

```
##                    0.5 %     99.5 %
## (Intercept) -0.777365500 -0.4176795
## Size2Toy    -0.565170129  0.8895790
## Size2Small  -0.009784176  0.5919405
## Size2Large  -0.114998038  0.4031611
## Size2Giant  -0.234824011  0.8661667
```

```
# Wald test
wald.test(b = coef(m), Sigma = vcov(m), Terms = 2)
```

```
## Wald test:
## ----------
## 
## Chi-squared test:
## X2 = 0.33, df = 1, P(> X2) = 0.57
```

```
## odds ratios and 95% CI
exp(cbind(OR = coef(m), confint(m, level=0.99)))
```

```
## Waiting for profiling to be done...
```

```
##                   OR     0.5 %   99.5 %
## (Intercept) 0.550173 0.4588380 0.657624
## Size2Toy    1.176101 0.5549742 2.409939
## Size2Small  1.337869 0.9896841 1.807134
## Size2Large  1.154978 0.8913456 1.496837
## Size2Giant  1.371180 0.7843506 2.370656
```

### Create ROCR from data

```
pred.mtt = predict(m, type = "response") #repeat risk predictions from model m
rocr.pred.mtt = ROCR::prediction(pred.mtt, labels = ml$Any_Health_Problem) #ROCR prediction object
roc.perf.mtt = ROCR::performance(rocr.pred.mtt, measure = "tpr", x.measure = "fpr") # #ROCR performance object
plot(roc.perf.mtt, col = "blue")
abline(a = 0, b = 1, lty = 2) #diagonal for random assignment
```

### Report AUC from ROC for training and test data

```
  # Train AUC
auc <- ROCR::performance(rocr.pred.mtt, measure = "auc")
  auc <- auc@y.values[[1]]
  print(auc)
```

```
## [1] 0.531286
```

### Calculate Nagelkerke R^2

```
NagelkerkeR2(m)
```

```
## $N
## [1] 2322
## 
## $R2
## [1] 0.004278308
```

### check assumptions of model

#### Cook’s distance

```
plot(m, which = 4, id.n = 3)
```

#### Extract model results and display data for top 3 values using Cook’s distance

```
model.data <- augment(m) %>% 
  mutate(index = 1:n()) 
model.data %>% top_n(3, .cooksd)
```

#### plot standardised residuals

```
ggplot(model.data, aes(index, .std.resid)) + 
  geom_point(aes(color = Any_Health_Problem), alpha = .5) +
  theme_bw()
```

#### Filter potential influential data points with abs(.std.res) > 3:

```
model.data %>% 
  filter(abs(.std.resid) > 3)
```

## SIZE\_GIANT Binary logistic regression for HEALTH

```
# fit binary logit model and store results 'm'
m <- glm(Any_Health_Problem ~ Size_Giant, data = ml,family = binomial)
# view a summary of the model
summary(m)
```

```
## 
## Call:
## glm(formula = Any_Health_Problem ~ Size_Giant, family = binomial, 
##     data = ml)
## 
## Coefficients:
##               Estimate Std. Error z value Pr(>|z|)    
## (Intercept)   -0.47879    0.04365 -10.969   <2e-16 ***
## Size_GiantYes  0.19694    0.20665   0.953    0.341    
## ---
## Signif. codes:  0 '***' 0.001 '**' 0.01 '*' 0.05 '.' 0.1 ' ' 1
## 
## (Dispersion parameter for binomial family taken to be 1)
## 
##     Null deviance: 3094.1  on 2321  degrees of freedom
## Residual deviance: 3093.2  on 2320  degrees of freedom
## AIC: 3097.2
## 
## Number of Fisher Scoring iterations: 4
```

```
# test model fit
with(m, null.deviance - deviance)
```

```
## [1] 0.8994133
```

```
with(m, df.null - df.residual)
```

```
## [1] 1
```

```
with(m, pchisq(null.deviance - deviance, df.null - df.residual, lower.tail = FALSE))
```

```
## [1] 0.3429391
```

```
BIC(m)
```

```
## [1] 3108.726
```

```
## CIs using profiled log-likelihood
confint(m, level=0.99)
```

```
## Waiting for profiling to be done...
```

```
##                    0.5 %     99.5 %
## (Intercept)   -0.5917498 -0.3668187
## Size_GiantYes -0.3442121  0.7259525
```

```
## CIs using standard errors
confint.default(m, level=0.99)
```

```
##                    0.5 %     99.5 %
## (Intercept)   -0.5912237 -0.3663532
## Size_GiantYes -0.3353627  0.7292373
```

```
# Wald test
wald.test(b = coef(m), Sigma = vcov(m), Terms = 2)
```

```
## Wald test:
## ----------
## 
## Chi-squared test:
## X2 = 0.91, df = 1, P(> X2) = 0.34
```

```
## odds ratios and 95% CI
exp(cbind(OR = coef(m), confint(m, level=0.99)))
```

```
## Waiting for profiling to be done...
```

```
##                      OR     0.5 %    99.5 %
## (Intercept)   0.6195335 0.5533582 0.6929353
## Size_GiantYes 1.2176677 0.7087786 2.0666987
```

### Create ROCR from data

```
pred.mtt = predict(m, type = "response") #repeat risk predictions from model m
rocr.pred.mtt = ROCR::prediction(pred.mtt, labels = ml$Any_Health_Problem) #ROCR prediction object
roc.perf.mtt = ROCR::performance(rocr.pred.mtt, measure = "tpr", x.measure = "fpr") # #ROCR performance object
plot(roc.perf.mtt, col = "blue")
abline(a = 0, b = 1, lty = 2) #diagonal for random assignment
```

### Report AUC from ROC for training and test data

```
  # Train AUC
auc <- ROCR::performance(rocr.pred.mtt, measure = "auc")
  auc <- auc@y.values[[1]]
  print(auc)
```

```
## [1] 0.5041321
```

### Calculate Nagelkerke R^2

```
NagelkerkeR2(m)
```

```
## $N
## [1] 2322
## 
## $R2
## [1] 0.0005260453
```

### check assumptions of model

#### Cook’s distance

```
plot(m, which = 4, id.n = 3)
```

#### Extract model results and display data for top 3 values using Cook’s distance

```
model.data <- augment(m) %>% 
  mutate(index = 1:n()) 
model.data %>% top_n(3, .cooksd)
```

#### plot standardised residuals

```
ggplot(model.data, aes(index, .std.resid)) + 
  geom_point(aes(color = Any_Health_Problem), alpha = .5) +
  theme_bw()
```

#### Filter potential influential data points with abs(.std.res) > 3:

```
model.data %>% 
  filter(abs(.std.resid) > 3)
```

## D\_AGE Binary logistic regression for HEALTH

```
# fit binary logit model and store results 'm'
m <- glm(Any_Health_Problem ~ D_Age, data = ml,family = binomial)
# view a summary of the model
summary(m)
```

```
## 
## Call:
## glm(formula = Any_Health_Problem ~ D_Age, family = binomial, 
##     data = ml)
## 
## Coefficients:
##             Estimate Std. Error z value Pr(>|z|)    
## (Intercept) -1.39520    0.09120  -15.30   <2e-16 ***
## D_Age        0.14541    0.01231   11.81   <2e-16 ***
## ---
## Signif. codes:  0 '***' 0.001 '**' 0.01 '*' 0.05 '.' 0.1 ' ' 1
## 
## (Dispersion parameter for binomial family taken to be 1)
## 
##     Null deviance: 3094.1  on 2321  degrees of freedom
## Residual deviance: 2945.3  on 2320  degrees of freedom
## AIC: 2949.3
## 
## Number of Fisher Scoring iterations: 4
```

```
# test model fit
with(m, null.deviance - deviance)
```

```
## [1] 148.847
```

```
with(m, df.null - df.residual)
```

```
## [1] 1
```

```
with(m, pchisq(null.deviance - deviance, df.null - df.residual, lower.tail = FALSE))
```

```
## [1] 3.097288e-34
```

```
BIC(m)
```

```
## [1] 2960.778
```

```
# Hosmer-Lemeshow Goodness-of-Fit Test
hltest(m)
```

```
## 
##    The Hosmer-Lemeshow goodness-of-fit test
## 
##  Group Size Observed  Expected
##      1  150       40  33.41058
##      2  255       72  63.47484
##      3  256       71  70.93387
##      4  241       72  74.01917
##      5  242       72  82.01681
##      6  204       71  75.93148
##      7  192       64  78.10033
##      8  177       83  78.28358
##      9  277      134 137.38228
##     10  235      154 136.29019
##     11   93       60  63.15689
## 
##          Statistic =  16.5731 
## degrees of freedom =  9 
##            p-value =  0.055836
```

```
## CIs using profiled log-likelihood
confint(m, level=0.99)
```

```
## Waiting for profiling to be done...
```

```
##                  0.5 %     99.5 %
## (Intercept) -1.6329469 -1.1628767
## D_Age        0.1139952  0.1774392
```

```
## CIs using standard errors
confint.default(m, level=0.99)
```

```
##                  0.5 %     99.5 %
## (Intercept) -1.6301137 -1.1602792
## D_Age        0.1137079  0.1771129
```

```
# Wald test
wald.test(b = coef(m), Sigma = vcov(m), Terms = 2)
```

```
## Wald test:
## ----------
## 
## Chi-squared test:
## X2 = 139.6, df = 1, P(> X2) = 0.0
```

```
## odds ratios and 95% CI
exp(cbind(OR = coef(m), confint(m, level=0.99)))
```

```
## Waiting for profiling to be done...
```

```
##                    OR    0.5 %    99.5 %
## (Intercept) 0.2477844 0.195353 0.3125857
## D_Age       1.1565141 1.120747 1.1941555
```

### Create ROCR from data

```
pred.mtt = predict(m, type = "response") #repeat risk predictions from model m
rocr.pred.mtt = ROCR::prediction(pred.mtt, labels = ml$Any_Health_Problem) #ROCR prediction object
roc.perf.mtt = ROCR::performance(rocr.pred.mtt, measure = "tpr", x.measure = "fpr") # #ROCR performance object
plot(roc.perf.mtt, col = "blue")
abline(a = 0, b = 1, lty = 2) #diagonal for random assignment
```

### Report AUC from ROC for training and test data

```
  # Train AUC
auc <- ROCR::performance(rocr.pred.mtt, measure = "auc")
  auc <- auc@y.values[[1]]
  print(auc)
```

```
## [1] 0.6416895
```

### Calculate Nagelkerke R^2

```
NagelkerkeR2(m)
```

```
## $N
## [1] 2322
## 
## $R2
## [1] 0.08434176
```

### Check age is linear with logit of outcome

#### Note lack of linearity

```
ypred = predict(m)
res = residuals(m, type = 'deviance')
plot(ypred,res)
```

### Box Tidwell test to check that D\_Age is linearly associated with the logit of the outcome

#### suggests not linear

```
boxTidwell(ml$Health_Binary ~ ml$D_Age)
```

```
##  MLE of lambda Score Statistic (t) Pr(>|t|)   
##          2.472              3.1802 0.001491 **
## ---
## Signif. codes:  0 '***' 0.001 '**' 0.01 '*' 0.05 '.' 0.1 ' ' 1
## 
## iterations =  4
```

### check assumptions of model

#### Cook’s distance

```
plot(m, which = 4, id.n = 3)
```

#### Extract model results and display data for top 3 values using Cook’s distance

```
model.data <- augment(m) %>% 
  mutate(index = 1:n()) 
model.data %>% top_n(3, .cooksd)
```

#### plot standardised residuals

```
ggplot(model.data, aes(index, .std.resid)) + 
  geom_point(aes(color = Any_Health_Problem), alpha = .5) +
  theme_bw()
```

#### Filter potential influential data points with abs(.std.res) > 3:

```
model.data %>% 
  filter(abs(.std.resid) > 3)
```

## D\_AGE Binary logistic regression for HEALTH

As age not linearly associated with logit of outcome, add a natural
cubic spline

```
# fit binary logit model and store results 'm'
m <- glm(Any_Health_Problem ~ bs(D_Age,degree=1,df=2), data = ml,family = binomial)
# view a summary of the model
summary(m)
```

```
## 
## Call:
## glm(formula = Any_Health_Problem ~ bs(D_Age, degree = 1, df = 2), 
##     family = binomial, data = ml)
## 
## Coefficients:
##                                Estimate Std. Error z value Pr(>|z|)    
## (Intercept)                     -1.0510     0.1115  -9.427   <2e-16 ***
## bs(D_Age, degree = 1, df = 2)1   0.3624     0.1577   2.298   0.0216 *  
## bs(D_Age, degree = 1, df = 2)2   3.0129     0.2571  11.716   <2e-16 ***
## ---
## Signif. codes:  0 '***' 0.001 '**' 0.01 '*' 0.05 '.' 0.1 ' ' 1
## 
## (Dispersion parameter for binomial family taken to be 1)
## 
##     Null deviance: 3094.1  on 2321  degrees of freedom
## Residual deviance: 2939.1  on 2319  degrees of freedom
## AIC: 2945.1
## 
## Number of Fisher Scoring iterations: 4
```

```
# test model fit
with(m, null.deviance - deviance)
```

```
## [1] 155.0527
```

```
with(m, df.null - df.residual)
```

```
## [1] 2
```

```
with(m, pchisq(null.deviance - deviance, df.null - df.residual, lower.tail = FALSE))
```

```
## [1] 2.141614e-34
```

```
BIC(m)
```

```
## [1] 2962.323
```

```
# Hosmer-Lemeshow Goodness-of-Fit Test
hltest(m)
```

```
## 
##    The Hosmer-Lemeshow goodness-of-fit test
## 
##  Group Size Observed  Expected
##      1  150       40  38.85407
##      2  255       72  69.66111
##      3  256       71  73.67872
##      4  241       72  72.99672
##      5  242       72  77.05558
##      6  204       71  68.20655
##      7  192       64  72.52034
##      8  177       83  74.89288
##      9  277      134 136.49149
##     10  235      154 141.31316
##     11   93       60  67.32937
## 
##          Statistic =  9.93557 
## degrees of freedom =  9 
##            p-value =  0.35573
```

```
## CIs using profiled log-likelihood
confint(m, level=0.99)
```

```
## Waiting for profiling to be done...
```

```
##                                      0.5 %     99.5 %
## (Intercept)                    -1.34304602 -0.7680786
## bs(D_Age, degree = 1, df = 2)1 -0.04166619  0.7715202
## bs(D_Age, degree = 1, df = 2)2  2.35846443  3.6844748
```

```
## CIs using standard errors
confint.default(m, level=0.99)
```

```
##                                      0.5 %     99.5 %
## (Intercept)                    -1.33822216 -0.7638399
## bs(D_Age, degree = 1, df = 2)1 -0.04387407  0.7687475
## bs(D_Age, degree = 1, df = 2)2  2.35048172  3.6752211
```

```
# Wald test
wald.test(b = coef(m), Sigma = vcov(m), Terms = 2)
```

```
## Wald test:
## ----------
## 
## Chi-squared test:
## X2 = 5.3, df = 1, P(> X2) = 0.022
```

```
## odds ratios and 95% CI
exp(cbind(OR = coef(m), confint(m, level=0.99)))
```

```
## Waiting for profiling to be done...
```

```
##                                        OR      0.5 %     99.5 %
## (Intercept)                     0.3495771  0.2610493  0.4639036
## bs(D_Age, degree = 1, df = 2)1  1.4368263  0.9591899  2.1630521
## bs(D_Age, degree = 1, df = 2)2 20.3453303 10.5747008 39.8242009
```

```
## To see what knots were selected
attr(terms(m), "predvars")
```

```
## list(Any_Health_Problem, bs(D_Age, degree = 1L, knots = 6, Boundary.knots = c(1, 
## 20), intercept = FALSE))
```

### Create ROCR from data

```
pred.mtt = predict(m, type = "response") #repeat risk predictions from model m
rocr.pred.mtt = ROCR::prediction(pred.mtt, labels = ml$Any_Health_Problem) #ROCR prediction object
roc.perf.mtt = ROCR::performance(rocr.pred.mtt, measure = "tpr", x.measure = "fpr") # #ROCR performance object
plot(roc.perf.mtt, col = "blue")
abline(a = 0, b = 1, lty = 2) #diagonal for random assignment
```

### Report AUC from ROC for training and test data

```
  # Train AUC
auc <- ROCR::performance(rocr.pred.mtt, measure = "auc")
  auc <- auc@y.values[[1]]
  print(auc)
```

```
## [1] 0.6416895
```

### Calculate Nagelkerke R^2

```
NagelkerkeR2(m)
```

```
## $N
## [1] 2322
## 
## $R2
## [1] 0.08774204
```

### check assumptions of model

#### Cook’s distance

```
plot(m, which = 4, id.n = 3)
```

#### Extract model results and display data for top 3 values using Cook’s distance

```
model.data <- augment(m) %>% 
  mutate(index = 1:n()) 
model.data %>% top_n(3, .cooksd)
```

#### plot standardised residuals

```
ggplot(model.data, aes(index, .std.resid)) + 
  geom_point(aes(color = Any_Health_Problem), alpha = .5) +
  theme_bw()
```

#### Filter potential influential data points with abs(.std.res) > 3:

```
model.data %>% 
  filter(abs(.std.resid) > 3)
```

## D\_Age\_quant logistic regression for HEALTH

#### Note better model fit than D-Age

```
# fit binary logit model and store results 'm'
m <- glm(Any_Health_Problem ~ D_Age_quant, data = ml,family = binomial)
# view a summary of the model
summary(m)
```

```
## 
## Call:
## glm(formula = Any_Health_Problem ~ D_Age_quant, family = binomial, 
##     data = ml)
## 
## Coefficients:
##                     Estimate Std. Error z value Pr(>|z|)    
## (Intercept)         -0.96012    0.08693 -11.045  < 2e-16 ***
## D_Age_quant(3,5]     0.10394    0.13210   0.787   0.4314    
## D_Age_quant(5,7]     0.30088    0.13710   2.195   0.0282 *  
## D_Age_quant(7,9.4]   0.82241    0.14183   5.799 6.69e-09 ***
## D_Age_quant(9.4,20]  1.40907    0.12907  10.917  < 2e-16 ***
## ---
## Signif. codes:  0 '***' 0.001 '**' 0.01 '*' 0.05 '.' 0.1 ' ' 1
## 
## (Dispersion parameter for binomial family taken to be 1)
## 
##     Null deviance: 3094.1  on 2321  degrees of freedom
## Residual deviance: 2936.5  on 2317  degrees of freedom
## AIC: 2946.5
## 
## Number of Fisher Scoring iterations: 4
```

```
# test model fit
with(m, null.deviance - deviance)
```

```
## [1] 157.6016
```

```
with(m, df.null - df.residual)
```

```
## [1] 4
```

```
with(m, pchisq(null.deviance - deviance, df.null - df.residual, lower.tail = FALSE))
```

```
## [1] 4.778141e-33
```

```
BIC(m)
```

```
## [1] 2975.274
```

```
# Hosmer-Lemeshow Goodness-of-Fit Test
hltest(m, G=4)
```

```
## 
##    The Hosmer-Lemeshow goodness-of-fit test
## 
##  Group Size Observed Expected
##      1  661      183      183
##      2  483      144      144
##      3  396      135      135
##      4  320      149      149
##      5  462      282      282
## 
##          Statistic =  0 
## degrees of freedom =  3 
##            p-value =  1
```

```
## CIs using profiled log-likelihood
confint(m, level=0.99)
```

```
## Waiting for profiling to be done...
```

```
##                           0.5 %     99.5 %
## (Intercept)         -1.18802517 -0.7397007
## D_Age_quant(3,5]    -0.23755543  0.4436395
## D_Age_quant(5,7]    -0.05347926  0.6534905
## D_Age_quant(7,9.4]   0.45754271  1.1888749
## D_Age_quant(9.4,20]  1.07932524  1.7446466
```

```
## CIs using standard errors
confint.default(m, level=0.99)
```

```
##                           0.5 %     99.5 %
## (Intercept)         -1.18403690 -0.7362123
## D_Age_quant(3,5]    -0.23633315  0.4442087
## D_Age_quant(5,7]    -0.05225721  0.6540151
## D_Age_quant(7,9.4]   0.45707624  1.1877384
## D_Age_quant(9.4,20]  1.07662139  1.7415282
```

```
# Wald test
wald.test(b = coef(m), Sigma = vcov(m), Terms = 2)
```

```
## Wald test:
## ----------
## 
## Chi-squared test:
## X2 = 0.62, df = 1, P(> X2) = 0.43
```

```
## odds ratios and 95% CI
exp(cbind(OR = coef(m), confint(m, level=0.99)))
```

```
## Waiting for profiling to be done...
```

```
##                            OR     0.5 %    99.5 %
## (Intercept)         0.3828452 0.3048226 0.4772567
## D_Age_quant(3,5]    1.1095314 0.7885532 1.5583685
## D_Age_quant(5,7]    1.3510458 0.9479256 1.9222388
## D_Age_quant(7,9.4]  2.2759723 1.5801862 3.2833849
## D_Age_quant(9.4,20] 4.0921676 2.9426933 5.7238785
```

### Create ROCR from data

```
pred.mtt = predict(m, type = "response") #repeat risk predictions from model m
rocr.pred.mtt = ROCR::prediction(pred.mtt, labels = ml$Any_Health_Problem) #ROCR prediction object
roc.perf.mtt = ROCR::performance(rocr.pred.mtt, measure = "tpr", x.measure = "fpr") # #ROCR performance object
plot(roc.perf.mtt, col = "blue")
abline(a = 0, b = 1, lty = 2) #diagonal for random assignment
```

### Report AUC from ROC for training and test data

```
  # Train AUC
auc <- ROCR::performance(rocr.pred.mtt, measure = "auc")
  auc <- auc@y.values[[1]]
  print(auc)
```

```
## [1] 0.6393315
```

### Calculate Nagelkerke R^2

```
NagelkerkeR2(m)
```

```
## $N
## [1] 2322
## 
## $R2
## [1] 0.08913605
```

### check assumptions of model

#### Cook’s distance

```
plot(m, which = 4, id.n = 3)
```

#### Extract model results and display data for top 3 values using Cook’s distance

```
model.data <- augment(m) %>% 
  mutate(index = 1:n()) 
model.data %>% top_n(3, .cooksd)
```

#### plot standardised residuals

```
ggplot(model.data, aes(index, .std.resid)) + 
  geom_point(aes(color = Any_Health_Problem), alpha = .5) +
  theme_bw()
```

#### Filter potential influential data points with abs(.std.res) > 3:

```
model.data %>% 
  filter(abs(.std.resid) > 3)
```

## DOG SEX binary logistic regression for HEALTH

```
# fit binary logit model and store results 'm'
m <- glm(Any_Health_Problem ~ D_Sex, data = ml,family = binomial)
# view a summary of the model
summary(m)
```

```
## 
## Call:
## glm(formula = Any_Health_Problem ~ D_Sex, family = binomial, 
##     data = ml)
## 
## Coefficients:
##             Estimate Std. Error z value Pr(>|z|)    
## (Intercept) -0.48606    0.06223  -7.810 5.71e-15 ***
## D_SexMale    0.03007    0.08547   0.352    0.725    
## ---
## Signif. codes:  0 '***' 0.001 '**' 0.01 '*' 0.05 '.' 0.1 ' ' 1
## 
## (Dispersion parameter for binomial family taken to be 1)
## 
##     Null deviance: 3094.1  on 2321  degrees of freedom
## Residual deviance: 3094.0  on 2320  degrees of freedom
## AIC: 3098
## 
## Number of Fisher Scoring iterations: 4
```

```
# test model fit
with(m, null.deviance - deviance)
```

```
## [1] 0.1238193
```

```
with(m, df.null - df.residual)
```

```
## [1] 1
```

```
with(m, pchisq(null.deviance - deviance, df.null - df.residual, lower.tail = FALSE))
```

```
## [1] 0.7249285
```

```
BIC(m)
```

```
## [1] 3109.501
```

```
## CIs using profiled log-likelihood
confint(m, level=0.99)
```

```
## Waiting for profiling to be done...
```

```
##                  0.5 %     99.5 %
## (Intercept) -0.6474746 -0.3266933
## D_SexMale   -0.1900106  0.2504411
```

```
## CIs using standard errors
confint.default(m, level=0.99)
```

```
##                  0.5 %     99.5 %
## (Intercept) -0.6463639 -0.3257582
## D_SexMale   -0.1900872  0.2502337
```

```
# Wald test
wald.test(b = coef(m), Sigma = vcov(m), Terms = 2)
```

```
## Wald test:
## ----------
## 
## Chi-squared test:
## X2 = 0.12, df = 1, P(> X2) = 0.72
```

```
## odds ratios and 95% CI
exp(cbind(OR = coef(m), confint(m, level=0.99)))
```

```
## Waiting for profiling to be done...
```

```
##                    OR     0.5 %    99.5 %
## (Intercept) 0.6150442 0.5233658 0.7213049
## D_SexMale   1.0305300 0.8269504 1.2845919
```

### Create ROCR from data

```
pred.mtt = predict(m, type = "response") #repeat risk predictions from model m
rocr.pred.mtt = ROCR::prediction(pred.mtt, labels = ml$Any_Health_Problem) #ROCR prediction object
roc.perf.mtt = ROCR::performance(rocr.pred.mtt, measure = "tpr", x.measure = "fpr") # #ROCR performance object
plot(roc.perf.mtt, col = "blue")
abline(a = 0, b = 1, lty = 2) #diagonal for random assignment
```

### Report AUC from ROC for training and test data

```
  # Train AUC
auc <- ROCR::performance(rocr.pred.mtt, measure = "auc")
  auc <- auc@y.values[[1]]
  print(auc)
```

```
## [1] 0.5037462
```

### Calculate Nagelkerke R^2

```
NagelkerkeR2(m)
```

```
## $N
## [1] 2322
## 
## $R2
## [1] 7.243104e-05
```

### check assumptions of model

#### Cook’s distance

```
plot(m, which = 4, id.n = 3)
```

#### Extract model results and display data for top 3 values using Cook’s distance

```
model.data <- augment(m) %>% 
  mutate(index = 1:n()) 
model.data %>% top_n(3, .cooksd)
```

#### plot standardised residuals

```
ggplot(model.data, aes(index, .std.resid)) + 
  geom_point(aes(color = Any_Health_Problem), alpha = .5) +
  theme_bw()
```

#### Filter potential influential data points with abs(.std.res) > 3:

```
model.data %>% 
  filter(abs(.std.resid) > 3)
```

## DOG NEUTER binary logistic regression for HEALTH

```
# fit binary logit model and store results 'm'
m <- glm(Any_Health_Problem ~ D_Neuter, data = ml,family = binomial)
# view a summary of the model
summary(m)
```

```
## 
## Call:
## glm(formula = Any_Health_Problem ~ D_Neuter, family = binomial, 
##     data = ml)
## 
## Coefficients:
##                  Estimate Std. Error z value Pr(>|z|)    
## (Intercept)      -0.95768    0.09872  -9.701  < 2e-16 ***
## D_NeuterNeutered  0.61400    0.10964   5.600 2.14e-08 ***
## ---
## Signif. codes:  0 '***' 0.001 '**' 0.01 '*' 0.05 '.' 0.1 ' ' 1
## 
## (Dispersion parameter for binomial family taken to be 1)
## 
##     Null deviance: 3094.1  on 2321  degrees of freedom
## Residual deviance: 3061.1  on 2320  degrees of freedom
## AIC: 3065.1
## 
## Number of Fisher Scoring iterations: 4
```

```
# test model fit
with(m, null.deviance - deviance)
```

```
## [1] 33.00163
```

```
with(m, df.null - df.residual)
```

```
## [1] 1
```

```
with(m, pchisq(null.deviance - deviance, df.null - df.residual, lower.tail = FALSE))
```

```
## [1] 9.208158e-09
```

```
BIC(m)
```

```
## [1] 3076.624
```

```
## CIs using profiled log-likelihood
confint(m, level=0.99)
```

```
## Waiting for profiling to be done...
```

```
##                      0.5 %     99.5 %
## (Intercept)      -1.217130 -0.7078452
## D_NeuterNeutered  0.335112  0.9005377
```

```
## CIs using standard errors
confint.default(m, level=0.99)
```

```
##                      0.5 %     99.5 %
## (Intercept)      -1.211953 -0.7033992
## D_NeuterNeutered  0.331589  0.8964135
```

```
# Wald test
wald.test(b = coef(m), Sigma = vcov(m), Terms = 2)
```

```
## Wald test:
## ----------
## 
## Chi-squared test:
## X2 = 31.4, df = 1, P(> X2) = 2.1e-08
```

```
## odds ratios and 95% CI
exp(cbind(OR = coef(m), confint(m, level=0.99)))
```

```
## Waiting for profiling to be done...
```

```
##                         OR     0.5 %    99.5 %
## (Intercept)      0.3837838 0.2960786 0.4927047
## D_NeuterNeutered 1.8478102 1.3980970 2.4609259
```

### Create ROCR from data

```
pred.mtt = predict(m, type = "response") #repeat risk predictions from model m
rocr.pred.mtt = ROCR::prediction(pred.mtt, labels = ml$Any_Health_Problem) #ROCR prediction object
roc.perf.mtt = ROCR::performance(rocr.pred.mtt, measure = "tpr", x.measure = "fpr") # #ROCR performance object
plot(roc.perf.mtt, col = "blue")
abline(a = 0, b = 1, lty = 2) #diagonal for random assignment
```

### Report AUC from ROC for training and test data

```
  # Train AUC
auc <- ROCR::performance(rocr.pred.mtt, measure = "auc")
  auc <- auc@y.values[[1]]
  print(auc)
```

```
## [1] 0.5499539
```

### Calculate Nagelkerke R^2

```
NagelkerkeR2(m)
```

```
## $N
## [1] 2322
## 
## $R2
## [1] 0.01916906
```

### check assumptions of model

#### Cook’s distance

```
plot(m, which = 4, id.n = 3)
```

#### Extract model results and display data for top 3 values using Cook’s distance

```
model.data <- augment(m) %>% 
  mutate(index = 1:n()) 
model.data %>% top_n(3, .cooksd)
```

#### plot standardised residuals

```
ggplot(model.data, aes(index, .std.resid)) + 
  geom_point(aes(color = Any_Health_Problem), alpha = .5) +
  theme_bw()
```

#### Filter potential influential data points with abs(.std.res) > 3:

```
model.data %>% 
  filter(abs(.std.resid) > 3)
```

## D\_Sex + D\_Neuter Binary logistic regression for HEALTH

```
# fit binary logit model and store results 'm'
m <- glm(Any_Health_Problem ~ D_Sex + D_Neuter, data = ml,family = binomial)
# view a summary of the model
summary(m)
```

```
## 
## Call:
## glm(formula = Any_Health_Problem ~ D_Sex + D_Neuter, family = binomial, 
##     data = ml)
## 
## Coefficients:
##                  Estimate Std. Error z value Pr(>|z|)    
## (Intercept)      -1.00022    0.11194  -8.935  < 2e-16 ***
## D_SexMale         0.07006    0.08634   0.811    0.417    
## D_NeuterNeutered  0.62091    0.11000   5.645 1.65e-08 ***
## ---
## Signif. codes:  0 '***' 0.001 '**' 0.01 '*' 0.05 '.' 0.1 ' ' 1
## 
## (Dispersion parameter for binomial family taken to be 1)
## 
##     Null deviance: 3094.1  on 2321  degrees of freedom
## Residual deviance: 3060.5  on 2319  degrees of freedom
## AIC: 3066.5
## 
## Number of Fisher Scoring iterations: 4
```

```
# test model fit
with(m, null.deviance - deviance)
```

```
## [1] 33.66045
```

```
with(m, df.null - df.residual)
```

```
## [1] 2
```

```
with(m, pchisq(null.deviance - deviance, df.null - df.residual, lower.tail = FALSE))
```

```
## [1] 4.905979e-08
```

```
BIC(m)
```

```
## [1] 3083.715
```

```
# Hosmer-Lemeshow Goodness-of-Fit Test
hltest(m)
```

```
## 
##    The Hosmer-Lemeshow goodness-of-fit test
## 
##  Group Size Observed  Expected
##      1  203       54  54.58641
##      2  309       88  87.41359
##      3  892      363 362.41359
##      4  918      388 388.58641
## 
##          Statistic =  0.01724 
## degrees of freedom =  2 
##            p-value =  0.99142
```

```
## CIs using profiled log-likelihood
confint(m, level=0.99)
```

```
## Waiting for profiling to be done...
```

```
##                       0.5 %     99.5 %
## (Intercept)      -1.2933079 -0.7160086
## D_SexMale        -0.1521601  0.2927547
## D_NeuterNeutered  0.3410984  0.9083689
```

```
## CIs using standard errors
confint.default(m, level=0.99)
```

```
##                       0.5 %     99.5 %
## (Intercept)      -1.2885499 -0.7118858
## D_SexMale        -0.1523330  0.2924448
## D_NeuterNeutered  0.3375717  0.9042434
```

```
# Wald test
wald.test(b = coef(m), Sigma = vcov(m), Terms = 2)
```

```
## Wald test:
## ----------
## 
## Chi-squared test:
## X2 = 0.66, df = 1, P(> X2) = 0.42
```

```
## odds ratios and 95% CI
exp(cbind(OR = coef(m), confint(m, level=0.99)))
```

```
## Waiting for profiling to be done...
```

```
##                         OR     0.5 %   99.5 %
## (Intercept)      0.3677993 0.2743617 0.488699
## D_SexMale        1.0725682 0.8588508 1.340114
## D_NeuterNeutered 1.8606159 1.4064917 2.480274
```

### Create ROCR from data

```
pred.mtt = predict(m, type = "response") #repeat risk predictions from model m
rocr.pred.mtt = ROCR::prediction(pred.mtt, labels = ml$Any_Health_Problem) #ROCR prediction object
roc.perf.mtt = ROCR::performance(rocr.pred.mtt, measure = "tpr", x.measure = "fpr") # #ROCR performance object
plot(roc.perf.mtt, col = "blue")
abline(a = 0, b = 1, lty = 2) #diagonal for random assignment
```

### Report AUC from ROC for training and test data

```
  # Train AUC
auc <- ROCR::performance(rocr.pred.mtt, measure = "auc")
  auc <- auc@y.values[[1]]
  print(auc)
```

```
## [1] 0.555455
```

### Calculate Nagelkerke R^2

```
NagelkerkeR2(m)
```

```
## $N
## [1] 2322
## 
## $R2
## [1] 0.01954897
```

### check assumptions of model

#### Cook’s distance

```
plot(m, which = 4, id.n = 3)
```

#### Extract model results and display data for top 3 values using Cook’s distance

```
model.data <- augment(m) %>% 
  mutate(index = 1:n()) 
model.data %>% top_n(3, .cooksd)
```

#### plot standardised residuals

```
ggplot(model.data, aes(index, .std.resid)) + 
  geom_point(aes(color = Any_Health_Problem), alpha = .5) +
  theme_bw()
```

#### Filter potential influential data points with abs(.std.res) > 3:

```
model.data %>% 
  filter(abs(.std.resid) > 3)
```

#### check for multicollinearity

```
car::vif(m)
```

```
##    D_Sex D_Neuter 
## 1.006304 1.006304
```

## D\_Sex \* D\_Neuter Binary logistic regression for HEALTH

```
# fit binary logit model and store results 'm'
m <- glm(Any_Health_Problem ~ D_Sex*D_Neuter, data = ml,family = binomial)
# view a summary of the model
summary(m)
```

```
## 
## Call:
## glm(formula = Any_Health_Problem ~ D_Sex * D_Neuter, family = binomial, 
##     data = ml)
## 
## Coefficients:
##                            Estimate Std. Error z value Pr(>|z|)    
## (Intercept)                -1.01496    0.15884  -6.390 1.66e-10 ***
## D_SexMale                   0.09414    0.20278   0.464 0.642478    
## D_NeuterNeutered            0.63838    0.17284   3.693 0.000221 ***
## D_SexMale:D_NeuterNeutered -0.02942    0.22412  -0.131 0.895552    
## ---
## Signif. codes:  0 '***' 0.001 '**' 0.01 '*' 0.05 '.' 0.1 ' ' 1
## 
## (Dispersion parameter for binomial family taken to be 1)
## 
##     Null deviance: 3094.1  on 2321  degrees of freedom
## Residual deviance: 3060.4  on 2318  degrees of freedom
## AIC: 3068.4
## 
## Number of Fisher Scoring iterations: 4
```

```
# test model fit
with(m, null.deviance - deviance)
```

```
## [1] 33.6777
```

```
with(m, df.null - df.residual)
```

```
## [1] 3
```

```
with(m, pchisq(null.deviance - deviance, df.null - df.residual, lower.tail = FALSE))
```

```
## [1] 2.317163e-07
```

```
BIC(m)
```

```
## [1] 3091.448
```

```
# Hosmer-Lemeshow Goodness-of-Fit Test
hltest(m)
```

```
## 
##    The Hosmer-Lemeshow goodness-of-fit test
## 
##  Group Size Observed Expected
##      1  203       54       54
##      2  309       88       88
##      3  892      363      363
##      4  918      388      388
## 
##          Statistic =  0 
## degrees of freedom =  2 
##            p-value =  1
```

```
## CIs using profiled log-likelihood
confint(m, level=0.99)
```

```
## Waiting for profiling to be done...
```

```
##                                 0.5 %     99.5 %
## (Intercept)                -1.4387518 -0.6174064
## D_SexMale                  -0.4243481  0.6229149
## D_NeuterNeutered            0.2027996  1.0958625
## D_SexMale:D_NeuterNeutered -0.6118949  0.5446604
```

```
## CIs using standard errors
confint.default(m, level=0.99)
```

```
##                                 0.5 %     99.5 %
## (Intercept)                -1.4241049 -0.6058196
## D_SexMale                  -0.4281818  0.6164545
## D_NeuterNeutered            0.1931599  1.0835934
## D_SexMale:D_NeuterNeutered -0.6067053  0.5478604
```

```
# Wald test
wald.test(b = coef(m), Sigma = vcov(m), Terms = 2)
```

```
## Wald test:
## ----------
## 
## Chi-squared test:
## X2 = 0.22, df = 1, P(> X2) = 0.64
```

```
## odds ratios and 95% CI
exp(cbind(OR = coef(m), confint(m, level=0.99)))
```

```
## Waiting for profiling to be done...
```

```
##                                   OR     0.5 %    99.5 %
## (Intercept)                0.3624161 0.2372237 0.5393415
## D_SexMale                  1.0987096 0.6541961 1.8643546
## D_NeuterNeutered           1.8934047 1.2248269 2.9917621
## D_SexMale:D_NeuterNeutered 0.9710062 0.5423222 1.7240227
```

### Create ROCR from data

```
pred.mtt = predict(m, type = "response") #repeat risk predictions from model m
rocr.pred.mtt = ROCR::prediction(pred.mtt, labels = ml$Any_Health_Problem) #ROCR prediction object
roc.perf.mtt = ROCR::performance(rocr.pred.mtt, measure = "tpr", x.measure = "fpr") # #ROCR performance object
plot(roc.perf.mtt, col = "blue")
abline(a = 0, b = 1, lty = 2) #diagonal for random assignment
```

### Report AUC from ROC for training and test data

```
  # Train AUC
auc <- ROCR::performance(rocr.pred.mtt, measure = "auc")
  auc <- auc@y.values[[1]]
  print(auc)
```

```
## [1] 0.555455
```

### Calculate Nagelkerke R^2

```
NagelkerkeR2(m)
```

```
## $N
## [1] 2322
## 
## $R2
## [1] 0.01955891
```

### check assumptions of model

#### Cook’s distance

```
plot(m, which = 4, id.n = 3)
```

#### Extract model results and display data for top 3 values using Cook’s distance

```
model.data <- augment(m) %>% 
  mutate(index = 1:n()) 
model.data %>% top_n(3, .cooksd)
```

#### plot standardised residuals

```
ggplot(model.data, aes(index, .std.resid)) + 
  geom_point(aes(color = Any_Health_Problem), alpha = .5) +
  theme_bw()
```

#### Filter potential influential data points with abs(.std.res) > 3:

```
model.data %>% 
  filter(abs(.std.resid) > 3)
```

#### check for multicollinearity

```
car::vif(m)
```

```
## there are higher-order terms (interactions) in this model
## consider setting type = 'predictor'; see ?vif
```

```
##          D_Sex       D_Neuter D_Sex:D_Neuter 
##       5.550631       2.484316       6.600013
```

# CHECK EFFECT OF DOG HEALTH CHARACTERISTICS - simple binary regression

## THERAPEUTIC FOOD Binary logistic regression for HEALTH

```
# fit binary logit model and store results 'm'
m <- glm(Any_Health_Problem ~ Therapeutic_Food, data = ml,family = binomial)
# view a summary of the model
summary(m)
```

```
## 
## Call:
## glm(formula = Any_Health_Problem ~ Therapeutic_Food, family = binomial, 
##     data = ml)
## 
## Coefficients:
##                     Estimate Std. Error z value Pr(>|z|)    
## (Intercept)         -0.55962    0.04421 -12.658  < 2e-16 ***
## Therapeutic_FoodYes  1.95721    0.24217   8.082 6.37e-16 ***
## ---
## Signif. codes:  0 '***' 0.001 '**' 0.01 '*' 0.05 '.' 0.1 ' ' 1
## 
## (Dispersion parameter for binomial family taken to be 1)
## 
##     Null deviance: 3094.1  on 2321  degrees of freedom
## Residual deviance: 3009.1  on 2320  degrees of freedom
## AIC: 3013.1
## 
## Number of Fisher Scoring iterations: 4
```

```
# test model fit
with(m, null.deviance - deviance)
```

```
## [1] 85.0518
```

```
with(m, df.null - df.residual)
```

```
## [1] 1
```

```
with(m, pchisq(null.deviance - deviance, df.null - df.residual, lower.tail = FALSE))
```

```
## [1] 2.9065e-20
```

```
BIC(m)
```

```
## [1] 3024.573
```

```
## CIs using profiled log-likelihood
confint(m, level=0.99)
```

```
## Waiting for profiling to be done...
```

```
##                         0.5 %     99.5 %
## (Intercept)         -0.674115 -0.4462975
## Therapeutic_FoodYes  1.365113  2.6232872
```

```
## CIs using standard errors
confint.default(m, level=0.99)
```

```
##                          0.5 %     99.5 %
## (Intercept)         -0.6734929 -0.4457387
## Therapeutic_FoodYes  1.3334281  2.5809913
```

```
# Wald test
wald.test(b = coef(m), Sigma = vcov(m), Terms = 2)
```

```
## Wald test:
## ----------
## 
## Chi-squared test:
## X2 = 65.3, df = 1, P(> X2) = 6.7e-16
```

```
## odds ratios and 95% CI
exp(cbind(OR = coef(m), confint(m, level=0.99)))
```

```
## Waiting for profiling to be done...
```

```
##                            OR     0.5 %     99.5 %
## (Intercept)         0.5714286 0.5096072  0.6399934
## Therapeutic_FoodYes 7.0795455 3.9161644 13.7809496
```

### Create ROCR from data

```
pred.mtt = predict(m, type = "response") #repeat risk predictions from model m
rocr.pred.mtt = ROCR::prediction(pred.mtt, labels = ml$Any_Health_Problem) #ROCR prediction object
roc.perf.mtt = ROCR::performance(rocr.pred.mtt, measure = "tpr", x.measure = "fpr") # #ROCR performance object
plot(roc.perf.mtt, col = "blue")
abline(a = 0, b = 1, lty = 2) #diagonal for random assignment
```

### Report AUC from ROC for training and test data

```
  # Train AUC
auc <- ROCR::performance(rocr.pred.mtt, measure = "auc")
  auc <- auc@y.values[[1]]
  print(auc)
```

```
## [1] 0.5421343
```

### Calculate Nagelkerke R^2

```
NagelkerkeR2(m)
```

```
## $N
## [1] 2322
## 
## $R2
## [1] 0.0488542
```

### check assumptions of model

#### Cook’s distance

```
plot(m, which = 4, id.n = 3)
```

#### Extract model results and display data for top 3 values using Cook’s distance

```
model.data <- augment(m) %>% 
  mutate(index = 1:n()) 
model.data %>% top_n(3, .cooksd)
```

#### plot standardised residuals

```
ggplot(model.data, aes(index, .std.resid)) + 
  geom_point(aes(color = Any_Health_Problem), alpha = .5) +
  theme_bw()
```

#### Filter potential influential data points with abs(.std.res) > 3:

```
model.data %>% 
  filter(abs(.std.resid) > 3)
```

## VISITS Binary logistic regression for HEALTH

```
# fit binary logit model and store results 'm'
m <- glm(Any_Health_Problem ~ Visits, data = ml,family = binomial)
# view a summary of the model
summary(m)
```

```
## 
## Call:
## glm(formula = Any_Health_Problem ~ Visits, family = binomial, 
##     data = ml)
## 
## Coefficients:
##             Estimate Std. Error z value Pr(>|z|)    
## (Intercept)  -1.7385     0.1388 -12.522  < 2e-16 ***
## Visits1       0.5511     0.1595   3.456 0.000548 ***
## Visits2       1.8334     0.1649  11.117  < 2e-16 ***
## Visits3       2.1648     0.2005  10.797  < 2e-16 ***
## Visits3<      2.9891     0.1961  15.239  < 2e-16 ***
## ---
## Signif. codes:  0 '***' 0.001 '**' 0.01 '*' 0.05 '.' 0.1 ' ' 1
## 
## (Dispersion parameter for binomial family taken to be 1)
## 
##     Null deviance: 3094.1  on 2321  degrees of freedom
## Residual deviance: 2618.5  on 2317  degrees of freedom
## AIC: 2628.5
## 
## Number of Fisher Scoring iterations: 4
```

```
# test model fit
with(m, null.deviance - deviance)
```

```
## [1] 475.6601
```

```
with(m, df.null - df.residual)
```

```
## [1] 4
```

```
with(m, pchisq(null.deviance - deviance, df.null - df.residual, lower.tail = FALSE))
```

```
## [1] 1.229755e-101
```

```
BIC(m)
```

```
## [1] 2657.216
```

```
# Hosmer-Lemeshow Goodness-of-Fit Test
hltest(m)
```

```
## 
##    The Hosmer-Lemeshow goodness-of-fit test
## 
##  Group Size Observed Expected
##      1  408       61       61
##      2  907      212      212
##      3  506      265      265
##      4  200      121      121
##      5  301      234      234
## 
##          Statistic =  0 
## degrees of freedom =  3 
##            p-value =  1
```

```
## CIs using profiled log-likelihood
confint(m, level=0.99)
```

```
## Waiting for profiling to be done...
```

```
##                  0.5 %     99.5 %
## (Intercept) -2.1121695 -1.3947188
## Visits1      0.1498021  0.9734713
## Visits2      1.4184435  2.2698654
## Visits3      1.6571940  2.6918309
## Visits3<     2.4964615  3.5084900
```

```
## CIs using standard errors
confint.default(m, level=0.99)
```

```
##                  0.5 %     99.5 %
## (Intercept) -2.0960674 -1.3808345
## Visits1      0.1403544  0.9618963
## Visits2      1.4085808  2.2581868
## Visits3      1.6483544  2.6812328
## Visits3<     2.4838350  3.4943238
```

```
# Wald test
wald.test(b = coef(m), Sigma = vcov(m), Terms = 2)
```

```
## Wald test:
## ----------
## 
## Chi-squared test:
## X2 = 11.9, df = 1, P(> X2) = 0.00055
```

```
## odds ratios and 95% CI
exp(cbind(OR = coef(m), confint(m, level=0.99)))
```

```
## Waiting for profiling to be done...
```

```
##                     OR      0.5 %     99.5 %
## (Intercept)  0.1757925  0.1209752  0.2479027
## Visits1      1.7352046  1.1616043  2.6471176
## Visits2      6.2550167  4.1306862  9.6780981
## Visits3      8.7128035  5.2445741 14.7586731
## Visits3<    19.8673844 12.1394619 33.3977987
```

### Create ROCR from data

```
pred.mtt = predict(m, type = "response") #repeat risk predictions from model m
rocr.pred.mtt = ROCR::prediction(pred.mtt, labels = ml$Any_Health_Problem) #ROCR prediction object
roc.perf.mtt = ROCR::performance(rocr.pred.mtt, measure = "tpr", x.measure = "fpr") # #ROCR performance object
plot(roc.perf.mtt, col = "blue")
abline(a = 0, b = 1, lty = 2) #diagonal for random assignment
```

### Report AUC from ROC for training and test data

```
  # Train AUC
auc <- ROCR::performance(rocr.pred.mtt, measure = "auc")
  auc <- auc@y.values[[1]]
  print(auc)
```

```
## [1] 0.746377
```

### Calculate Nagelkerke R^2

```
NagelkerkeR2(m)
```

```
## $N
## [1] 2322
## 
## $R2
## [1] 0.2516061
```

### check assumptions of model

#### Cook’s distance

```
plot(m, which = 4, id.n = 3)
```

#### Extract model results and display data for top 3 values using Cook’s distance

```
model.data <- augment(m) %>% 
  mutate(index = 1:n()) 
model.data %>% top_n(3, .cooksd)
```

#### plot standardised residuals

```
ggplot(model.data, aes(index, .std.resid)) + 
  geom_point(aes(color = Any_Health_Problem), alpha = .5) +
  theme_bw()
```

#### Filter potential influential data points with abs(.std.res) > 3:

```
model.data %>% 
  filter(abs(.std.resid) > 3)
```

## VISITS2 Binary logistic regression for HEALTH

#### Note Visits better fit than Visits2

```
# fit binary logit model and store results 'm'
m <- glm(Any_Health_Problem ~ Visits2, data = ml,family = binomial)
# view a summary of the model
summary(m)
```

```
## 
## Call:
## glm(formula = Any_Health_Problem ~ Visits2, family = binomial, 
##     data = ml)
## 
## Coefficients:
##             Estimate Std. Error z value Pr(>|z|)    
## (Intercept)  -1.7385     0.1388 -12.522  < 2e-16 ***
## Visits21v     0.5511     0.1595   3.456 0.000548 ***
## Visits22v     1.8334     0.1649  11.117  < 2e-16 ***
## Visits23v     2.6270     0.1701  15.442  < 2e-16 ***
## ---
## Signif. codes:  0 '***' 0.001 '**' 0.01 '*' 0.05 '.' 0.1 ' ' 1
## 
## (Dispersion parameter for binomial family taken to be 1)
## 
##     Null deviance: 3094.1  on 2321  degrees of freedom
## Residual deviance: 2635.6  on 2318  degrees of freedom
## AIC: 2643.6
## 
## Number of Fisher Scoring iterations: 4
```

```
# test model fit
with(m, null.deviance - deviance)
```

```
## [1] 458.5717
```

```
with(m, df.null - df.residual)
```

```
## [1] 3
```

```
with(m, pchisq(null.deviance - deviance, df.null - df.residual, lower.tail = FALSE))
```

```
## [1] 4.529119e-99
```

```
BIC(m)
```

```
## [1] 2666.554
```

```
# Hosmer-Lemeshow Goodness-of-Fit Test
hltest(m)
```

```
## 
##    The Hosmer-Lemeshow goodness-of-fit test
## 
##  Group Size Observed Expected
##      1  408       61       61
##      2  907      212      212
##      3  506      265      265
##      4  501      355      355
## 
##          Statistic =  0 
## degrees of freedom =  2 
##            p-value =  1
```

```
## CIs using profiled log-likelihood
confint(m, level=0.99)
```

```
## Waiting for profiling to be done...
```

```
##                  0.5 %     99.5 %
## (Intercept) -2.1121695 -1.3947188
## Visits21v    0.1498021  0.9734713
## Visits22v    1.4184435  2.2698654
## Visits23v    2.1994648  3.0774440
```

```
## CIs using standard errors
confint.default(m, level=0.99)
```

```
##                  0.5 %     99.5 %
## (Intercept) -2.0960674 -1.3808345
## Visits21v    0.1403544  0.9618963
## Visits22v    1.4085808  2.2581868
## Visits23v    2.1887569  3.0651673
```

```
# Wald test
wald.test(b = coef(m), Sigma = vcov(m), Terms = 2)
```

```
## Wald test:
## ----------
## 
## Chi-squared test:
## X2 = 11.9, df = 1, P(> X2) = 0.00055
```

```
## odds ratios and 95% CI
exp(cbind(OR = coef(m), confint(m, level=0.99)))
```

```
## Waiting for profiling to be done...
```

```
##                     OR     0.5 %     99.5 %
## (Intercept)  0.1757925 0.1209752  0.2479027
## Visits21v    1.7352046 1.1616043  2.6471176
## Visits22v    6.2550167 4.1306862  9.6780981
## Visits23v   13.8316865 9.0201845 21.7028597
```

### Create ROCR from data

```
pred.mtt = predict(m, type = "response") #repeat risk predictions from model m
rocr.pred.mtt = ROCR::prediction(pred.mtt, labels = ml$Any_Health_Problem) #ROCR prediction object
roc.perf.mtt = ROCR::performance(rocr.pred.mtt, measure = "tpr", x.measure = "fpr") # #ROCR performance object
plot(roc.perf.mtt, col = "blue")
abline(a = 0, b = 1, lty = 2) #diagonal for random assignment
```

### Report AUC from ROC for training and test data

```
  # Train AUC
auc <- ROCR::performance(rocr.pred.mtt, measure = "auc")
  auc <- auc@y.values[[1]]
  print(auc)
```

```
## [1] 0.7423103
```

### Calculate Nagelkerke R^2

```
NagelkerkeR2(m)
```

```
## $N
## [1] 2322
## 
## $R2
## [1] 0.2434312
```

### check assumptions of model

#### Cook’s distance

```
plot(m, which = 4, id.n = 3)
```

#### Extract model results and display data for top 3 values using Cook’s distance

```
model.data <- augment(m) %>% 
  mutate(index = 1:n()) 
model.data %>% top_n(3, .cooksd)
```

#### plot standardised residuals

```
ggplot(model.data, aes(index, .std.resid)) + 
  geom_point(aes(color = Any_Health_Problem), alpha = .5) +
  theme_bw()
```

#### Filter potential influential data points with abs(.std.res) > 3:

```
model.data %>% 
  filter(abs(.std.resid) > 3)
```

## MEDS Binary logistic regression for HEALTH

```
# fit binary logit model and store results 'm'
m <- glm(Any_Health_Problem ~ Meds, data = ml,family = binomial)
# view a summary of the model
summary(m)
```

```
## 
## Call:
## glm(formula = Any_Health_Problem ~ Meds, family = binomial, data = ml)
## 
## Coefficients:
##             Estimate Std. Error z value Pr(>|z|)    
## (Intercept) -1.52852    0.07019  -21.78   <2e-16 ***
## MedsYes      2.32944    0.09961   23.39   <2e-16 ***
## ---
## Signif. codes:  0 '***' 0.001 '**' 0.01 '*' 0.05 '.' 0.1 ' ' 1
## 
## (Dispersion parameter for binomial family taken to be 1)
## 
##     Null deviance: 3094.1  on 2321  degrees of freedom
## Residual deviance: 2457.9  on 2320  degrees of freedom
## AIC: 2461.9
## 
## Number of Fisher Scoring iterations: 4
```

```
# test model fit
with(m, null.deviance - deviance)
```

```
## [1] 636.273
```

```
with(m, df.null - df.residual)
```

```
## [1] 1
```

```
with(m, pchisq(null.deviance - deviance, df.null - df.residual, lower.tail = FALSE))
```

```
## [1] 2.160321e-140
```

```
BIC(m)
```

```
## [1] 2473.352
```

```
# Hosmer-Lemeshow Goodness-of-Fit Test
## CIs using profiled log-likelihood
confint(m, level=0.99)
```

```
## Waiting for profiling to be done...
```

```
##                 0.5 %    99.5 %
## (Intercept) -1.712966 -1.351095
## MedsYes      2.075553  2.588927
```

```
## CIs using standard errors
confint.default(m, level=0.99)
```

```
##                 0.5 %    99.5 %
## (Intercept) -1.709313 -1.347722
## MedsYes      2.072849  2.586023
```

```
# Wald test
wald.test(b = coef(m), Sigma = vcov(m), Terms = 2)
```

```
## Wald test:
## ----------
## 
## Chi-squared test:
## X2 = 546.8, df = 1, P(> X2) = 0.0
```

```
## odds ratios and 95% CI
exp(cbind(OR = coef(m), confint(m, level=0.99)))
```

```
## Waiting for profiling to be done...
```

```
##                     OR     0.5 %     99.5 %
## (Intercept)  0.2168569 0.1803301  0.2589566
## MedsYes     10.2721485 7.9689512 13.3154741
```

### Create ROCR from data

```
pred.mtt = predict(m, type = "response") #repeat risk predictions from model m
rocr.pred.mtt = ROCR::prediction(pred.mtt, labels = ml$Any_Health_Problem) #ROCR prediction object
roc.perf.mtt = ROCR::performance(rocr.pred.mtt, measure = "tpr", x.measure = "fpr") # #ROCR performance object
plot(roc.perf.mtt, col = "blue")
abline(a = 0, b = 1, lty = 2) #diagonal for random assignment
```

### Report AUC from ROC for training and test data

```
  # Train AUC
auc <- ROCR::performance(rocr.pred.mtt, measure = "auc")
  auc <- auc@y.values[[1]]
  print(auc)
```

```
## [1] 0.7602326
```

### Calculate Nagelkerke R^2

```
NagelkerkeR2(m)
```

```
## $N
## [1] 2322
## 
## $R2
## [1] 0.3255718
```

### check assumptions of model

#### Cook’s distance

```
plot(m, which = 4, id.n = 3)
```

#### Extract model results and display data for top 3 values using Cook’s distance

```
model.data <- augment(m) %>% 
  mutate(index = 1:n()) 
model.data %>% top_n(3, .cooksd)
```

#### plot standardised residuals

```
ggplot(model.data, aes(index, .std.resid)) + 
  geom_point(aes(color = Any_Health_Problem), alpha = .5) +
  theme_bw()
```

#### Filter potential influential data points with abs(.std.res) > 3:

```
model.data %>% 
  filter(abs(.std.resid) > 3)
```
