# Supplementary material for: Variables associated with owner perceptions of the health of their dog: Further analysis of data from a large international survey
Source: PLoS One. 2024 May 15;19(5):e0280173. doi: 10.1371/journal.pone.0280173 (PMC11095744; doi:10.1371/journal.pone.0280173)
Supplement: S9 File — (HTML) [file pone.0280173.s023.html]

 

 

 

 
 
 


 

 

 R stats for binary logistic regression on SIGNIFICANT or SERIOUS illness 

 
 
 
 
 
 
 
 
 
 
 
 
 

 

 
 


 


 

 

 


 

 


 


 


 R stats for binary logistic regression on
SIGNIFICANT or SERIOUS illness 
 Alex German 
 7 December 2023 

 


 
 Create data frame for analysis 
 
 NB need to run “Read_data_101.Rtm” first to create dataset 
 
 
 
 LET’S DO SOME INTIAL TABULATION AND CHI SQUARE TESTS 
 
 Tabulate D_Sex + D_Neuter + D_Diet 
  ## three way cross tabs (xtabs) and flatten the table
ftable(xtabs(~ D_Sex + D_Neuter + D_Diet, data = ml))  
  ##                 D_Diet Meat-based – conventional Meat-based – raw Vegan (consuming no animal products) Vegetarian (including eggs or milk, but not meat)
## D_Sex  D_Neuter                                                                                                                                         
## Female Intact                                 79               92                                   20                                                 1
##        Neutered                              465              239                                  133                                                13
## Male   Intact                                139              131                                   21                                                 3
##        Neutered                              469              270                                  121                                                15  
 
 
 Tabulate D_Neuter + D_Diet 
  ## two way cross tabs (xtabs) and flatten the table
ftable(xtabs(~ D_Neuter + D_Diet, data = ml))  
  ##          D_Diet Meat-based – conventional Meat-based – raw Vegan (consuming no animal products) Vegetarian (including eggs or milk, but not meat)
## D_Neuter                                                                                                                                         
## Intact                                218              223                                   41                                                 4
## Neutered                              934              509                                  254                                                28  
 
 
 Tabulate C_Diet_Vegan + D_Diet_Vegan 
  ## two way cross tabs (xtabs) and flatten the table
ftable(xtabs(~ C_Diet_Vegan + D_Diet_Vegan, data = ml))  
  ##              D_Diet_Vegan   No  Yes
## C_Diet_Vegan                       
## No                        1696   22
## Yes                        220  273  
 
 
 
 Tabulate C_Diet_Vegan_Veggie + D_Diet_Vegan_Veggie 
  ## two way cross tabs (xtabs) and flatten the table
ftable(xtabs(~ C_Diet_Vegan_Veggie + D_Diet_Vegan_Veggie, data = ml))  
  ##                     D_Diet_Vegan_Veggie   No  Yes
## C_Diet_Vegan_Veggie                              
## No                                      1473   24
## Yes                                      411  303  
 
 Tabulate D_Sex, D_Neuter and D_Raw 
  ## three way cross tabs (xtabs) and flatten the table
ftable(xtabs(~ D_Sex + D_Neuter + D_Diet_Raw, data = ml))  
  ##                 D_Diet_Raw  No Yes
## D_Sex  D_Neuter                   
## Female Intact              100  92
##        Neutered            611 239
## Male   Intact              163 131
##        Neutered            605 270  
 
 
 Tabulate D_Neuter and D_Raw 
  ## two way cross tabs (xtabs) and flatten the table
ftable(xtabs(~ D_Neuter + D_Diet_Raw, data = ml))  
  ##          D_Diet_Raw   No  Yes
## D_Neuter                     
## Intact               263  223
## Neutered            1216  509  
 
 
 Chi squared test of C_VEGAN vs D-VEGAN 
  table(ml$C_Diet_Vegan, ml$D_Diet_Vegan)  
  ##      
##         No  Yes
##   No  1696   22
##   Yes  220  273  
  chisq.test(ml$C_Diet_Vegan, ml$D_Diet_Vegan, correct=FALSE)  
  ## 
##  Pearson's Chi-squared test
## 
## data:  ml$C_Diet_Vegan and ml$D_Diet_Vegan
## X-squared = 969.51, df = 1, p-value < 2.2e-16  
 
 
 Chi squared test of NEUTERED vs D-RAW 
  table(ml$D_Neuter, ml$D_Diet_Raw)  
  ##           
##              No  Yes
##   Intact    263  223
##   Neutered 1216  509  
  chisq.test(ml$D_Neuter, ml$D_Diet_Raw, correct=FALSE)  
  ## 
##  Pearson's Chi-squared test
## 
## data:  ml$D_Neuter and ml$D_Diet_Raw
## X-squared = 45.923, df = 1, p-value = 1.23e-11  
 
 
 Tabulate Health_Binary and Income 
  ## three way cross tabs (xtabs) and flatten the table
ftable(xtabs(~ Health_Binary + Income, data = ml))  
  ##               Income  Low Medium High
## Health_Binary                        
## 0                     330   1445  314
## 1                      22     85   15  
 
 
 Chi squared test of Health_Binary and Income 
  table(ml$Health_Binary, ml$Income)  
  ##    
##      Low Medium High
##   0  330   1445  314
##   1   22     85   15  
  chisq.test(ml$Health_Binary, ml$Income, correct=FALSE)  
  ## 
##  Pearson's Chi-squared test
## 
## data:  ml$Health_Binary and ml$Income
## X-squared = 0.94597, df = 2, p-value = 0.6231  
 
 
 
 BINARY LOGISTIC REGRESSION ON SIGNIFICANT OR SERIOUS ILLNESS 
 
 CHECK EFFECT OF OWNER CHARACTERISTICS - simple binary logistic
regression 
 
 
 CLIENT DIET binary regression ON SIGNIFICANT OR SERIOUS ILLNESS 
  # fit binary logit model and store results 'm'
m <- glm(Health_Binary ~ C_Diet, data = ml_train,family = binomial)
# view a summary of the model
summary(m)  
  ## 
## Call:
## glm(formula = Health_Binary ~ C_Diet, family = binomial, data = ml_train)
## 
## Coefficients:
##                                                       Estimate Std. Error
## (Intercept)                                            -3.1448     0.1930
## C_DietOmnivore reducing animal product consumption      0.3486     0.2963
## C_DietPescatarian (including fish but no other meats)  -0.2564     0.6178
## C_DietVegan (consuming no animal products)              0.2210     0.3095
## C_DietVegetarian (consuming plants, eggs and milk)      0.5734     0.3563
##                                                       z value Pr(>|z|)    
## (Intercept)                                           -16.293   <2e-16 ***
## C_DietOmnivore reducing animal product consumption      1.177    0.239    
## C_DietPescatarian (including fish but no other meats)  -0.415    0.678    
## C_DietVegan (consuming no animal products)              0.714    0.475    
## C_DietVegetarian (consuming plants, eggs and milk)      1.609    0.108    
## ---
## Signif. codes:  0 '***' 0.001 '**' 0.01 '*' 0.05 '.' 0.1 ' ' 1
## 
## (Dispersion parameter for binomial family taken to be 1)
## 
##     Null deviance: 652.97  on 1657  degrees of freedom
## Residual deviance: 649.31  on 1653  degrees of freedom
## AIC: 659.31
## 
## Number of Fisher Scoring iterations: 6  
  # test model fit
with(m, null.deviance - deviance)  
  ## [1] 3.65785  
  with(m, df.null - df.residual)  
  ## [1] 4  
  with(m, pchisq(null.deviance - deviance, df.null - df.residual, lower.tail = FALSE))  
  ## [1] 0.4542861  
  BIC(m)  
  ## [1] 686.3752  
  ## CIs using profiled log-likelihood
confint(m, level=0.99)  
  ## Waiting for profiling to be done...  
  ##                                                            0.5 %    99.5 %
## (Intercept)                                           -3.6832623 -2.682371
## C_DietOmnivore reducing animal product consumption    -0.4352090  1.107237
## C_DietPescatarian (including fish but no other meats) -2.2828154  1.096500
## C_DietVegan (consuming no animal products)            -0.6068197  1.006441
## C_DietVegetarian (consuming plants, eggs and milk)    -0.4091992  1.455527  
  ## CIs using standard errors
confint.default(m, level=0.99)  
  ##                                                            0.5 %    99.5 %
## (Intercept)                                           -3.6419279 -2.647608
## C_DietOmnivore reducing animal product consumption    -0.4145047  1.111802
## C_DietPescatarian (including fish but no other meats) -1.8478180  1.334959
## C_DietVegan (consuming no animal products)            -0.5762231  1.018241
## C_DietVegetarian (consuming plants, eggs and milk)    -0.3443603  1.491218  
  # Wald test
wald.test(b = coef(m), Sigma = vcov(m), Terms = 2)  
  ## Wald test:
## ----------
## 
## Chi-squared test:
## X2 = 1.4, df = 1, P(> X2) = 0.24  
  ## odds ratios and 95% CI
exp(cbind(OR = coef(m), confint(m, level=0.99)))  
  ## Waiting for profiling to be done...  
  ##                                                               OR      0.5 %
## (Intercept)                                           0.04307692 0.02514082
## C_DietOmnivore reducing animal product consumption    1.41715116 0.64712943
## C_DietPescatarian (including fish but no other meats) 0.77380952 0.10199664
## C_DietVegan (consuming no animal products)            1.24733475 0.54508163
## C_DietVegetarian (consuming plants, eggs and milk)    1.77434031 0.66418193
##                                                           99.5 %
## (Intercept)                                           0.06840079
## C_DietOmnivore reducing animal product consumption    3.02598485
## C_DietPescatarian (including fish but no other meats) 2.99366930
## C_DietVegan (consuming no animal products)            2.73584567
## C_DietVegetarian (consuming plants, eggs and milk)    4.28674026  
 
 check assumptions of model 
 
 Cook’s distance 
  plot(m, which = 4, id.n = 3)  
   
 
 
 Extract model results and display data for top 3 values using Cook’s
distance 
  model.data <- augment(m) %>% 
  mutate(index = 1:n()) 
model.data %>% top_n(3, .cooksd)  
 
 
 
 
 
 plot standardised residuals 
  ggplot(model.data, aes(index, .std.resid)) + 
  geom_point(aes(color = Health_Binary), alpha = .5) +
  theme_bw()  
   
 
 
 Filter potential influential data points with abs(.std.res) >
3: 
  model.data %>% 
  filter(abs(.std.resid) > 3)  
 
 
 
 
 
 Calculate Nagelkerke R^2 
  NagelkerkeR2(m)  
  ## $N
## [1] 1658
## 
## $R2
## [1] 0.006769736  
 
 
 
 Create ROCR for training and test data 
  ## training data
pred.mtt = predict(m, type = "response") #repeat risk predictions from model m
rocr.pred.mtt = ROCR::prediction(pred.mtt, labels = ml_train$Health_Binary) #ROCR prediction object
roc.perf.mtt = ROCR::performance(rocr.pred.mtt, measure = "tpr", x.measure = "fpr") # #ROCR performance object
plot(roc.perf.mtt, col = "blue")


pred.te.1 = predict(m, newdata = ml_test, type = "response") #.te = "test"
rocr.pred.te.1 = ROCR::prediction(pred.te.1, labels = ml_test$Health_Binary)
roc.perf.te.1 = ROCR::performance(rocr.pred.te.1, measure = "tpr", x.measure = "fpr")
plot(roc.perf.te.1, col = "red", add = T)

abline(a = 0, b = 1, lty = 2) #diagonal for random assignment
legend("bottomright", legend = c("train","test"),
col = c("blue","red"), lty = c(2,1), lwd =1.5)  
   
 
 
 Report AUC from ROC for training and test data 
    # Train AUC
aucTr <- ROCR::performance(rocr.pred.mtt, measure = "auc")
  aucTr <- aucTr@y.values[[1]]
  print(aucTr)  
  ## [1] 0.5590488  
     # Test AUC
  aucTe <- ROCR::performance(rocr.pred.te.1, measure = "auc")
  aucTe <- aucTe@y.values[[1]]
  print(aucTe)  
  ## [1] 0.5775341  
 
 
 
 CLIENT DIET VEGAN binary regression ON SIGNIFICANT OR SERIOUS
ILLNESS 
  # fit binary logit model and store results 'm'
m <- glm(Health_Binary ~ C_Diet_Vegan, data = ml_train,family = binomial)
# view a summary of the model
summary(m)  
  ## 
## Call:
## glm(formula = Health_Binary ~ C_Diet_Vegan, family = binomial, 
##     data = ml_train)
## 
## Coefficients:
##                 Estimate Std. Error z value Pr(>|z|)    
## (Intercept)     -2.96479    0.12817  -23.13   <2e-16 ***
## C_Diet_VeganYes  0.04103    0.27380    0.15    0.881    
## ---
## Signif. codes:  0 '***' 0.001 '**' 0.01 '*' 0.05 '.' 0.1 ' ' 1
## 
## (Dispersion parameter for binomial family taken to be 1)
## 
##     Null deviance: 652.97  on 1657  degrees of freedom
## Residual deviance: 652.94  on 1656  degrees of freedom
## AIC: 656.94
## 
## Number of Fisher Scoring iterations: 5  
  # test model fit
with(m, null.deviance - deviance)  
  ## [1] 0.02230065  
  with(m, df.null - df.residual)  
  ## [1] 1  
  with(m, pchisq(null.deviance - deviance, df.null - df.residual, lower.tail = FALSE))  
  ## [1] 0.8812901  
  BIC(m)  
  ## [1] 667.7707  
  ## CIs using profiled log-likelihood
confint(m, level=0.99)  
  ## Waiting for profiling to be done...  
  ##                      0.5 %     99.5 %
## (Intercept)     -3.3123609 -2.6500878
## C_Diet_VeganYes -0.7139121  0.7111944  
  ## CIs using standard errors
confint.default(m, level=0.99)  
  ##                      0.5 %    99.5 %
## (Intercept)     -3.2949465 -2.634633
## C_Diet_VeganYes -0.6642212  0.746283  
  # Wald test
wald.test(b = coef(m), Sigma = vcov(m), Terms = 2)  
  ## Wald test:
## ----------
## 
## Chi-squared test:
## X2 = 0.022, df = 1, P(> X2) = 0.88  
  ## odds ratios and 95% CI
exp(cbind(OR = coef(m), confint(m, level=0.99)))  
  ## Waiting for profiling to be done...  
  ##                         OR      0.5 %     99.5 %
## (Intercept)     0.05157131 0.03643006 0.07064501
## C_Diet_VeganYes 1.04188432 0.48972461 2.03642207  
 
 Calculate Nagelkerke R^2 
  NagelkerkeR2(m)  
  ## $N
## [1] 1658
## 
## $R2
## [1] 4.131802e-05  
 
 
 check assumptions of model 
 
 Cook’s distance 
  plot(m, which = 4, id.n = 3)  
   
 
 
 Extract model results and display data for top 3 values using Cook’s
distance 
  model.data <- augment(m) %>% 
  mutate(index = 1:n()) 
model.data %>% top_n(3, .cooksd)  
 
 
 
 
 
 plot standardised residuals 
  ggplot(model.data, aes(index, .std.resid)) + 
  geom_point(aes(color = Health_Binary), alpha = .5) +
  theme_bw()  
   
 
 
 Filter potential influential data points with abs(.std.res) >
3: 
  model.data %>% 
  filter(abs(.std.resid) > 3)  
 
 
 
 
 
 Calculate Nagelkerke R^2 
  NagelkerkeR2(m)  
  ## $N
## [1] 1658
## 
## $R2
## [1] 4.131802e-05  
 
 
 
 Create ROCR for training and test data 
  ## training data
pred.mtt = predict(m, type = "response") #repeat risk predictions from model m
rocr.pred.mtt = ROCR::prediction(pred.mtt, labels = ml_train$Health_Binary) #ROCR prediction object
roc.perf.mtt = ROCR::performance(rocr.pred.mtt, measure = "tpr", x.measure = "fpr") # #ROCR performance object
plot(roc.perf.mtt, col = "blue")


pred.te.1 = predict(m, newdata = ml_test, type = "response") #.te = "test"
rocr.pred.te.1 = ROCR::prediction(pred.te.1, labels = ml_test$Health_Binary)
roc.perf.te.1 = ROCR::performance(rocr.pred.te.1, measure = "tpr", x.measure = "fpr")
plot(roc.perf.te.1, col = "red", add = T)

abline(a = 0, b = 1, lty = 2) #diagonal for random assignment
legend("bottomright", legend = c("train","test"),
col = c("blue","red"), lty = c(2,1), lwd =1.5)  
   
 
 
 Report AUC from ROC for training and test data 
    # Train AUC
aucTr <- ROCR::performance(rocr.pred.mtt, measure = "auc")
  aucTr <- aucTr@y.values[[1]]
  print(aucTr)  
  ## [1] 0.5034744  
     # Test AUC
  aucTe <- ROCR::performance(rocr.pred.te.1, measure = "auc")
  aucTe <- aucTe@y.values[[1]]
  print(aucTe)  
  ## [1] 0.4848197  
 
 
 
 C_Diet_Vegan_Veggie Binary logistic regression for HEALTH 
  # fit binary logit model and store results 'm'
m <- glm(Health_Binary ~ C_Diet_Vegan_Veggie, data = ml_train,family = binomial)
# view a summary of the model
summary(m)  
  ## 
## Call:
## glm(formula = Health_Binary ~ C_Diet_Vegan_Veggie, family = binomial, 
##     data = ml_train)
## 
## Coefficients:
##                        Estimate Std. Error z value Pr(>|z|)    
## (Intercept)             -3.0372     0.1419 -21.396   <2e-16 ***
## C_Diet_Vegan_VeggieYes   0.2399     0.2356   1.018    0.309    
## ---
## Signif. codes:  0 '***' 0.001 '**' 0.01 '*' 0.05 '.' 0.1 ' ' 1
## 
## (Dispersion parameter for binomial family taken to be 1)
## 
##     Null deviance: 652.97  on 1657  degrees of freedom
## Residual deviance: 651.95  on 1656  degrees of freedom
## AIC: 655.95
## 
## Number of Fisher Scoring iterations: 5  
  # test model fit
with(m, null.deviance - deviance)  
  ## [1] 1.015042  
  with(m, df.null - df.residual)  
  ## [1] 1  
  with(m, pchisq(null.deviance - deviance, df.null - df.residual, lower.tail = FALSE))  
  ## [1] 0.3136979  
  BIC(m)  
  ## [1] 666.7779  
  ## CIs using profiled log-likelihood
confint(m, level=0.99)  
  ## Waiting for profiling to be done...  
  ##                             0.5 %    99.5 %
## (Intercept)            -3.4244678 -2.690512
## C_Diet_Vegan_VeggieYes -0.3860177  0.836070  
  ## CIs using standard errors
confint.default(m, level=0.99)  
  ##                             0.5 %     99.5 %
## (Intercept)            -3.4028024 -2.6715365
## C_Diet_Vegan_VeggieYes -0.3670132  0.8467894  
  # Wald test
wald.test(b = coef(m), Sigma = vcov(m), Terms = 2)  
  ## Wald test:
## ----------
## 
## Chi-squared test:
## X2 = 1.0, df = 1, P(> X2) = 0.31  
  ## odds ratios and 95% CI
exp(cbind(OR = coef(m), confint(m, level=0.99)))  
  ## Waiting for profiling to be done...  
  ##                                OR      0.5 %     99.5 %
## (Intercept)            0.04797048 0.03256661 0.06784621
## C_Diet_Vegan_VeggieYes 1.27110691 0.67975852 2.30728161  
 
 Calculate Nagelkerke R^2 
  NagelkerkeR2(m)  
  ## $N
## [1] 1658
## 
## $R2
## [1] 0.001880079  
 
 
 check assumptions of model 
 
 Cook’s distance 
  plot(m, which = 4, id.n = 3)  
   
 
 
 Extract model results and display data for top 3 values using Cook’s
distance 
  model.data <- augment(m) %>% 
  mutate(index = 1:n()) 
model.data %>% top_n(3, .cooksd)  
 
 
 
 
 
 plot standardised residuals 
  ggplot(model.data, aes(index, .std.resid)) + 
  geom_point(aes(color = Health_Binary), alpha = .5) +
  theme_bw()  
   
 
 
 Filter potential influential data points with abs(.std.res) >
3: 
  model.data %>% 
  filter(abs(.std.resid) > 3)  
 
 
 
 
 
 
 Create ROCR for training and test data 
  ## training data
pred.mtt = predict(m, type = "response") #repeat risk predictions from model m
rocr.pred.mtt = ROCR::prediction(pred.mtt, labels = ml_train$Health_Binary) #ROCR prediction object
roc.perf.mtt = ROCR::performance(rocr.pred.mtt, measure = "tpr", x.measure = "fpr") # #ROCR performance object
plot(roc.perf.mtt, col = "blue")


pred.te.1 = predict(m, newdata = ml_test, type = "response") #.te = "test"
rocr.pred.te.1 = ROCR::prediction(pred.te.1, labels = ml_test$Health_Binary)
roc.perf.te.1 = ROCR::performance(rocr.pred.te.1, measure = "tpr", x.measure = "fpr")
plot(roc.perf.te.1, col = "red", add = T)

abline(a = 0, b = 1, lty = 2) #diagonal for random assignment
legend("bottomright", legend = c("train","test"),
col = c("blue","red"), lty = c(2,1), lwd =1.5)  
   
 
 
 Report AUC from ROC for training and test data 
    # Train AUC
aucTr <- ROCR::performance(rocr.pred.mtt, measure = "auc")
  aucTr <- aucTr@y.values[[1]]
  print(aucTr)  
  ## [1] 0.5268355  
     # Test AUC
  aucTe <- ROCR::performance(rocr.pred.te.1, measure = "auc")
  aucTe <- aucTe@y.values[[1]]
  print(aucTe)  
  ## [1] 0.5015107  
 
 
 
 LOCATION binary logistic regression ON SIGNIFICANT OR SERIOUS
ILLNESS 
  # fit binary logit model and store results 'm'
m <- glm(Health_Binary ~ Location, data = ml_train,family = binomial)
# view a summary of the model
summary(m)  
  ## 
## Call:
## glm(formula = Health_Binary ~ Location, family = binomial, data = ml_train)
## 
## Coefficients:
##                                       Estimate Std. Error z value Pr(>|z|)    
## (Intercept)                            -3.1695     0.1458 -21.735  < 2e-16 ***
## LocationOther European                  0.5808     0.2974   1.953  0.05084 .  
## LocationNorth America                   0.3130     0.4825   0.649  0.51643    
## LocationAustralia/New Zealand/Oceania   1.0593     0.4018   2.637  0.00837 ** 
## LocationOther                           0.7716     0.5422   1.423  0.15471    
## ---
## Signif. codes:  0 '***' 0.001 '**' 0.01 '*' 0.05 '.' 0.1 ' ' 1
## 
## (Dispersion parameter for binomial family taken to be 1)
## 
##     Null deviance: 652.97  on 1657  degrees of freedom
## Residual deviance: 643.73  on 1653  degrees of freedom
## AIC: 653.73
## 
## Number of Fisher Scoring iterations: 6  
  # test model fit
with(m, null.deviance - deviance)  
  ## [1] 9.232455  
  with(m, df.null - df.residual)  
  ## [1] 4  
  with(m, pchisq(null.deviance - deviance, df.null - df.residual, lower.tail = FALSE))  
  ## [1] 0.0555447  
  BIC(m)  
  ## [1] 680.8006  
  # Hosmer-Lemeshow Goodness-of-Fit Test
glmtoolbox::hltest(m)  
  ## 
##    The Hosmer-Lemeshow goodness-of-fit test
## 
##  Group Size Observed Expected
##      1 1215       49       49
##      2   92        5        5
##      3  229       16       16
##      4   48        4        4
##      5   74        8        8
## 
##          Statistic =  0 
## degrees of freedom =  3 
##            p-value =  1  
  ## CIs using profiled log-likelihood
confint(m, level=0.99)  
  ## Waiting for profiling to be done...  
  ##                                            0.5 %    99.5 %
## (Intercept)                           -3.5682706 -2.814167
## LocationOther European                -0.2382521  1.311675
## LocationNorth America                 -1.1696756  1.406789
## LocationAustralia/New Zealand/Oceania -0.1042263  2.011498
## LocationOther                         -0.9315919  1.989374  
  ## CIs using standard errors
confint.default(m, level=0.99)  
  ##                                             0.5 %    99.5 %
## (Intercept)                           -3.54514200 -2.793886
## LocationOther European                -0.18530107  1.346922
## LocationNorth America                 -0.92967044  1.555758
## LocationAustralia/New Zealand/Oceania  0.02441578  2.094186
## LocationOther                         -0.62502489  2.168262  
  # Wald test
wald.test(b = coef(m), Sigma = vcov(m), Terms = 2)  
  ## Wald test:
## ----------
## 
## Chi-squared test:
## X2 = 3.8, df = 1, P(> X2) = 0.051  
  ## odds ratios and 95% CI
exp(cbind(OR = coef(m), confint(m, level=0.99)))  
  ## Waiting for profiling to be done...  
  ##                                               OR      0.5 %     99.5 %
## (Intercept)                           0.04202401 0.02820459 0.05995464
## LocationOther European                1.78748683 0.78800405 3.71238849
## LocationNorth America                 1.36758152 0.31046765 4.08282502
## LocationAustralia/New Zealand/Oceania 2.88435374 0.90102135 7.47450792
## LocationOther                         2.16326531 0.39392612 7.31095354  
 
 Calculate Nagelkerke R^2 
  NagelkerkeR2(m)  
  ## $N
## [1] 1658
## 
## $R2
## [1] 0.01705821  
 
 
 check assumptions of model 
 
 Cook’s distance 
  plot(m, which = 4, id.n = 3)  
   
 
 
 Extract model results and display data for top 3 values using Cook’s
distance 
  model.data <- augment(m) %>% 
  mutate(index = 1:n()) 
model.data %>% top_n(3, .cooksd)  
 
 
 
 
 
 plot standardised residuals 
  ggplot(model.data, aes(index, .std.resid)) + 
  geom_point(aes(color = Health_Binary), alpha = .5) +
  theme_bw()  
   
 
 
 Filter potential influential data points with abs(.std.res) >
3: 
  model.data %>% 
  filter(abs(.std.resid) > 3)  
 
 
 
 
 
 
 Create ROCR for training and test data 
  ## training data
pred.mtt = predict(m, type = "response") #repeat risk predictions from model m
rocr.pred.mtt = ROCR::prediction(pred.mtt, labels = ml_train$Health_Binary) #ROCR prediction object
roc.perf.mtt = ROCR::performance(rocr.pred.mtt, measure = "tpr", x.measure = "fpr") # #ROCR performance object
plot(roc.perf.mtt, col = "blue")


pred.te.1 = predict(m, newdata = ml_test, type = "response") #.te = "test"
rocr.pred.te.1 = ROCR::prediction(pred.te.1, labels = ml_test$Health_Binary)
roc.perf.te.1 = ROCR::performance(rocr.pred.te.1, measure = "tpr", x.measure = "fpr")
plot(roc.perf.te.1, col = "red", add = T)

abline(a = 0, b = 1, lty = 2) #diagonal for random assignment
legend("bottomright", legend = c("train","test"),
col = c("blue","red"), lty = c(2,1), lwd =1.5)  
   
 
 
 Report AUC from ROC for training and test data 
    # Train AUC
aucTr <- ROCR::performance(rocr.pred.mtt, measure = "auc")
  aucTr <- aucTr@y.values[[1]]
  print(aucTr)  
  ## [1] 0.57774  
     # Test AUC
  aucTe <- ROCR::performance(rocr.pred.te.1, measure = "auc")
  aucTe <- aucTe@y.values[[1]]
  print(aucTe)  
  ## [1] 0.4813596  
 
 
 
 SETTING binary logistic regression ON SIGNIFICANT OR SERIOUS
ILLNESS 
  # fit binary logit model and store results 'm'
m <- glm(Health_Binary ~ setting, data = ml_train,family = binomial)
# view a summary of the model
summary(m)  
  ## 
## Call:
## glm(formula = Health_Binary ~ setting, family = binomial, data = ml_train)
## 
## Coefficients:
##                                Estimate Std. Error z value Pr(>|z|)    
## (Intercept)                     -2.8650     0.1846 -15.516   <2e-16 ***
## settingRural                    -0.1102     0.2702  -0.408    0.683    
## settingEqually urban and rural  -0.1776     0.2788  -0.637    0.524    
## ---
## Signif. codes:  0 '***' 0.001 '**' 0.01 '*' 0.05 '.' 0.1 ' ' 1
## 
## (Dispersion parameter for binomial family taken to be 1)
## 
##     Null deviance: 652.97  on 1657  degrees of freedom
## Residual deviance: 652.54  on 1655  degrees of freedom
## AIC: 658.54
## 
## Number of Fisher Scoring iterations: 5  
  # test model fit
with(m, null.deviance - deviance)  
  ## [1] 0.4222019  
  with(m, df.null - df.residual)  
  ## [1] 2  
  with(m, pchisq(null.deviance - deviance, df.null - df.residual, lower.tail = FALSE))  
  ## [1] 0.8096923  
  BIC(m)  
  ## [1] 674.7841  
  # Hosmer-Lemeshow Goodness-of-Fit Test
glmtoolbox::hltest(m)  
  ## 
##    The Hosmer-Lemeshow goodness-of-fit test
## 
##  Group Size Observed Expected
##      1  527       24       24
##      2  556       27       27
##      3  575       31       31
## 
##          Statistic =  0 
## degrees of freedom =  1 
##            p-value =  1  
  ## CIs using profiled log-likelihood
confint(m, level=0.99)  
  ## Waiting for profiling to be done...  
  ##                                     0.5 %     99.5 %
## (Intercept)                    -3.3772902 -2.4203342
## settingRural                   -0.8174051  0.5868581
## settingEqually urban and rural -0.9125871  0.5373710  
  ## CIs using standard errors
confint.default(m, level=0.99)  
  ##                                     0.5 %     99.5 %
## (Intercept)                    -3.3405807 -2.3893433
## settingRural                   -0.8062202  0.5858412
## settingEqually urban and rural -0.8957637  0.5406151  
  # Wald test
wald.test(b = coef(m), Sigma = vcov(m), Terms = 2)  
  ## Wald test:
## ----------
## 
## Chi-squared test:
## X2 = 0.17, df = 1, P(> X2) = 0.68  
  ## odds ratios and 95% CI
exp(cbind(OR = coef(m), confint(m, level=0.99)))  
  ## Waiting for profiling to be done...  
  ##                                        OR      0.5 %    99.5 %
## (Intercept)                    0.05698529 0.03413984 0.0888919
## settingRural                   0.89566438 0.44157601 1.7983293
## settingEqually urban and rural 0.83729881 0.40148419 1.7115014  
 
 Calculate Nagelkerke R^2 
  NagelkerkeR2(m)  
  ## $N
## [1] 1658
## 
## $R2
## [1] 0.0007821494  
 
 
 check assumptions of model 
 
 Cook’s distance 
  plot(m, which = 4, id.n = 3)  
   
 
 
 Extract model results and display data for top 3 values using Cook’s
distance 
  model.data <- augment(m) %>% 
  mutate(index = 1:n()) 
model.data %>% top_n(3, .cooksd)  
 
 
 
 
 
 plot standardised residuals 
  ggplot(model.data, aes(index, .std.resid)) + 
  geom_point(aes(color = Health_Binary), alpha = .5) +
  theme_bw()  
   
 
 
 Filter potential influential data points with abs(.std.res) >
3: 
  model.data %>% 
  filter(abs(.std.resid) > 3)  
 
 
 
 
 
 
 Create ROCR for training and test data 
  ## training data
pred.mtt = predict(m, type = "response") #repeat risk predictions from model m
rocr.pred.mtt = ROCR::prediction(pred.mtt, labels = ml_train$Health_Binary) #ROCR prediction object
roc.perf.mtt = ROCR::performance(rocr.pred.mtt, measure = "tpr", x.measure = "fpr") # #ROCR performance object
plot(roc.perf.mtt, col = "blue")


pred.te.1 = predict(m, newdata = ml_test, type = "response") #.te = "test"
rocr.pred.te.1 = ROCR::prediction(pred.te.1, labels = ml_test$Health_Binary)
roc.perf.te.1 = ROCR::performance(rocr.pred.te.1, measure = "tpr", x.measure = "fpr")
plot(roc.perf.te.1, col = "red", add = T)

abline(a = 0, b = 1, lty = 2) #diagonal for random assignment
legend("bottomright", legend = c("train","test"),
col = c("blue","red"), lty = c(2,1), lwd =1.5)  
   
 
 
 Report AUC from ROC for training and test data 
    # Train AUC
aucTr <- ROCR::performance(rocr.pred.mtt, measure = "auc")
  aucTr <- aucTr@y.values[[1]]
  print(aucTr)  
  ## [1] 0.5198596  
     # Test AUC
  aucTe <- ROCR::performance(rocr.pred.te.1, measure = "auc")
  aucTe <- aucTe@y.values[[1]]
  print(aucTe)  
  ## [1] 0.5423977  
 
 
 
 URBAN binary logistic regression ON SIGNIFICANT OR SERIOUS
ILLNESS 
  # fit binary logit model and store results 'm'
m <- glm(Health_Binary ~ Urban, data = ml_train,family = binomial)
# view a summary of the model
summary(m)  
  ## 
## Call:
## glm(formula = Health_Binary ~ Urban, family = binomial, data = ml_train)
## 
## Coefficients:
##             Estimate Std. Error z value Pr(>|z|)    
## (Intercept)  -3.0074     0.1434 -20.967   <2e-16 ***
## UrbanYes      0.1425     0.2338   0.609    0.542    
## ---
## Signif. codes:  0 '***' 0.001 '**' 0.01 '*' 0.05 '.' 0.1 ' ' 1
## 
## (Dispersion parameter for binomial family taken to be 1)
## 
##     Null deviance: 652.97  on 1657  degrees of freedom
## Residual deviance: 652.60  on 1656  degrees of freedom
## AIC: 656.6
## 
## Number of Fisher Scoring iterations: 5  
  # test model fit
with(m, null.deviance - deviance)  
  ## [1] 0.3671608  
  with(m, df.null - df.residual)  
  ## [1] 1  
  with(m, pchisq(null.deviance - deviance, df.null - df.residual, lower.tail = FALSE))  
  ## [1] 0.544556  
  BIC(m)  
  ## [1] 667.4258  
  ## CIs using profiled log-likelihood
confint(m, level=0.99)  
  ## Waiting for profiling to be done...  
  ##                  0.5 %    99.5 %
## (Intercept) -3.3989576 -2.657277
## UrbanYes    -0.4772074  0.735408  
  ## CIs using standard errors
confint.default(m, level=0.99)  
  ##                  0.5 %     99.5 %
## (Intercept) -3.3768908 -2.6379658
## UrbanYes    -0.4597926  0.7447251  
  # Wald test
wald.test(b = coef(m), Sigma = vcov(m), Terms = 2)  
  ## Wald test:
## ----------
## 
## Chi-squared test:
## X2 = 0.37, df = 1, P(> X2) = 0.54  
  ## odds ratios and 95% CI
exp(cbind(OR = coef(m), confint(m, level=0.99)))  
  ## Waiting for profiling to be done...  
  ##                     OR      0.5 %     99.5 %
## (Intercept) 0.04941861 0.03340808 0.07013894
## UrbanYes    1.15311417 0.62051385 2.08633315  
 
 Calculate Nagelkerke R^2 
  NagelkerkeR2(m)  
  ## $N
## [1] 1658
## 
## $R2
## [1] 0.0006801944  
 
 
 check assumptions of model 
 
 Cook’s distance 
  plot(m, which = 4, id.n = 3)  
   
 
 
 
 
 
 Extract model results and display data for top 3 values using Cook’s
distance 
  model.data <- augment(m) %>% 
  mutate(index = 1:n()) 
model.data %>% top_n(3, .cooksd)  
 
 
 
 
 plot standardised residuals 
  ggplot(model.data, aes(index, .std.resid)) + 
  geom_point(aes(color = Health_Binary), alpha = .5) +
  theme_bw()  
   
 
 
 Filter potential influential data points with abs(.std.res) >
3: 
  model.data %>% 
  filter(abs(.std.resid) > 3)  
 
 
 
 
 
 Create ROCR for training and test data 
  ## training data
pred.mtt = predict(m, type = "response") #repeat risk predictions from model m
rocr.pred.mtt = ROCR::prediction(pred.mtt, labels = ml_train$Health_Binary) #ROCR prediction object
roc.perf.mtt = ROCR::performance(rocr.pred.mtt, measure = "tpr", x.measure = "fpr") # #ROCR performance object
plot(roc.perf.mtt, col = "blue")


pred.te.1 = predict(m, newdata = ml_test, type = "response") #.te = "test"
rocr.pred.te.1 = ROCR::prediction(pred.te.1, labels = ml_test$Health_Binary)
roc.perf.te.1 = ROCR::performance(rocr.pred.te.1, measure = "tpr", x.measure = "fpr")
plot(roc.perf.te.1, col = "red", add = T)

abline(a = 0, b = 1, lty = 2) #diagonal for random assignment
legend("bottomright", legend = c("train","test"),
col = c("blue","red"), lty = c(2,1), lwd =1.5)  
   
 
 
 Report AUC from ROC for training and test data 
    # Train AUC
aucTr <- ROCR::performance(rocr.pred.mtt, measure = "auc")
  aucTr <- aucTr@y.values[[1]]
  print(aucTr)  
  ## [1] 0.5164356  
     # Test AUC
  aucTe <- ROCR::performance(rocr.pred.te.1, measure = "auc")
  aucTe <- aucTe@y.values[[1]]
  print(aucTe)  
  ## [1] 0.5157895  
 
 
 LOCATION + URBAN binary logistic regression ON SIGNIFICANT OR
SERIOUS ILLNESS 
  # fit binary logit model and store results 'm'
m <- glm(Health_Binary ~ Location + Urban, data = ml_train,family = binomial)
# view a summary of the model
summary(m)  
  ## 
## Call:
## glm(formula = Health_Binary ~ Location + Urban, family = binomial, 
##     data = ml_train)
## 
## Coefficients:
##                                       Estimate Std. Error z value Pr(>|z|)    
## (Intercept)                           -3.15853    0.16137 -19.573  < 2e-16 ***
## LocationOther European                 0.58636    0.29951   1.958  0.05026 .  
## LocationNorth America                  0.31827    0.48359   0.658  0.51046    
## LocationAustralia/New Zealand/Oceania  1.07428    0.41297   2.601  0.00929 ** 
## LocationOther                          0.79029    0.55515   1.424  0.15458    
## UrbanYes                              -0.03861    0.24604  -0.157  0.87531    
## ---
## Signif. codes:  0 '***' 0.001 '**' 0.01 '*' 0.05 '.' 0.1 ' ' 1
## 
## (Dispersion parameter for binomial family taken to be 1)
## 
##     Null deviance: 652.97  on 1657  degrees of freedom
## Residual deviance: 643.71  on 1652  degrees of freedom
## AIC: 655.71
## 
## Number of Fisher Scoring iterations: 6  
  # test model fit
with(m, null.deviance - deviance)  
  ## [1] 9.25715  
  with(m, df.null - df.residual)  
  ## [1] 5  
  with(m, pchisq(null.deviance - deviance, df.null - df.residual, lower.tail = FALSE))  
  ## [1] 0.09923653  
  BIC(m)  
  ## [1] 688.1893  
  # Hosmer-Lemeshow Goodness-of-Fit Test
hltest(m)  
  ## 
##    The Hosmer-Lemeshow goodness-of-fit test
## 
##  Group Size Observed  Expected
##      1  350       15 13.745715
##      2  865       34 35.254285
##      3  191       12 11.776350
##      4  167       13 12.281759
##      5   85        8  8.941891
## 
##          Statistic =  0.32639 
## degrees of freedom =  3 
##            p-value =  0.95499  
  ## CIs using profiled log-likelihood
confint(m, level=0.99)  
  ## Waiting for profiling to be done...  
  ##                                            0.5 %    99.5 %
## (Intercept)                           -3.5993496 -2.765207
## LocationOther European                -0.2373957  1.323103
## LocationNorth America                 -1.1665188  1.415773
## LocationAustralia/New Zealand/Oceania -0.1116908  2.060995
## LocationOther                         -0.9349076  2.052190
## UrbanYes                              -0.6915060  0.583433  
  ## CIs using standard errors
confint.default(m, level=0.99)  
  ##                                             0.5 %     99.5 %
## (Intercept)                           -3.57419503 -2.7428693
## LocationOther European                -0.18512430  1.3578494
## LocationNorth America                 -0.92738716  1.5639204
## LocationAustralia/New Zealand/Oceania  0.01053811  2.1380167
## LocationOther                         -0.63969429  2.2202690
## UrbanYes                              -0.67236486  0.5951481  
  # Wald test
wald.test(b = coef(m), Sigma = vcov(m), Terms = 2)  
  ## Wald test:
## ----------
## 
## Chi-squared test:
## X2 = 3.8, df = 1, P(> X2) = 0.05  
  ## odds ratios and 95% CI
exp(cbind(OR = coef(m), confint(m, level=0.99)))  
  ## Waiting for profiling to be done...  
  ##                                               OR     0.5 %     99.5 %
## (Intercept)                           0.04248806 0.0273415 0.06296305
## LocationOther European                1.79743843 0.7886791 3.75505535
## LocationNorth America                 1.37474273 0.3114493 4.11967147
## LocationAustralia/New Zealand/Oceania 2.92787650 0.8943207 7.85378350
## LocationOther                         2.20402963 0.3926221 7.78492908
## UrbanYes                              0.96212742 0.5008213 1.79218048  
 
 Calculate Nagelkerke R^2 
  NagelkerkeR2(m)  
  ## $N
## [1] 1658
## 
## $R2
## [1] 0.01710371  
 
 
 check assumptions of model 
 
 Cook’s distance 
  plot(m, which = 4, id.n = 3)  
   
 
 
 Extract model results and display data for top 3 values using Cook’s
distance 
  model.data <- augment(m) %>% 
  mutate(index = 1:n()) 
model.data %>% top_n(3, .cooksd)  
 
 
 
 
 
 plot standardised residuals 
  ggplot(model.data, aes(index, .std.resid)) + 
  geom_point(aes(color = Health_Binary), alpha = .5) +
  theme_bw()  
   
 
 
 Filter potential influential data points with abs(.std.res) >
3: 
  model.data %>% 
  filter(abs(.std.resid) > 3)  
 
 
 
 
 
 check for multicollinearity 
  car::vif(m)  
  ##              GVIF Df GVIF^(1/(2*Df))
## Location 1.099006  4        1.011871
## Urban    1.099006  1        1.048335  
 
 
 
 Create ROCR for training and test data 
  ## training data
pred.mtt = predict(m, type = "response") #repeat risk predictions from model m
rocr.pred.mtt = ROCR::prediction(pred.mtt, labels = ml_train$Health_Binary) #ROCR prediction object
roc.perf.mtt = ROCR::performance(rocr.pred.mtt, measure = "tpr", x.measure = "fpr") # #ROCR performance object
plot(roc.perf.mtt, col = "blue")


pred.te.1 = predict(m, newdata = ml_test, type = "response") #.te = "test"
rocr.pred.te.1 = ROCR::prediction(pred.te.1, labels = ml_test$Health_Binary)
roc.perf.te.1 = ROCR::performance(rocr.pred.te.1, measure = "tpr", x.measure = "fpr")
plot(roc.perf.te.1, col = "red", add = T)

abline(a = 0, b = 1, lty = 2) #diagonal for random assignment
legend("bottomright", legend = c("train","test"),
col = c("blue","red"), lty = c(2,1), lwd =1.5)  
   
 
 
 Report AUC from ROC for training and test data 
    # Train AUC
aucTr <- ROCR::performance(rocr.pred.mtt, measure = "auc")
  aucTr <- aucTr@y.values[[1]]
  print(aucTr)  
  ## [1] 0.5740683  
     # Test AUC
  aucTe <- ROCR::performance(rocr.pred.te.1, measure = "auc")
  aucTe <- aucTe@y.values[[1]]
  print(aucTe)  
  ## [1] 0.4670565  
 
 
 
 LOCATION * URBAN binary logistic regression ON SIGNIFICANT OR
SERIOUS ILLNESS 
  # fit binary logit model and store results 'm'
m <- glm(Health_Binary ~ Location*Urban, data = ml_train,family = binomial)
# view a summary of the model
summary(m)  
  ## 
## Call:
## glm(formula = Health_Binary ~ Location * Urban, family = binomial, 
##     data = ml_train)
## 
## Coefficients:
##                                                 Estimate Std. Error z value
## (Intercept)                                     -3.19627    0.17497 -18.267
## LocationOther European                           0.59770    0.38729   1.543
## LocationNorth America                            0.38286    0.61964   0.618
## LocationAustralia/New Zealand/Oceania            1.86127    0.53221   3.497
## LocationOther                                  -13.36980  723.48998  -0.018
## UrbanYes                                         0.09019    0.31665   0.285
## LocationOther European:UrbanYes                 -0.06750    0.61104  -0.110
## LocationNorth America:UrbanYes                  -0.19455    0.99027  -0.196
## LocationAustralia/New Zealand/Oceania:UrbanYes  -1.50672    0.84114  -1.791
## LocationOther:UrbanYes                          14.36567  723.49022   0.020
##                                                Pr(>|z|)    
## (Intercept)                                     < 2e-16 ***
## LocationOther European                          0.12276    
## LocationNorth America                           0.53666    
## LocationAustralia/New Zealand/Oceania           0.00047 ***
## LocationOther                                   0.98526    
## UrbanYes                                        0.77578    
## LocationOther European:UrbanYes                 0.91204    
## LocationNorth America:UrbanYes                  0.84425    
## LocationAustralia/New Zealand/Oceania:UrbanYes  0.07325 .  
## LocationOther:UrbanYes                          0.98416    
## ---
## Signif. codes:  0 '***' 0.001 '**' 0.01 '*' 0.05 '.' 0.1 ' ' 1
## 
## (Dispersion parameter for binomial family taken to be 1)
## 
##     Null deviance: 652.97  on 1657  degrees of freedom
## Residual deviance: 638.02  on 1648  degrees of freedom
## AIC: 658.02
## 
## Number of Fisher Scoring iterations: 15  
  # test model fit
with(m, null.deviance - deviance)  
  ## [1] 14.95107  
  with(m, df.null - df.residual)  
  ## [1] 9  
  with(m, pchisq(null.deviance - deviance, df.null - df.residual, lower.tail = FALSE))  
  ## [1] 0.09228869  
  BIC(m)  
  ## [1] 712.1488  
  # Hosmer-Lemeshow Goodness-of-Fit Test
hltest(m)  
  ## 
##    The Hosmer-Lemeshow goodness-of-fit test
## 
##  Group Size Observed     Expected
##      1   11        0 7.028142e-07
##      2  865       34 3.400000e+01
##      3  350       15 1.500000e+01
##      4  142        8 8.000000e+00
##      5  130        9 9.000000e+00
##      6  160       16 1.600000e+01
## 
##          Statistic =  0 
## degrees of freedom =  4 
##            p-value =  1  
  ## CIs using profiled log-likelihood
confint(m, level=0.99)  
  ## Waiting for profiling to be done...  
  ## Warning: glm.fit: fitted probabilities numerically 0 or 1 occurred

## Warning: glm.fit: fitted probabilities numerically 0 or 1 occurred

## Warning: glm.fit: fitted probabilities numerically 0 or 1 occurred

## Warning: glm.fit: fitted probabilities numerically 0 or 1 occurred

## Warning: glm.fit: fitted probabilities numerically 0 or 1 occurred

## Warning: glm.fit: fitted probabilities numerically 0 or 1 occurred

## Warning: glm.fit: fitted probabilities numerically 0 or 1 occurred

## Warning: glm.fit: fitted probabilities numerically 0 or 1 occurred

## Warning: glm.fit: fitted probabilities numerically 0 or 1 occurred

## Warning: glm.fit: fitted probabilities numerically 0 or 1 occurred

## Warning: glm.fit: fitted probabilities numerically 0 or 1 occurred

## Warning: glm.fit: fitted probabilities numerically 0 or 1 occurred

## Warning: glm.fit: fitted probabilities numerically 0 or 1 occurred

## Warning: glm.fit: fitted probabilities numerically 0 or 1 occurred

## Warning: glm.fit: fitted probabilities numerically 0 or 1 occurred

## Warning: glm.fit: fitted probabilities numerically 0 or 1 occurred

## Warning: glm.fit: fitted probabilities numerically 0 or 1 occurred

## Warning: glm.fit: fitted probabilities numerically 0 or 1 occurred

## Warning: glm.fit: fitted probabilities numerically 0 or 1 occurred

## Warning: glm.fit: fitted probabilities numerically 0 or 1 occurred

## Warning: glm.fit: fitted probabilities numerically 0 or 1 occurred

## Warning: glm.fit: fitted probabilities numerically 0 or 1 occurred

## Warning: glm.fit: fitted probabilities numerically 0 or 1 occurred

## Warning: glm.fit: fitted probabilities numerically 0 or 1 occurred

## Warning: glm.fit: fitted probabilities numerically 0 or 1 occurred

## Warning: glm.fit: fitted probabilities numerically 0 or 1 occurred

## Warning: glm.fit: fitted probabilities numerically 0 or 1 occurred

## Warning: glm.fit: fitted probabilities numerically 0 or 1 occurred

## Warning: glm.fit: fitted probabilities numerically 0 or 1 occurred

## Warning: glm.fit: fitted probabilities numerically 0 or 1 occurred

## Warning: glm.fit: fitted probabilities numerically 0 or 1 occurred

## Warning: glm.fit: fitted probabilities numerically 0 or 1 occurred

## Warning: glm.fit: fitted probabilities numerically 0 or 1 occurred

## Warning: glm.fit: fitted probabilities numerically 0 or 1 occurred

## Warning: glm.fit: fitted probabilities numerically 0 or 1 occurred

## Warning: glm.fit: fitted probabilities numerically 0 or 1 occurred

## Warning: glm.fit: fitted probabilities numerically 0 or 1 occurred  
  ##                                                       0.5 %     99.5 %
## (Intercept)                                      -3.6807941 -2.7744875
## LocationOther European                           -0.5065554  1.5290642
## LocationNorth America                            -1.6465366  1.7411684
## LocationAustralia/New Zealand/Oceania             0.2920021  3.1308995
## LocationOther                                            NA 68.2793456
## UrbanYes                                         -0.7763023  0.8764013
## LocationOther European:UrbanYes                  -1.6866086  1.5203410
## LocationNorth America:UrbanYes                   -3.1662012  2.4094772
## LocationAustralia/New Zealand/Oceania:UrbanYes   -3.9039060  0.6389850
## LocationOther:UrbanYes                         -119.7083077         NA  
  ## CIs using standard errors
confint.default(m, level=0.99)  
  ##                                                        0.5 %       99.5 %
## (Intercept)                                       -3.6469668   -2.7455718
## LocationOther European                            -0.3998802    1.5952868
## LocationNorth America                             -1.2132174    1.9789345
## LocationAustralia/New Zealand/Oceania              0.4903880    3.2321485
## LocationOther                                  -1876.9564806 1850.2168823
## UrbanYes                                          -0.7254460    0.9058239
## LocationOther European:UrbanYes                   -1.6414346    1.5064318
## LocationNorth America:UrbanYes                    -2.7453058    2.3562079
## LocationAustralia/New Zealand/Oceania:UrbanYes    -3.6733438    0.6598974
## LocationOther:UrbanYes                         -1849.2216382 1877.9529707  
  # Wald test
wald.test(b = coef(m), Sigma = vcov(m), Terms = 2)  
  ## Wald test:
## ----------
## 
## Chi-squared test:
## X2 = 2.4, df = 1, P(> X2) = 0.12  
  ## odds ratios and 95% CI
exp(cbind(OR = coef(m), confint(m, level=0.99)))  
  ## Waiting for profiling to be done...  
  ## Warning: glm.fit: fitted probabilities numerically 0 or 1 occurred

## Warning: glm.fit: fitted probabilities numerically 0 or 1 occurred

## Warning: glm.fit: fitted probabilities numerically 0 or 1 occurred

## Warning: glm.fit: fitted probabilities numerically 0 or 1 occurred

## Warning: glm.fit: fitted probabilities numerically 0 or 1 occurred

## Warning: glm.fit: fitted probabilities numerically 0 or 1 occurred

## Warning: glm.fit: fitted probabilities numerically 0 or 1 occurred

## Warning: glm.fit: fitted probabilities numerically 0 or 1 occurred

## Warning: glm.fit: fitted probabilities numerically 0 or 1 occurred

## Warning: glm.fit: fitted probabilities numerically 0 or 1 occurred

## Warning: glm.fit: fitted probabilities numerically 0 or 1 occurred

## Warning: glm.fit: fitted probabilities numerically 0 or 1 occurred

## Warning: glm.fit: fitted probabilities numerically 0 or 1 occurred

## Warning: glm.fit: fitted probabilities numerically 0 or 1 occurred

## Warning: glm.fit: fitted probabilities numerically 0 or 1 occurred

## Warning: glm.fit: fitted probabilities numerically 0 or 1 occurred

## Warning: glm.fit: fitted probabilities numerically 0 or 1 occurred

## Warning: glm.fit: fitted probabilities numerically 0 or 1 occurred

## Warning: glm.fit: fitted probabilities numerically 0 or 1 occurred

## Warning: glm.fit: fitted probabilities numerically 0 or 1 occurred

## Warning: glm.fit: fitted probabilities numerically 0 or 1 occurred

## Warning: glm.fit: fitted probabilities numerically 0 or 1 occurred

## Warning: glm.fit: fitted probabilities numerically 0 or 1 occurred

## Warning: glm.fit: fitted probabilities numerically 0 or 1 occurred

## Warning: glm.fit: fitted probabilities numerically 0 or 1 occurred

## Warning: glm.fit: fitted probabilities numerically 0 or 1 occurred

## Warning: glm.fit: fitted probabilities numerically 0 or 1 occurred

## Warning: glm.fit: fitted probabilities numerically 0 or 1 occurred

## Warning: glm.fit: fitted probabilities numerically 0 or 1 occurred

## Warning: glm.fit: fitted probabilities numerically 0 or 1 occurred

## Warning: glm.fit: fitted probabilities numerically 0 or 1 occurred

## Warning: glm.fit: fitted probabilities numerically 0 or 1 occurred

## Warning: glm.fit: fitted probabilities numerically 0 or 1 occurred

## Warning: glm.fit: fitted probabilities numerically 0 or 1 occurred

## Warning: glm.fit: fitted probabilities numerically 0 or 1 occurred

## Warning: glm.fit: fitted probabilities numerically 0 or 1 occurred

## Warning: glm.fit: fitted probabilities numerically 0 or 1 occurred  
  ##                                                          OR        0.5 %
## (Intercept)                                    4.091456e-02 2.520295e-02
## LocationOther European                         1.817939e+00 6.025676e-01
## LocationNorth America                          1.466471e+00 1.927162e-01
## LocationAustralia/New Zealand/Oceania          6.431889e+00 1.339106e+00
## LocationOther                                  1.561601e-06           NA
## UrbanYes                                       1.094381e+00 4.601042e-01
## LocationOther European:UrbanYes                9.347264e-01 1.851464e-01
## LocationNorth America:UrbanYes                 8.232059e-01 4.216346e-02
## LocationAustralia/New Zealand/Oceania:UrbanYes 2.216350e-01 2.016300e-02
## LocationOther:UrbanYes                         1.733523e+06 1.026461e-52
##                                                      99.5 %
## (Intercept)                                    6.238144e-02
## LocationOther European                         4.613857e+00
## LocationNorth America                          5.704004e+00
## LocationAustralia/New Zealand/Oceania          2.289456e+01
## LocationOther                                  4.501352e+29
## UrbanYes                                       2.402239e+00
## LocationOther European:UrbanYes                4.573785e+00
## LocationNorth America:UrbanYes                 1.112814e+01
## LocationAustralia/New Zealand/Oceania:UrbanYes 1.894557e+00
## LocationOther:UrbanYes                                   NA  
 
 Calculate Nagelkerke R^2 
  NagelkerkeR2(m)  
  ## $N
## [1] 1658
## 
## $R2
## [1] 0.02757659  
 
 
 check assumptions of model 
 
 Cook’s distance 
  plot(m, which = 4, id.n = 3)  
   
 
 
 Extract model results and display data for top 3 values using Cook’s
distance 
  model.data <- augment(m) %>% 
  mutate(index = 1:n()) 
model.data %>% top_n(3, .cooksd)  
 
 
 
 
 
 plot standardised residuals 
  ggplot(model.data, aes(index, .std.resid)) + 
  geom_point(aes(color = Health_Binary), alpha = .5) +
  theme_bw()  
   
 
 
 Filter potential influential data points with abs(.std.res) >
3: 
  model.data %>% 
  filter(abs(.std.resid) > 3)  
 
 
 
 
 
 check for multicollinearity 
  car::vif(m)  
  ## there are higher-order terms (interactions) in this model
## consider setting type = 'predictor'; see ?vif  
  ##                        GVIF Df GVIF^(1/(2*Df))
## Location       8.488131e+06  4        7.346856
## Urban          1.815889e+00  1        1.347549
## Location:Urban 1.261998e+07  4        7.720268  
 
 
 
 Create ROCR for training and test data 
  ## training data
pred.mtt = predict(m, type = "response") #repeat risk predictions from model m
rocr.pred.mtt = ROCR::prediction(pred.mtt, labels = ml_train$Health_Binary) #ROCR prediction object
roc.perf.mtt = ROCR::performance(rocr.pred.mtt, measure = "tpr", x.measure = "fpr") # #ROCR performance object
plot(roc.perf.mtt, col = "blue")


pred.te.1 = predict(m, newdata = ml_test, type = "response") #.te = "test"
rocr.pred.te.1 = ROCR::prediction(pred.te.1, labels = ml_test$Health_Binary)
roc.perf.te.1 = ROCR::performance(rocr.pred.te.1, measure = "tpr", x.measure = "fpr")
plot(roc.perf.te.1, col = "red", add = T)

abline(a = 0, b = 1, lty = 2) #diagonal for random assignment
legend("bottomright", legend = c("train","test"),
col = c("blue","red"), lty = c(2,1), lwd =1.5)  
   
 
 
 Report AUC from ROC for training and test data 
    # Train AUC
aucTr <- ROCR::performance(rocr.pred.mtt, measure = "auc")
  aucTr <- aucTr@y.values[[1]]
  print(aucTr)  
  ## [1] 0.5903956  
     # Test AUC
  aucTe <- ROCR::performance(rocr.pred.te.1, measure = "auc")
  aucTe <- aucTe@y.values[[1]]
  print(aucTe)  
  ## [1] 0.5020955  
 
 
 
 EDUCATION Binary logistic regression ON SIGNIFICANT OR SERIOUS
ILLNESS 
  # fit binary logit model and store results 'm'
m <- glm(Health_Binary ~ Education, data = ml_train,family = binomial)
# view a summary of the model
summary(m)  
  ## 
## Call:
## glm(formula = Health_Binary ~ Education, family = binomial, data = ml_train)
## 
## Coefficients:
##             Estimate Std. Error z value Pr(>|z|)
## (Intercept)   -5.164     83.390  -0.062    0.951
## Education.L    8.389    299.010   0.028    0.978
## Education.Q   -7.200    272.958  -0.026    0.979
## Education.C    5.057    186.466   0.027    0.978
## Education^4   -2.706     94.556  -0.029    0.977
## Education^5    1.039     31.519   0.033    0.974
## 
## (Dispersion parameter for binomial family taken to be 1)
## 
##     Null deviance: 652.97  on 1657  degrees of freedom
## Residual deviance: 648.62  on 1652  degrees of freedom
## AIC: 660.62
## 
## Number of Fisher Scoring iterations: 15  
  # test model fit
with(m, null.deviance - deviance)  
  ## [1] 4.343303  
  with(m, df.null - df.residual)  
  ## [1] 5  
  with(m, pchisq(null.deviance - deviance, df.null - df.residual, lower.tail = FALSE))  
  ## [1] 0.5011185  
  BIC(m)  
  ## [1] 693.1031  
  # Hosmer-Lemeshow Goodness-of-Fit Test
hltest(m)  
  ## 
##    The Hosmer-Lemeshow goodness-of-fit test
## 
##  Group Size Observed     Expected
##      1   23        0 1.469521e-06
##      2  482       19 1.900000e+01
##      3  496       26 2.600000e+01
##      4  265       14 1.400000e+01
##      5  332       19 1.900000e+01
##      6   60        4 4.000000e+00
## 
##          Statistic =  0 
## degrees of freedom =  4 
##            p-value =  1  
  ## CIs using profiled log-likelihood
confint(m, level=0.99)  
  ## Waiting for profiling to be done...  
  ## Warning: glm.fit: fitted probabilities numerically 0 or 1 occurred

## Warning: glm.fit: fitted probabilities numerically 0 or 1 occurred

## Warning: glm.fit: fitted probabilities numerically 0 or 1 occurred

## Warning: glm.fit: fitted probabilities numerically 0 or 1 occurred

## Warning: glm.fit: fitted probabilities numerically 0 or 1 occurred

## Warning: glm.fit: fitted probabilities numerically 0 or 1 occurred

## Warning: glm.fit: fitted probabilities numerically 0 or 1 occurred

## Warning: glm.fit: fitted probabilities numerically 0 or 1 occurred

## Warning: glm.fit: fitted probabilities numerically 0 or 1 occurred

## Warning: glm.fit: fitted probabilities numerically 0 or 1 occurred

## Warning: glm.fit: fitted probabilities numerically 0 or 1 occurred

## Warning: glm.fit: fitted probabilities numerically 0 or 1 occurred

## Warning: glm.fit: fitted probabilities numerically 0 or 1 occurred

## Warning: glm.fit: fitted probabilities numerically 0 or 1 occurred

## Warning: glm.fit: fitted probabilities numerically 0 or 1 occurred

## Warning: glm.fit: fitted probabilities numerically 0 or 1 occurred

## Warning: glm.fit: fitted probabilities numerically 0 or 1 occurred

## Warning: glm.fit: fitted probabilities numerically 0 or 1 occurred

## Warning: glm.fit: fitted probabilities numerically 0 or 1 occurred

## Warning: glm.fit: fitted probabilities numerically 0 or 1 occurred

## Warning: glm.fit: fitted probabilities numerically 0 or 1 occurred

## Warning: glm.fit: fitted probabilities numerically 0 or 1 occurred

## Warning: glm.fit: fitted probabilities numerically 0 or 1 occurred

## Warning: glm.fit: fitted probabilities numerically 0 or 1 occurred

## Warning: glm.fit: fitted probabilities numerically 0 or 1 occurred

## Warning: glm.fit: fitted probabilities numerically 0 or 1 occurred

## Warning: glm.fit: fitted probabilities numerically 0 or 1 occurred

## Warning: glm.fit: fitted probabilities numerically 0 or 1 occurred

## Warning: glm.fit: fitted probabilities numerically 0 or 1 occurred

## Warning: glm.fit: fitted probabilities numerically 0 or 1 occurred

## Warning: glm.fit: fitted probabilities numerically 0 or 1 occurred

## Warning: glm.fit: fitted probabilities numerically 0 or 1 occurred

## Warning: glm.fit: fitted probabilities numerically 0 or 1 occurred

## Warning: glm.fit: fitted probabilities numerically 0 or 1 occurred

## Warning: glm.fit: fitted probabilities numerically 0 or 1 occurred

## Warning: glm.fit: fitted probabilities numerically 0 or 1 occurred

## Warning: glm.fit: fitted probabilities numerically 0 or 1 occurred

## Warning: glm.fit: fitted probabilities numerically 0 or 1 occurred

## Warning: glm.fit: fitted probabilities numerically 0 or 1 occurred

## Warning: glm.fit: fitted probabilities numerically 0 or 1 occurred

## Warning: glm.fit: fitted probabilities numerically 0 or 1 occurred

## Warning: glm.fit: fitted probabilities numerically 0 or 1 occurred

## Warning: glm.fit: fitted probabilities numerically 0 or 1 occurred

## Warning: glm.fit: fitted probabilities numerically 0 or 1 occurred

## Warning: glm.fit: fitted probabilities numerically 0 or 1 occurred

## Warning: glm.fit: fitted probabilities numerically 0 or 1 occurred

## Warning: glm.fit: fitted probabilities numerically 0 or 1 occurred

## Warning: glm.fit: fitted probabilities numerically 0 or 1 occurred

## Warning: glm.fit: fitted probabilities numerically 0 or 1 occurred

## Warning: glm.fit: fitted probabilities numerically 0 or 1 occurred

## Warning: glm.fit: fitted probabilities numerically 0 or 1 occurred

## Warning: glm.fit: fitted probabilities numerically 0 or 1 occurred

## Warning: glm.fit: fitted probabilities numerically 0 or 1 occurred

## Warning: glm.fit: fitted probabilities numerically 0 or 1 occurred

## Warning: glm.fit: fitted probabilities numerically 0 or 1 occurred

## Warning: glm.fit: fitted probabilities numerically 0 or 1 occurred

## Warning: glm.fit: fitted probabilities numerically 0 or 1 occurred

## Warning: glm.fit: fitted probabilities numerically 0 or 1 occurred

## Warning: glm.fit: fitted probabilities numerically 0 or 1 occurred

## Warning: glm.fit: fitted probabilities numerically 0 or 1 occurred  
  ##                    0.5 %     99.5 %
## (Intercept)  -39.9892849  -1.136608
## Education.L   -7.5066022 125.280215
## Education.Q -113.9062122   7.311017
## Education.C   -4.8555757  77.951598
## Education^4  -39.6699153   2.321090
## Education^5   -0.6367929  13.360524  
  ## CIs using standard errors
confint.default(m, level=0.99)  
  ##                  0.5 %    99.5 %
## (Intercept) -219.96201 209.63494
## Education.L -761.81054 778.58859
## Education.Q -710.29245 695.89318
## Education.C -475.24698 485.36117
## Education^4 -246.26479 240.85366
## Education^5  -80.14944  82.22703  
  # Wald test
wald.test(b = coef(m), Sigma = vcov(m), Terms = 2)  
  ## Wald test:
## ----------
## 
## Chi-squared test:
## X2 = 0.00079, df = 1, P(> X2) = 0.98  
  ## odds ratios and 95% CI
exp(cbind(OR = coef(m), confint(m, level=0.99)))  
  ## Waiting for profiling to be done...  
  ## Warning: glm.fit: fitted probabilities numerically 0 or 1 occurred

## Warning: glm.fit: fitted probabilities numerically 0 or 1 occurred

## Warning: glm.fit: fitted probabilities numerically 0 or 1 occurred

## Warning: glm.fit: fitted probabilities numerically 0 or 1 occurred

## Warning: glm.fit: fitted probabilities numerically 0 or 1 occurred

## Warning: glm.fit: fitted probabilities numerically 0 or 1 occurred

## Warning: glm.fit: fitted probabilities numerically 0 or 1 occurred

## Warning: glm.fit: fitted probabilities numerically 0 or 1 occurred

## Warning: glm.fit: fitted probabilities numerically 0 or 1 occurred

## Warning: glm.fit: fitted probabilities numerically 0 or 1 occurred

## Warning: glm.fit: fitted probabilities numerically 0 or 1 occurred

## Warning: glm.fit: fitted probabilities numerically 0 or 1 occurred

## Warning: glm.fit: fitted probabilities numerically 0 or 1 occurred

## Warning: glm.fit: fitted probabilities numerically 0 or 1 occurred

## Warning: glm.fit: fitted probabilities numerically 0 or 1 occurred

## Warning: glm.fit: fitted probabilities numerically 0 or 1 occurred

## Warning: glm.fit: fitted probabilities numerically 0 or 1 occurred

## Warning: glm.fit: fitted probabilities numerically 0 or 1 occurred

## Warning: glm.fit: fitted probabilities numerically 0 or 1 occurred

## Warning: glm.fit: fitted probabilities numerically 0 or 1 occurred

## Warning: glm.fit: fitted probabilities numerically 0 or 1 occurred

## Warning: glm.fit: fitted probabilities numerically 0 or 1 occurred

## Warning: glm.fit: fitted probabilities numerically 0 or 1 occurred

## Warning: glm.fit: fitted probabilities numerically 0 or 1 occurred

## Warning: glm.fit: fitted probabilities numerically 0 or 1 occurred

## Warning: glm.fit: fitted probabilities numerically 0 or 1 occurred

## Warning: glm.fit: fitted probabilities numerically 0 or 1 occurred

## Warning: glm.fit: fitted probabilities numerically 0 or 1 occurred

## Warning: glm.fit: fitted probabilities numerically 0 or 1 occurred

## Warning: glm.fit: fitted probabilities numerically 0 or 1 occurred

## Warning: glm.fit: fitted probabilities numerically 0 or 1 occurred

## Warning: glm.fit: fitted probabilities numerically 0 or 1 occurred

## Warning: glm.fit: fitted probabilities numerically 0 or 1 occurred

## Warning: glm.fit: fitted probabilities numerically 0 or 1 occurred

## Warning: glm.fit: fitted probabilities numerically 0 or 1 occurred

## Warning: glm.fit: fitted probabilities numerically 0 or 1 occurred

## Warning: glm.fit: fitted probabilities numerically 0 or 1 occurred

## Warning: glm.fit: fitted probabilities numerically 0 or 1 occurred

## Warning: glm.fit: fitted probabilities numerically 0 or 1 occurred

## Warning: glm.fit: fitted probabilities numerically 0 or 1 occurred

## Warning: glm.fit: fitted probabilities numerically 0 or 1 occurred

## Warning: glm.fit: fitted probabilities numerically 0 or 1 occurred

## Warning: glm.fit: fitted probabilities numerically 0 or 1 occurred

## Warning: glm.fit: fitted probabilities numerically 0 or 1 occurred

## Warning: glm.fit: fitted probabilities numerically 0 or 1 occurred

## Warning: glm.fit: fitted probabilities numerically 0 or 1 occurred

## Warning: glm.fit: fitted probabilities numerically 0 or 1 occurred

## Warning: glm.fit: fitted probabilities numerically 0 or 1 occurred

## Warning: glm.fit: fitted probabilities numerically 0 or 1 occurred

## Warning: glm.fit: fitted probabilities numerically 0 or 1 occurred

## Warning: glm.fit: fitted probabilities numerically 0 or 1 occurred

## Warning: glm.fit: fitted probabilities numerically 0 or 1 occurred

## Warning: glm.fit: fitted probabilities numerically 0 or 1 occurred

## Warning: glm.fit: fitted probabilities numerically 0 or 1 occurred

## Warning: glm.fit: fitted probabilities numerically 0 or 1 occurred

## Warning: glm.fit: fitted probabilities numerically 0 or 1 occurred

## Warning: glm.fit: fitted probabilities numerically 0 or 1 occurred

## Warning: glm.fit: fitted probabilities numerically 0 or 1 occurred

## Warning: glm.fit: fitted probabilities numerically 0 or 1 occurred

## Warning: glm.fit: fitted probabilities numerically 0 or 1 occurred  
  ##                       OR        0.5 %       99.5 %
## (Intercept) 5.721439e-03 4.294120e-18 3.209056e-01
## Education.L 4.398518e+03 5.494448e-04 2.561569e+54
## Education.Q 7.468561e-04 3.397509e-50 1.496698e+03
## Education.C 1.571331e+02 7.784850e-03 7.144119e+33
## Education^4 6.683267e-02 5.909826e-18 1.018677e+01
## Education^5 2.825812e+00 5.289862e-01 6.344562e+05  
 
 Calculate Nagelkerke R^2 
  NagelkerkeR2(m)  
  ## $N
## [1] 1658
## 
## $R2
## [1] 0.008036673  
 
 
 check assumptions of model 
 
 Cook’s distance 
  plot(m, which = 4, id.n = 3)  
   
 
 
 Extract model results and display data for top 3 values using Cook’s
distance 
  model.data <- augment(m) %>% 
  mutate(index = 1:n()) 
model.data %>% top_n(3, .cooksd)  
 
 
 
 
 
 plot standardised residuals 
  ggplot(model.data, aes(index, .std.resid)) + 
  geom_point(aes(color = Health_Binary), alpha = .5) +
  theme_bw()  
   
 
 
 Filter potential influential data points with abs(.std.res) >
3: 
  model.data %>% 
  filter(abs(.std.resid) > 3)  
 
 
 
 
 
 
 Create ROCR for training and test data 
  ## training data
pred.mtt = predict(m, type = "response") #repeat risk predictions from model m
rocr.pred.mtt = ROCR::prediction(pred.mtt, labels = ml_train$Health_Binary) #ROCR prediction object
roc.perf.mtt = ROCR::performance(rocr.pred.mtt, measure = "tpr", x.measure = "fpr") # #ROCR performance object
plot(roc.perf.mtt, col = "blue")


pred.te.1 = predict(m, newdata = ml_test, type = "response") #.te = "test"
rocr.pred.te.1 = ROCR::prediction(pred.te.1, labels = ml_test$Health_Binary)
roc.perf.te.1 = ROCR::performance(rocr.pred.te.1, measure = "tpr", x.measure = "fpr")
plot(roc.perf.te.1, col = "red", add = T)

abline(a = 0, b = 1, lty = 2) #diagonal for random assignment
legend("bottomright", legend = c("train","test"),
col = c("blue","red"), lty = c(2,1), lwd =1.5)  
   
 
 
 Report AUC from ROC for training and test data 
    # Train AUC
aucTr <- ROCR::performance(rocr.pred.mtt, measure = "auc")
  aucTr <- aucTr@y.values[[1]]
  print(aucTr)  
  ## [1] 0.5480144  
     # Test AUC
  aucTe <- ROCR::performance(rocr.pred.te.1, measure = "auc")
  aucTe <- aucTe@y.values[[1]]
  print(aucTe)  
  ## [1] 0.5067008  
 
 
 
 EDUCATION_S Binary logistic regression ON SIGNIFICANT OR SERIOUS
ILLNESS 
  # fit binary logit model and store results 'm'
m <- glm(Health_Binary ~ Education_S, data = ml_train,family = binomial)
# view a summary of the model
summary(m)  
  ## 
## Call:
## glm(formula = Health_Binary ~ Education_S, family = binomial, 
##     data = ml_train)
## 
## Coefficients:
##                        Estimate Std. Error z value Pr(>|z|)    
## (Intercept)            -2.97407    0.27400 -10.854   <2e-16 ***
## Education_S1_College   -0.21922    0.36037  -0.608    0.543    
## Education_S2_Grad       0.07943    0.34010   0.234    0.815    
## Education_S3_PG_or_PhD  0.19877    0.34823   0.571    0.568    
## ---
## Signif. codes:  0 '***' 0.001 '**' 0.01 '*' 0.05 '.' 0.1 ' ' 1
## 
## (Dispersion parameter for binomial family taken to be 1)
## 
##     Null deviance: 652.97  on 1657  degrees of freedom
## Residual deviance: 651.09  on 1654  degrees of freedom
## AIC: 659.09
## 
## Number of Fisher Scoring iterations: 6  
  # test model fit
with(m, null.deviance - deviance)  
  ## [1] 1.872423  
  with(m, df.null - df.residual)  
  ## [1] 3  
  with(m, pchisq(null.deviance - deviance, df.null - df.residual, lower.tail = FALSE))  
  ## [1] 0.5993031  
  BIC(m)  
  ## [1] 680.7473  
  # Hosmer-Lemeshow Goodness-of-Fit Test
hltest(m, G=3)  
  ## 
##    The Hosmer-Lemeshow goodness-of-fit test
## 
##  Group Size Observed Expected
##      1  482       19       19
##      2  288       14       14
##      3  496       26       26
##      4  392       23       23
## 
##          Statistic =  0 
## degrees of freedom =  2 
##            p-value =  1  
  ## CIs using profiled log-likelihood
confint(m, level=0.99)  
  ## Waiting for profiling to be done...  
  ##                             0.5 %     99.5 %
## (Intercept)            -3.7650338 -2.3348239
## Education_S1_College   -1.1420486  0.7441126
## Education_S2_Grad      -0.7739333  1.0034292
## Education_S3_PG_or_PhD -0.6815995  1.1389402  
  ## CIs using standard errors
confint.default(m, level=0.99)  
  ##                             0.5 %     99.5 %
## (Intercept)            -3.6798584 -2.2682832
## Education_S1_College   -1.1474799  0.7090453
## Education_S2_Grad      -0.7966026  0.9554719
## Education_S3_PG_or_PhD -0.6982214  1.0957581  
  # Wald test
wald.test(b = coef(m), Sigma = vcov(m), Terms = 2)  
  ## Wald test:
## ----------
## 
## Chi-squared test:
## X2 = 0.37, df = 1, P(> X2) = 0.54  
  ## odds ratios and 95% CI
exp(cbind(OR = coef(m), confint(m, level=0.99)))  
  ## Waiting for profiling to be done...  
  ##                                OR      0.5 %     99.5 %
## (Intercept)            0.05109489 0.02316683 0.09682753
## Education_S1_College   0.80314718 0.31916451 2.10457301
## Education_S2_Grad      1.08267477 0.46119545 2.72761939
## Education_S3_PG_or_PhD 1.21989934 0.50580730 3.12345631  
 
 Calculate Nagelkerke R^2 
  NagelkerkeR2(m)  
  ## $N
## [1] 1658
## 
## $R2
## [1] 0.003467238  
 
 
 check assumptions of model 
 
 Cook’s distance 
  plot(m, which = 4, id.n = 3)  
   
 
 
 Extract model results and display data for top 3 values using Cook’s
distance 
  model.data <- augment(m) %>% 
  mutate(index = 1:n()) 
model.data %>% top_n(3, .cooksd)  
 
 
 
 
 
 plot standardised residuals 
  ggplot(model.data, aes(index, .std.resid)) + 
  geom_point(aes(color = Health_Binary), alpha = .5) +
  theme_bw()  
   
 
 
 Filter potential influential data points with abs(.std.res) >
3: 
  model.data %>% 
  filter(abs(.std.resid) > 3)  
 
 
 
 
 
 
 Create ROCR for training and test data 
  ## training data
pred.mtt = predict(m, type = "response") #repeat risk predictions from model m
rocr.pred.mtt = ROCR::prediction(pred.mtt, labels = ml_train$Health_Binary) #ROCR prediction object
roc.perf.mtt = ROCR::performance(rocr.pred.mtt, measure = "tpr", x.measure = "fpr") # #ROCR performance object
plot(roc.perf.mtt, col = "blue")


pred.te.1 = predict(m, newdata = ml_test, type = "response") #.te = "test"
rocr.pred.te.1 = ROCR::prediction(pred.te.1, labels = ml_test$Health_Binary)
roc.perf.te.1 = ROCR::performance(rocr.pred.te.1, measure = "tpr", x.measure = "fpr")
plot(roc.perf.te.1, col = "red", add = T)

abline(a = 0, b = 1, lty = 2) #diagonal for random assignment
legend("bottomright", legend = c("train","test"),
col = c("blue","red"), lty = c(2,1), lwd =1.5)  
   
 
 
 Report AUC from ROC for training and test data 
    # Train AUC
aucTr <- ROCR::performance(rocr.pred.mtt, measure = "auc")
  aucTr <- aucTr@y.values[[1]]
  print(aucTr)  
  ## [1] 0.5422419  
     # Test AUC
  aucTe <- ROCR::performance(rocr.pred.te.1, measure = "auc")
  aucTe <- aucTe@y.values[[1]]
  print(aucTe)  
  ## [1] 0.5301901  
 
 
 
 EDUCATION_S2 Binary logistic regression ON SIGNIFICANT OR SERIOUS
ILLNESS 
  # fit binary logit model and store results 'm'
m <- glm(Health_Binary ~ Education_S2, data = ml_train,family = binomial)
# view a summary of the model
summary(m)  
  ## 
## Call:
## glm(formula = Health_Binary ~ Education_S2, family = binomial, 
##     data = ml_train)
## 
## Coefficients:
##                         Estimate Std. Error z value Pr(>|z|)    
## (Intercept)             -2.97407    0.27400 -10.854   <2e-16 ***
## Education_S21_College   -0.21922    0.36037  -0.608    0.543    
## Education_S22_Grad       0.07943    0.34010   0.234    0.815    
## Education_S23_PG_or_PhD  0.19877    0.34823   0.571    0.568    
## ---
## Signif. codes:  0 '***' 0.001 '**' 0.01 '*' 0.05 '.' 0.1 ' ' 1
## 
## (Dispersion parameter for binomial family taken to be 1)
## 
##     Null deviance: 652.97  on 1657  degrees of freedom
## Residual deviance: 651.09  on 1654  degrees of freedom
## AIC: 659.09
## 
## Number of Fisher Scoring iterations: 6  
  # test model fit
with(m, null.deviance - deviance)  
  ## [1] 1.872423  
  with(m, df.null - df.residual)  
  ## [1] 3  
  with(m, pchisq(null.deviance - deviance, df.null - df.residual, lower.tail = FALSE))  
  ## [1] 0.5993031  
  BIC(m)  
  ## [1] 680.7473  
  # Hosmer-Lemeshow Goodness-of-Fit Test
hltest(m, G=3)  
  ## 
##    The Hosmer-Lemeshow goodness-of-fit test
## 
##  Group Size Observed Expected
##      1  482       19       19
##      2  288       14       14
##      3  496       26       26
##      4  392       23       23
## 
##          Statistic =  0 
## degrees of freedom =  2 
##            p-value =  1  
  ## CIs using profiled log-likelihood
confint(m, level=0.99)  
  ## Waiting for profiling to be done...  
  ##                              0.5 %     99.5 %
## (Intercept)             -3.7650338 -2.3348239
## Education_S21_College   -1.1420486  0.7441126
## Education_S22_Grad      -0.7739333  1.0034292
## Education_S23_PG_or_PhD -0.6815995  1.1389402  
  ## CIs using standard errors
confint.default(m, level=0.99)  
  ##                              0.5 %     99.5 %
## (Intercept)             -3.6798584 -2.2682832
## Education_S21_College   -1.1474799  0.7090453
## Education_S22_Grad      -0.7966026  0.9554719
## Education_S23_PG_or_PhD -0.6982214  1.0957581  
  # Wald test
wald.test(b = coef(m), Sigma = vcov(m), Terms = 2)  
  ## Wald test:
## ----------
## 
## Chi-squared test:
## X2 = 0.37, df = 1, P(> X2) = 0.54  
  ## odds ratios and 95% CI
exp(cbind(OR = coef(m), confint(m, level=0.99)))  
  ## Waiting for profiling to be done...  
  ##                                 OR      0.5 %     99.5 %
## (Intercept)             0.05109489 0.02316683 0.09682753
## Education_S21_College   0.80314718 0.31916451 2.10457301
## Education_S22_Grad      1.08267477 0.46119545 2.72761939
## Education_S23_PG_or_PhD 1.21989934 0.50580730 3.12345631  
 
 Calculate Nagelkerke R^2 
  NagelkerkeR2(m)  
  ## $N
## [1] 1658
## 
## $R2
## [1] 0.003467238  
 
 
 check assumptions of model 
 
 Cook’s distance 
  plot(m, which = 4, id.n = 3)  
   
 
 
 Extract model results and display data for top 3 values using Cook’s
distance 
  model.data <- augment(m) %>% 
  mutate(index = 1:n()) 
model.data %>% top_n(3, .cooksd)  
 
 
 
 
 
 plot standardised residuals 
  ggplot(model.data, aes(index, .std.resid)) + 
  geom_point(aes(color = Health_Binary), alpha = .5) +
  theme_bw()  
   
 
 
 Filter potential influential data points with abs(.std.res) >
3: 
  model.data %>% 
  filter(abs(.std.resid) > 3)  
 
 
 
 
 
 
 Create ROCR for training and test data 
  ## training data
pred.mtt = predict(m, type = "response") #repeat risk predictions from model m
rocr.pred.mtt = ROCR::prediction(pred.mtt, labels = ml_train$Health_Binary) #ROCR prediction object
roc.perf.mtt = ROCR::performance(rocr.pred.mtt, measure = "tpr", x.measure = "fpr") # #ROCR performance object
plot(roc.perf.mtt, col = "blue")


pred.te.1 = predict(m, newdata = ml_test, type = "response") #.te = "test"
rocr.pred.te.1 = ROCR::prediction(pred.te.1, labels = ml_test$Health_Binary)
roc.perf.te.1 = ROCR::performance(rocr.pred.te.1, measure = "tpr", x.measure = "fpr")
plot(roc.perf.te.1, col = "red", add = T)

abline(a = 0, b = 1, lty = 2) #diagonal for random assignment
legend("bottomright", legend = c("train","test"),
col = c("blue","red"), lty = c(2,1), lwd =1.5)  
   
 
 
 Report AUC from ROC for training and test data 
    # Train AUC
aucTr <- ROCR::performance(rocr.pred.mtt, measure = "auc")
  aucTr <- aucTr@y.values[[1]]
  print(aucTr)  
  ## [1] 0.5422419  
     # Test AUC
  aucTe <- ROCR::performance(rocr.pred.te.1, measure = "auc")
  aucTe <- aucTe@y.values[[1]]
  print(aucTe)  
  ## [1] 0.5301901  
 
 
 
 ANIMAL CAREER 2 Binary logistic regression ON SIGNIFICANT OR SERIOUS
ILLNESS 
  # fit binary logit model and store results 'm'
m <- glm(Health_Binary ~ Animal_Career2, data = ml_train,family = binomial)
# view a summary of the model
summary(m)  
  ## 
## Call:
## glm(formula = Health_Binary ~ Animal_Career2, family = binomial, 
##     data = ml_train)
## 
## Coefficients:
##                                   Estimate Std. Error z value Pr(>|z|)    
## (Intercept)                        -3.0477     0.1321 -23.066   <2e-16 ***
## Animal_Career2Vet professional      0.7169     0.4174   1.718   0.0858 .  
## Animal_Career2breeder/trainer      -0.2574     0.5260  -0.489   0.6246    
## Animal_Career2Pet industry worker   0.5704     0.3406   1.675   0.0940 .  
## ---
## Signif. codes:  0 '***' 0.001 '**' 0.01 '*' 0.05 '.' 0.1 ' ' 1
## 
## (Dispersion parameter for binomial family taken to be 1)
## 
##     Null deviance: 652.97  on 1657  degrees of freedom
## Residual deviance: 647.80  on 1654  degrees of freedom
## AIC: 655.8
## 
## Number of Fisher Scoring iterations: 6  
  # test model fit
with(m, null.deviance - deviance)  
  ## [1] 5.164077  
  with(m, df.null - df.residual)  
  ## [1] 3  
  with(m, pchisq(null.deviance - deviance, df.null - df.residual, lower.tail = FALSE))  
  ## [1] 0.1601694  
  BIC(m)  
  ## [1] 677.4556  
  # Hosmer-Lemeshow Goodness-of-Fit Test
## CIs using profiled log-likelihood
confint(m, level=0.99)  
  ## Waiting for profiling to be done...  
  ##                                        0.5 %     99.5 %
## (Intercept)                       -3.4066964 -2.7239071
## Animal_Career2Vet professional    -0.5163022  1.6892897
## Animal_Career2breeder/trainer     -1.9339674  0.9008759
## Animal_Career2Pet industry worker -0.3984047  1.3848233  
  ## CIs using standard errors
confint.default(m, level=0.99)  
  ##                                        0.5 %    99.5 %
## (Intercept)                       -3.3880312 -2.707353
## Animal_Career2Vet professional    -0.3581567  1.792029
## Animal_Career2breeder/trainer     -1.6121399  1.097417
## Animal_Career2Pet industry worker -0.3069077  1.447688  
  # Wald test
wald.test(b = coef(m), Sigma = vcov(m), Terms = 2)  
  ## Wald test:
## ----------
## 
## Chi-squared test:
## X2 = 3.0, df = 1, P(> X2) = 0.086  
  ## odds ratios and 95% CI
exp(cbind(OR = coef(m), confint(m, level=0.99)))  
  ## Waiting for profiling to be done...  
  ##                                           OR      0.5 %     99.5 %
## (Intercept)                       0.04746835 0.03315054 0.06561788
## Animal_Career2Vet professional    2.04814815 0.59672301 5.41563245
## Animal_Career2breeder/trainer     0.77308869 0.14457348 2.46175837
## Animal_Career2Pet industry worker 1.76895674 0.67139025 3.99411992  
 
 Calculate Nagelkerke R^2 
  NagelkerkeR2(m)  
  ## $N
## [1] 1658
## 
## $R2
## [1] 0.009553036  
 
 
 check assumptions of model 
 
 Cook’s distance 
  plot(m, which = 4, id.n = 3)  
   
 
 
 Extract model results and display data for top 3 values using Cook’s
distance 
  model.data <- augment(m) %>% 
  mutate(index = 1:n()) 
model.data %>% top_n(3, .cooksd)  
 
 
 
 
 
 plot standardised residuals 
  ggplot(model.data, aes(index, .std.resid)) + 
  geom_point(aes(color = Health_Binary), alpha = .5) +
  theme_bw()  
   
 
 
 
 
 
 Filter potential influential data points with abs(.std.res) >
3: 
  model.data %>% 
  filter(abs(.std.resid) > 3)  
 
 
 
 
 Create ROCR for training and test data 
  ## training data
pred.mtt = predict(m, type = "response") #repeat risk predictions from model m
rocr.pred.mtt = ROCR::prediction(pred.mtt, labels = ml_train$Health_Binary) #ROCR prediction object
roc.perf.mtt = ROCR::performance(rocr.pred.mtt, measure = "tpr", x.measure = "fpr") # #ROCR performance object
plot(roc.perf.mtt, col = "blue")


pred.te.1 = predict(m, newdata = ml_test, type = "response") #.te = "test"
rocr.pred.te.1 = ROCR::prediction(pred.te.1, labels = ml_test$Health_Binary)
roc.perf.te.1 = ROCR::performance(rocr.pred.te.1, measure = "tpr", x.measure = "fpr")
plot(roc.perf.te.1, col = "red", add = T)

abline(a = 0, b = 1, lty = 2) #diagonal for random assignment
legend("bottomright", legend = c("train","test"),
col = c("blue","red"), lty = c(2,1), lwd =1.5)  
   
 
 
 Report AUC from ROC for training and test data 
    # Train AUC
aucTr <- ROCR::performance(rocr.pred.mtt, measure = "auc")
  aucTr <- aucTr@y.values[[1]]
  print(aucTr)  
  ## [1] 0.5515778  
     # Test AUC
  aucTe <- ROCR::performance(rocr.pred.te.1, measure = "auc")
  aucTe <- aucTe@y.values[[1]]
  print(aucTe)  
  ## [1] 0.5247563  
 
 
 ANIMAL_CAREER_BINARY Binary logistic regression ON SIGNIFICANT OR
SERIOUS ILLNESS 
  # fit binary logit model and store results 'm'
m <- glm(Health_Binary ~ Animal_Career_BINARY, data = ml_train,family = binomial)
# view a summary of the model
summary(m)  
  ## 
## Call:
## glm(formula = Health_Binary ~ Animal_Career_BINARY, family = binomial, 
##     data = ml_train)
## 
## Coefficients:
##                         Estimate Std. Error z value Pr(>|z|)    
## (Intercept)              -3.0477     0.1321 -23.069   <2e-16 ***
## Animal_Career_BINARYYes   0.3957     0.2571   1.539    0.124    
## ---
## Signif. codes:  0 '***' 0.001 '**' 0.01 '*' 0.05 '.' 0.1 ' ' 1
## 
## (Dispersion parameter for binomial family taken to be 1)
## 
##     Null deviance: 652.97  on 1657  degrees of freedom
## Residual deviance: 650.73  on 1656  degrees of freedom
## AIC: 654.73
## 
## Number of Fisher Scoring iterations: 5  
  # test model fit
with(m, null.deviance - deviance)  
  ## [1] 2.236792  
  with(m, df.null - df.residual)  
  ## [1] 1  
  with(m, pchisq(null.deviance - deviance, df.null - df.residual, lower.tail = FALSE))  
  ## [1] 0.1347603  
  BIC(m)  
  ## [1] 665.5562  
  ## CIs using profiled log-likelihood
confint(m, level=0.99)  
  ## Waiting for profiling to be done...  
  ##                              0.5 %    99.5 %
## (Intercept)             -3.4066964 -2.723907
## Animal_Career_BINARYYes -0.3023523  1.033546  
  ## CIs using standard errors
confint.default(m, level=0.99)  
  ##                              0.5 %    99.5 %
## (Intercept)             -3.3879935 -2.707391
## Animal_Career_BINARYYes -0.2665788  1.058041  
  # Wald test
wald.test(b = coef(m), Sigma = vcov(m), Terms = 2)  
  ## Wald test:
## ----------
## 
## Chi-squared test:
## X2 = 2.4, df = 1, P(> X2) = 0.12  
  ## odds ratios and 95% CI
exp(cbind(OR = coef(m), confint(m, level=0.99)))  
  ## Waiting for profiling to be done...  
  ##                                 OR      0.5 %     99.5 %
## (Intercept)             0.04746836 0.03315053 0.06561789
## Animal_Career_BINARYYes 1.48547005 0.73907765 2.81101707  
 
 Calculate Nagelkerke R^2 
  NagelkerkeR2(m)  
  ## $N
## [1] 1658
## 
## $R2
## [1] 0.004141499  
 
 
 check assumptions of model 
 
 Cook’s distance 
  plot(m, which = 4, id.n = 3)  
   
 
 
 Extract model results and display data for top 3 values using Cook’s
distance 
  model.data <- augment(m) %>% 
  mutate(index = 1:n()) 
model.data %>% top_n(3, .cooksd)  
 
 
 
 
 
 plot standardised residuals 
  ggplot(model.data, aes(index, .std.resid)) + 
  geom_point(aes(color = Health_Binary), alpha = .5) +
  theme_bw()  
   
 
 
 Filter potential influential data points with abs(.std.res) >
3: 
  model.data %>% 
  filter(abs(.std.resid) > 3)  
 
 
 
 
 
 
 Create ROCR for training and test data 
  ## training data
pred.mtt = predict(m, type = "response") #repeat risk predictions from model m
rocr.pred.mtt = ROCR::prediction(pred.mtt, labels = ml_train$Health_Binary) #ROCR prediction object
roc.perf.mtt = ROCR::performance(rocr.pred.mtt, measure = "tpr", x.measure = "fpr") # #ROCR performance object
plot(roc.perf.mtt, col = "blue")


pred.te.1 = predict(m, newdata = ml_test, type = "response") #.te = "test"
rocr.pred.te.1 = ROCR::prediction(pred.te.1, labels = ml_test$Health_Binary)
roc.perf.te.1 = ROCR::performance(rocr.pred.te.1, measure = "tpr", x.measure = "fpr")
plot(roc.perf.te.1, col = "red", add = T)

abline(a = 0, b = 1, lty = 2) #diagonal for random assignment
legend("bottomright", legend = c("train","test"),
col = c("blue","red"), lty = c(2,1), lwd =1.5)  
   
 
 
 Report AUC from ROC for training and test data 
    # Train AUC
aucTr <- ROCR::performance(rocr.pred.mtt, measure = "auc")
  aucTr <- aucTr@y.values[[1]]
  print(aucTr)  
  ## [1] 0.5351616  
     # Test AUC
  aucTe <- ROCR::performance(rocr.pred.te.1, measure = "auc")
  aucTe <- aucTe@y.values[[1]]
  print(aucTe)  
  ## [1] 0.532578  
 
 
 
 INCOME Binary logistic regression ON SIGNIFICANT OR SERIOUS
ILLNESS 
  # fit binary logit model and store results 'm'
m <- glm(Health_Binary ~ Income, data = ml_train,family = binomial)
# view a summary of the model
summary(m)  
  ## 
## Call:
## glm(formula = Health_Binary ~ Income, family = binomial, data = ml_train)
## 
## Coefficients:
##             Estimate Std. Error z value Pr(>|z|)    
## (Intercept) -2.97289    0.14697 -20.228   <2e-16 ***
## Income.L    -0.04959    0.29686  -0.167    0.867    
## Income.Q    -0.03596    0.20365  -0.177    0.860    
## ---
## Signif. codes:  0 '***' 0.001 '**' 0.01 '*' 0.05 '.' 0.1 ' ' 1
## 
## (Dispersion parameter for binomial family taken to be 1)
## 
##     Null deviance: 652.97  on 1657  degrees of freedom
## Residual deviance: 652.91  on 1655  degrees of freedom
## AIC: 658.91
## 
## Number of Fisher Scoring iterations: 5  
  # test model fit
with(m, null.deviance - deviance)  
  ## [1] 0.05621822  
  with(m, df.null - df.residual)  
  ## [1] 2  
  with(m, pchisq(null.deviance - deviance, df.null - df.residual, lower.tail = FALSE))  
  ## [1] 0.9722823  
  BIC(m)  
  ## [1] 675.1501  
  # Hosmer-Lemeshow Goodness-of-Fit Test
glmtoolbox::hltest(m)  
  ## 
##    The Hosmer-Lemeshow goodness-of-fit test
## 
##  Group Size Observed Expected
##      1  237       11       11
##      2  262       13       13
##      3 1159       58       58
## 
##          Statistic =  0 
## degrees of freedom =  1 
##            p-value =  1  
  ## CIs using profiled log-likelihood
confint(m, level=0.99)  
  ## Waiting for profiling to be done...  
  ##                  0.5 %     99.5 %
## (Intercept) -3.3819062 -2.6191853
## Income.L    -0.8413580  0.7207372
## Income.Q    -0.5865219  0.4713246  
  ## CIs using standard errors
confint.default(m, level=0.99)  
  ##                  0.5 %     99.5 %
## (Intercept) -3.3514539 -2.5943290
## Income.L    -0.8142431  0.7150556
## Income.Q    -0.5605241  0.4886063  
  # Wald test
wald.test(b = coef(m), Sigma = vcov(m), Terms = 2)  
  ## Wald test:
## ----------
## 
## Chi-squared test:
## X2 = 0.028, df = 1, P(> X2) = 0.87  
  ## odds ratios and 95% CI
exp(cbind(OR = coef(m), confint(m, level=0.99)))  
  ## Waiting for profiling to be done...  
  ##                     OR      0.5 %    99.5 %
## (Intercept) 0.05115518 0.03398262 0.0728622
## Income.L    0.95161593 0.43112468 2.0559482
## Income.Q    0.96467992 0.55625864 1.6021150  
 
 Calculate Nagelkerke R^2 
  NagelkerkeR2(m)  
  ## $N
## [1] 1658
## 
## $R2
## [1] 0.0001041585  
 
 
 check assumptions of model 
 
 Cook’s distance 
  plot(m, which = 4, id.n = 3)  
   
 
 
 Extract model results and display data for top 3 values using Cook’s
distance 
  model.data <- augment(m) %>% 
  mutate(index = 1:n()) 
model.data %>% top_n(3, .cooksd)  
 
 
 
 
 
 plot standardised residuals 
  ggplot(model.data, aes(index, .std.resid)) + 
  geom_point(aes(color = Health_Binary), alpha = .5) +
  theme_bw()  
   
 
 
 Filter potential influential data points with abs(.std.res) >
3: 
  model.data %>% 
  filter(abs(.std.resid) > 3)  
 
 
 
 
 
 
 Create ROCR for training and test data 
  ## training data
pred.mtt = predict(m, type = "response") #repeat risk predictions from model m
rocr.pred.mtt = ROCR::prediction(pred.mtt, labels = ml_train$Health_Binary) #ROCR prediction object
roc.perf.mtt = ROCR::performance(rocr.pred.mtt, measure = "tpr", x.measure = "fpr") # #ROCR performance object
plot(roc.perf.mtt, col = "blue")


pred.te.1 = predict(m, newdata = ml_test, type = "response") #.te = "test"
rocr.pred.te.1 = ROCR::prediction(pred.te.1, labels = ml_test$Health_Binary)
roc.perf.te.1 = ROCR::performance(rocr.pred.te.1, measure = "tpr", x.measure = "fpr")
plot(roc.perf.te.1, col = "red", add = T)

abline(a = 0, b = 1, lty = 2) #diagonal for random assignment
legend("bottomright", legend = c("train","test"),
col = c("blue","red"), lty = c(2,1), lwd =1.5)  
   
 
 
 Report AUC from ROC for training and test data 
    # Train AUC
aucTr <- ROCR::performance(rocr.pred.mtt, measure = "auc")
  aucTr <- aucTr@y.values[[1]]
  print(aucTr)  
  ## [1] 0.5051264  
     # Test AUC
  aucTe <- ROCR::performance(rocr.pred.te.1, measure = "auc")
  aucTe <- aucTe@y.values[[1]]
  print(aucTe)  
  ## [1] 0.5136209  
 
 
 
 INCOME2 Binary logistic regression ON SIGNIFICANT OR SERIOUS
ILLNESS 
  # fit binary logit model and store results 'm'
m <- glm(Health_Binary ~ Income2, data = ml_train,family = binomial)
# view a summary of the model
summary(m)  
  ## 
## Call:
## glm(formula = Health_Binary ~ Income2, family = binomial, data = ml_train)
## 
## Coefficients:
##                Estimate Std. Error z value Pr(>|z|)    
## (Intercept)   -2.952504   0.284482 -10.379   <2e-16 ***
## Income2Medium  0.008972   0.314767   0.029    0.977    
## Income2High   -0.070136   0.419817  -0.167    0.867    
## ---
## Signif. codes:  0 '***' 0.001 '**' 0.01 '*' 0.05 '.' 0.1 ' ' 1
## 
## (Dispersion parameter for binomial family taken to be 1)
## 
##     Null deviance: 652.97  on 1657  degrees of freedom
## Residual deviance: 652.91  on 1655  degrees of freedom
## AIC: 658.91
## 
## Number of Fisher Scoring iterations: 5  
  # test model fit
with(m, null.deviance - deviance)  
  ## [1] 0.05621822  
  with(m, df.null - df.residual)  
  ## [1] 2  
  with(m, pchisq(null.deviance - deviance, df.null - df.residual, lower.tail = FALSE))  
  ## [1] 0.9722823  
  BIC(m)  
  ## [1] 675.1501  
  # Hosmer-Lemeshow Goodness-of-Fit Test
glmtoolbox::hltest(m)  
  ## 
##    The Hosmer-Lemeshow goodness-of-fit test
## 
##  Group Size Observed Expected
##      1  237       11       11
##      2  262       13       13
##      3 1159       58       58
## 
##          Statistic =  0 
## degrees of freedom =  1 
##            p-value =  1  
  ## CIs using profiled log-likelihood
confint(m, level=0.99)  
  ## Waiting for profiling to be done...  
  ##                    0.5 %    99.5 %
## (Intercept)   -3.7774335 -2.290924
## Income2Medium -0.7496268  0.894922
## Income2High   -1.1898598  1.019276  
  ## CIs using standard errors
confint.default(m, level=0.99)  
  ##                    0.5 %     99.5 %
## (Intercept)   -3.6852818 -2.2197253
## Income2Medium -0.8018128  0.8197576
## Income2High   -1.1515137  1.0112413  
  # Wald test
wald.test(b = coef(m), Sigma = vcov(m), Terms = 2)  
  ## Wald test:
## ----------
## 
## Chi-squared test:
## X2 = 0.00081, df = 1, P(> X2) = 0.98  
  ## odds ratios and 95% CI
exp(cbind(OR = coef(m), confint(m, level=0.99)))  
  ## Waiting for profiling to be done...  
  ##                       OR      0.5 %    99.5 %
## (Intercept)   0.05220884 0.02288134 0.1011729
## Income2Medium 1.00901278 0.47254285 2.4471450
## Income2High   0.93226686 0.30426391 2.7711884  
 
 Calculate Nagelkerke R^2 
  NagelkerkeR2(m)  
  ## $N
## [1] 1658
## 
## $R2
## [1] 0.0001041585  
 
 
 check assumptions of model 
 
 Cook’s distance 
  plot(m, which = 4, id.n = 3)  
   
 
 
 Extract model results and display data for top 3 values using Cook’s
distance 
  model.data <- augment(m) %>% 
  mutate(index = 1:n()) 
model.data %>% top_n(3, .cooksd)  
 
 
 
 
 
 plot standardised residuals 
  ggplot(model.data, aes(index, .std.resid)) + 
  geom_point(aes(color = Health_Binary), alpha = .5) +
  theme_bw()  
   
 
 
 Filter potential influential data points with abs(.std.res) >
3: 
  model.data %>% 
  filter(abs(.std.resid) > 3)  
 
 
 
 
 
 
 Create ROCR for training and test data 
  ## training data
pred.mtt = predict(m, type = "response") #repeat risk predictions from model m
rocr.pred.mtt = ROCR::prediction(pred.mtt, labels = ml_train$Health_Binary) #ROCR prediction object
roc.perf.mtt = ROCR::performance(rocr.pred.mtt, measure = "tpr", x.measure = "fpr") # #ROCR performance object
plot(roc.perf.mtt, col = "blue")


pred.te.1 = predict(m, newdata = ml_test, type = "response") #.te = "test"
rocr.pred.te.1 = ROCR::prediction(pred.te.1, labels = ml_test$Health_Binary)
roc.perf.te.1 = ROCR::performance(rocr.pred.te.1, measure = "tpr", x.measure = "fpr")
plot(roc.perf.te.1, col = "red", add = T)

abline(a = 0, b = 1, lty = 2) #diagonal for random assignment
legend("bottomright", legend = c("train","test"),
col = c("blue","red"), lty = c(2,1), lwd =1.5)  
   
 
 
 Report AUC from ROC for training and test data 
    # Train AUC
aucTr <- ROCR::performance(rocr.pred.mtt, measure = "auc")
  aucTr <- aucTr@y.values[[1]]
  print(aucTr)  
  ## [1] 0.5051264  
     # Test AUC
  aucTe <- ROCR::performance(rocr.pred.te.1, measure = "auc")
  aucTe <- aucTe@y.values[[1]]
  print(aucTe)  
  ## [1] 0.5136209  
 
 
 
 C_AGE Binary logistic regression ON SIGNIFICANT OR SERIOUS
ILLNESS 
  # fit binary logit model and store results 'm'
m <- glm(Health_Binary ~ C_Age, data = ml_train,family = binomial)
# view a summary of the model
summary(m)  
  ## 
## Call:
## glm(formula = Health_Binary ~ C_Age, family = binomial, data = ml_train)
## 
## Coefficients:
##             Estimate Std. Error z value Pr(>|z|)    
## (Intercept)  -2.9838     0.1168 -25.537   <2e-16 ***
## C_Age.L      -0.1974     0.2823  -0.699    0.484    
## C_Age.Q      -0.2860     0.2751  -1.040    0.298    
## C_Age.C       0.1177     0.2412   0.488    0.626    
## C_Age^4      -0.2556     0.2440  -1.048    0.295    
## ---
## Signif. codes:  0 '***' 0.001 '**' 0.01 '*' 0.05 '.' 0.1 ' ' 1
## 
## (Dispersion parameter for binomial family taken to be 1)
## 
##     Null deviance: 652.97  on 1657  degrees of freedom
## Residual deviance: 649.71  on 1653  degrees of freedom
## AIC: 659.71
## 
## Number of Fisher Scoring iterations: 6  
  # test model fit
with(m, null.deviance - deviance)  
  ## [1] 3.251264  
  with(m, df.null - df.residual)  
  ## [1] 4  
  with(m, pchisq(null.deviance - deviance, df.null - df.residual, lower.tail = FALSE))  
  ## [1] 0.5166909  
  BIC(m)  
  ## [1] 686.7818  
  # Hosmer-Lemeshow Goodness-of-Fit Test
hltest(m, G=4)  
  ## 
##    The Hosmer-Lemeshow goodness-of-fit test
## 
##  Group Size Observed Expected
##      1  323       12       12
##      2  273       12       12
##      3  342       16       16
##      4  372       19       19
##      5  348       23       23
## 
##          Statistic =  0 
## degrees of freedom =  3 
##            p-value =  1  
  ## CIs using profiled log-likelihood
confint(m, level=0.99)  
  ## Waiting for profiling to be done...  
  ##                  0.5 %     99.5 %
## (Intercept) -3.3003207 -2.6965181
## C_Age.L     -0.9370189  0.5414777
## C_Age.Q     -1.0222341  0.4081862
## C_Age.C     -0.5041754  0.7481486
## C_Age^4     -0.9088355  0.3611786  
  ## CIs using standard errors
confint.default(m, level=0.99)  
  ##                  0.5 %     99.5 %
## (Intercept) -3.2847985 -2.6828690
## C_Age.L     -0.9245325  0.5297265
## C_Age.Q     -0.9944603  0.4225091
## C_Age.C     -0.5036236  0.7389753
## C_Age^4     -0.8840710  0.3727733  
  # Wald test
wald.test(b = coef(m), Sigma = vcov(m), Terms = 2)  
  ## Wald test:
## ----------
## 
## Chi-squared test:
## X2 = 0.49, df = 1, P(> X2) = 0.48  
  ## odds ratios and 95% CI
exp(cbind(OR = coef(m), confint(m, level=0.99)))  
  ## Waiting for profiling to be done...  
  ##                     OR      0.5 %     99.5 %
## (Intercept) 0.05059848 0.03687134 0.06743992
## C_Age.L     0.82085975 0.39179408 1.71854443
## C_Age.Q     0.75128095 0.35979023 1.50408720
## C_Age.C     1.12487942 0.60400343 2.11308431
## C_Age^4     0.77441385 0.40299325 1.43501975  
 
 Calculate Nagelkerke R^2 
  NagelkerkeR2(m)  
  ## $N
## [1] 1658
## 
## $R2
## [1] 0.006017989  
 
 
 check assumptions of model 
 
 Cook’s distance 
  plot(m, which = 4, id.n = 3)  
   
 
 
 Extract model results and display data for top 3 values using Cook’s
distance 
  model.data <- augment(m) %>% 
  mutate(index = 1:n()) 
model.data %>% top_n(3, .cooksd)  
 
 
 
 
 
 plot standardised residuals 
  ggplot(model.data, aes(index, .std.resid)) + 
  geom_point(aes(color = Health_Binary), alpha = .5) +
  theme_bw()  
   
 
 
 Filter potential influential data points with abs(.std.res) >
3: 
  model.data %>% 
  filter(abs(.std.resid) > 3)  
 
 
 
 
 
 
 Create ROCR for training and test data 
  ## training data
pred.mtt = predict(m, type = "response") #repeat risk predictions from model m
rocr.pred.mtt = ROCR::prediction(pred.mtt, labels = ml_train$Health_Binary) #ROCR prediction object
roc.perf.mtt = ROCR::performance(rocr.pred.mtt, measure = "tpr", x.measure = "fpr") # #ROCR performance object
plot(roc.perf.mtt, col = "blue")


pred.te.1 = predict(m, newdata = ml_test, type = "response") #.te = "test"
rocr.pred.te.1 = ROCR::prediction(pred.te.1, labels = ml_test$Health_Binary)
roc.perf.te.1 = ROCR::performance(rocr.pred.te.1, measure = "tpr", x.measure = "fpr")
plot(roc.perf.te.1, col = "red", add = T)

abline(a = 0, b = 1, lty = 2) #diagonal for random assignment
legend("bottomright", legend = c("train","test"),
col = c("blue","red"), lty = c(2,1), lwd =1.5)  
   
 
 
 Report AUC from ROC for training and test data 
    # Train AUC
aucTr <- ROCR::performance(rocr.pred.mtt, measure = "auc")
  aucTr <- aucTr@y.values[[1]]
  print(aucTr)  
  ## [1] 0.5559768  
     # Test AUC
  aucTe <- ROCR::performance(rocr.pred.te.1, measure = "auc")
  aucTe <- aucTe@y.values[[1]]
  print(aucTe)  
  ## [1] 0.4879386  
 
 
 
 C_AGE2 Binary logistic regression ON SIGNIFICANT OR SERIOUS
ILLNESS 
  # fit binary logit model and store results 'm'
m <- glm(Health_Binary ~ C_Age2, data = ml_train,family = binomial)
# view a summary of the model
summary(m)  
  ## 
## Call:
## glm(formula = Health_Binary ~ C_Age2, family = binomial, data = ml_train)
## 
## Coefficients:
##             Estimate Std. Error z value Pr(>|z|)    
## (Intercept) -3.07961    0.29524 -10.431   <2e-16 ***
## C_Age230–39  0.43128    0.36568   1.179    0.238    
## C_Age240–49  0.06531    0.39081   0.167    0.867    
## C_Age250–59  0.15758    0.37766   0.417    0.676    
## C_Age260<   -0.17527    0.41679  -0.421    0.674    
## ---
## Signif. codes:  0 '***' 0.001 '**' 0.01 '*' 0.05 '.' 0.1 ' ' 1
## 
## (Dispersion parameter for binomial family taken to be 1)
## 
##     Null deviance: 652.97  on 1657  degrees of freedom
## Residual deviance: 649.71  on 1653  degrees of freedom
## AIC: 659.71
## 
## Number of Fisher Scoring iterations: 6  
  # test model fit
with(m, null.deviance - deviance)  
  ## [1] 3.251264  
  with(m, df.null - df.residual)  
  ## [1] 4  
  with(m, pchisq(null.deviance - deviance, df.null - df.residual, lower.tail = FALSE))  
  ## [1] 0.5166909  
  BIC(m)  
  ## [1] 686.7818  
  # Hosmer-Lemeshow Goodness-of-Fit Test
hltest(m, G=4)  
  ## 
##    The Hosmer-Lemeshow goodness-of-fit test
## 
##  Group Size Observed Expected
##      1  323       12       12
##      2  273       12       12
##      3  342       16       16
##      4  372       19       19
##      5  348       23       23
## 
##          Statistic =  0 
## degrees of freedom =  3 
##            p-value =  1  
  ## CIs using profiled log-likelihood
confint(m, level=0.99)  
  ## Waiting for profiling to be done...  
  ##                  0.5 %     99.5 %
## (Intercept) -3.9409874 -2.3965338
## C_Age230–39 -0.4835411  1.4317384
## C_Age240–49 -0.9376660  1.1133979
## C_Age250–59 -0.7992290  1.1807471
## C_Age260<   -1.2719374  0.9213437  
  ## CIs using standard errors
confint.default(m, level=0.99)  
  ##                  0.5 %     99.5 %
## (Intercept) -3.8400933 -2.3191342
## C_Age230–39 -0.5106397  1.3732053
## C_Age240–49 -0.9413540  1.0719642
## C_Age250–59 -0.8152102  1.1303796
## C_Age260<   -1.2488511  0.8983061  
  # Wald test
wald.test(b = coef(m), Sigma = vcov(m), Terms = 2)  
  ## Wald test:
## ----------
## 
## Chi-squared test:
## X2 = 1.4, df = 1, P(> X2) = 0.24  
  ## odds ratios and 95% CI
exp(cbind(OR = coef(m), confint(m, level=0.99)))  
  ## Waiting for profiling to be done...  
  ##                     OR      0.5 %     99.5 %
## (Intercept) 0.04597701 0.01942902 0.09103295
## C_Age230–39 1.53923077 0.61659607 4.18596985
## C_Age240–49 1.06748466 0.39154064 3.04468634
## C_Age250–59 1.17067989 0.44967554 3.25680649
## C_Age260<   0.83922830 0.28028807 2.51266440  
 
 
 Calculate Nagelkerke R^2 
  NagelkerkeR2(m)  
  ## $N
## [1] 1658
## 
## $R2
## [1] 0.006017989  
 
 
 check assumptions of model 
 
 Cook’s distance 
  plot(m, which = 4, id.n = 3)  
   
 
 
 Extract model results and display data for top 3 values using Cook’s
distance 
  model.data <- augment(m) %>% 
  mutate(index = 1:n()) 
model.data %>% top_n(3, .cooksd)  
 
 
 
 
 
 plot standardised residuals 
  ggplot(model.data, aes(index, .std.resid)) + 
  geom_point(aes(color = Health_Binary), alpha = .5) +
  theme_bw()  
   
 
 
 Filter potential influential data points with abs(.std.res) >
3: 
  model.data %>% 
  filter(abs(.std.resid) > 3)  
 
 
 
 
 
 Create ROCR for training and test data 
  ## training data
pred.mtt = predict(m, type = "response") #repeat risk predictions from model m
rocr.pred.mtt = ROCR::prediction(pred.mtt, labels = ml_train$Health_Binary) #ROCR prediction object
roc.perf.mtt = ROCR::performance(rocr.pred.mtt, measure = "tpr", x.measure = "fpr") # #ROCR performance object
plot(roc.perf.mtt, col = "blue")


pred.te.1 = predict(m, newdata = ml_test, type = "response") #.te = "test"
rocr.pred.te.1 = ROCR::prediction(pred.te.1, labels = ml_test$Health_Binary)
roc.perf.te.1 = ROCR::performance(rocr.pred.te.1, measure = "tpr", x.measure = "fpr")
plot(roc.perf.te.1, col = "red", add = T)

abline(a = 0, b = 1, lty = 2) #diagonal for random assignment
legend("bottomright", legend = c("train","test"),
col = c("blue","red"), lty = c(2,1), lwd =1.5)  
   
 
 
 Report AUC from ROC for training and test data 
    # Train AUC
aucTr <- ROCR::performance(rocr.pred.mtt, measure = "auc")
  aucTr <- aucTr@y.values[[1]]
  print(aucTr)  
  ## [1] 0.5559768  
     # Test AUC
  aucTe <- ROCR::performance(rocr.pred.te.1, measure = "auc")
  aucTe <- aucTe@y.values[[1]]
  print(aucTe)  
  ## [1] 0.4879386  
 
 
 
 C_GENDER Binary logistic regression ON SIGNIFICANT OR SERIOUS
ILLNESS 
  # fit binary logit model and store results 'm'
m <- glm(Health_Binary ~ C_Gender, data = ml_train,family = binomial)
# view a summary of the model
summary(m)  
  ## 
## Call:
## glm(formula = Health_Binary ~ C_Gender, family = binomial, data = ml_train)
## 
## Coefficients:
##              Estimate Std. Error z value Pr(>|z|)    
## (Intercept)   -2.9336     0.1162 -25.246   <2e-16 ***
## C_GenderMale  -0.3806     0.5221  -0.729    0.466    
## ---
## Signif. codes:  0 '***' 0.001 '**' 0.01 '*' 0.05 '.' 0.1 ' ' 1
## 
## (Dispersion parameter for binomial family taken to be 1)
## 
##     Null deviance: 652.97  on 1657  degrees of freedom
## Residual deviance: 652.37  on 1656  degrees of freedom
## AIC: 656.37
## 
## Number of Fisher Scoring iterations: 6  
  # test model fit
with(m, null.deviance - deviance)  
  ## [1] 0.5915496  
  with(m, df.null - df.residual)  
  ## [1] 1  
  with(m, pchisq(null.deviance - deviance, df.null - df.residual, lower.tail = FALSE))  
  ## [1] 0.4418205  
  BIC(m)  
  ## [1] 667.2014  
  ## CIs using profiled log-likelihood
confint(m, level=0.99)  
  ## Waiting for profiling to be done...  
  ##                  0.5 %     99.5 %
## (Intercept)  -3.247064 -2.6470157
## C_GenderMale -2.050946  0.7632282  
  ## CIs using standard errors
confint.default(m, level=0.99)  
  ##                  0.5 %     99.5 %
## (Intercept)  -3.232898 -2.6342705
## C_GenderMale -1.725455  0.9642508  
  # Wald test
wald.test(b = coef(m), Sigma = vcov(m), Terms = 2)  
  ## Wald test:
## ----------
## 
## Chi-squared test:
## X2 = 0.53, df = 1, P(> X2) = 0.47  
  ## odds ratios and 95% CI
exp(cbind(OR = coef(m), confint(m, level=0.99)))  
  ## Waiting for profiling to be done...  
  ##                     OR      0.5 %     99.5 %
## (Intercept)  0.0532060 0.03888821 0.07086237
## C_GenderMale 0.6834499 0.12861321 2.14519025  
 
 Calculate Nagelkerke R^2 
  NagelkerkeR2(m)  
  ## $N
## [1] 1658
## 
## $R2
## [1] 0.001095818  
 
 
 check assumptions of model 
 
 Cook’s distance 
  plot(m, which = 4, id.n = 3)  
   
 
 
 Extract model results and display data for top 3 values using Cook’s
distance 
  model.data <- augment(m) %>% 
  mutate(index = 1:n()) 
model.data %>% top_n(3, .cooksd)  
 
 
 
 
 
 plot standardised residuals 
  ggplot(model.data, aes(index, .std.resid)) + 
  geom_point(aes(color = Health_Binary), alpha = .5) +
  theme_bw()  
   
 
 
 Filter potential influential data points with abs(.std.res) >
3: 
  model.data %>% 
  filter(abs(.std.resid) > 3)  
 
 
 
 
 
 
 Create ROCR for training and test data 
  ## training data
pred.mtt = predict(m, type = "response") #repeat risk predictions from model m
rocr.pred.mtt = ROCR::prediction(pred.mtt, labels = ml_train$Health_Binary) #ROCR prediction object
roc.perf.mtt = ROCR::performance(rocr.pred.mtt, measure = "tpr", x.measure = "fpr") # #ROCR performance object
plot(roc.perf.mtt, col = "blue")


pred.te.1 = predict(m, newdata = ml_test, type = "response") #.te = "test"
rocr.pred.te.1 = ROCR::prediction(pred.te.1, labels = ml_test$Health_Binary)
roc.perf.te.1 = ROCR::performance(rocr.pred.te.1, measure = "tpr", x.measure = "fpr")
plot(roc.perf.te.1, col = "red", add = T)

abline(a = 0, b = 1, lty = 2) #diagonal for random assignment
legend("bottomright", legend = c("train","test"),
col = c("blue","red"), lty = c(2,1), lwd =1.5)  
   
 
 
 Report AUC from ROC for training and test data 
    # Train AUC
aucTr <- ROCR::performance(rocr.pred.mtt, measure = "auc")
  aucTr <- aucTr@y.values[[1]]
  print(aucTr)  
  ## [1] 0.5105082  
     # Test AUC
  aucTe <- ROCR::performance(rocr.pred.te.1, measure = "auc")
  aucTe <- aucTe@y.values[[1]]
  print(aucTe)  
  ## [1] 0.5005117  
 
 
 
 
 NOW CHECK ASSOCIATIONS BETWEEN SIGNIFICANT OR SERIOUS ILLNESS AND
DOG CHARACTERISTICS - simple BINARY LOGISTIC regression 
 
 DOG DIET VEGAN binary regression for ON SIGNIFICANT OR SERIOUS
ILLNESS 
  # fit binary logit model and store results 'm'
m <- glm(Health_Binary ~ D_Diet_Vegan, data = ml_train,family = binomial)
# view a summary of the model
summary(m)  
  ## 
## Call:
## glm(formula = Health_Binary ~ D_Diet_Vegan, family = binomial, 
##     data = ml_train)
## 
## Coefficients:
##                 Estimate Std. Error z value Pr(>|z|)    
## (Intercept)      -2.9094     0.1186 -24.537   <2e-16 ***
## D_Diet_VeganYes  -0.4430     0.4024  -1.101    0.271    
## ---
## Signif. codes:  0 '***' 0.001 '**' 0.01 '*' 0.05 '.' 0.1 ' ' 1
## 
## (Dispersion parameter for binomial family taken to be 1)
## 
##     Null deviance: 652.97  on 1657  degrees of freedom
## Residual deviance: 651.61  on 1656  degrees of freedom
## AIC: 655.61
## 
## Number of Fisher Scoring iterations: 6  
  # test model fit
with(m, null.deviance - deviance)  
  ## [1] 1.357535  
  with(m, df.null - df.residual)  
  ## [1] 1  
  with(m, pchisq(null.deviance - deviance, df.null - df.residual, lower.tail = FALSE))  
  ## [1] 0.2439652  
  BIC(m)  
  ## [1] 666.4354  
  ## CIs using profiled log-likelihood
confint(m, level=0.99)  
  ## Waiting for profiling to be done...  
  ##                     0.5 %     99.5 %
## (Intercept)     -3.229608 -2.6172396
## D_Diet_VeganYes -1.648358  0.4791607  
  ## CIs using standard errors
confint.default(m, level=0.99)  
  ##                     0.5 %     99.5 %
## (Intercept)     -3.214877 -2.6040184
## D_Diet_VeganYes -1.479445  0.5935266  
  # Wald test
wald.test(b = coef(m), Sigma = vcov(m), Terms = 2)  
  ## Wald test:
## ----------
## 
## Chi-squared test:
## X2 = 1.2, df = 1, P(> X2) = 0.27  
  ## odds ratios and 95% CI
exp(cbind(OR = coef(m), confint(m, level=0.99)))  
  ## Waiting for profiling to be done...  
  ##                         OR     0.5 %     99.5 %
## (Intercept)     0.05450581 0.0395730 0.07300411
## D_Diet_VeganYes 0.64213333 0.1923654 1.61471861  
 
 Calculate Nagelkerke R^2 
  NagelkerkeR2(m)  
  ## $N
## [1] 1658
## 
## $R2
## [1] 0.002514189  
 
 
 check assumptions of model 
 
 Cook’s distance 
  plot(m, which = 4, id.n = 3)  
   
 
 
 Extract model results and display data for top 3 values using Cook’s
distance 
  model.data <- augment(m) %>% 
  mutate(index = 1:n()) 
model.data %>% top_n(3, .cooksd)  
 
 
 
 
 
 plot standardised residuals 
  ggplot(model.data, aes(index, .std.resid)) + 
  geom_point(aes(color = Health_Binary), alpha = .5) +
  theme_bw()  
   
 
 
 Filter potential influential data points with abs(.std.res) >
3: 
  model.data %>% 
  filter(abs(.std.resid) > 3)  
 
 
 
 
 
 
 Create ROCR for training and test data 
  ## training data
pred.mtt = predict(m, type = "response") #repeat risk predictions from model mtt
rocr.pred.mtt = ROCR::prediction(pred.mtt, labels = ml_train$Health_Binary) #ROCR prediction object
roc.perf.mtt = ROCR::performance(rocr.pred.mtt, measure = "tpr", x.measure = "fpr") # #ROCR performance object
plot(roc.perf.mtt, col = "blue")


pred.te.1 = predict(m, newdata = ml_test, type = "response") #.te = "test"
rocr.pred.te.1 = ROCR::prediction(pred.te.1, labels = ml_test$Health_Binary)
roc.perf.te.1 = ROCR::performance(rocr.pred.te.1, measure = "tpr", x.measure = "fpr")
plot(roc.perf.te.1, col = "red", add = T)

abline(a = 0, b = 1, lty = 2) #diagonal for random assignment
legend("bottomright", legend = c("train","test"),
col = c("blue","red"), lty = c(2,1), lwd =1.5)  
   
 
 
 Report AUC from ROC for training and test data 
    # Train AUC
aucTr <- ROCR::performance(rocr.pred.mtt, measure = "auc")
  aucTr <- aucTr@y.values[[1]]
  print(aucTr)  
  ## [1] 0.5207688  
     # Test AUC
  aucTe <- ROCR::performance(rocr.pred.te.1, measure = "auc")
  aucTe <- aucTe@y.values[[1]]
  print(aucTe)  
  ## [1] 0.5183967  
 
 
 
 DOG DIET VEGAN_VEGGIE binary regression for ON SIGNIFICANT OR
SERIOUS ILLNESS 
  # fit binary logit model and store results 'm'
m <- glm(Health_Binary ~ D_Diet_Vegan_Veggie, data = ml_train,family = binomial)
# view a summary of the model
summary(m)  
  ## 
## Call:
## glm(formula = Health_Binary ~ D_Diet_Vegan_Veggie, family = binomial, 
##     data = ml_train)
## 
## Coefficients:
##                        Estimate Std. Error z value Pr(>|z|)    
## (Intercept)             -2.9189     0.1202  -24.29   <2e-16 ***
## D_Diet_Vegan_VeggieYes  -0.2955     0.3605   -0.82    0.412    
## ---
## Signif. codes:  0 '***' 0.001 '**' 0.01 '*' 0.05 '.' 0.1 ' ' 1
## 
## (Dispersion parameter for binomial family taken to be 1)
## 
##     Null deviance: 652.97  on 1657  degrees of freedom
## Residual deviance: 652.25  on 1656  degrees of freedom
## AIC: 656.25
## 
## Number of Fisher Scoring iterations: 5  
  # test model fit
with(m, null.deviance - deviance)  
  ## [1] 0.7207086  
  with(m, df.null - df.residual)  
  ## [1] 1  
  with(m, pchisq(null.deviance - deviance, df.null - df.residual, lower.tail = FALSE))  
  ## [1] 0.3959116  
  BIC(m)  
  ## [1] 667.0723  
  ## CIs using profiled log-likelihood
confint(m, level=0.99)  
  ## Waiting for profiling to be done...  
  ##                            0.5 %     99.5 %
## (Intercept)            -3.243545 -2.6229519
## D_Diet_Vegan_VeggieYes -1.348762  0.5466256  
  ## CIs using standard errors
confint.default(m, level=0.99)  
  ##                            0.5 %     99.5 %
## (Intercept)            -3.228378 -2.6093838
## D_Diet_Vegan_VeggieYes -1.224040  0.6329595  
  # Wald test
wald.test(b = coef(m), Sigma = vcov(m), Terms = 2)  
  ## Wald test:
## ----------
## 
## Chi-squared test:
## X2 = 0.67, df = 1, P(> X2) = 0.41  
  ## odds ratios and 95% CI
exp(cbind(OR = coef(m), confint(m, level=0.99)))  
  ## Waiting for profiling to be done...  
  ##                                OR      0.5 %     99.5 %
## (Intercept)            0.05399408 0.03902531 0.07258827
## D_Diet_Vegan_VeggieYes 0.74412933 0.25956128 1.72741427  
 
 Calculate Nagelkerke R^2 
  NagelkerkeR2(m)  
  ## $N
## [1] 1658
## 
## $R2
## [1] 0.001335027  
 
 
 check assumptions of model 
 
 Cook’s distance 
  plot(m, which = 4, id.n = 3)  
   
 
 
 Extract model results and display data for top 3 values using Cook’s
distance 
  model.data <- augment(m) %>% 
  mutate(index = 1:n()) 
model.data %>% top_n(3, .cooksd)  
 
 
 
 
 
 plot standardised residuals 
  ggplot(model.data, aes(index, .std.resid)) + 
  geom_point(aes(color = Health_Binary), alpha = .5) +
  theme_bw()  
   
 
 
 Filter potential influential data points with abs(.std.res) >
3: 
  model.data %>% 
  filter(abs(.std.resid) > 3)  
 
 
 
 
 
 
 Create ROCR for training and test data 
  ## training data
pred.mtt = predict(m, type = "response") #repeat risk predictions from model m
rocr.pred.mtt = ROCR::prediction(pred.mtt, labels = ml_train$Health_Binary) #ROCR prediction object
roc.perf.mtt = ROCR::performance(rocr.pred.mtt, measure = "tpr", x.measure = "fpr") # #ROCR performance object
plot(roc.perf.mtt, col = "blue")


pred.te.1 = predict(m, newdata = ml_test, type = "response") #.te = "test"
rocr.pred.te.1 = ROCR::prediction(pred.te.1, labels = ml_test$Health_Binary)
roc.perf.te.1 = ROCR::performance(rocr.pred.te.1, measure = "tpr", x.measure = "fpr")
plot(roc.perf.te.1, col = "red", add = T)

abline(a = 0, b = 1, lty = 2) #diagonal for random assignment
legend("bottomright", legend = c("train","test"),
col = c("blue","red"), lty = c(2,1), lwd =1.5)  
   
 
 
 Report AUC from ROC for training and test data 
    # Train AUC
aucTr <- ROCR::performance(rocr.pred.mtt, measure = "auc")
  aucTr <- aucTr@y.values[[1]]
  print(aucTr)  
  ## [1] 0.5161879  
     # Test AUC
  aucTe <- ROCR::performance(rocr.pred.te.1, measure = "auc")
  aucTe <- aucTe@y.values[[1]]
  print(aucTe)  
  ## [1] 0.51077  
 
 
 
 DOG DIET binary regression ON SIGNIFICANT OR SERIOUS ILLNESS 
  # fit binary logit model and store results 'm'
m <- glm(Health_Binary ~ D_Diet, data = ml_train,family = binomial)
# view a summary of the model
summary(m)  
  ## 
## Call:
## glm(formula = Health_Binary ~ D_Diet, family = binomial, data = ml_train)
## 
## Coefficients:
##                                                         Estimate Std. Error
## (Intercept)                                              -2.7230     0.1418
## D_DietMeat-based – raw                                   -0.5820     0.2682
## D_DietVegan (consuming no animal products)               -0.6294     0.4098
## D_DietVegetarian (including eggs or milk, but not meat)   0.2381     0.7495
##                                                         z value Pr(>|z|)    
## (Intercept)                                             -19.203   <2e-16 ***
## D_DietMeat-based – raw                                   -2.170    0.030 *  
## D_DietVegan (consuming no animal products)               -1.536    0.125    
## D_DietVegetarian (including eggs or milk, but not meat)   0.318    0.751    
## ---
## Signif. codes:  0 '***' 0.001 '**' 0.01 '*' 0.05 '.' 0.1 ' ' 1
## 
## (Dispersion parameter for binomial family taken to be 1)
## 
##     Null deviance: 652.97  on 1657  degrees of freedom
## Residual deviance: 646.25  on 1654  degrees of freedom
## AIC: 654.25
## 
## Number of Fisher Scoring iterations: 6  
  # test model fit
with(m, null.deviance - deviance)  
  ## [1] 6.712862  
  with(m, df.null - df.residual)  
  ## [1] 3  
  with(m, pchisq(null.deviance - deviance, df.null - df.residual, lower.tail = FALSE))  
  ## [1] 0.08163535  
  BIC(m)  
  ## [1] 675.9068  
  ## CIs using profiled log-likelihood
confint(m, level=0.99)  
  ## Waiting for profiling to be done...  
  ##                                                             0.5 %      99.5 %
## (Intercept)                                             -3.109132 -2.37606095
## D_DietMeat-based – raw                                  -1.312912  0.08211995
## D_DietVegan (consuming no animal products)              -1.848377  0.31847531
## D_DietVegetarian (including eggs or milk, but not meat) -2.401084  1.81918816  
  ## CIs using standard errors
confint.default(m, level=0.99)  
  ##                                                             0.5 %     99.5 %
## (Intercept)                                             -3.088283 -2.3577806
## D_DietMeat-based – raw                                  -1.272910  0.1088669
## D_DietVegan (consuming no animal products)              -1.685038  0.4262875
## D_DietVegetarian (including eggs or milk, but not meat) -1.692499  2.1687495  
  # Wald test
wald.test(b = coef(m), Sigma = vcov(m), Terms = 2)  
  ## Wald test:
## ----------
## 
## Chi-squared test:
## X2 = 4.7, df = 1, P(> X2) = 0.03  
  ## odds ratios and 95% CI
exp(cbind(OR = coef(m), confint(m, level=0.99)))  
  ## Waiting for profiling to be done...  
  ##                                                                 OR      0.5 %
## (Intercept)                                             0.06567534 0.04463970
## D_DietMeat-based – raw                                  0.55876753 0.26903554
## D_DietVegan (consuming no animal products)              0.53292453 0.15749254
## D_DietVegetarian (including eggs or milk, but not meat) 1.26886792 0.09061964
##                                                             99.5 %
## (Intercept)                                             0.09291586
## D_DietMeat-based – raw                                  1.08558602
## D_DietVegan (consuming no animal products)              1.37502967
## D_DietVegetarian (including eggs or milk, but not meat) 6.16684992  
 
 Calculate Nagelkerke R^2 
  NagelkerkeR2(m)  
  ## $N
## [1] 1658
## 
## $R2
## [1] 0.01241234  
 
 
 check assumptions of model 
 
 Cook’s distance 
  plot(m, which = 4, id.n = 3)  
   
 
 
 Extract model results and display data for top 3 values using Cook’s
distance 
  model.data <- augment(m) %>% 
  mutate(index = 1:n()) 
model.data %>% top_n(3, .cooksd)  
 
 
 
 
 
 plot standardised residuals 
  ggplot(model.data, aes(index, .std.resid)) + 
  geom_point(aes(color = Health_Binary), alpha = .5) +
  theme_bw()  
   
 
 
 Filter potential influential data points with abs(.std.res) >
3: 
  model.data %>% 
  filter(abs(.std.resid) > 3)  
 
 
 
 
 
 
 Create ROCR for training and test data 
  ## training data
pred.mtt = predict(m, type = "response") #repeat risk predictions from model m
rocr.pred.mtt = ROCR::prediction(pred.mtt, labels = ml_train$Health_Binary) #ROCR prediction object
roc.perf.mtt = ROCR::performance(rocr.pred.mtt, measure = "tpr", x.measure = "fpr") # #ROCR performance object
plot(roc.perf.mtt, col = "blue")


pred.te.1 = predict(m, newdata = ml_test, type = "response") #.te = "test"
rocr.pred.te.1 = ROCR::prediction(pred.te.1, labels = ml_test$Health_Binary)
roc.perf.te.1 = ROCR::performance(rocr.pred.te.1, measure = "tpr", x.measure = "fpr")
plot(roc.perf.te.1, col = "red", add = T)

abline(a = 0, b = 1, lty = 2) #diagonal for random assignment
legend("bottomright", legend = c("train","test"),
col = c("blue","red"), lty = c(2,1), lwd =1.5)  
   
 
 
 Report AUC from ROC for training and test data 
    # Train AUC
aucTr <- ROCR::performance(rocr.pred.mtt, measure = "auc")
  aucTr <- aucTr@y.values[[1]]
  print(aucTr)  
  ## [1] 0.5737627  
     # Test AUC
  aucTe <- ROCR::performance(rocr.pred.te.1, measure = "auc")
  aucTe <- aucTe@y.values[[1]]
  print(aucTe)  
  ## [1] 0.5958577  
 
 
 
 CLIENT DIET VEGAN + DOG DIET VEGAN binaryl regression ON SIGNIFICANT
OR SERIOUS ILLNESS 
  # fit binary logit model and store results 'm'
m <- glm(Health_Binary ~ C_Diet_Vegan + D_Diet_Vegan, data = ml_train,family = binomial)
# view a summary of the model
summary(m)  
  ## 
## Call:
## glm(formula = Health_Binary ~ C_Diet_Vegan + D_Diet_Vegan, family = binomial, 
##     data = ml_train)
## 
## Coefficients:
##                 Estimate Std. Error z value Pr(>|z|)    
## (Intercept)      -2.9585     0.1282 -23.073   <2e-16 ***
## C_Diet_VeganYes   0.3803     0.3275   1.161    0.246    
## D_Diet_VeganYes  -0.7507     0.4802  -1.563    0.118    
## ---
## Signif. codes:  0 '***' 0.001 '**' 0.01 '*' 0.05 '.' 0.1 ' ' 1
## 
## (Dispersion parameter for binomial family taken to be 1)
## 
##     Null deviance: 652.97  on 1657  degrees of freedom
## Residual deviance: 650.35  on 1655  degrees of freedom
## AIC: 656.35
## 
## Number of Fisher Scoring iterations: 6  
  # test model fit
with(m, null.deviance - deviance)  
  ## [1] 2.613526  
  with(m, df.null - df.residual)  
  ## [1] 2  
  with(m, pchisq(null.deviance - deviance, df.null - df.residual, lower.tail = FALSE))  
  ## [1] 0.2706949  
  BIC(m)  
  ## [1] 672.5928  
  # Hosmer-Lemeshow Goodness-of-Fit Test
glmtoolbox::hltest(m)  
  ## 
##    The Hosmer-Lemeshow goodness-of-fit test
## 
##  Group Size Observed   Expected
##      1   15        1  0.3586504
##      2  192        6  6.6413496
##      3 1290       63 63.6413496
##      4  161       12 11.3586504
## 
##          Statistic =  1.28489 
## degrees of freedom =  2 
##            p-value =  0.52601  
  ## CIs using profiled log-likelihood
confint(m, level=0.99)  
  ## Waiting for profiling to be done...  
  ##                      0.5 %     99.5 %
## (Intercept)     -3.3062053 -2.6437266
## C_Diet_VeganYes -0.5387699  1.1680253
## D_Diet_VeganYes -2.0987250  0.4384643  
  ## CIs using standard errors
confint.default(m, level=0.99)  
  ##                      0.5 %     99.5 %
## (Intercept)     -3.2888206 -2.6282618
## C_Diet_VeganYes -0.4634283  1.2239870
## D_Diet_VeganYes -1.9875685  0.4861373  
  # Wald test
wald.test(b = coef(m), Sigma = vcov(m), Terms = 2)  
  ## Wald test:
## ----------
## 
## Chi-squared test:
## X2 = 1.3, df = 1, P(> X2) = 0.25  
  ## odds ratios and 95% CI
exp(cbind(OR = coef(m), confint(m, level=0.99)))  
  ## Waiting for profiling to be done...  
  ##                         OR      0.5 %     99.5 %
## (Intercept)     0.05189457 0.03665501 0.07109583
## C_Diet_VeganYes 1.46269318 0.58346556 3.21563648
## D_Diet_VeganYes 0.47202865 0.12261266 1.55032449  
 
 Calculate Nagelkerke R^2 
  NagelkerkeR2(m)  
  ## $N
## [1] 1658
## 
## $R2
## [1] 0.004838484  
 
 
 check assumptions of model 
 
 Cook’s distance 
  plot(m, which = 4, id.n = 3)  
   
 
 
 Extract model results and display data for top 3 values using Cook’s
distance 
  model.data <- augment(m) %>% 
  mutate(index = 1:n()) 
model.data %>% top_n(3, .cooksd)  
 
 
 
 
 
 plot standardised residuals 
  ggplot(model.data, aes(index, .std.resid)) + 
  geom_point(aes(color = Health_Binary), alpha = .5) +
  theme_bw()  
   
 
 
 Filter potential influential data points with abs(.std.res) >
3: 
  model.data %>% 
  filter(abs(.std.resid) > 3)  
 
 
 
 
 
 check for multicollinearity 
  car::vif(m)  
  ## C_Diet_Vegan D_Diet_Vegan 
##     1.423581     1.423581  
 
 
 
 Create ROCR for training and test data 
  ## training data
pred.mtt = predict(m, type = "response") #repeat risk predictions from model m
rocr.pred.mtt = ROCR::prediction(pred.mtt, labels = ml_train$Health_Binary) #ROCR prediction object
roc.perf.mtt = ROCR::performance(rocr.pred.mtt, measure = "tpr", x.measure = "fpr") # #ROCR performance object
plot(roc.perf.mtt, col = "blue")


pred.te.1 = predict(m, newdata = ml_test, type = "response") #.te = "test"
rocr.pred.te.1 = ROCR::prediction(pred.te.1, labels = ml_test$Health_Binary)
roc.perf.te.1 = ROCR::performance(rocr.pred.te.1, measure = "tpr", x.measure = "fpr")
plot(roc.perf.te.1, col = "red", add = T)

abline(a = 0, b = 1, lty = 2) #diagonal for random assignment
legend("bottomright", legend = c("train","test"),
col = c("blue","red"), lty = c(2,1), lwd =1.5)  
   
 
 
 Report AUC from ROC for training and test data 
    # Train AUC
aucTr <- ROCR::performance(rocr.pred.mtt, measure = "auc")
  aucTr <- aucTr@y.values[[1]]
  print(aucTr)  
  ## [1] 0.5410231  
     # Test AUC
  aucTe <- ROCR::performance(rocr.pred.te.1, measure = "auc")
  aucTe <- aucTe@y.values[[1]]
  print(aucTe)  
  ## [1] 0.5142544  
 
 
 
 CLIENT DIET VEGAN * DOG DIET VEGAN binaryl regression ON SIGNIFICANT
OR SERIOUS ILLNESS 
  # fit binary logit model and store results 'm2'
m <- glm(Health_Binary ~ C_Diet_Vegan*D_Diet_Vegan, data = ml_train,family = binomial)
# view a summary of the model
summary(m)  
  ## 
## Call:
## glm(formula = Health_Binary ~ C_Diet_Vegan * D_Diet_Vegan, family = binomial, 
##     data = ml_train)
## 
## Coefficients:
##                                 Estimate Std. Error z value Pr(>|z|)    
## (Intercept)                      -2.9692     0.1292 -22.985   <2e-16 ***
## C_Diet_VeganYes                   0.4502     0.3267   1.378    0.168    
## D_Diet_VeganYes                   0.3301     1.0431   0.316    0.752    
## C_Diet_VeganYes:D_Diet_VeganYes  -1.2451     1.1620  -1.072    0.284    
## ---
## Signif. codes:  0 '***' 0.001 '**' 0.01 '*' 0.05 '.' 0.1 ' ' 1
## 
## (Dispersion parameter for binomial family taken to be 1)
## 
##     Null deviance: 652.97  on 1657  degrees of freedom
## Residual deviance: 649.44  on 1654  degrees of freedom
## AIC: 657.44
## 
## Number of Fisher Scoring iterations: 6  
  # test model fit
with(m, null.deviance - deviance)  
  ## [1] 3.521524  
  with(m, df.null - df.residual)  
  ## [1] 3  
  with(m, pchisq(null.deviance - deviance, df.null - df.residual, lower.tail = FALSE))  
  ## [1] 0.3179813  
  BIC(m)  
  ## [1] 679.0982  
  # Hosmer-Lemeshow Goodness-of-Fit Test
glmtoolbox::hltest(m)  
  ## 
##    The Hosmer-Lemeshow goodness-of-fit test
## 
##  Group Size Observed Expected
##      1  192        6        6
##      2 1290       63       63
##      3   15        1        1
##      4  161       12       12
## 
##          Statistic =  0 
## degrees of freedom =  2 
##            p-value =  1  
  ## CIs using profiled log-likelihood
confint(m, level=0.99)  
  ## Waiting for profiling to be done...  
  ##                                      0.5 %    99.5 %
## (Intercept)                     -3.3196268 -2.652159
## C_Diet_VeganYes                 -0.4742113  1.234056
## D_Diet_VeganYes                 -4.0170156  2.363436
## C_Diet_VeganYes:D_Diet_VeganYes -3.7790046  3.228218  
  ## CIs using standard errors
confint.default(m, level=0.99)  
  ##                                      0.5 %    99.5 %
## (Intercept)                     -3.3019437 -2.636442
## C_Diet_VeganYes                 -0.3913698  1.291676
## D_Diet_VeganYes                 -2.3567850  3.017056
## C_Diet_VeganYes:D_Diet_VeganYes -4.2381521  1.747986  
  # Wald test
wald.test(b = coef(m), Sigma = vcov(m), Terms = 2)  
  ## Wald test:
## ----------
## 
## Chi-squared test:
## X2 = 1.9, df = 1, P(> X2) = 0.17  
  ## odds ratios and 95% CI
exp(cbind(OR = coef(m), confint(m, level=0.99)))  
  ## Waiting for profiling to be done...  
  ##                                         OR      0.5 %      99.5 %
## (Intercept)                     0.05134474 0.03616633  0.07049886
## C_Diet_VeganYes                 1.56855225 0.62237574  3.43513384
## D_Diet_VeganYes                 1.39115646 0.01800662 10.62740983
## C_Diet_VeganYes:D_Diet_VeganYes 0.28791703 0.02284542 25.23463728  
 
 Calculate Nagelkerke R^2 
  NagelkerkeR2(m)  
  ## $N
## [1] 1658
## 
## $R2
## [1] 0.0065177  
 
 
 check assumptions of model 
 
 Cook’s distance 
  plot(m, which = 4, id.n = 3)  
   
 
 
 Extract model results and display data for top 3 values using Cook’s
distance 
  model.data <- augment(m) %>% 
  mutate(index = 1:n()) 
model.data %>% top_n(3, .cooksd)  
 
 
 
 
 
 plot standardised residuals 
  ggplot(model.data, aes(index, .std.resid)) + 
  geom_point(aes(color = Health_Binary), alpha = .5) +
  theme_bw()  
   
 
 
 Filter potential influential data points with abs(.std.res) >
3: 
  model.data %>% 
  filter(abs(.std.resid) > 3)  
 
 
 
 
 
 check for multicollinearity 
  car::vif(m)  
  ## there are higher-order terms (interactions) in this model
## consider setting type = 'predictor'; see ?vif  
  ##              C_Diet_Vegan              D_Diet_Vegan C_Diet_Vegan:D_Diet_Vegan 
##                  1.412924                  6.703605                  7.261551  
 
 
 
 Create ROCR for training and test data 
  ## training data
pred.mtt = predict(m, type = "response") #repeat risk predictions from model m
rocr.pred.mtt = ROCR::prediction(pred.mtt, labels = ml_train$Health_Binary) #ROCR prediction object
roc.perf.mtt = ROCR::performance(rocr.pred.mtt, measure = "tpr", x.measure = "fpr") # #ROCR performance object
plot(roc.perf.mtt, col = "blue")


pred.te.1 = predict(m, newdata = ml_test, type = "response") #.te = "test"
rocr.pred.te.1 = ROCR::prediction(pred.te.1, labels = ml_test$Health_Binary)
roc.perf.te.1 = ROCR::performance(rocr.pred.te.1, measure = "tpr", x.measure = "fpr")
plot(roc.perf.te.1, col = "red", add = T)

abline(a = 0, b = 1, lty = 2) #diagonal for random assignment
legend("bottomright", legend = c("train","test"),
col = c("blue","red"), lty = c(2,1), lwd =1.5)  
   
 
 
 Report AUC from ROC for training and test data 
    # Train AUC
aucTr <- ROCR::performance(rocr.pred.mtt, measure = "auc")
  aucTr <- aucTr@y.values[[1]]
  print(aucTr)  
  ## [1] 0.544482  
     # Test AUC
  aucTe <- ROCR::performance(rocr.pred.te.1, measure = "auc")
  aucTe <- aucTe@y.values[[1]]
  print(aucTe)  
  ## [1] 0.5019737  
 
 
 
 DOG DIET RAW binary regression ON SIGNIFICANT OR SERIOUS
ILLNESS 
  # fit binary logit model and store results 'm'
m <- glm(Health_Binary ~ D_Diet_Raw, data = ml_train,family = binomial)
# view a summary of the model
summary(m)  
  ## 
## Call:
## glm(formula = Health_Binary ~ D_Diet_Raw, family = binomial, 
##     data = ml_train)
## 
## Coefficients:
##               Estimate Std. Error z value Pr(>|z|)    
## (Intercept)    -2.8112     0.1308 -21.498   <2e-16 ***
## D_Diet_RawYes  -0.4939     0.2626  -1.881     0.06 .  
## ---
## Signif. codes:  0 '***' 0.001 '**' 0.01 '*' 0.05 '.' 0.1 ' ' 1
## 
## (Dispersion parameter for binomial family taken to be 1)
## 
##     Null deviance: 652.97  on 1657  degrees of freedom
## Residual deviance: 649.17  on 1656  degrees of freedom
## AIC: 653.17
## 
## Number of Fisher Scoring iterations: 6  
  # test model fit
with(m, null.deviance - deviance)  
  ## [1] 3.800504  
  with(m, df.null - df.residual)  
  ## [1] 1  
  with(m, pchisq(null.deviance - deviance, df.null - df.residual, lower.tail = FALSE))  
  ## [1] 0.05123716  
  BIC(m)  
  ## [1] 663.9925  
  ## CIs using profiled log-likelihood
confint(m, level=0.99)  
  ## Waiting for profiling to be done...  
  ##                   0.5 %    99.5 %
## (Intercept)   -3.165798 -2.490129
## D_Diet_RawYes -1.212952  0.152438  
  ## CIs using standard errors
confint.default(m, level=0.99)  
  ##                   0.5 %     99.5 %
## (Intercept)   -3.147973 -2.4743269
## D_Diet_RawYes -1.170194  0.1823869  
  # Wald test
wald.test(b = coef(m), Sigma = vcov(m), Terms = 2)  
  ## Wald test:
## ----------
## 
## Chi-squared test:
## X2 = 3.5, df = 1, P(> X2) = 0.06  
  ## odds ratios and 95% CI
exp(cbind(OR = coef(m), confint(m, level=0.99)))  
  ## Waiting for profiling to be done...  
  ##                       OR      0.5 %    99.5 %
## (Intercept)   0.06013579 0.04218045 0.0828993
## D_Diet_RawYes 0.61023972 0.29731828 1.1646703  
 
 Calculate Nagelkerke R^2 
  NagelkerkeR2(m)  
  ## $N
## [1] 1658
## 
## $R2
## [1] 0.007033449  
 
 
 check assumptions of model 
 
 Cook’s distance 
  plot(m, which = 4, id.n = 3)  
   
 
 
 Extract model results and display data for top 3 values using Cook’s
distance 
  model.data <- augment(m) %>% 
  mutate(index = 1:n()) 
model.data %>% top_n(3, .cooksd)  
 
 
 
 
 
 
 
 
 plot standardised residuals 
  ggplot(model.data, aes(index, .std.resid)) + 
  geom_point(aes(color = Health_Binary), alpha = .5) +
  theme_bw()  
   
 
 Filter potential influential data points with abs(.std.res) >
3: 
  model.data %>% 
  filter(abs(.std.resid) > 3)  
 
 
 
 
 
 Create ROCR for training and test data 
  ## training data
pred.mtt = predict(m, type = "response") #repeat risk predictions from model m
rocr.pred.mtt = ROCR::prediction(pred.mtt, labels = ml_train$Health_Binary) #ROCR prediction object
roc.perf.mtt = ROCR::performance(rocr.pred.mtt, measure = "tpr", x.measure = "fpr") # #ROCR performance object
plot(roc.perf.mtt, col = "blue")


pred.te.1 = predict(m, newdata = ml_test, type = "response") #.te = "test"
rocr.pred.te.1 = ROCR::prediction(pred.te.1, labels = ml_test$Health_Binary)
roc.perf.te.1 = ROCR::performance(rocr.pred.te.1, measure = "tpr", x.measure = "fpr")
plot(roc.perf.te.1, col = "red", add = T)

abline(a = 0, b = 1, lty = 2) #diagonal for random assignment
legend("bottomright", legend = c("train","test"),
col = c("blue","red"), lty = c(2,1), lwd =1.5)  
   
 
 
 Report AUC from ROC for training and test data 
    # Train AUC
aucTr <- ROCR::performance(rocr.pred.mtt, measure = "auc")
  aucTr <- aucTr@y.values[[1]]
  print(aucTr)  
  ## [1] 0.5509549  
     # Test AUC
  aucTe <- ROCR::performance(rocr.pred.te.1, measure = "auc")
  aucTe <- aucTe@y.values[[1]]
  print(aucTe)  
  ## [1] 0.5819201  
 
 
 DOG DIET + CLIENT DIET binary logistic regression ON SIGNIFICANT OR
SERIOUS ILLNESS 
  # fit binary logit model and store results 'm'
m <- glm(Health_Binary ~ D_Diet + C_Diet , data = ml_train,family = binomial)
# view a summary of the model
summary(m)  
  ## 
## Call:
## glm(formula = Health_Binary ~ D_Diet + C_Diet, family = binomial, 
##     data = ml_train)
## 
## Coefficients:
##                                                         Estimate Std. Error
## (Intercept)                                             -2.94019    0.21092
## D_DietMeat-based – raw                                  -0.55042    0.26995
## D_DietVegan (consuming no animal products)              -0.89615    0.48630
## D_DietVegetarian (including eggs or milk, but not meat)  0.05005    0.76250
## C_DietOmnivore reducing animal product consumption       0.35432    0.29681
## C_DietPescatarian (including fish but no other meats)   -0.27951    0.61876
## C_DietVegan (consuming no animal products)               0.50269    0.36265
## C_DietVegetarian (consuming plants, eggs and milk)       0.49359    0.36128
##                                                         z value Pr(>|z|)    
## (Intercept)                                             -13.940   <2e-16 ***
## D_DietMeat-based – raw                                   -2.039   0.0414 *  
## D_DietVegan (consuming no animal products)               -1.843   0.0654 .  
## D_DietVegetarian (including eggs or milk, but not meat)   0.066   0.9477    
## C_DietOmnivore reducing animal product consumption        1.194   0.2326    
## C_DietPescatarian (including fish but no other meats)    -0.452   0.6515    
## C_DietVegan (consuming no animal products)                1.386   0.1657    
## C_DietVegetarian (consuming plants, eggs and milk)        1.366   0.1719    
## ---
## Signif. codes:  0 '***' 0.001 '**' 0.01 '*' 0.05 '.' 0.1 ' ' 1
## 
## (Dispersion parameter for binomial family taken to be 1)
## 
##     Null deviance: 652.97  on 1657  degrees of freedom
## Residual deviance: 642.18  on 1650  degrees of freedom
## AIC: 658.18
## 
## Number of Fisher Scoring iterations: 6  
  # test model fit
with(m, null.deviance - deviance)  
  ## [1] 10.78744  
  with(m, df.null - df.residual)  
  ## [1] 7  
  with(m, pchisq(null.deviance - deviance, df.null - df.residual, lower.tail = FALSE))  
  ## [1] 0.1481634  
  BIC(m)  
  ## [1] 701.4857  
  # Hosmer-Lemeshow Goodness-of-Fit Test
hltest(m, G=7)  
  ## 
##    The Hosmer-Lemeshow goodness-of-fit test
## 
##  Group Size Observed   Expected
##      1   38        1  0.8324579
##      2  289        7  8.5487846
##      3  201        7  6.8884989
##      4  209        7  8.5177362
##      5   93        7  4.4434149
##      6  382       21 19.1772909
##      7  210       15 14.6415490
##      8  216       15 17.2745507
##      9   20        2  1.6757168
## 
##          Statistic =  2.73804 
## degrees of freedom =  7 
##            p-value =  0.90813  
  ## CIs using profiled log-likelihood
confint(m, level=0.99)  
  ## Waiting for profiling to be done...  
  ##                                                              0.5 %     99.5 %
## (Intercept)                                             -3.5236252 -2.4314377
## D_DietMeat-based – raw                                  -1.2851360  0.1187179
## D_DietVegan (consuming no animal products)              -2.2570963  0.3096985
## D_DietVegetarian (including eggs or milk, but not meat) -2.6071224  1.6789055
## C_DietOmnivore reducing animal product consumption      -0.4308421  1.1142849
## C_DietPescatarian (including fish but no other meats)   -2.3074154  1.0765831
## C_DietVegan (consuming no animal products)              -0.4893397  1.4021139
## C_DietVegetarian (consuming plants, eggs and milk)      -0.5012643  1.3881647  
  ## CIs using standard errors
confint.default(m, level=0.99)  
  ##                                                              0.5 %     99.5 %
## (Intercept)                                             -3.4834741 -2.3969009
## D_DietMeat-based – raw                                  -1.2457570  0.1449106
## D_DietVegan (consuming no animal products)              -2.1487760  0.3564692
## D_DietVegetarian (including eggs or milk, but not meat) -1.9140208  2.0141299
## C_DietOmnivore reducing animal product consumption      -0.4102139  1.1188464
## C_DietPescatarian (including fish but no other meats)   -1.8733427  1.3143154
## C_DietVegan (consuming no animal products)              -0.4314431  1.4368187
## C_DietVegetarian (consuming plants, eggs and milk)      -0.4370103  1.4241876  
  # Wald test
wald.test(b = coef(m), Sigma = vcov(m), Terms = 2)  
  ## Wald test:
## ----------
## 
## Chi-squared test:
## X2 = 4.2, df = 1, P(> X2) = 0.041  
  ## odds ratios and 95% CI
exp(cbind(OR = coef(m), confint(m, level=0.99)))  
  ## Waiting for profiling to be done...  
  ##                                                                 OR      0.5 %
## (Intercept)                                             0.05285582 0.02949233
## D_DietMeat-based – raw                                  0.57670572 0.27661297
## D_DietVegan (consuming no animal products)              0.40813659 0.10465393
## D_DietVegetarian (including eggs or milk, but not meat) 1.05132846 0.07374645
## C_DietOmnivore reducing animal product consumption      1.42520584 0.64996155
## C_DietPescatarian (including fish but no other meats)   0.75615139 0.09951813
## C_DietVegan (consuming no animal products)              1.65315866 0.61303106
## C_DietVegetarian (consuming plants, eggs and milk)      1.63818454 0.60576428
##                                                             99.5 %
## (Intercept)                                             0.08791035
## D_DietMeat-based – raw                                  1.12605221
## D_DietVegan (consuming no animal products)              1.36301407
## D_DietVegetarian (including eggs or milk, but not meat) 5.35968659
## C_DietOmnivore reducing animal product consumption      3.04738817
## C_DietPescatarian (including fish but no other meats)   2.93463503
## C_DietVegan (consuming no animal products)              4.06378124
## C_DietVegetarian (consuming plants, eggs and milk)      4.00748824  
 
 Calculate Nagelkerke R^2 
  NagelkerkeR2(m)  
  ## $N
## [1] 1658
## 
## $R2
## [1] 0.01992191  
 
 
 check assumptions of model 
 
 Cook’s distance 
  plot(m, which = 4, id.n = 3)  
   
 
 
 Extract model results and display data for top 3 values using Cook’s
distance 
  model.data <- augment(m) %>% 
  mutate(index = 1:n()) 
model.data %>% top_n(3, .cooksd)  
 
 
 
 
 
 plot standardised residuals 
  ggplot(model.data, aes(index, .std.resid)) + 
  geom_point(aes(color = Health_Binary), alpha = .5) +
  theme_bw()  
   
 
 
 Filter potential influential data points with abs(.std.res) >
3: 
  model.data %>% 
  filter(abs(.std.resid) > 3)  
 
 
 
 
 
 check for multicollinearity 
  car::vif(m)  
  ##            GVIF Df GVIF^(1/(2*Df))
## D_Diet 1.484848  3        1.068104
## C_Diet 1.484848  4        1.050655  
 
 
 
 Create ROCR for training and test data 
  ## training data
pred.mtt = predict(m, type = "response") #repeat risk predictions from model m
rocr.pred.mtt = ROCR::prediction(pred.mtt, labels = ml_train$Health_Binary) #ROCR prediction object
roc.perf.mtt = ROCR::performance(rocr.pred.mtt, measure = "tpr", x.measure = "fpr") # #ROCR performance object
plot(roc.perf.mtt, col = "blue")


pred.te.1 = predict(m, newdata = ml_test, type = "response") #.te = "test"
rocr.pred.te.1 = ROCR::prediction(pred.te.1, labels = ml_test$Health_Binary)
roc.perf.te.1 = ROCR::performance(rocr.pred.te.1, measure = "tpr", x.measure = "fpr")
plot(roc.perf.te.1, col = "red", add = T)

abline(a = 0, b = 1, lty = 2) #diagonal for random assignment
legend("bottomright", legend = c("train","test"),
col = c("blue","red"), lty = c(2,1), lwd =1.5)  
   
 
 
 Report AUC from ROC for training and test data 
    # Train AUC
aucTr <- ROCR::performance(rocr.pred.mtt, measure = "auc")
  aucTr <- aucTr@y.values[[1]]
  print(aucTr)  
  ## [1] 0.6062276  
     # Test AUC
  aucTe <- ROCR::performance(rocr.pred.te.1, measure = "auc")
  aucTe <- aucTe@y.values[[1]]
  print(aucTe)  
  ## [1] 0.620346  
 
 
 
 THERAPEUTIC DIET binary logistic regression ON SIGNIFICANT OR
SERIOUS ILLNESS 
  # fit binary logit model and store results 'm'
m <- glm(Health_Binary ~ Therapeutic_Food, data = ml_train,family = binomial)
# view a summary of the model
summary(m)  
  ## 
## Call:
## glm(formula = Health_Binary ~ Therapeutic_Food, family = binomial, 
##     data = ml_train)
## 
## Coefficients:
##                     Estimate Std. Error z value Pr(>|z|)    
## (Intercept)          -3.1362     0.1257 -24.942  < 2e-16 ***
## Therapeutic_FoodYes   1.8657     0.3096   6.026 1.68e-09 ***
## ---
## Signif. codes:  0 '***' 0.001 '**' 0.01 '*' 0.05 '.' 0.1 ' ' 1
## 
## (Dispersion parameter for binomial family taken to be 1)
## 
##     Null deviance: 652.97  on 1657  degrees of freedom
## Residual deviance: 625.58  on 1656  degrees of freedom
## AIC: 629.58
## 
## Number of Fisher Scoring iterations: 6  
  # test model fit
with(m, null.deviance - deviance)  
  ## [1] 27.39065  
  with(m, df.null - df.residual)  
  ## [1] 1  
  with(m, pchisq(null.deviance - deviance, df.null - df.residual, lower.tail = FALSE))  
  ## [1] 1.662326e-07  
  BIC(m)  
  ## [1] 640.4023  
  ## CIs using profiled log-likelihood
confint(m, level=0.99)  
  ## Waiting for profiling to be done...  
  ##                         0.5 %    99.5 %
## (Intercept)         -3.477014 -2.827434
## Therapeutic_FoodYes  1.020073  2.631890  
  ## CIs using standard errors
confint.default(m, level=0.99)  
  ##                         0.5 %    99.5 %
## (Intercept)         -3.460030 -2.812275
## Therapeutic_FoodYes  1.068207  2.663173  
  # Wald test
wald.test(b = coef(m), Sigma = vcov(m), Terms = 2)  
  ## Wald test:
## ----------
## 
## Chi-squared test:
## X2 = 36.3, df = 1, P(> X2) = 1.7e-09  
  ## odds ratios and 95% CI
exp(cbind(OR = coef(m), confint(m, level=0.99)))  
  ## Waiting for profiling to be done...  
  ##                             OR      0.5 %      99.5 %
## (Intercept)         0.04344964 0.03089954  0.05916446
## Therapeutic_FoodYes 6.46039341 2.77339713 13.90001911  
 
 Calculate Nagelkerke R^2 
  NagelkerkeR2(m)  
  ## $N
## [1] 1658
## 
## $R2
## [1] 0.05033208  
 
 
 check assumptions of model 
 
 Cook’s distance 
  plot(m, which = 4, id.n = 3)  
   
 
 
 Extract model results and display data for top 3 values using Cook’s
distance 
  model.data <- augment(m) %>% 
  mutate(index = 1:n()) 
model.data %>% top_n(3, .cooksd)  
 
 
 
 
 
 plot standardised residuals 
  ggplot(model.data, aes(index, .std.resid)) + 
  geom_point(aes(color = Health_Binary), alpha = .5) +
  theme_bw()  
   
 
 
 Filter potential influential data points with abs(.std.res) >
3: 
  model.data %>% 
  filter(abs(.std.resid) > 3)  
 
 
 
 
 
 
 Create ROCR for training and test data 
  ## training data
pred.mtt = predict(m, type = "response") #repeat risk predictions from model m
rocr.pred.mtt = ROCR::prediction(pred.mtt, labels = ml_train$Health_Binary) #ROCR prediction object
roc.perf.mtt = ROCR::performance(rocr.pred.mtt, measure = "tpr", x.measure = "fpr") # #ROCR performance object
plot(roc.perf.mtt, col = "blue")


pred.te.1 = predict(m, newdata = ml_test, type = "response") #.te = "test"
rocr.pred.te.1 = ROCR::prediction(pred.te.1, labels = ml_test$Health_Binary)
roc.perf.te.1 = ROCR::performance(rocr.pred.te.1, measure = "tpr", x.measure = "fpr")
plot(roc.perf.te.1, col = "red", add = T)

abline(a = 0, b = 1, lty = 2) #diagonal for random assignment
legend("bottomright", legend = c("train","test"),
col = c("blue","red"), lty = c(2,1), lwd =1.5)  
   
 
 
 Report AUC from ROC for training and test data 
    # Train AUC
aucTr <- ROCR::performance(rocr.pred.mtt, measure = "auc")
  aucTr <- aucTr@y.values[[1]]
  print(aucTr)  
  ## [1] 0.5794772  
     # Test AUC
  aucTe <- ROCR::performance(rocr.pred.te.1, measure = "auc")
  aucTe <- aucTe@y.values[[1]]
  print(aucTe)  
  ## [1] 0.5545322  
 
 
 
 DOG DIET + THERAPEUTIC DIET binary logistic regression ON
SIGNIFICANT OR SERIOUS ILLNESS 
  # fit binary logit model and store results 'm'
m <- glm(Health_Binary ~ D_Diet + Therapeutic_Food, data = ml_train,family = binomial)
# view a summary of the model
summary(m)  
  ## 
## Call:
## glm(formula = Health_Binary ~ D_Diet + Therapeutic_Food, family = binomial, 
##     data = ml_train)
## 
## Coefficients:
##                                                         Estimate Std. Error
## (Intercept)                                             -2.93813    0.15693
## D_DietMeat-based – raw                                  -0.41872    0.27398
## D_DietVegan (consuming no animal products)              -0.64324    0.41495
## D_DietVegetarian (including eggs or milk, but not meat)  0.07037    0.77301
## Therapeutic_FoodYes                                      1.79261    0.31628
##                                                         z value Pr(>|z|)    
## (Intercept)                                             -18.722  < 2e-16 ***
## D_DietMeat-based – raw                                   -1.528    0.126    
## D_DietVegan (consuming no animal products)               -1.550    0.121    
## D_DietVegetarian (including eggs or milk, but not meat)   0.091    0.927    
## Therapeutic_FoodYes                                       5.668 1.45e-08 ***
## ---
## Signif. codes:  0 '***' 0.001 '**' 0.01 '*' 0.05 '.' 0.1 ' ' 1
## 
## (Dispersion parameter for binomial family taken to be 1)
## 
##     Null deviance: 652.97  on 1657  degrees of freedom
## Residual deviance: 621.24  on 1653  degrees of freedom
## AIC: 631.24
## 
## Number of Fisher Scoring iterations: 6  
  # test model fit
with(m, null.deviance - deviance)  
  ## [1] 31.72411  
  with(m, df.null - df.residual)  
  ## [1] 4  
  with(m, pchisq(null.deviance - deviance, df.null - df.residual, lower.tail = FALSE))  
  ## [1] 2.178249e-06  
  BIC(m)  
  ## [1] 658.309  
  # Hosmer-Lemeshow Goodness-of-Fit Test
hltest(m, G=3)  
  ## 
##    The Hosmer-Lemeshow goodness-of-fit test
## 
##  Group Size Observed   Expected
##      1  195        5  5.2813019
##      2  558       19 18.7887269
##      3  809       41 40.6932471
##      4   23        1  1.2367241
##      5   12        2  1.7186981
##      6    7        1  1.2112731
##      7   51       12 12.3067529
##      8    3        1  0.7632759
## 
##          Statistic =  0.27503 
## degrees of freedom =  6 
##            p-value =  0.99961  
  ## CIs using profiled log-likelihood
confint(m, level=0.99)  
  ## Waiting for profiling to be done...  
  ##                                                              0.5 %     99.5 %
## (Intercept)                                             -3.3668499 -2.5555257
## D_DietMeat-based – raw                                  -1.1616497  0.2634369
## D_DietVegan (consuming no animal products)              -1.8726945  0.3197434
## D_DietVegetarian (including eggs or milk, but not meat) -2.6051813  1.7211018
## Therapeutic_FoodYes                                      0.9321526  2.5781662  
  ## CIs using standard errors
confint.default(m, level=0.99)  
  ##                                                              0.5 %     99.5 %
## (Intercept)                                             -3.3423616 -2.5338923
## D_DietMeat-based – raw                                  -1.1244459  0.2869989
## D_DietVegan (consuming no animal products)              -1.7120940  0.4256083
## D_DietVegetarian (including eggs or milk, but not meat) -1.9207687  2.0615067
## Therapeutic_FoodYes                                      0.9779336  2.6072863  
  # Wald test
wald.test(b = coef(m), Sigma = vcov(m), Terms = 2)  
  ## Wald test:
## ----------
## 
## Chi-squared test:
## X2 = 2.3, df = 1, P(> X2) = 0.13  
  ## odds ratios and 95% CI
exp(cbind(OR = coef(m), confint(m, level=0.99)))  
  ## Waiting for profiling to be done...  
  ##                                                                 OR      0.5 %
## (Intercept)                                             0.05296484 0.03449814
## D_DietMeat-based – raw                                  0.65788608 0.31296946
## D_DietVegan (consuming no animal products)              0.52558525 0.15370894
## D_DietVegetarian (including eggs or milk, but not meat) 1.07290396 0.07388974
## Therapeutic_FoodYes                                     6.00510507 2.53997071
##                                                             99.5 %
## (Intercept)                                              0.0776514
## D_DietMeat-based – raw                                   1.3013952
## D_DietVegan (consuming no animal products)               1.3767745
## D_DietVegetarian (including eggs or milk, but not meat)  5.5906851
## Therapeutic_FoodYes                                     13.1729589  
 
 Calculate Nagelkerke R^2 
  NagelkerkeR2(m)  
  ## $N
## [1] 1658
## 
## $R2
## [1] 0.05821917  
 
 
 check assumptions of model 
 
 Cook’s distance 
  plot(m, which = 4, id.n = 3)  
   
 
 
 Extract model results and display data for top 3 values using Cook’s
distance 
  model.data <- augment(m) %>% 
  mutate(index = 1:n()) 
model.data %>% top_n(3, .cooksd)  
 
 
 
 
 
 plot standardised residuals 
  ggplot(model.data, aes(index, .std.resid)) + 
  geom_point(aes(color = Health_Binary), alpha = .5) +
  theme_bw()  
   
 
 
 Filter potential influential data points with abs(.std.res) >
3: 
  model.data %>% 
  filter(abs(.std.resid) > 3)  
 
 
 
 
 
 check for multicollinearity 
  car::vif(m)  
  ##                      GVIF Df GVIF^(1/(2*Df))
## D_Diet           1.035323  3        1.005802
## Therapeutic_Food 1.035323  1        1.017508  
 
 
 
 Create ROCR for training and test data 
  ## training data
pred.mtt = predict(m, type = "response") #repeat risk predictions from model m
rocr.pred.mtt = ROCR::prediction(pred.mtt, labels = ml_train$Health_Binary) #ROCR prediction object
roc.perf.mtt = ROCR::performance(rocr.pred.mtt, measure = "tpr", x.measure = "fpr") # #ROCR performance object
plot(roc.perf.mtt, col = "blue")


pred.te.1 = predict(m, newdata = ml_test, type = "response") #.te = "test"
rocr.pred.te.1 = ROCR::prediction(pred.te.1, labels = ml_test$Health_Binary)
roc.perf.te.1 = ROCR::performance(rocr.pred.te.1, measure = "tpr", x.measure = "fpr")
plot(roc.perf.te.1, col = "red", add = T)

abline(a = 0, b = 1, lty = 2) #diagonal for random assignment
legend("bottomright", legend = c("train","test"),
col = c("blue","red"), lty = c(2,1), lwd =1.5)  
   
 
 
 Report AUC from ROC for training and test data 
    # Train AUC
aucTr <- ROCR::performance(rocr.pred.mtt, measure = "auc")
  aucTr <- aucTr@y.values[[1]]
  print(aucTr)  
  ## [1] 0.627983  
     # Test AUC
  aucTe <- ROCR::performance(rocr.pred.te.1, measure = "auc")
  aucTe <- aucTe@y.values[[1]]
  print(aucTe)  
  ## [1] 0.6221735  
 
 
 
 DOG DIET * THERAPEUTIC DIET binary logistic regression ON
SIGNIFICANT OR SERIOUS ILLNESS 
  # fit binary logit model and store results 'm'
m <- glm(Health_Binary ~ D_Diet*Therapeutic_Food, data = ml_train,family = binomial)
# view a summary of the model
summary(m)  
  ## 
## Call:
## glm(formula = Health_Binary ~ D_Diet * Therapeutic_Food, family = binomial, 
##     data = ml_train)
## 
## Coefficients:
##                                                                             Estimate
## (Intercept)                                                                  -2.9302
## D_DietMeat-based – raw                                                       -0.4151
## D_DietVegan (consuming no animal products)                                   -0.7074
## D_DietVegetarian (including eggs or milk, but not meat)                      -0.1608
## Therapeutic_FoodYes                                                           1.7516
## D_DietMeat-based – raw:Therapeutic_FoodYes                                   -0.1980
## D_DietVegan (consuming no animal products):Therapeutic_FoodYes                0.2766
## D_DietVegetarian (including eggs or milk, but not meat):Therapeutic_FoodYes   0.6463
##                                                                             Std. Error
## (Intercept)                                                                     0.1603
## D_DietMeat-based – raw                                                          0.2832
## D_DietVegan (consuming no animal products)                                      0.4806
## D_DietVegetarian (including eggs or milk, but not meat)                         1.0350
## Therapeutic_FoodYes                                                             0.3670
## D_DietMeat-based – raw:Therapeutic_FoodYes                                      1.1644
## D_DietVegan (consuming no animal products):Therapeutic_FoodYes                  0.9695
## D_DietVegetarian (including eggs or milk, but not meat):Therapeutic_FoodYes     1.6371
##                                                                             z value
## (Intercept)                                                                 -18.281
## D_DietMeat-based – raw                                                       -1.466
## D_DietVegan (consuming no animal products)                                   -1.472
## D_DietVegetarian (including eggs or milk, but not meat)                      -0.155
## Therapeutic_FoodYes                                                           4.773
## D_DietMeat-based – raw:Therapeutic_FoodYes                                   -0.170
## D_DietVegan (consuming no animal products):Therapeutic_FoodYes                0.285
## D_DietVegetarian (including eggs or milk, but not meat):Therapeutic_FoodYes   0.395
##                                                                             Pr(>|z|)
## (Intercept)                                                                  < 2e-16
## D_DietMeat-based – raw                                                         0.143
## D_DietVegan (consuming no animal products)                                     0.141
## D_DietVegetarian (including eggs or milk, but not meat)                        0.877
## Therapeutic_FoodYes                                                         1.81e-06
## D_DietMeat-based – raw:Therapeutic_FoodYes                                     0.865
## D_DietVegan (consuming no animal products):Therapeutic_FoodYes                 0.775
## D_DietVegetarian (including eggs or milk, but not meat):Therapeutic_FoodYes    0.693
##                                                                                
## (Intercept)                                                                 ***
## D_DietMeat-based – raw                                                         
## D_DietVegan (consuming no animal products)                                     
## D_DietVegetarian (including eggs or milk, but not meat)                        
## Therapeutic_FoodYes                                                         ***
## D_DietMeat-based – raw:Therapeutic_FoodYes                                     
## D_DietVegan (consuming no animal products):Therapeutic_FoodYes                 
## D_DietVegetarian (including eggs or milk, but not meat):Therapeutic_FoodYes    
## ---
## Signif. codes:  0 '***' 0.001 '**' 0.01 '*' 0.05 '.' 0.1 ' ' 1
## 
## (Dispersion parameter for binomial family taken to be 1)
## 
##     Null deviance: 652.97  on 1657  degrees of freedom
## Residual deviance: 620.97  on 1650  degrees of freedom
## AIC: 636.97
## 
## Number of Fisher Scoring iterations: 6  
  # test model fit
with(m, null.deviance - deviance)  
  ## [1] 31.997  
  with(m, df.null - df.residual)  
  ## [1] 7  
  with(m, pchisq(null.deviance - deviance, df.null - df.residual, lower.tail = FALSE))  
  ## [1] 4.066801e-05  
  BIC(m)  
  ## [1] 680.2762  
  # Hosmer-Lemeshow Goodness-of-Fit Test
hltest(m, G=7)  
  ## 
##    The Hosmer-Lemeshow goodness-of-fit test
## 
##  Group Size Observed Expected
##      1  195        5        5
##      2  558       19       19
##      3   23        1        1
##      4  809       41       41
##      5    7        1        1
##      6   12        2        2
##      7   51       12       12
##      8    3        1        1
## 
##          Statistic =  0 
## degrees of freedom =  6 
##            p-value =  1  
  ## CIs using profiled log-likelihood
confint(m, level=0.99)  
  ## Waiting for profiling to be done...  
  ##                                                                                  0.5 %
## (Intercept)                                                                 -3.3706173
## D_DietMeat-based – raw                                                      -1.1824944
## D_DietVegan (consuming no animal products)                                  -2.1865557
## D_DietVegetarian (including eggs or milk, but not meat)                     -4.5000779
## Therapeutic_FoodYes                                                          0.7449589
## D_DietMeat-based – raw:Therapeutic_FoodYes                                  -4.6725223
## D_DietVegan (consuming no animal products):Therapeutic_FoodYes              -2.6806193
## D_DietVegetarian (including eggs or milk, but not meat):Therapeutic_FoodYes -4.3399590
##                                                                                 99.5 %
## (Intercept)                                                                 -2.5411256
## D_DietMeat-based – raw                                                       0.2915238
## D_DietVegan (consuming no animal products)                                   0.3809495
## D_DietVegetarian (including eggs or milk, but not meat)                      1.8272744
## Therapeutic_FoodYes                                                          2.6616680
## D_DietMeat-based – raw:Therapeutic_FoodYes                                   2.3574015
## D_DietVegan (consuming no animal products):Therapeutic_FoodYes               2.6767049
## D_DietVegetarian (including eggs or milk, but not meat):Therapeutic_FoodYes  5.7188097  
  ## CIs using standard errors
confint.default(m, level=0.99)  
  ##                                                                                  0.5 %
## (Intercept)                                                                 -3.3430929
## D_DietMeat-based – raw                                                      -1.1444287
## D_DietVegan (consuming no animal products)                                  -1.9452468
## D_DietVegetarian (including eggs or milk, but not meat)                     -2.8267108
## Therapeutic_FoodYes                                                          0.8063116
## D_DietMeat-based – raw:Therapeutic_FoodYes                                  -3.1973332
## D_DietVegan (consuming no animal products):Therapeutic_FoodYes              -2.2206758
## D_DietVegetarian (including eggs or milk, but not meat):Therapeutic_FoodYes -3.5705770
##                                                                                 99.5 %
## (Intercept)                                                                 -2.5173425
## D_DietMeat-based – raw                                                       0.3143109
## D_DietVegan (consuming no animal products)                                   0.5305099
## D_DietVegetarian (including eggs or milk, but not meat)                      2.5050613
## Therapeutic_FoodYes                                                          2.6968137
## D_DietMeat-based – raw:Therapeutic_FoodYes                                   2.8012421
## D_DietVegan (consuming no animal products):Therapeutic_FoodYes               2.7738470
## D_DietVegetarian (including eggs or milk, but not meat):Therapeutic_FoodYes  4.8632422  
  # Wald test
wald.test(b = coef(m), Sigma = vcov(m), Terms = 2)  
  ## Wald test:
## ----------
## 
## Chi-squared test:
## X2 = 2.1, df = 1, P(> X2) = 0.14  
  ## odds ratios and 95% CI
exp(cbind(OR = coef(m), confint(m, level=0.99)))  
  ## Waiting for profiling to be done...  
  ##                                                                                     OR
## (Intercept)                                                                 0.05338542
## D_DietMeat-based – raw                                                      0.66030137
## D_DietVegan (consuming no animal products)                                  0.49293967
## D_DietVegetarian (including eggs or milk, but not meat)                     0.85144124
## Therapeutic_FoodYes                                                         5.76360225
## D_DietMeat-based – raw:Therapeutic_FoodYes                                  0.82033249
## D_DietVegan (consuming no animal products):Therapeutic_FoodYes              1.31861979
## D_DietVegetarian (including eggs or milk, but not meat):Therapeutic_FoodYes 1.90852865
##                                                                                  0.5 %
## (Intercept)                                                                 0.03436842
## D_DietMeat-based – raw                                                      0.30651322
## D_DietVegan (consuming no animal products)                                  0.11230288
## D_DietVegetarian (including eggs or milk, but not meat)                     0.01110813
## Therapeutic_FoodYes                                                         2.10635476
## D_DietMeat-based – raw:Therapeutic_FoodYes                                  0.00934866
## D_DietVegan (consuming no animal products):Therapeutic_FoodYes              0.06852071
## D_DietVegetarian (including eggs or milk, but not meat):Therapeutic_FoodYes 0.01303706
##                                                                                   99.5 %
## (Intercept)                                                                   0.07877768
## D_DietMeat-based – raw                                                        1.33846548
## D_DietVegan (consuming no animal products)                                    1.46367371
## D_DietVegetarian (including eggs or milk, but not meat)                       6.21691841
## Therapeutic_FoodYes                                                          14.32015494
## D_DietMeat-based – raw:Therapeutic_FoodYes                                   10.56346639
## D_DietVegan (consuming no animal products):Therapeutic_FoodYes               14.53711355
## D_DietVegetarian (including eggs or milk, but not meat):Therapeutic_FoodYes 304.54221081  
 
 Calculate Nagelkerke R^2 
  NagelkerkeR2(m)  
  ## $N
## [1] 1658
## 
## $R2
## [1] 0.05871515  
 
 
 check assumptions of model 
 
 Cook’s distance 
  plot(m, which = 4, id.n = 3)  
   
 
 
 Extract model results and display data for top 3 values using Cook’s
distance 
  model.data <- augment(m) %>% 
  mutate(index = 1:n()) 
model.data %>% top_n(3, .cooksd)  
 
 
 
 
 
 plot standardised residuals 
  ggplot(model.data, aes(index, .std.resid)) + 
  geom_point(aes(color = Health_Binary), alpha = .5) +
  theme_bw()  
   
 
 
 Filter potential influential data points with abs(.std.res) >
3: 
  model.data %>% 
  filter(abs(.std.resid) > 3)  
 
 
 
 
 
 check for multicollinearity 
 
 Note interactions 
  car::vif(m)  
  ## there are higher-order terms (interactions) in this model
## consider setting type = 'predictor'; see ?vif  
  ##                             GVIF Df GVIF^(1/(2*Df))
## D_Diet                  2.463172  3        1.162115
## Therapeutic_Food        1.392513  1        1.180048
## D_Diet:Therapeutic_Food 3.037464  3        1.203424  
 
 
 
 
 Create ROCR for training and test data 
  ## training data
pred.mtt = predict(m, type = "response") #repeat risk predictions from model m
rocr.pred.mtt = ROCR::prediction(pred.mtt, labels = ml_train$Health_Binary) #ROCR prediction object
roc.perf.mtt = ROCR::performance(rocr.pred.mtt, measure = "tpr", x.measure = "fpr") # #ROCR performance object
plot(roc.perf.mtt, col = "blue")


pred.te.1 = predict(m, newdata = ml_test, type = "response") #.te = "test"
rocr.pred.te.1 = ROCR::prediction(pred.te.1, labels = ml_test$Health_Binary)
roc.perf.te.1 = ROCR::performance(rocr.pred.te.1, measure = "tpr", x.measure = "fpr")
plot(roc.perf.te.1, col = "red", add = T)

abline(a = 0, b = 1, lty = 2) #diagonal for random assignment
legend("bottomright", legend = c("train","test"),
col = c("blue","red"), lty = c(2,1), lwd =1.5)  
   
 
 
 Report AUC from ROC for training and test data 
    # Train AUC
aucTr <- ROCR::performance(rocr.pred.mtt, measure = "auc")
  aucTr <- aucTr@y.values[[1]]
  print(aucTr)  
  ## [1] 0.6290354  
     # Test AUC
  aucTe <- ROCR::performance(rocr.pred.te.1, measure = "auc")
  aucTe <- aucTe@y.values[[1]]
  print(aucTe)  
  ## [1] 0.627924  
 
 
 
 SIZE Binary logistic regression ON SIGNIFICANT OR SERIOUS
ILLNESS 
  # fit binary logit model and store results 'm'
m <- glm(Health_Binary ~ Size, data = ml_train,family = binomial)
# view a summary of the model
summary(m)  
  ## 
## Call:
## glm(formula = Health_Binary ~ Size, family = binomial, data = ml_train)
## 
## Coefficients:
##             Estimate Std. Error z value Pr(>|z|)
## (Intercept)   -5.753    126.699  -0.045    0.964
## Size.L         9.917    400.658   0.025    0.980
## Size.Q        -7.242    338.618  -0.021    0.983
## Size.C         4.603    200.329   0.023    0.982
## Size^4        -1.794     75.718  -0.024    0.981
## 
## (Dispersion parameter for binomial family taken to be 1)
## 
##     Null deviance: 652.97  on 1657  degrees of freedom
## Residual deviance: 641.49  on 1653  degrees of freedom
## AIC: 651.49
## 
## Number of Fisher Scoring iterations: 16  
  # test model fit
with(m, null.deviance - deviance)  
  ## [1] 11.47761  
  with(m, df.null - df.residual)  
  ## [1] 4  
  with(m, pchisq(null.deviance - deviance, df.null - df.residual, lower.tail = FALSE))  
  ## [1] 0.02168956  
  BIC(m)  
  ## [1] 678.5555  
  # Hosmer-Lemeshow Goodness-of-Fit Test
hltest(m)  
  ## 
##    The Hosmer-Lemeshow goodness-of-fit test
## 
##  Group Size Observed     Expected
##      1   39        0 9.166805e-07
##      2  327       13 1.300000e+01
##      3  644       26 2.600000e+01
##      4  574       35 3.500000e+01
##      5   74        8 8.000000e+00
## 
##          Statistic =  0 
## degrees of freedom =  3 
##            p-value =  1  
  ## CIs using profiled log-likelihood
confint(m, level=0.99)  
  ## Waiting for profiling to be done...  
  ## Warning: glm.fit: fitted probabilities numerically 0 or 1 occurred

## Warning: glm.fit: fitted probabilities numerically 0 or 1 occurred

## Warning: glm.fit: fitted probabilities numerically 0 or 1 occurred

## Warning: glm.fit: fitted probabilities numerically 0 or 1 occurred

## Warning: glm.fit: fitted probabilities numerically 0 or 1 occurred

## Warning: glm.fit: fitted probabilities numerically 0 or 1 occurred

## Warning: glm.fit: fitted probabilities numerically 0 or 1 occurred

## Warning: glm.fit: fitted probabilities numerically 0 or 1 occurred

## Warning: glm.fit: fitted probabilities numerically 0 or 1 occurred

## Warning: glm.fit: fitted probabilities numerically 0 or 1 occurred

## Warning: glm.fit: fitted probabilities numerically 0 or 1 occurred

## Warning: glm.fit: fitted probabilities numerically 0 or 1 occurred

## Warning: glm.fit: fitted probabilities numerically 0 or 1 occurred

## Warning: glm.fit: fitted probabilities numerically 0 or 1 occurred

## Warning: glm.fit: fitted probabilities numerically 0 or 1 occurred

## Warning: glm.fit: fitted probabilities numerically 0 or 1 occurred

## Warning: glm.fit: fitted probabilities numerically 0 or 1 occurred

## Warning: glm.fit: fitted probabilities numerically 0 or 1 occurred

## Warning: glm.fit: fitted probabilities numerically 0 or 1 occurred

## Warning: glm.fit: fitted probabilities numerically 0 or 1 occurred

## Warning: glm.fit: fitted probabilities numerically 0 or 1 occurred

## Warning: glm.fit: fitted probabilities numerically 0 or 1 occurred

## Warning: glm.fit: fitted probabilities numerically 0 or 1 occurred

## Warning: glm.fit: fitted probabilities numerically 0 or 1 occurred

## Warning: glm.fit: fitted probabilities numerically 0 or 1 occurred

## Warning: glm.fit: fitted probabilities numerically 0 or 1 occurred

## Warning: glm.fit: fitted probabilities numerically 0 or 1 occurred

## Warning: glm.fit: fitted probabilities numerically 0 or 1 occurred

## Warning: glm.fit: fitted probabilities numerically 0 or 1 occurred

## Warning: glm.fit: fitted probabilities numerically 0 or 1 occurred

## Warning: glm.fit: fitted probabilities numerically 0 or 1 occurred

## Warning: glm.fit: fitted probabilities numerically 0 or 1 occurred

## Warning: glm.fit: fitted probabilities numerically 0 or 1 occurred

## Warning: glm.fit: fitted probabilities numerically 0 or 1 occurred

## Warning: glm.fit: fitted probabilities numerically 0 or 1 occurred

## Warning: glm.fit: fitted probabilities numerically 0 or 1 occurred

## Warning: glm.fit: fitted probabilities numerically 0 or 1 occurred

## Warning: glm.fit: fitted probabilities numerically 0 or 1 occurred

## Warning: glm.fit: fitted probabilities numerically 0 or 1 occurred

## Warning: glm.fit: fitted probabilities numerically 0 or 1 occurred

## Warning: glm.fit: fitted probabilities numerically 0 or 1 occurred

## Warning: glm.fit: fitted probabilities numerically 0 or 1 occurred

## Warning: glm.fit: fitted probabilities numerically 0 or 1 occurred

## Warning: glm.fit: fitted probabilities numerically 0 or 1 occurred

## Warning: glm.fit: fitted probabilities numerically 0 or 1 occurred

## Warning: glm.fit: fitted probabilities numerically 0 or 1 occurred

## Warning: glm.fit: fitted probabilities numerically 0 or 1 occurred

## Warning: glm.fit: fitted probabilities numerically 0 or 1 occurred

## Warning: glm.fit: fitted probabilities numerically 0 or 1 occurred

## Warning: glm.fit: fitted probabilities numerically 0 or 1 occurred  
  ##                   0.5 %      99.5 %
## (Intercept)  -64.398672  -0.6802746
## Size.L       -11.371505 166.6020863
## Size.Q      -139.664648  10.7504971
## Size.C        -6.041574  82.9452672
## Size^4       -31.404931   2.2290495  
  ## CIs using standard errors
confint.default(m, level=0.99)  
  ##                  0.5 %    99.5 %
## (Intercept)  -332.1083  320.6029
## Size.L      -1022.1093 1041.9442
## Size.Q       -879.4638  864.9798
## Size.C       -511.4107  520.6165
## Size^4       -196.8297  193.2413  
  # Wald test
wald.test(b = coef(m), Sigma = vcov(m), Terms = 2)  
  ## Wald test:
## ----------
## 
## Chi-squared test:
## X2 = 0.00061, df = 1, P(> X2) = 0.98  
  ## odds ratios and 95% CI
exp(cbind(OR = coef(m), confint(m, level=0.99)))  
  ## Waiting for profiling to be done...  
  ## Warning: glm.fit: fitted probabilities numerically 0 or 1 occurred

## Warning: glm.fit: fitted probabilities numerically 0 or 1 occurred

## Warning: glm.fit: fitted probabilities numerically 0 or 1 occurred

## Warning: glm.fit: fitted probabilities numerically 0 or 1 occurred

## Warning: glm.fit: fitted probabilities numerically 0 or 1 occurred

## Warning: glm.fit: fitted probabilities numerically 0 or 1 occurred

## Warning: glm.fit: fitted probabilities numerically 0 or 1 occurred

## Warning: glm.fit: fitted probabilities numerically 0 or 1 occurred

## Warning: glm.fit: fitted probabilities numerically 0 or 1 occurred

## Warning: glm.fit: fitted probabilities numerically 0 or 1 occurred

## Warning: glm.fit: fitted probabilities numerically 0 or 1 occurred

## Warning: glm.fit: fitted probabilities numerically 0 or 1 occurred

## Warning: glm.fit: fitted probabilities numerically 0 or 1 occurred

## Warning: glm.fit: fitted probabilities numerically 0 or 1 occurred

## Warning: glm.fit: fitted probabilities numerically 0 or 1 occurred

## Warning: glm.fit: fitted probabilities numerically 0 or 1 occurred

## Warning: glm.fit: fitted probabilities numerically 0 or 1 occurred

## Warning: glm.fit: fitted probabilities numerically 0 or 1 occurred

## Warning: glm.fit: fitted probabilities numerically 0 or 1 occurred

## Warning: glm.fit: fitted probabilities numerically 0 or 1 occurred

## Warning: glm.fit: fitted probabilities numerically 0 or 1 occurred

## Warning: glm.fit: fitted probabilities numerically 0 or 1 occurred

## Warning: glm.fit: fitted probabilities numerically 0 or 1 occurred

## Warning: glm.fit: fitted probabilities numerically 0 or 1 occurred

## Warning: glm.fit: fitted probabilities numerically 0 or 1 occurred

## Warning: glm.fit: fitted probabilities numerically 0 or 1 occurred

## Warning: glm.fit: fitted probabilities numerically 0 or 1 occurred

## Warning: glm.fit: fitted probabilities numerically 0 or 1 occurred

## Warning: glm.fit: fitted probabilities numerically 0 or 1 occurred

## Warning: glm.fit: fitted probabilities numerically 0 or 1 occurred

## Warning: glm.fit: fitted probabilities numerically 0 or 1 occurred

## Warning: glm.fit: fitted probabilities numerically 0 or 1 occurred

## Warning: glm.fit: fitted probabilities numerically 0 or 1 occurred

## Warning: glm.fit: fitted probabilities numerically 0 or 1 occurred

## Warning: glm.fit: fitted probabilities numerically 0 or 1 occurred

## Warning: glm.fit: fitted probabilities numerically 0 or 1 occurred

## Warning: glm.fit: fitted probabilities numerically 0 or 1 occurred

## Warning: glm.fit: fitted probabilities numerically 0 or 1 occurred

## Warning: glm.fit: fitted probabilities numerically 0 or 1 occurred

## Warning: glm.fit: fitted probabilities numerically 0 or 1 occurred

## Warning: glm.fit: fitted probabilities numerically 0 or 1 occurred

## Warning: glm.fit: fitted probabilities numerically 0 or 1 occurred

## Warning: glm.fit: fitted probabilities numerically 0 or 1 occurred

## Warning: glm.fit: fitted probabilities numerically 0 or 1 occurred

## Warning: glm.fit: fitted probabilities numerically 0 or 1 occurred

## Warning: glm.fit: fitted probabilities numerically 0 or 1 occurred

## Warning: glm.fit: fitted probabilities numerically 0 or 1 occurred

## Warning: glm.fit: fitted probabilities numerically 0 or 1 occurred

## Warning: glm.fit: fitted probabilities numerically 0 or 1 occurred

## Warning: glm.fit: fitted probabilities numerically 0 or 1 occurred  
  ##                       OR        0.5 %       99.5 %
## (Intercept) 3.174209e-03 1.076496e-28 5.064779e-01
## Size.L      2.028157e+04 1.151909e-05 2.261345e+72
## Size.Q      7.159005e-04 2.210111e-61 4.665321e+04
## Size.C      9.977498e+01 2.377813e-03 1.053590e+36
## Size^4      1.662597e-01 2.296210e-14 9.291030e+00  
 
 Calculate Nagelkerke R^2 
  NagelkerkeR2(m)  
  ## $N
## [1] 1658
## 
## $R2
## [1] 0.02119211  
 
 
 check assumptions of model 
 
 Cook’s distance 
  plot(m, which = 4, id.n = 3)  
   
 
 
 Extract model results and display data for top 3 values using Cook’s
distance 
  model.data <- augment(m) %>% 
  mutate(index = 1:n()) 
model.data %>% top_n(3, .cooksd)  
 
 
 
 
 
 plot standardised residuals 
  ggplot(model.data, aes(index, .std.resid)) + 
  geom_point(aes(color = Health_Binary), alpha = .5) +
  theme_bw()  
   
 
 
 Filter potential influential data points with abs(.std.res) >
3: 
  model.data %>% 
  filter(abs(.std.resid) > 3)  
 
 
 
 
 
 
 Create ROCR for training and test data 
  ## training data
pred.mtt = predict(m, type = "response") #repeat risk predictions from model m
rocr.pred.mtt = ROCR::prediction(pred.mtt, labels = ml_train$Health_Binary) #ROCR prediction object
roc.perf.mtt = ROCR::performance(rocr.pred.mtt, measure = "tpr", x.measure = "fpr") # #ROCR performance object
plot(roc.perf.mtt, col = "blue")


pred.te.1 = predict(m, newdata = ml_test, type = "response") #.te = "test"
rocr.pred.te.1 = ROCR::prediction(pred.te.1, labels = ml_test$Health_Binary)
roc.perf.te.1 = ROCR::performance(rocr.pred.te.1, measure = "tpr", x.measure = "fpr")
plot(roc.perf.te.1, col = "red", add = T)

abline(a = 0, b = 1, lty = 2) #diagonal for random assignment
legend("bottomright", legend = c("train","test"),
col = c("blue","red"), lty = c(2,1), lwd =1.5)  
   
 
 
 Report AUC from ROC for training and test data 
    # Train AUC
aucTr <- ROCR::performance(rocr.pred.mtt, measure = "auc")
  aucTr <- aucTr@y.values[[1]]
  print(aucTr)  
  ## [1] 0.584387  
     # Test AUC
  aucTe <- ROCR::performance(rocr.pred.te.1, measure = "auc")
  aucTe <- aucTe@y.values[[1]]
  print(aucTe)  
  ## [1] 0.5274854  
 
 
 
 SIZE2 Binary logistic regression for HEALTH 
  # fit binary logit model and store results 'm'
m <- glm(Health_Binary ~ Size2, data = ml_train,family = binomial)
# view a summary of the model
summary(m)  
  ## 
## Call:
## glm(formula = Health_Binary ~ Size2, family = binomial, data = ml_train)
## 
## Coefficients:
##              Estimate Std. Error z value Pr(>|z|)    
## (Intercept)  -3.16839    0.20020 -15.826   <2e-16 ***
## Size2Toy    -14.39768  633.49588  -0.023   0.9819    
## Size2Small   -0.01605    0.34668  -0.046   0.9631    
## Size2Large    0.43402    0.26553   1.635   0.1021    
## Size2Giant    1.05818    0.42454   2.493   0.0127 *  
## ---
## Signif. codes:  0 '***' 0.001 '**' 0.01 '*' 0.05 '.' 0.1 ' ' 1
## 
## (Dispersion parameter for binomial family taken to be 1)
## 
##     Null deviance: 652.97  on 1657  degrees of freedom
## Residual deviance: 641.49  on 1653  degrees of freedom
## AIC: 651.49
## 
## Number of Fisher Scoring iterations: 16  
  # test model fit
with(m, null.deviance - deviance)  
  ## [1] 11.47761  
  with(m, df.null - df.residual)  
  ## [1] 4  
  with(m, pchisq(null.deviance - deviance, df.null - df.residual, lower.tail = FALSE))  
  ## [1] 0.02168956  
  BIC(m)  
  ## [1] 678.5555  
  # Hosmer-Lemeshow Goodness-of-Fit Test
hltest(m)  
  ## 
##    The Hosmer-Lemeshow goodness-of-fit test
## 
##  Group Size Observed     Expected
##      1   39        0 9.166805e-07
##      2  327       13 1.300000e+01
##      3  644       26 2.600000e+01
##      4  574       35 3.500000e+01
##      5   74        8 8.000000e+00
## 
##          Statistic =  0 
## degrees of freedom =  3 
##            p-value =  1  
  ## CIs using profiled log-likelihood
confint(m, level=0.99)  
  ## Waiting for profiling to be done...  
  ## Warning: glm.fit: fitted probabilities numerically 0 or 1 occurred

## Warning: glm.fit: fitted probabilities numerically 0 or 1 occurred

## Warning: glm.fit: fitted probabilities numerically 0 or 1 occurred

## Warning: glm.fit: fitted probabilities numerically 0 or 1 occurred

## Warning: glm.fit: fitted probabilities numerically 0 or 1 occurred

## Warning: glm.fit: fitted probabilities numerically 0 or 1 occurred

## Warning: glm.fit: fitted probabilities numerically 0 or 1 occurred

## Warning: glm.fit: fitted probabilities numerically 0 or 1 occurred

## Warning: glm.fit: fitted probabilities numerically 0 or 1 occurred

## Warning: glm.fit: fitted probabilities numerically 0 or 1 occurred

## Warning: glm.fit: fitted probabilities numerically 0 or 1 occurred

## Warning: glm.fit: fitted probabilities numerically 0 or 1 occurred

## Warning: glm.fit: fitted probabilities numerically 0 or 1 occurred

## Warning: glm.fit: fitted probabilities numerically 0 or 1 occurred

## Warning: glm.fit: fitted probabilities numerically 0 or 1 occurred

## Warning: glm.fit: fitted probabilities numerically 0 or 1 occurred

## Warning: glm.fit: fitted probabilities numerically 0 or 1 occurred

## Warning: glm.fit: fitted probabilities numerically 0 or 1 occurred

## Warning: glm.fit: fitted probabilities numerically 0 or 1 occurred  
  ##                    0.5 %     99.5 %
## (Intercept)   -3.7287742 -2.6900815
## Size2Toy    -262.1377713 19.2631390
## Size2Small    -0.9646355  0.8491688
## Size2Large    -0.2452114  1.1343671
## Size2Giant    -0.1481627  2.0877175  
  ## CIs using standard errors
confint.default(m, level=0.99)  
  ##                     0.5 %       99.5 %
## (Intercept) -3.684071e+00   -2.6527133
## Size2Toy    -1.646175e+03 1617.3795843
## Size2Small  -9.090418e-01    0.8769384
## Size2Large  -2.499365e-01    1.1179853
## Size2Giant  -3.535465e-02    2.1517121  
  # Wald test
wald.test(b = coef(m), Sigma = vcov(m), Terms = 2)  
  ## Wald test:
## ----------
## 
## Chi-squared test:
## X2 = 0.00052, df = 1, P(> X2) = 0.98  
  ## odds ratios and 95% CI
exp(cbind(OR = coef(m), confint(m, level=0.99)))  
  ## Waiting for profiling to be done...  
  ## Warning: glm.fit: fitted probabilities numerically 0 or 1 occurred

## Warning: glm.fit: fitted probabilities numerically 0 or 1 occurred

## Warning: glm.fit: fitted probabilities numerically 0 or 1 occurred

## Warning: glm.fit: fitted probabilities numerically 0 or 1 occurred

## Warning: glm.fit: fitted probabilities numerically 0 or 1 occurred

## Warning: glm.fit: fitted probabilities numerically 0 or 1 occurred

## Warning: glm.fit: fitted probabilities numerically 0 or 1 occurred

## Warning: glm.fit: fitted probabilities numerically 0 or 1 occurred

## Warning: glm.fit: fitted probabilities numerically 0 or 1 occurred

## Warning: glm.fit: fitted probabilities numerically 0 or 1 occurred

## Warning: glm.fit: fitted probabilities numerically 0 or 1 occurred

## Warning: glm.fit: fitted probabilities numerically 0 or 1 occurred

## Warning: glm.fit: fitted probabilities numerically 0 or 1 occurred

## Warning: glm.fit: fitted probabilities numerically 0 or 1 occurred

## Warning: glm.fit: fitted probabilities numerically 0 or 1 occurred

## Warning: glm.fit: fitted probabilities numerically 0 or 1 occurred

## Warning: glm.fit: fitted probabilities numerically 0 or 1 occurred

## Warning: glm.fit: fitted probabilities numerically 0 or 1 occurred

## Warning: glm.fit: fitted probabilities numerically 0 or 1 occurred  
  ##                       OR         0.5 %       99.5 %
## (Intercept) 4.207120e-02  2.402226e-02 6.787541e-02
## Size2Toy    5.586869e-07 1.428935e-114 2.322068e+08
## Size2Small  9.840764e-01  3.811221e-01 2.337703e+00
## Size2Large  1.543457e+00  7.825391e-01 3.109205e+00
## Size2Giant  2.881119e+00  8.622908e-01 8.066483e+00  
 
 Calculate Nagelkerke R^2 
  NagelkerkeR2(m)  
  ## $N
## [1] 1658
## 
## $R2
## [1] 0.02119211  
 
 
 check assumptions of model 
 
 Cook’s distance 
  plot(m, which = 4, id.n = 3)  
   
 
 
 Extract model results and display data for top 3 values using Cook’s
distance 
  model.data <- augment(m) %>% 
  mutate(index = 1:n()) 
model.data %>% top_n(3, .cooksd)  
 
 
 
 
 
 plot standardised residuals 
  ggplot(model.data, aes(index, .std.resid)) + 
  geom_point(aes(color = Health_Binary), alpha = .5) +
  theme_bw()  
   
 
 
 Filter potential influential data points with abs(.std.res) >
3: 
  model.data %>% 
  filter(abs(.std.resid) > 3)  
 
 
 
 
 
 
 Create ROCR for training and test data 
  ## training data
pred.mtt = predict(m, type = "response") #repeat risk predictions from model m
rocr.pred.mtt = ROCR::prediction(pred.mtt, labels = ml_train$Health_Binary) #ROCR prediction object
roc.perf.mtt = ROCR::performance(rocr.pred.mtt, measure = "tpr", x.measure = "fpr") # #ROCR performance object
plot(roc.perf.mtt, col = "blue")


pred.te.1 = predict(m, newdata = ml_test, type = "response") #.te = "test"
rocr.pred.te.1 = ROCR::prediction(pred.te.1, labels = ml_test$Health_Binary)
roc.perf.te.1 = ROCR::performance(rocr.pred.te.1, measure = "tpr", x.measure = "fpr")
plot(roc.perf.te.1, col = "red", add = T)

abline(a = 0, b = 1, lty = 2) #diagonal for random assignment
legend("bottomright", legend = c("train","test"),
col = c("blue","red"), lty = c(2,1), lwd =1.5)  
   
 
 
 Report AUC from ROC for training and test data 
    # Train AUC
aucTr <- ROCR::performance(rocr.pred.mtt, measure = "auc")
  aucTr <- aucTr@y.values[[1]]
  print(aucTr)  
  ## [1] 0.584387  
     # Test AUC
  aucTe <- ROCR::performance(rocr.pred.te.1, measure = "auc")
  aucTe <- aucTe@y.values[[1]]
  print(aucTe)  
  ## [1] 0.5274854  
 
 
 
 SIZE_GIANT Binary logistic regression for HEALTH 
  # fit binary logit model and store results 'm'
m <- glm(Health_Binary ~ Size_Giant, data = ml_train,family = binomial)
# view a summary of the model
summary(m)  
  ## 
## Call:
## glm(formula = Health_Binary ~ Size_Giant, family = binomial, 
##     data = ml_train)
## 
## Coefficients:
##               Estimate Std. Error z value Pr(>|z|)    
## (Intercept)    -3.0158     0.1191 -25.332   <2e-16 ***
## Size_GiantYes   0.9056     0.3928   2.305   0.0212 *  
## ---
## Signif. codes:  0 '***' 0.001 '**' 0.01 '*' 0.05 '.' 0.1 ' ' 1
## 
## (Dispersion parameter for binomial family taken to be 1)
## 
##     Null deviance: 652.97  on 1657  degrees of freedom
## Residual deviance: 648.60  on 1656  degrees of freedom
## AIC: 652.6
## 
## Number of Fisher Scoring iterations: 5  
  # test model fit
with(m, null.deviance - deviance)  
  ## [1] 4.363058  
  with(m, df.null - df.residual)  
  ## [1] 1  
  with(m, pchisq(null.deviance - deviance, df.null - df.residual, lower.tail = FALSE))  
  ## [1] 0.03672633  
  BIC(m)  
  ## [1] 663.4299  
  ## CIs using profiled log-likelihood
confint(m, level=0.99)  
  ## Waiting for profiling to be done...  
  ##                    0.5 %    99.5 %
## (Intercept)   -3.3374992 -2.722595
## Size_GiantYes -0.2410338  1.828183  
  ## CIs using standard errors
confint.default(m, level=0.99)  
  ##                    0.5 %    99.5 %
## (Intercept)   -3.3224563 -2.709143
## Size_GiantYes -0.1063071  1.917480  
  # Wald test
wald.test(b = coef(m), Sigma = vcov(m), Terms = 2)  
  ## Wald test:
## ----------
## 
## Chi-squared test:
## X2 = 5.3, df = 1, P(> X2) = 0.021  
  ## odds ratios and 95% CI
exp(cbind(OR = coef(m), confint(m, level=0.99)))  
  ## Waiting for profiling to be done...  
  ##                       OR      0.5 %   99.5 %
## (Intercept)   0.04900662 0.03552569 0.065704
## Size_GiantYes 2.47338243 0.78581506 6.222568  
 
 Calculate Nagelkerke R^2 
  NagelkerkeR2(m)  
  ## $N
## [1] 1658
## 
## $R2
## [1] 0.008073177  
 
 
 check assumptions of model 
 
 Cook’s distance 
  plot(m, which = 4, id.n = 3)  
   
 
 
 Extract model results and display data for top 3 values using Cook’s
distance 
  model.data <- augment(m) %>% 
  mutate(index = 1:n()) 
model.data %>% top_n(3, .cooksd)  
 
 
 
 
 
 plot standardised residuals 
  ggplot(model.data, aes(index, .std.resid)) + 
  geom_point(aes(color = Health_Binary), alpha = .5) +
  theme_bw()  
   
 
 
 Filter potential influential data points with abs(.std.res) >
3: 
  model.data %>% 
  filter(abs(.std.resid) > 3)  
 
 
 
 
 
 
 Create ROCR for training and test data 
  ## training data
pred.mtt = predict(m, type = "response") #repeat risk predictions from model m
rocr.pred.mtt = ROCR::prediction(pred.mtt, labels = ml_train$Health_Binary) #ROCR prediction object
roc.perf.mtt = ROCR::performance(rocr.pred.mtt, measure = "tpr", x.measure = "fpr") # #ROCR performance object
plot(roc.perf.mtt, col = "blue")


pred.te.1 = predict(m, newdata = ml_test, type = "response") #.te = "test"
rocr.pred.te.1 = ROCR::prediction(pred.te.1, labels = ml_test$Health_Binary)
roc.perf.te.1 = ROCR::performance(rocr.pred.te.1, measure = "tpr", x.measure = "fpr")
plot(roc.perf.te.1, col = "red", add = T)

abline(a = 0, b = 1, lty = 2) #diagonal for random assignment
legend("bottomright", legend = c("train","test"),
col = c("blue","red"), lty = c(2,1), lwd =1.5)  
   
 
 
 Report AUC from ROC for training and test data 
    # Train AUC
aucTr <- ROCR::performance(rocr.pred.mtt, measure = "auc")
  aucTr <- aucTr@y.values[[1]]
  print(aucTr)  
  ## [1] 0.5278414  
     # Test AUC
  aucTe <- ROCR::performance(rocr.pred.te.1, measure = "auc")
  aucTe <- aucTe@y.values[[1]]
  print(aucTe)  
  ## [1] 0.5045322  
 
 
 
 D_AGE Binary logistic regression for HEALTH 
  # fit binary logit model and store results 'm'
m <- glm(Health_Binary ~ D_Age, data = ml_train,family = binomial)
# view a summary of the model
summary(m)  
  ## 
## Call:
## glm(formula = Health_Binary ~ D_Age, family = binomial, data = ml_train)
## 
## Coefficients:
##             Estimate Std. Error z value Pr(>|z|)    
## (Intercept) -4.11440    0.26338 -15.622  < 2e-16 ***
## D_Age        0.16182    0.02884   5.611 2.01e-08 ***
## ---
## Signif. codes:  0 '***' 0.001 '**' 0.01 '*' 0.05 '.' 0.1 ' ' 1
## 
## (Dispersion parameter for binomial family taken to be 1)
## 
##     Null deviance: 652.97  on 1657  degrees of freedom
## Residual deviance: 621.98  on 1656  degrees of freedom
## AIC: 625.98
## 
## Number of Fisher Scoring iterations: 6  
  # test model fit
with(m, null.deviance - deviance)  
  ## [1] 30.98204  
  with(m, df.null - df.residual)  
  ## [1] 1  
  with(m, pchisq(null.deviance - deviance, df.null - df.residual, lower.tail = FALSE))  
  ## [1] 2.604267e-08  
  BIC(m)  
  ## [1] 636.8109  
  # Hosmer-Lemeshow Goodness-of-Fit Test
hltest(m)  
  ## 
##    The Hosmer-Lemeshow goodness-of-fit test
## 
##  Group Size Observed  Expected
##      1  114        4  2.148114
##      2  177        5  3.908114
##      3  194        4  5.016388
##      4  173        4  5.235328
##      5  173        8  6.122349
##      6  143        7  5.912801
##      7  134        3  6.466895
##      8  130        8  7.313831
##      9  191        9 13.476691
##     10  159       16 15.819641
##     11   70       14 10.579849
## 
##          Statistic =  8.1824 
## degrees of freedom =  9 
##            p-value =  0.51587  
  ## CIs using profiled log-likelihood
confint(m, level=0.99)  
  ## Waiting for profiling to be done...  
  ##                   0.5 %     99.5 %
## (Intercept) -4.82998470 -3.4690959
## D_Age        0.08757854  0.2367154  
  ## CIs using standard errors
confint.default(m, level=0.99)  
  ##                   0.5 %     99.5 %
## (Intercept) -4.79281259 -3.4359933
## D_Age        0.08753881  0.2360963  
  # Wald test
wald.test(b = coef(m), Sigma = vcov(m), Terms = 2)  
  ## Wald test:
## ----------
## 
## Chi-squared test:
## X2 = 31.5, df = 1, P(> X2) = 2e-08  
  ## odds ratios and 95% CI
exp(cbind(OR = coef(m), confint(m, level=0.99)))  
  ## Waiting for profiling to be done...  
  ##                     OR       0.5 %     99.5 %
## (Intercept) 0.01633569 0.007986643 0.03114518
## D_Age       1.17564575 1.091527988 1.26708043  
 
 Calculate Nagelkerke R^2 
  NagelkerkeR2(m)  
  ## $N
## [1] 1658
## 
## $R2
## [1] 0.05687004  
 
 
 Check age is linear with logit of outcome 
 
 Note lack of linearity 
  ypred = predict(m)
res = residuals(m, type = 'deviance')
plot(ypred,res)  
   
 
 
 
 Box Tidwell test to check that D_Age is linearly associated with the
logit of the outcome 
 
 suggests not linear 
  boxTidwell(ml$Health_Binary ~ ml$D_Age)  
  ##  MLE of lambda Score Statistic (t) Pr(>|t|)   
##         2.5903              3.1841 0.001473 **
## ---
## Signif. codes:  0 '***' 0.001 '**' 0.01 '*' 0.05 '.' 0.1 ' ' 1
## 
## iterations =  4  
 
 
 
 check assumptions of model 
 
 Cook’s distance 
  plot(m, which = 4, id.n = 3)  
   
 
 
 Extract model results and display data for top 3 values using Cook’s
distance 
  model.data <- augment(m) %>% 
  mutate(index = 1:n()) 
model.data %>% top_n(3, .cooksd)  
 
 
 
 
 
 plot standardised residuals 
  ggplot(model.data, aes(index, .std.resid)) + 
  geom_point(aes(color = Health_Binary), alpha = .5) +
  theme_bw()  
   
 
 
 Filter potential influential data points with abs(.std.res) >
3: 
  model.data %>% 
  filter(abs(.std.resid) > 3)  
 
 
 
 
 
 
 Create ROCR for training and test data 
  ## training data
pred.mtt = predict(m, type = "response") #repeat risk predictions from model mtt
rocr.pred.mtt = ROCR::prediction(pred.mtt, labels = ml_train$Health_Binary) #ROCR prediction object
roc.perf.mtt = ROCR::performance(rocr.pred.mtt, measure = "tpr", x.measure = "fpr") # #ROCR performance object
plot(roc.perf.mtt, col = "blue")


pred.te.1 = predict(m, newdata = ml_test, type = "response") #.te = "test"
rocr.pred.te.1 = ROCR::prediction(pred.te.1, labels = ml_test$Health_Binary)
roc.perf.te.1 = ROCR::performance(rocr.pred.te.1, measure = "tpr", x.measure = "fpr")
plot(roc.perf.te.1, col = "red", add = T)

abline(a = 0, b = 1, lty = 2) #diagonal for random assignment
legend("bottomright", legend = c("train","test"),
col = c("blue","red"), lty = c(2,1), lwd =1.5)  
   
 
 
 Report AUC from ROC for training and test data 
    # Train AUC
aucTr <- ROCR::performance(rocr.pred.mtt, measure = "auc")
  aucTr <- aucTr@y.values[[1]]
  print(aucTr)  
  ## [1] 0.6619413  
     # Test AUC
  aucTe <- ROCR::performance(rocr.pred.te.1, measure = "auc")
  aucTe <- aucTe@y.values[[1]]
  print(aucTe)  
  ## [1] 0.7037524  
 
 
 
 D_AGE Binary logistic regression for HEALTH with splines 
  # fit binary logit model and store results 'm'
m <- glm(Health_Binary ~ bs(D_Age, degree=1,df=2), data = ml_train,family = binomial)
# view a summary of the model
summary(m)  
  ## 
## Call:
## glm(formula = Health_Binary ~ bs(D_Age, degree = 1, df = 2), 
##     family = binomial, data = ml_train)
## 
## Coefficients:
##                                Estimate Std. Error z value Pr(>|z|)    
## (Intercept)                    -3.58476    0.38203  -9.383  < 2e-16 ***
## bs(D_Age, degree = 1, df = 2)1  0.09878    0.48808   0.202     0.84    
## bs(D_Age, degree = 1, df = 2)2  2.97817    0.52654   5.656 1.55e-08 ***
## ---
## Signif. codes:  0 '***' 0.001 '**' 0.01 '*' 0.05 '.' 0.1 ' ' 1
## 
## (Dispersion parameter for binomial family taken to be 1)
## 
##     Null deviance: 652.97  on 1657  degrees of freedom
## Residual deviance: 620.72  on 1655  degrees of freedom
## AIC: 626.72
## 
## Number of Fisher Scoring iterations: 6  
  # test model fit
with(m, null.deviance - deviance)  
  ## [1] 32.24294  
  with(m, df.null - df.residual)  
  ## [1] 2  
  with(m, pchisq(null.deviance - deviance, df.null - df.residual, lower.tail = FALSE))  
  ## [1] 9.966311e-08  
  BIC(m)  
  ## [1] 642.9634  
  # Hosmer-Lemeshow Goodness-of-Fit Test
hltest(m)  
  ## 
##    The Hosmer-Lemeshow goodness-of-fit test
## 
##  Group Size Observed  Expected
##      1  114        4  3.077377
##      2  177        5  4.894193
##      3  194        4  5.494578
##      4  173        4  5.018758
##      5  173        8  5.140511
##      6  143        7  5.116121
##      7  134        3  5.765019
##      8  130        8  6.715371
##      9  191        9 12.929094
##     10  159       16 16.194812
##     11   70       14 11.654166
## 
##          Statistic =  6.77153 
## degrees of freedom =  9 
##            p-value =  0.66089  
  ## CIs using profiled log-likelihood
confint(m, level=0.99)  
  ## Waiting for profiling to be done...  
  ##                                    0.5 %    99.5 %
## (Intercept)                    -4.696502 -2.702680
## bs(D_Age, degree = 1, df = 2)1 -1.098649  1.444246
## bs(D_Age, degree = 1, df = 2)2  1.634019  4.377575  
  ## CIs using standard errors
confint.default(m, level=0.99)  
  ##                                    0.5 %    99.5 %
## (Intercept)                    -4.568805 -2.600706
## bs(D_Age, degree = 1, df = 2)1 -1.158422  1.355983
## bs(D_Age, degree = 1, df = 2)2  1.621885  4.334457  
  # Wald test
wald.test(b = coef(m), Sigma = vcov(m), Terms = 2)  
  ## Wald test:
## ----------
## 
## Chi-squared test:
## X2 = 0.041, df = 1, P(> X2) = 0.84  
  ## odds ratios and 95% CI
exp(cbind(OR = coef(m), confint(m, level=0.99)))  
  ## Waiting for profiling to be done...  
  ##                                         OR       0.5 %      99.5 %
## (Intercept)                     0.02774346 0.009127152  0.06702563
## bs(D_Age, degree = 1, df = 2)1  1.10382408 0.333321118  4.23865467
## bs(D_Age, degree = 1, df = 2)2 19.65183934 5.124426943 79.64469121  
 
 Calculate Nagelkerke R^2 
  NagelkerkeR2(m)  
  ## $N
## [1] 1658
## 
## $R2
## [1] 0.05916209  
 
 
 check assumptions of model 
 
 Cook’s distance 
  plot(m, which = 4, id.n = 3)  
   
 
 
 Extract model results and display data for top 3 values using Cook’s
distance 
  model.data <- augment(m) %>% 
  mutate(index = 1:n()) 
model.data %>% top_n(3, .cooksd)  
 
 
 
 
 
 plot standardised residuals 
  ggplot(model.data, aes(index, .std.resid)) + 
  geom_point(aes(color = Health_Binary), alpha = .5) +
  theme_bw()  
   
 
 
 Filter potential influential data points with abs(.std.res) >
3: 
  model.data %>% 
  filter(abs(.std.resid) > 3)  
 
 
 
 
 
 
 Create ROCR for training and test data 
  ## training data
pred.mtt = predict(m, type = "response") #repeat risk predictions from model mtt
rocr.pred.mtt = ROCR::prediction(pred.mtt, labels = ml_train$Health_Binary) #ROCR prediction object
roc.perf.mtt = ROCR::performance(rocr.pred.mtt, measure = "tpr", x.measure = "fpr") # #ROCR performance object
plot(roc.perf.mtt, col = "blue")


pred.te.1 = predict(m, newdata = ml_test, type = "response") #.te = "test"
rocr.pred.te.1 = ROCR::prediction(pred.te.1, labels = ml_test$Health_Binary)
roc.perf.te.1 = ROCR::performance(rocr.pred.te.1, measure = "tpr", x.measure = "fpr")
plot(roc.perf.te.1, col = "red", add = T)

abline(a = 0, b = 1, lty = 2) #diagonal for random assignment
legend("bottomright", legend = c("train","test"),
col = c("blue","red"), lty = c(2,1), lwd =1.5)  
   
 
 
 Report AUC from ROC for training and test data 
    # Train AUC
aucTr <- ROCR::performance(rocr.pred.mtt, measure = "auc")
  aucTr <- aucTr@y.values[[1]]
  print(aucTr)  
  ## [1] 0.6619413  
     # Test AUC
  aucTe <- ROCR::performance(rocr.pred.te.1, measure = "auc")
  aucTe <- aucTe@y.values[[1]]
  print(aucTe)  
  ## [1] 0.7037524  
 
 
 
 D_Age_quant logistic regression for HEALTH 
 
 Note better model fit that D_Age 
  # fit binary logit model and store results 'm'
m <- glm(Health_Binary ~ D_Age_quant, data = ml_train,family = binomial)
# view a summary of the model
summary(m)  
  ## 
## Call:
## glm(formula = Health_Binary ~ D_Age_quant, family = binomial, 
##     data = ml_train)
## 
## Coefficients:
##                     Estimate Std. Error z value Pr(>|z|)    
## (Intercept)          -3.5920     0.2811 -12.777  < 2e-16 ***
## D_Age_quant(3,5]      0.2658     0.4067   0.654   0.5134    
## D_Age_quant(5,7]      0.3074     0.4275   0.719   0.4722    
## D_Age_quant(7,9.4]    0.7771     0.4007   1.939   0.0525 .  
## D_Age_quant(9.4,20]   1.4624     0.3346   4.371 1.24e-05 ***
## ---
## Signif. codes:  0 '***' 0.001 '**' 0.01 '*' 0.05 '.' 0.1 ' ' 1
## 
## (Dispersion parameter for binomial family taken to be 1)
## 
##     Null deviance: 652.97  on 1657  degrees of freedom
## Residual deviance: 626.72  on 1653  degrees of freedom
## AIC: 636.72
## 
## Number of Fisher Scoring iterations: 6  
  # test model fit
with(m, null.deviance - deviance)  
  ## [1] 26.2418  
  with(m, df.null - df.residual)  
  ## [1] 4  
  with(m, pchisq(null.deviance - deviance, df.null - df.residual, lower.tail = FALSE))  
  ## [1] 2.828313e-05  
  BIC(m)  
  ## [1] 663.7913  
  # Hosmer-Lemeshow Goodness-of-Fit Test
hltest(m, G=4)  
  ## 
##    The Hosmer-Lemeshow goodness-of-fit test
## 
##  Group Size Observed Expected
##      1  485       13       13
##      2  346       12       12
##      3  277       10       10
##      4  230       13       13
##      5  320       34       34
## 
##          Statistic =  0 
## degrees of freedom =  3 
##            p-value =  1  
  ## CIs using profiled log-likelihood
confint(m, level=0.99)  
  ## Waiting for profiling to be done...  
  ##                          0.5 %    99.5 %
## (Intercept)         -4.4102803 -2.941318
## D_Age_quant(3,5]    -0.8107727  1.328297
## D_Age_quant(5,7]    -0.8450635  1.408914
## D_Age_quant(7,9.4]  -0.2753930  1.829761
## D_Age_quant(9.4,20]  0.6389720  2.387871  
  ## CIs using standard errors
confint.default(m, level=0.99)  
  ##                          0.5 %    99.5 %
## (Intercept)         -4.3162034 -2.867856
## D_Age_quant(3,5]    -0.7816780  1.313269
## D_Age_quant(5,7]    -0.7938909  1.408623
## D_Age_quant(7,9.4]  -0.2550908  1.809254
## D_Age_quant(9.4,20]  0.6005569  2.324240  
  # Wald test
wald.test(b = coef(m), Sigma = vcov(m), Terms = 2)  
  ## Wald test:
## ----------
## 
## Chi-squared test:
## X2 = 0.43, df = 1, P(> X2) = 0.51  
  ## odds ratios and 95% CI
exp(cbind(OR = coef(m), confint(m, level=0.99)))  
  ## Waiting for profiling to be done...  
  ##                             OR      0.5 %      99.5 %
## (Intercept)         0.02754237 0.01215177  0.05279607
## D_Age_quant(3,5]    1.30446799 0.44451446  3.77460825
## D_Age_quant(5,7]    1.35983866 0.42953008  4.09150997
## D_Age_quant(7,9.4]  2.17511521 0.75927364  6.23239996
## D_Age_quant(9.4,20] 4.31629909 1.89453224 10.89028182  
 
 
 Calculate Nagelkerke R^2 
  NagelkerkeR2(m)  
  ## $N
## [1] 1658
## 
## $R2
## [1] 0.04823765  
 
 
 check assumptions of model 
 
 Cook’s distance 
  plot(m, which = 4, id.n = 3)  
   
 
 
 Extract model results and display data for top 3 values using Cook’s
distance 
  model.data <- augment(m) %>% 
  mutate(index = 1:n()) 
model.data %>% top_n(3, .cooksd)  
 
 
 
 
 
 plot standardised residuals 
  ggplot(model.data, aes(index, .std.resid)) + 
  geom_point(aes(color = Health_Binary), alpha = .5) +
  theme_bw()  
   
 
 
 Filter potential influential data points with abs(.std.res) >
3: 
  model.data %>% 
  filter(abs(.std.resid) > 3)  
 
 
 
 
 
 
 Create ROCR for training and test data 
  ## training data
pred.mtt = predict(m, type = "response") #repeat risk predictions from model m
rocr.pred.mtt = ROCR::prediction(pred.mtt, labels = ml_train$Health_Binary) #ROCR prediction object
roc.perf.mtt = ROCR::performance(rocr.pred.mtt, measure = "tpr", x.measure = "fpr") # #ROCR performance object
plot(roc.perf.mtt, col = "blue")


pred.te.1 = predict(m, newdata = ml_test, type = "response") #.te = "test"
rocr.pred.te.1 = ROCR::prediction(pred.te.1, labels = ml_test$Health_Binary)
roc.perf.te.1 = ROCR::performance(rocr.pred.te.1, measure = "tpr", x.measure = "fpr")
plot(roc.perf.te.1, col = "red", add = T)

abline(a = 0, b = 1, lty = 2) #diagonal for random assignment
legend("bottomright", legend = c("train","test"),
col = c("blue","red"), lty = c(2,1), lwd =1.5)  
   
 
 
 Report AUC from ROC for training and test data 
    # Train AUC
aucTr <- ROCR::performance(rocr.pred.mtt, measure = "auc")
  aucTr <- aucTr@y.values[[1]]
  print(aucTr)  
  ## [1] 0.6516381  
     # Test AUC
  aucTe <- ROCR::performance(rocr.pred.te.1, measure = "auc")
  aucTe <- aucTe@y.values[[1]]
  print(aucTe)  
  ## [1] 0.6907895  
 
 
 
 D_SEX Binary logistic regression for HEALTH 
  # fit binary logit model and store results 'm'
m <- glm(Health_Binary ~ D_Sex, data = ml_train,family = binomial)
# view a summary of the model
summary(m)  
  ## 
## Call:
## glm(formula = Health_Binary ~ D_Sex, family = binomial, data = ml_train)
## 
## Coefficients:
##             Estimate Std. Error z value Pr(>|z|)    
## (Intercept)  -2.8319     0.1551 -18.255   <2e-16 ***
## D_SexMale    -0.2508     0.2271  -1.104     0.27    
## ---
## Signif. codes:  0 '***' 0.001 '**' 0.01 '*' 0.05 '.' 0.1 ' ' 1
## 
## (Dispersion parameter for binomial family taken to be 1)
## 
##     Null deviance: 652.97  on 1657  degrees of freedom
## Residual deviance: 651.74  on 1656  degrees of freedom
## AIC: 655.74
## 
## Number of Fisher Scoring iterations: 5  
  # test model fit
with(m, null.deviance - deviance)  
  ## [1] 1.222905  
  with(m, df.null - df.residual)  
  ## [1] 1  
  with(m, pchisq(null.deviance - deviance, df.null - df.residual, lower.tail = FALSE))  
  ## [1] 0.2687913  
  BIC(m)  
  ## [1] 666.5701  
  ## CIs using profiled log-likelihood
confint(m, level=0.99)  
  ## Waiting for profiling to be done...  
  ##                  0.5 %    99.5 %
## (Intercept) -3.2569157 -2.454350
## D_SexMale   -0.8432449  0.334089  
  ## CIs using standard errors
confint.default(m, level=0.99)  
  ##                  0.5 %   99.5 %
## (Intercept) -3.2314611 -2.43229
## D_SexMale   -0.8357568  0.33424  
  # Wald test
wald.test(b = coef(m), Sigma = vcov(m), Terms = 2)  
  ## Wald test:
## ----------
## 
## Chi-squared test:
## X2 = 1.2, df = 1, P(> X2) = 0.27  
  ## odds ratios and 95% CI
exp(cbind(OR = coef(m), confint(m, level=0.99)))  
  ## Waiting for profiling to be done...  
  ##                     OR      0.5 %   99.5 %
## (Intercept) 0.05890228 0.03850698 0.085919
## D_SexMale   0.77821036 0.43031193 1.396667  
 
 Calculate Nagelkerke R^2 
  NagelkerkeR2(m)  
  ## $N
## [1] 1658
## 
## $R2
## [1] 0.002264944  
 
 
 check assumptions of model 
 
 Cook’s distance 
  plot(m, which = 4, id.n = 3)  
   
 
 
 Extract model results and display data for top 3 values using Cook’s
distance 
  model.data <- augment(m) %>% 
  mutate(index = 1:n()) 
model.data %>% top_n(3, .cooksd)  
 
 
 
 
 
 plot standardised residuals 
  ggplot(model.data, aes(index, .std.resid)) + 
  geom_point(aes(color = Health_Binary), alpha = .5) +
  theme_bw()  
   
 
 
 Filter potential influential data points with abs(.std.res) >
3: 
  model.data %>% 
  filter(abs(.std.resid) > 3)  
 
 
 
 
 
 
 Create ROCR for training and test data 
  ## training data
pred.mtt = predict(m, type = "response") #repeat risk predictions from model m
rocr.pred.mtt = ROCR::prediction(pred.mtt, labels = ml_train$Health_Binary) #ROCR prediction object
roc.perf.mtt = ROCR::performance(rocr.pred.mtt, measure = "tpr", x.measure = "fpr") # #ROCR performance object
plot(roc.perf.mtt, col = "blue")


pred.te.1 = predict(m, newdata = ml_test, type = "response") #.te = "test"
rocr.pred.te.1 = ROCR::prediction(pred.te.1, labels = ml_test$Health_Binary)
roc.perf.te.1 = ROCR::performance(rocr.pred.te.1, measure = "tpr", x.measure = "fpr")
plot(roc.perf.te.1, col = "red", add = T)

abline(a = 0, b = 1, lty = 2) #diagonal for random assignment
legend("bottomright", legend = c("train","test"),
col = c("blue","red"), lty = c(2,1), lwd =1.5)  
   
 
 
 Report AUC from ROC for training and test data 
    # Train AUC
aucTr <- ROCR::performance(rocr.pred.mtt, measure = "auc")
  aucTr <- aucTr@y.values[[1]]
  print(aucTr)  
  ## [1] 0.5313003  
     # Test AUC
  aucTe <- ROCR::performance(rocr.pred.te.1, measure = "auc")
  aucTe <- aucTe@y.values[[1]]
  print(aucTe)  
  ## [1] 0.4979045  
 
 
 
 DOG NEUTER binary logistic regression ON SIGNIFICANT OR SERIOUS
ILLNESS 
  # fit binary logit model and store results 'm'
m <- glm(Health_Binary ~ D_Neuter, data = ml_train,family = binomial)
# view a summary of the model
summary(m)  
  ## 
## Call:
## glm(formula = Health_Binary ~ D_Neuter, family = binomial, data = ml_train)
## 
## Coefficients:
##                  Estimate Std. Error z value Pr(>|z|)    
## (Intercept)       -3.4854     0.3061 -11.387   <2e-16 ***
## D_NeuterNeutered   0.6440     0.3295   1.954   0.0507 .  
## ---
## Signif. codes:  0 '***' 0.001 '**' 0.01 '*' 0.05 '.' 0.1 ' ' 1
## 
## (Dispersion parameter for binomial family taken to be 1)
## 
##     Null deviance: 652.97  on 1657  degrees of freedom
## Residual deviance: 648.56  on 1656  degrees of freedom
## AIC: 652.56
## 
## Number of Fisher Scoring iterations: 6  
  # test model fit
with(m, null.deviance - deviance)  
  ## [1] 4.401392  
  with(m, df.null - df.residual)  
  ## [1] 1  
  with(m, pchisq(null.deviance - deviance, df.null - df.residual, lower.tail = FALSE))  
  ## [1] 0.0359096  
  BIC(m)  
  ## [1] 663.3916  
  ## CIs using profiled log-likelihood
confint(m, level=0.99)  
  ## Waiting for profiling to be done...  
  ##                       0.5 %    99.5 %
## (Intercept)      -4.3860728 -2.782573
## D_NeuterNeutered -0.1355077  1.590559  
  ## CIs using standard errors
confint.default(m, level=0.99)  
  ##                       0.5 %    99.5 %
## (Intercept)      -4.2738759 -2.696978
## D_NeuterNeutered -0.2048907  1.492817  
  # Wald test
wald.test(b = coef(m), Sigma = vcov(m), Terms = 2)  
  ## Wald test:
## ----------
## 
## Chi-squared test:
## X2 = 3.8, df = 1, P(> X2) = 0.051  
  ## odds ratios and 95% CI
exp(cbind(OR = coef(m), confint(m, level=0.99)))  
  ## Waiting for profiling to be done...  
  ##                          OR      0.5 %     99.5 %
## (Intercept)      0.03064067 0.01244953 0.06187907
## D_NeuterNeutered 1.90401135 0.87327246 4.90648933  
 
 Calculate Nagelkerke R^2 
  NagelkerkeR2(m)  
  ## $N
## [1] 1658
## 
## $R2
## [1] 0.008144016  
 
 
 check assumptions of model 
 
 Cook’s distance 
  plot(m, which = 4, id.n = 3)  
   
 
 
 Extract model results and display data for top 3 values using Cook’s
distance 
  model.data <- augment(m) %>% 
  mutate(index = 1:n()) 
model.data %>% top_n(3, .cooksd)  
 
 
 
 
 
 plot standardised residuals 
  ggplot(model.data, aes(index, .std.resid)) + 
  geom_point(aes(color = Health_Binary), alpha = .5) +
  theme_bw()  
   
 
 
 Filter potential influential data points with abs(.std.res) >
3: 
  model.data %>% 
  filter(abs(.std.resid) > 3)  
 
 
 
 
 
 
 Create ROCR for training and test data 
  ## training data
pred.mtt = predict(m, type = "response") #repeat risk predictions from model m
rocr.pred.mtt = ROCR::prediction(pred.mtt, labels = ml_train$Health_Binary) #ROCR prediction object
roc.perf.mtt = ROCR::performance(rocr.pred.mtt, measure = "tpr", x.measure = "fpr") # #ROCR performance object
plot(roc.perf.mtt, col = "blue")


pred.te.1 = predict(m, newdata = ml_test, type = "response") #.te = "test"
rocr.pred.te.1 = ROCR::prediction(pred.te.1, labels = ml_test$Health_Binary)
roc.perf.te.1 = ROCR::performance(rocr.pred.te.1, measure = "tpr", x.measure = "fpr")
plot(roc.perf.te.1, col = "red", add = T)

abline(a = 0, b = 1, lty = 2) #diagonal for random assignment
legend("bottomright", legend = c("train","test"),
col = c("blue","red"), lty = c(2,1), lwd =1.5)  
   
 
 
 Report AUC from ROC for training and test data 
    # Train AUC
aucTr <- ROCR::performance(rocr.pred.mtt, measure = "auc")
  aucTr <- aucTr@y.values[[1]]
  print(aucTr)  
  ## [1] 0.5468228  
     # Test AUC
  aucTe <- ROCR::performance(rocr.pred.te.1, measure = "auc")
  aucTe <- aucTe@y.values[[1]]
  print(aucTe)  
  ## [1] 0.5456871  
 
 
 
 D_SEX + D_NEUTER Binary logistic regression ON SIGNIFICANT OR
SERIOUS ILLNESS 
  # fit binary logit model and store results 'm'
m <- glm(Health_Binary ~ D_Sex + D_Neuter, data = ml_train,family = binomial)
# view a summary of the model
summary(m)  
  ## 
## Call:
## glm(formula = Health_Binary ~ D_Sex + D_Neuter, family = binomial, 
##     data = ml_train)
## 
## Coefficients:
##                  Estimate Std. Error z value Pr(>|z|)    
## (Intercept)       -3.3613     0.3295 -10.200   <2e-16 ***
## D_SexMale         -0.2213     0.2277  -0.972   0.3313    
## D_NeuterNeutered   0.6261     0.3301   1.897   0.0579 .  
## ---
## Signif. codes:  0 '***' 0.001 '**' 0.01 '*' 0.05 '.' 0.1 ' ' 1
## 
## (Dispersion parameter for binomial family taken to be 1)
## 
##     Null deviance: 652.97  on 1657  degrees of freedom
## Residual deviance: 647.62  on 1655  degrees of freedom
## AIC: 653.62
## 
## Number of Fisher Scoring iterations: 6  
  # test model fit
with(m, null.deviance - deviance)  
  ## [1] 5.348202  
  with(m, df.null - df.residual)  
  ## [1] 2  
  with(m, pchisq(null.deviance - deviance, df.null - df.residual, lower.tail = FALSE))  
  ## [1] 0.06896881  
  BIC(m)  
  ## [1] 669.8581  
  # Hosmer-Lemeshow Goodness-of-Fit Test
hltest(m)  
  ## 
##    The Hosmer-Lemeshow goodness-of-fit test
## 
##  Group Size Observed  Expected
##      1  217        8  5.870365
##      2  153        3  5.129635
##      3  650       30 32.129635
##      4  638       41 38.870365
## 
##          Statistic =  1.98163 
## degrees of freedom =  2 
##            p-value =  0.37127  
  ## CIs using profiled log-likelihood
confint(m, level=0.99)  
  ## Waiting for profiling to be done...  
  ##                       0.5 %    99.5 %
## (Intercept)      -4.3126242 -2.591127
## D_SexMale        -0.8151832  0.365191
## D_NeuterNeutered -0.1551920  1.573786  
  ## CIs using standard errors
confint.default(m, level=0.99)  
  ##                       0.5 %     99.5 %
## (Intercept)      -4.2101122 -2.5124908
## D_SexMale        -0.8078463  0.3653383
## D_NeuterNeutered -0.2242529  1.4763642  
  # Wald test
wald.test(b = coef(m), Sigma = vcov(m), Terms = 2)  
  ## Wald test:
## ----------
## 
## Chi-squared test:
## X2 = 0.94, df = 1, P(> X2) = 0.33  
  ## odds ratios and 95% CI
exp(cbind(OR = coef(m), confint(m, level=0.99)))  
  ## Waiting for profiling to be done...  
  ##                          OR      0.5 %     99.5 %
## (Intercept)      0.03469008 0.01339834 0.07493552
## D_SexMale        0.80151307 0.44255825 1.44078915
## D_NeuterNeutered 1.87021919 0.85625075 4.82488229  
 
 Calculate Nagelkerke R^2 
  NagelkerkeR2(m)  
  ## $N
## [1] 1658
## 
## $R2
## [1] 0.009893099  
 
 
 check assumptions of model 
 
 Cook’s distance 
  plot(m, which = 4, id.n = 3)  
   
 
 
 Extract model results and display data for top 3 values using Cook’s
distance 
  model.data <- augment(m) %>% 
  mutate(index = 1:n()) 
model.data %>% top_n(3, .cooksd)  
 
 
 
 
 
 plot standardised residuals 
  ggplot(model.data, aes(index, .std.resid)) + 
  geom_point(aes(color = Health_Binary), alpha = .5) +
  theme_bw()  
   
 
 
 Filter potential influential data points with abs(.std.res) >
3: 
  model.data %>% 
  filter(abs(.std.resid) > 3)  
 
 
 
 
 
 check for multicollinearity 
  car::vif(m)  
  ##    D_Sex D_Neuter 
## 1.003039 1.003039  
 
 
 
 Create ROCR for training and test data 
  ## training data
pred.mtt = predict(m, type = "response") #repeat risk predictions from model m
rocr.pred.mtt = ROCR::prediction(pred.mtt, labels = ml_train$Health_Binary) #ROCR prediction object
roc.perf.mtt = ROCR::performance(rocr.pred.mtt, measure = "tpr", x.measure = "fpr") # #ROCR performance object
plot(roc.perf.mtt, col = "blue")


pred.te.1 = predict(m, newdata = ml_test, type = "response") #.te = "test"
rocr.pred.te.1 = ROCR::prediction(pred.te.1, labels = ml_test$Health_Binary)
roc.perf.te.1 = ROCR::performance(rocr.pred.te.1, measure = "tpr", x.measure = "fpr")
plot(roc.perf.te.1, col = "red", add = T)

abline(a = 0, b = 1, lty = 2) #diagonal for random assignment
legend("bottomright", legend = c("train","test"),
col = c("blue","red"), lty = c(2,1), lwd =1.5)  
   
 
 
 Report AUC from ROC for training and test data 
    # Train AUC
aucTr <- ROCR::performance(rocr.pred.mtt, measure = "auc")
  aucTr <- aucTr@y.values[[1]]
  print(aucTr)  
  ## [1] 0.5736621  
     # Test AUC
  aucTe <- ROCR::performance(rocr.pred.te.1, measure = "auc")
  aucTe <- aucTe@y.values[[1]]
  print(aucTe)  
  ## [1] 0.5439815  
 
 
 
 D_SEX * D_NEUTER Binary logistic regression ON SIGNIFICANT OR
SERIOUS ILLNESS 
  # fit binary logit model and store results 'm'
m <- glm(Health_Binary ~ D_Sex*D_Neuter, data = ml_train,family = binomial)
# view a summary of the model
summary(m)  
  ## 
## Call:
## glm(formula = Health_Binary ~ D_Sex * D_Neuter, family = binomial, 
##     data = ml_train)
## 
## Coefficients:
##                            Estimate Std. Error z value Pr(>|z|)    
## (Intercept)                 -3.9120     0.5831  -6.710 1.95e-11 ***
## D_SexMale                    0.6491     0.6854   0.947   0.3436    
## D_NeuterNeutered             1.2337     0.6050   2.039   0.0414 *  
## D_SexMale:D_NeuterNeutered  -0.9993     0.7285  -1.372   0.1702    
## ---
## Signif. codes:  0 '***' 0.001 '**' 0.01 '*' 0.05 '.' 0.1 ' ' 1
## 
## (Dispersion parameter for binomial family taken to be 1)
## 
##     Null deviance: 652.97  on 1657  degrees of freedom
## Residual deviance: 645.56  on 1654  degrees of freedom
## AIC: 653.56
## 
## Number of Fisher Scoring iterations: 6  
  # test model fit
with(m, null.deviance - deviance)  
  ## [1] 7.408042  
  with(m, df.null - df.residual)  
  ## [1] 3  
  with(m, pchisq(null.deviance - deviance, df.null - df.residual, lower.tail = FALSE))  
  ## [1] 0.05996892  
  BIC(m)  
  ## [1] 675.2117  
  # Hosmer-Lemeshow Goodness-of-Fit Test
hltest(m)  
  ## 
##    The Hosmer-Lemeshow goodness-of-fit test
## 
##  Group Size Observed Expected
##      1  153        3        3
##      2  217        8        8
##      3  650       30       30
##      4  638       41       41
## 
##          Statistic =  0 
## degrees of freedom =  2 
##            p-value =  1  
  ## CIs using profiled log-likelihood
confint(m, level=0.99)  
  ## Waiting for profiling to be done...  
  ##                                  0.5 %     99.5 %
## (Intercept)                -5.88666871 -2.6956573
## D_SexMale                  -0.99195875  2.7741801
## D_NeuterNeutered           -0.06905063  3.2410769
## D_SexMale:D_NeuterNeutered -3.20142294  0.7624031  
  ## CIs using standard errors
confint.default(m, level=0.99)  
  ##                                 0.5 %     99.5 %
## (Intercept)                -5.4138711 -2.4101749
## D_SexMale                  -1.1162748  2.4145354
## D_NeuterNeutered           -0.3246828  2.7920387
## D_SexMale:D_NeuterNeutered -2.8758628  0.8772481  
  # Wald test
wald.test(b = coef(m), Sigma = vcov(m), Terms = 2)  
  ## Wald test:
## ----------
## 
## Chi-squared test:
## X2 = 0.9, df = 1, P(> X2) = 0.34  
  ## odds ratios and 95% CI
exp(cbind(OR = coef(m), confint(m, level=0.99)))  
  ## Waiting for profiling to be done...  
  ##                                   OR      0.5 %    99.5 %
## (Intercept)                0.0200000 0.00277621  0.067498
## D_SexMale                  1.9138756 0.37084958 16.025482
## D_NeuterNeutered           3.4338358 0.93327943 25.561233
## D_SexMale:D_NeuterNeutered 0.3681343 0.04070424  2.143421  
 
 Calculate Nagelkerke R^2 
  NagelkerkeR2(m)  
  ## $N
## [1] 1658
## 
## $R2
## [1] 0.01369489  
 
 
 check assumptions of model 
 
 Cook’s distance 
  plot(m, which = 4, id.n = 3)  
   
 
 
 Extract model results and display data for top 3 values using Cook’s
distance 
  model.data <- augment(m) %>% 
  mutate(index = 1:n()) 
model.data %>% top_n(3, .cooksd)  
 
 
 
 
 
 
 
 
 plot standardised residuals 
  ggplot(model.data, aes(index, .std.resid)) + 
  geom_point(aes(color = Health_Binary), alpha = .5) +
  theme_bw()  
   
 
 
 Filter potential influential data points with abs(.std.res) >
3: 
  model.data %>% 
  filter(abs(.std.resid) > 3)  
 
 
 
 
 check for multicollinearity 
  car::vif(m)  
  ## there are higher-order terms (interactions) in this model
## consider setting type = 'predictor'; see ?vif  
  ##          D_Sex       D_Neuter D_Sex:D_Neuter 
##       9.078481       3.362405       9.589043  
 
 
 Create ROCR for training and test data 
  ## training data
pred.mtt = predict(m, type = "response") #repeat risk predictions from model m
rocr.pred.mtt = ROCR::prediction(pred.mtt, labels = ml_train$Health_Binary) #ROCR prediction object
roc.perf.mtt = ROCR::performance(rocr.pred.mtt, measure = "tpr", x.measure = "fpr") # #ROCR performance object
plot(roc.perf.mtt, col = "blue")


pred.te.1 = predict(m, newdata = ml_test, type = "response") #.te = "test"
rocr.pred.te.1 = ROCR::prediction(pred.te.1, labels = ml_test$Health_Binary)
roc.perf.te.1 = ROCR::performance(rocr.pred.te.1, measure = "tpr", x.measure = "fpr")
plot(roc.perf.te.1, col = "red", add = T)

abline(a = 0, b = 1, lty = 2) #diagonal for random assignment
legend("bottomright", legend = c("train","test"),
col = c("blue","red"), lty = c(2,1), lwd =1.5)  
   
 
 
 Report AUC from ROC for training and test data 
    # Train AUC
aucTr <- ROCR::performance(rocr.pred.mtt, measure = "auc")
  aucTr <- aucTr@y.values[[1]]
  print(aucTr)  
  ## [1] 0.578096  
     # Test AUC
  aucTe <- ROCR::performance(rocr.pred.te.1, measure = "auc")
  aucTe <- aucTe@y.values[[1]]
  print(aucTe)  
  ## [1] 0.5478314  
 
 
 
 CHECK EFFECT OF DOG HEALTH CHARACTERISTICS ON SIGNIFICANT OR SERIOUS
ILLNESS - simple binary regression 
 
 THERAPEUTIC FOOD Binary logistic regression ON SIGNIFICANT OR
SERIOUS ILLNESS 
  # fit binary logit model and store results 'm'
m <- glm(Health_Binary ~ Therapeutic_Food, data = ml_train,family = binomial)
# view a summary of the model
summary(m)  
  ## 
## Call:
## glm(formula = Health_Binary ~ Therapeutic_Food, family = binomial, 
##     data = ml_train)
## 
## Coefficients:
##                     Estimate Std. Error z value Pr(>|z|)    
## (Intercept)          -3.1362     0.1257 -24.942  < 2e-16 ***
## Therapeutic_FoodYes   1.8657     0.3096   6.026 1.68e-09 ***
## ---
## Signif. codes:  0 '***' 0.001 '**' 0.01 '*' 0.05 '.' 0.1 ' ' 1
## 
## (Dispersion parameter for binomial family taken to be 1)
## 
##     Null deviance: 652.97  on 1657  degrees of freedom
## Residual deviance: 625.58  on 1656  degrees of freedom
## AIC: 629.58
## 
## Number of Fisher Scoring iterations: 6  
  # test model fit
with(m, null.deviance - deviance)  
  ## [1] 27.39065  
  with(m, df.null - df.residual)  
  ## [1] 1  
  with(m, pchisq(null.deviance - deviance, df.null - df.residual, lower.tail = FALSE))  
  ## [1] 1.662326e-07  
  BIC(m)  
  ## [1] 640.4023  
  ## CIs using profiled log-likelihood
confint(m, level=0.99)  
  ## Waiting for profiling to be done...  
  ##                         0.5 %    99.5 %
## (Intercept)         -3.477014 -2.827434
## Therapeutic_FoodYes  1.020073  2.631890  
  ## CIs using standard errors
confint.default(m, level=0.99)  
  ##                         0.5 %    99.5 %
## (Intercept)         -3.460030 -2.812275
## Therapeutic_FoodYes  1.068207  2.663173  
  # Wald test
wald.test(b = coef(m), Sigma = vcov(m), Terms = 2)  
  ## Wald test:
## ----------
## 
## Chi-squared test:
## X2 = 36.3, df = 1, P(> X2) = 1.7e-09  
  ## odds ratios and 95% CI
exp(cbind(OR = coef(m), confint(m, level=0.99)))  
  ## Waiting for profiling to be done...  
  ##                             OR      0.5 %      99.5 %
## (Intercept)         0.04344964 0.03089954  0.05916446
## Therapeutic_FoodYes 6.46039341 2.77339713 13.90001911  
 
 Calculate Nagelkerke R^2 
  NagelkerkeR2(m)  
  ## $N
## [1] 1658
## 
## $R2
## [1] 0.05033208  
 
 
 check assumptions of model 
 
 Cook’s distance 
  plot(m, which = 4, id.n = 3)  
   
 
 
 Extract model results and display data for top 3 values using Cook’s
distance 
  model.data <- augment(m) %>% 
  mutate(index = 1:n()) 
model.data %>% top_n(3, .cooksd)  
 
 
 
 
 
 plot standardised residuals 
  ggplot(model.data, aes(index, .std.resid)) + 
  geom_point(aes(color = Health_Binary), alpha = .5) +
  theme_bw()  
   
 
 
 Filter potential influential data points with abs(.std.res) >
3: 
  model.data %>% 
  filter(abs(.std.resid) > 3)  
 
 
 
 
 
 
 Create ROCR for training and test data 
  ## training data
pred.mtt = predict(m, type = "response") #repeat risk predictions from model m
rocr.pred.mtt = ROCR::prediction(pred.mtt, labels = ml_train$Health_Binary) #ROCR prediction object
roc.perf.mtt = ROCR::performance(rocr.pred.mtt, measure = "tpr", x.measure = "fpr") # #ROCR performance object
plot(roc.perf.mtt, col = "blue")


pred.te.1 = predict(m, newdata = ml_test, type = "response") #.te = "test"
rocr.pred.te.1 = ROCR::prediction(pred.te.1, labels = ml_test$Health_Binary)
roc.perf.te.1 = ROCR::performance(rocr.pred.te.1, measure = "tpr", x.measure = "fpr")
plot(roc.perf.te.1, col = "red", add = T)

abline(a = 0, b = 1, lty = 2) #diagonal for random assignment
legend("bottomright", legend = c("train","test"),
col = c("blue","red"), lty = c(2,1), lwd =1.5)  
   
 
 
 Report AUC from ROC for training and test data 
    # Train AUC
aucTr <- ROCR::performance(rocr.pred.mtt, measure = "auc")
  aucTr <- aucTr@y.values[[1]]
  print(aucTr)  
  ## [1] 0.5794772  
     # Test AUC
  aucTe <- ROCR::performance(rocr.pred.te.1, measure = "auc")
  aucTe <- aucTe@y.values[[1]]
  print(aucTe)  
  ## [1] 0.5545322  
 
 
 
 VISITS Binary logistic regression ON SIGNIFICANT OR SERIOUS
ILLNESS 
  # fit binary logit model and store results 'm'
m <- glm(Health_Binary ~ Visits, data = ml_train,family = binomial)
# view a summary of the model
summary(m)  
  ## 
## Call:
## glm(formula = Health_Binary ~ Visits, family = binomial, data = ml_train)
## 
## Coefficients:
##             Estimate Std. Error z value Pr(>|z|)    
## (Intercept)  -4.5951     0.5803  -7.919 2.39e-15 ***
## Visits1       0.3062     0.6703   0.457   0.6478    
## Visits2       1.2993     0.6453   2.013   0.0441 *  
## Visits3       1.6812     0.6981   2.408   0.0160 *  
## Visits3<      3.5443     0.6031   5.877 4.17e-09 ***
## ---
## Signif. codes:  0 '***' 0.001 '**' 0.01 '*' 0.05 '.' 0.1 ' ' 1
## 
## (Dispersion parameter for binomial family taken to be 1)
## 
##     Null deviance: 652.97  on 1657  degrees of freedom
## Residual deviance: 517.08  on 1653  degrees of freedom
## AIC: 527.08
## 
## Number of Fisher Scoring iterations: 7  
  # test model fit
with(m, null.deviance - deviance)  
  ## [1] 135.8838  
  with(m, df.null - df.residual)  
  ## [1] 4  
  with(m, pchisq(null.deviance - deviance, df.null - df.residual, lower.tail = FALSE))  
  ## [1] 2.146291e-28  
  BIC(m)  
  ## [1] 554.1493  
  # Hosmer-Lemeshow Goodness-of-Fit Test
hltest(m)  
  ## 
##    The Hosmer-Lemeshow goodness-of-fit test
## 
##  Group Size Observed Expected
##      1  300        3        3
##      2  665        9        9
##      3  364       13       13
##      4  136        7        7
##      5  193       50       50
## 
##          Statistic =  0 
## degrees of freedom =  3 
##            p-value =  1  
  ## CIs using profiled log-likelihood
confint(m, level=0.99)  
  ## Waiting for profiling to be done...  
  ##                   0.5 %    99.5 %
## (Intercept) -6.56553887 -3.390091
## Visits1     -1.27532223  2.408526
## Visits2     -0.17196310  3.365922
## Visits3     -0.01716993  3.824355
## Visits3<     2.25048111  5.548973  
  ## CIs using standard errors
confint.default(m, level=0.99)  
  ##                  0.5 %    99.5 %
## (Intercept) -6.0897555 -3.100484
## Visits1     -1.4204489  2.032816
## Visits2     -0.3630092  2.961575
## Visits3     -0.1168957  3.479331
## Visits3<     1.9909047  5.097692  
  # Wald test
wald.test(b = coef(m), Sigma = vcov(m), Terms = 2)  
  ## Wald test:
## ----------
## 
## Chi-squared test:
## X2 = 0.21, df = 1, P(> X2) = 0.65  
  ## odds ratios and 95% CI
exp(cbind(OR = coef(m), confint(m, level=0.99)))  
  ## Waiting for profiling to be done...  
  ##                      OR       0.5 %      99.5 %
## (Intercept)  0.01010101 0.001408065   0.0337056
## Visits1      1.35823171 0.279340942  11.1175635
## Visits2      3.66666667 0.842010242  28.9601957
## Visits3      5.37209302 0.982976636  45.8032268
## Visits3<    34.61538461 9.492301619 256.9735519  
 
 Calculate Nagelkerke R^2 
  NagelkerkeR2(m)  
  ## $N
## [1] 1658
## 
## $R2
## [1] 0.2417227  
 
 
 check assumptions of model 
 
 Cook’s distance 
  plot(m, which = 4, id.n = 3)  
   
 
 
 Extract model results and display data for top 3 values using Cook’s
distance 
  model.data <- augment(m) %>% 
  mutate(index = 1:n()) 
model.data %>% top_n(3, .cooksd)  
 
 
 
 
 
 plot standardised residuals 
  ggplot(model.data, aes(index, .std.resid)) + 
  geom_point(aes(color = Health_Binary), alpha = .5) +
  theme_bw()  
   
 
 
 Filter potential influential data points with abs(.std.res) >
3: 
  model.data %>% 
  filter(abs(.std.resid) > 3)  
 
 
 
 
 
 
 Create ROCR for training and test data 
  ## training data
pred.mtt = predict(m, type = "response") #repeat risk predictions from model m
rocr.pred.mtt = ROCR::prediction(pred.mtt, labels = ml_train$Health_Binary) #ROCR prediction object
roc.perf.mtt = ROCR::performance(rocr.pred.mtt, measure = "tpr", x.measure = "fpr") # #ROCR performance object
plot(roc.perf.mtt, col = "blue")


pred.te.1 = predict(m, newdata = ml_test, type = "response") #.te = "test"
rocr.pred.te.1 = ROCR::prediction(pred.te.1, labels = ml_test$Health_Binary)
roc.perf.te.1 = ROCR::performance(rocr.pred.te.1, measure = "tpr", x.measure = "fpr")
plot(roc.perf.te.1, col = "red", add = T)

abline(a = 0, b = 1, lty = 2) #diagonal for random assignment
legend("bottomright", legend = c("train","test"),
col = c("blue","red"), lty = c(2,1), lwd =1.5)  
   
 
 
 Report AUC from ROC for training and test data 
    # Train AUC
aucTr <- ROCR::performance(rocr.pred.mtt, measure = "auc")
  aucTr <- aucTr@y.values[[1]]
  print(aucTr)  
  ## [1] 0.8167134  
     # Test AUC
  aucTe <- ROCR::performance(rocr.pred.te.1, measure = "auc")
  aucTe <- aucTe@y.values[[1]]
  print(aucTe)  
  ## [1] 0.8413986  
 
 
 
 VISITS2 Binary logistic regression for HEALTH 
 
 Note Visits better fit than Visits2 
  # fit binary logit model and store results 'm'
m <- glm(Health_Binary ~ Visits2, data = ml_train,family = binomial)
# view a summary of the model
summary(m)  
  ## 
## Call:
## glm(formula = Health_Binary ~ Visits2, family = binomial, data = ml_train)
## 
## Coefficients:
##             Estimate Std. Error z value Pr(>|z|)    
## (Intercept)  -4.5951     0.5803  -7.919 2.39e-15 ***
## Visits21v     0.3062     0.6703   0.457   0.6478    
## Visits22v     1.2993     0.6453   2.013   0.0441 *  
## Visits23v     3.0324     0.5983   5.069 4.01e-07 ***
## ---
## Signif. codes:  0 '***' 0.001 '**' 0.01 '*' 0.05 '.' 0.1 ' ' 1
## 
## (Dispersion parameter for binomial family taken to be 1)
## 
##     Null deviance: 652.97  on 1657  degrees of freedom
## Residual deviance: 544.43  on 1654  degrees of freedom
## AIC: 552.43
## 
## Number of Fisher Scoring iterations: 7  
  # test model fit
with(m, null.deviance - deviance)  
  ## [1] 108.5323  
  with(m, df.null - df.residual)  
  ## [1] 3  
  with(m, pchisq(null.deviance - deviance, df.null - df.residual, lower.tail = FALSE))  
  ## [1] 2.270778e-23  
  BIC(m)  
  ## [1] 574.0874  
  # Hosmer-Lemeshow Goodness-of-Fit Test
hltest(m)  
  ## 
##    The Hosmer-Lemeshow goodness-of-fit test
## 
##  Group Size Observed Expected
##      1  300        3        3
##      2  665        9        9
##      3  364       13       13
##      4  329       57       57
## 
##          Statistic =  0 
## degrees of freedom =  2 
##            p-value =  1  
  ## CIs using profiled log-likelihood
confint(m, level=0.99)  
  ## Waiting for profiling to be done...  
  ##                  0.5 %    99.5 %
## (Intercept) -6.5655389 -3.390091
## Visits21v   -1.2753222  2.408526
## Visits22v   -0.1719631  3.365922
## Visits23v    1.7565935  5.029744  
  ## CIs using standard errors
confint.default(m, level=0.99)  
  ##                  0.5 %    99.5 %
## (Intercept) -6.0897555 -3.100484
## Visits21v   -1.4204489  2.032816
## Visits22v   -0.3630092  2.961575
## Visits23v    1.4913530  4.573385  
  # Wald test
wald.test(b = coef(m), Sigma = vcov(m), Terms = 2)  
  ## Wald test:
## ----------
## 
## Chi-squared test:
## X2 = 0.21, df = 1, P(> X2) = 0.65  
  ## odds ratios and 95% CI
exp(cbind(OR = coef(m), confint(m, level=0.99)))  
  ## Waiting for profiling to be done...  
  ##                      OR       0.5 %      99.5 %
## (Intercept)  0.01010101 0.001408065   0.0337056
## Visits21v    1.35823171 0.279340942  11.1175635
## Visits22v    3.66666667 0.842010242  28.9601957
## Visits23v   20.74632353 5.792671298 152.8938750  
 
 
 Calculate Nagelkerke R^2 
  NagelkerkeR2(m)  
  ## $N
## [1] 1658
## 
## $R2
## [1] 0.1946467  
 
 
 check assumptions of model 
 
 Cook’s distance 
  plot(m, which = 4, id.n = 3)  
   
 
 
 Extract model results and display data for top 3 values using Cook’s
distance 
  model.data <- augment(m) %>% 
  mutate(index = 1:n()) 
model.data %>% top_n(3, .cooksd)  
 
 
 
 
 
 plot standardised residuals 
  ggplot(model.data, aes(index, .std.resid)) + 
  geom_point(aes(color = Health_Binary), alpha = .5) +
  theme_bw()  
   
 
 
 Filter potential influential data points with abs(.std.res) >
3: 
  model.data %>% 
  filter(abs(.std.resid) > 3)  
 
 
 
 
 
 
 Create ROCR for training and test data 
  ## training data
pred.mtt = predict(m, type = "response") #repeat risk predictions from model m
rocr.pred.mtt = ROCR::prediction(pred.mtt, labels = ml_train$Health_Binary) #ROCR prediction object
roc.perf.mtt = ROCR::performance(rocr.pred.mtt, measure = "tpr", x.measure = "fpr") # #ROCR performance object
plot(roc.perf.mtt, col = "blue")


pred.te.1 = predict(m, newdata = ml_test, type = "response") #.te = "test"
rocr.pred.te.1 = ROCR::prediction(pred.te.1, labels = ml_test$Health_Binary)
roc.perf.te.1 = ROCR::performance(rocr.pred.te.1, measure = "tpr", x.measure = "fpr")
plot(roc.perf.te.1, col = "red", add = T)

abline(a = 0, b = 1, lty = 2) #diagonal for random assignment
legend("bottomright", legend = c("train","test"),
col = c("blue","red"), lty = c(2,1), lwd =1.5)  
   
 
 
 Report AUC from ROC for training and test data 
    # Train AUC
aucTr <- ROCR::performance(rocr.pred.mtt, measure = "auc")
  aucTr <- aucTr@y.values[[1]]
  print(aucTr)  
  ## [1] 0.7956311  
     # Test AUC
  aucTe <- ROCR::performance(rocr.pred.te.1, measure = "auc")
  aucTe <- aucTe@y.values[[1]]
  print(aucTe)  
  ## [1] 0.8253655  
 
 
 
 MEDS Binary logistic regression for HEALTH 
  # fit binary logit model and store results 'm'
m <- glm(Health_Binary ~ Meds, data = ml_train,family = binomial)
# view a summary of the model
summary(m)  
  ## 
## Call:
## glm(formula = Health_Binary ~ Meds, family = binomial, data = ml_train)
## 
## Coefficients:
##             Estimate Std. Error z value Pr(>|z|)    
## (Intercept)  -5.1150     0.4094 -12.495  < 2e-16 ***
## MedsYes       3.0879     0.4272   7.229 4.87e-13 ***
## ---
## Signif. codes:  0 '***' 0.001 '**' 0.01 '*' 0.05 '.' 0.1 ' ' 1
## 
## (Dispersion parameter for binomial family taken to be 1)
## 
##     Null deviance: 652.97  on 1657  degrees of freedom
## Residual deviance: 543.13  on 1656  degrees of freedom
## AIC: 547.13
## 
## Number of Fisher Scoring iterations: 7  
  # test model fit
with(m, null.deviance - deviance)  
  ## [1] 109.832  
  with(m, df.null - df.residual)  
  ## [1] 1  
  with(m, pchisq(null.deviance - deviance, df.null - df.residual, lower.tail = FALSE))  
  ## [1] 1.066554e-25  
  BIC(m)  
  ## [1] 557.9609  
  # Hosmer-Lemeshow Goodness-of-Fit Test

## CIs using profiled log-likelihood
confint(m, level=0.99)  
  ## Waiting for profiling to be done...  
  ##                 0.5 %    99.5 %
## (Intercept) -6.390137 -4.215499
## MedsYes      2.125183  4.394468  
  ## CIs using standard errors
confint.default(m, level=0.99)  
  ##                 0.5 %    99.5 %
## (Intercept) -6.169413 -4.060577
## MedsYes      1.987614  4.188158  
  # Wald test
wald.test(b = coef(m), Sigma = vcov(m), Terms = 2)  
  ## Wald test:
## ----------
## 
## Chi-squared test:
## X2 = 52.3, df = 1, P(> X2) = 4.9e-13  
  ## odds ratios and 95% CI
exp(cbind(OR = coef(m), confint(m, level=0.99)))  
  ## Waiting for profiling to be done...  
  ##                       OR       0.5 %      99.5 %
## (Intercept)  0.006006007 0.001678026  0.01476496
## MedsYes     21.930672003 8.374428522 81.00155615  
 
 Calculate Nagelkerke R^2 
  NagelkerkeR2(m)  
  ## $N
## [1] 1658
## 
## $R2
## [1] 0.1969013  
 
 
 check assumptions of model 
 
 Cook’s distance 
  plot(m, which = 4, id.n = 3)  
   
 
 
 Extract model results and display data for top 3 values using Cook’s
distance 
  model.data <- augment(m) %>% 
  mutate(index = 1:n()) 
model.data %>% top_n(3, .cooksd)  
 
 
 
 
 
 plot standardised residuals 
  ggplot(model.data, aes(index, .std.resid)) + 
  geom_point(aes(color = Health_Binary), alpha = .5) +
  theme_bw()  
   
 
 
 Filter potential influential data points with abs(.std.res) >
3: 
  model.data %>% 
  filter(abs(.std.resid) > 3)  
 
 
 
 
 
 
 Create ROCR for training and test data 
  ## training data
pred.mtt = predict(m, type = "response") #repeat risk predictions from model m
rocr.pred.mtt = ROCR::prediction(pred.mtt, labels = ml_train$Health_Binary) #ROCR prediction object
roc.perf.mtt = ROCR::performance(rocr.pred.mtt, measure = "tpr", x.measure = "fpr") # #ROCR performance object
plot(roc.perf.mtt, col = "blue")


pred.te.1 = predict(m, newdata = ml_test, type = "response") #.te = "test"
rocr.pred.te.1 = ROCR::prediction(pred.te.1, labels = ml_test$Health_Binary)
roc.perf.te.1 = ROCR::performance(rocr.pred.te.1, measure = "tpr", x.measure = "fpr")
plot(roc.perf.te.1, col = "red", add = T)

abline(a = 0, b = 1, lty = 2) #diagonal for random assignment
legend("bottomright", legend = c("train","test"),
col = c("blue","red"), lty = c(2,1), lwd =1.5)  
   
 
 
 Report AUC from ROC for training and test data 
    # Train AUC
aucTr <- ROCR::performance(rocr.pred.mtt, measure = "auc")
  aucTr <- aucTr@y.values[[1]]
  print(aucTr)  
  ## [1] 0.7803563  
     # Test AUC
  aucTe <- ROCR::performance(rocr.pred.te.1, measure = "auc")
  aucTe <- aucTe@y.values[[1]]
  print(aucTe)  
  ## [1] 0.7773148  
 
 
 
 
 MULTIPLE REGRESSION WITH BACKWARDS ELIMINATION 
 
 ROUND 1: Model with all variables 
  # fit binary logit model and store results 'm'
m <- glm(Health_Binary ~  Location + Urban + Education_S2 + Animal_Career_BINARY 
         + Income2 + C_Age2 + C_Gender + C_Diet + Size2 + D_Sex + D_Neuter  + D_Diet + Therapeutic_Food + Visits 
         + Meds + bs(D_Age, degree=1,df=2), data = ml_train,family = binomial)
# view a summary of the model
summary(m)  
  ## 
## Call:
## glm(formula = Health_Binary ~ Location + Urban + Education_S2 + 
##     Animal_Career_BINARY + Income2 + C_Age2 + C_Gender + C_Diet + 
##     Size2 + D_Sex + D_Neuter + D_Diet + Therapeutic_Food + Visits + 
##     Meds + bs(D_Age, degree = 1, df = 2), family = binomial, 
##     data = ml_train)
## 
## Coefficients:
##                                                          Estimate Std. Error
## (Intercept)                                              -5.33246    0.98360
## LocationOther European                                    0.94490    0.40914
## LocationNorth America                                    -0.03360    0.60117
## LocationAustralia/New Zealand/Oceania                     1.00775    0.56282
## LocationOther                                             0.72141    0.70478
## UrbanYes                                                 -0.24052    0.30076
## Education_S21_College                                    -0.49225    0.43900
## Education_S22_Grad                                        0.06811    0.42218
## Education_S23_PG_or_PhD                                  -0.44011    0.43431
## Animal_Career_BINARYYes                                   0.34877    0.32608
## Income2Medium                                            -0.12141    0.38426
## Income2High                                              -0.41880    0.51718
## C_Age230–39                                               0.38698    0.43514
## C_Age240–49                                              -0.40482    0.46666
## C_Age250–59                                               0.03374    0.46156
## C_Age260<                                                -0.67130    0.52631
## C_GenderMale                                             -0.16135    0.63670
## C_DietOmnivore reducing animal product consumption        0.16742    0.36106
## C_DietPescatarian (including fish but no other meats)    -1.08154    0.70150
## C_DietVegan (consuming no animal products)                0.12674    0.46145
## C_DietVegetarian (consuming plants, eggs and milk)        0.29415    0.44898
## Size2Toy                                                -14.82257  889.14711
## Size2Small                                               -0.42494    0.42316
## Size2Large                                                0.52606    0.31674
## Size2Giant                                                2.08918    0.57875
## D_SexMale                                                -0.40055    0.27703
## D_NeuterNeutered                                         -0.29872    0.43811
## D_DietMeat-based – raw                                    0.01882    0.33317
## D_DietVegan (consuming no animal products)               -0.76450    0.58121
## D_DietVegetarian (including eggs or milk, but not meat)  -0.49987    0.99053
## Therapeutic_FoodYes                                       0.79183    0.39210
## Visits1                                                  -0.89817    0.74999
## Visits2                                                  -0.54776    0.74813
## Visits3                                                  -0.28595    0.81102
## Visits3<                                                  1.48046    0.72569
## MedsYes                                                   2.53597    0.52101
## bs(D_Age, degree = 1, df = 2)1                            0.37991    0.57380
## bs(D_Age, degree = 1, df = 2)2                            3.19875    0.70753
##                                                         z value Pr(>|z|)    
## (Intercept)                                              -5.421 5.91e-08 ***
## LocationOther European                                    2.309 0.020918 *  
## LocationNorth America                                    -0.056 0.955433    
## LocationAustralia/New Zealand/Oceania                     1.791 0.073367 .  
## LocationOther                                             1.024 0.306021    
## UrbanYes                                                 -0.800 0.423887    
## Education_S21_College                                    -1.121 0.262162    
## Education_S22_Grad                                        0.161 0.871841    
## Education_S23_PG_or_PhD                                  -1.013 0.310881    
## Animal_Career_BINARYYes                                   1.070 0.284805    
## Income2Medium                                            -0.316 0.752039    
## Income2High                                              -0.810 0.418073    
## C_Age230–39                                               0.889 0.373835    
## C_Age240–49                                              -0.867 0.385672    
## C_Age250–59                                               0.073 0.941735    
## C_Age260<                                                -1.275 0.202138    
## C_GenderMale                                             -0.253 0.799943    
## C_DietOmnivore reducing animal product consumption        0.464 0.642866    
## C_DietPescatarian (including fish but no other meats)    -1.542 0.123135    
## C_DietVegan (consuming no animal products)                0.275 0.783587    
## C_DietVegetarian (consuming plants, eggs and milk)        0.655 0.512363    
## Size2Toy                                                 -0.017 0.986699    
## Size2Small                                               -1.004 0.315280    
## Size2Large                                                1.661 0.096739 .  
## Size2Giant                                                3.610 0.000306 ***
## D_SexMale                                                -1.446 0.148216    
## D_NeuterNeutered                                         -0.682 0.495337    
## D_DietMeat-based – raw                                    0.056 0.954949    
## D_DietVegan (consuming no animal products)               -1.315 0.188389    
## D_DietVegetarian (including eggs or milk, but not meat)  -0.505 0.613802    
## Therapeutic_FoodYes                                       2.019 0.043441 *  
## Visits1                                                  -1.198 0.231085    
## Visits2                                                  -0.732 0.464063    
## Visits3                                                  -0.353 0.724406    
## Visits3<                                                  2.040 0.041342 *  
## MedsYes                                                   4.867 1.13e-06 ***
## bs(D_Age, degree = 1, df = 2)1                            0.662 0.507917    
## bs(D_Age, degree = 1, df = 2)2                            4.521 6.16e-06 ***
## ---
## Signif. codes:  0 '***' 0.001 '**' 0.01 '*' 0.05 '.' 0.1 ' ' 1
## 
## (Dispersion parameter for binomial family taken to be 1)
## 
##     Null deviance: 652.97  on 1657  degrees of freedom
## Residual deviance: 411.36  on 1620  degrees of freedom
## AIC: 487.36
## 
## Number of Fisher Scoring iterations: 17  
  # test model fit
with(m, null.deviance - deviance)  
  ## [1] 241.6046  
  with(m, df.null - df.residual)  
  ## [1] 37  
  with(m, pchisq(null.deviance - deviance, df.null - df.residual, lower.tail = FALSE))  
  ## [1] 7.31347e-32  
  BIC(m)  
  ## [1] 693.0696  
  # Hosmer-Lemeshow Goodness-of-Fit Test
hltest(m)  
  ## 
##    The Hosmer-Lemeshow goodness-of-fit test
## 
##  Group Size Observed    Expected
##      1  166        1  0.07496339
##      2  166        0  0.20726053
##      3  166        0  0.33429905
##      4  166        0  0.53194419
##      5  166        1  0.90356257
##      6  166        0  1.64053625
##      7  166        4  3.14351933
##      8  166        8  5.97357473
##      9  166       13 14.33850918
##     10  164       55 54.85183125
## 
##          Statistic =  15.25169 
## degrees of freedom =  8 
##            p-value =  0.054432  
  ## CIs using profiled log-likelihood
confint(m, level=0.99)  
  ## Waiting for profiling to be done...  
  ##                                                                0.5 %     99.5 %
## (Intercept)                                               -8.1308806 -2.9897849
## LocationOther European                                    -0.1393624  1.9850243
## LocationNorth America                                     -1.7818359  1.3812588
## LocationAustralia/New Zealand/Oceania                     -0.5440562  2.3934691
## LocationOther                                             -1.2964560  2.4353603
## UrbanYes                                                  -1.0391901  0.5195767
## Education_S21_College                                     -1.6241106  0.6608002
## Education_S22_Grad                                        -1.0016499  1.1935968
## Education_S23_PG_or_PhD                                   -1.5538012  0.7047734
## Animal_Career_BINARYYes                                   -0.5156005  1.1757060
## Income2Medium                                             -1.0720430  0.9287228
## Income2High                                               -1.7879866  0.9114816
## C_Age230–39                                               -0.7142158  1.5530667
## C_Age240–49                                               -1.6175682  0.8177638
## C_Age250–59                                               -1.1458971  1.2588252
## C_Age260<                                                 -2.0503483  0.6920966
## C_GenderMale                                              -2.0708574  1.3135497
## C_DietOmnivore reducing animal product consumption        -0.7821564  1.0928842
## C_DietPescatarian (including fish but no other meats)     -3.2539676  0.5318847
## C_DietVegan (consuming no animal products)                -1.1030527  1.2922506
## C_DietVegetarian (consuming plants, eggs and milk)        -0.9149750  1.4223924
## Size2Toy                                                -406.8780446 24.3336109
## Size2Small                                                -1.5727823  0.6322620
## Size2Large                                                -0.2820615  1.3608243
## Size2Giant                                                 0.5262716  3.5479646
## D_SexMale                                                 -1.1234891  0.3114915
## D_NeuterNeutered                                          -1.3876983  0.8983575
## D_DietMeat-based – raw                                    -0.8699340  0.8606778
## D_DietVegan (consuming no animal products)                -2.3496891  0.6933745
## D_DietVegetarian (including eggs or milk, but not meat)   -3.5827672  1.7809353
## Therapeutic_FoodYes                                       -0.2528850  1.7823832
## Visits1                                                   -2.7482959  1.3323711
## Visits2                                                   -2.3599111  1.6879781
## Visits3                                                   -2.3166941  2.0560268
## Visits3<                                                  -0.2388561  3.6840617
## MedsYes                                                    1.3185031  4.0546145
## bs(D_Age, degree = 1, df = 2)1                            -1.0556957  1.9280008
## bs(D_Age, degree = 1, df = 2)2                             1.4069221  5.0759805  
  ## CIs using standard errors
confint.default(m, level=0.99)  
  ##                                                                 0.5 %
## (Intercept)                                                -7.8660447
## LocationOther European                                     -0.1089840
## LocationNorth America                                      -1.5821079
## LocationAustralia/New Zealand/Oceania                      -0.4419739
## LocationOther                                              -1.0939704
## UrbanYes                                                   -1.0152166
## Education_S21_College                                      -1.6230268
## Education_S22_Grad                                         -1.0193565
## Education_S23_PG_or_PhD                                    -1.5588101
## Animal_Career_BINARYYes                                    -0.4911597
## Income2Medium                                              -1.1111869
## Income2High                                                -1.7509610
## C_Age230–39                                                -0.7338734
## C_Age240–49                                                -1.6068561
## C_Age250–59                                                -1.1551576
## C_Age260<                                                  -2.0269775
## C_GenderMale                                               -1.8013817
## C_DietOmnivore reducing animal product consumption         -0.7626124
## C_DietPescatarian (including fish but no other meats)      -2.8884825
## C_DietVegan (consuming no animal products)                 -1.0618831
## C_DietVegetarian (consuming plants, eggs and milk)         -0.8623324
## Size2Toy                                                -2305.1137467
## Size2Small                                                 -1.5149358
## Size2Large                                                 -0.2897992
## Size2Giant                                                  0.5984137
## D_SexMale                                                  -1.1141299
## D_NeuterNeutered                                           -1.4272160
## D_DietMeat-based – raw                                     -0.8393639
## D_DietVegan (consuming no animal products)                 -2.2616061
## D_DietVegetarian (including eggs or milk, but not meat)    -3.0513034
## Therapeutic_FoodYes                                        -0.2181589
## Visits1                                                    -2.8300224
## Visits2                                                    -2.4748287
## Visits3                                                    -2.3749870
## Visits3<                                                   -0.3887822
## MedsYes                                                     1.1939407
## bs(D_Age, degree = 1, df = 2)1                             -1.0981146
## bs(D_Age, degree = 1, df = 2)2                              1.3762653
##                                                               99.5 %
## (Intercept)                                               -2.7988747
## LocationOther European                                     1.9987804
## LocationNorth America                                      1.5149140
## LocationAustralia/New Zealand/Oceania                      2.4574797
## LocationOther                                              2.5368001
## UrbanYes                                                   0.5341865
## Education_S21_College                                      0.6385354
## Education_S22_Grad                                         1.1555691
## Education_S23_PG_or_PhD                                    0.6785823
## Animal_Career_BINARYYes                                    1.1887076
## Income2Medium                                              0.8683731
## Income2High                                                0.9133700
## C_Age230–39                                                1.5078271
## C_Age240–49                                                0.7972106
## C_Age250–59                                                1.2226280
## C_Age260<                                                  0.6843823
## C_GenderMale                                               1.4786761
## C_DietOmnivore reducing animal product consumption         1.0974579
## C_DietPescatarian (including fish but no other meats)      0.7254088
## C_DietVegan (consuming no animal products)                 1.3153561
## C_DietVegetarian (consuming plants, eggs and milk)         1.4506361
## Size2Toy                                                2275.4686046
## Size2Small                                                 0.6650521
## Size2Large                                                 1.3419116
## Size2Giant                                                 3.5799395
## D_SexMale                                                  0.3130349
## D_NeuterNeutered                                           0.8297709
## D_DietMeat-based – raw                                     0.8770074
## D_DietVegan (consuming no animal products)                 0.7325992
## D_DietVegetarian (including eggs or milk, but not meat)    2.0515561
## Therapeutic_FoodYes                                        1.8018158
## Visits1                                                    1.0336856
## Visits2                                                    1.3793009
## Visits3                                                    1.8030957
## Visits3<                                                   3.3497079
## MedsYes                                                    3.8780074
## bs(D_Age, degree = 1, df = 2)1                             1.8579280
## bs(D_Age, degree = 1, df = 2)2                             5.0212367  
  # Wald test
wald.test(b = coef(m), Sigma = vcov(m), Terms = 2)  
  ## Wald test:
## ----------
## 
## Chi-squared test:
## X2 = 5.3, df = 1, P(> X2) = 0.021  
  ## odds ratios and 95% CI
exp(cbind(OR = coef(m), confint(m, level=0.99)))  
  ## Waiting for profiling to be done...  
  ##                                                                   OR
## (Intercept)                                             4.832170e-03
## LocationOther European                                  2.572551e+00
## LocationNorth America                                   9.669612e-01
## LocationAustralia/New Zealand/Oceania                   2.739438e+00
## LocationOther                                           2.057342e+00
## UrbanYes                                                7.862228e-01
## Education_S21_College                                   6.112522e-01
## Education_S22_Grad                                      1.070479e+00
## Education_S23_PG_or_PhD                                 6.439631e-01
## Animal_Career_BINARYYes                                 1.417329e+00
## Income2Medium                                           8.856735e-01
## Income2High                                             6.578387e-01
## C_Age230–39                                             1.472522e+00
## C_Age240–49                                             6.670950e-01
## C_Age250–59                                             1.034311e+00
## C_Age260<                                               5.110450e-01
## C_GenderMale                                            8.509918e-01
## C_DietOmnivore reducing animal product consumption      1.182254e+00
## C_DietPescatarian (including fish but no other meats)   3.390740e-01
## C_DietVegan (consuming no animal products)              1.135118e+00
## C_DietVegetarian (consuming plants, eggs and milk)      1.341988e+00
## Size2Toy                                                3.652912e-07
## Size2Small                                              6.538078e-01
## Size2Large                                              1.692245e+00
## Size2Giant                                              8.078261e+00
## D_SexMale                                               6.699532e-01
## D_NeuterNeutered                                        7.417652e-01
## D_DietMeat-based – raw                                  1.019000e+00
## D_DietVegan (consuming no animal products)              4.655651e-01
## D_DietVegetarian (including eggs or milk, but not meat) 6.066073e-01
## Therapeutic_FoodYes                                     2.207429e+00
## Visits1                                                 4.073150e-01
## Visits2                                                 5.782414e-01
## Visits3                                                 7.513034e-01
## Visits3<                                                4.394979e+00
## MedsYes                                                 1.262873e+01
## bs(D_Age, degree = 1, df = 2)1                          1.462148e+00
## bs(D_Age, degree = 1, df = 2)2                          2.450191e+01
##                                                                 0.5 %
## (Intercept)                                              2.943089e-04
## LocationOther European                                   8.699127e-01
## LocationNorth America                                    1.683288e-01
## LocationAustralia/New Zealand/Oceania                    5.803893e-01
## LocationOther                                            2.734994e-01
## UrbanYes                                                 3.537411e-01
## Education_S21_College                                    1.970869e-01
## Education_S22_Grad                                       3.672730e-01
## Education_S23_PG_or_PhD                                  2.114427e-01
## Animal_Career_BINARYYes                                  5.971419e-01
## Income2Medium                                            3.423085e-01
## Income2High                                              1.672967e-01
## C_Age230–39                                              4.895759e-01
## C_Age240–49                                              1.983805e-01
## C_Age250–59                                              3.179386e-01
## C_Age260<                                                1.286901e-01
## C_GenderMale                                             1.260776e-01
## C_DietOmnivore reducing animal product consumption       4.574185e-01
## C_DietPescatarian (including fish but no other meats)    3.862067e-02
## C_DietVegan (consuming no animal products)               3.318565e-01
## C_DietVegetarian (consuming plants, eggs and milk)       4.005266e-01
## Size2Toy                                                1.972924e-177
## Size2Small                                               2.074671e-01
## Size2Large                                               7.542273e-01
## Size2Giant                                               1.692610e+00
## D_SexMale                                                3.251434e-01
## D_NeuterNeutered                                         2.496493e-01
## D_DietMeat-based – raw                                   4.189792e-01
## D_DietVegan (consuming no animal products)               9.539882e-02
## D_DietVegetarian (including eggs or milk, but not meat)  2.779867e-02
## Therapeutic_FoodYes                                      7.765572e-01
## Visits1                                                  6.403689e-02
## Visits2                                                  9.442861e-02
## Visits3                                                  9.859901e-02
## Visits3<                                                 7.875282e-01
## MedsYes                                                  3.737822e+00
## bs(D_Age, degree = 1, df = 2)1                           3.479503e-01
## bs(D_Age, degree = 1, df = 2)2                           4.083368e+00
##                                                               99.5 %
## (Intercept)                                             5.029826e-02
## LocationOther European                                  7.279225e+00
## LocationNorth America                                   3.979908e+00
## LocationAustralia/New Zealand/Oceania                   1.095142e+01
## LocationOther                                           1.141993e+01
## UrbanYes                                                1.681316e+00
## Education_S21_College                                   1.936341e+00
## Education_S22_Grad                                      3.298925e+00
## Education_S23_PG_or_PhD                                 2.023388e+00
## Animal_Career_BINARYYes                                 3.240430e+00
## Income2Medium                                           2.531274e+00
## Income2High                                             2.488006e+00
## C_Age230–39                                             4.725941e+00
## C_Age240–49                                             2.265428e+00
## C_Age250–59                                             3.521282e+00
## C_Age260<                                               1.997900e+00
## C_GenderMale                                            3.719353e+00
## C_DietOmnivore reducing animal product consumption      2.982865e+00
## C_DietPescatarian (including fish but no other meats)   1.702137e+00
## C_DietVegan (consuming no animal products)              3.640972e+00
## C_DietVegetarian (consuming plants, eggs and milk)      4.147030e+00
## Size2Toy                                                3.697881e+10
## Size2Small                                              1.881863e+00
## Size2Large                                              3.899406e+00
## Size2Giant                                              3.474253e+01
## D_SexMale                                               1.365460e+00
## D_NeuterNeutered                                        2.455566e+00
## D_DietMeat-based – raw                                  2.364763e+00
## D_DietVegan (consuming no animal products)              2.000455e+00
## D_DietVegetarian (including eggs or milk, but not meat) 5.935405e+00
## Therapeutic_FoodYes                                     5.944005e+00
## Visits1                                                 3.790019e+00
## Visits2                                                 5.408534e+00
## Visits3                                                 7.814858e+00
## Visits3<                                                3.980775e+01
## MedsYes                                                 5.766293e+01
## bs(D_Age, degree = 1, df = 2)1                          6.875750e+00
## bs(D_Age, degree = 1, df = 2)2                          1.601291e+02  
 
 Calculate Nagelkerke R^2 
  NagelkerkeR2(m)  
  ## $N
## [1] 1658
## 
## $R2
## [1] 0.4165541  
 
 
 check assumptions of model 
 
 Cook’s distance 
  plot(m, which = 4, id.n = 3)  
   
 
 
 Extract model results and display data for top 3 values using Cook’s
distance 
  model.data <- augment(m) %>% 
  mutate(index = 1:n()) 
model.data %>% top_n(3, .cooksd)  
 
 
 
 
 
 plot standardised residuals 
  ggplot(model.data, aes(index, .std.resid)) + 
  geom_point(aes(color = Health_Binary), alpha = .5) +
  theme_bw()  
   
 
 
 Filter potential influential data points with abs(.std.res) >
3: 
  model.data %>% 
  filter(abs(.std.resid) > 3)  
 
 
 
 
 
 check for multicollinearity 
  car::vif(m)  
  ##                                   GVIF Df GVIF^(1/(2*Df))
## Location                      1.978738  4        1.089052
## Urban                         1.194840  1        1.093088
## Education_S2                  1.333438  3        1.049129
## Animal_Career_BINARY          1.168300  1        1.080879
## Income2                       1.153154  2        1.036267
## C_Age2                        1.631480  4        1.063097
## C_Gender                      1.148240  1        1.071560
## C_Diet                        2.260935  4        1.107353
## Size2                         1.497943  4        1.051809
## D_Sex                         1.092754  1        1.045349
## D_Neuter                      1.325796  1        1.151432
## D_Diet                        2.100238  3        1.131648
## Therapeutic_Food              1.184251  1        1.088233
## Visits                        1.664887  4        1.065794
## Meds                          1.418004  1        1.190800
## bs(D_Age, degree = 1, df = 2) 1.466703  2        1.100489  
 
 
 
 Create ROCR for training and test data 
  ## training data
pred.mtt = predict(m, type = "response") #repeat risk predictions from model m
rocr.pred.mtt = ROCR::prediction(pred.mtt, labels = ml_train$Health_Binary) #ROCR prediction object
roc.perf.mtt = ROCR::performance(rocr.pred.mtt, measure = "tpr", x.measure = "fpr") # #ROCR performance object
plot(roc.perf.mtt, col = "blue")


pred.te.1 = predict(m, newdata = ml_test, type = "response") #.te = "test"
rocr.pred.te.1 = ROCR::prediction(pred.te.1, labels = ml_test$Health_Binary)
roc.perf.te.1 = ROCR::performance(rocr.pred.te.1, measure = "tpr", x.measure = "fpr")
plot(roc.perf.te.1, col = "red", add = T)

abline(a = 0, b = 1, lty = 2) #diagonal for random assignment
legend("bottomright", legend = c("train","test"),
col = c("blue","red"), lty = c(2,1), lwd =1.5)  
   
 
 
 Report AUC from ROC for training and test data 
    # Train AUC
aucTr <- ROCR::performance(rocr.pred.mtt, measure = "auc")
  aucTr <- aucTr@y.values[[1]]
  print(aucTr)  
  ## [1] 0.9167234  
     # Test AUC
  aucTe <- ROCR::performance(rocr.pred.te.1, measure = "auc")
  aucTe <- aucTe@y.values[[1]]
  print(aucTe)  
  ## [1] 0.8634503  
 
 
 
 
 MULTIPLE REGRESSION WITH BACKWARDS ELIMINATION 
 
 ROUND 2: Model with all variables 
 Remove Education as least significant in round 1 
  # fit binary logit model and store results 'm'
m <- glm(Health_Binary ~  Location + Urban + Animal_Career_BINARY 
         + Income2 + C_Age2 + C_Gender + C_Diet + Size2 + D_Sex + D_Neuter  + D_Diet + Therapeutic_Food + Visits 
         + Meds + bs(D_Age, degree=1,df=2), data = ml_train,family = binomial)
# view a summary of the model
summary(m)  
  ## 
## Call:
## glm(formula = Health_Binary ~ Location + Urban + Animal_Career_BINARY + 
##     Income2 + C_Age2 + C_Gender + C_Diet + Size2 + D_Sex + D_Neuter + 
##     D_Diet + Therapeutic_Food + Visits + Meds + bs(D_Age, degree = 1, 
##     df = 2), family = binomial, data = ml_train)
## 
## Coefficients:
##                                                          Estimate Std. Error
## (Intercept)                                              -5.48515    0.93712
## LocationOther European                                    0.93146    0.40584
## LocationNorth America                                    -0.03131    0.58457
## LocationAustralia/New Zealand/Oceania                     1.07393    0.55642
## LocationOther                                             0.74392    0.69786
## UrbanYes                                                 -0.23432    0.29833
## Animal_Career_BINARYYes                                   0.32977    0.32281
## Income2Medium                                            -0.13346    0.38215
## Income2High                                              -0.49733    0.51348
## C_Age230–39                                               0.36391    0.43351
## C_Age240–49                                              -0.42456    0.46601
## C_Age250–59                                               0.02234    0.45700
## C_Age260<                                                -0.61544    0.51691
## C_GenderMale                                             -0.20625    0.64594
## C_DietOmnivore reducing animal product consumption        0.12705    0.35737
## C_DietPescatarian (including fish but no other meats)    -1.05233    0.69453
## C_DietVegan (consuming no animal products)                0.07941    0.45718
## C_DietVegetarian (consuming plants, eggs and milk)        0.22648    0.44385
## Size2Toy                                                -14.82472  891.17062
## Size2Small                                               -0.39959    0.42024
## Size2Large                                                0.52700    0.31627
## Size2Giant                                                2.00899    0.57612
## D_SexMale                                                -0.35439    0.27467
## D_NeuterNeutered                                         -0.26775    0.43582
## D_DietMeat-based – raw                                    0.03962    0.32949
## D_DietVegan (consuming no animal products)               -0.70481    0.57678
## D_DietVegetarian (including eggs or milk, but not meat)  -0.41717    0.96996
## Therapeutic_FoodYes                                       0.74769    0.38671
## Visits1                                                  -0.95663    0.74716
## Visits2                                                  -0.62641    0.74496
## Visits3                                                  -0.32586    0.80849
## Visits3<                                                  1.39325    0.72125
## MedsYes                                                   2.52721    0.52154
## bs(D_Age, degree = 1, df = 2)1                            0.41099    0.57516
## bs(D_Age, degree = 1, df = 2)2                            3.10990    0.70206
##                                                         z value Pr(>|z|)    
## (Intercept)                                              -5.853 4.82e-09 ***
## LocationOther European                                    2.295 0.021725 *  
## LocationNorth America                                    -0.054 0.957282    
## LocationAustralia/New Zealand/Oceania                     1.930 0.053598 .  
## LocationOther                                             1.066 0.286423    
## UrbanYes                                                 -0.785 0.432198    
## Animal_Career_BINARYYes                                   1.022 0.306982    
## Income2Medium                                            -0.349 0.726913    
## Income2High                                              -0.969 0.332766    
## C_Age230–39                                               0.839 0.401219    
## C_Age240–49                                              -0.911 0.362270    
## C_Age250–59                                               0.049 0.961018    
## C_Age260<                                                -1.191 0.233807    
## C_GenderMale                                             -0.319 0.749493    
## C_DietOmnivore reducing animal product consumption        0.356 0.722192    
## C_DietPescatarian (including fish but no other meats)    -1.515 0.129733    
## C_DietVegan (consuming no animal products)                0.174 0.862106    
## C_DietVegetarian (consuming plants, eggs and milk)        0.510 0.609870    
## Size2Toy                                                 -0.017 0.986728    
## Size2Small                                               -0.951 0.341673    
## Size2Large                                                1.666 0.095657 .  
## Size2Giant                                                3.487 0.000488 ***
## D_SexMale                                                -1.290 0.196968    
## D_NeuterNeutered                                         -0.614 0.538984    
## D_DietMeat-based – raw                                    0.120 0.904290    
## D_DietVegan (consuming no animal products)               -1.222 0.221716    
## D_DietVegetarian (including eggs or milk, but not meat)  -0.430 0.667128    
## Therapeutic_FoodYes                                       1.933 0.053181 .  
## Visits1                                                  -1.280 0.200420    
## Visits2                                                  -0.841 0.400424    
## Visits3                                                  -0.403 0.686912    
## Visits3<                                                  1.932 0.053396 .  
## MedsYes                                                   4.846 1.26e-06 ***
## bs(D_Age, degree = 1, df = 2)1                            0.715 0.474876    
## bs(D_Age, degree = 1, df = 2)2                            4.430 9.44e-06 ***
## ---
## Signif. codes:  0 '***' 0.001 '**' 0.01 '*' 0.05 '.' 0.1 ' ' 1
## 
## (Dispersion parameter for binomial family taken to be 1)
## 
##     Null deviance: 652.97  on 1657  degrees of freedom
## Residual deviance: 414.79  on 1623  degrees of freedom
## AIC: 484.79
## 
## Number of Fisher Scoring iterations: 17  
  # test model fit
with(m, null.deviance - deviance)  
  ## [1] 238.1792  
  with(m, df.null - df.residual)  
  ## [1] 34  
  with(m, pchisq(null.deviance - deviance, df.null - df.residual, lower.tail = FALSE))  
  ## [1] 1.719589e-32  
  BIC(m)  
  ## [1] 674.2549  
  # Hosmer-Lemeshow Goodness-of-Fit Test
hltest(m)  
  ## 
##    The Hosmer-Lemeshow goodness-of-fit test
## 
##  Group Size Observed    Expected
##      1  166        0  0.08393176
##      2  166        1  0.22676174
##      3  166        0  0.36707525
##      4  166        0  0.54161643
##      5  166        1  0.92168198
##      6  166        1  1.70916273
##      7  166        3  3.14386301
##      8  166        7  6.17301777
##      9  166       16 14.51076916
##     10  164       53 54.32212062
## 
##          Statistic =  4.27691 
## degrees of freedom =  8 
##            p-value =  0.83132  
  ## CIs using profiled log-likelihood
confint(m, level=0.99)  
  ## Waiting for profiling to be done...  
  ##                                                                0.5 %     99.5 %
## (Intercept)                                               -8.1804294 -3.2716428
## LocationOther European                                    -0.1451109  1.9618937
## LocationNorth America                                     -1.7337574  1.3475874
## LocationAustralia/New Zealand/Oceania                     -0.4595579  2.4445439
## LocationOther                                             -1.2599577  2.4415985
## UrbanYes                                                  -1.0262036  0.5198763
## Animal_Career_BINARYYes                                   -0.5260181  1.1482043
## Income2Medium                                             -1.0784040  0.9117281
## Income2High                                               -1.8572512  0.8233128
## C_Age230–39                                               -0.7330210  1.5260027
## C_Age240–49                                               -1.6350944  0.7968304
## C_Age250–59                                               -1.1439139  1.2375704
## C_Age260<                                                 -1.9687747  0.7263580
## C_GenderMale                                              -2.1398543  1.2886568
## C_DietOmnivore reducing animal product consumption        -0.8138359  1.0420943
## C_DietPescatarian (including fish but no other meats)     -3.2101808  0.5430888
## C_DietVegan (consuming no animal products)                -1.1399314  1.2324830
## C_DietVegetarian (consuming plants, eggs and milk)        -0.9707920  1.3395026
## Size2Toy                                                -368.3862814 31.6058119
## Size2Small                                                -1.5377821  0.6517545
## Size2Large                                                -0.2795537  1.3609145
## Size2Giant                                                 0.4528679  3.4615726
## D_SexMale                                                 -1.0703757  0.3523040
## D_NeuterNeutered                                          -1.3499071  0.9244003
## D_DietMeat-based – raw                                    -0.8384289  0.8729990
## D_DietVegan (consuming no animal products)                -2.2804790  0.7400632
## D_DietVegetarian (including eggs or milk, but not meat)   -3.4400616  1.8298080
## Therapeutic_FoodYes                                       -0.2849054  1.7223761
## Visits1                                                   -2.7968470  1.2691404
## Visits2                                                   -2.4244370  1.6054427
## Visits3                                                   -2.3486506  2.0119902
## Visits3<                                                  -0.3080342  3.5907990
## MedsYes                                                    1.3086799  4.0462921
## bs(D_Age, degree = 1, df = 2)1                            -1.0276884  1.9623544
## bs(D_Age, degree = 1, df = 2)2                             1.3316795  4.9725405  
  ## CIs using standard errors
confint.default(m, level=0.99)  
  ##                                                                 0.5 %
## (Intercept)                                                -7.8989983
## LocationOther European                                     -0.1139131
## LocationNorth America                                      -1.5370783
## LocationAustralia/New Zealand/Oceania                      -0.3593104
## LocationOther                                              -1.0536439
## UrbanYes                                                   -1.0027816
## Animal_Career_BINARYYes                                    -0.5017215
## Income2Medium                                              -1.1178000
## Income2High                                                -1.8199665
## C_Age230–39                                                -0.7527380
## C_Age240–49                                                -1.6249177
## C_Age250–59                                                -1.1548161
## C_Age260<                                                  -1.9469081
## C_GenderMale                                               -1.8700791
## C_DietOmnivore reducing animal product consumption         -0.7934596
## C_DietPescatarian (including fish but no other meats)      -2.8413238
## C_DietVegan (consuming no animal products)                 -1.0981990
## C_DietVegetarian (consuming plants, eggs and milk)         -0.9167943
## Size2Toy                                                -2310.3281314
## Size2Small                                                 -1.4820514
## Size2Large                                                 -0.2876651
## Size2Giant                                                  0.5250091
## D_SexMale                                                  -1.0618888
## D_NeuterNeutered                                           -1.3903495
## D_DietMeat-based – raw                                     -0.8090953
## D_DietVegan (consuming no animal products)                 -2.1904901
## D_DietVegetarian (including eggs or milk, but not meat)    -2.9156118
## Therapeutic_FoodYes                                        -0.2484161
## Visits1                                                    -2.8811902
## Visits2                                                    -2.5453016
## Visits3                                                    -2.4083804
## Visits3<                                                   -0.4645781
## MedsYes                                                     1.1838223
## bs(D_Age, degree = 1, df = 2)1                             -1.0705196
## bs(D_Age, degree = 1, df = 2)2                              1.3015199
##                                                               99.5 %
## (Intercept)                                               -3.0712989
## LocationOther European                                     1.9768295
## LocationNorth America                                      1.4744524
## LocationAustralia/New Zealand/Oceania                      2.5071753
## LocationOther                                              2.5414772
## UrbanYes                                                   0.5341360
## Animal_Career_BINARYYes                                    1.1612604
## Income2Medium                                              0.8508833
## Income2High                                                0.8253001
## C_Age230–39                                                1.4805534
## C_Age240–49                                                0.7758042
## C_Age250–59                                                1.1994890
## C_Age260<                                                  0.7160328
## C_GenderMale                                               1.4575734
## C_DietOmnivore reducing animal product consumption         1.0475689
## C_DietPescatarian (including fish but no other meats)      0.7366731
## C_DietVegan (consuming no animal products)                 1.2570167
## C_DietVegetarian (consuming plants, eggs and milk)         1.3697460
## Size2Toy                                                2280.6786883
## Size2Small                                                 0.6828726
## Size2Large                                                 1.3416679
## Size2Giant                                                 3.4929773
## D_SexMale                                                  0.3531118
## D_NeuterNeutered                                           0.8548550
## D_DietMeat-based – raw                                     0.8883340
## D_DietVegan (consuming no animal products)                 0.7808722
## D_DietVegetarian (including eggs or milk, but not meat)    2.0812700
## Therapeutic_FoodYes                                        1.7437950
## Visits1                                                    0.9679278
## Visits2                                                    1.2924806
## Visits3                                                    1.7566610
## Visits3<                                                   3.2510806
## MedsYes                                                    3.8706037
## bs(D_Age, degree = 1, df = 2)1                             1.8924982
## bs(D_Age, degree = 1, df = 2)2                             4.9182846  
  # Wald test
wald.test(b = coef(m), Sigma = vcov(m), Terms = 2)  
  ## Wald test:
## ----------
## 
## Chi-squared test:
## X2 = 5.3, df = 1, P(> X2) = 0.022  
  ## odds ratios and 95% CI
exp(cbind(OR = coef(m), confint(m, level=0.99)))  
  ## Waiting for profiling to be done...  
  ##                                                                   OR
## (Intercept)                                             4.147919e-03
## LocationOther European                                  2.538208e+00
## LocationNorth America                                   9.691722e-01
## LocationAustralia/New Zealand/Oceania                   2.926867e+00
## LocationOther                                           2.104161e+00
## UrbanYes                                                7.911064e-01
## Animal_Career_BINARYYes                                 1.390647e+00
## Income2Medium                                           8.750639e-01
## Income2High                                             6.081503e-01
## C_Age230–39                                             1.438941e+00
## C_Age240–49                                             6.540596e-01
## C_Age250–59                                             1.022588e+00
## C_Age260<                                               5.404044e-01
## C_GenderMale                                            8.136273e-01
## C_DietOmnivore reducing animal product consumption      1.135479e+00
## C_DietPescatarian (including fish but no other meats)   3.491250e-01
## C_DietVegan (consuming no animal products)              1.082647e+00
## C_DietVegetarian (consuming plants, eggs and milk)      1.254172e+00
## Size2Toy                                                3.645065e-07
## Size2Small                                              6.705953e-01
## Size2Large                                              1.693846e+00
## Size2Giant                                              7.455807e+00
## D_SexMale                                               7.016023e-01
## D_NeuterNeutered                                        7.651012e-01
## D_DietMeat-based – raw                                  1.040415e+00
## D_DietVegan (consuming no animal products)              4.942030e-01
## D_DietVegetarian (including eggs or milk, but not meat) 6.589083e-01
## Therapeutic_FoodYes                                     2.112114e+00
## Visits1                                                 3.841849e-01
## Visits2                                                 5.345070e-01
## Visits3                                                 7.219065e-01
## Visits3<                                                4.027925e+00
## MedsYes                                                 1.251857e+01
## bs(D_Age, degree = 1, df = 2)1                          1.508309e+00
## bs(D_Age, degree = 1, df = 2)2                          2.241885e+01
##                                                                 0.5 %
## (Intercept)                                              2.800816e-04
## LocationOther European                                   8.649263e-01
## LocationNorth America                                    1.766195e-01
## LocationAustralia/New Zealand/Oceania                    6.315628e-01
## LocationOther                                            2.836660e-01
## UrbanYes                                                 3.583649e-01
## Animal_Career_BINARYYes                                  5.909534e-01
## Income2Medium                                            3.401379e-01
## Income2High                                              1.561011e-01
## C_Age230–39                                              4.804553e-01
## C_Age240–49                                              1.949340e-01
## C_Age250–59                                              3.185697e-01
## C_Age260<                                                1.396278e-01
## C_GenderMale                                             1.176720e-01
## C_DietOmnivore reducing animal product consumption       4.431549e-01
## C_DietPescatarian (including fish but no other meats)    4.034932e-02
## C_DietVegan (consuming no animal products)               3.198410e-01
## C_DietVegetarian (consuming plants, eggs and milk)       3.787829e-01
## Size2Toy                                                1.027710e-160
## Size2Small                                               2.148571e-01
## Size2Large                                               7.561211e-01
## Size2Giant                                               1.572816e+00
## D_SexMale                                                3.428797e-01
## D_NeuterNeutered                                         2.592644e-01
## D_DietMeat-based – raw                                   4.323893e-01
## D_DietVegan (consuming no animal products)               1.022352e-01
## D_DietVegetarian (including eggs or milk, but not meat)  3.206271e-02
## Therapeutic_FoodYes                                      7.520854e-01
## Visits1                                                  6.100210e-02
## Visits2                                                  8.852795e-02
## Visits3                                                  9.549794e-02
## Visits3<                                                 7.348902e-01
## MedsYes                                                  3.701284e+00
## bs(D_Age, degree = 1, df = 2)1                           3.578332e-01
## bs(D_Age, degree = 1, df = 2)2                           3.787399e+00
##                                                               99.5 %
## (Intercept)                                             3.794404e-02
## LocationOther European                                  7.112784e+00
## LocationNorth America                                   3.848130e+00
## LocationAustralia/New Zealand/Oceania                   1.152529e+01
## LocationOther                                           1.149140e+01
## UrbanYes                                                1.681820e+00
## Animal_Career_BINARYYes                                 3.152527e+00
## Income2Medium                                           2.488619e+00
## Income2High                                             2.278034e+00
## C_Age230–39                                             4.599753e+00
## C_Age240–49                                             2.218498e+00
## C_Age250–59                                             3.447228e+00
## C_Age260<                                               2.067537e+00
## C_GenderMale                                            3.627910e+00
## C_DietOmnivore reducing animal product consumption      2.835148e+00
## C_DietPescatarian (including fish but no other meats)   1.721315e+00
## C_DietVegan (consuming no animal products)              3.429735e+00
## C_DietVegetarian (consuming plants, eggs and milk)      3.817145e+00
## Size2Toy                                                5.323898e+13
## Size2Small                                              1.918905e+00
## Size2Large                                              3.899758e+00
## Size2Giant                                              3.186705e+01
## D_SexMale                                               1.422341e+00
## D_NeuterNeutered                                        2.520356e+00
## D_DietMeat-based – raw                                  2.394080e+00
## D_DietVegan (consuming no animal products)              2.096068e+00
## D_DietVegetarian (including eggs or milk, but not meat) 6.232690e+00
## Therapeutic_FoodYes                                     5.597814e+00
## Visits1                                                 3.557793e+00
## Visits2                                                 4.980064e+00
## Visits3                                                 7.478186e+00
## Visits3<                                                3.626304e+01
## MedsYes                                                 5.718503e+01
## bs(D_Age, degree = 1, df = 2)1                          7.116062e+00
## bs(D_Age, degree = 1, df = 2)2                          1.443933e+02  
 
 Calculate Nagelkerke R^2 
  NagelkerkeR2(m)  
  ## $N
## [1] 1658
## 
## $R2
## [1] 0.4110625  
 
 
 check assumptions of model 
 
 Cook’s distance 
  plot(m, which = 4, id.n = 3)  
   
 
 
 Extract model results and display data for top 3 values using Cook’s
distance 
  model.data <- augment(m) %>% 
  mutate(index = 1:n()) 
model.data %>% top_n(3, .cooksd)  
 
 
 
 
 
 plot standardised residuals 
  ggplot(model.data, aes(index, .std.resid)) + 
  geom_point(aes(color = Health_Binary), alpha = .5) +
  theme_bw()  
   
 
 
 Filter potential influential data points with abs(.std.res) >
3: 
  model.data %>% 
  filter(abs(.std.resid) > 3)  
 
 
 
 
 
 check for multicollinearity 
  car::vif(m)  
  ##                                   GVIF Df GVIF^(1/(2*Df))
## Location                      1.899530  4        1.083505
## Urban                         1.185786  1        1.088938
## Animal_Career_BINARY          1.160842  1        1.077424
## Income2                       1.123731  2        1.029593
## C_Age2                        1.513179  4        1.053140
## C_Gender                      1.151447  1        1.073055
## C_Diet                        2.167690  4        1.101538
## Size2                         1.478838  4        1.050123
## D_Sex                         1.082539  1        1.040451
## D_Neuter                      1.316566  1        1.147417
## D_Diet                        1.998287  3        1.122302
## Therapeutic_Food              1.177718  1        1.085227
## Visits                        1.646423  4        1.064309
## Meds                          1.420736  1        1.191946
## bs(D_Age, degree = 1, df = 2) 1.446481  2        1.096676  
 
 
 
 Create ROCR for training and test data 
  ## training data
pred.mtt = predict(m, type = "response") #repeat risk predictions from model m
rocr.pred.mtt = ROCR::prediction(pred.mtt, labels = ml_train$Health_Binary) #ROCR prediction object
roc.perf.mtt = ROCR::performance(rocr.pred.mtt, measure = "tpr", x.measure = "fpr") # #ROCR performance object
plot(roc.perf.mtt, col = "blue")


pred.te.1 = predict(m, newdata = ml_test, type = "response") #.te = "test"
rocr.pred.te.1 = ROCR::prediction(pred.te.1, labels = ml_test$Health_Binary)
roc.perf.te.1 = ROCR::performance(rocr.pred.te.1, measure = "tpr", x.measure = "fpr")
plot(roc.perf.te.1, col = "red", add = T)

abline(a = 0, b = 1, lty = 2) #diagonal for random assignment
legend("bottomright", legend = c("train","test"),
col = c("blue","red"), lty = c(2,1), lwd =1.5)  
   
 
 
 Report AUC from ROC for training and test data 
    # Train AUC
aucTr <- ROCR::performance(rocr.pred.mtt, measure = "auc")
  aucTr <- aucTr@y.values[[1]]
  print(aucTr)  
  ## [1] 0.9153577  
     # Test AUC
  aucTe <- ROCR::performance(rocr.pred.te.1, measure = "auc")
  aucTe <- aucTe@y.values[[1]]
  print(aucTe)  
  ## [1] 0.861501  
 
 
 
 
 MULTIPLE REGRESSION WITH BACKWARDS ELIMINATION 
 
 ROUND 3: Model with all variables 
 Remove C_Age2 as least significant in round 2 
  # fit binary logit model and store results 'm'
m <- glm(Health_Binary ~  Location + Urban + Animal_Career_BINARY 
         + Income2 + C_Gender + C_Diet + Size2 + D_Sex + D_Neuter  + D_Diet + Therapeutic_Food + Visits 
         + Meds + bs(D_Age, degree=1,df=2), data = ml_train,family = binomial)
# view a summary of the model
summary(m)  
  ## 
## Call:
## glm(formula = Health_Binary ~ Location + Urban + Animal_Career_BINARY + 
##     Income2 + C_Gender + C_Diet + Size2 + D_Sex + D_Neuter + 
##     D_Diet + Therapeutic_Food + Visits + Meds + bs(D_Age, degree = 1, 
##     df = 2), family = binomial, data = ml_train)
## 
## Coefficients:
##                                                          Estimate Std. Error
## (Intercept)                                              -5.59113    0.87898
## LocationOther European                                    0.99627    0.39511
## LocationNorth America                                     0.08783    0.57014
## LocationAustralia/New Zealand/Oceania                     1.05371    0.54097
## LocationOther                                             0.87769    0.68304
## UrbanYes                                                 -0.19953    0.29532
## Animal_Career_BINARYYes                                   0.43546    0.31301
## Income2Medium                                            -0.15581    0.37725
## Income2High                                              -0.51844    0.50716
## C_GenderMale                                             -0.26951    0.62809
## C_DietOmnivore reducing animal product consumption        0.05475    0.35212
## C_DietPescatarian (including fish but no other meats)    -1.31022    0.68522
## C_DietVegan (consuming no animal products)                0.08499    0.45004
## C_DietVegetarian (consuming plants, eggs and milk)        0.18946    0.43535
## Size2Toy                                                -14.70299  903.31690
## Size2Small                                               -0.39308    0.41351
## Size2Large                                                0.48068    0.31324
## Size2Giant                                                1.85604    0.56403
## D_SexMale                                                -0.26677    0.26896
## D_NeuterNeutered                                         -0.18554    0.42650
## D_DietMeat-based – raw                                    0.01614    0.32567
## D_DietVegan (consuming no animal products)               -0.64304    0.57195
## D_DietVegetarian (including eggs or milk, but not meat)  -0.56186    0.96802
## Therapeutic_FoodYes                                       0.66225    0.38364
## Visits1                                                  -0.80201    0.73666
## Visits2                                                  -0.53969    0.74003
## Visits3                                                  -0.21848    0.80048
## Visits3<                                                  1.46278    0.71618
## MedsYes                                                   2.48100    0.51567
## bs(D_Age, degree = 1, df = 2)1                            0.28928    0.56780
## bs(D_Age, degree = 1, df = 2)2                            2.92865    0.67152
##                                                         z value Pr(>|z|)    
## (Intercept)                                              -6.361 2.01e-10 ***
## LocationOther European                                    2.521 0.011686 *  
## LocationNorth America                                     0.154 0.877566    
## LocationAustralia/New Zealand/Oceania                     1.948 0.051439 .  
## LocationOther                                             1.285 0.198803    
## UrbanYes                                                 -0.676 0.499265    
## Animal_Career_BINARYYes                                   1.391 0.164160    
## Income2Medium                                            -0.413 0.679598    
## Income2High                                              -1.022 0.306664    
## C_GenderMale                                             -0.429 0.667854    
## C_DietOmnivore reducing animal product consumption        0.155 0.876431    
## C_DietPescatarian (including fish but no other meats)    -1.912 0.055862 .  
## C_DietVegan (consuming no animal products)                0.189 0.850205    
## C_DietVegetarian (consuming plants, eggs and milk)        0.435 0.663419    
## Size2Toy                                                 -0.016 0.987014    
## Size2Small                                               -0.951 0.341817    
## Size2Large                                                1.535 0.124896    
## Size2Giant                                                3.291 0.000999 ***
## D_SexMale                                                -0.992 0.321262    
## D_NeuterNeutered                                         -0.435 0.663540    
## D_DietMeat-based – raw                                    0.050 0.960462    
## D_DietVegan (consuming no animal products)               -1.124 0.260892    
## D_DietVegetarian (including eggs or milk, but not meat)  -0.580 0.561632    
## Therapeutic_FoodYes                                       1.726 0.084306 .  
## Visits1                                                  -1.089 0.276283    
## Visits2                                                  -0.729 0.465826    
## Visits3                                                  -0.273 0.784897    
## Visits3<                                                  2.042 0.041103 *  
## MedsYes                                                   4.811 1.50e-06 ***
## bs(D_Age, degree = 1, df = 2)1                            0.509 0.610418    
## bs(D_Age, degree = 1, df = 2)2                            4.361 1.29e-05 ***
## ---
## Signif. codes:  0 '***' 0.001 '**' 0.01 '*' 0.05 '.' 0.1 ' ' 1
## 
## (Dispersion parameter for binomial family taken to be 1)
## 
##     Null deviance: 652.97  on 1657  degrees of freedom
## Residual deviance: 421.02  on 1627  degrees of freedom
## AIC: 483.02
## 
## Number of Fisher Scoring iterations: 17  
  # test model fit
with(m, null.deviance - deviance)  
  ## [1] 231.9493  
  with(m, df.null - df.residual)  
  ## [1] 30  
  with(m, pchisq(null.deviance - deviance, df.null - df.residual, lower.tail = FALSE))  
  ## [1] 4.454662e-33  
  BIC(m)  
  ## [1] 650.8313  
  # Hosmer-Lemeshow Goodness-of-Fit Test
hltest(m)  
  ## 
##    The Hosmer-Lemeshow goodness-of-fit test
## 
##  Group Size Observed   Expected
##      1  166        0  0.1141662
##      2  166        0  0.2674077
##      3  166        1  0.4026544
##      4  166        0  0.6048547
##      5  166        0  0.9615624
##      6  166        3  1.7925540
##      7  166        4  3.2950453
##      8  166        3  6.2614499
##      9  166       20 14.3024663
##     10  164       51 53.9978397
## 
##          Statistic =  8.31792 
## degrees of freedom =  8 
##            p-value =  0.40305  
  ## CIs using profiled log-likelihood
confint(m, level=0.99)  
  ## Waiting for profiling to be done...  
  ##                                                                 0.5 %
## (Intercept)                                               -8.15354226
## LocationOther European                                    -0.05160909
## LocationNorth America                                     -1.58295690
## LocationAustralia/New Zealand/Oceania                     -0.44100285
## LocationOther                                             -1.08865854
## UrbanYes                                                  -0.98276000
## Animal_Career_BINARYYes                                   -0.39604734
## Income2Medium                                             -1.08891769
## Income2High                                               -1.86374088
## C_GenderMale                                              -2.15249892
## C_DietOmnivore reducing animal product consumption        -0.87378788
## C_DietPescatarian (including fish but no other meats)     -3.45202083
## C_DietVegan (consuming no animal products)                -1.11654741
## C_DietVegetarian (consuming plants, eggs and milk)        -0.98725813
## Size2Toy                                                -386.70971179
## Size2Small                                                -1.51272974
## Size2Large                                                -0.31850556
## Size2Giant                                                 0.32941904
## D_SexMale                                                 -0.96574608
## D_NeuterNeutered                                          -1.24340547
## D_DietMeat-based – raw                                    -0.85238084
## D_DietVegan (consuming no animal products)                -2.20720591
## D_DietVegetarian (including eggs or milk, but not meat)   -3.57999029
## Therapeutic_FoodYes                                       -0.36256402
## Visits1                                                   -2.60685285
## Visits2                                                   -2.32216826
## Visits3                                                   -2.21536526
## Visits3<                                                  -0.22176083
## MedsYes                                                    1.27515049
## bs(D_Age, degree = 1, df = 2)1                            -1.13200068
## bs(D_Age, degree = 1, df = 2)2                             1.22481073
##                                                             99.5 %
## (Intercept)                                             -3.5303075
## LocationOther European                                   2.0004271
## LocationNorth America                                    1.4304600
## LocationAustralia/New Zealand/Oceania                    2.3850510
## LocationOther                                            2.5379861
## UrbanYes                                                 0.5475243
## Animal_Career_BINARYYes                                  1.2280189
## Income2Medium                                            0.8762657
## Income2High                                              0.7845658
## C_GenderMale                                             1.1869625
## C_DietOmnivore reducing animal product consumption       0.9547455
## C_DietPescatarian (including fish but no other meats)    0.2545236
## C_DietVegan (consuming no animal products)               1.2192253
## C_DietVegetarian (consuming plants, eggs and milk)       1.2792465
## Size2Toy                                                29.8745208
## Size2Small                                               0.6419272
## Size2Large                                               1.3062968
## Size2Giant                                               3.2752852
## D_SexMale                                                0.4272003
## D_NeuterNeutered                                         0.9822450
## D_DietMeat-based – raw                                   0.8387971
## D_DietVegan (consuming no animal products)               0.7897689
## D_DietVegetarian (including eggs or milk, but not meat)  1.6684519
## Therapeutic_FoodYes                                      1.6285521
## Visits1                                                  1.4067236
## Visits2                                                  1.6834992
## Visits3                                                  2.1056045
## Visits3<                                                 3.6515414
## MedsYes                                                  3.9842787
## bs(D_Age, degree = 1, df = 2)1                           1.8206904
## bs(D_Age, degree = 1, df = 2)2                           4.7080853  
  ## CIs using standard errors
confint.default(m, level=0.99)  
  ##                                                                 0.5 %
## (Intercept)                                                -7.8552341
## LocationOther European                                     -0.0214697
## LocationNorth America                                      -1.3807600
## LocationAustralia/New Zealand/Oceania                      -0.3397505
## LocationOther                                              -0.8817111
## UrbanYes                                                   -0.9602140
## Animal_Career_BINARYYes                                    -0.3707904
## Income2Medium                                              -1.1275496
## Income2High                                                -1.8248026
## C_GenderMale                                               -1.8873500
## C_DietOmnivore reducing animal product consumption         -0.8522458
## C_DietPescatarian (including fish but no other meats)      -3.0752267
## C_DietVegan (consuming no animal products)                 -1.0742219
## C_DietVegetarian (consuming plants, eggs and milk)         -0.9319215
## Size2Toy                                                -2341.4931358
## Size2Small                                                 -1.4582224
## Size2Large                                                 -0.3261715
## Size2Giant                                                  0.4031905
## D_SexMale                                                  -0.9595638
## D_NeuterNeutered                                           -1.2841439
## D_DietMeat-based – raw                                     -0.8227233
## D_DietVegan (consuming no animal products)                 -2.1162957
## D_DietVegetarian (including eggs or milk, but not meat)    -3.0553199
## Therapeutic_FoodYes                                        -0.3259393
## Visits1                                                    -2.6995280
## Visits2                                                    -2.4458835
## Visits3                                                    -2.2803720
## Visits3<                                                   -0.3819616
## MedsYes                                                     1.1527097
## bs(D_Age, degree = 1, df = 2)1                             -1.1732676
## bs(D_Age, degree = 1, df = 2)2                              1.1989345
##                                                               99.5 %
## (Intercept)                                               -3.3270357
## LocationOther European                                     2.0140024
## LocationNorth America                                      1.5564270
## LocationAustralia/New Zealand/Oceania                      2.4471670
## LocationOther                                              2.6370850
## UrbanYes                                                   0.5611550
## Animal_Career_BINARYYes                                    1.2417063
## Income2Medium                                              0.8159296
## Income2High                                                0.7879151
## C_GenderMale                                               1.3483335
## C_DietOmnivore reducing animal product consumption         0.9617515
## C_DietPescatarian (including fish but no other meats)      0.4547957
## C_DietVegan (consuming no animal products)                 1.2442058
## C_DietVegetarian (consuming plants, eggs and milk)         1.3108469
## Size2Toy                                                2312.0871500
## Size2Small                                                 0.6720634
## Size2Large                                                 1.2875281
## Size2Giant                                                 3.3088814
## D_SexMale                                                  0.4260205
## D_NeuterNeutered                                           0.9130586
## D_DietMeat-based – raw                                     0.8550124
## D_DietVegan (consuming no animal products)                 0.8302158
## D_DietVegetarian (including eggs or milk, but not meat)    1.9316011
## Therapeutic_FoodYes                                        1.6504429
## Visits1                                                    1.0955082
## Visits2                                                    1.3664960
## Visits3                                                    1.8434034
## Visits3<                                                   3.3075283
## MedsYes                                                    3.8092824
## bs(D_Age, degree = 1, df = 2)1                             1.7518268
## bs(D_Age, degree = 1, df = 2)2                             4.6583673  
  # Wald test
wald.test(b = coef(m), Sigma = vcov(m), Terms = 2)  
  ## Wald test:
## ----------
## 
## Chi-squared test:
## X2 = 6.4, df = 1, P(> X2) = 0.012  
  ## odds ratios and 95% CI
exp(cbind(OR = coef(m), confint(m, level=0.99)))  
  ## Waiting for profiling to be done...  
  ##                                                                   OR
## (Intercept)                                             3.730791e-03
## LocationOther European                                  2.708152e+00
## LocationNorth America                                   1.091806e+00
## LocationAustralia/New Zealand/Oceania                   2.868268e+00
## LocationOther                                           2.405330e+00
## UrbanYes                                                8.191160e-01
## Animal_Career_BINARYYes                                 1.545671e+00
## Income2Medium                                           8.557217e-01
## Income2High                                             5.954465e-01
## C_GenderMale                                            7.637550e-01
## C_DietOmnivore reducing animal product consumption      1.056280e+00
## C_DietPescatarian (including fish but no other meats)   2.697619e-01
## C_DietVegan (consuming no animal products)              1.088708e+00
## C_DietVegetarian (consuming plants, eggs and milk)      1.208600e+00
## Size2Toy                                                4.116909e-07
## Size2Small                                              6.749751e-01
## Size2Large                                              1.617171e+00
## Size2Giant                                              6.398323e+00
## D_SexMale                                               7.658479e-01
## D_NeuterNeutered                                        8.306534e-01
## D_DietMeat-based – raw                                  1.016276e+00
## D_DietVegan (consuming no animal products)              5.256919e-01
## D_DietVegetarian (including eggs or milk, but not meat) 5.701480e-01
## Therapeutic_FoodYes                                     1.939154e+00
## Visits1                                                 4.484268e-01
## Visits2                                                 5.829267e-01
## Visits3                                                 8.037361e-01
## Visits3<                                                4.317961e+00
## MedsYes                                                 1.195316e+01
## bs(D_Age, degree = 1, df = 2)1                          1.335465e+00
## bs(D_Age, degree = 1, df = 2)2                          1.870238e+01
##                                                                 0.5 %
## (Intercept)                                              2.877144e-04
## LocationOther European                                   9.497000e-01
## LocationNorth America                                    2.053670e-01
## LocationAustralia/New Zealand/Oceania                    6.433909e-01
## LocationOther                                            3.366678e-01
## UrbanYes                                                 3.742767e-01
## Animal_Career_BINARYYes                                  6.729748e-01
## Income2Medium                                            3.365806e-01
## Income2High                                              1.550914e-01
## C_GenderMale                                             1.161934e-01
## C_DietOmnivore reducing animal product consumption       4.173676e-01
## C_DietPescatarian (including fish but no other meats)    3.168155e-02
## C_DietVegan (consuming no animal products)               3.274083e-01
## C_DietVegetarian (consuming plants, eggs and milk)       3.725969e-01
## Size2Toy                                                1.132677e-168
## Size2Small                                               2.203078e-01
## Size2Large                                               7.272350e-01
## Size2Giant                                               1.390160e+00
## D_SexMale                                                3.806991e-01
## D_NeuterNeutered                                         2.884004e-01
## D_DietMeat-based – raw                                   4.263985e-01
## D_DietVegan (consuming no animal products)               1.100076e-01
## D_DietVegetarian (including eggs or milk, but not meat)  2.787597e-02
## Therapeutic_FoodYes                                      6.958898e-01
## Visits1                                                  7.376633e-02
## Visits2                                                  9.806073e-02
## Visits3                                                  1.091137e-01
## Visits3<                                                 8.011069e-01
## MedsYes                                                  3.579240e+00
## bs(D_Age, degree = 1, df = 2)1                           3.223876e-01
## bs(D_Age, degree = 1, df = 2)2                           3.403522e+00
##                                                               99.5 %
## (Intercept)                                             2.929591e-02
## LocationOther European                                  7.392213e+00
## LocationNorth America                                   4.180622e+00
## LocationAustralia/New Zealand/Oceania                   1.085962e+01
## LocationOther                                           1.265416e+01
## UrbanYes                                                1.728967e+00
## Animal_Career_BINARYYes                                 3.414458e+00
## Income2Medium                                           2.401913e+00
## Income2High                                             2.191455e+00
## C_GenderMale                                            3.277112e+00
## C_DietOmnivore reducing animal product consumption      2.598009e+00
## C_DietPescatarian (including fish but no other meats)   1.289847e+00
## C_DietVegan (consuming no animal products)              3.384565e+00
## C_DietVegetarian (consuming plants, eggs and milk)      3.593931e+00
## Size2Toy                                                9.426263e+12
## Size2Small                                              1.900139e+00
## Size2Large                                              3.692474e+00
## Size2Giant                                              2.645077e+01
## D_SexMale                                               1.532960e+00
## D_NeuterNeutered                                        2.670445e+00
## D_DietMeat-based – raw                                  2.313582e+00
## D_DietVegan (consuming no animal products)              2.202887e+00
## D_DietVegetarian (including eggs or milk, but not meat) 5.303951e+00
## Therapeutic_FoodYes                                     5.096490e+00
## Visits1                                                 4.082557e+00
## Visits2                                                 5.384364e+00
## Visits3                                                 8.212066e+00
## Visits3<                                                3.853402e+01
## MedsYes                                                 5.374651e+01
## bs(D_Age, degree = 1, df = 2)1                          6.176121e+00
## bs(D_Age, degree = 1, df = 2)2                          1.108397e+02  
 
 Calculate Nagelkerke R^2 
  NagelkerkeR2(m)  
  ## $N
## [1] 1658
## 
## $R2
## [1] 0.4010457  
 
 
 check assumptions of model 
 
 Cook’s distance 
  plot(m, which = 4, id.n = 3)  
   
 
 
 Extract model results and display data for top 3 values using Cook’s
distance 
  model.data <- augment(m) %>% 
  mutate(index = 1:n()) 
model.data %>% top_n(3, .cooksd)  
 
 
 
 
 
 plot standardised residuals 
  ggplot(model.data, aes(index, .std.resid)) + 
  geom_point(aes(color = Health_Binary), alpha = .5) +
  theme_bw()  
   
 
 
 Filter potential influential data points with abs(.std.res) >
3: 
  model.data %>% 
  filter(abs(.std.resid) > 3)  
 
 
 
 
 
 check for multicollinearity 
  car::vif(m)  
  ##                                   GVIF Df GVIF^(1/(2*Df))
## Location                      1.772390  4        1.074162
## Urban                         1.176735  1        1.084774
## Animal_Career_BINARY          1.105893  1        1.051614
## Income2                       1.105111  2        1.025301
## C_Gender                      1.120108  1        1.058352
## C_Diet                        1.994434  4        1.090128
## Size2                         1.412671  4        1.044131
## D_Sex                         1.053211  1        1.026261
## D_Neuter                      1.279025  1        1.130940
## D_Diet                        1.929869  3        1.115804
## Therapeutic_Food              1.163050  1        1.078448
## Visits                        1.578183  4        1.058692
## Meds                          1.393364  1        1.180409
## bs(D_Age, degree = 1, df = 2) 1.330932  2        1.074086  
 
 
 
 Create ROCR for training and test data 
  ## training data
pred.mtt = predict(m, type = "response") #repeat risk predictions from model m
rocr.pred.mtt = ROCR::prediction(pred.mtt, labels = ml_train$Health_Binary) #ROCR prediction object
roc.perf.mtt = ROCR::performance(rocr.pred.mtt, measure = "tpr", x.measure = "fpr") # #ROCR performance object
plot(roc.perf.mtt, col = "blue")


pred.te.1 = predict(m, newdata = ml_test, type = "response") #.te = "test"
rocr.pred.te.1 = ROCR::prediction(pred.te.1, labels = ml_test$Health_Binary)
roc.perf.te.1 = ROCR::performance(rocr.pred.te.1, measure = "tpr", x.measure = "fpr")
plot(roc.perf.te.1, col = "red", add = T)

abline(a = 0, b = 1, lty = 2) #diagonal for random assignment
legend("bottomright", legend = c("train","test"),
col = c("blue","red"), lty = c(2,1), lwd =1.5)  
   
 
 
 Report AUC from ROC for training and test data 
    # Train AUC
aucTr <- ROCR::performance(rocr.pred.mtt, measure = "auc")
  aucTr <- aucTr@y.values[[1]]
  print(aucTr)  
  ## [1] 0.9131291  
     # Test AUC
  aucTe <- ROCR::performance(rocr.pred.te.1, measure = "auc")
  aucTe <- aucTe@y.values[[1]]
  print(aucTe)  
  ## [1] 0.8682261  
 
 
 
 
 MULTIPLE REGRESSION WITH BACKWARDS ELIMINATION 
 
 ROUND 4: Model with all variables 
 Remove Income2 as least significant in round 3 
  # fit binary logit model and store results 'm'
m <- glm(Health_Binary ~  Location + Urban + Animal_Career_BINARY 
         + C_Gender + C_Diet + Size2 + D_Sex + D_Neuter  + D_Diet + Therapeutic_Food + Visits 
         + Meds + bs(D_Age, degree=1,df=2), data = ml_train,family = binomial)
# view a summary of the model
summary(m)  
  ## 
## Call:
## glm(formula = Health_Binary ~ Location + Urban + Animal_Career_BINARY + 
##     C_Gender + C_Diet + Size2 + D_Sex + D_Neuter + D_Diet + Therapeutic_Food + 
##     Visits + Meds + bs(D_Age, degree = 1, df = 2), family = binomial, 
##     data = ml_train)
## 
## Coefficients:
##                                                          Estimate Std. Error
## (Intercept)                                              -5.74635    0.83386
## LocationOther European                                    0.98160    0.39401
## LocationNorth America                                     0.05266    0.57070
## LocationAustralia/New Zealand/Oceania                     0.95919    0.53609
## LocationOther                                             0.88179    0.67513
## UrbanYes                                                 -0.19061    0.29393
## Animal_Career_BINARYYes                                   0.44577    0.31277
## C_GenderMale                                             -0.28377    0.62684
## C_DietOmnivore reducing animal product consumption        0.01982    0.34974
## C_DietPescatarian (including fish but no other meats)    -1.32639    0.68092
## C_DietVegan (consuming no animal products)                0.05282    0.44756
## C_DietVegetarian (consuming plants, eggs and milk)        0.16304    0.43269
## Size2Toy                                                -13.66750  548.65147
## Size2Small                                               -0.39282    0.41107
## Size2Large                                                0.46169    0.31254
## Size2Giant                                                1.86296    0.55975
## D_SexMale                                                -0.26992    0.26840
## D_NeuterNeutered                                         -0.18077    0.42312
## D_DietMeat-based – raw                                    0.01215    0.32601
## D_DietVegan (consuming no animal products)               -0.60132    0.57098
## D_DietVegetarian (including eggs or milk, but not meat)  -0.56274    0.95316
## Therapeutic_FoodYes                                       0.63846    0.38288
## Visits1                                                  -0.83019    0.73725
## Visits2                                                  -0.57614    0.74036
## Visits3                                                  -0.24882    0.80138
## Visits3<                                                  1.41657    0.71598
## MedsYes                                                   2.50521    0.51637
## bs(D_Age, degree = 1, df = 2)1                            0.30239    0.56773
## bs(D_Age, degree = 1, df = 2)2                            2.97275    0.66999
##                                                         z value Pr(>|z|)    
## (Intercept)                                              -6.891 5.53e-12 ***
## LocationOther European                                    2.491 0.012727 *  
## LocationNorth America                                     0.092 0.926486    
## LocationAustralia/New Zealand/Oceania                     1.789 0.073574 .  
## LocationOther                                             1.306 0.191519    
## UrbanYes                                                 -0.648 0.516678    
## Animal_Career_BINARYYes                                   1.425 0.154088    
## C_GenderMale                                             -0.453 0.650767    
## C_DietOmnivore reducing animal product consumption        0.057 0.954800    
## C_DietPescatarian (including fish but no other meats)    -1.948 0.051423 .  
## C_DietVegan (consuming no animal products)                0.118 0.906061    
## C_DietVegetarian (consuming plants, eggs and milk)        0.377 0.706312    
## Size2Toy                                                 -0.025 0.980126    
## Size2Small                                               -0.956 0.339272    
## Size2Large                                                1.477 0.139618    
## Size2Giant                                                3.328 0.000874 ***
## D_SexMale                                                -1.006 0.314576    
## D_NeuterNeutered                                         -0.427 0.669206    
## D_DietMeat-based – raw                                    0.037 0.970277    
## D_DietVegan (consuming no animal products)               -1.053 0.292283    
## D_DietVegetarian (including eggs or milk, but not meat)  -0.590 0.554928    
## Therapeutic_FoodYes                                       1.668 0.095411 .  
## Visits1                                                  -1.126 0.260134    
## Visits2                                                  -0.778 0.436462    
## Visits3                                                  -0.310 0.756188    
## Visits3<                                                  1.979 0.047870 *  
## MedsYes                                                   4.852 1.23e-06 ***
## bs(D_Age, degree = 1, df = 2)1                            0.533 0.594288    
## bs(D_Age, degree = 1, df = 2)2                            4.437 9.12e-06 ***
## ---
## Signif. codes:  0 '***' 0.001 '**' 0.01 '*' 0.05 '.' 0.1 ' ' 1
## 
## (Dispersion parameter for binomial family taken to be 1)
## 
##     Null deviance: 652.97  on 1657  degrees of freedom
## Residual deviance: 422.20  on 1629  degrees of freedom
## AIC: 480.2
## 
## Number of Fisher Scoring iterations: 16  
  # test model fit
with(m, null.deviance - deviance)  
  ## [1] 230.7701  
  with(m, df.null - df.residual)  
  ## [1] 28  
  with(m, pchisq(null.deviance - deviance, df.null - df.residual, lower.tail = FALSE))  
  ## [1] 8.994181e-34  
  BIC(m)  
  ## [1] 637.1838  
  # Hosmer-Lemeshow Goodness-of-Fit Test
hltest(m)  
  ## 
##    The Hosmer-Lemeshow goodness-of-fit test
## 
##  Group Size Observed   Expected
##      1  166        0  0.1168830
##      2  166        1  0.2668931
##      3  166        0  0.4022338
##      4  166        0  0.5896590
##      5  166        0  0.9549781
##      6  166        3  1.7923339
##      7  166        4  3.3509806
##      8  166        3  6.3047161
##      9  166       21 14.4968349
##     10  164       50 53.7244888
## 
##          Statistic =  10.4213 
## degrees of freedom =  8 
##            p-value =  0.23669  
  ## CIs using profiled log-likelihood
confint(m, level=0.99)  
  ## Waiting for profiling to be done...  
  ##                                                                 0.5 %
## (Intercept)                                               -8.21323715
## LocationOther European                                    -0.06378422
## LocationNorth America                                     -1.62081831
## LocationAustralia/New Zealand/Oceania                     -0.52432670
## LocationOther                                             -1.06986867
## UrbanYes                                                  -0.96990349
## Animal_Career_BINARYYes                                   -0.38506929
## C_GenderMale                                              -2.16487599
## C_DietOmnivore reducing animal product consumption        -0.90329104
## C_DietPescatarian (including fish but no other meats)     -3.46039752
## C_DietVegan (consuming no animal products)                -1.14358099
## C_DietVegetarian (consuming plants, eggs and milk)        -1.00775130
## Size2Toy                                                -241.81739876
## Size2Small                                                -1.50563324
## Size2Large                                                -0.33587516
## Size2Giant                                                 0.34788526
## D_SexMale                                                 -0.96743967
## D_NeuterNeutered                                          -1.23055564
## D_DietMeat-based – raw                                    -0.85741136
## D_DietVegan (consuming no animal products)                -2.16643960
## D_DietVegetarian (including eggs or milk, but not meat)   -3.55061842
## Therapeutic_FoodYes                                       -0.38504351
## Visits1                                                   -2.63654017
## Visits2                                                   -2.35933687
## Visits3                                                   -2.24791231
## Visits3<                                                  -0.26697826
## MedsYes                                                    1.29833641
## bs(D_Age, degree = 1, df = 2)1                            -1.11825345
## bs(D_Age, degree = 1, df = 2)2                             1.27302167
##                                                             99.5 %
## (Intercept)                                             -3.8119447
## LocationOther European                                   1.9828211
## LocationNorth America                                    1.3953450
## LocationAustralia/New Zealand/Oceania                    2.2748922
## LocationOther                                            2.5166262
## UrbanYes                                                 0.5531908
## Animal_Career_BINARYYes                                  1.2377550
## C_GenderMale                                             1.1680597
## C_DietOmnivore reducing animal product consumption       0.9129114
## C_DietPescatarian (including fish but no other meats)    0.2255585
## C_DietVegan (consuming no animal products)               1.1791770
## C_DietVegetarian (consuming plants, eggs and milk)       1.2451936
## Size2Toy                                                13.0059151
## Size2Small                                               0.6362284
## Size2Large                                               1.2854122
## Size2Giant                                               3.2720737
## D_SexMale                                                0.4225938
## D_NeuterNeutered                                         0.9772059
## D_DietMeat-based – raw                                   0.8353650
## D_DietVegan (consuming no animal products)               0.8273861
## D_DietVegetarian (including eggs or milk, but not meat)  1.6288023
## Therapeutic_FoodYes                                      1.6021207
## Visits1                                                  1.3795485
## Visits2                                                  1.6477353
## Visits3                                                  2.0771148
## Visits3<                                                 3.6050348
## MedsYes                                                  4.0107074
## bs(D_Age, degree = 1, df = 2)1                           1.8340125
## bs(D_Age, degree = 1, df = 2)2                           4.7489243  
  ## CIs using standard errors
confint.default(m, level=0.99)  
  ##                                                                 0.5 %
## (Intercept)                                             -7.894231e+00
## LocationOther European                                  -3.330081e-02
## LocationNorth America                                   -1.417380e+00
## LocationAustralia/New Zealand/Oceania                   -4.216707e-01
## LocationOther                                           -8.572365e-01
## UrbanYes                                                -9.477190e-01
## Animal_Career_BINARYYes                                 -3.598654e-01
## C_GenderMale                                            -1.898406e+00
## C_DietOmnivore reducing animal product consumption      -8.810595e-01
## C_DietPescatarian (including fish but no other meats)   -3.080321e+00
## C_DietVegan (consuming no animal products)              -1.100014e+00
## C_DietVegetarian (consuming plants, eggs and milk)      -9.514948e-01
## Size2Toy                                                -1.426900e+03
## Size2Small                                              -1.451681e+00
## Size2Large                                              -3.433626e-01
## Size2Giant                                               4.211419e-01
## D_SexMale                                               -9.612639e-01
## D_NeuterNeutered                                        -1.270660e+00
## D_DietMeat-based – raw                                  -8.275994e-01
## D_DietVegan (consuming no animal products)              -2.072074e+00
## D_DietVegetarian (including eggs or milk, but not meat) -3.017918e+00
## Therapeutic_FoodYes                                     -3.477737e-01
## Visits1                                                 -2.729212e+00
## Visits2                                                 -2.483188e+00
## Visits3                                                 -2.313029e+00
## Visits3<                                                -4.276588e-01
## MedsYes                                                  1.175112e+00
## bs(D_Age, degree = 1, df = 2)1                          -1.159991e+00
## bs(D_Age, degree = 1, df = 2)2                           1.246983e+00
##                                                               99.5 %
## (Intercept)                                               -3.5984730
## LocationOther European                                     1.9965036
## LocationNorth America                                      1.5226940
## LocationAustralia/New Zealand/Oceania                      2.3400566
## LocationOther                                              2.6208156
## UrbanYes                                                   0.5665067
## Animal_Career_BINARYYes                                    1.2514005
## C_GenderMale                                               1.3308682
## C_DietOmnivore reducing animal product consumption         0.9207063
## C_DietPescatarian (including fish but no other meats)      0.4275449
## C_DietVegan (consuming no animal products)                 1.2056443
## C_DietVegetarian (consuming plants, eggs and milk)         1.2775830
## Size2Toy                                                1399.5650208
## Size2Small                                                 0.6660322
## Size2Large                                                 1.2667469
## Size2Giant                                                 3.3047764
## D_SexMale                                                  0.4214272
## D_NeuterNeutered                                           0.9091132
## D_DietMeat-based – raw                                     0.8518943
## D_DietVegan (consuming no animal products)                 0.8694379
## D_DietVegetarian (including eggs or milk, but not meat)    1.8924400
## Therapeutic_FoodYes                                        1.6246968
## Visits1                                                    1.0688257
## Visits2                                                    1.3309139
## Visits3                                                    1.8153899
## Visits3<                                                   3.2608048
## MedsYes                                                    3.8353000
## bs(D_Age, degree = 1, df = 2)1                             1.7647782
## bs(D_Age, degree = 1, df = 2)2                             4.6985185  
  # Wald test
wald.test(b = coef(m), Sigma = vcov(m), Terms = 2)  
  ## Wald test:
## ----------
## 
## Chi-squared test:
## X2 = 6.2, df = 1, P(> X2) = 0.013  
  ## odds ratios and 95% CI
exp(cbind(OR = coef(m), confint(m, level=0.99)))  
  ## Waiting for profiling to be done...  
  ##                                                                   OR
## (Intercept)                                             3.194412e-03
## LocationOther European                                  2.668726e+00
## LocationNorth America                                   1.054068e+00
## LocationAustralia/New Zealand/Oceania                   2.609590e+00
## LocationOther                                           2.415218e+00
## UrbanYes                                                8.264580e-01
## Animal_Career_BINARYYes                                 1.561688e+00
## C_GenderMale                                            7.529406e-01
## C_DietOmnivore reducing animal product consumption      1.020021e+00
## C_DietPescatarian (including fish but no other meats)   2.654343e-01
## C_DietVegan (consuming no animal products)              1.054235e+00
## C_DietVegetarian (consuming plants, eggs and milk)      1.177089e+00
## Size2Toy                                                1.159521e-06
## Size2Small                                              6.751472e-01
## Size2Large                                              1.586757e+00
## Size2Giant                                              6.442774e+00
## D_SexMale                                               7.634418e-01
## D_NeuterNeutered                                        8.346246e-01
## D_DietMeat-based – raw                                  1.012222e+00
## D_DietVegan (consuming no animal products)              5.480887e-01
## D_DietVegetarian (including eggs or milk, but not meat) 5.696468e-01
## Therapeutic_FoodYes                                     1.893566e+00
## Visits1                                                 4.359651e-01
## Visits2                                                 5.620654e-01
## Visits3                                                 7.797208e-01
## Visits3<                                                4.122967e+00
## MedsYes                                                 1.224608e+01
## bs(D_Age, degree = 1, df = 2)1                          1.353094e+00
## bs(D_Age, degree = 1, df = 2)2                          1.954561e+01
##                                                                 0.5 %
## (Intercept)                                              2.710419e-04
## LocationOther European                                   9.382074e-01
## LocationNorth America                                    1.977368e-01
## LocationAustralia/New Zealand/Oceania                    5.919538e-01
## LocationOther                                            3.430536e-01
## UrbanYes                                                 3.791196e-01
## Animal_Career_BINARYYes                                  6.804035e-01
## C_GenderMale                                             1.147642e-01
## C_DietOmnivore reducing animal product consumption       4.052338e-01
## C_DietPescatarian (including fish but no other meats)    3.141727e-02
## C_DietVegan (consuming no animal products)               3.186758e-01
## C_DietVegetarian (consuming plants, eggs and milk)       3.650389e-01
## Size2Toy                                                9.550763e-106
## Size2Small                                               2.218767e-01
## Size2Large                                               7.147123e-01
## Size2Giant                                               1.416070e+00
## D_SexMale                                                3.800549e-01
## D_NeuterNeutered                                         2.921302e-01
## D_DietMeat-based – raw                                   4.242589e-01
## D_DietVegan (consuming no animal products)               1.145849e-01
## D_DietVegetarian (including eggs or milk, but not meat)  2.870688e-02
## Therapeutic_FoodYes                                      6.804210e-01
## Visits1                                                  7.160860e-02
## Visits2                                                  9.448286e-02
## Visits3                                                  1.056195e-01
## Visits3<                                                 7.656897e-01
## MedsYes                                                  3.663198e+00
## bs(D_Age, degree = 1, df = 2)1                           3.268502e-01
## bs(D_Age, degree = 1, df = 2)2                           3.571629e+00
##                                                               99.5 %
## (Intercept)                                             2.210515e-02
## LocationOther European                                  7.263204e+00
## LocationNorth America                                   4.036367e+00
## LocationAustralia/New Zealand/Oceania                   9.726870e+00
## LocationOther                                           1.238674e+01
## UrbanYes                                                1.738792e+00
## Animal_Career_BINARYYes                                 3.447864e+00
## C_GenderMale                                            3.215747e+00
## C_DietOmnivore reducing animal product consumption      2.491566e+00
## C_DietPescatarian (including fish but no other meats)   1.253022e+00
## C_DietVegan (consuming no animal products)              3.251697e+00
## C_DietVegetarian (consuming plants, eggs and milk)      3.473607e+00
## Size2Toy                                                4.450381e+05
## Size2Small                                              1.889342e+00
## Size2Large                                              3.616158e+00
## Size2Giant                                              2.636596e+01
## D_SexMale                                               1.525914e+00
## D_NeuterNeutered                                        2.657022e+00
## D_DietMeat-based – raw                                  2.305656e+00
## D_DietVegan (consuming no animal products)              2.287332e+00
## D_DietVegetarian (including eggs or milk, but not meat) 5.097766e+00
## Therapeutic_FoodYes                                     4.963547e+00
## Visits1                                                 3.973107e+00
## Visits2                                                 5.195201e+00
## Visits3                                                 7.981408e+00
## Visits3<                                                3.678296e+01
## MedsYes                                                 5.518590e+01
## bs(D_Age, degree = 1, df = 2)1                          6.258951e+00
## bs(D_Age, degree = 1, df = 2)2                          1.154600e+02  
 
 Calculate Nagelkerke R^2 
  NagelkerkeR2(m)  
  ## $N
## [1] 1658
## 
## $R2
## [1] 0.3991453  
 
 
 check assumptions of model 
 
 Cook’s distance 
  plot(m, which = 4, id.n = 3)  
   
 
 
 Extract model results and display data for top 3 values using Cook’s
distance 
  model.data <- augment(m) %>% 
  mutate(index = 1:n()) 
model.data %>% top_n(3, .cooksd)  
 
 
 
 
 
 plot standardised residuals 
  ggplot(model.data, aes(index, .std.resid)) + 
  geom_point(aes(color = Health_Binary), alpha = .5) +
  theme_bw()  
   
 
 
 Filter potential influential data points with abs(.std.res) >
3: 
  model.data %>% 
  filter(abs(.std.resid) > 3)  
 
 
 
 
 
 check for multicollinearity 
  car::vif(m)  
  ##                                   GVIF Df GVIF^(1/(2*Df))
## Location                      1.706100  4        1.069056
## Urban                         1.171848  1        1.082519
## Animal_Career_BINARY          1.101245  1        1.049402
## C_Gender                      1.123197  1        1.059810
## C_Diet                        1.946388  4        1.086810
## Size2                         1.392838  4        1.042288
## D_Sex                         1.052260  1        1.025797
## D_Neuter                      1.273231  1        1.128375
## D_Diet                        1.908393  3        1.113725
## Therapeutic_Food              1.155707  1        1.075038
## Visits                        1.568811  4        1.057904
## Meds                          1.396185  1        1.181603
## bs(D_Age, degree = 1, df = 2) 1.329132  2        1.073722  
 
 
 
 Create ROCR for training and test data 
  ## training data
pred.mtt = predict(m, type = "response") #repeat risk predictions from model m
rocr.pred.mtt = ROCR::prediction(pred.mtt, labels = ml_train$Health_Binary) #ROCR prediction object
roc.perf.mtt = ROCR::performance(rocr.pred.mtt, measure = "tpr", x.measure = "fpr") # #ROCR performance object
plot(roc.perf.mtt, col = "blue")


pred.te.1 = predict(m, newdata = ml_test, type = "response") #.te = "test"
rocr.pred.te.1 = ROCR::prediction(pred.te.1, labels = ml_test$Health_Binary)
roc.perf.te.1 = ROCR::performance(rocr.pred.te.1, measure = "tpr", x.measure = "fpr")
plot(roc.perf.te.1, col = "red", add = T)

abline(a = 0, b = 1, lty = 2) #diagonal for random assignment
legend("bottomright", legend = c("train","test"),
col = c("blue","red"), lty = c(2,1), lwd =1.5)  
   
 
 
 Report AUC from ROC for training and test data 
    # Train AUC
aucTr <- ROCR::performance(rocr.pred.mtt, measure = "auc")
  aucTr <- aucTr@y.values[[1]]
  print(aucTr)  
  ## [1] 0.9126842  
     # Test AUC
  aucTe <- ROCR::performance(rocr.pred.te.1, measure = "auc")
  aucTe <- aucTe@y.values[[1]]
  print(aucTe)  
  ## [1] 0.8651072  
 
 
 
 
 MULTIPLE REGRESSION WITH BACKWARDS ELIMINATION 
 
 ROUND 5: Model with all variables 
 Remove C_Gender as least significant in round 4 
  # fit binary logit model and store results 'm'
m <- glm(Health_Binary ~  Location + Urban + Animal_Career_BINARY 
         + C_Diet + Size2 + D_Sex + D_Neuter  + D_Diet + Therapeutic_Food + Visits 
         + Meds + bs(D_Age, degree=1,df=2), data = ml_train,family = binomial)
# view a summary of the model
summary(m)  
  ## 
## Call:
## glm(formula = Health_Binary ~ Location + Urban + Animal_Career_BINARY + 
##     C_Diet + Size2 + D_Sex + D_Neuter + D_Diet + Therapeutic_Food + 
##     Visits + Meds + bs(D_Age, degree = 1, df = 2), family = binomial, 
##     data = ml_train)
## 
## Coefficients:
##                                                          Estimate Std. Error
## (Intercept)                                              -5.75789    0.83331
## LocationOther European                                    0.96766    0.39352
## LocationNorth America                                     0.05720    0.56921
## LocationAustralia/New Zealand/Oceania                     0.95412    0.53528
## LocationOther                                             0.83271    0.66816
## UrbanYes                                                 -0.20419    0.29295
## Animal_Career_BINARYYes                                   0.45206    0.31224
## C_DietOmnivore reducing animal product consumption        0.03092    0.34884
## C_DietPescatarian (including fish but no other meats)    -1.30852    0.67937
## C_DietVegan (consuming no animal products)                0.05172    0.44799
## C_DietVegetarian (consuming plants, eggs and milk)        0.18108    0.43060
## Size2Toy                                                -13.64201  548.89871
## Size2Small                                               -0.37868    0.40992
## Size2Large                                                0.46388    0.31258
## Size2Giant                                                1.86861    0.55970
## D_SexMale                                                -0.28214    0.26721
## D_NeuterNeutered                                         -0.18042    0.42310
## D_DietMeat-based – raw                                    0.02326    0.32500
## D_DietVegan (consuming no animal products)               -0.59973    0.57256
## D_DietVegetarian (including eggs or milk, but not meat)  -0.54231    0.95034
## Therapeutic_FoodYes                                       0.62652    0.38250
## Visits1                                                  -0.84320    0.73737
## Visits2                                                  -0.58554    0.74055
## Visits3                                                  -0.24420    0.80132
## Visits3<                                                  1.41019    0.71611
## MedsYes                                                   2.52224    0.51546
## bs(D_Age, degree = 1, df = 2)1                            0.29297    0.56710
## bs(D_Age, degree = 1, df = 2)2                            2.95529    0.66886
##                                                         z value Pr(>|z|)    
## (Intercept)                                              -6.910 4.86e-12 ***
## LocationOther European                                    2.459 0.013932 *  
## LocationNorth America                                     0.100 0.919960    
## LocationAustralia/New Zealand/Oceania                     1.782 0.074673 .  
## LocationOther                                             1.246 0.212661    
## UrbanYes                                                 -0.697 0.485798    
## Animal_Career_BINARYYes                                   1.448 0.147676    
## C_DietOmnivore reducing animal product consumption        0.089 0.929370    
## C_DietPescatarian (including fish but no other meats)    -1.926 0.054096 .  
## C_DietVegan (consuming no animal products)                0.115 0.908090    
## C_DietVegetarian (consuming plants, eggs and milk)        0.421 0.674097    
## Size2Toy                                                 -0.025 0.980172    
## Size2Small                                               -0.924 0.355588    
## Size2Large                                                1.484 0.137802    
## Size2Giant                                                3.339 0.000842 ***
## D_SexMale                                                -1.056 0.291013    
## D_NeuterNeutered                                         -0.426 0.669803    
## D_DietMeat-based – raw                                    0.072 0.942934    
## D_DietVegan (consuming no animal products)               -1.047 0.294890    
## D_DietVegetarian (including eggs or milk, but not meat)  -0.571 0.568239    
## Therapeutic_FoodYes                                       1.638 0.101426    
## Visits1                                                  -1.144 0.252817    
## Visits2                                                  -0.791 0.429130    
## Visits3                                                  -0.305 0.760558    
## Visits3<                                                  1.969 0.048928 *  
## MedsYes                                                   4.893 9.92e-07 ***
## bs(D_Age, degree = 1, df = 2)1                            0.517 0.605428    
## bs(D_Age, degree = 1, df = 2)2                            4.418 9.94e-06 ***
## ---
## Signif. codes:  0 '***' 0.001 '**' 0.01 '*' 0.05 '.' 0.1 ' ' 1
## 
## (Dispersion parameter for binomial family taken to be 1)
## 
##     Null deviance: 652.97  on 1657  degrees of freedom
## Residual deviance: 422.41  on 1630  degrees of freedom
## AIC: 478.41
## 
## Number of Fisher Scoring iterations: 16  
  # test model fit
with(m, null.deviance - deviance)  
  ## [1] 230.5559  
  with(m, df.null - df.residual)  
  ## [1] 27  
  with(m, pchisq(null.deviance - deviance, df.null - df.residual, lower.tail = FALSE))  
  ## [1] 3.337834e-34  
  BIC(m)  
  ## [1] 629.9846  
  # Hosmer-Lemeshow Goodness-of-Fit Test
hltest(m)  
  ## 
##    The Hosmer-Lemeshow goodness-of-fit test
## 
##  Group Size Observed   Expected
##      1  166        0  0.1160347
##      2  168        0  0.2678174
##      3  167        1  0.4048365
##      4  166        0  0.5909860
##      5  166        0  0.9668381
##      6  167        3  1.8337046
##      7  166        4  3.4421714
##      8  166        3  6.4399985
##      9  166       21 14.8045833
##     10  160       50 53.1330309
## 
##          Statistic =  8.7042 
## degrees of freedom =  8 
##            p-value =  0.36786  
  ## CIs using profiled log-likelihood
confint(m, level=0.99)  
  ## Waiting for profiling to be done...  
  ##                                                                 0.5 %
## (Intercept)                                               -8.22350704
## LocationOther European                                    -0.07618084
## LocationNorth America                                     -1.61247394
## LocationAustralia/New Zealand/Oceania                     -0.52728535
## LocationOther                                             -1.10372370
## UrbanYes                                                  -0.98063633
## Animal_Career_BINARYYes                                   -0.37733090
## C_DietOmnivore reducing animal product consumption        -0.88977421
## C_DietPescatarian (including fish but no other meats)     -3.43969522
## C_DietVegan (consuming no animal products)                -1.14601416
## C_DietVegetarian (consuming plants, eggs and milk)        -0.98401345
## Size2Toy                                                -239.68010357
## Size2Small                                                -1.48839926
## Size2Large                                                -0.33368690
## Size2Giant                                                 0.35380198
## D_SexMale                                                 -0.97658406
## D_NeuterNeutered                                          -1.23007062
## D_DietMeat-based – raw                                    -0.84357371
## D_DietVegan (consuming no animal products)                -2.16927822
## D_DietVegetarian (including eggs or milk, but not meat)   -3.52434510
## Therapeutic_FoodYes                                       -0.39577323
## Visits1                                                   -2.64975771
## Visits2                                                   -2.36906773
## Visits3                                                   -2.24304959
## Visits3<                                                  -0.27391077
## MedsYes                                                    1.31827243
## bs(D_Age, degree = 1, df = 2)1                            -1.12609661
## bs(D_Age, degree = 1, df = 2)2                             1.25827140
##                                                             99.5 %
## (Intercept)                                             -3.8248587
## LocationOther European                                   1.9676429
## LocationNorth America                                    1.3962827
## LocationAustralia/New Zealand/Oceania                    2.2680546
## LocationOther                                            2.4467683
## UrbanYes                                                 0.5371956
## Animal_Career_BINARYYes                                  1.2428170
## C_DietOmnivore reducing animal product consumption       0.9218021
## C_DietPescatarian (including fish but no other meats)    0.2390468
## C_DietVegan (consuming no animal products)               1.1789208
## C_DietVegetarian (consuming plants, eggs and milk)       1.2581756
## Size2Toy                                                13.4474431
## Size2Small                                               0.6474196
## Size2Large                                               1.2878240
## Size2Giant                                               3.2776812
## D_SexMale                                                0.4072615
## D_NeuterNeutered                                         0.9776485
## D_DietMeat-based – raw                                   0.8440035
## D_DietVegan (consuming no animal products)               0.8319969
## D_DietVegetarian (including eggs or milk, but not meat)  1.6427515
## Therapeutic_FoodYes                                      1.5893320
## Visits1                                                  1.3667639
## Visits2                                                  1.6387302
## Visits3                                                  2.0816424
## Visits3<                                                 3.5988061
## MedsYes                                                  4.0259020
## bs(D_Age, degree = 1, df = 2)1                           1.8231178
## bs(D_Age, degree = 1, df = 2)2                           4.7283894  
  ## CIs using standard errors
confint.default(m, level=0.99)  
  ##                                                                 0.5 %
## (Intercept)                                             -7.904350e+00
## LocationOther European                                  -4.597468e-02
## LocationNorth America                                   -1.408990e+00
## LocationAustralia/New Zealand/Oceania                   -4.246692e-01
## LocationOther                                           -8.883454e-01
## UrbanYes                                                -9.587890e-01
## Animal_Career_BINARYYes                                 -3.522239e-01
## C_DietOmnivore reducing animal product consumption      -8.676346e-01
## C_DietPescatarian (including fish but no other meats)   -3.058465e+00
## C_DietVegan (consuming no animal products)              -1.102220e+00
## C_DietVegetarian (consuming plants, eggs and milk)      -9.280628e-01
## Size2Toy                                                -1.427511e+03
## Size2Small                                              -1.434559e+00
## Size2Large                                              -3.412800e-01
## Size2Giant                                               4.269243e-01
## D_SexMale                                               -9.704240e-01
## D_NeuterNeutered                                        -1.270249e+00
## D_DietMeat-based – raw                                  -8.138772e-01
## D_DietVegan (consuming no animal products)              -2.074544e+00
## D_DietVegetarian (including eggs or milk, but not meat) -2.990232e+00
## Therapeutic_FoodYes                                     -3.587246e-01
## Visits1                                                 -2.742533e+00
## Visits2                                                 -2.493064e+00
## Visits3                                                 -2.308272e+00
## Visits3<                                                -4.343987e-01
## MedsYes                                                  1.194515e+00
## bs(D_Age, degree = 1, df = 2)1                          -1.167778e+00
## bs(D_Age, degree = 1, df = 2)2                           1.232413e+00
##                                                               99.5 %
## (Intercept)                                               -3.6114338
## LocationOther European                                     1.9812961
## LocationNorth America                                      1.5233825
## LocationAustralia/New Zealand/Oceania                      2.3329120
## LocationOther                                              2.5537675
## UrbanYes                                                   0.5504077
## Animal_Career_BINARYYes                                    1.2563489
## C_DietOmnivore reducing animal product consumption         0.9294759
## C_DietPescatarian (including fish but no other meats)      0.4414279
## C_DietVegan (consuming no animal products)                 1.2056581
## C_DietVegetarian (consuming plants, eggs and milk)         1.2902208
## Size2Toy                                                1400.2273833
## Size2Small                                                 0.6771930
## Size2Large                                                 1.2690467
## Size2Giant                                                 3.3103036
## D_SexMale                                                  0.4061354
## D_NeuterNeutered                                           0.9094142
## D_DietMeat-based – raw                                     0.8604060
## D_DietVegan (consuming no animal products)                 0.8750857
## D_DietVegetarian (including eggs or milk, but not meat)    1.9056116
## Therapeutic_FoodYes                                        1.6117634
## Visits1                                                    1.0561276
## Visits2                                                    1.3219885
## Visits3                                                    1.8198678
## Visits3<                                                   3.2547692
## MedsYes                                                    3.8499741
## bs(D_Age, degree = 1, df = 2)1                             1.7537165
## bs(D_Age, degree = 1, df = 2)2                             4.6781755  
  # Wald test
wald.test(b = coef(m), Sigma = vcov(m), Terms = 2)  
  ## Wald test:
## ----------
## 
## Chi-squared test:
## X2 = 6.0, df = 1, P(> X2) = 0.014  
  ## odds ratios and 95% CI
exp(cbind(OR = coef(m), confint(m, level=0.99)))  
  ## Waiting for profiling to be done...  
  ##                                                                   OR
## (Intercept)                                             3.157761e-03
## LocationOther European                                  2.631781e+00
## LocationNorth America                                   1.058864e+00
## LocationAustralia/New Zealand/Oceania                   2.596388e+00
## LocationOther                                           2.299544e+00
## UrbanYes                                                8.153069e-01
## Animal_Career_BINARYYes                                 1.571550e+00
## C_DietOmnivore reducing animal product consumption      1.031404e+00
## C_DietPescatarian (including fish but no other meats)   2.702200e-01
## C_DietVegan (consuming no animal products)              1.053080e+00
## C_DietVegetarian (consuming plants, eggs and milk)      1.198510e+00
## Size2Toy                                                1.189462e-06
## Size2Small                                              6.847625e-01
## Size2Large                                              1.590237e+00
## Size2Giant                                              6.479309e+00
## D_SexMale                                               7.541649e-01
## D_NeuterNeutered                                        8.349216e-01
## D_DietMeat-based – raw                                  1.023537e+00
## D_DietVegan (consuming no animal products)              5.489603e-01
## D_DietVegetarian (including eggs or milk, but not meat) 5.814035e-01
## Therapeutic_FoodYes                                     1.871087e+00
## Visits1                                                 4.303301e-01
## Visits2                                                 5.568062e-01
## Visits3                                                 7.833294e-01
## Visits3<                                                4.096714e+00
## MedsYes                                                 1.245652e+01
## bs(D_Age, degree = 1, df = 2)1                          1.340401e+00
## bs(D_Age, degree = 1, df = 2)2                          1.920737e+01
##                                                                 0.5 %
## (Intercept)                                              2.682726e-04
## LocationOther European                                   9.266486e-01
## LocationNorth America                                    1.993937e-01
## LocationAustralia/New Zealand/Oceania                    5.902050e-01
## LocationOther                                            3.316339e-01
## UrbanYes                                                 3.750724e-01
## Animal_Career_BINARYYes                                  6.856891e-01
## C_DietOmnivore reducing animal product consumption       4.107485e-01
## C_DietPescatarian (including fish but no other meats)    3.207446e-02
## C_DietVegan (consuming no animal products)               3.179014e-01
## C_DietVegetarian (consuming plants, eggs and milk)       3.738078e-01
## Size2Toy                                                8.095685e-105
## Size2Small                                               2.257337e-01
## Size2Large                                               7.162780e-01
## Size2Giant                                               1.424473e+00
## D_SexMale                                                3.765953e-01
## D_NeuterNeutered                                         2.922719e-01
## D_DietMeat-based – raw                                   4.301705e-01
## D_DietVegan (consuming no animal products)               1.142601e-01
## D_DietVegetarian (including eggs or milk, but not meat)  2.947110e-02
## Therapeutic_FoodYes                                      6.731593e-01
## Visits1                                                  7.066833e-02
## Visits2                                                  9.356792e-02
## Visits3                                                  1.061343e-01
## Visits3<                                                 7.603999e-01
## MedsYes                                                  3.736960e+00
## bs(D_Age, degree = 1, df = 2)1                           3.242966e-01
## bs(D_Age, degree = 1, df = 2)2                           3.519333e+00
##                                                               99.5 %
## (Intercept)                                             2.182152e-02
## LocationOther European                                  7.153795e+00
## LocationNorth America                                   4.040154e+00
## LocationAustralia/New Zealand/Oceania                   9.660589e+00
## LocationOther                                           1.155096e+01
## UrbanYes                                                1.711201e+00
## Animal_Career_BINARYYes                                 3.465361e+00
## C_DietOmnivore reducing animal product consumption      2.513816e+00
## C_DietPescatarian (including fish but no other meats)   1.270038e+00
## C_DietVegan (consuming no animal products)              3.250864e+00
## C_DietVegetarian (consuming plants, eggs and milk)      3.518995e+00
## Size2Toy                                                6.920705e+05
## Size2Small                                              1.910604e+00
## Size2Large                                              3.624890e+00
## Size2Giant                                              2.651422e+01
## D_SexMale                                               1.502697e+00
## D_NeuterNeutered                                        2.658198e+00
## D_DietMeat-based – raw                                  2.325659e+00
## D_DietVegan (consuming no animal products)              2.297903e+00
## D_DietVegetarian (including eggs or milk, but not meat) 5.169373e+00
## Therapeutic_FoodYes                                     4.900475e+00
## Visits1                                                 3.922636e+00
## Visits2                                                 5.148628e+00
## Visits3                                                 8.017627e+00
## Visits3<                                                3.655457e+01
## MedsYes                                                 5.603083e+01
## bs(D_Age, degree = 1, df = 2)1                          6.191131e+00
## bs(D_Age, degree = 1, df = 2)2                          1.131132e+02  
 
 Calculate Nagelkerke R^2 
  NagelkerkeR2(m)  
  ## $N
## [1] 1658
## 
## $R2
## [1] 0.3988  
 
 
 check assumptions of model 
 
 Cook’s distance 
  plot(m, which = 4, id.n = 3)  
   
 
 
 Extract model results and display data for top 3 values using Cook’s
distance 
  model.data <- augment(m) %>% 
  mutate(index = 1:n()) 
model.data %>% top_n(3, .cooksd)  
 
 
 
 
 
 plot standardised residuals 
  ggplot(model.data, aes(index, .std.resid)) + 
  geom_point(aes(color = Health_Binary), alpha = .5) +
  theme_bw()  
   
 
 
 Filter potential influential data points with abs(.std.res) >
3: 
  model.data %>% 
  filter(abs(.std.resid) > 3)  
 
 
 
 
 
 check for multicollinearity 
  car::vif(m)  
  ##                                   GVIF Df GVIF^(1/(2*Df))
## Location                      1.660241  4        1.065421
## Urban                         1.163469  1        1.078642
## Animal_Career_BINARY          1.098620  1        1.048151
## C_Diet                        1.922319  4        1.085121
## Size2                         1.387478  4        1.041785
## D_Sex                         1.043386  1        1.021463
## D_Neuter                      1.275409  1        1.129340
## D_Diet                        1.895670  3        1.112484
## Therapeutic_Food              1.150482  1        1.072605
## Visits                        1.556523  4        1.056865
## Meds                          1.391978  1        1.179821
## bs(D_Age, degree = 1, df = 2) 1.323918  2        1.072668  
 
 
 
 Create ROCR for training and test data 
  ## training data
pred.mtt = predict(m, type = "response") #repeat risk predictions from model m
rocr.pred.mtt = ROCR::prediction(pred.mtt, labels = ml_train$Health_Binary) #ROCR prediction object
roc.perf.mtt = ROCR::performance(rocr.pred.mtt, measure = "tpr", x.measure = "fpr") # #ROCR performance object
plot(roc.perf.mtt, col = "blue")


pred.te.1 = predict(m, newdata = ml_test, type = "response") #.te = "test"
rocr.pred.te.1 = ROCR::prediction(pred.te.1, labels = ml_test$Health_Binary)
roc.perf.te.1 = ROCR::performance(rocr.pred.te.1, measure = "tpr", x.measure = "fpr")
plot(roc.perf.te.1, col = "red", add = T)

abline(a = 0, b = 1, lty = 2) #diagonal for random assignment
legend("bottomright", legend = c("train","test"),
col = c("blue","red"), lty = c(2,1), lwd =1.5)  
   
 
 
 Report AUC from ROC for training and test data 
    # Train AUC
aucTr <- ROCR::performance(rocr.pred.mtt, measure = "auc")
  aucTr <- aucTr@y.values[[1]]
  print(aucTr)  
  ## [1] 0.913783  
     # Test AUC
  aucTe <- ROCR::performance(rocr.pred.te.1, measure = "auc")
  aucTe <- aucTe@y.values[[1]]
  print(aucTe)  
  ## [1] 0.8655945  
 
 
 
 
 MULTIPLE REGRESSION WITH BACKWARDS ELIMINATION 
 
 ROUND 6: Model with all variables 
 Remove Urban as least significant in round 5 
  # fit binary logit model and store results 'm'
m <- glm(Health_Binary ~  Location + Animal_Career_BINARY 
         + C_Diet + Size2 + D_Sex + D_Neuter  + D_Diet + Therapeutic_Food + Visits 
         + Meds + bs(D_Age, degree=1,df=2), data = ml_train,family = binomial)
# view a summary of the model
summary(m)  
  ## 
## Call:
## glm(formula = Health_Binary ~ Location + Animal_Career_BINARY + 
##     C_Diet + Size2 + D_Sex + D_Neuter + D_Diet + Therapeutic_Food + 
##     Visits + Meds + bs(D_Age, degree = 1, df = 2), family = binomial, 
##     data = ml_train)
## 
## Coefficients:
##                                                           Estimate Std. Error
## (Intercept)                                              -5.775255   0.831374
## LocationOther European                                    0.957324   0.394215
## LocationNorth America                                     0.022875   0.569486
## LocationAustralia/New Zealand/Oceania                     0.905131   0.531078
## LocationOther                                             0.719560   0.648804
## Animal_Career_BINARYYes                                   0.457532   0.312359
## C_DietOmnivore reducing animal product consumption        0.020511   0.348383
## C_DietPescatarian (including fish but no other meats)    -1.311014   0.679998
## C_DietVegan (consuming no animal products)               -0.003904   0.441985
## C_DietVegetarian (consuming plants, eggs and milk)        0.178167   0.429915
## Size2Toy                                                -13.682475 547.214227
## Size2Small                                               -0.382995   0.409556
## Size2Large                                                0.462344   0.312824
## Size2Giant                                                1.871964   0.561256
## D_SexMale                                                -0.293148   0.266696
## D_NeuterNeutered                                         -0.190017   0.423336
## D_DietMeat-based – raw                                    0.038760   0.323830
## D_DietVegan (consuming no animal products)               -0.566848   0.569870
## D_DietVegetarian (including eggs or milk, but not meat)  -0.566219   0.955291
## Therapeutic_FoodYes                                       0.639489   0.382458
## Visits1                                                  -0.853210   0.737673
## Visits2                                                  -0.599304   0.741288
## Visits3                                                  -0.253104   0.801214
## Visits3<                                                  1.402458   0.716705
## MedsYes                                                   2.504395   0.514982
## bs(D_Age, degree = 1, df = 2)1                            0.288626   0.567404
## bs(D_Age, degree = 1, df = 2)2                            2.959839   0.669369
##                                                         z value Pr(>|z|)    
## (Intercept)                                              -6.947 3.74e-12 ***
## LocationOther European                                    2.428 0.015164 *  
## LocationNorth America                                     0.040 0.967959    
## LocationAustralia/New Zealand/Oceania                     1.704 0.088320 .  
## LocationOther                                             1.109 0.267406    
## Animal_Career_BINARYYes                                   1.465 0.142985    
## C_DietOmnivore reducing animal product consumption        0.059 0.953053    
## C_DietPescatarian (including fish but no other meats)    -1.928 0.053859 .  
## C_DietVegan (consuming no animal products)               -0.009 0.992952    
## C_DietVegetarian (consuming plants, eggs and milk)        0.414 0.678563    
## Size2Toy                                                 -0.025 0.980052    
## Size2Small                                               -0.935 0.349713    
## Size2Large                                                1.478 0.139416    
## Size2Giant                                                3.335 0.000852 ***
## D_SexMale                                                -1.099 0.271687    
## D_NeuterNeutered                                         -0.449 0.653535    
## D_DietMeat-based – raw                                    0.120 0.904727    
## D_DietVegan (consuming no animal products)               -0.995 0.319884    
## D_DietVegetarian (including eggs or milk, but not meat)  -0.593 0.553370    
## Therapeutic_FoodYes                                       1.672 0.094515 .  
## Visits1                                                  -1.157 0.247426    
## Visits2                                                  -0.808 0.418824    
## Visits3                                                  -0.316 0.752078    
## Visits3<                                                  1.957 0.050369 .  
## MedsYes                                                   4.863 1.16e-06 ***
## bs(D_Age, degree = 1, df = 2)1                            0.509 0.610977    
## bs(D_Age, degree = 1, df = 2)2                            4.422 9.79e-06 ***
## ---
## Signif. codes:  0 '***' 0.001 '**' 0.01 '*' 0.05 '.' 0.1 ' ' 1
## 
## (Dispersion parameter for binomial family taken to be 1)
## 
##     Null deviance: 652.97  on 1657  degrees of freedom
## Residual deviance: 422.90  on 1631  degrees of freedom
## AIC: 476.9
## 
## Number of Fisher Scoring iterations: 16  
  # test model fit
with(m, null.deviance - deviance)  
  ## [1] 230.0643  
  with(m, df.null - df.residual)  
  ## [1] 26  
  with(m, pchisq(null.deviance - deviance, df.null - df.residual, lower.tail = FALSE))  
  ## [1] 1.377317e-34  
  BIC(m)  
  ## [1] 623.0629  
  # Hosmer-Lemeshow Goodness-of-Fit Test
hltest(m)  
  ## 
##    The Hosmer-Lemeshow goodness-of-fit test
## 
##  Group Size Observed   Expected
##      1  166        0  0.1171745
##      2  167        0  0.2704688
##      3  166        1  0.4093524
##      4  166        0  0.5975685
##      5  166        0  0.9697457
##      6  166        4  1.8120244
##      7  166        0  3.4241603
##      8  166        6  6.3120978
##      9  166       20 14.7044358
##     10  163       51 53.3829731
## 
##          Statistic =  11.25173 
## degrees of freedom =  8 
##            p-value =  0.18784  
  ## CIs using profiled log-likelihood
confint(m, level=0.99)  
  ## Waiting for profiling to be done...  
  ##                                                                 0.5 %
## (Intercept)                                               -8.23716723
## LocationOther European                                    -0.08796783
## LocationNorth America                                     -1.64691708
## LocationAustralia/New Zealand/Oceania                     -0.56833629
## LocationOther                                             -1.17843910
## Animal_Career_BINARYYes                                   -0.37225716
## C_DietOmnivore reducing animal product consumption        -0.89924584
## C_DietPescatarian (including fish but no other meats)     -3.44353872
## C_DietVegan (consuming no animal products)                -1.18594978
## C_DietVegetarian (consuming plants, eggs and milk)        -0.98610013
## Size2Toy                                                -258.16353879
## Size2Small                                                -1.49176144
## Size2Large                                                -0.33573736
## Size2Giant                                                 0.35230035
## D_SexMale                                                 -0.98647250
## D_NeuterNeutered                                          -1.24036500
## D_DietMeat-based – raw                                    -0.82476074
## D_DietVegan (consuming no animal products)                -2.12956734
## D_DietVegetarian (including eggs or milk, but not meat)   -3.56026155
## Therapeutic_FoodYes                                       -0.38266444
## Visits1                                                   -2.66093067
## Visits2                                                   -2.38487765
## Visits3                                                   -2.25123832
## Visits3<                                                  -0.28355587
## MedsYes                                                    1.30292659
## bs(D_Age, degree = 1, df = 2)1                            -1.13114433
## bs(D_Age, degree = 1, df = 2)2                             1.26176721
##                                                             99.5 %
## (Intercept)                                             -3.8481796
## LocationOther European                                   1.9592207
## LocationNorth America                                    1.3626523
## LocationAustralia/New Zealand/Oceania                    2.2043738
## LocationOther                                            2.2775192
## Animal_Career_BINARYYes                                  1.2485058
## C_DietOmnivore reducing animal product consumption       0.9099513
## C_DietPescatarian (including fish but no other meats)    0.2378042
## C_DietVegan (consuming no animal products)               1.1081802
## C_DietVegetarian (consuming plants, eggs and milk)       1.2528096
## Size2Toy                                                 9.8327345
## Size2Small                                               0.6422451
## Size2Large                                               1.2870681
## Size2Giant                                               3.2838488
## D_SexMale                                                0.3947060
## D_NeuterNeutered                                         0.9680699
## D_DietMeat-based – raw                                   0.8568648
## D_DietVegan (consuming no animal products)               0.8583881
## D_DietVegetarian (including eggs or milk, but not meat)  1.6309798
## Therapeutic_FoodYes                                      1.6021126
## Visits1                                                  1.3571326
## Visits2                                                  1.6262878
## Visits3                                                  2.0726563
## Visits3<                                                 3.5919847
## MedsYes                                                  4.0083889
## bs(D_Age, degree = 1, df = 2)1                           1.8198184
## bs(D_Age, degree = 1, df = 2)2                           4.7343696  
  ## CIs using standard errors
confint.default(m, level=0.99)  
  ##                                                                 0.5 %
## (Intercept)                                             -7.916734e+00
## LocationOther European                                  -5.810565e-02
## LocationNorth America                                   -1.444024e+00
## LocationAustralia/New Zealand/Oceania                   -4.628343e-01
## LocationOther                                           -9.516493e-01
## Animal_Career_BINARYYes                                 -3.470513e-01
## C_DietOmnivore reducing animal product consumption      -8.768651e-01
## C_DietPescatarian (including fish but no other meats)   -3.062573e+00
## C_DietVegan (consuming no animal products)              -1.142383e+00
## C_DietVegetarian (consuming plants, eggs and milk)      -9.292206e-01
## Size2Toy                                                -1.423213e+03
## Size2Small                                              -1.437940e+00
## Size2Large                                              -3.434368e-01
## Size2Giant                                               4.262643e-01
## D_SexMale                                               -9.801116e-01
## D_NeuterNeutered                                        -1.280458e+00
## D_DietMeat-based – raw                                  -7.953709e-01
## D_DietVegan (consuming no animal products)              -2.034736e+00
## D_DietVegetarian (including eggs or milk, but not meat) -3.026886e+00
## Therapeutic_FoodYes                                     -3.456585e-01
## Visits1                                                 -2.753332e+00
## Visits2                                                 -2.508735e+00
## Visits3                                                 -2.316895e+00
## Visits3<                                                -4.436510e-01
## MedsYes                                                  1.177890e+00
## bs(D_Age, degree = 1, df = 2)1                          -1.172908e+00
## bs(D_Age, degree = 1, df = 2)2                           1.235658e+00
##                                                               99.5 %
## (Intercept)                                               -3.6337764
## LocationOther European                                     1.9727536
## LocationNorth America                                      1.4897742
## LocationAustralia/New Zealand/Oceania                      2.2730957
## LocationOther                                              2.3907694
## Animal_Career_BINARYYes                                    1.2621158
## C_DietOmnivore reducing animal product consumption         0.9178864
## C_DietPescatarian (including fish but no other meats)      0.4405444
## C_DietVegan (consuming no animal products)                 1.1345743
## C_DietVegetarian (consuming plants, eggs and milk)         1.2855551
## Size2Toy                                                1395.8479676
## Size2Small                                                 0.6719507
## Size2Large                                                 1.2681254
## Size2Giant                                                 3.3176634
## D_SexMale                                                  0.3938148
## D_NeuterNeutered                                           0.9004237
## D_DietMeat-based – raw                                     0.8728909
## D_DietVegan (consuming no animal products)                 0.9010400
## D_DietVegetarian (including eggs or milk, but not meat)    1.8944487
## Therapeutic_FoodYes                                        1.6246359
## Visits1                                                    1.0469105
## Visits2                                                    1.3101274
## Visits3                                                    1.8106866
## Visits3<                                                   3.2485676
## MedsYes                                                    3.8309007
## bs(D_Age, degree = 1, df = 2)1                             1.7501611
## bs(D_Age, degree = 1, df = 2)2                             4.6840202  
  # Wald test
wald.test(b = coef(m), Sigma = vcov(m), Terms = 2)  
  ## Wald test:
## ----------
## 
## Chi-squared test:
## X2 = 5.9, df = 1, P(> X2) = 0.015  
  ## odds ratios and 95% CI
exp(cbind(OR = coef(m), confint(m, level=0.99)))  
  ## Waiting for profiling to be done...  
  ##                                                                   OR
## (Intercept)                                             3.103406e-03
## LocationOther European                                  2.604717e+00
## LocationNorth America                                   1.023139e+00
## LocationAustralia/New Zealand/Oceania                   2.472255e+00
## LocationOther                                           2.053530e+00
## Animal_Career_BINARYYes                                 1.580170e+00
## C_DietOmnivore reducing animal product consumption      1.020722e+00
## C_DietPescatarian (including fish but no other meats)   2.695465e-01
## C_DietVegan (consuming no animal products)              9.961034e-01
## C_DietVegetarian (consuming plants, eggs and milk)      1.195025e+00
## Size2Toy                                                1.142291e-06
## Size2Small                                              6.818165e-01
## Size2Large                                              1.587792e+00
## Size2Giant                                              6.501051e+00
## D_SexMale                                               7.459114e-01
## D_NeuterNeutered                                        8.269449e-01
## D_DietMeat-based – raw                                  1.039521e+00
## D_DietVegan (consuming no animal products)              5.673109e-01
## D_DietVegetarian (including eggs or milk, but not meat) 5.676679e-01
## Therapeutic_FoodYes                                     1.895511e+00
## Visits1                                                 4.260449e-01
## Visits2                                                 5.491939e-01
## Visits3                                                 7.763869e-01
## Visits3<                                                4.065181e+00
## MedsYes                                                 1.223616e+01
## bs(D_Age, degree = 1, df = 2)1                          1.334593e+00
## bs(D_Age, degree = 1, df = 2)2                          1.929487e+01
##                                                                 0.5 %
## (Intercept)                                              2.646328e-04
## LocationOther European                                   9.157903e-01
## LocationNorth America                                    1.926429e-01
## LocationAustralia/New Zealand/Oceania                    5.664671e-01
## LocationOther                                            3.077587e-01
## Animal_Career_BINARYYes                                  6.891770e-01
## C_DietOmnivore reducing animal product consumption       4.068764e-01
## C_DietPescatarian (including fish but no other meats)    3.195142e-02
## C_DietVegan (consuming no animal products)               3.054559e-01
## C_DietVegetarian (consuming plants, eggs and milk)       3.730286e-01
## Size2Toy                                                7.603257e-113
[truncated: 397,022 more chars]
