# Supplementary material for: Variables associated with owner perceptions of the health of their dog: Further analysis of data from a large international survey
Source: PLoS One. 2024 May 15;19(5):e0280173. doi: 10.1371/journal.pone.0280173 (PMC11095744; doi:10.1371/journal.pone.0280173)
Supplement: S10 File — (HTML) [file pone.0280173.s024.html]

R stats for binary logistic regression on SERIOUS ILLNESS outcome variiable for the all-owner dataset


Code 

- Show All Code
- Hide All Code
- Download Rmd

# R stats for binary logistic regression on SERIOUS ILLNESS outcome variiable for the all-owner dataset

#### Alex German

#### 7 December 2023

# Create data frame for analysis

### NB need to run “Read\_data\_102.Rtm” first to create dataset

## Chi squared test of Health\_Binary and Primary\_Decision\_Maker


```
table(ml$Health_Binary, ml$Primary_Decision_Maker)
chisq.test(ml$Health_Binary, ml$Primary_Decision_Maker, correct=FALSE)
```

# BINARY LOGISTIC REGRESSION ON SIGNIFICANT OR SERIOUS ILLNESS

## CHECK EFFECT OF OWNER CHARACTERISTICS - simple binary logistic regression

## DECISION MAKER Binary regression for Any\_Health\_Problem


```
# fit binary logit model and store results 'm'
m <- glm(Health_Binary ~ Primary_Decision_Maker, data = ml,family = binomial)
# view a summary of the model
summary(m)
# test model fit
with(m, null.deviance - deviance)
with(m, df.null - df.residual)
with(m, pchisq(null.deviance - deviance, df.null - df.residual, lower.tail = FALSE))
BIC(m)
## CIs using profiled log-likelihood
confint(m, level=0.99)
## CIs using standard errors
confint.default(m, level=0.99)
# Wald test
wald.test(b = coef(m), Sigma = vcov(m), Terms = 2)
## odds ratios and 95% CI
exp(cbind(OR = coef(m), confint(m, level=0.99)))
```


### Create ROCR from data


```
## training data
pred.mtt = predict(m, type = "response") #repeat risk predictions from model m
rocr.pred.mtt = ROCR::prediction(pred.mtt, labels = ml$Health_Binary) #ROCR prediction object
roc.perf.mtt = ROCR::performance(rocr.pred.mtt, measure = "tpr", x.measure = "fpr") # #ROCR performance object
plot(roc.perf.mtt, col = "blue")
abline(a = 0, b = 1, lty = 2) #diagonal for random assignment
```

### Report AUC from ROC for training and test data


```
  # Train AUC
auc <- ROCR::performance(rocr.pred.mtt, measure = "auc")
  auc <- auc@y.values[[1]]
  print(auc)
```

### Calculate Nagelkerke R^2


```
NagelkerkeR2(m)
```

### check assumptions of model

#### Cook’s distance


```
plot(m, which = 4, id.n = 3)
```

#### Extract model results and display data for top 3 values using Cook’s distance


```
model.data <- augment(m) %>% 
  mutate(index = 1:n()) 
model.data %>% top_n(3, .cooksd)
```

#### plot standardised residuals


```
ggplot(model.data, aes(index, .std.resid)) + 
  geom_point(aes(color = Health_Binary), alpha = .5) +
  theme_bw()
```

#### Filter potential influential data points with abs(.std.res) > 3:


```
model.data %>% 
  filter(abs(.std.resid) > 3)
```

## CLIENT DIET binary regression ON SIGNIFICANT OR SERIOUS ILLNESS


```
# fit binary logit model and store results 'm'
m <- glm(Health_Binary ~ C_Diet, data = ml,family = binomial)
# view a summary of the model
summary(m)
# test model fit
with(m, null.deviance - deviance)
with(m, df.null - df.residual)
with(m, pchisq(null.deviance - deviance, df.null - df.residual, lower.tail = FALSE))
BIC(m)
## CIs using profiled log-likelihood
confint(m, level=0.99)
## CIs using standard errors
confint.default(m, level=0.99)
# Wald test
wald.test(b = coef(m), Sigma = vcov(m), Terms = 2)
## odds ratios and 95% CI
exp(cbind(OR = coef(m), confint(m, level=0.99)))
```


### Create ROCR from data


```
## training data
pred.mtt = predict(m, type = "response") #repeat risk predictions from model m
rocr.pred.mtt = ROCR::prediction(pred.mtt, labels = ml$Health_Binary) #ROCR prediction object
roc.perf.mtt = ROCR::performance(rocr.pred.mtt, measure = "tpr", x.measure = "fpr") # #ROCR performance object
plot(roc.perf.mtt, col = "blue")
abline(a = 0, b = 1, lty = 2) #diagonal for random assignment
```

### Report AUC from ROC for training and test data


```
  # Train AUC
auc <- ROCR::performance(rocr.pred.mtt, measure = "auc")
  auc <- auc@y.values[[1]]
  print(auc)
```

### Calculate Nagelkerke R^2


```
NagelkerkeR2(m)
```

### check assumptions of model

#### Cook’s distance


```
plot(m, which = 4, id.n = 3)
```

#### Extract model results and display data for top 3 values using Cook’s distance


```
model.data <- augment(m) %>% 
  mutate(index = 1:n()) 
model.data %>% top_n(3, .cooksd)
```

#### plot standardised residuals


```
ggplot(model.data, aes(index, .std.resid)) + 
  geom_point(aes(color = Health_Binary), alpha = .5) +
  theme_bw()
```

#### Filter potential influential data points with abs(.std.res) > 3:


```
model.data %>% 
  filter(abs(.std.resid) > 3)
```

#### Calculate Nagelkerke R^2


```
NagelkerkeR2(m)
```

## CLIENT DIET VEGAN binary regression ON SIGNIFICANT OR SERIOUS ILLNESS


```
# fit binary logit model and store results 'm'
m <- glm(Health_Binary ~ C_Diet_Vegan, data = ml,family = binomial)
# view a summary of the model
summary(m)
# test model fit
with(m, null.deviance - deviance)
with(m, df.null - df.residual)
with(m, pchisq(null.deviance - deviance, df.null - df.residual, lower.tail = FALSE))
BIC(m)
## CIs using profiled log-likelihood
confint(m, level=0.99)
## CIs using standard errors
confint.default(m, level=0.99)
# Wald test
wald.test(b = coef(m), Sigma = vcov(m), Terms = 2)
## odds ratios and 95% CI
exp(cbind(OR = coef(m), confint(m, level=0.99)))
```


### Create ROCR from data


```
## training data
pred.mtt = predict(m, type = "response") #repeat risk predictions from model m
rocr.pred.mtt = ROCR::prediction(pred.mtt, labels = ml$Health_Binary) #ROCR prediction object
roc.perf.mtt = ROCR::performance(rocr.pred.mtt, measure = "tpr", x.measure = "fpr") # #ROCR performance object
plot(roc.perf.mtt, col = "blue")
abline(a = 0, b = 1, lty = 2) #diagonal for random assignment
```

### Report AUC from ROC for training and test data


```
  # Train AUC
auc <- ROCR::performance(rocr.pred.mtt, measure = "auc")
  auc <- auc@y.values[[1]]
  print(auc)
```

### Calculate Nagelkerke R^2


```
NagelkerkeR2(m)
```

### check assumptions of model

#### Cook’s distance


```
plot(m, which = 4, id.n = 3)
```

#### Extract model results and display data for top 3 values using Cook’s distance


```
model.data <- augment(m) %>% 
  mutate(index = 1:n()) 
model.data %>% top_n(3, .cooksd)
```

#### plot standardised residuals


```
ggplot(model.data, aes(index, .std.resid)) + 
  geom_point(aes(color = Health_Binary), alpha = .5) +
  theme_bw()
```

#### Filter potential influential data points with abs(.std.res) > 3:


```
model.data %>% 
  filter(abs(.std.resid) > 3)
```

#### Calculate Nagelkerke R^2


```
NagelkerkeR2(m)
```

## C\_Diet\_Vegan\_Veggie Binary logistic regression for HEALTH


```
# fit binary logit model and store results 'm'
m <- glm(Health_Binary ~ C_Diet_Vegan_Veggie, data = ml,family = binomial)
# view a summary of the model
summary(m)
# test model fit
with(m, null.deviance - deviance)
with(m, df.null - df.residual)
with(m, pchisq(null.deviance - deviance, df.null - df.residual, lower.tail = FALSE))
BIC(m)
## CIs using profiled log-likelihood
confint(m, level=0.99)
## CIs using standard errors
confint.default(m, level=0.99)
# Wald test
wald.test(b = coef(m), Sigma = vcov(m), Terms = 2)
## odds ratios and 95% CI
exp(cbind(OR = coef(m), confint(m, level=0.99)))
```


### Create ROCR from data


```
## training data
pred.mtt = predict(m, type = "response") #repeat risk predictions from model m
rocr.pred.mtt = ROCR::prediction(pred.mtt, labels = ml$Health_Binary) #ROCR prediction object
roc.perf.mtt = ROCR::performance(rocr.pred.mtt, measure = "tpr", x.measure = "fpr") # #ROCR performance object
plot(roc.perf.mtt, col = "blue")
abline(a = 0, b = 1, lty = 2) #diagonal for random assignment
```

### Report AUC from ROC for training and test data


```
  # Train AUC
auc <- ROCR::performance(rocr.pred.mtt, measure = "auc")
  auc <- auc@y.values[[1]]
  print(auc)
```

### Calculate Nagelkerke R^2


```
NagelkerkeR2(m)
```

### check assumptions of model

#### Cook’s distance


```
plot(m, which = 4, id.n = 3)
```

#### Extract model results and display data for top 3 values using Cook’s distance


```
model.data <- augment(m) %>% 
  mutate(index = 1:n()) 
model.data %>% top_n(3, .cooksd)
```

#### plot standardised residuals


```
ggplot(model.data, aes(index, .std.resid)) + 
  geom_point(aes(color = Health_Binary), alpha = .5) +
  theme_bw()
```

#### Filter potential influential data points with abs(.std.res) > 3:


```
model.data %>% 
  filter(abs(.std.resid) > 3)
```

## LOCATION binary logistic regression ON SIGNIFICANT OR SERIOUS ILLNESS


```
# fit binary logit model and store results 'm'
m <- glm(Health_Binary ~ Location, data = ml,family = binomial)
# view a summary of the model
summary(m)
# test model fit
with(m, null.deviance - deviance)
with(m, df.null - df.residual)
with(m, pchisq(null.deviance - deviance, df.null - df.residual, lower.tail = FALSE))
BIC(m)
# Hosmer-Lemeshow Goodness-of-Fit Test
glmtoolbox::hltest(m)
## CIs using profiled log-likelihood
confint(m, level=0.99)
## CIs using standard errors
confint.default(m, level=0.99)
# Wald test
wald.test(b = coef(m), Sigma = vcov(m), Terms = 2)
## odds ratios and 95% CI
exp(cbind(OR = coef(m), confint(m, level=0.99)))
```


### Create ROCR from data


```
## training data
pred.mtt = predict(m, type = "response") #repeat risk predictions from model m
rocr.pred.mtt = ROCR::prediction(pred.mtt, labels = ml$Health_Binary) #ROCR prediction object
roc.perf.mtt = ROCR::performance(rocr.pred.mtt, measure = "tpr", x.measure = "fpr") # #ROCR performance object
plot(roc.perf.mtt, col = "blue")
abline(a = 0, b = 1, lty = 2) #diagonal for random assignment
```

### Report AUC from ROC for training and test data


```
  # Train AUC
auc <- ROCR::performance(rocr.pred.mtt, measure = "auc")
  auc <- auc@y.values[[1]]
  print(auc)
```

### Calculate Nagelkerke R^2


```
NagelkerkeR2(m)
```

### check assumptions of model

#### Cook’s distance


```
plot(m, which = 4, id.n = 3)
```

#### Extract model results and display data for top 3 values using Cook’s distance


```
model.data <- augment(m) %>% 
  mutate(index = 1:n()) 
model.data %>% top_n(3, .cooksd)
```

#### plot standardised residuals


```
ggplot(model.data, aes(index, .std.resid)) + 
  geom_point(aes(color = Health_Binary), alpha = .5) +
  theme_bw()
```

#### Filter potential influential data points with abs(.std.res) > 3:


```
model.data %>% 
  filter(abs(.std.resid) > 3)
```

## SETTING binary logistic regression ON SIGNIFICANT OR SERIOUS ILLNESS


```
# fit binary logit model and store results 'm'
m <- glm(Health_Binary ~ setting, data = ml,family = binomial)
# view a summary of the model
summary(m)
# test model fit
with(m, null.deviance - deviance)
with(m, df.null - df.residual)
with(m, pchisq(null.deviance - deviance, df.null - df.residual, lower.tail = FALSE))
BIC(m)
# Hosmer-Lemeshow Goodness-of-Fit Test
glmtoolbox::hltest(m)
## CIs using profiled log-likelihood
confint(m, level=0.99)
## CIs using standard errors
confint.default(m, level=0.99)
# Wald test
wald.test(b = coef(m), Sigma = vcov(m), Terms = 2)
## odds ratios and 95% CI
exp(cbind(OR = coef(m), confint(m, level=0.99)))
```


### Create ROCR from data


```
## training data
pred.mtt = predict(m, type = "response") #repeat risk predictions from model m
rocr.pred.mtt = ROCR::prediction(pred.mtt, labels = ml$Health_Binary) #ROCR prediction object
roc.perf.mtt = ROCR::performance(rocr.pred.mtt, measure = "tpr", x.measure = "fpr") # #ROCR performance object
plot(roc.perf.mtt, col = "blue")
abline(a = 0, b = 1, lty = 2) #diagonal for random assignment
```

### Report AUC from ROC for training and test data


```
  # Train AUC
auc <- ROCR::performance(rocr.pred.mtt, measure = "auc")
  auc <- auc@y.values[[1]]
  print(auc)
```

### Calculate Nagelkerke R^2


```
NagelkerkeR2(m)
```

### check assumptions of model

#### Cook’s distance


```
plot(m, which = 4, id.n = 3)
```

#### Extract model results and display data for top 3 values using Cook’s distance


```
model.data <- augment(m) %>% 
  mutate(index = 1:n()) 
model.data %>% top_n(3, .cooksd)
```

#### plot standardised residuals


```
ggplot(model.data, aes(index, .std.resid)) + 
  geom_point(aes(color = Health_Binary), alpha = .5) +
  theme_bw()
```

#### Filter potential influential data points with abs(.std.res) > 3:


```
model.data %>% 
  filter(abs(.std.resid) > 3)
```

## URBAN binary logistic regression ON SIGNIFICANT OR SERIOUS ILLNESS


```
# fit binary logit model and store results 'm'
m <- glm(Health_Binary ~ Urban, data = ml,family = binomial)
# view a summary of the model
summary(m)
# test model fit
with(m, null.deviance - deviance)
with(m, df.null - df.residual)
with(m, pchisq(null.deviance - deviance, df.null - df.residual, lower.tail = FALSE))
BIC(m)
## CIs using profiled log-likelihood
confint(m, level=0.99)
## CIs using standard errors
confint.default(m, level=0.99)
# Wald test
wald.test(b = coef(m), Sigma = vcov(m), Terms = 2)
## odds ratios and 95% CI
exp(cbind(OR = coef(m), confint(m, level=0.99)))
```


### Create ROCR from data


```
## training data
pred.mtt = predict(m, type = "response") #repeat risk predictions from model m
rocr.pred.mtt = ROCR::prediction(pred.mtt, labels = ml$Health_Binary) #ROCR prediction object
roc.perf.mtt = ROCR::performance(rocr.pred.mtt, measure = "tpr", x.measure = "fpr") # #ROCR performance object
plot(roc.perf.mtt, col = "blue")
abline(a = 0, b = 1, lty = 2) #diagonal for random assignment
```

### Report AUC from ROC for training and test data


```
  # Train AUC
auc <- ROCR::performance(rocr.pred.mtt, measure = "auc")
  auc <- auc@y.values[[1]]
  print(auc)
```

### Calculate Nagelkerke R^2


```
NagelkerkeR2(m)
```

### check assumptions of model

#### Cook’s distance


```
plot(m, which = 4, id.n = 3)
```

# Extract model results and display data for top 3 values using Cook’s distance


```
model.data <- augment(m) %>% 
  mutate(index = 1:n()) 
model.data %>% top_n(3, .cooksd)
```


#### plot standardised residuals


```
ggplot(model.data, aes(index, .std.resid)) + 
  geom_point(aes(color = Health_Binary), alpha = .5) +
  theme_bw()
```

#### Filter potential influential data points with abs(.std.res) > 3:


```
model.data %>% 
  filter(abs(.std.resid) > 3)
```

## LOCATION + URBAN binary logistic regression ON SIGNIFICANT OR SERIOUS ILLNESS


```
# fit binary logit model and store results 'm'
m <- glm(Health_Binary ~ Location + Urban, data = ml,family = binomial)
# view a summary of the model
summary(m)
# test model fit
with(m, null.deviance - deviance)
with(m, df.null - df.residual)
with(m, pchisq(null.deviance - deviance, df.null - df.residual, lower.tail = FALSE))
BIC(m)
# Hosmer-Lemeshow Goodness-of-Fit Test
hltest(m)
## CIs using profiled log-likelihood
confint(m, level=0.99)
## CIs using standard errors
confint.default(m, level=0.99)
# Wald test
wald.test(b = coef(m), Sigma = vcov(m), Terms = 2)
## odds ratios and 95% CI
exp(cbind(OR = coef(m), confint(m, level=0.99)))
```


### Create ROCR from data


```
## training data
pred.mtt = predict(m, type = "response") #repeat risk predictions from model m
rocr.pred.mtt = ROCR::prediction(pred.mtt, labels = ml$Health_Binary) #ROCR prediction object
roc.perf.mtt = ROCR::performance(rocr.pred.mtt, measure = "tpr", x.measure = "fpr") # #ROCR performance object
plot(roc.perf.mtt, col = "blue")
abline(a = 0, b = 1, lty = 2) #diagonal for random assignment
```

### Report AUC from ROC for training and test data


```
  # Train AUC
auc <- ROCR::performance(rocr.pred.mtt, measure = "auc")
  auc <- auc@y.values[[1]]
  print(auc)
```

### Calculate Nagelkerke R^2


```
NagelkerkeR2(m)
```

### check assumptions of model

#### Cook’s distance


```
plot(m, which = 4, id.n = 3)
```

#### Extract model results and display data for top 3 values using Cook’s distance


```
model.data <- augment(m) %>% 
  mutate(index = 1:n()) 
model.data %>% top_n(3, .cooksd)
```

#### plot standardised residuals


```
ggplot(model.data, aes(index, .std.resid)) + 
  geom_point(aes(color = Health_Binary), alpha = .5) +
  theme_bw()
```

#### Filter potential influential data points with abs(.std.res) > 3:


```
model.data %>% 
  filter(abs(.std.resid) > 3)
```

#### check for multicollinearity


```
car::vif(m)
```

## LOCATION \* URBAN binary logistic regression ON SIGNIFICANT OR SERIOUS ILLNESS


```
# fit binary logit model and store results 'm'
m <- glm(Health_Binary ~ Location*Urban, data = ml,family = binomial)
# view a summary of the model
summary(m)
# test model fit
with(m, null.deviance - deviance)
with(m, df.null - df.residual)
with(m, pchisq(null.deviance - deviance, df.null - df.residual, lower.tail = FALSE))
BIC(m)
# Hosmer-Lemeshow Goodness-of-Fit Test
hltest(m)
## CIs using profiled log-likelihood
confint(m, level=0.99)
## CIs using standard errors
confint.default(m, level=0.99)
# Wald test
wald.test(b = coef(m), Sigma = vcov(m), Terms = 2)
## odds ratios and 95% CI
exp(cbind(OR = coef(m), confint(m, level=0.99)))
```


### Create ROCR from data


```
## training data
pred.mtt = predict(m, type = "response") #repeat risk predictions from model m
rocr.pred.mtt = ROCR::prediction(pred.mtt, labels = ml$Health_Binary) #ROCR prediction object
roc.perf.mtt = ROCR::performance(rocr.pred.mtt, measure = "tpr", x.measure = "fpr") # #ROCR performance object
plot(roc.perf.mtt, col = "blue")
abline(a = 0, b = 1, lty = 2) #diagonal for random assignment
```

### Report AUC from ROC for training and test data


```
  # Train AUC
auc <- ROCR::performance(rocr.pred.mtt, measure = "auc")
  auc <- auc@y.values[[1]]
  print(auc)
```

### Calculate Nagelkerke R^2


```
NagelkerkeR2(m)
```

### check assumptions of model

#### Cook’s distance


```
plot(m, which = 4, id.n = 3)
```

#### Extract model results and display data for top 3 values using Cook’s distance


```
model.data <- augment(m) %>% 
  mutate(index = 1:n()) 
model.data %>% top_n(3, .cooksd)
```

#### plot standardised residuals


```
ggplot(model.data, aes(index, .std.resid)) + 
  geom_point(aes(color = Health_Binary), alpha = .5) +
  theme_bw()
```

#### Filter potential influential data points with abs(.std.res) > 3:


```
model.data %>% 
  filter(abs(.std.resid) > 3)
```

#### check for multicollinearity


```
car::vif(m)
```

## EDUCATION Binary logistic regression ON SIGNIFICANT OR SERIOUS ILLNESS


```
# fit binary logit model and store results 'm'
m <- glm(Health_Binary ~ Education, data = ml,family = binomial)
# view a summary of the model
summary(m)
# test model fit
with(m, null.deviance - deviance)
with(m, df.null - df.residual)
with(m, pchisq(null.deviance - deviance, df.null - df.residual, lower.tail = FALSE))
BIC(m)
# Hosmer-Lemeshow Goodness-of-Fit Test
hltest(m)
## CIs using profiled log-likelihood
confint(m, level=0.99)
## CIs using standard errors
confint.default(m, level=0.99)
# Wald test
wald.test(b = coef(m), Sigma = vcov(m), Terms = 2)
## odds ratios and 95% CI
exp(cbind(OR = coef(m), confint(m, level=0.99)))
```


### Create ROCR from data


```
## training data
pred.mtt = predict(m, type = "response") #repeat risk predictions from model m
rocr.pred.mtt = ROCR::prediction(pred.mtt, labels = ml$Health_Binary) #ROCR prediction object
roc.perf.mtt = ROCR::performance(rocr.pred.mtt, measure = "tpr", x.measure = "fpr") # #ROCR performance object
plot(roc.perf.mtt, col = "blue")
abline(a = 0, b = 1, lty = 2) #diagonal for random assignment
```

### Report AUC from ROC for training and test data


```
  # Train AUC
auc <- ROCR::performance(rocr.pred.mtt, measure = "auc")
  auc <- auc@y.values[[1]]
  print(auc)
```

### Calculate Nagelkerke R^2


```
NagelkerkeR2(m)
```

### check assumptions of model

#### Cook’s distance


```
plot(m, which = 4, id.n = 3)
```

#### Extract model results and display data for top 3 values using Cook’s distance


```
model.data <- augment(m) %>% 
  mutate(index = 1:n()) 
model.data %>% top_n(3, .cooksd)
```

#### plot standardised residuals


```
ggplot(model.data, aes(index, .std.resid)) + 
  geom_point(aes(color = Health_Binary), alpha = .5) +
  theme_bw()
```

#### Filter potential influential data points with abs(.std.res) > 3:


```
model.data %>% 
  filter(abs(.std.resid) > 3)
```

## EDUCATION\_S Binary logistic regression ON SIGNIFICANT OR SERIOUS ILLNESS


```
# fit binary logit model and store results 'm'
m <- glm(Health_Binary ~ Education_S, data = ml,family = binomial)
# view a summary of the model
summary(m)
# test model fit
with(m, null.deviance - deviance)
with(m, df.null - df.residual)
with(m, pchisq(null.deviance - deviance, df.null - df.residual, lower.tail = FALSE))
BIC(m)
# Hosmer-Lemeshow Goodness-of-Fit Test
hltest(m, G=3)
## CIs using profiled log-likelihood
confint(m, level=0.99)
## CIs using standard errors
confint.default(m, level=0.99)
# Wald test
wald.test(b = coef(m), Sigma = vcov(m), Terms = 2)
## odds ratios and 95% CI
exp(cbind(OR = coef(m), confint(m, level=0.99)))
```


### Create ROCR from data


```
## training data
pred.mtt = predict(m, type = "response") #repeat risk predictions from model m
rocr.pred.mtt = ROCR::prediction(pred.mtt, labels = ml$Health_Binary) #ROCR prediction object
roc.perf.mtt = ROCR::performance(rocr.pred.mtt, measure = "tpr", x.measure = "fpr") # #ROCR performance object
plot(roc.perf.mtt, col = "blue")
abline(a = 0, b = 1, lty = 2) #diagonal for random assignment
```

### Report AUC from ROC for training and test data


```
  # Train AUC
auc <- ROCR::performance(rocr.pred.mtt, measure = "auc")
  auc <- auc@y.values[[1]]
  print(auc)
```

### Calculate Nagelkerke R^2


```
NagelkerkeR2(m)
```

### check assumptions of model

#### Cook’s distance


```
plot(m, which = 4, id.n = 3)
```

#### Extract model results and display data for top 3 values using Cook’s distance


```
model.data <- augment(m) %>% 
  mutate(index = 1:n()) 
model.data %>% top_n(3, .cooksd)
```

#### plot standardised residuals


```
ggplot(model.data, aes(index, .std.resid)) + 
  geom_point(aes(color = Health_Binary), alpha = .5) +
  theme_bw()
```

#### Filter potential influential data points with abs(.std.res) > 3:


```
model.data %>% 
  filter(abs(.std.resid) > 3)
```

## EDUCATION\_S2 Binary logistic regression ON SIGNIFICANT OR SERIOUS ILLNESS


```
# fit binary logit model and store results 'm'
m <- glm(Health_Binary ~ Education_S2, data = ml,family = binomial)
# view a summary of the model
summary(m)
# test model fit
with(m, null.deviance - deviance)
with(m, df.null - df.residual)
with(m, pchisq(null.deviance - deviance, df.null - df.residual, lower.tail = FALSE))
BIC(m)
# Hosmer-Lemeshow Goodness-of-Fit Test
hltest(m, G=3)
## CIs using profiled log-likelihood
confint(m, level=0.99)
## CIs using standard errors
confint.default(m, level=0.99)
# Wald test
wald.test(b = coef(m), Sigma = vcov(m), Terms = 2)
## odds ratios and 95% CI
exp(cbind(OR = coef(m), confint(m, level=0.99)))
```


### Create ROCR from data


```
## training data
pred.mtt = predict(m, type = "response") #repeat risk predictions from model m
rocr.pred.mtt = ROCR::prediction(pred.mtt, labels = ml$Health_Binary) #ROCR prediction object
roc.perf.mtt = ROCR::performance(rocr.pred.mtt, measure = "tpr", x.measure = "fpr") # #ROCR performance object
plot(roc.perf.mtt, col = "blue")
abline(a = 0, b = 1, lty = 2) #diagonal for random assignment
```

### Report AUC from ROC for training and test data


```
  # Train AUC
auc <- ROCR::performance(rocr.pred.mtt, measure = "auc")
  auc <- auc@y.values[[1]]
  print(auc)
```

### Calculate Nagelkerke R^2


```
NagelkerkeR2(m)
```

### check assumptions of model

#### Cook’s distance


```
plot(m, which = 4, id.n = 3)
```

#### Extract model results and display data for top 3 values using Cook’s distance


```
model.data <- augment(m) %>% 
  mutate(index = 1:n()) 
model.data %>% top_n(3, .cooksd)
```

#### plot standardised residuals


```
ggplot(model.data, aes(index, .std.resid)) + 
  geom_point(aes(color = Health_Binary), alpha = .5) +
  theme_bw()
```

#### Filter potential influential data points with abs(.std.res) > 3:


```
model.data %>% 
  filter(abs(.std.resid) > 3)
```

## ANIMAL CAREER 2 Binary logistic regression ON SIGNIFICANT OR SERIOUS ILLNESS


```
# fit binary logit model and store results 'm'
m <- glm(Health_Binary ~ Animal_Career2, data = ml,family = binomial)
# view a summary of the model
summary(m)
# test model fit
with(m, null.deviance - deviance)
with(m, df.null - df.residual)
with(m, pchisq(null.deviance - deviance, df.null - df.residual, lower.tail = FALSE))
BIC(m)
# Hosmer-Lemeshow Goodness-of-Fit Test
## CIs using profiled log-likelihood
confint(m, level=0.99)
## CIs using standard errors
confint.default(m, level=0.99)
# Wald test
wald.test(b = coef(m), Sigma = vcov(m), Terms = 2)
## odds ratios and 95% CI
exp(cbind(OR = coef(m), confint(m, level=0.99)))
```


### Create ROCR from data


```
## training data
pred.mtt = predict(m, type = "response") #repeat risk predictions from model m
rocr.pred.mtt = ROCR::prediction(pred.mtt, labels = ml$Health_Binary) #ROCR prediction object
roc.perf.mtt = ROCR::performance(rocr.pred.mtt, measure = "tpr", x.measure = "fpr") # #ROCR performance object
plot(roc.perf.mtt, col = "blue")
abline(a = 0, b = 1, lty = 2) #diagonal for random assignment
```

### Report AUC from ROC for training and test data


```
  # Train AUC
auc <- ROCR::performance(rocr.pred.mtt, measure = "auc")
  auc <- auc@y.values[[1]]
  print(auc)
```

### Calculate Nagelkerke R^2


```
NagelkerkeR2(m)
```

### check assumptions of model

#### Cook’s distance


```
plot(m, which = 4, id.n = 3)
```

#### Extract model results and display data for top 3 values using Cook’s distance


```
model.data <- augment(m) %>% 
  mutate(index = 1:n()) 
model.data %>% top_n(3, .cooksd)
```

#### plot standardised residuals


```
ggplot(model.data, aes(index, .std.resid)) + 
  geom_point(aes(color = Health_Binary), alpha = .5) +
  theme_bw()
```

# Filter potential influential data points with abs(.std.res) > 3:


```
model.data %>% 
  filter(abs(.std.resid) > 3)
```


## ANIMAL\_CAREER\_BINARY Binary logistic regression ON SIGNIFICANT OR SERIOUS ILLNESS


```
# fit binary logit model and store results 'm'
m <- glm(Health_Binary ~ Animal_Career_BINARY, data = ml,family = binomial)
# view a summary of the model
summary(m)
# test model fit
with(m, null.deviance - deviance)
with(m, df.null - df.residual)
with(m, pchisq(null.deviance - deviance, df.null - df.residual, lower.tail = FALSE))
BIC(m)

## CIs using profiled log-likelihood
confint(m, level=0.99)
## CIs using standard errors
confint.default(m, level=0.99)
# Wald test
wald.test(b = coef(m), Sigma = vcov(m), Terms = 2)
## odds ratios and 95% CI
exp(cbind(OR = coef(m), confint(m, level=0.99)))
```


### Create ROCR from data


```
## training data
pred.mtt = predict(m, type = "response") #repeat risk predictions from model m
rocr.pred.mtt = ROCR::prediction(pred.mtt, labels = ml$Health_Binary) #ROCR prediction object
roc.perf.mtt = ROCR::performance(rocr.pred.mtt, measure = "tpr", x.measure = "fpr") # #ROCR performance object
plot(roc.perf.mtt, col = "blue")
abline(a = 0, b = 1, lty = 2) #diagonal for random assignment
```

### Report AUC from ROC for training and test data


```
  # Train AUC
auc <- ROCR::performance(rocr.pred.mtt, measure = "auc")
  auc <- auc@y.values[[1]]
  print(auc)
```

### Calculate Nagelkerke R^2


```
NagelkerkeR2(m)
```

### check assumptions of model

#### Cook’s distance


```
plot(m, which = 4, id.n = 3)
```

#### Extract model results and display data for top 3 values using Cook’s distance


```
model.data <- augment(m) %>% 
  mutate(index = 1:n()) 
model.data %>% top_n(3, .cooksd)
```

#### plot standardised residuals


```
ggplot(model.data, aes(index, .std.resid)) + 
  geom_point(aes(color = Health_Binary), alpha = .5) +
  theme_bw()
```

#### Filter potential influential data points with abs(.std.res) > 3:


```
model.data %>% 
  filter(abs(.std.resid) > 3)
```

## INCOME Binary logistic regression ON SIGNIFICANT OR SERIOUS ILLNESS


```
# fit binary logit model and store results 'm'
m <- glm(Health_Binary ~ Income, data = ml,family = binomial)
# view a summary of the model
summary(m)
# test model fit
with(m, null.deviance - deviance)
with(m, df.null - df.residual)
with(m, pchisq(null.deviance - deviance, df.null - df.residual, lower.tail = FALSE))
BIC(m)
# Hosmer-Lemeshow Goodness-of-Fit Test
glmtoolbox::hltest(m)
## CIs using profiled log-likelihood
confint(m, level=0.99)
## CIs using standard errors
confint.default(m, level=0.99)
# Wald test
wald.test(b = coef(m), Sigma = vcov(m), Terms = 2)
## odds ratios and 95% CI
exp(cbind(OR = coef(m), confint(m, level=0.99)))
```


### Create ROCR from data


```
## training data
pred.mtt = predict(m, type = "response") #repeat risk predictions from model m
rocr.pred.mtt = ROCR::prediction(pred.mtt, labels = ml$Health_Binary) #ROCR prediction object
roc.perf.mtt = ROCR::performance(rocr.pred.mtt, measure = "tpr", x.measure = "fpr") # #ROCR performance object
plot(roc.perf.mtt, col = "blue")
abline(a = 0, b = 1, lty = 2) #diagonal for random assignment
```

### Report AUC from ROC for training and test data


```
  # Train AUC
auc <- ROCR::performance(rocr.pred.mtt, measure = "auc")
  auc <- auc@y.values[[1]]
  print(auc)
```

### Calculate Nagelkerke R^2


```
NagelkerkeR2(m)
```

### check assumptions of model

#### Cook’s distance


```
plot(m, which = 4, id.n = 3)
```

#### Extract model results and display data for top 3 values using Cook’s distance


```
model.data <- augment(m) %>% 
  mutate(index = 1:n()) 
model.data %>% top_n(3, .cooksd)
```

#### plot standardised residuals


```
ggplot(model.data, aes(index, .std.resid)) + 
  geom_point(aes(color = Health_Binary), alpha = .5) +
  theme_bw()
```

#### Filter potential influential data points with abs(.std.res) > 3:


```
model.data %>% 
  filter(abs(.std.resid) > 3)
```

## INCOME2 Binary logistic regression ON SIGNIFICANT OR SERIOUS ILLNESS


```
# fit binary logit model and store results 'm'
m <- glm(Health_Binary ~ Income2, data = ml,family = binomial)
# view a summary of the model
summary(m)
# test model fit
with(m, null.deviance - deviance)
with(m, df.null - df.residual)
with(m, pchisq(null.deviance - deviance, df.null - df.residual, lower.tail = FALSE))
BIC(m)
# Hosmer-Lemeshow Goodness-of-Fit Test
glmtoolbox::hltest(m)
## CIs using profiled log-likelihood
confint(m, level=0.99)
## CIs using standard errors
confint.default(m, level=0.99)
# Wald test
wald.test(b = coef(m), Sigma = vcov(m), Terms = 2)
## odds ratios and 95% CI
exp(cbind(OR = coef(m), confint(m, level=0.99)))
```


### Create ROCR from data


```
## training data
pred.mtt = predict(m, type = "response") #repeat risk predictions from model m
rocr.pred.mtt = ROCR::prediction(pred.mtt, labels = ml$Health_Binary) #ROCR prediction object
roc.perf.mtt = ROCR::performance(rocr.pred.mtt, measure = "tpr", x.measure = "fpr") # #ROCR performance object
plot(roc.perf.mtt, col = "blue")
abline(a = 0, b = 1, lty = 2) #diagonal for random assignment
```

### Report AUC from ROC for training and test data


```
  # Train AUC
auc <- ROCR::performance(rocr.pred.mtt, measure = "auc")
  auc <- auc@y.values[[1]]
  print(auc)
```

### Calculate Nagelkerke R^2


```
NagelkerkeR2(m)
```

### check assumptions of model

#### Cook’s distance


```
plot(m, which = 4, id.n = 3)
```

#### Extract model results and display data for top 3 values using Cook’s distance


```
model.data <- augment(m) %>% 
  mutate(index = 1:n()) 
model.data %>% top_n(3, .cooksd)
```

#### plot standardised residuals


```
ggplot(model.data, aes(index, .std.resid)) + 
  geom_point(aes(color = Health_Binary), alpha = .5) +
  theme_bw()
```

#### Filter potential influential data points with abs(.std.res) > 3:


```
model.data %>% 
  filter(abs(.std.resid) > 3)
```

## C\_AGE Binary logistic regression ON SIGNIFICANT OR SERIOUS ILLNESS


```
# fit binary logit model and store results 'm'
m <- glm(Health_Binary ~ C_Age, data = ml,family = binomial)
# view a summary of the model
summary(m)
# test model fit
with(m, null.deviance - deviance)
with(m, df.null - df.residual)
with(m, pchisq(null.deviance - deviance, df.null - df.residual, lower.tail = FALSE))
BIC(m)
# Hosmer-Lemeshow Goodness-of-Fit Test
hltest(m, G=4)
## CIs using profiled log-likelihood
confint(m, level=0.99)
## CIs using standard errors
confint.default(m, level=0.99)
# Wald test
wald.test(b = coef(m), Sigma = vcov(m), Terms = 2)
## odds ratios and 95% CI
exp(cbind(OR = coef(m), confint(m, level=0.99)))
```


### Create ROCR from data


```
## training data
pred.mtt = predict(m, type = "response") #repeat risk predictions from model m
rocr.pred.mtt = ROCR::prediction(pred.mtt, labels = ml$Health_Binary) #ROCR prediction object
roc.perf.mtt = ROCR::performance(rocr.pred.mtt, measure = "tpr", x.measure = "fpr") # #ROCR performance object
plot(roc.perf.mtt, col = "blue")
abline(a = 0, b = 1, lty = 2) #diagonal for random assignment
```

### Report AUC from ROC for training and test data


```
  # Train AUC
auc <- ROCR::performance(rocr.pred.mtt, measure = "auc")
  auc <- auc@y.values[[1]]
  print(auc)
```

### Calculate Nagelkerke R^2


```
NagelkerkeR2(m)
```

### check assumptions of model

#### Cook’s distance


```
plot(m, which = 4, id.n = 3)
```

#### Extract model results and display data for top 3 values using Cook’s distance


```
model.data <- augment(m) %>% 
  mutate(index = 1:n()) 
model.data %>% top_n(3, .cooksd)
```

#### plot standardised residuals


```
ggplot(model.data, aes(index, .std.resid)) + 
  geom_point(aes(color = Health_Binary), alpha = .5) +
  theme_bw()
```

#### Filter potential influential data points with abs(.std.res) > 3:


```
model.data %>% 
  filter(abs(.std.resid) > 3)
```

## C\_AGE2 Binary logistic regression ON SIGNIFICANT OR SERIOUS ILLNESS


```
# fit binary logit model and store results 'm'
m <- glm(Health_Binary ~ C_Age2, data = ml,family = binomial)
# view a summary of the model
summary(m)
# test model fit
with(m, null.deviance - deviance)
with(m, df.null - df.residual)
with(m, pchisq(null.deviance - deviance, df.null - df.residual, lower.tail = FALSE))
BIC(m)
# Hosmer-Lemeshow Goodness-of-Fit Test
hltest(m, G=4)
## CIs using profiled log-likelihood
confint(m, level=0.99)
## CIs using standard errors
confint.default(m, level=0.99)
# Wald test
wald.test(b = coef(m), Sigma = vcov(m), Terms = 2)
## odds ratios and 95% CI
exp(cbind(OR = coef(m), confint(m, level=0.99)))
```


### Create ROCR from data


```
## training data
pred.mtt = predict(m, type = "response") #repeat risk predictions from model m
rocr.pred.mtt = ROCR::prediction(pred.mtt, labels = ml$Health_Binary) #ROCR prediction object
roc.perf.mtt = ROCR::performance(rocr.pred.mtt, measure = "tpr", x.measure = "fpr") # #ROCR performance object
plot(roc.perf.mtt, col = "blue")
abline(a = 0, b = 1, lty = 2) #diagonal for random assignment
```

### Report AUC from ROC for training and test data


```
  # Train AUC
auc <- ROCR::performance(rocr.pred.mtt, measure = "auc")
  auc <- auc@y.values[[1]]
  print(auc)
```

## Calculate Nagelkerke R^2


```
NagelkerkeR2(m)
```

## check assumptions of model

#### Cook’s distance


```
plot(m, which = 4, id.n = 3)
```

#### Extract model results and display data for top 3 values using Cook’s distance


```
model.data <- augment(m) %>% 
  mutate(index = 1:n()) 
model.data %>% top_n(3, .cooksd)
```

#### plot standardised residuals


```
ggplot(model.data, aes(index, .std.resid)) + 
  geom_point(aes(color = Health_Binary), alpha = .5) +
  theme_bw()
```

#### Filter potential influential data points with abs(.std.res) > 3:


```
model.data %>% 
  filter(abs(.std.resid) > 3)
```

## C\_GENDER Binary logistic regression ON SIGNIFICANT OR SERIOUS ILLNESS


```
# fit binary logit model and store results 'm'
m <- glm(Health_Binary ~ C_Gender, data = ml,family = binomial)
# view a summary of the model
summary(m)
# test model fit
with(m, null.deviance - deviance)
with(m, df.null - df.residual)
with(m, pchisq(null.deviance - deviance, df.null - df.residual, lower.tail = FALSE))
BIC(m)
## CIs using profiled log-likelihood
confint(m, level=0.99)
## CIs using standard errors
confint.default(m, level=0.99)
# Wald test
wald.test(b = coef(m), Sigma = vcov(m), Terms = 2)
## odds ratios and 95% CI
exp(cbind(OR = coef(m), confint(m, level=0.99)))
```


### Create ROCR from data


```
## training data
pred.mtt = predict(m, type = "response") #repeat risk predictions from model m
rocr.pred.mtt = ROCR::prediction(pred.mtt, labels = ml$Health_Binary) #ROCR prediction object
roc.perf.mtt = ROCR::performance(rocr.pred.mtt, measure = "tpr", x.measure = "fpr") # #ROCR performance object
plot(roc.perf.mtt, col = "blue")
abline(a = 0, b = 1, lty = 2) #diagonal for random assignment
```

### Report AUC from ROC for training and test data


```
  # Train AUC
auc <- ROCR::performance(rocr.pred.mtt, measure = "auc")
  auc <- auc@y.values[[1]]
  print(auc)
```

### Calculate Nagelkerke R^2


```
NagelkerkeR2(m)
```

### check assumptions of model

#### Cook’s distance


```
plot(m, which = 4, id.n = 3)
```

#### Extract model results and display data for top 3 values using Cook’s distance


```
model.data <- augment(m) %>% 
  mutate(index = 1:n()) 
model.data %>% top_n(3, .cooksd)
```

#### plot standardised residuals


```
ggplot(model.data, aes(index, .std.resid)) + 
  geom_point(aes(color = Health_Binary), alpha = .5) +
  theme_bw()
```

#### Filter potential influential data points with abs(.std.res) > 3:


```
model.data %>% 
  filter(abs(.std.resid) > 3)
```

# NOW CHECK ASSOCIATIONS BETWEEN SIGNIFICANT OR SERIOUS ILLNESS AND DOG CHARACTERISTICS - simple BINARY LOGISTIC regression

## DOG DIET VEGAN binary regression for ON SIGNIFICANT OR SERIOUS ILLNESS


```
# fit binary logit model and store results 'm'
m <- glm(Health_Binary ~ D_Diet_Vegan, data = ml,family = binomial)
# view a summary of the model
summary(m)
# test model fit
with(m, null.deviance - deviance)
with(m, df.null - df.residual)
with(m, pchisq(null.deviance - deviance, df.null - df.residual, lower.tail = FALSE))
BIC(m)
## CIs using profiled log-likelihood
confint(m, level=0.99)
## CIs using standard errors
confint.default(m, level=0.99)
# Wald test
wald.test(b = coef(m), Sigma = vcov(m), Terms = 2)
## odds ratios and 95% CI
exp(cbind(OR = coef(m), confint(m, level=0.99)))
```


### Create ROCR from data


```
## training data
pred.mtt = predict(m, type = "response") #repeat risk predictions from model m
rocr.pred.mtt = ROCR::prediction(pred.mtt, labels = ml$Health_Binary) #ROCR prediction object
roc.perf.mtt = ROCR::performance(rocr.pred.mtt, measure = "tpr", x.measure = "fpr") # #ROCR performance object
plot(roc.perf.mtt, col = "blue")
abline(a = 0, b = 1, lty = 2) #diagonal for random assignment
```

### Report AUC from ROC for training and test data


```
  # Train AUC
auc <- ROCR::performance(rocr.pred.mtt, measure = "auc")
  auc <- auc@y.values[[1]]
  print(auc)
```

### Calculate Nagelkerke R^2


```
NagelkerkeR2(m)
```

### check assumptions of model

#### Cook’s distance


```
plot(m, which = 4, id.n = 3)
```

#### Extract model results and display data for top 3 values using Cook’s distance


```
model.data <- augment(m) %>% 
  mutate(index = 1:n()) 
model.data %>% top_n(3, .cooksd)
```

#### plot standardised residuals


```
ggplot(model.data, aes(index, .std.resid)) + 
  geom_point(aes(color = Health_Binary), alpha = .5) +
  theme_bw()
```

#### Filter potential influential data points with abs(.std.res) > 3:


```
model.data %>% 
  filter(abs(.std.resid) > 3)
```

## DOG DIET VEGAN\_VEGGIE binary regression for ON SIGNIFICANT OR SERIOUS ILLNESS


```
# fit binary logit model and store results 'm'
m <- glm(Health_Binary ~ D_Diet_Vegan_Veggie, data = ml,family = binomial)
# view a summary of the model
summary(m)
# test model fit
with(m, null.deviance - deviance)
with(m, df.null - df.residual)
with(m, pchisq(null.deviance - deviance, df.null - df.residual, lower.tail = FALSE))
BIC(m)
## CIs using profiled log-likelihood
confint(m, level=0.99)
## CIs using standard errors
confint.default(m, level=0.99)
# Wald test
wald.test(b = coef(m), Sigma = vcov(m), Terms = 2)
## odds ratios and 95% CI
exp(cbind(OR = coef(m), confint(m, level=0.99)))
```


### Create ROCR from data


```
## training data
pred.mtt = predict(m, type = "response") #repeat risk predictions from model m
rocr.pred.mtt = ROCR::prediction(pred.mtt, labels = ml$Health_Binary) #ROCR prediction object
roc.perf.mtt = ROCR::performance(rocr.pred.mtt, measure = "tpr", x.measure = "fpr") # #ROCR performance object
plot(roc.perf.mtt, col = "blue")
abline(a = 0, b = 1, lty = 2) #diagonal for random assignment
```

### Report AUC from ROC for training and test data


```
  # Train AUC
auc <- ROCR::performance(rocr.pred.mtt, measure = "auc")
  auc <- auc@y.values[[1]]
  print(auc)
```

### Calculate Nagelkerke R^2


```
NagelkerkeR2(m)
```

### check assumptions of model

#### Cook’s distance


```
plot(m, which = 4, id.n = 3)
```

#### Extract model results and display data for top 3 values using Cook’s distance


```
model.data <- augment(m) %>% 
  mutate(index = 1:n()) 
model.data %>% top_n(3, .cooksd)
```

#### plot standardised residuals


```
ggplot(model.data, aes(index, .std.resid)) + 
  geom_point(aes(color = Health_Binary), alpha = .5) +
  theme_bw()
```

#### Filter potential influential data points with abs(.std.res) > 3:


```
model.data %>% 
  filter(abs(.std.resid) > 3)
```

## DOG DIET binary regression ON SIGNIFICANT OR SERIOUS ILLNESS


```
# fit binary logit model and store results 'm'
m <- glm(Health_Binary ~ D_Diet, data = ml,family = binomial)
# view a summary of the model
summary(m)
# test model fit
with(m, null.deviance - deviance)
with(m, df.null - df.residual)
with(m, pchisq(null.deviance - deviance, df.null - df.residual, lower.tail = FALSE))
BIC(m)
## CIs using profiled log-likelihood
confint(m, level=0.99)
## CIs using standard errors
confint.default(m, level=0.99)
# Wald test
wald.test(b = coef(m), Sigma = vcov(m), Terms = 2)
## odds ratios and 95% CI
exp(cbind(OR = coef(m), confint(m, level=0.99)))
```


### Create ROCR from data


```
## training data
pred.mtt = predict(m, type = "response") #repeat risk predictions from model m
rocr.pred.mtt = ROCR::prediction(pred.mtt, labels = ml$Health_Binary) #ROCR prediction object
roc.perf.mtt = ROCR::performance(rocr.pred.mtt, measure = "tpr", x.measure = "fpr") # #ROCR performance object
plot(roc.perf.mtt, col = "blue")
abline(a = 0, b = 1, lty = 2) #diagonal for random assignment
```

### Report AUC from ROC for training and test data


```
  # Train AUC
auc <- ROCR::performance(rocr.pred.mtt, measure = "auc")
  auc <- auc@y.values[[1]]
  print(auc)
```

### Calculate Nagelkerke R^2


```
NagelkerkeR2(m)
```

### check assumptions of model

#### Cook’s distance


```
plot(m, which = 4, id.n = 3)
```

#### Extract model results and display data for top 3 values using Cook’s distance


```
model.data <- augment(m) %>% 
  mutate(index = 1:n()) 
model.data %>% top_n(3, .cooksd)
```

#### plot standardised residuals


```
ggplot(model.data, aes(index, .std.resid)) + 
  geom_point(aes(color = Health_Binary), alpha = .5) +
  theme_bw()
```

#### Filter potential influential data points with abs(.std.res) > 3:


```
model.data %>% 
  filter(abs(.std.resid) > 3)
```

## CLIENT DIET VEGAN + DOG DIET VEGAN binaryl regression ON SIGNIFICANT OR SERIOUS ILLNESS


```
# fit binary logit model and store results 'm2'
m <- glm(Health_Binary ~ C_Diet_Vegan + D_Diet_Vegan, data = ml,family = binomial)
# view a summary of the model
summary(m)
# test model fit
with(m, null.deviance - deviance)
with(m, df.null - df.residual)
with(m, pchisq(null.deviance - deviance, df.null - df.residual, lower.tail = FALSE))
BIC(m)
# Hosmer-Lemeshow Goodness-of-Fit Test
glmtoolbox::hltest(m)
## CIs using profiled log-likelihood
confint(m, level=0.99)
## CIs using standard errors
confint.default(m, level=0.99)
# Wald test
wald.test(b = coef(m), Sigma = vcov(m), Terms = 2)
## odds ratios and 95% CI
exp(cbind(OR = coef(m), confint(m, level=0.99)))
```


### Create ROCR from data


```
## training data
pred.mtt = predict(m, type = "response") #repeat risk predictions from model m
rocr.pred.mtt = ROCR::prediction(pred.mtt, labels = ml$Health_Binary) #ROCR prediction object
roc.perf.mtt = ROCR::performance(rocr.pred.mtt, measure = "tpr", x.measure = "fpr") # #ROCR performance object
plot(roc.perf.mtt, col = "blue")
abline(a = 0, b = 1, lty = 2) #diagonal for random assignment
```

### Report AUC from ROC for training and test data


```
  # Train AUC
auc <- ROCR::performance(rocr.pred.mtt, measure = "auc")
  auc <- auc@y.values[[1]]
  print(auc)
```

### Calculate Nagelkerke R^2


```
NagelkerkeR2(m)
```

### check assumptions of model

#### Cook’s distance


```
plot(m, which = 4, id.n = 3)
```

#### Extract model results and display data for top 3 values using Cook’s distance


```
model.data <- augment(m) %>% 
  mutate(index = 1:n()) 
model.data %>% top_n(3, .cooksd)
```

#### plot standardised residuals


```
ggplot(model.data, aes(index, .std.resid)) + 
  geom_point(aes(color = Health_Binary), alpha = .5) +
  theme_bw()
```

#### Filter potential influential data points with abs(.std.res) > 3:


```
model.data %>% 
  filter(abs(.std.resid) > 3)
```

#### check for multicollinearity


```
car::vif(m)
```

## CLIENT DIET VEGAN \* DOG DIET VEGAN binaryl regression ON SIGNIFICANT OR SERIOUS ILLNESS


```
# fit binary logit model and store results 'm2'
m <- glm(Health_Binary ~ C_Diet_Vegan*D_Diet_Vegan, data = ml,family = binomial)
# view a summary of the model
summary(m)
# test model fit
with(m, null.deviance - deviance)
with(m, df.null - df.residual)
with(m, pchisq(null.deviance - deviance, df.null - df.residual, lower.tail = FALSE))
BIC(m)
# Hosmer-Lemeshow Goodness-of-Fit Test
glmtoolbox::hltest(m)
## CIs using profiled log-likelihood
confint(m, level=0.99)
## CIs using standard errors
confint.default(m, level=0.99)
# Wald test
wald.test(b = coef(m), Sigma = vcov(m), Terms = 2)
## odds ratios and 95% CI
exp(cbind(OR = coef(m), confint(m, level=0.99)))
```


### Create ROCR from data


```
## training data
pred.mtt = predict(m, type = "response") #repeat risk predictions from model m
rocr.pred.mtt = ROCR::prediction(pred.mtt, labels = ml$Health_Binary) #ROCR prediction object
roc.perf.mtt = ROCR::performance(rocr.pred.mtt, measure = "tpr", x.measure = "fpr") # #ROCR performance object
plot(roc.perf.mtt, col = "blue")
abline(a = 0, b = 1, lty = 2) #diagonal for random assignment
```

### Report AUC from ROC for training and test data


```
  # Train AUC
auc <- ROCR::performance(rocr.pred.mtt, measure = "auc")
  auc <- auc@y.values[[1]]
  print(auc)
```

### Calculate Nagelkerke R^2


```
NagelkerkeR2(m)
```

### check assumptions of model

#### Cook’s distance


```
plot(m, which = 4, id.n = 3)
```

#### Extract model results and display data for top 3 values using Cook’s distance


```
model.data <- augment(m) %>% 
  mutate(index = 1:n()) 
model.data %>% top_n(3, .cooksd)
```

#### plot standardised residuals


```
ggplot(model.data, aes(index, .std.resid)) + 
  geom_point(aes(color = Health_Binary), alpha = .5) +
  theme_bw()
```

#### Filter potential influential data points with abs(.std.res) > 3:


```
model.data %>% 
  filter(abs(.std.resid) > 3)
```

#### check for multicollinearity


```
car::vif(m)
```

## DOG DIET RAW binary regression ON SIGNIFICANT OR SERIOUS ILLNESS


```
# fit binary logit model and store results 'm'
m <- glm(Health_Binary ~ D_Diet_Raw, data = ml,family = binomial)
# view a summary of the model
summary(m)
# test model fit
with(m, null.deviance - deviance)
with(m, df.null - df.residual)
with(m, pchisq(null.deviance - deviance, df.null - df.residual, lower.tail = FALSE))
BIC(m)
## CIs using profiled log-likelihood
confint(m, level=0.99)
## CIs using standard errors
confint.default(m, level=0.99)
# Wald test
wald.test(b = coef(m), Sigma = vcov(m), Terms = 2)
## odds ratios and 95% CI
exp(cbind(OR = coef(m), confint(m, level=0.99)))
```


### Create ROCR from data


```
## training data
pred.mtt = predict(m, type = "response") #repeat risk predictions from model m
rocr.pred.mtt = ROCR::prediction(pred.mtt, labels = ml$Health_Binary) #ROCR prediction object
roc.perf.mtt = ROCR::performance(rocr.pred.mtt, measure = "tpr", x.measure = "fpr") # #ROCR performance object
plot(roc.perf.mtt, col = "blue")
abline(a = 0, b = 1, lty = 2) #diagonal for random assignment
```

### Report AUC from ROC for training and test data


```
  # Train AUC
auc <- ROCR::performance(rocr.pred.mtt, measure = "auc")
  auc <- auc@y.values[[1]]
  print(auc)
```

### Calculate Nagelkerke R^2


```
NagelkerkeR2(m)
```

### check assumptions of model

#### Cook’s distance


```
plot(m, which = 4, id.n = 3)
```

#### Extract model results and display data for top 3 values using Cook’s distance


```
model.data <- augment(m) %>% 
  mutate(index = 1:n()) 
model.data %>% top_n(3, .cooksd)
```

# plot standardised residuals


```
ggplot(model.data, aes(index, .std.resid)) + 
  geom_point(aes(color = Health_Binary), alpha = .5) +
  theme_bw()
```


#### Filter potential influential data points with abs(.std.res) > 3:


```
model.data %>% 
  filter(abs(.std.resid) > 3)
```

## DOG DIET + CLIENT DIET binary logistic regression ON SIGNIFICANT OR SERIOUS ILLNESS


```
# fit binary logit model and store results 'm'
m <- glm(Health_Binary ~ D_Diet + C_Diet , data = ml,family = binomial)
# view a summary of the model
summary(m)
# test model fit
with(m, null.deviance - deviance)
with(m, df.null - df.residual)
with(m, pchisq(null.deviance - deviance, df.null - df.residual, lower.tail = FALSE))
BIC(m)
# Hosmer-Lemeshow Goodness-of-Fit Test
hltest(m, G=7)
## CIs using profiled log-likelihood
confint(m, level=0.99)
## CIs using standard errors
confint.default(m, level=0.99)
# Wald test
wald.test(b = coef(m), Sigma = vcov(m), Terms = 2)
## odds ratios and 95% CI
exp(cbind(OR = coef(m), confint(m, level=0.99)))
```


### Create ROCR from data


```
## training data
pred.mtt = predict(m, type = "response") #repeat risk predictions from model m
rocr.pred.mtt = ROCR::prediction(pred.mtt, labels = ml$Health_Binary) #ROCR prediction object
roc.perf.mtt = ROCR::performance(rocr.pred.mtt, measure = "tpr", x.measure = "fpr") # #ROCR performance object
plot(roc.perf.mtt, col = "blue")
abline(a = 0, b = 1, lty = 2) #diagonal for random assignment
```

### Report AUC from ROC for training and test data


```
  # Train AUC
auc <- ROCR::performance(rocr.pred.mtt, measure = "auc")
  auc <- auc@y.values[[1]]
  print(auc)
```

### Calculate Nagelkerke R^2


```
NagelkerkeR2(m)
```

### check assumptions of model

#### Cook’s distance


```
plot(m, which = 4, id.n = 3)
```

#### Extract model results and display data for top 3 values using Cook’s distance


```
model.data <- augment(m) %>% 
  mutate(index = 1:n()) 
model.data %>% top_n(3, .cooksd)
```

#### plot standardised residuals


```
ggplot(model.data, aes(index, .std.resid)) + 
  geom_point(aes(color = Health_Binary), alpha = .5) +
  theme_bw()
```

#### Filter potential influential data points with abs(.std.res) > 3:


```
model.data %>% 
  filter(abs(.std.resid) > 3)
```

#### check for multicollinearity


```
car::vif(m)
```

## THERAPEUTIC DIET binary logistic regression ON SIGNIFICANT OR SERIOUS ILLNESS


```
# fit binary logit model and store results 'm'
m <- glm(Health_Binary ~ Therapeutic_Food, data = ml,family = binomial)
# view a summary of the model
summary(m)
# test model fit
with(m, null.deviance - deviance)
with(m, df.null - df.residual)
with(m, pchisq(null.deviance - deviance, df.null - df.residual, lower.tail = FALSE))
BIC(m)

## CIs using profiled log-likelihood
confint(m, level=0.99)
## CIs using standard errors
confint.default(m, level=0.99)
# Wald test
wald.test(b = coef(m), Sigma = vcov(m), Terms = 2)
## odds ratios and 95% CI
exp(cbind(OR = coef(m), confint(m, level=0.99)))
```


### Create ROCR from data


```
## training data
pred.mtt = predict(m, type = "response") #repeat risk predictions from model m
rocr.pred.mtt = ROCR::prediction(pred.mtt, labels = ml$Health_Binary) #ROCR prediction object
roc.perf.mtt = ROCR::performance(rocr.pred.mtt, measure = "tpr", x.measure = "fpr") # #ROCR performance object
plot(roc.perf.mtt, col = "blue")
abline(a = 0, b = 1, lty = 2) #diagonal for random assignment
```

### Report AUC from ROC for training and test data


```
  # Train AUC
auc <- ROCR::performance(rocr.pred.mtt, measure = "auc")
  auc <- auc@y.values[[1]]
  print(auc)
```

### Calculate Nagelkerke R^2


```
NagelkerkeR2(m)
```

### check assumptions of model

#### Cook’s distance


```
plot(m, which = 4, id.n = 3)
```

#### Extract model results and display data for top 3 values using Cook’s distance


```
model.data <- augment(m) %>% 
  mutate(index = 1:n()) 
model.data %>% top_n(3, .cooksd)
```

#### plot standardised residuals


```
ggplot(model.data, aes(index, .std.resid)) + 
  geom_point(aes(color = Health_Binary), alpha = .5) +
  theme_bw()
```

#### Filter potential influential data points with abs(.std.res) > 3:


```
model.data %>% 
  filter(abs(.std.resid) > 3)
```

## DOG DIET + THERAPEUTIC DIET binary logistic regression ON SIGNIFICANT OR SERIOUS ILLNESS


```
# fit binary logit model and store results 'm'
m <- glm(Health_Binary ~ D_Diet + Therapeutic_Food, data = ml,family = binomial)
# view a summary of the model
summary(m)
# test model fit
with(m, null.deviance - deviance)
with(m, df.null - df.residual)
with(m, pchisq(null.deviance - deviance, df.null - df.residual, lower.tail = FALSE))
BIC(m)
# Hosmer-Lemeshow Goodness-of-Fit Test
hltest(m, G=3)
## CIs using profiled log-likelihood
confint(m, level=0.99)
## CIs using standard errors
confint.default(m, level=0.99)
# Wald test
wald.test(b = coef(m), Sigma = vcov(m), Terms = 2)
## odds ratios and 95% CI
exp(cbind(OR = coef(m), confint(m, level=0.99)))
```


### Create ROCR from data


```
## training data
pred.mtt = predict(m, type = "response") #repeat risk predictions from model m
rocr.pred.mtt = ROCR::prediction(pred.mtt, labels = ml$Health_Binary) #ROCR prediction object
roc.perf.mtt = ROCR::performance(rocr.pred.mtt, measure = "tpr", x.measure = "fpr") # #ROCR performance object
plot(roc.perf.mtt, col = "blue")
abline(a = 0, b = 1, lty = 2) #diagonal for random assignment
```

### Report AUC from ROC for training and test data


```
  # Train AUC
auc <- ROCR::performance(rocr.pred.mtt, measure = "auc")
  auc <- auc@y.values[[1]]
  print(auc)
```

### Calculate Nagelkerke R^2


```
NagelkerkeR2(m)
```

### check assumptions of model

#### Cook’s distance


```
plot(m, which = 4, id.n = 3)
```

#### Extract model results and display data for top 3 values using Cook’s distance


```
model.data <- augment(m) %>% 
  mutate(index = 1:n()) 
model.data %>% top_n(3, .cooksd)
```

#### plot standardised residuals


```
ggplot(model.data, aes(index, .std.resid)) + 
  geom_point(aes(color = Health_Binary), alpha = .5) +
  theme_bw()
```

#### Filter potential influential data points with abs(.std.res) > 3:


```
model.data %>% 
  filter(abs(.std.resid) > 3)
```

#### check for multicollinearity


```
car::vif(m)
```

## DOG DIET \* THERAPEUTIC DIET binary logistic regression ON SIGNIFICANT OR SERIOUS ILLNESS


```
# fit binary logit model and store results 'm'
m <- glm(Health_Binary ~ D_Diet*Therapeutic_Food, data = ml,family = binomial)
# view a summary of the model
summary(m)
# test model fit
with(m, null.deviance - deviance)
with(m, df.null - df.residual)
with(m, pchisq(null.deviance - deviance, df.null - df.residual, lower.tail = FALSE))
BIC(m)
# Hosmer-Lemeshow Goodness-of-Fit Test
hltest(m, G=7)
## CIs using profiled log-likelihood
confint(m, level=0.99)
## CIs using standard errors
confint.default(m, level=0.99)
# Wald test
wald.test(b = coef(m), Sigma = vcov(m), Terms = 2)
## odds ratios and 95% CI
exp(cbind(OR = coef(m), confint(m, level=0.99)))
```


### Create ROCR from data


```
## training data
pred.mtt = predict(m, type = "response") #repeat risk predictions from model m
rocr.pred.mtt = ROCR::prediction(pred.mtt, labels = ml$Health_Binary) #ROCR prediction object
roc.perf.mtt = ROCR::performance(rocr.pred.mtt, measure = "tpr", x.measure = "fpr") # #ROCR performance object
plot(roc.perf.mtt, col = "blue")
abline(a = 0, b = 1, lty = 2) #diagonal for random assignment
```

### Report AUC from ROC for training and test data


```
  # Train AUC
auc <- ROCR::performance(rocr.pred.mtt, measure = "auc")
  auc <- auc@y.values[[1]]
  print(auc)
```

### Calculate Nagelkerke R^2


```
NagelkerkeR2(m)
```

### check assumptions of model

#### Cook’s distance


```
plot(m, which = 4, id.n = 3)
```

#### Extract model results and display data for top 3 values using Cook’s distance


```
model.data <- augment(m) %>% 
  mutate(index = 1:n()) 
model.data %>% top_n(3, .cooksd)
```

#### plot standardised residuals


```
ggplot(model.data, aes(index, .std.resid)) + 
  geom_point(aes(color = Health_Binary), alpha = .5) +
  theme_bw()
```

#### Filter potential influential data points with abs(.std.res) > 3:


```
model.data %>% 
  filter(abs(.std.resid) > 3)
```

#### check for multicollinearity

##### Note interactions


```
car::vif(m)
```

## SIZE Binary logistic regression ON SIGNIFICANT OR SERIOUS ILLNESS


```
# fit binary logit model and store results 'm'
m <- glm(Health_Binary ~ Size, data = ml,family = binomial)
# view a summary of the model
summary(m)
# test model fit
with(m, null.deviance - deviance)
with(m, df.null - df.residual)
with(m, pchisq(null.deviance - deviance, df.null - df.residual, lower.tail = FALSE))
BIC(m)
# Hosmer-Lemeshow Goodness-of-Fit Test
hltest(m)
## CIs using profiled log-likelihood
confint(m, level=0.99)
## CIs using standard errors
confint.default(m, level=0.99)
# Wald test
wald.test(b = coef(m), Sigma = vcov(m), Terms = 2)
## odds ratios and 95% CI
exp(cbind(OR = coef(m), confint(m, level=0.99)))
```


### Create ROCR from data


```
## training data
pred.mtt = predict(m, type = "response") #repeat risk predictions from model m
rocr.pred.mtt = ROCR::prediction(pred.mtt, labels = ml$Health_Binary) #ROCR prediction object
roc.perf.mtt = ROCR::performance(rocr.pred.mtt, measure = "tpr", x.measure = "fpr") # #ROCR performance object
plot(roc.perf.mtt, col = "blue")
abline(a = 0, b = 1, lty = 2) #diagonal for random assignment
```

### Report AUC from ROC for training and test data


```
  # Train AUC
auc <- ROCR::performance(rocr.pred.mtt, measure = "auc")
  auc <- auc@y.values[[1]]
  print(auc)
```

### Calculate Nagelkerke R^2


```
NagelkerkeR2(m)
```

### check assumptions of model

#### Cook’s distance


```
plot(m, which = 4, id.n = 3)
```

#### Extract model results and display data for top 3 values using Cook’s distance


```
model.data <- augment(m) %>% 
  mutate(index = 1:n()) 
model.data %>% top_n(3, .cooksd)
```

#### plot standardised residuals


```
ggplot(model.data, aes(index, .std.resid)) + 
  geom_point(aes(color = Health_Binary), alpha = .5) +
  theme_bw()
```

#### Filter potential influential data points with abs(.std.res) > 3:


```
model.data %>% 
  filter(abs(.std.resid) > 3)
```

## SIZE2 Binary logistic regression for HEALTH


```
# fit binary logit model and store results 'm'
m <- glm(Health_Binary ~ Size2, data = ml,family = binomial)
# view a summary of the model
summary(m)
# test model fit
with(m, null.deviance - deviance)
with(m, df.null - df.residual)
with(m, pchisq(null.deviance - deviance, df.null - df.residual, lower.tail = FALSE))
BIC(m)
# Hosmer-Lemeshow Goodness-of-Fit Test
hltest(m)
## CIs using profiled log-likelihood
confint(m, level=0.99)
## CIs using standard errors
confint.default(m, level=0.99)
# Wald test
wald.test(b = coef(m), Sigma = vcov(m), Terms = 2)
## odds ratios and 95% CI
exp(cbind(OR = coef(m), confint(m, level=0.99)))
```


### Create ROCR from data


```
## training data
pred.mtt = predict(m, type = "response") #repeat risk predictions from model m
rocr.pred.mtt = ROCR::prediction(pred.mtt, labels = ml$Health_Binary) #ROCR prediction object
roc.perf.mtt = ROCR::performance(rocr.pred.mtt, measure = "tpr", x.measure = "fpr") # #ROCR performance object
plot(roc.perf.mtt, col = "blue")
abline(a = 0, b = 1, lty = 2) #diagonal for random assignment
```

### Report AUC from ROC for training and test data


```
  # Train AUC
auc <- ROCR::performance(rocr.pred.mtt, measure = "auc")
  auc <- auc@y.values[[1]]
  print(auc)
```

### Calculate Nagelkerke R^2


```
NagelkerkeR2(m)
```

### check assumptions of model

#### Cook’s distance


```
plot(m, which = 4, id.n = 3)
```

#### Extract model results and display data for top 3 values using Cook’s distance


```
model.data <- augment(m) %>% 
  mutate(index = 1:n()) 
model.data %>% top_n(3, .cooksd)
```

#### plot standardised residuals


```
ggplot(model.data, aes(index, .std.resid)) + 
  geom_point(aes(color = Health_Binary), alpha = .5) +
  theme_bw()
```

#### Filter potential influential data points with abs(.std.res) > 3:


```
model.data %>% 
  filter(abs(.std.resid) > 3)
```

## SIZE\_GIANT Binary logistic regression for HEALTH


```
# fit binary logit model and store results 'm'
m <- glm(Health_Binary ~ Size_Giant, data = ml,family = binomial)
# view a summary of the model
summary(m)
# test model fit
with(m, null.deviance - deviance)
with(m, df.null - df.residual)
with(m, pchisq(null.deviance - deviance, df.null - df.residual, lower.tail = FALSE))
BIC(m)

## CIs using profiled log-likelihood
confint(m, level=0.99)
## CIs using standard errors
confint.default(m, level=0.99)
# Wald test
wald.test(b = coef(m), Sigma = vcov(m), Terms = 2)
## odds ratios and 95% CI
exp(cbind(OR = coef(m), confint(m, level=0.99)))
```


### Create ROCR from data


```
## training data
pred.mtt = predict(m, type = "response") #repeat risk predictions from model m
rocr.pred.mtt = ROCR::prediction(pred.mtt, labels = ml$Health_Binary) #ROCR prediction object
roc.perf.mtt = ROCR::performance(rocr.pred.mtt, measure = "tpr", x.measure = "fpr") # #ROCR performance object
plot(roc.perf.mtt, col = "blue")
abline(a = 0, b = 1, lty = 2) #diagonal for random assignment
```

### Report AUC from ROC for training and test data


```
  # Train AUC
auc <- ROCR::performance(rocr.pred.mtt, measure = "auc")
  auc <- auc@y.values[[1]]
  print(auc)
```

### Calculate Nagelkerke R^2


```
NagelkerkeR2(m)
```

### check assumptions of model

#### Cook’s distance


```
plot(m, which = 4, id.n = 3)
```

#### Extract model results and display data for top 3 values using Cook’s distance


```
model.data <- augment(m) %>% 
  mutate(index = 1:n()) 
model.data %>% top_n(3, .cooksd)
```

#### plot standardised residuals


```
ggplot(model.data, aes(index, .std.resid)) + 
  geom_point(aes(color = Health_Binary), alpha = .5) +
  theme_bw()
```

#### Filter potential influential data points with abs(.std.res) > 3:


```
model.data %>% 
  filter(abs(.std.resid) > 3)
```

## D\_AGE Binary logistic regression for HEALTH


```
# fit binary logit model and store results 'm'
m <- glm(Health_Binary ~ D_Age, data = ml,family = binomial)
# view a summary of the model
summary(m)
# test model fit
with(m, null.deviance - deviance)
with(m, df.null - df.residual)
with(m, pchisq(null.deviance - deviance, df.null - df.residual, lower.tail = FALSE))
BIC(m)
# Hosmer-Lemeshow Goodness-of-Fit Test
hltest(m)
## CIs using profiled log-likelihood
confint(m, level=0.99)
## CIs using standard errors
confint.default(m, level=0.99)
# Wald test
wald.test(b = coef(m), Sigma = vcov(m), Terms = 2)
## odds ratios and 95% CI
exp(cbind(OR = coef(m), confint(m, level=0.99)))
```


### Create ROCR from data


```
## training data
pred.mtt = predict(m, type = "response") #repeat risk predictions from model m
rocr.pred.mtt = ROCR::prediction(pred.mtt, labels = ml$Health_Binary) #ROCR prediction object
roc.perf.mtt = ROCR::performance(rocr.pred.mtt, measure = "tpr", x.measure = "fpr") # #ROCR performance object
plot(roc.perf.mtt, col = "blue")
abline(a = 0, b = 1, lty = 2) #diagonal for random assignment
```

### Report AUC from ROC for training and test data


```
  # Train AUC
auc <- ROCR::performance(rocr.pred.mtt, measure = "auc")
  auc <- auc@y.values[[1]]
  print(auc)
```

### Calculate Nagelkerke R^2


```
NagelkerkeR2(m)
```

### Check age is linear with logit of outcome

##### Note lack of linearity


```
ypred = predict(m)
res = residuals(m, type = 'deviance')
plot(ypred,res)
```

### Box Tidwell test to check that D\_Age is linearly associated with the logit of the outcome

##### suggests not linear


```
boxTidwell(ml$Health_Binary ~ ml$D_Age)
```

### check assumptions of model

#### Cook’s distance


```
plot(m, which = 4, id.n = 3)
```

#### Extract model results and display data for top 3 values using Cook’s distance


```
model.data <- augment(m) %>% 
  mutate(index = 1:n()) 
model.data %>% top_n(3, .cooksd)
```

#### plot standardised residuals


```
ggplot(model.data, aes(index, .std.resid)) + 
  geom_point(aes(color = Health_Binary), alpha = .5) +
  theme_bw()
```

#### Filter potential influential data points with abs(.std.res) > 3:


```
model.data %>% 
  filter(abs(.std.resid) > 3)
```

## D\_AGE Binary logistic regression for HEALTH


```
# fit binary logit model and store results 'm'
m <- glm(Health_Binary ~ bs(D_Age, degree=1,df=2), data = ml,family = binomial)
# view a summary of the model
summary(m)
# test model fit
with(m, null.deviance - deviance)
with(m, df.null - df.residual)
with(m, pchisq(null.deviance - deviance, df.null - df.residual, lower.tail = FALSE))
BIC(m)
# Hosmer-Lemeshow Goodness-of-Fit Test
hltest(m)
## CIs using profiled log-likelihood
confint(m, level=0.99)
## CIs using standard errors
confint.default(m, level=0.99)
# Wald test
wald.test(b = coef(m), Sigma = vcov(m), Terms = 2)
## odds ratios and 95% CI
exp(cbind(OR = coef(m), confint(m, level=0.99)))
```


### Create ROCR from data


```
## training data
pred.mtt = predict(m, type = "response") #repeat risk predictions from model m
rocr.pred.mtt = ROCR::prediction(pred.mtt, labels = ml$Health_Binary) #ROCR prediction object
roc.perf.mtt = ROCR::performance(rocr.pred.mtt, measure = "tpr", x.measure = "fpr") # #ROCR performance object
plot(roc.perf.mtt, col = "blue")
abline(a = 0, b = 1, lty = 2) #diagonal for random assignment
```

### Report AUC from ROC for training and test data


```
  # Train AUC
auc <- ROCR::performance(rocr.pred.mtt, measure = "auc")
  auc <- auc@y.values[[1]]
  print(auc)
```

### Calculate Nagelkerke R^2


```
NagelkerkeR2(m)
```

### check assumptions of model

#### Cook’s distance


```
plot(m, which = 4, id.n = 3)
```

#### Extract model results and display data for top 3 values using Cook’s distance


```
model.data <- augment(m) %>% 
  mutate(index = 1:n()) 
model.data %>% top_n(3, .cooksd)
```

#### plot standardised residuals


```
ggplot(model.data, aes(index, .std.resid)) + 
  geom_point(aes(color = Health_Binary), alpha = .5) +
  theme_bw()
```

#### Filter potential influential data points with abs(.std.res) > 3:


```
model.data %>% 
  filter(abs(.std.resid) > 3)
```

## D\_Age\_quant logistic regression for HEALTH

#### Note better model fit that D\_Age


```
# fit binary logit model and store results 'm'
m <- glm(Health_Binary ~ D_Age_quant, data = ml,family = binomial)
# view a summary of the model
summary(m)
# test model fit
with(m, null.deviance - deviance)
with(m, df.null - df.residual)
with(m, pchisq(null.deviance - deviance, df.null - df.residual, lower.tail = FALSE))
BIC(m)
# Hosmer-Lemeshow Goodness-of-Fit Test
hltest(m, G=4)
## CIs using profiled log-likelihood
confint(m, level=0.99)
## CIs using standard errors
confint.default(m, level=0.99)
# Wald test
wald.test(b = coef(m), Sigma = vcov(m), Terms = 2)
## odds ratios and 95% CI
exp(cbind(OR = coef(m), confint(m, level=0.99)))
```

### Create ROCR from data


```
## training data
pred.mtt = predict(m, type = "response") #repeat risk predictions from model m
rocr.pred.mtt = ROCR::prediction(pred.mtt, labels = ml$Health_Binary) #ROCR prediction object
roc.perf.mtt = ROCR::performance(rocr.pred.mtt, measure = "tpr", x.measure = "fpr") # #ROCR performance object
plot(roc.perf.mtt, col = "blue")
abline(a = 0, b = 1, lty = 2) #diagonal for random assignment
```

### Report AUC from ROC for training and test data


```
  # Train AUC
auc <- ROCR::performance(rocr.pred.mtt, measure = "auc")
  auc <- auc@y.values[[1]]
  print(auc)
```

### Calculate Nagelkerke R^2


```
NagelkerkeR2(m)
```

### check assumptions of model

#### Cook’s distance


```
plot(m, which = 4, id.n = 3)
```

#### Extract model results and display data for top 3 values using Cook’s distance


```
model.data <- augment(m) %>% 
  mutate(index = 1:n()) 
model.data %>% top_n(3, .cooksd)
```

#### plot standardised residuals


```
ggplot(model.data, aes(index, .std.resid)) + 
  geom_point(aes(color = Health_Binary), alpha = .5) +
  theme_bw()
```

#### Filter potential influential data points with abs(.std.res) > 3:


```
model.data %>% 
  filter(abs(.std.resid) > 3)
```

## D\_SEX Binary logistic regression for HEALTH


```
# fit binary logit model and store results 'm'
m <- glm(Health_Binary ~ D_Sex, data = ml,family = binomial)
# view a summary of the model
summary(m)
# test model fit
with(m, null.deviance - deviance)
with(m, df.null - df.residual)
with(m, pchisq(null.deviance - deviance, df.null - df.residual, lower.tail = FALSE))
BIC(m)
## CIs using profiled log-likelihood
confint(m, level=0.99)
## CIs using standard errors
confint.default(m, level=0.99)
# Wald test
wald.test(b = coef(m), Sigma = vcov(m), Terms = 2)
## odds ratios and 95% CI
exp(cbind(OR = coef(m), confint(m, level=0.99)))
```


### Create ROCR from data


```
## training data
pred.mtt = predict(m, type = "response") #repeat risk predictions from model m
rocr.pred.mtt = ROCR::prediction(pred.mtt, labels = ml$Health_Binary) #ROCR prediction object
roc.perf.mtt = ROCR::performance(rocr.pred.mtt, measure = "tpr", x.measure = "fpr") # #ROCR performance object
plot(roc.perf.mtt, col = "blue")
abline(a = 0, b = 1, lty = 2) #diagonal for random assignment
```

### Report AUC from ROC for training and test data


```
  # Train AUC
auc <- ROCR::performance(rocr.pred.mtt, measure = "auc")
  auc <- auc@y.values[[1]]
  print(auc)
```

### Calculate Nagelkerke R^2


```
NagelkerkeR2(m)
```

### check assumptions of model

#### Cook’s distance


```
plot(m, which = 4, id.n = 3)
```

#### Extract model results and display data for top 3 values using Cook’s distance


```
model.data <- augment(m) %>% 
  mutate(index = 1:n()) 
model.data %>% top_n(3, .cooksd)
```

#### plot standardised residuals


```
ggplot(model.data, aes(index, .std.resid)) + 
  geom_point(aes(color = Health_Binary), alpha = .5) +
  theme_bw()
```

#### Filter potential influential data points with abs(.std.res) > 3:


```
model.data %>% 
  filter(abs(.std.resid) > 3)
```

## DOG NEUTER binary logistic regression ON SIGNIFICANT OR SERIOUS ILLNESS


```
# fit binary logit model and store results 'm'
m <- glm(Health_Binary ~ D_Neuter, data = ml,family = binomial)
# view a summary of the model
summary(m)
# test model fit
with(m, null.deviance - deviance)
with(m, df.null - df.residual)
with(m, pchisq(null.deviance - deviance, df.null - df.residual, lower.tail = FALSE))
BIC(m)
## CIs using profiled log-likelihood
confint(m, level=0.99)
## CIs using standard errors
confint.default(m, level=0.99)
# Wald test
wald.test(b = coef(m), Sigma = vcov(m), Terms = 2)
## odds ratios and 95% CI
exp(cbind(OR = coef(m), confint(m, level=0.99)))
```


### Create ROCR from data


```
## training data
pred.mtt = predict(m, type = "response") #repeat risk predictions from model m
rocr.pred.mtt = ROCR::prediction(pred.mtt, labels = ml$Health_Binary) #ROCR prediction object
roc.perf.mtt = ROCR::performance(rocr.pred.mtt, measure = "tpr", x.measure = "fpr") # #ROCR performance object
plot(roc.perf.mtt, col = "blue")
abline(a = 0, b = 1, lty = 2) #diagonal for random assignment
```

### Report AUC from ROC for training and test data


```
  # Train AUC
auc <- ROCR::performance(rocr.pred.mtt, measure = "auc")
  auc <- auc@y.values[[1]]
  print(auc)
```

### Calculate Nagelkerke R^2


```
NagelkerkeR2(m)
```

### check assumptions of model

#### Cook’s distance


```
plot(m, which = 4, id.n = 3)
```

#### Extract model results and display data for top 3 values using Cook’s distance


```
model.data <- augment(m) %>% 
  mutate(index = 1:n()) 
model.data %>% top_n(3, .cooksd)
```

#### plot standardised residuals


```
ggplot(model.data, aes(index, .std.resid)) + 
  geom_point(aes(color = Health_Binary), alpha = .5) +
  theme_bw()
```

#### Filter potential influential data points with abs(.std.res) > 3:


```
model.data %>% 
  filter(abs(.std.resid) > 3)
```

## D\_SEX + D\_NEUTER Binary logistic regression ON SIGNIFICANT OR SERIOUS ILLNESS


```
# fit binary logit model and store results 'm'
m <- glm(Health_Binary ~ D_Sex + D_Neuter, data = ml,family = binomial)
# view a summary of the model
summary(m)
# test model fit
with(m, null.deviance - deviance)
with(m, df.null - df.residual)
with(m, pchisq(null.deviance - deviance, df.null - df.residual, lower.tail = FALSE))
BIC(m)
# Hosmer-Lemeshow Goodness-of-Fit Test
hltest(m)
## CIs using profiled log-likelihood
confint(m, level=0.99)
## CIs using standard errors
confint.default(m, level=0.99)
# Wald test
wald.test(b = coef(m), Sigma = vcov(m), Terms = 2)
## odds ratios and 95% CI
exp(cbind(OR = coef(m), confint(m, level=0.99)))
```


### Create ROCR from data


```
## training data
pred.mtt = predict(m, type = "response") #repeat risk predictions from model m
rocr.pred.mtt = ROCR::prediction(pred.mtt, labels = ml$Health_Binary) #ROCR prediction object
roc.perf.mtt = ROCR::performance(rocr.pred.mtt, measure = "tpr", x.measure = "fpr") # #ROCR performance object
plot(roc.perf.mtt, col = "blue")
abline(a = 0, b = 1, lty = 2) #diagonal for random assignment
```

### Report AUC from ROC for training and test data


```
  # Train AUC
auc <- ROCR::performance(rocr.pred.mtt, measure = "auc")
  auc <- auc@y.values[[1]]
  print(auc)
```

### Calculate Nagelkerke R^2


```
NagelkerkeR2(m)
```

### check assumptions of model

#### Cook’s distance


```
plot(m, which = 4, id.n = 3)
```

#### Extract model results and display data for top 3 values using Cook’s distance


```
model.data <- augment(m) %>% 
  mutate(index = 1:n()) 
model.data %>% top_n(3, .cooksd)
```

#### plot standardised residuals


```
ggplot(model.data, aes(index, .std.resid)) + 
  geom_point(aes(color = Health_Binary), alpha = .5) +
  theme_bw()
```

#### Filter potential influential data points with abs(.std.res) > 3:


```
model.data %>% 
  filter(abs(.std.resid) > 3)
```

#### check for multicollinearity


```
car::vif(m)
```

## D\_SEX \* D\_NEUTER Binary logistic regression ON SIGNIFICANT OR SERIOUS ILLNESS


```
# fit binary logit model and store results 'm'
m <- glm(Health_Binary ~ D_Sex*D_Neuter, data = ml,family = binomial)
# view a summary of the model
summary(m)
# test model fit
with(m, null.deviance - deviance)
with(m, df.null - df.residual)
with(m, pchisq(null.deviance - deviance, df.null - df.residual, lower.tail = FALSE))
BIC(m)
# Hosmer-Lemeshow Goodness-of-Fit Test
hltest(m)
## CIs using profiled log-likelihood
confint(m, level=0.99)
## CIs using standard errors
confint.default(m, level=0.99)
# Wald test
wald.test(b = coef(m), Sigma = vcov(m), Terms = 2)
## odds ratios and 95% CI
exp(cbind(OR = coef(m), confint(m, level=0.99)))
```


### Create ROCR from data


```
## training data
pred.mtt = predict(m, type = "response") #repeat risk predictions from model m
rocr.pred.mtt = ROCR::prediction(pred.mtt, labels = ml$Health_Binary) #ROCR prediction object
roc.perf.mtt = ROCR::performance(rocr.pred.mtt, measure = "tpr", x.measure = "fpr") # #ROCR performance object
plot(roc.perf.mtt, col = "blue")
abline(a = 0, b = 1, lty = 2) #diagonal for random assignment
```

### Report AUC from ROC for training and test data


```
  # Train AUC
auc <- ROCR::performance(rocr.pred.mtt, measure = "auc")
  auc <- auc@y.values[[1]]
  print(auc)
```

### Calculate Nagelkerke R^2


```
NagelkerkeR2(m)
```

### check assumptions of model

#### Cook’s distance


```
plot(m, which = 4, id.n = 3)
```

#### Extract model results and display data for top 3 values using Cook’s distance


```
model.data <- augment(m) %>% 
  mutate(index = 1:n()) 
model.data %>% top_n(3, .cooksd)
```

# plot standardised residuals


```
ggplot(model.data, aes(index, .std.resid)) + 
  geom_point(aes(color = Health_Binary), alpha = .5) +
  theme_bw()
```

# Filter potential influential data points with abs(.std.res) > 3:


```
model.data %>% 
  filter(abs(.std.resid) > 3)
```


#### check for multicollinearity


```
car::vif(m)
```

# CHECK EFFECT OF DOG HEALTH CHARACTERISTICS ON SIGNIFICANT OR SERIOUS ILLNESS - simple binary regression

## THERAPEUTIC FOOD Binary logistic regression ON SIGNIFICANT OR SERIOUS ILLNESS


```
# fit binary logit model and store results 'm'
m <- glm(Health_Binary ~ Therapeutic_Food, data = ml,family = binomial)
# view a summary of the model
summary(m)
# test model fit
with(m, null.deviance - deviance)
with(m, df.null - df.residual)
with(m, pchisq(null.deviance - deviance, df.null - df.residual, lower.tail = FALSE))
BIC(m)

## CIs using profiled log-likelihood
confint(m, level=0.99)
## CIs using standard errors
confint.default(m, level=0.99)
# Wald test
wald.test(b = coef(m), Sigma = vcov(m), Terms = 2)
## odds ratios and 95% CI
exp(cbind(OR = coef(m), confint(m, level=0.99)))
```


### Create ROCR from data


```
## training data
pred.mtt = predict(m, type = "response") #repeat risk predictions from model m
rocr.pred.mtt = ROCR::prediction(pred.mtt, labels = ml$Health_Binary) #ROCR prediction object
roc.perf.mtt = ROCR::performance(rocr.pred.mtt, measure = "tpr", x.measure = "fpr") # #ROCR performance object
plot(roc.perf.mtt, col = "blue")
abline(a = 0, b = 1, lty = 2) #diagonal for random assignment
```

### Report AUC from ROC for training and test data


```
  # Train AUC
auc <- ROCR::performance(rocr.pred.mtt, measure = "auc")
  auc <- auc@y.values[[1]]
  print(auc)
```

### Calculate Nagelkerke R^2


```
NagelkerkeR2(m)
```

### check assumptions of model

#### Cook’s distance


```
plot(m, which = 4, id.n = 3)
```

#### Extract model results and display data for top 3 values using Cook’s distance


```
model.data <- augment(m) %>% 
  mutate(index = 1:n()) 
model.data %>% top_n(3, .cooksd)
```

#### plot standardised residuals


```
ggplot(model.data, aes(index, .std.resid)) + 
  geom_point(aes(color = Health_Binary), alpha = .5) +
  theme_bw()
```

#### Filter potential influential data points with abs(.std.res) > 3:


```
model.data %>% 
  filter(abs(.std.resid) > 3)
```

## VISITS Binary logistic regression ON SIGNIFICANT OR SERIOUS ILLNESS


```
# fit binary logit model and store results 'm'
m <- glm(Health_Binary ~ Visits, data = ml,family = binomial)
# view a summary of the model
summary(m)
# test model fit
with(m, null.deviance - deviance)
with(m, df.null - df.residual)
with(m, pchisq(null.deviance - deviance, df.null - df.residual, lower.tail = FALSE))
BIC(m)
# Hosmer-Lemeshow Goodness-of-Fit Test
hltest(m)
## CIs using profiled log-likelihood
confint(m, level=0.99)
## CIs using standard errors
confint.default(m, level=0.99)
# Wald test
wald.test(b = coef(m), Sigma = vcov(m), Terms = 2)
## odds ratios and 95% CI
exp(cbind(OR = coef(m), confint(m, level=0.99)))
```


### Create ROCR from data


```
## training data
pred.mtt = predict(m, type = "response") #repeat risk predictions from model m
rocr.pred.mtt = ROCR::prediction(pred.mtt, labels = ml$Health_Binary) #ROCR prediction object
roc.perf.mtt = ROCR::performance(rocr.pred.mtt, measure = "tpr", x.measure = "fpr") # #ROCR performance object
plot(roc.perf.mtt, col = "blue")
abline(a = 0, b = 1, lty = 2) #diagonal for random assignment
```

### Report AUC from ROC for training and test data


```
  # Train AUC
auc <- ROCR::performance(rocr.pred.mtt, measure = "auc")
  auc <- auc@y.values[[1]]
  print(auc)
```

### Calculate Nagelkerke R^2


```
NagelkerkeR2(m)
```

### check assumptions of model

#### Cook’s distance


```
plot(m, which = 4, id.n = 3)
```

#### Extract model results and display data for top 3 values using Cook’s distance


```
model.data <- augment(m) %>% 
  mutate(index = 1:n()) 
model.data %>% top_n(3, .cooksd)
```

#### plot standardised residuals


```
ggplot(model.data, aes(index, .std.resid)) + 
  geom_point(aes(color = Health_Binary), alpha = .5) +
  theme_bw()
```

#### Filter potential influential data points with abs(.std.res) > 3:


```
model.data %>% 
  filter(abs(.std.resid) > 3)
```

## VISITS2 Binary logistic regression for HEALTH

#### Note Visits better fit than Visits2


```
# fit binary logit model and store results 'm'
m <- glm(Health_Binary ~ Visits2, data = ml,family = binomial)
# view a summary of the model
summary(m)
# test model fit
with(m, null.deviance - deviance)
with(m, df.null - df.residual)
with(m, pchisq(null.deviance - deviance, df.null - df.residual, lower.tail = FALSE))
BIC(m)
# Hosmer-Lemeshow Goodness-of-Fit Test
hltest(m)
## CIs using profiled log-likelihood
confint(m, level=0.99)
## CIs using standard errors
confint.default(m, level=0.99)
# Wald test
wald.test(b = coef(m), Sigma = vcov(m), Terms = 2)
## odds ratios and 95% CI
exp(cbind(OR = coef(m), confint(m, level=0.99)))
```

### Create ROCR from data


```
## training data
pred.mtt = predict(m, type = "response") #repeat risk predictions from model m
rocr.pred.mtt = ROCR::prediction(pred.mtt, labels = ml$Health_Binary) #ROCR prediction object
roc.perf.mtt = ROCR::performance(rocr.pred.mtt, measure = "tpr", x.measure = "fpr") # #ROCR performance object
plot(roc.perf.mtt, col = "blue")
abline(a = 0, b = 1, lty = 2) #diagonal for random assignment
```

### Report AUC from ROC for training and test data


```
  # Train AUC
auc <- ROCR::performance(rocr.pred.mtt, measure = "auc")
  auc <- auc@y.values[[1]]
  print(auc)
```

### Calculate Nagelkerke R^2


```
NagelkerkeR2(m)
```

### check assumptions of model

#### Cook’s distance


```
plot(m, which = 4, id.n = 3)
```

#### Extract model results and display data for top 3 values using Cook’s distance


```
model.data <- augment(m) %>% 
  mutate(index = 1:n()) 
model.data %>% top_n(3, .cooksd)
```

#### plot standardised residuals


```
ggplot(model.data, aes(index, .std.resid)) + 
  geom_point(aes(color = Health_Binary), alpha = .5) +
  theme_bw()
```

#### Filter potential influential data points with abs(.std.res) > 3:


```
model.data %>% 
  filter(abs(.std.resid) > 3)
```

## MEDS Binary logistic regression for HEALTH


```
# fit binary logit model and store results 'm'
m <- glm(Health_Binary ~ Meds, data = ml,family = binomial)
# view a summary of the model
summary(m)
# test model fit
with(m, null.deviance - deviance)
with(m, df.null - df.residual)
with(m, pchisq(null.deviance - deviance, df.null - df.residual, lower.tail = FALSE))
BIC(m)
# Hosmer-Lemeshow Goodness-of-Fit Test

## CIs using profiled log-likelihood
confint(m, level=0.99)
## CIs using standard errors
confint.default(m, level=0.99)
# Wald test
wald.test(b = coef(m), Sigma = vcov(m), Terms = 2)
## odds ratios and 95% CI
exp(cbind(OR = coef(m), confint(m, level=0.99)))
```


### Create ROCR from data


```
## training data
pred.mtt = predict(m, type = "response") #repeat risk predictions from model m
rocr.pred.mtt = ROCR::prediction(pred.mtt, labels = ml$Health_Binary) #ROCR prediction object
roc.perf.mtt = ROCR::performance(rocr.pred.mtt, measure = "tpr", x.measure = "fpr") # #ROCR performance object
plot(roc.perf.mtt, col = "blue")
abline(a = 0, b = 1, lty = 2) #diagonal for random assignment
```

### Report AUC from ROC for training and test data


```
  # Train AUC
auc <- ROCR::performance(rocr.pred.mtt, measure = "auc")
  auc <- auc@y.values[[1]]
  print(auc)
```

### Calculate Nagelkerke R^2


```
NagelkerkeR2(m)
```

### check assumptions of model

#### Cook’s distance


```
plot(m, which = 4, id.n = 3)
```

#### Extract model results and display data for top 3 values using Cook’s distance


```
model.data <- augment(m) %>% 
  mutate(index = 1:n()) 
model.data %>% top_n(3, .cooksd)
```

#### plot standardised residuals


```
ggplot(model.data, aes(index, .std.resid)) + 
  geom_point(aes(color = Health_Binary), alpha = .5) +
  theme_bw()
```

#### Filter potential influential data points with abs(.std.res) > 3:


```
model.data %>% 
  filter(abs(.std.resid) > 3)
```

LS0tCnRpdGxlOiAiUiBzdGF0cyBmb3IgYmluYXJ5IGxvZ2lzdGljIHJlZ3Jlc3Npb24gb24gU0VSSU9VUyBJTExORVNTIG91dGNvbWUgdmFyaWlhYmxlIGZvciB0aGUgYWxsLW93bmVyIGRhdGFzZXQiCmF1dGhvcjogIkFsZXggR2VybWFuIgpkYXRlOiAiNyBEZWNlbWJlciAyMDIzIgpvdXRwdXQ6CiAgaHRtbF9kb2N1bWVudDoKICAgIGRmX3ByaW50OiBwYWdlZAogIHdvcmRfZG9jdW1lbnQ6IGRlZmF1bHQKICBodG1sX25vdGVib29rOiBkZWZhdWx0Ci0tLQoKCiMgQ3JlYXRlIGRhdGEgZnJhbWUgZm9yIGFuYWx5c2lzCiMjIyBOQiBuZWVkIHRvIHJ1biAiUmVhZF9kYXRhXzEwMi5SdG0iIGZpcnN0IHRvIGNyZWF0ZSBkYXRhc2V0CmBgYHtyIGluY2x1ZGU9RkFMU0V9CmtuaXRyOjprbml0KCJSZWFkX2RhdGFfMTAyLnJtZCIpCmBgYAoKCiMjIENoaSBzcXVhcmVkIHRlc3Qgb2YgSGVhbHRoX0JpbmFyeSBhbmQgUHJpbWFyeV9EZWNpc2lvbl9NYWtlcgpgYGB7ciBJNTh4fQp0YWJsZShtbCRIZWFsdGhfQmluYXJ5LCBtbCRQcmltYXJ5X0RlY2lzaW9uX01ha2VyKQpjaGlzcS50ZXN0KG1sJEhlYWx0aF9CaW5hcnksIG1sJFByaW1hcnlfRGVjaXNpb25fTWFrZXIsIGNvcnJlY3Q9RkFMU0UpCmBgYAoKCgoKIyBCSU5BUlkgTE9HSVNUSUMgUkVHUkVTU0lPTiBPTiBTSUdOSUZJQ0FOVCBPUiBTRVJJT1VTIElMTE5FU1MKIyMgQ0hFQ0sgRUZGRUNUIE9GIE9XTkVSIENIQVJBQ1RFUklTVElDUyAtIHNpbXBsZSBiaW5hcnkgbG9naXN0aWMgcmVncmVzc2lvbgoKIyMgREVDSVNJT04gTUFLRVIgQmluYXJ5IHJlZ3Jlc3Npb24gZm9yIEFueV9IZWFsdGhfUHJvYmxlbQpgYGB7ciA5Z0R9CiMgZml0IGJpbmFyeSBsb2dpdCBtb2RlbCBhbmQgc3RvcmUgcmVzdWx0cyAnbScKbSA8LSBnbG0oSGVhbHRoX0JpbmFyeSB+IFByaW1hcnlfRGVjaXNpb25fTWFrZXIsIGRhdGEgPSBtbCxmYW1pbHkgPSBiaW5vbWlhbCkKIyB2aWV3IGEgc3VtbWFyeSBvZiB0aGUgbW9kZWwKc3VtbWFyeShtKQojIHRlc3QgbW9kZWwgZml0CndpdGgobSwgbnVsbC5kZXZpYW5jZSAtIGRldmlhbmNlKQp3aXRoKG0sIGRmLm51bGwgLSBkZi5yZXNpZHVhbCkKd2l0aChtLCBwY2hpc3EobnVsbC5kZXZpYW5jZSAtIGRldmlhbmNlLCBkZi5udWxsIC0gZGYucmVzaWR1YWwsIGxvd2VyLnRhaWwgPSBGQUxTRSkpCkJJQyhtKQojIyBDSXMgdXNpbmcgcHJvZmlsZWQgbG9nLWxpa2VsaWhvb2QKY29uZmludChtLCBsZXZlbD0wLjk5KQojIyBDSXMgdXNpbmcgc3RhbmRhcmQgZXJyb3JzCmNvbmZpbnQuZGVmYXVsdChtLCBsZXZlbD0wLjk5KQojIFdhbGQgdGVzdAp3YWxkLnRlc3QoYiA9IGNvZWYobSksIFNpZ21hID0gdmNvdihtKSwgVGVybXMgPSAyKQojIyBvZGRzIHJhdGlvcyBhbmQgOTUlIENJCmV4cChjYmluZChPUiA9IGNvZWYobSksIGNvbmZpbnQobSwgbGV2ZWw9MC45OSkpKQpgYGAKCgojIyMgQ3JlYXRlIFJPQ1IgZnJvbSBkYXRhCmBgYHtyIFJPQzF9CiMjIHRyYWluaW5nIGRhdGEKcHJlZC5tdHQgPSBwcmVkaWN0KG0sIHR5cGUgPSAicmVzcG9uc2UiKSAjcmVwZWF0IHJpc2sgcHJlZGljdGlvbnMgZnJvbSBtb2RlbCBtCnJvY3IucHJlZC5tdHQgPSBST0NSOjpwcmVkaWN0aW9uKHByZWQubXR0LCBsYWJlbHMgPSBtbCRIZWFsdGhfQmluYXJ5KSAjUk9DUiBwcmVkaWN0aW9uIG9iamVjdApyb2MucGVyZi5tdHQgPSBST0NSOjpwZXJmb3JtYW5jZShyb2NyLnByZWQubXR0LCBtZWFzdXJlID0gInRwciIsIHgubWVhc3VyZSA9ICJmcHIiKSAjICNST0NSIHBlcmZvcm1hbmNlIG9iamVjdApwbG90KHJvYy5wZXJmLm10dCwgY29sID0gImJsdWUiKQphYmxpbmUoYSA9IDAsIGIgPSAxLCBsdHkgPSAyKSAjZGlhZ29uYWwgZm9yIHJhbmRvbSBhc3NpZ25tZW50CmBgYAoKIyMjIFJlcG9ydCBBVUMgZnJvbSBST0MgZm9yIHRyYWluaW5nIGFuZCB0ZXN0IGRhdGEKYGBge3IgQVVDMX0KICAjIFRyYWluIEFVQwphdWMgPC0gUk9DUjo6cGVyZm9ybWFuY2Uocm9jci5wcmVkLm10dCwgbWVhc3VyZSA9ICJhdWMiKQogIGF1YyA8LSBhdWNAeS52YWx1ZXNbWzFdXQogIHByaW50KGF1YykKYGBgCgoKCgojIyMgQ2FsY3VsYXRlIE5hZ2Vsa2Vya2UgUl4yCmBgYHtyIDlhZ3p9Ck5hZ2Vsa2Vya2VSMihtKQpgYGAKCiMjIyBjaGVjayBhc3N1bXB0aW9ucyBvZiBtb2RlbAojIyMjIENvb2sncyBkaXN0YW5jZQpgYGB7ciAxMGd6fQpwbG90KG0sIHdoaWNoID0gNCwgaWQubiA9IDMpCmBgYAoKIyMjIyBFeHRyYWN0IG1vZGVsIHJlc3VsdHMgYW5kIGRpc3BsYXkgZGF0YSBmb3IgdG9wIDMgdmFsdWVzIHVzaW5nIENvb2sncyBkaXN0YW5jZQpgYGB7ciAxMWd6fQptb2RlbC5kYXRhIDwtIGF1Z21lbnQobSkgJT4lIAogIG11dGF0ZShpbmRleCA9IDE6bigpKSAKbW9kZWwuZGF0YSAlPiUgdG9wX24oMywgLmNvb2tzZCkKYGBgCgojIyMjIHBsb3Qgc3RhbmRhcmRpc2VkIHJlc2lkdWFscwpgYGB7ciAxMmd6fQpnZ3Bsb3QobW9kZWwuZGF0YSwgYWVzKGluZGV4LCAuc3RkLnJlc2lkKSkgKyAKICBnZW9tX3BvaW50KGFlcyhjb2xvciA9IEhlYWx0aF9CaW5hcnkpLCBhbHBoYSA9IC41KSArCiAgdGhlbWVfYncoKQpgYGAKCiMjIyMgRmlsdGVyIHBvdGVudGlhbCBpbmZsdWVudGlhbCBkYXRhIHBvaW50cyB3aXRoIGFicyguc3RkLnJlcykgPiAzOgpgYGB7ciAxM2d6fQptb2RlbC5kYXRhICU+JSAKICBmaWx0ZXIoYWJzKC5zdGQucmVzaWQpID4gMykKYGBgCgoKCgojIyBDTElFTlQgRElFVCBiaW5hcnkgcmVncmVzc2lvbiBPTiBTSUdOSUZJQ0FOVCBPUiBTRVJJT1VTIElMTE5FU1MKYGBge3IgOWd9CiMgZml0IGJpbmFyeSBsb2dpdCBtb2RlbCBhbmQgc3RvcmUgcmVzdWx0cyAnbScKbSA8LSBnbG0oSGVhbHRoX0JpbmFyeSB+IENfRGlldCwgZGF0YSA9IG1sLGZhbWlseSA9IGJpbm9taWFsKQojIHZpZXcgYSBzdW1tYXJ5IG9mIHRoZSBtb2RlbApzdW1tYXJ5KG0pCiMgdGVzdCBtb2RlbCBmaXQKd2l0aChtLCBudWxsLmRldmlhbmNlIC0gZGV2aWFuY2UpCndpdGgobSwgZGYubnVsbCAtIGRmLnJlc2lkdWFsKQp3aXRoKG0sIHBjaGlzcShudWxsLmRldmlhbmNlIC0gZGV2aWFuY2UsIGRmLm51bGwgLSBkZi5yZXNpZHVhbCwgbG93ZXIudGFpbCA9IEZBTFNFKSkKQklDKG0pCiMjIENJcyB1c2luZyBwcm9maWxlZCBsb2ctbGlrZWxpaG9vZApjb25maW50KG0sIGxldmVsPTAuOTkpCiMjIENJcyB1c2luZyBzdGFuZGFyZCBlcnJvcnMKY29uZmludC5kZWZhdWx0KG0sIGxldmVsPTAuOTkpCiMgV2FsZCB0ZXN0CndhbGQudGVzdChiID0gY29lZihtKSwgU2lnbWEgPSB2Y292KG0pLCBUZXJtcyA9IDIpCiMjIG9kZHMgcmF0aW9zIGFuZCA5NSUgQ0kKZXhwKGNiaW5kKE9SID0gY29lZihtKSwgY29uZmludChtLCBsZXZlbD0wLjk5KSkpCmBgYAojIyMgQ3JlYXRlIFJPQ1IgZnJvbSBkYXRhCmBgYHtyIFJPQzJ9CiMjIHRyYWluaW5nIGRhdGEKcHJlZC5tdHQgPSBwcmVkaWN0KG0sIHR5cGUgPSAicmVzcG9uc2UiKSAjcmVwZWF0IHJpc2sgcHJlZGljdGlvbnMgZnJvbSBtb2RlbCBtCnJvY3IucHJlZC5tdHQgPSBST0NSOjpwcmVkaWN0aW9uKHByZWQubXR0LCBsYWJlbHMgPSBtbCRIZWFsdGhfQmluYXJ5KSAjUk9DUiBwcmVkaWN0aW9uIG9iamVjdApyb2MucGVyZi5tdHQgPSBST0NSOjpwZXJmb3JtYW5jZShyb2NyLnByZWQubXR0LCBtZWFzdXJlID0gInRwciIsIHgubWVhc3VyZSA9ICJmcHIiKSAjICNST0NSIHBlcmZvcm1hbmNlIG9iamVjdApwbG90KHJvYy5wZXJmLm10dCwgY29sID0gImJsdWUiKQphYmxpbmUoYSA9IDAsIGIgPSAxLCBsdHkgPSAyKSAjZGlhZ29uYWwgZm9yIHJhbmRvbSBhc3NpZ25tZW50CmBgYAoKIyMjIFJlcG9ydCBBVUMgZnJvbSBST0MgZm9yIHRyYWluaW5nIGFuZCB0ZXN0IGRhdGEKYGBge3IgQVVDMn0KICAjIFRyYWluIEFVQwphdWMgPC0gUk9DUjo6cGVyZm9ybWFuY2Uocm9jci5wcmVkLm10dCwgbWVhc3VyZSA9ICJhdWMiKQogIGF1YyA8LSBhdWNAeS52YWx1ZXNbWzFdXQogIHByaW50KGF1YykKYGBgCgoKCiMjIyBDYWxjdWxhdGUgTmFnZWxrZXJrZSBSXjIKYGBge3IgOWFnfQpOYWdlbGtlcmtlUjIobSkKYGBgCgojIyMgY2hlY2sgYXNzdW1wdGlvbnMgb2YgbW9kZWwKIyMjIyBDb29rJ3MgZGlzdGFuY2UKYGBge3IgMTBnfQpwbG90KG0sIHdoaWNoID0gNCwgaWQubiA9IDMpCmBgYAoKIyMjIyBFeHRyYWN0IG1vZGVsIHJlc3VsdHMgYW5kIGRpc3BsYXkgZGF0YSBmb3IgdG9wIDMgdmFsdWVzIHVzaW5nIENvb2sncyBkaXN0YW5jZQpgYGB7ciAxMWd9Cm1vZGVsLmRhdGEgPC0gYXVnbWVudChtKSAlPiUgCiAgbXV0YXRlKGluZGV4ID0gMTpuKCkpIAptb2RlbC5kYXRhICU+JSB0b3BfbigzLCAuY29va3NkKQpgYGAKCiMjIyMgcGxvdCBzdGFuZGFyZGlzZWQgcmVzaWR1YWxzCmBgYHtyIDFnMn0KZ2dwbG90KG1vZGVsLmRhdGEsIGFlcyhpbmRleCwgLnN0ZC5yZXNpZCkpICsgCiAgZ2VvbV9wb2ludChhZXMoY29sb3IgPSBIZWFsdGhfQmluYXJ5KSwgYWxwaGEgPSAuNSkgKwogIHRoZW1lX2J3KCkKYGBgCgojIyMjIEZpbHRlciBwb3RlbnRpYWwgaW5mbHVlbnRpYWwgZGF0YSBwb2ludHMgd2l0aCBhYnMoLnN0ZC5yZXMpID4gMzoKYGBge3IgMTNnfQptb2RlbC5kYXRhICU+JSAKICBmaWx0ZXIoYWJzKC5zdGQucmVzaWQpID4gMykKYGBgCgojIyMjIENhbGN1bGF0ZSBOYWdlbGtlcmtlIFJeMgpgYGB7ciBnfQpOYWdlbGtlcmtlUjIobSkKYGBgCgoKCiMjIENMSUVOVCBESUVUIFZFR0FOIGJpbmFyeSByZWdyZXNzaW9uIE9OIFNJR05JRklDQU5UIE9SIFNFUklPVVMgSUxMTkVTUwpgYGB7ciA5fQojIGZpdCBiaW5hcnkgbG9naXQgbW9kZWwgYW5kIHN0b3JlIHJlc3VsdHMgJ20nCm0gPC0gZ2xtKEhlYWx0aF9CaW5hcnkgfiBDX0RpZXRfVmVnYW4sIGRhdGEgPSBtbCxmYW1pbHkgPSBiaW5vbWlhbCkKIyB2aWV3IGEgc3VtbWFyeSBvZiB0aGUgbW9kZWwKc3VtbWFyeShtKQojIHRlc3QgbW9kZWwgZml0CndpdGgobSwgbnVsbC5kZXZpYW5jZSAtIGRldmlhbmNlKQp3aXRoKG0sIGRmLm51bGwgLSBkZi5yZXNpZHVhbCkKd2l0aChtLCBwY2hpc3EobnVsbC5kZXZpYW5jZSAtIGRldmlhbmNlLCBkZi5udWxsIC0gZGYucmVzaWR1YWwsIGxvd2VyLnRhaWwgPSBGQUxTRSkpCkJJQyhtKQojIyBDSXMgdXNpbmcgcHJvZmlsZWQgbG9nLWxpa2VsaWhvb2QKY29uZmludChtLCBsZXZlbD0wLjk5KQojIyBDSXMgdXNpbmcgc3RhbmRhcmQgZXJyb3JzCmNvbmZpbnQuZGVmYXVsdChtLCBsZXZlbD0wLjk5KQojIFdhbGQgdGVzdAp3YWxkLnRlc3QoYiA9IGNvZWYobSksIFNpZ21hID0gdmNvdihtKSwgVGVybXMgPSAyKQojIyBvZGRzIHJhdGlvcyBhbmQgOTUlIENJCmV4cChjYmluZChPUiA9IGNvZWYobSksIGNvbmZpbnQobSwgbGV2ZWw9MC45OSkpKQpgYGAKCiMjIyBDcmVhdGUgUk9DUiBmcm9tIGRhdGEKYGBge3IgUk9DM30KIyMgdHJhaW5pbmcgZGF0YQpwcmVkLm10dCA9IHByZWRpY3QobSwgdHlwZSA9ICJyZXNwb25zZSIpICNyZXBlYXQgcmlzayBwcmVkaWN0aW9ucyBmcm9tIG1vZGVsIG0Kcm9jci5wcmVkLm10dCA9IFJPQ1I6OnByZWRpY3Rpb24ocHJlZC5tdHQsIGxhYmVscyA9IG1sJEhlYWx0aF9CaW5hcnkpICNST0NSIHByZWRpY3Rpb24gb2JqZWN0CnJvYy5wZXJmLm10dCA9IFJPQ1I6OnBlcmZvcm1hbmNlKHJvY3IucHJlZC5tdHQsIG1lYXN1cmUgPSAidHByIiwgeC5tZWFzdXJlID0gImZwciIpICMgI1JPQ1IgcGVyZm9ybWFuY2Ugb2JqZWN0CnBsb3Qocm9jLnBlcmYubXR0LCBjb2wgPSAiYmx1ZSIpCmFibGluZShhID0gMCwgYiA9IDEsIGx0eSA9IDIpICNkaWFnb25hbCBmb3IgcmFuZG9tIGFzc2lnbm1lbnQKYGBgCgojIyMgUmVwb3J0IEFVQyBmcm9tIFJPQyBmb3IgdHJhaW5pbmcgYW5kIHRlc3QgZGF0YQpgYGB7ciBBVUMzfQogICMgVHJhaW4gQVVDCmF1YyA8LSBST0NSOjpwZXJmb3JtYW5jZShyb2NyLnByZWQubXR0LCBtZWFzdXJlID0gImF1YyIpCiAgYXVjIDwtIGF1Y0B5LnZhbHVlc1tbMV1dCiAgcHJpbnQoYXVjKQpgYGAKCgojIyMgQ2FsY3VsYXRlIE5hZ2Vsa2Vya2UgUl4yCmBgYHtyIDlhfQpOYWdlbGtlcmtlUjIobSkKYGBgCgojIyMgY2hlY2sgYXNzdW1wdGlvbnMgb2YgbW9kZWwKIyMjIyBDb29rJ3MgZGlzdGFuY2UKYGBge3IgMTB9CnBsb3QobSwgd2hpY2ggPSA0LCBpZC5uID0gMykKYGBgCgojIyMjIEV4dHJhY3QgbW9kZWwgcmVzdWx0cyBhbmQgZGlzcGxheSBkYXRhIGZvciB0b3AgMyB2YWx1ZXMgdXNpbmcgQ29vaydzIGRpc3RhbmNlCmBgYHtyIDExfQptb2RlbC5kYXRhIDwtIGF1Z21lbnQobSkgJT4lIAogIG11dGF0ZShpbmRleCA9IDE6bigpKSAKbW9kZWwuZGF0YSAlPiUgdG9wX24oMywgLmNvb2tzZCkKYGBgCgojIyMjIHBsb3Qgc3RhbmRhcmRpc2VkIHJlc2lkdWFscwpgYGB7ciAxMn0KZ2dwbG90KG1vZGVsLmRhdGEsIGFlcyhpbmRleCwgLnN0ZC5yZXNpZCkpICsgCiAgZ2VvbV9wb2ludChhZXMoY29sb3IgPSBIZWFsdGhfQmluYXJ5KSwgYWxwaGEgPSAuNSkgKwogIHRoZW1lX2J3KCkKYGBgCgojIyMjIEZpbHRlciBwb3RlbnRpYWwgaW5mbHVlbnRpYWwgZGF0YSBwb2ludHMgd2l0aCBhYnMoLnN0ZC5yZXMpID4gMzoKYGBge3IgMTN9Cm1vZGVsLmRhdGEgJT4lIAogIGZpbHRlcihhYnMoLnN0ZC5yZXNpZCkgPiAzKQpgYGAKCiMjIyMgQ2FsY3VsYXRlIE5hZ2Vsa2Vya2UgUl4yCmBgYHtyfQpOYWdlbGtlcmtlUjIobSkKYGBgCgoKCiMjIENfRGlldF9WZWdhbl9WZWdnaWUgQmluYXJ5IGxvZ2lzdGljIHJlZ3Jlc3Npb24gZm9yIEhFQUxUSApgYGB7ciBJODR9CiMgZml0IGJpbmFyeSBsb2dpdCBtb2RlbCBhbmQgc3RvcmUgcmVzdWx0cyAnbScKbSA8LSBnbG0oSGVhbHRoX0JpbmFyeSB+IENfRGlldF9WZWdhbl9WZWdnaWUsIGRhdGEgPSBtbCxmYW1pbHkgPSBiaW5vbWlhbCkKIyB2aWV3IGEgc3VtbWFyeSBvZiB0aGUgbW9kZWwKc3VtbWFyeShtKQojIHRlc3QgbW9kZWwgZml0CndpdGgobSwgbnVsbC5kZXZpYW5jZSAtIGRldmlhbmNlKQp3aXRoKG0sIGRmLm51bGwgLSBkZi5yZXNpZHVhbCkKd2l0aChtLCBwY2hpc3EobnVsbC5kZXZpYW5jZSAtIGRldmlhbmNlLCBkZi5udWxsIC0gZGYucmVzaWR1YWwsIGxvd2VyLnRhaWwgPSBGQUxTRSkpCkJJQyhtKQojIyBDSXMgdXNpbmcgcHJvZmlsZWQgbG9nLWxpa2VsaWhvb2QKY29uZmludChtLCBsZXZlbD0wLjk5KQojIyBDSXMgdXNpbmcgc3RhbmRhcmQgZXJyb3JzCmNvbmZpbnQuZGVmYXVsdChtLCBsZXZlbD0wLjk5KQojIFdhbGQgdGVzdAp3YWxkLnRlc3QoYiA9IGNvZWYobSksIFNpZ21hID0gdmNvdihtKSwgVGVybXMgPSAyKQojIyBvZGRzIHJhdGlvcyBhbmQgOTUlIENJCmV4cChjYmluZChPUiA9IGNvZWYobSksIGNvbmZpbnQobSwgbGV2ZWw9MC45OSkpKQpgYGAKCiMjIyBDcmVhdGUgUk9DUiBmcm9tIGRhdGEKYGBge3IgUk9DNH0KIyMgdHJhaW5pbmcgZGF0YQpwcmVkLm10dCA9IHByZWRpY3QobSwgdHlwZSA9ICJyZXNwb25zZSIpICNyZXBlYXQgcmlzayBwcmVkaWN0aW9ucyBmcm9tIG1vZGVsIG0Kcm9jci5wcmVkLm10dCA9IFJPQ1I6OnByZWRpY3Rpb24ocHJlZC5tdHQsIGxhYmVscyA9IG1sJEhlYWx0aF9CaW5hcnkpICNST0NSIHByZWRpY3Rpb24gb2JqZWN0CnJvYy5wZXJmLm10dCA9IFJPQ1I6OnBlcmZvcm1hbmNlKHJvY3IucHJlZC5tdHQsIG1lYXN1cmUgPSAidHByIiwgeC5tZWFzdXJlID0gImZwciIpICMgI1JPQ1IgcGVyZm9ybWFuY2Ugb2JqZWN0CnBsb3Qocm9jLnBlcmYubXR0LCBjb2wgPSAiYmx1ZSIpCmFibGluZShhID0gMCwgYiA9IDEsIGx0eSA9IDIpICNkaWFnb25hbCBmb3IgcmFuZG9tIGFzc2lnbm1lbnQKYGBgCgojIyMgUmVwb3J0IEFVQyBmcm9tIFJPQyBmb3IgdHJhaW5pbmcgYW5kIHRlc3QgZGF0YQpgYGB7ciBBVUM0fQogICMgVHJhaW4gQVVDCmF1YyA8LSBST0NSOjpwZXJmb3JtYW5jZShyb2NyLnByZWQubXR0LCBtZWFzdXJlID0gImF1YyIpCiAgYXVjIDwtIGF1Y0B5LnZhbHVlc1tbMV1dCiAgcHJpbnQoYXVjKQpgYGAKCgojIyMgQ2FsY3VsYXRlIE5hZ2Vsa2Vya2UgUl4yCmBgYHtyIEk4NGF9Ck5hZ2Vsa2Vya2VSMihtKQpgYGAKCiMjIyBjaGVjayBhc3N1bXB0aW9ucyBvZiBtb2RlbAojIyMjIENvb2sncyBkaXN0YW5jZQpgYGB7ciBJODV9CnBsb3QobSwgd2hpY2ggPSA0LCBpZC5uID0gMykKYGBgCgojIyMjIEV4dHJhY3QgbW9kZWwgcmVzdWx0cyBhbmQgZGlzcGxheSBkYXRhIGZvciB0b3AgMyB2YWx1ZXMgdXNpbmcgQ29vaydzIGRpc3RhbmNlCmBgYHtyIEk4Nn0KbW9kZWwuZGF0YSA8LSBhdWdtZW50KG0pICU+JSAKICBtdXRhdGUoaW5kZXggPSAxOm4oKSkgCm1vZGVsLmRhdGEgJT4lIHRvcF9uKDMsIC5jb29rc2QpCmBgYAoKIyMjIyBwbG90IHN0YW5kYXJkaXNlZCByZXNpZHVhbHMKYGBge3IgSTg3fQpnZ3Bsb3QobW9kZWwuZGF0YSwgYWVzKGluZGV4LCAuc3RkLnJlc2lkKSkgKyAKICBnZW9tX3BvaW50KGFlcyhjb2xvciA9IEhlYWx0aF9CaW5hcnkpLCBhbHBoYSA9IC41KSArCiAgdGhlbWVfYncoKQpgYGAKCiMjIyMgRmlsdGVyIHBvdGVudGlhbCBpbmZsdWVudGlhbCBkYXRhIHBvaW50cyB3aXRoIGFicyguc3RkLnJlcykgPiAzOgpgYGB7ciBJODh9Cm1vZGVsLmRhdGEgJT4lIAogIGZpbHRlcihhYnMoLnN0ZC5yZXNpZCkgPiAzKQpgYGAKCgoKIyMgTE9DQVRJT04gYmluYXJ5IGxvZ2lzdGljIHJlZ3Jlc3Npb24gT04gU0lHTklGSUNBTlQgT1IgU0VSSU9VUyBJTExORVNTCmBgYHtyIEk5fQojIGZpdCBiaW5hcnkgbG9naXQgbW9kZWwgYW5kIHN0b3JlIHJlc3VsdHMgJ20nCm0gPC0gZ2xtKEhlYWx0aF9CaW5hcnkgfiBMb2NhdGlvbiwgZGF0YSA9IG1sLGZhbWlseSA9IGJpbm9taWFsKQojIHZpZXcgYSBzdW1tYXJ5IG9mIHRoZSBtb2RlbApzdW1tYXJ5KG0pCiMgdGVzdCBtb2RlbCBmaXQKd2l0aChtLCBudWxsLmRldmlhbmNlIC0gZGV2aWFuY2UpCndpdGgobSwgZGYubnVsbCAtIGRmLnJlc2lkdWFsKQp3aXRoKG0sIHBjaGlzcShudWxsLmRldmlhbmNlIC0gZGV2aWFuY2UsIGRmLm51bGwgLSBkZi5yZXNpZHVhbCwgbG93ZXIudGFpbCA9IEZBTFNFKSkKQklDKG0pCiMgSG9zbWVyLUxlbWVzaG93IEdvb2RuZXNzLW9mLUZpdCBUZXN0CmdsbXRvb2xib3g6OmhsdGVzdChtKQojIyBDSXMgdXNpbmcgcHJvZmlsZWQgbG9nLWxpa2VsaWhvb2QKY29uZmludChtLCBsZXZlbD0wLjk5KQojIyBDSXMgdXNpbmcgc3RhbmRhcmQgZXJyb3JzCmNvbmZpbnQuZGVmYXVsdChtLCBsZXZlbD0wLjk5KQojIFdhbGQgdGVzdAp3YWxkLnRlc3QoYiA9IGNvZWYobSksIFNpZ21hID0gdmNvdihtKSwgVGVybXMgPSAyKQojIyBvZGRzIHJhdGlvcyBhbmQgOTUlIENJCmV4cChjYmluZChPUiA9IGNvZWYobSksIGNvbmZpbnQobSwgbGV2ZWw9MC45OSkpKQpgYGAKCiMjIyBDcmVhdGUgUk9DUiBmcm9tIGRhdGEKYGBge3IgUk9DNX0KIyMgdHJhaW5pbmcgZGF0YQpwcmVkLm10dCA9IHByZWRpY3QobSwgdHlwZSA9ICJyZXNwb25zZSIpICNyZXBlYXQgcmlzayBwcmVkaWN0aW9ucyBmcm9tIG1vZGVsIG0Kcm9jci5wcmVkLm10dCA9IFJPQ1I6OnByZWRpY3Rpb24ocHJlZC5tdHQsIGxhYmVscyA9IG1sJEhlYWx0aF9CaW5hcnkpICNST0NSIHByZWRpY3Rpb24gb2JqZWN0CnJvYy5wZXJmLm10dCA9IFJPQ1I6OnBlcmZvcm1hbmNlKHJvY3IucHJlZC5tdHQsIG1lYXN1cmUgPSAidHByIiwgeC5tZWFzdXJlID0gImZwciIpICMgI1JPQ1IgcGVyZm9ybWFuY2Ugb2JqZWN0CnBsb3Qocm9jLnBlcmYubXR0LCBjb2wgPSAiYmx1ZSIpCmFibGluZShhID0gMCwgYiA9IDEsIGx0eSA9IDIpICNkaWFnb25hbCBmb3IgcmFuZG9tIGFzc2lnbm1lbnQKYGBgCgojIyMgUmVwb3J0IEFVQyBmcm9tIFJPQyBmb3IgdHJhaW5pbmcgYW5kIHRlc3QgZGF0YQpgYGB7ciBBVUM1fQogICMgVHJhaW4gQVVDCmF1YyA8LSBST0NSOjpwZXJmb3JtYW5jZShyb2NyLnByZWQubXR0LCBtZWFzdXJlID0gImF1YyIpCiAgYXVjIDwtIGF1Y0B5LnZhbHVlc1tbMV1dCiAgcHJpbnQoYXVjKQpgYGAKCgojIyMgQ2FsY3VsYXRlIE5hZ2Vsa2Vya2UgUl4yCmBgYHtyIEk5YX0KTmFnZWxrZXJrZVIyKG0pCmBgYAoKIyMjIGNoZWNrIGFzc3VtcHRpb25zIG9mIG1vZGVsCiMjIyMgQ29vaydzIGRpc3RhbmNlCmBgYHtyIEkxMH0KcGxvdChtLCB3aGljaCA9IDQsIGlkLm4gPSAzKQpgYGAKCiMjIyMgRXh0cmFjdCBtb2RlbCByZXN1bHRzIGFuZCBkaXNwbGF5IGRhdGEgZm9yIHRvcCAzIHZhbHVlcyB1c2luZyBDb29rJ3MgZGlzdGFuY2UKYGBge3IgSTExfQptb2RlbC5kYXRhIDwtIGF1Z21lbnQobSkgJT4lIAogIG11dGF0ZShpbmRleCA9IDE6bigpKSAKbW9kZWwuZGF0YSAlPiUgdG9wX24oMywgLmNvb2tzZCkKYGBgCgojIyMjIHBsb3Qgc3RhbmRhcmRpc2VkIHJlc2lkdWFscwpgYGB7ciBJMTJ9CmdncGxvdChtb2RlbC5kYXRhLCBhZXMoaW5kZXgsIC5zdGQucmVzaWQpKSArIAogIGdlb21fcG9pbnQoYWVzKGNvbG9yID0gSGVhbHRoX0JpbmFyeSksIGFscGhhID0gLjUpICsKICB0aGVtZV9idygpCmBgYAoKIyMjIyBGaWx0ZXIgcG90ZW50aWFsIGluZmx1ZW50aWFsIGRhdGEgcG9pbnRzIHdpdGggYWJzKC5zdGQucmVzKSA+IDM6CmBgYHtyIEkxM30KbW9kZWwuZGF0YSAlPiUgCiAgZmlsdGVyKGFicyguc3RkLnJlc2lkKSA+IDMpCmBgYAoKCgojIyBTRVRUSU5HIGJpbmFyeSBsb2dpc3RpYyByZWdyZXNzaW9uIE9OIFNJR05JRklDQU5UIE9SIFNFUklPVVMgSUxMTkVTUwpgYGB7ciBJMTR9CiMgZml0IGJpbmFyeSBsb2dpdCBtb2RlbCBhbmQgc3RvcmUgcmVzdWx0cyAnbScKbSA8LSBnbG0oSGVhbHRoX0JpbmFyeSB+IHNldHRpbmcsIGRhdGEgPSBtbCxmYW1pbHkgPSBiaW5vbWlhbCkKIyB2aWV3IGEgc3VtbWFyeSBvZiB0aGUgbW9kZWwKc3VtbWFyeShtKQojIHRlc3QgbW9kZWwgZml0CndpdGgobSwgbnVsbC5kZXZpYW5jZSAtIGRldmlhbmNlKQp3aXRoKG0sIGRmLm51bGwgLSBkZi5yZXNpZHVhbCkKd2l0aChtLCBwY2hpc3EobnVsbC5kZXZpYW5jZSAtIGRldmlhbmNlLCBkZi5udWxsIC0gZGYucmVzaWR1YWwsIGxvd2VyLnRhaWwgPSBGQUxTRSkpCkJJQyhtKQojIEhvc21lci1MZW1lc2hvdyBHb29kbmVzcy1vZi1GaXQgVGVzdApnbG10b29sYm94OjpobHRlc3QobSkKIyMgQ0lzIHVzaW5nIHByb2ZpbGVkIGxvZy1saWtlbGlob29kCmNvbmZpbnQobSwgbGV2ZWw9MC45OSkKIyMgQ0lzIHVzaW5nIHN0YW5kYXJkIGVycm9ycwpjb25maW50LmRlZmF1bHQobSwgbGV2ZWw9MC45OSkKIyBXYWxkIHRlc3QKd2FsZC50ZXN0KGIgPSBjb2VmKG0pLCBTaWdtYSA9IHZjb3YobSksIFRlcm1zID0gMikKIyMgb2RkcyByYXRpb3MgYW5kIDk1JSBDSQpleHAoY2JpbmQoT1IgPSBjb2VmKG0pLCBjb25maW50KG0sIGxldmVsPTAuOTkpKSkKYGBgCgojIyMgQ3JlYXRlIFJPQ1IgZnJvbSBkYXRhCmBgYHtyIFJPQzZ9CiMjIHRyYWluaW5nIGRhdGEKcHJlZC5tdHQgPSBwcmVkaWN0KG0sIHR5cGUgPSAicmVzcG9uc2UiKSAjcmVwZWF0IHJpc2sgcHJlZGljdGlvbnMgZnJvbSBtb2RlbCBtCnJvY3IucHJlZC5tdHQgPSBST0NSOjpwcmVkaWN0aW9uKHByZWQubXR0LCBsYWJlbHMgPSBtbCRIZWFsdGhfQmluYXJ5KSAjUk9DUiBwcmVkaWN0aW9uIG9iamVjdApyb2MucGVyZi5tdHQgPSBST0NSOjpwZXJmb3JtYW5jZShyb2NyLnByZWQubXR0LCBtZWFzdXJlID0gInRwciIsIHgubWVhc3VyZSA9ICJmcHIiKSAjICNST0NSIHBlcmZvcm1hbmNlIG9iamVjdApwbG90KHJvYy5wZXJmLm10dCwgY29sID0gImJsdWUiKQphYmxpbmUoYSA9IDAsIGIgPSAxLCBsdHkgPSAyKSAjZGlhZ29uYWwgZm9yIHJhbmRvbSBhc3NpZ25tZW50CmBgYAoKIyMjIFJlcG9ydCBBVUMgZnJvbSBST0MgZm9yIHRyYWluaW5nIGFuZCB0ZXN0IGRhdGEKYGBge3IgQVVDNn0KICAjIFRyYWluIEFVQwphdWMgPC0gUk9DUjo6cGVyZm9ybWFuY2Uocm9jci5wcmVkLm10dCwgbWVhc3VyZSA9ICJhdWMiKQogIGF1YyA8LSBhdWNAeS52YWx1ZXNbWzFdXQogIHByaW50KGF1YykKYGBgCgoKIyMjIENhbGN1bGF0ZSBOYWdlbGtlcmtlIFJeMgpgYGB7ciBJMTRhfQpOYWdlbGtlcmtlUjIobSkKYGBgCgojIyMgY2hlY2sgYXNzdW1wdGlvbnMgb2YgbW9kZWwKIyMjIyBDb29rJ3MgZGlzdGFuY2UKYGBge3IgSTE1fQpwbG90KG0sIHdoaWNoID0gNCwgaWQubiA9IDMpCmBgYAoKIyMjIyBFeHRyYWN0IG1vZGVsIHJlc3VsdHMgYW5kIGRpc3BsYXkgZGF0YSBmb3IgdG9wIDMgdmFsdWVzIHVzaW5nIENvb2sncyBkaXN0YW5jZQpgYGB7ciBJMTZ9Cm1vZGVsLmRhdGEgPC0gYXVnbWVudChtKSAlPiUgCiAgbXV0YXRlKGluZGV4ID0gMTpuKCkpIAptb2RlbC5kYXRhICU+JSB0b3BfbigzLCAuY29va3NkKQpgYGAKCiMjIyMgcGxvdCBzdGFuZGFyZGlzZWQgcmVzaWR1YWxzCmBgYHtyIEkxN30KZ2dwbG90KG1vZGVsLmRhdGEsIGFlcyhpbmRleCwgLnN0ZC5yZXNpZCkpICsgCiAgZ2VvbV9wb2ludChhZXMoY29sb3IgPSBIZWFsdGhfQmluYXJ5KSwgYWxwaGEgPSAuNSkgKwogIHRoZW1lX2J3KCkKYGBgCgojIyMjIEZpbHRlciBwb3RlbnRpYWwgaW5mbHVlbnRpYWwgZGF0YSBwb2ludHMgd2l0aCBhYnMoLnN0ZC5yZXMpID4gMzoKYGBge3IgSTE4fQptb2RlbC5kYXRhICU+JSAKICBmaWx0ZXIoYWJzKC5zdGQucmVzaWQpID4gMykKYGBgCgoKCiMjIFVSQkFOIGJpbmFyeSBsb2dpc3RpYyByZWdyZXNzaW9uIE9OIFNJR05JRklDQU5UIE9SIFNFUklPVVMgSUxMTkVTUwpgYGB7ciBJMTl9CiMgZml0IGJpbmFyeSBsb2dpdCBtb2RlbCBhbmQgc3RvcmUgcmVzdWx0cyAnbScKbSA8LSBnbG0oSGVhbHRoX0JpbmFyeSB+IFVyYmFuLCBkYXRhID0gbWwsZmFtaWx5ID0gYmlub21pYWwpCiMgdmlldyBhIHN1bW1hcnkgb2YgdGhlIG1vZGVsCnN1bW1hcnkobSkKIyB0ZXN0IG1vZGVsIGZpdAp3aXRoKG0sIG51bGwuZGV2aWFuY2UgLSBkZXZpYW5jZSkKd2l0aChtLCBkZi5udWxsIC0gZGYucmVzaWR1YWwpCndpdGgobSwgcGNoaXNxKG51bGwuZGV2aWFuY2UgLSBkZXZpYW5jZSwgZGYubnVsbCAtIGRmLnJlc2lkdWFsLCBsb3dlci50YWlsID0gRkFMU0UpKQpCSUMobSkKIyMgQ0lzIHVzaW5nIHByb2ZpbGVkIGxvZy1saWtlbGlob29kCmNvbmZpbnQobSwgbGV2ZWw9MC45OSkKIyMgQ0lzIHVzaW5nIHN0YW5kYXJkIGVycm9ycwpjb25maW50LmRlZmF1bHQobSwgbGV2ZWw9MC45OSkKIyBXYWxkIHRlc3QKd2FsZC50ZXN0KGIgPSBjb2VmKG0pLCBTaWdtYSA9IHZjb3YobSksIFRlcm1zID0gMikKIyMgb2RkcyByYXRpb3MgYW5kIDk1JSBDSQpleHAoY2JpbmQoT1IgPSBjb2VmKG0pLCBjb25maW50KG0sIGxldmVsPTAuOTkpKSkKYGBgCgojIyMgQ3JlYXRlIFJPQ1IgZnJvbSBkYXRhCmBgYHtyIFJPQzd9CiMjIHRyYWluaW5nIGRhdGEKcHJlZC5tdHQgPSBwcmVkaWN0KG0sIHR5cGUgPSAicmVzcG9uc2UiKSAjcmVwZWF0IHJpc2sgcHJlZGljdGlvbnMgZnJvbSBtb2RlbCBtCnJvY3IucHJlZC5tdHQgPSBST0NSOjpwcmVkaWN0aW9uKHByZWQubXR0LCBsYWJlbHMgPSBtbCRIZWFsdGhfQmluYXJ5KSAjUk9DUiBwcmVkaWN0aW9uIG9iamVjdApyb2MucGVyZi5tdHQgPSBST0NSOjpwZXJmb3JtYW5jZShyb2NyLnByZWQubXR0LCBtZWFzdXJlID0gInRwciIsIHgubWVhc3VyZSA9ICJmcHIiKSAjICNST0NSIHBlcmZvcm1hbmNlIG9iamVjdApwbG90KHJvYy5wZXJmLm10dCwgY29sID0gImJsdWUiKQphYmxpbmUoYSA9IDAsIGIgPSAxLCBsdHkgPSAyKSAjZGlhZ29uYWwgZm9yIHJhbmRvbSBhc3NpZ25tZW50CmBgYAoKIyMjIFJlcG9ydCBBVUMgZnJvbSBST0MgZm9yIHRyYWluaW5nIGFuZCB0ZXN0IGRhdGEKYGBge3IgQVVDN30KICAjIFRyYWluIEFVQwphdWMgPC0gUk9DUjo6cGVyZm9ybWFuY2Uocm9jci5wcmVkLm10dCwgbWVhc3VyZSA9ICJhdWMiKQogIGF1YyA8LSBhdWNAeS52YWx1ZXNbWzFdXQogIHByaW50KGF1YykKYGBgCgoKIyMjIENhbGN1bGF0ZSBOYWdlbGtlcmtlIFJeMgpgYGB7ciBJMTlhfQpOYWdlbGtlcmtlUjIobSkKYGBgCgojIyMgY2hlY2sgYXNzdW1wdGlvbnMgb2YgbW9kZWwKIyMjIyBDb29rJ3MgZGlzdGFuY2UKYGBge3IgSTIwfQpwbG90KG0sIHdoaWNoID0gNCwgaWQubiA9IDMpCmBgYAoKIyBFeHRyYWN0IG1vZGVsIHJlc3VsdHMgYW5kIGRpc3BsYXkgZGF0YSBmb3IgdG9wIDMgdmFsdWVzIHVzaW5nIENvb2sncyBkaXN0YW5jZQpgYGB7ciBJMjF9Cm1vZGVsLmRhdGEgPC0gYXVnbWVudChtKSAlPiUgCiAgbXV0YXRlKGluZGV4ID0gMTpuKCkpIAptb2RlbC5kYXRhICU+JSB0b3BfbigzLCAuY29va3NkKQpgYGAKCiMjIyMgcGxvdCBzdGFuZGFyZGlzZWQgcmVzaWR1YWxzCmBgYHtyIEkyMn0KZ2dwbG90KG1vZGVsLmRhdGEsIGFlcyhpbmRleCwgLnN0ZC5yZXNpZCkpICsgCiAgZ2VvbV9wb2ludChhZXMoY29sb3IgPSBIZWFsdGhfQmluYXJ5KSwgYWxwaGEgPSAuNSkgKwogIHRoZW1lX2J3KCkKYGBgCgojIyMjIEZpbHRlciBwb3RlbnRpYWwgaW5mbHVlbnRpYWwgZGF0YSBwb2ludHMgd2l0aCBhYnMoLnN0ZC5yZXMpID4gMzoKYGBge3IgSTIzfQptb2RlbC5kYXRhICU+JSAKICBmaWx0ZXIoYWJzKC5zdGQucmVzaWQpID4gMykKYGBgCgoKCiMjIExPQ0FUSU9OICsgVVJCQU4gYmluYXJ5IGxvZ2lzdGljIHJlZ3Jlc3Npb24gT04gU0lHTklGSUNBTlQgT1IgU0VSSU9VUyBJTExORVNTCmBgYHtyIEkyNH0KIyBmaXQgYmluYXJ5IGxvZ2l0IG1vZGVsIGFuZCBzdG9yZSByZXN1bHRzICdtJwptIDwtIGdsbShIZWFsdGhfQmluYXJ5IH4gTG9jYXRpb24gKyBVcmJhbiwgZGF0YSA9IG1sLGZhbWlseSA9IGJpbm9taWFsKQojIHZpZXcgYSBzdW1tYXJ5IG9mIHRoZSBtb2RlbApzdW1tYXJ5KG0pCiMgdGVzdCBtb2RlbCBmaXQKd2l0aChtLCBudWxsLmRldmlhbmNlIC0gZGV2aWFuY2UpCndpdGgobSwgZGYubnVsbCAtIGRmLnJlc2lkdWFsKQp3aXRoKG0sIHBjaGlzcShudWxsLmRldmlhbmNlIC0gZGV2aWFuY2UsIGRmLm51bGwgLSBkZi5yZXNpZHVhbCwgbG93ZXIudGFpbCA9IEZBTFNFKSkKQklDKG0pCiMgSG9zbWVyLUxlbWVzaG93IEdvb2RuZXNzLW9mLUZpdCBUZXN0CmhsdGVzdChtKQojIyBDSXMgdXNpbmcgcHJvZmlsZWQgbG9nLWxpa2VsaWhvb2QKY29uZmludChtLCBsZXZlbD0wLjk5KQojIyBDSXMgdXNpbmcgc3RhbmRhcmQgZXJyb3JzCmNvbmZpbnQuZGVmYXVsdChtLCBsZXZlbD0wLjk5KQojIFdhbGQgdGVzdAp3YWxkLnRlc3QoYiA9IGNvZWYobSksIFNpZ21hID0gdmNvdihtKSwgVGVybXMgPSAyKQojIyBvZGRzIHJhdGlvcyBhbmQgOTUlIENJCmV4cChjYmluZChPUiA9IGNvZWYobSksIGNvbmZpbnQobSwgbGV2ZWw9MC45OSkpKQpgYGAKCiMjIyBDcmVhdGUgUk9DUiBmcm9tIGRhdGEKYGBge3IgUk9DOH0KIyMgdHJhaW5pbmcgZGF0YQpwcmVkLm10dCA9IHByZWRpY3QobSwgdHlwZSA9ICJyZXNwb25zZSIpICNyZXBlYXQgcmlzayBwcmVkaWN0aW9ucyBmcm9tIG1vZGVsIG0Kcm9jci5wcmVkLm10dCA9IFJPQ1I6OnByZWRpY3Rpb24ocHJlZC5tdHQsIGxhYmVscyA9IG1sJEhlYWx0aF9CaW5hcnkpICNST0NSIHByZWRpY3Rpb24gb2JqZWN0CnJvYy5wZXJmLm10dCA9IFJPQ1I6OnBlcmZvcm1hbmNlKHJvY3IucHJlZC5tdHQsIG1lYXN1cmUgPSAidHByIiwgeC5tZWFzdXJlID0gImZwciIpICMgI1JPQ1IgcGVyZm9ybWFuY2Ugb2JqZWN0CnBsb3Qocm9jLnBlcmYubXR0LCBjb2wgPSAiYmx1ZSIpCmFibGluZShhID0gMCwgYiA9IDEsIGx0eSA9IDIpICNkaWFnb25hbCBmb3IgcmFuZG9tIGFzc2lnbm1lbnQKYGBgCgojIyMgUmVwb3J0IEFVQyBmcm9tIFJPQyBmb3IgdHJhaW5pbmcgYW5kIHRlc3QgZGF0YQpgYGB7ciBBVUM4fQogICMgVHJhaW4gQVVDCmF1YyA8LSBST0NSOjpwZXJmb3JtYW5jZShyb2NyLnByZWQubXR0LCBtZWFzdXJlID0gImF1YyIpCiAgYXVjIDwtIGF1Y0B5LnZhbHVlc1tbMV1dCiAgcHJpbnQoYXVjKQpgYGAKCgojIyMgQ2FsY3VsYXRlIE5hZ2Vsa2Vya2UgUl4yCmBgYHtyIEkyNGF9Ck5hZ2Vsa2Vya2VSMihtKQpgYGAKCiMjIyBjaGVjayBhc3N1bXB0aW9ucyBvZiBtb2RlbAojIyMjIENvb2sncyBkaXN0YW5jZQpgYGB7ciBJMjV9CnBsb3QobSwgd2hpY2ggPSA0LCBpZC5uID0gMykKYGBgCgojIyMjIEV4dHJhY3QgbW9kZWwgcmVzdWx0cyBhbmQgZGlzcGxheSBkYXRhIGZvciB0b3AgMyB2YWx1ZXMgdXNpbmcgQ29vaydzIGRpc3RhbmNlCmBgYHtyIEkyNn0KbW9kZWwuZGF0YSA8LSBhdWdtZW50KG0pICU+JSAKICBtdXRhdGUoaW5kZXggPSAxOm4oKSkgCm1vZGVsLmRhdGEgJT4lIHRvcF9uKDMsIC5jb29rc2QpCmBgYAoKIyMjIyBwbG90IHN0YW5kYXJkaXNlZCByZXNpZHVhbHMKYGBge3IgSTI3fQpnZ3Bsb3QobW9kZWwuZGF0YSwgYWVzKGluZGV4LCAuc3RkLnJlc2lkKSkgKyAKICBnZW9tX3BvaW50KGFlcyhjb2xvciA9IEhlYWx0aF9CaW5hcnkpLCBhbHBoYSA9IC41KSArCiAgdGhlbWVfYncoKQpgYGAKCiMjIyMgRmlsdGVyIHBvdGVudGlhbCBpbmZsdWVudGlhbCBkYXRhIHBvaW50cyB3aXRoIGFicyguc3RkLnJlcykgPiAzOgpgYGB7ciBJMjh9Cm1vZGVsLmRhdGEgJT4lIAogIGZpbHRlcihhYnMoLnN0ZC5yZXNpZCkgPiAzKQpgYGAKCiMjIyMgY2hlY2sgZm9yIG11bHRpY29sbGluZWFyaXR5CmBgYHtyIEkyOX0KY2FyOjp2aWYobSkKYGBgCgoKCiMjIExPQ0FUSU9OICogVVJCQU4gYmluYXJ5IGxvZ2lzdGljIHJlZ3Jlc3Npb24gT04gU0lHTklGSUNBTlQgT1IgU0VSSU9VUyBJTExORVNTCmBgYHtyIEkzMH0KIyBmaXQgYmluYXJ5IGxvZ2l0IG1vZGVsIGFuZCBzdG9yZSByZXN1bHRzICdtJwptIDwtIGdsbShIZWFsdGhfQmluYXJ5IH4gTG9jYXRpb24qVXJiYW4sIGRhdGEgPSBtbCxmYW1pbHkgPSBiaW5vbWlhbCkKIyB2aWV3IGEgc3VtbWFyeSBvZiB0aGUgbW9kZWwKc3VtbWFyeShtKQojIHRlc3QgbW9kZWwgZml0CndpdGgobSwgbnVsbC5kZXZpYW5jZSAtIGRldmlhbmNlKQp3aXRoKG0sIGRmLm51bGwgLSBkZi5yZXNpZHVhbCkKd2l0aChtLCBwY2hpc3EobnVsbC5kZXZpYW5jZSAtIGRldmlhbmNlLCBkZi5udWxsIC0gZGYucmVzaWR1YWwsIGxvd2VyLnRhaWwgPSBGQUxTRSkpCkJJQyhtKQojIEhvc21lci1MZW1lc2hvdyBHb29kbmVzcy1vZi1GaXQgVGVzdApobHRlc3QobSkKIyMgQ0lzIHVzaW5nIHByb2ZpbGVkIGxvZy1saWtlbGlob29kCmNvbmZpbnQobSwgbGV2ZWw9MC45OSkKIyMgQ0lzIHVzaW5nIHN0YW5kYXJkIGVycm9ycwpjb25maW50LmRlZmF1bHQobSwgbGV2ZWw9MC45OSkKIyBXYWxkIHRlc3QKd2FsZC50ZXN0KGIgPSBjb2VmKG0pLCBTaWdtYSA9IHZjb3YobSksIFRlcm1zID0gMikKIyMgb2RkcyByYXRpb3MgYW5kIDk1JSBDSQpleHAoY2JpbmQoT1IgPSBjb2VmKG0pLCBjb25maW50KG0sIGxldmVsPTAuOTkpKSkKYGBgCgojIyMgQ3JlYXRlIFJPQ1IgZnJvbSBkYXRhCmBgYHtyIFJPQzl9CiMjIHRyYWluaW5nIGRhdGEKcHJlZC5tdHQgPSBwcmVkaWN0KG0sIHR5cGUgPSAicmVzcG9uc2UiKSAjcmVwZWF0IHJpc2sgcHJlZGljdGlvbnMgZnJvbSBtb2RlbCBtCnJvY3IucHJlZC5tdHQgPSBST0NSOjpwcmVkaWN0aW9uKHByZWQubXR0LCBsYWJlbHMgPSBtbCRIZWFsdGhfQmluYXJ5KSAjUk9DUiBwcmVkaWN0aW9uIG9iamVjdApyb2MucGVyZi5tdHQgPSBST0NSOjpwZXJmb3JtYW5jZShyb2NyLnByZWQubXR0LCBtZWFzdXJlID0gInRwciIsIHgubWVhc3VyZSA9ICJmcHIiKSAjICNST0NSIHBlcmZvcm1hbmNlIG9iamVjdApwbG90KHJvYy5wZXJmLm10dCwgY29sID0gImJsdWUiKQphYmxpbmUoYSA9IDAsIGIgPSAxLCBsdHkgPSAyKSAjZGlhZ29uYWwgZm9yIHJhbmRvbSBhc3NpZ25tZW50CmBgYAoKIyMjIFJlcG9ydCBBVUMgZnJvbSBST0MgZm9yIHRyYWluaW5nIGFuZCB0ZXN0IGRhdGEKYGBge3IgQVVDOX0KICAjIFRyYWluIEFVQwphdWMgPC0gUk9DUjo6cGVyZm9ybWFuY2Uocm9jci5wcmVkLm10dCwgbWVhc3VyZSA9ICJhdWMiKQogIGF1YyA8LSBhdWNAeS52YWx1ZXNbWzFdXQogIHByaW50KGF1YykKYGBgCgoKIyMjIENhbGN1bGF0ZSBOYWdlbGtlcmtlIFJeMgpgYGB7ciBJMzBhfQpOYWdlbGtlcmtlUjIobSkKYGBgCgojIyMgY2hlY2sgYXNzdW1wdGlvbnMgb2YgbW9kZWwKIyMjIyBDb29rJ3MgZGlzdGFuY2UKYGBge3IgSTMxfQpwbG90KG0sIHdoaWNoID0gNCwgaWQubiA9IDMpCmBgYAoKIyMjIyBFeHRyYWN0IG1vZGVsIHJlc3VsdHMgYW5kIGRpc3BsYXkgZGF0YSBmb3IgdG9wIDMgdmFsdWVzIHVzaW5nIENvb2sncyBkaXN0YW5jZQpgYGB7ciBJMzJ9Cm1vZGVsLmRhdGEgPC0gYXVnbWVudChtKSAlPiUgCiAgbXV0YXRlKGluZGV4ID0gMTpuKCkpIAptb2RlbC5kYXRhICU+JSB0b3BfbigzLCAuY29va3NkKQpgYGAKCiMjIyMgcGxvdCBzdGFuZGFyZGlzZWQgcmVzaWR1YWxzCmBgYHtyIEkzM30KZ2dwbG90KG1vZGVsLmRhdGEsIGFlcyhpbmRleCwgLnN0ZC5yZXNpZCkpICsgCiAgZ2VvbV9wb2ludChhZXMoY29sb3IgPSBIZWFsdGhfQmluYXJ5KSwgYWxwaGEgPSAuNSkgKwogIHRoZW1lX2J3KCkKYGBgCgojIyMjIEZpbHRlciBwb3RlbnRpYWwgaW5mbHVlbnRpYWwgZGF0YSBwb2ludHMgd2l0aCBhYnMoLnN0ZC5yZXMpID4gMzoKYGBge3IgSTM0fQptb2RlbC5kYXRhICU+JSAKICBmaWx0ZXIoYWJzKC5zdGQucmVzaWQpID4gMykKYGBgCgojIyMjIGNoZWNrIGZvciBtdWx0aWNvbGxpbmVhcml0eQpgYGB7ciBJMzV9CmNhcjo6dmlmKG0pCmBgYAoKCgojIyBFRFVDQVRJT04gQmluYXJ5IGxvZ2lzdGljIHJlZ3Jlc3Npb24gT04gU0lHTklGSUNBTlQgT1IgU0VSSU9VUyBJTExORVNTCmBgYHtyIEkzN30KIyBmaXQgYmluYXJ5IGxvZ2l0IG1vZGVsIGFuZCBzdG9yZSByZXN1bHRzICdtJwptIDwtIGdsbShIZWFsdGhfQmluYXJ5IH4gRWR1Y2F0aW9uLCBkYXRhID0gbWwsZmFtaWx5ID0gYmlub21pYWwpCiMgdmlldyBhIHN1bW1hcnkgb2YgdGhlIG1vZGVsCnN1bW1hcnkobSkKIyB0ZXN0IG1vZGVsIGZpdAp3aXRoKG0sIG51bGwuZGV2aWFuY2UgLSBkZXZpYW5jZSkKd2l0aChtLCBkZi5udWxsIC0gZGYucmVzaWR1YWwpCndpdGgobSwgcGNoaXNxKG51bGwuZGV2aWFuY2UgLSBkZXZpYW5jZSwgZGYubnVsbCAtIGRmLnJlc2lkdWFsLCBsb3dlci50YWlsID0gRkFMU0UpKQpCSUMobSkKIyBIb3NtZXItTGVtZXNob3cgR29vZG5lc3Mtb2YtRml0IFRlc3QKaGx0ZXN0KG0pCiMjIENJcyB1c2luZyBwcm9maWxlZCBsb2ctbGlrZWxpaG9vZApjb25maW50KG0sIGxldmVsPTAuOTkpCiMjIENJcyB1c2luZyBzdGFuZGFyZCBlcnJvcnMKY29uZmludC5kZWZhdWx0KG0sIGxldmVsPTAuOTkpCiMgV2FsZCB0ZXN0CndhbGQudGVzdChiID0gY29lZihtKSwgU2lnbWEgPSB2Y292KG0pLCBUZXJtcyA9IDIpCiMjIG9kZHMgcmF0aW9zIGFuZCA5NSUgQ0kKZXhwKGNiaW5kKE9SID0gY29lZihtKSwgY29uZmludChtLCBsZXZlbD0wLjk5KSkpCmBgYAoKIyMjIENyZWF0ZSBST0NSIGZyb20gZGF0YQpgYGB7ciBST0MxMH0KIyMgdHJhaW5pbmcgZGF0YQpwcmVkLm10dCA9IHByZWRpY3QobSwgdHlwZSA9ICJyZXNwb25zZSIpICNyZXBlYXQgcmlzayBwcmVkaWN0aW9ucyBmcm9tIG1vZGVsIG0Kcm9jci5wcmVkLm10dCA9IFJPQ1I6OnByZWRpY3Rpb24ocHJlZC5tdHQsIGxhYmVscyA9IG1sJEhlYWx0aF9CaW5hcnkpICNST0NSIHByZWRpY3Rpb24gb2JqZWN0CnJvYy5wZXJmLm10dCA9IFJPQ1I6OnBlcmZvcm1hbmNlKHJvY3IucHJlZC5tdHQsIG1lYXN1cmUgPSAidHByIiwgeC5tZWFzdXJlID0gImZwciIpICMgI1JPQ1IgcGVyZm9ybWFuY2Ugb2JqZWN0CnBsb3Qocm9jLnBlcmYubXR0LCBjb2wgPSAiYmx1ZSIpCmFibGluZShhID0gMCwgYiA9IDEsIGx0eSA9IDIpICNkaWFnb25hbCBmb3IgcmFuZG9tIGFzc2lnbm1lbnQKYGBgCgojIyMgUmVwb3J0IEFVQyBmcm9tIFJPQyBmb3IgdHJhaW5pbmcgYW5kIHRlc3QgZGF0YQpgYGB7ciBBVUMxMH0KICAjIFRyYWluIEFVQwphdWMgPC0gUk9DUjo6cGVyZm9ybWFuY2Uocm9jci5wcmVkLm10dCwgbWVhc3VyZSA9ICJhdWMiKQogIGF1YyA8LSBhdWNAeS52YWx1ZXNbWzFdXQogIHByaW50KGF1YykKYGBgCgoKIyMjIENhbGN1bGF0ZSBOYWdlbGtlcmtlIFJeMgpgYGB7ciBJMzdhfQpOYWdlbGtlcmtlUjIobSkKYGBgCgojIyMgY2hlY2sgYXNzdW1wdGlvbnMgb2YgbW9kZWwKIyMjIyBDb29rJ3MgZGlzdGFuY2UKYGBge3IgSTM4fQpwbG90KG0sIHdoaWNoID0gNCwgaWQubiA9IDMpCmBgYAoKIyMjIyBFeHRyYWN0IG1vZGVsIHJlc3VsdHMgYW5kIGRpc3BsYXkgZGF0YSBmb3IgdG9wIDMgdmFsdWVzIHVzaW5nIENvb2sncyBkaXN0YW5jZQpgYGB7ciBJMzl9Cm1vZGVsLmRhdGEgPC0gYXVnbWVudChtKSAlPiUgCiAgbXV0YXRlKGluZGV4ID0gMTpuKCkpIAptb2RlbC5kYXRhICU+JSB0b3BfbigzLCAuY29va3NkKQpgYGAKCiMjIyMgcGxvdCBzdGFuZGFyZGlzZWQgcmVzaWR1YWxzCmBgYHtyIEk0MH0KZ2dwbG90KG1vZGVsLmRhdGEsIGFlcyhpbmRleCwgLnN0ZC5yZXNpZCkpICsgCiAgZ2VvbV9wb2ludChhZXMoY29sb3IgPSBIZWFsdGhfQmluYXJ5KSwgYWxwaGEgPSAuNSkgKwogIHRoZW1lX2J3KCkKYGBgCgojIyMjIEZpbHRlciBwb3RlbnRpYWwgaW5mbHVlbnRpYWwgZGF0YSBwb2ludHMgd2l0aCBhYnMoLnN0ZC5yZXMpID4gMzoKYGBge3IgSTQxfQptb2RlbC5kYXRhICU+JSAKICBmaWx0ZXIoYWJzKC5zdGQucmVzaWQpID4gMykKYGBgCgoKCiMjIEVEVUNBVElPTl9TIEJpbmFyeSBsb2dpc3RpYyByZWdyZXNzaW9uIE9OIFNJR05JRklDQU5UIE9SIFNFUklPVVMgSUxMTkVTUwpgYGB7ciBJNDJ9CiMgZml0IGJpbmFyeSBsb2dpdCBtb2RlbCBhbmQgc3RvcmUgcmVzdWx0cyAnbScKbSA8LSBnbG0oSGVhbHRoX0JpbmFyeSB+IEVkdWNhdGlvbl9TLCBkYXRhID0gbWwsZmFtaWx5ID0gYmlub21pYWwpCiMgdmlldyBhIHN1bW1hcnkgb2YgdGhlIG1vZGVsCnN1bW1hcnkobSkKIyB0ZXN0IG1vZGVsIGZpdAp3aXRoKG0sIG51bGwuZGV2aWFuY2UgLSBkZXZpYW5jZSkKd2l0aChtLCBkZi5udWxsIC0gZGYucmVzaWR1YWwpCndpdGgobSwgcGNoaXNxKG51bGwuZGV2aWFuY2UgLSBkZXZpYW5jZSwgZGYubnVsbCAtIGRmLnJlc2lkdWFsLCBsb3dlci50YWlsID0gRkFMU0UpKQpCSUMobSkKIyBIb3NtZXItTGVtZXNob3cgR29vZG5lc3Mtb2YtRml0IFRlc3QKaGx0ZXN0KG0sIEc9MykKIyMgQ0lzIHVzaW5nIHByb2ZpbGVkIGxvZy1saWtlbGlob29kCmNvbmZpbnQobSwgbGV2ZWw9MC45OSkKIyMgQ0lzIHVzaW5nIHN0YW5kYXJkIGVycm9ycwpjb25maW50LmRlZmF1bHQobSwgbGV2ZWw9MC45OSkKIyBXYWxkIHRlc3QKd2FsZC50ZXN0KGIgPSBjb2VmKG0pLCBTaWdtYSA9IHZjb3YobSksIFRlcm1zID0gMikKIyMgb2RkcyByYXRpb3MgYW5kIDk1JSBDSQpleHAoY2JpbmQoT1IgPSBjb2VmKG0pLCBjb25maW50KG0sIGxldmVsPTAuOTkpKSkKYGBgCgojIyMgQ3JlYXRlIFJPQ1IgZnJvbSBkYXRhCmBgYHtyIFJPQzExfQojIyB0cmFpbmluZyBkYXRhCnByZWQubXR0ID0gcHJlZGljdChtLCB0eXBlID0gInJlc3BvbnNlIikgI3JlcGVhdCByaXNrIHByZWRpY3Rpb25zIGZyb20gbW9kZWwgbQpyb2NyLnByZWQubXR0ID0gUk9DUjo6cHJlZGljdGlvbihwcmVkLm10dCwgbGFiZWxzID0gbWwkSGVhbHRoX0JpbmFyeSkgI1JPQ1IgcHJlZGljdGlvbiBvYmplY3QKcm9jLnBlcmYubXR0ID0gUk9DUjo6cGVyZm9ybWFuY2Uocm9jci5wcmVkLm10dCwgbWVhc3VyZSA9ICJ0cHIiLCB4Lm1lYXN1cmUgPSAiZnByIikgIyAjUk9DUiBwZXJmb3JtYW5jZSBvYmplY3QKcGxvdChyb2MucGVyZi5tdHQsIGNvbCA9ICJibHVlIikKYWJsaW5lKGEgPSAwLCBiID0gMSwgbHR5ID0gMikgI2RpYWdvbmFsIGZvciByYW5kb20gYXNzaWdubWVudApgYGAKCiMjIyBSZXBvcnQgQVVDIGZyb20gUk9DIGZvciB0cmFpbmluZyBhbmQgdGVzdCBkYXRhCmBgYHtyIEFVQzExfQogICMgVHJhaW4gQVVDCmF1YyA8LSBST0NSOjpwZXJmb3JtYW5jZShyb2NyLnByZWQubXR0LCBtZWFzdXJlID0gImF1YyIpCiAgYXVjIDwtIGF1Y0B5LnZhbHVlc1tbMV1dCiAgcHJpbnQoYXVjKQpgYGAKCgojIyMgQ2FsY3VsYXRlIE5hZ2Vsa2Vya2UgUl4yCmBgYHtyIEk0MmF9Ck5hZ2Vsa2Vya2VSMihtKQpgYGAKCiMjIyBjaGVjayBhc3N1bXB0aW9ucyBvZiBtb2RlbAojIyMjIENvb2sncyBkaXN0YW5jZQpgYGB7ciBJNDN9CnBsb3QobSwgd2hpY2ggPSA0LCBpZC5uID0gMykKYGBgCgojIyMjIEV4dHJhY3QgbW9kZWwgcmVzdWx0cyBhbmQgZGlzcGxheSBkYXRhIGZvciB0b3AgMyB2YWx1ZXMgdXNpbmcgQ29vaydzIGRpc3RhbmNlCmBgYHtyIEk0NH0KbW9kZWwuZGF0YSA8LSBhdWdtZW50KG0pICU+JSAKICBtdXRhdGUoaW5kZXggPSAxOm4oKSkgCm1vZGVsLmRhdGEgJT4lIHRvcF9uKDMsIC5jb29rc2QpCmBgYAoKIyMjIyBwbG90IHN0YW5kYXJkaXNlZCByZXNpZHVhbHMKYGBge3IgSTQ1fQpnZ3Bsb3QobW9kZWwuZGF0YSwgYWVzKGluZGV4LCAuc3RkLnJlc2lkKSkgKyAKICBnZW9tX3BvaW50KGFlcyhjb2xvciA9IEhlYWx0aF9CaW5hcnkpLCBhbHBoYSA9IC41KSArCiAgdGhlbWVfYncoKQpgYGAKCiMjIyMgRmlsdGVyIHBvdGVudGlhbCBpbmZsdWVudGlhbCBkYXRhIHBvaW50cyB3aXRoIGFicyguc3RkLnJlcykgPiAzOgpgYGB7ciBJNDZ9Cm1vZGVsLmRhdGEgJT4lIAogIGZpbHRlcihhYnMoLnN0ZC5yZXNpZCkgPiAzKQpgYGAKCgoKIyMgRURVQ0FUSU9OX1MyIEJpbmFyeSBsb2dpc3RpYyByZWdyZXNzaW9uIE9OIFNJR05JRklDQU5UIE9SIFNFUklPVVMgSUxMTkVTUwpgYGB7ciBJNDJpfQojIGZpdCBiaW5hcnkgbG9naXQgbW9kZWwgYW5kIHN0b3JlIHJlc3VsdHMgJ20nCm0gPC0gZ2xtKEhlYWx0aF9CaW5hcnkgfiBFZHVjYXRpb25fUzIsIGRhdGEgPSBtbCxmYW1pbHkgPSBiaW5vbWlhbCkKIyB2aWV3IGEgc3VtbWFyeSBvZiB0aGUgbW9kZWwKc3VtbWFyeShtKQojIHRlc3QgbW9kZWwgZml0CndpdGgobSwgbnVsbC5kZXZpYW5jZSAtIGRldmlhbmNlKQp3aXRoKG0sIGRmLm51bGwgLSBkZi5yZXNpZHVhbCkKd2l0aChtLCBwY2hpc3EobnVsbC5kZXZpYW5jZSAtIGRldmlhbmNlLCBkZi5udWxsIC0gZGYucmVzaWR1YWwsIGxvd2VyLnRhaWwgPSBGQUxTRSkpCkJJQyhtKQojIEhvc21lci1MZW1lc2hvdyBHb29kbmVzcy1vZi1GaXQgVGVzdApobHRlc3QobSwgRz0zKQojIyBDSXMgdXNpbmcgcHJvZmlsZWQgbG9nLWxpa2VsaWhvb2QKY29uZmludChtLCBsZXZlbD0wLjk5KQojIyBDSXMgdXNpbmcgc3RhbmRhcmQgZXJyb3JzCmNvbmZpbnQuZGVmYXVsdChtLCBsZXZlbD0wLjk5KQojIFdhbGQgdGVzdAp3YWxkLnRlc3QoYiA9IGNvZWYobSksIFNpZ21hID0gdmNvdihtKSwgVGVybXMgPSAyKQojIyBvZGRzIHJhdGlvcyBhbmQgOTUlIENJCmV4cChjYmluZChPUiA9IGNvZWYobSksIGNvbmZpbnQobSwgbGV2ZWw9MC45OSkpKQpgYGAKIyMjIENyZWF0ZSBST0NSIGZyb20gZGF0YQpgYGB7ciBST0MxMn0KIyMgdHJhaW5pbmcgZGF0YQpwcmVkLm10dCA9IHByZWRpY3QobSwgdHlwZSA9ICJyZXNwb25zZSIpICNyZXBlYXQgcmlzayBwcmVkaWN0aW9ucyBmcm9tIG1vZGVsIG0Kcm9jci5wcmVkLm10dCA9IFJPQ1I6OnByZWRpY3Rpb24ocHJlZC5tdHQsIGxhYmVscyA9IG1sJEhlYWx0aF9CaW5hcnkpICNST0NSIHByZWRpY3Rpb24gb2JqZWN0CnJvYy5wZXJmLm10dCA9IFJPQ1I6OnBlcmZvcm1hbmNlKHJvY3IucHJlZC5tdHQsIG1lYXN1cmUgPSAidHByIiwgeC5tZWFzdXJlID0gImZwciIpICMgI1JPQ1IgcGVyZm9ybWFuY2Ugb2JqZWN0CnBsb3Qocm9jLnBlcmYubXR0LCBjb2wgPSAiYmx1ZSIpCmFibGluZShhID0gMCwgYiA9IDEsIGx0eSA9IDIpICNkaWFnb25hbCBmb3IgcmFuZG9tIGFzc2lnbm1lbnQKYGBgCgojIyMgUmVwb3J0IEFVQyBmcm9tIFJPQyBmb3IgdHJhaW5pbmcgYW5kIHRlc3QgZGF0YQpgYGB7ciBBVUMxMn0KICAjIFRyYWluIEFVQwphdWMgPC0gUk9DUjo6cGVyZm9ybWFuY2Uocm9jci5wcmVkLm10dCwgbWVhc3VyZSA9ICJhdWMiKQogIGF1YyA8LSBhdWNAeS52YWx1ZXNbWzFdXQogIHByaW50KGF1YykKYGBgCgoKIyMjIENhbGN1bGF0ZSBOYWdlbGtlcmtlIFJeMgpgYGB7ciBJNDJhaX0KTmFnZWxrZXJrZVIyKG0pCmBgYAoKIyMjIGNoZWNrIGFzc3VtcHRpb25zIG9mIG1vZGVsCiMjIyMgQ29vaydzIGRpc3RhbmNlCmBgYHtyIEk0M2l9CnBsb3QobSwgd2hpY2ggPSA0LCBpZC5uID0gMykKYGBgCgojIyMjIEV4dHJhY3QgbW9kZWwgcmVzdWx0cyBhbmQgZGlzcGxheSBkYXRhIGZvciB0b3AgMyB2YWx1ZXMgdXNpbmcgQ29vaydzIGRpc3RhbmNlCmBgYHtyIEk0NGl9Cm1vZGVsLmRhdGEgPC0gYXVnbWVudChtKSAlPiUgCiAgbXV0YXRlKGluZGV4ID0gMTpuKCkpIAptb2RlbC5kYXRhICU+JSB0b3BfbigzLCAuY29va3NkKQpgYGAKCiMjIyMgcGxvdCBzdGFuZGFyZGlzZWQgcmVzaWR1YWxzCmBgYHtyIEk0NWl9CmdncGxvdChtb2RlbC5kYXRhLCBhZXMoaW5kZXgsIC5zdGQucmVzaWQpKSArIAogIGdlb21fcG9pbnQoYWVzKGNvbG9yID0gSGVhbHRoX0JpbmFyeSksIGFscGhhID0gLjUpICsKICB0aGVtZV9idygpCmBgYAoKIyMjIyBGaWx0ZXIgcG90ZW50aWFsIGluZmx1ZW50aWFsIGRhdGEgcG9pbnRzIHdpdGggYWJzKC5zdGQucmVzKSA+IDM6CmBgYHtyIEk0Nml9Cm1vZGVsLmRhdGEgJT4lIAogIGZpbHRlcihhYnMoLnN0ZC5yZXNpZCkgPiAzKQpgYGAKCgoKIyMgQU5JTUFMIENBUkVFUiAyIEJpbmFyeSBsb2dpc3RpYyByZWdyZXNzaW9uIE9OIFNJR05JRklDQU5UIE9SIFNFUklPVVMgSUxMTkVTUwpgYGB7ciBJNDd9CiMgZml0IGJpbmFyeSBsb2dpdCBtb2RlbCBhbmQgc3RvcmUgcmVzdWx0cyAnbScKbSA8LSBnbG0oSGVhbHRoX0JpbmFyeSB+IEFuaW1hbF9DYXJlZXIyLCBkYXRhID0gbWwsZmFtaWx5ID0gYmlub21pYWwpCiMgdmlldyBhIHN1bW1hcnkgb2YgdGhlIG1vZGVsCnN1bW1hcnkobSkKIyB0ZXN0IG1vZGVsIGZpdAp3aXRoKG0sIG51bGwuZGV2aWFuY2UgLSBkZXZpYW5jZSkKd2l0aChtLCBkZi5udWxsIC0gZGYucmVzaWR1YWwpCndpdGgobSwgcGNoaXNxKG51bGwuZGV2aWFuY2UgLSBkZXZpYW5jZSwgZGYubnVsbCAtIGRmLnJlc2lkdWFsLCBsb3dlci50YWlsID0gRkFMU0UpKQpCSUMobSkKIyBIb3NtZXItTGVtZXNob3cgR29vZG5lc3Mtb2YtRml0IFRlc3QKIyMgQ0lzIHVzaW5nIHByb2ZpbGVkIGxvZy1saWtlbGlob29kCmNvbmZpbnQobSwgbGV2ZWw9MC45OSkKIyMgQ0lzIHVzaW5nIHN0YW5kYXJkIGVycm9ycwpjb25maW50LmRlZmF1bHQobSwgbGV2ZWw9MC45OSkKIyBXYWxkIHRlc3QKd2FsZC50ZXN0KGIgPSBjb2VmKG0pLCBTaWdtYSA9IHZjb3YobSksIFRlcm1zID0gMikKIyMgb2RkcyByYXRpb3MgYW5kIDk1JSBDSQpleHAoY2JpbmQoT1IgPSBjb2VmKG0pLCBjb25maW50KG0sIGxldmVsPTAuOTkpKSkKYGBgCgojIyMgQ3JlYXRlIFJPQ1IgZnJvbSBkYXRhCmBgYHtyIFJPQzEzfQojIyB0cmFpbmluZyBkYXRhCnByZWQubXR0ID0gcHJlZGljdChtLCB0eXBlID0gInJlc3BvbnNlIikgI3JlcGVhdCByaXNrIHByZWRpY3Rpb25zIGZyb20gbW9kZWwgbQpyb2NyLnByZWQubXR0ID0gUk9DUjo6cHJlZGljdGlvbihwcmVkLm10dCwgbGFiZWxzID0gbWwkSGVhbHRoX0JpbmFyeSkgI1JPQ1IgcHJlZGljdGlvbiBvYmplY3QKcm9jLnBlcmYubXR0ID0gUk9DUjo6cGVyZm9ybWFuY2Uocm9jci5wcmVkLm10dCwgbWVhc3VyZSA9ICJ0cHIiLCB4Lm1lYXN1cmUgPSAiZnByIikgIyAjUk9DUiBwZXJmb3JtYW5jZSBvYmplY3QKcGxvdChyb2MucGVyZi5tdHQsIGNvbCA9ICJibHVlIikKYWJsaW5lKGEgPSAwLCBiID0gMSwgbHR5ID0gMikgI2RpYWdvbmFsIGZvciByYW5kb20gYXNzaWdubWVudApgYGAKCiMjIyBSZXBvcnQgQVVDIGZyb20gUk9DIGZvciB0cmFpbmluZyBhbmQgdGVzdCBkYXRhCmBgYHtyIEFVQzEzfQogICMgVHJhaW4gQVVDCmF1YyA8LSBST0NSOjpwZXJmb3JtYW5jZShyb2NyLnByZWQubXR0LCBtZWFzdXJlID0gImF1YyIpCiAgYXVjIDwtIGF1Y0B5LnZhbHVlc1tbMV1dCiAgcHJpbnQoYXVjKQpgYGAKCgojIyMgQ2FsY3VsYXRlIE5hZ2Vsa2Vya2UgUl4yCmBgYHtyIEk0N2F9Ck5hZ2Vsa2Vya2VSMihtKQpgYGAKCiMjIyBjaGVjayBhc3N1bXB0aW9ucyBvZiBtb2RlbAojIyMjIENvb2sncyBkaXN0YW5jZQpgYGB7ciBJNDh9CnBsb3QobSwgd2hpY2ggPSA0LCBpZC5uID0gMykKYGBgCgojIyMjIEV4dHJhY3QgbW9kZWwgcmVzdWx0cyBhbmQgZGlzcGxheSBkYXRhIGZvciB0b3AgMyB2YWx1ZXMgdXNpbmcgQ29vaydzIGRpc3RhbmNlCmBgYHtyIEk0OX0KbW9kZWwuZGF0YSA8LSBhdWdtZW50KG0pICU+JSAKICBtdXRhdGUoaW5kZXggPSAxOm4oKSkgCm1vZGVsLmRhdGEgJT4lIHRvcF9uKDMsIC5jb29rc2QpCmBgYAoKIyMjIyBwbG90IHN0YW5kYXJkaXNlZCByZXNpZHVhbHMKYGBge3IgSTUwfQpnZ3Bsb3QobW9kZWwuZGF0YSwgYWVzKGluZGV4LCAuc3RkLnJlc2lkKSkgKyAKICBnZW9tX3BvaW50KGFlcyhjb2xvciA9IEhlYWx0aF9CaW5hcnkpLCBhbHBoYSA9IC41KSArCiAgdGhlbWVfYncoKQpgYGAKCiMgRmlsdGVyIHBvdGVudGlhbCBpbmZsdWVudGlhbCBkYXRhIHBvaW50cyB3aXRoIGFicyguc3RkLnJlcykgPiAzOgpgYGB7ciBJNTF9Cm1vZGVsLmRhdGEgJT4lIAogIGZpbHRlcihhYnMoLnN0ZC5yZXNpZCkgPiAzKQpgYGAKCgoKIyMgQU5JTUFMX0NBUkVFUl9CSU5BUlkgQmluYXJ5IGxvZ2lzdGljIHJlZ3Jlc3Npb24gT04gU0lHTklGSUNBTlQgT1IgU0VSSU9VUyBJTExORVNTCmBgYHtyIEk1Mn0KIyBmaXQgYmluYXJ5IGxvZ2l0IG1vZGVsIGFuZCBzdG9yZSByZXN1bHRzICdtJwptIDwtIGdsbShIZWFsdGhfQmluYXJ5IH4gQW5pbWFsX0NhcmVlcl9CSU5BUlksIGRhdGEgPSBtbCxmYW1pbHkgPSBiaW5vbWlhbCkKIyB2aWV3IGEgc3VtbWFyeSBvZiB0aGUgbW9kZWwKc3VtbWFyeShtKQojIHRlc3QgbW9kZWwgZml0CndpdGgobSwgbnVsbC5kZXZpYW5jZSAtIGRldmlhbmNlKQp3aXRoKG0sIGRmLm51bGwgLSBkZi5yZXNpZHVhbCkKd2l0aChtLCBwY2hpc3EobnVsbC5kZXZpYW5jZSAtIGRldmlhbmNlLCBkZi5udWxsIC0gZGYucmVzaWR1YWwsIGxvd2VyLnRhaWwgPSBGQUxTRSkpCkJJQyhtKQoKIyMgQ0lzIHVzaW5nIHByb2ZpbGVkIGxvZy1saWtlbGlob29kCmNvbmZpbnQobSwgbGV2ZWw9MC45OSkKIyMgQ0lzIHVzaW5nIHN0YW5kYXJkIGVycm9ycwpjb25maW50LmRlZmF1bHQobSwgbGV2ZWw9MC45OSkKIyBXYWxkIHRlc3QKd2FsZC50ZXN0KGIgPSBjb2VmKG0pLCBTaWdtYSA9IHZjb3YobSksIFRlcm1zID0gMikKIyMgb2RkcyByYXRpb3MgYW5kIDk1JSBDSQpleHAoY2JpbmQoT1IgPSBjb2VmKG0pLCBjb25maW50KG0sIGxldmVsPTAuOTkpKSkKYGBgCgojIyMgQ3JlYXRlIFJPQ1IgZnJvbSBkYXRhCmBgYHtyIFJPQzE0fQojIyB0cmFpbmluZyBkYXRhCnByZWQubXR0ID0gcHJlZGljdChtLCB0eXBlID0gInJlc3BvbnNlIikgI3JlcGVhdCByaXNrIHByZWRpY3Rpb25zIGZyb20gbW9kZWwgbQpyb2NyLnByZWQubXR0ID0gUk9DUjo6cHJlZGljdGlvbihwcmVkLm10dCwgbGFiZWxzID0gbWwkSGVhbHRoX0JpbmFyeSkgI1JPQ1IgcHJlZGljdGlvbiBvYmplY3QKcm9jLnBlcmYubXR0ID0gUk9DUjo6cGVyZm9ybWFuY2Uocm9jci5wcmVkLm10dCwgbWVhc3VyZSA9ICJ0cHIiLCB4Lm1lYXN1cmUgPSAiZnByIikgIyAjUk9DUiBwZXJmb3JtYW5jZSBvYmplY3QKcGxvdChyb2MucGVyZi5tdHQsIGNvbCA9ICJibHVlIikKYWJsaW5lKGEgPSAwLCBiID0gMSwgbHR5ID0gMikgI2RpYWdvbmFsIGZvciByYW5kb20gYXNzaWdubWVudApgYGAKCiMjIyBSZXBvcnQgQVVDIGZyb20gUk9DIGZvciB0cmFpbmluZyBhbmQgdGVzdCBkYXRhCmBgYHtyIEFVQzE0fQogICMgVHJhaW4gQVVDCmF1YyA8LSBST0NSOjpwZXJmb3JtYW5jZShyb2NyLnByZWQubXR0LCBtZWFzdXJlID0gImF1YyIpCiAgYXVjIDwtIGF1Y0B5LnZhbHVlc1tbMV1dCiAgcHJpbnQoYXVjKQpgYGAKCgojIyMgQ2FsY3VsYXRlIE5hZ2Vsa2Vya2UgUl4yCmBgYHtyIEk1MmF9Ck5hZ2Vsa2Vya2VSMihtKQpgYGAKCiMjIyBjaGVjayBhc3N1bXB0aW9ucyBvZiBtb2RlbAojIyMjIENvb2sncyBkaXN0YW5jZQpgYGB7ciBJNTN9CnBsb3QobSwgd2hpY2ggPSA0LCBpZC5uID0gMykKYGBgCgojIyMjIEV4dHJhY3QgbW9kZWwgcmVzdWx0cyBhbmQgZGlzcGxheSBkYXRhIGZvciB0b3AgMyB2YWx1ZXMgdXNpbmcgQ29vaydzIGRpc3RhbmNlCmBgYHtyIEk1NH0KbW9kZWwuZGF0YSA8LSBhdWdtZW50KG0pICU+JSAKICBtdXRhdGUoaW5kZXggPSAxOm4oKSkgCm1vZGVsLmRhdGEgJT4lIHRvcF9uKDMsIC5jb29rc2QpCmBgYAoKIyMjIyBwbG90IHN0YW5kYXJkaXNlZCByZXNpZHVhbHMKYGBge3IgSTU1fQpnZ3Bsb3QobW9kZWwuZGF0YSwgYWVzKGluZGV4LCAuc3RkLnJlc2lkKSkgKyAKICBnZW9tX3BvaW50KGFlcyhjb2xvciA9IEhlYWx0aF9CaW5hcnkpLCBhbHBoYSA9IC41KSArCiAgdGhlbWVfYncoKQpgYGAKCiMjIyMgRmlsdGVyIHBvdGVudGlhbCBpbmZsdWVudGlhbCBkYXRhIHBvaW50cyB3aXRoIGFicyguc3RkLnJlcykgPiAzOgpgYGB7ciBJNTZ9Cm1vZGVsLmRhdGEgJT4lIAogIGZpbHRlcihhYnMoLnN0ZC5yZXNpZCkgPiAzKQpgYGAKCgoKIyMgSU5DT01FIEJpbmFyeSBsb2dpc3RpYyByZWdyZXNzaW9uIE9OIFNJR05JRklDQU5UIE9SIFNFUklPVVMgSUxMTkVTUwpgYGB7ciBJNTl9CiMgZml0IGJpbmFyeSBsb2dpdCBtb2RlbCBhbmQgc3RvcmUgcmVzdWx0cyAnbScKbSA8LSBnbG0oSGVhbHRoX0JpbmFyeSB+IEluY29tZSwgZGF0YSA9IG1sLGZhbWlseSA9IGJpbm9taWFsKQojIHZpZXcgYSBzdW1tYXJ5IG9mIHRoZSBtb2RlbApzdW1tYXJ5KG0pCiMgdGVzdCBtb2RlbCBmaXQKd2l0aChtLCBudWxsLmRldmlhbmNlIC0gZGV2aWFuY2UpCndpdGgobSwgZGYubnVsbCAtIGRmLnJlc2lkdWFsKQp3aXRoKG0sIHBjaGlzcShudWxsLmRldmlhbmNlIC0gZGV2aWFuY2UsIGRmLm51bGwgLSBkZi5yZXNpZHVhbCwgbG93ZXIudGFpbCA9IEZBTFNFKSkKQklDKG0pCiMgSG9zbWVyLUxlbWVzaG93IEdvb2RuZXNzLW9mLUZpdCBUZXN0CmdsbXRvb2xib3g6OmhsdGVzdChtKQojIyBDSXMgdXNpbmcgcHJvZmlsZWQgbG9nLWxpa2VsaWhvb2QKY29uZmludChtLCBsZXZlbD0wLjk5KQojIyBDSXMgdXNpbmcgc3RhbmRhcmQgZXJyb3JzCmNvbmZpbnQuZGVmYXVsdChtLCBsZXZlbD0wLjk5KQojIFdhbGQgdGVzdAp3YWxkLnRlc3QoYiA9IGNvZWYobSksIFNpZ21hID0gdmNvdihtKSwgVGVybXMgPSAyKQojIyBvZGRzIHJhdGlvcyBhbmQgOTUlIENJCmV4cChjYmluZChPUiA9IGNvZWYobSksIGNvbmZpbnQobSwgbGV2ZWw9MC45OSkpKQpgYGAKCiMjIyBDcmVhdGUgUk9DUiBmcm9tIGRhdGEKYGBge3IgUk9DMTV9CiMjIHRyYWluaW5nIGRhdGEKcHJlZC5tdHQgPSBwcmVkaWN0KG0sIHR5cGUgPSAicmVzcG9uc2UiKSAjcmVwZWF0IHJpc2sgcHJlZGljdGlvbnMgZnJvbSBtb2RlbCBtCnJvY3IucHJlZC5tdHQgPSBST0NSOjpwcmVkaWN0aW9uKHByZWQubXR0LCBsYWJlbHMgPSBtbCRIZWFsdGhfQmluYXJ5KSAjUk9DUiBwcmVkaWN0aW9uIG9iamVjdApyb2MucGVyZi5tdHQgPSBST0NSOjpwZXJmb3JtYW5jZShyb2NyLnByZWQubXR0LCBtZWFzdXJlID0gInRwciIsIHgubWVhc3VyZSA9ICJmcHIiKSAjICNST0NSIHBlcmZvcm1hbmNlIG9iamVjdApwbG90KHJvYy5wZXJmLm10dCwgY29sID0gImJsdWUiKQphYmxpbmUoYSA9IDAsIGIgPSAxLCBsdHkgPSAyKSAjZGlhZ29uYWwgZm9yIHJhbmRvbSBhc3NpZ25tZW50CmBgYAoKIyMjIFJlcG9ydCBBVUMgZnJvbSBST0MgZm9yIHRyYWluaW5nIGFuZCB0ZXN0IGRhdGEKYGBge3IgQVVDMTV9CiAgIyBUcmFpbiBBVUMKYXVjIDwtIFJPQ1I6OnBlcmZvcm1hbmNlKHJvY3IucHJlZC5tdHQsIG1lYXN1cmUgPSAiYXVjIikKICBhdWMgPC0gYXVjQHkudmFsdWVzW1sxXV0KICBwcmludChhdWMpCmBgYAoKCiMjIyBDYWxjdWxhdGUgTmFnZWxrZXJrZSBSXjIKYGBge3IgSTU5YX0KTmFnZWxrZXJrZVIyKG0pCmBgYAoKIyMjIGNoZWNrIGFzc3VtcHRpb25zIG9mIG1vZGVsCiMjIyMgQ29vaydzIGRpc3RhbmNlCmBgYHtyIEk2MH0KcGxvdChtLCB3aGljaCA9IDQsIGlkLm4gPSAzKQpgYGAKCiMjIyMgRXh0cmFjdCBtb2RlbCByZXN1bHRzIGFuZCBkaXNwbGF5IGRhdGEgZm9yIHRvcCAzIHZhbHVlcyB1c2luZyBDb29rJ3MgZGlzdGFuY2UKYGBge3IgSTYxfQptb2RlbC5kYXRhIDwtIGF1Z21lbnQobSkgJT4lIAogIG11dGF0ZShpbmRleCA9IDE6bigpKSAKbW9kZWwuZGF0YSAlPiUgdG9wX24oMywgLmNvb2tzZCkKYGBgCgojIyMjIHBsb3Qgc3RhbmRhcmRpc2VkIHJlc2lkdWFscwpgYGB7ciBJNjJ9CmdncGxvdChtb2RlbC5kYXRhLCBhZXMoaW5kZXgsIC5zdGQucmVzaWQpKSArIAogIGdlb21fcG9pbnQoYWVzKGNvbG9yID0gSGVhbHRoX0JpbmFyeSksIGFscGhhID0gLjUpICsKICB0aGVtZV9idygpCmBgYAoKIyMjIyBGaWx0ZXIgcG90ZW50aWFsIGluZmx1ZW50aWFsIGRhdGEgcG9pbnRzIHdpdGggYWJzKC5zdGQucmVzKSA+IDM6CmBgYHtyIEk2M30KbW9kZWwuZGF0YSAlPiUgCiAgZmlsdGVyKGFicyguc3RkLnJlc2lkKSA+IDMpCmBgYAoKCgojIyBJTkNPTUUyIEJpbmFyeSBsb2dpc3RpYyByZWdyZXNzaW9uIE9OIFNJR05JRklDQU5UIE9SIFNFUklPVVMgSUxMTkVTUwpgYGB7ciBJNTlpfQojIGZpdCBiaW5hcnkgbG9naXQgbW9kZWwgYW5kIHN0b3JlIHJlc3VsdHMgJ20nCm0gPC0gZ2xtKEhlYWx0aF9CaW5hcnkgfiBJbmNvbWUyLCBkYXRhID0gbWwsZmFtaWx5ID0gYmlub21pYWwpCiMgdmlldyBhIHN1bW1hcnkgb2YgdGhlIG1vZGVsCnN1bW1hcnkobSkKIyB0ZXN0IG1vZGVsIGZpdAp3aXRoKG0sIG51bGwuZGV2aWFuY2UgLSBkZXZpYW5jZSkKd2l0aChtLCBkZi5udWxsIC0gZGYucmVzaWR1YWwpCndpdGgobSwgcGNoaXNxKG51bGwuZGV2aWFuY2UgLSBkZXZpYW5jZSwgZGYubnVsbCAtIGRmLnJlc2lkdWFsLCBsb3dlci50YWlsID0gRkFMU0UpKQpCSUMobSkKIyBIb3NtZXItTGVtZXNob3cgR29vZG5lc3Mtb2YtRml0IFRlc3QKZ2xtdG9vbGJveDo6aGx0ZXN0KG0pCiMjIENJcyB1c2luZyBwcm9maWxlZCBsb2ctbGlrZWxpaG9vZApjb25maW50KG0sIGxldmVsPTAuOTkpCiMjIENJcyB1c2luZyBzdGFuZGFyZCBlcnJvcnMKY29uZmludC5kZWZhdWx0KG0sIGxldmVsPTAuOTkpCiMgV2FsZCB0ZXN0CndhbGQudGVzdChiID0gY29lZihtKSwgU2lnbWEgPSB2Y292KG0pLCBUZXJtcyA9IDIpCiMjIG9kZHMgcmF0aW9zIGFuZCA5NSUgQ0kKZXhwKGNiaW5kKE9SID0gY29lZihtKSwgY29uZmludChtLCBsZXZlbD0wLjk5KSkpCmBgYAoKIyMjIENyZWF0ZSBST0NSIGZyb20gZGF0YQpgYGB7ciBST0MxNn0KIyMgdHJhaW5pbmcgZGF0YQpwcmVkLm10dCA9IHByZWRpY3QobSwgdHlwZSA9ICJyZXNwb25zZSIpICNyZXBlYXQgcmlzayBwcmVkaWN0aW9ucyBmcm9tIG1vZGVsIG0Kcm9jci5wcmVkLm10dCA9IFJPQ1I6OnByZWRpY3Rpb24ocHJlZC5tdHQsIGxhYmVscyA9IG1sJEhlYWx0aF9CaW5hcnkpICNST0NSIHByZWRpY3Rpb24gb2JqZWN0CnJvYy5wZXJmLm10dCA9IFJPQ1I6OnBlcmZvcm1hbmNlKHJvY3IucHJlZC5tdHQsIG1lYXN1cmUgPSAidHByIiwgeC5tZWFzdXJlID0gImZwciIpICMgI1JPQ1IgcGVyZm9ybWFuY2Ugb2JqZWN0CnBsb3Qocm9jLnBlcmYubXR0LCBjb2wgPSAiYmx1ZSIpCmFibGluZShhID0gMCwgYiA9IDEsIGx0eSA9IDIpICNkaWFnb25hbCBmb3IgcmFuZG9tIGFzc2lnbm1lbnQKYGBgCgojIyMgUmVwb3J0IEFVQyBmcm9tIFJPQyBmb3IgdHJhaW5pbmcgYW5kIHRlc3QgZGF0YQpgYGB7ciBBVUMxNn0KICAjIFRyYWluIEFVQwphdWMgPC0gUk9DUjo6cGVyZm9ybWFuY2Uocm9jci5wcmVkLm10dCwgbWVhc3VyZSA9ICJhdWMiKQogIGF1YyA8LSBhdWNAeS52YWx1ZXNbWzFdXQogIHByaW50KGF1YykKYGBgCgoKIyMjIENhbGN1bGF0ZSBOYWdlbGtlcmtlIFJeMgpgYGB7ciBJNTlhaX0KTmFnZWxrZXJrZVIyKG0pCmBgYAoKIyMjIGNoZWNrIGFzc3VtcHRpb25zIG9mIG1vZGVsCiMjIyMgQ29vaydzIGRpc3RhbmNlCmBgYHtyIEk2MGl9CnBsb3QobSwgd2hpY2ggPSA0LCBpZC5uID0gMykKYGBgCgojIyMjIEV4dHJhY3QgbW9kZWwgcmVzdWx0cyBhbmQgZGlzcGxheSBkYXRhIGZvciB0b3AgMyB2YWx1ZXMgdXNpbmcgQ29vaydzIGRpc3RhbmNlCmBgYHtyIEk2MWl9Cm1vZGVsLmRhdGEgPC0gYXVnbWVudChtKSAlPiUgCiAgbXV0YXRlKGluZGV4ID0gMTpuKCkpIAptb2RlbC5kYXRhICU+JSB0b3BfbigzLCAuY29va3NkKQpgYGAKCiMjIyMgcGxvdCBzdGFuZGFyZGlzZWQgcmVzaWR1YWxzCmBgYHtyIEk2Mml9CmdncGxvdChtb2RlbC5kYXRhLCBhZXMoaW5kZXgsIC5zdGQucmVzaWQpKSArIAogIGdlb21fcG9pbnQoYWVzKGNvbG9yID0gSGVhbHRoX0JpbmFyeSksIGFscGhhID0gLjUpICsKICB0aGVtZV9idygpCmBgYAoKIyMjIyBGaWx0ZXIgcG90ZW50aWFsIGluZmx1ZW50aWFsIGRhdGEgcG9pbnRzIHdpdGggYWJzKC5zdGQucmVzKSA+IDM6CmBgYHtyIEk2M2l9Cm1vZGVsLmRhdGEgJT4lIAogIGZpbHRlcihhYnMoLnN0ZC5yZXNpZCkgPiAzKQpgYGAKCgoKIyMgQ19BR0UgQmluYXJ5IGxvZ2lzdGljIHJlZ3Jlc3Npb24gT04gU0lHTklGSUNBTlQgT1IgU0VSSU9VUyBJTExORVNTCmBgYHtyIEk2NH0KIyBmaXQgYmluYXJ5IGxvZ2l0IG1vZGVsIGFuZCBzdG9yZSByZXN1bHRzICdtJwptIDwtIGdsbShIZWFsdGhfQmluYXJ5IH4gQ19BZ2UsIGRhdGEgPSBtbCxmYW1pbHkgPSBiaW5vbWlhbCkKIyB2aWV3IGEgc3VtbWFyeSBvZiB0aGUgbW9kZWwKc3VtbWFyeShtKQojIHRlc3QgbW9kZWwgZml0CndpdGgobSwgbnVsbC5kZXZpYW5jZSAtIGRldmlhbmNlKQp3aXRoKG0sIGRmLm51bGwgLSBkZi5yZXNpZHVhbCkKd2l0aChtLCBwY2hpc3EobnVsbC5kZXZpYW5jZSAtIGRldmlhbmNlLCBkZi5udWxsIC0gZGYucmVzaWR1YWwsIGxvd2VyLnRhaWwgPSBGQUxTRSkpCkJJQyhtKQojIEhvc21lci1MZW1lc2hvdyBHb29kbmVzcy1vZi1GaXQgVGVzdApobHRlc3QobSwgRz00KQojIyBDSXMgdXNpbmcgcHJvZmlsZWQgbG9nLWxpa2VsaWhvb2QKY29uZmludChtLCBsZXZlbD0wLjk5KQojIyBDSXMgdXNpbmcgc3RhbmRhcmQgZXJyb3JzCmNvbmZpbnQuZGVmYXVsdChtLCBsZXZlbD0wLjk5KQojIFdhbGQgdGVzdAp3YWxkLnRlc3QoYiA9IGNvZWYobSksIFNpZ21hID0gdmNvdihtKSwgVGVybXMgPSAyKQojIyBvZGRzIHJhdGlvcyBhbmQgOTUlIENJCmV4cChjYmluZChPUiA9IGNvZWYobSksIGNvbmZpbnQobSwgbGV2ZWw9MC45OSkpKQpgYGAKCiMjIyBDcmVhdGUgUk9DUiBmcm9tIGRhdGEKYGBge3IgUk9DMTd9CiMjIHRyYWluaW5nIGRhdGEKcHJlZC5tdHQgPSBwcmVkaWN0KG0sIHR5cGUgPSAicmVzcG9uc2UiKSAjcmVwZWF0IHJpc2sgcHJlZGljdGlvbnMgZnJvbSBtb2RlbCBtCnJvY3IucHJlZC5tdHQgPSBST0NSOjpwcmVkaWN0aW9uKHByZWQubXR0LCBsYWJlbHMgPSBtbCRIZWFsdGhfQmluYXJ5KSAjUk9DUiBwcmVkaWN0aW9uIG9iamVjdApyb2MucGVyZi5tdHQgPSBST0NSOjpwZXJmb3JtYW5jZShyb2NyLnByZWQubXR0LCBtZWFzdXJlID0gInRwciIsIHgubWVhc3VyZSA9ICJmcHIiKSAjICNST0NSIHBlcmZvcm1hbmNlIG9iamVjdApwbG90KHJvYy5wZXJmLm10dCwgY29sID0gImJsdWUiKQphYmxpbmUoYSA9IDAsIGIgPSAxLCBsdHkgPSAyKSAjZGlhZ29uYWwgZm9yIHJhbmRvbSBhc3NpZ25tZW50CmBgYAoKIyMjIFJlcG9ydCBBVUMgZnJvbSBST0MgZm9yIHRyYWluaW5nIGFuZCB0ZXN0IGRhdGEKYGBge3IgQVVDMTd9CiAgIyBUcmFpbiBBVUMKYXVjIDwtIFJPQ1I6OnBlcmZvcm1hbmNlKHJvY3IucHJlZC5tdHQsIG1lYXN1cmUgPSAiYXVjIikKICBhdWMgPC0gYXVjQHkudmFsdWVzW1sxXV0KICBwcmludChhdWMpCmBgYAoKCiMjIyBDYWxjdWxhdGUgTmFnZWxrZXJrZSBSXjIKYGBge3IgSTY0YX0KTmFnZWxrZXJrZVIyKG0pCmBgYAoKIyMjIGNoZWNrIGFzc3VtcHRpb25zIG9mIG1vZGVsCiMjIyMgQ29vaydzIGRpc3RhbmNlCmBgYHtyIEk2NX0KcGxvdChtLCB3aGljaCA9IDQsIGlkLm4gPSAzKQpgYGAKCiMjIyMgRXh0cmFjdCBtb2RlbCByZXN1bHRzIGFuZCBkaXNwbGF5IGRhdGEgZm9yIHRvcCAzIHZhbHVlcyB1c2luZyBDb29rJ3MgZGlzdGFuY2UKYGBge3IgSTY2fQptb2RlbC5kYXRhIDwtIGF1Z21lbnQobSkgJT4lIAogIG11dGF0ZShpbmRleCA9IDE6bigpKSAKbW9kZWwuZGF0YSAlPiUgdG9wX24oMywgLmNvb2tzZCkKYGBgCgojIyMjIHBsb3Qgc3RhbmRhcmRpc2VkIHJlc2lkdWFscwpgYGB7ciBJNjd9CmdncGxvdChtb2RlbC5kYXRhLCBhZXMoaW5kZXgsIC5zdGQucmVzaWQpKSArIAogIGdlb21fcG9pbnQoYWVzKGNvbG9yID0gSGVhbHRoX0JpbmFyeSksIGFscGhhID0gLjUpICsKICB0aGVtZV9idygpCmBgYAoKIyMjIyBGaWx0ZXIgcG90ZW50aWFsIGluZmx1ZW50aWFsIGRhdGEgcG9pbnRzIHdpdGggYWJzKC5zdGQucmVzKSA+IDM6CmBgYHtyIEk2OH0KbW9kZWwuZGF0YSAlPiUgCiAgZmlsdGVyKGFicyguc3RkLnJlc2lkKSA+IDMpCmBgYAoKCgojIyBDX0FHRTIgQmluYXJ5IGxvZ2lzdGljIHJlZ3Jlc3Npb24gT04gU0lHTklGSUNBTlQgT1IgU0VSSU9VUyBJTExORVNTCmBgYHtyIEk2NGl9CiMgZml0IGJpbmFyeSBsb2dpdCBtb2RlbCBhbmQgc3RvcmUgcmVzdWx0cyAnbScKbSA8LSBnbG0oSGVhbHRoX0JpbmFyeSB+IENfQWdlMiwgZGF0YSA9IG1sLGZhbWlseSA9IGJpbm9taWFsKQojIHZpZXcgYSBzdW1tYXJ5IG9mIHRoZSBtb2RlbApzdW1tYXJ5KG0pCiMgdGVzdCBtb2RlbCBmaXQKd2l0aChtLCBudWxsLmRldmlhbmNlIC0gZGV2aWFuY2UpCndpdGgobSwgZGYubnVsbCAtIGRmLnJlc2lkdWFsKQp3aXRoKG0sIHBjaGlzcShudWxsLmRldmlhbmNlIC0gZGV2aWFuY2UsIGRmLm51bGwgLSBkZi5yZXNpZHVhbCwgbG93ZXIudGFpbCA9IEZBTFNFKSkKQklDKG0pCiMgSG9zbWVyLUxlbWVzaG93IEdvb2RuZXNzLW9mLUZpdCBUZXN0CmhsdGVzdChtLCBHPTQpCiMjIENJcyB1c2luZyBwcm9maWxlZCBsb2ctbGlrZWxpaG9vZApjb25maW50KG0sIGxldmVsPTAuOTkpCiMjIENJcyB1c2luZyBzdGFuZGFyZCBlcnJvcnMKY29uZmludC5kZWZhdWx0KG0sIGxldmVsPTAuOTkpCiMgV2FsZCB0ZXN0CndhbGQudGVzdChiID0gY29lZihtKSwgU2lnbWEgPSB2Y292KG0pLCBUZXJtcyA9IDIpCiMjIG9kZHMgcmF0aW9zIGFuZCA5NSUgQ0kKZXhwKGNiaW5kKE9SID0gY29lZihtKSwgY29uZmludChtLCBsZXZlbD0wLjk5KSkpCmBgYAoKIyMjIENyZWF0ZSBST0NSIGZyb20gZGF0YQpgYGB7ciBST0MxOH0KIyMgdHJhaW5pbmcgZGF0YQpwcmVkLm10dCA9IHByZWRpY3QobSwgdHlwZSA9ICJyZXNwb25zZSIpICNyZXBlYXQgcmlzayBwcmVkaWN0aW9ucyBmcm9tIG1vZGVsIG0Kcm9jci5wcmVkLm10dCA9IFJPQ1I6OnByZWRpY3Rpb24ocHJlZC5tdHQsIGxhYmVscyA9IG1sJEhlYWx0aF9CaW5hcnkpICNST0NSIHByZWRpY3Rpb24gb2JqZWN0CnJvYy5wZXJmLm10dCA9IFJPQ1I6OnBlcmZvcm1hbmNlKHJvY3IucHJlZC5tdHQsIG1lYXN1cmUgPSAidHByIiwgeC5tZWFzdXJlID0gImZwciIpICMgI1JPQ1IgcGVyZm9ybWFuY2Ugb2JqZWN0CnBsb3Qocm9jLnBlcmYubXR0LCBjb2wgPSAiYmx1ZSIpCmFibGluZShhID0gMCwgYiA9IDEsIGx0eSA9IDIpICNkaWFnb25hbCBmb3IgcmFuZG9tIGFzc2lnbm1lbnQKYGBgCgojIyMgUmVwb3J0IEFVQyBmcm9tIFJPQyBmb3IgdHJhaW5pbmcgYW5kIHRlc3QgZGF0YQpgYGB7ciBBVUMxOH0KICAjIFRyYWluIEFVQwphdWMgPC0gUk9DUjo6cGVyZm9ybWFuY2Uocm9jci5wcmVkLm10dCwgbWVhc3VyZSA9ICJhdWMiKQogIGF1YyA8LSBhdWNAeS52YWx1ZXNbWzFdXQogIHByaW50KGF1YykKYGBgCgoKIyMgQ2FsY3VsYXRlIE5hZ2Vsa2Vya2UgUl4yCmBgYHtyIEk2NGFpfQpOYWdlbGtlcmtlUjIobSkKYGBgCgojIyBjaGVjayBhc3N1bXB0aW9ucyBvZiBtb2RlbAojIyMjIENvb2sncyBkaXN0YW5jZQpgYGB7ciBJNjVpfQpwbG90KG0sIHdoaWNoID0gNCwgaWQubiA9IDMpCmBgYAoKIyMjIyBFeHRyYWN0IG1vZGVsIHJlc3VsdHMgYW5kIGRpc3BsYXkgZGF0YSBmb3IgdG9wIDMgdmFsdWVzIHVzaW5nIENvb2sncyBkaXN0YW5jZQpgYGB7ciBJNjZpfQptb2RlbC5kYXRhIDwtIGF1Z21lbnQobSkgJT4lIAogIG11dGF0ZShpbmRleCA9IDE6bigpKSAKbW9kZWwuZGF0YSAlPiUgdG9wX24oMywgLmNvb2tzZCkKYGBgCgojIyMjIHBsb3Qgc3RhbmRhcmRpc2VkIHJlc2lkdWFscwpgYGB7ciBJNjdpfQpnZ3Bsb3QobW9kZWwuZGF0YSwgYWVzKGluZGV4LCAuc3RkLnJlc2lkKSkgKyAKICBnZW9tX3BvaW50KGFlcyhjb2xvciA9IEhlYWx0aF9CaW5hcnkpLCBhbHBoYSA9IC41KSArCiAgdGhlbWVfYncoKQpgYGAKCiMjIyMgRmlsdGVyIHBvdGVudGlhbCBpbmZsdWVudGlhbCBkYXRhIHBvaW50cyB3aXRoIGFicyguc3RkLnJlcykgPiAzOgpgYGB7ciBJNjhpfQptb2RlbC5kYXRhICU+JSAKICBmaWx0ZXIoYWJzKC5zdGQucmVzaWQpID4gMykKYGBgCgoKCiMjIENfR0VOREVSIEJpbmFyeSBsb2dpc3RpYyByZWdyZXNzaW9uIE9OIFNJR05JRklDQU5UIE9SIFNFUklPVVMgSUxMTkVTUwpgYGB7ciBJNjl9CiMgZml0IGJpbmFyeSBsb2dpdCBtb2RlbCBhbmQgc3RvcmUgcmVzdWx0cyAnbScKbSA8LSBnbG0oSGVhbHRoX0JpbmFyeSB+IENfR2VuZGVyLCBkYXRhID0gbWwsZmFtaWx5ID0gYmlub21pYWwpCiMgdmlldyBhIHN1bW1hcnkgb2YgdGhlIG1vZGVsCnN1bW1hcnkobSkKIyB0ZXN0IG1vZGVsIGZpdAp3aXRoKG0sIG51bGwuZGV2aWFuY2UgLSBkZXZpYW5jZSkKd2l0aChtLCBkZi5udWxsIC0gZGYucmVzaWR1YWwpCndpdGgobSwgcGNoaXNxKG51bGwuZGV2aWFuY2UgLSBkZXZpYW5jZSwgZGYubnVsbCAtIGRmLnJlc2lkdWFsLCBsb3dlci50YWlsID0gRkFMU0UpKQpCSUMobSkKIyMgQ0lzIHVzaW5nIHByb2ZpbGVkIGxvZy1saWtlbGlob29kCmNvbmZpbnQobSwgbGV2ZWw9MC45OSkKIyMgQ0lzIHVzaW5nIHN0YW5kYXJkIGVycm9ycwpjb25maW50LmRlZmF1bHQobSwgbGV2ZWw9MC45OSkKIyBXYWxkIHRlc3QKd2FsZC50ZXN0KGIgPSBjb2VmKG0pLCBTaWdtYSA9IHZjb3YobSksIFRlcm1zID0gMikKIyMgb2RkcyByYXRpb3MgYW5kIDk1JSBDSQpleHAoY2JpbmQoT1IgPSBjb2VmKG0pLCBjb25maW50KG0sIGxldmVsPTAuOTkpKSkKYGBgCgojIyMgQ3JlYXRlIFJPQ1IgZnJvbSBkYXRhCmBgYHtyIFJPQzE5fQojIyB0cmFpbmluZyBkYXRhCnByZWQubXR0ID0gcHJlZGljdChtLCB0eXBlID0gInJlc3BvbnNlIikgI3JlcGVhdCByaXNrIHByZWRpY3Rpb25zIGZyb20gbW9kZWwgbQpyb2NyLnByZWQubXR0ID0gUk9DUjo6cHJlZGljdGlvbihwcmVkLm10dCwgbGFiZWxzID0gbWwkSGVhbHRoX0JpbmFyeSkgI1JPQ1IgcHJlZGljdGlvbiBvYmplY3QKcm9jLnBlcmYubXR0ID0gUk9DUjo6cGVyZm9ybWFuY2Uocm9jci5wcmVkLm10dCwgbWVhc3VyZSA9ICJ0cHIiLCB4Lm1lYXN1cmUgPSAiZnByIikgIyAjUk9DUiBwZXJmb3JtYW5jZSBvYmplY3QKcGxvdChyb2MucGVyZi5tdHQsIGNvbCA9ICJibHVlIikKYWJsaW5lKGEgPSAwLCBiID0gMSwgbHR5ID0gMikgI2RpYWdvbmFsIGZvciByYW5kb20gYXNzaWdubWVudApgYGAKCiMjIyBSZXBvcnQgQVVDIGZyb20gUk9DIGZvciB0cmFpbmluZyBhbmQgdGVzdCBkYXRhCmBgYHtyIEFVQzE5fQogICMgVHJhaW4gQVVDCmF1YyA8LSBST0NSOjpwZXJmb3JtYW5jZShyb2NyLnByZWQubXR0LCBtZWFzdXJlID0gImF1YyIpCiAgYXVjIDwtIGF1Y0B5LnZhbHVlc1tbMV1dCiAgcHJpbnQoYXVjKQpgYGAKCgojIyMgQ2FsY3VsYXRlIE5hZ2Vsa2Vya2UgUl4yCmBgYHtyIEk2OWF9Ck5hZ2Vsa2Vya2VSMihtKQpgYGAKCiMjIyBjaGVjayBhc3N1bXB0aW9ucyBvZiBtb2RlbAojIyMjIENvb2sncyBkaXN0YW5jZQpgYGB7ciBJNzB9CnBsb3QobSwgd2hpY2ggPSA0LCBpZC5uID0gMykKYGBgCgojIyMjIEV4dHJhY3QgbW9kZWwgcmVzdWx0cyBhbmQgZGlzcGxheSBkYXRhIGZvciB0b3AgMyB2YWx1ZXMgdXNpbmcgQ29vaydzIGRpc3RhbmNlCmBgYHtyIEk3MX0KbW9kZWwuZGF0YSA8LSBhdWdtZW50KG0pICU+JSAKICBtdXRhdGUoaW5kZXggPSAxOm4oKSkgCm1vZGVsLmRhdGEgJT4lIHRvcF9uKDMsIC5jb29rc2QpCmBgYAoKIyMjIyBwbG90IHN0YW5kYXJkaXNlZCByZXNpZHVhbHMKYGBge3IgSTcyfQpnZ3Bsb3QobW9kZWwuZGF0YSwgYWVzKGluZGV4LCAuc3RkLnJlc2lkKSkgKyAKICBnZW9tX3BvaW50KGFlcyhjb2xvciA9IEhlYWx0aF9CaW5hcnkpLCBhbHBoYSA9IC41KSArCiAgdGhlbWVfYncoKQpgYGAKCiMjIyMgRmlsdGVyIHBvdGVudGlhbCBpbmZsdWVudGlhbCBkYXRhIHBvaW50cyB3aXRoIGFicyguc3RkLnJlcykgPiAzOgpgYGB7ciBJNzN9Cm1vZGVsLmRhdGEgJT4lIAogIGZpbHRlcihhYnMoLnN0ZC5yZXNpZCkgPiAzKQpgYGAKCgoKCiMgTk9XIENIRUNLIEFTU09DSUFUSU9OUyBCRVRXRUVOIFNJR05JRklDQU5UIE9SIFNFUklPVVMgSUxMTkVTUyBBTkQgRE9HIENIQVJBQ1RFUklTVElDUyAtIHNpbXBsZSBCSU5BUlkgTE9HSVNUSUMgcmVncmVzc2lvbgoKCiMjIERPRyBESUVUIFZFR0FOIGJpbmFyeSByZWdyZXNzaW9uIGZvciBPTiBTSUdOSUZJQ0FOVCBPUiBTRVJJT1VTIElMTE5FU1MKYGBge3IgMTR0fQojIGZpdCBiaW5hcnkgbG9naXQgbW9kZWwgYW5kIHN0b3JlIHJlc3VsdHMgJ20nCm0gPC0gZ2xtKEhlYWx0aF9CaW5hcnkgfiBEX0RpZXRfVmVnYW4sIGRhdGEgPSBtbCxmYW1pbHkgPSBiaW5vbWlhbCkKIyB2aWV3IGEgc3VtbWFyeSBvZiB0aGUgbW9kZWwKc3VtbWFyeShtKQojIHRlc3QgbW9kZWwgZml0CndpdGgobSwgbnVsbC5kZXZpYW5jZSAtIGRldmlhbmNlKQp3aXRoKG0sIGRmLm51bGwgLSBkZi5yZXNpZHVhbCkKd2l0aChtLCBwY2hpc3EobnVsbC5kZXZpYW5jZSAtIGRldmlhbmNlLCBkZi5udWxsIC0gZGYucmVzaWR1YWwsIGxvd2VyLnRhaWwgPSBGQUxTRSkpCkJJQyhtKQojIyBDSXMgdXNpbmcgcHJvZmlsZWQgbG9nLWxpa2VsaWhvb2QKY29uZmludChtLCBsZXZlbD0wLjk5KQojIyBDSXMgdXNpbmcgc3RhbmRhcmQgZXJyb3JzCmNvbmZpbnQuZGVmYXVsdChtLCBsZXZlbD0wLjk5KQojIFdhbGQgdGVzdAp3YWxkLnRlc3QoYiA9IGNvZWYobSksIFNpZ21hID0gdmNvdihtKSwgVGVybXMgPSAyKQojIyBvZGRzIHJhdGlvcyBhbmQgOTUlIENJCmV4cChjYmluZChPUiA9IGNvZWYobSksIGNvbmZpbnQobSwgbGV2ZWw9MC45OSkpKQpgYGAKCiMjIyBDcmVhdGUgUk9DUiBmcm9tIGRhdGEKYGBge3IgUk9DMjB9CiMjIHRyYWluaW5nIGRhdGEKcHJlZC5tdHQgPSBwcmVkaWN0KG0sIHR5cGUgPSAicmVzcG9uc2UiKSAjcmVwZWF0IHJpc2sgcHJlZGljdGlvbnMgZnJvbSBtb2RlbCBtCnJvY3IucHJlZC5tdHQgPSBST0NSOjpwcmVkaWN0aW9uKHByZWQubXR0LCBsYWJlbHMgPSBtbCRIZWFsdGhfQmluYXJ5KSAjUk9DUiBwcmVkaWN0aW9uIG9iamVjdApyb2MucGVyZi5tdHQgPSBST0NSOjpwZXJmb3JtYW5jZShyb2NyLnByZWQubXR0LCBtZWFzdXJlID0gInRwciIsIHgubWVhc3VyZSA9ICJmcHIiKSAjICNST0NSIHBlcmZvcm1hbmNlIG9iamVjdApwbG90KHJvYy5wZXJmLm10dCwgY29sID0gImJsdWUiKQphYmxpbmUoYSA9IDAsIGIgPSAxLCBsdHkgPSAyKSAjZGlhZ29uYWwgZm9yIHJhbmRvbSBhc3NpZ25tZW50CmBgYAoKIyMjIFJlcG9ydCBBVUMgZnJvbSBST0MgZm9yIHRyYWluaW5nIGFuZCB0ZXN0IGRhdGEKYGBge3IgQVVDMjB9CiAgIyBUcmFpbiBBVUMKYXVjIDwtIFJPQ1I6OnBlcmZvcm1hbmNlKHJvY3IucHJlZC5tdHQsIG1lYXN1cmUgPSAiYXVjIikKICBhdWMgPC0gYXVjQHkudmFsdWVzW1sxXV0KICBwcmludChhdWMpCmBgYAoKCiMjIyBDYWxjdWxhdGUgTmFnZWxrZXJrZSBSXjIKYGBge3IgMTRhdH0KTmFnZWxrZXJrZVIyKG0pCmBgYAoKIyMjIGNoZWNrIGFzc3VtcHRpb25zIG9mIG1vZGVsCiMjIyMgQ29vaydzIGRpc3RhbmNlCmBgYHtyIDE1dH0KcGxvdChtLCB3aGljaCA9IDQsIGlkLm4gPSAzKQpgYGAKCiMjIyMgRXh0cmFjdCBtb2RlbCByZXN1bHRzIGFuZCBkaXNwbGF5IGRhdGEgZm9yIHRvcCAzIHZhbHVlcyB1c2luZyBDb29rJ3MgZGlzdGFuY2UKYGBge3IgMTZ0fQptb2RlbC5kYXRhIDwtIGF1Z21lbnQobSkgJT4lIAogIG11dGF0ZShpbmRleCA9IDE6bigpKSAKbW9kZWwuZGF0YSAlPiUgdG9wX24oMywgLmNvb2tzZCkKYGBgCgojIyMjIHBsb3Qgc3RhbmRhcmRpc2VkIHJlc2lkdWFscwpgYGB7ciAxN3R9CmdncGxvdChtb2RlbC5kYXRhLCBhZXMoaW5kZXgsIC5zdGQucmVzaWQpKSArIAogIGdlb21fcG9pbnQoYWVzKGNvbG9yID0gSGVhbHRoX0JpbmFyeSksIGFscGhhID0gLjUpICsKICB0aGVtZV9idygpCmBgYAoKIyMjIyBGaWx0ZXIgcG90ZW50aWFsIGluZmx1ZW50aWFsIGRhdGEgcG9pbnRzIHdpdGggYWJzKC5zdGQucmVzKSA+IDM6CmBgYHtyIDE4dH0KbW9kZWwuZGF0YSAlPiUgCiAgZmlsdGVyKGFicyguc3RkLnJlc2lkKSA+IDMpCmBgYAoKCiMjIERPRyBESUVUIFZFR0FOX1ZFR0dJRSBiaW5hcnkgcmVncmVzc2lvbiBmb3IgT04gU0lHTklGSUNBTlQgT1IgU0VSSU9VUyBJTExORVNTCmBgYHtyIDE0dHh9CiMgZml0IGJpbmFyeSBsb2dpdCBtb2RlbCBhbmQgc3RvcmUgcmVzdWx0cyAnbScKbSA8LSBnbG0oSGVhbHRoX0JpbmFyeSB+IERfRGlldF9WZWdhbl9WZWdnaWUsIGRhdGEgPSBtbCxmYW1pbHkgPSBiaW5vbWlhbCkKIyB2aWV3IGEgc3VtbWFyeSBvZiB0aGUgbW9kZWwKc3VtbWFyeShtKQojIHRlc3QgbW9kZWwgZml0CndpdGgobSwgbnVsbC5kZXZpYW5jZSAtIGRldmlhbmNlKQp3aXRoKG0sIGRmLm51bGwgLSBkZi5yZXNpZHVhbCkKd2l0aChtLCBwY2hpc3EobnVsbC5kZXZpYW5jZSAtIGRldmlhbmNlLCBkZi5udWxsIC0gZGYucmVzaWR1YWwsIGxvd2VyLnRhaWwgPSBGQUxTRSkpCkJJQyhtKQojIyBDSXMgdXNpbmcgcHJvZmlsZWQgbG9nLWxpa2VsaWhvb2QKY29uZmludChtLCBsZXZlbD0wLjk5KQojIyBDSXMgdXNpbmcgc3RhbmRhcmQgZXJyb3JzCmNvbmZpbnQuZGVmYXVsdChtLCBsZXZlbD0wLjk5KQojIFdhbGQgdGVzdAp3YWxkLnRlc3QoYiA9IGNvZWYobSksIFNpZ21hID0gdmNvdihtKSwgVGVybXMgPSAyKQojIyBvZGRzIHJhdGlvcyBhbmQgOTUlIENJCmV4cChjYmluZChPUiA9IGNvZWYobSksIGNvbmZpbnQobSwgbGV2ZWw9MC45OSkpKQpgYGAKCiMjIyBDcmVhdGUgUk9DUiBmcm9tIGRhdGEKYGBge3IgUk9DMjF9CiMjIHRyYWluaW5nIGRhdGEKcHJlZC5tdHQgPSBwcmVkaWN0KG0sIHR5cGUgPSAicmVzcG9uc2UiKSAjcmVwZWF0IHJpc2sgcHJlZGljdGlvbnMgZnJvbSBtb2RlbCBtCnJvY3IucHJlZC5tdHQgPSBST0NSOjpwcmVkaWN0aW9uKHByZWQubXR0LCBsYWJlbHMgPSBtbCRIZWFsdGhfQmluYXJ5KSAjUk9DUiBwcmVkaWN0aW9uIG9iamVjdApyb2MucGVyZi5tdHQgPSBST0NSOjpwZXJmb3JtYW5jZShyb2NyLnByZWQubXR0LCBtZWFzdXJlID0gInRwciIsIHgubWVhc3VyZSA9ICJmcHIiKSAjICNST0NSIHBlcmZvcm1hbmNlIG9iamVjdApwbG90KHJvYy5wZXJmLm10dCwgY29sID0gImJsdWUiKQphYmxpbmUoYSA9IDAsIGIgPSAxLCBsdHkgPSAyKSAjZGlhZ29uYWwgZm9yIHJhbmRvbSBhc3NpZ25tZW50CmBgYAoKIyMjIFJlcG9ydCBBVUMgZnJvbSBST0MgZm9yIHRyYWluaW5nIGFuZCB0ZXN0IGRhdGEKYGBge3IgQVVDMjF9CiAgIyBUcmFpbiBBVUMKYXVjIDwtIFJPQ1I6OnBlcmZvcm1hbmNlKHJvY3IucHJlZC5tdHQsIG1lYXN1cmUgPSAiYXVjIikKICBhdWMgPC0gYXVjQHkudmFsdWVzW1sxXV0KICBwcmludChhdWMpCmBgYAoKCiMjIyBDYWxjdWxhdGUgTmFnZWxrZXJrZSBSXjIKYGBge3IgMTRhdHh9Ck5hZ2Vsa2Vya2VSMihtKQpgYGAKCiMjIyBjaGVjayBhc3N1bXB0aW9ucyBvZiBtb2RlbAojIyMjIENvb2sncyBkaXN0YW5jZQpgYGB7ciAxNXR4fQpwbG90KG0sIHdoaWNoID0gNCwgaWQubiA9IDMpCmBgYAoKIyMjIyBFeHRyYWN0IG1vZGVsIHJlc3VsdHMgYW5kIGRpc3BsYXkgZGF0YSBmb3IgdG9wIDMgdmFsdWVzIHVzaW5nIENvb2sncyBkaXN0YW5jZQpgYGB7ciAxNnR4fQptb2RlbC5kYXRhIDwtIGF1Z21lbnQobSkgJT4lIAogIG11dGF0ZShpbmRleCA9IDE6bigpKSAKbW9kZWwuZGF0YSAlPiUgdG9wX24oMywgLmNvb2tzZCkKYGBgCgojIyMjIHBsb3Qgc3RhbmRhcmRpc2VkIHJlc2lkdWFscwpgYGB7ciAxN3R4fQpnZ3Bsb3QobW9kZWwuZGF0YSwgYWVzKGluZGV4LCAuc3RkLnJlc2lkKSkgKyAKICBnZW9tX3BvaW50KGFlcyhjb2xvciA9IEhlYWx0aF9CaW5hcnkpLCBhbHBoYSA9IC41KSArCiAgdGhlbWVfYncoKQpgYGAKCiMjIyMgRmlsdGVyIHBvdGVudGlhbCBpbmZsdWVudGlhbCBkYXRhIHBvaW50cyB3aXRoIGFicyguc3RkLnJlcykgPiAzOgpgYGB7ciAxOHR4fQptb2RlbC5kYXRhICU+JSAKICBmaWx0ZXIoYWJzKC5zdGQucmVzaWQpID4gMykKYGBgCgoKCgojIyBET0cgRElFVCBiaW5hcnkgcmVncmVzc2lvbiBPTiBTSUdOSUZJQ0FOVCBPUiBTRVJJT1VTIElMTE5FU1MKYGBge3IgMTRpdH0KIyBmaXQgYmluYXJ5IGxvZ2l0IG1vZGVsIGFuZCBzdG9yZSByZXN1bHRzICdtJwptIDwtIGdsbShIZWFsdGhfQmluYXJ5IH4gRF9EaWV0LCBkYXRhID0gbWwsZmFtaWx5ID0gYmlub21pYWwpCiMgdmlldyBhIHN1bW1hcnkgb2YgdGhlIG1vZGVsCnN1bW1hcnkobSkKIyB0ZXN0IG1vZGVsIGZpdAp3aXRoKG0sIG51bGwuZGV2aWFuY2UgLSBkZXZpYW5jZSkKd2l0aChtLCBkZi5udWxsIC0gZGYucmVzaWR1YWwpCndpdGgobSwgcGNoaXNxKG51bGwuZGV2aWFuY2UgLSBkZXZpYW5jZSwgZGYubnVsbCAtIGRmLnJlc2lkdWFsLCBsb3dlci50YWlsID0gRkFMU0UpKQpCSUMobSkKIyMgQ0lzIHVzaW5nIHByb2ZpbGVkIGxvZy1saWtlbGlob29kCmNvbmZpbnQobSwgbGV2ZWw9MC45OSkKIyMgQ0lzIHVzaW5nIHN0YW5kYXJkIGVycm9ycwpjb25maW50LmRlZmF1bHQobSwgbGV2ZWw9MC45OSkKIyBXYWxkIHRlc3QKd2FsZC50ZXN0KGIgPSBjb2VmKG0pLCBTaWdtYSA9IHZjb3YobSksIFRlcm1zID0gMikKIyMgb2RkcyByYXRpb3MgYW5kIDk1JSBDSQpleHAoY2JpbmQoT1IgPSBjb2VmKG0pLCBjb25maW50KG0sIGxldmVsPTAuOTkpKSkKYGBgCgojIyMgQ3JlYXRlIFJPQ1IgZnJvbSBkYXRhCmBgYHtyIFJPQzIyfQojIyB0cmFpbmluZyBkYXRhCnByZWQubXR0ID0gcHJlZGljdChtLCB0eXBlID0gInJlc3BvbnNlIikgI3JlcGVhdCByaXNrIHByZWRpY3Rpb25zIGZyb20gbW9kZWwgbQpyb2NyLnByZWQubXR0ID0gUk9DUjo6cHJlZGljdGlvbihwcmVkLm10dCwgbGFiZWxzID0gbWwkSGVhbHRoX0JpbmFyeSkgI1JPQ1IgcHJlZGljdGlvbiBvYmplY3QKcm9jLnBlcmYubXR0ID0gUk9DUjo6cGVyZm9ybWFuY2Uocm9jci5wcmVkLm10dCwgbWVhc3VyZSA9ICJ0cHIiLCB4Lm1lYXN1cmUgPSAiZnByIikgIyAjUk9DUiBwZXJmb3JtYW5jZSBvYmplY3QKcGxvdChyb2MucGVyZi5tdHQsIGNvbCA9ICJibHVlIikKYWJsaW5lKGEgPSAwLCBiID0gMSwgbHR5ID0gMikgI2RpYWdvbmFsIGZvciByYW5kb20gYXNzaWdubWVudApgYGAKCiMjIyBSZXBvcnQgQVVDIGZyb20gUk9DIGZvciB0cmFpbmluZyBhbmQgdGVzdCBkYXRhCmBgYHtyIEFVQzIyfQogICMgVHJhaW4gQVVDCmF1YyA8LSBST0NSOjpwZXJmb3JtYW5jZShyb2NyLnByZWQubXR0LCBtZWFzdXJlID0gImF1YyIpCiAgYXVjIDwtIGF1Y0B5LnZhbHVlc1tbMV1dCiAgcHJpbnQoYXVjKQpgYGAKCgojIyMgQ2FsY3VsYXRlIE5hZ2Vsa2Vya2UgUl4yCmBgYHtyIDE0YXRpfQpOYWdlbGtlcmtlUjIobSkKYGBgCgojIyMgY2hlY2sgYXNzdW1wdGlvbnMgb2YgbW9kZWwKIyMjIyBDb29rJ3MgZGlzdGFuY2UKYGBge3IgMTV0aX0KcGxvdChtLCB3aGljaCA9IDQsIGlkLm4gPSAzKQpgYGAKCiMjIyMgRXh0cmFjdCBtb2RlbCByZXN1bHRzIGFuZCBkaXNwbGF5IGRhdGEgZm9yIHRvcCAzIHZhbHVlcyB1c2luZyBDb29rJ3MgZGlzdGFuY2UKYGBge3IgMTZ0aX0KbW9kZWwuZGF0YSA8LSBhdWdtZW50KG0pICU+JSAKICBtdXRhdGUoaW5kZXggPSAxOm4oKSkgCm1vZGVsLmRhdGEgJT4lIHRvcF9uKDMsIC5jb29rc2QpCmBgYAoKIyMjIyBwbG90IHN0YW5kYXJkaXNlZCByZXNpZHVhbHMKYGBge3IgMTd0aX0KZ2dwbG90KG1vZGVsLmRhdGEsIGFlcyhpbmRleCwgLnN0ZC5yZXNpZCkpICsgCiAgZ2VvbV9wb2ludChhZXMoY29sb3IgPSBIZWFsdGhfQmluYXJ5KSwgYWxwaGEgPSAuNSkgKwogIHRoZW1lX2J3KCkKYGBgCgojIyMjIEZpbHRlciBwb3RlbnRpYWwgaW5mbHVlbnRpYWwgZGF0YSBwb2ludHMgd2l0aCBhYnMoLnN0ZC5yZXMpID4gMzoKYGBge3IgMTh0aX0KbW9kZWwuZGF0YSAlPiUgCiAgZmlsdGVyKGFicyguc3RkLnJlc2lkKSA+IDMpCmBgYAoKCgojIyBDTElFTlQgRElFVCBWRUdBTiArIERPRyBESUVUIFZFR0FOIGJpbmFyeWwgcmVncmVzc2lvbiBPTiBTSUdOSUZJQ0FOVCBPUiBTRVJJT1VTIElMTE5FU1MKYGBge3IgMjVzcX0KIyBmaXQgYmluYXJ5IGxvZ2l0IG1vZGVsIGFuZCBzdG9yZSByZXN1bHRzICdtMicKbSA8LSBnbG0oSGVhbHRoX0JpbmFyeSB+IENfRGlldF9WZWdhbiArIERfRGlldF9WZWdhbiwgZGF0YSA9IG1sLGZhbWlseSA9IGJpbm9taWFsKQojIHZpZXcgYSBzdW1tYXJ5IG9mIHRoZSBtb2RlbApzdW1tYXJ5KG0pCiMgdGVzdCBtb2RlbCBmaXQKd2l0aChtLCBudWxsLmRldmlhbmNlIC0gZGV2aWFuY2UpCndpdGgobSwgZGYubnVsbCAtIGRmLnJlc2lkdWFsKQp3aXRoKG0sIHBjaGlzcShudWxsLmRldmlhbmNlIC0gZGV2aWFuY2UsIGRmLm51bGwgLSBkZi5yZXNpZHVhbCwgbG93ZXIudGFpbCA9IEZBTFNFKSkKQklDKG0pCiMgSG9zbWVyLUxlbWVzaG93IEdvb2RuZXNzLW9mLUZpdCBUZXN0CmdsbXRvb2xib3g6OmhsdGVzdChtKQojIyBDSXMgdXNpbmcgcHJvZmlsZWQgbG9nLWxpa2VsaWhvb2QKY29uZmludChtLCBsZXZlbD0wLjk5KQojIyBDSXMgdXNpbmcgc3RhbmRhcmQgZXJyb3JzCmNvbmZpbnQuZGVmYXVsdChtLCBsZXZlbD0wLjk5KQojIFdhbGQgdGVzdAp3YWxkLnRlc3QoYiA9IGNvZWYobSksIFNpZ21hID0gdmNvdihtKSwgVGVybXMgPSAyKQojIyBvZGRzIHJhdGlvcyBhbmQgOTUlIENJCmV4cChjYmluZChPUiA9IGNvZWYobSksIGNvbmZpbnQobSwgbGV2ZWw9MC45OSkpKQpgYGAKCiMjIyBDcmVhdGUgUk9DUiBmcm9tIGRhdGEKYGBge3IgUk9DMjN9CiMjIHRyYWluaW5nIGRhdGEKcHJlZC5tdHQgPSBwcmVkaWN0KG0sIHR5cGUgPSAicmVzcG9uc2UiKSAjcmVwZWF0IHJpc2sgcHJlZGljdGlvbnMgZnJvbSBtb2RlbCBtCnJvY3IucHJlZC5tdHQgPSBST0NSOjpwcmVkaWN0aW9uKHByZWQubXR0LCBsYWJlbHMgPSBtbCRIZWFsdGhfQmluYXJ5KSAjUk9DUiBwcmVkaWN0aW9uIG9iamVjdApyb2MucGVyZi5tdHQgPSBST0NSOjpwZXJmb3JtYW5jZShyb2NyLnByZWQubXR0LCBtZWFzdXJlID0gInRwciIsIHgubWVhc3VyZSA9ICJmcHIiKSAjICNST0NSIHBlcmZvcm1hbmNlIG9iamVjdApwbG90KHJvYy5wZXJmLm10dCwgY29sID0gImJsdWUiKQphYmxpbmUoYSA9IDAsIGIgPSAxLCBsdHkgPSAyKSAjZGlhZ29uYWwgZm9yIHJhbmRvbSBhc3NpZ25tZW50CmBgYAoKIyMjIFJlcG9ydCBBVUMgZnJvbSBST0MgZm9yIHRyYWluaW5nIGFuZCB0ZXN0IGRhdGEKYGBge3IgQVVDMjN9CiAgIyBUcmFpbiBBVUMKYXVjIDwtIFJPQ1I6OnBlcmZvcm1hbmNlKHJvY3IucHJlZC5tdHQsIG1lYXN1cmUgPSAiYXVjIikKICBhdWMgPC0gYXVjQHkudmFsdWVzW1sxXV0KICBwcmludChhdWMpCmBgYAoKCiMjIyBDYWxjdWxhdGUgTmFnZWxrZXJrZSBSXjIKYGBge3IgMjVhc3F9Ck5hZ2Vsa2Vya2VSMihtKQpgYGAKCiMjIyBjaGVjayBhc3N1bXB0aW9ucyBvZiBtb2RlbAojIyMjIENvb2sncyBkaXN0YW5jZQpgYGB7ciAyNnNxfQpwbG90KG0sIHdoaWNoID0gNCwgaWQubiA9IDMpCmBgYAoKIyMjIyBFeHRyYWN0IG1vZGVsIHJlc3VsdHMgYW5kIGRpc3BsYXkgZGF0YSBmb3IgdG9wIDMgdmFsdWVzIHVzaW5nIENvb2sncyBkaXN0YW5jZQpgYGB7ciAyN3NxfQptb2RlbC5kYXRhIDwtIGF1Z21lbnQobSkgJT4lIAogIG11dGF0ZShpbmRleCA9IDE6bigpKSAKbW9kZWwuZGF0YSAlPiUgdG9wX24oMywgLmNvb2tzZCkKYGBgCgojIyMjIHBsb3Qgc3RhbmRhcmRpc2VkIHJlc2lkdWFscwpgYGB7ciAyOHNxfQpnZ3Bsb3QobW9kZWwuZGF0YSwgYWVzKGluZGV4LCAuc3RkLnJlc2lkKSkgKyAKICBnZW9tX3BvaW50KGFlcyhjb2xvciA9IEhlYWx0aF9CaW5hcnkpLCBhbHBoYSA9IC41KSArCiAgdGhlbWVfYncoKQpgYGAKCiMjIyMgRmlsdGVyIHBvdGVudGlhbCBpbmZsdWVudGlhbCBkYXRhIHBvaW50cyB3aXRoIGFicyguc3RkLnJlcykgPiAzOgpgYGB7ciAyOXNxfQptb2RlbC5kYXRhICU+JSAKICBmaWx0ZXIoYWJzKC5zdGQucmVzaWQpID4gMykKYGBgCgojIyMjIGNoZWNrIGZvciBtdWx0aWNvbGxpbmVhcml0eQpgYGB7ciAzMHNxfQpjYXI6OnZpZihtKQpgYGAKCgoKIyMgQ0xJRU5UIERJRVQgVkVHQU4gKiBET0cgRElFVCBWRUdBTiBiaW5hcnlsIHJlZ3Jlc3Npb24gT04gU0lHTklGSUNBTlQgT1IgU0VSSU9VUyBJTExORVNTCmBgYHtyIDI1c30KIyBmaXQgYmluYXJ5IGxvZ2l0IG1vZGVsIGFuZCBzdG9yZSByZXN1bHRzICdtMicKbSA8LSBnbG0oSGVhbHRoX0JpbmFyeSB+IENfRGlldF9WZWdhbipEX0RpZXRfVmVnYW4sIGRhdGEgPSBtbCxmYW1pbHkgPSBiaW5vbWlhbCkKIyB2aWV3IGEgc3VtbWFyeSBvZiB0aGUgbW9kZWwKc3VtbWFyeShtKQojIHRlc3QgbW9kZWwgZml0CndpdGgobSwgbnVsbC5kZXZpYW5jZSAtIGRldmlhbmNlKQp3aXRoKG0sIGRmLm51bGwgLSBkZi5yZXNpZHVhbCkKd2l0aChtLCBwY2hpc3EobnVsbC5kZXZpYW5jZSAtIGRldmlhbmNlLCBkZi5udWxsIC0gZGYucmVzaWR1YWwsIGxvd2VyLnRhaWwgPSBGQUxTRSkpCkJJQyhtKQojIEhvc21lci1MZW1lc2hvdyBHb29kbmVzcy1vZi1GaXQgVGVzdApnbG10b29sYm94OjpobHRlc3QobSkKIyMgQ0lzIHVzaW5nIHByb2ZpbGVkIGxvZy1saWtlbGlob29kCmNvbmZpbnQobSwgbGV2ZWw9MC45OSkKIyMgQ0lzIHVzaW5nIHN0YW5kYXJkIGVycm9ycwpjb25maW50LmRlZmF1bHQobSwgbGV2ZWw9MC45OSkKIyBXYWxkIHRlc3QKd2FsZC50ZXN0KGIgPSBjb2VmKG0pLCBTaWdtYSA9IHZjb3YobSksIFRlcm1zID0gMikKIyMgb2RkcyByYXRpb3MgYW5kIDk1JSBDSQpleHAoY2JpbmQoT1IgPSBjb2VmKG0pLCBjb25maW50KG0sIGxldmVsPTAuOTkpKSkKYGBgCgojIyMgQ3JlYXRlIFJPQ1IgZnJvbSBkYXRhCmBgYHtyIFJPQzI0fQojIyB0cmFpbmluZyBkYXRhCnByZWQubXR0ID0gcHJlZGljdChtLCB0eXBlID0gInJlc3BvbnNlIikgI3JlcGVhdCByaXNrIHByZWRpY3Rpb25zIGZyb20gbW9kZWwgbQpyb2NyLnByZWQubXR0ID0gUk9DUjo6cHJlZGljdGlvbihwcmVkLm10dCwgbGFiZWxzID0gbWwkSGVhbHRoX0JpbmFyeSkgI1JPQ1IgcHJlZGljdGlvbiBvYmplY3QKcm9jLnBlcmYubXR0ID0gUk9DUjo6cGVyZm9ybWFuY2Uocm9jci5wcmVkLm10dCwgbWVhc3VyZSA9ICJ0cHIiLCB4Lm1lYXN1cmUgPSAiZnByIikgIyAjUk9DUiBwZXJmb3JtYW5jZSBvYmplY3QKcGxvdChyb2MucGVyZi5tdHQsIGNvbCA9ICJibHVlIikKYWJsaW5lKGEgPSAwLCBiID0gMSwgbHR5ID0gMikgI2RpYWdvbmFsIGZvciByYW5kb20gYXNzaWdubWVudApgYGAKCiMjIyBSZXBvcnQgQVVDIGZyb20gUk9DIGZvciB0cmFpbmluZyBhbmQgdGVzdCBkYXRhCmBgYHtyIEFVQzI0fQogICMgVHJhaW4gQVVDCmF1YyA8LSBST0NSOjpwZXJmb3JtYW5jZShyb2NyLnByZWQubXR0LCBtZWFzdXJlID0gImF1YyIpCiAgYXVjIDwtIGF1Y0B5LnZhbHVlc1tbMV1dCiAgcHJpbnQoYXVjKQpgYGAKCgojIyMgQ2FsY3VsYXRlIE5hZ2Vsa2Vya2UgUl4yCmBgYHtyIDI1YXN9Ck5hZ2Vsa2Vya2VSMihtKQpgYGAKCiMjIyBjaGVjayBhc3N1bXB0aW9ucyBvZiBtb2RlbAojIyMjIENvb2sncyBkaXN0YW5jZQpgYGB7ciAyNnN9CnBsb3QobSwgd2hpY2ggPSA0LCBpZC5uID0gMykKYGBgCgojIyMjIEV4dHJhY3QgbW9kZWwgcmVzdWx0cyBhbmQgZGlzcGxheSBkYXRhIGZvciB0b3AgMyB2YWx1ZXMgdXNpbmcgQ29vaydzIGRpc3RhbmNlCmBgYHtyIDI3c30KbW9kZWwuZGF0YSA8LSBhdWdtZW50KG0pICU+JSAKICBtdXRhdGUoaW5kZXggPSAxOm4oKSkgCm1vZGVsLmRhdGEgJT4lIHRvcF9uKDMsIC5jb29rc2QpCmBgYAoKIyMjIyBwbG90IHN0YW5kYXJkaXNlZCByZXNpZHVhbHMKYGBge3IgMjhzfQpnZ3Bsb3QobW9kZWwuZGF0YSwgYWVzKGluZGV4LCAuc3RkLnJlc2lkKSkgKyAKICBnZW9tX3BvaW50KGFlcyhjb2xvciA9IEhlYWx0aF9CaW5hcnkpLCBhbHBoYSA9IC41KSArCiAgdGhlbWVfYncoKQpgYGAKCiMjIyMgRmlsdGVyIHBvdGVudGlhbCBpbmZsdWVudGlhbCBkYXRhIHBvaW50cyB3aXRoIGFicyguc3RkLnJlcykgPiAzOgpgYGB7ciAyOXN9Cm1vZGVsLmRhdGEgJT4lIAogIGZpbHRlcihhYnMoLnN0ZC5yZXNpZCkgPiAzKQpgYGAKCiMjIyMgY2hlY2sgZm9yIG11bHRpY29sbGluZWFyaXR5CmBgYHtyIDMwc30KY2FyOjp2aWYobSkKYGBgCgoKCiMjIERPRyBESUVUIFJBVyBiaW5hcnkgcmVncmVzc2lvbiBPTiBTSUdOSUZJQ0FOVCBPUiBTRVJJT1VTIElMTE5FU1MKYGBge3IgMzJkfQojIGZpdCBiaW5hcnkgbG9naXQgbW9kZWwgYW5kIHN0b3JlIHJlc3VsdHMgJ20nCm0gPC0gZ2xtKEhlYWx0aF9CaW5hcnkgfiBEX0RpZXRfUmF3LCBkYXRhID0gbWwsZmFtaWx5ID0gYmlub21pYWwpCiMgdmlldyBhIHN1bW1hcnkgb2YgdGhlIG1vZGVsCnN1bW1hcnkobSkKIyB0ZXN0IG1vZGVsIGZpdAp3aXRoKG0sIG51bGwuZGV2aWFuY2UgLSBkZXZpYW5jZSkKd2l0aChtLCBkZi5udWxsIC0gZGYucmVzaWR1YWwpCndpdGgobSwgcGNoaXNxKG51bGwuZGV2aWFuY2UgLSBkZXZpYW5jZSwgZGYubnVsbCAtIGRmLnJlc2lkdWFsLCBsb3dlci50YWlsID0gRkFMU0UpKQpCSUMobSkKIyMgQ0lzIHVzaW5nIHByb2ZpbGVkIGxvZy1saWtlbGlob29kCmNvbmZpbnQobSwgbGV2ZWw9MC45OSkKIyMgQ0lzIHVzaW5nIHN0YW5kYXJkIGVycm9ycwpjb25maW50LmRlZmF1bHQobSwgbGV2ZWw9MC45OSkKIyBXYWxkIHRlc3QKd2FsZC50ZXN0KGIgPSBjb2VmKG0pLCBTaWdtYSA9IHZjb3YobSksIFRlcm1zID0gMikKIyMgb2RkcyByYXRpb3MgYW5kIDk1JSBDSQpleHAoY2JpbmQoT1IgPSBjb2VmKG0pLCBjb25maW50KG0sIGxldmVsPTAuOTkpKSkKYGBgCgojIyMgQ3JlYXRlIFJPQ1IgZnJvbSBkYXRhCmBgYHtyIFJPQzI1fQojIyB0cmFpbmluZyBkYXRhCnByZWQubXR0ID0gcHJlZGljdChtLCB0eXBlID0gInJlc3BvbnNlIikgI3JlcGVhdCByaXNrIHByZWRpY3Rpb25zIGZyb20gbW9kZWwgbQpyb2NyLnByZWQubXR0ID0gUk9DUjo6cHJlZGljdGlvbihwcmVkLm10dCwgbGFiZWxzID0gbWwkSGVhbHRoX0JpbmFyeSkgI1JPQ1IgcHJlZGljdGlvbiBvYmplY3QKcm9jLnBlcmYubXR0ID0gUk9DUjo6cGVyZm9ybWFuY2Uocm9jci5wcmVkLm10dCwgbWVhc3VyZSA9ICJ0cHIiLCB4Lm1lYXN1cmUgPSAiZnByIikgIyAjUk9DUiBwZXJmb3JtYW5jZSBvYmplY3QKcGxvdChyb2MucGVyZi5tdHQsIGNvbCA9ICJibHVlIikKYWJsaW5lKGEgPSAwLCBiID0gMSwgbHR5ID0gMikgI2RpYWdvbmFsIGZvciByYW5kb20gYXNzaWdubWVudApgYGAKCiMjIyBSZXBvcnQgQVVDIGZyb20gUk9DIGZvciB0cmFpbmluZyBhbmQgdGVzdCBkYXRhCmBgYHtyIEFVQzI1fQogICMgVHJhaW4gQVVDCmF1YyA8LSBST0NSOjpwZXJmb3JtYW5jZShyb2NyLnByZWQubXR0LCBtZWFzdXJlID0gImF1YyIpCiAgYXVjIDwtIGF1Y0B5LnZhbHVlc1tbMV1dCiAgcHJpbnQoYXVjKQpgYGAKCgojIyMgQ2FsY3VsYXRlIE5hZ2Vsa2Vya2UgUl4yCmBgYHtyIDMyYWR9Ck5hZ2Vsa2Vya2VSMihtKQpgYGAKCiMjIyBjaGVjayBhc3N1bXB0aW9ucyBvZiBtb2RlbAojIyMjIENvb2sncyBkaXN0YW5jZQpgYGB7ciAzM2R9CnBsb3QobSwgd2hpY2ggPSA0LCBpZC5uID0gMykKYGBgCgojIyMjIEV4dHJhY3QgbW9kZWwgcmVzdWx0cyBhbmQgZGlzcGxheSBkYXRhIGZvciB0b3AgMyB2YWx1ZXMgdXNpbmcgQ29vaydzIGRpc3RhbmNlCmBgYHtyIDM0ZH0KbW9kZWwuZGF0YSA8LSBhdWdtZW50KG0pICU+JSAKICBtdXRhdGUoaW5kZXggPSAxOm4oKSkgCm1vZGVsLmRhdGEgJT4lIHRvcF9uKDMsIC5jb29rc2QpCmBgYAoKIyBwbG90IHN0YW5kYXJkaXNlZCByZXNpZHVhbHMKYGBge3IgMzVkfQpnZ3Bsb3QobW9kZWwuZGF0YSwgYWVzKGluZGV4LCAuc3RkLnJlc2lkKSkgKyAKICBnZW9tX3BvaW50KGFlcyhjb2xvciA9IEhlYWx0aF9CaW5hcnkpLCBhbHBoYSA9IC41KSArCiAgdGhlbWVfYncoKQpgYGAKCiMjIyMgRmlsdGVyIHBvdGVudGlhbCBpbmZsdWVudGlhbCBkYXRhIHBvaW50cyB3aXRoIGFicyguc3RkLnJlcykgPiAzOgpgYGB7ciAzNmR9Cm1vZGVsLmRhdGEgJT4lIAogIGZpbHRlcihhYnMoLnN0ZC5yZXNpZCkgPiAzKQpgYGAKCgoKIyMgRE9HIERJRVQgKyBDTElFTlQgRElFVCBiaW5hcnkgbG9naXN0aWMgcmVncmVzc2lvbiBPTiBTSUdOSUZJQ0FOVCBPUiBTRVJJT1VTIElMTE5FU1MKYGBge3IgNzh9CiMgZml0IGJpbmFyeSBsb2dpdCBtb2RlbCBhbmQgc3RvcmUgcmVzdWx0cyAnbScKbSA8LSBnbG0oSGVhbHRoX0JpbmFyeSB+IERfRGlldCArIENfRGlldCAsIGRhdGEgPSBtbCxmYW1pbHkgPSBiaW5vbWlhbCkKIyB2aWV3IGEgc3VtbWFyeSBvZiB0aGUgbW9kZWwKc3VtbWFyeShtKQojIHRlc3QgbW9kZWwgZml0CndpdGgobSwgbnVsbC5kZXZpYW5jZSAtIGRldmlhbmNlKQp3aXRoKG0sIGRmLm51bGwgLSBkZi5yZXNpZHVhbCkKd2l0aChtLCBwY2hpc3EobnVsbC5kZXZpYW5jZSAtIGRldmlhbmNlLCBkZi5udWxsIC0gZGYucmVzaWR1YWwsIGxvd2VyLnRhaWwgPSBGQUxTRSkpCkJJQyhtKQojIEhvc21lci1MZW1lc2hvdyBHb29kbmVzcy1vZi1GaXQgVGVzdApobHRlc3QobSwgRz03KQojIyBDSXMgdXNpbmcgcHJvZmlsZWQgbG9nLWxpa2VsaWhvb2QKY29uZmludChtLCBsZXZlbD0wLjk5KQojIyBDSXMgdXNpbmcgc3RhbmRhcmQgZXJyb3JzCmNvbmZpbnQuZGVmYXVsdChtLCBsZXZlbD0wLjk5KQojIFdhbGQgdGVzdAp3YWxkLnRlc3QoYiA9IGNvZWYobSksIFNpZ21hID0gdmNvdihtKSwgVGVybXMgPSAyKQojIyBvZGRzIHJhdGlvcyBhbmQgOTUlIENJCmV4cChjYmluZChPUiA9IGNvZWYobSksIGNvbmZpbnQobSwgbGV2ZWw9MC45OSkpKQpgYGAKCiMjIyBDcmVhdGUgUk9DUiBmcm9tIGRhdGEKYGBge3IgUk9DMjZ9CiMjIHRyYWluaW5nIGRhdGEKcHJlZC5tdHQgPSBwcmVkaWN0KG0sIHR5cGUgPSAicmVzcG9uc2UiKSAjcmVwZWF0IHJpc2sgcHJlZGljdGlvbnMgZnJvbSBtb2RlbCBtCnJvY3IucHJlZC5tdHQgPSBST0NSOjpwcmVkaWN0aW9uKHByZWQubXR0LCBsYWJlbHMgPSBtbCRIZWFsdGhfQmluYXJ5KSAjUk9DUiBwcmVkaWN0aW9uIG9iamVjdApyb2MucGVyZi5tdHQgPSBST0NSOjpwZXJmb3JtYW5jZShyb2NyLnByZWQubXR0LCBtZWFzdXJlID0gInRwciIsIHgubWVhc3VyZSA9ICJmcHIiKSAjICNST0NSIHBlcmZvcm1hbmNlIG9iamVjdApwbG90KHJvYy5wZXJmLm10dCwgY29sID0gImJsdWUiKQphYmxpbmUoYSA9IDAsIGIgPSAxLCBsdHkgPSAyKSAjZGlhZ29uYWwgZm9yIHJhbmRvbSBhc3NpZ25tZW50CmBgYAoKIyMjIFJlcG9ydCBBVUMgZnJvbSBST0MgZm9yIHRyYWluaW5nIGFuZCB0ZXN0IGRhdGEKYGBge3IgQVVDMjZ9CiAgIyBUcmFpbiBBVUMKYXVjIDwtIFJPQ1I6OnBlcmZvcm1hbmNlKHJvY3IucHJlZC5tdHQsIG1lYXN1cmUgPSAiYXVjIikKICBhdWMgPC0gYXVjQHkudmFsdWVzW1sxXV0KICBwcmludChhdWMpCmBgYAoKCiMjIyBDYWxjdWxhdGUgTmFnZWxrZXJrZSBSXjIKYGBge3IgNzhhfQpOYWdlbGtlcmtlUjIobSkKYGBgCgojIyMgY2hlY2sgYXNzdW1wdGlvbnMgb2YgbW9kZWwKIyMjIyBDb29rJ3MgZGlzdGFuY2UKYGBge3IgNzl9CnBsb3QobSwgd2hpY2ggPSA0LCBpZC5uID0gMykKYGBgCgojIyMjIEV4dHJhY3QgbW9kZWwgcmVzdWx0cyBhbmQgZGlzcGxheSBkYXRhIGZvciB0b3AgMyB2YWx1ZXMgdXNpbmcgQ29vaydzIGRpc3RhbmNlCmBgYHtyIDgwfQptb2RlbC5kYXRhIDwtIGF1Z21lbnQobSkgJT4lIAogIG11dGF0ZShpbmRleCA9IDE6bigpKSAKbW9kZWwuZGF0YSAlPiUgdG9wX24oMywgLmNvb2tzZCkKYGBgCgojIyMjIHBsb3Qgc3RhbmRhcmRpc2VkIHJlc2lkdWFscwpgYGB7ciA4MX0KZ2dwbG90KG1vZGVsLmRhdGEsIGFlcyhpbmRleCwgLnN0ZC5yZXNpZCkpICsgCiAgZ2VvbV9wb2ludChhZXMoY29sb3IgPSBIZWFsdGhfQmluYXJ5KSwgYWxwaGEgPSAuNSkgKwogIHRoZW1lX2J3KCkKYGBgCgojIyMjIEZpbHRlciBwb3RlbnRpYWwgaW5mbHVlbnRpYWwgZGF0YSBwb2ludHMgd2l0aCBhYnMoLnN0ZC5yZXMpID4gMzoKYGBge3IgODJ9Cm1vZGVsLmRhdGEgJT4lIAogIGZpbHRlcihhYnMoLnN0ZC5yZXNpZCkgPiAzKQpgYGAKCiMjIyMgY2hlY2sgZm9yIG11bHRpY29sbGluZWFyaXR5CmBgYHtyIDg5fQpjYXI6OnZpZihtKQpgYGAKCgoKIyMgVEhFUkFQRVVUSUMgRElFVCBiaW5hcnkgbG9naXN0aWMgcmVncmVzc2lvbiBPTiBTSUdOSUZJQ0FOVCBPUiBTRVJJT1VTIElMTE5FU1MKYGBge3IgOTB9CiMgZml0IGJpbmFyeSBsb2dpdCBtb2RlbCBhbmQgc3RvcmUgcmVzdWx0cyAnbScKbSA8LSBnbG0oSGVhbHRoX0JpbmFyeSB+IFRoZXJhcGV1dGljX0Zvb2QsIGRhdGEgPSBtbCxmYW1pbHkgPSBiaW5vbWlhbCkKIyB2aWV3IGEgc3VtbWFyeSBvZiB0aGUgbW9kZWwKc3VtbWFyeShtKQojIHRlc3QgbW9kZWwgZml0CndpdGgobSwgbnVsbC5kZXZpYW5jZSAtIGRldmlhbmNlKQp3aXRoKG0sIGRmLm51bGwgLSBkZi5yZXNpZHVhbCkKd2l0aChtLCBwY2hpc3EobnVsbC5kZXZpYW5jZSAtIGRldmlhbmNlLCBkZi5udWxsIC0gZGYucmVzaWR1YWwsIGxvd2VyLnRhaWwgPSBGQUxTRSkpCkJJQyhtKQoKIyMgQ0lzIHVzaW5nIHByb2ZpbGVkIGxvZy1saWtlbGlob29kCmNvbmZpbnQobSwgbGV2ZWw9MC45OSkKIyMgQ0lzIHVzaW5nIHN0YW5kYXJkIGVycm9ycwpjb25maW50LmRlZmF1bHQobSwgbGV2ZWw9MC45OSkKIyBXYWxkIHRlc3QKd2FsZC50ZXN0KGIgPSBjb2VmKG0pLCBTaWdtYSA9IHZjb3YobSksIFRlcm1zID0gMikKIyMgb2RkcyByYXRpb3MgYW5kIDk1JSBDSQpleHAoY2JpbmQoT1IgPSBjb2VmKG0pLCBjb25maW50KG0sIGxldmVsPTAuOTkpKSkKYGBgCgojIyMgQ3JlYXRlIFJPQ1IgZnJvbSBkYXRhCmBgYHtyIFJPQzI3fQojIyB0cmFpbmluZyBkYXRhCnByZWQubXR0ID0gcHJlZGljdChtLCB0eXBlID0gInJlc3BvbnNlIikgI3JlcGVhdCByaXNrIHByZWRpY3Rpb25zIGZyb20gbW9kZWwgbQpyb2NyLnByZWQubXR0ID0gUk9DUjo6cHJlZGljdGlvbihwcmVkLm10dCwgbGFiZWxzID0gbWwkSGVhbHRoX0JpbmFyeSkgI1JPQ1IgcHJlZGljdGlvbiBvYmplY3QKcm9jLnBlcmYubXR0ID0gUk9DUjo6cGVyZm9ybWFuY2Uocm9jci5wcmVkLm10dCwgbWVhc3VyZSA9ICJ0cHIiLCB4Lm1lYXN1cmUgPSAiZnByIikgIyAjUk9DUiBwZXJmb3JtYW5jZSBvYmplY3QKcGxvdChyb2MucGVyZi5tdHQsIGNvbCA9ICJibHVlIikKYWJsaW5lKGEgPSAwLCBiID0gMSwgbHR5ID0gMikgI2RpYWdvbmFsIGZvciByYW5kb20gYXNzaWdubWVudApgYGAKCiMjIyBSZXBvcnQgQVVDIGZyb20gUk9DIGZvciB0cmFpbmluZyBhbmQgdGVzdCBkYXRhCmBgYHtyIEFVQzI3fQogICMgVHJhaW4gQVVDCmF1YyA8LSBST0NSOjpwZXJmb3JtYW5jZShyb2NyLnByZWQubXR0LCBtZWFzdXJlID0gImF1YyIpCiAgYXVjIDwtIGF1Y0B5LnZhbHVlc1tbMV1dCiAgcHJpbnQoYXVjKQpgYGAKCgojIyMgQ2FsY3VsYXRlIE5hZ2Vsa2Vya2UgUl4yCmBgYHtyIDkwYX0KTmFnZWxrZXJrZVIyKG0pCmBgYAoKIyMjIGNoZWNrIGFzc3VtcHRpb25zIG9mIG1vZGVsCiMjIyMgQ29vaydzIGRpc3RhbmNlCmBgYHtyIDkxfQpwbG90KG0sIHdoaWNoID0gNCwgaWQubiA9IDMpCmBgYAoKIyMjIyBFeHRyYWN0IG1vZGVsIHJlc3VsdHMgYW5kIGRpc3BsYXkgZGF0YSBmb3IgdG9wIDMgdmFsdWVzIHVzaW5nIENvb2sncyBkaXN0YW5jZQpgYGB7ciA5Mn0KbW9kZWwuZGF0YSA8LSBhdWdtZW50KG0pICU+JSAKICBtdXRhdGUoaW5kZXggPSAxOm4oKSkgCm1vZGVsLmRhdGEgJT4lIHRvcF9uKDMsIC5jb29rc2QpCmBgYAoKIyMjIyBwbG90IHN0YW5kYXJkaXNlZCByZXNpZHVhbHMKYGBge3IgOTN9CmdncGxvdChtb2RlbC5kYXRhLCBhZXMoaW5kZXgsIC5zdGQucmVzaWQpKSArIAogIGdlb21fcG9pbnQoYWVzKGNvbG9yID0gSGVhbHRoX0JpbmFyeSksIGFscGhhID0gLjUpICsKICB0aGVtZV9idygpCmBgYAoKIyMjIyBGaWx0ZXIgcG90ZW50aWFsIGluZmx1ZW50aWFsIGRhdGEgcG9pbnRzIHdpdGggYWJzKC5zdGQucmVzKSA+IDM6CmBgYHtyIDk0fQptb2RlbC5kYXRhICU+JSAKICBmaWx0ZXIoYWJzKC5zdGQucmVzaWQpID4gMykKYGBgCgoKCiMjIERPRyBESUVUICsgVEhFUkFQRVVUSUMgRElFVCBiaW5hcnkgbG9naXN0aWMgcmVncmVzc2lvbiBPTiBTSUdOSUZJQ0FOVCBPUiBTRVJJT1VTIElMTE5FU1MKYGBge3IgOTV9CiMgZml0IGJpbmFyeSBsb2dpdCBtb2RlbCBhbmQgc3RvcmUgcmVzdWx0cyAnbScKbSA8LSBnbG0oSGVhbHRoX0JpbmFyeSB+IERfRGlldCArIFRoZXJhcGV1dGljX0Zvb2QsIGRhdGEgPSBtbCxmYW1pbHkgPSBiaW5vbWlhbCkKIyB2aWV3IGEgc3VtbWFyeSBvZiB0aGUgbW9kZWwKc3VtbWFyeShtKQojIHRlc3QgbW9kZWwgZml0CndpdGgobSwgbnVsbC5kZXZpYW5jZSAtIGRldmlhbmNlKQp3aXRoKG0sIGRmLm51bGwgLSBkZi5yZXNpZHVhbCkKd2l0aChtLCBwY2hpc3EobnVsbC5kZXZpYW5jZSAtIGRldmlhbmNlLCBkZi5udWxsIC0gZGYucmVzaWR1YWwsIGxvd2VyLnRhaWwgPSBGQUxTRSkpCkJJQyhtKQojIEhvc21lci1MZW1lc2hvdyBHb29kbmVzcy1vZi1GaXQgVGVzdApobHRlc3QobSwgRz0zKQojIyBDSXMgdXNpbmcgcHJvZmlsZWQgbG9nLWxpa2VsaWhvb2QKY29uZmludChtLCBsZXZlbD0wLjk5KQojIyBDSXMgdXNpbmcgc3RhbmRhcmQgZXJyb3JzCmNvbmZpbnQuZGVmYXVsdChtLCBsZXZlbD0wLjk5KQojIFdhbGQgdGVzdAp3YWxkLnRlc3QoYiA9IGNvZWYobSksIFNpZ21hID0gdmNvdihtKSwgVGVybXMgPSAyKQojIyBvZGRzIHJhdGlvcyBhbmQgOTUlIENJCmV4cChjYmluZChPUiA9IGNvZWYobSksIGNvbmZpbnQobSwgbGV2ZWw9MC45OSkpKQpgYGAKCiMjIyBDcmVhdGUgUk9DUiBmcm9tIGRhdGEKYGBge3IgUk9DMjh9CiMjIHRyYWluaW5nIGRhdGEKcHJlZC5tdHQgPSBwcmVkaWN0KG0sIHR5cGUgPSAicmVzcG9uc2UiKSAjcmVwZWF0IHJpc2sgcHJlZGljdGlvbnMgZnJvbSBtb2RlbCBtCnJvY3IucHJlZC5tdHQgPSBST0NSOjpwcmVkaWN0aW9uKHByZWQubXR0LCBsYWJlbHMgPSBtbCRIZWFsdGhfQmluYXJ5KSAjUk9DUiBwcmVkaWN0aW9uIG9iamVjdApyb2MucGVyZi5tdHQgPSBST0NSOjpwZXJmb3JtYW5jZShyb2NyLnByZWQubXR0LCBtZWFzdXJlID0gInRwciIsIHgubWVhc3VyZSA9ICJmcHIiKSAjICNST0NSIHBlcmZvcm1hbmNlIG9iamVjdApwbG90KHJvYy5wZXJmLm10dCwgY29sID0gImJsdWUiKQphYmxpbmUoYSA9IDAsIGIgPSAxLCBsdHkgPSAyKSAjZGlhZ29uYWwgZm9yIHJhbmRvbSBhc3NpZ25tZW50CmBgYAoKIyMjIFJlcG9ydCBBVUMgZnJvbSBST0MgZm9yIHRyYWluaW5nIGFuZCB0ZXN0IGRhdGEKYGBge3IgQVVDMjh9CiAgIyBUcmFpbiBBVUMKYXVjIDwtIFJPQ1I6OnBlcmZvcm1hbmNlKHJvY3IucHJlZC5tdHQsIG1lYXN1cmUgPSAiYXVjIikKICBhdWMgPC0gYXVjQHkudmFsdWVzW1sxXV0KICBwcmludChhdWMpCmBgYAoKCiMjIyBDYWxjdWxhdGUgTmFnZWxrZXJrZSBSXjIKYGBge3IgOTVhfQpOYWdlbGtlcmtlUjIobSkKYGBgCgojIyMgY2hlY2sgYXNzdW1wdGlvbnMgb2YgbW9kZWwKIyMjIyBDb29rJ3MgZGlzdGFuY2UKYGBge3IgOTZ9CnBsb3QobSwgd2hpY2ggPSA0LCBpZC5uID0gMykKYGBgCgojIyMjIEV4dHJhY3QgbW9kZWwgcmVzdWx0cyBhbmQgZGlzcGxheSBkYXRhIGZvciB0b3AgMyB2YWx1ZXMgdXNpbmcgQ29vaydzIGRpc3RhbmNlCmBgYHtyIDk3fQptb2RlbC5kYXRhIDwtIGF1Z21lbnQobSkgJT4lIAogIG11dGF0ZShpbmRleCA9IDE6bigpKSAKbW9kZWwuZGF0YSAlPiUgdG9wX24oMywgLmNvb2tzZCkKYGBgCgojIyMjIHBsb3Qgc3RhbmRhcmRpc2VkIHJlc2lkdWFscwpgYGB7ciA5OH0KZ2dwbG90KG1vZGVsLmRhdGEsIGFlcyhpbmRleCwgLnN0ZC5yZXNpZCkpICsgCiAgZ2VvbV9wb2ludChhZXMoY29sb3IgPSBIZWFsdGhfQmluYXJ5KSwgYWxwaGEgPSAuNSkgKwogIHRoZW1lX2J3KCkKYGBgCgojIyMjIEZpbHRlciBwb3RlbnRpYWwgaW5mbHVlbnRpYWwgZGF0YSBwb2ludHMgd2l0aCBhYnMoLnN0ZC5yZXMpID4gMzoKYGBge3IgOTl9Cm1vZGVsLmRhdGEgJT4lIAogIGZpbHRlcihhYnMoLnN0ZC5yZXNpZCkgPiAzKQpgYGAKCiMjIyMgY2hlY2sgZm9yIG11bHRpY29sbGluZWFyaXR5CmBgYHtyIEkxfQpjYXI6OnZpZihtKQpgYGAKCgoKIyMgRE9HIERJRVQgKiBUSEVSQVBFVVRJQyBESUVUIGJpbmFyeSBsb2dpc3RpYyByZWdyZXNzaW9uIE9OIFNJR05JRklDQU5UIE9SIFNFUklPVVMgSUxMTkVTUwpgYGB7ciBJMn0KIyBmaXQgYmluYXJ5IGxvZ2l0IG1vZGVsIGFuZCBzdG9yZSByZXN1bHRzICdtJwptIDwtIGdsbShIZWFsdGhfQmluYXJ5IH4gRF9EaWV0KlRoZXJhcGV1dGljX0Zvb2QsIGRhdGEgPSBtbCxmYW1pbHkgPSBiaW5vbWlhbCkKIyB2aWV3IGEgc3VtbWFyeSBvZiB0aGUgbW9kZWwKc3VtbWFyeShtKQojIHRlc3QgbW9kZWwgZml0CndpdGgobSwgbnVsbC5kZXZpYW5jZSAtIGRldmlhbmNlKQp3aXRoKG0sIGRmLm51bGwgLSBkZi5yZXNpZHVhbCkKd2l0aChtLCBwY2hpc3EobnVsbC5kZXZpYW5jZSAtIGRldmlhbmNlLCBkZi5udWxsIC0gZGYucmVzaWR1YWwsIGxvd2VyLnRhaWwgPSBGQUxTRSkpCkJJQyhtKQojIEhvc21lci1MZW1lc2hvdyBHb29kbmVzcy1vZi1GaXQgVGVzdApobHRlc3QobSwgRz03KQojIyBDSXMgdXNpbmcgcHJvZmlsZWQgbG9nLWxpa2VsaWhvb2QKY29uZmludChtLCBsZXZlbD0wLjk5KQojIyBDSXMgdXNpbmcgc3RhbmRhcmQgZXJyb3JzCmNvbmZpbnQuZGVmYXVsdChtLCBsZXZlbD0wLjk5KQojIFdhbGQgdGVzdAp3YWxkLnRlc3QoYiA9IGNvZWYobSksIFNpZ21hID0gdmNvdihtKSwgVGVybXMgPSAyKQojIyBvZGRzIHJhdGlvcyBhbmQgOTUlIENJCmV4cChjYmluZChPUiA9IGNvZWYobSksIGNvbmZpbnQobSwgbGV2ZWw9MC45OSkpKQpgYGAKCiMjIyBDcmVhdGUgUk9DUiBmcm9tIGRhdGEKYGBge3IgUk9DMjl9CiMjIHRyYWluaW5nIGRhdGEKcHJlZC5tdHQgPSBwcmVkaWN0KG0sIHR5cGUgPSAicmVzcG9uc2UiKSAjcmVwZWF0IHJpc2sgcHJlZGljdGlvbnMgZnJvbSBtb2RlbCBtCnJvY3IucHJlZC5tdHQgPSBST0NSOjpwcmVkaWN0aW9uKHByZWQubXR0LCBsYWJlbHMgPSBtbCRIZWFsdGhfQmluYXJ5KSAjUk9DUiBwcmVkaWN0aW9uIG9iamVjdApyb2MucGVyZi5tdHQgPSBST0NSOjpwZXJmb3JtYW5jZShyb2NyLnByZWQubXR0LCBtZWFzdXJlID0gInRwciIsIHgubWVhc3VyZSA9ICJmcHIiKSAjICNST0NSIHBlcmZvcm1hbmNlIG9iamVjdApwbG90KHJvYy5wZXJmLm10dCwgY29sID0gImJsdWUiKQphYmxpbmUoYSA9IDAsIGIgPSAxLCBsdHkgPSAyKSAjZGlhZ29uYWwgZm9yIHJhbmRvbSBhc3NpZ25tZW50CmBgYAoKIyMjIFJlcG9ydCBBVUMgZnJvbSBST0MgZm9yIHRyYWluaW5nIGFuZCB0ZXN0IGRhdGEKYGBge3IgQVVDMjl9CiAgIyBUcmFpbiBBVUMKYXVjIDwtIFJPQ1I6OnBlcmZvcm1hbmNlKHJvY3IucHJlZC5tdHQsIG1lYXN1cmUgPSAiYXVjIikKICBhdWMgPC0gYXVjQHkudmFsdWVzW1sxXV0KICBwcmludChhdWMpCmBgYAoKCiMjIyBDYWxjdWxhdGUgTmFnZWxrZXJrZSBSXjIKYGBge3IgSTJhfQpOYWdlbGtlcmtlUjIobSkKYGBgCgojIyMgY2hlY2sgYXNzdW1wdGlvbnMgb2YgbW9kZWwKIyMjIyBDb29rJ3MgZGlzdGFuY2UKYGBge3IgSTN9CnBsb3QobSwgd2hpY2ggPSA0LCBpZC5uID0gMykKYGBgCgojIyMjIEV4dHJhY3QgbW9kZWwgcmVzdWx0cyBhbmQgZGlzcGxheSBkYXRhIGZvciB0b3AgMyB2YWx1ZXMgdXNpbmcgQ29vaydzIGRpc3RhbmNlCmBgYHtyIEk0fQptb2RlbC5kYXRhIDwtIGF1Z21lbnQobSkgJT4lIAogIG11dGF0ZShpbmRleCA9IDE6bigpKSAKbW9kZWwuZGF0YSAlPiUgdG9wX24oMywgLmNvb2tzZCkKYGBgCgojIyMjIHBsb3Qgc3RhbmRhcmRpc2VkIHJlc2lkdWFscwpgYGB7ciBJNn0KZ2dwbG90KG1vZGVsLmRhdGEsIGFlcyhpbmRleCwgLnN0ZC5yZXNpZCkpICsgCiAgZ2VvbV9wb2ludChhZXMoY29sb3IgPSBIZWFsdGhfQmluYXJ5KSwgYWxwaGEgPSAuNSkgKwogIHRoZW1lX2J3KCkKYGBgCgojIyMjIEZpbHRlciBwb3RlbnRpYWwgaW5mbHVlbnRpYWwgZGF0YSBwb2ludHMgd2l0aCBhYnMoLnN0ZC5yZXMpID4gMzoKYGBge3IgSTd9Cm1vZGVsLmRhdGEgJT4lIAogIGZpbHRlcihhYnMoLnN0ZC5yZXNpZCkgPiAzKQpgYGAKCiMjIyMgY2hlY2sgZm9yIG11bHRpY29sbGluZWFyaXR5CiMjIyMjIE5vdGUgaW50ZXJhY3Rpb25zCmBgYHtyIEk4fQpjYXI6OnZpZihtKQpgYGAKCgoKIyMgU0laRSBCaW5hcnkgbG9naXN0aWMgcmVncmVzc2lvbiBPTiBTSUdOSUZJQ0FOVCBPUiBTRVJJT1VTIElMTE5FU1MKYGBge3IgSTg5fQojIGZpdCBiaW5hcnkgbG9naXQgbW9kZWwgYW5kIHN0b3JlIHJlc3VsdHMgJ20nCm0gPC0gZ2xtKEhlYWx0aF9CaW5hcnkgfiBTaXplLCBkYXRhID0gbWwsZmFtaWx5ID0gYmlub21pYWwpCiMgdmlldyBhIHN1bW1hcnkgb2YgdGhlIG1vZGVsCnN1bW1hcnkobSkKIyB0ZXN0IG1vZGVsIGZpdAp3aXRoKG0sIG51bGwuZGV2aWFuY2UgLSBkZXZpYW5jZSkKd2l0aChtLCBkZi5udWxsIC0gZGYucmVzaWR1YWwpCndpdGgobSwgcGNoaXNxKG51bGwuZGV2aWFuY2UgLSBkZXZpYW5jZSwgZGYubnVsbCAtIGRmLnJlc2lkdWFsLCBsb3dlci50YWlsID0gRkFMU0UpKQpCSUMobSkKIyBIb3NtZXItTGVtZXNob3cgR29vZG5lc3Mtb2YtRml0IFRlc3QKaGx0ZXN0KG0pCiMjIENJcyB1c2luZyBwcm9maWxlZCBsb2ctbGlrZWxpaG9vZApjb25maW50KG0sIGxldmVsPTAuOTkpCiMjIENJcyB1c2luZyBzdGFuZGFyZCBlcnJvcnMKY29uZmludC5kZWZhdWx0KG0sIGxldmVsPTAuOTkpCiMgV2FsZCB0ZXN0CndhbGQudGVzdChiID0gY29lZihtKSwgU2lnbWEgPSB2Y292KG0pLCBUZXJtcyA9IDIpCiMjIG9kZHMgcmF0aW9zIGFuZCA5NSUgQ0kKZXhwKGNiaW5kKE9SID0gY29lZihtKSwgY29uZmludChtLCBsZXZlbD0wLjk5KSkpCmBgYAoKIyMjIENyZWF0ZSBST0NSIGZyb20gZGF0YQpgYGB7ciBST0MzMH0KIyMgdHJhaW5pbmcgZGF0YQpwcmVkLm10dCA9IHByZWRpY3QobSwgdHlwZSA9ICJyZXNwb25zZSIpICNyZXBlYXQgcmlzayBwcmVkaWN0aW9ucyBmcm9tIG1vZGVsIG0Kcm9jci5wcmVkLm10dCA9IFJPQ1I6OnByZWRpY3Rpb24ocHJlZC5tdHQsIGxhYmVscyA9IG1sJEhlYWx0aF9CaW5hcnkpICNST0NSIHByZWRpY3Rpb24gb2JqZWN0CnJvYy5wZXJmLm10dCA9IFJPQ1I6OnBlcmZvcm1hbmNlKHJvY3IucHJlZC5tdHQsIG1lYXN1cmUgPSAidHByIiwgeC5tZWFzdXJlID0gImZwciIpICMgI1JPQ1IgcGVyZm9ybWFuY2Ugb2JqZWN0CnBsb3Qocm9jLnBlcmYubXR0LCBjb2wgPSAiYmx1ZSIpCmFibGluZShhID0gMCwgYiA9IDEsIGx0eSA9IDIpICNkaWFnb25hbCBmb3IgcmFuZG9tIGFzc2lnbm1lbnQKYGBgCgojIyMgUmVwb3J0IEFVQyBmcm9tIFJPQyBmb3IgdHJhaW5pbmcgYW5kIHRlc3QgZGF0YQpgYGB7ciBBVUMzMH0KICAjIFRyYWluIEFVQwphdWMgPC0gUk9DUjo6cGVyZm9ybWFuY2Uocm9jci5wcmVkLm10dCwgbWVhc3VyZSA9ICJhdWMiKQogIGF1YyA8LSBhdWNAeS52YWx1ZXNbWzFdXQogIHByaW50KGF1YykKYGBgCgojIyMgQ2FsY3VsYXRlIE5hZ2Vsa2Vya2UgUl4yCmBgYHtyIEkyYXF9Ck5hZ2Vsa2Vya2VSMihtKQpgYGAKCgoKIyMjIGNoZWNrIGFzc3VtcHRpb25zIG9mIG1vZGVsCiMjIyMgQ29vaydzIGRpc3RhbmNlCmBgYHtyIEk5MH0KcGxvdChtLCB3aGljaCA9IDQsIGlkLm4gPSAzKQpgYGAKCiMjIyMgRXh0cmFjdCBtb2RlbCByZXN1bHRzIGFuZCBkaXNwbGF5IGRhdGEgZm9yIHRvcCAzIHZhbHVlcyB1c2luZyBDb29rJ3MgZGlzdGFuY2UKYGBge3IgSTkxfQptb2RlbC5kYXRhIDwtIGF1Z21lbnQobSkgJT4lIAogIG11dGF0ZShpbmRleCA9IDE6bigpKSAKbW9kZWwuZGF0YSAlPiUgdG9wX24oMywgLmNvb2tzZCkKYGBgCgojIyMjIHBsb3Qgc3RhbmRhcmRpc2VkIHJlc2lkdWFscwpgYGB7ciBJOTJ9CmdncGxvdChtb2RlbC5kYXRhLCBhZXMoaW5kZXgsIC5zdGQucmVzaWQpKSArIAogIGdlb21fcG9pbnQoYWVzKGNvbG9yID0gSGVhbHRoX0JpbmFyeSksIGFscGhhID0gLjUpICsKICB0aGVtZV9idygpCmBgYAoKIyMjIyBGaWx0ZXIgcG90ZW50aWFsIGluZmx1ZW50aWFsIGRhdGEgcG9pbnRzIHdpdGggYWJzKC5zdGQucmVzKSA+IDM6CmBgYHtyIEk5M30KbW9kZWwuZGF0YSAlPiUgCiAgZmlsdGVyKGFicyguc3RkLnJlc2lkKSA+IDMpCmBgYAoKCgojIyBTSVpFMiBCaW5hcnkgbG9naXN0aWMgcmVncmVzc2lvbiBmb3IgSEVBTFRICmBgYHtyIEk4OWl9CiMgZml0IGJpbmFyeSBsb2dpdCBtb2RlbCBhbmQgc3RvcmUgcmVzdWx0cyAnbScKbSA8LSBnbG0oSGVhbHRoX0JpbmFyeSB+IFNpemUyLCBkYXRhID0gbWwsZmFtaWx5ID0gYmlub21pYWwpCiMgdmlldyBhIHN1bW1hcnkgb2YgdGhlIG1vZGVsCnN1bW1hcnkobSkKIyB0ZXN0IG1vZGVsIGZpdAp3aXRoKG0sIG51bGwuZGV2aWFuY2UgLSBkZXZpYW5jZSkKd2l0aChtLCBkZi5udWxsIC0gZGYucmVzaWR1YWwpCndpdGgobSwgcGNoaXNxKG51bGwuZGV2aWFuY2UgLSBkZXZpYW5jZSwgZGYubnVsbCAtIGRmLnJlc2lkdWFsLCBsb3dlci50YWlsID0gRkFMU0UpKQpCSUMobSkKIyBIb3NtZXItTGVtZXNob3cgR29vZG5lc3Mtb2YtRml0IFRlc3QKaGx0ZXN0KG0pCiMjIENJcyB1c2luZyBwcm9maWxlZCBsb2ctbGlrZWxpaG9vZApjb25maW50KG0sIGxldmVsPTAuOTkpCiMjIENJcyB1c2luZyBzdGFuZGFyZCBlcnJvcnMKY29uZmludC5kZWZhdWx0KG0sIGxldmVsPTAuOTkpCiMgV2FsZCB0ZXN0CndhbGQudGVzdChiID0gY29lZihtKSwgU2lnbWEgPSB2Y292KG0pLCBUZXJtcyA9IDIpCiMjIG9kZHMgcmF0aW9zIGFuZCA5NSUgQ0kKZXhwKGNiaW5kKE9SID0gY29lZihtKSwgY29uZmludChtLCBsZXZlbD0wLjk5KSkpCmBgYAoKIyMjIENyZWF0ZSBST0NSIGZyb20gZGF0YQpgYGB7ciBST0MzMX0KIyMgdHJhaW5pbmcgZGF0YQpwcmVkLm10dCA9IHByZWRpY3QobSwgdHlwZSA9ICJyZXNwb25zZSIpICNyZXBlYXQgcmlzayBwcmVkaWN0aW9ucyBmcm9tIG1vZGVsIG0Kcm9jci5wcmVkLm10dCA9IFJPQ1I6OnByZWRpY3Rpb24ocHJlZC5tdHQsIGxhYmVscyA9IG1sJEhlYWx0aF9CaW5hcnkpICNST0NSIHByZWRpY3Rpb24gb2JqZWN0CnJvYy5wZXJmLm10dCA9IFJPQ1I6OnBlcmZvcm1hbmNlKHJvY3IucHJlZC5tdHQsIG1lYXN1cmUgPSAidHByIiwgeC5tZWFzdXJlID0gImZwciIpICMgI1JPQ1IgcGVyZm9ybWFuY2Ugb2JqZWN0CnBsb3Qocm9jLnBlcmYubXR0LCBjb2wgPSAiYmx1ZSIpCmFibGluZShhID0gMCwgYiA9IDEsIGx0eSA9IDIpICNkaWFnb25hbCBmb3IgcmFuZG9tIGFzc2lnbm1lbnQKYGBgCgojIyMgUmVwb3J0IEFVQyBmcm9tIFJPQyBmb3IgdHJhaW5pbmcgYW5kIHRlc3QgZGF0YQpgYGB7ciBBVUMzMX0KICAjIFRyYWluIEFVQwphdWMgPC0gUk9DUjo6cGVyZm9ybWFuY2Uocm9jci5wcmVkLm10dCwgbWVhc3VyZSA9ICJhdWMiKQogIGF1YyA8LSBhdWNAeS52YWx1ZXNbWzFdXQogIHByaW50KGF1YykKYGBgCgoKIyMjIENhbGN1bGF0ZSBOYWdlbGtlcmtlIFJeMgpgYGB7ciBJODlpaWl9Ck5hZ2Vsa2Vya2VSMihtKQpgYGAKCiMjIyBjaGVjayBhc3N1bXB0aW9ucyBvZiBtb2RlbAojIyMjIENvb2sncyBkaXN0YW5jZQpgYGB7ciBJOTBpfQpwbG90KG0sIHdoaWNoID0gNCwgaWQubiA9IDMpCmBgYAoKIyMjIyBFeHRyYWN0IG1vZGVsIHJlc3VsdHMgYW5kIGRpc3BsYXkgZGF0YSBmb3IgdG9wIDMgdmFsdWVzIHVzaW5nIENvb2sncyBkaXN0YW5jZQpgYGB7ciBJOTFpfQptb2RlbC5kYXRhIDwtIGF1Z21lbnQobSkgJT4lIAogIG11dGF0ZShpbmRleCA9IDE6bigpKSAKbW9kZWwuZGF0YSAlPiUgdG9wX24oMywgLmNvb2tzZCkKYGBgCgojIyMjIHBsb3Qgc3RhbmRhcmRpc2VkIHJlc2lkdWFscwpgYGB7ciBJOTJpfQpnZ3Bsb3QobW9kZWwuZGF0YSwgYWVzKGluZGV4LCAuc3RkLnJlc2lkKSkgKyAKICBnZW9tX3BvaW50KGFlcyhjb2xvciA9IEhlYWx0aF9CaW5hcnkpLCBhbHBoYSA9IC41KSArCiAgdGhlbWVfYncoKQpgYGAKCiMjIyMgRmlsdGVyIHBvdGVudGlhbCBpbmZsdWVudGlhbCBkYXRhIHBvaW50cyB3aXRoIGFicyguc3RkLnJlcykgPiAzOgpgYGB7ciBJOTNpfQptb2RlbC5kYXRhICU+JSAKICBmaWx0ZXIoYWJzKC5zdGQucmVzaWQpID4gMykKYGBgCgoKCiMjIFNJWkVfR0lBTlQgQmluYXJ5IGxvZ2lzdGljIHJlZ3Jlc3Npb24gZm9yIEhFQUxUSApgYGB7ciBJODlpaX0KIyBmaXQgYmluYXJ5IGxvZ2l0IG1vZGVsIGFuZCBzdG9yZSByZXN1bHRzICdtJwptIDwtIGdsbShIZWFsdGhfQmluYXJ5IH4gU2l6ZV9HaWFudCwgZGF0YSA9IG1sLGZhbWlseSA9IGJpbm9taWFsKQojIHZpZXcgYSBzdW1tYXJ5IG9mIHRoZSBtb2RlbApzdW1tYXJ5KG0pCiMgdGVzdCBtb2RlbCBmaXQKd2l0aChtLCBudWxsLmRldmlhbmNlIC0gZGV2aWFuY2UpCndpdGgobSwgZGYubnVsbCAtIGRmLnJlc2lkdWFsKQp3aXRoKG0sIHBjaGlzcShudWxsLmRldmlhbmNlIC0gZGV2aWFuY2UsIGRmLm51bGwgLSBkZi5yZXNpZHVhbCwgbG93ZXIudGFpbCA9IEZBTFNFKSkKQklDKG0pCgojIyBDSXMgdXNpbmcgcHJvZmlsZWQgbG9nLWxpa2VsaWhvb2QKY29uZmludChtLCBsZXZlbD0wLjk5KQojIyBDSXMgdXNpbmcgc3RhbmRhcmQgZXJyb3JzCmNvbmZpbnQuZGVmYXVsdChtLCBsZXZlbD0wLjk5KQojIFdhbGQgdGVzdAp3YWxkLnRlc3QoYiA9IGNvZWYobSksIFNpZ21hID0gdmNvdihtKSwgVGVybXMgPSAyKQojIyBvZGRzIHJhdGlvcyBhbmQgOTUlIENJCmV4cChjYmluZChPUiA9IGNvZWYobSksIGNvbmZpbnQobSwgbGV2ZWw9MC45OSkpKQpgYGAKCiMjIyBDcmVhdGUgUk9DUiBmcm9tIGRhdGEKYGBge3IgUk9DMzJ9CiMjIHRyYWluaW5nIGRhdGEKcHJlZC5tdHQgPSBwcmVkaWN0KG0sIHR5cGUgPSAicmVzcG9uc2UiKSAjcmVwZWF0IHJpc2sgcHJlZGljdGlvbnMgZnJvbSBtb2RlbCBtCnJvY3IucHJlZC5tdHQgPSBST0NSOjpwcmVkaWN0aW9uKHByZWQubXR0LCBsYWJlbHMgPSBtbCRIZWFsdGhfQmluYXJ5KSAjUk9DUiBwcmVkaWN0aW9uIG9iamVjdApyb2MucGVyZi5tdHQgPSBST0NSOjpwZXJmb3JtYW5jZShyb2NyLnByZWQubXR0LCBtZWFzdXJlID0gInRwciIsIHgubWVhc3VyZSA9ICJmcHIiKSAjICNST0NSIHBlcmZvcm1hbmNlIG9iamVjdApwbG90KHJvYy5wZXJmLm10dCwgY29sID0gImJsdWUiKQphYmxpbmUoYSA9IDAsIGIgPSAxLCBsdHkgPSAyKSAjZGlhZ29uYWwgZm9yIHJhbmRvbSBhc3NpZ25tZW50CmBgYAoKIyMjIFJlcG9ydCBBVUMgZnJvbSBST0MgZm9yIHRyYWluaW5nIGFuZCB0ZXN0IGRhdGEKYGBge3IgQVVDMzJ9CiAgIyBUcmFpbiBBVUMKYXVjIDwtIFJPQ1I6OnBlcmZvcm1hbmNlKHJvY3IucHJlZC5tdHQsIG1lYXN1cmUgPSAiYXVjIikKICBhdWMgPC0gYXVjQHkudmFsdWVzW1sxXV0KICBwcmludChhdWMpCmBgYAoKCiMjIyBDYWxjdWxhdGUgTmFnZWxrZXJrZSBSXjIKYGBge3IgSTg5aXZ9Ck5hZ2Vsa2Vya2VSMihtKQpgYGAKCiMjIyBjaGVjayBhc3N1bXB0aW9ucyBvZiBtb2RlbAojIyMjIENvb2sncyBkaXN0YW5jZQpgYGB7ciBJOTBpaX0KcGxvdChtLCB3aGljaCA9IDQsIGlkLm4gPSAzKQpgYGAKCiMjIyMgRXh0cmFjdCBtb2RlbCByZXN1bHRzIGFuZCBkaXNwbGF5IGRhdGEgZm9yIHRvcCAzIHZhbHVlcyB1c2luZyBDb29rJ3MgZGlzdGFuY2UKYGBge3IgSTkxaWl9Cm1vZGVsLmRhdGEgPC0gYXVnbWVudChtKSAlPiUgCiAgbXV0YXRlKGluZGV4ID0gMTpuKCkpIAptb2RlbC5kYXRhICU+JSB0b3BfbigzLCAuY29va3NkKQpgYGAKCiMjIyMgcGxvdCBzdGFuZGFyZGlzZWQgcmVzaWR1YWxzCmBgYHtyIEk5MmlpfQpnZ3Bsb3QobW9kZWwuZGF0YSwgYWVzKGluZGV4LCAuc3RkLnJlc2lkKSkgKyAKICBnZW9tX3BvaW50KGFlcyhjb2xvciA9IEhlYWx0aF9CaW5hcnkpLCBhbHBoYSA9IC41KSArCiAgdGhlbWVfYncoKQpgYGAKCiMjIyMgRmlsdGVyIHBvdGVudGlhbCBpbmZsdWVudGlhbCBkYXRhIHBvaW50cyB3aXRoIGFicyguc3RkLnJlcykgPiAzOgpgYGB7ciBJOTNpaX0KbW9kZWwuZGF0YSAlPiUgCiAgZmlsdGVyKGFicyguc3RkLnJlc2lkKSA+IDMpCmBgYAoKCgojIyBEX0FHRSBCaW5hcnkgbG9naXN0aWMgcmVncmVzc2lvbiBmb3IgSEVBTFRICmBgYHtyIEk5NH0KIyBmaXQgYmluYXJ5IGxvZ2l0IG1vZGVsIGFuZCBzdG9yZSByZXN1bHRzICdtJwptIDwtIGdsbShIZWFsdGhfQmluYXJ5IH4gRF9BZ2UsIGRhdGEgPSBtbCxmYW1pbHkgPSBiaW5vbWlhbCkKIyB2aWV3IGEgc3VtbWFyeSBvZiB0aGUgbW9kZWwKc3VtbWFyeShtKQojIHRlc3QgbW9kZWwgZml0CndpdGgobSwgbnVsbC5kZXZpYW5jZSAtIGRldmlhbmNlKQp3aXRoKG0sIGRmLm51bGwgLSBkZi5yZXNpZHVhbCkKd2l0aChtLCBwY2hpc3EobnVsbC5kZXZpYW5jZSAtIGRldmlhbmNlLCBkZi5udWxsIC0gZGYucmVzaWR1YWwsIGxvd2VyLnRhaWwgPSBGQUxTRSkpCkJJQyhtKQojIEhvc21lci1MZW1lc2hvdyBHb29kbmVzcy1vZi1GaXQgVGVzdApobHRlc3QobSkKIyMgQ0lzIHVzaW5nIHByb2ZpbGVkIGxvZy1saWtlbGlob29kCmNvbmZpbnQobSwgbGV2ZWw9MC45OSkKIyMgQ0lzIHVzaW5nIHN0YW5kYXJkIGVycm9ycwpjb25maW50LmRlZmF1bHQobSwgbGV2ZWw9MC45OSkKIyBXYWxkIHRlc3QKd2FsZC50ZXN0KGIgPSBjb2VmKG0pLCBTaWdtYSA9IHZjb3YobSksIFRlcm1zID0gMikKIyMgb2RkcyByYXRpb3MgYW5kIDk1JSBDSQpleHAoY2JpbmQoT1IgPSBjb2VmKG0pLCBjb25maW50KG0sIGxldmVsPTAuOTkpKSkKYGBgCgojIyMgQ3JlYXRlIFJPQ1IgZnJvbSBkYXRhCmBgYHtyIFJPQzMzfQojIyB0cmFpbmluZyBkYXRhCnByZWQubXR0ID0gcHJlZGljdChtLCB0eXBlID0gInJlc3BvbnNlIikgI3JlcGVhdCByaXNrIHByZWRpY3Rpb25zIGZyb20gbW9kZWwgbQpyb2NyLnByZWQubXR0ID0gUk9DUjo6cHJlZGljdGlvbihwcmVkLm10dCwgbGFiZWxzID0gbWwkSGVhbHRoX0JpbmFyeSkgI1JPQ1IgcHJlZGljdGlvbiBvYmplY3QKcm9jLnBlcmYubXR0ID0gUk9DUjo6cGVyZm9ybWFuY2Uocm9jci5wcmVkLm10dCwgbWVhc3VyZSA9ICJ0cHIiLCB4Lm1lYXN1cmUgPSAiZnByIikgIyAjUk9DUiBwZXJmb3JtYW5jZSBvYmplY3QKcGxvdChyb2MucGVyZi5tdHQsIGNvbCA9ICJibHVlIikKYWJsaW5lKGEgPSAwLCBiID0gMSwgbHR5ID0gMikgI2RpYWdvbmFsIGZvciByYW5kb20gYXNzaWdubWVudApgYGAKCiMjIyBSZXBvcnQgQVVDIGZyb20gUk9DIGZvciB0cmFpbmluZyBhbmQgdGVzdCBkYXRhCmBgYHtyIEFVQzMzfQogICMgVHJhaW4gQVVDCmF1YyA8LSBST0NSOjpwZXJmb3JtYW5jZShyb2NyLnByZWQubXR0LCBtZWFzdXJlID0gImF1YyIpCiAgYXVjIDwtIGF1Y0B5LnZhbHVlc1tbMV1dCiAgcHJpbnQoYXVjKQpgYGAKCgojIyMgQ2FsY3VsYXRlIE5hZ2Vsa2Vya2UgUl4yCmBgYHtyIEk5NGF9Ck5hZ2Vsa2Vya2VSMihtKQpgYGAKCiMjIyBDaGVjayBhZ2UgaXMgbGluZWFyIHdpdGggbG9naXQgb2Ygb3V0Y29tZQojIyMjIyBOb3RlIGxhY2sgb2YgbGluZWFyaXR5CmBgYHtyIEk5NX0KeXByZWQgPSBwcmVkaWN0KG0pCnJlcyA9IHJlc2lkdWFscyhtLCB0eXBlID0gJ2RldmlhbmNlJykKcGxvdCh5cHJlZCxyZXMpCmBgYAoKIyMjIEJveCBUaWR3ZWxsIHRlc3QgdG8gY2hlY2sgdGhhdCBEX0FnZSBpcyBsaW5lYXJseSBhc3NvY2lhdGVkIHdpdGggdGhlIGxvZ2l0IG9mIHRoZSBvdXRjb21lCiMjIyMjIHN1Z2dlc3RzIG5vdCBsaW5lYXIKYGBge3IgSTk2fQpib3hUaWR3ZWxsKG1sJEhlYWx0aF9CaW5hcnkgfiBtbCREX0FnZSkKYGBgCgojIyMgY2hlY2sgYXNzdW1wdGlvbnMgb2YgbW9kZWwKIyMjIyBDb29rJ3MgZGlzdGFuY2UKYGBge3IgSTk3fQpwbG90KG0sIHdoaWNoID0gNCwgaWQubiA9IDMpCmBgYAoKIyMjIyBFeHRyYWN0IG1vZGVsIHJlc3VsdHMgYW5kIGRpc3BsYXkgZGF0YSBmb3IgdG9wIDMgdmFsdWVzIHVzaW5nIENvb2sncyBkaXN0YW5jZQpgYGB7ciBJOTh9Cm1vZGVsLmRhdGEgPC0gYXVnbWVudChtKSAlPiUgCiAgbXV0YXRlKGluZGV4ID0gMTpuKCkpIAptb2RlbC5kYXRhICU+JSB0b3BfbigzLCAuY29va3NkKQpgYGAKCiMjIyMgcGxvdCBzdGFuZGFyZGlzZWQgcmVzaWR1YWxzCmBgYHtyIEk5OX0KZ2dwbG90KG1vZGVsLmRhdGEsIGFlcyhpbmRleCwgLnN0ZC5yZXNpZCkpICsgCiAgZ2VvbV9wb2ludChhZXMoY29sb3IgPSBIZWFsdGhfQmluYXJ5KSwgYWxwaGEgPSAuNSkgKwogIHRoZW1lX2J3KCkKYGBgCgojIyMjIEZpbHRlciBwb3RlbnRpYWwgaW5mbHVlbnRpYWwgZGF0YSBwb2ludHMgd2l0aCBhYnMoLnN0ZC5yZXMpID4gMzoKYGBge3IgSTEwMH0KbW9kZWwuZGF0YSAlPiUgCiAgZmlsdGVyKGFicyguc3RkLnJlc2lkKSA+IDMpCmBgYAoKCgojIyBEX0FHRSBCaW5hcnkgbG9naXN0aWMgcmVncmVzc2lvbiBmb3IgSEVBTFRICmBgYHtyIEk5NHF9CiMgZml0IGJpbmFyeSBsb2dpdCBtb2RlbCBhbmQgc3RvcmUgcmVzdWx0cyAnbScKbSA8LSBnbG0oSGVhbHRoX0JpbmFyeSB+IGJzKERfQWdlLCBkZWdyZWU9MSxkZj0yKSwgZGF0YSA9IG1sLGZhbWlseSA9IGJpbm9taWFsKQojIHZpZXcgYSBzdW1tYXJ5IG9mIHRoZSBtb2RlbApzdW1tYXJ5KG0pCiMgdGVzdCBtb2RlbCBmaXQKd2l0aChtLCBudWxsLmRldmlhbmNlIC0gZGV2aWFuY2UpCndpdGgobSwgZGYubnVsbCAtIGRmLnJlc2lkdWFsKQp3aXRoKG0sIHBjaGlzcShudWxsLmRldmlhbmNlIC0gZGV2aWFuY2UsIGRmLm51bGwgLSBkZi5yZXNpZHVhbCwgbG93ZXIudGFpbCA9IEZBTFNFKSkKQklDKG0pCiMgSG9zbWVyLUxlbWVzaG93IEdvb2RuZXNzLW9mLUZpdCBUZXN0CmhsdGVzdChtKQojIyBDSXMgdXNpbmcgcHJvZmlsZWQgbG9nLWxpa2VsaWhvb2QKY29uZmludChtLCBsZXZlbD0wLjk5KQojIyBDSXMgdXNpbmcgc3RhbmRhcmQgZXJyb3JzCmNvbmZpbnQuZGVmYXVsdChtLCBsZXZlbD0wLjk5KQojIFdhbGQgdGVzdAp3YWxkLnRlc3QoYiA9IGNvZWYobSksIFNpZ21hID0gdmNvdihtKSwgVGVybXMgPSAyKQojIyBvZGRzIHJhdGlvcyBhbmQgOTUlIENJCmV4cChjYmluZChPUiA9IGNvZWYobSksIGNvbmZpbnQobSwgbGV2ZWw9MC45OSkpKQpgYGAKCiMjIyBDcmVhdGUgUk9DUiBmcm9tIGRhdGEKYGBge3IgUk9DMzNxfQojIyB0cmFpbmluZyBkYXRhCnByZWQubXR0ID0gcHJlZGljdChtLCB0eXBlID0gInJlc3BvbnNlIikgI3JlcGVhdCByaXNrIHByZWRpY3Rpb25zIGZyb20gbW9kZWwgbQpyb2NyLnByZWQubXR0ID0gUk9DUjo6cHJlZGljdGlvbihwcmVkLm10dCwgbGFiZWxzID0gbWwkSGVhbHRoX0JpbmFyeSkgI1JPQ1IgcHJlZGljdGlvbiBvYmplY3QKcm9jLnBlcmYubXR0ID0gUk9DUjo6cGVyZm9ybWFuY2Uocm9jci5wcmVkLm10dCwgbWVhc3VyZSA9ICJ0cHIiLCB4Lm1lYXN1cmUgPSAiZnByIikgIyAjUk9DUiBwZXJmb3JtYW5jZSBvYmplY3QKcGxvdChyb2MucGVyZi5tdHQsIGNvbCA9ICJibHVlIikKYWJsaW5lKGEgPSAwLCBiID0gMSwgbHR5ID0gMikgI2RpYWdvbmFsIGZvciByYW5kb20gYXNzaWdubWVudApgYGAKCiMjIyBSZXBvcnQgQVVDIGZyb20gUk9DIGZvciB0cmFpbmluZyBhbmQgdGVzdCBkYXRhCmBgYHtyIEFVQzMzcX0KICAjIFRyYWluIEFVQwphdWMgPC0gUk9DUjo6cGVyZm9ybWFuY2Uocm9jci5wcmVkLm10dCwgbWVhc3VyZSA9ICJhdWMiKQogIGF1YyA8LSBhdWNAeS52YWx1ZXNbWzFdXQogIHByaW50KGF1YykKYGBgCgoKIyMjIENhbGN1bGF0ZSBOYWdlbGtlcmtlIFJeMgpgYGB7ciBJOTRhcX0KTmFnZWxrZXJrZVIyKG0pCmBgYAoKCiMjIyBjaGVjayBhc3N1bXB0aW9ucyBvZiBtb2RlbAojIyMjIENvb2sncyBkaXN0YW5jZQpgYGB7ciBJOTdxfQpwbG90KG0sIHdoaWNoID0gNCwgaWQubiA9IDMpCmBgYAoKIyMjIyBFeHRyYWN0IG1vZGVsIHJlc3VsdHMgYW5kIGRpc3BsYXkgZGF0YSBmb3IgdG9wIDMgdmFsdWVzIHVzaW5nIENvb2sncyBkaXN0YW5jZQpgYGB7ciBJOThxfQptb2RlbC5kYXRhIDwtIGF1Z21lbnQobSkgJT4lIAogIG11dGF0ZShpbmRleCA9IDE6bigpKSAKbW9kZWwuZGF0YSAlPiUgdG9wX24oMywgLmNvb2tzZCkKYGBgCgojIyMjIHBsb3Qgc3RhbmRhcmRpc2VkIHJlc2lkdWFscwpgYGB7ciBJOTlxfQpnZ3Bsb3QobW9kZWwuZGF0YSwgYWVzKGluZGV4LCAuc3RkLnJlc2lkKSkgKyAKICBnZW9tX3BvaW50KGFlcyhjb2xvciA9IEhlYWx0aF9CaW5hcnkpLCBhbHBoYSA9IC41KSArCiAgdGhlbWVfYncoKQpgYGAKCiMjIyMgRmlsdGVyIHBvdGVudGlhbCBpbmZsdWVudGlhbCBkYXRhIHBvaW50cyB3aXRoIGFicyguc3RkLnJlcykgPiAzOgpgYGB7ciBJMTAwcX0KbW9kZWwuZGF0YSAlPiUgCiAgZmlsdGVyKGFicyguc3RkLnJlc2lkKSA+IDMpCmBgYAoKCgoKCgojIyBEX0FnZV9xdWFudCBsb2dpc3RpYyByZWdyZXNzaW9uIGZvciBIRUFMVEgKIyMjIyBOb3RlIGJldHRlciBtb2RlbCBmaXQgdGhhdCBEX0FnZQpgYGB7ciBJSTF9CiMgZml0IGJpbmFyeSBsb2dpdCBtb2RlbCBhbmQgc3RvcmUgcmVzdWx0cyAnbScKbSA8LSBnbG0oSGVhbHRoX0JpbmFyeSB+IERfQWdlX3F1YW50LCBkYXRhID0gbWwsZmFtaWx5ID0gYmlub21pYWwpCiMgdmlldyBhIHN1bW1hcnkgb2YgdGhlIG1vZGVsCnN1bW1hcnkobSkKIyB0ZXN0IG1vZGVsIGZpdAp3aXRoKG0sIG51bGwuZGV2aWFuY2UgLSBkZXZpYW5jZSkKd2l0aChtLCBkZi5udWxsIC0gZGYucmVzaWR1YWwpCndpdGgobSwgcGNoaXNxKG51bGwuZGV2aWFuY2UgLSBkZXZpYW5jZSwgZGYubnVsbCAtIGRmLnJlc2lkdWFsLCBsb3dlci50YWlsID0gRkFMU0UpKQpCSUMobSkKIyBIb3NtZXItTGVtZXNob3cgR29vZG5lc3Mtb2YtRml0IFRlc3QKaGx0ZXN0KG0sIEc9NCkKIyMgQ0lzIHVzaW5nIHByb2ZpbGVkIGxvZy1saWtlbGlob29kCmNvbmZpbnQobSwgbGV2ZWw9MC45OSkKIyMgQ0lzIHVzaW5nIHN0YW5kYXJkIGVycm9ycwpjb25maW50LmRlZmF1bHQobSwgbGV2ZWw9MC45OSkKIyBXYWxkIHRlc3QKd2FsZC50ZXN0KGIgPSBjb2VmKG0pLCBTaWdtYSA9IHZjb3YobSksIFRlcm1zID0gMikKIyMgb2RkcyByYXRpb3MgYW5kIDk1JSBDSQpleHAoY2JpbmQoT1IgPSBjb2VmKG0pLCBjb25maW50KG0sIGxldmVsPTAuOTkpKSkKYGBgCgojIyMgQ3JlYXRlIFJPQ1IgZnJvbSBkYXRhCmBgYHtyIFJPQzM0fQojIyB0cmFpbmluZyBkYXRhCnByZWQubXR0ID0gcHJlZGljdChtLCB0eXBlID0gInJlc3BvbnNlIikgI3JlcGVhdCByaXNrIHByZWRpY3Rpb25zIGZyb20gbW9kZWwgbQpyb2NyLnByZWQubXR0ID0gUk9DUjo6cHJlZGljdGlvbihwcmVkLm10dCwgbGFiZWxzID0gbWwkSGVhbHRoX0JpbmFyeSkgI1JPQ1IgcHJlZGljdGlvbiBvYmplY3QKcm9jLnBlcmYubXR0ID0gUk9DUjo6cGVyZm9ybWFuY2Uocm9jci5wcmVkLm10dCwgbWVhc3VyZSA9ICJ0cHIiLCB4Lm1lYXN1cmUgPSAiZnByIikgIyAjUk9DUiBwZXJmb3JtYW5jZSBvYmplY3QKcGxvdChyb2MucGVyZi5tdHQsIGNvbCA9ICJibHVlIikKYWJsaW5lKGEgPSAwLCBiID0gMSwgbHR5ID0gMikgI2RpYWdvbmFsIGZvciByYW5kb20gYXNzaWdubWVudApgYGAKCiMjIyBSZXBvcnQgQVVDIGZyb20gUk9DIGZvciB0cmFpbmluZyBhbmQgdGVzdCBkYXRhCmBgYHtyIEFVQzM0fQogICMgVHJhaW4gQVVDCmF1YyA8LSBST0NSOjpwZXJmb3JtYW5jZShyb2NyLnByZWQubXR0LCBtZWFzdXJlID0gImF1YyIpCiAgYXVjIDwtIGF1Y0B5LnZhbHVlc1tbMV1dCiAgcHJpbnQoYXVjKQpgYGAKCgojIyMgQ2FsY3VsYXRlIE5hZ2Vsa2Vya2UgUl4yCmBgYHtyIElJMWFhfQpOYWdlbGtlcmtlUjIobSkKYGBgCgojIyMgY2hlY2sgYXNzdW1wdGlvbnMgb2YgbW9kZWwKIyMjIyBDb29rJ3MgZGlzdGFuY2UKYGBge3IgSUkxYX0KcGxvdChtLCB3aGljaCA9IDQsIGlkLm4gPSAzKQpgYGAKCiMjIyMgRXh0cmFjdCBtb2RlbCByZXN1bHRzIGFuZCBkaXNwbGF5IGRhdGEgZm9yIHRvcCAzIHZhbHVlcyB1c2luZyBDb29rJ3MgZGlzdGFuY2UKYGBge3IgSUkyfQptb2RlbC5kYXRhIDwtIGF1Z21lbnQobSkgJT4lIAogIG11dGF0ZShpbmRleCA9IDE6bigpKSAKbW9kZWwuZGF0YSAlPiUgdG9wX24oMywgLmNvb2tzZCkKYGBgCgojIyMjIHBsb3Qgc3RhbmRhcmRpc2VkIHJlc2lkdWFscwpgYGB7ciBJSTN9CmdncGxvdChtb2RlbC5kYXRhLCBhZXMoaW5kZXgsIC5zdGQucmVzaWQpKSArIAogIGdlb21fcG9pbnQoYWVzKGNvbG9yID0gSGVhbHRoX0JpbmFyeSksIGFscGhhID0gLjUpICsKICB0aGVtZV9idygpCmBgYAoKIyMjIyBGaWx0ZXIgcG90ZW50aWFsIGluZmx1ZW50aWFsIGRhdGEgcG9pbnRzIHdpdGggYWJzKC5zdGQucmVzKSA+IDM6CmBgYHtyIElJNH0KbW9kZWwuZGF0YSAlPiUgCiAgZmlsdGVyKGFicyguc3RkLnJlc2lkKSA+IDMpCmBgYAoKCgojIyBEX1NFWCBCaW5hcnkgbG9naXN0aWMgcmVncmVzc2lvbiBmb3IgSEVBTFRICmBgYHtyIElJMTd9CiMgZml0IGJpbmFyeSBsb2dpdCBtb2RlbCBhbmQgc3RvcmUgcmVzdWx0cyAnbScKbSA8LSBnbG0oSGVhbHRoX0JpbmFyeSB+IERfU2V4LCBkYXRhID0gbWwsZmFtaWx5ID0gYmlub21pYWwpCiMgdmlldyBhIHN1bW1hcnkgb2YgdGhlIG1vZGVsCnN1bW1hcnkobSkKIyB0ZXN0IG1vZGVsIGZpdAp3aXRoKG0sIG51bGwuZGV2aWFuY2UgLSBkZXZpYW5jZSkKd2l0aChtLCBkZi5udWxsIC0gZGYucmVzaWR1YWwpCndpdGgobSwgcGNoaXNxKG51bGwuZGV2aWFuY2UgLSBkZXZpYW5jZSwgZGYubnVsbCAtIGRmLnJlc2lkdWFsLCBsb3dlci50YWlsID0gRkFMU0UpKQpCSUMobSkKIyMgQ0lzIHVzaW5nIHByb2ZpbGVkIGxvZy1saWtlbGlob29kCmNvbmZpbnQobSwgbGV2ZWw9MC45OSkKIyMgQ0lzIHVzaW5nIHN0YW5kYXJkIGVycm9ycwpjb25maW50LmRlZmF1bHQobSwgbGV2ZWw9MC45OSkKIyBXYWxkIHRlc3QKd2FsZC50ZXN0KGIgPSBjb2VmKG0pLCBTaWdtYSA9IHZjb3YobSksIFRlcm1zID0gMikKIyMgb2RkcyByYXRpb3MgYW5kIDk1JSBDSQpleHAoY2JpbmQoT1IgPSBjb2VmKG0pLCBjb25maW50KG0sIGxldmVsPTAuOTkpKSkKYGBgCgojIyMgQ3JlYXRlIFJPQ1IgZnJvbSBkYXRhCmBgYHtyIFJPQzM1fQojIyB0cmFpbmluZyBkYXRhCnByZWQubXR0ID0gcHJlZGljdChtLCB0eXBlID0gInJlc3BvbnNlIikgI3JlcGVhdCByaXNrIHByZWRpY3Rpb25zIGZyb20gbW9kZWwgbQpyb2NyLnByZWQubXR0ID0gUk9DUjo6cHJlZGljdGlvbihwcmVkLm10dCwgbGFiZWxzID0gbWwkSGVhbHRoX0JpbmFyeSkgI1JPQ1IgcHJlZGljdGlvbiBvYmplY3QKcm9jLnBlcmYubXR0ID0gUk9DUjo6cGVyZm9ybWFuY2Uocm9jci5wcmVkLm10dCwgbWVhc3VyZSA9ICJ0cHIiLCB4Lm1lYXN1cmUgPSAiZnByIikgIyAjUk9DUiBwZXJmb3JtYW5jZSBvYmplY3QKcGxvdChyb2MucGVyZi5tdHQsIGNvbCA9ICJibHVlIikKYWJsaW5lKGEgPSAwLCBiID0gMSwgbHR5ID0gMikgI2RpYWdvbmFsIGZvciByYW5kb20gYXNzaWdubWVudApgYGAKCiMjIyBSZXBvcnQgQVVDIGZyb20gUk9DIGZvciB0cmFpbmluZyBhbmQgdGVzdCBkYXRhCmBgYHtyIEFVQzM1fQogICMgVHJhaW4gQVVDCmF1YyA8LSBST0NSOjpwZXJmb3JtYW5jZShyb2NyLnByZWQubXR0LCBtZWFzdXJlID0gImF1YyIpCiAgYXVjIDwtIGF1Y0B5LnZhbHVlc1tbMV1dCiAgcHJpbnQoYXVjKQpgYGAKCgojIyMgQ2FsY3VsYXRlIE5hZ2Vsa2Vya2UgUl4yCmBgYHtyIElJMTdhfQpOYWdlbGtlcmtlUjIobSkKYGBgCgojIyMgY2hlY2sgYXNzdW1wdGlvbnMgb2YgbW9kZWwKIyMjIyBDb29rJ3MgZGlzdGFuY2UKYGBge3IgSUkxOH0KcGxvdChtLCB3aGljaCA9IDQsIGlkLm4gPSAzKQpgYGAKCiMjIyMgRXh0cmFjdCBtb2RlbCByZXN1bHRzIGFuZCBkaXNwbGF5IGRhdGEgZm9yIHRvcCAzIHZhbHVlcyB1c2luZyBDb29rJ3MgZGlzdGFuY2UKYGBge3IgSUkxOX0KbW9kZWwuZGF0YSA8LSBhdWdtZW50KG0pICU+JSAKICBtdXRhdGUoaW5kZXggPSAxOm4oKSkgCm1vZGVsLmRhdGEgJT4lIHRvcF9uKDMsIC5jb29rc2QpCmBgYAoKIyMjIyBwbG90IHN0YW5kYXJkaXNlZCByZXNpZHVhbHMKYGBge3IgSUkyMH0KZ2dwbG90KG1vZGVsLmRhdGEsIGFlcyhpbmRleCwgLnN0ZC5yZXNpZCkpICsgCiAgZ2VvbV9wb2ludChhZXMoY29sb3IgPSBIZWFsdGhfQmluYXJ5KSwgYWxwaGEgPSAuNSkgKwogIHRoZW1lX2J3KCkKYGBgCgojIyMjIEZpbHRlciBwb3RlbnRpYWwgaW5mbHVlbnRpYWwgZGF0YSBwb2ludHMgd2l0aCBhYnMoLnN0ZC5yZXMpID4gMzoKYGBge3IgSUkyMX0KbW9kZWwuZGF0YSAlPiUgCiAgZmlsdGVyKGFicyguc3RkLnJlc2lkKSA+IDMpCmBgYAoKCgojIyBET0cgTkVVVEVSIGJpbmFyeSBsb2dpc3RpYyByZWdyZXNzaW9uIE9OIFNJR05JRklDQU5UIE9SIFNFUklPVVMgSUxMTkVTUwpgYGB7ciAzN2R9CiMgZml0IGJpbmFyeSBsb2dpdCBtb2RlbCBhbmQgc3RvcmUgcmVzdWx0cyAnbScKbSA8LSBnbG0oSGVhbHRoX0JpbmFyeSB+IERfTmV1dGVyLCBkYXRhID0gbWwsZmFtaWx5ID0gYmlub21pYWwpCiMgdmlldyBhIHN1bW1hcnkgb2YgdGhlIG1vZGVsCnN1bW1hcnkobSkKIyB0ZXN0IG1vZGVsIGZpdAp3aXRoKG0sIG51bGwuZGV2aWFuY2UgLSBkZXZpYW5jZSkKd2l0aChtLCBkZi5udWxsIC0gZGYucmVzaWR1YWwpCndpdGgobSwgcGNoaXNxKG51bGwuZGV2aWFuY2UgLSBkZXZpYW5jZSwgZGYubnVsbCAtIGRmLnJlc2lkdWFsLCBsb3dlci50YWlsID0gRkFMU0UpKQpCSUMobSkKIyMgQ0lzIHVzaW5nIHByb2ZpbGVkIGxvZy1saWtlbGlob29kCmNvbmZpbnQobSwgbGV2ZWw9MC45OSkKIyMgQ0lzIHVzaW5nIHN0YW5kYXJkIGVycm9ycwpjb25maW50LmRlZmF1bHQobSwgbGV2ZWw9MC45OSkKIyBXYWxkIHRlc3QKd2FsZC50ZXN0KGIgPSBjb2VmKG0pLCBTaWdtYSA9IHZjb3YobSksIFRlcm1zID0gMikKIyMgb2RkcyByYXRpb3MgYW5kIDk1JSBDSQpleHAoY2JpbmQoT1IgPSBjb2VmKG0pLCBjb25maW50KG0sIGxldmVsPTAuOTkpKSkKYGBgCgojIyMgQ3JlYXRlIFJPQ1IgZnJvbSBkYXRhCmBgYHtyIFJPQzM2fQojIyB0cmFpbmluZyBkYXRhCnByZWQubXR0ID0gcHJlZGljdChtLCB0eXBlID0gInJlc3BvbnNlIikgI3JlcGVhdCByaXNrIHByZWRpY3Rpb25zIGZyb20gbW9kZWwgbQpyb2NyLnByZWQubXR0ID0gUk9DUjo6cHJlZGljdGlvbihwcmVkLm10dCwgbGFiZWxzID0gbWwkSGVhbHRoX0JpbmFyeSkgI1JPQ1IgcHJlZGljdGlvbiBvYmplY3QKcm9jLnBlcmYubXR0ID0gUk9DUjo6cGVyZm9ybWFuY2Uocm9jci5wcmVkLm10dCwgbWVhc3VyZSA9ICJ0cHIiLCB4Lm1lYXN1cmUgPSAiZnByIikgIyAjUk9DUiBwZXJmb3JtYW5jZSBvYmplY3QKcGxvdChyb2MucGVyZi5tdHQsIGNvbCA9ICJibHVlIikKYWJsaW5lKGEgPSAwLCBiID0gMSwgbHR5ID0gMikgI2RpYWdvbmFsIGZvciByYW5kb20gYXNzaWdubWVudApgYGAKCiMjIyBSZXBvcnQgQVVDIGZyb20gUk9DIGZvciB0cmFpbmluZyBhbmQgdGVzdCBkYXRhCmBgYHtyIEFVQzM2fQogICMgVHJhaW4gQVVDCmF1YyA8LSBST0NSOjpwZXJmb3JtYW5jZShyb2NyLnByZWQubXR0LCBtZWFzdXJlID0gImF1YyIpCiAgYXVjIDwtIGF1Y0B5LnZhbHVlc1tbMV1dCiAgcHJpbnQoYXVjKQpgYGAKCgojIyMgQ2FsY3VsYXRlIE5hZ2Vsa2Vya2UgUl4yCmBgYHtyIDM3YWR9Ck5hZ2Vsa2Vya2VSMihtKQpgYGAKCiMjIyBjaGVjayBhc3N1bXB0aW9ucyBvZiBtb2RlbAojIyMjIENvb2sncyBkaXN0YW5jZQpgYGB7ciAzOGR9CnBsb3QobSwgd2hpY2ggPSA0LCBpZC5uID0gMykKYGBgCgojIyMjIEV4dHJhY3QgbW9kZWwgcmVzdWx0cyBhbmQgZGlzcGxheSBkYXRhIGZvciB0b3AgMyB2YWx1ZXMgdXNpbmcgQ29vaydzIGRpc3RhbmNlCmBgYHtyIDM5ZH0KbW9kZWwuZGF0YSA8LSBhdWdtZW50KG0pICU+JSAKICBtdXRhdGUoaW5kZXggPSAxOm4oKSkgCm1vZGVsLmRhdGEgJT4lIHRvcF9uKDMsIC5jb29rc2QpCmBgYAoKIyMjIyBwbG90IHN0YW5kYXJkaXNlZCByZXNpZHVhbHMKYGBge3IgNDBkfQpnZ3Bsb3QobW9kZWwuZGF0YSwgYWVzKGluZGV4LCAuc3RkLnJlc2lkKSkgKyAKICBnZW9tX3BvaW50KGFlcyhjb2xvciA9IEhlYWx0aF9CaW5hcnkpLCBhbHBoYSA9IC41KSArCiAgdGhlbWVfYncoKQpgYGAKCiMjIyMgRmlsdGVyIHBvdGVudGlhbCBpbmZsdWVudGlhbCBkYXRhIHBvaW50cyB3aXRoIGFicyguc3RkLnJlcykgPiAzOgpgYGB7ciA0MWR9Cm1vZGVsLmRhdGEgJT4lIAogIGZpbHRlcihhYnMoLnN0ZC5yZXNpZCkgPiAzKQpgYGAKCgoKIyMgRF9TRVggKyBEX05FVVRFUiBCaW5hcnkgbG9naXN0aWMgcmVncmVzc2lvbiBPTiBTSUdOSUZJQ0FOVCBPUiBTRVJJT1VTIElMTE5FU1MKYGBge3IgSUk1fQojIGZpdCBiaW5hcnkgbG9naXQgbW9kZWwgYW5kIHN0b3JlIHJlc3VsdHMgJ20nCm0gPC0gZ2xtKEhlYWx0aF9CaW5hcnkgfiBEX1NleCArIERfTmV1dGVyLCBkYXRhID0gbWwsZmFtaWx5ID0gYmlub21pYWwpCiMgdmlldyBhIHN1bW1hcnkgb2YgdGhlIG1vZGVsCnN1bW1hcnkobSkKIyB0ZXN0IG1vZGVsIGZpdAp3aXRoKG0sIG51bGwuZGV2aWFuY2UgLSBkZXZpYW5jZSkKd2l0aChtLCBkZi5udWxsIC0gZGYucmVzaWR1YWwpCndpdGgobSwgcGNoaXNxKG51bGwuZGV2aWFuY2UgLSBkZXZpYW5jZSwgZGYubnVsbCAtIGRmLnJlc2lkdWFsLCBsb3dlci50YWlsID0gRkFMU0UpKQpCSUMobSkKIyBIb3NtZXItTGVtZXNob3cgR29vZG5lc3Mtb2YtRml0IFRlc3QKaGx0ZXN0KG0pCiMjIENJcyB1c2luZyBwcm9maWxlZCBsb2ctbGlrZWxpaG9vZApjb25maW50KG0sIGxldmVsPTAuOTkpCiMjIENJcyB1c2luZyBzdGFuZGFyZCBlcnJvcnMKY29uZmludC5kZWZhdWx0KG0sIGxldmVsPTAuOTkpCiMgV2FsZCB0ZXN0CndhbGQudGVzdChiID0gY29lZihtKSwgU2lnbWEgPSB2Y292KG0pLCBUZXJtcyA9IDIpCiMjIG9kZHMgcmF0aW9zIGFuZCA5NSUgQ0kKZXhwKGNiaW5kKE9SID0gY29lZihtKSwgY29uZmludChtLCBsZXZlbD0wLjk5KSkpCmBgYAoKIyMjIENyZWF0ZSBST0NSIGZyb20gZGF0YQpgYGB7ciBST0MzN30KIyMgdHJhaW5pbmcgZGF0YQpwcmVkLm10dCA9IHByZWRpY3QobSwgdHlwZSA9ICJyZXNwb25zZSIpICNyZXBlYXQgcmlzayBwcmVkaWN0aW9ucyBmcm9tIG1vZGVsIG0Kcm9jci5wcmVkLm10dCA9IFJPQ1I6OnByZWRpY3Rpb24ocHJlZC5tdHQsIGxhYmVscyA9IG1sJEhlYWx0aF9CaW5hcnkpICNST0NSIHByZWRpY3Rpb24gb2JqZWN0CnJvYy5wZXJmLm10dCA9IFJPQ1I6OnBlcmZvcm1hbmNlKHJvY3IucHJlZC5tdHQsIG1lYXN1cmUgPSAidHByIiwgeC5tZWFzdXJlID0gImZwciIpICMgI1JPQ1IgcGVyZm9ybWFuY2Ugb2JqZWN0CnBsb3Qocm9jLnBlcmYubXR0LCBjb2wgPSAiYmx1ZSIpCmFibGluZShhID0gMCwgYiA9IDEsIGx0eSA9IDIpICNkaWFnb25hbCBmb3IgcmFuZG9tIGFzc2lnbm1lbnQKYGBgCgojIyMgUmVwb3J0IEFVQyBmcm9tIFJPQyBmb3IgdHJhaW5pbmcgYW5kIHRlc3QgZGF0YQpgYGB7ciBBVUMzN30KICAjIFRyYWluIEFVQwphdWMgPC0gUk9DUjo6cGVyZm9ybWFuY2Uocm9jci5wcmVkLm10dCwgbWVhc3VyZSA9ICJhdWMiKQogIGF1YyA8LSBhdWNAeS52YWx1ZXNbWzFdXQogIHByaW50KGF1YykKYGBgCgoKIyMjIENhbGN1bGF0ZSBOYWdlbGtlcmtlIFJeMgpgYGB7ciBJSTVhfQpOYWdlbGtlcmtlUjIobSkKYGBgCgojIyMgY2hlY2sgYXNzdW1wdGlvbnMgb2YgbW9kZWwKIyMjIyBDb29rJ3MgZGlzdGFuY2UKYGBge3IgSUk2fQpwbG90KG0sIHdoaWNoID0gNCwgaWQubiA9IDMpCmBgYAoKIyMjIyBFeHRyYWN0IG1vZGVsIHJlc3VsdHMgYW5kIGRpc3BsYXkgZGF0YSBmb3IgdG9wIDMgdmFsdWVzIHVzaW5nIENvb2sncyBkaXN0YW5jZQpgYGB7ciBJSTd9Cm1vZGVsLmRhdGEgPC0gYXVnbWVudChtKSAlPiUgCiAgbXV0YXRlKGluZGV4ID0gMTpuKCkpIAptb2RlbC5kYXRhICU+JSB0b3BfbigzLCAuY29va3NkKQpgYGAKCiMjIyMgcGxvdCBzdGFuZGFyZGlzZWQgcmVzaWR1YWxzCmBgYHtyIElJOH0KZ2dwbG90KG1vZGVsLmRhdGEsIGFlcyhpbmRleCwgLnN0ZC5yZXNpZCkpICsgCiAgZ2VvbV9wb2ludChhZXMoY29sb3IgPSBIZWFsdGhfQmluYXJ5KSwgYWxwaGEgPSAuNSkgKwogIHRoZW1lX2J3KCkKYGBgCgojIyMjIEZpbHRlciBwb3RlbnRpYWwgaW5mbHVlbnRpYWwgZGF0YSBwb2ludHMgd2l0aCBhYnMoLnN0ZC5yZXMpID4gMzoKYGBge3IgSUk5fQptb2RlbC5kYXRhICU+JSAKICBmaWx0ZXIoYWJzKC5zdGQucmVzaWQpID4gMykKYGBgCgojIyMjIGNoZWNrIGZvciBtdWx0aWNvbGxpbmVhcml0eQpgYGB7ciBJSTEwfQpjYXI6OnZpZihtKQpgYGAKCgoKIyMgRF9TRVggKiBEX05FVVRFUiBCaW5hcnkgbG9naXN0aWMgcmVncmVzc2lvbiBPTiBTSUdOSUZJQ0FOVCBPUiBTRVJJT1VTIElMTE5FU1MKYGBge3IgSUkxMX0KIyBmaXQgYmluYXJ5IGxvZ2l0IG1vZGVsIGFuZCBzdG9yZSByZXN1bHRzICdtJwptIDwtIGdsbShIZWFsdGhfQmluYXJ5IH4gRF9TZXgqRF9OZXV0ZXIsIGRhdGEgPSBtbCxmYW1pbHkgPSBiaW5vbWlhbCkKIyB2aWV3IGEgc3VtbWFyeSBvZiB0aGUgbW9kZWwKc3VtbWFyeShtKQojIHRlc3QgbW9kZWwgZml0CndpdGgobSwgbnVsbC5kZXZpYW5jZSAtIGRldmlhbmNlKQp3aXRoKG0sIGRmLm51bGwgLSBkZi5yZXNpZHVhbCkKd2l0aChtLCBwY2hpc3EobnVsbC5kZXZpYW5jZSAtIGRldmlhbmNlLCBkZi5udWxsIC0gZGYucmVzaWR1YWwsIGxvd2VyLnRhaWwgPSBGQUxTRSkpCkJJQyhtKQojIEhvc21lci1MZW1lc2hvdyBHb29kbmVzcy1vZi1GaXQgVGVzdApobHRlc3QobSkKIyMgQ0lzIHVzaW5nIHByb2ZpbGVkIGxvZy1saWtlbGlob29kCmNvbmZpbnQobSwgbGV2ZWw9MC45OSkKIyMgQ0lzIHVzaW5nIHN0YW5kYXJkIGVycm9ycwpjb25maW50LmRlZmF1bHQobSwgbGV2ZWw9MC45OSkKIyBXYWxkIHRlc3QKd2FsZC50ZXN0KGIgPSBjb2VmKG0pLCBTaWdtYSA9IHZjb3YobSksIFRlcm1zID0gMikKIyMgb2RkcyByYXRpb3MgYW5kIDk1JSBDSQpleHAoY2JpbmQoT1IgPSBjb2VmKG0pLCBjb25maW50KG0sIGxldmVsPTAuOTkpKSkKYGBgCgojIyMgQ3JlYXRlIFJPQ1IgZnJvbSBkYXRhCmBgYHtyIFJPQzM4fQojIyB0cmFpbmluZyBkYXRhCnByZWQubXR0ID0gcHJlZGljdChtLCB0eXBlID0gInJlc3BvbnNlIikgI3JlcGVhdCByaXNrIHByZWRpY3Rpb25zIGZyb20gbW9kZWwgbQpyb2NyLnByZWQubXR0ID0gUk9DUjo6cHJlZGljdGlvbihwcmVkLm10dCwgbGFiZWxzID0gbWwkSGVhbHRoX0JpbmFyeSkgI1JPQ1IgcHJlZGljdGlvbiBvYmplY3QKcm9jLnBlcmYubXR0ID0gUk9DUjo6cGVyZm9ybWFuY2Uocm9jci5wcmVkLm10dCwgbWVhc3VyZSA9ICJ0cHIiLCB4Lm1lYXN1cmUgPSAiZnByIikgIyAjUk9DUiBwZXJmb3JtYW5jZSBvYmplY3QKcGxvdChyb2MucGVyZi5tdHQsIGNvbCA9ICJibHVlIikKYWJsaW5lKGEgPSAwLCBiID0gMSwgbHR5ID0gMikgI2RpYWdvbmFsIGZvciByYW5kb20gYXNzaWdubWVudApgYGAKCiMjIyBSZXBvcnQgQVVDIGZyb20gUk9DIGZvciB0cmFpbmluZyBhbmQgdGVzdCBkYXRhCmBgYHtyIEFVQzM4fQogICMgVHJhaW4gQVVDCmF1YyA8LSBST0NSOjpwZXJmb3JtYW5jZShyb2NyLnByZWQubXR0LCBtZWFzdXJlID0gImF1YyIpCiAgYXVjIDwtIGF1Y0B5LnZhbHVlc1tbMV1dCiAgcHJpbnQoYXVjKQpgYGAKCgojIyMgQ2FsY3VsYXRlIE5hZ2Vsa2Vya2UgUl4yCmBgYHtyIElJMTFhfQpOYWdlbGtlcmtlUjIobSkKYGBgCgojIyMgY2hlY2sgYXNzdW1wdGlvbnMgb2YgbW9kZWwKIyMjIyBDb29rJ3MgZGlzdGFuY2UKYGBge3IgSUkxMn0KcGxvdChtLCB3aGljaCA9IDQsIGlkLm4gPSAzKQpgYGAKCiMjIyMgRXh0cmFjdCBtb2RlbCByZXN1bHRzIGFuZCBkaXNwbGF5IGRhdGEgZm9yIHRvcCAzIHZhbHVlcyB1c2luZyBDb29rJ3MgZGlzdGFuY2UKYGBge3IgSUkxM30KbW9kZWwuZGF0YSA8LSBhdWdtZW50KG0pICU+JSAKICBtdXRhdGUoaW5kZXggPSAxOm4oKSkgCm1vZGVsLmRhdGEgJT4lIHRvcF9uKDMsIC5jb29rc2QpCmBgYAoKIyBwbG90IHN0YW5kYXJkaXNlZCByZXNpZHVhbHMKYGBge3IgSUkxNH0KZ2dwbG90KG1vZGVsLmRhdGEsIGFlcyhpbmRleCwgLnN0ZC5yZXNpZCkpICsgCiAgZ2VvbV9wb2ludChhZXMoY29sb3IgPSBIZWFsdGhfQmluYXJ5KSwgYWxwaGEgPSAuNSkgKwogIHRoZW1lX2J3KCkKYGBgCgojIEZpbHRlciBwb3RlbnRpYWwgaW5mbHVlbnRpYWwgZGF0YSBwb2ludHMgd2l0aCBhYnMoLnN0ZC5yZXMpID4gMzoKYGBge3IgSUkxNX0KbW9kZWwuZGF0YSAlPiUgCiAgZmlsdGVyKGFicyguc3RkLnJlc2lkKSA+IDMpCmBgYAoKIyMjIyBjaGVjayBmb3IgbXVsdGljb2xsaW5lYXJpdHkKYGBge3IgSUkxNn0KY2FyOjp2aWYobSkKYGBgCgoKCgojIENIRUNLIEVGRkVDVCBPRiBET0cgSEVBTFRIIENIQVJBQ1RFUklTVElDUyBPTiBTSUdOSUZJQ0FOVCBPUiBTRVJJT1VTIElMTE5FU1MgLSBzaW1wbGUgYmluYXJ5IHJlZ3Jlc3Npb24KCiMjIFRIRVJBUEVVVElDIEZPT0QgQmluYXJ5IGxvZ2lzdGljIHJlZ3Jlc3Npb24gT04gU0lHTklGSUNBTlQgT1IgU0VSSU9VUyBJTExORVNTCmBgYHtyIElJMjd9CiMgZml0IGJpbmFyeSBsb2dpdCBtb2RlbCBhbmQgc3RvcmUgcmVzdWx0cyAnbScKbSA8LSBnbG0oSGVhbHRoX0JpbmFyeSB+IFRoZXJhcGV1dGljX0Zvb2QsIGRhdGEgPSBtbCxmYW1pbHkgPSBiaW5vbWlhbCkKIyB2aWV3IGEgc3VtbWFyeSBvZiB0aGUgbW9kZWwKc3VtbWFyeShtKQojIHRlc3QgbW9kZWwgZml0CndpdGgobSwgbnVsbC5kZXZpYW5jZSAtIGRldmlhbmNlKQp3aXRoKG0sIGRmLm51bGwgLSBkZi5yZXNpZHVhbCkKd2l0aChtLCBwY2hpc3EobnVsbC5kZXZpYW5jZSAtIGRldmlhbmNlLCBkZi5udWxsIC0gZGYucmVzaWR1YWwsIGxvd2VyLnRhaWwgPSBGQUxTRSkpCkJJQyhtKQoKIyMgQ0lzIHVzaW5nIHByb2ZpbGVkIGxvZy1saWtlbGlob29kCmNvbmZpbnQobSwgbGV2ZWw9MC45OSkKIyMgQ0lzIHVzaW5nIHN0YW5kYXJkIGVycm9ycwpjb25maW50LmRlZmF1bHQobSwgbGV2ZWw9MC45OSkKIyBXYWxkIHRlc3QKd2FsZC50ZXN0KGIgPSBjb2VmKG0pLCBTaWdtYSA9IHZjb3YobSksIFRlcm1zID0gMikKIyMgb2RkcyByYXRpb3MgYW5kIDk1JSBDSQpleHAoY2JpbmQoT1IgPSBjb2VmKG0pLCBjb25maW50KG0sIGxldmVsPTAuOTkpKSkKYGBgCgojIyMgQ3JlYXRlIFJPQ1IgZnJvbSBkYXRhCmBgYHtyIFJPQzM5fQojIyB0cmFpbmluZyBkYXRhCnByZWQubXR0ID0gcHJlZGljdChtLCB0eXBlID0gInJlc3BvbnNlIikgI3JlcGVhdCByaXNrIHByZWRpY3Rpb25zIGZyb20gbW9kZWwgbQpyb2NyLnByZWQubXR0ID0gUk9DUjo6cHJlZGljdGlvbihwcmVkLm10dCwgbGFiZWxzID0gbWwkSGVhbHRoX0JpbmFyeSkgI1JPQ1IgcHJlZGljdGlvbiBvYmplY3QKcm9jLnBlcmYubXR0ID0gUk9DUjo6cGVyZm9ybWFuY2Uocm9jci5wcmVkLm10dCwgbWVhc3VyZSA9ICJ0cHIiLCB4Lm1lYXN1cmUgPSAiZnByIikgIyAjUk9DUiBwZXJmb3JtYW5jZSBvYmplY3QKcGxvdChyb2MucGVyZi5tdHQsIGNvbCA9ICJibHVlIikKYWJsaW5lKGEgPSAwLCBiID0gMSwgbHR5ID0gMikgI2RpYWdvbmFsIGZvciByYW5kb20gYXNzaWdubWVudApgYGAKCiMjIyBSZXBvcnQgQVVDIGZyb20gUk9DIGZvciB0cmFpbmluZyBhbmQgdGVzdCBkYXRhCmBgYHtyIEFVQzM5fQogICMgVHJhaW4gQVVDCmF1YyA8LSBST0NSOjpwZXJmb3JtYW5jZShyb2NyLnByZWQubXR0LCBtZWFzdXJlID0gImF1YyIpCiAgYXVjIDwtIGF1Y0B5LnZhbHVlc1tbMV1dCiAgcHJpbnQoYXVjKQpgYGAKCgojIyMgQ2FsY3VsYXRlIE5hZ2Vsa2Vya2UgUl4yCmBgYHtyIElJMjdhfQpOYWdlbGtlcmtlUjIobSkKYGBgCgojIyMgY2hlY2sgYXNzdW1wdGlvbnMgb2YgbW9kZWwKIyMjIyBDb29rJ3MgZGlzdGFuY2UKYGBge3IgSUkyOH0KcGxvdChtLCB3aGljaCA9IDQsIGlkLm4gPSAzKQpgYGAKCiMjIyMgRXh0cmFjdCBtb2RlbCByZXN1bHRzIGFuZCBkaXNwbGF5IGRhdGEgZm9yIHRvcCAzIHZhbHVlcyB1c2luZyBDb29rJ3MgZGlzdGFuY2UKYGBge3IgSUkyOX0KbW9kZWwuZGF0YSA8LSBhdWdtZW50KG0pICU+JSAKICBtdXRhdGUoaW5kZXggPSAxOm4oKSkgCm1vZGVsLmRhdGEgJT4lIHRvcF9uKDMsIC5jb29rc2QpCmBgYAoKIyMjIyBwbG90IHN0YW5kYXJkaXNlZCByZXNpZHVhbHMKYGBge3IgSUkzMH0KZ2dwbG90KG1vZGVsLmRhdGEsIGFlcyhpbmRleCwgLnN0ZC5yZXNpZCkpICsgCiAgZ2VvbV9wb2ludChhZXMoY29sb3IgPSBIZWFsdGhfQmluYXJ5KSwgYWxwaGEgPSAuNSkgKwogIHRoZW1lX2J3KCkKYGBgCgojIyMjIEZpbHRlciBwb3RlbnRpYWwgaW5mbHVlbnRpYWwgZGF0YSBwb2ludHMgd2l0aCBhYnMoLnN0ZC5yZXMpID4gMzoKYGBge3IgSUkzMX0KbW9kZWwuZGF0YSAlPiUgCiAgZmlsdGVyKGFicyguc3RkLnJlc2lkKSA+IDMpCmBgYAoKCgojIyBWSVNJVFMgQmluYXJ5IGxvZ2lzdGljIHJlZ3Jlc3Npb24gT04gU0lHTklGSUNBTlQgT1IgU0VSSU9VUyBJTExORVNTCmBgYHtyIElJMzJ9CiMgZml0IGJpbmFyeSBsb2dpdCBtb2RlbCBhbmQgc3RvcmUgcmVzdWx0cyAnbScKbSA8LSBnbG0oSGVhbHRoX0JpbmFyeSB+IFZpc2l0cywgZGF0YSA9IG1sLGZhbWlseSA9IGJpbm9taWFsKQojIHZpZXcgYSBzdW1tYXJ5IG9mIHRoZSBtb2RlbApzdW1tYXJ5KG0pCiMgdGVzdCBtb2RlbCBmaXQKd2l0aChtLCBudWxsLmRldmlhbmNlIC0gZGV2aWFuY2UpCndpdGgobSwgZGYubnVsbCAtIGRmLnJlc2lkdWFsKQp3aXRoKG0sIHBjaGlzcShudWxsLmRldmlhbmNlIC0gZGV2aWFuY2UsIGRmLm51bGwgLSBkZi5yZXNpZHVhbCwgbG93ZXIudGFpbCA9IEZBTFNFKSkKQklDKG0pCiMgSG9zbWVyLUxlbWVzaG93IEdvb2RuZXNzLW9mLUZpdCBUZXN0CmhsdGVzdChtKQojIyBDSXMgdXNpbmcgcHJvZmlsZWQgbG9nLWxpa2VsaWhvb2QKY29uZmludChtLCBsZXZlbD0wLjk5KQojIyBDSXMgdXNpbmcgc3RhbmRhcmQgZXJyb3JzCmNvbmZpbnQuZGVmYXVsdChtLCBsZXZlbD0wLjk5KQojIFdhbGQgdGVzdAp3YWxkLnRlc3QoYiA9IGNvZWYobSksIFNpZ21hID0gdmNvdihtKSwgVGVybXMgPSAyKQojIyBvZGRzIHJhdGlvcyBhbmQgOTUlIENJCmV4cChjYmluZChPUiA9IGNvZWYobSksIGNvbmZpbnQobSwgbGV2ZWw9MC45OSkpKQpgYGAKCiMjIyBDcmVhdGUgUk9DUiBmcm9tIGRhdGEKYGBge3IgUk9DNDB9CiMjIHRyYWluaW5nIGRhdGEKcHJlZC5tdHQgPSBwcmVkaWN0KG0sIHR5cGUgPSAicmVzcG9uc2UiKSAjcmVwZWF0IHJpc2sgcHJlZGljdGlvbnMgZnJvbSBtb2RlbCBtCnJvY3IucHJlZC5tdHQgPSBST0NSOjpwcmVkaWN0aW9uKHByZWQubXR0LCBsYWJlbHMgPSBtbCRIZWFsdGhfQmluYXJ5KSAjUk9DUiBwcmVkaWN0aW9uIG9iamVjdApyb2MucGVyZi5tdHQgPSBST0NSOjpwZXJmb3JtYW5jZShyb2NyLnByZWQubXR0LCBtZWFzdXJlID0gInRwciIsIHgubWVhc3VyZSA9ICJmcHIiKSAjICNST0NSIHBlcmZvcm1hbmNlIG9iamVjdApwbG90KHJvYy5wZXJmLm10dCwgY29sID0gImJsdWUiKQphYmxpbmUoYSA9IDAsIGIgPSAxLCBsdHkgPSAyKSAjZGlhZ29uYWwgZm9yIHJhbmRvbSBhc3NpZ25tZW50CmBgYAoKIyMjIFJlcG9ydCBBVUMgZnJvbSBST0MgZm9yIHRyYWluaW5nIGFuZCB0ZXN0IGRhdGEKYGBge3IgQVVDNDB9CiAgIyBUcmFpbiBBVUMKYXVjIDwtIFJPQ1I6OnBlcmZvcm1hbmNlKHJvY3IucHJlZC5tdHQsIG1lYXN1cmUgPSAiYXVjIikKICBhdWMgPC0gYXVjQHkudmFsdWVzW1sxXV0KICBwcmludChhdWMpCmBgYAoKCiMjIyBDYWxjdWxhdGUgTmFnZWxrZXJrZSBSXjIKYGBge3IgSUkzMmF9Ck5hZ2Vsa2Vya2VSMihtKQpgYGAKCiMjIyBjaGVjayBhc3N1bXB0aW9ucyBvZiBtb2RlbAojIyMjIENvb2sncyBkaXN0YW5jZQpgYGB7ciBJSTMzfQpwbG90KG0sIHdoaWNoID0gNCwgaWQubiA9IDMpCmBgYAoKIyMjIyBFeHRyYWN0IG1vZGVsIHJlc3VsdHMgYW5kIGRpc3BsYXkgZGF0YSBmb3IgdG9wIDMgdmFsdWVzIHVzaW5nIENvb2sncyBkaXN0YW5jZQpgYGB7ciBJSTM0fQptb2RlbC5kYXRhIDwtIGF1Z21lbnQobSkgJT4lIAogIG11dGF0ZShpbmRleCA9IDE6bigpKSAKbW9kZWwuZGF0YSAlPiUgdG9wX24oMywgLmNvb2tzZCkKYGBgCgojIyMjIHBsb3Qgc3RhbmRhcmRpc2VkIHJlc2lkdWFscwpgYGB7ciBJSTM1fQpnZ3Bsb3QobW9kZWwuZGF0YSwgYWVzKGluZGV4LCAuc3RkLnJlc2lkKSkgKyAKICBnZW9tX3BvaW50KGFlcyhjb2xvciA9IEhlYWx0aF9CaW5hcnkpLCBhbHBoYSA9IC41KSArCiAgdGhlbWVfYncoKQpgYGAKCiMjIyMgRmlsdGVyIHBvdGVudGlhbCBpbmZsdWVudGlhbCBkYXRhIHBvaW50cyB3aXRoIGFicyguc3RkLnJlcykgPiAzOgpgYGB7ciBJSTM2fQptb2RlbC5kYXRhICU+JSAKICBmaWx0ZXIoYWJzKC5zdGQucmVzaWQpID4gMykKYGBgCgoKCiMjIFZJU0lUUzIgQmluYXJ5IGxvZ2lzdGljIHJlZ3Jlc3Npb24gZm9yIEhFQUxUSAojIyMjIE5vdGUgVmlzaXRzIGJldHRlciBmaXQgdGhhbiBWaXNpdHMyCmBgYHtyIElJMzd9CiMgZml0IGJpbmFyeSBsb2dpdCBtb2RlbCBhbmQgc3RvcmUgcmVzdWx0cyAnbScKbSA8LSBnbG0oSGVhbHRoX0JpbmFyeSB+IFZpc2l0czIsIGRhdGEgPSBtbCxmYW1pbHkgPSBiaW5vbWlhbCkKIyB2aWV3IGEgc3VtbWFyeSBvZiB0aGUgbW9kZWwKc3VtbWFyeShtKQojIHRlc3QgbW9kZWwgZml0CndpdGgobSwgbnVsbC5kZXZpYW5jZSAtIGRldmlhbmNlKQp3aXRoKG0sIGRmLm51bGwgLSBkZi5yZXNpZHVhbCkKd2l0aChtLCBwY2hpc3EobnVsbC5kZXZpYW5jZSAtIGRldmlhbmNlLCBkZi5udWxsIC0gZGYucmVzaWR1YWwsIGxvd2VyLnRhaWwgPSBGQUxTRSkpCkJJQyhtKQojIEhvc21lci1MZW1lc2hvdyBHb29kbmVzcy1vZi1GaXQgVGVzdApobHRlc3QobSkKIyMgQ0lzIHVzaW5nIHByb2ZpbGVkIGxvZy1saWtlbGlob29kCmNvbmZpbnQobSwgbGV2ZWw9MC45OSkKIyMgQ0lzIHVzaW5nIHN0YW5kYXJkIGVycm9ycwpjb25maW50LmRlZmF1bHQobSwgbGV2ZWw9MC45OSkKIyBXYWxkIHRlc3QKd2FsZC50ZXN0KGIgPSBjb2VmKG0pLCBTaWdtYSA9IHZjb3YobSksIFRlcm1zID0gMikKIyMgb2RkcyByYXRpb3MgYW5kIDk1JSBDSQpleHAoY2JpbmQoT1IgPSBjb2VmKG0pLCBjb25maW50KG0sIGxldmVsPTAuOTkpKSkKYGBgCgojIyMgQ3JlYXRlIFJPQ1IgZnJvbSBkYXRhCmBgYHtyIFJPQzQxfQojIyB0cmFpbmluZyBkYXRhCnByZWQubXR0ID0gcHJlZGljdChtLCB0eXBlID0gInJlc3BvbnNlIikgI3JlcGVhdCByaXNrIHByZWRpY3Rpb25zIGZyb20gbW9kZWwgbQpyb2NyLnByZWQubXR0ID0gUk9DUjo6cHJlZGljdGlvbihwcmVkLm10dCwgbGFiZWxzID0gbWwkSGVhbHRoX0JpbmFyeSkgI1JPQ1IgcHJlZGljdGlvbiBvYmplY3QKcm9jLnBlcmYubXR0ID0gUk9DUjo6cGVyZm9ybWFuY2Uocm9jci5wcmVkLm10dCwgbWVhc3VyZSA9ICJ0cHIiLCB4Lm1lYXN1cmUgPSAiZnByIikgIyAjUk9DUiBwZXJmb3JtYW5jZSBvYmplY3QKcGxvdChyb2MucGVyZi5tdHQsIGNvbCA9ICJibHVlIikKYWJsaW5lKGEgPSAwLCBiID0gMSwgbHR5ID0gMikgI2RpYWdvbmFsIGZvciByYW5kb20gYXNzaWdubWVudApgYGAKCiMjIyBSZXBvcnQgQVVDIGZyb20gUk9DIGZvciB0cmFpbmluZyBhbmQgdGVzdCBkYXRhCmBgYHtyIEFVQzQxfQogICMgVHJhaW4gQVVDCmF1YyA8LSBST0NSOjpwZXJmb3JtYW5jZShyb2NyLnByZWQubXR0LCBtZWFzdXJlID0gImF1YyIpCiAgYXVjIDwtIGF1Y0B5LnZhbHVlc1tbMV1dCiAgcHJpbnQoYXVjKQpgYGAKCgojIyMgQ2FsY3VsYXRlIE5hZ2Vsa2Vya2UgUl4yCmBgYHtyIElJMzdhfQpOYWdlbGtlcmtlUjIobSkKYGBgCgojIyMgY2hlY2sgYXNzdW1wdGlvbnMgb2YgbW9kZWwKIyMjIyBDb29rJ3MgZGlzdGFuY2UKYGBge3IgSUkzN2FhfQpwbG90KG0sIHdoaWNoID0gNCwgaWQubiA9IDMpCmBgYAoKIyMjIyBFeHRyYWN0IG1vZGVsIHJlc3VsdHMgYW5kIGRpc3BsYXkgZGF0YSBmb3IgdG9wIDMgdmFsdWVzIHVzaW5nIENvb2sncyBkaXN0YW5jZQpgYGB7ciBJSTM4fQptb2RlbC5kYXRhIDwtIGF1Z21lbnQobSkgJT4lIAogIG11dGF0ZShpbmRleCA9IDE6bigpKSAKbW9kZWwuZGF0YSAlPiUgdG9wX24oMywgLmNvb2tzZCkKYGBgCgojIyMjIHBsb3Qgc3RhbmRhcmRpc2VkIHJlc2lkdWFscwpgYGB7ciBJSTM5fQpnZ3Bsb3QobW9kZWwuZGF0YSwgYWVzKGluZGV4LCAuc3RkLnJlc2lkKSkgKyAKICBnZW9tX3BvaW50KGFlcyhjb2xvciA9IEhlYWx0aF9CaW5hcnkpLCBhbHBoYSA9IC41KSArCiAgdGhlbWVfYncoKQpgYGAKCiMjIyMgRmlsdGVyIHBvdGVudGlhbCBpbmZsdWVudGlhbCBkYXRhIHBvaW50cyB3aXRoIGFicyguc3RkLnJlcykgPiAzOgpgYGB7ciBJSTQwfQptb2RlbC5kYXRhICU+JSAKICBmaWx0ZXIoYWJzKC5zdGQucmVzaWQpID4gMykKYGBgCgoKCiMjIE1FRFMgQmluYXJ5IGxvZ2lzdGljIHJlZ3Jlc3Npb24gZm9yIEhFQUxUSApgYGB7ciBJSTQxfQojIGZpdCBiaW5hcnkgbG9naXQgbW9kZWwgYW5kIHN0b3JlIHJlc3VsdHMgJ20nCm0gPC0gZ2xtKEhlYWx0aF9CaW5hcnkgfiBNZWRzLCBkYXRhID0gbWwsZmFtaWx5ID0gYmlub21pYWwpCiMgdmlldyBhIHN1bW1hcnkgb2YgdGhlIG1vZGVsCnN1bW1hcnkobSkKIyB0ZXN0IG1vZGVsIGZpdAp3aXRoKG0sIG51bGwuZGV2aWFuY2UgLSBkZXZpYW5jZSkKd2l0aChtLCBkZi5udWxsIC0gZGYucmVzaWR1YWwpCndpdGgobSwgcGNoaXNxKG51bGwuZGV2aWFuY2UgLSBkZXZpYW5jZSwgZGYubnVsbCAtIGRmLnJlc2lkdWFsLCBsb3dlci50YWlsID0gRkFMU0UpKQpCSUMobSkKIyBIb3NtZXItTGVtZXNob3cgR29vZG5lc3Mtb2YtRml0IFRlc3QKCiMjIENJcyB1c2luZyBwcm9maWxlZCBsb2ctbGlrZWxpaG9vZApjb25maW50KG0sIGxldmVsPTAuOTkpCiMjIENJcyB1c2luZyBzdGFuZGFyZCBlcnJvcnMKY29uZmludC5kZWZhdWx0KG0sIGxldmVsPTAuOTkpCiMgV2FsZCB0ZXN0CndhbGQudGVzdChiID0gY29lZihtKSwgU2lnbWEgPSB2Y292KG0pLCBUZXJtcyA9IDIpCiMjIG9kZHMgcmF0aW9zIGFuZCA5NSUgQ0kKZXhwKGNiaW5kKE9SID0gY29lZihtKSwgY29uZmludChtLCBsZXZlbD0wLjk5KSkpCmBgYAoKIyMjIENyZWF0ZSBST0NSIGZyb20gZGF0YQpgYGB7ciBST0M0Mn0KIyMgdHJhaW5pbmcgZGF0YQpwcmVkLm10dCA9IHByZWRpY3QobSwgdHlwZSA9ICJyZXNwb25zZSIpICNyZXBlYXQgcmlzayBwcmVkaWN0aW9ucyBmcm9tIG1vZGVsIG0Kcm9jci5wcmVkLm10dCA9IFJPQ1I6OnByZWRpY3Rpb24ocHJlZC5tdHQsIGxhYmVscyA9IG1sJEhlYWx0aF9CaW5hcnkpICNST0NSIHByZWRpY3Rpb24gb2JqZWN0CnJvYy5wZXJmLm10dCA9IFJPQ1I6OnBlcmZvcm1hbmNlKHJvY3IucHJlZC5tdHQsIG1lYXN1cmUgPSAidHByIiwgeC5tZWFzdXJlID0gImZwciIpICMgI1JPQ1IgcGVyZm9ybWFuY2Ugb2JqZWN0CnBsb3Qocm9jLnBlcmYubXR0LCBjb2wgPSAiYmx1ZSIpCmFibGluZShhID0gMCwgYiA9IDEsIGx0eSA9IDIpICNkaWFnb25hbCBmb3IgcmFuZG9tIGFzc2lnbm1lbnQKYGBgCgojIyMgUmVwb3J0IEFVQyBmcm9tIFJPQyBmb3IgdHJhaW5pbmcgYW5kIHRlc3QgZGF0YQpgYGB7ciBBVUM0Mn0KICAjIFRyYWluIEFVQwphdWMgPC0gUk9DUjo6cGVyZm9ybWFuY2Uocm9jci5wcmVkLm10dCwgbWVhc3VyZSA9ICJhdWMiKQogIGF1YyA8LSBhdWNAeS52YWx1ZXNbWzFdXQogIHByaW50KGF1YykKYGBgCgoKIyMjIENhbGN1bGF0ZSBOYWdlbGtlcmtlIFJeMgpgYGB7ciBJSTQxYX0KTmFnZWxrZXJrZVIyKG0pCmBgYAoKIyMjIGNoZWNrIGFzc3VtcHRpb25zIG9mIG1vZGVsCiMjIyMgQ29vaydzIGRpc3RhbmNlCmBgYHtyIElJNDJ9CnBsb3QobSwgd2hpY2ggPSA0LCBpZC5uID0gMykKYGBgCgojIyMjIEV4dHJhY3QgbW9kZWwgcmVzdWx0cyBhbmQgZGlzcGxheSBkYXRhIGZvciB0b3AgMyB2YWx1ZXMgdXNpbmcgQ29vaydzIGRpc3RhbmNlCmBgYHtyIElJNDN9Cm1vZGVsLmRhdGEgPC0gYXVnbWVudChtKSAlPiUgCiAgbXV0YXRlKGluZGV4ID0gMTpuKCkpIAptb2RlbC5kYXRhICU+JSB0b3BfbigzLCAuY29va3NkKQpgYGAKCiMjIyMgcGxvdCBzdGFuZGFyZGlzZWQgcmVzaWR1YWxzCmBgYHtyIElJNDR9CmdncGxvdChtb2RlbC5kYXRhLCBhZXMoaW5kZXgsIC5zdGQucmVzaWQpKSArIAogIGdlb21fcG9pbnQoYWVzKGNvbG9yID0gSGVhbHRoX0JpbmFyeSksIGFscGhhID0gLjUpICsKICB0aGVtZV9idygpCmBgYAoKIyMjIyBGaWx0ZXIgcG90ZW50aWFsIGluZmx1ZW50aWFsIGRhdGEgcG9pbnRzIHdpdGggYWJzKC5zdGQucmVzKSA+IDM6CmBgYHtyIElJNDV9Cm1vZGVsLmRhdGEgJT4lIAogIGZpbHRlcihhYnMoLnN0ZC5yZXNpZCkgPiAzKQpgYGAKCgoKCgoKCgoKCgoKCgoK
