# Supplementary material for: Variables associated with owner perceptions of the health of their dog: Further analysis of data from a large international survey
Source: PLoS One. 2024 May 15;19(5):e0280173. doi: 10.1371/journal.pone.0280173 (PMC11095744; doi:10.1371/journal.pone.0280173)
Supplement: Attachment — Submitted filename: Response to Reviewers.pdf [file pone.0280173.s026.pdf]

Response to reviewer comments

PONE-D-22-34984

Variables associated with owner perceptions of the health of their dog: further analysis of data from a large international survey

Barrett-Jolly and German

We would like to thank the editor and both reviewers for their time in reviewing our manuscript and for the constructive comments that have been made. Below, we have addressed all points highlighted and, where relevant, have also indicated how the manuscript has been revised as a result. Given that the responses are detailed, we have also created a table summarising how each of the points has been addressed.

We hope that these explanations are useful to the editor.

Summary of point-by-point responses

Editor / reviewer comment	Author response	Changes made
Editorial comments		
1. Ensure style guide is met.	Minor changes to style made.	Manuscript updated to ensure compliance with the PLOS ONE style.
2. Correct *competing interests* section.	Sentence added to the *competing interests* section.	Manuscript updated to include the required additional sentence.
Reviewer 1		
1. No changes requested.	Acknowledged and thanked reviewer for their detailed review	No changes needed.
Reviewer 2		
	Acknowledged and thanked reviewer for their detailed review	
1. Authors did not consult with the authors of the original study (Knight et al., 2022).	Acknowledged this as a limitation, but explained the reason why it was not done:<br>• Study information data from Knight et al. (2022) was clear so not necessary.<br>• preferable that scientific replication is independent of authors of the original study.	Discussion updated to add this limitation, as well as explaining and justifying the approach taken.
2. Results sometimes reinforce those of Knight et al. (2022); differences might be due to different parameter choice and statistical methods.	• Agree that results of preliminary, simple (i.e., univariable) regression are similar to results from Knight et al. (2022).<br>• This is expected because both used univariable tests and similar data.<br>• The fact that these results are similar suggests that the dataset for the current study was representative of the original dataset.<br>• However, significant diet effects were NOT seen with multiple regression or XGBoost, when other variables were accounted for. It is these findings that are most important.	Discussion updated so similarities between Knight et results and simple regression are better emphasised.

3.	Dispute the conclusion that the effect of vegan diet was minimal.	• Reviewer has incorrectly assumed that Table 3 shows multiple regression; instead, these results are from simple regression.<br>• Clarified why we conclude that the vegan diet effect is minimal, because it explained **only 0.5%** of model variance and was not included in any final multiple regression models because model fit was not improved.	Amended titles and legends of Tables 2-4 to make it clear that these are simple regression results
4.	Only one health metric was studied; why not use veterinary assessments?	• Whilst only analysing owner-reported dog health metric is a possible limitation of the study, other metrics reported by Knight et al. (2022) were less reliable.<br>• The *veterinary assessment metric* was not obtained directly from the veterinarians and was uniquely flawed because it required the owner to speculate on the opinion of their vet.<br>• Data on the 22 health disorder metrics were also flawed because of lack of standardised definitions and small numbers (<50 dogs) in over half of the categories.<br>• Therefore, it was justifiable to focus on the owner-reported dog health metric.	Discussion updated to add this limitation, and to explain and justify approach taken.
5.	Exclusion of 111 owners that played either no role or a limited role in diet decisions.	• Agree with the reviewer that we had not properly explained and justified the reasons for excluding these 111 owners in our study.<br>• Decided to excluded these respondents because of concerns about the reliability of both the diet information and health data from these dogs, given the respondent's lesser (and often limited) involvement.<br>• Although this resulted in a small decrease in sample size, data loss was minimal (only ~5%). Therefore, any disadvantage in study power would be more than offset by the advantage of data reliability.	Discussion updated to add this limitation, and also to explain and justify approach taken
6.	Combined dogs fed vegetarian and vegan diets in the vegan diet category.	• This point reflects a misunderstanding from the reviewer about how the dog diet was handled.<br>• Three diet variables were assessed separately in initial simple (univariable) regression: *dog diet* (conventional, raw, vegetarian, vegan as separate categories), a *vegan diet* binary and a *vegan-vegetarian* binary.	Methods section updated to clarify the statistical methods and explain how the *vegan diet* and *vegan-vegetarian diet* variables were handled.

		• The *vegan-vegetarian* variable was only used in initial simple regression but then excluded in favour of the *vegan diet* variable which performed better.<br>• Therefore, the comment made by the reviewer is **factually incorrect**.<br>• Nonetheless, we acknowledge this could be more clearly articulated in the statistical methods of the original manuscript.	
7.	Removing or combining categories with small numbers can risk losing finer discrimination within data.	• The reviewer is correct that removing or combining categories makes it more challenging to assess effect in these groups statistically.<br>• Explained why this approach was necessary: to ensure that datasets were balanced to ensure our modelling was valid.<br>• Emphasised that we grouped many variables in different ways to ensure that we optimised model fit. For example, we compared different groupings (4 vs 5 categories for vet visits; vegan vs vegan-vegetarian (as above) and age (as continuous vs ordinal categories).<br>• We then chose the coding method that led to best model fit, ensuring that the grouping / removal of categories with small numbers was justified.	Discussion updated to add this limitation, and to explain and justify approach taken.
8.	Several analysis steps were statistically flawed including identical inclusion of the same data in different predictor variables leading to issues with multicollinearity.	This point is **factually incorrect** for the following reasons:<br>• Predictor variables that used the same data were **not** included in the same multiple regression models.<br>• As explained above, we tested different versions of the same predictor variable in different ways (e.g., continuous vs. ordinal; number of categories etc), and selected the version that best fitted the data, before using this in any further analyses.<br>• As described in the original manuscript, multicollinearity **was** examined in the our study using variance inflation factors VIFs).<br>• Based on these analyses, multicollinearity was never an issue in any of the multiple regression model reported.<br>• We have re-read the text included in the original manuscript about multicollinearity in our original methods and believe it to be explicit and clear.	No changes necessary as multicollinearity had already been adequately addressed in the original manuscript.

9.	Including both veterinary visits and receiving medication in models invalidated them.	• This point is effectively the same criticism as the previous one (point 8) and is **factually incorrect** for the same reasons.<br>• Most notability, possible multicollinearity was assessed using VIFs and was **not** an issue in any models containing both these predictor variables.	No changes necessary as multicollinearity had already been adequately addressed in the original manuscript.
10.	Unfair criticism of the sampling method used in the Knight et al. (2022).	• Acknowledge the reviewer's criticism and accept that the discussion in their original manuscript mainly focused on limitations of the sampling method used by Knight et al. (2022), and did not adequately explain their reasons, leading to an unbalanced discussion.	Discussion expanded to emphasise both the reasons and benefits of the sampling approach by Knight et al (2022), in addition to the limitations.
11.	Difference in approach between current study and that of Knight et al (2022); the aim of that study was to explore the effect of diet, whereas the current study also assessed owner demographic variables.	• This point is more of a justification of the approach taken by Knight et al. (2022), rather than a criticism of the current work.<br>• We agree that the Knight et al (2022) only focused on diet but emphasised that this was a key reason for undertaking the current study, namely that other variables had not been adequately assessed by Knight et al.<br>• Justified the inclusion of owner variables using previous studies which have demonstrated associations between such owner variables and either diet choice or dog health.<br>• Concluded that studying these additional variables in the current study was valid.	Discussion updated (including adding 2 new references) better to explain and justify the reasons for including owner variables in the modelling.
12.	The discussion of the limitation of using owner opinions overlapped with the discussion of the Knight et al study.	• Agreed with the reviewer that the Knight et al (2022) study also discussed the limitation of using owner reports to assess dog health.<br>• Explained that this was necessary because the same data were used in both studies and, therefore, the limitation is pertinent to both.<br>• However, emphasised the text complemented rather than simply reiterated the previous discussion.	Discussion updated to emphasise better that the Knight et al (2022) study had also stated this limitation.
13.	Overall reviewer conclusion that statistical methods were flawed, data used did not accurately represent the	As summarised above, and discussed in detail below, the conclusions of the reviewers are disputed:<br>• Whilst, as expected, there is similarity between the simple regression and previous results of Knight et al (2022), the results of both multiple	See above and below for changes made.

original data and conclusions were unsupported.	regression and XGBoost suggest that diet type has a minimal effect on owner-reported dog health; therefore, conclusions ARE justified.<br>• The similarity between simple regression results and the Knight et al results confirms that the dataset was representative of the original dataset and refutes the reviewer's statement.<br>• The reviewer misunderstood the methods in the original version of our manuscript and, therefore, their claim that statistical methods were flawed is incorrect. For example, multicollinearity was already addressed in the original manuscript.<br>• Dogs on vegetarian diets were only grouped with the vegan diet dogs in the preliminary analyses of simple logistic regression; this vegan-vegetarian diet variable was then discarded because the vegan diet variable performed better.<br>• We dispute that it was incorrect to remove or combine categories containing small numbers; not only was this appropriate for the statistical methods used, but it improved model fit.<br>• We stand by the choice of outcome variable (owner-reported dog health), which was superior to other health metrics reported by Knight et al (2022), as discussed below.<br>• We thank Knight et al, once again, for promoting the open science principle, by making the original data from their study freely available.

Editorial comments

When submitting your revision, we need you to address these additional requirements:

1. Please ensure that your manuscript meets PLOS ONE's style requirements, including those for file naming. The PLOS ONE style templates can be found at https://journals.plos.org/plosone/s/file?id=wjVg/PLOSOne_formatting_sample_main_body.pdf and https://journals.plos.org/plosone/s/file?id=ba62/PLOSOne_formatting_sample_title_authors_affiliations.pdf

Authors' response:
We have rechecked the formatting of the manuscript and made some minor style changes (as indicated in the tracked changes version of the document. We are happy that the style is now compliant.

*2. Thank you for stating the following in the Competing Interests section:
"AJG is an employee of the University of Liverpool, but his position is financially-supported by Royal Canin. AJG has also received financial remuneration and gifts for providing educational material, speaking at conferences, and consultancy work, all unrelated to the current study. Royal Canin had no involvement in any aspect of the current work including study design, data analysis, drafting the manuscript or the decision to submit the work for publication.
RBJ is an employee of the University of Liverpool, whose salary is financially-supported by the Higher Education Funding Council for England. RBJ also receives a stipend from United Kingdom Research and Innovation for work as a panellist for the Biotechnology and Biological Sciences Research Council."*

Please confirm that this does not alter your adherence to all PLOS ONE policies on sharing data and materials, by including the following statement: ""This does not alter our adherence to PLOS ONE policies on sharing data and materials." (as detailed online in our guide for authors http://journals.plos.org/plosone/s/competing-interests). If there are restrictions on sharing of data and/or materials, please state these. Please note that we cannot proceed with consideration of your article until this information has been declared.

Please include your updated Competing Interests statement in your cover letter; we will change the online submission form on your behalf.

Authors' response:

We can confirm that none of these competing interests alters the authors' adherence to all PLOS ONE policies on sharing data and materials. We have updated the 'competing interests' section in the revised manuscript, and have also included the full competing interests statement in the cover letter.

Reviewer 1 comments

Happy to have reviewed the paper titled "variables associated with owner perceptions of the health of their dog: further analysis of data from a large international survey".
A timely and well-written paper with extensive analysis of data.
The authors have produced additional information on owner-reported dog health status and their diet using different statistical techniques to create models that best predicted owner opinions of health, and to identify the relative importance of the variables that contributed to the final model.

Authors' response:

Many thanks for reviewing our manuscript. We are pleased that you were happy with the data analysis and conclusions, as well as your opinion that this work provides additional information about variables associated with owner-reported health status.

Reviewer 2 comments

Reviewer comment 1

This authors of this current study responded to our prior Knight et al (2022) study (https://doi.org/10.1371/journal.pone.0265662), after reanalysing the dataset we collected, which is available open access. Unfortunately, within this current study there are substantial flaws in both the statistical analysis, and the representation of the results. It is unfortunate that the authors did not choose to consult with Knight et al. during design of their study, which would have allowed the following flaws to be identified in advance and avoided. Our Knight et al (2022) study was large and complex in some respects, and the authors are encouraged to do this, if planning any further reanalyses of our data.

Authors' response:

As stated in our manuscript, we would like to reiterate our gratitude to Knight et al., for making their original data available for independent analysis. This approach is consistent with the open science principle, which we welcome. We took a similar approach for the current study, not only concerning data access, but also by providing full details of our statistical analysis and code.

We note the reviewer's concerns about us not contacting Knight et al. before conducting our analysis. To the original authors' credit, Knight et al, (2022) study was well written, with clear study methodology and results. They also provided full details of the questionnaire used (including the exact wording of all questions), whilst the associated dataset was appropriately laid out, with clear labelling of all variables. As a result, it was not necessary for us to have to contact the original authors, either to request further information or to clarify ambiguities. Moreover, since independent replication is critical to the scientific method, it was arguably preferable for the primary study authors not to be involved in this further analysis. That said and, recognising the reviewer's concern, we believe it would be sensible to include this as a possible study limitation; therefore, the following text has been added to the discussion:

"A third limitation was the fact that we did not contact the authors of the original study before conducting our data analyses, to clarify any uncertainties with the dataset and seek guidance on our planned approach. This was not necessary because the original paper was well written, with clearly presented study methodology and results. Further, both the questionnaire and original dataset were also available, both of which were appropriately formatted with clear labelling of variables. This meant that nothing needed to be clarified prior to data analysis. Moreover, since independent replication is critical to the scientific method, it was arguably better for the primary study authors not to have been involved in, or provide guidance for, any such replication."

Reviewer comment 2

The authors of this current study concluded that diet has, at most, minimal association with owner-perceived canine health. They stated that these results conflicted with those of our previous Knight et al (2022) study. On closer examination, however, it becomes apparent that their results sometimes reinforce those of our previous study, and where they differ, this appears due to differences in parameters chosen for study, and the statistical methods used.

Authors' response:

We agree with the reviewer that some of the study findings are consistent with those of the Knight et al. (2022) study, most notably, the results of simple logistic regression, which we used to screen variables for inclusion in multiple logistic regression. This is not surprising and, indeed, is what was expected since these were univariable analyses, and those using dog diet variables were very similar to the tests performed in the previous study (odds ratios and ANOVA). However, differences emerged when we took the analysis further by using multiple logistic regression and machine learning predictive modelling using XGBoost. These models took other variables (beyond diet type) into account and, in so doing, associations with diet

were curtailed. Therefore, rather than conflicting with the previous findings, the current study should instead be viewed as an extension of the previous work, providing the reader with a greater understanding of the possible reasons for associations between diet variables and owner opinions dog health. This approach was briefly explained in the discussion from the original version of our manuscript. However, given the reviewer's concerns, we have rewritten the opening section of the discussion to explain better both the similarities and differences between the two studies:

"In the current study, we examined associations between owner perceptions of the health of their dog, and a range of owner-related, animal-related and healthcare variables. We utilised data from a previous study, which had used a questionnaire to gather the opinions of owners about the health of their dogs [15]. The primary aim of that study was to examine associations between owner opinions of dog health status and the type of diet that owners predominantly fed, with the key finding being that health was positively-associated with feeding either a vegan or raw diet, compared with other diet types (including conventional and vegetarian). **Given the focus of that study,** *other information gathered in the questionnaire was not assessed and no account was taken of possible confounding amongst variables.* **Therefore, to extend the findings of the previous work, additional animal, owner and healthcare variables were studied. Previous work has reported associations between such variables and owner decisions about feeding. For example, animal variables, such as age and neuter status, were associated with the owner feeding choice in the previous study of Knight et al. [15]. In the same study, maintenance of pet health was the most-common reason cited by owners for choosing a particular food [15] whilst, in other research, owner characteristics (such as geographic location) are also associated with food choice [64]. Finally, owner characteristics can also be associated with aspects of dog health; for example, both owner age and income are associated with the prevalence of obesity in dogs [65]. Considering these previous findings, our decision to examine variables beyond those of diet type was justified.**

Using both simple ordinal and simple binary logistic regression, we identified associations between owner-reported dog health and several owner (e.g., gender, diet), animal (e.g., age, breed, sex, neuter status) or healthcare (e.g., veterinary visits, prescribed medication by vet, switched to a therapeutic food) variables. Further, owner-reported dog health was associated with the dog diet variable, albeit not for the significant illness outcome variable. These findings are not surprising and, indeed would be expected, because they arose from univariable analyses (simple logistic regression) and, therefore, were broadly similar to the univariable analyses (e.g., odds ratios, one-way ANOVA) conducted in the previous study [15]. In the current study, and as described in the methods section, data pre-processing was necessary to ensure that our statistical analyses were valid. The approach taken was to remove or combine groups where numbers of dogs was small, to ensure better balance amongst categories within a predictor variable. The fact that the results obtained from our univariable analyses (simple logistic regression) were similar to those of Knight et al. [15], suggests that, despite this pre-processing, the final dataset remained representative of the dataset from which it was drawn.

Given that our simple logistic regressions analyses only considered the independent (i.e., uncorrected) effect of each predictor variable, *we* **next** *used a combination of* **multiple logistic regression and machine learning predictive modelling, so that** *multiple variables* **could be analysed** *concurrently*. **This enabled the creation of** *creating models that best predicted owner opinions of* **dog health**, *and to determine the relative importance of variables*

contributing to the final models. **We chose this combined approach** *to maximise the benefits of both approaches, whilst minimising disadvantages."*

[Please note that, since we have added two new references (64 and 65), other references have been renumbered.]

We believe that this expanded discussion better represents the valuable contribution of the Knight et al. study and highlights the similarity with our study whilst, at the same time, both justifying why our new study was necessary and highlighting where the studies differ.

Reviewer comment 3

Our previous study found statistically significant associations between diet and all seven general illness indicators studied. In every case the effect size was small. After controlling for numerous demographic factors and other variables via logistic regression analyses, the authors of the current study found the vegan diet lowered the risk of 'any health problem' by 43.2% (Table 3). Far from conflicting with our previous results, this key result appears to have strengthened our conclusion that the healthiest and least hazardous diet was nutritionally-sound vegan dog food. Hence the conclusion that only minimal benefits were associated with a vegan diet, is not supported by the study results. A 43.2% risk reduction is far from minimal.

Authors' response:
Thank you for this comment, to which we have the following responses:

- First, the reviewer should be aware that the results in Table 3 (as well as Tables 2 and 4) are the results of simple (univariable) logistic regression and not multiple logisitic regression. Here, each of the individual independent variables was tested separately in a regression model. Therefore, it is completely incorrect to state that these analyses "controlled [sic] for numerous demographic factors and other variables". Further, it would be expected that the results of such uncorrected analyses would parallel those reported by Knight et al. (2022), because their analyses (odds ratios, ANOVA etc) were also univariable and uncorrected and the same original data were used. As the reviewer will no doubt know, these simple regression analyses are solely used as a preliminary screening step ahead of the multiple regression modelling, where associations amongst predictor variables can be explored (i.e., "controlled for"); it is these latter results that are more meaningful and, therefore, the ones on which we base our conclusions.

  The multiple regression results that correspond to the simple regression in Table 3 are shown in Fig 4 with full numerical details given in S4 Table. Here, the best-fit model did not include vegan diet as a predictor variable because its inclusion did not improve the generalisability of the model. Therefore, in contrast to what the

reviewer suggests, these results **do not** strengthen the conclusions of Knight et al. (2022).

- Second, to assess the effect of a predictor variable in a regression model, it is necessary both to determine whether it independently improves the fit of the model, and also to determine the proportion of the variance that is explained by its inclusion. Although, on simple regression, the odds ratio for the *vegan diet* variable was 0.689 and this was statistically significant (*P*=0.006), the model fit was poor (based on the Bayesian information criterion, BIC) and pseudo R^2 was only 0.0049. This latter result suggests that only ~0.5% of the variance in the outcome variable is explained by the *vegan diet* variable and, therefore, we can conclude that whilst this might have statistical significance, its clinical significance is minimal.

  By way of comparison, the pseudo R^2 of the *dog age* variable from the same model was 0.0873 (~9% of variance explained), whilst that of the *prescribed medication by vet* was 0.3242 (~32% of variance explained); also, model fit for both variables was much better (*dog age:* BIC 2650; *prescribed medication by vet:* BIC 2346, vs. BIC 2940 for the *vegan diet* variable). Further, when multiple regression analysis was used, inclusion of the *vegan diet* variable led to **worse generalisability of the model, and this was why it was not included in the final best-fit model.** For comparison, the BIC of the best-fit model was 2058, and pseudo R^2 was 0.428.

  Moreover, using machine learning predictive modelling, the diet of the dog diet was low down in order of importance. Therefore, we were correct to conclude that our findings do indeed suggest that diet has only a minimal association with dog health.

- Third, the reviewer's interpretation of the results from both the Knight et al. (2022) study and the current work is incorrect. Specifically, it is wrong to conclude that vegan diets (or any other predictor variable, for that matter) "lowered the risk" of a dog having *'any health problem'*. The original survey was cross-sectional and observational in design and, therefore, correlations **do not** confirm causation. Therefore, the most we can ever say is that a predictor variable might be "associated with" the outcome variable, whilst making it clear that the reasons for any associations are not known. We are certain that the reviewer will agree with the scientific principle of not overstating the findings of scientific studies, not least when they are observational in nature, since they can mislead others and reduce credibility in research.

In conclusion, we disagree with the reviewer's comment, and stand by the conclusions of our study. Nonetheless, we have reviewed the discussion of our manuscript and accept that we did not emphasise enough the similarities between our simple (univariable) regression analyses and the findings reported in Knight et al. (2022). Therefore, as explained above for an earlier point, we have amended the opening paragraphs of the discussion to address this better.

As well as this, we believe that it would be sensible to ensure that readers understand that the results in Tables 2-4 are from simple LR, where each variable is tested separately. To clarify this, we have altered the title of Table 3 as follows:

"Results of simple **(ie univariable)** *binary logistic regression analyses…"*

And, for the table legend, we have now added the following explanation:

"Results presented are from simple (ie univariable) logistic regression, whereby each independent predictor variable is tested separately in a logistic regression model. These results were then used to determine the variables to include in subsequent multiple regression analysis, as shown in Fig 7 and S5 Table."

Similar wording has also been added to Tables 2 and 4. We hope that these changes will reduce the chances of confusion for readers.

Reviewer comment 4

Unlike our prior Knight et al (2022) study, which assessed seven general indicators of illness, and also considered the prevalence of 22 specific health disorders, the current study did not consider any specific health disorders, and it created and assessed only two general indicators of illness from the raw data. For one of them ('any health problem'), guardian opinions of health were used to create this indicator. However, guardian opinions of health status are normally less reliable than veterinary assessments. The indicator that should have been used, to achieve greater reliability, was reported veterinary assessment of being unwell.

Authors' response:
Thank you for this comment. We agree with the reviewer that there are limitations with relying on any health metrics utilising subjective owner opinions and recollections which can be inaccurate, misleading or biased. Whilst the reviewer is also correct that data from veterinary assessments would have been a better health metric to use, this was **not** what was reported in the Knight et al. (2022) study. In this respect, the *veterinary assessment of health* metric used in the previous was simply another owner opinion, as explained in the Methods section of the Knight et al. (2022) paper:

"They were asked to report their own opinion of their dog's health status, and also to report what they believed their veterinarian's assessment to be. Guardians were asked to "Think about your veterinarian. Which of the following would most likely describe their opinions about your animal's medical condition over the previous 12 months?"

This is surely an inferior dog health metric because it requires owners to speculate about what their veterinarian's opinion about their dog's health might be. Not only would such responses be biased by the owner's own beliefs, but such speculative, 'hearsay' responses will almost-certainly be less reliable than polling the owner's own opinion of their dog's health directly. For that reason, rejecting the *veterinary health* metric as our outcome variable was justified.

The reviewer also mentions other possible health metrics including classifying dogs according to the presence of 22 specific health disorders. Whilst a veterinarian's definitive diagnosis of a specific disease would certainly have been a superior outcome variable to study, this was again **not** what the Knight et al. (2022) study reported. Instead, these health metrics relied on owner opinions and not veterinary assessments:

"If veterinarians reportedly considered dogs to be suffering from health disorder(s), guardians were asked which disorder(s) these were, from among 18 disorders indicated to be among the most common disorders experienced by companion dogs [22–26]. Guardians were able to select multiple disorders, and to provide details of additional disorders by selecting 'other'. Details for each 'other' entry were examined, with these entries then reclassified into 18 existing or four new disorder types, giving a total of 22 possible health disorders."

Therefore, all the *specific health disorder* metrics will suffer the same limitations as for the *veterinary health* metric and, again, will be inferior to owner-reported dog health whilst, perhaps, creating the illusion of being a robust health measure.

Further, rather than being definitive veterinary diagnoses of specific diseases, these metrics were just disease categories, many of which are very broad (e.g., allergy, eyes, heart, mobility). The criteria used for including dogs in these categories was not standardised, and the severity of each condition was not reported. Therefore, it is likely that each category contained multiple differing and diverse diseases, making it unclear as to what was actually being reported by owners.

Moreover, although many dogs were positive for one or more of these health disorders (1,477 from 931 dogs; see S1 Table below, reproduced from Knight et al. 2022), many categories contained small numbers (e.g., allergy 20 dogs, hormonal 31 dogs, kidney 17 dogs, liver 15 dogs, epilepsy 17 dogs).

S1 Table. 1,477 cases of 22 specific disorders or affected bodily systems, in 931 dogs fed three main diets, based on reported assessments of veterinarians
[from Knight et al, 2022]

Disorders (22)	Conventional meat	Raw meat	Vegan	Total
Allergy	13	7		20
Anal glands	69	22	9	100
Behavioural	43	3	4	50
Body weight	61	12	7	80
Cancer/tumours	25	18	7	50
Dental/oral (teeth/mouth)	75	23	12	110
Ears	85	48	9	142
Epilepsy	11	5	1	17
Eyes	37	17	4	58

Gastrointestinal (e.g., diarrhoea, vomiting)	129	35	15	179
Heart	28	8	9	45
Hormonal (e.g., diabetes, hyper-/hypothyroidism, Addison's, Cushing's)	21	6	4	31
Injury	13	12	2	27
Internal parasites	7	2	9	18
Kidney	7	7	3	17
Liver	9	3	3	15
Lower urinary tract	18	5	4	27
Mobility	90	29	16	135
Other medical	18	18	4	40
Other musculoskeletal (muscle or bone) disease	92	41	12	145
Respiratory tract (airways/lungs)	11	10	3	24
Skin/coat	85	46	16	147
Total	947	377	153	1477

For these reasons, we decided it was best to limit our analyses to the *owner-reported dog health* metric. Nonetheless, in the absence of reliable veterinarian disease diagnoses, we accept that limiting the analyses to one health metric was a study limitation and have emphasised this more clearly in the discussion by adding the following text:

"Fourth, the outcome variables tested in the current study were derived from only one of the health metrics examined in the original study, namely owner-reported dog health [15]. Other health metrics reported by Knight et al. included asking owners "to report what they believed their veterinarian's assessment [of their dog's health] to be" [15] and owner reports of 22 possible health disorders, grouped into broad categories (e.g., allergy, cancer/tumours, hormonal, skin/coat) [15]. Our conclusions might have differed if one or more of these alternative health metrics had been analysed, but we decided against this because, in our opinion, all were inferior to owner-reported dog health. Not only were these metrics also based on subjective owner reports, but owners were required to speculate on what their veterinarian's opinion about the health of their dog would be. Further, there were no clear definitions of the health disorder categories, meaning that these would likely be broad, comprising many different diseases with differing aetiology and severity. Finally, the number of dogs included in many categories was small, being ≤50 in 12 of the 22 [15]. Whilst further work could be considered where these additional metrics are analysed using the techniques of the current study, it is questionable as to whether anything further would be gained. Instead, it would be preferable instead to conduct a

prospective study utilising objective, veterinary-derived measures of health, as discussed above."

Reviewer comment 5

Additionally, this study excluded data from 111 guardians that reported no or a lesser role in decision making for their dog "given uncertainty with reliability of these data". However, such decision making was limited to pet food purchasing decisions. There is no reason to suggest such guardians might not provide reliable data concerning health outcomes (all respondents confirmed they were aged 18+); hence, their selective exclusion has reduced the data set considered, without adequate justification.

Authors' response:

Thank you for this comment, and we accept the reviewer's criticism that we did not adequately justify why we chose to exclude these 111 owner-dog pairs. The reason for this was because these respondents reported playing either *"no role"* or *"some lesser role"* (a poorly-define category) in decision-making about the dog's food, raising valid concerns about the accuracy of any diet information provided. Further, given a lesser, and sometimes limited to negligible, involvement in diet decisions, it is plausible that many of these owners were also less involved in other aspects of the dog's life, including health and veterinary matters; therefore, there are also valid concerns about the reliability of this information. Unfortunately, there is no way of knowing whether this was the case because this was not checked in the original study [15].

Beyond valid concerns over data reliability, it is also possible that the opinions/attitudes of these respondents towards dog health and veterinary care might have differed systematically from respondents who took primary responsibility for diet. Indeed, previous research has indicated that the diet choice of owners is strongly associated with perceived health benefits (Morgan et al 2017), whilst owners who feed unconventional diets such as raw diet are less likely to base decisions on advice from a veterinary professional (Morgan et al 2022).

Given these concerns, we decided that it was safer not to include these responses, whilst recognising that this would mean a marginal decrease in overall sample size, increasing the possibility of analyses being underpowered (type II error). However, excluding these 111 owner-dog pairs, only reduced the dataset by ~5% (from 2,322 to 2,211) and, therefore, the effect on study power is minimal. Any downside is likely to be more than made up for by the more robust dataset that was used.

To address this point in our revised manuscript, we have expanded the discussion about both the limitations of and reasons for excluding owners who were not primary decision-makers in the discussion. The additional text reads as follows:

"Related to this, a second limitation was that we excluded data from 111 respondents who were not the primary decision makers about the dog's diet. Some of these owners reported

playing "some lesser role", whilst others reported playing "no role" in diet decision-making [15]. This raised concerns about the accuracy of the diet information. Given this lesser (and possibly limited or negligible) involvement in diet decisions, it is plausible that these owners might also be less involved in other aspects of the dog's life, including health and veterinary matters; therefore, this information might also be unreliable. Given that there was no way of knowing whether this was the case, because it was not checked in the original study [15], we decided that it was safer to exclude these data. A possible concern would be a marginal reduction in sample size although, given that the final dataset was only ~5% smaller (2,211 vs. 2,322), any negative effect on study power would likely to be minimal and offset by the benefits of greater data reliability."

Reviewer comment 6

On the other hand, this study also included a number of dogs fed vegetarian diets (n = 35). In our prior Knight et al (2022) study we excluded these as our intention was to study only dogs fed vegan or meat-based diets. By including these dogs fed vegetarian diets within the 'vegan' group, the current study misreports all results relating to 'vegan' diets. The results actually refer to 'vegetarian and vegan diets'. These cannot be accurately compared to our prior results relating [only] to 'vegan diets'.

Authors' response:

This particular comment from reviewer 2 is **factually incorrect** and has arisen from a misunderstanding about how diet variables were handled in our logistic regression models. We hope that the following point-by-point clarification will be helpful:

- In preliminary statistical analyses (simple binary LR and simple ordinal LR), we assessed the effect of diet in different ways (see supplementary information for full details). This included assessing *dog diet* as a 4-category variable (conventional [reference], raw, vegan and vegetarian) and also testing binary predictor variables e.g. *raw diet*, *vegan die*t, and *vegan-vegetarian diet*. This last variable did include dogs fed either vegan or vegetarian diets, but was **only** used in preliminary analyses, and subsequently rejected.

- In this respect, when performing logistic regression, we started by testing the performance of each of all our predictor variables separately using simple (i.e., univariable, only one predictor variable) regression, to identify variables for inclusion in multiple regression modelling (based on $P<0.2$).

- However, given that the *vegan diet* and *vegan-vegetarian diet* variables utilised overlapping data, we compared their relative performance. Given that the *vegan diet* variable performed marginally better (i.e. lower BIC), than the *vegan-vegetarian diet* variable, we selected the *vegan diet* variable for further testing, whilst the *vegan-vegetarian diet* variable was rejected and NOT tested further. For full transparency,

we show the statistical results obtained in these preliminary analyses in the figures below:

Only vegan dogs

```
# fit binary logit model and store results 'm'
m <- glm(Health_Binary ~ D_Diet_Vegan, data = ml,family = binomial)
# view a summary of the model
summary(m)
```

```
##
## Call:
## glm(formula = Health_Binary ~ D_Diet_Vegan, family = binomial,
##     data = ml)
##
## Coefficients:
##                 Estimate Std. Error z value Pr(>|z|)
## (Intercept)     -2.79839    0.09821 -28.495   <2e-16 ***
## D_Diet_VeganYes -0.36215    0.31060  -1.166    0.244
## ---
## Signif. codes:  0 '***' 0.001 '**' 0.01 '*' 0.05 '.' 0.1 ' ' 1
##
## (Dispersion parameter for binomial family taken to be 1)
##
##     Null deviance: 944.05  on 2210  degrees of freedom
## Residual deviance: 942.57  on 2209  degrees of freedom
## AIC: 946.57
##
## Number of Fisher Scoring iterations: 5
```

```
BIC(m)
```

```
## [1] 957.9707
```

Vegan and vegetarian combined

```
# fit binary logit model and store results 'm'
m <- glm(Health_Binary ~ D_Diet_Vegan_Veggie, data = ml,family = binomial)
# view a summary of the model
summary(m)
```

```
##
## Call:
## glm(formula = Health_Binary ~ D_Diet_Vegan_Veggie, family = binomial,
##     data = ml)
##
## Coefficients:
##                        Estimate Std. Error z value Pr(>|z|)
## (Intercept)            -2.80985    0.09954 -28.228   <2e-16 ***
## D_Diet_Vegan_VeggieYes -0.22510    0.28243  -0.797    0.425
## ---
## Signif. codes:  0 '***' 0.001 '**' 0.01 '*' 0.05 '.' 0.1 ' ' 1
##
## (Dispersion parameter for binomial family taken to be 1)
##
##     Null deviance: 944.05  on 2210  degrees of freedom
## Residual deviance: 943.38  on 2209  degrees of freedom
## AIC: 947.38
##
## Number of Fisher Scoring iterations: 5
```

```
BIC(m)
```

```
## [1] 958.7867
```

- These reports indicate that the *vegan diet* variable produced a model with a BIC which was marginally less than that of the *vegan-vegetarian* diet variable. This was the basis for favouring the *vegan diet* variable.

- The only other time that dogs fed vegetarian diets were assessed was when the *dog diet* variable was tested, with an example shown in the figure below:

```
# fit binary logit model and store results 'm'
m <- glm(Health_Binary ~ D_Diet, data = ml,family = binomial)
# view a summary of the model
summary(m)
```

```
##
## Call:
## glm(formula = Health_Binary ~ D_Diet, family = binomial, data = ml)
##
## Coefficients:
##                                                      Estimate Std. Error
## (Intercept)                                           -2.5819     0.1152
## D_DietMeat-based - raw                                -0.7196     0.2306
## D_DietVegan (consuming no animal products)            -0.5786     0.3165
## D_DietVegetarian (including eggs or milk, but not meat)  0.3132     0.6173
##                                                      z value Pr(>|z|)
## (Intercept)                                          -22.405   <2e-16 ***
## D_DietMeat-based - raw                                -3.121   0.0018 **
## D_DietVegan (consuming no animal products)            -1.828   0.0675 .
## D_DietVegetarian (including eggs or milk, but not meat)  0.507   0.6119
## ---
## Signif. codes:  0 '***' 0.001 '**' 0.01 '*' 0.05 '.' 0.1 ' ' 1
##
## (Dispersion parameter for binomial family taken to be 1)
##
##     Null deviance: 944.05  on 2210  degrees of freedom
## Residual deviance: 931.14  on 2207  degrees of freedom
## AIC: 939.14
##
## Number of Fisher Scoring iterations: 6
```

```
BIC(m)
```

```
## [1] 961.9416
```

- As stated above, there were 4 categories in this predictor variable, **which included separate vegetarian and vegan categories**.

Therefore, in contrast to what the reviewer claims, the analyses performed using dog diet variables were comparable with those of the early (Knight et al. (2022)) study. This is supported by the fact that the findings of our simple logistic regression analysis (ordinal LR and binary LR on any health outcome) revealed similar associations to the analyses reported the previous study (where significant effects of both the raw and vegan, but not vegetarian, diets were seen. Of course, in our study, the diet effects were curtailed when multiple regression was employed.

Nonetheless, the reviewer's misunderstanding on this point enabled us to reflect on how the methodological details of variable selection for modelling were described, not least for variables which used the same data coded in different ways (e.g., dog diet). To improve clarity,

we have rewritten the text in the methods so as better to explain the process and to avoid any further confusion. The revised text now reads:

"Ordinal logistic regression models were created using either the 'polr' function of the 'MASS' package [39] or the 'vglm' function of the 'VGAM' package [52]. The outcome variable was illness severity (none = 0, mild =1, serious = 2), whilst all owner, animal and healthcare variables were tested as predictor variables. Initially, simple ordinal regression was used to test associations between illness severity and single predictor variables. Initially, simple ordinal regression was used to test associations between illness severity and single predictor variables, **to determine their unadjusted effect on the outcome variable and to select variables for inclusion in multiple regression modelling***. Variables* **where the same original data had been coded in** *different ways (e.g., dog age* **coded** *as both a continuous and ordinal variable***; veterinary visits coded as both a 4- or 5-category variable; dog diet both as a 4-category variable [conventional, raw, vegetarian and vegan] or as separate binary variables for raw diet, vegan diet or a combined vegan-vegetarian binary variable [see above];** *breed* **coded both** *as* **the original** *breed size category* **and as a** *giant breed* **binary variable***) were tested* **separately in simple regression models, and only the coding approach that** *fitted the data best, based on the Bayesian Information Criterion (BIC; see below) [58]***, was selected for use in any further analyses. For example, the vegan diet binary variable and dog age ordinal variable performed better than the vegan-vegetarian and dog age continuous variables, respectively.**

After this initial screening stage, a *'best fit' multiple regression model was* **then** *created which initially included variables that met the threshold of P <0.2 on simple regression. This model was refined in a backwards and forwards stepwise fashion, with the BIC being used to select the model within the same family with the best generalisability (a measure of its goodness of fit compared with its complexity) [58]. With this approach, the existing model was repeatedly refined with addition or removal of variables until the model with the smallest BIC was found, according to previously published rules [59]. Models with interaction terms were also tested when these were clinically relevant (e.g., between dog sex and neuter status, between owner and dog diet categories and between location and setting). Results are reported as odds ratios (OR) with the associated 99% confidence intervals (99%-CI). For variables that did not meet the proportional odds assumption, separate OR and 99%-CI are reported for the different levels of illness severity ('minor' and 'significant').* **For comparison with the best-fit multiple regression model, an 'all-variable' multiple regression model was also built which contained all variables (owner-animal metadata and healthcare variables), except for those variables where the same original data were coded in different ways, when only the best-fitting variable (as determined above) was used.**

Reviewer comment 7

This study also grouped several reporting categories, including guardian and animal ages. Such data grouping can aid statistical analysis, but can also risk losing finer discrimination within data. The authors noted this limitation: "The disadvantage of such an approach was that many smaller groups needed to be

excluded, meaning that we might have missed some variables with a potential impact on owner-reported canine health".

Authors' response:
We agree with the reviewer that the decision to combine categories was a limitation since some genuine associations might have been missed. However, grouping data in this way was necessary, given the statistical methods used, not least since including groups with markedly different sizes can unbalance models and lead to poor performance. Of course, whilst there might have been genuine differences with such small groups, but it is unlikely that these would have been identified anyway because, given the small sample size, any analyses would almost-certainly be under-powered to detect them (type II error). To ensure that we any grouping of data was justified, and as explained above, we tested different groupings of such same predictor variables (e.g., veterinary visits, which was grouped either into a 4- or 5- category variable) and then selected the one that performed best in terms of model fit. Therefore, our choice of grouping can be justified from a statistical point of view.

Reviewer 2 was particularly concerned about how the *dog age* variable was handled, and this warrants further discussion. Data on dog age were originally tested (in simple LR) either as a continuous variable or as an ordinal categorical variable with 5 categories (1-3 years [reference category], 3-5 years, 5-7 years, 7-9 years and 9-20 years). The *categorical dog age* variable performed better than the *continuous dog age* variable in all LR modelling (ordinal LR and binary LR on both the *any health problem* and *significant illness* variables). For example, here are the statistical outputs for simple binary LR on the *significant illness* variable:

Dog age as a continuous variable

```
# fit binary logit model and store results 'm'
m <- glm(Health_Binary ~ D_Age, data = ml,family = binomial)
# view a summary of the model
summary(m)
```

```
##
## Call:
## glm(formula = Health_Binary ~ D_Age, family = binomial, data = ml)
##
## Coefficients:
##             Estimate Std. Error z value Pr(>|z|)
## (Intercept) -4.09067    0.22096 -18.513  < 2e-16 ***
## D_Age        0.17219    0.02388   7.211 5.54e-13 ***
## ---
## Signif. codes:  0 '***' 0.001 '**' 0.01 '*' 0.05 '.' 0.1 ' ' 1
##
## (Dispersion parameter for binomial family taken to be 1)
##
##     Null deviance: 944.05  on 2210  degrees of freedom
## Residual deviance: 892.40  on 2209  degrees of freedom
## AIC: 896.4
##
## Number of Fisher Scoring iterations: 6
```

```
BIC(m)
```

```
## [1] 907.8042
```

Dog age as an ordinal 5-category variable

```
# fit binary logit model and store results 'm'
m <- glm(Health_Binary ~ D_Age_quant, data = ml,family = binomial)
# view a summary of the model
summary(m)
```

```
##
## Call:
## glm(formula = Health_Binary ~ D_Age_quant, family = binomial,
##     data = ml)
##
## Coefficients:
##                    Estimate Std. Error z value Pr(>|z|)
## (Intercept)         -3.5984     0.2811 -12.800  < 2e-16 ***
## D_Age_quant(3,5]     0.2796     0.3792   0.737   0.4608
## D_Age_quant(5,7]     0.5399     0.3750   1.440   0.1499
## D_Age_quant(7,9.4]   0.9311     0.3640   2.558   0.0105 *
## D_Age_quant(9.4,20]  1.6016     0.3176   5.043 4.59e-07 ***
## ---
## Signif. codes:  0 '***' 0.001 '**' 0.01 '*' 0.05 '.' 0.1 ' ' 1
##
## (Dispersion parameter for binomial family taken to be 1)
##
##     Null deviance: 904.96  on 2067  degrees of freedom
## Residual deviance: 863.93  on 2063  degrees of freedom
##   (143 observations deleted due to missingness)
## AIC: 873.93
##
## Number of Fisher Scoring iterations: 6
```

```
BIC(m)
```

```
## [1] 902.0998
```

As can be seen, model generalisability is better with the ordinal categorical age variable (BIC 902) compared with the continuous age variable (BIC 907). The reasons for this are not clear but might be because the age effect is not linear, or because there are problems with accuracy of age data. In this respect, many owners estimate their dog's age (if date of both is not known, for example, if they were rescue dogs) and sometimes round up and down to the nearest year.

Therefore, whilst we agree with the reviewer that combining categories might mean we missed some genuine effects of small groups, we did carefully consider how best to code each predictor variable to ensure best performance. To ensure that we have properly explained and justified our statistical approach to testing predictor variables, we have modified the following text in the discussion, as follows:

"The main limitations to the current study have already been discussed above, including the fact that owner-reported health was the main outcome measure and the fact that the study population was not representative of the general dog-owning population. Further, significant data pre-processing was required to ensure adequate group sizes for statistical analyses. The disadvantage of such an approach was that many smaller groups needed to be excluded, meaning that we might have missed some variables with a potential impact on owner-reported canine health. **That said, in preliminary testing, we coded several of our variables in different ways (e.g., dog age as a continuous or ordinal 5-category variable; veterinary visits as a 4- or 5-category ordinal variable), and tested their performance, selecting the approach that produced the best model fit. Therefore, whilst some genuine effects from**

small categories might have been missed when competing variables, the final best-fit models we selected were those that best fitted the data.”

Reviewer comment 8

Beyond the interpretation of strong diet effects as ‘minimal’ (see above), unfortunately this study also took several steps that are statistically flawed. One example was the inclusion of identical information within multiple variables (e.g., Table 3: ‘Dog diet’ and ‘Dog on vegan diet’), causing severe correlation between these ‘independent’ variables (‘multicollinearity’) and thus ambiguous effect estimates (Gogtay, Deshpande and Thatte, 2017).

As discussed above, this point from reviewer 2 is **factually incorrect**. Table 3 (like Tables 2 and 4) only reports the results of the initial simple (i.e., univariable) logistic regression, where each of the predictor variables were tested in **separate** LR models. As also explained above, variables that used the same original data were **never** analysed together in the same multiple regression model. Therefore, the concerns that the reviewer raises about multicollinearity are not relevant in this context.

Nonetheless, in multiple logistic regression, there was the potential for multicollinearity amongst any of the predictor variables to adversely affect model performance. As explained in the methods section of our original manuscript, we addressed this by testing for possible multicollinearity in ALL multiple regression models using **variance inflation factors (VIFs**; Fox and Monette, 1992). All these results are included in the accompanying statistical reports (supplementary information), but one such example (for the multiple LR regression model for the *significant illness* variable (Fig 4, S4 Table) is shown in the image below:

check for multicollinearity

```
car::vif(m)
```

```
##                 GVIF Df GVIF^(1/(2*Df))
## D_Age_quant 1.019052  4        1.002362
## Visits      1.445970  4        1.047177
## Meds        1.422167  1        1.192547
```

By way of interpretation, multicollinearity is not considered to be a concern when VIF or GVIF is <4 or GVIF(1/(2×Df) is <2. On this basis, we can confirm that multicollinearity was **never** an issue in any of our models.

We have reviewed the text included in the original manuscript about testing for multicollinearity, which was as follows:

For model validation, the proportionality assumption was examined graphically, with partial proportional odds models being used when models include variables that did not meet this assumption. Influential datapoints were identified and assessed using Cook’s distance; given that the data were from a secondary source, such datapoints were not removed, as their

validity could not be checked. Possible multicollinearity in all models was explored using the variance inflation factor (VIF) for predictors with 1 degree of freedom [Df]) or generalised variance inflation factor (GVIF) and GVIF$^{(1/(2\times Df)}$ for predictors with >1 Df (e.g., polynomial variables) [60]. A rule of thumb was applied whereby multicollinearity was not considered to be a concern when VIF or GVIF was <4 or GVIF$^{(1/(2\times Df)}$ was <2. If necessary, multicollinearity was resolved by removing the variable with the greatest VIF or creating a new dummy variable by combining variables with high collinearity. Goodness of fit was tested by visual inspection of observed and expected results and 3 tests for goodness of fit: the Lipsitz goodness of fit test for ordinal response models, the Pulkstenis-Robinson chi-squared test and the Hosmer and Lemeshow test for large datasets. The coefficient of determination (pseudo-R^2) of the overall model was calculated using a modification of MacFadden's R^2 proposed by Ugba and Gertheiss [61]."

We believe that this explanation is clear and, therefore, have decided not to amend it.

Reviewer comment 9

Similarly, it used 'veterinary visits and receiving medication' as 'independent' variables to predict the 'significant illness' outcome. It is trivial that dogs with more veterinary visits tend to be significantly ill more often, and including such indicators of illness as independent variables creates similar invalidity.

Thank you for this comment. We certainly agree that there might be associations between the frequency of veterinary visits and the chance of a dog receiving medication. Indeed, this was highlighted in our preliminary correlation analyses shown in Fig 2 (with further details in S1 Table and S2 Table), where a strong positive association between the *veterinary visits* variable and the *prescribed medication by vet* variable was seen (Kendall's tau 0.52, P<0.001). To ensure that including both variables together in multiple regression was appropriate, we first checked whether model fit might be affected by multicollinearity, as described in the previous response, and this was **never** an issue. For example, here is the statistical output for the multicollinearity check on the multiple binary LR regression model for the *significant illness* variable:

check for multicollinearity

```
car::vif(m)
```

```
##                 GVIF Df GVIF^(1/(2*Df))
## D_Age_quant 1.019052  4        1.002362
## Visits      1.445970  4        1.047177
## Meds        1.422167  1        1.192547
```

As can be seen, there is no evidence of multicollinearity. Further, in addition to testing models where these variables were included separately, we also tested models that included an interaction term (*veterinary visits* * *prescribed medication* by vet). However, adding such interaction terms led to models with worse generalisability (higher BIC) and, therefore, such interactions were not included in the final best-fit models. As an example, here is the

statistical output for the best-fit multiple binary LR regression model for the *significant illness* variable:

```
# fit binary logit model and store results 'm'
m <- glm(Health_Binary ~  D_Age_quant + Visits + Meds, data = ml,family = binomial)
# view a summary of the model
summary(m)
```

```
##
## Call:
## glm(formula = Health_Binary ~ D_Age_quant + Visits + Meds, family = binomial,
##     data = ml)
##
## Coefficients:
##                      Estimate Std. Error z value Pr(>|z|)
## (Intercept)           -5.3909     0.5472  -9.852  < 2e-16 ***
## D_Age_quant(3,5]       0.5301     0.4030   1.315  0.18838
## D_Age_quant(5,7]       0.6845     0.4005   1.709  0.08746 .
## D_Age_quant(7,9.4]     0.8114     0.3895   2.083  0.03721 *
## D_Age_quant(9.4,20]    1.5530     0.3422   4.538 5.68e-06 ***
## Visits1               -0.9584     0.5945  -1.612  0.10691
## Visits2               -0.4766     0.5917  -0.805  0.42053
## Visits3                0.1709     0.6128   0.279  0.78032
## Visits3<               1.5589     0.5633   2.767  0.00565 **
## MedsYes                2.2409     0.4227   5.301 1.15e-07 ***
## ---
## Signif. codes:  0 '***' 0.001 '**' 0.01 '*' 0.05 '.' 0.1 ' ' 1
##
## (Dispersion parameter for binomial family taken to be 1)
##
##     Null deviance: 904.96  on 2067  degrees of freedom
## Residual deviance: 631.68  on 2058  degrees of freedom
##   (143 observations deleted due to missingness)
## AIC: 651.68
##
## Number of Fisher Scoring iterations: 7
```

```
BIC(m)
```

```
## [1] 708.0255
```

Our conclusion from these analyses is that both the *veterinary visits* and *prescribed medication by vet* variables are independently associated with *owner-reported dog health*, and that there is no evidence of multicollinearity in the model. Therefore, we disagree with the reviewer that it was inappropriate to include both in the same model.

We have reviewed the text included in the original manuscript about testing for multicollinearity, as discussed above. Given that we believe this was clear, we have decided that changes to the manuscript are not needed.

Reviewer comment 10

Finally, this study critiqued our prior Knight et al (2022) study on the basis that our sample of respondents was not representative of the typical dog-owning public, because unconventional diet use was greater than normal. But as the authors also noted, we deliberately targeted guardians more likely to feed such diets, in an effort to increase respondent numbers within these groups, to

increase the statistical reliability of our results (this proved successful; our numbers were sufficient to yield reliable results).

Authors' response:

Thank you for this comment. As the reviewer will know, one requirement of the discussion section of any paper is to consider how generalisable the results of the study are to the population from which they are drawn. Indeed, this is a stipulation of many reporting guidelines, including those for humans (CONSORT, https://www.equator-network.org/reporting-guidelines/consort/; STROBE, https://www.equator-network.org/reporting-guidelines/strobe/) and those for companion animals (PETSORT, https://www.frontiersin.org/articles/10.3389/fvets.2023.1137781/full).

For the current study, it was necessary to consider how the dogs included in the original study population (from the Knight et al. [2022] study) had been recruited, and the impact that this might have had on generalisability of results. The targeting of specific groups of pet owners is particularly relevant here because the dogs studied would then not have been fully representative of the general pet dog population. Whilst this does not necessarily invalidate the results of either the Knight et al. (2022) study or the current study, it does mean that there is a need to be more cautious in how the findings are interpreted. Therefore, we believe that the comments in the discussion of the current study were necessary, although we have chosen to modify them (see below).

In this respect, we agree that the targeting strategy employed by the Knight et al. (2022) study succeeded in its stated aim of ensuring that dogs from minority diet groups were sufficiently represented. This meant that, in both studies, there was sufficient statistical power to detect differences between groups. However, our original discussion did not adequately emphasise this point and, therefore, we have reworded it as follows:

"Although the study was large, the population studied was not representative of the typical dog-owning public. In this respect, 33% and 13% of owners reportedly fed their dog raw and vegan diets, respectively, which is greater than expected; for example, in a recent UK survey, the proportion of owners feeding raw and vegan diets were 7% and <1%, respectively [1]. Further, the proportion of the UK human population reportedly consuming a vegan diet is estimated to be between 2 and 3% [2] whilst, in the current study, 10% were of owners identified as vegan. This was of particular significance given the identified of clustering of owner-animal metadata based on the owner's diet choice. Besides this, and despite involving responders from many countries, the geographical split was unbalanced with 73% of survey responses coming from the UK. Although the reasons for such imbalances are not clear, it might well have resulted from the method of owner recruitment. In this respect, the study was widely advertised via social media but, given concerns that owners feeding dogs unconventional diets might be under-represented, relevant interest groups were actively targeted and invited to **participate. This strategy was successful in ensuring that adequate numbers of dog owners feeding unconventional diets were included, ensuring that statistical comparisons were meaningful in both the previous [15] and current studies. However,** *the recruitment strategy might have inadvertently generated an unrepresentative study population which, in turn, may have influenced the results obtained, as discussed above."*

We believe that these changes bring a better balance to this discussion point.

Reviewer comment 11

This study also found weak associations of some guardian demographic factors – such as education level and geographic location – with their indicators of illness. These factors are interesting, but our intentions were not to explore the links between human demographic factors and dog health outcomes. Rather, our study was designed specifically to explore the effects of diet – including the use of unconventional diets, notably vegan and raw meat diets.

Arguably, this point is more a justification of the approach taken in the Knight et al. (2022) study, rather than a comment about the current manuscript. Nonetheless, we have addressed it as best we can. We certainly agree with the reviewer that the primary aim of the Knight et al. (2022) study was to examine the effect of diet choice on dog health. Of course, given the study design (cross-sectional, observational study), causality cannot be inferred from any associations identified. Instead, the identified association might be related to another unmeasured variable. As described in the Introduction, Methods and Discussion of our paper, this was the very reason that we chose the approach we did, namely, to assess associations between a wider range of predictor variables and owner-perceived dog health, and to determine their relative importance. In so doing, we extended rather than repeated the work of Knight et al. (2022), and it is this that makes our study novel.

To fulfil our aim, testing associations with owner demographic variables (e.g., education level, geographic location, income, setting [urban-rural] etc) was important, not least since previous studies have identified associations between owner variables are associated and both diet choice and dog health. For example, in one previous study (Hoummady et al 2022), associations were identified between owner variables (including where they lived) and the diet choices they made. Further, some owner variables are also associated with the presence of health conditions in dogs,; for example, previous research has demonstrated associations between owner factors (such as age and income) the odds of their doing having obesity (Courcier et al 2010). Therefore, we believe that we were justified in exploring predictor variables other than diet-related variables in the current study.

Nonetheless, given the reviewer's concern, we have amended the Discussion to emphasise that the aim of the Knight et al. (2022) study was to examine associations with diet, and better explain why it was justified to look at other variables (including owner variables) as part of the current study. Therefore, the opening part of the discussion now reads:

"In the current study, we examined associations between owner perceptions of the health of their dog, and a range of owner-related, animal-related and healthcare variables. We utilised data from a previous study, which had used a questionnaire to gather the opinions of owners about the health of their dogs [15]. The primary aim of that study was to examine associations between owner opinions of dog health status and the type of diet that owners predominantly

fed, with the key finding being that health was positively-associated with feeding either a vegan or raw diet, compared with other diet types (including conventional and vegetarian). **Given the focus of that study,** *other information gathered in the questionnaire was not assessed and no account was taken of possible confounding amongst variables.* **Therefore, to extend the findings of the previous work, additional animal, owner and healthcare variables were studied. Previous work has reported associations between such variables and owner decisions about feeding. For example, animal variables, such as age and neuter status, were associated with the owner feeding choice in the previous study of Knight et al. [15]. In the same study, maintenance of pet health was the most-common reason cited by owners for choosing a particular food [15], whilst owner characteristics (such as geographic location) are also associated with food choice [64]. Finally, owner characteristics can also be associated with aspects of dog health; for example, both owner age and income are associated with the prevalence of obesity in dogs [65]. Considering these previous findings, our decision to examine variables beyond those of diet type was justified."**

Reviewer comment 12

Finally, the authors noted that reliance on guardian reported data and opinions was a major limitation of our study. We already discussed this limitation extensively within our prior study.

Authors' response:

We agree with the reviewer that these limitations had already been discussed in the Knight et al. (2022) study. However, given that the same data were used in our study, it was also necessary for us to consider those same limitations when discussing the implications of the results. However, we were careful not simply to repeat the discussion of Knight et al. (2022) but, instead, to complement and expand it. We believe that we were successful in this regard. For example, Knight et al. (2022) discussed concerns about possible unconscious bias from owner assessments, as follows:

"Another source of potential error, when relying on guardian answers, is unconscious bias. This could occur if a guardian using a conventional or unconventional pet diet expected a better health outcome as a result, and if this expectation exerted an unconscious effect on their answers about pet health indicators. Our study included more vegans than reported in some other studies [53]. It is conceivable that vegans, or respondents following other dietary groups, such as omnivores, might have had greater subconscious expectations of good health, when animals were fed diets similar to their own. We acknowledge such possible unconscious bias effects cannot be fully eliminated, but to minimise their effects on reported results, we ensured that survey questions asking about animal health were positioned prior to questions about ani- mal diets. This minimises chances that answers might be affected by prior answers about dietary choices, e.g., if a guardian reporting use of an unconventional diet, subsequently became more likely to consciously or unconsciously under-report health problems. Additionally, by careful wording choice, no bias for or against any particular diet was implied within survey advertising materials, or within the survey questions or explanatory text. We do not consider that any remaining unconscious bias effects would be appreciably greater in one

dietary group than another; hence consider that their effect on our results was probably minimal, overall."

In the discussion of the current study, we extended the discussion of the issue of unconscious bias (for example, by discussing possible differences between owners feeding a conventional vs an unconventional diet), but also by considering other types of bias such as recall bias.

It is comforting that we agree with the reviewer about the importance of considering these limitations. Therefore, to ensure we reflect this between-study agreement better, and specifically to acknowledge Knight et al. (2022) also discussed these limitations, we have decided to modify the discussion paragraph as follows:

*"It is also feasible that the attitudes and beliefs of owners might either consciously or unconsciously have influenced responses about the health of their dog***, a point that the authors of the previous study were also keen emphasise [15]***. For example, owners who believe a particular type of diet to be optimal for* **dog health***, might be more likely to perceive their dog to be healthy, whether or not this* **was** *actually the case. Whilst such a bias might feasibly affect any diet type, a greater bias might be expected with diets perceived to be 'unconventional'***, again, as acknowledged in the previous study [15]***. Although increasing in popularity [1], both vegan diets and raw meat diets are still an uncommon choice for owners, and many veterinary professionals either do not recommend them or might even advise against their use. Current evidence suggests that both such diet types are often not formulated appropriately to be complete and balanced for essential nutrients [5,8,9-11]. Further, there are also concerns with feeding raw food given the potential for contamination either with bacteria of possible pathogenic potential or bacteria resistant to antimicrobials, both of which might pose a risk to the health of owners and other in-contact people [11-13]. Given such health concerns, owners who make an active choice to feed either a raw or vegan diet could be more defensive about this diet choice, compared with owners who feed conventional diets, and this attitude which might unconsciously have biased responses about the health of their dog, for example, minimising the significance of any illnesses. In the original study questionnaire, the authors did attempt to minimise* **the** *influence of canine diet variables on reported health status, by placing the health-related questions before most other variables [15]. However, it is unclear as to whether this would adequately eliminate pre-existing unconscious biases resulting from diet choice.*

Reviewer comment 13

In short, the conclusion of the current study that the results conflicted with those of our previous Knight et al (2022) study, often seems unsupported, when the results of our respective studies are examined. Multiple differences in the variables and data chosen for consideration, and the statistical methods used, have resulted in the differences observed. Unfortunately, the data chosen for analysis within this study do not accurately represent results of [only] vegan diets, and several statistical steps taken were significantly flawed.

Authors' response:
Whilst we agree with the reviewer that the conclusions of the current study were distinct from those of Knight et al. (2022), and that we used different inclusion criteria for the current study (necessitated by the statistical approach that we took), we fundamentally disagree that these conclusions were unsupported. As discussed in detail in our responses above:

- **The statement that our that our findings conflicted with those of the Knight et al. (2022) study is not entirely correct.** In fact, some findings are consistent with those of the Knight et al. (2022) study, most notably, the results of simple logistic regression models. This is not surprising, not least given that the univariable analyses involving dietary type were equivalent to those performed by Knight et al. (e.g., odds ratios and ANOVA).

  However, differences between studies were see with both multiple regression and XGBoost where, after accounting for other variables, associations with diet were curtailed. These models clarified the minimal impact of dog diet on health. For example, on simple logistic regression, feeding a vegan diet was only associated with 0.2-0.5% of the variance in owner-perceived health, depending on the type of model used. Further, including diet-related variables in multiple logistic regression analysis, did not improve overall model fit, whilst modelling using XGBoost suggested that diet-related variables were of low importance.

  Therefore, rather than conflicting with the previous work, the current study should instead be viewed as an extension of the previous work, providing the reader with a greater understanding of the possible reasons for owner opinions on owner-observed health as well as the impact of the variables studied.

- **We disagree that the statistical steps were significantly flawed**. For example, we did not, as the reviewer stated, include variables utilising the same data (e.g., diet choice) in the same multiple regression model and, as part of our model validation procedures we assessed for and excluded the possibility of multicollinearity using a recognised statistical technique (analysis of VIFs).

- **The reviewer's claim that dogs fed vegetarian diets were included within the *vegan diet* variable is not entirely correct.** Whilst a combined *vegan-vegetarian diet* variable was tested in preliminary analyses, this was dropped in favour the *vegan diet* variable, where only vegan dogs were included. Therefore, this diet variable was an accurate representation of the vegan diet data in the Knight et al. (2022) study.

- **We stand by our decision to exclude categories within variables where the number of datapoints were small**. Where group sizes differ markedly within a variable, models can be unbalanced and performance can be affected. In any case, given such small groups, any statistical analyses are likely to be underpowered to detect differences between these groups and others (type II error). Given that the grouping approach invariably improved model fit, we believe that it was statistically justified.

- **Related to this, the decision to exclude results from caregivers who were not the primary decision makers was also justified**. Data loss was minimal (~5%), and the final dataset still large (>2,000 owner-dog pairs) meaning that any limitation was more

than outweighed by the benefit of improved model balance and performance. Besides this, the validity of the data from individuals who played either no part at all the diet choice for their dog or played some lesser role (a very variable category), was likely to be unreliable.

- **Whilst recognising it was a subjective assessment and was flawed, we stand by our choice of outcome measure (owner-reported dog health)**. It is highly likely that the subjectivity and flaws of all other health outcome metrics available in the dataset (and studied by Knight et al.) would be worse. However, we agree with the reviewer that this was a possible a study limitation, and now discuss this at length in the paper. Further, we agree with the reviewer that further studies on the health implications of feeding vegan and raw diets are now required, that utilise more reliable health outcome measures, which are evidence-based and derived from veterinarians.

We would like to conclude by thanking Knight et al. (2022) for having engaged in open science principles by making their original dataset available. It is a truly informative dataset, and we are grateful to have had the opportunity to have analysed it.

Other changes to manuscript

After having made these extensive changes, we have thoroughly re-read the entire manuscript for errors and readability. A small number of grammatical and typographical errors were identified (see tracked changes version), for which we apologise. Full details can be seen in the tracked changes version of the manuscript, and include:

- Very minor improvements to grammar
- Correction of spelling mistake or duplicated words
- Ensuring consistency with decimal places (all 1 DCP)
- Ensuring consistency throughout the manuscript with the naming and formatting (italics) of outcome and predictor variables.
- Correcting the legend to Table 1 where the assignment of the dog diet variable had not properly been explained.

All these changes are minor and do not affect the meaning of the text. Once again, we apologise for the errors, but hope that correcting them now will save time required in the future during editing.

References

Courcier EA, Thomson RM, Mellor DJ, Yam PS. An epidemiological study of environmental factors associated with canine obesity. J Small Anim Pract. 2010 Jul;51(7):362-7. doi: 10.1111/j.1748-5827.2010.00933.x

Fox J, Monette G. Generalized Collinearity Diagnostics. J Am Stat Assoc. 1992;87:417, 178-183, DOI: 10.1080/01621459.1992.10475190

Hoummady, S., Fantinati, M., Maso, D. et al. Comparison of canine owner profile according to food choice: an online preliminary survey in France. BMC Vet Res 18, 163 (2022). https://doi.org/10.1186/s12917-022-03258-9

Knight A, Huang E, Rai N, Brown H. Vegan versus meat-based dog food: Guardian-reported indicators of health. PLoS One. 2022 Apr 13;17(4):e0265662. doi: 10.1371/journal.pone.0265662

Morgan SK, Willis S, Shepherd ML. Survey of owner motivations and veterinary input of owners feeding diets containing raw animal products. PeerJ. 2017 Mar 2;5:e3031. doi: 10.7717/peerj.3031

Morgan G, Williams N, Schmidt V, Cookson D, Symington C, Pinchbeck G. A Dog's Dinner: Factors affecting food choice and feeding practices for UK dog owners feeding raw meat-based or conventional cooked diets. Prev Vet Med. 2022 Nov;208:105741. doi: 10.1016/j.prevetmed.2022.105741
