# Supplementary material for: Variables associated with owner perceptions of the health of their dog: Further analysis of data from a large international survey
Source: PLoS One. 2024 May 15;19(5):e0280173. doi: 10.1371/journal.pone.0280173 (PMC11095744; doi:10.1371/journal.pone.0280173)
Supplement: Attachment — Submitted filename: review 2023 05 24.docx [file pone.0280173.s025.docx]

Response to Barrett-Jolley and German (2022)

This authors of this current study responded to our prior Knight et al (2022) study (<https://doi.org/10.1371/journal.pone.0265662>), after reanalysing the dataset we collected, which is available open access.

Unfortunately, within this current study there are substantial flaws in both the statistical analysis, and the representation of the results. It is unfortunate that the authors did not choose to consult with Knight et al. during design of their study, which would have allowed the following flaws to be identified in advance and avoided. Our Knight et al (2022) study was large and complex in some respects, and the authors are encouraged to do this, if planning any further reanalyses of our data.

The authors of this current study concluded that diet has, at most, minimal association with owner-perceived canine health. They stated that these results conflicted with those of our previous Knight et al (2022) study. On closer examination, however, it becomes apparent that their results sometimes reinforce those of our previous study, and where they differ, this appears due to differences in parameters chosen for study, and the statistical methods used.

Our previous study found statistically significant associations between diet and all seven general illness indicators studied. In every case the effect size was small. After controlling for numerous demographic factors and other variables via logistic regression analyses, the authors of the current study found the vegan diet lowered the risk of 'any health problem' by 43.2% (Table 3). Far from conflicting with our previous results, this key result appears to have strengthened our conclusion that the healthiest and least hazardous diet was nutritionally-sound vegan dog food. Hence the conclusion that only minimal benefits were associated with a vegan diet, is not supported by the study results. A 43.2% risk reduction is far from minimal.

Unlike our prior Knight et al (2022) study, which assessed seven general indicators of illness, and also considered the prevalence of 22 specific health disorders, the current study did not consider any specific health disorders, and it created and assessed only two general indicators of illness from the raw data. For one of them (‘any health problem’), guardian opinions of health were used to create this indicator. However, guardian opinions of health status are normally less reliable than veterinary assessments. The indicator that should have been used, to achieve greater reliability, was reported veterinary assessment of being unwell.

Additionally, this study excluded data from 111 guardians that reported no or a lesser role in decision making for their dog “given uncertainty with reliability of these data”. However, such decision making was limited to pet food purchasing decisions. There is no reason to suggest such guardians might not provide reliable data concerning health outcomes (all respondents confirmed they were aged 18+); hence, their selective exclusion has reduced the data set considered, without adequate justification.

On the other hand, this study also included a number of dogs fed vegetarian diets (n = 35). In our prior Knight et al (2022) study we excluded these as our intention was to study only dogs fed vegan or meat-based diets. By including these dogs fed vegetarian diets within the ‘vegan’ group, the current study misreports all results relating to ‘vegan’ diets. The results actually refer to ‘vegetarian and vegan diets’. These cannot be accurately compared to our prior results relating [only] to ‘vegan diets’.

This study also grouped several reporting categories, including guardian and animal ages. Such data grouping can aid statistical analysis, but can also risk losing finer discrimination within data. The authors noted this limitation: “The disadvantage of such an approach was that many smaller groups needed to be excluded, meaning that we might have missed some variables with a potential impact on owner-reported canine health”.

Beyond the interpretation of strong diet effects as ‘minimal’ (see above), unfortunately this study also took several steps that are statistically flawed. One example was the inclusion of identical information within multiple variables (e.g., Table 3: ‘Dog diet’ and ‘Dog on vegan diet’), causing severe correlation between these ‘independent’ variables (‘multicollinearity’) and thus ambiguous effect estimates (Gogtay, Deshpande and Thatte, 2017).

Similarly, it used ‘veterinary visits and receiving medication’ as ‘independent’ variables to predict the ‘significant illness’ outcome. It is trivial that dogs with more veterinary visits tend to be significantly ill more often, and including such indicators of illness as independent variables creates similar invalidity.

Finally, this study critiqued our prior Knight et al (2022) study on the basis that our sample of respondents was not representative of the typical dog-owning public, because unconventional diet use was greater than normal. But as the authors also noted, we deliberately targeted guardians more likely to feed such diets, in an effort to increase respondent numbers within these groups, to increase the statistical reliability of our results (this proved successful; our numbers were sufficient to yield reliable results). This study also found weak associations of some guardian demographic factors – such as education level and geographic location – with their indicators of illness. These factors are interesting, but our intentions were not to explore the links between human demographic factors and dog health outcomes. Rather, our study was designed specifically to explore the effects of diet – including the use of unconventional diets, notably vegan and raw meat diets. Finally, the authors noted that reliance on guardian reported data and opinions was a major limitation of our study. We already discussed this limitation extensively within our prior study.

In short, the conclusion of the current study that the results conflicted with those of our previous Knight et al (2022) study, often seems unsupported, when the results of our respective studies are examined. Multiple differences in the variables and data chosen for consideration, and the statistical methods used, have resulted in the differences observed. Unfortunately, the data chosen for analysis within this study do not accurately represent results of [only] vegan diets, and several statistical steps taken were significantly flawed.
