# Supplementary material for: Variables associated with owner perceptions of the health of their dog: Further analysis of data from a large international survey
Source: PLoS One. 2024 May 15;19(5):e0280173. doi: 10.1371/journal.pone.0280173 (PMC11095744; doi:10.1371/journal.pone.0280173)
Supplement: Attachment — Submitted filename: review 2023 07 19.docx [file pone.0280173.s027.docx]

Review of Barrett-Jolley and German (2023) Revision 1

The authors of this current study responded to our prior Knight et al (2022) study (<https://doi.org/10.1371/journal.pone.0265662>), after reanalysing the dataset we collected, which is available open access.

**Conclusions and Abstract revisions**

Re: “The results of the current study conflict with previously-published research using the same dataset” (abstract) and “These results conflict with previously-published research” (Conclusions).

These statements are not correct, because (i) the authors have agreed (in several locations) that the results of their preliminary, simple (i.e., univariable) regression modelling were in fact similar to results from the previously-published research (Knight et al. 2022), and (ii) the previously-published research did not undertake multiple regression or XGBoost analyses as the authors have done, and so did not have any comparable results to compare with their own research.

The authors correctly noted in the Abstract, “The aim of the current study was to extend the earlier findings, using different modelling techniques and including multiple variables, to identify the most important predictors of owner-reported dog health.”

Hence, statements that “The results of the current study conflict with previously-published research…” are incorrect. They should be deleted or modified to clarify that the current study provides additional research findings, examining different aspects (e.g., guardian location), that extend the results of the previously-published research.

**Statistical analysis**

First, I thank the authors for answering all prior critique points in an unusual level of detail. I highly appreciate the effort put into this, including the compact overview table, facilitating the revision process, given that so many different points are discussed. This was very helpful.

Regarding my original comments #2, #3, #6 and #8:
Thank you for clarifying that the results I was referring to came from the univariate regression models. I apologize for my misunderstanding and also appreciate that you now have clarified the univariate character of the results in the respective table captions. The character of the model selection process and how differently encoded variables are handled is now explained in a significantly clearer way, within the manuscript.

Nonetheless, very central issues remain, with the statistical methodology of the study.

**Major statistical issues requiring revision**

1. *Use of health outcomes as independent variables*
   Thank you for the insights given in the answer to comment #9. Perhaps I didn’t phrase my initial comment clearly enough. What I see as being critical, is not a potential correlation between health outcome variables, but the inclusion of such health outcome variables as independent variables in the models per sé, potentially leading to hidden correlations caused by other variables. It could be that one variable (e.g., diet) is affecting both your response variable (overall health) and the other two health variables (vet visits and medication), but this effect is not visible in the final models because it is hidden by the correlations between the health variables. You correctly state this issue yourself in line 731. Hence I would suggest removing health outcome variables from the independent variable list.

Alternatively, given a key aim of this study (analysing the effect of diet on overall health), you at least need to perform a so-called ‘sensitivity analysis’ to check if any indirect effect/hidden correlation is present. For such a sensitivity analysis you could re-estimate your model selection approach by leaving out the health outcome variables from the independent variable list, reporting these results in the Supplementary materials and stating any identified potential differences in the manuscript.

2. *Model selection process*
   It is commendable that you utilized two different modelling approaches (classical regression estimation and the boosting approach) to more robustly analyse the data. However, from a statistical point of view, the variable selection approach used for the regression modelling is not state-of-the-art anymore. The main problem is that you base the initial decision on ‘which variables should be included within the available set of variables’, on univariate regression model results, which you yourself state are (potentially) wrong. In this way, you potentially miss individual variables which may not be included in the stepwise procedure.

In statistical research, the solution to this exact problem you are facing is exactly the alternative method that you already use: boosting. Boosting is the solution applied when a dataset is too small to estimate a regression model with a lot of variables. In this case, it is the better method compared to any stepwise selection procedure. Accordingly, I suggest dropping the stepwise-selected regression approach (maybe keep something like the ‘all-variable’ model, if you deem it meaningful), and fully concentrate on the boosting approach, as long as you stick to your research question with a bigger set of independent variables. This way, the manuscript will also become much easier to read and follow. While reading the (revised) manuscript, I found it quite hard to grasp which of the models is ‘the final model’ (selected regression model vs. Selected boosting model vs. All-variable model) and is the base for the main interpretation.

Nb: As an additional, minor note: in boosting there’s also the option in some packages to fix one independent variable as the main variable in the model. E.g., you could fix dog diet, so that it is always included in the model, in this way ensuring that you end with an effect estimate that you can compare with other publications.

3. *Exclusion of 111 data points (comment #5)*
   While I see your point that people with less involvement in the dog food purchase decision process might also be less involved/informed in health matters of the dog, and I appreciate the reasoning given in the discussion, deleting data from a study dataset is a very hazardous statistical step and should be treated with great caution. I still disagree with the reasoning to exclude these data points, but could accept this if the effect of the exclusion of these 111 data points was appropriately documented. Please perform a sensitivity analysis for your main statistical model(s), to A) run the analysis one time excluding the data points, and B) run the analysis another time including the data points. The results of B) can be placed in the Supplementary materials and briefly referenced within the manuscript, to show that the exclusion (hopefully) doesn’t alter results.

**Further issues requiring revision**

1. *Regarding comment #1:*
   I appreciate the paragraph added in line 798. I think it is not needed in this length and can be shortened quite a bit, also to keep the focus in the discussion. E.g., in rough summary, “We did not contact the authors for this study because A) the original study was well written, replicable etc. and B) we wanted to retain independent in the study process given the importance of independent replication to the scientific method” (or similar).
2. *Further data exclusion:*
   Referring to line 283 (“Influential datapoints were […] not removed unless there was a good reason to do so.“), it remains unclear if you did remove further points in addition to the above discussed 111 data points. Please specify more clearly which data points were excluded and provide the reasoning.
3. *Dimension reduction visualization (UMAP & clustering):*
   1. I do appreciate the effort in using modern statistical tools to facilitate the interpretation of potential complex multidimensional correlation structures in this dataset. However, I think the UMAP figures are minimally interpretable by many likely audiences (e.g., most veterinary readers), and (correct me if I’m wrong) as far as I see it the only clear finding from applying this method is that dog owners who are vegan themselves also tend to feed their dogs vegan/vegetarian more often. This latter statement could equally be made using an easily understandable table of relative frequencies in the data, which I would favour in this case, and – if deemed meaningful – a comment that the application of the clustering approach didn’t lead to further insights. Otherwise, for the sake of generalist reader comprehension, it would be necessary to explain the interpretation of the method and of the results in more detail (either in the main part of the paper, or in the Supplementary materials while quoting the most important findings in the main part).
   2. How big is the share of variability represented by the first two UMAP dimensions? Please calculate it and add a statement about it in the Statistics section. The confidence one can put in the interpretation of dimension reduction methods highly depends on the share of variability the shown dimensions explain.
   3. Please add a statement about the goodness of the clustering, regarding how well it separates the clusters from one another. One standard measure would be to add a silhouette plot and the average silhouette coefficient.
4. *Correlation matrix*
   I concur with the use of correlation matrices because they give a good overview of the data. Do I understand correctly that some bubbles in the correlation matrix are not shown because of some p-value based (in your case -LogP) or FDR-based criterion? This remains slightly unclear to me in the text.

In any case, I would suggest dropping both the LogP and FDR criteria to simplify the analysis, and showing all bubbles in the matrix. In all other analyses, you do a really nice job of not interpreting p-values (rightly so, after variable selection). In the correlation matrix, I would find it most informative and easiest understandable if you would 1) show all bubbles in the correlation matrix, and 2) state that this is an exploratory analysis. Individual correlation values shouldn’t be overinterpreted regardless, so you don’t gain anything, but mostly add complexity to your analysis when including LogP and FDR.

5. *Model evaluation*
   1. Please report AUC values (based on cross-validation or a hold-out test set) for all logistic regression models, instead of pseudo R², since AUC is the most established goodness of fit measure for logistic regression. (e.g., lines 41 & 54).
   2. In lines 271 ff. and 288 ff. you state “Goodness of fit was tested by […] 3 tests for goodness of fit“.
      In statistical research, it is the standard now to not perform such goodness of fit tests anymore to judge the overall goodness of a model, but instead to visualize and judge the distribution of the model errors (or in the logistic case looking at the ROC curve and the test set AUC). This is especially due to the fact that such tests are well-designed to detect strong deviations from the model assumptions, but have only low power in situations where model assumptions are more or less fulfilled. Accordingly, you generally gain more insights in the model structure by simply visually evaluating it with model evaluation plots (which can also be shown purely in the Supplementary materials, and referenced in the main manuscript). I would therefore suggest dropping all three tests and keep the analysis simple with ROC curves and AUC values.
6. *Results interpretation*
   1. Around line 829 you state that the “possibility [of owners feeding unconventional dog food being sceptical about visiting the veterinarian or allowing their dog to receive medication] is supported by the fact that there were negative associations between the use of medications and feeding a raw or vegan diet […]“.

It is possible that guardians feeding vegan diets were sceptical about veterinary healthcare but this is very unlikely (other than with respect to anti-vegan dietary advice) – these guardians were more likely than the other two groups to comply with common veterinary advice to have their animals neutered (the opposite was true for guardians feeding raw meat, who were less likely to have their animals neutered, and also much less likely to visit vets, and who are also known to be more likely to gather information from non-veterinary sources). Hence if including this comment (‘possibility X is supported by Y’), then for balance you need to add that at least in the case of vegan diets, lowered rates of medication use may simply reflect better health (i.e. this is another possibility supported by these same results).

2. In line 844 you state the “vegan diet variable was only the 7th most important predictor variable“. Please clarify this statement referring to the absolute effect size / effect strength and not the relative rank in effect sizes. If 10 variables all had really strong effects the 7th would also have a strong one, so referring only to “7th most important“ doesn’t provide the necessary information.

7. *Reproducibility of your results*

I would highly appreciate if the R code for all analyses would be published along with the manuscript.

**Minor feedback**

1. Medication terminology: terminology ‘on medication’ or similar has been changed in many locations (incl. table and supplementary tables) to ‘prescribed medication by vet’. This reflects an incorrect assumption that all medication received must have been prescribed by a vet. This is not the case. Regardless of whether or not they should do so, pet guardians can and do receive and dispense medication, from sources other than veterinary prescriptions, e.g., online vendors. The actual question asked in the survey was “Has your animal received medications in the last year, apart from routine vaccinations and treatments for skin or internal parasites, such as fleas, ticks, lice, heartworm and intestinal worms? Please do not include neutering operations (spay/castration) or microchipping.” Hence all of this text should be changed back.
2. Line 43: please correct the wording of the sentence (“… albeit having limited although their importance was limited.“)
3. Line 93: change “and well as“ to “as well as“
4. Line 224: Ordinal logit models were either estimated with function MASS::polr or VGAM::vglm. I assume one of these functions was used for estimating the partial proportional odds models when the proportional odds assumption wasn’t fulfilled? Please clarify this in the text.
5. Line 324: “dog“ is a typo and should be ”dogs“
6. Line 374: change “been“ to “between“
7. Line 628: change “regressions analyses“ to “regression analyses“
