# Supplementary material for: Variables associated with owner perceptions of the health of their dog: Further analysis of data from a large international survey
Source: PLoS One. 2024 May 15;19(5):e0280173. doi: 10.1371/journal.pone.0280173 (PMC11095744; doi:10.1371/journal.pone.0280173)
Supplement: Attachment — Submitted filename: Response to Reviewers.docx [file pone.0280173.s028.docx]

**Response to reviewer comments**

**PONE-D-22-34984**

**Variables associated with owner perceptions of the health of their dog: further analysis of data from a large international survey**

*Barrett-Jolly and German*

We would like to thank the editor and all three reviewers for their time in reviewing our manuscript and for the constructive comments that have been made. Below, we have addressed all points raised and, where relevant, have also indicated how the manuscript has been revised. Given that, between them, the reviewers had many comments (41 in total!) and our responses are detailed, we have again created a table summarising how each of the points was addressed.

We hope that these explanations, responses and corrections are acceptable.

**Summary of point-by-point responses**

| **Editor / reviewer comment** | | **Author response** | **Changes made** |
| --- | --- | --- | --- |
|  | |  |  |
| *Reviewer 1* |  |  |  |
| 1.1 Again, no changes requested. | | Acknowledged and thanked reviewer for their second review | No changes needed. |
|  | |  |  |
| *Reviewer 2* | |  |  |
| 2.1 Statements in the abstract suggesting that the results conflict with previously-published research are incorrect. | | - Acknowledged this point | Corrected text in both abstract and conclusions from ‘conflict with’ to ‘extend’. |
| 2.2 Acknowledged the previous detailed point-by-point response fully explaining the original revision. | | - Thanked the reviewer for this point. - Stated that we have attempted to do the same for the current review | Detailed point-by-point response created with responses and changes explained for the 37 reviewer comments that were made. |
| 2.3 Reviewer apologised for their previous misunderstandings but explained that they had continuing issues with statistical methodology. | | - Thanked the reviewer for their apology. - Acknowledged the complexity of the original manuscript, which might have contributed to their misunderstanding. - Taken all new comments in good faith and have provided detailed responses and as well as manuscript amendments to address them. - Where we disagreed, we have justified our position, but have mostly still made the changes requested in an effort to reach a compromise position with the reviewer. | Complete redraft of the paper which includes new statistical analyses as requested by the reviewer. Given the increased detail, the ordinal LR has been removed as results were similar to the binary LR and made the revised manuscript unnecessarily long. |
| 2.4 Use of healthcare variables outcomes as independent variables. | | - Noted that this reviewer comment contradicts the position they take in a later point (2.6) where they argue against excluding variables from analyses because it might mean that important correlations are not accounted for. - Provided further explanation and justification for why healthcare variables are valid predictor variables because they differ from the outcomes (owner perceptions of health) and would be considered by owners when forming these impressions. - Explained in detail, using diagrams, the impact of not including these variables in modelling, in that they create more unexplained variance in the model and cant lead to erroneous estimates of the effects of other variables - Accepted that a compromise was needed with the reviewer to reach a position on which we could both agree. - As result, additional analyses are now reported for models which include only the owner-animal metadata. | Multiple LR analyses now reported in the supplementary information, for models using only owner-animal metadata.  Methods and results amended to report on these analyses and compare findings with models containing healthcare variables.  Reasons for differences also considered in the discussion. |
| 2.5 Consider performing a sensitivity analysis by repeating multiple LR after leaving healthcare variables out. | | - This point is follows on the previous one. - Have complied with this request (see above), so as to reach a compromise with the reviewer. | As above |
| 2.6 Model selection process might have missed variables of potential importance. | | - Noted that this point contradicts a previous point (2.4) where they argue for the removal of some variables, because their inclusion may hide correlations amongst other variables. - Acknowledged the fact that the reviewer commended our statistical approach of using two different methods for analysis (LR and XG Boost). Reiterated our reasons for doing this. - Disagreed with the reviewer’s comment that the selection process might have meant important variables were unfairly excluded. - However, acknowledged need for compromise and, therefore, have amended the statistical analysis so that the stepwise regression starts with a model containing all variables. - Noted that the final multiple LR models remain the same. - Further, to ensure that the *dog diet vegan* variable had not been erroneously removed during the selection process, the effect of adding this variable to best-fit models has now been tested; are now shown as an additional model; for comparison, the effect of adding the *client diet vegan* was also tested. *[Note that was done in the original manuscript, but the result was not actually shown].* | Stepwise multiple LR analyses revised so that they start with models containing all variables (rather than using a threshold for inclusion of variables).  New models where either *dog diet vegan* or *client diet vegan* were added to the best-fit model are now reported. |
| 2.7 Remove most of the logistic regression models and focus on XG Boost instead. | | - Noted that this point was related to the previous one where the main concern of the reviewer was inappropriate selection of initial variables for the stepwise regression. - Noted that this issue had been addressed by removing the threshold for inclusion and starting with a model containing all variables (above), meaning that retaining the stepwise regression results could be justified. - Acknowledged that it can be confusing for readers given the many models reported, and this needed to be addressed, with changes including removal of the ordinal LR (to reduce the number of models reported) and rewording for clarity. | Changes made to the stepwise regression process as described above. Ordinal regression results removed and methods and results reworded to improve clarity. |
| 2.8 Consider always forcing the XG Boost models to retain the dog diet variable so an estimate of its impact can be provided. | | - Clarified that XGBoost selects model features in an unbiased manner and, therefore, coercing it to ‘fix’ a particular variable in the model is inappropriate. - However, the number of trees used in modls can be increased, thereby increases the chances that a particular variable (e.g., *dog diet vegan*) is retained. This has now been done. | XGBoost hyperparameters changed to encourage use of more trees, thereby increasing the number of features as far as possible without severe decrease in model performance.  *Dog Vegan Diet* does now appear in most models. |
| 2.9 Continuing concern over removal of data from the 111 owners who were not primary decision makers | | - Acknowledged the reviewer’s acceptance that data from owners who are not primary decision makers might be unreliable. - Explained that we have now assessed the effect of ‘decision maker status’ in our statistical analysis and there is a significant association between this and the *any health problem* binary variable, thereby confirming material differences as a result of decision-maker status, justifying their exclusion. - Explained in detail, using diagrams, how decision-maker status might adversely affect results, and how removing owners who are not primary decision makers can address this concern without impact on other variables. - Despite this, the need for compromise was accepted. - As a result, further analyses conducted on the dataset with all owners, which are now compared with those of the data from primary decision makers only. | Methods and results altered to provide further details of how the issue of decision-maker status was addressed including analyses using a new decision-maker status variable.  Additional analyses conducted on the dataset of all owners, and included in the supplementary information.  Discussion also amended to explain the new results and consider the impact of focusing on primary decision makers. |
| 2.10 Too much detail provided about why the authors of the original study previous had not been contacted. | | - Reviewer thanked for the suggestion. | Text amended to explain the reasons for not contacting the primary authors more concisely. |
| 2.11 Lack of clarity about how influential datapoints were handled. | | - Accepted that the approach lacked clarity and that further details were needed. | Methods section updated to explain the approach to influential datapoints more clearly. |
| 2.12 Concern that the UMAP might be too complex for a generalist reader and findings are minimally interpretable. | | - Accepted that generalists might be less familiar with UMAP compared with other visualisation methods. - However, its use can be justified because of its strengths (use of different date types, ability to reduce dimensions of a multivariate dataset, non-linear technique, improved performance over methods such as PCA). - Therefore, accepted the need to explain both the technique better (methods) and what the results show (results). | Methods section updated with a more detailed explanation of methodology.  Results explained in more detail. |
| 2.13 Question about the share of variability in the first two dimensions of the UMAP. | | - Explained that the reviewer might be confusing the UMAP output with that from a PCA; with UMAP, only two dimensions “x” and “y” are used. With PCA, there are many but the top 2-3 are displayed by convention. Therefore, all variability is captured in these to dimensions. | This is now explained more clearly in the methods and results. |
| 2.14 Include a statement about the goodness of clustering in the UMAP. | | - Explained Clustering was performed by DBSCAN and, therefore, the clustering function using kmeans was not needed. - However the reviewer is correct that performance silhouette plots and metrics can be still be calculated after with clustering has been generated by DBSCAN. | We now calculate and include a full silhouette analysis (including a new figure), and also calculate the model independent Davies-Bouldin’s index (a cluster separation measure). |
| 2.15 Clarify what the bubbles in the correlation matrix mean. | | - We agree that the meaning of the bubble size and colour intensity and size were not defined, and that variables below the FDR-corrected *P*-value threshold were not shown. - Stated that we are happy to remove the threshold to show bubbles. - Noted that, despite this, some bubbles are not clearly seen because the correlation coefficient is so small (meaning tiny bubbles/ colour intensity). | Figures updated and an explanation provided in figure legends as to what the bubbles show. |
| 2.16 Consider removing the FDR-based threshold for including bubbles in the correlation matrices. | | - Disagree with the reviewer about removing FDR, which was used to correct for multiple comparisions; given the number of comparisons, such a correction is considered to be good statistical practice. - However, showing more bubbles in the correlation matrix would be useful for readers (see above). - Agreed that it would be helpful to have the absolute *P*-values in the results text (as well as log*P*). | We have removed the threshold for bubble inclusion in the correlation matrix, but continue to use FDR to correct for multiple comparisons.  Added absolute *P*-values to the text in the results, whilst keeping log*P* in supplementary information. |
| 2.17 Issues with tests used to determine goodness of fit. Report AUC values (based on either cross-validation or a hold-out test set) for logistic regression models. | | - Disagreed with the suggestion that AUC was now the preferred measure of LR model performance and emphasised that different metrics assess performance in different ways (generalisability, amount of variance explained, prediction accuracy). - Acknowledged need for compromise and, therefore, have decided to include ROC curves and AUC created for all logistic regression models. - For the primary decision-maker dataset, and as requested, the ROCR and AUC are calculated from a hold-out test set, which necessitated the splitting of the original dataset during the data pre-processing stage. For completeness, ROC and AUC for both training and test datasets are shown in statistical reports (and are generally similar). - ROCR and AUC have also been generated for the new LR regressions on the all-owner dataset (added in response to point 2.9). However, because we wanted to ensure the largest dataset for training, we did not split it into training and test sets. Therefore, these AUC are from ROC curves on the original dataset. The over-estimate is expected to be small given the similarity between training and test AUC from the models using the primary decision-maker dataset. | Methods updated to explain the creation of ROC curves and AUC for logistic regression.  All results and tables amended to include AUC, and AUC also used when comparing performance of different models. |
| 2.18 Query over interpretation of associations between dog diet and healthcare variables in the discussion. | | - Acknowledged that the original discussion was misleading and did not adequately differentiate between possible effects for raw-fed and vegan-fed dogs. - Also acknowledged the reports reported by Knight et al where there were differences amongst diet types in the proportion of dogs that were neutered. - As a result, a correction to the statements is required. | Text revised to explain better the associations between healthcare variables and diet type (now differentiating raw and vegan), as well as the need for further research. |
| 2.19 Report absolute effect size for variables (such as dog diet vegan) for the XG Boost rather than just order of importance. | | - Agree with this suggestion. - Explained that XGBoost uses 3 metrics to rank the effect of ‘features’ (variables) in the model: importance, cover and frequency. These were shown in the figures, where variables were ranked using a sum of these metrics (stacked columns). - However, accepted that we did not provide any results for these metrics in the results. | In the results section, we now report the actual values for these metrics for the most important variables, and also for the dog diet variables. |
| 2.20 Acknowledgement that publishing R code would be helpful. | | - Confirmed our intention to publish all could as well as our statistical reports. | No changes required. |
| 2.21 Correct the wording in line 43. | | - Acknowledged and apologised for the error | Wording corrected. |
| 2.22 Clarify terminology on use of medication (‘received medication’ cf ‘prescribed medication by vet’. | | - Acknowledged and apologised for the error | Wording corrected. |
| 2.23 Correct the wording in line 93. | | - Acknowledged and apologised for the error | Wording corrected. |
| 2.24 Clarify packages used for ordinal regression. | | - Acknowledged and apologised for the error | Wording corrected. |
| 2.25 Correct the typo in line 324. | | - Acknowledged and apologised for the error | Wording corrected. |
| 2.26 Correct the typo in line 374. | | - Acknowledged and apologised for the error | Wording corrected. |
| 2.27 Correct the typo in line 626. | | - Acknowledged and apologised for the error | Wording corrected. |
|  | |  |  |
| *Reviewer 3* | |  |  |
| 3.1 Summary about the methods and results of the paper, with an acknowledgement that providing statistical code was helpful, but stating that there were some concerns with the approach. | | - Thanked the reviewer for their detailed review and constructive suggestions. - Explained that we have attempted to address all points and made changes as a result, which are explained in detail in the point-by-point document. | Manuscript extensively revised to address the concerns raised by the reviewer, including reporting new statistical analyses. |
| 3.2 Concern that categorical variables were handled as numerical variables in the UMAP. | | - Accepted that the methods were unclear, not least regarding how variables were handled. - Explained that UMAP can handle categorical variables, with the correct distance metric (Hamming). - However, realised that there was an error with our original statistics in that appropriate direction metrics (e.g., Hamming) had not been used for categorical variables. This has now been corrected with new analyses, that have changed the results slightly. - Also, accept that the methods and results needed to be explained more clearly. | Repeated UMAP using the correct distance metrics (e.g., Hamming for categorical data).  Updated methods to provide a better explanation of methodology.  Updated results, which are now slightly different. |
| 3.3 Question about the use of healthcare variables as predictor variables rather than as outcomes. | | - Acknowledged the comment and the need to clarify as well as the approach taken for the healthcare variables. - Accepted that both the outcome variables (owner perceptions of health) and the healthcare variables would be associated with actual dog health. - However, emphasised that they measure different things (perceptions of health vs factual knowledge about aspects of vet care the dog receives). - Emphasised that our paper was specifically interested in perception of health rather than actual health. - Explained that healthcare variables would form part of the knowledge base that an owner would use when forming their perception of their dog’s health; as such, their inclusion as predictor variables can be justified. - Clarified the statistical methods used to ensure that their inclusion did not have an adverse effect on the other variables (VIFs). - Nevertheless, acknowledged the need for a compromise. - Therefore, new statistical analyses performed where healthcare variables have been excluded. These are now reported alongside the existing models (where healthcare variables are included). - Expressed the hope that the reviewer would accept this compromise. | Methods, results and supplementary information have been updated to include the new statistical models where only owner-animal metadata are used as predictor variables. Results are compared with models where healthcare variables are included, and reasons for differences considered in the discussion. |
| 3.4 Correct the wording in lines 43-44. | | - Acknowledged and apologised for the error. | Wording corrected. |
| 3.5 Correct the wording in line 50. | | - Acknowledged and apologised for the error. | Wording corrected. |
| 3.6 Correct the error over reversal of numbers for vegetarian and vegan diets. | | - Acknowledged and apologised for the error. | Numbers corrected to ensure they are accurate. |
| 3.7 Inconsistency in the number of owners reported as vegan in text and tables. | | - Acknowledged errors with the figures and apologised for these. | Results section revised so as to indicate study numbers more clearly.  All cluster results for the UMAP have now been updated. |
| 3.8 Question about why processed variables not used for UMAP. | | - The UMAP was undertaken before the categorisation of most variables, and hence used the initial dataset. - This was because it is intended to be a global visual overview of the non-health metadata prior to any significant pre-processing. - Accepted that this was not clear and needed to better explain this in both the methods and results. | Methods revised so as better to explain the data pre-processing stages, and where the UMAP fitted.  Results updated so that the numbers used for the UMAP were clear. |
| 3.9 Question about how diets for those on a therapeutic diet were handled. | | - Explained that the handling of therapeutic diets was explained in the original (Knight et al) study. - Specifically, for those dogs on a therapeutic diet, owners were asked to consider the diet they fed before switching the diet. - Accepted the need also to make this point clear in our manuscript. | Methods amended to clarify how the use of therapeutic diets were handled as part of the original study. |
| 3.10 Only report the version of the variables selected for LR in the tables and results. | | - Accepted that it would be sensible only to report the versions of the variables that were selected for LR modelling. - Explained that it would be good to make an exception for *dog diet vegan* and *client diet vegan* because these had particular prominence in the original study, not least given the additional analyses reported (where these variables are added back into the best-fit models). - Expressed the hope that the reviewer would be happy with this compromise. | Tables, figures and results amended so that only the final variable choices are shown. |
| 3.11 Inconsistencies in the variables selected for multiple LR and those that were eligible from the simple LR. | | - Acknowledged and apologised for this error. However, the text in question has now been removed as the approach to variable selection for the stepwise regression has changed in response to a comment from reviewer 2 (where we now start with a model containing all variables). - Also acknowledged that the use of the giant breed variable was not properly explained. | All tables and results checked to ensure consistency. Further details provided in the statistical methods to explain and justify use of the giant breed variable |
| 3.12 False negative rate should be false-positive rate | | - Acknowledged and apologised for the error. | Corrected in the revised manuscript. |
| 3.13 Incorrect statement that dog diet was not associated with the significant illness outcome when raw diet was. | | - Acknowledged and apologised for the error. | Results revised and corrected to ensure that the text is accurate and consistent with results in the tables. |
|  | |  |  |

**Reviewer 1 comments**

*No comments*

***Authors’ response:***

Once again, we would like to thank this reviewer for the time taken to review the revised manuscript. We are happy that they are happy with it and are recommending publication.

**Reviewer 2 comments**

**Reviewer comment 2.1**

*The authors of this current study responded to our prior Knight et al (2022) study (https://doi.org/10.1371/journal.pone.0265662), after reanalysing the dataset we collected, which is available open access.*

*Conclusions and Abstract revisions*

*Re: “The results of the current study conflict with previously-published research using the same dataset” (abstract) and “These results conflict with previously-published research” (Conclusions).*

*These statements are not correct, because (i) the authors have agreed (in several locations) that the results of their preliminary, simple (i.e., univariable) regression modelling were in fact similar to results from the previously-published research (Knight et al. 2022), and (ii) the previously-published research did not undertake multiple regression or XGBoost analyses as the authors have done, and so did not have any comparable results to compare with their own research.*

*The authors correctly noted in the Abstract, “The aim of the current study was to extend the earlier findings, using different modelling techniques and including multiple variables, to identify the most important predictors of owner-reported dog health.”*

*Hence, statements that “The results of the current study conflict with previously-published research…” are incorrect. They should be deleted or modified to clarify that the current study provides additional research findings, examining different aspects (e.g., guardian location), that extend the results of the previously-published research.*

Many thanks for this comment. We have amended the text as follows:

**Abstract**

“The results of the current study extend the previous research using the same dataset and suggest that diet has limited impact on owner-perceived dog health status…”

**Conclusions**

“These results extend previously-published research [15] and suggest that…”

**Reviewer comment 2.2**

*Statistical analysis*

*First, I thank the authors for answering all prior critique points in an unusual level of detail. I highly appreciate the effort put into this, including the compact overview table, facilitating the revision process, given that so many different points are discussed. This was very helpful.*

Many thanks for acknowledging the considerable work that was required in creating the original point-by-point response. In this second review, many new concerns have been raised that, arguably, should have been included first time around. Nonetheless, we have taken them in good faith and have done our best to address them. As with the last round, we have been as detailed as possible in our responses and, once again, have included a summary table. We hope that this helps clarify exactly where we agree and disagree, as well as how the manuscript has been revised.

**Reviewer comment 2.3**

*Regarding my original comments #2, #3, #6 and #8:*

*Thank you for clarifying that the results I was referring to came from the univariate regression models. I apologize for my misunderstanding and also appreciate that you now have clarified the univariate character of the results in the respective table captions. The character of the model selection process and how differently encoded variables are handled is now explained in a significantly clearer way, within the manuscript.*

*Nonetheless, very central issues remain, with the statistical methodology of the study.*

We accept that the original paper was complex, not least given that we used several methods (ordinal logistic regression, binary logistic regression, XG Boost etc) to analyse the same outcome variables. As a result, some misunderstandings are understandable, and we are glad that these have now been clarified. That said, and echoing a point the reviewer makes later, we accept that the revised manuscript was still very complicated. Further, the new changes required for this review round have added even more complexity. As a result, we have decided to remove one of the modelling approaches originally used (ordinal logistic regression). We felt that using three separate approaches to assess the same outcome measures (ordinal LR, binary LR and XGBoost) was excessive. Removing the ordinal LR does not detract from the paper because the findings from this approach were similar to those of binary logistic regression, but model fit was worse. Further, the advantage of removing the ordinal LR is that the additional analyses the reviewer requests are easier to accommodate.

Removing some of the logistic regression analyses is also a compromise for one of the points this reviewer makes below about removing all logistic regression modelling. Whilst we do not agree with this position, as explained below, removing some of the regression modelling is a compromise that we hope the reviewer will accept.

**Reviewer comment 2.4**

*Major statistical issues requiring revision*

*1.	Use of health outcomes as independent variables*

*Thank you for the insights given in the answer to comment #9. Perhaps I didn’t phrase my initial comment clearly enough. What I see as being critical, is not a potential correlation between health outcome variables, but the inclusion of such health outcome variables as independent variables in the models per sé, potentially leading to hidden correlations caused by other variables. It could be that one variable (e.g., diet) is affecting both your response variable (overall health) and the other two health variables (vet visits and medication), but this effect is not visible in the final models because it is hidden by the correlations between the health variables. You correctly state this issue yourself in line 731. Hence I would suggest removing health outcome variables from the independent variable list.*

Before addressing this comment, we note that it contradicts a later point from the same reviewer (2.6. “model selection process”). For this comment, they express concerns about including some variables (ie those associated with healthcare), given that doing this might *“potentially lead [sic] to hidden correlations caused by other variables.”* However, in the later comment, they express concerns about excluding variables from the initial stepwise multiple LR because this could, *“potentially miss individual variables which may not be included in the stepwise procedure.”* It is unfair to argue for limiting the inclusion of variables they do not like in this comment, whilst arguing against limiting inclusion of other variables in the later point. Despite this obvious contradiction, we have taken the comment in good faith and done our best to address it below.

First, we thought it best to clarify our rationale for including the healthcare variables as predictors. The primary aim of our study was to explore associations with *owner perceptions about the health of their dog*. It was for this reason that we chose the data from question 18 of the original study: *“In your own opinion, which best describes your animal over the previous 12 months”*, rather than the other health-related variables (owner opinions of their veterinarian’s opinion, number of veterinary visits, receiving medications etc).

As the reviewer correctly states, there are likely to be many possible associations amongst variables in the dataset, as illustrated by the following diagram:

Clearly, one factor associated with owner opinions of health is the actual health of the dog; however, the effect of this outcome variable will be influenced by the knowledge and recollections of owners (which is pertinent to decision-maker status; see below), and owner perceptions of health will also be affected by their beliefs and attitudes (for example on the type of diet that they believe is ‘healthiest’). As an aside, dog diet is unlikely to have a **direct** **effect** on the healthcare variables as the reviewer suggests; instead, its effect would be mediated indirectly through effects on the actual health, which in turn would affect what healthcare is necessary. So, the inclusion of healthcare variables will not necessarily eliminate the diet effect as suggested.

The inclusion of healthcare variables as predictors in our models on owner perception of health is justified by the fact that they measure different things. In contrast to owner perceptions of health, the healthcare variables were derived from questions in the original survey that required factual recall, for example:

- *“Has your animal received medications in the last year, apart from routine vaccinations and treatments for skin or internal parasites, such as fleas, ticks, lice, heartworm and intestinal worms? Please do not include neutering operations (spay/castration) or microchipping.”*
- *“How many times has your animal visited a veterinarian (or received a vet home visit) in the previous 12 months?”*

Such responses do not require an owner to form a subjective, ‘holistic’ opinion about their dog’s health and, therefore, they **do not** measure the same thing as the owner perception of health variables. Further, these variables will form part of the knowledge base that an owner will call on when formulating their opinion about how healthy their dog is. For this reason, it is important for these variables to be included within in the modelling, and excluding them can itself be problematic. In this respect, any effects on owner perception of dog health resulting from the healthcare variables will remain in the system, irrespective of whether they are included as predictors in a particular model, as illustrated in this next diagram:

Put another way, leaving the healthcare variables out of a model will not actually remove their associated variance; instead, we are simply ignoring it in the statistical modelling. This increases the amount of variance that is not properly accounted for, leading to a model that performs poorly (because of this ‘unexplained’ variance), with the added danger that some of this variance might be inappropriately attributed to variables that are included, leading to erroneous estimates of their regression coefficients. This will skew interpretation of the actual impact of owner-animal metadata variables, since we are wrongly assuming that healthcare matters had had absolutely have no impact on these opinions.

A further point of note; in this comment, the reviewer has reiterated their concern that corelations between the healthcare variables and other variables (such as dog diet) could mean that the correlations between these other variables and the outcome measure are ‘hidden’. There are two solutions to this, the first being to investigate for such ‘hidden correlations’ (aka multicollinearity) using variance inflation factors (VIFs). We addressed this in our previous point-by-point response, but this was not acknowledged by the reviewer. Therefore, to reiterate, VIFs determine the extent to which a particular variable is ‘inflated’ (or ‘deflated’) by inclusion of other variables in the model. Since all VIFs were within acceptable limits, there is **no evidence** of significant multicollinearity in any of our final models (see the statistical reports). Whilst we continue to acknowledge that this does not altogether eliminate any possibility of variance being incorrectly assigned amongst variables, it reassures us that any such misclassification is likely to be minimal.

Further, a second solution to the problem of multicollinearity is to perform statistical analyses using methods that are better able to handle complex interactions amongst variables, for example XG Boost. In fact, we made this exact point immediately after the sentence the reviewer refers to above:

*Therefore, a downside of including the healthcare variables in multiple logistic regression analyses is that their presence might have adversely affected the associations seen with the owner-animal metadata leading to biased models.* ***Arguably, however, the use of XGBoost should offset the limitation of confounding from healthcare variables given that these techniques do not make prior assumptions about data distribution and can take account of complexity in interactions amongst variables [69].***

By using VIFs in multiple regression as well as using XG Boost, we believe that we did take sufficient care to avoid possible issues with multicollinearity in our models.

It is likely that we will never reach complete consensus with the reviewer on this point. Given that we accept the need to compromise, we have nonetheless complied with their request and have now performed additional multiple logistic regression models which only contain the owner-animal metadata variables, as discussed below. These models have been included as supplementary information but are also referred to in the methods, results and discussion as described in our next response (2.5), below. We hope that the reviewer will accept this compromise.

**Reviewer comment 2.5**

*Alternatively, given a key aim of this study (analysing the effect of diet on overall health), you at least need to perform a so-called ‘sensitivity analysis’ to check if any indirect effect/hidden correlation is present. For such a sensitivity analysis you could re-estimate your model selection approach by leaving out the health outcome variables from the independent variable list, reporting these results in the Supplementary materials and stating any identified potential differences in the manuscript.*

Thank you for this comment. It should first be noted that, in the original manuscript, we reported the results of all simple regression analyses, enabling the reader to see the (unadjusted) effect of each predictor variable. In addition, two of our original XG Boost models only contained *owner-animal metadata* variables (original Figs 6 and 9). As the reviewer acknowledges, this approach is a better able to handle complex interactions amongst predictors variables. The fact that these models performed poorly in prediction confirms our original conclusions that the influence of diet choice on owner opinions of health are negligible.

That said, it was an oversight not to have included multiple LR models using only the owner-animal metadata (and not healthcare variables). Therefore, in the spirit of compromise, we now include such models as supplementary information (S6 Fig, S8 Fig). In the revised manuscript, we compare these models with the equivalent multiple regression models containing all variables in terms of which variables have the strongest association with the outcome measures and model performance. As with the XG Boost models we originally included, the owner-animal metadata models perform poorly. These additional statistical analyses have required us to revise our manuscript in the following ways:

- First, we explain these additional analyses in the results, for both the *any health problem* binary variable:

Next, we created two multiple regression models, again on data from primary decision-makers, with *any health problem* binary as the outcome variable and that either contained all predictor variables or just the owner-animal metadata. For the *all-variable* model (S3a Fig), the variables with the strongest associations with the outcome variable were *dog age* (both the ‘1-5y’ and ‘6-20y’ categories), *received medication*, *veterinary visits* (the ‘1 visit’, ‘2 visits’, ‘3 visits’ and ≥4 visits’ categories) and *switched to a therapeutic diet*. In terms of model performance, pseudo-R^2^ was 0.439, BIC was 1836 and AUC on the test dataset was 0.852 (S5 Fig). For the *owner-animal metadata* model (S3b Fig), the variables with the strongest associations with the outcome variable were *owner age* (‘50-59y’ and ‘≥60y’ categories), *dog age* (‘6-20y’ category), *breed size category* (‘giant breed’ category) and *dog diet* (‘vegan diet' category). However, performance of this model was considerably worse in terms of generalisability (BIC 2244), the proportion of variance explained (pseudo-R^2^ 0.150) and prediction accuracy (AUC on the test dataset: 0.668).

- and also for the *significant illness* variable:

Next, we created both an *all-variable* and an *owner-animal metadata* model, using data from primary decision-makers, with *significant illness* binary as the outcome variable. For the *all-variable* model (S4a Fig), the variables with the strongest associations with the outcome variable were *dog age* (‘6-20y’ category), *giant breed*, and *received medication*. In terms of model performance, pseudo-R^2^ was 0.403, BIC was 679 and AUC on the test dataset was 0.890. For the *owner-animal metadata* model (S4b Fig), the variables with the strongest associations with the outcome variable were *dog age* (‘6-20y’ category) and *giant breed*. Once again, the performance of this model was considerably worse in terms of generalisability (BIC 799), the proportion of variance explained (pseudo-R^2^ 0.124) and prediction accuracy (AUC on the test dataset: 0.692).

- We have also expanded the discussion section to consider these new results as well as to explain the reasoning for including the healthcare variables:

The approach of the current study also differed by our decision to include healthcare variables (*received medication*, *veterinary visits*, *switched to a therapeutic diet*) as predictors. In the previous study [15], these variables were instead used as proxy measures for dog health and compared amongst dogs fed different diet types. Whilst both owner perceptions of health and healthcare variables will be associated with actual dog health, they are materially different. As already discussed, owner perceptions of health were derived from a question where owners subjectively rated their dog’s health [15], and these perceptions will be influenced by other factors including owner knowledge and recollections, as well as their beliefs and attitudes (Fig 9). Conversely, the healthcare variables were derived from survey questions that required factual recall rather than the formulation of a subjective, holistic opinion about health. For example, owners were asked whether their dog “had received medications in the last year” and how many times the dog had “visited a veterinarian or received a home visit” [15]. Given that such healthcare information will contribute to the knowledge an owner utilises when constructing opinions about the health of their dog, we reasoned that they were valid predictor variables in our statistical modelling. Further, excluding them from statistical models could be problematic as illustrated in Fig 9c; unlike removing observations from owners who were not primary decision-makers (as discussed above), where any variance associated with the variable is removed, simply excluding a variable from a statistical model will not eliminate any associated variance. The ‘hidden’ variance associated with the healthcare variables might then be incorrectly attributed to other variables leading to erroneous estimates of their regression coefficients. Any unassigned variance also adversely affects model performance, which likely explains why the models that only included owner-animal metadata performed poorly as discussed below.

**Reviewer comment 2.6**

*2.	Model selection process*

*It is commendable that you utilized two different modelling approaches (classical regression estimation and the boosting approach) to more robustly analyse the data. However, from a statistical point of view, the variable selection approach used for the regression modelling is not state-of-the-art anymore. The main problem is that you base the initial decision on ‘which variables should be included within the available set of variables’, on univariate regression model results, which you yourself state are (potentially) wrong. In this way, you potentially miss individual variables which may not be included in the stepwise procedure.*

As mentioned above, this comment contradicts the reviewer’s earlier comment (2.4) about the inclusion of variables in multiple LR models. Previously, they expressed concerns that including some variables, could hide effects in the result models; here, they are now criticising being selective about variable inclusion because this might be important effects are missed! Once again, however, we have ignored the contradiction, taken the comment in good faith and done our best to address.

First, many thanks for commending our approach of using two different statistical approaches: an established method (LR) and a state-of-the-art, machine-learning method involving boosting. We agree with the reviewer that this ensures that our data analysis is robust. For example, although automated modelling approaches may be better able to spot complex patterns within data sets, they have their weaknesses, not least that they do not take account what is biologically plausible or clinically relevant. This is exactly why we chose to complement XGBoost with a supervised stepwise logistical regression approach. Overseeing the stepwise selection process manually might be laborious but it can ensure that biological and clinical relevance are properly considered. Multiple logistic regression remains widely respected by statisticians and is still commonly used in both biomedical and veterinary research and we disagree with excluding it.

In this comment, the only reason that the reviewer gives for criticising multiple regression (and suggesting it be removed) is the initial step of deciding which variables to include, because this might have *“potentially missed [sic] individual variables which may not be included in the stepwise procedure.”* We disagree with this comment, as the threshold for selection we chose (*P*<0.2) is a ‘low bar’ for variable inclusion. Nonetheless, given the reviewer’s concern, we have decided to remove it and, instead, now start the stepwise elimination process with the all-variable model. Not surprisingly, the variables we had previously excluded were eliminated during the stepwise process and, as a consequence, the final best-fit models for both outcome variables remain the same.

The revised manuscript has been altered as follows:

**Methods**

In the first step of the multiple logistic regression modelling, two multiple regression models were created for each outcome variable (*any health problem*, *significant illness*); all predictor variables were included in the first of these models (*all-variable* model), whilst only owner and animal variables were included in the second model (*owner-animal metadata* model). The performance of *all-variable* and *owner-animal metadata* models were then compared with each other and with the *best-fit* models that were created subsequently. For these *best-fit* models, we used a supervised, stepwise approach starting with all variables. This initial model was manually refined in both a backwards and forwards stepwise fashion, with the BIC being used to select the model within the same family with the best generalisability (a measure of its goodness of fit compared with its complexity) [60]. Using this approach, variables could be added or removed until the model with the smallest BIC was found, according to previously published rules [61], whereby model generalisability was deemed to be superior if the BIC of the new model was less than the previous model by at least 2 units.

***Results (any health problem outcome variable)***

Supervised backwards and forwards stepwise regression was then performed to create a best-fit multiple regression model for the *any health problem* binary outcome variable, starting with the model containing all variables. After refinement by backwards and forwards stepwise elimination, the best-fit model contained 4 variables: *dog age*, *veterinary visits*, *received medication* and *switched to a therapeutic diet* (Fig 3a; model 1, S5 Table). Compared with the all-variable model, generalisability was markedly improved (BIC 1656), with only slight reductions in both the amount of variability explained (pseudo-R^2^ 0.420) and prediction accuracy (AUC for the test dataset:0.842).

***Results (serious illness outcome variable)***

Supervised backwards and forwards stepwise logistic regression was again used to create a best-fit multiple regression model for the *significant illness* outcome variable, starting with all variables. After refinement by backwards and forwards stepwise elimination, the best-fit model contained 3 variables: *dog age*, *veterinary visits* and *received medication* (Fig 6a; model 1, S5 Table). Compared with the *all-variable* model, generalisability was markedly improved BIC 517), with better prediction accuracy (AUC for the test dataset: 0.903), and only a small decreased in the amount of variability explained (pseudo-R^2^ 0.342).

Finally, to be absolutely certain that we did not inadvertently remove the *dog diet vegan* variable during stepwise selection of variables, we added this variable back into the final best-fit models and determined the effect. This was something we had done for the original manuscript (for example: *“Inclusion of any of the other variables identified on simple regression, either individually or in combinations and with interactions, led to models with worse generalisability.”*). However, in the spirit of full disclosure, we now report these results in the manuscript (results, figures and supplementary information). For comparison, we have also included models where *client diet vegan* is instead added to the best-fit models. The corrected text reads as follows:

**Any health problem outcome variable**

As an additional multiple logistic regression step, the effect of adding the *dog diet vegan* variable to this best-fit model was assessed (Fig 3b; model 2; S4 Table); generalisability was worse (BIC 1660), whilst the amount of variance explained (pseudo-R^2^ 0.422) and prediction accuracy (AUC for the test dataset 0.845) were similar. Equivalent results were obtained when the *owner diet vegan diet* variable was instead added to the final best-fit model (BIC 1660, pseudo-R^2^ 0.421, AUC 0.844; Fig 3c; model 2; S4 Table).

**Serious illness outcome variable**

As a further multiple logistic regression step, the effect of adding the *dog diet vegan diet* variable to this best-fit model was assessed (Fig 6b; model 2; S6 Table); generalisability was worse (BIC 524), whilst the amount of variance explained (pseudo-R^2^ 0.342) and prediction accuracy (AUC for the test dataset 0.904) were similar. Equivalent results were obtained when the *owner diet vegan diet* variable was instead added to the final best-fit model (BIC 524, pseudo-R^2^ 0.342, AUC 0.903; Fig 6c; model 2; S6 Table).

We believe that all these changes now fully address the specific concern of the reviewer about our selection process potentially missing important variables.

**Reviewer comment 2.7**

*In statistical research, the solution to this exact problem you are facing is exactly the alternative method that you already use: boosting. Boosting is the solution applied when a dataset is too small to estimate a regression model with a lot of variables. In this case, it is the better method compared to any stepwise selection procedure. Accordingly, I suggest dropping the stepwise-selected regression approach (maybe keep something like the ‘all-variable’ model, if you deem it meaningful), and fully concentrate on the boosting approach, as long as you stick to your research question with a bigger set of independent variables. This way, the manuscript will also become much easier to read and follow. While reading the (revised) manuscript, I found it quite hard to grasp which of the models is ‘the final model’ (selected regression model vs. Selected boosting model vs. All-variable model) and is the base for the main interpretation.*

This comment follows on from the previous one, about the selection process for initial inclusion of variables to determine the best-fit model. Given that we have fully addressed this concern (by starting with the all-variable model), we disagree with dropping the stepwise selection approach altogether because it adds useful additional information (namely to highlight which variables are most important in predicting the opinions of owners about the health of their dog). In this respect, it is telling that prediction accuracy for the best fit models (*any health problem* AUC 0.842; *significant illness* AUC 0.903) was very similar to those of the all-variable models (*any health problem* AUC 0.853; *significant illness* AUC 891) despite only including 3-4 variables. It is also noteworthy that the prediction accuracy of our logistic regression modelling was equivalent to that of the XG Boost for both outcome variables (*any health problem* AUC 0.853; *significant illness* AUC 891). Therefore, even though some scientists might suggest these tests as *“not state of the art anymore”*, they performed at least as well as the newer approaches. This conclusively demonstrates that the reviewer’s theoretical concern about the validity of our multiple regression modelling is not supported by the evidence. As a consequence, we strongly refute their recommendation to remove it.

Nevertheless, as discussed above, we do agree that the original manuscript was complicated, given that we used three different modelling methods (ordinal LR, binary LR and XG Boost). We have reflected on this and have decided to remove the ordinal logistic regressions, as well as to rewrite the results to improve clarity further. We hope that this will make it easier for readers to follow our approach and the results obtained.

**Reviewer comment 2.8**

*Nb: As an additional, minor note: in boosting there’s also the option in some packages to fix one independent variable as the main variable in the model. E.g., you could fix dog diet, so that it is always included in the model, in this way ensuring that you end with an effect estimate that you can compare with other publications.*

Many thanks for raising this point. We are sure that the reviewer is already aware that a major feature of the powerful XGBoost machine learning tool is its ability to choose important features automatically (in an unbiased manner) and score their importance. Therefore, the whole point of using it is to allow unbiased selection and ranking of important features. Therefore, whilst it might be acceptable to retain some variables in multiple logistic regression models (as we do for some of our models; see response to comment 2.6 above), coercing XGBoost models to fix a particular feature conflicts with a key reason for using the method and, therefore, is inappropriate.

Not surprisingly, the XGBoost package in R does not offer a way to define what features to include in the final model *a priori*. However, it is possible to increase the number of trees used, thereby increasing the chances that low-importance features will be retained in the final model. We have now have revised all our XGBoost models in this way, and this ensures that the *dog diet vegan* variable is included in most models. The only model where it is still not included is the all-variable model for the *serious illness* outcome because the this is because its contribution is so minimal.

Please note that changing our XGBoost models has altered our results, although they are broadly similar.

Therefore, the revised text for XG Boost in the results section is as follows:

**Machine learning predictive modelling with XGBoost** [*any health problem* outcome]

The ability of owner, animal and healthcare variables to predict the *any health problem* variable was further assessed using machine learning predictive modelling using the XGBoost algorithm. Two separate models were created using data from primary decision-makers; the first contained all variables, whilst the second was a reduced model containing just the owner-animal metadata (i.e., without healthcare variables). Based on ROC analysis, the prediction accuracy of the all-variable model was reasonable (AUC 0.836; Fig 4a). The XGBoost procedure also enables the most important variables contributing to the prediction to be identified, for the all-variable model (Fig 4b); the variables that most strongly predicted the *any health problem* outcome variable were (in order, based on the sum of the 3 metrics): *received medication* (importance 0.393, cover 0.129, frequency 0.065), *dog age* (importance 0.175, cover 0.182, frequency 0.188) and *veterinary visits* (importance 0.153, cover 0.181, frequency 0.155), followed by *owner age* (importance 0.048, cover 0.083, frequency 0.106) and then *breed size category* (importance 0.046, cover 0.070, frequency 0.087). Other variables followed these including *education*, *dog sex*, *location*, and *dog diet*; for the latter variable, *dog diet raw* was 10^th^ in order of importance (importance 0.015, cover 0.026, frequency 0.032), whilst *dog diet vegan* was 15^th^ in order of importance (importance 0.008, cover 0.021, frequency 0.013).

**Machine learning predictive modelling with XGBoost** [*serious illness* outcome]

Using machine learning predictive modelling with XGBoost, and data from primary decision-makers, all-variable and reduced (owner-animal metadata) models were created (S2 File) for the *serious illness* outcome variable. Based on ROC analysis, the prediction accuracy of the all-variable model was reasonable (AUC 0.887; Fig 7a). In order (Fig 7b), the variables that most strongly predicted the *significant illness* outcome variable were: *veterinary visits* (importance 0.530, cover 0.473, frequency 0.285), *dog age* (importance 0.182, cover 0.219, frequency 0.302) and *received medication* (importance 0.175, cover 0.190, frequency 0.143), followed by *breed size category* (importance 0.050, cover 0.034, frequency 0.079), *education* (importance 0.016, cover 0.020, frequency 0.050), *owner age* (importance 0.012, cover 0.014, frequency 0.036) and *dog sex* (importance 0.009, cover 0.010, frequency 0.034)*.* Other variables followed these including *location, urban setting*, *owner sex*, *animal career* and *therapeutic food*. For the *dog diet* variable, *dog diet raw* was 17^th^ in order of importance (importance 6.14 x10^-5^, cover 4.09 x10^-5^, frequency 1.55 x10^-4^) relative importance), whilst the *dog diet vegan* variable was of insufficient importance to be included in the model (i.e., relative importance less than that of the *dog diet raw*).

**Reviewer comment 2.9**

*3.	Exclusion of 111 data points (comment #5)*

*While I see your point that people with less involvement in the dog food purchase decision process might also be less involved/informed in health matters of the dog, and I appreciate the reasoning given in the discussion, deleting data from a study dataset is a very hazardous statistical step and should be treated with great caution. I still disagree with the reasoning to exclude these data points, but could accept this if the effect of the exclusion of these 111 data points was appropriately documented. Please perform a sensitivity analysis for your main statistical model(s), to A) run the analysis one time excluding the data points, and B) run the analysis another time including the data points. The results of B) can be placed in the Supplementary materials and briefly referenced within the manuscript, to show that the exclusion (hopefully) doesn’t alter results.*

First, we thank the reviewer for acknowledging our concern about the data from the 111 owners who were not primary decision makers. As discussed in our previous response, there are genuine concerns about the reliability of information these owners provided, not least that there might plausibly be differences in their attitudes and opinions about health matters in general. However, given the reviewer’s continued concern, we have undertaken further work to address this point more thoroughly. Although the reviewer’s assertation that *“deleting data from a study dataset is a very hazardous statistical step*” is arguably overstated, we agree with them that this should be undertaken cautiously, be justifiable and any impacts be properly considered.

To address this, we have first created a flow diagram to illustrate where *decision-maker status* could interact with other variables in the dataset:

As discussed above, such a variable could affect the knowledge/recollection of owners about their dog’s health, as well as being associated with different beliefs and attitudes. However, we cannot think of any plausible biological mechanism through which decision maker status would directly impact the actual health of the dog.

Next, we have created a new predictor variable (*decision maker status*; primary vs. other) and have tested its associations with our outcome variables using logistic regression. Notably, the odds of primary decision makers recording their dog as having *any health problem* were **less** than for owners who were not primary decision makers (OR 0.576: 99%-CI 0.347, 0.958; P=0.005, pseudo-R2 0.0046). Such a difference validates our concerns that the responses of owners who are primary decision makers are materially different from those who are not. Further, given the lack of a plausible direct association with actual dog health, this is most likely to be mediated either through differences in knowledge/recollection, differences in beliefs/attitudes or both.

In theory, there are two options for addressing this issue. First, we could try to account for the effect of decision maker status by adding add the *primary decision maker* variable as a covariate to all models. However, this is problematic because the groups are so imbalanced (2,211 primary decision makers vs. 111 other owners), and this could adversely affect the performance of procedures such as logistic regression. The second way to address this issue would be to exclude the small number of owners who are not primary decision makers, which was our original choice. Based on our flow diagram (see above), this would have the effect of eliminating the pathways associated with the *decision-maker* variable and simplifying the overall structure, but without affecting any other associations amongst variables. The reason for this is that removing all data associated with owners who are not primary decision makers effectively ‘collapses’ the decision-maker variable into a single category and, as such, no variance is then associated with it; put another way, the variable is no longer ‘variable’ and can be safely ignored.

For these reasons, it is justifiable to focus our main modelling studies on the dataset from primary decision makers only, and these are the findings we present in our results section. However, as suggested by the reviewer, we have conducted additional logistic regression modelling on the all-owner dataset, and these are now reported in the supplementary information (S1 Table, S2 Table). Such results have also been compared with those for the primary decision makers (Tables 2 and 3). Since comparisons of this kind are best undertaken using estimates of regression coefficients (β) from the different models, we have now added these to all our tables.

Overall, the following changes have been made to the manuscript:

**Methods**

As a final pre-processing step, a *decision-maker* (primary vs. other) variable was created from data about the role owners played choosing a diet for their dog; for this, the ‘primary decision-maker’ category was classified as ‘primary’, whilst the other two categories (‘play no role’, n=15; ‘play some lesser role’, n=96) were classified as ‘other’. Given concerns about the reliability of information from owners who were not primary decision-makers and healthcare received, its effect on the outcome variables used in modelling studies (both binary illness variables) was first assessed by logistic regression, before the other modelling studies, as described below.

**Results**

We were concerned with the reliability of data from the 111 owners (4.8%) who were not the primary decision-makers, because their knowledge about the health of their dog could well be less reliable and their beliefs and behaviours might also be different, all of which would likely impact perceptions about the health of their dog. To examine this, we created separate binary logistic regression models for the *any health problem* and *significant illness* outcome variables, with *decision-maker status* as the sole predictor variable. There was no difference in the odds of an owner recording their dog as having a *serious illness*, whether they were a primary decision-maker or not (OR 0.662: 99%-CI 0.286, 1.896; *P*=0.252, pseudo-R^2^ 0.0015). However, the odds of primary decision-makers recording their dog as having *any health problem* were less than for owners who were not primary decision-makers (OR 0.576: 99%-CI 0.347, 0.958; *P*=0.005, pseudo-R^2^ 0.0046). As a result of this difference, we decided to limit further analyses to data from the 2,211 owners who were primary decision-makers. However, to determine the effect of this decision, simple logistic regression was also performed on the data from all owners (irrespective of decision-maker status; S1 Table; S2 Table), enabling comparisons to be made with simple logistic regression performed on the data from primary decision-makers only (see below).

*Any health problem binary variable: simple regression*

Within the *any health problem* binary variable, 894 (38.5%) and 1429 (61.5%) dogs were classified as ‘yes’ and ‘no’, respectively. Simple binary logistic regression was performed first to assess the unadjusted effects of each predictor variable separately using data from primary decision-makers only (Table 2). Broadly similar results were obtained when logistical regression was performed on the dataset from all owners (irrespective of decision-maker status; S1 Table). For example, estimates of the regression coefficients (β) for the *dog diet vegan* variable was ‑0.43 (SE 0.131) using data from all owners and β -0.30 (SE 0.159) using data from primary decision-makers only.

*Serious binary variable: simple regression*

Within the *significant illness* binary variable, 122 (5.5%) and 2089 (94.5%) dogs were classified as ‘yes’ and ‘no’, respectively. Results of the simple binary logistic regression stage, to assess the unadjusted effects of each predictor variable separately using data from primary decision-makers only, are shown in Table 3, whilst the results of equivalent analyses on the dataset from all owners (irrespective of decision-maker status) are shown in S2 Table. Once again, estimates of the regression coefficients were broadly similar from both data sets. For example, estimates of the regression coefficients (β) for the *dog diet vegan* variable was -0.46 (SE 0.309) using data from all owners and β -0.44 (SE 0.402) using data from primary decision-makers only.

**Discussion**

We were also concerned about the small numbers (n=111) of respondents who were not make the primary decision about their dog’s diet. Some of these owners reported playing “some lesser role”, whilst others reported playing “no role” in diet decision-making [15]. Not only would this raise concerns about the accuracy of the diet information, but these owners might also be less involved in other aspects of the dog’s life, including health and veterinary matters, which might adversely affect the accuracy of other information provided (Fig 9a). There might also be differences in their opinions and attitudes towards health matters in general. Analyses performed using *decision-maker status* as an outcome variable supported these concerns, not least since the odds of a dog being recorded as having a health problem were less for owners who were primary decision-makers than for those who were not. One way to account for this would be to include *decision-maker status* as a covariate in all analyses but the marked imbalance in group size (2,211 vs. 111) could adversely affect the performance of procedures such as logistic regression. Therefore, we instead decided to limit further analyses to owners who were primary decision-makers only. Decisions about excluding observations from a dataset should be made with caution, properly justified and consider the possible impact on results. Based upon our flow diagram of associations within in the dataset (Fig 9b), we determined that it would be possible to eliminate all pathways associated with the *decision-maker status* variable, thereby simplifying the overall structure without affecting any other associations amongst variables. This was because removing all data from owners who are not primary decision-makers effectively ‘collapses’ the *decision-maker status* variable into a single category such that variance is no longer associated with it. Estimates of regression coefficients and odds ratios were broadly similar in logistic regression models using the all-owner dataset (S1 Table, S2 Table), compared with models using the primary decision-maker dataset (Tables 2 and 3). Any differences are likely to be the result of differences in the characteristics, attitudes and beliefs of the owners that were not primary decision-makers. Of course, one main disadvantage of focusing on data from owners who are primary decision-makers is that it limits the generalisability of findings to the wider pet-owning public, not least to other members of a dog-owning family. However, given the original concern about data reliability, further studies involving all family members would be required to explore such opinions properly.

**Reviewer comment 2.10**

*Further issues requiring revision*

*1.	Regarding comment #1:*

*I appreciate the paragraph added in line 798. I think it is not needed in this length and can be shortened quite a bit, also to keep the focus in the discussion. E.g., in rough summary, “We did not contact the authors for this study because A) the original study was well written, replicable etc. and B) we wanted to retain independent in the study process given the importance of independent replication to the scientific method” (or similar).*

Many thanks for this suggestion. We have shortened the paragraph as follows:

A third limitation was the fact that we did not contact the authors of the original study before conducting our data analyses, to clarify any uncertainties with the dataset and seek guidance on our planned approach. We chose not to do so because the original paper was well written, with clearly presented study methodology and results. Further, since independent replication is critical to the scientific method, it was arguably better not to have direct contact with the primary study authors when conducting statistical analyses.

**Reviewer comment 2.11**

*2.	Further data exclusion:*

*Referring to line 283 (“Influential datapoints were […] not removed unless there was a good reason to do so.“), it remains unclear if you did remove further points in addition to the above discussed 111 data points. Please specify more clearly which data points were excluded and provide the reasoning.*

We apologise that there was a lack of clarity in this section. In short, we used Cook’s distance to identify influential datapoints. In most models, none were identified. In the occasional cases where outliers were identified (see statistical reports), we checked them in the original data to make sure there were no obvious errors (e.g., unrealistic results such as dog age >20 years). No such errors were found. Given that we had used data from a secondary source and had no further means of verifying them (e.g., by comparing against the original questionnaire information), we decided not to remove them as there was no valid justification to do so (e.g., a typographical error at the time of data entry).

We hope that this makes sense and have amended the methods section to clarify this:

Influential datapoints were identified and assessed using Cook’s distance and, in most models, none were identified. In the occasional cases where outliers were identified, we checked them in the original data to make sure there were no obvious errors (e.g., unrealistic results such as dog age >20 years); no such errors were found. Given that we were using data from a secondary source, had no further means of verifying them (e.g., by cross-checking against the original questionnaire response), we decided not to remove them as there was no valid justification to do so (e.g., a typographical error created when the original data were entered).

**Reviewer comment 2.12**

*3.	Dimension reduction visualization (UMAP & clustering):*

1. *I do appreciate the effort in using modern statistical tools to facilitate the interpretation of potential complex multidimensional correlation structures in this dataset. However, I think the UMAP figures are minimally interpretable by many likely audiences (e.g., most veterinary readers), and (correct me if I’m wrong) as far as I see it the only clear finding from applying this method is that dog owners who are vegan themselves also tend to feed their dogs vegan/vegetarian more often. This latter statement could equally be made using an easily understandable table of relative frequencies in the data, which I would favour in this case, and – if deemed meaningful – a comment that the application of the clustering approach didn’t lead to further insights. Otherwise, for the sake of generalist reader comprehension, it would be necessary to explain the interpretation of the method and of the results in more detail (either in the main part of the paper, or in the Supplementary materials while quoting the most important findings in the main part).*

Many thanks for this comment, and we accept that many generalist readers may be unfamiliar with this methodology. However, because it is such a powerful visualisation technique, we believe that the manuscript is stronger with these results, rather than a table of relative frequencies of data. Its key strengths are the ability to use different data types (continuous, ordinal categorical, unordered categorical etc), the ability to reduce multiple dimensions of a multivariate dataset down to just two and the non-linear method for calculating distances amongst datapoints. Given these strengths, the method out-performs many other visualisation techniques, including principal component analysis (McInnes et al 2018; <https://doi.org/10.48550/arXiv.1802.03426>). Therefore, rather than removing UMAP, we have instead taken the reviewer’s second suggestion and provide more details about the method and have better explained the results.

Details of changes to the manuscript are as follows:

**Methods**

Unsupervised uniform manifold approximation and projection (UMAP) [52] was used to reduce the multivariate owner and dog metadata data to two dimensions for visualisation purposes (using the ‘uwot’ package [51]). After this, density-based spatial clustering (DBSCAN, using the ‘dbscan’ package [26]) was applied to the UMAP outputs to identify potential groupings within these owner-animal metadata, prior to the data pre-processing necessary for the statistical and machine learning modelling, as explained above. The UMAP approach was specifically developed to be applicable to mixed datasets with categorical, ordinal and numeric data [52]. It is a potent, non-linear dimension-reduction technique that outperforms principal component analysis (PCA) and t-distributed stochastic neighbour embedding (TSNE) in its ability to preserve the global and local structure of data [52]. It employs a specific type of manifold projection known as Riemannian geometry, which has the strength of enabling meaningful clusters to be identified and visualised in a reduced dimensional space. This approach differs from PCA in that it typically reduces multivariate data to two parameters (arbitrarily called x and y); further, rather than calculating variability, UMAP instead non-linearly transforms the data to create a lower-dimensional representation, allowing more complex data patterns to be captured, compared with linear methods like PCA. The axes in UMAP do not represent ‘principal components’ and cannot be interpreted in the same way; instead, they help visualise high-dimensional data in a lower-dimensional space.

Each record (individual row of the dataset with one owner-dog combination) contributes one point to the UMAP graph. The following animal variables were included: *dog age*, *dog sex*, *neuter status*, *diet* and *breed size category*. In addition, the following owner variables were included: *owner age*, *owner gender*, *owner diet*, *location*, *setting*, *education*, *income* and *decision-maker status*. Following best practise [53], different distance metrics were used including Euclidian (for continuous data e.g., *dog age*), Manhattan (for ordered categorical data e.g., *owner age*, *education*) and Hamming (for non-ordered categorical data e.g. *dog diet*, *dog sex* etc)

The quality of DBSCAN clustering was assessed with a silhouette plot along with silhouette scores [53] and also by calculating Davies-Bouldin’s cluster separation measure [54]. The silhouette plot provides a visual representation of how well points within the UMAP have been classified into their respective clusters, whilst average silhouette widths (silhouette scores) quantify how similar the point is to its own cluster relative to other clusters; silhouette scores can range from +1 to -1, with positive and negative values indicating good and poor matching, respectively; further, average cluster silhouette scores of 0.26-0.50, 0.51-0.70 and 0.71-1.00 represent weak, reasonable and strong clustering, respectively [55]. Davies-Bouldin’s cluster separation measure is determined from the ratio between the scatter within and the separation between clusters, with smaller values suggesting better clustering and values <1 representing very good clustering.

**Results**

The original dataset comprised information on 2,639 owner-dog pairs [15]. After the initial pre-processing stage to create separate dog sex and neuter status variables, and also removing dogs without age data (n=3) or that were <1 year of age (n=27), the number of owner-dog pairs with available data was 2,609. We first used a UMAP projection and density-based clustering to create a simple overall visualisation of relationships in these owner-animal metadata (Fig 1; S1 and S2 Figs). Six distinct clusters were identified, with a Davies-Bouldin’s cluster separation measure of 0.652 (good). Good clustering was evident within 3 clusters (average silhouette scores: cluster 2, 0.86; cluster 5, 0.84; cluster 6, 0.80), there was reasonable clustering in two others (average silhouette scores; cluster 3, 0.68; cluster 4, 0.59), but clustering was poor in the final cluster (cluster 1, average silhouette score 0.36).

Examining the owner-animal metadata, cluster 2 was most distinctive, almost-exclusively comprising dogs fed either a vegan (328/374 [87.7%] points in the cluster, 97.6% of all vegan dogs) or vegetarian (31/374 [8.3%] points in the cluster, 88.6% of all vegetarian dogs) diet, and also containing a predominance of owners consuming a vegan diet (332/374 [88.8%] points in the cluster, 57.1% of all vegan owners). In contrast, cluster 3 comprised owners who consumed either a vegan (249/608, 41.0%), vegetarian (240/608, 39.5%) or pescatarian (119/608, 19.6%) diet, but fed their dog either a conventional meat-based (423/608, 69.6%) or raw (175/608, 28.8%) diet. Cluster 6 was also distinctive in that it mostly comprised owners who consumed an omnivore (214/316, 67.7%) or omnivore reducing (98/316, 31.0%) diet and fed their dogs a raw diet (299/316, 94.6%). In the remaining 3 clusters (clusters 1, 4 and 5), most owners consumed either an omnivore or omnivore-reducing diet and usually fed their dog either a conventional meat-based diet or raw diet. Besides owner and dog diet, there were no major differences amongst clusters for the other variables, although some variables did appear to separate some clusters (e.g., cluster 4 for the setting variable; cluster 5 for the location variable; S1 and S2 Figs).

Please note that the results presented are different from before because of differences in how the variables are included (categorical vs. continuous), which was in response to comment 3.2 from reviewer 3. We hope that this reviewer will agree that the clustering of owner-animal pairs on the basis of the diet consumed is truly fascinating!

**Reviewer comment 2.13**

2. *How big is the share of variability represented by the first two UMAP dimensions? Please calculate it and add a statement about it in the Statistics section. The confidence one can put in the interpretation of dimension reduction methods highly depends on the share of variability the shown dimensions explain.*

Many thanks for the comment, which we suspect has arisen for the reviewer inadvertently mistaking the UMAP output with outputs from a method such as principal component analysis (PCA). This was partly our fault because we did not provide enough detail about UMAP methodology in our methods section and did not properly explain the results; we apologise to the reviewer for this. First, we ran our UMAP in *unsupervised* mode, which is intended for visualisation purposes, rather than training for prediction. Second, UMAP reduces multivariate data (from all variables included in the model) to two parameters (arbitrarily called x and y). Therefore, unlike PCA, all variability is included within these two dimensions. A further difference from PCA is that UMAP performs a non-linear transformation of the data to create a lower-dimensional representation. This process allows UMAP to capture more complex patterns in the data compared with linear methods like PCA.

As a result of the non-linear nature of the transformation used in UMAP, interpretation of the UMAP dimensions (x and y in this case) is more nuance in that they do not have a direct (ie linear) interpretation. Instead, the relative distances between points in this space can be used to infer similarity between data points, thereby visualising high-dimensional data in a lower-dimensional space.

We have addressed this confusion by expanding the methods section, to ensure that the UMAP approach is properly explained, including how it differs from PCA. The revised methods reads as follows:

**Methods**

Unsupervised uniform manifold approximation and projection (UMAP) [52] was used to reduce the multivariate owner and dog metadata data to two dimensions for visualisation purposes (using the ‘uwot’ package [51]). After this, density-based spatial clustering (DBSCAN, using the ‘dbscan’ package [26]) was applied to the UMAP outputs to identify potential groupings within these owner-animal metadata, prior to the data pre-processing necessary for the statistical and machine learning modelling, as explained above. The UMAP approach was specifically developed to be applicable to mixed datasets with categorical, ordinal and numeric data [52]. It is a potent, non-linear dimension-reduction technique that outperforms principal component analysis (PCA) and t-distributed stochastic neighbour embedding (TSNE) in its ability to preserve the global and local structure of data [52]. It employs a specific type of manifold projection known as Riemannian geometry, which has the strength of enabling meaningful clusters to be identified and visualised in a reduced dimensional space. This approach differs from PCA in that it typically reduces multivariate data to two parameters (arbitrarily called x and y); further, rather than calculating variability, UMAP instead non-linearly transforms the data to create a lower-dimensional representation, allowing more complex data patterns to be captured, compared with linear methods like PCA. The axes in UMAP do not represent ‘principal components’ and cannot be interpreted in the same way; instead, they help visualise high-dimensional data in a lower-dimensional space.

Each record (individual row of the dataset with one owner-dog combination) contributes one point to the UMAP graph. The following animal variables were included: *dog age*, *dog sex*, *neuter status*, *diet* and *breed size category*. In addition, the following owner variables were included: *owner age*, *owner gender*, *owner diet*, *location*, *setting*, *education*, *income* and *decision-maker status*. Following best practise [53], different distance metrics were used including Euclidian (for continuous data e.g., *dog age*), Manhattan (for ordered categorical data e.g., *owner age*, *education*) and Hamming (for non-ordered categorical data e.g. *dog diet*, *dog sex* etc)

**Reviewer comment 2.14**

3. *Please add a statement about the goodness of the clustering, regarding how well it separates the clusters from one another. One standard measure would be to add a silhouette plot and the average silhouette coefficient.*

Silhouette plots are indeed standard methods for kmeans clustering, which is a traditional clustering method. Not only do such plots provide an indication of goodness of clustering, they can also be used as a method for choosing an appropriate number of clusters. Since we instead used density-based clustering (DBSCAN) to determine the optimal cluster number, we did not original create a silhouette plot. However, the reviewer is correct that, whatever the clustering method, a silhouette plot is useful to show the quality of clustering, and have now created one. As well as the silhouette plot, we report the associated silhouette scores and have also included the familiar Davies-Bouldin index, as a measure of quality of the clustering. The relevant parts of the methods and results are as follows:

**Methods**

The quality of DBSCAN clustering was assessed with a silhouette plot along with silhouette scores [53] and also by calculating Davies-Bouldin’s cluster separation measure [54]. The silhouette plot provides a visual representation of how well points within the UMAP have been classified into their respective clusters, whilst average silhouette widths (silhouette scores) quantify how similar the point is to its own cluster relative to other clusters; silhouette scores can range from +1 to -1, with positive and negative values indicating good and poor matching, respectively; further, average cluster silhouette scores of 0.26-0.50, 0.51-0.70 and 0.71-1.00 represent weak, reasonable and strong clustering, respectively [55]. Davies-Bouldin’s cluster separation measure is determined from the ratio between the scatter within and the separation between clusters, with smaller values suggesting better clustering and values <1 representing very good clustering.

**Reviewer comment 2.15**

*4.	Correlation matrix*

*I concur with the use of correlation matrices because they give a good overview of the data. Do I understand correctly that some bubbles in the correlation matrix are not shown because of some p-value based (in your case -LogP) or FDR-based criterion? This remains slightly unclear to me in the text.*

The reviewer is correct that we applied a threshold for including bubbles in the correlation matrix, which was based on FDR-corrected *P*-values. Bubbles were not displayed for those correlations that did not reach the threshold for statistical significance. We apologise that this was not clear in the original text, most notably, the Figure legend for the correlation matrix. In response to the next reviewer comment (see below for details), we have now removed the threshold for including bubbles in the matrix and the figure has been revised accordingly. We have also better explained what the correlation matrix shows in the legend for Fig 2:

**Fig 2. Naïve correlation amongst owner-animal metadata variables.** Data were first coerced into a numerical ordinal form, except for the Location variable which was not ordinal and, therefore, one-hot encoding was instead used. Correlations between variables were calculated using Kendall’s tau, with positive and negative correlations coloured in blue and red, respectively. Both the size and intensity of the colour are proportional to the size of Kendall’s tau. Not surprisingly, a large, dark blue circle is evident when variables are correlated with themselves. Hierarchical clustering, using the Ward method [58], is shown on the left side of the figure. All pairs are shown whether statistically significant or not; however, correlation coefficients and associated log *P*-values, corrected for false-discovery rate using the Benjamini-Hochberg procedure, are shown in full in the supplementary tables (S3 and S4 Tables).

**Reviewer comment 2.16**

*In any case, I would suggest dropping both the LogP and FDR criteria to simplify the analysis, and showing all bubbles in the matrix. In all other analyses, you do a really nice job of not interpreting p-values (rightly so, after variable selection). In the correlation matrix, I would find it most informative and easiest understandable if you would 1) show all bubbles in the correlation matrix, and 2) state that this is an exploratory analysis. Individual correlation values shouldn’t be overinterpreted regardless, so you don’t gain anything, but mostly add complexity to your analysis when including LogP and FDR.*

We disagree with the reviewer about dropping the FDR correction since this is required to correct for multiple comparisons and would be considered to be best practice amongst statisticians. Therefore, we have decided to retain FDR-corrected *P*-values in S4 Table. That said, and as discussed in our response to comment 2.15, we agree that showing all bubbles in the correlation matrix is helpful to the reader and, as a result, have removed the threshold we originally applied for displaying them (which was based on the FDR-adjusted *P*-values). We hope that the reviewer accepts this compromise. We also agree that readers might find Log*P* harder to comprehend than standard *P*-values. Therefore, we have now also included the latter in our results:

Results of Kendall’s tau correlations are shown in Fig 2, whilst datasets of all correlation coefficients and FDR-corrected -Log*P* are presented in the supplementary information (S3 and S4 Tables, respectively). Several associations were identified including a large positive association between owners and dogs consuming *vegan diets* (Kendall’s tau 0.66, -Log*P* 212.0 [*P*<0.001]), a small positive association between UK location and dogs being fed a *raw diet* (Kendall’s tau 0.24, -Log*P* 29.5 0 [*P*<0.001]), as well as positive associations amongst healthcare variables (*received medication* and *veterinary visits*, Kendall’s tau 0.51, *-*Log*P* 154.8 0 [*P*<0.001]; *received medication* and *switched to therapeutic diet*, Kendall’s tau 0.17, *-*Log*P* 14.9 0 [*P*<0.001]; *switched to a therapeutic diet* and *veterinary visits*, Kendall’s tau 0.16, *-*Log*P* 15.3 0 [*P*<0.001]). In contrast, weak associations were observed between the *dog vegan diet* variable and the healthcare variables (*dog vegan diet* and *veterinary visits*, Kendall’s tau -0.02, *-*Log*P* 0.5 [*P*=0.340]; *dog vegan diet* and *received medication*, Kendall’s tau -0.11, *-*Log*P* 2.7 0 [*P*=0.002]; *dog vegan diet* and *switched to a therapeutic diet*, Kendall’s tau 0.00, *-*Log*P* 0.1 0 [*P*=0.843]).

**Reviewer comment 2.17**

*5.	Model evaluation*

1. *Please report AUC values (based on cross-validation or a hold-out test set) for all logistic regression models, instead of pseudo R², since AUC is the most established goodness of fit measure for logistic regression. (e.g., lines 41 & 54).*
2.1 *In lines 271 ff. and 288 ff. you state “Goodness of fit was tested by […] 3 tests for goodness of fit“. In statistical research, it is the standard now to not perform such goodness of fit tests anymore to judge the overall goodness of a model, but instead to visualize and judge the distribution of the model errors (or in the logistic case looking at the ROC curve and the test set AUC). This is especially due to the fact that such tests are well-designed to detect strong deviations from the model assumptions, but have only low power in situations where model assumptions are more or less fulfilled. Accordingly, you generally gain more insights in the model structure by simply visually evaluating it with model evaluation plots (which can also be shown purely in the Supplementary materials, and referenced in the main manuscript). I would therefore suggest dropping all three tests and keep the analysis simple with ROC curves and AUC values.*

**Please note that these two points are similar and, therefore, have been answered together.**

Whilst we respect the reviewer’s opinion, it is certainly not the consensus amongst statisticians. In fact, pseudo-R^2^, AUC and BIC all assess different aspects of model performance and so are not interchangeable:

- R^2^ is a proxy measure of the amount of variance that the model explains.
- AUC from ROC curves is an indication prediction accuracy, namely how well the model correctly predicts the status of individuals.
- Other goodness of fit tests analyse this statistically, whilst measures such as BIC indicate how generalisable the model is.

Further pseudo-R^2^ is, arguably, the measure that is most familiar to those without much statistical training and is still commonly used, not least in veterinary science publications that use logistic regression. Therefore, it would be wrong to report AUC from ROC curves as the only performance measure.

That said, we agree that it would be helpful if this metric were included as **an additional** performance check, and hope that the reviewer is happy with such a compromise. Therefore, we have re-analysed all our logistic regression models, with additional code to accommodate ROC curves and AUC from a hold-out test dataset. This required an additional step at the data pre-processing stage, namely to divide the original dataset randomly into training (75%) and test (25%) sets; the former is used to generate all the models, whilst the latter is used for the creation of ROC curves and associated AUC. Note that this has only been done for the data from primary decision makers and not for the dataset of all owners, a point explained further below.

The relevant passages covering this in the methods are as follows:

**Binary logistic regression** (paragraph 1)

Binary logistic regression models were created using the ‘glm’ function in R. Both binary illness variables (any health problem and significant illness) were used separately as outcome variables, whilst all owner, animal and healthcare variables were tested as predictor variables. We used a combination of simple and multiple logistic regression. Simple logistic regression analyses were initially performed using data from all owners (irrespective of decision-maker status), with results shown in the supplementary information (S1 and S2 Tables). However, these models revealed concerns over the reliability of data from owners who were not primary decision-makers (see below) and, as a result, all remaining logistic regression analyses were instead conducted with the primary decision-maker dataset. For these analyses, the dataset was first randomly divided into training (75%) and test (25%) datasets, using the ‘sample’ function in R, a step that was necessary to facilitate model validation by receiver operating characteristic (ROC) curves (see below).

**Binary logistic regression** (paragraph 5)

Results are reported as estimates of the regression coefficients (β) with the associated standard error (SE), and with odds ratios (OR) with the associated 99% confidence intervals (99%-CI). Influential datapoints were identified and assessed using Cook’s distance and, in most models, none were identified. In the occasional cases where outliers were identified, we checked them in the original data to make sure there were no obvious errors (e.g., unrealistic results such as dog age >20 years); no such errors were found. Given that we were using data from a secondary source, had no further means of verifying them (e.g., by cross-checking against the original questionnaire response), we decided not to remove them as there was no valid justification to do so (e.g., a typographical error created when the original data were entered). Possible multicollinearity in all models was assessed using the generalised variance inflation factor (GVIF) and GVIF(1/(2×𝐷𝑓); these were deemed to be acceptable when all values were <4 and <2 for GVIF and GVIF(1/(2×𝐷𝑓), respectively [62]. If necessary, multicollinearity was resolved by removing the variable with the greatest GVIF. Goodness of fit was tested by a visual inspection of observed and expected results and the Hosmer and Lemeshow test for large datasets. In addition, the proportion of variance explained in the overall model was assessed by the coefficient of determination (pseudo-R^2^) based on the method reported by Nagelkerke [63]. Further, the log-odds of the outcome variable and dog age was plotted to enable visual assessment of whether the relationship was linear, and this was further assessed with the Box-Tidwell test. Finally, prediction accuracy was assessed by creating ROC curves, using the ROCR package [44], and then calculating their area under the curve (AUC). Values could range from 0 to 1; a model that performed no better than chance would have an AUC of 0.5, and models predicting better than by chance would have AUC >0.5, with an AUC of 1.0 suggesting perfect prediction. The test dataset was used to generate the ROC curves and AUC for models using data from owners who were primary decision-makers; however, because the all-owner dataset containing was not subdivided, AUC are based in ROC curves from the original dataset.

Further, all relevant tables and passages in the results section have been redrafted to include the AUC results in addition to the other measures of performance (BIC and pseudo-R^2^). As can be seen, all measures correlate well with one another; therefore, the models with the best generalisability (BIC) tend to explain more of the variance (pseudo-R^2^) and have a better predictive performance (AUC). We hope that this reassures the reviewer that model performance has been thoroughly and appropriately assessed.

One final point to note, here. As can be seen from these revised methods sections, and for the reasons we discussed earlier, we have now included logistic regression results from analyses conducted using data from all owners (not just primary decision makers) within the supplementary information. For these analyses, we chose not to divide the data into training and test sets but, instead to perform these on using the original data. The reason for this was that we wanted to ensure the maximum power to identify all significant associations. For this reason, we have generated ROC curves and estimated AUC using the data that were used to train the model (i.e., the complete dataset) rather than using a separate dataset. To illustrate the likely magnitude of any such over-estimate, we decided to perform ROC curves and AUC from BOTH the test and training datasets for the **primary decision maker** dataset. All such curves are included in the statistical reports within the supplementary information. However, two examples are shown below to illustrate:

**Dog diet vegan and the any health problem outcome**

**Veterinary visits variable and the any health problem outcome**

As can be seen, the AUC are broadly similar from the training and test sets. Therefore, whilst we only have the AUC from the training data for the data from all owners, it is likely that these are reasonable, at the very least, to enable a crude comparison with the data from the primary decision makers. We hope that this is acceptable to the reviewer.

**Reviewer comment 2.18**

*6.	Results interpretation*

1. *Around line 829 you state that the “possibility [of owners feeding unconventional dog food being sceptical about visiting the veterinarian or allowing their dog to receive medication] is supported by the fact that there were negative associations between the use of medications and feeding a raw or vegan diet […]“.*

*It is possible that guardians feeding vegan diets were sceptical about veterinary healthcare but this is very unlikely (other than with respect to anti-vegan dietary advice) – these guardians were more likely than the other two groups to comply with common veterinary advice to have their animals neutered (the opposite was true for guardians feeding raw meat, who were less likely to have their animals neutered, and also much less likely to visit vets, and who are also known to be more likely to gather information from non-veterinary sources). Hence if including this comment (‘possibility X is supported by Y’), then for balance you need to add that at least in the case of vegan diets, lowered rates of medication use may simply reflect better health (i.e. this is another possibility supported by these same results).*

Many thanks for this comment, which we have carefully considered. We accept that the original passage was misleading because we did not properly separate comments about dogs fed raw and vegan diets. The reviewer is also correct that, in their original study, differences in neutering were evident (raw < conventional < vegan) and we did not properly consider this. As a result, we have reworded this paragraph to highlight a greater complexity in associations between diet fed and healthcare variables, better emphasising differences between raw and vegan diets. The revised paragraph now reads as follows:

A final limitation was the fact that there might be confounding between healthcare variables (e.g., *veterinary visits* and *received medication*) and dietary choices. For example, owners who are sceptical about conventional diets and instead choose to feed either vegan or raw food, might also be less willing either visiting their veterinarian or allowing their dog to receive medication. In support of this, negative associations were identified in the current study between feeding a raw diet and both veterinary visit frequency and the use of medications; further, in the previous study, dogs fed raw diets were less likely to be neutered than dogs fed conventional diets [15]. However, results were inconsistent for dogs fed vegan diets; whilst there was a negative association between feeding a vegan diet and the odds of a dog receiving medications, there was no association with frequency of veterinary visits. Further, in the previous study, dogs fed vegan diets were more likely to be neutered than those fed conventional diets [15]. Given these complexities, further research would be required to investigate associations between different diet choices and healthcare variables.

**Reviewer comment 2.19**

2. *In line 844 you state the “vegan diet variable was only the 7th most important predictor variable“. Please clarify this statement referring to the absolute effect size / effect strength and not the relative rank in effect sizes. If 10 variables all had really strong effects the 7th would also have a strong one, so referring only to “7th most important“ doesn’t provide the necessary information.*

We accept this concern and agree with the reviewer that, with hindsight, we did not provide enough details about the effect of individual predictor variables within the XG Boost models. XG Boost provides different metrics to determine the contribution of the different variables (aka ‘features’) in the model including:

- ‘Importance’ (aka gain) represents fractional contribution of each feature to the model, based on the total gain from including each feature;
- ‘Cover’ represents the number of observations related to this feature in the model);
- ‘Frequency’ represents the relative number of times a feature has been used in trees).

We did include these in the original figures, although we inadvertently included both importance and gain (which are the same thing), and have now corrected this. However, with hindsight, we should have also reported these results for variables included in each of the models as this helps to show their actual contribution (in addition to their order).

We have revised the results section to explain these metrics:

Different measures of the contribution of the variables (‘features’) included in the final models were also calculated and depicted graphically. These included ‘feature importance’ (a metric of the fractional contribution of each feature to the model, based on the total gain from including each feature), ‘cover’ (a metric of the number of observations related to this feature in the model) and ‘frequency’ (a metric that represents the relative number of times a feature has been used in trees). Since these metrics are normalised, the sum of scores for all features in the model is 1.0.

The results have also been updated as follows:

**XG Boost for the *any health problem* outcome**

The ability of owner, animal and healthcare variables to predict the *any health problem* variable was further assessed using machine learning predictive modelling using the XGBoost algorithm. Two separate models were created using data from primary decision-makers; the first contained all variables, whilst the second was a reduced model containing just the owner-animal metadata (i.e., without healthcare variables). Based on ROC analysis, the prediction accuracy of the all-variable model was reasonable (AUC 0.836; Fig 4a). The XGBoost procedure also enables the most important variables contributing to the prediction to be identified, for the all-variable model (Fig 4b); the variables that most strongly predicted the *any health problem* outcome variable were (in order, based on the sum of the 3 metrics): *received medication* (importance 0.393, cover 0.129, frequency 0.065), *dog age* (importance 0.175, cover 0.182, frequency 0.188) and *veterinary visits* (importance 0.153, cover 0.181, frequency 0.155), followed by *owner age* (importance 0.048, cover 0.083, frequency 0.106) and then *breed size category* (importance 0.046, cover 0.070, frequency 0.087). Other variables followed these including *education*, *dog sex*, *location*, and *dog diet*; for the latter variable, *dog diet raw* was 10^th^ in order of importance (importance 0.015, cover 0.026, frequency 0.032), whilst *dog diet vegan* was 15^th^ in order of importance (importance 0.008, cover 0.021, frequency 0.013).

The order of importance of predictor variables was broadly similar in the reduced (owner-animal metadata) model although, based on ROC analysis, prediction accuracy was poor (AUC 0.674, Fig 5a). In this reduced model, *dog age* was overwhelmingly most important (importance 0.261, cover 0.196, frequency 0.157), followed by *owner age* (importance 0.115, cover 0.123, frequency 0.141, *education level* (importance 0.095, cover 0.089, frequency 0.124), *breed size category* (importance 0.091, cover 0.099, frequency 0.108) and *neuter status* (importance 0.060, cover 0.062, frequency 0.047). Other variables including *dog diet*, *urban setting*, *location* and *owner income* and followed these (Fig 5b); for the dog diet variable, *dog diet raw* was 7^th^ in order of importance (importance 0.054, cover 0.048, frequency 0.060), whilst *dog diet vegan* was 12^th^ in order of importance (importance 0.039, cover 0.045, frequency 0.031).

**XG Boost for the *significant illness* outcome**

Using machine learning predictive modelling with XGBoost, and data from primary decision-makers, all-variable and reduced (owner-animal metadata) models were created (S2 File) for the *serious illness* outcome variable. Based on ROC analysis, the prediction accuracy of the all-variable model was reasonable (AUC 0.887; Fig 7a). In order (Fig 7b), the variables that most strongly predicted the *significant illness* outcome variable were: *veterinary visits* (importance 0.530, cover 0.473, frequency 0.285), *dog age* (importance 0.182, cover 0.219, frequency 0.302) and *received medication* (importance 0.175, cover 0.190, frequency 0.143), followed by *breed size category* (importance 0.050, cover 0.034, frequency 0.079), *education* (importance 0.016, cover 0.020, frequency 0.050), *owner age* (importance 0.012, cover 0.014, frequency 0.036) and *dog sex* (importance 0.009, cover 0.010, frequency 0.034)*.* Other variables followed these including *location, urban setting*, *owner sex*, *animal career* and *therapeutic food*. For the *dog diet* variable, *dog diet raw* was 17^th^ in order of importance (importance 6.14 x10^-5^, cover 4.09 x10^-5^, frequency 1.55 x10^-4^) relative importance), whilst the *dog diet vegan* variable was of insufficient importance to be included in the model (i.e., relative importance less than that of the *dog diet raw*).

As with the *any health problem* binary, ROC analysis suggested that prediction accuracy of the reduced (owner-animal metadata) model was poor (AUC 0.689, Fig 8a). Once again, *dog age* (importance 0.304, cover 0.362, frequency 0.224) was the most important predictor, followed by *breed size category* (importance 0.195, cover 0.226, frequency 0.186), *owner age* (importance 0.091, cover 0.082, frequency 0.125), *urban setting* (importance 0.077, cover 0.066, frequency 0.086) and *education* (importance 0.072, cover 0.060, frequency 0.094); other variables followed these including *dog sex*, *dog diet*, *location*, *animal career* and *neuter status* (Fig 8b); for the dog diet variable, *dog diet raw* was 7^th^ in order of importance (importance 0.059, cover 0.042, frequency 0.047), whilst *dog diet vegan* was 12^th^ in order of importance (importance 0.018, cover 0.014, frequency 0.016).

**Reviewer comment 2.20**

*7.	Reproducibility of your results*

*I would highly appreciate if the R code for all analyses would be published along with the manuscript.*

We can confirm our plan to publish all R code via GitHub, along with including all our statistical reports in the supplementary information.

**Reviewer comment 2.21**

*Minor feedback*

1. *Medication terminology: terminology ‘on medication’ or similar has been changed in many locations (incl. table and supplementary tables) to ‘prescribed medication by vet’. This reflects an incorrect assumption that all medication received must have been prescribed by a vet. This is not the case. Regardless of whether or not they should do so, pet guardians can and do receive and dispense medication, from sources other than veterinary prescriptions, e.g., online vendors. The actual question asked in the survey was “Has your animal received medications in the last year, apart from routine vaccinations and treatments for skin or internal parasites, such as fleas, ticks, lice, heartworm and intestinal worms? Please do not include neutering operations (spay/castration) or microchipping.” Hence all of this text should be changed back.*

Many thanks for pointing this out and apologies for the error. We have now changed this to “received medication” throughout the manuscript.

**Reviewer comment 2.22**

2. *Line 43: please correct the wording of the sentence (“… albeit having limited although their importance was limited.“)*

Many thanks for spotting this error. We have corrected the sentence as follows:

For the *significant illness* outcome, received medication, veterinary visits, dog age and were again the most important predictors for both logistic regression (AUC 0.903) and XGBoost (AUC 0.887), whilst breed size category, education and owner age also featured in the latter. Any contribution from the dog vegan diet variable was negligible.

**Reviewer comment 2.23**

*Line 93: change “and well as“ to “as well as“*

Many thanks for spotting this typographical error. We have now corrected it.

**Reviewer comment 2.24**

3. *Line 224: Ordinal logit models were either estimated with function MASS::polr or VGAM::vglm. I assume one of these functions was used for estimating the partial proportional odds models when the proportional odds assumption wasn’t fulfilled? Please clarify this in the text.*

The reviewer is correct about our use of R packages for the ordinal logistic regression analyses. However, as we have now decided to remove these analyses from the latest revision of the manuscript, we no longer reference them in the statistical software section of the methods.

**Reviewer comment 2.25**

4. *Line 324: “dog“ is a typo and should be ”dogs“*

Many thanks for this suggestion. We have reworded the sentence as follows:

Regarding the dogs in this dataset (Table 1), a range of ages and breed sizes were included with the majority of male (294/1169, 74.9%) and female (192/1042, 81.6%) dogs being neutered.

We hope that this makes the sentence clearer.

**Reviewer comment 2.26**

5. *Line 374: change “been“ to “between“*

Many thanks for spotting this typographical error. However, in altering our approach to variable selection for multiple regression, this sentence has been deleted.

**Reviewer comment 2.27**

*7.	Line 628: change “regressions analyses“ to “regression analyses“*

Many thanks for spotting this typographical error. As part of our revisions to improve clarity, this sentence has instead been reworded as follows:

We next used a combination of multiple logistic regression and machine learning predictive modelling so that multiple variables could be analysed concurrently.

**Reviewer 3**

**Reviewer comment 3.1**

The authors present a secondary analysis of a dataset from a previously published study on dog diet and health outcomes. The authors' goal was to incorporate more dog and owner information into models to account for possible confounding. The authors provide extensive detail in supplemental material (including statistical code), in addition to the manuscript itself, enabling the readers to follow along and understand exactly what was done. Although the authors approached the question using multiple approaches, generally arriving at similar conclusions, I do have some concerns about the approach.

Many thanks for your careful review of the manuscript and for your constructive suggestions. Below we have responded to each of the points you made and have outlined the changes to the manuscript in response. We hope that these responses and alterations address your concerns.

**Reviewer comment 3.2**

The manuscript will be strengthened if the authors consider the following points:

1. The authors use UMAP and DBSCAN as exploratory tools to assess the underlying structure of the data. They state they coerced all variables to be numeric in order to use these techniques. It is not clear to me that this is a sufficient way to handle the non-ordinal categorical variables (such as diet, gender and location). All examples I looked up for UMAP and DBSCAN focused only on variables that were inherently numeric and since these methods appear to have some sort of "distance" that is calculated, the numeric values matter. Do authors arrive at the same clusters if other orderings (numeric assignments) of the non-ordinal data are used?

Many thanks for this comment and apologies that the approach taken was not clear. Whilst both UMAP and DBSCAN do make use of information that is numeric, they are able to handle a range of different variable types (continuous, ordinal categorical, unordered categorical), and this was as explained in the original UMAP paper (McInnes et al 2018, <https://doi.org/10.48550/arXiv.1802.03426>). This is done by using different ‘distance metrics’ in the modelling process (e.g., Euclidian, Manhattan, Hamming etc). Specifically, the ‘Hamming distance’ is appropriate for use with unordered categorical data.

That said, in reviewing our methods, we realised that we had not actually used the appropriate distance metrics for our categorical variables (e.g., Hamming distance), and sincerely apologise for this error. To address this, we have now repeated our UMAP analyses using a different R package (‘uwot’). Our revised results are now different, in that there are now 6 clusters, but with better clustering and separation (mostly separated by dog diet and owner diet). As well as this statistical error, we also realised that we had not properly explained our methods.

Therefore, to address this point, we have now altered and expanded the methods section as follows:

Unsupervised uniform manifold approximation and projection (UMAP) [52] was used to reduce the multivariate owner and dog metadata data to two dimensions for visualisation purposes (using the ‘uwot’ package [51]). After this, density-based spatial clustering (DBSCAN, using the ‘dbscan’ package [26]) was applied to the UMAP outputs to identify potential groupings within these owner-animal metadata, prior to the data pre-processing necessary for the statistical and machine learning modelling, as explained above. The UMAP approach was specifically developed to be applicable to mixed datasets with categorical, ordinal and numeric data [52]. It is a potent, non-linear dimension-reduction technique that outperforms principal component analysis (PCA) and t-distributed stochastic neighbour embedding (TSNE) in its ability to preserve the global and local structure of data [52]. It employs a specific type of manifold projection known as Riemannian geometry, which has the strength of enabling meaningful clusters to be identified and visualised in a reduced dimensional space. This approach differs from PCA in that it typically reduces multivariate data to two parameters (arbitrarily called x and y); further, rather than calculating variability, UMAP instead non-linearly transforms the data to create a lower-dimensional representation, allowing more complex data patterns to be captured, compared with linear methods like PCA. The axes in UMAP do not represent ‘principal components’ and cannot be interpreted in the same way; instead, they help visualise high-dimensional data in a lower-dimensional space.

Each record (individual row of the dataset with one owner-dog combination) contributes one point to the UMAP graph. The following animal variables were included: *dog age*, *dog sex*, *neuter status*, *diet* and *breed size category*. In addition, the following owner variables were included: *owner age*, *owner gender*, *owner diet*, *location*, *setting*, *education*, *income* and *decision-maker status*. Following best practise [53], different distance metrics were used including Euclidian (for continuous data e.g., *dog age*), Manhattan (for ordered categorical data e.g., *owner age*, *education*) and Hamming (for non-ordered categorical data e.g. *dog diet*, *dog sex* etc)

The quality of DBSCAN clustering was assessed with a silhouette plot along with silhouette scores [53] and also by calculating Davies-Bouldin’s cluster separation measure [54]. The silhouette plot provides a visual representation of how well points within the UMAP have been classified into their respective clusters, whilst average silhouette widths (silhouette scores) quantify how similar the point is to its own cluster relative to other clusters; silhouette scores can range from +1 to -1, with positive and negative values indicating good and poor matching, respectively; further, average cluster silhouette scores of 0.26-0.50, 0.51-0.70 and 0.71-1.00 represent weak, reasonable and strong clustering, respectively [55]. Davies-Bouldin’s cluster separation measure is determined from the ratio between the scatter within and the separation between clusters, with smaller values suggesting better clustering and values <1 representing very good clustering.

**Reviewer comment 3.3**

2. Using healthcare variables (# of vet visits, prescribed medication by vet, on therapeutic diet) as predictors of owner perceived health of their pet seems not all that informative, since likely these variables are a part of an owner's assessment of the health of their pet. For example, I find it hard to believe that very many owners who had to take their pet to the vet numerous times in a year would characterize their pet as healthy. So, it seems obvious that these would end up being the most predictive of the outcome. Since, to me, they seem to be part of the outcome, why consider them as predictors at all? Authors could instead use those as alternative outcome measures of health and see how diet and dog and owner characteristics are associated with perceived health.

Many thanks for this comment which we have considered carefully. We agree it is obvious that healthcare variables (received medication, veterinary visits) are highly likely to be associated with the actual health of the dog. However, we decided against using these healthcare metrics as outcome variables for the following reasons. Firstly, this was something that had already been done in the Knight *et al.* study, and we were keen to avoid unnecessary overlap. However, more importantly, we specifically wanted to focus on **owner perceptions about health** rather than **actual dog health**. We tried to make this as clear as we could in the original drafts of our original manuscript, including how the title was worded (*“Variables associated with owner* ***perceptions of the health*** *of their dog: further analysis of data from a large international survey”*), aims (*“Therefore, the aim of the current study was to… create models that best predicted* ***owner opinions*** ***of health****…”*) and conclusions (*“Variables most strongly associated with owner* ***perceptions of health*** *are age of dog and the healthcare variables…”*).

In the questionnaire from the original study, question 18 best reflected owner perspectives:

*“In your own opinion, which best describes your animal over the previous 12 months.”*

… and the possible responses were:

- *Healthy*
- *Generally healthy with minor or infrequent problems*
- *Significant or frequent problems*
- *Seriously ill*

Therefore, the owner is required to formulate a subjective, holistic opinion about their dog’s health, which will necessarily require them to take many factors into account (including aspects of healthcare). We have tried to indicate how these factors could affect the owner perception the following diagram:

Whilst owner’s responses will be associated with the actual health of their dog (which in turn is determined by other factors), they will also be influenced by owner *knowledge and recollection* as well as their *attitudes, beliefs and behaviours.*

In contrast, the healthcare variables were derived from questions in the Knight et al. survey that required factual recall (rather than a holistic opinion of health), for example:

- *“Has your animal received medications in the last year, apart from routine vaccinations and treatments for skin or internal parasites, such as fleas, ticks, lice, heartworm and intestinal worms? Please do not include neutering operations (spay/castration) or microchipping.”*
- *“How many times has your animal visited a veterinarian (or received a vet home visit) in the previous 12 months?”*

Unlike the *owner perceptions of health*, these do not require an owner to formulate a holistic opinion about their dog’s health and, therefore, they **do not** measure the same thing. Further, these variables will be included in the knowledge base that an owner uses to construct their opinion about how healthy their dog is. For this reason, it is important that they also be considered as predictor variables in our modelling studies. In fact, ignoring them might have adverse effects on model performance. In this respect, their effect on owner perceptions of dog health will be present in the system irrespective of whether they are included in a particular model, as illustrated in this next diagram:

Since this increases the amount of unassigned variance, it will lead to the generation of models that perform poorly (because of the extra ‘unexplained’ variance in the system). A further danger is that some of this variance might then be incorrectly attributed to the remaining variables leading to erroneous estimates of their regression coefficients.

Another advantage of including healthcare variables as predictors is that they can then provide a benchmark against which other associations can be compared. For example, all our studies, the next most important variable affecting owner perceptions of health was dog age, but this was weaker than the associations with medications and visit frequency. Compared with dog age, associations with all other variables were weaker still.

One concern with including healthcare variables as predictors is the fact that their strong associations with the outcome measure could hide much weaker associations with other variables. This was addressed in our logistic regression models by calculating *variance inflation factors* (VIFs; Fox and Monette, 1992), which provide an indication of how much the variance of one variable is ‘inflated’ (or ‘deflated’) by the inclusion of other variables in the model. In our final models, VIFs were always well within acceptable limits, suggesting that the impact of including the healthcare variables on other (owner-animal metadata) variables was minimal.

For these reasons, we believe it appropriate to use healthcare variables as predictors. However, by way of compromise, we have decided to include additional models without the healthcare variables (owner-animal metadata models). These models can then be compared with the all-variable models (that include healthcare variables). We hope that the reviewer will accept this compromise.

Details of the resulting changes are as follows:

**Methods**

In the first step of the multiple logistic regression modelling, two multiple regression models were created for each outcome variable (*any health problem*, *significant illness*); all predictor variables were included in the first of these models (*all-variable* model), whilst only owner and animal variables were included in the second model (*owner-animal metadata* model). The performance of *all-variable* and *owner-animal metadata* models were then compared with each other and with the *best-fit* models that were created subsequently.

**Results**

*Any health problem binary variable*

Next, we created two multiple regression models, again on data from primary decision-makers, with *any health problem* binary as the outcome variable and that either contained all predictor variables or just the owner-animal metadata. For the *all-variable* model (S3a Fig), the variables with the strongest associations with the outcome variable were *dog age* (both the ‘1-5y’ and ‘6-20y’ categories), *received medication*, *veterinary visits* (the ‘1 visit’, ‘2 visits’, ‘3 visits’ and ≥4 visits’ categories) and *switched to a therapeutic diet*. In terms of model performance, pseudo-R^2^ was 0.439, BIC was 1836 and AUC on the test dataset was 0.852 (S5 Fig). For the *owner-animal metadata* model (S3b Fig), the variables with the strongest associations with the outcome variable were *owner age* (‘50-59y’ and ‘≥60y’ categories), *dog age* (‘6-20y’ category), *breed size category* (‘giant breed’ category) and *dog diet* (‘vegan diet' category). However, performance of this model was considerably worse in terms of generalisability (BIC 2244), the proportion of variance explained (pseudo-R^2^ 0.150) and prediction accuracy (AUC on the test dataset: 0.668).

*Serious illness*

Next, we created both an *all-variable* and an *owner-animal metadata* model, using data from primary decision-makers, with *significant illness* binary as the outcome variable. For the *all-variable* model (S4a Fig), the variables with the strongest associations with the outcome variable were *dog age* (‘6-20y’ category), *giant breed*, and *received medication*. In terms of model performance, pseudo-R^2^ was 0.403, BIC was 679 and AUC on the test dataset was 0.890. For the *owner-animal metadata* model (S4b Fig), the variables with the strongest associations with the outcome variable were *dog age* (‘6-20y’ category) and *giant breed*. Once again, the performance of this model was considerably worse in terms of generalisability (BIC 799), the proportion of variance explained (pseudo-R^2^ 0.124) and prediction accuracy (AUC on the test dataset: 0.692).

Finally, we made considerable amendments to the discussion, partly to explain better the reasons for including the healthcare variables, but also to consider reasons for differences in the results obtained with the different models. We have also decided that the figures we created for this point-by-point response would be helpful to explain the reasoning and can be used as a base for the explanations. The relevant section in the discussion is as follows:

The approach of the current study also differed by our decision to include healthcare variables (*received medication*, *veterinary visits*, *switched to a therapeutic diet*) as predictors. In the previous study [15], these variables were instead used as proxy measures for dog health and compared amongst dogs fed different diet types. Whilst both owner perceptions of health and healthcare variables will be associated with actual dog health, they are materially different. As already discussed, owner perceptions of health were derived from a question where owners subjectively rated their dog’s health [15], and these perceptions will be influenced by other factors including owner knowledge and recollections, as well as their beliefs and attitudes (Fig 9). Conversely, the healthcare variables were derived from survey questions that required factual recall rather than the formulation of a subjective, holistic opinion about health. For example, owners were asked whether their dog “had received medications in the last year” and how many times the dog had “visited a veterinarian or received a home visit” [15]. Given that such healthcare information will contribute to the knowledge an owner utilises when constructing opinions about the health of their dog, we reasoned that they were valid predictor variables in our statistical modelling. Further, excluding them from statistical models could be problematic as illustrated in Fig 9c; unlike removing observations from owners who were not primary decision-makers (as discussed above), where any variance associated with the variable is removed, simply excluding a variable from a statistical model will not eliminate any associated variance. The ‘hidden’ variance associated with the healthcare variables might then be incorrectly attributed to other variables leading to erroneous estimates of their regression coefficients. Any unassigned variance also adversely affects model performance, which likely explains why the models that only included owner-animal metadata performed poorly as discussed below.

**Reviewer comment 3.4**

**Minor points:**

1. line 43-44: "albeit having limited although... was limited" is awkwardly phrased.

Many thanks for spotting this error. We have corrected the sentence as follows:

For the *significant illness* outcome, received medication, veterinary visits, dog age and were again the most important predictors for both logistic regression (AUC 0.903) and XGBoost (AUC 0.887), whilst breed size category, education and owner age also featured in the latter. Any contribution from the dog vegan diet variable was negligible.

**Reviewer comment 3.5**

2. line 50: "that diet the fed" is awkwardly phrased

We now chosen to rephrase as follows:

The results of the current study extend the previous research using the same dataset and suggest that diet has limited impact on owner-perceived dog health status; instead, dog age, frequency of veterinary visits and receiving medication are most important.

**Reviewer comment 3.6**

3. line 318 - it appears as though authors have switched vegan and vegetarian (Table 1 has 493 vegetarians and 221 vegans, while the text has them reversed).

Many thanks for spotting this error. We have now corrected it.

**Reviewer comment 3.7**

4. lines 325-328: the numbers given for owners identifying as vegan (324) does not make sense given the numbers in Table 1 - even if the labels are reversed in Table 1, 324 would not be 56% of all vegan owners. Also, the numbers of vegetarian and vegan diets for the dogs do not match those presented in Table 1. Finally, it is not clear why the number of observations in the cluster mentioned in this section changes depending on whether it is being illustrated by owner diet or by dog diet (381 vs 383).

**Reviewer comment 3.8**

5. In supplemental figures S1 and S2, why are authors not using the processed variables (with collapsed categories) for client age and education as described in lines 161-163?

**Please note that these two points are related and, therefore, have been answered together.**

Many thanks for this comment, which has partly arisen because we had not properly explained the use of data in the original manuscript, but also because of some typographical errors, as we explain below.

The main reason for the difference in numbers for the UMAP (in both the results section and Fig S1/S2) was that this analysis was performed before most of the pre-processing where we created the new variables. The reason for this is because this approach was intended to be an initial exploratory analysis and visualisation; as a result, it was more appropriate to use the original dataset. As can be seen in the statistical file (see 101), the only pre-processing that occurred before UMAP was to separate sex into sex and neuter variables and to remove dogs <1 year (because we were interested in adult dogs). We apologise for not having made this clear originally and have now reworded both the methods and initial results to explain this:

**Methods**

*Initial dataset pre-processing*

Initially, data were imported into the statistical environment using the ‘readxl’ package [42], and then manipulated using the ‘dplyr’ package [28]. Columns were renamed for clarity and to improve compatibility with use in R (see below). Counts of all categorical data were examined, and categories with small groups were either removed or combined with others to exclude missing data or to reduce dataset imbalance. The variables included in the dataset were owner variables and animal variables (collectively owner-animal metadata), as well as healthcare variables and health outcomes variables.

Data pre-processing occurred in two stages: in the first stage (prior to Uniform Manifold Approximation and Projection; UMAP; see below), the original dog sex variable (male sexually intact, male castrated, female sexually intact, female spayed) was separated into two binary variables, *dog sex* (male, female) and *neuter status* (yes, no). In addition, data were excluded from dogs where age was reported as ‘unsure’ (n=3) or were aged <1 year (n=26), to ensure a focus on adult dogs.

To facilitate the modelling studies (logistic regression and XGBoost), further data pre-processing steps were undertaken after UMAP. For owner variables, pre-processing included…

And...

*Owner-dog metadata visualised by Uniform Manifold Approximation and Projection with Density-Based Spatial Clustering*

Uniform Manifold Approximation and Projection (UMAP) [52] was used for initial dimension reduction (using the ‘uwot’ package [51]), whilst density-based spatial clustering (DBSCAN, using the ‘dbscan’ package [26]) was applied to identify groupings within the owner-animal metadata, but before most data pre-processing, as explained above.

**Results**

*Owner-dog metadata visualised by Uniform Manifold Approximation and Projection*

The original dataset comprised information on 2,639 owner-dog pairs [15]. After the initial pre-processing stage to create separate dog sex and neuter status variables, and also removing dogs without age data (n=3) or that were <1 year of age (n=27), the number of owner-dog pairs with available data was 2,609. We first used a UMAP projection and density-based clustering to create a simple overall visualisation of relationships in these owner-animal metadata (Fig 1; S1 and S2 Figs).

Finally, there was an error in the size reported for the original cluster 2, which contained 383 owner-dog pairs (381). This has now been corrected in the revised manuscript, although the size (and shape) of the clusters has changed, given our different methodology and revised analyses. The relevant part of the results has also been expanded in response to comments from reviewer 2, but the cluster numbers are now all correct. The revised wording for this section is as follows:

Six distinct clusters were identified, with a Davies-Bouldin’s cluster separation measure of 0.652 (good). Good clustering was evident within 3 clusters (average silhouette scores: cluster 2, 0.86; cluster 5, 0.84; cluster 6, 0.80), there was reasonable clustering in two others (average silhouette scores; cluster 3, 0.68; cluster 4, 0.59), but clustering was poor in the final cluster (cluster 1, average silhouette score 0.36).

Examining the owner-animal metadata, cluster 2 was most distinctive, almost-exclusively comprising dogs fed either a vegan (328/374 [87.7%] points in the cluster, 97.6% of all vegan dogs) or vegetarian (31/374 [8.3%] points in the cluster, 88.6% of all vegetarian dogs) diet, and also containing a predominance of owners consuming a vegan diet (332/374 [88.8%] points in the cluster, 57.1% of all vegan owners). In contrast, cluster 3 comprised owners who consumed either a vegan (249/608, 41.0%), vegetarian (240/608, 39.5%) or pescatarian (119/608, 19.6%) diet, but fed their dog either a conventional meat-based (423/608, 69.6%) or raw (175/608, 28.8%) diet. Cluster 6 was also distinctive in that it mostly comprised owners who consumed an omnivore (214/316, 67.7%) or omnivore reducing (98/316, 31.0%) diet and fed their dogs a raw diet (299/316, 94.6%). In the remaining 3 clusters (clusters 1, 4 and 5), most owners consumed either an omnivore or omnivore-reducing diet and usually fed their dog either a conventional meat-based diet or raw diet. Besides owner and dog diet, there were no major differences amongst clusters for the other variables, although some variables did appear to separate some clusters (e.g., cluster 4 for the setting variable; cluster 5 for the location variable; S1 and S2 Figs).

**Reviewer comment 3.9**

6. How were diets for those on a therapeutic diet handled? Those would be at the recommendation of the veterinarian, so not exactly the complete choice of the owner.

Many thanks for this comment. This was explained in the original paper, which we have reproduced here:

*“Where animals were fed a prescription or therapeutic diet, guardians were asked to base answers on the diet in use prior to the commencement of the therapeutic diet.”*

Therefore, the information gathered from such owners was about their chosen diet before starting the therapeutic food. We hope that makes sense. As this point could be confusing to reads who are less familiar with the original paper, we have explained this better in the methods as follows:

Animal information used for the current study included: age, sex, neuter status, breed size category (e.g., toy, small, medium, large and giant) and main diet type (including conventional, raw, vegetarian, vegan, *in vitro* meat, insect, fungal or algal). We also used responses from owners about the healthcare their dog received including number of veterinary visits in the last 12 months, whether the dog had received medication (besides those used in preventive care e.g. routine vaccinations and endo- or ecto-parasite treatments) in the last 12 months, and whether the dog was on a therapeutic diet; when dogs were fed a therapeutic diet, owners were asked to base the information on their dog’s diet on their previous diet [15].

**Reviewer comment 3.10**

7. To avoid confusion in the usage of the variables that are derived from the same information, authors should only mention the "selected" one for consideration in the multivariable models. For example, in lines 395-396, authors do not need to mention both breed size category and giant breed - their methods describe how they chose the "best" variable. I do wonder, however, in a situation like this where giant breed results in a lower BIC, if authors are in fact combining too many breeds together by just using this single binary variable to represent breed.

Many thanks for this comment. We have now revised the results so that, for most variables, only the version of the predictor variable that we selected is shown. However, we hope that the reviewer will allow us to make an exception for both the *owner diet vegan* and *dog diet vegan* variables, which are of particular interest, given the conclusions from the Knight et al study. Further, in order to address a concern of reviewer 2 above, we have created additional multiple LR models where these variables have been added back to the best-fit multiple LR models (to determine their effect on model performance). As a result, we believe it to be of interest to the reader to see the unadjusted odds ratio from simple regression for these variables as well as the *owner diet* and *dog diet* variables.

Also, apologies that the handling of the *giant breed* variable was not clear from the methods and results. There were two reasons for creating a binary variable for this category. First, we found that this was the often only category of significance amongst breeds when refining the *serious illness* multiple LR model and switching from the 5-category breed variable improved model fit. Further, we identified some issues with model convergence when the 5-category variable was included, and we were concerned that the calculation of OR and 95%-CI could be inaccurate. Therefore, the giant breed variable was then used in the stepwise regression procedure for this outcome. This is now explained in the methods section as follows:

Further, for the *significant illness* outcome variable, the *giant breed* predictor variable performed better than the *breed size* *category* variable, and there were also some issues with model convergence for the latter.

**Reviewer comment 3.11**

8. The list of variables considered for the multiple regression in lines 435-437 does not seem to correspond to the results in Table 3 and the approach described in the Methods section for selection of those variables. For example, owner gender, animal related career and neuter status all seem to be variables that should be considered...and it is not clear why giant breed is being considered. The same can be said for the list of variables give in lines 513-515. Authors need to make sure what they state matches their results and the approach they say they took.

Many thanks for spotting this error. In response to a comment from reviewer 2, we have now amended the approach to variable selection for multiple regression modelling. Since we now start the process with a model containing all variables, the sentence in the results no longer exists. We have explained the use of the giant breed variable in the response to the previous comment and hope that this is now clear.

**Reviewer comment 3.12**

9. captions to figures 5, 6, 8, 9 - authors refer to a false-negative rate (lines 488, 501,560, 573), but these should be false positive rates based on your figure.

The reviewer is correct that these captions should instead read “false-positive rate”. We apologise for this error and have now corrected it.

**Reviewer comment 3.13**

10. lines 603: authors state that dog diet was not associated with the significant illness outcome, but based on Table 4, raw diet was.

Many thanks for spotting this; it was indeed an error, and we have corrected it in the revised manuscript.

**Other changes to manuscript**

*Dog age*

Please note that, in performing further statistical analyses for this revision, we spotted an error which had occurred when we classified dog age into quintiles, whereby data from 165 dogs who were 1 year of age were inadvertently omitted during data pre-processing (read_data_101.rmd). Below is an output from this stage, where these dogs have become “NA”.

This error occurred because, by default, values at the lower boundary are excluded when using the ‘cut’ function. We apologise for this error, and have now revised the code to correct this:

After this correction, the continuous dog age variable now performs better in logistic regression analyses than this categorical variable, albeit that it is necessary to add basic splines to address one of the assumptions of logistic regression (that the logit of the response and the predictor variable was not linear). By adding splines, we now report two odds ratios for dog age; the first is the OR **per year** for dogs between 1 and 5 years of age; the second is the odds ratio **per age** for dogs between 6 and 20 years; for example (Table 2; simple LR on the any health problem variable):

We hope that this makes sense.

*Removal of the ordinal logistic regression modelling*

In order to address the issues of all reviewers, we have had to perform additional statistical analyses, provide more details in both methods and results, and add clarifications in the discussion. Our concern was that this made the manuscript too long, and reviewer 2 had already commented that they found it hard to follow which model was which. As a result, we made the decision to remove the ordinal logistic regression modelling; results from this were very similar to those of binary logistic regression meaning that there was unnecessary repetition. We hope that this change improves the clarity of both methods and results, as well as enabling us to accommodate the extra detail that the reviewers requested.

*Minor text editing*

In this revision, it was necessary to make substantial alterations and to improve clarity throughout. As a result, we have copy edited the whole manuscript and made minor changes to wording to correct typographical and grammatical errors and improve the final version. All such changes are shown in the point-by-point response.
