# Supplementary material for: Variables associated with owner perceptions of the health of their dog: Further analysis of data from a large international survey
Source: PLoS One. 2024 May 15;19(5):e0280173. doi: 10.1371/journal.pone.0280173 (PMC11095744; doi:10.1371/journal.pone.0280173)
Supplement: Attachment — Submitted filename: Review 2024 02 26.docx [file pone.0280173.s029.docx]

Variables associated with owner perceptions of the health of their dog: further analysis of data from a large international survey

PONE-D-22-34984R2

Review 26/02/24

Minor revisions required

Comments

I appreciate the many improvements made. As acknowledged in places, the current study has not assessed the same outcomes as the prior study by Knight et al (citation 15). The current study has assessed correlation of various factors with owner opinion of illness (and its severity), within two created variables: ‘any healthcare problem’ and ‘significant illness’. The prior study (15) drew conclusions about which of three canine diets were healthiest and least hazardous overall, based on 7 general indicators of illness (including owner opinion, but also including more objective data such as medication usage), the reported prevalence of 22 specific healthcare disorders, and also prior studies indicating hazards (pathogens and nutritional imbalances) associated with raw meat diets. As also noted in the current study, the data sets examined are also slightly different (the current study excluded data from dogs aged < 1 yr (n = 26) and 111 respondents who were not primary decision-makers with respect to dog diet choice). Hence, the new wording ‘we extend the findings of the prior study…’ is correct. I appreciate the change from the old wording ‘our findings contrast/conflict with those of the prior study,’ which was not really correct.

I also appreciate the statistical changes made, and also the clarifications, which will help non-statistician readers to understand the study.

However, some additional edits are required, for accuracy, consistency, and clarity of communication.

Required edits

nb: line numbers were taken from the ‘tracked change’ version.

Introduction

l. 86-88 “However, besides being reliant on owner-reported health, rather than more objective health measures, no account was taken of possible confounding from other variables.”

This description of the prior study (15) is not correct. The prior study did rely on owner-reported health, but it also relied on a range of other indicators of illness. Hence the sentence should simply become “However, no account was taken of possible confounding from other variables.”

Materials and Methods

l. 105 OSF have now provided a permanent link to this data repository at <https://osf.io/nbepu> and this link should instead be used.

l. 129 The description of R as an “online open-access statistical language and environment” is confusing and misleading. I would suggest “open-source statistical software”, but at least dropping “online open-access” in favour of the technically correct term “open-source”.

l. 147 The methods section is sometimes unnecessarily detailed in the exact data analysis steps that were performed. The two sentences from line 147 to line 149 could be dropped. I’d suggest to drop or shorten such specific sentences in favour of conciseness. One other example for this can be found in line 354, where the words “and saved to file” can be dropped.

Discussion

l. 1035-1037 “The primary aim of that study was to examine associations between owner opinions of dog health status and the type of diet that owners predominantly fed…”

This description of the prior study (15) is not correct. The aim was not limited to examining associations between owner opinion and diet. It was to examine variation of a (wider) range of illness indicators with diet, as stated in that study. Hence those words must be deleted, and the sentence can simply start with the following words “The key finding of that prior study was that that health status was…”

 l. 1302 “in contrast to the findings of the previous study [15]”

This is not correct as the prior study did not assess the same outcomes/associations, as noted above. These words need to be simply deleted.

l. 1306 “association with dog health status” needs to be corrected to “association with owner opinion of dog health status.”

l. 1364 “Current evidence suggests that both such diet types are often not

formulated appropriately to be complete and balanced for essential nutrients”

That concern is true and well-supported for raw meat-based diets. However there is no evidence those concerns are more prevalent for vegan diets, than for conventional meat-based diets. There are significant bodies of studies indicating past problems with both of these diet groups (e.g. within meat-based diets: <https://avmajournals.avma.org/view/journals/javma/234/3/javma.234.3.347.xml>, <https://pubmed.ncbi.nlm.nih.gov/30410174/>, <https://www.nature.com/articles/s41598-017-17159-7>), and some of those studies of conventional meat-based diets have been much larger than any studies of vegan diets. Hence, it is probable that this problem has been identified for more conventional meat-based diets, than for vegan diets. Additionally, there appear to be few systemic differences in steps taken to ensure nutritional soundness and quality. The only study comparing the two groups <https://www.veterinaria.org/index.php/REDVET/article/view/92> found that both groups were normally produced to fairly good standards overall, but companies producing plant-based diets had slightly better standards overall.

Hence, the words above must be deleted, or alternatively discussion of this point requires expansion, to include the points and references above.

Other

Diets ‘raw’ and ‘conventional’ should be clarified as meat-based throughout, e.g. ‘raw meat diet’, ‘conventional meat-based diet’. This is to ensure clarity/future-proofing in case any raw vegan diet exists or is soon created.
