# Supplementary material for: Variables associated with owner perceptions of the health of their dog: Further analysis of data from a large international survey
Source: PLoS One. 2024 May 15;19(5):e0280173. doi: 10.1371/journal.pone.0280173 (PMC11095744; doi:10.1371/journal.pone.0280173)
Supplement: Attachment — Submitted filename: Response to Reviewers.docx [file pone.0280173.s030.docx]

**Response to reviewer comments**

**PONE-D-22-34984**

**Variables associated with owner perceptions of the health of their dog: further analysis of data from a large international survey**

*Barrett-Jolley and German*

We would like to thank the editor and all reviewer 2 for, once again taking time to review this manuscript, and are pleased that we are close to a consensus. Once again, we have carefully considered their additional comments and have addressed them in the manuscript (see revised manuscript with changes highlighted), as well as explaining below how this has been done. On this occasion, since there were only 11 comments to address, we have not included a summary table.

**Editorial and journal requests**

*If you would like to make changes to your financial disclosure, please include your updated statement in your cover letter. Guidelines for resubmitting your figure files are available below the reviewer comments at the end of this letter.*

We have checked the financial disclosure statement and this is still accurate; no changes are required.

*If applicable, we recommend that you deposit your laboratory protocols in* [*protocols.io*](http://protocols.io) *to enhance the reproducibility of your results.* [*Protocols.io*](http://Protocols.io) *assigns your protocol its own identifier (DOI) so that it can be cited independently in the future. For instructions see:* [*https://journals.plos.org/plosone/s/submission-guidelines#loc-laboratory-protocols*](https://journals.plos.org/plosone/s/submission-guidelines#loc-laboratory-protocols)*. Additionally, PLOS ONE offers an option for publishing peer-reviewed Lab Protocol articles, which describe protocols hosted on* [*protocols.io*](http://protocols.io)*. Read more information on sharing protocols at* [*https://plos.org/protocols?utm_medium=editorial-email&utm_source=authorletters&utm_campaign=protocols*](https://plos.org/protocols?utm_medium=editorial-email&utm_source=authorletters&utm_campaign=protocols)*.*

Many thanks for this information. As this study involved statistical analysis of secondary data, there are no laboratory protocols to deposit. However, we will consider this for future manuscripts.

*Please review your reference list to ensure that it is complete and correct. If you have cited papers that have been retracted, please include the rationale for doing so in the manuscript text, or remove these references and replace them with relevant current references. Any changes to the reference list should be mentioned in the rebuttal letter that accompanies your revised manuscript. If you need to cite a retracted article, indicate the article’s retracted status in the References list and also include a citation and full reference for the retraction notice.*

We have rechecked the reference list, and made some minor edits to ensure correct formatting, as well as to ensure accuracy. The changes made are shown in the tracked changes of the revised manuscript. The only more substantive change is that a full paper is now available for reference 83 (lines 1441-1444), and details have been updated for this.

We have checked the current status of all references used and, as far as we can tell, none have been retracted. In the original version of the manuscript, we included details of one erratum (reference 10). However, since originally submitting in December 2022, an erratum has been published for reference 15. Details of this erratum have now been added to the reference list (lines 1225-1226).

**Reviewer 2 comments**

***General comments***

*I appreciate the many improvements made. As acknowledged in places, the current study has not assessed the same outcomes as the prior study by Knight et al (citation 15). The current study has assessed correlation of various factors with owner opinion of illness (and its severity), within two created variables: ‘any healthcare problem’ and ‘significant illness’. The prior study (15) drew conclusions about which of three canine diets were healthiest and least hazardous overall, based on 7 general indicators of illness (including owner opinion, but also including more objective data such as medication usage), the reported prevalence of 22 specific healthcare disorders, and also prior studies indicating hazards (pathogens and nutritional imbalances) associated with raw meat diets. As also noted in the current study, the data sets examined are also slightly different (the current study excluded data from dogs aged < 1 yr (n = 26) and 111 respondents who were not primary decision-makers with respect to dog diet choice). Hence, the new wording ‘we extend the findings of the prior study…’ is correct. I appreciate the change from the old wording ‘our findings contrast/conflict with those of the prior study,’ which was not really correct.*

*I also appreciate the statistical changes made, and also the clarifications, which will help non-statistician readers to understand the study.*

*However, some additional edits are required, for accuracy, consistency, and clarity of communication.*

We are glad that the detailed changes made during the second revision were appreciated and are acceptable to the reviewer. We strongly agree with them about the need for accuracy, consistency and clarity of communications. As a result, we have accepted many of their suggestions without modification. However, where we believe that clarity and accuracy can be improved beyond what is suggested, we have explained and justified our reasons for adapting some of their recommendations.

***Introduction***

*l. 86-88 “However, besides being reliant on owner-reported health, rather than more objective health measures, no account was taken of possible confounding from other variables.”*

*This description of the prior study (15) is not correct. The prior study did rely on owner-reported health, but it also relied on a range of other indicators of illness. Hence the sentence should simply become “However, no account was taken of possible confounding from other variables.”*

We agree with the reviewer that owners were not just asked for their opinions on health, but also to report other health-related information such as medication usage and the presence of different illnesses. However, since this information still relies on owner recollections, rather than being gathered independently from more objective sources (e.g., verified diagnoses made by a veterinarian; information gathered from electronic patient records), it could still have suffered from recall bias and have been modified by their owner biases and beliefs (as discussed in the manuscript). As a result, rather than deleting the first part of the sentence altogether, we would prefer to reword it as follows (lines 79-83):

*“However, besides being reliant on owner-reported health* **and their recollections of other health-related information (e.g., medication usage; the presences of specific health disorders)***, rather than more objective health measures* **(e.g., verified diagnoses made by a veterinarian; information gathered from electronic patient records)***, no account was taken of possible confounding from other variables.”*

We believe that this compromise wording is a more accurate explanation the methods used in the original manuscript, than either the original wording or the suggestion from the reviewer.

***Materials and Methods***

*l. 105 OSF have now provided a permanent link to this data repository at* [*https://osf.io/nbepu*](https://osf.io/nbepu) *and this link should instead be used.*

Many thanks for informing us of this permanent link. We have updated the revised manuscript accordingly (lines 85, 100 and 1168).

*l. 129 The description of R as an “online open-access statistical language and environment” is confusing and misleading. I would suggest “open-source statistical software”, but at least dropping “online open-access” in favour of the technically correct term “open-source”.*

Many thanks for this suggestion. We have corrected this sentence in the manuscript, instead using the term **“open-source statistical software”** (lines 133-134).

*l. 147 The methods section is sometimes unnecessarily detailed in the exact data analysis steps that were performed. The two sentences from line 147 to line 149 could be dropped. I’d suggest to drop or shorten such specific sentences in favour of conciseness.*

Many thanks for this suggestion which we have carefully considered. Unfortunately, removing these two sentences altogether would be problematic because they introduce the rest of the data pre-processing section. Therefore, instead, we have created a single more concise sentences as follows (lines 153-154):

**“Data were imported into the statistical software using the ‘readxl’ package [42] and manipulated using the ‘dplyr’ package [28], with columns renamed for use in R (see below).”**

*One other example for this can be found in line 354, where the words “and saved to file” can be dropped.*

Many thanks for this suggestion. On re-reading this and the preceding sentence, it did not make sense as there was a typographical error. Therefore, we have reworded this section as follows (lines 381-383):

*“The model* **was trained** *using the augmented training data***, and then tested against the validation training subset with area-under the curve (AUC) as the evaluation metric, enabling the final model to be built***.****”***

We hope that this is acceptable.

***Discussion***

*l. 1035-1037 “The primary aim of that study was to examine associations between owner opinions of dog health status and the type of diet that owners predominantly fed…”*

*This description of the prior study (15) is not correct. The aim was not limited to examining associations between owner opinion and diet. It was to examine variation of a (wider) range of illness indicators with diet, as stated in that study. Hence those words must be deleted, and the sentence can simply start with the following words “The key finding of that prior study was that that health status was…”*

This comment is related the earlier one on lines 86-88 of the manuscript (see above). As with that earlier point, rather than delete the comment altogether we believe it to be more accurate to amend the wording to ensure we properly reflect the aims of the earlier study. Therefore, we have reworded this section as follows (lines 788-792):

**“One of the aims** *of that study was to examine associations between* **the type of diet that owners predominantly fed, and both subjective** *owner opinions* **about their** *dog***’s** *health* **as well as their recollection of other health information (e.g., medication usage and the presence of specific health disorders). A key conclusion of that study was** *that health status was positively associated with feeding either a vegan or raw diet, compared with other diet types (including conventional and vegetarian).”*

Once again, we believe this compromise wording to be more accurate than both the wording in the original manuscript and the wording were we to adopt the reviewer’s suggestion of removing the sentence altogether.

*l. 1302 “in contrast to the findings of the previous study [15]”*

*This is not correct as the prior study did not assess the same outcomes/associations, as noted above. These words need to be simply deleted.*

Many thanks for this suggestion. We have deleted this statement, and the revised sentence now reads (lines 954-955):

*“***However, the** *diet of the dog had little to no effect in all models, except for those that only included owner-animal metadata, albeit that these models performed poorly.”*

*l. 1306 “association with dog health status” needs to be corrected to “association with owner opinion of dog health status.”*

Many thanks for this suggestion. We have made this change. Please note that a hyphen has also been added between ‘*clinically’* and *‘meaningful’* i.e. **‘clinically-meaningful’** (lines 956-958):

*“Therefore, we could not find convincing evidence that particular food types (conventional, vegan, vegetarian or raw) have any* **clinically-meaningful** *positive or negative association with* **owner opinions of** *dog health status.”*

*l. 1364 “Current evidence suggests that both such diet types are often not formulated appropriately to be complete and balanced for essential nutrients”*

*That concern is true and well-supported for raw meat-based diets. However there is no evidence those concerns are more prevalent for vegan diets, than for conventional meat-based diets. There are significant bodies of studies indicating past problems with both of these diet groups (e.g. within meat-based diets:* [*https://avmajournals.avma.org/view/journals/javma/234/3/javma.234.3.347.xml*](https://avmajournals.avma.org/view/journals/javma/234/3/javma.234.3.347.xml)*,* [*https://pubmed.ncbi.nlm.nih.gov/30410174/*](https://pubmed.ncbi.nlm.nih.gov/30410174/)*,* [*https://www.nature.com/articles/s41598-017-17159-7*](https://www.nature.com/articles/s41598-017-17159-7)*), and some of those studies of conventional meat-based diets have been much larger than any studies of vegan diets. Hence, it is probable that this problem has been identified for more conventional meat-based diets, than for vegan diets. Additionally, there appear to be few systemic differences in steps taken to ensure nutritional soundness and quality. The only study comparing the two groups* [*https://www.veterinaria.org/index.php/REDVET/article/view/92*](https://www.veterinaria.org/index.php/REDVET/article/view/92) *found that both groups were normally produced to fairly good standards overall, but companies producing plant-based diets had slightly better standards overall.*

*Hence, the words above must be deleted, or alternatively discussion of this point requires expansion, to include the points and references above.*

Many thanks for the comments which we have again considered carefully. On balance, we believe that it is best to remove this part of the discussion altogether, as this can be done without much loss of meaning. In doing so, some minor wording changes were required to improve understanding. Therefore, the revised section (lines 978-994) now reads as follows:

*“As discussed above, it is also feasible that the attitudes and beliefs of owners might either consciously or unconsciously have influenced responses about the health of their dog, a point that the authors of the previous study emphasised [15]. For example, owners who believe a particular type of diet to be optimal for dog health, might be more likely to perceive their dog to be healthy, whether or not this was objectively true. Whilst such a bias would feasibly affect any diet type, a greater bias might be expected with diets perceived to be ‘unconventional’, again, as acknowledged in the previous study [15]. Although increasing in popularity [1], both vegan diets and raw meat diets are still uncommon choices for owners, and many veterinary professionals either do not recommend them or might even advise against their* **use. Given** *that owners who make an active choice to feed either a raw or vegan diet* **might be acting contrary to veterinary advice, they would arguably** *be more defensive about this* **decision than would an** *owner who feed***s a** *conventional* **diet. This in turn** *might unconsciously have biased responses about the health of their dog, for example, minimising the significance of any illnesses.”*

***Other***

*Diets ‘raw’ and ‘conventional’ should be clarified as meat-based throughout, e.g. ‘raw meat diet’, ‘conventional meat-based diet’. This is to ensure clarity/future-proofing in case any raw vegan diet exists or is soon created.*

Many thanks for this comment which we have also carefully considered. With the greatest of respect, we do have concerns about the way in which the diet types named in the original study. In this respect, the terms “raw meat-based diet” and “conventional meat-based diet”, arguably, do not accurately represent the constituents of either of these diet types. Regarding conventional diets, wet and dry formulations can contain a range of raw materials from both animal and plant sources. Further, whilst some of the materials from animal sources will be from meat (e.g., muscle tissue, including chicken and fish), they might also come from other parts of the animal including organ tissue and bone meal. Moreover, dairy products and egg can also be a key part of the formulation in some conventional diets, but these would not be considered to be ‘meat’. This can also be the case for raw diets, not least those formulated at home, where they might include organ meat, bone, animal fat, eggs and dairy products.

Nonetheless, we accept that there is a need to be consistent with the categories used for the original survey question in the previous study. Therefore, as a compromise, whilst we have now explained in detail the four diet categories we used (vegan, vegetarian, raw and conventional), and related them to those used in the original study, as well as the survey question that this was based on. In this regard, paragraph 4 of the “initial data pre-processing” section has been reworded as follows (lines 192-208):

*For animal variables, in addition to having a continuous dog age variable, an ordinal variable was also created with data grouped into quintiles (1-3y, 3-5y, 5-7y, 7-9y, 9-20y).* **Further, a giant breed binary variable (yes, no) was created from the breed size category variable. Moreover, a dog diet variable was created from owner responses to a question where owners selected from different diet categories based on a question where they were asked “to consider the main ingredients within your pet's normal diet (‘meat’ includes land animals, poultry and fish)” [15]. T***he ‘unsure’ (n=8), ‘insect-based’ (n=5), ‘meat-based (lab-grown)’ (n=6) and ‘mixture’ (n=13) categories were all removed since numbers were again small.* **This left four diet categories whose names were adapted from the original study [15] as follows: ‘vegan’ (original category: “vegan [consuming no animal products]”); ‘vegetarian’ (original category: “vegetarian [including eggs or milk, but not meat]”); ‘raw’ (original category: “meat-based raw”); and ‘conventional’ (“meat-based conventional”). From these same categories,** *binary variables were created for dogs consuming a vegan (yes vs. no), vegan or vegetarian (yes or no) or a raw (yes vs. no) diet.*

We hope that this will enable readers to relate our chosen category names to those of the original survey.

**Additional changes made to the manuscript**

Once again, we have carefully reviewed the whole manuscript to identify any typographical or other errors. As a result, the following additional minor changes have been made:

1. A full stop was missing from the sentence in lines 259-262 of the revised manuscript: *“Following best practise [53], different distance metrics were used including Euclidian (for continuous data e.g., dog age), Manhattan (for ordered categorical data e.g., owner age, education) and Hamming (for non-ordered categorical data e.g. dog diet, dog sex etc)***.***”*
2. The sentence in lines 331-334 has been reworded slightly (moving the brackets) so that it makes more sense: *“This initial model was manually refined in both a backwards and forwards stepwise fashion, with the BIC* **(a measure of its goodness of fit compared with its complexity)** *being used to select the model within the same family with the best generalisability [60].”*
3. Typographical error corrected in the sentence lines 348-352: *“Given that we were using data from a secondary source,* **and** *had no further means of verifying them (e.g., by cross-checking against the original questionnaire response), we decided not to remove them as there was no valid justification to do so (e.g., a typographical error created when the original data were entered).”*
4. In line 411, there was an error in the details of the clustering information, namely the average silhouette score for cluster 3. This has been corrected to **0.62** rather than *0.68*.
5. There was a double full stop in the legend for Figure 8 (line 761), which has been corrected.
6. There was an error with the arrow colours in Figure 9c, from the healthcare variables box; the arrow to the *“knowledge and recollection of owner box”* is now black, whilst the one to the *“animal variables”* box is now red.
7. There was also a typographical error in the final sentence of the legend for Figure 9, which las been corrected as follows (line 838): *“Possible associations amongst variables in models on the primary decision-maker dataset where the healthcare variables are not included; as illustrated, the impact of this is different from (b) because no data or variance are removed; therefore, although the healthcare variables are ignored in the modelling, the variance associated within them remains within the dataset, with the potential* ***to affect performance****.”*
8. Lines 914-915. We have added a hyphen between the words *‘combined’* and *‘methods’* i.e. **“…combined-methods approach…”**.
9. Lines 921-924. There were typographical errors in this sentence, which have been corrected as follows: *“For example, in our exploratory analyses, we tested interactions* **with possible** *clinical (e.g., between the veterinary visit and received medication variables), biological (between the dog sex and neuter status variables)***,** *epidemiological (between the location and setting variables) and psychological (between owner diet and dog diet variables)* **explanations***.”*
10. Lines 1042-1043. We have corrected some typographical errors as follows: *“This raises a concern about how representative* **the** *study population* **was, which** *is of particular significance given the strong clustering based on owner diet.”*
11. Lines 1080-1082. We have corrected a typographical error as follows: *“A final approach we took, to ensure* **we had** *fully accounted for any possible effect of the dog diet vegan variable, was to add it back into the best-fit models for both the any health problem and serious illness outcome variables.”*
12. Lines 1114-1116. This sentence has been slightly reworded to improve clarity, and better explain the intended point. Differences are as follows:

- **Original:** *“Finally, the number of dogs included in many categories was small, being ≤50 in 12 of the 22 [15].”*
- **Edited:** *“Finally,* **very few (≤50) dogs were reported to be suffering from many (12/22) of the health-disorders studied** *[15],* **and, therefore, any statistical analyses would likely be underpowered to identify associations with predictor variables***.”*

13. The same typographical error was present in the table legends for Table 2 (line 538), Table 3 (line 673), S1 Table and S2 Table. In each place, we have corrected *“estimated (β)”* to “**estimates of regression coefficients (β)”** in sentence 3.

We apologise for these errors and hope that the editor will allow their correction.
